# Supplementary material for: An Evolutionary Perspective on the Origin, Conservation and Binding Partner Acquisition of Tankyrases
Source: Biomolecules. 2022 Nov 15;12(11):1688. doi: 10.3390/biom12111688 (PMC9688111; doi:10.3390/biom12111688)
Supplement: Supplementary file 1 [file biomolecules-12-01688-s001.zip › biomolecules-1913719-supplementary-11.17 -done/Data_1.pdf]

Supplementary Data S1

>TNKS1[H.sapiens][NP_003738.2]
EFKGHSLLQAAREADLAKVKKTLALEIINFKQPQSHETALHCAVASLHPKRKQVTELLLRKGANVNEKNKDFMTPLHVAAERA
HNDVMEVLHKHGAKMNALDTLGQTALHRAALAGHLQTCRLLLSYGSDPSIISLQGFTAAQMGNEAVQQILSESTPIRTSDVDY
RLLEASKAGDLETVKQLCSSQNVNCRDLEGRHSTPLHFAAGYNRVSVVEYLLHHGADVHAKDKGGLVPLHNACSYGHYEVAEL
LVRHGASVNVADLWKFTPLHEAAAKGKYEICKLLLKHGADPTKKNRDGNTPLDLVKEGDTDIQDLLRGDAALLDAAKKGCLAR
VQKLCTPENINCRDTQGRNSTPLHLAAGYNNLEVAEYLLEHGADVNAQDKGGLIPLHNAASYGHVDIAALLIKYNTCVNATDK
WAFTPLHEAAQKGRTQLCALLLAHGADPTMKNQEGQTPLDLATADDIRALLIDAMPPEALPTCFKPQATVVSASLISPASTPS
CLSAASSIDNLTGPLAELAVGGASNAGDGAAGTERKEGEVAGLDMNISQFLKSLGLEHLRDIFETEQITLDVLADMGHEELKE
IGINAYGHRHKLIKGVERLLGGQQGTNPYLTFHCVNQGTILLDLAPEDKEYQSVEEEMQSTIREHRDGGNAGGIFNRYNVIRI
QKVVNKKLRERFCHRQKEVSEENHNHHNERMLFHGSPFINAIIHKGFDERHAYIGGMFGAGIYFAENSSKSNQYVYGIGGGTG
CPTHKDRSCYICHRQMLFCRVTLGKSFLQFSTMKMAHAPPGHHSVIGRPSVNGLAYAEYVIYRGEQAYPEYLITYQIMKPEAP
SQTATAAEQKT

>TNKS2[H.sapiens][AAK13463.1]
EFKGHSLLQAAREADVTRIKKHLSLEMVNFKHPQTHETALHCAAASPYPKRKQICELLLRKGANINEKTKEFLTPLHVASEKA
HNDVVEVVVKHEAKVNALDNLGQTSLHRAAYCGHLQTCRLLLSYGCDPNIISLQGFTALQMGNENVQQLLQEGISLGNSEADR
QLLEAAKAGDVETVKKLCTVQSVNCRDIEGRQSTPLHFAAGYNRVSVVEYLLQHGADVHAKDKGGLVPLHNACSYGHYEVAEL
LVKHGAVVNVADLWKFTPLHEAAAKGKYEICKLLLQHGADPTKKNRDGNTPLDLVKDGDTDIQDLLRGDAALLDAAKKGCLAR
VKKLSSPDNVNCRDTQGRHSTPLHLAAGYNNLEVAEYLLQHGADVNAQDKGGLIPLHNAASYGHVDVAALLIKYNACVNATDK
WAFTPLHEAAQKGRTQLCALLLAHGADPTLKNQEGQTPLDLVSADDVSALLTAAMPPSALPSCYKPQVLNGVRSPGATADALS
SGPSSPSSLSAASSLDNLSGSFSELSSVVSSSGTEGASSLEKKEVPGVDFSITQFVRNLGLEHLMDIFEREQITLDVLVEMGH
KELKEIGINAYGHRHKLIKGVERLISGQQGLNPYLTLNTSGSGTILIDLSPDDKEFQSVEEEMQSTVREHRDGGHAGGIFNRY
NILKIQKVCNKKLWERYTHRRKEVSEENHNHANERMLFHGSPFVNAIIHKGFDERHAYIGGMFGAGIYFAENSSKSNQYVYGI
GGGTGCPVHKDRSCYICHRQLLFCRVTLGKSFLQFSAMKMAHSPPGHHSVTGRPSVNGLALAEYVIYRGEQAYPEYLITYQIM
RPEGMVDG

>TNKS1[G.gallus][NP_989671.1]
EFKGHSLLQAAREADLAKVKKTLALEIINFKQPQSHETALHCAVAAVHPKRKQVTELLLRKGANVNEKNKDFMTPLHVAAEKA
HNDVMEVLHKHGAKMNALDTLGQTALHRAALAGHLQTCRLLLNYGSDPSIISLQGFTAAQIGNEAVQQILSESTPVRTSDVDY
RLLEASKAGDLETVKQLCSPQNVNCRDLEGRHSTPLHFAAGYNRVSVVEYLLHHGADVHAKDKGGLVPLHNACSYGHYEVAEL
LVRHGASVNVADLWKFTPLHEAAAKGKYEICKLLLKHGADPTKKNRDGNTPLDLVKEGDTDIQDLLRGDAALLDAAKKGCLAR
VQKLCTPENINCRDTQGRNSTPLHLAAGYNNLEVAEYLLEHGADVNAQDKGGLIPLHNAASYGHVDIAALLIKYNTCVNATDK
WAFTPLHEAAQKGRTQLCALLLAHGADPTMKNQEGQTPLDLATADDIRALLIDAMPPEALPTCFKLQATVVSASLISPASTPS
CLSAASSIDNLTGPLAELAVGGASNTGDGAAGTERKEGEVSGLDMNITQFLKSLGLEHLRDIFETEQITLDVLADMGHEELKE
IGINAYGHRHKLIKGVERLLGGQQGTNPYLTFHCVSQGTILLDLAPDDKEYQSVEEEMQSTIREHRDGGNAGGIFNRYNVIRI
QKVVNKKLRERFCHRQKEVSEENHNHHNERMLFHGSPFINAIIHKGFDERHAYIGGMFGAGIYFAENSSKSNQYVYGIGGGTG
CPTHKDRSCYICHRQMLFCRVTLGKSFLQFSTMKMAHAPPGHHSVIGRPSVNGLAYAEYVIYRGEQAYPEYLITYQIVKPEAP
SQTGTAAEQKT

>TNKS2[G.gallus][NP_989672.1]
EFKGHSLLQAARESDVARIKKHLSLETVNFKHPQTHETALHCAAASPYPKRKQVCELLLRKGANINEKTKDFLTPLHVASEKA
HNDVVEVVVKHEAKVNALDNLGQTSLHRAAHCGHLQTCRLLLSSGCDPSIVSLQGFTALQMGNESVQQLLQEGIPLGNSDADR
QLLEAAKAGDVDTVKKLCTVQSVNCRDIEGRQSTPLHFAAGYNRVSVVEYLLQHGADVHAKDKGGLVPLHNACSYGHYEVAEL
LVKHGAVVNVADLWKFTPLHEAAAKGKYEICKLLLQHGADPPKKNRDGNTPLDLVKDGDTDIQDLLRGDAALLDAAKKGCLAR
VKKLCSPDNVNCRDTQGRHSTPLHLAAGYNNLEVAEYLLQHGADVNAQDKGGLIPLHNAASYGHVDVAALLIKYNACVNATDK
WAFTPLHEAAQKGRTQLCALLLAHGADPTLKNQEGQTPLDLVTADDVSALLTAAMPPSALPSCYKPQVISVSQTASSTADPLS
SVPSSPSSLSAASSLDNLSGSFSELPSVVGTNSAEGATVLEKKEVSGVDFSINQFVRNLGLEHLIDIFEREQITLDVLVEMGH
KELKEIGINAYGHRHKIIKGVERLISGQQGLNPYLTLNTSSSGTLLIDLSSEDKEFQSVEEEMQSTVREHRDGGHAGGVFNRY
NILKIQKVCNKKLWERYTHRRKEVSEENHNHANERMLFHGSPFVNAIIHKGFDERHAYIGGMFGAGIYFAENSSKSNQYVYGI
GGGTGCPIHKDRSCYVCHRQLLFCRVTLGKSFLQFSAMKMAHSPPGHHSVTGRPSVNGLALAEYVIYRGEQAYPEYLITYQIV
KPEATTEA

>TNKS1[X.laevis][XP_018099068.1]
EFKGHSLLQAAREADLAKVKKTLALEIINFKQPQSHETALHCAVASLHPKRKQITELLLRKGASVNEKNKDFMTPLHVAAERA
HNDVVEVLHKHGAKMNALDTLGQTALHRAALGGHLQTCRLLLSFGSDASIVSLQGFTAAQMGNEAVQQILNESTPVRTSDVDY
RLLEASKAGDLDIVKQLCSSQNVNCRDLEGRHSTPLHFAAGYNRVSVVEYLLHHGADVHAKDKGGLVPLHNACSYGHYEVAEL
LVRHGASVNVADLWKFTPLHEAAAKGKYEICKLLLKHGADPTKKNRDGNTPLDLVKDGDTDIQDLLRGDAALLDAAKKGCLAR
VQKLCTQENINCRDTQGRNSTPLHLAAGYNNLEVAEYLLEHGADVNAQDKGGLIPLHNAASYGHVDIAALLIKYNTCVNATDK
WAFTPLHEAAQKGRTQLCALLLAHGADPTMKNQESQTPLDLATADDIRALLIDAMPPEALPSCFKPQATVVSASIISPPSTPS
CLSAASSIDNLTGPLAELAVGGVSNAGDGAAGTERKEGEVTVLDININQFLKSLGLEHLRDIFETEQITLDVLADMGHEELKE
IGINAYGHRHKLIKGVERLLGGQQGTNPYLTFHCVSQGTVLLDLASDDKEYQSVEEEMQNSIREHRDGGNAGGVFNRYNVIRI
QKVVNKKLRERFCHRQKEVSEENHNHHNERMLFHGSPFINAIIHKGFDERHAYIGGMFGAGIYFAENSSKSNQYVYGIGGGTG
CPTHKDRSCYICHRQMLFCRVTLGKSFLQFSTMKMAHAPPGHHSVIGRPSVNGLAYAEYVIYRGEQAYPEYLITYQIMKPETP
LQAATTAEQKT

>TNKS2[X.laevis][XP_018082988.1]
EFKGHSLLQAAREADLTRIKKHLSLEIVNFKHPQTHDTALHCAASSPYPKRKQVCELLFRKGANVNEKTKDFLTPLHIASDKA
HNDIVELLIKHEAKVNALDNLGQTSLHRAAHCGHLQTCRLLLNSGCDPSIVSLQGFTALQMGNESVQQLLQEGLPFSNSDADS
QLLEAAKAGDLDVVKKLCTSQSVNCRDVEGRQSTPLHFAAGYNRVAVVEYLLQHGADVHAKDKGGLVALHNACSYGHYEVTEL
LVKHGAVINVADLWKFTPLHEAAAKGKYEICKLLLLHGADSTKKNRDGNTPLDLVKDGDTDIQDLLRGDAALLDAAKKGCVSR
VKKLCTPENVNCRDTQGRHSTPLHLAAGYNNLEVAEYLLQHGADVNAADKGGLIPLHNAASYGHVDVAALLIKYNACVNANDK
WAFTPLHEAAQKGRTQLCALLLAHGADPALKNQEGQAPLDLVTADDVRALLTAAMPPAALPTFYKPQLLNVPQPSTTAIAALS
SVPLSSPTLSAASSLDNLASSFSETTSVSSGGAEGASGLDKKDEGVDLNINQFLRNLGLEHLIDVFEREQITLDVLIEMGHKE
FKEIGISAYGHRHKIIKGVERLISGQHGLNPYLTLNTSNSGTILIDLAPDDKEFLSVEEEMQSTVREHRDGGHAGGVFTKYNI
LKIQKVCNKKLWEKYTHRQKEVTEENHNHSNERMLFHGSPFVNAIIHKGFDERHAYIGGMFGAGIYFAENSSKSNQYVYGIGG
GTGCPIHKDRSCYVCHRQLLFCRVTLGKSFLQFSAMKMAHSPPGHHSVTGRPSVNGLALAEYVIYRGEQAYPEYIITYQIMKP
EAVAEG

>TNKS1[S.salar][XP_014017013.1]
EFKGHSLLQAAREADMAKVKKTLALEIISFKHPQTNETALHCAVASPHPKRKQVTELLLRKGANINEKNKDFMTALHVASERA
HNDILEVLQKHGAKVNAVDTLGQTALHRAALAGHIQTCRLLLSYGADPAIVSLQGFTASQMGNEAVQQILNENVPTRNSDVDY
RFLEAAKAGDLDTVQQLCTPQNVNCRDLEGRHSTPLHFAAGYNRVAVVEYLLHHGADVHAKDKGGLVPLHNACSYGHYEVAEL
LVRHGASVNVADLWKFTPLHEAAAKGKYEICKLLLKHGADPTKKNRDGNMPLDMVKDGDTDIQDLLRGDAALLDAAKKGCLAR
VQKLCSPENINCRDTQGRNSTPLHLAAGYNNLEVAEYLLEHGADVNAQDKGGLIPLHNAASYGHVDIAALLIKFNTCVNATDK
WAFTPLHEAAQKGRTQLCALLLAHGADPTMKNQEGQTALDLATADDIRALLMDAMPPDALPSCFKPQATVVSAGSVISPASTP
SCLSAASSIDNLAGPLNELGAAGTSGVADGATGSDRKEGELVMLDMNISQFLKSLGLEHLRDIFEREQISLDVLADMGHEELK
EIGINAYGHRHKLIKGVERLLGGQQGANPYLTFHCANQGTVLIDLAPDDKEGQSVEEEMQSTIREHRDGGNAGGVFSRYNIIK
IQKVVNKKLRERYTHRQKEISDENHNHHNERMLFHGSPFINAIIHKGFDERHAYIGGMFGAGIYFAENSSKSNQYVYGIGGGT
GCPTHKDRSCYLCHRQMLFCRVTLGKSFLQFSAMKMAHAPPGHHSVIGRPSVNGLAYAEYVIYRGEQAYPEYLITYQILKPES
TATSAAGEDQKS

>TNKS2[S.salar][XP_014034742.1]
EFRGHALLQAAREADLPRLKKHLSLETITFKHPLTHETALHCAATSPYPKRKQVCELLLRKGANVNEKTKDFLTPLHLASEKS
HNDIIEVLVKHEAKVNALDNLGQTALHRAAHCGHLQTCRLLLKAGCDPLVMSLQGFSPSQMGNESVQEILHEGTLIGNSDVDW
QLLEASKSGDLEIVKKLCTMQNVNCRDVEGRQSTPLHFAAGYNRVSVVEYLLLHGADVHAKDKGGLVPLHNACSYGHYEVAEL
LVIHGAVVNIADLWKFTPLHEAAAKGKYEICKLLLQHGADPTKKNRDGSTPLDLVKDGDTDMQDLLRGDAALLDAAKKGCLAR
VEKLCSPDNVNCRDAQGRHSTLLHLAAGYNNLEVAEYLLQHGAEVNSQDKGGLIPLHNAASYGHVDVAALLIKYDACVNATDK
WAFTPLHEAAQKGRTQLCALLLAHGADPALRNQEGQSPLDLVTVDDVRALLTAAMPPSALPGCYKPQVISMSSPVGVVVPPSL
SASSTPLSTLASRNSLDNQATASTCTAFPELPALLGPSGAVGTDNKEVPGVDLSIGQFLNNLGLEHLLEIFEREQITLDVLVE
MGHKELKEIGINAYGHRHKIIKGVERLISGPQSLNPYLTLNTANSGTILIDLVSDDKEFQLVEEELQSTIREHRDGGLAGGVF
NRYNIVKIQKVCNKKLWERYTHRRKEVSEENHNHSNERMLFHGSPFVNAIIHKGFDERHAYIGGMFGAGIYFAENSSKSNQYV
YGIGGGTGCPLHKDRSCYVCQRHLLFCRVTLGKSFLQFSAMKMAHSPPGHHSVTGRPSVNGLSLAEYVIYRGEQAYPEYLITY
QILKPDASVDG

>TNKS[P.marinus][XP_032806710.1]
EFRGHSLLQAAREADVTRVKKHLGLDIINFKHPQTHETALHCAVASPYPKRKQVSELLLRKGANVNEKTKDLLTPLHVASDKA
HNDVIDVLLKHGAKINVTDSLGQTSLHRAAHAGHLTTCRLLLSHGCDPNLPSLQGFTAAQLGNESVQQILHESSLPVRNSDVE
HQLLEAAKAGDLDTVKQLCTTQTVNCRDMEGRHSTPLHFAAGYNRVSVVEYLLQHGADVHAKDKGGLVPLHNACSYGHYEVAE
LLVKRGAVVNVADLWKFTPLHEAAAKGKYEICKLLLKHGADPTKKNRDGNTPLDLVKEGDTDIQDLLRGDAALLDAAKKGCLA
RVQKLCSGENVNCRDSQGRNSTPLHLAAGYNNLEVAEYLLEHGADVNAQDKGGLIPLHNAASYGHVDIAALLIKYSACVNATD
KWAFTPLHEAAQKGRTQLCALLLAHGADPSMKNQEGQTPLDLATADDVRALLIDAMPPEALPVTHKPPSIPTIAATSAAATAA
LALGPTPALASLAPSPVPSGASASGAGGVPLLASPSSTPASLSAASSMDSLTGACGVAPSPVPAGAIGAGAAGDGTLLGACGV
SAADGATGPADKPDRSADFEIPGLDMNINMFLKGLGLDHLRDIFEKEQITLDVLADMGHEELKEIGINAYGHRHKLIKGVERL
LGAQQGGLAYPGANPYLSYHTTAQGTLLIDLPPDDKEYQSVEDEMQSTIREHKDGGQAGGVFNRYNVIKIQKVRNKKLWERYT
HRRKEVTEENHNHHNERMLFHGSPFINAIINKGFDERHAYIGGMFGAGIYFAENSSKSNQYVYGIGGGTGCPVHKDRSCYICQ
RQMLFCRVTLGKSFLQFSAMKMAHSPPGHHSVIGRPSVNGLAFAEYVIYRGEQAYPEYLITYQILKPECTPDLTSAAGQKS

>TNKS[B.belcheri][XP_019641281.1]
EFKGHCLLDGARQTDMAKVKKHLQLDIVNFKHPYTHDTALHCAALSPYPKRRQITELLIRKGANLNDKNKEFLTPLHVASDKC
YIDVMEVLLKHGAKVNALDSLGQTALHRGAHCGHVQACRLLLSFGVDPSIVSLQGVTAAQMATEAVQQMLHEDPPSGPADECD
QLLEAAKAGDLEAVKVLCNPHTVNCRDVNGRHSTPLHFAAGYNRVAVVEYLLQHGADVHAKDKGGLVPLHNACSYGHYEVCEL
LLKHGAVVNVADLWKFTPLHEAAAKGKYEICKLLLKHGADPNKKNRDGNTPLDLVRDGDTDIQDLLRGDAALLDAAKKGNLAR
VQKLATPENINCRDTQGRNSTPLHLAAGYNNVEVAEFLLENGADVNAQDKGGLIPLHNASSYGHVEIAQLLIKYGTCVNATDR
WNFTPLHEAAQKGRTQLCALLLAHGADPTMKNQEGQTPLDLATAEDVRALLVDAMPPQSLPAAITAATVKAASPISSPSTTPS
STPGVLSTASSMDHLVGAVGGAGQGDGAIDRSAVEGESLLDMTISSFLQQLGLNQLLDIFNKEQITLDILGEMGHEELKEIGV
NAYGHRHKLIKGVERILGGTGTTLNPYLTPALGSHGTILTDLSPDDKEYQSVEEEVQSTIRQHKDQGQAGGIFNRYNVIKIQK
VRNKRLWDRYVHRRKEVSEENHNQSNERMLFHGSPFINAIINKGFDERHAYIGGMFGAGIYFAENSSKSNQYVYGIGGGTGCP
IHKDRSCYICQRQMLFCRVTLGKAFLQFSAMKMAHAPPGHHSVIGRPSVGGLNFAEYVIYRGEQAFPEYLITYQIVKPEAPAA
PSPDQK

>TNKS[D.melanogaster][NP_651410.1]
EYKGHCLLDACRKCDVSRAKKLVCAEIVNFVHPYTGDTPLHLAVVSPDGKRKQLMELLTRKGSLLNEKNKAFLTPLHLAAELL
HYDAMEVLLKQGAKVNALDSLGQTPLHRCARDEQAVRLLLSYAADTNIVSLEGLTAAQLASDSVLKLLKNPPDSETHLLEAAK

AGDLDTVRRIVLNNPISVNCRDLDGRHSTPLHFAAGFNRVPVVQFLLEHGAEVYAADKGGLVPLHNACSYGHYEVTELLVKHG
ANVNVSDLWKFTPLHEAAAKGKYDICKLLLKHGADPMKKNRDGATPADLVKESDHDVAELLRGPSALLDAAKKGNLARVQRLV
TPESINCRDAQGRNSTPLHLAAGYNNFECAEYLLENGADVNAQDKGGLIPLHNASSYGHLDIAALLIKHKTVVNATDKWGFTP
LHEAAQKGRTQLCSLLLAHGADAYMKNQEGQTPIELATADDVKCLLQDAMATSLSQQALSASTQSLTSSSPAPDATAAAAPGT
SSSSSSAILSPTTETVLLPTGASMILSVPVPLPLSSSTRISPAQGAEANGAEGSSSDDLLPDADTITNVSGFLSSQQLHHLIE
LFEREQITLDILAEMGHDDLKQVGVSAYGFRHKILKGIAQLRSTTGIGNNVNLCTLLVDLLPDDKEFVAVEEEMQATIREHRD
NGQAGGYFTRYNIIRVQKVQNRKLWERYAHRRQEIAEENFLQSNERMLFHGSPFINAIVQRGFDERHAYIGGMFGAGIYFAEH
SSKSNQYVYGIGGGIGCPSHKDKSCYVCPRQLLLCRVALGKSFLQYSAMKMAHAPPGHHSVVGRPSAGGLHFAEYVVYRGEQS
YPEYLITYQIVKPDDSSSGTEDTR

>TNKS[N.vectensis][XP_032220531.1]
EFKGHQILEAAKGCDTAKIKKLITTETINFQHPLTLDTPLHVAVASSSPKKKGVIETLLKKGADPNLQNKSSYAPLHVAAEKG
LFEAMELLLKHGAKVNAIDSAGQTALHVAAIAGQVQACRVLMSNGADPTCQTFQGYTAFEVAPEPVQKVLHVNEESPSPVSDA
EKQLLEAAKTGDLETVKNLCTAQTVNCRDLDGRCSTPLHFAAGYNRVRVVEYLLLNGADVHAKDKGGLVPLHNACSYGHYEVA
ELLVKHGAIVNVADLWKFTPLHEAAAKGKYEICQLLLKHGADPQKKNRDGYTPLDLVKEGDSDVADLLRGDAALLDAAKKGNL
IRITKLATPENINCRDTQGRNSTPLHLAAGYNHLEVAEYLLEHGADVNAQDKGGLIPLHNASSYGHVDIAALLIRYNTDVNAT
DRWLFTPLHEAAQKGRTQLCALLLAHGAEPSMKNQEGQSPVDLATAEDVKCLLGDAMLSTQTTSVSTAPVAKVPAPVTATAGA
ATPATGPISGMAPLSQANSSLLANSPAGHGDGAFSNRSVSEAQGEAVRSISAVHPGLDVDVGQFLDGLQLNNLKEIFEREQIS
WDVLVDMGHEELKEIGIHAYGHRHKILKAVKEKISGMGLGLGPFSTSQVQGSVIQELSMVDKDFASVADEMQSTIVEHRDSGT
AGGVFTSYTILKIERVVNTKLWEKYVYRRREIADSNHNHANERMLFHGSPFINAIVQKGFDQRHAYIGGMFGAGIYFAENSSK
SNQYVYGIGGGSGCPTHKDRSCYSCERQLLLCRVALGKPFYQFSAVKMAHAPPGHNSVIGRPSSGGLSFAEYVIYRGEQAYPE
YLITYKINKPDT

>TNKS[A.queenslandica][XP_019848937.1]
EYRGYQLLAAAEDGGIILLKKLLSSQLLKFQHHQTLDTLLHKAVLSKSSNRQSVIDALLKRGINLNIGNKENMTPLICAAKKG
ILEVVEQLVQRGANINHQDINGMTSLHWAVQNEHAQICRYLLSSGANPSIVNNQGQTIYQLKTSDTIQLILKNEPPVSQFEIE
QQLLEAARNSDLEILKKICTPQNVNCRDTKGRMSTPLHFAAGYNRVTVVEFLLENGADVHAKDKGGLVPLHNACSYGHYEVAE
LLVKYKANVNAMDLWKFTPLHEAAAKEKYDICKLLLKNGANVHSKNRDNLTPIDLVKDPKSDLADLLRGEPALLDAAKKGEIE
RVKKLLTEDNVNCRDEYGRNSTPLHLAAGYNHLDVVEYLLENKADVNAKDKGGLVPLHNASSYGHVDVASLLIRYNSVINATD
RWNFTPLHEAAQKGRTQVCSLLIIHGADVYLKNQEGQIPLDLATADDVIALLQDAMMKDIPLTIPPAEKEAKSNIVNKGLTAA
GASLLASELVLGDGVDDKNGMVTQQRGGPAGVGDGSDKGFMSYRGGGDAPPPWHNVTVKDILTELELGHLVELFEREQITIDI
LIEMNGDDLQSIGITAFGVRHRLLKRIRELVQGNNEEYPVGVTTTKPTQGTQLIELSSDDKEFIDTADLMQSTICEHRDDGKA
GGVFDSYEILKIERIVNTKVWERYKYRRKEVAESNNNCANELMLFHGSPFVPYIVHNGFDERHAYIGGMFGAGIYFAEHSSKS
NQYVYGIGGGNGCPEHKNRSCYTCLRKLLLCRVVLGKPVEQYTAVRIAHAPPGHHSVIGRPSAGGLNYPEYVIYRGEQAYPEY
IITFRIKKPSATDSMSSSSSLDMSNNT

>TNKS[S.macrocollata][GGOT01036632.1]
EAAGCVLLAALRTNDVSKIRRTLQPETANYCAHGTRQTPLHVAVQCARRAAVAAVRLLLEWKVDVNAQDARGRTPLHEACENG
NAEVVELLLAHNADHSLEDDKGHTAGVLALEHGQDDVVNVLRAHGAAVGVIDDPGRLRQVARLQSMMLRAAKDDDVDTMKRVL
QSPLVLQEPSLRQELLDCRDHEGRHSAPLHFAAGYNRVAACRFLLSEGATVHVRDKGGLVPLHNACSYGHHEIMKLLVEHHAS
VNEVDQWQYTPLHEAANKGKKDIVRFLLEHGADPNVKNSEGKRAVDLVPPELTDLKDMLSGDAALLEAAKLGQLDRVIKLCTP
TNVNCRDCEGRNSTALHLAAGYNQLSVVEYLLEHGADVNARDKGLLVPLHNAASYGHAEVAELLIGRGADVNATDRWGFTPLH
EAAQKGRTQLCTLLLNHGAMLHKCNQDGKTALDLATASDIRALLSDLSLHTPVPAAVVAKEGEPELARRMPAAGVSMVPAPDM
EGAQDHSTMEKWLHSLGLDEYTRLFEKERITFDVLVDMSHDDLEKIGVQAFGDRARLVKAVRQRATAIPASWLGEQKIVSLQA
SDIDFVQTSKMVLETIKRHGTQAGGVYRTFAITAIKRIQNIGLWKRYAFRRDEIEAENGGTANERLLFHGSPFVDSITQRGFD
ERHAYFGGMFGAGIYFAEHSSKSNQYVYGVGGGFGCAAHSDTSCYVCERQLLLCRVALGKSCVHTTAQRAAHAPPGHHSVIGE
PSKNGLQYPEYIIYRGEQAYPEFLIAYRLVKPS

>TNKS[S.kvevrii][GGOX01000296.1]
HVRGGKLLAAIRDQDLAAVRDLLAADPELLLSFASTNTHTTPLHIAAESTTPEAEGIFDLLVSAAPSTVLNAKDAAGRTCLHV
AALNSNLNGVKKLIAAGININLQDGQGATALLLSAEHKKHDIMRCLLRHGADPSVPNASGARAEDVASAAGRALLHGEVPDVE
LQVLEAAKTGNLAALNRLLDANNELVNCRDPEGRNSTALHFAAGFNRLAAAKLLISRGADVHCEDKGGLVPLHNACSYGHFEI
CKLLVESGASVNTADMWNFTPLHEAAHKGKLDIAKLLIEHGADLNKRNCDGKRPMDLVGDDQDLIDLLSGDAALLEAAKLGQL
DRVRKLVRPDNVNCRDAQGRNSTPLHFAAGYNQLAVVQCLIDAGADVNAQDRGGLIPLHNAASYGHNEVAEVLIDNGSEVNAK
DKWSFSPLHEAASKGRTQMCTLLLQHGADANSRNEDGKTPLQLATAEDVIVLLNAVSAQTRIATAPTAAAPRQSQLSAATASS
LQGASTQKTKQADPHLGFRSKTSDGAAESERAMLIGEDEDDVQHLPPPAEEVPVQEDPDGSILLKGFLEKLSLQSLYPLLSAV
PWTQLCTMGHIELEELGILDADHRSALVSSFWRDCAPRTLDAIVAPPQDWTDEELVSVPPDTPTYAGIEKRILSTIQQHEGAA
GGNYSTFKITSMQRIQNRGLWQQYAFRRQLVAMENDGDPGERLLFHGSPFAETIALRGFDERHAFFGGMFGAGIYFAQDSSKS
NQYVHGIGGGTGCKQHQNTSCYECERIMLVCRVTLGHACYQASSNKVAHAPPGHHSVVGKPTPGGLAFYEYVVYRGEQAYPAF
RVTYKLVPP
