# Supplementary material for: An Evolutionary Perspective on the Origin, Conservation and Binding Partner Acquisition of Tankyrases
Source: Biomolecules. 2022 Nov 15;12(11):1688. doi: 10.3390/biom12111688 (PMC9688111; doi:10.3390/biom12111688)
Supplement: Supplementary file 1 [file biomolecules-12-01688-s001.zip › biomolecules-1913719-supplementary-11.17 -done/Data_2/CytochromeC.pdf]

Cytochrome C

Sequences:

```
>NP_061820.1 cytochrome c [Homo sapiens]
MGDVEKGKKIFIMKCSQCHTVEKGGKHKTGPNLHGLFGRKTGQAPGYSYTAANKNKGIIWGEDTLMEYLE
NPKKYIPGTKMIFVGIKKKEERADLIAYLKKATNE

>NP_001124639.1 cytochrome c [Pongo abelii]
MGDVEKGKKIFIMKCSQCHTVEKGGKHKTGPNLHGLFGRKTGQAPGYSYTAANKNKGIIWGEDTLMEYLE
NPKKYIPGTKMIFVGIKKKEERADLIAYLKKATNE

>NP_031834.1 cytochrome c, somatic [Mus musculus]
MGDVEKGKKIFVQKCAQCHTVEKGGKHKTGPNLHGLFGRKTGQAAGFSYTDANKNKGITWGEDTLMEYLE
NPKKYIPGTKMIFAGIKKKGERADLIAYLKKATNE

>NP_001072946.1 cytochrome c [Gallus gallus]
MGDIEKGKKIFVQKCSQCHTVEKGGKHKTGPNLHGLFGRKTGQAEGFSYTDANKNKGITWGEDTLMEYLE
NPKKYIPGTKMIFAGIKKKSERVDLIAYLKDATSK

>NP_001085462.1 cytochrome c, somatic A [Xenopus laevis]
MGDAEKGKKIFIQKCAQCHTVEKTGKHKTGPNLWGLFGRKTGQAPGFSYTDANKSKGIVWGEDTLFEYLE
NPKKYIPGTKMIFAGIKKKNERLDLIAYLKKSTSE

>XP_014028790.1 cytochrome c [Salmo salar]
MGDIAKGKKAFVQKCAQCHTVENGGKHKVGPNLWGLFGRKTGQAEGYSYTDANKSKGIIWETDTLMTYLE
NPKKYIPGTKMIFAGIKKKGERADLIAYLKSATS

>XP_032813079.1 cytochrome c, somatic-like [Petromyzon marinus]
MADVEKGKKVFVQKCSQCHTVESGGKHKTGPNLWGLFGRKTGQAPGYSYTDANKSKGITWEEDTLYVYLE
NPKKYIPGTKMIFAGIKKKDERNNLIAYLKKSTSD

>XP_019613432.1 PREDICTED: cytochrome c, testis-specific [Branchiostoma belcheri]
MADAVKNGNPEKGKKVFVQKCAQCHTVEAGGKHKTGPNLNGLFGRKTGQAPGFSYTDANKNKGITWAEDT
LWIYLENPKKYIPGTKMVFAGLKKKGERADLISYLKESTSS

>NP_001285984.1 cytochrome c proximal, isoform B [Drosophila melanogaster]
MGVPAGDVEKGKKLFVQRCAQCHTVEAGGKHKVGPNLHGLIGRKTGQAAGFAYTDANKAKGITWNEDTLF
EYLENPKKYIPGTKMIFAGLKKPNERGDLIAYLKSATK

>XP_001635381.1 cytochrome c [Nematostella vectensis]
MGDATKGAKVFKTKCAQCHTVEAGGKHKTGPNLHGLFGRKTGQAEGFSYTTANQNKGITWGEDTLMVYLE
NPKKYIPGTKMVFAGIKKKNERLDLIAYLKQETS

>XP_004209990.1 cytochrome c [Hydra vulgaris]
MGAEKIPTGDATKGAKVFKQRCAQCHVIDQSGKHKTGPNLYGLIGRKTGQAPGFSYTQANQNKGITWNKD
TLWVYLENPSKYIPGTKMVFAGIKKGQERADLIAYLEEECKK

>XP_003388937.1 PREDICTED: cytochrome c-like [Amphimedon queenslandica]
MRSFLQRTLDSVVTKEKGVVTGYHCISLKAEEKEKALQRRSILQLLGKMSIPQGDPANGAKIFKQRCAQC
HTTEAGGKHKTGPNLHGLFGRKTGQAPGFTYTQANKSKGITWGEDTLFVYLEAPKKYIPGTKMVFAGLKK
PQERADLIAHLKEATK
```

Alignment

```
XP_003388937.1[A.queenslandica]      MRSFLQRTLDSVVTKEKGVVTGYHCISLKAEEKEKALQRRSILQLLGKMSIPQGDPANGA           60
XP_004209990.1[H.vulgaris]           ---------------------------------------------MGAEKIPTGDATKGA           15
NP_001285984.1[D.melanogaster]       ------------------------------------------------MGVPAGDVEKGK           12
XP_019613432.1[B.belcheri]           ----------------------------------------------MADAVKNGNPEKGK           14
XP_001635381.1[N.vectensis]          ----------------------------------------------------MGDATKGA           8
XP_032813079.1[P.marinus]            ----------------------------------------------------MADVEKGK           8
XP_014028790.1[S.salar]              ----------------------------------------------------MGDIAKGK           8
NP_001085462.1[X.laevis]             ----------------------------------------------------MGDAEKGK           8
NP_061820.1[H.sapiens]               ----------------------------------------------------MGDVEKGK           8
NP_001124639.1[P.abelii]             ----------------------------------------------------MGDVEKGK           8
NP_031834.1[M.musculus]              ----------------------------------------------------MGDVEKGK           8
NP_001072946.1[G.gallus]             ----------------------------------------------------MGDIEKGK           8
                                                                                          .:  :*

XP_003388937.1[A.queenslandica]      KIFKQRCAQCHTTEAGGKHKTGPNLHGLFGRKTGQAPGFTYTQANKSKGITWGEDTLFVY           120
XP_004209990.1[H.vulgaris]           KVFKQRCAQCHVIDQSGKHKTGPNLYGLIGRKTGQAPGFSYTQANQNKGITWNKDTLWVY           75
NP_001285984.1[D.melanogaster]       KLFVQRCAQCHTVEAGGKHKVGPNLHGLIGRKTGQAAGFAYTDANKAKGITWNEDTLFEY           72
XP_019613432.1[B.belcheri]           KVFVQKCAQCHTVEAGGKHKTGPNLNGLFGRKTGQAPGFSYTDANKNKGITWAEDTLWIY           74
XP_001635381.1[N.vectensis]          KVFKTKCAQCHTVEAGGKHKTGPNLHGLFGRKTGQAEGFSYTTANQNKGITWGEDTLMVY           68
XP_032813079.1[P.marinus]            KVFVQKCSQCHTVESGGKHKTGPNLWGLFGRKTGQAPGYSYTDANKSKGITWEEDTLYVY           68
XP_014028790.1[S.salar]              KAFVQKCAQCHTVENGGKHKVGPNLWGLFGRKTGQAEGYSYTDANKSKGIIWETDTLMTY           68
NP_001085462.1[X.laevis]             KIFIQKCAQCHTVEKTGKHKTGPNLWGLFGRKTGQAPGFSYTDANKSKGIVWGEDTLFEY           68
NP_061820.1[H.sapiens]               KIFIMKCSQCHTVEKGGKHKTGPNLHGLFGRKTGQAPGYSYTAANKNKGIIWGEDTLMEY           68
NP_001124639.1[P.abelii]             KIFIMKCSQCHTVEKGGKHKTGPNLHGLFGRKTGQAPGYSYTAANKNKGIIWGEDTLMEY           68
NP_031834.1[M.musculus]              KIFVQKCAQCHTVEKGGKHKTGPNLHGLFGRKTGQAAGFSYTDANKNKGITWGEDTLMEY           68
NP_001072946.1[G.gallus]             KIFVQKCSQCHTVEKGGKHKTGPNLHGLFGRKTGQAEGFSYTDANKNKGITWGEDTLMEY           68
                                     * *  :*:***. :  ****.**** **:******* *::** **: *** *  ***  *

XP_003388937.1[A.queenslandica]      LEAPKKYIPGTKMVFAGLKKPQERADLIAHLKEATK-          156
XP_004209990.1[H.vulgaris]           LENPSKYIPGTKMVFAGIKKGQERADLIAYLEEECKK          112
NP_001285984.1[D.melanogaster]       LENPKKYIPGTKMIFAGLKKPNERGDLIAYLKSATK-          108
XP_019613432.1[B.belcheri]           LENPKKYIPGTKMVFAGLKKKGERADLISYLKESTSS          111
XP_001635381.1[N.vectensis]          LENPKKYIPGTKMVFAGIKKKNERLDLIAYLKQETS-          104
XP_032813079.1[P.marinus]            LENPKKYIPGTKMIFAGIKKKDERNNLIAYLKKSTSD          105
XP_014028790.1[S.salar]              LENPKKYIPGTKMIFAGIKKKGERADLIAYLKSATS-          104
NP_001085462.1[X.laevis]             LENPKKYIPGTKMIFAGIKKKNERLDLIAYLKKSTSE          105
NP_061820.1[H.sapiens]               LENPKKYIPGTKMIFVGIKKKEERADLIAYLKKATNE          105
NP_001124639.1[P.abelii]             LENPKKYIPGTKMIFVGIKKKEERADLIAYLKKATNE          105
NP_031834.1[M.musculus]              LENPKKYIPGTKMIFAGIKKKGERADLIAYLKKATNE          105
NP_001072946.1[G.gallus]             LENPKKYIPGTKMIFAGIKKKSERVDLIAYLKDATSK          105
                                     ** *.********:*.*:**  ** :**::*:.  .
```

Percent identity matrix:

```
#
#
#  Percent Identity  Matrix - created by Clustal2.1
#
#
```

1: XP_003388937.1[A.queenslandica]	100.00	72.97	77.78	73.64	77.88	72.12	73.08	75.96	74.04	74.04	77.88	75.96
2: XP_004209990.1[H.vulgaris]	72.97	100.00	68.52	69.37	77.88	67.62	67.31	69.52	66.67	66.67	70.48	69.52
3: NP_001285984.1[D.melanogaster]	77.78	68.52	100.00	76.85	75.96	76.92	78.85	79.81	76.92	76.92	84.62	81.73
4: XP_019613432.1[B.belcheri]	73.64	69.37	76.85	100.00	80.77	80.95	79.81	80.95	77.14	77.14	83.81	81.90
5: XP_001635381.1[N.vectensis]	77.88	77.88	75.96	80.77	100.00	78.85	78.85	82.69	79.81	79.81	83.65	84.62
6: XP_032813079.1[P.marinus]	72.12	67.62	76.92	80.95	78.85	100.00	83.65	84.76	81.90	81.90	83.81	83.81
7: XP_014028790.1[S.salar]	73.08	67.31	78.85	79.81	78.85	83.65	100.00	82.69	81.73	81.73	85.58	85.58
8: NP_001085462.1[X.laevis]	75.96	69.52	79.81	80.95	82.69	84.76	82.69	100.00	85.71	85.71	88.57	86.67
9: NP_061820.1[H.sapiens]	74.04	66.67	76.92	77.14	79.81	81.90	81.73	85.71	100.00	100.00	91.43	87.62
10: NP_001124639.1[P.abelii]	74.04	66.67	76.92	77.14	79.81	81.90	81.73	85.71	100.00	100.00	91.43	87.62
11: NP_031834.1[M.musculus]	77.88	70.48	84.62	83.81	83.65	83.81	85.58	88.57	91.43	91.43	100.00	92.38
12: NP_001072946.1[G.gallus]	75.96	69.52	81.73	81.90	84.62	83.81	85.58	86.67	87.62	87.62	92.38	100.00
