# Supplementary material for: An Evolutionary Perspective on the Origin, Conservation and Binding Partner Acquisition of Tankyrases
Source: Biomolecules. 2022 Nov 15;12(11):1688. doi: 10.3390/biom12111688 (PMC9688111; doi:10.3390/biom12111688)
Supplement: Supplementary file 1 [file biomolecules-12-01688-s001.zip › biomolecules-1913719-supplementary-11.17 -done/Data_2/PARP1.pdf]

PARP1

Sequences:

```
>NP_001609.2 poly [ADP-ribose] polymerase 1 [Homo sapiens]
MAESSDKLYRVEYAKSGRASCKKCSESIPKDSLRMAIMVQSPMFDGKVPHWYHFSCFWKVGHSIRHPDVE
VDGFSELRWDDQQKVKKTAEAGGVTGKGQDGIGSKAEKTLGDFAAEYAKSNRSTCKGCMEKIEKGQVRLS
KKMVDPEKPQLGMIDRWYHPGCFVKNREELGFRPEYSASQLKGFSLLATEDKEALKKQLPGVKSEGKRKG
DEVDGVDEVAKKKSKKEKDKDSKLEKALKAQNDLIWNIKDELKKVCSTNDLKELLIFNKQQVPSGESAIL
DRVADGMVFGALLPCEECSGQLVFKSDAYYCTGDVTAWTKCMVKTQTPNRKEWVTPKEFREISYLKKLKV
KKQDRIFPPETSASVAATPPPSTASAPAAVNSSASADKPLSNMKILTLGKLSRNKDEVKAMIEKLGGKLT
GTANKASLCISTKKEVEKMNKKMEEVKEANIRVVSEDFLQDVSASTKSLQELFLAHILSPWGAEVKAEPV
EVVAPRGKSGAALSKKSKGQVKEEGINKSEKRMKLTLKGGAAVDPDSGLEHSAHVLEKGGKVFSATLGLV
DIVKGTNSYYKLQLLEDDKENRYWIFRSWGRVGTVIGSNKLEQMPSKEDAIEHFMKLYEEKTGNAWHSKN
FTKYPKKFYPLEIDYGQDEEAVKKLTVNPGTKSKLPKPVQDLIKMIFDVESMKKAMVEYEIDLQKMPLGK
LSKRQIQAAYSILSEVQQAVSQGSSDSQILDLSNRFYTLIPHDFGMKKPPLLNNADSVQAKVEMLDNLLD
IEVAYSLLRGGSDDSSKDPIDVNYEKLKTDIKVVDRDSEEAEIIRKYVKNTHATTHNAYDLEVIDIFKIE
REGECQRYKPFKQLHNRRLLWHGSRTTNFAGILSQGLRIAPPEAPVTGYMFGKGIYFADMVSKSANYCHT
SQGDPIGLILLGEVALGNMYELKHASHISKLPKGKHSVKGLGKTTPDPSANISLDGVDVPLGTGISSGVN
DTSLLYNEYIVYDIAQVNLKYLLKLKFNFKTSLW

>XP_024101750.1 poly [ADP-ribose] polymerase 1 [Pongo abelii]
MAESSDKLYRVEYAKSGRASCKKCSESIPKDSLRMAIMVQSPMFDGKVPHWYHFSCFWKVGHSIRHPDVE
VDGFSELRWDDQQKVKKTAEAGGVTGKGQDGIGSKAEKTLGDFAAEYAKSNRSTCKGCMEKIEKGQVRLS
KKMLDPEKPQLGMIDRWYHPGCFVKNREELGFRPEYSASQLKGFSLLATEDKEALKKQLPGVKSEGKRKG
DEVDGVDEVAKKKSKKEKDKDSKLEKALKAQNDLIWNIKDELKKVCSTNDLKELLIFNKQQVPSGESAIL
DRVADGMVFGALLPCEECSGQLVFKSDAYYCTGDVTAWTKCMVKTQTPNRKEWVTPKEFREISYLKKLKV
KKQDRIFPPETSAPVAATPPPSTASAPAAVNSSASADKPLSNMKILTLGKLSRNKDEVKAMIEKLGGKLT
GTANKASLCISTKKEVEKMNKKMEEVKEANIRVVSEDFLQDVSASTKSLQELFLAHILSPWGAEVKAEPV
EVVAPRGKSGAALSKKSKGQVKEEGINKSEKRMKLTLKGGAAVDPDSGLEHSAHVLEKGGKVFSATLGLV
DIVKGTNSYYKLQLLEDDKENRYWIFRSWGRVGTVIGSNKLEQMPSKEDAIEHFMKLYEEKTGNAWHSKN
FTKYPKKFYPLEIDYGQDEEAVKKLTVNPGTKSKLPKPVQDLIKMIFDVESMKKAMVEYEIDLQKMPLGK
LSKRQIQAAYSILSEVQQAVSQGSSDSQILDLSNRFYTLIPHDFGMRKPPLLNNADSVQAKVEMLDNLLD
IEVAYSLLRGGSDDSSKDPIDVNYEKLKTDIKVVDRDSEEAEIIRKYVKNTHATTHNAYDLEVIDIFKIE
REGECQRYKPFKQLHNRRLLWHGSRTTNFAGILSQGLRIAPPEAPVTGYMFGKGIYFADMVSKSANYCHT
SQGDPIGLILLGEVALGNMYELKHASHISKLPKGKHSVKGLGKTTPDPSASISLDGVEVPLGTGISSGVN
DTCLLYNEYIVYDIAQVNLKYLLKLKFNFKTSLW

>BAE39743.1 unnamed protein product [Mus musculus]
MAEASERLYRVEYAKSGRASCKKCSESIPKDSLRMAIMVQSPMFDGKVPHWYHFSCFWKVGHSIRQPDVE
VDGFSELRWDDQQKVKKTAEAGGVAGKGQDGSGGKAEKTLGDFLAEYAKSNRSMCKGCLEKIEKGQMRLS
KKMVDPEKPQLGMIDRWYHPTCFVKKRDELGFRPEYSASQLKGFSLLSAEDKEALKKQLPAIKNEGKRKG
DEVDGTDEVAKKKSKKGKDKDSSKLEKALKAQNELIWNIKDELKKACSTNDLKELLIFNQQQVPSGESAI
LDRVADGMAFGALLPCKECSGQLVFKSDAYYCTGDVTAWTKCMVKTQNPSRKEWVTPKEFREISYLKKLK
VKKQDRIFPPESSAPAPLALPLSVTSAPTAVNSSAPADKPLSNMKILTLGKLSQNKDEAKAVIEKLGGKL
TGSANKASLCISTKKEVEKMSKKMEEVKAANVRVVCEDFLQDVSASTKSLQELLSAHSLSSWGAEVKAEP
GEVVASKGKSAAPSKKSKGAVKEEGVNKSEKRMKLTLKGGAAVDPDSGLEHSAHVLEKGGKVFSATLGLV
DIVKGTNSYYKLQLLEDDKESRYWIFRSWGRVGTVIGSNKLEQMPSKEDAVEHFMKLYEEKTGNAWHSKN
FTKYPKKFYPLEIDYGQDEEAVKKLTVKPGTKSKLPKPVQELVGMIFDVESMKKALVEYEIDLQKMPLGK
LSRRQIQAAYSILSEVQQAVSQGSSESQILDLSNRFYTLIPHDFGMKKPPLLNNADSVQAKVEMLDNLLD
IEVAYSLLRGGSDDSSKDPIDVNYEKLKTDIKVVDRDSEEAEVIRKYVKNTHATTHNAYDLEVIDIFKIE
REGESQRYKPFRQLHNRRLLWHGSRTTNFAGILSQGLRIAPPEAPVTGYMFGKGIYFADMVSKSANYCHT
SQGDPIGLILLGEVALGNMYELKHASHISKLPKGKHSVKGLGKTTPDPSASITLEGVEVPLGTGIPSGVN
DTCLLYNEYIVYDIAQVNLKYLLKLKFNFKTSLW

>P26446.2 [Gallus gallus]
MAETGDKPYRAEYAKSGRASCKKCGESIAKDSLRLALMVQSPMFDGKVPHWHHYSCFWKRARIVSHTDID
GFPELRWEDQEKIKKAIETGALQEEKGGTRKEVGKAEKSLTDFAAEYAKSNRSTCKGCEQKIEKGQIRIS
KKMVHPEKPQLGMIDNWYHPDCFVSRRAELGFLPAYGATQLLGFSILKAEDKETLKKQLPATKTEGKRKG
EEVDGNVVAKKKSRKEKEKESKQEKQLKEQTELIWGIKDELRKVCSTNDLKELLIANKQEVPSGENAILD
RVADGMAFGALLPCEECKGQFVFKSDAYYCSGDITAWTKCVAKTQTPNRKDWVIPKEFREIPYLKKFKCK
KQDRIFPPEAATVNSAPPPPASAPLTETVTAPQDKPLTNMKILTLGKLSKNKEEVKNIVEELGGKMTTTA
NKATLCISTQKEVEKMSKKMEEVKDAKVRVVSEEFLKDVKSSNKGFQELLSLHAISPWGAEVKTEHQEVA
VDGKCSKPANMKSAGKVKEEQGPSKSEKKMKLTVKGGAAVDPDSGLEDSAHVFEKGGKIFSATLGLVDIV
KGTNSYYKLQLLEDDRESRYWVFRSWGRVGTVIGSNKLEQMPSKEDAVEHFLNLYEEKTGNSWHSKNFTK
YPKKFYPLEIDYGQDEEAVRKLTVSAGTKSKLAKPIQDLIKMIFDVESMKKAMVEFEIDLQKMPLGKLSK
RQIQSAYSILNEVQQAVSDGGSESQILDLSNRFYTLIPHDFGMKKPPLLSNLEYIQAKVQMLDNLLDIEV
AYSLLRGGNEDGDKDPIDINYEKLRTDIKVVDKDSEEAKIIKQYVKNTHAATHNAYDLKVVEIFRIEREG
ESQRYKPFKQLHNRQLLWHGSRTTNFAGILSQGLRIAPPEAPVTGYMFGKGIYFADMVSKSANYCHTSQA
DPIGLILLGEVALGNMYELKNASHITKLPKGKHSVKGLGKTAPDPTATTTLDGVEVPLGNGISTGINDTC
LLYNEYIVYDVAQVNLKYLLKLKFNYKTSLW

>NP_001081571.1 poly [ADP-ribose] polymerase 1 [Xenopus laevis]
MAEASDKLYRAEYAKSGRASCKKCGDNIAKESLRLAIMVQSPMFDGKVPHWHHYSCFWKRARVLSQGDIY
GYTELRWEDQEMIKKAIETGGAAAGAGGDSKGGKGEMTLNDFAAEYAKSNRSACKGCEQKIEKGQIRISK
KSVDVERPQLGMIDRWYHPDCFVSSREELGFLPSYSASQLKGFTILSAEDKDSLKKKLPAVKNEGKRKAD
EVDGHSAATKKKIKKEKEKESKLEKLLKEQTELIWHIKDELKKVCSTNDLKELLIANKQQVPSGETNIVD
RVSDGMAFGALLPCEECSGQFVFKGDAYYCTGDLSAWTKCVAKTQTPNRKDWVTPKEFHEIPYLKKFKFK
RHDRAFPPCAAPTPISPPAAPEPKPTVEETFPEGKPLTNTKVLLIGKLSKNKDEVKTLIEGLGGKVAGSA
HKANLCISTNKEVEKMSKKMEEVKAANVRVVSDDFLKEVESGKSVQELLSQFGISSWGAEIKQEAVQPTE
KQPSSGPVAGKSSGKVKEEKGSNKSEKKMKLTVKGGAAIDPDSELEDSCHVLETGGKIFSATLGLVDITR
GTNSYYKLQLIEHDRDSRYWVFRSWGRVGTVIGSKKLEEMSSKEDAIEHFLNLYQDKTGNAWHSPNFTKY
PKKFYPLEIDYGQEEDVVKKLSVGAGTKSKLAKPVQELIKLIFDVESMKKAMVEFEIDLQKMPLGKLSKR
QIQSAYSILSQVQQAVSESLSEARLLDLSNQFYTLIPHDFGMKKPPLLNNLEYIQAKVQMLDNLLDIEVA
YSLLRGGADDGEKDPIDVKYEKIKTDIKVVAKDSEESRIICDYVKNTHADTHNAYDLEVLEIFKIDREGE
YQRYKPFKQLHNRQLLWHGSRTTNFAGILSQGLRIAPPEAPVTGYMFGKGIYFADMVSKSANYCHAMPGS
PIGLILLGEVALGNMHELKAASQITKLPKGKHSVKGLGRTAPDPSATVQLDGVDVPLGKGTSANISDTSL
LYNEYIVYDIAQVNLKYLLKLKFNYKGGMMW

>XP_014061166.1 poly [ADP-ribose] polymerase 1 isoform X1 [Salmo salar]
MADAQEDKLYKAEYAKSGRASCKKCKENIAKDSLRMAIMVQSPMFDGKVPHWHHFSCFWLRASAQSPADI
AGFSDLRWEDQEKVKKAIETGGAAGGKGDVKGGAKGEKTLNDFAVEYAKSNRSTCKGCEQKIEKDQIRVS
KKTVDPEKPQLGLIDRWYHTACFVGRREELVFKPEYSAAQLKGFNTLRVEDKEELKRRLPAVKSKGKRKT
DEVDGEGASKKKKDEEEEKKKVELQMKEQSQLIWGIKDKLRQFCSTNDMKELLIANGQEVPSGESNVVDS
LGDCMAFGALKPCEECQGQLVFKSDAYYCTGDLSAWTKCVFKTQLPNRKDWVIPKEFHELPFLKKFKFKR
QDRVFPKVAPAPPPASSAAVPCASSAPSIPLPEGAPADKPLTGMKLLTVGKLRQNKDELKAAVEELGGKI
TGTANKATLCLSTKKEVEKMSKKMEEAEEVGVRVVSEDFLTDIKSSGKALQELVSLHAISPWGAEVKLET
QAQSAASRSGAMAGKSTKRVKEEEGVSKASKKMKLTVKGGAAVDPDSGLENSAHVLDQNGKIFSATLGLV
DIVRGTNSYYKLQLLEDDVHKRYWVFRSWGRVGTTIGGKKLDKFNDKNSAMDNFLGVYEEKTGNAWGSSH
FTKYPNRFYPLEIDYGQDEEAVKRLTASAGTNSLLAKPVQELIRTIFDVESMKKAMVEFEIDLQKMPLGK
LSKRQIQSAYALLSEVQQAVSDSSSESHILDLSNRFYTLIPHDFGMKKPPLLRNLDYIQAKVQMLDNLLD
IEVAYSLLKGGAEDNKKDPIDINYEKLKTKIEVVDKTTKEAEIILQYVKNTHAATHNTYTLEVEEIFKIV
REGEYQKYRPFQDLPNRQLLWHGSRATNYAGILSQGLRIAPPEAPVTGYMFGKGVYFADMVSKSANYCHT
SQSDPTGLLLLGEVALGNMHELKKASHITKLPKGKHSVKGLGRNAPDPSATVTLDGVQVPLGKGTNTNID
DTSLLYNEYIVYDVAQVNLKYLLKIKFNYQMSMW

>XP_032819895.1 poly [ADP-ribose] polymerase 1 [Petromyzon marinus]
MADKPFKAEYAKSSRASCKKCGGNIEKDCLRLAVMVQSPRFDGKVPQWHHFQCFWKRARPLTQADVDGFS
```

ELRSADQDRIKKHIEGSADGEGKSSGKANGGAAASNTLPDFLVEYAKSNRSKCKGCDDKIEKDDVRISRK
MINIEKPHLGMIDHWYHTNCFVEHRDTLGFLSTYGANQIGGFTRLNANDKKAVETLLGTVVVDGKRKAED
QVDEVAKKIKLESMQQKEEEEKMLKKQSNVIWSFKDGLKSCSDEIDLKELLIANKQAVPSGESAVLERLA
DGMAFGALLPCAECKDGQLVCMSDAYRCSGNISGWTKCTFSTKTPKRNEWIIPKEFKDVPFLKKYKYKQR
ERVFMSAQATTSPPRAATVATATVATAATKSGEAPGTSAGAVPGKLLAGKALCLVGKLQKSRDDIKALVE
KLGGKMTSTVPKADFCISTKKEVEKMSKKIAEAQTCNVCVVAEDLLTEASGRPLAELVSAHRLSTWGAPI
KVEQEPTVKTEPVLEVPKEASAKSSKRKSEGQKAEANKLKLTVKGRAAVDPLSNLVDVAHVLEDRGMIYG
AAMSLVDVERGTNSYYKLQLLECDLNRKYWVFRSWGRLGTTIGGTKLEKMKSREDAVEHFCQLYYDKTGN
KWNARNFVKYPNKFYPVETDYGQDDEDIHDLKAMAGTNSKLAKPIQELLRMIFDIDSMKRTMVEFEIDLC
KMPLGKLSRKQIQSAYSVLGDAQKLVSESGLTSLIQDLSNKFYTLIPHDFGMKKPTLLDNLDYIKMKVDM
LDNLMDIEVAYSLLNPKDETSGGKKDLLDASYEKLHTTIEVLGKDTEEFRVILEYVTNTHAHTHDAYELE
VEEVFKVSRDGEFQRYRPFRELHNRQLLWHGSRMTNYAGILSKGLRIAPKEAPVTGYMFGKGVYFADMVS
KSANYCHTSPSNPTGLILLAEVALGNMHELKQSESITKLPKGTHSVKGVGKTAPDPKKTVTLNGVQVPIG
NPSATGINDTSLLYNEYIVYDVAQVNIKYLLKLNFKYKMQLW

>XP_019618726.1 PREDICTED: poly [ADP-ribose] polymerase 1-like isoform X1 [Branchiostoma belcheri]
MLAYRVQEAAKISMADDEGKAYKAEYAKSNRASCKLCKGNITKDSLRLARMVQSPHFDGKVPNWFHYSCF
FKKCKPSSTVEFSGVTGLRWDDQEKLKKTIGGEGSSAGADAGGDEVDAPGVQVFSVEYAKSNRSACRGCS
EKIDKGLVRISKKVDEGDQWGPKDLWHHVDCFVEKREELGFTTDMAPSVIQGYKKLSKDDQDILVKKLGS
GTTGKRKAKGSNGAAAKKVKAEETEEEKKLKEQSKLVWKIRDELSKSMENSDLKELLLANDQDIPSGESA
LLDRLSDGMAFGALQRCPECKHGQLFYRSDGYHCSGNLTEWTKCIYTTREPKRKKWIIPDDLKEEVPFLK
KFKSKVAARVFSAAHVAAASESTDSFSSSSRADEKKPLHHVKVVLGKTTKSKAEMTKAIEKLGGTVASKV
DSTVACVISSKEDVQKMSKKIKDAKAADVHVVSEDFVTDVEKGGAALLIMQKSIASWGSDPHSRIATVEE
KPSKSKSKSKYEEMESGTKKLKMMVKGGAAVDPDSGIEHSAHVLEDKGKVYNAVLGLVDLVRGTNSYYKL
QVLEADKGNRWYVFRAWGRVGTTVGGNKLETFHSRQGALEQFLNLYEEKTGNEFGTKNFVKYPKRFYPLD
IDYGQEEEDLQKLKIKPGSKSTLAREVQEIIQMIFDIESMKKAMVEFEIDLKKMPLGKLSRKQIESAYSV
LTELNGILTGERSATRILDASNRFYTLIPHDFGMKKPPMLDNEEVIKAKTTMLDNLLEIEVAYNLLKSGD
DGEGKDPIDAHYEKLKCKMEVVEKASDEFAMVQEYVKNTHAKTHSHYTLEVEELFKIAREGEASRYRPFQ
QLHNRQLLWHGSRVTNYAGILSQGLRIAPPEAPVTGYMFGKGLYFADMVSKSANYCATSTASPTGLLLLC
EVALGNMYERKHAEYVSKLPKGMHSTKGLGATGPDPGATKTLPNGTQVPIGHGVPSGVSGSSLLYNEYIV
YDVAQVEMKYLIRMKFNYKSLW

>NP_001104452.1 Poly-(ADP-ribose) polymerase [Drosophila melanogaster]
MDIELPYLAEYARTGRATCKGCKSTISKDTLRIAVMVQSAFHDAKVPNWFHKTCFFKNQRPSSVGDIQNI
GNLRFADQKELTDLVENIQEVISAQLGKKRSKAFNLALKDFGIEYAKSSRSTCRGCEQKINKDLVRLRKT
VYDTEVGMKYGGQPLWHHLECFAQLRSELGWFASGEDMPGFQSLADDDQAKVKNAIPPIKSEELPDTKRA
KMELSDTNEEGEKKQRLKDQNDAYFRFRDDIKNKMKKKDIDILLKFNNQQPVTGDTEKLFDQTADLLTFG
AIESCSECNSCQFIVNKSGYICNGNHSEWTKCNKLLKEPTRSACIVPKELKALYNFLNTVKEIPSTRIFN
NFPPNKSTFSRSLLKTNKNNDVLVRPTIPRISPPLYNLKFSIIGLKNQHKELRKRIENLGGKFEVKISEN
TIAIISTELEIQKKSTRMKFAEELGIHIVPIEFLDFVEADTEGAIKYINSTCICSWGTDPKSRIPKETTK
SLNSNSIYTKSMPVSRTFKVKDGLAVDPDSGLEDIAHVYVDSNNKYSVVLGLTDIQRNKNSYYKVQLLKA
DKKEKYWIFRSWGRIGTNIGNSKLEEFDTSESAKRNFKEIYADKTGNEYEQRDNFVKRTGRMYPIEIQYD
DDQKLVKHESHFFTSKLEISVQNLIKLIFDIDSMNKTLMEFHIDMDKMPLGKLSAHQIQSAYRVVKEIYN
VLECGSNTAKLIDATNRFYTLIPHNFGVQLPTLIETHQQIEDLRQMLDSLAEIEVAYSIIKSEDVSDACN
PLDNHYAQIKTQLVALDKNSEEFSILSQYVKNTHASTHKSYDLKIVDVFKVSRQGEARRFKPFKKLHNRK
LLWHGSRLTNFVGILSHGLRIAPPEAPPTGYMFGKGIYFADMVSKSANYCCTSQQNSTGLMLLSEVALGD
MMECTSAKYINKLSNNKHSCFGRGRTMPDPTKSYIRSDGVEIPYGETITDEHLKSSLLYNEYIVYDVAQV
NIQYLFRMEFKYSY

>XP_032219514.1 poly [ADP-ribose] polymerase 1 isoform X1 [Nematostella vectensis]
MSENDVPFKTEYAKSNRSSCKSCKSNIGKDSLRVARMVQSPHFDGKVPNWFHFSCFFKKQFKPKSLAEIA
GIDGLRWDDQEKFRAQVYGGASDVPDTTAAATVSVEQPDLLAEYAKSSRSTCKHCDEQIVKGELRLAKVM
DGEKYGPVPKWHHVPCFLKAMPDLDISGILTAQHFTGFQKLGKDDQKLLIDKFGSQKQISKKKGKKKSLE
ASAAMPVKSASEEKEEQQLKEQSEAIWKIRDELKTNCSLSELREMLEDNEINPQGGETSVFDRCSDGMLF
GRLLPCHECHGGMLVYRSDGYHCTGNVSGWTKCAFTTQDPKRTKWVISKELKKTNDFLKTFKCKEMKRLF
PKLTAIQPGAIPGKPLSNMMVAVIGKLKRTKADVASCVAQLGGTTMDRVTSKVDCCISTQAEVSKSSKKM
KDAEKFNIPVVSEEFLDAIKDGEFNSNIAKHSLVSWGKIKALDIVDGPLPLRRKASDSKYELGASQPKKV
KLSVKGGAAVDPASEMEDDCHVLEVKGVVYSATLGMVDISRGTNSYYKLQVLKKDKSSRCYLFRSWGRVG
TTIGGSKLEDCGNSHNSAVQSFEELYREKTGNEWSERDNFVKHPNRFYPLEIDYGQAHEDVVKAKIEMGS
KSKLPSQIQELIKMIFDVESMKKTLIEFEIDLKKMPLGKLTKRQIEKAYSVLGEAQQILSEAASKTAVLD
VSNRFYTLIPHDFGMRKPPLLDNQELIKTKIEMLDNLIDIEVAFSLLQSSDGEDAEDPIDINYKKLKTDI
EVLDHKSAEFGLISEYVSNTHADTHRQYELELLDVFKIKREGEEALFKPFKQLHNRQLLWHGSRTTNYAG
ILSQGLRIAPPEAPVTGYMFGKGVYFADMVSKSANYCCTSPSNNIGLLLLCEVALGNMHELKHASFIKKV
PKGKHSVKGLGKTAPDPSATHTFEDGTIVPKGKGCPAPVKDSSLLYNEYIVYDTAQINMKYLLKTKFKYK
YGW

>XP_047140981.1 poly [ADP-ribose] polymerase 1 [Hydra vulgaris]
MSEDLPYNAEYAKSGRAGCKSCKCNIGKDSLRIAKMVQSPHFDGKIPQWYHYSCFFKKFKPLNVSDIKNF
DSLRWDDQEKLREQIGCPQKKDVVDGPSNSTVNKDFCIEYAKSGRAKCRKCEEKIEKDIIRIAKIMESEE
SKFKGLIPFWYHVKCFALSKSELEAQNLTSNEIDGWGELSEEDQEKVGLEIKFKKHGKLKIKKGNKINIK
DECKDEEKKEIKDEKVEKFKLQNKEYWDMRDKLQKNVPNNVLRKLLDANNYNTTGGENTLLERCADGICF
GALLTCSECKTGNLVYRTEGYQCTGHVSGWTSCTFLTQTPKRKKWHIPKELLLENDFLSTIKPKVEERTF
MTTTKMNTDIKESQKRPLENRKVIVIGKLKESKGVIENKIQLLGGTLSAAVSKSCYCCISTQDEIEKSTK
KMQTVKTYNIPVVTENFLDAVQNGEIDALMKNSIAPWGTNREMLDDKMDNFKSMKRKTSSSIESSKKAKV
IMKGGAVVDPDSGLEGKCHILSVRGELFTAVLGLVDLLRGTNSYYKIQALEHDTKKKYYVFRSWGRVGTT
IGGNKLEHFVNQEDAIENFKEVYGEKTGNTWSNRKDFVKYPNKFYPLDIDYGQNDEMLQSSITPGSLSIL
PNPVKELIKLIFDVEAMKHALVEFEIDMKKMPLGKLSKKQIETAYGCLGDCQKFLANNDPGSKILDASNR
FYTLIPHDFGMKKPPLLDNEELIKLKIGMLDSLMEIEVAYNLIKGTKDNGAKDPIDLHYETLKTDIKVVD
KESKDFELIVEYVKNTHAKTHSNYSLDVIEVFSIERAGEKQRYRPFKDLFNKKLLWHGSRTTNYAGILSQ
GLRIAPPEAPVTGYMFGKGIYFADMASKSANYCCTTRSNNTGLMLLCEVALGNMYELKQSEHISKLPPGK
HSCKGLGSTSPDPSMDKVIDGEVLVPLGKPISTNIKDSTLLYNEYIVYDVAQVNIKYLVQLKFNYCY

>XP_003385071.1 PREDICTED: poly [ADP-ribose] polymerase 1-like [Amphimedon queenslandica]
MADDDPPYRTEYAKSNRSSCKACRETIAKDSLRMARMVQAPNFDAKIPHWYHFSCFFGPKMKLVSVSQIG
GMGGLRWDDQQKIQAKIDGGGGATAGKKRKKGKSADGEENATKKQKKTESPEDKALREQNEMIWKIRDKL
ESHLTKGELKDLLDANGQAIPKGESCILDACADGMVFGSLGLCEECGGQLRVRTHSYSCAGFISDWTKCT
NTTTEAKRRKWVIPKQLKEYDFLKSYKYVPRTRVFPEAPPTPSPSVGSIASSSSQQPIDPSKPLSGFKIA
SSGRLSKTKGELKKLIVSLGGEMSSTVNRNTTLLISNEAEVEENSSTAICAASRYQIPVVDIEYLDDVAS
GDALGKVKSHTISSWTASPHLEGSNGREETDSAVFEKVPLTVKMKVKGGGVVDPDSGLDSSHHIYEQSGE
KYTAVLGLVDITKGTNSYYKLQLLESDSGKSYYLFRAWGRVGTTIGGNKLEDFGYDLDDALSEFLRLYQE
KTGTSWHIRKHARKVPGKFYPIDIDYGEEEGPSLSLSGAGSNSKLAPEIQDLIKMIFDIQKMKDAMMEFE
IDMKKMPLGKLSKRQILSAYSVLNELQNELTGAKNPSRILDGSNRFYTLIPHDFGMAKPPLLETEEMIKS
KILMLDNLKEIEIAYSLLKTEGEGSADKDPLDIHYEKLKTNMSVLDKESEEFKRLVEYVANTHAATHNQY
QLEVQEVFNLERHGEKKRYKPFSKLENRMLLWHGSRLTNFVGILSQGLRIAPPEAPVTGYMFGKGVYFAD
MVSKSANYCWTSPQSPVGLMLLCEVALGNMYEREHADYITSLPAGKHSTKGIGRTAPDPTSNYVSESGAI
IPMGKGTTSSSRSTSLLYNEYIVYDVAQINMKYLIKMNFKYRR

Alignment

```
NP_001104452.1[D.melanogaster]       ---------------MDIELPYLAEYARTGRATCKGCKSTISKDTLRIAVMVQSAFHDAK           45
XP_003385071.1[A.queenslandica]      --------------MADDDPPYRTEYAKSNRSSCKACRETIAKDSLRMARMVQAPNFDAK           46
XP_032819895.1[P.marinus]            ----------------MADKPFKAEYAKSSRASCKKCGGNIEKDCLRLAVMVQSPRFDGK           44
XP_014061166.1[S.salar]              ------------MADAQEDKLYKAEYAKSGRASCKKCKENIAKDSLRMAIMVQSPMFDGK           48
NP_001081571.1[X.laevis]             -------------MAEASDKLYRAEYAKSGRASCKKCGDNIAKESLRLAIMVQSPMFDGK           47
P26446.2[G.gallus]                   -------------MAETGDKPYRAEYAKSGRASCKKCGESIAKDSLRLALMVQSPMFDGK           47
BAE39743.1[M.musculus]               -------------MAEASERLYRVEYAKSGRASCKKCSESIPKDSLRMAIMVQSPMFDGK           47
NP_001609.2[H.sapiens]               -------------MAESSDKLYRVEYAKSGRASCKKCSESIPKDSLRMAIMVQSPMFDGK           47
XP_024101750.1[P.abelii]             -------------MAESSDKLYRVEYAKSGRASCKKCSESIPKDSLRMAIMVQSPMFDGK           47
XP_047140981.1[H.vulgaris]           ---------------MSEDLPYNAEYAKSGRAGCKSCKCNIGKDSLRIAKMVQSPHFDGK           45
XP_019618726.1[B.belcheri]           MLAYRVQEAAKISMADDEGKAYKAEYAKSNRASCKLCKGNITKDSLRLARMVQSPHFDGK           60
XP_032219514.1[N.vectensis]          --------------MSENDVPFKTEYAKSNRSSCKSCKSNIGKDSLRVARMVQSPHFDGK           46
                                                          : .***::.*: ** *  .* *: **:* ***:  .*.*

NP_001104452.1[D.melanogaster]       VPNWFHKTCFFKN-QRPSSVG--DIQNIGNLRFADQKELTDLVENIQEVISAQLGK-KRS           101
XP_003385071.1[A.queenslandica]      IPHWYHFSCFFGPKMKLVSV--SQIGGMGGLRWDDQQKIQAKIDGGGGATAG--KKR---           99
XP_032819895.1[P.marinus]            VPQWHHFQCFWKR-ARPLTQA--DVDGFSELRSADQDRIKKHIEGSADGEGKSSGKANGG           101
XP_014061166.1[S.salar]              VPHWHHFSCFWLR-ASAQSP--ADIAGFSDLRWEDQEKVKKAIETGGAAGGK-GD-VKGG           103
NP_001081571.1[X.laevis]             VPHWHHYSCFWKR-ARVLSQG--DIYGYTELRWEDQEMIKKAIETGGAAAGA-GG-DSKG           102
P26446.2[G.gallus]                   VPHWHHYSCFWKR-ARIVSHT--DIDGFPELRWEDQEKIKKAIETGALQEEK-GGTRKEV           103
BAE39743.1[M.musculus]               VPHWYHFSCFWKV-GHSIRQPDVEVDGFSELRWDDQQKVKKTAEAGGVAGK---GQDGSG           103
NP_001609.2[H.sapiens]               VPHWYHFSCFWKV-GHSIRHPDVEVDGFSELRWDDQQKVKKTAEAGGVTGK---GQDGIG           103
XP_024101750.1[P.abelii]             VPHWYHFSCFWKV-GHSIRHPDVEVDGFSELRWDDQQKVKKTAEAGGVTGK---GQDGIG           103
XP_047140981.1[H.vulgaris]           IPQWYHYSCFFKK-FKPLNV--SDIKNFDSLRWDDQEKLREQIGCPQKKDVV---D---G           96
XP_019618726.1[B.belcheri]           VPNWFHYSCFFKK-CKPSST--VEFSGVTGLRWDDQEKLKKTIGGEGSSAGA---DAGGD           114
XP_032219514.1[N.vectensis]          VPNWFHFSCFFKKQFKPKSL--AEIAGIDGLRWDDQEKFRAQVYGGASDVPD---TTAAA           101
                                     :*:*.*  **:            :. .   **  **. .

NP_001104452.1[D.melanogaster]       KAFNLALKDFGIEYAKSSRSTCRGCEQKINKDLVRLRKTVYDTEVGMKYGGQPLWHHLEC           161
XP_003385071.1[A.queenslandica]      ------------------------------------------------------------           99
XP_032819895.1[P.marinus]            AAASNTLPDFLVEYAKSNRSKCKGCDDKIEKDDVRISRKMINIE-KPHLGMIDHWYHTNC           160
XP_014061166.1[S.salar]              AKGEKTLNDFAVEYAKSNRSTCKGCEQKIEKDQIRVSKKTVDPE-KPQLGLIDRWYHTAC           162
NP_001081571.1[X.laevis]             GKGEMTLNDFAAEYAKSNRSACKGCEQKIEKGQIRISKKSVDVE-RPQLGMIDRWYHPDC           161
P26446.2[G.gallus]                   GKAEKSLTDFAAEYAKSNRSTCKGCEQKIEKGQIRISKKMVHPE-KPQLGMIDNWYHPDC           162
BAE39743.1[M.musculus]               GKAEKTLGDFLAEYAKSNRSMCKGCLEKIEKGQMRLSKKMVDPE-KPQLGMIDRWYHPTC           162
NP_001609.2[H.sapiens]               SKAEKTLGDFAAEYAKSNRSTCKGCMEKIEKGQVRLSKKMVDPE-KPQLGMIDRWYHPGC           162
XP_024101750.1[P.abelii]             SKAEKTLGDFAAEYAKSNRSTCKGCMEKIEKGQVRLSKKMLDPE-KPQLGMIDRWYHPGC           162
XP_047140981.1[H.vulgaris]           PSNSTVNKDFCIEYAKSGRAKCRKCEEKIEKDIIRIAKIMESEE-SKFKGLIPFWYHVKC           155
XP_019618726.1[B.belcheri]           EVDAPGVQVFSVEYAKSNRSACRGCSEKIDKGLVRISKKVDE---GDQWGPKDLWHHVDC           171
XP_032219514.1[N.vectensis]          TVS-VEQPDLLAEYAKSSRSTCKHCDEQIVKGELRLAK-VMD---GEKYGPVPKWHHVPC           156

NP_001104452.1[D.melanogaster]       FAQLRSELGWFA--SGEDMPGFQSLADDDQAKVKNAIPPIKSEELPDTKRA---------           210
XP_003385071.1[A.queenslandica]      ---------------------------------------------KKGKSADGEENAT           112
XP_032819895.1[P.marinus]            FVEHRDTLGFLSTYGANQIGGFTRLNANDKKAVETLLGTVVVDGKR--KAEDQVDEVA--           216
XP_014061166.1[S.salar]              FVGRREELVFKPEYSAAQLKGFNTLRVEDKEELKRRLPAVKSKGKR--KTDEVDGEGASK           220
NP_001081571.1[X.laevis]             FVSSREELGFLPSYSASQLKGFTILSAEDKDSLKKKLPAVKNEGKR--KADEVDGHSAAT           219
P26446.2[G.gallus]                   FVSRRAELGFLPAYGATQLLGFSILKAEDKETLKKQLPATKTEGKR--KGEEVDGNVV-A           219
BAE39743.1[M.musculus]               FVKKRDELGFRPEYSASQLKGFSLLSAEDKEALKKQLPAIKNEGKR--KGDEVDGTDEVA           220
NP_001609.2[H.sapiens]               FVKNREELGFRPEYSASQLKGFSLLATEDKEALKKQLPGVKSEGKR--KGDEVDGVDEVA           220
XP_024101750.1[P.abelii]             FVKNREELGFRPEYSASQLKGFSLLATEDKEALKKQLPGVKSEGKR--KGDEVDGVDEVA           220
XP_047140981.1[H.vulgaris]           FALSKSELEAQ-NLTSNEIDGWGELSEEDQEKVGLEIKFKKHGKLKIKKGNKINIKDECK           214
XP_019618726.1[B.belcheri]           FVEKREELGFTTDMAPSVIQGYKKLSKDDQDILVKKLGSGTTGKRK--AKG---SNG-AA           225
XP_032219514.1[N.vectensis]          FLKAMPDLDISGILTAQHFTGFQKLGKDDQKLLIDKFGSQKQISKK--KGKKKSLEASAA           214

NP_001104452.1[D.melanogaster]       --KMELSDTNEEGEKKQRLKDQNDAYFRFRDDIKNKMKKKDIDILLKFNNQQPVTGDTEK           268
XP_003385071.1[A.queenslandica]      K-----KQKKTESPEDKALREQNEMIWKIRDKLESHLTKGELKDLLDANGQAIPKGE-SC           166
XP_032819895.1[P.marinus]            -KKIKLESMQQKEEEEKMLKKQSNVIWSFKDGLKSCSDEIDLKELLIANKQAVPSGE-SA           274
XP_014061166.1[S.salar]              KKKD---EEEEKKKVELQMKEQSQLIWGIKDKLRQFCSTNDMKELLIANGQEVPSGE-SN           276
NP_001081571.1[X.laevis]             KKKIKKEKEK-ESKLEKLLKEQTELIWHIKDELKKVCSTNDLKELLIANKQQVPSGE-TN           277
P26446.2[G.gallus]                   KKKSRKEKEK-ESKQEKQLKEQTELIWGIKDELRKVCSTNDLKELLIANKQEVPSGE-NA           277
BAE39743.1[M.musculus]               KKKSKKGKDKDSSKLEKALKAQNELIWNIKDELKKACSTNDLKELLIFNQQQVPSGE-SA           279
NP_001609.2[H.sapiens]               KKKSKKEKDKD-SKLEKALKAQNDLIWNIKDELKKVCSTNDLKELLIFNKQQVPSGE-SA           278
XP_024101750.1[P.abelii]             KKKSKKEKDKD-SKLEKALKAQNDLIWNIKDELKKVCSTNDLKELLIFNKQQVPSGE-SA           278
XP_047140981.1[H.vulgaris]           D----EEKKEIKDEKVEKFKLQNKEYWDMRDKLQKNVPNNVLRKLLDANNYNTTGGE-NT           269
XP_019618726.1[B.belcheri]           A----KKVKAEETEEEKKLKEQSKLVWKIRDELSKSMENSDLKELLLANDQDIPSGE-SA           280
XP_032219514.1[N.vectensis]          M----PVKSASEEKEEQQLKEQSEAIWKIRDELKTNCSLSELREMLEDNEINPQGGE-TS           269
                                                       :: *..  : ::* :        :  :*  *      *:

NP_001104452.1[D.melanogaster]       LFDQTADLLTFGAIESCSECNSCQFIVNKSGYICNGNHSEWTKCNKLLKEPTRSACIVPK           328
XP_003385071.1[A.queenslandica]      ILDACADGMVFGSLGLCEECG-GQLRVRTHSYSCAGFISDWTKCTNTTTEAKRRKWVIPK           225
XP_032819895.1[P.marinus]            VLERLADGMAFGALLPCAECKDGQLVCMSDAYRCSGNISGWTKCTFSTKTPKRNEWIIPK           334
XP_014061166.1[S.salar]              VVDSLGDCMAFGALKPCEECQ-GQLVFKSDAYYCTGDLSAWTKCVFKTQLPNRKDWVIPK           335
NP_001081571.1[X.laevis]             IVDRVSDGMAFGALLPCEECS-GQFVFKGDAYYCTGDLSAWTKCVAKTQTPNRKDWVTPK           336
P26446.2[G.gallus]                   ILDRVADGMAFGALLPCEECK-GQFVFKSDAYYCSGDITAWTKCVAKTQTPNRKDWVIPK           336
BAE39743.1[M.musculus]               ILDRVADGMAFGALLPCKECS-GQLVFKSDAYYCTGDVTAWTKCMVKTQNPSRKEWVTPK           338
NP_001609.2[H.sapiens]               ILDRVADGMVFGALLPCEECS-GQLVFKSDAYYCTGDVTAWTKCMVKTQTPNRKEWVTPK           337
XP_024101750.1[P.abelii]             ILDRVADGMVFGALLPCEECS-GQLVFKSDAYYCTGDVTAWTKCMVKTQTPNRKEWVTPK           337
XP_047140981.1[H.vulgaris]           LLERCADGICFGALLTCSECKTGNLVYRTEGYQCTGHVSGWTSCTFLTQTPKRKKWHIPK           329
XP_019618726.1[B.belcheri]           LLDRLSDGMAFGALQRCPECKHGQLFYRSDGYHCSGNLTEWTKCIYTTREPKRKKWIIPD           340
XP_032219514.1[N.vectensis]          VFDRCSDGMLFGRLLPCHECHGGMLVYRSDGYHCTGNVSGWTKCAFTTQDPKRTKWVISK           329
                                     :.:  .* : ** :  * **    :     .* * *  : **.*       .*      .

NP_001104452.1[D.melanogaster]       ELKALYNFLNTVKEIPSTRIFNNFPPNKSTFSRSLLKTNK-------NNDVLVRPTIPRI           381
XP_003385071.1[A.queenslandica]      QLK-EYDFLKSYKYVPRTRVFPEAPPTPSPSVGS--------------IASSSSQQPIDP           270
XP_032819895.1[P.marinus]            EFK-DVPFLKKYKYKQRERVFMSAQATTSPPRAATVATATVATAATKSGEAPGTSAGAVP           393
XP_014061166.1[S.salar]              EFH-ELPFLKKFKFKRQDRVFPKVAPAPPPASSAAVPCAS-------SAPSIPLPEGAPA           387
NP_001081571.1[X.laevis]             EFH-EIPYLKKFKFKRHDRAFPPCAAPT-PIS---PPAA--------PEPKPTVEETFPE           383
P26446.2[G.gallus]                   EFR-EIPYLKKFKCKKQDRIFPPEAATVNSAP---PPP---------ASAPLTETVTAPQ           383
BAE39743.1[M.musculus]               EFR-EISYLKKLKVKKQDRIFPPESSAPAPLA---LPLSV-------TSAPTAVNSSAPA           387
NP_001609.2[H.sapiens]               EFR-EISYLKKLKVKKQDRIFPPETSASVAAT---PPPST-------ASAPAAVNSSASA           386
XP_024101750.1[P.abelii]             EFR-EISYLKKLKVKKQDRIFPPETSAPVAAT---PPPST-------ASAPAAVNSSASA           386
XP_047140981.1[H.vulgaris]           ELLLENDFLSTIKPKVEERTFMTTTKMNT------------------------DIKESQ           364
XP_019618726.1[B.belcheri]           DLKEEVPFLKKFKSKVAARVFSAAHVAAASES----------------TDSFSSSSRADE           384
XP_032219514.1[N.vectensis]          ELKKTNDFLKTFKCKEMKRLFPKLTAIQPG--------------------------AIP           362
                                     ::     :*.. *     * *

NP_001104452.1[D.melanogaster]       SPPLYNLKFSIIGL-KNQHKELRKRIENLGGKFEVKISENTIAIISTELEIQKKS-TRMK           439
XP_003385071.1[A.queenslandica]      SKPLSGFKIASSGRLSKTKGELKKLIVSLGGEMSSTVNRNTTLLISNEAEVEENSSTAIC           330
XP_032819895.1[P.marinus]            GKLLAGKALCLVGKLQKSRDDIKALVEKLGGKMTSTVP-KADFCISTKKEVEKMS-KKIA           451
XP_014061166.1[S.salar]              DKPLTGMKLLTVGKLRQNKDELKAAVEELGGKITGTAN-KATLCLSTKKEVEKMS-KKME           445
NP_001081571.1[X.laevis]             GKPLTNTKVLLIGKLSKNKDEVKTLIEGLGGKVAGSAH-KANLCISTNKEVEKMS-KKME           441
P26446.2[G.gallus]                   DKPLTNMKILTLGKLSKNKEEVKNIVEELGGKMTTTAN-KATLCISTQKEVEKMS-KKME           441
BAE39743.1[M.musculus]               DKPLSNMKILTLGKLSQNKDEAKAVIEKLGGKLTGSAN-KASLCISTKKEVEKMS-KKME           445
NP_001609.2[H.sapiens]               DKPLSNMKILTLGKLSRNKDEVKAMIEKLGGKLTGTAN-KASLCISTKKEVEKMN-KKME           444
XP_024101750.1[P.abelii]             DKPLSNMKILTLGKLSRNKDEVKAMIEKLGGKLTGTAN-KASLCISTKKEVEKMN-KKME           444
XP_047140981.1[H.vulgaris]           KRPLENRKVIVIGKLKESKGVIENKIQLLGGTLSAAVSKSCYCCISTQDEIEKST-KKMQ           423
XP_019618726.1[B.belcheri]           KKPLH-HVKVVLGKTTKSKAEMTKAIEKLGGTVASKVDSTVACVISSKEDVQKMS-KKIK           442
XP_032219514.1[N.vectensis]          GKPLSNMMVAVIGKLKRTKADVASCVAQLGGTTMDRVTSKVDCCISTQAEVSKSS-KKMK           421
                                        *        *   . :      :  ***        .    :*.: ::.: . . :
```

```
NP_001104452.1[D.melanogaster]       FAEELGIHIVPIEFLDFVEADTEGAIKYINSTCICSWGTDPKSRIPKETTKS--------      491
XP_003385071.1[A.queenslandica]      AASRYQIPVVDIEYLDDVASG--DALGKVKSHTISSWTASPHLEGSNGREET--------      380
XP_032819895.1[P.marinus]            EAQTCNVCVVAEDLLTE--ASGRPLAELVSAHRLSTWGAPIKVEQEPTVKTEPVLEVPKE      509
XP_014061166.1[S.salar]              EAEEVGVRVVSEDFLTDIKSSGKALQELVSLHAISPWGAEVKLETQAQSAA--SRSGAMA      503
NP_001081571.1[X.laevis]             EVKAANVRVVSDDFLKEVES-GKSVQELLSQFGISSWGAEIKQEAVQPTEKQ-PSSGPVA      499
P26446.2[G.gallus]                   EVKDAKVRVVSEEFLKDVKSSNKGFQELLSLHAISPWGAEVKTEHQEVAVDG-KCSKPAN      500
BAE39743.1[M.musculus]               EVKAANVRVVCEDFLQDVSASTKSLQELLSAHSLSSWGAEVKAEPGEVVASKGKSAA-PS      504
NP_001609.2[H.sapiens]               EVKEANIRVVSEDFLQDVSASTKSLQELFLAHILSPWGAEVKAEPVEVVAPRGKSGAALS      504
XP_024101750.1[P.abelii]             EVKEANIRVVSEDFLQDVSASTKSLQELFLAHILSPWGAEVKAEPVEVVAPRGKSGAALS      504
XP_047140981.1[H.vulgaris]           TVKTYNIPVVTENFLDAVQNG--EIDA-LMKNSIAPWGTNREMLDDKMDN---------F      471
XP_019618726.1[B.belcheri]           DAKAADVHVVSEDFVTDVEKG--GAALLIMQKSIASWGSDPHSRIATVEE---------K      491
XP_032219514.1[N.vectensis]          DAEKFNIPVVSEEFLDAIKDG--EFNSNIAKHSLVSWGKIKALDIVDGPL---------P      470
                                      ..   : :*  : :             .    :  *

NP_001104452.1[D.melanogaster]       -----LNSNSIYTKSMPVSRTFKVKDGLAVDPDSGLEDIAHVYVDSNNKYSVVLGLTDIQ      546
XP_003385071.1[A.queenslandica]      --------DSAVFEKVPLTVKMKVKGGGVVDPDSGLDSSHHIYEQSGEKYTAVLGLVDIT      432
XP_032819895.1[P.marinus]            ASAKSSKRKSEGQKAEANKLKLTVKGRAAVDPLSNLVDVAHVLEDRGMIYGAAMSLVDVE      569
XP_014061166.1[S.salar]              GKSTKRVKEEEGVSKASKKMKLTVKGGAAVDPDSGLENSAHVLDQNGKIFSATLGLVDIV      563
NP_001081571.1[X.laevis]             GKSSGKVKEEKGSNKSEKKMKLTVKGGAAIDPDSELEDSCHVLETGGKIFSATLGLVDIT      559
P26446.2[G.gallus]                   MKSAGKVKEEQGPSKSEKKMKLTVKGGAAVDPDSGLEDSAHVFEKGGKIFSATLGLVDIV      560
BAE39743.1[M.musculus]               KKSKGAV-KEEGVNKSEKRMKLTLKGGAAVDPDSGLEHSAHVLEKGGKVFSATLGLVDIV      563
NP_001609.2[H.sapiens]               KKSKGQV-KEEGINKSEKRMKLTLKGGAAVDPDSGLEHSAHVLEKGGKVFSATLGLVDIV      563
XP_024101750.1[P.abelii]             KKSKGQV-KEEGINKSEKRMKLTLKGGAAVDPDSGLEHSAHVLEKGGKVFSATLGLVDIV      563
XP_047140981.1[H.vulgaris]           K---SMKRKTSSSIESSKKAKVIMKGGAVVDPDSGLEGKCHILSVRGELFTAVLGLVDLL      528
XP_019618726.1[B.belcheri]           PSKSKSKSKYEEMESGTKKLKMMVKGGAAVDPDSGIEHSAHVLEDKGKVYNAVLGLVDLV      551
XP_032219514.1[N.vectensis]          LRRKASDSKYELGASQPKKVKLSVKGGAAVDPASEMEDDCHVLEVKGVVYSATLGMVDIS      530
                                             .           .. :*.  .:** * :    *:    .  : ..:.:.*:

NP_001104452.1[D.melanogaster]       RNKNSYYKVQLLKADKKEKYWIFRSWGRIGTNIGNSKLEEFDT-SESAKRNFKEIYADKT      605
XP_003385071.1[A.queenslandica]      KGTNSYYKLQLLESDSGKSYYLFRAWGRVGTTIGGNKLEDFGYDLDDALSEFLRLYQEKT      492
XP_032819895.1[P.marinus]            RGTNSYYKLQLLECDLNRKYWVFRSWGRLGTTIGGTKLEKMKS-REDAVEHFCQLYYDKT      628
XP_014061166.1[S.salar]              RGTNSYYKLQLLEDDVHKRYWVFRSWGRVGTTIGGKKLDKFND-KNSAMDNFLGVYEEKT      622
NP_001081571.1[X.laevis]             RGTNSYYKLQLIEHDRDSRYWVFRSWGRVGTVIGSKKLEEMSS-KEDAIEHFLNLYQDKT      618
P26446.2[G.gallus]                   KGTNSYYKLQLLEDDRESRYWVFRSWGRVGTVIGSNKLEQMPS-KEDAVEHFLNLYEEKT      619
BAE39743.1[M.musculus]               KGTNSYYKLQLLEDDKESRYWIFRSWGRVGTVIGSNKLEQMPS-KEDAVEHFMKLYEEKT      622
NP_001609.2[H.sapiens]               KGTNSYYKLQLLEDDKENRYWIFRSWGRVGTVIGSNKLEQMPS-KEDAIEHFMKLYEEKT      622
XP_024101750.1[P.abelii]             KGTNSYYKLQLLEDDKENRYWIFRSWGRVGTVIGSNKLEQMPS-KEDAIEHFMKLYEEKT      622
XP_047140981.1[H.vulgaris]           RGTNSYYKIQALEHDTKKKYYVFRSWGRVGTTIGGNKLEHFVN-QEDAIENFKEVYGEKT      587
XP_019618726.1[B.belcheri]           RGTNSYYKLQVLEADKGNRWYVFRAWGRVGTTVGGNKLETFHS-RQGALEQFLNLYEEKT      610
XP_032219514.1[N.vectensis]          RGTNSYYKLQVLKKDKSSRCYLFRSWGRVGTTIGGSKLEDCGNSHNSAVQSFEELYREKT      590
                                     :..*****:* :: *     ::**:***:** :*..**:      :.*   *  :* :**

NP_001104452.1[D.melanogaster]       GNEYEQRDNFVKRTGRMYPIEIQYDDDQKLVKHES--HFFTSKLEISVQNLIKLIFDIDS      663
XP_003385071.1[A.queenslandica]      GTSWHIRKHARKVPGKFYPIDIDYGEEEGPSLS-LSGAGSNSKLAPEIQDLIKMIFDIQK      551
XP_032819895.1[P.marinus]            GNKWNA-RNFVKYPNKFYPVETDYGQDDEDIHDLKAMAGTNSKLAKPIQELLRMIFDIDS      687
XP_014061166.1[S.salar]              GNAWGS-SHFTKYPNRFYPLEIDYGQDEEAVKRLTASAGTNSLLAKPVQELIRTIFDVES      681
NP_001081571.1[X.laevis]             GNAWHS-PNFTKYPKKFYPLEIDYGQEEDVVKKLSVGAGTKSKLAKPVQELIKLIFDVES      677
P26446.2[G.gallus]                   GNSWHS-KNFTKYPKKFYPLEIDYGQDEEAVRKLTVSAGTKSKLAKPIQDLIKMIFDVES      678
BAE39743.1[M.musculus]               GNAWHS-KNFTKYPKKFYPLEIDYGQDEEAVKKLTVKPGTKSKLPKPVQELVGMIFDVES      681
NP_001609.2[H.sapiens]               GNAWHS-KNFTKYPKKFYPLEIDYGQDEEAVKKLTVNPGTKSKLPKPVQDLIKMIFDVES      681
XP_024101750.1[P.abelii]             GNAWHS-KNFTKYPKKFYPLEIDYGQDEEAVKKLTVNPGTKSKLPKPVQDLIKMIFDVES      681
XP_047140981.1[H.vulgaris]           GNTWSNRKDFVKYPNKFYPLDIDYGQNDEML-QSSITPGSLSILPNPVKELIKLIFDVEA      646
XP_019618726.1[B.belcheri]           GNEFGT-KNFVKYPKRFYPLDIDYGQEEEDLQKLKIKPGSKSTLAREVQEIIQMIFDIES      669
XP_032219514.1[N.vectensis]          GNEWSERDNFVKHPNRFYPLEIDYGQAHEDVVKAKIEMGSKSKLPSQIQELIKMIFDVES      650
                                     *. :    .  *   ::**:: :*.: .             * *   :::::  ***::

NP_001104452.1[D.melanogaster]       MNKTLMEFHIDMDKMPLGKLSAHQIQSAYRVVKEIYNVLECGSNTAKLIDATNRFYTLIP      723
XP_003385071.1[A.queenslandica]      MKDAMMEFEIDMKKMPLGKLSKRQILSAYSVLNELQNELTGAKNPSRILDGSNRFYTLIP      611
XP_032819895.1[P.marinus]            MKRTMVEFEIDLCKMPLGKLSRKQIQSAYSVLGDAQKLVSESGLTSLIQDLSNKFYTLIP      747
XP_014061166.1[S.salar]              MKKAMVEFEIDLQKMPLGKLSKRQIQSAYALLSEVQQAVSDSSSESHILDLSNRFYTLIP      741
NP_001081571.1[X.laevis]             MKKAMVEFEIDLQKMPLGKLSKRQIQSAYSILSQVQQAVSESLSEARLLDLSNQFYTLIP      737
P26446.2[G.gallus]                   MKKAMVEFEIDLQKMPLGKLSKRQIQSAYSILNEVQQAVSDGGSESQILDLSNRFYTLIP      738
BAE39743.1[M.musculus]               MKKALVEYEIDLQKMPLGKLSRRQIQAAYSILSEVQQAVSQGSSESQILDLSNRFYTLIP      741
NP_001609.2[H.sapiens]               MKKAMVEYEIDLQKMPLGKLSKRQIQAAYSILSEVQQAVSQGSSDSQILDLSNRFYTLIP      741
XP_024101750.1[P.abelii]             MKKAMVEYEIDLQKMPLGKLSKRQIQAAYSILSEVQQAVSQGSSDSQILDLSNRFYTLIP      741
XP_047140981.1[H.vulgaris]           MKHALVEFEIDMKKMPLGKLSKKQIETAYGCLGDCQKFLANNDPGSKILDASNRFYTLIP      706
XP_019618726.1[B.belcheri]           MKKAMVEFEIDLKKMPLGKLSRKQIESAYSVLTELNGILTGERSATRILDASNRFYTLIP      729
XP_032219514.1[N.vectensis]          MKKTLIEFEIDLKKMPLGKLTKRQIEKAYSVLGEAQQILSEAASKTAVLDVSNRFYTLIP      710
                                     *: :::*:.**: *******: :**  **  : :    :      : : * :*:******

NP_001104452.1[D.melanogaster]       HNFGVQLPTLIETHQQIEDLRQMLDSLAEIEVAYSIIKSED--VSDACNPLDNHYAQIKT      781
XP_003385071.1[A.queenslandica]      HDFGMAKPPLLETEEMIKSKILMLDNLKEIEIAYSLLKTEGE-GSADKDPLDIHYEKLKT      670
XP_032819895.1[P.marinus]            HDFGMKKPTLLDNLDYIKMKVDMLDNLMDIEVAYSLLNPKDETSGGKKDLLDASYEKLHT      807
XP_014061166.1[S.salar]              HDFGMKKPPLLRNLDYIQAKVQMLDNLLDIEVAYSLLKGGA--EDNKKDPIDINYEKLKT      799
NP_001081571.1[X.laevis]             HDFGMKKPPLLNNLEYIQAKVQMLDNLLDIEVAYSLLRGGA--DDGEKDPIDVKYEKIKT      795
P26446.2[G.gallus]                   HDFGMKKPPLLSNLEYIQAKVQMLDNLLDIEVAYSLLRGGN--EDGDKDPIDINYEKLRT      796
BAE39743.1[M.musculus]               HDFGMKKPPLLNNADSVQAKVEMLDNLLDIEVAYSLLRGGS--DDSSKDPIDVNYEKLKT      799
NP_001609.2[H.sapiens]               HDFGMKKPPLLNNADSVQAKVEMLDNLLDIEVAYSLLRGGS--DDSSKDPIDVNYEKLKT      799
XP_024101750.1[P.abelii]             HDFGMRKPPLLNNADSVQAKVEMLDNLLDIEVAYSLLRGGS--DDSSKDPIDVNYEKLKT      799
XP_047140981.1[H.vulgaris]           HDFGMKKPPLLDNEELIKLKIGMLDSLMEIEVAYNLIKGTK--DNGAKDPIDLHYETLKT      764
XP_019618726.1[B.belcheri]           HDFGMKKPPMLDNEEVIKAKTTMLDNLLEIEVAYNLLKSGD--DGEGKDPIDAHYEKLKC      787
XP_032219514.1[N.vectensis]          HDFGMRKPPLLDNQELIKTKIEMLDNLIDIEVAFSLLQSSD--GEDAEDPIDINYKKLKT      768
                                     *:**:  * :: . : ::    ***.* :**:*:.::.          : :*  *  ::

NP_001104452.1[D.melanogaster]       QLVALDKNSEEFSILSQYVKNTHASTHKSYDLKIVDVFKVSRQGEARRFKPFKKLHNRKL      841
XP_003385071.1[A.queenslandica]      NMSVLDKESEEFKRLVEYVANTHAATHNQYQLEVQEVFNLERHGEKKRYKPFSKLENRML      730
XP_032819895.1[P.marinus]            TIEVLGKDTEEFRVILEYVTNTHAHTHDAYELEVEEVFKVSRDGEFQRYRPFRELHNRQL      867
XP_014061166.1[S.salar]              KIEVVDKTTKEAEIILQYVKNTHAATHNTYTLEVEEIFKIVREGEYQKYRPFQDLPNRQL      859
NP_001081571.1[X.laevis]             DIKVVAKDSEESRIICDYVKNTHADTHNAYDLEVLEIFKIDREGEYQRYKPFKQLHNRQL      855
P26446.2[G.gallus]                   DIKVVDKDSEEAKIIKQYVKNTHAATHNAYDLKVVEIFRIEREGESQRYKPFKQLHNRQL      856
BAE39743.1[M.musculus]               DIKVVDRDSEEAEVIRKYVKNTHATTHNAYDLEVIDIFKIEREGESQRYKPFRQLHNRRL      859
NP_001609.2[H.sapiens]               DIKVVDRDSEEAEIIRKYVKNTHATTHNAYDLEVIDIFKIEREGECQRYKPFKQLHNRRL      859
XP_024101750.1[P.abelii]             DIKVVDRDSEEAEIIRKYVKNTHATTHNAYDLEVIDIFKIEREGECQRYKPFKQLHNRRL      859
XP_047140981.1[H.vulgaris]           DIKVVDKESKDFELIVEYVKNTHAKTHSNYSLDVIEVFSIERAGEKQRYRPFKDLFNKKL      824
XP_019618726.1[B.belcheri]           KMEVVEKASDEFAMVQEYVKNTHAKTHSHYTLEVEELFKIAREGEASRYRPFQQLHNRQL      847
XP_032219514.1[N.vectensis]          DIEVLDHKSAEFGLISEYVSNTHADTHRQYELELLDVFKIKREGEEALFKPFKQLHNRQL      828
                                      : .: : : :   : .** **** **  * *.: ::* : * **   ::** .* *: *

NP_001104452.1[D.melanogaster]       LWHGSRLTNFVGILSHGLRIAPPEAPPTGYMFGKGIYFADMVSKSANYCCTSQQNSTGLM      901
XP_003385071.1[A.queenslandica]      LWHGSRLTNFVGILSQGLRIAPPEAPVTGYMFGKGVYFADMVSKSANYCWTSPQSPVGLM      790
XP_032819895.1[P.marinus]            LWHGSRMTNYAGILSKGLRIAPKEAPVTGYMFGKGVYFADMVSKSANYCHTSPSNPTGLI      927
XP_014061166.1[S.salar]              LWHGSRATNYAGILSQGLRIAPPEAPVTGYMFGKGVYFADMVSKSANYCHTSQSDPTGLL      919
NP_001081571.1[X.laevis]             LWHGSRTTNFAGILSQGLRIAPPEAPVTGYMFGKGIYFADMVSKSANYCHAMPGSPIGLI      915
P26446.2[G.gallus]                   LWHGSRTTNFAGILSQGLRIAPPEAPVTGYMFGKGIYFADMVSKSANYCHTSQADPIGLI      916
BAE39743.1[M.musculus]               LWHGSRTTNFAGILSQGLRIAPPEAPVTGYMFGKGIYFADMVSKSANYCHTSQGDPIGLI      919
NP_001609.2[H.sapiens]               LWHGSRTTNFAGILSQGLRIAPPEAPVTGYMFGKGIYFADMVSKSANYCHTSQGDPIGLI      919
XP_024101750.1[P.abelii]             LWHGSRTTNFAGILSQGLRIAPPEAPVTGYMFGKGIYFADMVSKSANYCHTSQGDPIGLI      919
XP_047140981.1[H.vulgaris]           LWHGSRTTNYAGILSQGLRIAPPEAPVTGYMFGKGIYFADMASKSANYCCTTRSNNTGLM      884
XP_019618726.1[B.belcheri]           LWHGSRVTNYAGILSQGLRIAPPEAPVTGYMFGKGLYFADMVSKSANYCATSTASPTGLL      907
XP_032219514.1[N.vectensis]          LWHGSRTTNYAGILSQGLRIAPPEAPVTGYMFGKGVYFADMVSKSANYCCTSPSNNIGLL      888
                                     ****** **:.****:****** *** ********:*****.******* :   .  **:

NP_001104452.1[D.melanogaster]       LLSEVALGDMMECTSAKYINKLSNNKHSCFGRGRTMPDPTKSYIRSDGVEIPYGETITDE      961
```

```
XP_003385071.1[A.queenslandica]      LLCEVALGNMYEREHADYITSLPAGKHSTKGIGRTAPDPTSNYVSESGAIIPMGKGTTSS           850
XP_032819895.1[P.marinus]            LLAEVALGNMHELKQSESITKLPKGTHSVKGVGKTAPDPKKTVTL-NGVQVPIGNPSATG           986
XP_014061166.1[S.salar]              LLGEVALGNMHELKKASHITKLPKGKHSVKGLGRNAPDPSATVTL-DGVQVPLGKGTNTN           978
NP_001081571.1[X.laevis]             LLGEVALGNMHELKAASQITKLPKGKHSVKGLGRTAPDPSATVQL-DGVDVPLGKGTSAN           974
P26446.2[G.gallus]                   LLGEVALGNMYELKNASHITKLPKGKHSVKGLGKTAPDPTATTTL-DGVEVPLGNGISTG           975
BAE39743.1[M.musculus]               LLGEVALGNMYELKHASHISKLPKGKHSVKGLGKTTPDPSASITL-EGVEVPLGTGIPSG           978
NP_001609.2[H.sapiens]               LLGEVALGNMYELKHASHISKLPKGKHSVKGLGKTTPDPSANISL-DGVDVPLGTGISSG           978
XP_024101750.1[P.abelii]             LLGEVALGNMYELKHASHISKLPKGKHSVKGLGKTTPDPSASISL-DGVEVPLGTGISSG           978
XP_047140981.1[H.vulgaris]           LLCEVALGNMYELKQSEHISKLPPGKHSCKGLGSTSPDPSMDKVIDGEVLVPLGKPISTN           944
XP_019618726.1[B.belcheri]           LLCEVALGNMYERKHAEYVSKLPKGMHSTKGLGATGPDPGATKTLPNGTQVPIGHGVPSG           967
XP_032219514.1[N.vectensis]          LLCEVALGNMHELKHASFIKKVPKGKHSVKGLGKTAPDPSATHTFEDGTIVPKGKGCPAP           948
                                     ** *****:* *   :. :..:  . **  * * . ***         . :* *

NP_001104452.1[D.melanogaster]       HLKSSLLYNEYIVYDVAQVNIQYLFRMEFKYSY----          994
XP_003385071.1[A.queenslandica]      SRSTSLLYNEYIVYDVAQINMKYLIKMNFKYRR----          883
XP_032819895.1[P.marinus]            INDTSLLYNEYIVYDVAQVNIKYLLKLNFKYKMQLW-          1022
XP_014061166.1[S.salar]              IDDTSLLYNEYIVYDVAQVNLKYLLKIKFNYQMSMW-          1014
NP_001081571.1[X.laevis]             ISDTSLLYNEYIVYDIAQVNLKYLLKLKFNYKGGMMW          1011
P26446.2[G.gallus]                   INDTCLLYNEYIVYDVAQVNLKYLLKLKFNYKTSLW-          1011
BAE39743.1[M.musculus]               VNDTCLLYNEYIVYDIAQVNLKYLLKLKFNFKTSLW-          1014
NP_001609.2[H.sapiens]               VNDTSLLYNEYIVYDIAQVNLKYLLKLKFNFKTSLW-          1014
XP_024101750.1[P.abelii]             VNDTCLLYNEYIVYDIAQVNLKYLLKLKFNFKTSLW-          1014
XP_047140981.1[H.vulgaris]           IKDSTLLYNEYIVYDVAQVNIKYLVQLKFNYCY----          977
XP_019618726.1[B.belcheri]           VSGSSLLYNEYIVYDVAQVEMKYLIRMKFNYKSLW--          1002
XP_032219514.1[N.vectensis]          VKDSSLLYNEYIVYDTAQINMKYLLKTKFKYKYGW--          983
                                        : ********** **::::**.: :*::
```

Percent identity matrix:

#
#
Percent Identity Matrix - created by Clustal2.1
#
#

1: NP_001104452.1[D.melanogaster]	100.00	45.19	40.18	41.21	42.21	42.13	41.10	41.66	41.76	43.42	42.96	43.56
2: XP_003385071.1[A.queenslandica]	45.19	100.00	49.08	51.72	51.15	54.02	50.46	51.21	51.09	49.59	53.75	52.20
3: XP_032819895.1[P.marinus]	40.18	49.08	100.00	59.56	58.58	59.76	57.07	56.67	56.47	48.76	53.02	50.57
4: XP_014061166.1[S.salar]	41.21	51.72	59.56	100.00	72.14	73.63	69.18	69.88	69.68	50.16	54.22	52.52
5: NP_001081571.1[X.laevis]	42.21	51.15	58.58	72.14	100.00	77.98	73.66	74.06	73.66	50.47	55.10	52.94
6: P26446.2[G.gallus]	42.13	54.02	59.76	73.63	77.98	100.00	77.86	78.95	78.95	51.40	55.09	53.34
7: BAE39743.1[M.musculus]	41.10	50.46	57.07	69.18	73.66	77.86	100.00	92.40	92.60	50.31	54.93	52.87
8: NP_001609.2[H.sapiens]	41.66	51.21	56.67	69.88	74.06	78.95	92.40	100.00	99.41	51.29	54.38	53.24
9: XP_024101750.1[P.abelii]	41.76	51.09	56.47	69.68	73.66	78.95	92.60	99.41	100.00	51.19	54.18	53.24
10: XP_047140981.1[H.vulgaris]	43.42	49.59	48.76	50.16	50.47	51.40	50.31	51.29	51.19	100.00	51.71	52.38
11: XP_019618726.1[B.belcheri]	42.96	53.75	53.02	54.22	55.10	55.09	54.93	54.38	54.18	51.71	100.00	55.38
12: XP_032219514.1[N.vectensis]	43.56	52.20	50.57	52.52	52.94	53.34	52.87	53.24	53.24	52.38	55.38	100.00
