# Supplementary material for: An Evolutionary Perspective on the Origin, Conservation and Binding Partner Acquisition of Tankyrases
Source: Biomolecules. 2022 Nov 15;12(11):1688. doi: 10.3390/biom12111688 (PMC9688111; doi:10.3390/biom12111688)
Supplement: Supplementary file 1 [file biomolecules-12-01688-s001.zip › biomolecules-1913719-supplementary-11.17 -done/Data_2/PARP12.pdf]

PARP12

Sequences:

>NP_073587.1 protein mono-ADP-ribosyltransferase PARP12 [Homo sapiens]
MAQAGVVGEVTQVLCAAGGALELPELRRRLRMGLSADALERLLRQRGRFVVAVRAGGAAAAPERVVLAAS
PLRLCRAHQGSKPGCVGLCAQLHLCRFMVYGACKFLRAGKNCRNSHSLTTEHNLSVLRTHGVDHLSYNEL
CQLLFQNDPWLLPEICQHYNKGDGPHGSCAFQKQCIKLHICQYFLQGECKFGTSCKRSHDFSNSENLEKL
EKLGMSSDLVSRLPTIYRNAHDIKNKSSAPSRVPPLFVPQGTSERKDSSGSVSPNTLSQEEGDQICLYHI
RKSCSFQDKCHRVHFHLPYRWQFLDRGKWEDLDNMELIEEAYCNPKIERILCSESASTFHSHCLNFNAMT
YGATQARRLSTASSVTKPPHFILTTDWIWYWSDEFGSWQEYGRQGTVHPVTTVSSSDVEKAYLAYCTPGS
DGQAATLKFQAGKHNYELDFKAFVQKNLVYGTTKKVCRRPKYVSPQDVTTMQTCNTKFPGPKSIPDYWDS
SALPDPGFQKITLSSSSEEYQKVWNLFNRTLPFYFVQKIERVQNLALWEVYQWQKGQMQKQNGGKAVDER
QLFHGTSAIFVDAICQQNFDWRVCGVHGTSYGKGSYFARDAAYSHHYSKSDTQTHTMFLARVLVGEFVRG
NASFVRPPAKEGWSNAFYDSCVNSVSDPSIFVIFEKHQVYPEYVIQYTTSSKPSVTPSILLALGSLFSSR
Q

>XP_024106109.1 poly [ADP-ribose] polymerase 12 [Pongo abelii]
MAQAGVVGEVTQVLCAAGGALELPELRRRLRMGLSADALERLLRQRGRFVVAVRAGDAAAAPERVVLAVS
PLRLCRAHQGSKPGCVGLCAQLHLCRFMVYGACKFLKAGKNCRNSHSLATEHNLSVLRTHGVDHLSYNEL
CQLLFQNDPWLLPEICQHYNKGDGPHGSCAFQKQCIKLHICQYFLQGECKFGTSCKRSHDFSNSENLEKL
EKLGMSSDLVSRLPTIYRNAHDIKNKSSAPSRVPPPFVPQGTSERKDSSGSVSPNTLSQEESDQICLYHI
RKSCSFQDKCHRVHFHLPYRWQFLDRGKWKDLDNMELIEEAYCNPKIERILCSESASTFHSDCLNFNAMT
YGATQARRLSTASSVTKPPHFILTTDWIWYWSDEFGSWQEYGRQGTAHPVTTVSSSDVEKAYLAYCTPGS
DGQAATLKFQAGKHNYELDFKAFVQKNLVYGTTKKVCRRPKYMSPQDVTTMQTCNTKFPGPKSIPDYWDS
SALPDPGFQKITLSSSSEEYQKVWNLFNRTLPFYFVQKIERVQNLALWEVYQWQKGQMQKQNGGKAVDER
QLFHGTSTIFVDSICQQNFDWRVCGVHGTSYGKGSYFARDAAYSHHYSKSDTQTHTMFLARVLVGEFVRG
NASFVRPPAKEGWSNAFYDSCVNSVSDPSIFVIFEKHQVYPEYLIQYSTSSKPSVTPSILLALGSLFSRQ

>NP_766481.2 protein mono-ADP-ribosyltransferase PARP12 [Mus musculus]
MAQAAVAVAEVTQLLCAAGGALELAELRRRLRTSLGTDALERLLRDCGRFVVASRAVVAVGAGREAAAAA
SERLVLAVSSLRLCRAHQGPKPGCTGLCAQLHLCKFLIYGNCKFLKTGKNCRNGHNLKTDHNLSVLRTHG
VDHLTYTELCQLLLQNDPSLLPDICLHYNKGDGPFGSCSFQKQCIKLHICQYFLQGECKFGTSCKRSHEF
TNSESLEQLERLGLSSDLVSRLLSTYRNAYDIKNKGSALSKVSPSPAGPQGSSERKDSSGPVSPGTPSQE
ESEQICLYHIRKSCSFQEKCHRVHFHLPYRWQFLDGGKWKDLDNMELIEEAYSNPSKDRIVYTESAAGFH
FDNLDFNSMKFGNTLARRLSTASSVTKPPHFILTTDWIWYWMDEFGSWQEYGRQGSGHPVTTISSSDVER
AYLAFCAPGADAQAATLKFQAGKHNYELHFKAFLQKNLVYGTIRKVCRRPKYVSPQDVQMKQSCNTKLHG
PKSIPDYWDPAALPDLGFKKITLSSSSEEYQKVWNIFNRTLPFYFVQKIERIQNMGLWEVYQWQKCQMQK
QNGGKEVDERQLFHGTSANFVDAICQQNFDWRVCGLHGTSYGKGSYFARDAAYSHHYSKSDTHSHMMFLA
RVLVGDFVRGSTSFVRPPAKEGQSNAFYDSCVNSMSDPTIFVVFEKHQVYPEYLIQYSTSSKPPASPSIF
VALGNLFTSRQ

>XP_416333.5 protein mono-ADP-ribosyltransferase PARP12 isoform X1 [Gallus gallus]
MRAMRAAQRAQCPLGRQRGMTAIPAEPGALRSSRGRRDRSRSAARPFPLARPSRRQFRFRFLAQEAREWR
RRSRFRARPAGSGSAGGKQPGPAMVLPTQVLRVLCAGGGSLELEELLRRLPGRPTAEQLAAVLRDPQRFT
LVRTPDEAEAGGAAAAAAAAAEETVLVVATSSLRLCLEHGAGCRGGCGRLHLCKYHLKGVCRNQQARKEC
KFVHDFYSPHNHSVLRQHGLDSTFSNDELRQLLLQNDPTLLPEVCLHYNKGDGPYGSCTFKKMCTKLHVC
QYFLRGQCRFGSSCKRSHDFSKPECCEKLERQGMSSDIIQKLPSIYRNMYAIQNSKGSKHDTEDNKSLPC
KERKHSSSQESTSTSNEVEQICLYNLYTSCIFEDKCIRTHFHLPYRWQISDGTTWKDLESMEEIEKEYCD
PNNIRSSKPISESGFIFPYISFVDMQSGFKKVRRLSTASSVTKPPHFILTTEWIWYWKDEHGVWREYGKK
DSDRAAATVSSDDLEKAYTVGNSAKLNFKAGVHEYELDFGAMTQKNLRYKTERKVCRRPKFVSQSEVERI
RKRGFKNTEELRRIPSHWDKSALPELGFKLIELDSSSEEYNKVKGDFQRTMPKTHIKRICRIQNPSLWEL
YQWQKEQMQKSNGGKTDERFLFHGTSKKHIDAICHQNFDWRICGLNGTVYGKGSYFARDASYSDAYCREG
SCSKTMFQARVLVGEFTVGRSSYVRPPLKDNQNFYDSCVNSSSNPSIFVIFEKQQVYPEYLIEYLDQACA
RML

>XP_018096331.1 protein mono-ADP-ribosyltransferase PARP12 [Xenopus laevis]
MWTNQLIMVHTARLSRRLCSAGGSMELGQLGHSLGLNGQQLERLVEVEEGRSLVLRVQEGDQKVAVYRSD
LRLCQTPKCAGDCGQLHLCRFYILGSCNRSPCKFNHCIRNGHNLSVLQKFHLDSMPIDELRQLLLQNDPN
LLPDICSHYNRGDGPHGSCTYKNKCNKLHVCQHFLQGDCKFGEKCKRCHDLSEEETLKKLTKWQLSDSLL
PGLLETYTNAHTLKSSCDRPPRNVVKSAEKKTKPGPSKSGTSQEIEEICLYFIRNSCSFKEKCVREHFHL
PYRWQVYTNGTWKDLDNMEMIEKSYCDPNSRAVLVNLDFEAMTYQSNKVKRLSTPSSASKPPHFVFTTDW
KWYWMDEYNKWVEYGTESDRHANSTICSSDLENFYQSNQTADVKFKAGKHKYSLSFKNMVQRNLQYETKR
RVCRRPVFVSGKDVKKKRSSTSEPSKEDKNTPEHWDKGQTPDLGYKLVLLSPLSEEYSKIEAMFCRTLRT
IRIHSIERIQNLALWEVYQWQKEQMKKLNEGKDPEERQLFHGTKVKLIDAICQQNFDWRICGVHGTAYGK
GSYFARDSSYSHNYCKRSSSQANIMFVARVLVGNFTRGHSSYLRPPSKSEFRSTSFYDSCVDSKSNPSIF
VIFEKHQIYPEYLIKYSDK

>XP_014003371.1 protein mono-ADP-ribosyltransferase PARP12 [Salmo salar]
MSSIVSQYVTKILCGNQGCLDFKRLDQIVGQKFTVADDVLLGILCDREKYAISKGEGKASRLAMSQNSVI
VAKTSLRVCQSPPGECLHCENLHLCRYFVCGNCRFGNKCKNAHSLDSPHNTAVIRSLGHHDLGEAELFPL
LLQNDPYLMPEICSHYNKGVGEHGSCKFKTSCTSLHLCLHFLQGDCKFGAGCKRAHTFDATTMKILNGRG
FSQENIRILDKIYNNKFIIIGQGHEEKPSVVVPSPASAIEPVGKERSRKPSSRSISETDRNEICLFFIRR
HCSFKEKCVRIHYHLPYKWQILERDGVTWRDLPNVEEIEKAYCIPGKDTSGGIQPVNFLTMTCGRSPVRR
LSTVSSVTKPPHFILTTEWLWYWSGELGQWIEYGQETYDGKEKVASVTSKTLENVYQADPDSEIPFGSGH
NQYILRFKEMYQQNVRFKTKREVRRRPCFLSGQDVEAKLKSGSPESTSSSTVSVPPHWDKGALPDFTYKL
VPLLDQMTEYQMVQSLFKRTMSTSTIHKINRIQNPSLWRVFQWQKEQMKVKNGGKLVDERHLFHGTEPSI
VDAICEQNFDWRVCGIHGSHYGKGSYFARDALYSNRFSKSSVSGKKIMFVALVLVGEFTKGNRDYLRPPQ
KGTKKRLYDSCVDSESNPAIFVVFEKQQIYPEFVIEYS

>XP_032833032.1 protein mono-ADP-ribosyltransferase PARP12 [Petromyzon marinus]
MQSSYPQEGQQQQEKPPLPLDRALDTLHDSGCALPLSALASALGFPDSALLAALRSKPELFALVEGSGSS
SSSLSGGQQQQPERGSLDWRVLAVSALRVCPRYRERTGCPDGEGCAKLHVCRRFVLGSCKFGPDCKFSHD
FRDGHNRDLCYKSGVEALPFDPMQTLLLQNDPTLLPQICKTYKVLDAAGECKYGNKCARLHLCRPFVLGV
CKFGDKCRRNHGLSDAGLRRLLKQHGVCAWPDAALLGLLRRREQIHAAAGGGADAADDGATIAPLGFAAR
AAARGRKAGGSGGDGWPAGAAGRDGEGEICLYHLLSKCHYKDRCLKSHAALPYRWQERSHGGGAWQDLPG
TEAMERHYCDPASVSHDSVDFIHMKSVHGTVRRLSTANAVAMPDNFLLTTRWSWYWQECVGTWIKYGDEA
TTNDRAKSNVSSLDLEKAYHEDKEGSFKFTAGRFSYRLDFKDMTQTNLEVGTRRCVRRRPLLVTARDVEV
ARERHASLSRTGVGSVPAHWDRASLSDVDISLVELVNTTSEYKEVATLFKNTLSQPSINILNVKRVQNLE
LWDAFQRKREWMRKKNDGVEVEERKLFHGTRPKHVVPICQQNIDWRLRGAHGTAYGEGSYFARDASYSHT
YSKGRKGTAPASAAMFLARVLVGRYTCGKAGYVRPPAIKASPSTSAGQPAPEVFYDSCVNTEIDPSIFVV
FERDQIYPEYVIEYQF

>XP_019640190.1 PREDICTED: poly [ADP-ribose] polymerase 12-like [Branchiostoma belcheri]
MKPATNGQDGSPNGGGARGAFSCSPRGRGGNRGRGGRGRGFRGPSPSPDPAGRGGRGGARGGRGMDRGRG
RGGFYSPQQVPPPASGPMVANEAKFSMNDVMQLMQAMHMGAAPQMPQAQPEAAMVHPQAPPPSGGARSRP
RSRQRTAHNDNPPPVPAKLPQACTWYNNRQRGCTKGDACTFLHVCGRYLVGGCIFDKGQCEFSHNILTQQ
NKGILQKFGIPCNSEKAVIDIFKRDFVNKRKAEQGDKVPEKLDEICIFNIRQRCSYKTLCERWHCRMPYQ
WQYRAPAADRWRHFGHDLNVMVEEAYCKVTEDSYSPVRLDGKTCAVNFTDMKAVATDGSTGDTRQEFEVR
RLSTSSSVKSAPGHLFVTEWLWYWHDVDGTWVEYGQMNKSGEVRGTSSLTSRDIEDRYQDQRDADVTFST
SHHQYVLRLRDMCQENTRVGTRRDVRRRPKFVSEEDVKNPPKPKPASRAKSARSASAWDESAPAHWSPMS
DDDEFVKVDVVGTSAEYRNVKTLFEQGGMAGTAIAGVKRVQNAFLWSAYNRKKAQLKKQNGGKDAEERLL
FHGTQDAVVDAICQQNFDWRLSGSRVGQLYGQGTYFSASAQYSHSYAQQASNGRRYMFVVRVLVGAYTQG
DAGIRRPPPVNPGEPYGRMYDSCVNDTANPNIFVIFDNAQCYPEHIIEY

Alignment

```
XP_019640190.1[B.belcheri]      MKPATNGQDGSPNGGGARG--AFSCSPRGRGGNRGRGGRGRGFRGPSPSPDPA-------    51
XP_032833032.1[P.marinus]       ------------------------------------------------------------    0
XP_014003371.1[S.salar]         ------------------------------------------------------------    0
XP_018096331.1[X.laevis]        ------------------------------------------------------------    0
XP_416333.5[G.gallus]           MRAMRAAQRAQCPLGRQRGMTAIPAEPGALRSSRGRRDRSRSAARPFPLARPSRRQFRFR    60
NP_766481.2[M.musculus]         ------------------------------------------------------------    0
NP_073587.1[H.sapiens]          ------------------------------------------------------------    0
XP_024106109.1[P.abelii]        ------------------------------------------------------------    0


XP_019640190.1[B.belcheri]      --------------------GRGGRGGARGGR-------GMDRGRGRGGFYSPQQV----    80
XP_032833032.1[P.marinus]       --------------MQS---SYPQEGQQQQEKPPLPLDRALDTLHDSGCALPLSALASAL    43
XP_014003371.1[S.salar]         -------------------------------MSSIVSQYVTKILCGNQGCLDFKRLDQIV    29
XP_018096331.1[X.laevis]        -------MW-------------------TNQLIMVHTARLSRRLCSAGGSMELGQLGHSL    34
XP_416333.5[G.gallus]           FLAQEAREWRRRSRFRARPAGSGSAGGKQPGPAMVLPTQVLRVLCAGGGSLELEELLRRL    120
NP_766481.2[M.musculus]         -----------------------------MAQAAVAVAEVTQLLCAAGGALELAELRRRL    31
NP_073587.1[H.sapiens]          -----------------------------MA-QAGVVGEVTQVLCAAGGALELPELRRRL    30
XP_024106109.1[P.abelii]        -----------------------------MA-QAGVVGEVTQVLCAAGGALELPELRRRL    30
                                                                       :

XP_019640190.1[B.belcheri]      ------PPPA-SGPMVANEAKFSM-------------------------NDVM-------    101
XP_032833032.1[P.marinus]       GF----PDSALLAALRSKPELFALVEGSGSSSSSLSGGQQ-QQPERGSLDWRVLAVSALR    98
XP_014003371.1[S.salar]         GQKFTVADDVLLG-ILCDREKYAI--------S---KGEGKASRLAMSQNSVIVAKTSLR    77
XP_018096331.1[X.laevis]        G--LN--GQQLER-LVEVEEGRSL-----------------VLRVQEGDQKVAVYRSDLR    72
XP_416333.5[G.gallus]           PGRPT--AEQLAA-VLRDPQRFTLVRTPDEAEA---GGAAAAAAAAAEETVLVVATSSLR    174
NP_766481.2[M.musculus]         RTSLG--TDALER-LLRDCGRFVVASRAVVAVG---AGREA---AAAASERLVLAVSSLR    82
NP_073587.1[H.sapiens]          RMGLS--ADALER-LLRQRGRFVVAVRAG-----------G---AAAAPERVVLAASPLR    73
XP_024106109.1[P.abelii]        RMGLS--ADALER-LLRQRGRFVVAVRAG-----------D---AAAAPERVVLAVSPLR    73
                                              :        :

XP_019640190.1[B.belcheri]      ---------------------QLMQAMHMGAAPQMPQAQPEAAMVHPQAPPPS------G    134
XP_032833032.1[P.marinus]       VCPRYRE-RTGCPDGEGCAKLHVCRRFVLGSCK-F---GPDCKFSHDFRDGHNRDLCYKS    153
XP_014003371.1[S.salar]         VCQSP----PGE--CLHCENLHLCRYFVCGNCR-F---GNKCKNAHSLDSPHNTAVIRSL    127
XP_018096331.1[X.laevis]        LCQT-----PKC--AGDCGQLHLCRFYILGSCN-----RSPCKFNHCIRNGHNLSVLQKF    120
XP_416333.5[G.gallus]           LCLEHG---AGC--RGGCGRLHLCKYHLKGVCR-NQQARKECKFVHDFYSPHNHSVLRQH    228
NP_766481.2[M.musculus]         LCRAHQGPKPGC--TGLCAQLHLCKFLIYGNCK-FLKTGKNCRNGHNLKTDHNLSVLRTH    139
NP_073587.1[H.sapiens]          LCRAHQGSKPGC--VGLCAQLHLCRFMVYGACK-FLRAGKNCRNSHSLTTEHNLSVLRTH    130
XP_024106109.1[P.abelii]        LCRAHQGSKPGC--VGLCAQLHLCRFMVYGACK-FLKAGKNCRNSHSLATEHNLSVLRTH    130
                                                     :: :    * .         .   *      .

XP_019640190.1[B.belcheri]      GARS-RPRSRQRTAHNDNPPPVPAKLPQACTWYNNRQ---RGCTKGDACTFLHVCGRYLV    190
XP_032833032.1[P.marinus]       GVEA-LPFDPMQTL---LLQNDPTLLPQICKTYKVLD-AAGECKYGNKCARLHLCRPFVL    208
XP_014003371.1[S.salar]         GHHD-LGEAELFPL---LLQNDPYLMPEICSHYNKGVGEHGSCKFKTSCTSLHLCLHFLQ    183
XP_018096331.1[X.laevis]        HLDS-MPIDELRQL---LLQNDPNLLPDICSHYNRGDGPHGSCTYKNKCNKLHVCQHFLQ    176
XP_416333.5[G.gallus]           GLDSTFSNDELRQL---LLQNDPTLLPEVCLHYNKGDGPYGSCTFKKMCTKLHVCQYFLR    285
NP_766481.2[M.musculus]         GVDH-LTYTELCQL---LLQNDPSLLPDICLHYNKGDGPFGSCSFQKQCIKLHICQYFLQ    195
NP_073587.1[H.sapiens]          GVDH-LSYNELCQL---LFQNDPWLLPEICQHYNKGDGPHGSCAFQKQCIKLHICQYFLQ    186
XP_024106109.1[P.abelii]        GVDH-LSYNELCQL---LFQNDPWLLPEICQHYNKGDGPHGSCAFQKQCIKLHICQYFLQ    186
                                                      *  :*: *  *:        *     *  **:*  ::

XP_019640190.1[B.belcheri]      GGCIFDKGQCEFSHNILTQQNKGILQKFGIPCNSEKAVIDIFKRDFVNKRKAEQ------    244
XP_032833032.1[P.marinus]       GVCKFGD-KCRRNHGLSDAGLRRLLKQHGVCAWPDAALLGLLRRREQIHAAAGGGADAAD    267
XP_014003371.1[S.salar]         GDCKFGA-GCKRAHTFDAT-TMKILNGRGFSQENIRILDKIYNNKFIIIGQGHEEKPSVV    241
XP_018096331.1[X.laevis]        GDCKFGE-KCKRCHDLSEEETLKKLTKWQLSDSLLPGLLETYTNAHTLKSSCDR--PPRN    233
XP_416333.5[G.gallus]           GQCRFGS-SCKRSHDFSKPECCEKLERQGMSSDIIQKLPSIYRNMYAIQNSKGS--KHDT    342
NP_766481.2[M.musculus]         GECKFGT-SCKRSHEFTNSESLEQLERLGLSSDLVSRLLSTYRNAYDIKNKGSA--LSKV    252
NP_073587.1[H.sapiens]          GECKFGT-SCKRSHDFSNSENLEKLEKLGMSSDLVSRLPTIYRNAHDIKNKSSA--PSRV    243
XP_024106109.1[P.abelii]        GECKFGT-SCKRSHDFSNSENLEKLEKLGMSSDLVSRLPTIYRNAHDIKNKSSA--PSRV    243
                                * * *.   *.  * :        *    .       :     .

XP_019640190.1[B.belcheri]      -------------------------------GDKVPEKLDEICIFNIRQRCSYKTLCERW    273
XP_032833032.1[P.marinus]       DGATIAPLGFAARAAARGRKAGGSGGDGWPAGAAGRDGEGEICLYHLLSKCHYKDRCLKS    327
XP_014003371.1[S.salar]         VPSPASAIEPVGKERSRK----------PSSRSISETDRNEICLFFIRRHCSFKEKCVRI    291
XP_018096331.1[X.laevis]        V----V--KSAEKKTKPGP---------S--KSGTSQEIEEICLYFIRNSCSFKEKCVRE    276
XP_416333.5[G.gallus]           EDNKSLPC--KERKHSSS-----------QESTSTSNEVEQICLYNLYTSCIFEDKCIRT    389
NP_766481.2[M.musculus]         SPSPAGPQGSSERKDSSGP---------VSPGTPSQEESEQICLYHIRKSCSFQEKCHRV    303
NP_073587.1[H.sapiens]          PP-LFVPQGTSERKDSSGS---------VSPNTLSQEEGDQICLYHIRKSCSFQDKCHRV    293
XP_024106109.1[P.abelii]        PP-PFVPQGTSERKDSSGS---------VSPNTLSQEESDQICLYHIRKSCSFQDKCHRV    293
                                                                        :**:: :   * ::  * :

XP_019640190.1[B.belcheri]      HCRMPYQWQYRAPAADRWRHFGHDLNVMVEEAYCKVTEDSYSPVRL--DGKTCAVNFTDM    331
XP_032833032.1[P.marinus]       HAALPYRWQERSHGGGAWQDLPG--TEAMERHYCDPASVS-----------HDSVDFIHM    374
XP_014003371.1[S.salar]         HYHLPYKWQILERDGVTWRDLPN--VEEIEKAYCIPGKDTSGG--------IQPVNFLTM    341
XP_018096331.1[X.laevis]        HFHLPYRWQVY--TNGTWKDLDN--MEMIEKSYCDPNSRAV----------LVNLDFEAM    322
XP_416333.5[G.gallus]           HFHLPYRWQIS--DGTTWKDLES--MEEIEKEYCDPNNIRSSKPISESGFIFPYISFVDM    445
NP_766481.2[M.musculus]         HFHLPYRWQFL--DGGKWKDLDN--MELIEEAYSNPSKDRIVYTESAAGFHFDNLDFNSM    359
NP_073587.1[H.sapiens]          HFHLPYRWQFL--DRGKWEDLDN--MELIEEAYCNPKIERILCSESASTFHSHCLNFNAM    349
XP_024106109.1[P.abelii]        HFHLPYRWQFL--DRGKWKDLDN--MELIEEAYCNPKIERILCSESASTFHSDCLNFNAM    349
                                *  :**:**        *..:       :*. *.                    :.*  *

XP_019640190.1[B.belcheri]      KAVATDGSTGDTRQEFEVRRLSTSSSVKSAPGHLFVTEWLWYWHDVDGTWVEYGQMNKSG    391
XP_032833032.1[P.marinus]       KSV-----------HGTVRRLSTANAVAMPDNFLLTTRWSWYWQECVGTWIKYGDEATTN    423
XP_014003371.1[S.salar]         TCG-----------RSPVRRLSTVSSVTKPPHFILTTEWLWYWSGELGQWIEYGQETYDG    390
XP_018096331.1[X.laevis]        TYQ-----------SNKVKRLSTPSSASKPPHFVFTTDWKWYWMDEYNKWVEYGTESDR-    370
XP_416333.5[G.gallus]           QSG-----------FKKVRRLSTASSVTKPPHFILTTEWIWYWKDEHGVWREYGKKDSD-    493
NP_766481.2[M.musculus]         KFG-----------NTLARRLSTASSVTKPPHFILTTDWIWYWMDEFGSWQEYGRQGSG-    407
NP_073587.1[H.sapiens]          TYG-----------ATQARRLSTASSVTKPPHFILTTDWIWYWSDEFGSWQEYGRQGTV-    397
XP_024106109.1[P.abelii]        TYG-----------ATQARRLSTASSVTKPPHFILTTDWIWYWSDEFGSWQEYGRQGTA-    397
                                                 .:**** .:.     .::.* * ***    . * :**

XP_019640190.1[B.belcheri]      EVRGTSSLTSRDIEDRYQD-------QRDADVTFSTSHHQYVLRLRDMCQENTRVGTRRD    444
XP_032833032.1[P.marinus]       D-RAKSNVSSLDLEKAYHE-------DKEGSFKFTAGRFSYRLDFKDMTQTNLEVGTRRC    475
XP_014003371.1[S.salar]         K-EKVASVTSKTLENVYQA-------DPDSEIPFGSGHNQYILRFKEMYQQNVRFKTKRE    442
XP_018096331.1[X.laevis]        --HANSTICSSDLENFYQS-------NQTADVKFKAGKHKYSLSFKNMVQRNLQYETKRR    421
XP_416333.5[G.gallus]           --RAAATVSSDDLEKAYTV-------GNSAKLNFKAGVHEYELDFGAMTQKNLRYKTERK    544
NP_766481.2[M.musculus]         --HPVTTISSSDVERAYLAFCAPGADAQAATLKFQAGKHNYELHFKAFLQKNLVYGTIRK    465
NP_073587.1[H.sapiens]          --HPVTTVSSSDVEKAYLAYCTPGSDGQAATLKFQAGKHNYELDFKAFVQKNLVYGTTKK    455
XP_024106109.1[P.abelii]        --HPVTTVSSSDVEKAYLAYCTPGSDGQAATLKFQAGKHNYELDFKAFVQKNLVYGTTKK    455
                                  .  :.: *  :*  *            . . * :.  .* * :  : * *    * :

XP_019640190.1[B.belcheri]      VRRRPKFVSEEDVKNPPKPKPASRAKSARSASAWDESAPAHWSPMSD-DDEFVKVDVVGT    503
XP_032833032.1[P.marinus]       VRRRPLLVTARDVEVARERHAS-------LSRTGVGSVPAHWDRASLSDVDISLVELVNT    528
XP_014003371.1[S.salar]         VRRRPCFLSGQDVEAKLKSGSP------ESTSSSTVSVPPHWDKGALPDFTYKLVPLLDQ    496
XP_018096331.1[X.laevis]        VCRRPVFVSGKDVKKKRSSTSE------P--SKEDKNTPEHWDKGQTPDLGYKLVLLSPL    473
XP_416333.5[G.gallus]           VCRRPKFVSQSEVERIRKRGFK------N--TEELRRIPSHWDKSALPELGFKLIELDSS    596
NP_766481.2[M.musculus]         VCRRPKYVSPQDVQMKQSCNT-------K--LHGPKSIPDYWDPAALPDLGFKKITLSSS    516
NP_073587.1[H.sapiens]          VCRRPKYVSPQDVTTMQTCNT-------K--FPGPKSIPDYWDSSALPDPGFQKITLSSS    506
XP_024106109.1[P.abelii]        VCRRPKYMSPQDVTTMQTCNT-------K--FPGPKSIPDYWDSSALPDPGFQKITLSSS    506
                                * ***  ::  :*                         * :*.     :     : :

XP_019640190.1[B.belcheri]      SAEYRNVKTLFEQGGM-AGTAIAGVKRVQNAFLWSAYNRKKAQLKKQNGGKDAEERLLFH    562
```

```
XP_032833032.1[P.marinus]       TSEYKEVATLFKNTLSQPSINILNVKRVQNLELWDAFQRKREWMRKKNDGVEVEERKLFH    588
XP_014003371.1[S.salar]         MTEYQMVQSLFKRTMST--STIHKINRIQNPSLWRVFQWQKEQMKVKNGGKLVDERHLFH    554
XP_018096331.1[X.laevis]        SEEYSKIEAMFCRTLRT--IRIHSIERIQNLALWEVYQWQKEQMKKLNEGKDPEERQLFH    531
XP_416333.5[G.gallus]           SEEYNKVKGDFQRTMPK--THIKRICRIQNPSLWELYQWQKEQMQKSNGGK-TDERFLFH    653
NP_766481.2[M.musculus]         SEEYQKVWNIFNRTLPF--YFVQKIERIQNMGLWEVYQWQKCQMQKQNGGKEVDERQLFH    574
NP_073587.1[H.sapiens]          SEEYQKVWNLFNRTLPF--YFVQKIERVQNLALWEVYQWQKGQMQKQNGGKAVDERQLFH    564
XP_024106109.1[P.abelii]        SEEYQKVWNLFNRTLPF--YFVQKIERVQNLALWEVYQWQKGQMQKQNGGKAVDERQLFH    564
                                  **  :   * .        :  : *:**  **  :: ::  ::  * *   :** ***

XP_019640190.1[B.belcheri]      GTQDAVVDAICQQNFDWRLSGSRVGQLYGQGTYFSASAQYSHSYAQQA---SNGRRYMFV    619
XP_032833032.1[P.marinus]       GTRPKHVVPICQQNIDWRLRGA-HGTAYGEGSYFARDASYSHTYSKGRKGTAPASAAMFL    647
XP_014003371.1[S.salar]         GTEPSIVDAICEQNFDWRVCGI-HGSHYGKGSYFARDALYSNRFSKSS---VSGKKIMFV    610
XP_018096331.1[X.laevis]        GTKVKLIDAICQQNFDWRICGV-HGTAYGKGSYFARDSSYSHNYCKRS---SSQANIMFV    587
XP_416333.5[G.gallus]           GTSKKHIDAICHQNFDWRICGL-NGTVYGKGSYFARDASYSDAYCREG----SCSKTMFQ    708
NP_766481.2[M.musculus]         GTSANFVDAICQQNFDWRVCGL-HGTSYGKGSYFARDAAYSHHYSKSD----THSHMMFL    629
NP_073587.1[H.sapiens]          GTSAIFVDAICQQNFDWRVCGV-HGTSYGKGSYFARDAAYSHHYSKSD----TQTHTMFL    619
XP_024106109.1[P.abelii]        GTSTIFVDSICQQNFDWRVCGV-HGTSYGKGSYFARDAAYSHHYSKSD----TQTHTMFL    619
                                **    :  **.**:***: *   *  **:*:**: .: **. :.:           **

XP_019640190.1[B.belcheri]      VRVLVGAYTQGDAGIRRPPPVNPG-------EPYGRMYDSCVNDTANPNIFVIFDNAQCY    672
XP_032833032.1[P.marinus]       ARVLVGRYTCGKAGYVRPPAIKASPSTSAGQPAPEVFYDSCVNTEIDPSIFVVFERDQIY    707
XP_014003371.1[S.salar]         ALVLVGEFTKGNRDYLRPPQKGTK----------KRLYDSCVDSESNPAIFVVFEKQQIY    660
XP_018096331.1[X.laevis]        ARVLVGNFTRGHSSYLRPPSKSEF--------RSTSFYDSCVDSKSNPSIFVIFEKHQIY    639
XP_416333.5[G.gallus]           ARVLVGEFTVGRSSYVRPPLKDN-----------QNFYDSCVNSSSNPSIFVIFEKQQVY    757
NP_766481.2[M.musculus]         ARVLVGDFVRGSTSFVRPPAKEGQ---------SNAFYDSCVNSMSDPTIFVVFEKHQVY    680
NP_073587.1[H.sapiens]          ARVLVGEFVRGNASFVRPPAKEGW---------SNAFYDSCVNSVSDPSIFVIFEKHQVY    670
XP_024106109.1[P.abelii]        ARVLVGEFVRGNASFVRPPAKEGW---------SNAFYDSCVNSVSDPSIFVIFEKHQVY    670
                                . **** :. *  .  ***                 :*****:   :* ***:*:. * *

XP_019640190.1[B.belcheri]      PEHIIEY------------------------         679
XP_032833032.1[P.marinus]       PEYVIEYQF----------------------         716
XP_014003371.1[S.salar]         PEFVIEYS-----------------------         668
XP_018096331.1[X.laevis]        PEYLIKYSDK---------------------         649
XP_416333.5[G.gallus]           PEYLIEYLDQACARML---------------         773
NP_766481.2[M.musculus]         PEYLIQYSTSSKPPASPSIFVALGNLFTSRQ         711
NP_073587.1[H.sapiens]          PEYVIQYTTSSKPSVTPSILLALGSLFSSRQ         701
XP_024106109.1[P.abelii]        PEYLIQYSTSSKPSVTPSILLALGSLFSRQ-         700
                                **.:*:*
```

Percent identity matrix:

```
#
#
#  Percent Identity  Matrix - created by Clustal2.1
#
#

     1: XP_019640190.1[B.belcheri]  100.00   33.28   34.61   33.98   32.97   31.43   33.04   32.87
     2: XP_032833032.1[P.marinus]    33.28  100.00   37.08   39.00   38.62   38.58   39.63   39.63
     3: XP_014003371.1[S.salar]      34.61   37.08  100.00   45.92   45.40   45.64   46.13   46.13
     4: XP_018096331.1[X.laevis]     33.98   39.00   45.92  100.00   50.94   51.88   51.49   51.64
     5: XP_416333.5[G.gallus]        32.97   38.62   45.40   50.94  100.00   54.20   54.41   54.41
     6: NP_766481.2[M.musculus]      31.43   38.58   45.64   51.88   54.20  100.00   81.03   81.43
     7: NP_073587.1[H.sapiens]       33.04   39.63   46.13   51.49   54.41   81.03  100.00   97.71
     8: XP_024106109.1[P.abelii]     32.87   39.63   46.13   51.64   54.41   81.43   97.71  100.00
```
