# Supplementary material for: An Evolutionary Perspective on the Origin, Conservation and Binding Partner Acquisition of Tankyrases
Source: Biomolecules. 2022 Nov 15;12(11):1688. doi: 10.3390/biom12111688 (PMC9688111; doi:10.3390/biom12111688)
Supplement: Supplementary file 1 [file biomolecules-12-01688-s001.zip › biomolecules-1913719-supplementary-11.17 -done/Data_2/PARP16.pdf]

# PARP16

## **Sequences:**

```
>NP_001303872.1 protein mono-ADP-ribosyltransferase PARP16 isoform 2 [Homo sapiens]
MQPSGWAAAREAAGRDMLAADLRCSLFASALQSYKRDSVLRPFPASYARGDCKDFEALLADASKLPNLKE
LLQSSGDNHKRAWDLVSWILSSKVLTIHSAGKAEFEKIQKLTGAPHTPVPAPDFLFEIEYFDPANAKFYE
TKGERDLIYAFHGSRLENFHSIIHNGLHCHLNKTSLFGEGTYLTSDLSLALIYSPHGHGWQHSLLGPILS
CVAVCEVIDHPDVKCQTKKKDSKEIDRRRARIKHSEGGDIPPKYFVVTNNQLLRVKYLLVYSQKPPKRAS
SQLSWFSSHWFTVMISLYLLLLLIVSVINSSAFQHFWNRAKR

>XP_009248197.1 mono [ADP-ribose] polymerase PARP16 isoform X2 [Pongo abelii]
MQPSGWAAAREAAGRDMLAADLRCSLFASALQSYKRDSVLRPFPSSYARGDCKDFESLLADASKLPNLKE
LLQSSGDNHKRAWDLVSWILSSKVLTIHSAGKAEFEKIQKLTGAPHTPVPAPDFLFEIEYFDPANAKFYE
TKGERDLIYAFHGSRLENFHSIIHNGLHCHLNKTSLFGEGTYLTSDLSLALIYSPHGHGWQHSLLGPILS
CVAVCEVIDHPDVKCQTKKKDSKEIDRRRARIKHSEGGDIPPKYFVVTNNQLLRVKYLLVYSQKPPKRAS
SQLSWFSSHWFTIMISLYLLLLLIVSVINSSAFQHFWNRAKK

>AAH55447.1 Poly (ADP-ribose) polymerase family, member 16 [Mus musculus]
MQLSNRAAAREAASRDVLAADLRCSLFASALQSYKRDSVLRPFPASYARHDCKDFEALLADTGRLPNLKE
LLQSSRDTDKQAWDLVSWILSSKILTIHSAKKAEFEKIQQLTGAPHTPVPTPDFLFEIEYFDPANSRFYE
TKGERDLIYAFHGSRLENFHSIIHNGLHCHLNKTSLFGEGTYLTSDLSLALIYSPHGHGWQHSLLGPILS
CVAVCEVIDHPDVKCQIKKKDSKEIDRSRARIKHSEGGEIPPKYFVVTNNQLLRVKYLLVYSQKQPKRAS
SQLSWLSSHWFVIMMSLYLLLLLIVSVTNSSAFHHFWNRVKR

>XP_015147741.1 protein mono-ADP-ribosyltransferase PARP16 isoform X2 [Gallus gallus]
MGPPRLGEAVREAVRRDPLGAELRCSLFAAALQSYRRDSVLRPFPSRYGGADAASKDFEALLADTEALPG
LKELLGAAPSAEERTWDLLSWILSPKTFTIQSIKKQEYEKIQELTGMSGAAVPAPDFLFEVTYCDQMNAK
FAETRGEQDLIYAFHGSRLENFHSILHNGLHCHLNRTSLFGEGTYLTSDLSLALLYSPHGLGWQRSTLGS
ILSCVAVCEIIDHPDVKCQVKKKDSEEIDRKRARVKNSEGGDVPQKYFVVTNNQLLQVKYLLVYSQKQHR
RPSGQSSWFSTHRFAITMMLYLLLLMVIGASNSPAFLYYWHRMFYSEG

>XP_018108776.1 protein mono-ADP-ribosyltransferase PARP16 [Xenopus laevis]
MVEEIITRDLLSADLKCSLFVAALKSYKRDSVLRPFPALYTSDQYKDFDALITDSSALPGLPEIIQDKNN
RDQKVLNLLRWILSDKLFTVKSVPKDKYEEIQALPGSPSHALPTPDFLFELEYCEKLSAKFQETRGDRDL
IYAFHGSRLENFHSILHNGLHCHLNKTSLFGEGTYLTSDLGLALLYSPHGQGWTRSMLGPVLSCVAMCEM
IDHPDVKCQAKKKDSSEIDRKRARARNSEGGDVPQKYFVVTNNQLLRVKYLMVYSQEQPRRRPRQPTWFS
KHRFAIMMTLYLFILILIGLSSSKAFTYHWNRLLGWRP

>XP_045544515.1 protein mono-ADP-ribosyltransferase PARP16-like isoform X2 [Salmo salar]
MQPPLPPDAVRELVCSCLHRDPVAADLRCSLFVAAAQNYKRDSVLRPYPPRYIRDRDTKEFDELLSDVSS
LPGVRELVRLRPGEADHHLALTHWVLSSKSFAVKTLQKDEYAKLCNLTESEGISTPVPDFLFELEYCDQM
NAKFEKTRAGRDLFYAFHGSRLENFHSIIHNGLHCHLNKTSLFGEGTYLTSDLSMAVLYSPHGNGWRDSL
LGPLLSCVAVCEVIDHPDVKCQVKRKDSETIDRQRSRARNSEGGEVPQKYFVVTNNQLLRVKYLLVYSQR
RHLSRHSRGRSWLVRHHFAIMMSLYLLLLIVIGAFNSTTFLSFWNRLFR

>XP_032804273.1 protein mono-ADP-ribosyltransferase PARP16 isoform X2 [Petromyzon marinus]
MLAEEARGWEPANQAAEEEGGGGGGEGGDDHEDGDRVGPRGPGDAVREALRGDPGAADLRLSLVAAALLG
YRRHSLLRPFPSGYGNGGEDGKDFTALLADMSAVPSVQQLLREDVDVAPRILELLHWALTSKHFTVCSQR
KDEFSKIEALTGSPGVPVPAPDFVFSVTYSETAERRFREMRGELDILYAYHGSRLENFHSILHNGLHCHL
NKTALFGEGTYLTSDLSLALLYSPHSQAWRLSALGAALSCVAVCEVIDHPDVKCQVKRKDADGLDRRRAR
ARHSEGGDVPQKYYVVTNNQLLRVRYLLLYARKSQHHSIPWQRSWLGRHRFAVGMAVYGLILLLLGALNS
PTVLHYWRRYLSGD

>XP_019627055.1 PREDICTED: mono [ADP-ribose] polymerase PARP16-like isoform X2 [Branchiostoma belcheri]
MTEDVRTNVREKLEGDVLAADLQLSLFAAALLSYRHDTVLRPFPPGFARQNDEKDFPALKSVWKGLSGVK
ELLQDSRVTSDAQTLQLVHWVLETRNFHLRSCEPAEYKEVQRLTGYTSTNIPPPSHMFEVVHSESTDARF
EETRQDRSLFYAFHGSRLDNFHSILHNGLHAHLNKNSLFGEGTYLSGDLGVSIIYSHKGQGWDRSMLGET
MSCVAVCEVIMDPKYVKSKVEEENGRAKNKDKHEVPEKYYIVSNNELLRVKYVLVYAERKARPRVQAPSF
FQRHKFFILMTLYVIVLAVIGAANSPNLQYYLRKFFR

>XP_032233915.1 protein mono-ADP-ribosyltransferase PARP16 [Nematostella vectensis]
MDDSVEKETQLERVIGKLRSDGFACDLRMCLFVAALESYRHDSILRPYPPVGLREDGSKNIQKLTNLSSS
IPSLSALNNDSCLEPGVWSLLDWVLLKKFDVKTLDKSMFQEIEKKTGHSSYNSTEPDYIFEIQYHENNAL
NARFLALSEECEVLYAYHGSRLENFHSILHNGLHSHLNKNSLFGEGTYLSSDLNVSIMYSTYSQAWSNSG
LGEKLSCVAVCEVLNHPDVKCSLEDPRAATSSPERRRSGSGASSVRARAKGSEGGDVPDRYYVVQNNQLL
RVKYLLVYAEKRPQRNTQRTPPGTWFARLLIIYIIGLVLLGLSRTRAFHKLASYLQKEDTHL

>XP_002164087.1 protein mono-ADP-ribosyltransferase PARP16 [Hydra vulgaris]
MTSISEQVSIILSNDLLAAELKLALFTSALNSYRHDSCLRPFPNIFGDTCDKNIAVKQMKSLLSEFRPLA
EMVNIISLMSEDCQKMIHWALDSKIYSSKTCNKHVFDDVQKKTGCSSYQVQPDYIFEIKYNTFSSQKFDD
LAKMYGVKYGFHGSRMDNFHSIVNNGLQVHMVKNAVFGEGVYLSEDLSVSMPYISSGFTWQHSKLGQQIS
CVAVCEVLDHPGVKCTMKTVNGANRSRANNSEAGEVPKKYFVVTSSDLVRVKYIFVFTDKTEKRNSAKCG
MLCFRYPVLSIMLAYMLLLAFVSIWNSKTFQLIWRNYWYKKKF

>XP_019855808.1 PREDICTED: mono [ADP-ribose] polymerase PARP16-like [Amphimedon queenslandica]
MSKDDGGDASTGTLQLKVNELREALSKDMAGCDCLLSLFWSALSSYRHDTVLRPYPNQFILSNGDKDIDG
LRSVFKTLPGLNEVVQQLDSLDDRVITLLYDIICTKGFRLSFKPGDKFSLIESQTSVPAHKVQPYCFFEL
QYSSEREEKWQRTASEYGTFLAYHGSRMENFHSIAHNGLLSHMNKVGIFGEGTYLSTDLSVCMNFSPSAT
SWTKSSLPPLCSMMAVCEVVSHPDIRSRVQHGGEPSINQKVPERYFVVTNDEMLRVKYILVFAQERGRPS
PTRRWFSNHKWVLMVFLYLFLLLFLGLWNSRYVSRLYNQYTRRYITL
```

## Alignment

```
XP_019855808.1[A.queenslandica]      ---------------MSKDDGGDASTG----------TLQLKVNELREALSKDMAGCDCL          35
XP_002164087.1[H.vulgaris]           --------------------------------------MTSISEQVSIILSNDLLAAELK          22
XP_032233915.1[N.vectensis]          --------------------------------MDDSVEKETQLERVIGKLRSDGFACDLR          28
XP_019627055.1[B.belcheri]           -------------------------------------MTEDVRTNVREKLEGDVLAADLQ          23
XP_032804273.1[P.marinus]            MLAEEARGWEPANQAAEEEGGGGGGEGGDDHEDGDRVGPRGPGDAVREALRGDPGAADLR          60
XP_045544515.1[S.salar]              --------------------------------MQPPLPPDAVRELVCSCLHRDPVAADLR          28
XP_018108776.1[X.laevis]             --------------------------------------------MVEEIITRDLLSADLK          16
XP_015147741.1[G.gallus]             ------------------------------------MGPPRLGEAVREAVRRDPLGAELR          24
AAH55447.1[M.musculus]               -------------------------------------MQLSNRAAAREAASRDVLAADLR          23
NP_001303872.1[H.sapiens]            -------------------------------------MQPSGWAAAREAAGRDMLAADLR          23
XP_009248197.1[P.abelii]             -------------------------------------MQPSGWAAAREAAGRDMLAADLR          23
                                                                                         *  ..:

XP_019855808.1[A.queenslandica]      LSLFWSALSSYRHDTVLRPYPNQFILSN-GDKDIDGLRSVFKTLPGLNEVVQQLD-SLDD          93
XP_002164087.1[H.vulgaris]           LALFTSALNSYRHDSCLRPFPNIFGDTCDKNIAVKQMKSLLSEFRPLAEMVNIIS-LMSE          81
XP_032233915.1[N.vectensis]          MCLFVAALESYRHDSILRPYPPVGLRED-GSKNIQKLTNLSSSIPSLSALNNDSC--LEP          85
XP_019627055.1[B.belcheri]           LSLFAAALLSYRHDTVLRPFPPGFARQN-DEKDFPALKSVWKGLSGVKELLQDSRVTSDA          82
XP_032804273.1[P.marinus]            LSLVAAALLGYRRHSLLRPFPSGYGNGGEDGKDFTALLADMSAVPSVQQLLREDV-DVAP          119
XP_045544515.1[S.salar]              CSLFVAAAQNYKRDSVLRPYPPRYIRDR-DTKEFDELLSDVSSLPGVRELVRLRP-GEAD          86
XP_018108776.1[X.laevis]             CSLFVAALKSYKRDSVLRPFPALYTSDQ--YKDFDALITDSSALPGLPEIIQDKN-NRDQ          73
XP_015147741.1[G.gallus]             CSLFAAALQSYRRDSVLRPFPSRYGGADAASKDFEALLADTEALPGLKELLGAAP-SAEE          83
AAH55447.1[M.musculus]               CSLFASALQSYKRDSVLRPFPASYARHD--CKDFEALLADTGRLPNLKELLQSSR-DTDK          80
NP_001303872.1[H.sapiens]            CSLFASALQSYKRDSVLRPFPASYARGD--CKDFEALLADASKLPNLKELLQSSG-DNHK          80
XP_009248197.1[P.abelii]             CSLFASALQSYKRDSVLRPFPSSYARGD--CKDFESLLADASKLPNLKELLQSSG-DNHK          80
                                      .*. :*  .*::.: ***:*            .  :      .  :  :

XP_019855808.1[A.queenslandica]      RVITLLYDIICTKGFRLSFKPGDKFSLIESQTSVPAHKV-QPYCFFELQYSSE--REEKW          150
XP_002164087.1[H.vulgaris]           DCQKMIHWALDSKIYSSKTCNKHVFDDVQKKTGCSSYQV-QPDYIFEIKYNTF--SSQKF          138
XP_032233915.1[N.vectensis]          GVWSLLDWVLL-KKFDVKTLDKSMFQEIEKKTGHSSYNSTEPDYIFEIQYHENNALNARF          144
XP_019627055.1[B.belcheri]           QTLQLVHWVLETRNFHLRSCEPAEYKEVQRLTGYTSTNIPPPSHMFEVVHSES--TDARF          140
XP_032804273.1[P.marinus]            RILELLHWALTSKHFTVCSQRKDEFSKIEALTGSPGVPVPAPDFVFSVTYSET--AERRF          177
XP_045544515.1[S.salar]              HHLALTHWVLSSKSFAVKTLQKDEYAKLCNLTESEGISTPVPDFLFELEYCDQ--MNAKF          144
XP_018108776.1[X.laevis]             KVLNLLRWILSDKLFTVKSVPKDKYEEIQALPGSPSHALPTPDFLFELEYCEK--LSAKF          131
XP_015147741.1[G.gallus]             RTWDLLSWILSPKTFTIQSIKKQEYEKIQELTGMSGAAVPAPDFLFEVTYCDQ--MNAKF          141
AAH55447.1[M.musculus]               QAWDLVSWILSSKILTIHSAKKAEFEKIQQLTGAPHTPVPTPDFLFEIEYFDP--ANSRF          138
NP_001303872.1[H.sapiens]            RAWDLVSWILSSKVLTIHSAGKAEFEKIQKLTGAPHTPVPAPDFLFEIEYFDP--ANAKF          138
XP_009248197.1[P.abelii]             RAWDLVSWILSSKVLTIHSAGKAEFEKIQKLTGAPHTPVPAPDFLFEIEYFDP--ANAKF          138
                                         :    :  :           :  :             *  .*.: :      . ::

XP_019855808.1[A.queenslandica]      QRTASEYGTFLAYHGSRMENFHSIAHNGLLSHMNKVGIFGEGTYLSTDLSVCMNFSPSAT          210
XP_002164087.1[H.vulgaris]           DDLAKMYGVKYGFHGSRMDNFHSIVNNGLQVHMVKNAVFGEGVYLSEDLSVSMPYISSGF          198
XP_032233915.1[N.vectensis]          LALSEECEVLYAYHGSRLENFHSILHNGLHSHLNKNSLFGEGTYLSSDLNVSIMYSTYSQ          204
XP_019627055.1[B.belcheri]           EETRQDRSLFYAFHGSRLDNFHSILHNGLHAHLNKNSLFGEGTYLSGDLGVSIIYSHKGQ          200
XP_032804273.1[P.marinus]            REMRGELDILYAYHGSRLENFHSILHNGLHCHLNKTALFGEGTYLTSDLSLALLYSPHSQ          237
XP_045544515.1[S.salar]              EKTRAGRDLFYAFHGSRLENFHSIIHNGLHCHLNKTSLFGEGTYLTSDLSMAVLYSPHGN          204
XP_018108776.1[X.laevis]             QETRGDRDLIYAFHGSRLENFHSILHNGLHCHLNKTSLFGEGTYLTSDLGLALLYSPHGQ          191
XP_015147741.1[G.gallus]             AETRGEQDLIYAFHGSRLENFHSILHNGLHCHLNRTSLFGEGTYLTSDLSLALLYSPHGL          201
AAH55447.1[M.musculus]               YETKGERDLIYAFHGSRLENFHSIIHNGLHCHLNKTSLFGEGTYLTSDLSLALIYSPHGH          198
NP_001303872.1[H.sapiens]            YETKGERDLIYAFHGSRLENFHSIIHNGLHCHLNKTSLFGEGTYLTSDLSLALIYSPHGH          198
XP_009248197.1[P.abelii]             YETKGERDLIYAFHGSRLENFHSIIHNGLHCHLNKTSLFGEGTYLTSDLSLALIYSPHGH          198
                                                .:****::***** :***  *: : .:****.**: **.:.: :   .

XP_019855808.1[A.queenslandica]      SWTKSSLPPLCSMMAVCEVVSHPDIRSRVQHGG---------------------EPSIN          248
XP_002164087.1[H.vulgaris]           TWQHSKLGQQISCVAVCEVLDHPGVKCTMKTVN---------------GANRSRANNSEA          243
XP_032233915.1[N.vectensis]          AWSNSGLGEKLSCVAVCEVLNHPDVKCSLEDPRAATSSPERRRSGSGASSVRARAKGSEG          264
XP_019627055.1[B.belcheri]           GWDRSMLGETMSCVAVCEVIMDPKYVK------------------SKVEEENGRAKNKDK          242
XP_032804273.1[P.marinus]            AWRLSALGAALSCVAVCEVIDHPDVKCQVKRKD-----------ADGLDRRRARARHSEG          286
XP_045544515.1[S.salar]              GWRDSLLGPLLSCVAVCEVIDHPDVKCQVKRKD-----------SETIDRQRSRARNSEG          253
XP_018108776.1[X.laevis]             GWTRSMLGPVLSCVAMCEMIDHPDVKCQAKKKD-----------SSEIDRKRARARNSEG          240
XP_015147741.1[G.gallus]             GWQRSTLGSILSCVAVCEIIDHPDVKCQVKKKD-----------SEEIDRKRARVKNSEG          250
AAH55447.1[M.musculus]               GWQHSLLGPILSCVAVCEVIDHPDVKCQIKKKD-----------SKEIDRSRARIKHSEG          247
NP_001303872.1[H.sapiens]            GWQHSLLGPILSCVAVCEVIDHPDVKCQTKKKD-----------SKEIDRRRARIKHSEG          247
XP_009248197.1[P.abelii]             GWQHSLLGPILSCVAVCEVIDHPDVKCQTKKKD-----------SKEIDRRRARIKHSEG          247
                                      *  * *    * :*:**:: .*                                . .

XP_019855808.1[A.queenslandica]      QKVPERYFVVTNDEMLRVKYILVFAQERGR--PSPTRRWFSNHKWVLMVFLYLFLLLFLG          306
XP_002164087.1[H.vulgaris]           GEVPKKYFVVTSSDLVRVKYIFVFTDKTEKRNSAKCGMLCFRYPVLSIMLAYMLLLAFVS          303
XP_032233915.1[N.vectensis]          GDVPDRYYVVQNNQLLRVKYLLVYAEKRPQRNTQRTP---PGTWFARLLIIYIIGLVLLG          321
XP_019627055.1[B.belcheri]           HEVPEKYYIVSNNELLRVKYVLVYAERKAR-PRVQAPSFFQRHKFFILMTLYVIVLAVIG          301
XP_032804273.1[P.marinus]            GDVPQKYYVVTNNQLLRVRYLLLYARKSQHHSIPWQRSWLGRHRFAVGMAVYGLILLLLG          346
XP_045544515.1[S.salar]              GEVPQKYFVVTNNQLLRVKYLLVYSQRRHLSRHSRGRSWLVRHHFAIMMSLYLLLLIVIG          313
XP_018108776.1[X.laevis]             GDVPQKYFVVTNNQLLRVKYLMVYSQEQPR-RRPRQPTWFSKHRFAIMMTLYLFILILIG          299
XP_015147741.1[G.gallus]             GDVPQKYFVVTNNQLLQVKYLLVYSQKQHR-RPSGQSSWFSTHRFAITMMLYLLLLMVIG          309
AAH55447.1[M.musculus]               GEIPPKYFVVTNNQLLRVKYLLVYSQKQPK-RASSQLSWLSSHWFVIMMSLYLLLLLIVS          306
NP_001303872.1[H.sapiens]            GDIPPKYFVVTNNQLLRVKYLLVYSQKPPK-RASSQLSWFSSHWFTVMISLYLLLLLIVS          306
XP_009248197.1[P.abelii]             GDIPPKYFVVTNNQLLRVKYLLVYSQKPPK-RASSQLSWFSSHWFTIMISLYLLLLLIVS          306
                                      .:* :*::* ..::::*:*::::: .                     :  * : * .:.

XP_019855808.1[A.queenslandica]      LWNSRYVSRLYNQYTRRYITL  327
XP_002164087.1[H.vulgaris]           IWNSKTFQLIWRNYWYKKKF-  323
XP_032233915.1[N.vectensis]          LSRTRAFHKLASYLQKEDTHL  342
XP_019627055.1[B.belcheri]           AANSPNLQYYLRKFFR-----  317
XP_032804273.1[P.marinus]            ALNSPTVLHYWRRYLSGD---  364
XP_045544515.1[S.salar]              AFNSTTFLSFWNRLFR-----  329
XP_018108776.1[X.laevis]             LSSSKAFTYHWNRLLGWRP--  318
XP_015147741.1[G.gallus]             ASNSPAFLYYWHRMFYSEG--  328
AAH55447.1[M.musculus]               VTNSSAFHHFWNRVKR-----  322
NP_001303872.1[H.sapiens]            VINSSAFQHFWNRAKR-----  322
XP_009248197.1[P.abelii]             VINSSAFQHFWNRAKK-----  322
                                        :  .
```

## Percent identity matrix:

```
#
#
#  Percent Identity  Matrix - created by Clustal2.1
#
#
```

| | | | | | | | | | | | |
|---|---|---|---|---|---|---|---|---|---|---|---|
| 1: XP_019855808.1[A.queenslandica] | 100.00 | 31.95 | 35.48 | 36.51 | 34.88 | 37.74 | 39.34 | 37.06 | 38.19 | 38.51 | 38.19 |
| 2: XP_002164087.1[H.vulgaris] | 31.95 | 100.00 | 37.22 | 34.41 | 34.27 | 36.79 | 35.46 | 36.14 | 38.29 | 38.29 | 38.29 |
| 3: XP_032233915.1[N.vectensis] | 35.48 | 37.22 | 100.00 | 41.80 | 44.79 | 42.90 | 46.01 | 42.86 | 42.27 | 42.90 | 43.22 |
| 4: XP_019627055.1[B.belcheri] | 36.51 | 34.41 | 41.80 | 100.00 | 43.35 | 45.89 | 47.73 | 46.52 | 46.35 | 44.44 | 44.13 |
| 5: XP_032804273.1[P.marinus] | 34.88 | 34.27 | 44.79 | 43.35 | 100.00 | 54.71 | 55.21 | 59.94 | 55.90 | 57.14 | 56.83 |
| 6: XP_045544515.1[S.salar] | 37.74 | 36.79 | 42.90 | 45.89 | 54.71 | 100.00 | 62.22 | 62.96 | 60.25 | 59.63 | 59.63 |
| 7: XP_018108776.1[X.laevis] | 39.34 | 35.46 | 46.01 | 47.73 | 55.21 | 62.22 | 100.00 | 66.35 | 62.86 | 62.86 | 62.54 |
| 8: XP_015147741.1[G.gallus] | 37.06 | 36.14 | 42.86 | 46.52 | 59.94 | 62.96 | 66.35 | 100.00 | 68.63 | 69.25 | 69.57 |
| 9: AAH55447.1[M.musculus] | 38.19 | 38.29 | 42.27 | 46.35 | 55.90 | 60.25 | 62.86 | 68.63 | 100.00 | 90.68 | 90.06 |
| 10: NP_001303872.1[H.sapiens] | 38.51 | 38.29 | 42.90 | 44.44 | 57.14 | 59.63 | 62.86 | 69.25 | 90.68 | 100.00 | 98.76 |
| 11: XP_009248197.1[P.abelii] | 38.19 | 38.29 | 43.22 | 44.13 | 56.83 | 59.63 | 62.54 | 69.57 | 90.06 | 98.76 | 100.00 |
