# Supplementary material for: An Evolutionary Perspective on the Origin, Conservation and Binding Partner Acquisition of Tankyrases
Source: Biomolecules. 2022 Nov 15;12(11):1688. doi: 10.3390/biom12111688 (PMC9688111; doi:10.3390/biom12111688)
Supplement: Supplementary file 1 [file biomolecules-12-01688-s001.zip › biomolecules-1913719-supplementary-11.17 -done/Data_2/PARP3.pdf]

PARP3

Sequences:

>NP_001003931.4 protein mono-ADP-ribosyltransferase PARP3 isoform a [Homo sapiens]
MAPKPKPWVQTEGPEKKKGRQAGREEDPFRSTAEALKAIPAEKRIIRVDPTCPLSSNPGTQVYEDYNCTL
NQTNIENNNNKFYIIQLLQDSNRFFTCWNRWGRVGEVGQSKINHFTRLEDAKKDFEKKFREKTKNNWAER
DHFVSHPGKYTLIEVQAEDEAQEAVVKVDRGPVRTVTKRVQPCSLDPATQKLITNIFSKEMFKNTMALMD
LDVKKMPLGKLSKQQIARGFEALEALEEALKGPTDGGQSLEELSSHFYTVIPHNFGHSQPPPINSPELLQ
AKKDMLLVLADIELAQALQAVSEQEKTVEEVPHPLDRDYQLLKCQLQLLDSGAPEYKVIQTYLEQTGSNH
RCPTLQHIWKVNQEGEEDRFQAHSKLGNRKLLWHGTNMAVVAAILTSGLRIMPHSGGRVGKGIYFASENS
KSAGYVIGMKCGAHHVGYMFLGEVALGREHHINTDNPSLKSPPPGFDSVIARGHTEPDPTQDTELELDGQ
QVVVPQGQPVPCPEFSSSTFSQSEYLIYQESQCRLRYLLEVHL

>XP_002813722.3 poly [ADP-ribose] polymerase 3 isoform X1 [Pongo abelii]
MSLLFLAMAPKRKPSVQTEGPEKKGRQAGREEDPFRSAAEALKAIPAEKRIIRVDPTCPLSSNPGTQVYE
DYDCTLNQTNIGNNNNKFYIIQLLQDSDRFTCWNRWGRVGEVGQSKINHFTRLEDAKKDFEKKFREKTKN
NWAERDRFVSHPGKYTLIEVQAEDEAQEAVVKVDGGPVRTVAKRVQPCSLDPATQKLITNIFSKEMFKNT
MALMDLDVKKMPLGKLSKQQIARGFEALEALEEALKGPTDGGQSLEELSSHFYTVIPHNFGRSQPPPINS
PELLQAKKDMLLVLADIELAQALQAASEQEKMVEEVPHPLDRDYQLLKCQLQLLDSGAPEYKVIQTYLEQ
TGSNHRCPTLQHIWKVNREGEEDRFQAHSKLGNRKLLWHGTNMAVVAAILTSGLRIMPHSGGRVGKGIYF
ASENSKSAGYVTGMKCGAHHVGYMFLGEVALGREHHINTDNPSLKSPPPGFDSVIARGHTEPDPTQDTEL
ELDGQQVVVPQGQPVPCPEFSRSTFSQSEYLIYQESQCRLRYLLEVHL

>EDL21147.1 poly (ADP-ribose) polymerase family, member 3, isoform CRA_a [Mus musculus]
MAPKRKASVQTEGSKKQRQGTEEEDSFRSTAEALRAAPADNRVIRVDPSCPFSRNPGIQVHEDYDCTLNQ
TNIGNNNNKFYIIQLLEEGSRFFCWNRWGRVGEVGQSKMNHFTCLEDAKKDFKKKFWEKTKNKWEERDRF
VAQPNKYTLIEVQGEAESQEAVVKVYSGPVRTVVKPCSLDPATQNLITNIFSKEMFKNAMTLMNLDVKKM
PLGKLTKQQIARGFEALEALEEAMKNPTGDGQSLEELSSCFYTVIPHNFGRSRPPPINSPDVLQAKKDML
LVLADIELAQTLQAAPGEEEEKVEEVPHPLDRDYQLLRCQLQLLDSGESEYKAIQTYLKQTGNSYRCPNL
RHVWKVNREGEGDRFQAHSKLGNRRLLWHGTNVAVVAAILTSGLRIMPHSGGRVGKGIYFASENSKSAGY
VTTMHCGGHQVGYMFLGEVALGKEHHITIDDPSLKSPPPGFDSVIARGQTEPDPAQDIELELDGQPVVVP
QGPPVQCPSFKSSSFSQSEYLIYKESQCRLRYLLEIHL

>XP_040537823.1 protein mono-ADP-ribosyltransferase PARP3 [Gallus gallus]
MAPKRRAPPASQPADGGKKAKGGQEEEEDAWSSALTALKTAPREKPPATIDGQCPLSAGPDAKVYEDYDC
TLNQTNISANNNKFYIIQLIEHGGTYSTWNRWGRVGEVGQSKLLPFTSLEAAKKDFEKKFWEKTKNRWAA
RDNFVAQPGKYTLIEVQPGAGQEVALRVDGAGDEKVSKRRVLPCALDETTQKLVALIFSSDMFRHAMQAM
NIDVKKMPLGKLSKQQIARGFEALEELEAALGEQPRSMSRLEELSSRFYTIVPHNFGRARPPPIDSPELL
RAKKDMLLVLADIEVAQSLQAQKVEEEEVVAHPLDRDYALLCCQLTLLEDTSQEYEMILNYVAQTGGQVY
VLNVWRVAREGEDKLFQAHDHLEHRRLLWHGTNVAVVAAILKNGLRIMPHSGGRVGKGIYFASENSKSAC
YVGCTSKRVGLMFLTEVALGKPYCITRDEPTLQQPPNGYDSVQACGRTEPDPAQDVEVTLDGKKVLVCQG
KPIPMPAYKDSSFFQSEYLIYQESQCRIRYLVQLHF

>XP_018112381.1 poly(ADP-ribose) polymerase family member 3 L homeolog isoform X1 [Xenopus laevis]
MAPKRKAATQAKASGKIKKTKVKEEVKEEEIEQPVKATDRFQSAVQALKAASGKKGKAKVDSCCHLSGCG
DYEVYEDYDCMLNQTNIGSNNNKFYVIQLITSKKESYCWNRWGRVGELGQSKLSPFPNLAGAQKDFEKKF
KDKTKNSWSERENFTAHPGKYTMIEVQHNDDDDDEGSGEATVKADTVDGTVQKVKPCSLDKPTQDLMSLI
FSSDMFKEAMQTMNLDIKKMPLGKLSKAQIAKGFEALDELQAALDRKANKGVLSDLSSRFYTIVPHNFGR
RTPPVIDTVEVLQAKKDMLLVLADIELAQTLQADKVKKEEEEEKVQEVPHPLDVDYGLLKCDLSLLDTKS
EEYKVINTYVKNTGPSYSPLKILNVWSVNREKEEERLNAHKDIDNRRLLWHGTNIAVVVAILKSGLRIMP
HSGGRVGKGIYFASENSKSAGYVGCTSKNLGIMFLNEVALGKEHHITMDDCSLKSPPKGYDSVVARGCTE
PDPAKDRVLALDGRKITVPQGPPIKMEKYNCTSFSQSEYLVYKESQARLRYLLLLQF

>XP_045560960.1 poly [ADP-ribose] polymerase 3 isoform X1 [Salmo salar]
MAPKRRAASSTKANKTGGKKVKQEPDTPKDAFTSAKEALKAAGPEVKGKRKADEHCLLSEQHSGRVHEDY
DCMLNQTNIGNNNNKFYVIQVLCIDDSYYCWTRWGRVGETGQSNLSDLSNSPDKAIKDFEKKFKDKTKNS
WKERDNFESHPGKYTLIEVDGDQDAEVKVDCVDGKIVKGPRNILPCTLDSPTQKLIQLIFSNDMFKEAME
CMNLDIKKMPLGKLSKMQIAKGFEVLEEIEGAMNASRPKLEELSSKFFTTIPHNFGRTRPPVIDSSEIVE
KKKEMLLVLADIEIAQNLKAETEKAQEQMEVEKVPHALDQNYLSLSCKLSLLQRDTQEFKVIERYLKATA
CSHNQPKIIDVWEVDRETEAERFRENDGLENRRLLWHGTNVAVVAAILKGGLRIMPQSGGRVGRGIYFAS
ENSKSAAYVRTSKDRGVMFLNEVALGKENTITMDDSSLKEAPTGYNCVVARGQLEPDPSKDIFLTLDGKQ
VAVPQGEPIKQPQYKDSFFSNSEYLVYKENQCRIRYLLELKF

>XP_032819077.1 protein mono-ADP-ribosyltransferase PARP3 [Petromyzon marinus]
MAPKRKAASAKGKRQKKVKAEVEPEVPPAVDKVTETRKALKTEPGSKAKAKIDSACNLANKTGLEVHEDY
DCMLNQTNIGHNNNKFYIIQLIAGDGKFYTWTRWGRVGEGGQNAMKTFGNVAEAIKDFEKKFKDKTKNNW
QQRDEFTAHPGKYTLIEMQHEDDEEEGEKPVVSVDAVDGSLTKKVIKPCSLPKQTQDLMSLIFSNDMFKD
AMKEMNLDVKKMPLGKLSKAQIGRGFDALEAIEEALRTAAGQRNLESLSSTFFTIIPHNFGRMRPPVINS
QEIIRNKKDMLLVLADIVLAQSLQADKDRAAAAAGGEGDVGTVVEVPHPLNADYDLLKCELELLGENSHE
LAVIKQYVAATAPSYMKVNCVWKVNREGECERFKAHEELSNRRLLWHGTNIAVVAAILKSGLRIMPHSGG
RVGRGIYFASQNEKSAQYMGYACDGRGVMFLNEVVLGNEFTITKDDCSLKQAPAGYDSVVARGHVEPDPK
KDVILELDGKQVTVPQGRPVQVAKFKQSNFSHSEYLVYNENQCRIRYLLLVSNK

>XP_019634302.1 PREDICTED: poly [ADP-ribose] polymerase 3-like [Branchiostoma belcheri]
MPRAAKRKAAATKGGDSASPDLSKMTVAQLKQECTKQGLGTAGTKAVLLKRLQENTGAGGKKVKTEDTAP
TTAKAAAEKLKKADSPTKKKVYKVDSPCPLNGRGQVQDDYDCMLNQTNIGHNNNKYYIIQLIRTAGVSPY
YVWNRWGRVGEPGQSALKGPWDLETAKKDFEKKFKDKTKNDWSKRGSFVPFKGKYTLLEMDTEETDEDEQ
DTAEKIALLDQLDGPSKNVLPSSLNSQTQSLIKLIFDNDMFKEQMAKMEIDVRKMPLGKLSKAQIAKGFE
VLDEIEEVLKTGRTSGLTELSSRFYTVIPHDFGRRVPPVINDQEKLRKKMDMLLVLGDIEIAQAMQKDKD
KDDGSEATDVKHPLDLNYDLLNCGLELLKPSSEEFKVIEKYTEATKYSGWRDPKILNVWKVDRSGEGDRF
KEHDHLENRKLLWHGTNVAVVAAILKTGLRIMPHSGGRVGRGIYFASENSKSAGYVGCASGNVGIMFLNE
VALGKEKRITRDDSSLTKAPSGFDSVVAVGHREPDPKKNTTMKLDGRTVVVPQGQPINQTEGQSSSFSQS
EYLVYKENQCRIRYLLKMKFGS

>AAM50807.1 LD31274p [Drosophila melanogaster]
MKFAEELGIHIVPIEFLDFVEADTEGAIKYINSTCICSWGTDPKSRIPKETTKSLNSNSIYTKSMPVSRT
FKVKDGLAVDPDSGLEDIAHVYVDSNNKYSVVLGLTDIQRNKNSYYKVQLLKADKKEKYWIFRSWGRIGT
NIGNSKLEEFDTSESAKRNFKEIYADKTGNEYEQRDNFVKRTGRMYPIEIQYDDDQKLVKHESHFFTSKL
EISVQNLIKLIFDIDSMNKTLMEFHIDMDKMPLGKLSAHQIQSAYRVVKEIYNVLECGSNTAKLIDATNR
FYTLIPHNFGVQLPTLIETHQQIEDLRQMLDSLAEIEVAYSIIKSEDVSDACNPLDNHYAQIKTQLVALD
KNSEEFSILSQYVKNTHASTHKSYDLKIVDVFKVSRQGEARRFKPFKKLHNRKLLWHGSRLTNFVGILSH
GLRIAPPEAPPTGYMFGKGIYFADMVSKSANYCCTSQQNSTGLMLLSEVALGDMMECTSAKYINKLSNNK
HSCFGRGRTMPDPTKSYIRSDGVEIPYGETITDEHLKSSLLYNEYIVYDVAQVNIQYLFRMEFKYSY

>XP_047143363.1 protein mono-ADP-ribosyltransferase PARP3 [Hydra vulgaris]
MPPKRKVAQQKASSIEETLPIKFSDLKVPELKAECLKRNLDEKGKKADLIERLEEYENQSSNTSSTKKVK
KEEEKEDEKPVTIKDKLKAIAEKEKADKKKMFKVDSLCTLSNASVVDDYDCMLNQTNVGHNNNKFYVIQL
ICSGAGYYVWNRWGRVGEAGANAIKGPFNDQQYAIKEFTKKFKEKTKNNWEDKDNFQPVAGKYTLLEMDG
EDEPGYTEKNLDAVDGSPLTVKVKTRPCKLDKPTESLIKLIFDNDMFKEAMANMNIDVKKMPLGKLSKSQ
IAKGFEVLEEIQVEINKNKSSKLQDLSSRFFTLIPHNFGRQRPPTISDQETLQMKMDMLTVLGDIEIAQS
LQNVVVKKEEVEDDHPLDTNYGLLKCDLKHIDCKSDTFKIINNYIEQTKSSYGNIKLLDVWEVDRMGEDE
RFSAHNDIKNRKLLWHGTNVAVVVAILKSGLRIMPHSGGRVGRGIYFASEHAKSAGYVGTARDGTGIMFL
NEVAIGKQHIITQDDSSLKAPPKGYDSVLAKGRQEPDPSMDTSLIINGNTVTVPQGKPVETDAHSSSFHQ
SEYLIYKESQNRIRYLLKMKFH

>XP_019856101.1 PREDICTED: poly [ADP-ribose] polymerase 3-like [Amphimedon queenslandica]
MPPKKRQRKTAAAKEEDEPTTSAVAPLNPAALTVTQLRAELSKKGLDTTGKKALLVQRLTDSLKSSDEPS

TTKKAKTEDPAEDEGEEEIDTTEGELTYSKAAAALRQADKKEKGKRQRTVDTHIPWYTNYEVVDDWDCML
NQTNIGHNNNKFYVIQLLSNKNSGYFHVWNRWGRVGEPGMNAMKGPFHAMDAATKEFAKKFKDKTKNDWS
DRDSFTPHAGKYTLIEMGGDDDEDEPMDVIEDVDTGKPKKIKPCTLDKPTQNLVKLIFDNDMFREAMKTL
EIDTKKMPLGKLSKAQIAKGFEALEALESALQSKASRAKLSELSSKFYTIIPHDFGRRIPPVIDNQEHLQ
KKFDMLTVLGDIEIAQSLQKDKIKTEQPDIEEVPHEFDVNYASLKAKLEHVKKEDSDYKIIETYLRQTEP
SYRKLEIVDIWRVSRDGEAERFSVHDSIENRRLLWHGTNVAVVAAILKSGLRIMPHSGGRVGRGIYFASE
NSKSAGYVGCANNIGIMFLNEVALGKEHSITRDDSSLVKAPAGHDSVVARGHTEPDPALDTTITIDGKTV
TVPQGKPKSQQKYSTSSFSQTEYLIYKESQNRIRYLLKLKFGGHSFFF

Alignment

```
AAM50807.1[D.melanogaster]              ------------MKFAEE-LGIHIVPIEFLDFVEA-----------DTEGAIKYINSTCI          36
XP_047143363.1[H.vulgaris]              MPPKRKVAQQKAS------SIEETLPIKFSDLKVPELKAECLKRNLDEKGKKADL-----          49
XP_019634302.1[B.belcheri]              MP-RAAKRKAAATKGGD------SASPDLSKMTVAQLKQECTKQGLGTAGTKAVL-----          48
XP_019856101.1[A.queenslandica]         MPPKKRQRKTAAAKEEDEPTTSAVAPLNPAALTVTQLRAELSKKGLDTTGKKALL-----          55
XP_045560960.1[S.salar]                 ------------------------------------------------------------          0
XP_040537823.1[G.gallus]                ------------------------------------------------------------          0
EDL21147.1[M.musculus]                  ------------------------------------------------------------          0
NP_001003931.4[H.sapiens]               ------------------------------------------------------------          0
XP_002813722.3[P.abelii]                ---------------------------------------------------MSLL-----          4
XP_018112381.1[X.laevis]                ------------------------------------------------------------          0
XP_032819077.1[P.marinus]               ------------------------------------------------------------          0


AAM50807.1[D.melanogaster]              CSWGTDPKSRIPKETT---------------------KSLNSNSIYTKSMPVSRT--FK          72
XP_047143363.1[H.vulgaris]              -IERLEEYENQSSNTSST---KKVKKEE-----EKEDEKPVTIKDKLKAIAE---KEKAD          97
XP_019634302.1[B.belcheri]              -LKRLQENTGA--------GGKKVKTED------------TAPTTAKAAAEKLKKADSPT          87
XP_019856101.1[A.queenslandica]         -VQRLTDSLKSSDE--P-STTKKAKTEDPAEDEGEEEIDTTEGELTYSKAAAALRQADKK          111
XP_045560960.1[S.salar]                 ----MAPKRRAASSTKANKTGGK-KVK----------QEPDTPKDAFTSAKEALKAAGPE          45
XP_040537823.1[G.gallus]                ----MAPKRRAPPASQPADGGKKAKGG------------QEEEEDAWSSALTALKTA-PR          43
EDL21147.1[M.musculus]                  ----MAPKRKASVQTE---GSK--KQR-----------QGTEEEDSFRSTAEALRAA-PA          39
NP_001003931.4[H.sapiens]               ----MAPKPKPWVQTE---GPEKKKGR-----------QAGREEDPFRSTAEALKAI-PA          41
XP_002813722.3[P.abelii]                -FLAMAPKRKPSVQTE---GPEK-KGR-----------QAGREEDPFRSAAEALKAI-PA          47
XP_018112381.1[X.laevis]                ----MAPKRKAATQAKASGKIKKTKVKE--EVKEEEIEQPVKATDRFQSAVQALKAA-SG          53
XP_032819077.1[P.marinus]               ----MAPKRKAASAKGKRQK----KVKA--EVE----PEVPPAVDKVTETRKALKTE-PG          45


AAM50807.1[D.melanogaster]              VKDGLAVDPDSGLEDIAHVYVDSNNKYSVVLGLTDIQRNKNSYYKVQLLKADKKEKYWIF          132
XP_047143363.1[H.vulgaris]              KKKMFKVDSLCTLSN-----ASVVDDYDCMLNQTNVGHNNNKFYVIQLICSGA-G-YYVW          150
XP_019634302.1[B.belcheri]              KKKVYKVDSPCPLNG----RGQVQDDYDCMLNQTNIGHNNNKYYIIQLIRTAGVSPYYVW          143
XP_019856101.1[A.queenslandica]         EKGKRQRTVDTHIPWY--TNYEVVDDWDCMLNQTNIGHNNNKFYVIQLLSNKNSGYFHVW          169
XP_045560960.1[S.salar]                 VKGKRKADEHCLLSEQ--HSGRVHEDYDCMLNQTNIGNNNNKFYVIQVLCIDD-S-YYCW          101
XP_040537823.1[G.gallus]                EKPPATIDGQCPLSAG--PDAKVYEDYDCTLNQTNISANNNKFYIIQLIEHGG-T-YSTW          99
EDL21147.1[M.musculus]                  DNRVIRVDPSCPFSRN--PGIQVHEDYDCTLNQTNIGNNNNKFYIIQLLEEGS-RF-FCW          95
NP_001003931.4[H.sapiens]               EKRIIRVDPTCPLSSN--PGTQVYEDYNCTLNQTNIENNNNKFYIIQLLQDSN-RFFTCW          98
XP_002813722.3[P.abelii]                EKRIIRVDPTCPLSSN--PGTQVYEDYDCTLNQTNIGNNNNKFYIIQLLQDSD-R-FTCW          103
XP_018112381.1[X.laevis]                KKGKAKVDSCCHLSGC--GDYEVYEDYDCMLNQTNIGSNNNKFYVIQLITSKK-E-SYCW          109
XP_032819077.1[P.marinus]               SKAKAKIDSACNLANK--TGLEVHEDYDCMLNQTNIGHNNNKFYIIQLIAGDG-K-FYTW          101
                                         :          :           :.:.  *. *::  *:*.:* :*::          :

AAM50807.1[D.melanogaster]              RSWGRIGTNIGNSKLEEF-DTSESAKRNFKEIYADKTGNEYEQRDNFVKRTGRMYPIEIQ          191
XP_047143363.1[H.vulgaris]              NRWGRVGEA-GANAIKGPFNDQQYAIKEFTKKFKEKTKNNWEDKDNFQPVAGKYTLLEMD          209
XP_019634302.1[B.belcheri]              NRWGRVGEP-GQSALKGPWDL-ETAKKDFEKKFKDKTKNDWSKRGSFVPFKGKYTLLEMD          201
XP_019856101.1[A.queenslandica]         NRWGRVGEP-GMNAMKGPFHAMDAATKEFAKKFKDKTKNDWSDRDSFTPHAGKYTLIEMG          228
XP_045560960.1[S.salar]                 TRWGRVGET-GQSNLSDLSNSPDKAIKDFEKKFKDKTKNSWKERDNFESHPGKYTLIEVD          160
XP_040537823.1[G.gallus]                NRWGRVGEV-GQSKLLPFTSL-EAAKKDFEKKFWEKTKNRWAARDNFVAQPGKYTLIEVQ          157
EDL21147.1[M.musculus]                  NRWGRVGEV-GQSKMNHFTCL-EDAKKDFKKKFWEKTKNKWEERDRFVAQPNKYTLIEVQ          153
NP_001003931.4[H.sapiens]               NRWGRVGEV-GQSKINHFTRL-EDAKKDFEKKFREKTKNNWAERDHFVSHPGKYTLIEVQ          156
XP_002813722.3[P.abelii]                NRWGRVGEV-GQSKINHFTRL-EDAKKDFEKKFREKTKNNWAERDRFVSHPGKYTLIEVQ          161
XP_018112381.1[X.laevis]                NRWGRVGEL-GQSKLSPFPNL-AGAQKDFEKKFKDKTKNSWSERENFTAHPGKYTMIEVQ          167
XP_032819077.1[P.marinus]               TRWGRVGEG-GQNAMKTFGNV-AEAIKDFEKKFKDKTKNNWQQRDEFTAHPGKYTLIEMQ          159
                                          ***:*   * . :         * ::* : : :** * :  :  *     .:  :*:

AAM50807.1[D.melanogaster]              YDDDQKLV------------------KHESHFFTSKLEISVQNLIKLIFDIDSMNKTLME          233
XP_047143363.1[H.vulgaris]              GEDEPGYT------EKNLDAVDGSPLTVKVKTRPCKLDKPTESLIKLIFDNDMFKEAMAN          263
XP_019634302.1[B.belcheri]              TEETDEDEQDTAEKIALLD----QLDGPSKNVLPSSLNSQTQSLIKLIFDNDMFKEQMAK          257
XP_019856101.1[A.queenslandica]         GDDDEDEP------MDVI---EDVDTGKPKKIKPCTLDKPTQNLVKLIFDNDMFREAMKT          279
XP_045560960.1[S.salar]                 GDQDAE---------VKVDCVDGKIVKGPRNILPCTLDSPTQKLIQLIFSNDMFKEAMEC          211
XP_040537823.1[G.gallus]                PGAGQE------VALRVDGAGDEKVS--KRRVLPCALDETTQKLVALIFSSDMFRHAMQA          209
EDL21147.1[M.musculus]                  GEAESQ------EAVVKVYSGPVRTV-----VKPCSLDPATQNLITNIFSKEMFKNAMTL          202
NP_001003931.4[H.sapiens]               AEDEAQ------EAVVKVDRGPVRTV--TKRVQPCSLDPATQKLITNIFSKEMFKNTMAL          208
XP_002813722.3[P.abelii]                AEDEAQ------EAVVKVDGGPVRTV--AKRVQPCSLDPATQKLITNIFSKEMFKNTMAL          213
XP_018112381.1[X.laevis]                HNDDDDDEGS-GEATVKADTVDGT----VQKVKPCSLDKPTQDLMSLIFSSDMFKEAMQT          222
XP_032819077.1[P.marinus]               HEDDEEEGEK---PVVSVDAVDGSLT--KKVIKPCSLPKQTQDLMSLIFSNDMFKDAMKE          214
                                                                          . *   .:.*:  **. : :.. :

AAM50807.1[D.melanogaster]              FHIDMDKMPLGKLSAHQIQSAYRVVKEIYNVLECGSN-TAKLIDATNRFYTLIPHNFGVQ          292
XP_047143363.1[H.vulgaris]              MNIDVKKMPLGKLSKSQIAKGFEVLEEIQVEINKNK--SSKLQDLSSRFFTLIPHNFGRQ          321
XP_019634302.1[B.belcheri]              MEIDVRKMPLGKLSKAQIAKGFEVLDEIEEVLKTGR--TSGLTELSSRFYTVIPHDFGRR          315
XP_019856101.1[A.queenslandica]         LEIDTKKMPLGKLSKAQIAKGFEALEALESALQSKAS-RAKLSELSSKFYTIIPHDFGRR          338
XP_045560960.1[S.salar]                 MNLDIKKMPLGKLSKMQIAKGFEVLEEIEGAMNAS---RPKLEELSSKFFTTIPHNFGRT          268
XP_040537823.1[G.gallus]                MNIDVKKMPLGKLSKQQIARGFEALEELEAALGEQPRSMSRLEELSSRFYTIVPHNFGRA          269
EDL21147.1[M.musculus]                  MNLDVKKMPLGKLTKQQIARGFEALEALEEAMKNPTGDGQSLEELSSCFYTVIPHNFGRS          262
NP_001003931.4[H.sapiens]               MDLDVKKMPLGKLSKQQIARGFEALEALEEALKGPTDGGQSLEELSSHFYTVIPHNFGHS          268
XP_002813722.3[P.abelii]                MDLDVKKMPLGKLSKQQIARGFEALEALEEALKGPTDGGQSLEELSSHFYTVIPHNFGRS          273
XP_018112381.1[X.laevis]                MNLDIKKMPLGKLSKAQIAKGFEALDELQAALDRKAN-KGVLSDLSSRFYTIVPHNFGRR          281
XP_032819077.1[P.marinus]               MNLDVKKMPLGKLSKAQIGRGFDALEAIEEALRTAAG-QRNLESLSSTFFTIIPHNFGRM          273
                                        :.:*  *******:  **  .: .:. :   :         * . :. *:* :**:**

AAM50807.1[D.melanogaster]              LPTLIETHQQIEDLRQMLDSLAEIEVAYSIIKSE--------------DVSDACNPLDNH          338
XP_047143363.1[H.vulgaris]              RPPTISDQETLQMKMDMLTVLGDIEIAQSLQNVVVKK-----------EEVEDDHPLDTN          370
XP_019634302.1[B.belcheri]              VPPVINDQEKLRKKMDMLLVLGDIEIAQAMQKDKDK--------DDGSEATDVKHPLDLN          367
XP_019856101.1[A.queenslandica]         IPPVIDNQEHLQKKFDMLTVLGDIEIAQSLQKDKIK--------TEQPDIEEVPHEFDVN          390
XP_045560960.1[S.salar]                 RPPVIDSSEIVEKKKEMLLVLADIEIAQNLKAETEKA-------QEQMEVEKVPHALDQN          321
XP_040537823.1[G.gallus]                RPPPIDSPELLRAKKDMLLVLADIEVAQSLQAQKV---------EEE---EVVAHPLDRD          317
EDL21147.1[M.musculus]                  RPPPINSPDVLQAKKDMLLVLADIELAQTLQAAPG---------EEEEKVEEVPHPLDRD          313
NP_001003931.4[H.sapiens]               QPPPINSPELLQAKKDMLLVLADIELAQALQAVS----------EQEKTVEEVPHPLDRD          318
XP_002813722.3[P.abelii]                QPPPINSPELLQAKKDMLLVLADIELAQALQAAS----------EQEKMVEEVPHPLDRD          323
XP_018112381.1[X.laevis]                TPPVIDTVEVLQAKKDMLLVLADIELAQTLQADKVKK------EEEEEKVQEVPHPLDVD          335
XP_032819077.1[P.marinus]               RPPVINSQEIIRNKKDMLLVLADIVLAQSLQADKDRAAAAAGGEGDVGTVVEVPHPLNAD          333
                                         *  *.  : :.   :**  *.:* :*  :                        : :: .

AAM50807.1[D.melanogaster]              YAQIKTQLVALDKNSEEFSILSQYVKNTHASTHKSYDLKIVDVFKVSRQGEARRFKPFKK          398
XP_047143363.1[H.vulgaris]              YGLLKCDLKHIDCKSDTFKIINNYIEQTK-SS--YGNIKLLDVWEVDRMGEDERFSAHND          427
XP_019634302.1[B.belcheri]              YDLLNCGLELLKPSSEEFKVIEKYTEATKYSG--WRDPKILNVWKVDRSGEGDRFKEHDH          425
XP_019856101.1[A.queenslandica]         YASLKAKLEHVKKEDSDYKIIETYLRQTE-PS--YRKLEIVDIWRVSRDGEAERFSVHDS          447
XP_045560960.1[S.salar]                 YLSLSCKLSLLQRDTQEFKVIERYLKATA-CS--HNQPKIIDVWEVDRETEAERFRENDG          378
XP_040537823.1[G.gallus]                YALLCCQLTLLEDTSQEYEMILNYVAQTG-GQ--VY---VLNVWRVAREGEDKLFQAHDH          371
EDL21147.1[M.musculus]                  YQLLRCQLQLLDSGESEYKAIQTYLKQTG-NS--YRCPNLRHVWKVNREGEGDRFQAHSK          370
NP_001003931.4[H.sapiens]               YQLLKCQLQLLDSGAPEYKVIQTYLEQTG-SN--HRCPTLQHIWKVNQEGEEDRFQAHSK          375
XP_002813722.3[P.abelii]                YQLLKCQLQLLDSGAPEYKVIQTYLEQTG-SN--HRCPTLQHIWKVNREGEEDRFQAHSK          380
XP_018112381.1[X.laevis]                YGLLKCDLSLLDTKSEEYKVINTYVKNTG-PS--YSPLKILNVWSVNREKEEERLNAHKD          392
XP_032819077.1[P.marinus]               YDLLKCELELLGENSHELAVIKQYVAATA-PS--YMK--VNCVWKVNREGECERFKAHEE          388
                                        *  :   *  :         :  *   *           :  :: * :  *   :   .

AAM50807.1[D.melanogaster]              LHNRKLLWHGSRLTNFVGILSHGLRIAPPEAPPTGYMFGKGIYFADMVSKSANYCCT--S          456
XP_047143363.1[H.vulgaris]              IKNRKLLWHGTNVAVVVAILKSGLRIMPH----SGGRVGRGIYFASEHAKSAGYV---GT          480
XP_019634302.1[B.belcheri]              LENRKLLWHGTNVAVVAAILKTGLRIMPH----SGGRVGRGIYFASENSKSAGYV---GC          478
XP_019856101.1[A.queenslandica]         IENRRLLWHGTNVAVVAAILKSGLRIMPH----SGGRVGRGIYFASENSKSAGYV---GC          500
XP_045560960.1[S.salar]                 LENRRLLWHGTNVAVVAAILKGGLRIMPQ----SGGRVGRGIYFASENSKSAAYV----R          430
XP_040537823.1[G.gallus]                LEHRRLLWHGTNVAVVAAILKNGLRIMPH----SGGRVGKGIYFASENSKSACYV---GC          424
EDL21147.1[M.musculus]                  LGNRRLLWHGTNVAVVAAILTSGLRIMPH----SGGRVGKGIYFASENSKSAGYVTTMHC          426
```

```
NP_001003931.4[H.sapiens]            LGNRKLLWHGTNMAVVAAILTSGLRIMPH----SGGRVGKGIYFASENSKSAGYVIGMKC           431
XP_002813722.3[P.abelii]             LGNRKLLWHGTNMAVVAAILTSGLRIMPH----SGGRVGKGIYFASENSKSAGYVTGMKC           436
XP_018112381.1[X.laevis]             IDNRRLLWHGTNIAVVVAILKSGLRIMPH----SGGRVGKGIYFASENSKSAGYV---GC           445
XP_032819077.1[P.marinus]            LSNRRLLWHGTNIAVVAAILKSGLRIMPH----SGGRVGRGIYFASQNEKSAQYM---GY           441
                                     : :*:*****:.:: ...**. **** *     :*  .*:*****.   *** *

AAM50807.1[D.melanogaster]           QQNSTGLMLLSEVALGDMMECTSAK-YINKLSNNKHSCFGRGRTMPDPTKSY---IRSDG           512
XP_047143363.1[H.vulgaris]           ARDGTGIMFLNEVAIGKQHIITQDDSSLKAPPKGYDSVLAKGRQEPDPSMDTSLIINGNT           540
XP_019634302.1[B.belcheri]           ASGNVGIMFLNEVALGKEKRITRDDSSLTKAPSGFDSVVAVGHREPDPKKNTTMKLDGRT           538
XP_019856101.1[A.queenslandica]      -ANNIGIMFLNEVALGKEHSITRDDSSLVKAPAGHDSVVARGHTEPDPALDTTITIDGKT           559
XP_045560960.1[S.salar]              TSKDRGVMFLNEVALGKENTITMDDSSLKEAPTGYNCVVARGQLEPDPSKDIFLTLDGKQ           490
XP_040537823.1[G.gallus]             TSKRVGLMFLTEVALGKPYCITRDEPTLQQPPNGYDSVQACGRTEPDPAQDVEVTLDGKK           484
EDL21147.1[M.musculus]               GGHQVGYMFLGEVALGKEHHITIDDPSLKSPPPGFDSVIARGQTEPDPAQDIELELDGQP           486
NP_001003931.4[H.sapiens]            GAHHVGYMFLGEVALGREHHINTDNPSLKSPPPGFDSVIARGHTEPDPTQDTELELDGQQ           491
XP_002813722.3[P.abelii]             GAHHVGYMFLGEVALGREHHINTDNPSLKSPPPGFDSVIARGHTEPDPTQDTELELDGQQ           496
XP_018112381.1[X.laevis]             TSKNLGIMFLNEVALGKEHHITMDDCSLKSPPKGYDSVVARGCTEPDPAKDRVLALDGRK           505
XP_032819077.1[P.marinus]            ACDGRGVMFLNEVVLGNEFTITKDDCSLKQAPAGYDSVVARGHVEPDPKKDVILELDGKQ           501
                                          * *:* **.:*     .  .  :    . ..  . *   ***  .    : .

AAM50807.1[D.melanogaster]           VEIPYGETITDEHLK-SSLLYNEYIVYDVAQVNIQYLFRMEFKYSY---           557
XP_047143363.1[H.vulgaris]           VTVPQGKPVETDA-HSSSFHQSEYLIYKESQNRIRYLLKMKFH------           582
XP_019634302.1[B.belcheri]           VVVPQGQPINQTEGQSSSFSQSEYLVYKENQCRIRYLLKMKFGS-----           582
XP_019856101.1[A.queenslandica]      VTVPQGKPKSQQKYSTSSFSQTEYLIYKESQNRIRYLLKLKFGGHSFFF           608
XP_045560960.1[S.salar]              VAVPQGEPIKQPQYKDSFFSNSEYLVYKENQCRIRYLLELKF-------           532
XP_040537823.1[G.gallus]             VLVCQGKPIPMPAYKDSSFFQSEYLIYQESQCRIRYLVQLHF-------           526
EDL21147.1[M.musculus]               VVVPQGPPVQCPSFKSSSFSQSEYLIYKESQCRLRYLLEIHL-------           528
NP_001003931.4[H.sapiens]            VVVPQGQPVPCPEFSSSTFSQSEYLIYQESQCRLRYLLEVHL-------           533
XP_002813722.3[P.abelii]             VVVPQGQPVPCPEFSRSTFSQSEYLIYQESQCRLRYLLEVHL-------           538
XP_018112381.1[X.laevis]             ITVPQGPPIKMEKYNCTSFSQSEYLVYKESQARLRYLLLLQF-------           547
XP_032819077.1[P.marinus]            VTVPQGRPVQVAKFKQSNFSHSEYLVYNENQCRIRYLLLVSNK------           544
                                     : :  *          : :  .**::*.  * .::**. :
```

Percent identity matrix:

```
#
#
#  Percent Identity  Matrix - created by Clustal2.1
#
#
```

1: AAM50807.1[D.melanogaster]	100.00	31.67	33.52	30.97	33.13	33.81	31.64	31.39	31.21	33.67	31.72
2: XP_047143363.1[H.vulgaris]	31.67	100.00	57.27	55.57	54.05	51.47	51.87	50.59	50.77	56.98	53.58
3: XP_019634302.1[B.belcheri]	33.52	57.27	100.00	56.89	57.89	55.21	54.31	54.76	55.19	57.12	56.12
4: XP_019856101.1[A.queenslandica]	30.97	55.57	56.89	100.00	55.34	52.91	52.91	50.67	51.52	57.60	53.99
5: XP_045560960.1[S.salar]	33.13	54.05	57.89	55.34	100.00	57.14	57.45	55.09	56.43	61.98	60.04
6: XP_040537823.1[G.gallus]	33.81	51.47	55.21	52.91	57.14	100.00	63.44	61.30	62.38	60.99	55.77
7: EDL21147.1[M.musculus]	31.64	51.87	54.31	52.91	57.45	63.44	100.00	81.40	82.70	62.19	57.34
8: NP_001003931.4[H.sapiens]	31.39	50.59	54.76	50.67	55.09	61.30	81.40	100.00	97.18	59.13	56.62
9: XP_002813722.3[P.abelii]	31.21	50.77	55.19	51.52	56.43	62.38	82.70	97.18	100.00	60.38	57.20
10: XP_018112381.1[X.laevis]	33.67	56.98	57.12	57.60	61.98	60.99	62.19	59.13	60.38	100.00	61.50
11: XP_032819077.1[P.marinus]	31.72	53.58	56.12	53.99	60.04	55.77	57.34	56.62	57.20	61.50	100.00
