# Supplementary material for: An Evolutionary Perspective on the Origin, Conservation and Binding Partner Acquisition of Tankyrases
Source: Biomolecules. 2022 Nov 15;12(11):1688. doi: 10.3390/biom12111688 (PMC9688111; doi:10.3390/biom12111688)
Supplement: Supplementary file 1 [file biomolecules-12-01688-s001.zip › biomolecules-1913719-supplementary-11.17 -done/Data_2/PARP4.pdf]

PARP4

Sequences:

>NP_006428.2 protein mono-ADP-ribosyltransferase PARP4 [Homo sapiens]
MVMGIFANCIFCLKVKYLPQQQKKKLQTDIKENGGKFSFSLNPQCTHIILDNADVLSQYQLNSIQKNHVH
IANPDFIWKSIREKRLLDVKNYDPYKPLDITPPPDQKASSSEVKTEGLCPDSATEEEDTVELTEFGMQNV
EIPHLPQDFEVAKYNTLEKVGMEGGQEAVVVELQCSRDSRDCPFLISSHFLLDDGMETRRQFAIKKTSED
ASEYFENYIEELKKQGFLLREHFTPEATQLASEQLQALLLEEVMNSSTLSQEVSDLVEMIWAEALGHLEH
MLLKPVNRISLNDVSKAEGILLLVKAALKNGETAEQLQKMMTEFYRLIPHKGTMPKEVNLGLLAKKADLC
QLIRDMVNVCETNLSKPNPPSLAKYRALRCKIEHVEQNTEEFLRVRKEVLQNHHSKSPVDVLQIFRVGRV
NETTEFLSKLGNVRPLLHGSPVQNIVGILCRGLLLPKVVEDRGVQRTDVGNLGSGIYFSDSLSTSIKYSH
PGETDGTRLLLICDVALGKCMDLHEKDFSLTEAPPGYDSVHGVSQTASVTTDFEDDEFVVYKTNQVKMKY
IIKFSMPGDQIKDFHPSDHTELEEYRPEFSNFSKVEDYQLPDAKTSSSTKAGLQDASGNLVPLEDVHIKG
RIIDTVAQVIVFQTYTNKSHVPIEAKYIFPLDDKAAVCGFEAFINGKHIVGEIKEKEEAQQEYLEAVTQG
HGAYLMSQDAPDVFTVSVGNLPPKAKVLIKITYITELSILGTVGVFFMPATVAPWQQDKALNENLQDTVE
KICIKEIGTKQSFSLTMSIEMPYVIEFIFSDTHELKQKRTDCKAVISTMEGSSLDSSGFSLHIGLSAAYL
PRMWVEKHPEKESEACMLVFQPDLDVDLPDLASESEVIICLDCSSSMEGVTFLQAKQIALHALSLVGEKQ
KVNIIQFGTGYKELFSYPKHITSNTMAAEFIMSATPTMGNTDFWKTLRYLSLLYPARGSRNILLVSDGHL
QDESLTLQLVKRSRPHTRLFACGIGSTANRHVLRILSQCGAGVFEYFNAKSKHSWRKQIEDQMTRLCSPS
CHSVSVKWQQLNPDVPEALQAPAQVPSLFLNDRLLVYGFIPHCTQATLCALIQEKEFRTMVSTTELQKTT
GTMIHKLAARALIRDYEDGILHENETSHEMKKQTLKSLIIKLSKENSLITQFTSFVAVEKRDENESPFPD
IPKVSELIAKEDVDFLPYMSWQGEPQEAVRNQSLLASSEWPELRLSKRKHRKIPFSKRKMELSQPEVSED
FEEDGLGVLPAFTSNLERGGVEKLLDLSWTESCKPTATEPLFKKVSPWETSTSSFFPILAPAVGSYLPPT
ARAHSPASLSFASYRQVASFGSAAPPRQFDASQFSQGPVPGTCADWIPQSASCPTGPPQNPPSSPYCGIV
FSGSSLSSAQSAPLQHPGGFTTRPSAGTFPELDSPQLHFSLPTDPDPIRGFGSYHPSASSPFHFQPSAAS
LTANLRLPMASALPEALCSQSRTTPVDLCLLEESVGSLEGSRCPVFAFQSSDTESDELSEVLQDSCFLQI
KCDTKDDSILCFLEVKEEDEIVCIQHWQDAVPWTELLSLQTEDGFWKLTPELGLILNLNTNGLHSFLKQK
GIQSLGVKGRECLLDLIATMLVLQFIRTRLEKEGIVFKSLMKMDDASISRNIPWAFEAIKQASEWVRRTE
GQYPSICPRLELGNDWDSATKQLLGLQPISTVSPLHRVLHYSQG

>NP_001125927.1 protein mono-ADP-ribosyltransferase PARP4 [Pongo abelii]
MMMGIFANCIFCLKVKYLPRQQKKKLQTDIKENGGKFSFSLNPQCTHIILDNADVLSQYQLNSIQKNHVH
IANPDFIWESVREKRLLDVKNYDPYKPLDIIPPPDQKPSSPEVKTEGLCPDSATEQEDTVELTEFGTQNV
EIPHLPQDFEVAKYNTLEKVGMEGGQEAVVVELQCSRDSRDCPFLISSHFLLDDGMQTRRQFAIKKTSED
ASEYYENYIEELKKQGFLLREHFTPEATQLASEQLQALLLEEVMNSSTLSQKVSDLVEMIWAEALGHLEH
MLLKPVNRISLNDVSKAEGILLLVKAALKNGETAEQLQKMMAEFYRLIPHKGTTPKEVNLGLLAKKADLC
QLIRDMVNVCETNLSKPSPPSLAKYQALRCKIEHVEQNTEEFLRVRKEVLQNHHSKSPVDVLQIFRVGRV
NETTEFLSKLGNVRPLLHGSPVRNIVGILCRGLLLPKVVEDHGVQRTDVGNLGSGIYFSDSLSTSIKYSH
PGETDGTRLLLICDVALGKCMDLHKKDFSLTEAPPGYDSVHGVSQTASVTTDFEDDEFVVYKTNQVKMKY
IIKFSMPGDQIKDFHPSDHTELEEYRPEFSNFSKVEDYQLPDAKTSSSTKAGLQDASGNLVPLEDVHIKG
RIIDTVAQVIVFQTYTNKSHVPIEAKYIFPLDDKAAVCGFEAFINGKHIVGEIKEKEEAQREYREAVTKG
HGAYLMSQDAPDVFTVSVGNLPPKAKVLIKIAYITELSILGTVGVFFMPATVAPWQQDKALNENLQDTVE
KICIKEIGTKQSFSLTMSIEMPYVIELIFSDTHELKQKRTDCKAVISTMEGSSLDSSGFSLHIGLSAAYL
PRMWVEKHPEKESEACMLVFQPDLDVDLPDLANESEVIICLDCSSSMEGVTFLQAKQIALHALSLVGEKQ
KVNIIQFGTGYKELFSYPKHITNNTAAAEFIMSATPTMGNTDFWKTLRYLSLLYPARGSRNILLVSDGHL
QDESLTLQLVKRSRPHTRLFACGIGSTANRHILRILSQCGAGVFEYFNAKSKHSWRKQIEDQMTRLCSPS
CHSVSVKWQQLNPDGPEALQAPAQVPSLFRNDRLLVYGFIPHCTQATLCALIQEKEFCTMVSTTELQKTT
GTMIHKLAARALIRDYEDGILHENETSHEMKKQTLKSLIIKLSKENSLITQFTSFVAVEKRDENESPFPD
IPKVSELIAKEDVDFLPYMSWQGEPQGAIRNQSLLASSEWQKLYSSKPKLRAKRKKKLSQPEVSEDFEED
GLGVLPAFTSNLERGGVEKLSDLSWTESCKPTATEPLFKKVSPWETSTSSLFGVSPPVVGSYLRPTAHAP
RAASMPFASYCQAASFGSAAPPRQFDAPQFSQSPAPGSCADWIPQSASCPTGPPQNLSFAPFCGIAFSGS
SLSPTQLPPPQFPGGCTTRPSAGTSPELDSPQLLFSLPTDPDPIRGFGSYHLSASSPFHFQPSAASLTAN
LRLPIASALPEALCSQSQTTPVDLCLLQESVGSLEGSRCPVFVFRSSDTESDELSEVLQDSRILQIKCDT
KDDSIPCFLEVIEEDEIVCTQHWQDAVPWTELFSLQTEDGFWKLTPELGLILNLNTNALHSFLKQKGIQS
LGIKGRERLLDLIATMLVLQFIRTRLEKEGIVFKSLMKMDDPSISRNIPWAFEAIKKASEWVRRTEGQYP
SICPRLELGKDWDSATKQLLGLQPVNTVSPLHRVLHYSQG

>EDL36193.1 mCG130836, isoform CRA_a [Mus musculus]
MTLGIFANCIFCLKVKYLPRQQKKKLQTDIKENGGKFSFLLNPQCTHVIVDSADVLSRCHLNSIQKNDVQ
IANPAFIQDSVRQRRLLDVRNYDPLSPAPAAPPAERSRSEVQSEYLPSDNTPEKENTEVTEVSAENVEIP
PFLQDFEVVKYNILEKVGGPETVVVELQSSQDPESCPFVITAHFLLADQKTRRESTGKQTSEGAIEYYES
YVEDLKRQGFLLQEHFTAEATQLASEKLQALLLEEVISSGALSQEVSDLLEVIWTEALGHLENTLLKPVN
SMSLNDVSKAEGILLLVKTALKNGDSPGQLQKTMAEFYRLLPHRHPASEEVNLRLLAQKEDLCQLVRDMV
NVCETNLSKPNPPSLAKYRALRCKIEHVDQNTEEFSRVRKEVLQNNRSEQPVDILQIFRVGRVNEATEFL
SKLGNVRLLFHGSPVRNILGILSRGLLLPKVAEDRGVQRTDVGNLGSGIYFSDSLSTSIKYAHAGETDGS
RLLVVCDVALGKCVNLFKKDFSLTEAPPGYDSVHGVSETTSVPTDFQDDEFVVYKTNQVKMKYIVKFCTP
GDQIKEFHPHENTEVEEQRAEPSSVPEAGDFQLPDIKPFTNIKAGLQDASANPVPLDSVHIKGRVIDFVA
QVIVFQTYTNQSHVPIEAKYIFPLDDKAAVCGFEAFINGKHIVGEIKEKEEARQEYREAVSQGHGAYLMD
QDTPDVFTVSVGNLPPRAKVLIKITYITELSIQSPVAIFFIPGTVAPWQQDKALNENLQDTVETIRIKEI
GAEQSFSLAMSIEMPYMIEFISSDTHELRQKSTDCKAVVSTVEGSSLDSGGFSLHIGLRDAYLPRMWVEK
HPEKESEACMLVFQPELADVLPDLRGKNEVIICLDCSSSMEGVTFTQAKQVALYALSLLGEEQKVNIMQF
GTGYKELFSYPKCITDSKMATEFIMSAAPSMGNTDFWKVLRYLSLLYPSEGFRNILLISDGHLQSESLTL
QLVKRNIQHTRVFTCAVGSTANRHILRTLSQCGAGVFEYFNSKSKHSWKKQIEAQMTRIRSPSCHSVSVK
WQQLSRDAPEPLQAPAWVPSLFHNDRLLVYGFIPHCTQATLQAFIQEKEFCTMVSTTELQKTTGTMIHKL
AARALIRDYEDGILHDDETNHEMKKNIMKSLIIELSKENSLITQFTSFVAVEKRDVNEIPFANVPNISEL
VAKEDVDFLPYVSWQEKQPEASISQTEIDSSRLKHNKLSDGHGVLQLSHSPINLSEIPTIHDSEPPLLGF
KDLCSRDMGFSCGTAFSGSFASSKDFDPGKFSQGPNNISFSPKAPEMGVLHQSPFCSPPKPPSAPPLVTN
VLCSEAPQSYFLNLQSAAVHQSPNNRVSEIIMESVESSLPSDYSSRDASSYLALEGAEDSLLGGSSFETD
TDEAAAFIANDLLTSIETSSDEECAFCDEDQESPVPWASLFALQTENGFWKLTPELGLILNLNVNALLTS
LEEKGIRSLGTKGRERLLDLIATLLVLQFLYTKLEQEGMVAKSLIKMDDAFISRNIPWAFENIKKAREWA
RKTEGQYPSICQRLELGKDWESATKQLLGIQPQANTSLHRILYYSQG

>XP_046764289.1 protein mono-ADP-ribosyltransferase PARP4 isoform X1 [Gallus gallus]
MTIFANCVFFIKLKYSSIQKKNSLKACIEENGGAIDFVLNNKCTHVLVDNANVLSHHNLKITQKYQLPVL
YADFVWKSVENGKLLVTDAFEVNTSQGNISNQWPGGHGLEDLSSSENKIATNQNESNDSNLTVIGESDKR
IEFKVGTLRFHSENASVFPQDFEVAKYNVLEKINSKGSKDFVVIELHCSQEQYEFLFRLCAHFSASNGTK
KDKQFIINKTADEIRENYDILVANLKSQDFLLRETFPPEAECLASTKLQKLLLEESINSSAICQEVSDFV
ELIWAEALGHLDSLLLESVNNISLNDVSKAEGILLQVKNALNEGANETALQEMMMEFYQLIRHKTEIDYE
VTKRLLSRKQDLCQLMRDMLNIRETSMSCPNPSSLAKYQALRCKIDAVDPMSDEFLGVERQVLKNNYNGC
SVRILQVYRIGRVSETAEFLGSLGNVRSLFHASSVRNFMGILSRGLLMPKVVVEDHGVERTDTGNLGSGI
YFSDSVSASVKYSSPSEMDGTRLLAVCDVALGSCLDLYERDFSLNAAPSGYNSVHGVRKTADVSSDFEDD
EFVVYKTTQVKIRYVVKFCLAEDQIKQFQPGTEVEIEQDNTEPESQCHLQNESYELPSANISDTVKTGLQ
DRLGNPVPLESVHIKARIIDFVAQVVMFQTYTNQNSSPIEAKYVFPLDDKAAVCGFEAFVNGRHIIGQVK
EKKQAHREYRKAISEGDGAYLMDQDAPDIFVITVGNICPNSTVLIKVTYITELSCLNGCITFHMPASVSP
WQQDKALNENTQDTIKKICVKQVETLKKFSLDMSIEMPYSIESIHSWTHKLKIKKTECKAVIKTVENSSL
DSSGFGLDIWISHAYLPRMWVEKHPNKNSEACMLVFQPEFEAAFDEEQLSSEIIIILLDCSNSMAGSALLQ
AKQIALHALKQFSSRQNVNLIKFGTNFSEFSSFSKNTSKDLASLTEFITSATATMGNTDLWKTLRYLSLL
FPSQGHRNILLISDGHIQNESVTFQLVKDNVHHTRLFTCGVGSTANRHMLRSLSQYGAGAFEYFDSKSKY
NWEAKIQNQVSRIFSPGCSSVSIKWQQFDINAPKPMQAPAQIQSLFTNERLLVYGFVPHCTQATLRAIIN
DQELQTVVSTTELQKTTGTVLHKLTARAFIRDYEDGILHENETEHEMKKQILKNMIIHLSMENSIITQFT
SFIAIEKRDANEICSANVPNILEIIAQEDVDFLPYMNWEQKPMGSGTLFSGEPYGIPPFDAVLQDSDAQS
DCFTTKRCETEGLQLQPEDLTVTKESISQEVIPQDYGFHDDDIATVKFAAAMPFVPCKPFVMSYCNTAPL
FGSAVGAAYMTQKNSFPTPALASSGAHLRLCGSPVYSPPSPLVGMKYDNSFVDHIPNTIMFGSCASTYSS
DKTNAEKQVTHNSPVPQHHKKTKLKAKYRKKMVTGLQDATASEQEFPERPKRLLQPSCFITTEKERSQQK
LELTPVMWEEIFDLQNQDGSWNLSPQLGKILKFDVDYLINHFLIRNGIQSLGIKGKEKLLKLIATLLVLQ
FIRCANELQGIVFKSLMKLDNLTTSSGVHWAFESIKKATEWVKRVEGQFPSICYRLELGKDWDSATKMIL
GIKCN

>XP_041440458.1 protein mono-ADP-ribosyltransferase PARP4 isoform X2 [Xenopus laevis]
MTVAIFSDCVFFLKINDLSFKERKALKSTITSNNGVISFVLNQKCTHVVVNTIAFLSASQLKTVQKHQIQ
IVDSHFIWKCLEEKCLILDYQYLTAGVTPSEKEPQSWQADPEKTVFPENVPTLQNKDELWDRDYSEFDSE
TFTDPPDTEVAKYSFFHKGSNVAFLELLCFPESSSFLFKISKVICDSNGAEPDSSFQFANTAEASCQKYE
KCTEALEKQGFKRVKRIPPKVALLTSEALQKVLLEEAINATKLSPVVGYFVESVWTEALGHLDKILSRPV
NSISLNDVSKAEGILLQVRKALDTGTEPGDISKAMAEFYRLIPHKKPMLVNINKKLLSTKQDLCQLIRDM
VSVCETNLSKPNPSILAKYQALRCQIEHVESDTEEFHQVSQQIRENKHCEESIKILKIFRVGKLSEATGF
QRNLGNVKSLLHASSPSHFVGILSRGLLLPKNVEDDLGLERTDIGNLGSGIYFSDSISTGVKYSEPSVTN
GARLLLVCDVALGNCKDLYKRDFSLTEAPNGFDSVHGVRQVPGRKSDFEDDEFVVYSKDQVKMRYVVQFC
TAKDQVNMEHDKLPVWVEPTEEDVGEEPVQAEDPMLENLPEPRSIKGGLQSVQGNQIPLENIHVKGRMMD
LVVEVIIFQTYINKSSVPIEAKYVFPLDSTAAVCGFEAFINGKHVIGEVKEKKQAHQEYRAAIREGHGAY
LMDQDADDIFTVSVGNLPPNATVIIKITYITELNLEYETVDFSIPGTVASWQKDKALKENTQDTVEKICV
EESGVSKGCFSLEMSVEMSFKIHSIMSYTHNILMKKTDCKAVIATEKDSSFDDGGFRLIIYLEPTFLQSS
WNQPRMWVEKHPDEDSEACMLVFLPDFETNIQSWDLTILLDCSNSMESTFQSAKQIALLAANFFNSWLSI
NIISFGTSFKEFCVCPKEKNNLIPELVQFIKMAKPNMGNTELWKPLQSLCLLAPSSGTMQNVLLISDGHI
QNENRVFQILKKNAGKVRLFTCGVGATANRHMLRCLAQYGAGFFEFFEDKSKSNWEKKMEAQLEKMNSPA
CTSASVKWVQFSENDPEPVQAPAHIPALFKNSCLLVYGFAPRCTQAKLQALIDDKELDTMASTTELQKTT
GTMLHKLTARAVIRDYEDGFLHEKEHEHEMKKQQTKSLIIELSKKYSIVTQFTSFVAVEKRDAQEKPKDY
VPNVLEIISAEVVDILPYMTWETEKNKKIGVDFPGWKENFCGCYGGPVSVDERASLDEEEESLPFSFAPP
PPPDEEEESLIYNSPAVAFSFAPPPPPPPLSVAKTFDSPTPAVPFSFAPPPPPPFSFAPPPPPPPLALGM
PFSFAPPPPPPPLALGMPFSFAPPPPPPPLPAAKSHSTDEVYSLDVALLGAQQVSVCAPPPPPPPPGSIC
APPPPGFICAPSPPAFDELLQVLEQTQLAVSMIETEPEKAEHEGADFVRPARKKKIYYNTVEEPMGRLHG
SRRCVRGEEVALSQKRERAPPVFNALLDLQCEGSVCAPPPIRFISVTPPVALSQKRGRALPAFNALLDLQ
CEEGYWLLNEELGCLLNMNVNYLIEVFLSKNGIQSLGKKGKDEVLKLIATLLVLQTICTYELVNVTFKNL
MKLDESFCTSPFYETIEKSMKWARKRDKQYPGICYRLGLGKDWDSATRKLLSIDPVDPSSDLFPAVESSV
ESIV

>XP_014020296.2 protein mono-ADP-ribosyltransferase PARP4 isoform X1 [Salmo salar]
MDKSTYKSMAPFDNCFVVLELKTLPYKEKKRLKLAITENGGSISYVINKQCTLVVTISMSSLSASRVRSI
KKHQTPVVGVEYVSRCLEKGVLLPVEDYCLPPPPSTSLIPPRPASPLPSLSVLKGTSQPAEVQIQTVNAV
NQAESAGNQERTYLGKFRVYTESDNDLPTYPTNFQVAKYSIFGKANSSTWSVLELQSGSTEGGQQQYRVV
RYWREDHGAKSAAVQDKQVFLSMSEEAVEVYQTLRKELQVRGLTLRTTLPPEAPYLGSASLQQLLLEEKL
SCSSLSQEVGVFVELLWAEALGCLDNVLKVPITSLSLNDVSRAEGLLLQVQRKLRARQGEGPDTEVASLM
EEVYTLLPHKTDQSHLPLHTKLISSQLDLCQLIRDVLNVSEATLRSPIPSCLGKYRALRCSIERVPPGPE
LLYVTELLQDRQVQILQVLRVNRGVELQMFREDLGNVKPLLHSSSPSSFVGILSRGLLLPRVGVEHHGIE
RTDIGNLGGGIYFSDAMSTSLKYSKPSVTDGSRLLLVCDVALGRCTDLQKRDSSLTRSPEGYHSVHGVRH
TPKKPSEFDDDEYVVYSPEQVRLQYMVQFRLEEDQLKDFQPTIDTSSTELPLPVSPSDLLSCGEAEEDVG
SCKNPLEEVTAGLLDCSGQPLPLQAVHVRARLMDLLSQVIIFQTYTNQSTAPIEAKYVFPLDDSAAVCGF
EAFINGKHVMGQVKEKQQARKEYKQAIERGHGAYLMDQDAPDVFTISVGNLPAGATVLIKVTFVTELVVM
EGLINFSLPGSVAPWQQSSALNQRTQVTLEKVCVTNLDSQGEFSLDMSIEMPNEIINLHCLTHKVKIKRT
DCKAVVRTCPGETLGPDGFLVCFSLAQIHLPRMWVEKHPDKDSQACMLVFYPDFEPCPTSSPMSGSSEVI
LLLDSSESMRGDALQNARRIALQLVNTMDHHSVRVNVISFGSDQKDAFLSANPLVEALQPAKKFIMSSPP
VGGSTELWRPLRSLSLLPPSQGVRNLLLLSDGHVQNQALTLQLVRQHAQHSRLFTCGLSPTANRHMLRAL
AQAGGGAYEFFDTKTKHTWREKVACQVKRIMSPGCSSVSVKWQQFNPSAPPPVQAPSQLHALFTDCHTLV
YGFVPHCTQATLLGDLSGQNIETMVSTTELQKTRGTFLHKLTARAVIRDYEDGSLHTNEAEHEGKKSVMK
SFIIELSKEFSILSQFTSFVAIEERDSEQPNVGFTDVPKLISDEDVDFLPYLSWLEEEYEGDGMDLGSNS
MSNLLDGGPGRVLCRNICMEEMVMGEEHESLVQLQVDDESWEGLNYSYLMGSMVFGSPVTVSAATKPPDM
TLKSEGAVTDEEMECFNDMYFGLQESEHPLRGSVIMHSEALTSFYSPTLHTITGSQSTPVTPYLATKMLA
MTVETDFIPELSETIQSLPEIVRHAKSRQGDFRRADRHRKVCADPAFDTVETQKKSLSVADVPRPPPSVL
QLKSCAVADALLPPPASVVGSPGPPPPPASVLSSPGPPPSRLQSFSAAVSDALPPPPPAFVLSSPGPPPS
RLQSFSATVSDALPPTPPASVLSSPGPPPSRLQSFSATVSDALPPPPPAFVLSSPGPPPSRLQSFSATVS
DALPPTPPASVLSSPGPPPSRLQSFSDAVSDSLPPPSALVSNEFRAIKHKMRGHPKPGVGAISHTATFPK
LQAGKDLLLSKPREPYPVELCETSKMWTVDNSLSEDKDCGLVEMDHWQPSVVPASVPPSGFSFGGGRVGS
QSSQHHGSWFGDTANACYIPGLFEASSAGSLFGPTLPYLGSSGRSMGLSFEAPLTGGHLNSPLPTTHGFN
LTHPLGASLGSSLFGSSLRTLQQHHVLPQAQKPLDIKKNIQKLVGDSERREGLEFRKNTLDVVKWTEIFA
LQHSEGYWECSASLGSLLGVDVDYFANVFLKNRGISSLGVRAHADILRLVASLLVLQLMRVRRLEEGKLL
LSLFRLDLDHSPPQPRCERWEAVRRAVDWVCWADREYPCVCSRLEFGWDWESSTRQLLGIDPPHPLSPLI
LLGTTGGVRAQ

>XP_032828733.1 protein mono-ADP-ribosyltransferase PARP4 isoform X1 [Petromyzon marinus]
MGLFSACFIVLDLPSWPRRQRLQLKEQLTAHGAIISHAVTSHCTHLVVSAEGAGSPSRLKLAAKHGVRVV
CPEFLDRCFATGQLLEPALADDPADNNNIAVGNEERENGVVTSAIRTKTRDHKRGPPAEETFELNKVKLW
VDDDEDINRPAFPEEFEMVRSAIFCKTSEKEQTKEQYWAVVEVQAAPHSSGLVFRVAAYHATCKNSDKVT
SRLTKVRMAGSGREAEVFLGHACEVLTGERCGMVKVDQLPGDLQLGSRMFQQAQTEISMANFHLSKEVGR
FVSTVWSEALGHLSQILQVPILLIEPKQVTKAEAILLQLKKAVLSKDVHDEGALERLSNEFYKIIPQKHV
AVCPLTGMASISAKLDLCQLIRDVVDANEATRNKNTGGDDSSMRKENLALKKLTASSHSATTLAQFTALR
CSLLHLEPNSSDFNSIRNQLLQSSHGRLDGFKILKIFRVDRPAEIGQFRSEVGNTRSLFHAFAPRSLLGI
LSRGLLLPKTVVGEFGVERTDIGMLGCGIYFSDSISTSVKYARRAAARDGGRVLAVCDVALGRCHLVRRR
DRSLAAPPQGHDSLHGARATPGTASEFEDDEFVIYDPRQQKLRYIVQFCLESDVVKEEENSDAEEFYEAP
ETPADERILPEPNIPELLPEQGEPKQRLSGLHSGVGSPVPLKAVHVRANILDFTAQVVVMQEYDNNSTEP
IEAKYTFPLDEMAAVCSFEAFINGKHIVGQVKEKETAHQQYRNAVSQGHGAYLMDQESPDVFVVSVGNLP
PACRVLIKVTYVCELAAEGAAVRFRLPGNLAPWQQRLATGDVTQETVETLAVAKHSGRLSVQVSVEMPYE
IINVTSPTHAIRVKRTACKAVVELASSDDSLGSEFQLLVFPAQIHEPRMWVEQNPETDSQACILTFYPEF
APSHSEGVDIVLCLDCSASMTTQPLTDVKRLALFALRHLPPGSRFNVLKFGTHYKELFPVFRESSPSNIA
TAVSFIKRLHADAGNTELWRPLRSVALLAGGVGGSRDAAAPPVSVLLLTDGVAQSEARATREARENVRLL
RVLACGVGETPNRYFLNALARNSGGACEFFTAKNRSTWTDKMLALMEKATQPVCRDVTVEWQRFDRGALG
GDRVEMQAPRMIRSVFCGTRLVVYGIVPHCTQATLHAIINNQEFSTMVSTSNLQITRGKMLHRLTAHAII
QDYEDGNLHPDETQHEVIIAEMKSRIIELSKEYSIITPYTSFVAVEERDSTLADEATGPALDELLSKESV
DFLPYVGWVEEEKARKKKGSMQFHLRAEKTKAPVGGRGGLASSKIKYRKTRPACFSLIPEKTEQTPEIEF
GSAAERANLSPFEIEAAADTASQYPSEIQPKFAHASVEAVPCSNEGPLNLSRRDVKDHGVSASRITTMSA
SSRFGERHLLHRAAPVILKEAFECRRSQQGFGSSAYAPRSVVHQLNSAETPVPLIRAQAFAPLTIFDNVQ
PPGVSLGVIAMETPSRFTPPYQDTFERGQARRQSDRFTNSAYPFKPQKCLARVKKPPPPLLCFTARSPSS
AALMPDVHELRAAGLMSRVPECAKRDVCALQLPNRDSPPTDFPEPPRLPPPPPAQHASRFGQPMHDDFQS
STCQAASRLCEESPFGGSSNLSESFSSEDDYGMPFESCLMFHTSPGVSLCGSSGSSEFSDTTPWQHADLS
KITCLQNKDGSWDGGVELSSLVGWSPDHITVILKKQGIQSLGLRMEEAIVRLVWTLLVLQILRLRMGLAS
SLNEFVLLLLRRDVSTLAELDMFMPGSADAFARAASWIRSTESVCPCVCTRLELGPNWQAATCSLLGVA

>XP_019636962.1 PREDICTED: poly [ADP-ribose] polymerase 4-like [Branchiostoma belcheri]
MIFQPPEASSKEKVWKEGEKGPDGAFKHDPMEGSVSDPLLVDGRFEVARYVFLQKDHCKVTKKGAAAPVV
QVLELQVASGDPDSSKGEFKYRVMTQETGGAKEYRYMQTAGEAVQVYGILYDRCTKHPHSYSRLAPDGDT
PWTVGSPQLHQMLREVEARPSSVSPRVLGLVDSLWEETLGNLSHILTGDVLNINLDQIEKAEAILLQLKQ
AVERKAPNDELQKLSSEFFSAIPHRPDHKDTIKNKREIARKQDMCQLIRDMVSVSEATGWTSHPSAVARY
HALRCNIQSLARDSEDYRTLESMIHSSQENGTPPIKVESVFRVCRSMEETGFTSAIGNIQTKFHSSQAQN
FVGILSRGLMLPKVVVDDFGGTRTDAGMLGHGIYFSDASTSAKYSSPSKTRGRRYMVVSEVALGKCKDVY
QQDTSLTQPPQGYNSVHGVKNTDDIPSEFKDNEYVIYNVQQQKMRYLVEFSLPGDQVTPVDEAAYLHLLT
PEVEIEEDQDLNKISLEDVQNIADPLDKVKGGLISKSGEEMPVPLKSVHVRAKLLDLAAQVIVLQSYKNE
NNVAIEAKYVFPLDDMAAVVGFEAFINGKHVVGEVKEKEEAHREYRQAISEGHGAYLMDEETPDVFTVSV
GNLPPRASVLIKITYVSELAVEGENICFRLPGSVAPWQKDSLSEEIQKDVETVKVGKDAGKEFSLQVAME
MPFDIRTIQSPSHKIRVKRTASKAVVELEKNCMLGDGFLLQVGLAEIHVPRMWVERHPDKDSQACMLTFY
PEFQAEVMQGHEVILLLDASNSMRGSPLEAAKKVALLCLSHLPKECSFNVVAFGTGYEELFAVSQPRIKS
NVSKAENFIQDLKASKGNTDAWRPLHGFFLLPPSPGKTRNVFLISDGHVNNADQTLSDIHAHYLETRVFS
CGVGSTSNKHLLRAIARVGGGAFEYFDDKTKSKWERKVKSQLWKAAQPGLTSVSVDWQQWDDDAPPPVQA
PNQIVSLFNGSRQVVYGYVPHCTQATLKAVINNREISTMVSTSELSITAGKILHQLTARAVIRDWEDGTL
HVDRTHHETKKMGLKPYIISLSKEYSIVTQFTSFVAVEKREQGEDLTTGDGPTIKELVQKESVDILSYMG
WQQDEGQLDSTELFEVKVGFLGLSDGKRDPEIVRVQLTDDSLVLQREETVYGNSLAAISSQVNNFDIDSN
RERSVILRRQKSGGLGLSIMGGAEHNIPVLISSIVKDQAADQTGQLFVGDAIIKVNGVNVEHSTHDDVVQ
HLKNSPDDVKLTVRYYRAATPYLRAPAKWSRYSSEQCNGNASPADNETTSSSEWKAEVTDYEDMSCEMVT
EIMTLGYSEGVQKMAGKILDSSVELEEESESDEDMEFGLFDQEGWMAADEEKEILAKPEMEKKKVALLEK
CSRLASPVMSRSAVATSTAYSPTSPTKLVAYSVYSKPSPQKMALKRAEKELEAPRPETDEVTVDVTLKAS
LDGIQARGDRLEDLIEKSDTLSAEAPVFFQASTAISEELFAATAEPTVHEATLVESKPAVLRKSGRAPPK
GARFVREVEPEPVSEEVAQSAPSAASRSAPAFGFIPPGASFGKKSAGLVGGPPGTTAEETKTKEVAFGQG
FGSDAVSQGAGFGATPFGAPPLDATPFGAPPFGAPQFGAPQFGAAKTKTQEAQRFGSDAVSQGAGFGATP
FGAPPLGATPFGAPLFGATPFGAPQFGAAKIKTQEAAFGQGFSSNAVSRGADVSTTPFGATSFGATPSIA

TPFGGTPFASTISLFGHSTTAAKEPEAALFGFASSATPSGGGGFSFGSTSSDIPLKDEDKSSVPIDSSKG
LKFGAPLEEKGGTTGFRFGGSSQAKMIPQQGGFISHPQMQQQQQAQSNVPDRARIPGRKAQSARKTTGGK
ALRPAADKVASDGVKADSSPDTVVFMDESDKAKETKTPTSAKSPLKMIPATCCMVCYDMPPLYQQGSPPP
PAPSRGPPPLPPTSQQEPPPPPPPPCPAGPPLPAGAPPPPPPQFRKSLLLALSQEKLKGKSGQSPTEMIQP
PPSRAPPPSMGGRPPPPPSMGRRPPPPMMTRAPPPNMARLRVDADALYVYGLGETTKRMGARIGARIGAR
SLKKAAELPKGEEEEPMEVEESPAICGEQVEEESMDLKLRKLKKKKVLELRAEFEKKYESLPVDEREERD
GRSSLLNQIMRGTSLRRGPGAYHSASFHPTSDSAGYFSGTETVVVPIVTTRGSGLTEKTVDQLFALLKHT
DQGSYWEFSPELDQLLGISSSTCVQIFNTAGLRSLGAKVAKQLLQLVATLLVLQLLMLHLPQLFPSCSSL
LHLDVMQVAAEWRSAVQQVLAWAKGVDTMHPSVYSRLELGKSWDDLTKKLIGVGVSLVHE

>XP_032219396.1 protein mono-ADP-ribosyltransferase PARP4 isoform X1 [Nematostella vectensis]
MSVIFSGLLFVLDSSGLPGFSFKQKQELRSKITSNGGTISYILTKKTDYVLTSDGERTLSSYKGRTAAKL
GVPVVHIDFIDSCLSSGQLSAPDPTYILSETPESKQFSSGKIVTAPKQGGCFNKKVSSSVQVLQADLSKI
RFFPYKGSKGPSYDEKSYEIAKHVLYQRRADAGEVLVFSAIELHVIPPSTCFDGHHYRVFHHHGNFKALK
EGNDAEQCTKECSYHGSSEEALGTYIALCAKVTQPPHNLTCCWKTLSSKIGSDKMKEESMDTRSSGEGES
VSQEVASLVDYLWTEAIGCLNSTLSVPLESIKLQKVQEAEAILMSLKEEFDNTQNKEKISKLWEEFDSLI
PHQIKIKDSTVNKGLIAEKQNLCQLIRDMVTISEATNMSTVSSHLAKYKALRCHIEALHPEEAEYKAIKE
LAIEEQTGSNDIIVLNVFSLHRPVEDINYRHGLGRQKLLFHASRVRNFVGILSRGLLLPTIVVDDFGGQR
SDAGMLGHGIYFADRASTSAKYSSPGMLGSRFMLVNKVALGKSKEYRKFTKDLTRPPDGYNSVIGVKATE
TQPSDFKDDEFVVYDTKQQQMRYLVEFALPEDDIIARDNVSVDSGFVEEPLDNEDEIEVDLSDVMSIPDP
LLKVEAGLKGTADKPVPLKAVHIRAKLLDLAAQVVVMQAYCNNSDVPIEAKYVFPLDDMAAVCGFEAFIN
GKHIIGEVKEKEQARKEYREAISAGHGAYLMDEETPDVFTVNVGNLPPAASVLIKITYVAELTVEGELIC
FRLPGSVAPWQRTQAHDDVTQTDVDTICVDEENQSNTSVQVSVEMPFEIRAILCPGHDVKVKRTSTKAVV
EMAPGGTLGSGFQLQIQLAEIHVPRMWVERHDTEADSQACMLAFYPEFESGPPHHVEVIFVLDASCSMKG
KALQEAKKLTLMCLSLMEEEWAFNIVVFGSNYSELFTQSQKKDKETVARAAKFVKSVKAVKGSTDLWRVL
RSLYLLRCNSTADYPSNVFLFTDGHVTEESTTLAYIRDIRLRTRVFTFGVGPTCNRHFLRSMARVGAGAY
ELFDSKVKSKWERKVESQLSKARQPVLTSVKVAWQQHDSYAKPPVQAPHDIVSLFNGSRQVVYGLVDNCT
QASLTAEVDGRVVSTMVSTSELSITTGKVLHQLTARAIIRDWEEGSLDPNHTKHEMAKRDMKDFIISLSK
RFSIVSQFTSFVAIEHRKKDEKFDASLGPSIDELVSHEDVDVIPYVSWEEPESRDVGKPESSSKSTGDRV
AGLVSEARVLERFSVLQAETLYKRACDTALSAESPDNRATYKAFLSLAQFYVDVHGDLQKAEALTMMLHG
ALGGIEHDVSSRSNFDDLLEHLRQDSSYSDEVGRLVAEFNPMKDINKSKRKVIASPFPNVDYPWSQTVVN
KLFEACRSESSTETGSTASSTSSSESSSEEELSDNSEDSDWGSDEFYEDNIDDEVYQDAVGEEEEEEGED
DVYGMCLEEAEEVERSEAPTPLVIDVGSHMWKVGFAGDDAPKGVFPPIVGRPRHQGVMVGMGQKDSYVGW
EAHSKRGILSLSTPQKREARPTPPLQDRPPPPLQDRPPPAMEGRALTKDRVIWPIEEAPLDHTASIDLEE
QEDLYVDMDKGEVLDDDGLFEAMCNNAQKAASLAMGKRRQDVDQLTLDSYDDIHEVLPSNTQVAATLDTD
DMYVDMKNRATSSALKERAVSYDIQDSLCEVAEDAVKEEEAERPIAIFGLDSCIARVSSSEEKSSLYDEI
PSPPNRPFKKKVANIPSPKPRPPRASKIAAPPAPSLQAVCIDLPEPPLKAGDSPLKRSVKAPLPPPPPQQ
ASRIDYRASYGAAPPAPSLQAVCIDLPEPPLKAGDSPLKRSVKKPLPPPPPQQAARIDYRASYGAPPLPP
KRGGGPPLPPKRGGGPPLFGAMALRTGAPSKASKPSFGGRGQPQVFATSASEILHPRLARTPSELAPKGV
PSQTNDEAPVFMSRLQLLESIRAKKKTKETDREDIMKQDDSGEADILRREAHQHSMETELEADRNEAEMG
VRPVPRKRGKRMLEKKAAEQKKEAMEEPSESLQNRMRKWMTADKLLELFASQHNDGYWSEKELEVAFRID
CSFLSRLLVNAGAQSLGDEAFETILKMLTTIITLAILKTYLPDQFPVSLVQGVSSEGLVTSEWRNQVGRA
LSWLRKMDRKMPSVCSRLELGPDWDVVAKGIIDDKVHLMIGVR

>XP_047129290.1 protein mono-ADP-ribosyltransferase PARP4 isoform X5 [Hydra vulgaris]
MFSGCIVTLDLATNASFREKEILRKAIVNNGGVVSYIITKKSTHVVVASKEFAQTSYRCERALKYGVPVV
TSDFITKSVEAGFLLNHESFLAFKIEKESVDELKSGKLSTKSSNNNNCMKKVSEAEVLLNCRVWSDGKGP
WNNDILFEVPKYFLLLENSKSDGNRPFCLIELHVSCDGAWRVWCQYGNNVTEKETSKNVCEYRAAKDAYE
CEILFGACINQYKKEFQNKNELSEPLSKSYAIGSKYLYEWPGRNSEVRTEMTSNLSHLVNTIWADAQGNL
KDLLANSIHSYILADVVRAEGILLQIKECFEKKDEKLKTLSEEFYSIIKLKKNNNVLDNAKIISRMFDLC
QVIKDVVGVSESTNWKTIPSLNAKYKALGCNIKELHYEALGELKKTISMSSMEAKEIEVLSAFELNRISE
KGGVDFVGPFFHSTSTENLVGILSRGLLLPKVVVDQHGGERTDVGMLGNGIYFASAASTCIKYSKPSQTS
GSRFMLLCELNLGKTKTYRKFQKDLNEAPAGYDSTHGVKSTSNEPSDFDDDEFCVYDVSRQRITHIVEFK
LASDNVHHTLNNDVSEDEGIESDFDSESELSIKDVDSVQDPLSKVVSGLQSSTNQPVPLTAVHIRAKLQE
FSSEVVVLQAYRNDSINPIEAKYVFPLESSAAVCSFEAFINGKHIVGKVKEKEQARKEYKEAIQQGHGAY
LMEEQEESPDVFTVTVGNLPPNSDVLIKITYVAELSLEDDQISFSLPGCVAPWVKDQALATKMQNELETI
NVNSFGEFSLEISLEMPCAIRSIVSPTHGNILIKKTATKAVVKMNDPLGTSFQLLIALAEIHVPRMWVQK
SPQDSDKQACMLSFFPEFDGDIGDHEVIFLIDASNSMKDHFIELQKLVYLTYEKMPQSTKFNMVIFNDTY
KELFPYSRFKDESKQQVMSLLKLLKNCNGGTHLWSVLKMIEMLSCESTMKNVFLFSDGHIFDEISTLARI
RNLKKNVRLFTFGVGTTPNKHILQKMACLGSGYFEYFDKNAKSKWQKKVELQLEKCQQPVLTNIKIHWIQ
HDEDAPKPLQAPNEITTLFNGNRLVVYGFVPHCTMANLSAILNGKELNTIVSTSELSTTVGLTIHRLAAK
SVINDWNDGMLHQDQYKHEAIKAEMKGKIIQLSIEYSIVTKFTSFIAVEEREKNEGKDVTAPTINELVLM
ETVDILPYMGFETKTKSVPMDKAKFSLSEILDGMHMLKDELSLSEDDYDNDDSSLSRQESEEEKKLYKKP
IILDIGMAETKAGFAGCRAPKVVIPTLVGRPRHQGVMVGMGQKDAYVGNEALSKRGILTLLSPFKLDKGS
ETDSTKPLQLIGDTGNIVKPETSKAINPKFKIAKCKRELSKGLSTLQHSSVQQSINIDSDSVDYQKSYIM
LTKQKECAEIEQTSDDFLSMQSDNLMMDYCEMLASKESIYLESDSVKDIHQFKLREKKSATPPPSSTRNV
SSLRKKQPFPEPMVSKVCLQAKMPDLPMPPPPQLMPLFGAKQPSAEPMLLKACSPAKMPDLPMPPPPQLM
PLFGAKQPSAEPMLLKACSPGKTSFFSQPPPPPPAQSMYLFGAKQPLPEPTLSKDCLPGKTSFFSQPPPP
PPAQSMYLFGAKQPLPEPTLSKDCLPGLMGKKLAAPPPAAQSVSLFGSNKSSLMFSRNSCFSDDLGMPKR
EEHSLDLCDDLEMLADEKLSLDYRPAATGTISACHFVKPISCAYYEPCFLERKKTSIENGLKSVFDCFFK
DEKFERPFLDGRSFVFLNKTSNEDLINNTLEFLVENQHSDGYWELEDIFQDQQSTISFDILFVDVCRKLA
LSLCGEKYSSEIQRLIATIIVCVIFKLALSNDKALWSYPYHLRESNLKASDKLTSAFFKGKSYILHEDCK
FPSIHTRLGLGYSLEEMTFQVYRLASQSFDKRMELRL

>XP_019851276.1 PREDICTED: uncharacterized protein LOC100633591 isoform X2 [Amphimedon queenslandica]
MENLDSLFRGCQITLELDSSIPFKKKSALKQSVIQYGGIVSFIVTKKTTHLVVNNVEKAQDSYKSRMAQK
WGIPVVSIKFIEDCIENGKLLEPDQFVVAGKTPSEELSSGKIVASMEDKSNSDRKKRRPKSNVNLNQIKV
WPWPHADGMKTPLFPEDGYEIVRHAFFKKYEKMSKLTSFVAIEIHVVPTSVTEDKPLYRVFVHKGHLEQR
LKTGELGTCECRYVETSLDAEAVYAYLCKQYTSSPYSMERVQYLSFMIGSPGLRKLMSETWPRGVAGSRE
DLDPLVSKLVEYVWSEAIGEIEGFLTVPLQNIKLEQVEKAEAALMSIRKLLDEGRTGDDEDIKKYTKDYN
SALPNSKSAPIDSKKVIANRQEFCQVIRDLVSVSESTNWSHKSGPVAKYHALRCQMCPIDPVSKEYSDVK
EHIMSSLTNDSNDPEILSIYSVQRPVEESQFSHSLGNRKLLFHSSQLQNFVGILSRGLLLPKIVVDDFGG
SRSDPGMLGSGIYFADSASLSVKFSGPGKKRHGTRLMLVNEVALGNVKEFTSYQKELLAAPNGFHSVKGV
KGTTEEPSDFKENEYVVYKTEQQRMRYLIEFRLPQDEEIDQDETESEKYENEEIEEDEESNIVTDGVSLS
DVKNVTDPLSKVEAGLKVDGGVVPLKAVHVKAQLIDLAAKVIVLQSYKNESLVPIEAKYVFPLDDMAAVC
GFEAFINGKHIIGEVKEKEQAHREYKQAISEGHGAYLMDEETPDVFTVSVGNLPPGASVLIKITYVAELQ
AEGENVVFSLPGSVAPWKQEAALDETTQVDVEKVKVRSDADSLSVQISIDMPFTIRTIESPAHKIKMKKT
DTKATVELCPGEKLGTGFQLLIGLAEIHVPRMWVEENDKGHHACMLTFYPEFEAESIVENEIIFLLDVSN
SMKGDALDNAKKVLLLLLHHLPPKTYFNVFTFGAMFESIFPSSVQINNETLSFATKQVQSCQAVMGNTDI
WRPLHSLYLLSEAASSSTGGLTNSSTLPPCSVFVISDGHMTEEAPSLSAIKDGAQNYRVFTFGVSSTANR
HFLRSMARVGGGCSEFFDSQKKSRWERKVKEQLSKAFQPALTGVDVQWQQHDENAPPPIQAPKSITSLFS
GSRQVVYGFVPHCKQASLKAFIGRKEIQTMVSTSDLATTSGQTLHQLTAQALIRDWSEGSLNDDRTEHEI
MKRDLKTFIINTSKEYSIVSQFTSFVAIEKREKDEKFDATKGPSIEDLVSKENVDKLKYMGWEIDQGADP
VKIAAESIDDVIASMAGETDVIKLDTAFNKVWESTQNTLPNSHPARVKLLKMRLEVLKKMDKLSDGQELL
EGLITDLHLRGSIDSTRLINVLEGIRDGFSRMIDAKRKAAEAEAARRSRMMIVAEEAKSLRQSTLETERD
GGIIGEAKMELKSRIAFKQKEIESLESKLHLITRRRGKALKKDIPPAEERKASPKVPLKKKKAVAKNRGP
IREQESPEKDEDISVAEEGRRSIRMEEEPEEEDIPVAVIDTGMDTVKAGFAGDDGPRSVFPALVGRPRHQ
GVMVGMGQKDSYVGDEAKSKRGILTLKSPFERPSKPMAAMSYSQPIVQVEEKEKESSATVEEDVLNDLFG
YDGRNYEISDFSMAENYQVNEVLMDELEMDESLMEQSLMVESAELYEELEQQSEETIAVNQKELRSRKVK
KSIPGTSPRTSLLKGLGSKEPQEGQLGTGLAGFASKPPEGYLSLDIEGLAMPANKETYSPLSQTYNPVSP
VYSPTSVTSPAYSPTSPSYSPSLPSYVPTCTSPAYNAPTVNGKLSAPSVPGLTFNRSSDKVSSDSSETSR
NISQLSSAYLGSGKSRRFESVSLEKSAPLSQPTAAPPSRSRIVRFEAGALTFGSSSQAPLEPVPPPPLPP
RAVMTKSAPSEPIYPSFDGIPAFSVRNAISSSPEGSPPELTPWSSPSFKSPPLPRSAPLPGGPPAKPSRS
SGRALMQSPGGPPPPPPPPPGTGAPMQLSEKSPPPPPVPLRSRDRGPMPVPQSTVLSSGRPPPPPPHFMLC
GEVPMPSGGPPPPLPYLSRGIAQRRLPGGPPPPPPPPPPGAAGAPLPGSSKVAPKGKSKVSYYAGLADKK
TTQASPFSKAGGKKEPSGPTGRGALLSSIKKGVSLQKVTVEKKEEEKSVSASLMQALQSRKAPEAFNLKL
VDYSKWKSVEMSDDDSWDDDEEDDDEGGGGGGGRSRLLVNEFFAFQDQERAEDKVQGQEEEEEESDDNMA
FGLFDSFDEEALSNASYYSMSENAQESDGEKVIEVQNQFPVINSSQIYMMYSLQKDDGSWLLSDLDTIVM
PGTGDRVLKVLNEAGAKSLGSRVYKELLSLIATCFVLRLLNLNFPDAYKITFSPHFEIDMSNGDPGDIAT
KMNKSLVLCNKLHKQNPSVVTRLELGYSLIDAVDKILKLCA

Alignment

```
XP_047129290.1[H.vulgaris]           ----------MFSGCIVTLDLA--TNASFREKEILRKAIVNNGGVVSYIITKKSTHVVVA           48
XP_032828733.1[P.marinus]            --------MGLFSACFIVLDLP---SWPRRQRLQLKEQLTAHGAIISHAVTSHCTHLVVS           49
XP_032219396.1[N.vectensis]          -------MSVIFSGLLFVLDSSGLPGFSFKQKQELRSKITSNGGTISYILTKKTDYVLTS           53
XP_019851276.1[A.queenslandica]      ----MENLDSLFRGCQITLELD--SSIPFKKKSALKQSVIQYGGIVSFIVTKKTTHLVVN           54
XP_019636962.1[B.belcheri]           ------------------------------------------------------------           0
XP_014020296.2[S.salar]              MDKSTYKSMAPFDNCFVVLELK---TLPYKEKKRLKLAITENGGSISYVINKQCTLVVTI           57
XP_041440458.1[X.laevis]             ------MTVAIFSDCVFFLKIN---DLSFKERKALKSTITSNNGVISFVLNQKCTHVVVN           51
XP_046764289.1[G.gallus]             --------MTIFANCVFFIKLK---YSSIQKKNSLKACIEENGGAIDFVLNNKCTHVLVD           49
EDL36193.1[M.musculus]               ------MTLGIFANCIFCLKVK---YLPRQQKKKLQTDIKENGGKFSFLLNPQCTHVIVD           51
NP_006428.2[H.sapiens]               ------MVMGIFANCIFCLKVK---YLPQQQKKKLQTDIKENGGKFSFSLNPQCTHIILD           51
NP_001125927.1[P.abelii]             ------MMMGIFANCIFCLKVK---YLPRQQKKKLQTDIKENGGKFSFSLNPQCTHIILD           51


XP_047129290.1[H.vulgaris]           SKEFAQTSYRCERALKYGVPVVTSDFITKSVEAGFLLNHESFLAFKIEKESVDE---LKS           105
XP_032828733.1[P.marinus]            AEG-AGSPSRLKLAAKHGVRVVCPEFLDRCFATGQLLEPALADDPADNNNIAVGNEEREN           108
XP_032219396.1[N.vectensis]          DGERTLSSYKGRTAAKLGVPVVHIDFIDSCLSSGQLSAPDPTYILSETPESKQFS-S---           109
XP_019851276.1[A.queenslandica]      NVEKAQDSYKSRMAQKWGIPVVSIKFIEDCIENGKLLEPDQFVVAGKTP-SEELS-S---           109
XP_019636962.1[B.belcheri]           ------------------------------------------------------------           0
XP_014020296.2[S.salar]              SMS-SLSASRVRSIKKHQTPVVGVEYVSRCLEKGVLLPVEDYCLPPPPSTSLIPP--RPA           114
XP_041440458.1[X.laevis]             TIA-FLSASQLKTVQKHQIQIVDSHFIWKCLEEKCLILDYQYLTAGVTPSEKEPQ-SWQA           109
XP_046764289.1[G.gallus]             NAN-VLSHHNLKITQKYQLPVLYADFVWKSVENGKLLVTDAFEV--NTSQGNISN-QWPG           105
EDL36193.1[M.musculus]               SAD-VLSRCHLNSIQKNDVQIANPAFIQDSVRQRRLLDVRNYDP--LSPAPAAP----PA           104
NP_006428.2[H.sapiens]               NAD-VLSQYQLNSIQKNHVHIANPDFIWKSIREKRLLDVKNYDP--YKPLDITP----PP           104
NP_001125927.1[P.abelii]             NAD-VLSQYQLNSIQKNHVHIANPDFIWESVREKRLLDVKNYDP--YKPLDIIP----PP           104


XP_047129290.1[H.vulgaris]           GKLSTKSSNNNNCMKKVS------EAEVLLNCRVWSDGK----------------GPWN           142
XP_032828733.1[P.marinus]            GV-VTSAIRT-KTRD-HKRGPPAEETFELNKVKLWVDDD--------------EDINRPA           151
XP_032219396.1[N.vectensis]          GKIVTAPKQG-GCFNKKVSSSVQVLQADLSKIRFFPY---------------KGSKGPSY           153
XP_019851276.1[A.queenslandica]      GKIVASMEDK-SNSDRKKRR--PKSNVNLNQIKVWPWPHA------------DGMKTPLF           154
XP_019636962.1[B.belcheri]           ------MIF-----Q-PPEA--------SSKEKVWKEGEKGPDGAFKHDPMEGSVSDPLL           40
XP_014020296.2[S.salar]              SPLPSLSVLK-GTSQ-PAEVQIQTVN-AVN--QAESAGNQERTYLGKFRVYTESDNDLPT           169
XP_041440458.1[X.laevis]             D--PEKTVF-----P---------E-------NVPTLQNKDELWDRDYSE---FDSETFT           143
XP_046764289.1[G.gallus]             G--HGLEDL-----S-SSENKIATNQNESNDSNLTVIGESDKRIEFKVGTLRFHSENASV           157
EDL36193.1[M.musculus]               E--R---SR-----S---EVQ--SE--------YLPSDNTPEKENTEVTEVSAENVEIPP           141
NP_006428.2[H.sapiens]               D--Q---KA-----S-SSEVK--TE-------GLCPDSATEEEDTVELTEFGMQNVEIPH           144
NP_001125927.1[P.abelii]             D--Q---KP-----S-SPEVK--TE-------GLCPDSATEQEDTVELTEFGTQNVEIPH           144


XP_047129290.1[H.vulgaris]           NDILFEVPKYFLLLENSKSDG-----NRPFCLIELHVSCDG--------AWRVWCQYGN-           188
XP_032828733.1[P.marinus]            FPEEFEMVRSAIFCKTSEKEQ---TKEQYWAVVEVQAAPHS-----SGLVFRVAAYHATC           203
XP_032219396.1[N.vectensis]          DEKSYEIAKHVLYQRRADAGE-----VLVFSAIELHVIPPS--TCFDGHHYRVFHHHGNF           206
XP_019851276.1[A.queenslandica]      PEDGYEIVRHAFFKKYEKMSK-----LTSFVAIEIHVVPTS--VTEDKPLYRVFVHKGHL           207
XP_019636962.1[B.belcheri]           VDGRFEVARYVFLQKDHCKVTKKGAAAPVVQVLELQVASGDPDSSKGEFKYRVMT--QE-           97
XP_014020296.2[S.salar]              YPTNFQVAKYSIFGKANS---------STWSVLELQSGSTE----GGQQQYRVVRYWRE-           215
XP_041440458.1[X.laevis]             DPPDTEVAKYSFFHK-----------GSNVAFLELLCFPE-----SSSFLFKISKVICD-           186
XP_046764289.1[G.gallus]             FPQDFEVAKYNVLEKINSKG------SKDFVVIELHCSQE-----QYEFLFRLCAHFSA-           205
EDL36193.1[M.musculus]               FLQDFEVVKYNILEKV---G------GPETVVVELQSSQD-----PESCPFVITAHFLL-           186
NP_006428.2[H.sapiens]               LPQDFEVAKYNTLEKVGMEG------GQEAVVVELQCSRD-----SRDCPFLISSHFLL-           192
NP_001125927.1[P.abelii]             LPQDFEVAKYNTLEKVGMEG------GQEAVVVELQCSRD-----SRDCPFLISSHFLL-           192
                                          :: :     .                 :*:               : :

XP_047129290.1[H.vulgaris]           --NVTEKETSKNVCEYRAAKDAYECEILFGACINQYKKEFQNKNELSEPLSKSYAIGSK-           245
XP_032828733.1[P.marinus]            KNSDKV---TSRLTKVRMAGSGREAEVFLGHACEVLTGERCGMVKVDQ-LPGDLQLGSRM           259
XP_032219396.1[N.vectensis]          KALKEGNDAEQCTKECSYHGSSEEALGTYIALCAKVTQPPHNLTCCWK--TLSSKIGS--           262
XP_019851276.1[A.queenslandica]      EQRLKTGEL--GTCECRYVETSLDAEAVYAYLCKQYTSSPYSMERV-Q--YLSFMIGSPG           262
XP_019636962.1[B.belcheri]           ---TGG------AKEYRYMQTAGEAVQVYGILYDRCTKHPHSYSRLAPDGDTPWTVGSPQ           148
XP_014020296.2[S.salar]              ---DHGAKSAAVQDKQVFLSMSEEAVEVYQTLRKELQVRGLTLRTTLP--PEAPYLGSAS           270
XP_041440458.1[X.laevis]             ---SNGAEP---DSSFQFANTAEASCQKYEKCTEALEKQGFKRVKRIP--PKVALLTSEA           238
XP_046764289.1[G.gallus]             ---SNGTKK---DKQFIINKTADEIRENYDILVANLKSQDFLLRETFP--PEAECLASTK           257
EDL36193.1[M.musculus]               ---ADQK-T---RRESTGKQTSEGAIEYYESYVEDLKRQGFLLQEHFT--AEATQLASEK           237
NP_006428.2[H.sapiens]               ---DDGMET---RRQFAIKKTSEDASEYFENYIEELKKQGFLLREHFT--PEATQLASEQ           244
NP_001125927.1[P.abelii]             ---DDGMQT---RRQFAIKKTSEDASEYYENYIEELKKQGFLLREHFT--PEATQLASEQ           244
                                                   .      .                                 : *

XP_047129290.1[H.vulgaris]           ----YLYEWPGRNSEVRTEMTSNLSHLVNTIWADAQGNLKDLLANSIHSYILADVVRAEG           301
XP_032828733.1[P.marinus]            FQQAQTE-----ISMANFHLSKEVGRFVSTVWSEALGHLSQILQVPILLIEPKQVTKAEA           314
XP_032219396.1[N.vectensis]          -DKMKEESMDTRSSGEGESVSQEVASLVDYLWTEAIGCLNSTLSVPLESIKLQKVQEAEA           321
XP_019851276.1[A.queenslandica]      LRKLMSETWPRGVAGSREDLDPLVSKLVEYVWSEAIGEIEGFLTVPLQNIKLEQVEKAEA           322
XP_019636962.1[B.belcheri]           LHQMLREVE-----ARPSSVSPRVLGLVDSLWEETLGNLSHILTGDVLNINLDQIEKAEA           203
XP_014020296.2[S.salar]              LQQLLLEEK-----LSCSSLSQEVGVFVELLWAEALGCLDNVLKVPITSLSLNDVSRAEG           325
XP_041440458.1[X.laevis]             LQKVLLEEA-----INATKLSPVVGYFVESVWTEALGHLDKILSRPVNSISLNDVSKAEG           293
XP_046764289.1[G.gallus]             LQKLLLEES-----INSSAICQEVSDFVELIWAEALGHLDSLLLESVNNISLNDVSKAEG           312
EDL36193.1[M.musculus]               LQALLLEEV-----ISSGALSQEVSDLLEVIWTEALGHLENTLLKPVNSMSLNDVSKAEG           292
NP_006428.2[H.sapiens]               LQALLLEEV-----MNSSTLSQEVSDLVEMIWAEALGHLEHMLLKPVNRISLNDVSKAEG           299
NP_001125927.1[P.abelii]             LQALLLEEV-----MNSSTLSQKVSDLVEMIWAEALGHLEHMLLKPVNRISLNDVSKAEG           299
                                                        :   :  ::. :* :: * :.  *   :      .: .**.

XP_047129290.1[H.vulgaris]           ILLQIKECFEKKD-----EKLKTLSEEFYSIIKLKKNNN-VLDNAKIISRMFDLCQVIKD           355
XP_032828733.1[P.marinus]            ILLQLKKAVLSKDVH-DEGALERLSNEFYKIIPQKHVAVCPLTGMASISAKLDLCQLIRD           373
XP_032219396.1[N.vectensis]          ILMSLKEEFDNTQ---NKEKISKLWEEFDSLIPHQIKIKDSTVNKGLIAEKQNLCQLIRD           378
XP_019851276.1[A.queenslandica]      ALMSIRKLLDEGRT-GDDEDIKKYTKDYNSALPNSKS--APIDSKKVIANRQEFCQVIRD           379
XP_019636962.1[B.belcheri]           ILLQLKQAVERKA---PNDELQKLSSEFFSAIPHRPDHKDTIKNKREIARKQDMCQLIRD           260
XP_014020296.2[S.salar]              LLLQVQRKLRARQGEGPDTEVASLMEEVYTLLPHKTDQSHLPLHTKLISSQLDLCQLIRD           385
XP_041440458.1[X.laevis]             ILLQVRKALDTGTEP---GDISKAMAEFYRLIPHKKPML-VNINKKLLSTKQDLCQLIRD           349
XP_046764289.1[G.gallus]             ILLQVKNALNEGANE---TALQEMMMEFYQLIRHKTEID-YEVTKRLLSRKQDLCQLMRD           368
EDL36193.1[M.musculus]               ILLLVKTALKNGDSP---GQLQKTMAEFYRLLPHRHPAS-EEVNLRLLAQKEDLCQLVRD           348
NP_006428.2[H.sapiens]               ILLLVKAALKNGETA---EQLQKMMTEFYRLIPHKGTMP-KEVNLGLLAKKADLCQLIRD           355
NP_001125927.1[P.abelii]             ILLLVKAALKNGETA---EQLQKMMAEFYRLIPHKGTTP-KEVNLGLLAKKADLCQLIRD           355
                                      *: ::  .           :     :    :               ::   ::**:::*

XP_047129290.1[H.vulgaris]           VVGVSESTN-----------------------WKTIPSLNAKYKALGCNIKELHYEAL--           390
XP_032828733.1[P.marinus]            VVDANEATRNKNTGGDDSSMRKENLALKKLTASSHSATTLAQFTALRCSLLHLEPNSSDF           433
XP_032219396.1[N.vectensis]          MVTISEATN-----------------------MSTVSSHLAKYKALRCHIEALHPEEAEY           415
XP_019851276.1[A.queenslandica]      LVSVSESTN-----------------------WSHKSGPVAKYHALRCQMCPIDPVSKEY           416
XP_019636962.1[B.belcheri]           MVSVSEATG-----------------------WTSHPSAVARYHALRCNIQSLARDSEDY           297
XP_014020296.2[S.salar]              VLNVSEATL-----------------------RSPIPSCLGKYRALRCSIERVPPGPELL           422
XP_041440458.1[X.laevis]             MVSVCETNL-----------------------SKPNPSILAKYQALRCQIEHVESDTEEF           386
XP_046764289.1[G.gallus]             MLNIRETSM-----------------------SCPNPSSLAKYQALRCKIDAVDPMSDEF           405
EDL36193.1[M.musculus]               MVNVCETNL-----------------------SKPNPPSLAKYRALRCKIEHVDQNTEEF           385
NP_006428.2[H.sapiens]               MVNVCETNL-----------------------SKPNPPSLAKYRALRCKIEHVEQNTEEF           392
NP_001125927.1[P.abelii]             MVNVCETNL-----------------------SKPSPPSLAKYQALRCKIEHVEQNTEEF           392
                                     ::   *:.                                .:: ** * :  :

XP_047129290.1[H.vulgaris]           GELKKTISMS-SMEAKEIEVLSAFELNRISEKGG----VDFVGPFFHSTSTENLVGILSR           445
XP_032828733.1[P.marinus]            NSIRNQLLQSSHGRLDGFKILKIFRVDRPAEIGQFRSEVGNTRSLFHAFAPRSLLGILSR           493
XP_032219396.1[N.vectensis]          KAIKELAIEE-QTGSNDIIVLNVFSLHRPVEDINYRHGLGRQKLLFHASRVRNFVGILSR           474
XP_019851276.1[A.queenslandica]      SDVKEHIMSSLTNDSNDPEILSIYSVQRPVEESQFSHSLGNRKLLFHSSQLQNFVGILSR           476
XP_019636962.1[B.belcheri]           RTLESMIHSSQENGTPPIKVESVFRVCRSMEETGFTSAIGNIQTKFHSSQAQNFVGILSR           357
XP_014020296.2[S.salar]              YVT------EL-LQDRQVQILQVLRVNRGVELQMFREDLGNVKPLLHSSSPSSFVGILSR           475
XP_041440458.1[X.laevis]             HQVSQQIRENK-HCEESIKILKIFRVGKLSEATGFQRNLGNVKSLLHASSPSHFVGILSR           445
```

```
XP_046764289.1[G.gallus]            LGVERQVLKNN-YNGCSVRILQVYRIGRVSETAEFLGSLGNVRSLFHASSVRNFMGILSR        464
EDL36193.1[M.musculus]              SRVRKEVLQNN-RSEQPVDILQIFRVGRVNEATEFLSKLGNVRLLFHGSPVRNILGILSR        444
NP_006428.2[H.sapiens]              LRVRKEVLQNH-HSKSPVDVLQIFRVGRVNETTEFLSKLGNVRPLLHGSPVQNIVGILCR        451
NP_001125927.1[P.abelii]            LRVRKEVLQNH-HSKSPVDVLQIFRVGRVNETTEFLSKLGNVRPLLHGSPVRNIVGILCR        451
                                             .         : .   : :  *      :.     :*.      ::***.*

XP_047129290.1[H.vulgaris]          GLLLPKVVVDQHGGERTDVGMLGNGIYFASAASTCIKYSKPSQT-SGSRFMLLCELNLGK        504
XP_032828733.1[P.marinus]           GLLLPKTVVGEFGVERTDIGMLGCGIYFSDSISTSVKYARRAAARDGGRVLAVCDVALGR        553
XP_032219396.1[N.vectensis]         GLLLPTIVVDDFGGQRSDAGMLGHGIYFADRASTSAKYSSPGM--LGSRFMLVNKVALGK        532
XP_019851276.1[A.queenslandica]     GLLLPKIVVDDFGGSRSDPGMLGSGIYFADSASLSVKFSGPGKKRHGTRLMLVNEVALGN        536
XP_019636962.1[B.belcheri]          GLMLPKVVVDDFGGTRTDAGMLGHGIYFSDA-STSAKYSSPSKT-RGRRYMVVSEVALGK        415
XP_014020296.2[S.salar]             GLLLPRVGVEHHGIERTDIGNLGGGIYFSDAMSTSLKYSKPSVT-DGSRLLLVCDVALGR        534
XP_041440458.1[X.laevis]            GLLLPKNVEDDLGLERTDIGNLGSGIYFSDSISTGVKYSEPSVT-NGARLLLVCDVALGN        504
XP_046764289.1[G.gallus]            GLLMPKVVVEDHGVERTDTGNLGSGIYFSDSVSASVKYSSPSEM-DGTRLLAVCDVALGS        523
EDL36193.1[M.musculus]              GLLLPKV-AEDRGVQRTDVGNLGSGIYFSDSLSTSIKYAHAGET-DGSRLLVVCDVALGK        502
NP_006428.2[H.sapiens]              GLLLPKV-VEDRGVQRTDVGNLGSGIYFSDSLSTSIKYSHPGET-DGTRLLLICDVALGK        509
NP_001125927.1[P.abelii]            GLLLPKV-VEDHGVQRTDVGNLGSGIYFSDSLSTSIKYSHPGET-DGTRLLLICDVALGK        509
                                    **::*     . *  *:* * ** ****:.  *   *::  .    * * : : .: **

XP_047129290.1[H.vulgaris]          TKTYRKFQKDLNEAPAGYDSTHGVKSTSNEPSDFDDDEFCVYDVSRQRITHIVEFKLASD        564
XP_032828733.1[P.marinus]           CHLVRRRDRSLAAPPQGHDSLHGARATPGTASEFEDDEFVIYDPRQQKLRYIVQFCLESD        613
XP_032219396.1[N.vectensis]         SKEYRKFTKDLTRPPDGYNSVIGVKATETQPSDFKDDEFVVYDTKQQQMRYLVEFALPED        592
XP_019851276.1[A.queenslandica]     VKEFTSYQKELLAAPNGFHSVKGVKGTTEEPSDFKENEYVVYKTEQQRMRYLIEFRLPQD        596
XP_019636962.1[B.belcheri]          CKDVYQQDTSLTQPPQGYNSVHGVKNTDDIPSEFKDNEYVIYNVQQQKMRYLVEFSLPGD        475
XP_014020296.2[S.salar]             CTDLQKRDSSLTRSPEGYHSVHGVRHTPKKPSEFDDDEYVVYSPEQVRLQYMVQFRLEED        594
XP_041440458.1[X.laevis]            CKDLYKRDFSLTEAPNGFDSVHGVRQVPGRKSDFEDDEFVVYSKDQVKMRYVVQFCTAKD        564
XP_046764289.1[G.gallus]            CLDLYERDFSLNAAPSGYNSVHGVRKTADVSSDFEDDEFVVYKTTQVKIRYVVKFCLAED        583
EDL36193.1[M.musculus]              CVNLFKKDFSLTEAPPGYDSVHGVSETTSVPTDFQDDEFVVYKTNQVKMKYIVKFCTPGD        562
NP_006428.2[H.sapiens]              CMDLHEKDFSLTEAPPGYDSVHGVSQTASVTTDFEDDEFVVYKTNQVKMKYIIKFSMPGD        569
NP_001125927.1[P.abelii]            CMDLHKKDFSLTEAPPGYDSVHGVSQTASVTTDFEDDEFVVYKTNQVKMKYIIKFSMPGD        569
                                             .*   * *..*  *.   .   ::*.::*:  :*.  : :: ::::*    *

XP_047129290.1[H.vulgaris]          NVHHTLNNDVSED------EGIESDFDSESELSIKDVDSVQDPLSKVVSGLQSSTNQ--P        616
XP_032828733.1[P.marinus]           VVKEEENSDAEEFYEAPETPAD-ER-IL-PEPNIPELLPEQGEPKQRLSGLHSGVGS--P        668
XP_032219396.1[N.vectensis]         DIIARDNVSVDSGFVEEPLDN-----EDEIEVDLSDVMSIPDPLLKVEAGLKGTADK--P        645
XP_019851276.1[A.queenslandica]     EEIDQDETESEK-YENEEIEEDEESNIVTDGVSLSDVKNVTDPLSKVEAGLKVD-GG--V        652
XP_019636962.1[B.belcheri]          QVTPVDEAAYLHL--LTPEVEIEEDQDLN-KISLEDVQNIADPLDKVKGGLISKSGEEMP        532
XP_014020296.2[S.salar]             QLKDFQPTIDTSS--TELPLPVSPSDLLSCGEAEEDVGSCKNPLEEVTAGLLDCSGQ--P        650
XP_041440458.1[X.laevis]            QVNMEHDKLPVWV--EPTEEDV-GEEPV---QAEDPMLENLPEPRSIKGGLQSVQGN--Q        616
XP_046764289.1[G.gallus]            QIKQFQPGTEVEI--EQDNTEPESQCHL---QNESYELPSANISDTVKTGLQDRLGN--P        636
EDL36193.1[M.musculus]              QIKEFHPHENTEV--EEQRAEPSS--VP---EAGDFQLPDIKPFTNIKAGLQDASAN--P        613
NP_006428.2[H.sapiens]              QIKDFHPSDHTEL--EEYRPEFSN--FS---KVEDYQLPDAKTSSSTKAGLQDASGN--L        620
NP_001125927.1[P.abelii]            QIKDFHPSDHTEL--EEYRPEFSN--FS---KVEDYQLPDAKTSSSTKAGLQDASGN--L        620
                                                                                    **

XP_047129290.1[H.vulgaris]          VPLTAVHIRAKLQEFSSEVVVLQAYRNDSINPIEAKYVFPLESSAAVCSFEAFINGKHIV        676
XP_032828733.1[P.marinus]           VPLKAVHVRANILDFTAQVVVMQEYDNNSTEPIEAKYTFPLDEMAAVCSFEAFINGKHIV        728
XP_032219396.1[N.vectensis]         VPLKAVHIRAKLLDLAAQVVVMQAYCNNSDVPIEAKYVFPLDDMAAVCGFEAFINGKHII        705
XP_019851276.1[A.queenslandica]     VPLKAVHVKAQLIDLAAKVIVLQSYKNESLVPIEAKYVFPLDDMAAVCGFEAFINGKHII        712
XP_019636962.1[B.belcheri]          VPLKSVHVRAKLLDLAAQVIVLQSYKNENNVAIEAKYVFPLDDMAAVVGFEAFINGKHVV        592
XP_014020296.2[S.salar]             LPLQAVHVRARLMDLLSQVIIFQTYTNQSTAPIEAKYVFPLDDSAAVCGFEAFINGKHVM        710
XP_041440458.1[X.laevis]            IPLENIHVKGRMMDLVVEVIIFQTYINKSSVPIEAKYVFPLDSTAAVCGFEAFINGKHVI        676
XP_046764289.1[G.gallus]            VPLESVHIKARIIDFVAQVVMFQTYTNQNSSPIEAKYVFPLDDKAAVCGFEAFVNGRHII        696
EDL36193.1[M.musculus]              VPLDSVHIKGRVIDFVAQVIVFQTYTNQSHVPIEAKYIFPLDDKAAVCGFEAFINGKHIV        673
NP_006428.2[H.sapiens]              VPLEDVHIKGRIIDTVAQVIVFQTYTNKSHVPIEAKYIFPLDDKAAVCGFEAFINGKHIV        680
NP_001125927.1[P.abelii]            VPLEDVHIKGRIIDTVAQVIVFQTYTNKSHVPIEAKYIFPLDDKAAVCGFEAFINGKHIV        680
                                    :**  :*::..: :   :*:::* * *..   ***** ***:. *** .****:**:*::

XP_047129290.1[H.vulgaris]          GKVKEKEQARKEYKEAIQQGHGAYLMEEQEESPDVFTVTVGNLPPNSDVLIKITYVAELS        736
XP_032828733.1[P.marinus]           GQVKEKETAHQQYRNAVSQGHGAYLMDQ--ESPDVFVVSVGNLPPACRVLIKVTYVCELA        786
XP_032219396.1[N.vectensis]         GEVKEKEQARKEYREAISAGHGAYLMDE--ETPDVFTVNVGNLPPAASVLIKITYVAELT        763
XP_019851276.1[A.queenslandica]     GEVKEKEQAHREYKQAISEGHGAYLMDE--ETPDVFTVSVGNLPPGASVLIKITYVAELQ        770
XP_019636962.1[B.belcheri]          GEVKEKEEAHREYRQAISEGHGAYLMDE--ETPDVFTVSVGNLPPRASVLIKITYVSELA        650
XP_014020296.2[S.salar]             GQVKEKQQARKEYKQAIERGHGAYLMDQ--DAPDVFTISVGNLPAGATVLIKVTFVTELV        768
XP_041440458.1[X.laevis]            GEVKEKKQAHQEYRAAIREGHGAYLMDQ--DADDIFTVSVGNLPPNATVIIKITYITELN        734
XP_046764289.1[G.gallus]            GQVKEKKQAHREYRKAISEGDGAYLMDQ--DAPDIFVITVGNICPNSTVLIKVTYITELS        754
EDL36193.1[M.musculus]              GEIKEKEEARQEYREAVSQGHGAYLMDQ--DTPDVFTVSVGNLPPRAKVLIKITYITELS        731
NP_006428.2[H.sapiens]              GEIKEKEEAQQEYLEAVTQGHGAYLMSQ--DAPDVFTVSVGNLPPKAKVLIKITYITELS        738
NP_001125927.1[P.abelii]            GEIKEKEEAQREYREAVTKGHGAYLMSQ--DAPDVFTVSVGNLPPKAKVLIKIAYITELS        738
                                    *::***: *:::*  *:  *.*****.:  :: *:*.:.***:   . *:**:::: **

XP_047129290.1[H.vulgaris]          LEDDQISFSLPGCVAPWVKDQALATKMQNELETINVNSF----GEFSLEISLEMPCAIRS        792
XP_032828733.1[P.marinus]           AEGAAVRFRLPGNLAPWQQRLATGDVTQETVETLAVAKH---SGRLSVQVSVEMPYEIIN        843
XP_032219396.1[N.vectensis]         VEGELICFRLPGSVAPWQRTQAHDDVTQTDVDTICVDEEN--QSNTSVQVSVEMPFEIRA        821
XP_019851276.1[A.queenslandica]     AEGENVVFSLPGSVAPWKQEAALDETTQVDVEKVKVRSD---ADSLSVQISIDMPFTIRT        827
XP_019636962.1[B.belcheri]          VEGENICFRLPGSVAPWQKDSL-SEEIQKDVETVKVGKD--AGKEFSLQVAMEMPFDIRT        707
XP_014020296.2[S.salar]             VMEGLINFSLPGSVAPWQQSSALNQRTQVTLEKVCVTNLDSQ-GEFSLDMSIEMPNEIIN        827
XP_041440458.1[X.laevis]            LEYETVDFSIPGTVASWQKDKALKENTQDTVEKICVEESGVSKGCFSLEMSVEMSFKIHS        794
XP_046764289.1[G.gallus]            CLNGCITFHMPASVSPWQQDKALNENTQDTIKKICVKQVETL-KKFSLDMSIEMPYSIES        813
EDL36193.1[M.musculus]              IQSPVAIFFIPGTVAPWQQDKALNENLQDTVETIRIKEIGAE-QSFSLAMSIEMPYMIEF        790
NP_006428.2[H.sapiens]              ILGTVGVFFMPATVAPWQQDKALNENLQDTVEKICIKEIGTK-QSFSLTMSIEMPYVIEF        797
NP_001125927.1[P.abelii]            ILGTVGVFFMPATVAPWQQDKALNENLQDTVEKICIKEIGTK-QSFSLTMSIEMPYVIEL        797
                                           * :*. :: * :        *  :..: : .        *: ::::*  *

XP_047129290.1[H.vulgaris]          IVSPTHGNILIKKTATKAVVKMND--P-LGTSFQLLIALAEIHV------PRMWVQKSPQ        843
XP_032828733.1[P.marinus]           VTSPTHA-IRVKRTACKAVVELASSDDSLGSEFQLLVFPAQIHE------PRMWVEQNPE        896
XP_032219396.1[N.vectensis]         ILCPGHD-VKVKRTSTKAVVEMAPGGTL-GSGFQLQIQLAEIHV------PRMWVERHDT        873
XP_019851276.1[A.queenslandica]     IESPAHK-IKMKKTDTKATVELCPGEKL-GTGFQLLIGLAEIHV------PRMWVEENDK        879
XP_019636962.1[B.belcheri]          IQSPSHK-IRVKRTASKAVVELEKNCML-GDGFLLQVGLAEIHV------PRMWVERHPD        759
XP_014020296.2[S.salar]             LHCLTHK-VKIKRTDCKAVVRTCPGETLGPDGFLVCFSLAQIHL------PRMWVEKHPD        880
XP_041440458.1[X.laevis]            IMSYTHN-ILMKKTDCKAVIATEKDSSFDDGGFRLIIYLEPTFLQSSWNQPRMWVEKHPD        853
XP_046764289.1[G.gallus]            IHSWTHK-LKIKKTECKAVIKTVENSSLDSSGFGLDIWISHAYL------PRMWVEKHPN        866
EDL36193.1[M.musculus]              ISSDTHE-LRQKSTDCKAVVSTVEGSSLDSGGFSLHIGLRDAYL------PRMWVEKHPE        843
NP_006428.2[H.sapiens]              IFSDTHE-LKQKRTDCKAVISTMEGSSLDSSGFSLHIGLSAAYL------PRMWVEKHPE        850
NP_001125927.1[P.abelii]            IFSDTHE-LKQKRTDCKAVISTMEGSSLDSSGFSLHIGLSAAYL------PRMWVEKHPE        850
                                    : .  *  :  * *  **.:            * : .     .      *****:.

XP_047129290.1[H.vulgaris]          DSDKQACMLSFFPEFDGDI------GDHEVIFLIDASNSMKDHFI-ELQKLVYLTYEKMP        896
XP_032828733.1[P.marinus]           -TDSQACILTFYPEFAPSHS-----EGVDIVLCLDCSASMTTQPLTDVKRLALFALRHLP        950
XP_032219396.1[N.vectensis]         EADSQACMLAFYPEFESGPP-----HHVEVIFVLDASCSMKGKALQEAKKLTLMCLSLME        928
XP_019851276.1[A.queenslandica]     --GHHACMLTFYPEFEAESI-----VENEIIFLLDVSNSMKGDALDNAKKVLLLLLHHLP        932
XP_019636962.1[B.belcheri]          -KDSQACMLTFYPEFQAEVM-----QGHEVILLLDASNSMRGSPLEAAKKVALLCLSHLP        813
XP_014020296.2[S.salar]             -KDSQACMLVFYPDFEPCPTSSPMSGSSEVILLLDSSESMRGDALQNARRIALQLVNTMD        939
XP_041440458.1[X.laevis]            -EDSEACMLVFLPDFETNIQ--S----WDLTILLDCSNSMES-TFQSAKQIALLAANFFN        905
XP_046764289.1[G.gallus]            -KNSEACMLVFQPEFEAAFD--EEQLSSEIIILLDCSNSMAGSALLQAKQIALHALKQFS        923
EDL36193.1[M.musculus]              -KESEACMLVFQPELADVLP--DLRGKNEVIICLDCSSSMEGVTFTQAKQVALYALSLLG        900
NP_006428.2[H.sapiens]              -KESEACMLVFQPDLDVDLP--DLASESEVIICLDCSSSMEGVTFLQAKQIALHALSLVG        907
NP_001125927.1[P.abelii]            -KESEACMLVFQPDLDVDLP--DLANESEVIICLDCSSSMEGVTFLQAKQIALHALSLVG        907
                                        .**:* * *::             :: : :* * **    :   :::      .

XP_047129290.1[H.vulgaris]          -QSTKFNMVIFNDTYKELFPYSRFKDES-KQQVMSLLKLLKNCNGGTHLWSVLKMIEMLS        954
XP_032828733.1[P.marinus]           -PGSRFNVLKFGTHYKELFPVFRESSPSNIATAVSFIKRLHADAGNTELWRPLRSVALLA        1009
XP_032219396.1[N.vectensis]         -EEWAFNIVVFGSNYSELFTQSQKKDKETVARAAKFVKSVKAVKGSTDLWRVLRSLYLLR        987
XP_019851276.1[A.queenslandica]     -PKTYFNVFTFGAMFESIFPSSVQINNETLSFATKQVQSCQAVMGNTDIWRPLHSLYLLS        991
```

```
XP_019636962.1[B.belcheri]           -KECSFNVVAFGTGYEELFAVSQPRIKSNVSKAENFIQDLKASKGNTDAWRPLHGFFLLP           872
XP_014020296.2[S.salar]              HHSVRVNVISFGSDQKDAFLSANPLVE-ALQPAKKFIMSSPPVGGSTELWRPLRSLSLLP           998
XP_041440458.1[X.laevis]             -SWLSINIISFGTSFKEFCVCPKEKNN-LIPELVQFIKMAKPNMGNTELWKPLQSLCLLA           963
XP_046764289.1[G.gallus]             -SRQNVNLIKFGTNFSEFSSFSKNTSK-DLASLTEFITSATATMGNTDLWKTLRYLSLLF           981
EDL36193.1[M.musculus]               -EEQKVNIMQFGTGYKELFSYPKCITD--SKMATEFIMSAAPSMGNTDFWKVLRYLSLLY           957
NP_006428.2[H.sapiens]               -EKQKVNIIQFGTGYKELFSYPKHITS--NTMAAEFIMSATPTMGNTDFWKTLRYLSLLY           964
NP_001125927.1[P.abelii]             -EKQKVNIIQFGTGYKELFSYPKHITN--NTAAAEFIMSATPTMGNTDFWKTLRYLSLLY           964
                                          .*:. *.   ..                 . :       *.*. *  *: . :*

XP_047129290.1[H.vulgaris]           CES-------------TMKNVFLFSDGHIFDEISTLARIRNLKKNVRLFTFGVGTTPNKH           1001
XP_032828733.1[P.marinus]            GGV----G-GSRDAAAPPVSVLLLTDGVAQSEARATREARENVRLLRVLACGVGETPNRY           1064
XP_032219396.1[N.vectensis]          CNS----------TADYPSNVFLFTDGHVTEESTTLAYIRDIRLRTRVFTFGVGPTCNRH           1037
XP_019851276.1[A.queenslandica]      EAASSSTGGLTNSSTLPPCSVFVISDGHMTEEAPSLSAIKDGAQNYRVFTFGVSSTANRH           1051
XP_019636962.1[B.belcheri]           PSP------------GKTRNVFLISDGHVNNADQTLSDIHAHYLETRVFSCGVGSTSNKH           920
XP_014020296.2[S.salar]              PSQ------------GV-RNLLLLSDGHVQNQALTLQLVRQHAQHSRLFTCGLSPTANRH           1045
XP_041440458.1[X.laevis]             PSS------------GTMQNVLLISDGHIQNENRVFQILKKNAGKVRLFTCGVGATANRH           1011
XP_046764289.1[G.gallus]             PSQ------------GH-RNILLISDGHIQNESVTFQLVKDNVHHTRLFTCGVGSTANRH           1028
EDL36193.1[M.musculus]               PSE------------GF-RNILLISDGHLQSESLTLQLVKRNIQHTRVFTCAVGSTANRH           1004
NP_006428.2[H.sapiens]               PAR------------GS-RNILLVSDGHLQDESLTLQLVKRSRPHTRLFACGIGSTANRH           1011
NP_001125927.1[P.abelii]             PAR------------GS-RNILLVSDGHLQDESLTLQLVKRSRPHTRLFACGIGSTANRH           1011
                                                        .:::.:**   .        :      *::: .:. * *::

XP_047129290.1[H.vulgaris]           ILQKMACLGSGYFEYFDKNAKSKWQKKVELQLEKCQQPVLTNIKIHWIQHDEDA----PK           1057
XP_032828733.1[P.marinus]            FLNALARNSGGACEFFTAKNRSTWTDKMLALMEKATQPVCRDVTVEWQRFDRGALGGDRV           1124
XP_032219396.1[N.vectensis]          FLRSMARVGAGAYELFDSKVKSKWERKVESQLSKARQPVLTSVKVAWQQHDSYA----KP           1093
XP_019851276.1[A.queenslandica]      FLRSMARVGGGCSEFFDSQKKSRWERKVKEQLSKAFQPALTGVDVQWQQHDENA----PP           1107
XP_019636962.1[B.belcheri]           LLRAIARVGGGAFEYFDDKTKSKWERKVKSQLWKAAQPGLTSVSVDWQQWDDDA----PP           976
XP_014020296.2[S.salar]              MLRALAQAGGGAYEFFDTKTKHTWREKVACQVKRIMSPGCSSVSVKWQQFNPSA----PP           1101
XP_041440458.1[X.laevis]             MLRCLAQYGAGFFEFFEDKSKSNWEKKMEAQLEKMNSPACTSASVKWVQFSEND----PE           1067
XP_046764289.1[G.gallus]             MLRSLSQYGAGAFEYFDSKSKYNWEAKIQNQVSRIFSPGCSSVSIKWQQFDINA----PK           1084
EDL36193.1[M.musculus]               ILRTLSQCGAGVFEYFNSKSKHSWKKQIEAQMTRIRSPSCHSVSVKWQQLSRDA----PE           1060
NP_006428.2[H.sapiens]               VLRILSQCGAGVFEYFNAKSKHSWRKQIEDQMTRLCSPSCHSVSVKWQQLNPDV----PE           1067
NP_001125927.1[P.abelii]             ILRILSQCGAGVFEYFNAKSKHSWRKQIEDQMTRLCSPSCHSVSVKWQQLNPDG----PE           1067
                                     .*. ::  ..*  * *  : :  *  ::   : :  .*   .  : * : .

XP_047129290.1[H.vulgaris]           PLQAPNEITTLFNGNRLVVYGFVPHCTMANLSAILNGKELNTIVSTSELSTTVGLTIHRL           1117
XP_032828733.1[P.marinus]            EMQAPRMIRSVFCGTRLVVYGIVPHCTQATLHAIINNQEFSTMVSTSNLQITRGKMLHRL           1184
XP_032219396.1[N.vectensis]          PVQAPHDIVSLFNGSRQVVYGLVDNCTQASLTAEVDGRVVSTMVSTSELSITTGKVLHQL           1153
XP_019851276.1[A.queenslandica]      PIQAPKSITSLFSGSRQVVYGFVPHCKQASLKAFIGRKEIQTMVSTSDLATTSGQTLHQL           1167
XP_019636962.1[B.belcheri]           PVQAPNQIVSLFNGSRQVVYGYVPHCTQATLKAVINNREISTMVSTSELSITAGKILHQL           1036
XP_014020296.2[S.salar]              PVQAPSQLHALFTDCHTLVYGFVPHCTQATLLGDLSGQNIETMVSTTELQKTRGTFLHKL           1161
XP_041440458.1[X.laevis]             PVQAPAHIPALFKNSCLLVYGFAPRCTQAKLQALIDDKELDTMASTTELQKTTGTMLHKL           1127
XP_046764289.1[G.gallus]             PMQAPAQIQSLFTNERLLVYGFVPHCTQATLRAIINDQELQTVVSTTELQKTTGTVLHKL           1144
EDL36193.1[M.musculus]               PLQAPAWVPSLFHNDRLLVYGFIPHCTQATLQAFIQEKEFCTMVSTTELQKTTGTMIHKL           1120
NP_006428.2[H.sapiens]               ALQAPAQVPSLFLNDRLLVYGFIPHCTQATLCALIQEKEFRTMVSTTELQKTTGTMIHKL           1127
NP_001125927.1[P.abelii]             ALQAPAQVPSLFRNDRLLVYGFIPHCTQATLCALIQEKEFCTMVSTTELQKTTGTMIHKL           1127
                                      :***  : ::* .   :***   .*. *.* . :  : . *:.**::*  * *  :*:*

XP_047129290.1[H.vulgaris]           AAKSVINDWNDGMLHQDQYKHEAIKAEMKGKIIQLSIEYSIVTKFTSFIAVEEREKNEG-           1176
XP_032828733.1[P.marinus]            TAHAIIQDYEDGNLHPDETQHEVIIAEMKSRIIELSKEYSIITPYTSFVAVEERDSTLA-           1243
XP_032219396.1[N.vectensis]          TARAIIRDWEEGSLDPNHTKHEMAKRDMKDFIISLSKRFSIVSQFTSFVAIEHRKKDEKF           1213
XP_019851276.1[A.queenslandica]      TAQALIRDWSEGSLNDDRTEHEIMKRDLKTFIINTSKEYSIVSQFTSFVAIEKREKDEKF           1227
XP_019636962.1[B.belcheri]           TARAVIRDWEDGTLHVDRTHHETKKMGLKPYIISLSKEYSIVTQFTSFVAVEKREQGEDL           1096
XP_014020296.2[S.salar]              TARAVIRDYEDGSLHTNEAEHEGKKSVMKSFIIELSKEFSILSQFTSFVAIEERDSE-Q-           1219
XP_041440458.1[X.laevis]             TARAVIRDYEDGFLHEKEHEHEMKKQQTKSLIIELSKKYSIVTQFTSFVAVEKRDAQEK-           1186
XP_046764289.1[G.gallus]             TARAFIRDYEDGILHENETEHEMKKQILKNMIIHLSMENSIITQFTSFIAIEKRDANEI-           1203
EDL36193.1[M.musculus]               AARALIRDYEDGILHDDETNHEMKKNIMKSLIIELSKENSLITQFTSFVAVEKRDVNEI-           1179
NP_006428.2[H.sapiens]               AARALIRDYEDGILHENETSHEMKKQTLKSLIIKLSKENSLITQFTSFVAVEKRDENES-           1186
NP_001125927.1[P.abelii]             AARALIRDYEDGILHENETSHEMKKQTLKSLIIKLSKENSLITQFTSFVAVEKRDENES-           1186
                                     :*::.*.*:.:* *. ..   **     *  **  * . *::: :***:*:*.*.

XP_047129290.1[H.vulgaris]           KDVTAPTINELVLMETVDILPYMGFETKTKSVPMDKAK---------FSLSEILD-----           1222
XP_032828733.1[P.marinus]            DEATGPALDELLSKESVDFLPYVGWVEEEKARKKKGSMQF------------HLR-----           1286
XP_032219396.1[N.vectensis]          DASLGPSIDELVSHEDVDVIPYVSWEEPESRDVGKPESSSKSTGDRVAGLVSEAR-----           1268
XP_019851276.1[A.queenslandica]      DATKGPSIEDLVSKENVDKLKYMGWEIDQGADPVKIAA--ESIDDVIASMAGETD-----           1280
XP_019636962.1[B.belcheri]           TTGDGPTIKELVQKESVDILSYMGWQQDEGQLDSTELF------EVKVGFLGLSDGKRDP           1150
XP_014020296.2[S.salar]              PNVGFTDVPKLISDEDVDFLPYLSWLEEEYEGDGMDLG------SN--SMSNLLDGGPGR           1271
XP_041440458.1[X.laevis]             PKDYVPNVLEIISAEVVDILPYMTWETEKNKKIGV------------------D-----           1222
XP_046764289.1[G.gallus]             CSANVPNILEIIAQEDVDFLPYMNWEQKPMGSGTLFS-----------------------           1240
EDL36193.1[M.musculus]               PFANVPNISELVAKEDVDFLPYVSWQEKQPEASISQT---------------EID-----           1219
NP_006428.2[H.sapiens]               PFPDIPKVSELIAKEDVDFLPYMSWQGEPQEAVRNQS---------------LLA-----           1226
NP_001125927.1[P.abelii]             PFPDIPKVSELIAKEDVDFLPYMSWQGEPQGAIRNQS---------------LLA-----           1226
                                            : .::  * ** : *: :

XP_047129290.1[H.vulgaris]           ------------------------------------------------------------           1222
XP_032828733.1[P.marinus]            -----------------------------------------------------------A           1287
XP_032219396.1[N.vectensis]          VLERFSV-------LQAETLYKRACDTA---LSAESPDNRATYKAFLSLAQFYVDVHGDL           1318
XP_019851276.1[A.queenslandica]      VI-------------KLDTAFNKVWESTQNTLPNSHPARV-------KLLKMRLEVLKKM           1320
XP_019636962.1[B.belcheri]           EIVRVQLTDDSLVLQREETVYGNSLAAISSQVNNFDIDSNR----------ER-SVILRR           1199
XP_014020296.2[S.salar]              VLCRNICM--------EEM-----------------------------------------           1282
XP_041440458.1[X.laevis]             ------------------------------------------------------------           1222
XP_046764289.1[G.gallus]             ------------------------------------------------------------           1240
EDL36193.1[M.musculus]               ------------------------------------------------------------           1219
NP_006428.2[H.sapiens]               ------------------------------------------------------------           1226
NP_001125927.1[P.abelii]             ------------------------------------------------------------           1226


XP_047129290.1[H.vulgaris]           ---------------GMHML----------------------------------------           1227
XP_032828733.1[P.marinus]            EKTKA----PVGGRGGLASS------K---------------------------------           1304
XP_032219396.1[N.vectensis]          QKAEALTMMLHGALGGIEHD------------VSSRSNFDDLLEHLRQDSSYS-DEVGRL           1365
XP_019851276.1[A.queenslandica]      DKLSDGQELLEGLITDLHLRGSIDSTRLINVLEGIRDGFSRMIDAKRKAAEAEAARRSRM           1380
XP_019636962.1[B.belcheri]           QKSGG---LGLSIMGGAEHN------------------IPVLISSIVKDQAA--DQTGQL           1236
XP_014020296.2[S.salar]              ------------VMG-EEH-----------------------------------------           1288
XP_041440458.1[X.laevis]             ------------------------------------------------------------           1222
XP_046764289.1[G.gallus]             ------------------------------------------------------------           1240
EDL36193.1[M.musculus]               ------------------------------------------------------------           1219
NP_006428.2[H.sapiens]               ------------------------------------------------------------           1226
NP_001125927.1[P.abelii]             ------------------------------------------------------------           1226


XP_047129290.1[H.vulgaris]           --K-----D-----------ELS-------------------------------------           1232
XP_032828733.1[P.marinus]            ------IKY--------------------------------RKTRP--------------           1312
XP_032219396.1[N.vectensis]          VAE-----FN-------PMKDINKSK------R----KVIASPFP--NVDYPWSQTVVN-           1400
XP_019851276.1[A.queenslandica]      MIV-----AE-------EAKSLRQSTLETERDG----GIIGEAKMELKSRIAFKQKEIES           1424
XP_019636962.1[B.belcheri]           FVGDAIIKVNGVNVEHSTHDDVVQHLKNSPDDVKLTVRYYRAATPYLRAPAKWSRYSSE-           1295
XP_014020296.2[S.salar]              -------------------ESLVQLQVD--------------------------------           1297
XP_041440458.1[X.laevis]             ------------------------------------------------------------           1222
XP_046764289.1[G.gallus]             ------------------------------------------------------------           1240
EDL36193.1[M.musculus]               ------------------------------------------------------------           1219
NP_006428.2[H.sapiens]               ------------------------------------------------------------           1226
NP_001125927.1[P.abelii]             ------------------------------------------------------------           1226


XP_047129290.1[H.vulgaris]           -----------------------------LS---------EDDYD---------------           1239
```

```
XP_032828733.1[P.marinus]            -------ACFS-------------------------------------------------      1316
XP_032219396.1[N.vectensis]          ---KLFEACRSESSTETGSTASSTSSSESSSEEELSDNSEDSDWGSD-------------      1444
XP_019851276.1[A.queenslandica]      LESKLHLITRR-----RGKALKKD---IPPAEER--KASPKVPLKKK-------------      1461
XP_019636962.1[B.belcheri]           -------QCN-------GNAS--------PADN---ETTSSSEWKAEVTDYEDMSCEMVT      1330
XP_014020296.2[S.salar]              ----------------------------------------DESWEGL-------NYSYLM      1310
XP_041440458.1[X.laevis]             ----------------------------------------FPGWKEN-------FCGCY-      1234
XP_046764289.1[G.gallus]             ----------------------------------------GEPYG---------------      1245
EDL36193.1[M.musculus]               ----------------------------------------SSRLK---------------      1224
NP_006428.2[H.sapiens]               ----------------------------------------SSEWPEL-------RLSKRK      1239
NP_001125927.1[P.abelii]             ----------------------------------------SSEWQKL-------YSSKPK      1239


XP_047129290.1[H.vulgaris]           ------------------------NDDSSLSRQES-------------------EEEKKL      1256
XP_032828733.1[P.marinus]            -----------------------------LIPEKTEQTPEIEFGSAAER----------A      1337
XP_032219396.1[N.vectensis]          ------------EFYEDNIDDEV-YQDAVGEEEEEEGEDDVY----GMCLEEAEEVERSE      1487
XP_019851276.1[A.queenslandica]      ------------KAVAK-------NRGPIREQESPEKDEDISVAEEGRRSIRMEEEPEEE      1502
XP_019636962.1[B.belcheri]           EIMTLGYSEGVQKMAGKILD------SSVELEEESESDEDMEFGLFDQEGW-MAADEEKE      1383
XP_014020296.2[S.salar]              GSMVFGSPVTVSAA-TKPPDMTLKSEG-AVTDEEMECFNDMYFGLQE-SEH---------      1358
XP_041440458.1[X.laevis]             -----GGPVSVD--------------ERASLDEEEE-----S---LP-------------      1254
XP_046764289.1[G.gallus]             -----IPPFDAVLQ-----DSDAQSDCFTTKRCETE-----GLQLQPEDLT---------      1281
EDL36193.1[M.musculus]               H---NKL---------------------------SD-----GHGVLQLSHS---------      1240
NP_006428.2[H.sapiens]               H---RKIPFSKRKM-----EL---SQPEVSEDFEED-----GLGVLPAFTS---------      1274
NP_001125927.1[P.abelii]             L---R----AKRKK-----KL---SQPEVSEDFEED-----GLGVLPAFTS---------      1270


XP_047129290.1[H.vulgaris]           YKKPIILDIGMAETKAGFAGCRAPKVVIPTLVGRPRHQGVMVGMGQKDAYVGNEALSKRG      1316
XP_032828733.1[P.marinus]            NLSPFEIEAAAD----------TASQYPSEIQPKFAHAS---------------------      1366
XP_032219396.1[N.vectensis]          APTPLVIDVGSHMWKVGFAGDDAPKGVFPPIVGRPRHQGVMVGMGQKDSYVGWEAHSKRG      1547
XP_019851276.1[A.queenslandica]      DIPVAVIDTGMDTVKAGFAGDDGPRSVFPALVGRPRHQGVMVGMGQKDSYVGDEAKSKRG      1562
XP_019636962.1[B.belcheri]           ILAKPEM----E--KKKVALLEKCSRLASPVMSRS-------AVATSTAYSPTSPTKLVA      1430
XP_014020296.2[S.salar]              PLRGSVI-----------MHSEALTSFYSPTLHTI-------TGSQSTPVTPYLATKMLA      1400
XP_041440458.1[X.laevis]             ------------------------FSFAPPPPPDE-------EEE---------------      1268
XP_046764289.1[G.gallus]             V--------------------------TK--------------ESISQ------------      1289
EDL36193.1[M.musculus]               PINLSEI-----------PTI-H-------------------------------------      1251
NP_006428.2[H.sapiens]               NLERGGV-----------EKL-LDLSWTESCKPTA-------TEPLFKKVSPWETST---      1312
NP_001125927.1[P.abelii]             NLERGGV-----------EKL-SDLSWTESCKPTA-------TEPLFKKVSPWETST---      1308


XP_047129290.1[H.vulgaris]           I--LTLLSPFKLDKGSETD--STKPLQLI-------------------GDTG--NIVKPE      1351
XP_032828733.1[P.marinus]            --VEAVP-----------CSNEGPLNLSRRDV-------KDHGV--SASR----------      1394
XP_032219396.1[N.vectensis]          I--LSLSTPQKREARPTPPLQDRPPPPLQDRPP-----------PAMEGRA----LTKDR      1590
XP_019851276.1[A.queenslandica]      I--LTLKSPFERPSKPMAAMSYSQPIVQVEEKE-------KESSATVEEDVLNDLFGYDG      1613
XP_019636962.1[B.belcheri]           YSVYSKPSPQKMALKRAE-KELEAPRPETDEVTVDVTLKASLDGIQARGDRLEDLIEKSD      1489
XP_014020296.2[S.salar]              MTVETD------------------------------------------------------      1406
XP_041440458.1[X.laevis]             ----SL------------------------------------------------------      1270
XP_046764289.1[G.gallus]             ------------------------------------------------------------      1289
EDL36193.1[M.musculus]               ------------------------------------------------------------      1251
NP_006428.2[H.sapiens]               ----SS------------------------------------------------------      1314
NP_001125927.1[P.abelii]             ----SS------------------------------------------------------      1310


XP_047129290.1[H.vulgaris]           TSKAINPKFKIAKCKRE---LSKGLST-LQHSSVQQSI---------------------N      1386
XP_032828733.1[P.marinus]            -ITTMS-----ASSRFGERHLLHRAAP------------V--------------------      1416
XP_032219396.1[N.vectensis]          VIWPIE-EAPLDHTASI------DLEE-------QEDL-------------YVDMDKGEV      1623
XP_019851276.1[A.queenslandica]      RNYEIS-DFSMAENYQVNEVLMDELEM-------DESL----------------MEQSLM      1649
XP_019636962.1[B.belcheri]           TLSAEAPVFFQASTAISEELFAATAEPTVHEATLVESKPAVLRKSGRA--PPKGARFVRE      1547
XP_014020296.2[S.salar]              --------------------FIPELSETI------QSLPEIVRHAKSRQGDFRRADRHRK      1440
XP_041440458.1[X.laevis]             --------------------IYN--SPAV-----A-------------------------      1278
XP_046764289.1[G.gallus]             -----------------------------------EVIPQDYGFHDDD------------      1302
EDL36193.1[M.musculus]               -----------------------------------DSEPPLLGFKD--------------      1262
NP_006428.2[H.sapiens]               --------------------FFPILAPAV-----GSYLPPTARAHS--------------      1335
NP_001125927.1[P.abelii]             --------------------LFGVSPPVV-----GSYLRPTAHAPR--------------      1331


XP_047129290.1[H.vulgaris]           IDSDSV----DYQK------------------------S--YIMLTKQKECAEIEQTSD-      1415
XP_032828733.1[P.marinus]            -----------------------------------------ILKEAFECRRS--------      1427
XP_032219396.1[N.vectensis]          LDDDGLFEAMCNNAQKAA-----------------------SL--AMGKRRQDVDQLT--      1656
XP_019851276.1[A.queenslandica]      VESAELYEELEQQSEETIAVNQKELRSRKVKKSIPGTSPRTSLLKGLGSKEPQEGQLGTG      1709
XP_019636962.1[B.belcheri]           VEPEPVSEEVAQSAPSAAS---------RSAPAFGFIPPGAS----FGKKSA--------      1586
XP_014020296.2[S.salar]              VCADPAFDTVETQK--KSL---------SVA-DVPRPPPSVL----QLKSCA--------      1476
XP_041440458.1[X.laevis]             ------------------F---------SFAPPPP--PPPL----------S--------      1291
XP_046764289.1[G.gallus]             -------------I--ATV---------KFAAAMPFVPCKPF----VMSYCN--------      1326
EDL36193.1[M.musculus]               -------------------------------------LCSRD----MGFSCG--------      1273
NP_006428.2[H.sapiens]               -------------------------------------PASLS----FASYRQ--------      1346
NP_001125927.1[P.abelii]             -------------------------------------AASMP----FASYCQ--------      1342


XP_047129290.1[H.vulgaris]           --DFLSMQ--------------------SDNL----------------------------      1425
XP_032828733.1[P.marinus]            -----------------------QQGFGSSAYAPRSV-------VHQLNSAET-------      1450
XP_032219396.1[N.vectensis]          L--------DSYD---------------D--I--HEVLPSNTQVAATLDT-----DD---      1681
XP_019851276.1[A.queenslandica]      LAGFASKPPEGYL---------------SLDI-EGLAMPANKETYSPLSQTYNPVSP---      1750
XP_019636962.1[B.belcheri]           --GLVGGPPGTTAEETKTKEVAFGQGFGSDAVSQGAGFGATPFGAPPLDATPFGAPPFGA      1644
XP_014020296.2[S.salar]              VADALLPPPAS--------------VVGS------------PGPPPPPASVLSSPG----      1506
XP_041440458.1[X.laevis]             VAKT----------------------FDS------------PTPAVPFSFAP--PP----      1311
XP_046764289.1[G.gallus]             TAPL----------------------FGS------------AVGAAYMTQKN--SF----      1346
EDL36193.1[M.musculus]               TA-F----------------------SGS------------FASSKDFDPGKFSQG----      1294
NP_006428.2[H.sapiens]               VA-S----------------------FGS------------AAPPRQFDASQFSQG----      1367
NP_001125927.1[P.abelii]             AA-S----------------------FGS------------AAPPRQFDAPQFSQS----      1363
                                                                 .

XP_047129290.1[H.vulgaris]           -----------------------------------------------MMDYCEMLASKES      1438
XP_032828733.1[P.marinus]            --------------------------------------PVP---------LIRAQAFAP-      1462
XP_032219396.1[N.vectensis]          ------MYVDMKN------RATSSALKE----RAVSYDI--------QDSLCEV------      1711
XP_019851276.1[A.queenslandica]      ------VYSPT--------SVTSPAYSP----TSPSYSP----SLPSYVPTCTSPAYNAP      1788
XP_019636962.1[B.belcheri]           PQFGAPQFGAAKTKTQEAQRFGSDAVSQGAGFGATPFGAPPLGATPFGAPLFGATPFGAP      1704
XP_014020296.2[S.salar]              --------------------------------------PPPS------------------      1510
XP_041440458.1[X.laevis]             --------------------------------------PPP-------------------      1314
XP_046764289.1[G.gallus]             --------------------------------------PTP-------------------      1349
EDL36193.1[M.musculus]               --------------------------------------PNN-------------------      1297
NP_006428.2[H.sapiens]               --------------------------------------PVP-------------------      1370
NP_001125927.1[P.abelii]             --------------------------------------PAP-------------------      1366


XP_047129290.1[H.vulgaris]           ---------------IYLESDSVK--------------------------------DIHQ      1451
XP_032828733.1[P.marinus]            ------------------------------------------------------------      1462
XP_032219396.1[N.vectensis]          ------------------AEDAVK------------------------------------      1717
XP_019851276.1[A.queenslandica]      TVNGKLSAPSVPGLTFNRSSDKVS------------------------------------      1812
XP_019636962.1[B.belcheri]           QFGAAKIKTQEAAFGQGFSSNAVSRGADVSTTPFGATSFGATPSIATPFGGTPFASTISL      1764
XP_014020296.2[S.salar]              --------------------------------------------------------RLQS      1514
XP_041440458.1[X.laevis]             ------------------------------------------------------------      1314
XP_046764289.1[G.gallus]             ------------------------------------------------------------      1349
EDL36193.1[M.musculus]               ------------------------------------------------------------      1297
NP_006428.2[H.sapiens]               ------------------------------------------------------------      1370
NP_001125927.1[P.abelii]             ------------------------------------------------------------      1366
```

```
XP_047129290.1[H.vulgaris]          FKL------------REKKSATPPPSST-RNV--SSLRKKQPFPEPMVS-----------          1485
XP_032828733.1[P.marinus]           ------------L--TIFDNV-QPPG---VSLGVIAMETP------------------SR          1486
XP_032219396.1[N.vectensis]         -----EEEAERPIAIFGLDSCIARVS--------SS-EEKSSLYDEI--PSPPNRPFKKK          1761
XP_019851276.1[A.queenslandica]     -----SDSSETSRNISQLSSAYLGSGKS-RRFESVSLEKSAPLSQPT--AAPPSRSRIVR          1864
XP_019636962.1[B.belcheri]          FGHSTTAAKEPEAALFGFASSATPSGGGGFSFGSTS--SDIPLKDEDKSSVPIDSSKGLK          1822
XP_014020296.2[S.salar]             FSAAVSDALPPPPPAFVLSSPGPPPSRL-QSFSATV------------------------          1549
XP_041440458.1[X.laevis]            ---------------FSFAPPPPPPPLA-L------------------------------          1328
XP_046764289.1[G.gallus]            ---------------AL-ASSGAHLRLC-G------------------------------          1362
EDL36193.1[M.musculus]              ---------------ISFSPKAPEMGVL-H------------------------------          1311
NP_006428.2[H.sapiens]              ---------------GTCADWIPQSASC-P------------------------------          1384
NP_001125927.1[P.abelii]            ---------------GSCADWIPQSASC-P------------------------------          1380

XP_047129290.1[H.vulgaris]          ---------------------------------KV------CLQAKMP------------          1494
XP_032828733.1[P.marinus]           FTPPYQDTFERGQARRQS------------------------------------------          1504
XP_032219396.1[N.vectensis]         -------------------VA---NI---------------------PSPKPRPPRASKI          1778
XP_019851276.1[A.queenslandica]     --------FEAGALTFGSSSQAPLEP---------------------VPPPPLPPRAVMT          1895
XP_019636962.1[B.belcheri]          FGAPLEEKGGTTGFRFGGSSQAKMIPQQGGFISHPQMQQQQQAQSNVPDRARIPGRKAQS          1882
XP_014020296.2[S.salar]             ------------------------------------------------------------          1549
XP_041440458.1[X.laevis]            ------------------------------------------------------------          1328
XP_046764289.1[G.gallus]            ------------------------------------------------------------          1362
EDL36193.1[M.musculus]              ------------------------------------------------------------          1311
NP_006428.2[H.sapiens]              ------------------------------------------------------------          1384
NP_001125927.1[P.abelii]            ------------------------------------------------------------          1380

XP_047129290.1[H.vulgaris]          -----------------------------------------------DLPMPPPPQLMPL          1507
XP_032828733.1[P.marinus]           ----------------------------------------------DRFT------NSAY          1512
XP_032219396.1[N.vectensis]         --AA-----------------------------------------------PPAPSLQAV          1789
XP_019851276.1[A.queenslandica]     --KSAPSEPIYPSFDGIPAFSVRNAISSSPEGSPPELTPWSS----PSFKSPPLPRSAPL          1949
XP_019636962.1[B.belcheri]          ARKTTGGKALRPAADKVASDGVK-------ADSSPDTVVFMDESDKAKETKTPTSAKSPL          1935
XP_014020296.2[S.salar]             -----------------------------------------------SDALPPTPPA---          1559
XP_041440458.1[X.laevis]            --------------------------------------------------------G---          1329
XP_046764289.1[G.gallus]            ------------------------------------------------------------          1362
EDL36193.1[M.musculus]              --------------------------------------------------------Q---          1312
NP_006428.2[H.sapiens]              --------------------------------------------------------T---          1385
NP_001125927.1[P.abelii]            --------------------------------------------------------T---          1381

XP_047129290.1[H.vulgaris]          FGAKQPSAEPMLLKACSPAKMPDLPMP--PPPQ-----LMPLFGAKQPSAEPM---LLK-          1556
XP_032828733.1[P.marinus]           PF--KP----QKCL-----ARVKKPPP--P-------LLC--FTARSPSSAALMPDVHEL          1550
XP_032219396.1[N.vectensis]         CI--DLPEPPLKAGDSPLKRSVKAPLP-PPPPQQA---SRIDYRAS--------------          1829
XP_019851276.1[A.queenslandica]     PG--GPPAKPSRSSGRALMQSPGGPPPPPPPPGTG---APMQLSEKS-PPPPPVP--LRS          2001
XP_019636962.1[B.belcheri]          KM--IPATCCMVCYDMPPLYQQGSPPP--PAPSRGPPPLPPTSQQEPPPPPPP-------          1984
XP_014020296.2[S.salar]             ----------------SVLSSPGPPPS--R-----LQSFSATVSDALPPPPPA-------          1589
XP_041440458.1[X.laevis]            ----------------MPFSFAPPPPP--P-----PLALGMPFSFAPPPPPPP-------          1359
XP_046764289.1[G.gallus]            ----------------SPVYSPPS------------PLVGMKYDNSFV------------          1382
EDL36193.1[M.musculus]              ----------------SPFCSPPKPPS--A-----PPLVTNVLCSE--------------          1335
NP_006428.2[H.sapiens]              ----------------G---PPQNPPS--S------PYCGIVFSGSSL------------          1406
NP_001125927.1[P.abelii]            ----------------G---PPQNLSF--A------PFCGIAFSGSSL------------          1402

XP_047129290.1[H.vulgaris]          ------ACSPGKTSFFSQPPPPPPAQSMYLFGAKQPLPEPTLSKD--CLPGKTSFFSQPP          1608
XP_032828733.1[P.marinus]           RAAGLM-----------------SRVPECAK---RDVCALQLP--NRDSPPTD---FPEP          1585
XP_032219396.1[N.vectensis]         ---------------YGAAPPAPSLQAVCID---LPEPPLK----AGDSPLKRSVKKPLP          1867
XP_019851276.1[A.queenslandica]     RDRGPMPVPQSTVLSSGRPPPPPPHFMLCGE---VPMPS-------G----------GPP          2041
XP_019636962.1[B.belcheri]          -------CPAGPPLPAGAPPPPPPQFRKSLL---LALSQEKLKGKSGQSPTEMI--QPPP          2032
XP_014020296.2[S.salar]             -------FVLS------SPGPPPSRLQ-SFS-----------------ATVSDA--LPP-          1615
XP_041440458.1[X.laevis]            -------LPAA------KSH-STDEVY-SLD---VALL--------GAQQVSVC--APPP          1391
XP_046764289.1[G.gallus]            ----------------------------------------------------DH--IP--          1386
EDL36193.1[M.musculus]              ------------------------------------------------------------          1335
NP_006428.2[H.sapiens]              ----------------------------------------------------SS--AQSA          1412
NP_001125927.1[P.abelii]            ----------------------------------------------------SP--TQLP          1408

XP_047129290.1[H.vulgaris]          PPPPAQ-SM----YLFGA---------KQPLPEPTLS---------------------KD          1633
XP_032828733.1[P.marinus]           PRLPPP--------------PPAQHASR--------FGQP--------------MHD---          1606
XP_032219396.1[N.vectensis]         PPPPQQAARIDYRASYGAPPLPPKRGGGPPLPPKRGGGPPLFGAMALR--TGAPSKASKP          1925
XP_019851276.1[A.queenslandica]     PPLPYLSRGIAQRRLPGGPPPP------PPPPPPGAAGAPLPGSSKVA--PKGKSKV--S          2091
XP_019636962.1[B.belcheri]          SRAPPP-------SMGGRPPPPPSMGRRPPPPMMTRAPPP----------NMARLRVDAD          2075
XP_014020296.2[S.salar]             --TPPA-------SVLSSPGPPPSRLQSFSDAVSDSLPPPSALVSNEFRAIKHKMRG--H          1664
XP_041440458.1[X.laevis]            PPPPPG-------SICA--PPPPGFICAP-----------------SPPAFDELLQV---          1422
XP_046764289.1[G.gallus]            -----N-------TIMF--GSCASTYSSD-----------------KTNAEKQV------          1409
EDL36193.1[M.musculus]              --------------------APQSYFLNL-----------------QSAAVHQS------          1352
NP_006428.2[H.sapiens]              PLQHPG-------GFTT--RPSAGTFPEL-----------------DSPQLHFSLPTDPD          1446
NP_001125927.1[P.abelii]            PPQFPG-------GCTT--RPSAGTSPEL-----------------DSPQLLFSLPTDPD          1442

XP_047129290.1[H.vulgaris]          CLPGLMGKKLAAPPPAAQSVSLFGSN-----KSSLMFSRNSCFS----------------          1672
XP_032828733.1[P.marinus]           -----------------------------------DFQSSTC------------------          1613
XP_032219396.1[N.vectensis]         SFGGRGQPQVFAT--SASEILHPRLA----RTPSEL-----A------------------          1956
XP_019851276.1[A.queenslandica]     YYAGLADKKT--T--QASPFSKAGGK----KEPSGP-----TGRGAL---LSSI------          2129
XP_019636962.1[B.belcheri]          A----------------------------------L------------------------          2077
XP_014020296.2[S.salar]             PKPGVGAI--SHT--ATFPKLQAGKD-LLLSKPREPYPVELCETSKMWTVDNSLSEDKDC          1719
XP_041440458.1[X.laevis]            -----------------------LEQ----TQLAVS-------------MIETE------          1436
XP_046764289.1[G.gallus]            ------------T--HNSPVPQHHKKTKLKAKYRKK-------------MVTGL------          1436
EDL36193.1[M.musculus]              -----------------------PNN-RVSEIIMES-------------VESSL------          1369
NP_006428.2[H.sapiens]              PIRGFGSY--HPS--ASSPFHFQPSAASLTANLRLP-------------MASAL------          1483
NP_001125927.1[P.abelii]            PIRGFGSY--HLS--ASSPFHFQPSAASLTANLRLP-------------IASAL------          1479

XP_047129290.1[H.vulgaris]          --------------------------------------DDL---------GMPKREE---          1682
XP_032828733.1[P.marinus]           --------------------------------------------------Q---------          1614
XP_032219396.1[N.vectensis]         -------------PKGVP--------------------------SQ--------TNDEAP          1969
XP_019851276.1[A.queenslandica]     -------------KKGVS--------------------------LQ--KVTVEKKEEEKS          2148
XP_019636962.1[B.belcheri]          ---------------------YVYGLGETTKRMGARIGARIGARSLKKAAELPKGEEEEP          2116
XP_014020296.2[S.salar]             GLVEMDHWQPSVVPASVPPSGFSFGGGRVGSQSSQHHGSWFGDTAN--ACYIPGLFEASS          1777
XP_041440458.1[X.laevis]            -------------PE-----------------KAEHEGADFVRPAR--KKKIYYNTVEEP          1464
XP_046764289.1[G.gallus]            ------------------------------------------------------------          1436
EDL36193.1[M.musculus]              -------------P------------------------SDYSSRDA--SS----------          1380
NP_006428.2[H.sapiens]              -------------P------------------------EALCSQSR--TTPVDLCLLEES          1504
NP_001125927.1[P.abelii]            -------------P------------------------EALCSQSQ--TTPVDLCLLQES          1500

XP_047129290.1[H.vulgaris]          ---------HSLDLCDDL-------------------EMLADEKLSL-----DYRPAATG          1709
XP_032828733.1[P.marinus]           ---------AASRLCEESPFGGSSNLS-------------ESF-------SSED------          1639
XP_032219396.1[N.vectensis]         VFMSRLQLLESIR------AK------------KKTKETDRED--I----MKQDDS----          2001
XP_019851276.1[A.queenslandica]     VSASLMQALQSRK------APEAFNLKLVDYSKWKSVEMSDDD--SWDDDEEDDDEGGGG          2200
XP_019636962.1[B.belcheri]          M-----EVEESPAICGEQVEEESMDLKLRKLKKKKVLELRAEFEKKYESLPVDER---EE          2168
XP_014020296.2[S.salar]             A-----GSLFGPTLPYLGSSGRSMGLS---------------FEAPLT------------          1805
XP_041440458.1[X.laevis]            M-----GRLHGSRRCVRGEEVA---LS---------------QK----------------          1485
XP_046764289.1[G.gallus]            ------------QDATASEQ------E---------------F-----------------          1446
```

```
EDL36193.1[M.musculus]               Y-----LALEGAEDSLLGGSSF---ET---------------D-----------------           1400
NP_006428.2[H.sapiens]               V-----GSLEGSRCPVFAFQSS---DT---------------E-----------------           1524
NP_001125927.1[P.abelii]             V-----GSLEGSRCPVFVFRSS---DT---------------E-----------------           1520


XP_047129290.1[H.vulgaris]           TISACHFVKP---------------------------------------ISCAYYE-PCF           1729
XP_032828733.1[P.marinus]            -----------------------------------DYGMPF--------ESCLMFH----           1652
XP_032219396.1[N.vectensis]          --GEADILRREAHQH----SMETEL-------EADRNEAEMGVRPVPRKRGKRMLE-KK-           2046
XP_019851276.1[A.queenslandica]      GGGRSRLLVNEFFAFQDQERAEDKVQGQEEEEEESDDNMAFGLFDSF--DEEALSN-ASY           2257
XP_019636962.1[B.belcheri]           RDGRSSLLNQIMRGTSL-------------------------------RRGPGAYHSASF           2197
XP_014020296.2[S.salar]              -GGHLNSPLPTTHGFNLTH-------------------------PLGASLGSSLFG-SSL           1838
XP_041440458.1[X.laevis]             -----------------------------------------------RERAPPVF--NAL           1496
XP_046764289.1[G.gallus]             -----------------------------------------------PERPKRLLQPSCF           1459
EDL36193.1[M.musculus]               -----------------------------------------------TDEAAAFIANDLL           1413
NP_006428.2[H.sapiens]               -----------------------------------------------SDELSEVLQDSCF           1537
NP_001125927.1[P.abelii]             -----------------------------------------------SDELSEVLQDSRI           1533


XP_047129290.1[H.vulgaris]           LERKKTSIENG------LKSVFDCFFKDEKFERPFLDGRSFVFLNKTSNEDLINNTLEFL           1783
XP_032828733.1[P.marinus]            --TS----------------PGVSLCGSSG-----------SSEFSDTTPWQ-HADLSKI           1682
XP_032219396.1[N.vectensis]          --AA---------------EQK-----KEAMEEPS------ESLQNRMRKWMTADKLLEL           2078
XP_019851276.1[A.queenslandica]      YSMS---------------ENA-----QESDGEKV------IEVQNQF-PVINSSQIYMM           2290
XP_019636962.1[B.belcheri]           HPTS---------------DSAGYFSGTETVVVPI------VTTRGSGLTEKTVDQLFAL           2236
XP_014020296.2[S.salar]              RTLQQHHVLPQAQKPLDIKKNIQKLVGDSER-REG------LEFRKNTL---DVVKWTEI           1888
XP_041440458.1[X.laevis]             LDLQC--------EGSVCAPPPIRFI-SVTP--PV------ALSQKRGR---ALPAFNAL           1536
XP_046764289.1[G.gallus]             ITT--------------------------EK-E--------RSQQKLEL---TPVMWEEI           1481
EDL36193.1[M.musculus]               TSI----------------------E-TSSD-EEC------AFCDEDQE---SPVPWASL           1440
NP_006428.2[H.sapiens]               LQIKC--------DTKD--DSILCFL-EVKE-EDE------IVCIQHWQ---DAVPWTEL           1576
NP_001125927.1[P.abelii]             LQIKC--------DTKD--DSIPCFL-EVIE-EDE------IVCTQHWQ---DAVPWTEL           1572
                                                                                        :

XP_047129290.1[H.vulgaris]           VENQHSDGYWELEDIFQDQQSTISFDILFVDVCRKLALSLCGEKYSSEIQRLIATIIVCV           1843
XP_032828733.1[P.marinus]            TCLQNKDGSWDGGVELSSL-VGWSPDH-ITVILKKQGIQSLGLRMEEAIVRLVWTLLVLQ           1740
XP_032219396.1[N.vectensis]          FASQHNDGYWSEK-ELEVA-FRI-DCSFLSRLLVNAGAQSLGDEAFETILKMLTTIITLA           2135
XP_019851276.1[A.queenslandica]      YSLQKDDGSWLLS-DLDTI-VMPGTGDRVLKVLNEAGAKSLGSRVYKELLSLIATCFVLR           2348
XP_019636962.1[B.belcheri]           LKHTDQGSYWEFSPELDQL-LGISSSTCVQ-IFNTAGLRSLGAKVAKQLLQLVATLLVLQ           2294
XP_014020296.2[S.salar]              FALQHSEGYWECSASLGSL-LGVDVDYFANVFLKNRGISSLGVRAHADILRLVASLLVLQ           1947
XP_041440458.1[X.laevis]             LDLQCEEGYWLLNEELGCL-LNMNVNYLIEVFLSKNGIQSLGKKGKDEVLKLIATLLVLQ           1595
XP_046764289.1[G.gallus]             FDLQNQDGSWNLSPQLGKI-LKFDVDYLINHFLIRNGIQSLGIKGKEKLLKLIATLLVLQ           1540
EDL36193.1[M.musculus]               FALQTENGFWKLTPELGLI-LNLNVNALL-TSLEEKGIRSLGTKGRERLLDLIATLLVLQ           1498
NP_006428.2[H.sapiens]               LSLQTEDGFWKLTPELGLI-LNLNTNGLH-SFLKQKGIQSLGVKGRECLLDLIATMLVLQ           1634
NP_001125927.1[P.abelii]             FSLQTEDGFWKLTPELGLI-LNLNTNALH-SFLKQKGIQSLGIKGRERLLDLIATMLVLQ           1630
                                          . . *     :                    .    * .    :  :: : :.

XP_047129290.1[H.vulgaris]           IFKLALSNDKALWSY--P-YHLRESNLKA----SDKLTSAFFKGKSYILHEDCKFPSIHT           1896
XP_032828733.1[P.marinus]            ILRLRMGLASSLNEFVLLLLRRDVSTLAELDMFMPGSADAFARAASWIRSTESVCPCVCT           1800
XP_032219396.1[N.vectensis]          ILKTYLPDQFPVSLV--QGVSSEG--LV-----TSEWRNQVGRALSWLRKMDRKMPSVCS           2186
XP_019851276.1[A.queenslandica]      LLNLNFPDAYKITFS--PHFEIDMSNGD-----PGDIATKMNKSLVLCNKLHKQNPSVVT           2401
XP_019636962.1[B.belcheri]           LLMLHLPQLFPSCSS--L-LHLDVMQVA-----AE-WRSAVQQVLAWAKGVDTMHPSVYS           2345
XP_014020296.2[S.salar]              LMRVRRLEEGKLLLS--L-FRLDLDHSPP--QPRCERWEAVRRAVDWVCWADREYPCVCS           2002
XP_041440458.1[X.laevis]             TI-CTYELVNVTFKN--L-MKLDESFCT---SP---FYETIEKSMKWARKRDKQYPGICY           1645
XP_046764289.1[G.gallus]             FIRCANELQGIVFKS--L-MKLDNLTTS---SGVHWAFESIKKATEWVKRVEGQFPSICY           1594
EDL36193.1[M.musculus]               FLYTKLEQEGMVAKS--L-IKMDDAFIS---RNIPWAFENIKKAREWARKTEGQYPSICQ           1552
NP_006428.2[H.sapiens]               FIRTRLEKEGIVFKS--L-MKMDDASIS---RNIPWAFEAIKQASEWVRRTEGQYPSICP           1688
NP_001125927.1[P.abelii]             FIRTRLEKEGIVFKS--L-MKMDDPSIS---RNIPWAFEAIKKASEWVRRTEGQYPSICP           1684
                                      :                                      . :        .   * :

XP_047129290.1[H.vulgaris]           RLGLGYSLEEMTFQVYRLASQSFDKRMELRL----------      1927
XP_032828733.1[P.marinus]            RLELGPNWQAATCSLLGVA----------------------      1819
XP_032219396.1[N.vectensis]          RLELGPDWDVVAKGIIDDKVHLMIGVR--------------      2213
XP_019851276.1[A.queenslandica]      RLELGYSLIDAVDKILKL----CA-----------------      2421
XP_019636962.1[B.belcheri]           RLELGKSWDDLTKKLIGVGVSLVHE----------------      2370
XP_014020296.2[S.salar]              RLEFGWDWESSTRQLLGIDPPHPLSPLILLGT--TGGVRAQ      2041
XP_041440458.1[X.laevis]             RLGLGKDWDSATRKLLSIDPVDPSSDLFPAVESSVESIV--      1684
XP_046764289.1[G.gallus]             RLELGKDWDSATKMILGIKCN--------------------      1615
EDL36193.1[M.musculus]               RLELGKDWESATKQLLGIQPQAN-TSLHRILYYSQG-----      1587
NP_006428.2[H.sapiens]               RLELGNDWDSATKQLLGLQPISTVSPLHRVLHYSQG-----      1724
NP_001125927.1[P.abelii]             RLELGKDWDSATKQLLGLQPVNTVSPLHRVLHYSQG-----      1720
                                     ** :* .    .  :
```

Percent identity matrix:

```
#
#
#  Percent Identity  Matrix - created by Clustal2.1
#
#
```

1: XP_047129290.1[H.vulgaris]	100.00	32.26	38.59	36.70	35.72	31.35	33.51	32.90	32.98	31.27	31.01
2: XP_032828733.1[P.marinus]	32.26	100.00	35.34	33.67	38.19	37.01	37.08	36.52	37.52	35.76	35.56
3: XP_032219396.1[N.vectensis]	38.59	35.34	100.00	40.86	39.21	34.87	34.69	35.85	35.81	33.82	33.88
4: XP_019851276.1[A.queenslandica]	36.70	33.67	40.86	100.00	37.82	33.09	35.17	34.30	34.63	31.77	31.83
5: XP_019636962.1[B.belcheri]	35.72	38.19	39.21	37.82	100.00	37.25	39.20	38.54	38.25	35.98	35.56
6: XP_014020296.2[S.salar]	31.35	37.01	34.87	33.09	37.25	100.00	41.80	43.30	41.68	40.36	39.81
7: XP_041440458.1[X.laevis]	33.51	37.08	34.69	35.17	39.20	41.80	100.00	48.16	47.33	47.32	46.94
8: XP_046764289.1[G.gallus]	32.90	36.52	35.85	34.30	38.54	43.30	48.16	100.00	52.14	53.34	53.41
9: EDL36193.1[M.musculus]	32.98	37.52	35.81	34.63	38.25	41.68	47.33	52.14	100.00	70.25	70.02
10: NP_006428.2[H.sapiens]	31.27	35.76	33.82	31.77	35.98	40.36	47.32	53.34	70.25	100.00	94.36
11: NP_001125927.1[P.abelii]	31.01	35.56	33.88	31.83	35.56	39.81	46.94	53.41	70.02	94.36	100.00
