# Supplementary material for: An Evolutionary Perspective on the Origin, Conservation and Binding Partner Acquisition of Tankyrases
Source: Biomolecules. 2022 Nov 15;12(11):1688. doi: 10.3390/biom12111688 (PMC9688111; doi:10.3390/biom12111688)
Supplement: Supplementary file 1 [file biomolecules-12-01688-s001.zip › biomolecules-1913719-supplementary-11.17 -done/Data_2/PARP6.pdf]

PARP6

Sequences:

>NP_001310451.1 protein mono-ADP-ribosyltransferase PARP6 isoform 1 [Homo sapiens]
MDIKGQFWNDDDSEGDNESEEFLYGVQGSCAADLYRHPQLDADIEAVKEIYSENSVSIREYGTIDDVDID
LHINISFLDEEVSTAWKVLRTEPIVLRLRFSLSQYLDGPEPSIEVFQPSNKEGFGLGLQLKKILGMFTSQ
QWKHLSNDFLKTQQEKRHSWFKASGTIKKFRAGLSIFSPIPKSPSFPIIQDSMLKGKLGVPELRVGRLMN
RSISCTMKNPKVEVFGYPPSPQAGLLCPQHVGLPPPARTSPLVSGHCKNIPTLEYGFLVQIMKYAEQRIP
TLNEYCVVCDEQHVFQNGSMLKPAVCTRELCVFSFYTLGVMSGAAEEVATGAEVVDLLVAMCRAALESPR
KSIIFEPYPSVVDPTDPKTLAFNPKKKNYERLQKALDSVMSIREMTQGSYLEIKKQMDKLDPLAHPLLQW
IISSNRSHIVKLPLSRLKFMHTSHQFLLLSSPPAKEARFRTAKKLYGSTFAFHGSHIENWHSILRNGLVN
ASYTKLQLHGAAYGKGIYLSPISSISFGYSGMGKGQHRMPSKDELVQRYNRMNTIPQTRSIQSRFLQSRN
LNCIALCEVITSKDLQKHGNIWVCPVSDHVCTRFFFVYEDGQVGDANINTQDPKIQKEIMRVIGTQVYTN

>XP_024087899.1 poly [ADP-ribose] polymerase 6 isoform X2 [Pongo abelii]
MDIKGQFWNDDDSEGDNESEEFLYGVQGSCAADLYRHPQLDADIEAVKEIYSENSVSIREYGTIDDVDID
LHINISFLDEEVSTAWKVLRTEPIVLRLRFSLSQYLDGPEPSIEVFQPSNKEGFGLGLQLKKILGMFTSQ
QWKHLSNDFLKTQQEKRHSWFKASGTIKKFRAGLSIFSPIPKSPSFPIIQDSMLKGKLGVPELRVGRLMN
RSISCTMKNPKVEVFGYPPSPQAGLLCPQHVGLPPPARTSPLVSGHCKNIPTLEYGFLVQIMKYAEQRIP
TLNEYCVVCDEQHVFQNGSMLKPAVCTRELCVFSFYTLGVMSGAAEEVATGAEVVDLLVAMCRAALESPR
KSIIFEPYPSVVDPTDPKTLAFNPKKKNYERLQKALDSVMSIREMTQGSYLEIKKQMDKLDPLAHPLLQW
IISSNRSHIVKLPLSRLKFMHTSHQFLLLSSPPAKEARFRTAKKLYGSTFAFHGSHIENWHSILRNGLVN
ASYTKLQLHGAAYGKGIYLSPISSISFGYSGMGKGQHRMPSKDELVQRYNRMNTIPQTRSIQSRFLQSRN
LNCIALCEVITSKDLQKHGNIWVCPVSDHVCTRFFFVYEDGQVGDANINTQDPKIQKEIMRVIGTQVYTN

>NP_001192168.1 protein mono-ADP-ribosyltransferase PARP6 isoform 1 [Mus musculus]
MDIKGQFWNDDDSEGDNESEEFLYGVQGSCAADLYRHPQLDADIEAVKEIYSENSVSIREYGTIDDVDLD
LHINISFLDEEVSTAWKVLRTEPIVLRLRFSLSQYLDGPEPSIEVFQPSNKEGFGLGLQLKKILCMFTSQ
QWKHLSNDFLKTQQEKRHSWFKASGTIKKFRAGLSIFSPIPKSPSFPIIQDSMLKGKLGVPELRVGRLMN
RSISCTMKNPKVEVFGYPPSPQAGLLCPQHVGLPPPARTSPLVSGHCKNIPTLEYGFLVQIMKYAEQRIP
TLNEYCVVCDEQHVFQNGSMLKPAVCTRELCVFSFYTLGVMSGAAEEVATGAEVVDLLVAMCRAALESPR
KSIIFEPYPSVVDPTDPKTLAFNPKKKNYERLQKALDSVMSIREMTQGSYLEIKKQMDKLDPLAHPLLQW
IISSNRSHIVKLPLSRLKFMHTSHQFLLLSSPPAKEARFRTAKKLYGSTFAFHGSHIENWHSILRNGLVN
ASYTKLQLHGAAYGKGIYLSPISSISFGYSGMGKGQHRMPSKDELVQRYNRMNTIPQTRSIQSRFLQSRN
LNCIALCEVITSKDLQKHGNIWVCPVSDHVCTRFFFVYEDGQVGDANINTQDPKIQKEIMRVIGTQVYTN

>XP_015134546.2 protein mono-ADP-ribosyltransferase PARP6 isoform X9 [Gallus gallus]
MGTQRGAAARPHSRHGADGRDGSGFEAGVPAVTSPPARRCRATEARGPDVKARWPPARPAAWRRQGRGQA
AGSAEMDLKGQYWTDDDSDGDNESEEFLYGVQGTCAADLYRHPQLDADIEAVKEIYSENAVSVREYGTID
DVDIDLHVNISFLDEEVATAWKVLRTEPIVLRLRFSLSQYLDGPEPSIEVFQPSNKEGFGLGLQLKKILG
MFTSQQWKHLSNDFLKTQQEKRHSWFKTSGTIKKFRAGLSIFSPIPKSPSFPVIPDSVLKGKLGAPEVRV
NRLMNRSVSCTVKNPKGEVFGYTPSTQAGVAPFNILVGGHCKNVPTLEYGFLVQIMKYAEQRIPTLNEYC
VVCDEQHVFQNGSMLKPAVCTRELCVFSFYTLGVMSGAAEEVATGAEVVDLLVAMCRAALESPRKSIIFE
PYPSVVDPNDPKTLAFNPKKKNYERLQKALDSVMSIREMTQGSYLEIKKQMDKLDPLAHPLLQWIISSNR
SHIVKLPLSRLKFMHTSHQFLLLSSPPAKEARFRTAKKLYGSTFAFHGSHIENWHSILRNGLVNASYTKL
QLHGAAYGKGIYLSPISSISFGYSGMGKGQHRMPSKDELVQRYNRMNTIPQTRSIQSRFLQSRNLNCIAL
CEVITSKDLQKHGNIWVCPVSDHVCTRFFFVYEDGQVGDANINTQDPKIQKEIMRVIGTQVYTN

>XP_041444306.1 protein mono-ADP-ribosyltransferase PARP6 isoform X2 [Xenopus laevis]
MDIKGHNWPEEDSDGDNECGEFLYGVQGTCCADLYRHPQLDADIEAVKEIYSENAVSVREYGTIDDVDID
LHINIGFLDEEVATAWKVIRTEPIVLRLRFSLSQYLDGPEPTIDVFQPSNRDAFGLGIQLKKIVSLFSSH
QWRYLSNEVLRSQQERRLRWLRVSGSIKRFRAGLSIFSPVPKSPSFPGVQDSVVRSGLTGSDLRSGLTGS
DLRGPRLMNRSVSCTLRNPKSELYGSHNPTTQPLSSIQVSGHCKTVPTLEYGFLVQIMKYAEQRLPTLNE
YCVVCDEQHVFQNASMLKPAVCTRELCVFSFYTLGVMSGAAEEVATGAEVVGLLVAMCRAALESPRKSII
FEPYPSVVDPNDPKTLAFNPKKKNYERLQKALDSVMSIREMTQGSYLEIKKQMDRLDPLAHPLLQWIISS
NRSHIVKLPLSRLKFMHTSHQFLLLSSPPAKEARFRTAKKLYGSTFAFHGSHIENWHSILRNGLVNASYT
KLQLHGAAYGKGIYLSPISSISFGYSGMGKGQHRMPSKDELVQRYNRMNTIPQTRCLQSRFLQSRNLNCI
ALCEVITSKDLQKHGNIWVCPVSDHVCTRFFFVYEDGQVGDANINTQDPKIQKEIMRVIGTQVYSG

>XP_014030859.1 protein mono-ADP-ribosyltransferase PARP6 isoform X4 [Salmo salar]
MDIKGQSWTDEESDGENESEQFLYGIQGSCAADLYRHPQLDADIEAVKDIYTDSAVSVREYGTIDDVDID
LQINISFLDEEVATAWKVIRTEPIILRLRFSLSQYLDGPEPSVEVFQPSNKEGFSLGLQLKKILSTFTSQ
QWKHLSNEFLKAQQEKRHSWFKAGGTIKKFRAGLSIFSPIPKSPSYPLIQDTVLKGKLSVPELRVTRLMN
RSISCTMKNPKGELFSYPPNSQTVAVPAARAPAQITTRQLIELFFSSQAGGHCKNIPTLEYGFLVQIMKY
SEQRIPTLNEYCVVCDEQHVFQNGSMLKPAVCTRELCVFSFYTLGVMSGAAEEVATGAEVVDLLVAMCRA
ALESPRKSIIFEPYPSVVDPNDPKTLAFNPKKKNYERLQKALDSVMSIREMTQGSYLEIKKQMDKLDPLA
HPLLQWIISSNRSHIVKLPLSRQLKFMHTSHQFLLLSSPPAKEARFRTAKKLYGSTFAFHGSHIENWHSV
LRNGLVNASYTKLQLHGAAYGKGIYLSPISSISFGYSGMGKGQHRMPTKDELVQRYNRMNTIPQSRPIQS
RFLQSRNLNCIALCEVITSKDLQKHGNIWVCPVSDHVCTRFFFVYEDGQVGDANINTQEPKVQKEIMRVI
GTQIYSS

>XP_032806551.1 protein mono-ADP-ribosyltransferase PARP6-like isoform X1 [Petromyzon marinus]
MDQDLSAPKWTGEESDGETESDEFFCGVQVNADGETHRHPQLDADIESVREKYGDGAVQIREYGAIDDVA
VDLHINVGFLDEEIATAWKVMRTEAVVLRLHFSLSQYLDGPEPSVDVFQPSNKDSFGIGLQLKKVLKAFV
SQQWKHLSNDCLQSQHSKRLNLLKSSRNAKKLRSPLSLLSHLHRSPSFLFHQDGGGRGKRNLEQNGSSQQ
GNATKTLPILEHGFLVQTMLYGEQRIPTLNEFCVVCDEPHVFQNGPMLKPAVCARELCVFAFQTLGVMTG
AAAEVATGPEVVDLLLAMCRSALESPRKSIIFDPYPSVVDPNDPKALAFNPKKKNYERLQRALDSIMSVR
EMTQGSYLDIKKQMDKQDSLAHPLLQWVISSNRSHIVKLQSSRQLKFMHTPHQFLLLSSPPAKEARFRAA
KKLYGSSFAFHGSHTENWHSILRNGLVNASYTKLQLHGAAYGKGIYLSPISSISFGYSGMGKGQHRMESK
EKLVQNCNRVNTTPQIKTMQSRFLQSRNLNCIALCEVITSKDLQKHGNIWVCPMSDHVCTRFFFVYEDGQ
VGDANINTQDHKVQAEILRILGMQSVSL

>XP_019614591.1 PREDICTED: poly [ADP-ribose] polymerase 6-like isoform X5 [Branchiostoma belcheri]
MHPQLDNDIKSVEQRFGAHAINTRIFSSIDDVDAELNIDMSFLDEEIAKAWKVNRQEPIVIRLHLSFSQY
LDANEPPKVEVFQPSNKETFGLGMQIQKIMETFLSKEWKDLSNDRVTAICVPLRPISARSSHSHKGASSH
GKKLKQLHEMSKSFQSLVSSWKVRPVKKIHDRHVAQLVDMGFTVEMARNALFMTRNNLEEAINLLLTQPE
SCRPAGGGGAGSSSFRGTWTSALFLAPSVAQQHQVVLPARPSQPLKPATIGDELHLVPCSTERGRTAKTI
PGLEYGFLAQCLIYARNRIPTLNEYCVVCDEPHIFQNGAMLKPVVCEREVCVFAFQQLGVMRGAADDIAT
GAEVVDLLLAMVKASSHSPRKDLILDPFPSVVDPDNAKEMVLSPKKKNYDRVNAILDSLMSVREMTQASV
DMKKQLDKKDKYAWPFLSWVISSNRSHIVKLPESRRIKFMHTPHQFLLLSSPPAKEQAFREAKRQHGSLF
AFHGSHIENWHSILRHGLINASGTKHQLHGAAYGSGIYLSPNSSVSFGYSCMGHGSHKAARNKALLVTPH
RKGFLQGCKALGCTNQTTAASEKTGKKENTKRFLESDNLTCIALCEVIHSKELRKHNNIWVVPNPDHVCT
RFFFVYEDGQVGDSMVDTTQEKYQKEILRAIGNMTATD

>XP_019849465.1 PREDICTED: poly [ADP-ribose] polymerase 6-like [Amphimedon queenslandica]
MLPEDRQLKSMDTKYQFLLISSPPAKEMAFQQAKLKHGSTFAFHGSSIENWHSIMRKGLINASGTKYQLN
GAAYGSGIYLSPHAMTSFGYSNRYYYDPSSQKKGSSGKQLETLAGKLNITCIALCEVVTSKELRRSGDIW
VCSNSEHVVTRFFFVYDTNSATSEASSLHTETNRAFKQEIMHAVALAKKMKLDNSTASKTK

Alignment

```
XP_019849465.1[A.queenslandica]      ------------------------------------------------------------      0
XP_019614591.1[B.belcheri]           ------------------------------------------------------------      0
XP_032806551.1[P.marinus]            ------------------------------------------------------------      0
XP_041444306.1[X.laevis]             ------------------------------------------------------------      0
XP_014030859.1[S.salar]              ------------------------------------------------------------      0
XP_015134546.2[G.gallus]             MGTQRGAAARPHSRHGADGRDGSGFEAGVPAVTSPPARRCRATEARGPDVKARWPPARPA      60
NP_001310451.1[H.sapiens]            ------------------------------------------------------------      0
XP_024087899.1[P.abelii]             ------------------------------------------------------------      0
NP_001192168.1[M.musculus]           ------------------------------------------------------------      0


XP_019849465.1[A.queenslandica]      ------------------------------------------------------------      0
XP_019614591.1[B.belcheri]           --------------------------------------------------MHPQLDNDIK      10
XP_032806551.1[P.marinus]            -------------MDQDLSAPKWTGEESDGETESDEFFCGVQVNADGETHRHPQLDADIE      47
XP_041444306.1[X.laevis]             ---------------MDIKGHNWPEEDSDGDNECGEFLYGVQGTCCADLYRHPQLDADIE      45
XP_014030859.1[S.salar]              ---------------MDIKGQSWTDEESDGENESEQFLYGIQGSCAADLYRHPQLDADIE      45
XP_015134546.2[G.gallus]             AWRRQGRGQAAGSAEMDLKGQYWTDDDSDGDNESEEFLYGVQGTCAADLYRHPQLDADIE      120
NP_001310451.1[H.sapiens]            ---------------MDIKGQFWNDDDSEGDNESEEFLYGVQGSCAADLYRHPQLDADIE      45
XP_024087899.1[P.abelii]             ---------------MDIKGQFWNDDDSEGDNESEEFLYGVQGSCAADLYRHPQLDADIE      45
NP_001192168.1[M.musculus]           ---------------MDIKGQFWNDDDSEGDNESEEFLYGVQGSCAADLYRHPQLDADIE      45


XP_019849465.1[A.queenslandica]      ------------------------------------------------------------      0
XP_019614591.1[B.belcheri]           SVEQRFGAHAINTRIFSSIDDVDAELNIDMSFLDEEIAKAWKVNRQEPIVIRLHLSFSQY      70
XP_032806551.1[P.marinus]            SVREKYGDGAVQIREYGAIDDVAVDLHINVGFLDEEIATAWKVMRTEAVVLRLHFSLSQY      107
XP_041444306.1[X.laevis]             AVKEIYSENAVSVREYGTIDDVDIDLHINIGFLDEEVATAWKVIRTEPIVLRLRFSLSQY      105
XP_014030859.1[S.salar]              AVKDIYTDSAVSVREYGTIDDVDIDLQINISFLDEEVATAWKVIRTEPIILRLRFSLSQY      105
XP_015134546.2[G.gallus]             AVKEIYSENAVSVREYGTIDDVDIDLHVNISFLDEEVATAWKVLRTEPIVLRLRFSLSQY      180
NP_001310451.1[H.sapiens]            AVKEIYSENSVSIREYGTIDDVDIDLHINISFLDEEVSTAWKVLRTEPIVLRLRFSLSQY      105
XP_024087899.1[P.abelii]             AVKEIYSENSVSIREYGTIDDVDIDLHINISFLDEEVSTAWKVLRTEPIVLRLRFSLSQY      105
NP_001192168.1[M.musculus]           AVKEIYSENSVSIREYGTIDDVDLDLHINISFLDEEVSTAWKVLRTEPIVLRLRFSLSQY      105


XP_019849465.1[A.queenslandica]      ------------------------------------------------------------      0
XP_019614591.1[B.belcheri]           LDANEPPKVEVFQPSNKETFGLGMQIQKIMETFLSKEWKDLSNDRVTAICVPLRPISARS      130
XP_032806551.1[P.marinus]            LDGPE-PSVDVFQPSNKDSFGIGLQLKKVLKAFVSQQWKHLSNDCLQSQHSKRLNL----      162
XP_041444306.1[X.laevis]             LDGPE-PTIDVFQPSNRDAFGLGIQLKKIVSLFSSHQWRYLSNEVLRSQQERRLRW----      160
XP_014030859.1[S.salar]              LDGPE-PSVEVFQPSNKEGFSLGLQLKKILSTFTSQQWKHLSNEFLKAQQEKRHSW----      160
XP_015134546.2[G.gallus]             LDGPE-PSIEVFQPSNKEGFGLGLQLKKILGMFTSQQWKHLSNDFLKTQQEKRHSW----      235
NP_001310451.1[H.sapiens]            LDGPE-PSIEVFQPSNKEGFGLGLQLKKILGMFTSQQWKHLSNDFLKTQQEKRHSW----      160
XP_024087899.1[P.abelii]             LDGPE-PSIEVFQPSNKEGFGLGLQLKKILGMFTSQQWKHLSNDFLKTQQEKRHSW----      160
NP_001192168.1[M.musculus]           LDGPE-PSIEVFQPSNKEGFGLGLQLKKILCMFTSQQWKHLSNDFLKTQQEKRHSW----      160


XP_019849465.1[A.queenslandica]      ------------------------------------------------------------      0
XP_019614591.1[B.belcheri]           SHSHKGASSHGKKLKQLHEMSKSFQSLVSSWK---VRPVKKIH-D-----RHVAQLVDMG      181
XP_032806551.1[P.marinus]            ----LKSSRN---A-------KKLRSPLSLLSHLHRSPSFLFHQDGGGRGKRN-------      201
XP_041444306.1[X.laevis]             ----LRVSGS---I-------KRFRAGLSIFSPVPKSPSFPGVQDSVVRSGLTGSDLRSG      206
XP_014030859.1[S.salar]              ----FKAGGT---I-------KKFRAGLSIFSPIPKSPSYPLIQDTVLKGKLS-------      199
XP_015134546.2[G.gallus]             ----FKTSGT---I-------KKFRAGLSIFSPIPKSPSFPVIPDSVLKGKLG-------      274
NP_001310451.1[H.sapiens]            ----FKASGT---I-------KKFRAGLSIFSPIPKSPSFPIIQDSMLKGKLG-------      199
XP_024087899.1[P.abelii]             ----FKASGT---I-------KKFRAGLSIFSPIPKSPSFPIIQDSMLKGKLG-------      199
NP_001192168.1[M.musculus]           ----FKASGT---I-------KKFRAGLSIFSPIPKSPSFPIIQDSMLKGKLG-------      199


XP_019849465.1[A.queenslandica]      ------------------------------------------------------------      0
XP_019614591.1[B.belcheri]           FTVEMARNALFMTRNNLEEAINLLLTQPESCRPAGGGGAGSSSFRGTWTSALFLAPSVAQ      241
XP_032806551.1[P.marinus]            ------------------------------------------------------------      201
XP_041444306.1[X.laevis]             LTGSDLR-------------GPRLMNRSVSCTLRNPKS------------ELYGSHNPTT      241
XP_014030859.1[S.salar]              --VPELR-------------VTRLMNRSISCTMKNPKG------------ELFSYPPNSQ      232
XP_015134546.2[G.gallus]             --APEVR-------------VNRLMNRSVSCTVKNPKG------------EVFGYTPSTQ      307
NP_001310451.1[H.sapiens]            --VPELR-------------VGRLMNRSISCTMKNPKV------------EVFGYPPSPQ      232
XP_024087899.1[P.abelii]             --VPELR-------------VGRLMNRSISCTMKNPKV------------EVFGYPPSPQ      232
NP_001192168.1[M.musculus]           --VPELR-------------VGRLMNRSISCTMKNPKV------------EVFGYPPSPQ      232


XP_019849465.1[A.queenslandica]      ------------------------------------------------------------      0
XP_019614591.1[B.belcheri]           QHQVVLPARPSQPLKPATI-GDELHLVPCSTERGRTAKTIPGLEYGFLAQCLIYARNRIP      300
XP_032806551.1[P.marinus]            ------------------------LEQNGSSQQGNATKTLPILEHGFLVQTMLYGEQRIP      237
XP_041444306.1[X.laevis]             --Q-----------------------PLSSIQVSGHCKTVPTLEYGFLVQIMKYAEQRLP      276
XP_014030859.1[S.salar]              --TVAVPAA----RAPAQITTRQLIELFFSSQAGGHCKNIPTLEYGFLVQIMKYSEQRIP      286
XP_015134546.2[G.gallus]             --AGVA---------------------PFNILVGGHCKNVPTLEYGFLVQIMKYAEQRIP      344
NP_001310451.1[H.sapiens]            --AGLLCPQ----HVG------LPPPARTSPLVSGHCKNIPTLEYGFLVQIMKYAEQRIP      280
XP_024087899.1[P.abelii]             --AGLLCPQ----HVG------LPPPARTSPLVSGHCKNIPTLEYGFLVQIMKYAEQRIP      280
NP_001192168.1[M.musculus]           --AGLLCPQ----HVG------LPPPARTSPLVSGHCKNIPTLEYGFLVQIMKYAEQRIP      280


XP_019849465.1[A.queenslandica]      ------------------------------------------------------------      0
XP_019614591.1[B.belcheri]           TLNEYCVVCDEPHIFQNGAMLKPVVCEREVCVFAFQQLGVMRGAADDIATGAEVVDLLLA      360
XP_032806551.1[P.marinus]            TLNEFCVVCDEPHVFQNGPMLKPAVCARELCVFAFQTLGVMTGAAAEVATGPEVVDLLLA      297
XP_041444306.1[X.laevis]             TLNEYCVVCDEQHVFQNASMLKPAVCTRELCVFSFYTLGVMSGAAEEVATGAEVVGLLVA      336
XP_014030859.1[S.salar]              TLNEYCVVCDEQHVFQNGSMLKPAVCTRELCVFSFYTLGVMSGAAEEVATGAEVVDLLVA      346
XP_015134546.2[G.gallus]             TLNEYCVVCDEQHVFQNGSMLKPAVCTRELCVFSFYTLGVMSGAAEEVATGAEVVDLLVA      404
NP_001310451.1[H.sapiens]            TLNEYCVVCDEQHVFQNGSMLKPAVCTRELCVFSFYTLGVMSGAAEEVATGAEVVDLLVA      340
XP_024087899.1[P.abelii]             TLNEYCVVCDEQHVFQNGSMLKPAVCTRELCVFSFYTLGVMSGAAEEVATGAEVVDLLVA      340
NP_001192168.1[M.musculus]           TLNEYCVVCDEQHVFQNGSMLKPAVCTRELCVFSFYTLGVMSGAAEEVATGAEVVDLLVA      340


XP_019849465.1[A.queenslandica]      ------------------------------------------------------------      0
XP_019614591.1[B.belcheri]           MVKASSHSPRKDLILDPFPSVVDPDNAKEMVLSPKKKNYDRVNAILDSLMSVREMTQAS-      419
XP_032806551.1[P.marinus]            MCRSALESPRKSIIFDPYPSVVDPNDPKALAFNPKKKNYERLQRALDSIMSVREMTQGSY      357
XP_041444306.1[X.laevis]             MCRAALESPRKSIIFEPYPSVVDPNDPKTLAFNPKKKNYERLQKALDSVMSIREMTQGSY      396
XP_014030859.1[S.salar]              MCRAALESPRKSIIFEPYPSVVDPNDPKTLAFNPKKKNYERLQKALDSVMSIREMTQGSY      406
XP_015134546.2[G.gallus]             MCRAALESPRKSIIFEPYPSVVDPNDPKTLAFNPKKKNYERLQKALDSVMSIREMTQGSY      464
NP_001310451.1[H.sapiens]            MCRAALESPRKSIIFEPYPSVVDPTDPKTLAFNPKKKNYERLQKALDSVMSIREMTQGSY      400
XP_024087899.1[P.abelii]             MCRAALESPRKSIIFEPYPSVVDPTDPKTLAFNPKKKNYERLQKALDSVMSIREMTQGSY      400
NP_001192168.1[M.musculus]           MCRAALESPRKSIIFEPYPSVVDPTDPKTLAFNPKKKNYERLQKALDSVMSIREMTQGSY      400


XP_019849465.1[A.queenslandica]      ------------------------------MLPEDRQLKSMDTKYQFLLISSPPAKEMAF      30
XP_019614591.1[B.belcheri]           VDMKKQLDKKDKYAWPFLSWVISSNRSHIVKLPESRRIKFMHTPHQFLLLSSPPAKEQAF      479
XP_032806551.1[P.marinus]            LDIKKQMDKQDSLAHPLLQWVISSNRSHIVKLQSSRQLKFMHTPHQFLLLSSPPAKEARF      417
XP_041444306.1[X.laevis]             LEIKKQMDRLDPLAHPLLQWIISSNRSHIVKLPLSR-LKFMHTSHQFLLLSSPPAKEARF      455
XP_014030859.1[S.salar]              LEIKKQMDKLDPLAHPLLQWIISSNRSHIVKLPLSRQLKFMHTSHQFLLLSSPPAKEARF      466
XP_015134546.2[G.gallus]             LEIKKQMDKLDPLAHPLLQWIISSNRSHIVKLPLSR-LKFMHTSHQFLLLSSPPAKEARF      523
NP_001310451.1[H.sapiens]            LEIKKQMDKLDPLAHPLLQWIISSNRSHIVKLPLSR-LKFMHTSHQFLLLSSPPAKEARF      459
XP_024087899.1[P.abelii]             LEIKKQMDKLDPLAHPLLQWIISSNRSHIVKLPLSR-LKFMHTSHQFLLLSSPPAKEARF      459
NP_001192168.1[M.musculus]           LEIKKQMDKLDPLAHPLLQWIISSNRSHIVKLPLSR-LKFMHTSHQFLLLSSPPAKEARF      459
                                                                    *  .* :* *.* :****:*******  *

XP_019849465.1[A.queenslandica]      QQAKLKHGSTFAFHGSSIENWHSIMRKGLINASGTKYQLNGAAYGSGIYLSPHAMTSFGY      90
```

```
XP_019614591.1[B.belcheri]          REAKRQHGSLFAFHGSHIENWHSILRHGLINASGTKHQLHGAAYGSGIYLSPNSSVSFGY          539
XP_032806551.1[P.marinus]           RAAKKLYGSSFAFHGSHTENWHSILRNGLVNASYTKLQLHGAAYGKGIYLSPISSISFGY          477
XP_041444306.1[X.laevis]            RTAKKLYGSTFAFHGSHIENWHSILRNGLVNASYTKLQLHGAAYGKGIYLSPISSISFGY          515
XP_014030859.1[S.salar]             RTAKKLYGSTFAFHGSHIENWHSVLRNGLVNASYTKLQLHGAAYGKGIYLSPISSISFGY          526
XP_015134546.2[G.gallus]            RTAKKLYGSTFAFHGSHIENWHSILRNGLVNASYTKLQLHGAAYGKGIYLSPISSISFGY          583
NP_001310451.1[H.sapiens]           RTAKKLYGSTFAFHGSHIENWHSILRNGLVNASYTKLQLHGAAYGKGIYLSPISSISFGY          519
XP_024087899.1[P.abelii]            RTAKKLYGSTFAFHGSHIENWHSILRNGLVNASYTKLQLHGAAYGKGIYLSPISSISFGY          519
NP_001192168.1[M.musculus]          RTAKKLYGSTFAFHGSHIENWHSILRNGLVNASYTKLQLHGAAYGKGIYLSPISSISFGY          519
                                    : **  :** ******  *****::*:**:*** ** **:*****.****** :  ****

XP_019849465.1[A.queenslandica]     SNRYYYDPSSQKK------------------------------GSSGKQLETLAGKLNI          119
XP_019614591.1[B.belcheri]          SCMGHGSHKAARNKALLVTPHRKGFLQGCKALGCTNQTTAASEKTGKKENTKRFLESDNL          599
XP_032806551.1[P.marinus]           SGMGKGQHRMESKEKLV---------QNCNRVNTTP---------QIKTMQSRFLQSRNL          519
XP_041444306.1[X.laevis]            SGMGKGQHRMPSKDELV---------QRYNRMNTIP---------QTRCLQSRFLQSRNL          557
XP_014030859.1[S.salar]             SGMGKGQHRMPTKDELV---------QRYNRMNTIP---------QSRPIQSRFLQSRNL          568
XP_015134546.2[G.gallus]            SGMGKGQHRMPSKDELV---------QRYNRMNTIP---------QTRSIQSRFLQSRNL          625
NP_001310451.1[H.sapiens]           SGMGKGQHRMPSKDELV---------QRYNRMNTIP---------QTRSIQSRFLQSRNL          561
XP_024087899.1[P.abelii]            SGMGKGQHRMPSKDELV---------QRYNRMNTIP---------QTRSIQSRFLQSRNL          561
NP_001192168.1[M.musculus]          SGMGKGQHRMPSKDELV---------QRYNRMNTIP---------QTRSIQSRFLQSRNL          561
                                    *     .     :                                      . :  . *:

XP_019849465.1[A.queenslandica]     TCIALCEVVTSKELRRSGDIWVCSNSEHVVTRFFFVYDTNSATSEASSLHTETNRAFKQE          179
XP_019614591.1[B.belcheri]          TCIALCEVIHSKELRKHNNIWVVPNPDHVCTRFFFVYEDGQVGDSMV---DTTQEKYQKE          656
XP_032806551.1[P.marinus]           NCIALCEVITSKDLQKHGNIWVCPMSDHVCTRFFFVYEDGQVGDANI---NTQDHKVQAE          576
XP_041444306.1[X.laevis]            NCIALCEVITSKDLQKHGNIWVCPVSDHVCTRFFFVYEDGQVGDANI---NTQDPKIQKE          614
XP_014030859.1[S.salar]             NCIALCEVITSKDLQKHGNIWVCPVSDHVCTRFFFVYEDGQVGDANI---NTQEPKVQKE          625
XP_015134546.2[G.gallus]            NCIALCEVITSKDLQKHGNIWVCPVSDHVCTRFFFVYEDGQVGDANI---NTQDPKIQKE          682
NP_001310451.1[H.sapiens]           NCIALCEVITSKDLQKHGNIWVCPVSDHVCTRFFFVYEDGQVGDANI---NTQDPKIQKE          618
XP_024087899.1[P.abelii]            NCIALCEVITSKDLQKHGNIWVCPVSDHVCTRFFFVYEDGQVGDANI---NTQDPKIQKE          618
NP_001192168.1[M.musculus]          NCIALCEVITSKDLQKHGNIWVCPVSDHVCTRFFFVYEDGQVGDANI---NTQDPKIQKE          618
                                    .*******: **:*:: .:***    :** *******: ... .         :   : *

XP_019849465.1[A.queenslandica]     IMHAVALAKKMKLDNSTASKTK 201
XP_019614591.1[B.belcheri]          ILRAIGNMTATD---------- 668
XP_032806551.1[P.marinus]           ILRILGMQSVSL---------- 588
XP_041444306.1[X.laevis]            IMRVIGTQVYSG---------- 626
XP_014030859.1[S.salar]             IMRVIGTQIYSS---------- 637
XP_015134546.2[G.gallus]            IMRVIGTQVYTN---------- 694
NP_001310451.1[H.sapiens]           IMRVIGTQVYTN---------- 630
XP_024087899.1[P.abelii]            IMRVIGTQVYTN---------- 630
NP_001192168.1[M.musculus]          IMRVIGTQVYTN---------- 630
                                    *:: :.
```

Percent identity matrix:

```
#
#
#  Percent Identity  Matrix - created by Clustal2.1
#
#

     1: XP_019849465.1[A.queenslandica]  100.00   52.13   48.66   50.54   50.80   50.54   50.54   50.54   50.54
     2: XP_019614591.1[B.belcheri]        52.13  100.00   59.15   54.22   56.35   56.79   55.90   55.90   55.90
     3: XP_032806551.1[P.marinus]         48.66   59.15  100.00   73.58   74.57   74.66   74.02   74.02   74.02
     4: XP_041444306.1[X.laevis]          50.54   54.22   73.58  100.00   84.60   87.50   86.55   86.55   86.39
     5: XP_014030859.1[S.salar]           50.80   56.35   74.57   84.60  100.00   90.47   89.21   89.21   89.05
     6: XP_015134546.2[G.gallus]          50.54   56.79   74.66   87.50   90.47  100.00   95.15   95.15   94.83
     7: NP_001310451.1[H.sapiens]         50.54   55.90   74.02   86.55   89.21   95.15  100.00  100.00   99.68
     8: XP_024087899.1[P.abelii]          50.54   55.90   74.02   86.55   89.21   95.15  100.00  100.00   99.68
     9: NP_001192168.1[M.musculus]        50.54   55.90   74.02   86.39   89.05   94.83   99.68   99.68  100.00
```
