# Supplementary material for: An Evolutionary Perspective on the Origin, Conservation and Binding Partner Acquisition of Tankyrases
Source: Biomolecules. 2022 Nov 15;12(11):1688. doi: 10.3390/biom12111688 (PMC9688111; doi:10.3390/biom12111688)
Supplement: Supplementary file 1 [file biomolecules-12-01688-s001.zip › biomolecules-1913719-supplementary-11.17 -done/Data_2/PARP9.pdf]

PARP9

Sequences:

```
>NP_001139574.1 protein mono-ADP-ribosyltransferase PARP9 isoform a [Homo sapiens]
MDFSMVAGAAAYNEKSGRITSLSLLFQKVFAQIFPQWRKGNTEECLPYKCSETGALGENYSWQIPINHND
FKILKNNERQLCEVLQNKFGCISTLVSPVQEGNSKSLQVFRKMLTPRIELSVWKDDLTTHAVDAVVNAAN
EDLLHGGGLALALVKAGGFEIQEESKQFVARYGKVSAGEIAVTGAGRLPCKQIIHAVGPRWMEWDKQGCT
GKLQRAIVSILNYVIYKNTHIKTVAIPALSSGIFQFPLNLCTKTIVETIRVSLQGKPMMSNLKEIHLVSN
EDPTVAAFKAASEFILGKSELGQETTPSFNAMVVNNLTLQIVQGHIEWQTADVIVNSVNPHDITVGPVAK
SILQQAGVEMKSEFLATKAKQFQRSQLVLVTKGFNLFCKYIYHVLWHSEFPKPQILKHAMKECLEKCIEQ
NITSISFPALGTGNMEIKKETAAEILFDEVLTFAKDHVKHQLTVKFVIFPTDLEIYKAFSSEMAKRSKML
SLNNYSVPQSTREEKRENGLEARSPAINLMGFNVEEMYEAHAWIQRILSLQNHHIIENNHILYLGRKEHD
ILSQLQKTSSVSITEIISPGRTELEIEGARADLIEVVMNIEDMLCKVQEEMARKKERGLWRSLGQWTIQQ
QKTQDEMKENIIFLKCPVPPTQELLDQKKQFEKCGLQVLKVEKIDNEVLMAAFQRKKKMMEEKLHRQPVS
HRLFQQVPYQFCNVVCRVGFQRMYSTPCDPKYGAGIYFTKNLKNLAEKAKKISAADKLIYVFEAEVLTGF
FCQGHPLNIVPPPLSPGAIDGHDSVVDNVSSPETFVIFSGMQAIPQYLWTCTQEYVQSQDYSSGPMRPFA
QHPWRGFASGSPVD
```

```
>XP_002813263.1 poly [ADP-ribose] polymerase 9 isoform X1 [Pongo abelii]
MDFSMVAGATAYNEKSETGALGENYSWQIPINHNDFNILKNNERQLCEVLQNKFGCISTLVSPVREGNSK
SLQVFRKMLTPRIELSVWKDDLTTHAVDAVVNAANEDLLHGGGLALALVKAGGFEIQEESKQFVARYGKV
SAGEIAVTGAGRLPCKHIIHAVGPRWMEWDTQGCIGKLQRAIVSILNYVIYENTHIKTVAIPALSSGIFQ
FPLNLCTTTIVETIRVNLQGRPMMSNLKEIHLVSNEDPTVAAFKAASEFILGKSELGQETTPSFNAMVVN
NLTLQIVQGHIERQTADVIVNSVNPHDITVGPVAKSILQQAGVEMKSEFLATKAKQFQRSRLVLVTKGFN
LSCRYIYHVLWHSEFPKPQVLKHAMKECLEKCIEQNITSISFPALGTGNMEIKKETAAEILFDEVLTFAK
DHVKHQLTVKFVIFPTDLEIYKAFSAEMAKRSKMLSLNNYSVPQSTREEKRENGLEARSPAINLMGFNME
EVYEAHAWIQRILSLQNHHIIENNHILYLGRKEHDILSQLQKTSSVSITEIISPGRTELEIEGAQADLIE
VVMNIEDMLCKVQEEVARKKERGLWRLLGRRTNQQQKTQDEMKENIIFLKYPAPPTQELLDQKKQFEKCG
LQVMKVEKIDNEVLMAAFQRKKKMMEEKLHRQPVSHRLFQQVPYQFCNVVCRVGFQRMYSVPCDPKYGAG
IYFTKNLKNLAEKAKKISAADKLICVFEAEVLTGSFCQGHLLNIVPPPLSPGAIDGHDSVVDNVSSPETF
VIFSGMQAIPQYLWTCTQEYVQSQDYSSGPMRPFAQHPWWGFTSGSPVD
```

```
>XP_006522818.1 protein mono-ADP-ribosyltransferase PARP9 isoform X1 [Mus musculus]
MAYYMDTWAAAPAERPGMIASLSLSFKKAFAELFPQRRRGHSEGDYPPLRGSANNSLEEHYRWQIPIKHN
VFEILKSNESQLCEVLQNKFGCISTLSCPTLAGSSSPAQRVFRRTLIPGIELSVWKDDLTRHVVDAVVNA
ANENLLHGSGLAGSLVKTGGFEIQEESKRIIANVGKISVGGIAITGAGRLPCHLIIHAVGPRWTVTNSQT
AIELLKFAIRNILDYVTKYDLRIKTVAIPALSSGIFQFPLDLCTSIILETIRLYFQDKQMFGNLREIHLV
SNEDPTVASFKSASESILGRDLSSWGGPETDPASTMTLRIGRGLTLQIVQGCIEMQTTDVIVNSGYMQDF
KSGRVAQSILRQAGVEMEKELDKVNLSTDYQEVWVTKGFKLSCQYVFHVAWHSQINKYQILKDAMKSCLE
KCLKPDINSISFPALGTGLMDLKKSTAAQIMFEEVFAFAKEHKEKTLTVKIVIFPVDVETYKIFYAEMTK
RSNELNLSGNSGALALQWSSGEQRRGGLEAGSPAINLMGVKVGEMCEAQEWIERLLVSLDHHIIENNHIL
YLGKKEHDVLSELQTSTRVSISETVSPRTATLEIKGPQADLIDAVMRIECMLCDVQEEVAGKREKNLWSL
SGQGTNQQEKLDKMEESYTFQRYPASLTQELQDRKKQFEKCGLWVVQVEQIDNKVLLAAFQEKKKMMEER
TPKGSGSQRLFQQVPHQFCNTVCRVGFHRMYSTSYNPVYGAGIYFTKSLKNLADKVKKTSSTDKLIYVFE
AEVLTGSFCQGNSSNIIPPPLSPGALDVNDSVVDNVSSPETIVVFNGMQAMPLYLWTCTQDRTFSQHPMW
SQGYSSGPGMVSSLQSWEWVLNGSSV
```

```
>XP_040531610.1 protein mono-ADP-ribosyltransferase PARP9 isoform X1 [Gallus gallus]
MGTYQSKQSETPKDESGTGPRMDEGSVMIPVNKDTYEALKKRENDLRNLVYKKFACTLTCTKRAAEVYRK
VLGEGIHLLVYKDDLTSHKADAVVNAANESLEHSGALALALLNAGGPEIAEESRNFIRKHGKVPTGKIAV
TGGGKLPCKKIIHAIGPMWYPSEKEKCCVLLEEAVVNVLKYASDPKNNIKSVAIPAVSSGVFGFPVNLCA
QVIVMSIKLFVETQPSCLKEIHLVNICEQTVAEIKRACEMILGEGSSLQETVPASLSLPVPVIKHGNIRL
RIIKGYLEKIRTTAIVSSVSSDGEFCSQISTAMLQKAGPTLQAEILSQLKHLHSSKELIVTSGYNLPSDF
VLHVLWPCFNHVVLLCEQLKEIVNRCLYFVRNYPLPSIAFPEKNWSLKLPVAIVAEIMIEEVLDFARKYP
ETKIDVQFVLHPDDDTTYQVFQEKMNLAASKLENNNRSDYLSTESSSQGRKETTYNKMAIELQGRTHFAL
KAAELWVQSLVQTQKSHSAVIENNYIFSLGKKEYTELSRVKYSNVSMSEKVKDGKASLEFHGPPDAVIDA
VLTTEKLLLFMQEQTTAKQEELLRLIGQADTDQLSEGHLDKTNTTKQFQMLQVDSHLQAFKDRQKQFERT
GLHILKIEKIHNPLLSAAFQQTKKNLEQKGVTSKGTHKLYQRVPAQFCTLVCQTGFHRIYSPPPDQRYGA
GIYFKRNPNSLIEDDGKKETESKIYVFEADVLTGLYTKGKPSYIMPPAVEGDASMLYDSLVDDINNPDTF
VICNSLQALPCYLLTCSQVKESPMDL
```

```
>XP_041434303.1 protein mono-ADP-ribosyltransferase PARP9 isoform X2 [Xenopus laevis]
MEHSITFPISEELYRPMQGIKETLTKLFAKKFSCQIEVKGPKYLALPGAQGSKLVYEKKMPGGFRVSTWK
GDLTMQDVDAVVNAANDQLEHIGGLALALANAGGPVVEEESRRHIEKNGKVKTGSIAVTSAGNLPCSWII
HAVGPKWVDYKHSQCESELKEVITSILRHVNKDSRITSVAIPAVSSGIFGFPLTLCANILVQAIENFSSQ
VKCDHLQEIRLVNNDDKTVEAMRAACEKILGRSQNWSQGIAQAANNQAAAPPYLSPSLSINGLNLFLKKG
NIEEQSTNIIVNSTSSSLDLHQGQISNAILNKAGPELQHEIRKYLPYPPTGSMLPTLGYRLPCQFVYHTI
LPFYSEWFKGDPKQILNMLILQCLTQAANIPSQGQRSISFPTLGTGALGFPIKTVAEIMIHTFLQFAYNY
PTMLDVYFVIHPKDEAYYKVFQDTVQTIQKSNNIRVSAEEPRETSSQDEKICIIIRGNSSDVQEAETFLT
QNILVPESIHIQNNHIFLLGKKEHRSLSSLHFSQAEISEVLSNGKASLEIKGSQKDLASAAVQVEMMLLD
AQEEHAEKLEDELFESAVQWMYEGRKQMDCFPGRDNRKIEEAYMSKSNTELSISGKQMSFDFINLTVKAA
GTTYRLYRECHLARDYIPRAHKGGKTVAPLFLLDLVNPSTKEFKDRAKEFMKANLELVKMVKIQNKVLSA
VYQSIKSDNKGLQVYKRVPSDFCKLIARVGFNRLYMGSGDAEYEGCICFNKQLDEVLEDPTGTKGLVCIF
QAEVAPGTLSETWNMLPTPLASDVFSLYDSYMDSIKDPKKVVIFDGHRANPQYWFICKWKGSKV
```

```
>XP_014034922.2 protein mono-ADP-ribosyltransferase PARP9 isoform X2 [Salmo salar]
MAKSDNIPVEEEMVSILGQCGPALTYALQSKFGCTAVLHGVNAASAGAGQSMKPEITFSTQLSKGLRVSV
WRDDLTTHCGVDAVVNAANEHLSHVGGLAKALSDAGGPDIQRESDRYINAHGRLLTGYAIVASPGYLPCK
KIIHVVGPSLPYKPQKDDVNIATPKLEKAVRNILEEMKKHNLKSVAIPAISSGLFNFPLPLCADVIVKTL
KQYHDHNYSGAPLEVRLVNHDDLSVGEMKRACCEILGSTVSHSQPAAAWGLPPKASYSKVVSHGNGSTSM
KVNNVSLNLKKGYIETQKTSGIVNTISSDLDLSYGAISKAILQKAGKGIQEEISKCYNYNKYGDVIETTG
HKLSCRFVYHTICPNRKQAFEKILGDVISKCLSMAQKKTLSSISFPAIGTGMLGFNKQEVAQIMVDTAVK
FAKKNNGTIMDIYYVIYPADTETYRAFEDKLKSLQGATQYSSSFNSASAQGFQPTTGYFDSRDPRESAQP
YIELSSAFDETLREARHWYFSVLYPPFKKDFTIRNNFIQHFGQHEFEKLLTFQTKFKVSIEVFFKDGCAG
MNIHGASRDVIAAVFEVEAMCCKVQEDFAKEEERDIGLRTSTSSPRNPVSESSSDYRHSRLDFSGLEIVR
VEKVENSALKQVFELKKKQLAVSTSQRMYQRLPAQYCGLLNRVGFQREFAPPDVQNYGEGIYFSGSVDGA
KKQWKRLADEVYLYFVEAQVLTGKSTHGSPGLIVPPVIPSGNPITLYDSVKGGVDTCVIFNGHQALPEYL
IICKNNHAMSSDKN
```

Alignment

```
XP_040531610.1[G.gallus]        ---------------------------------MGT---YQSKQSETPKDESGTGPRMDE    24
XP_006522818.1[M.musculus]      MAYYMDTWAAAPAERPGMIASLSLSFKKAFAELFPQRRRGHSEGDYPPLRGSANNSLEEH    60
NP_001139574.1[H.sapiens]       MDFSMVAGAAAYNEKSGRITSLSLLFQKVFAQIFPQWRKGNTEEC-LPYKCSETGALGEN    59
XP_002813263.1[P.abelii]        MDFSMVAGATAYNEK------------------------------------SETGALGEN    24
XP_041434303.1[X.laevis]        ----------------------------------------------------------ME    2
XP_014034922.2[S.salar]         ----------------------------------------------------------MA    2


XP_040531610.1[G.gallus]        GSVMIPVNKDTYEALKKRENDLRNLVYKKFACTLTCT------------KRAAEVYRKVL    72
XP_006522818.1[M.musculus]      YRWQIPIKHNVFEILKSNESQLCEVLQNKFGCISTLSCPTL----AGSSSPAQRVFRRTL    116
NP_001139574.1[H.sapiens]       YSWQIPINHNDFKILKNNERQLCEVLQNKFGCISTLVSPVQ----EGNSK-SLQVFRKML    114
XP_002813263.1[P.abelii]        YSWQIPINHNDFNILKNNERQLCEVLQNKFGCISTLVSPVR----EGNSK-SLQVFRKML    79
XP_041434303.1[X.laevis]        HSITFPISEELYRPMQGIKETLTKLFAKKFSCQIEVKGPKYLALPG--AQGSKLVYEKKM    60
XP_014034922.2[S.salar]         KSDNIPVEEEMVSILGQCGPALTYALQSKFGCTAVLHGVNAASAGAGQSMKPEITFSTQL    62
                                    :*:..:    :      *   . .**.*                      .:   :

XP_040531610.1[G.gallus]        GEGIHLLVYKDDLTS-HKADAVVNAANESLEHSGALALALLNAGGPEIAEESRNFIRKHG    131
XP_006522818.1[M.musculus]      IPGIELSVWKDDLTR-HVVDAVVNAANENLLHGSGLAGSLVKTGGFEIQEESKRIIANVG    175
NP_001139574.1[H.sapiens]       TPRIELSVWKDDLTT-HAVDAVVNAANEDLLHGGGLALALVKAGGFEIQEESKQFVARYG    173
XP_002813263.1[P.abelii]        TPRIELSVWKDDLTT-HAVDAVVNAANEDLLHGGGLALALVKAGGFEIQEESKQFVARYG    138
XP_041434303.1[X.laevis]        PGGFRVSTWKGDLTM-QDVDAVVNAANDQLEHIGGLALALANAGGPVVEEESRRHIEKNG    119
XP_014034922.2[S.salar]         SKGLRVSVWRDDLTTHCGVDAVVNAANEHLSHVGGLAKALSDAGGPDIQRESDRYINAHG    122
                                   :.: .::.***    .********: * * ..** :* .:**  : .** . :   *

XP_040531610.1[G.gallus]        KVPTGKIAVTGGGKLPCKKIIHAIGPMWYPSEKEK----CCVLLEEAVVNVLKYASDPKN    187
XP_006522818.1[M.musculus]      KISVGGIAITGAGRLPCHLIIHAVGPRWTVTNSQT----AIELLKFAIRNILDYVTKYDL    231
NP_001139574.1[H.sapiens]       KVSAGEIAVTGAGRLPCKQIIHAVGPRWMEWDKQG----CTGKLQRAIVSILNYVIYKNT    229
XP_002813263.1[P.abelii]        KVSAGEIAVTGAGRLPCKHIIHAVGPRWMEWDTQG----CIGKLQRAIVSILNYVIYENT    194
XP_041434303.1[X.laevis]        KVKTGSIAVTSAGNLPCSWIIHAVGPKWVDYKHSQ----CESELKEVITSILRHVN-KDS    174
XP_014034922.2[S.salar]         RLLTGYAIVASPGYLPCKKIIHVVGPSLPYKPQKDDVNIATPKLEKAVRNILEE-M-KKH    180
                                :: .*   ::. * ***  ***.:**       .     .   *: .: .:*      .

XP_040531610.1[G.gallus]        NIKSVAIPAVSSGVFGFPVNLCAQVIVMSIKLFVETQP--SCLKEIHLVNICEQTVAEIK    245
XP_006522818.1[M.musculus]      RIKTVAIPALSSGIFQFPLDLCTSIILETIRLYFQDKQMFGNLREIHLVSNEDPTVASFK    291
NP_001139574.1[H.sapiens]       HIKTVAIPALSSGIFQFPLNLCTKTIVETIRVSLQGKPMMSNLKEIHLVSNEDPTVAAFK    289
XP_002813263.1[P.abelii]        HIKTVAIPALSSGIFQFPLNLCTTTIVETIRVNLQGRPMMSNLKEIHLVSNEDPTVAAFK    254
XP_041434303.1[X.laevis]        RITSVAIPAVSSGIFGFPLTLCANILVQAIENFS-SQVKCDHLQEIRLVNNDDKTVEAMR    233
XP_014034922.2[S.salar]         NLKSVAIPAISSGLFNFPLPLCADVIVKTLKQYH-DHNYSGAPLEVRLVNHDDLSVGEMK    239
                                .:.:*****:***:* **: **:  :: ::.     :   .   *::**.  : :*  ::

XP_040531610.1[G.gallus]        RACEMILGEGSSL---------QETV----PASLSLPVPVIKHGNIRLRIIKGYLEKIRT    292
XP_006522818.1[M.musculus]      SASESILGRDLSS------WGGPETD----PAS---TMTLRIGRGLTLQIVQGCIEMQTT    338
NP_001139574.1[H.sapiens]       AASEFILGKSELG---------QETT----P-----SFNAMVVNNLTLQIVQGHIEWQTA    331
XP_002813263.1[P.abelii]        AASEFILGKSELG---------QETT----P-----SFNAMVVNNLTLQIVQGHIERQTA    296
XP_041434303.1[X.laevis]        AACEKILGRSQNWSQGIAQAAN------NQAAAPPYLSPSLSINGLNLFLKKGNIEEQST    287
XP_014034922.2[S.salar]         RACCEILGSTVSHSQPAAAWGLPPKASYSKVVSHGNGSTSMKVNNVSLNLKKGYIETQKT    299
                                 *.  ***                                    .: * : :* :*   :

XP_040531610.1[G.gallus]        TAIVSSVSSD-GEFCSQISTAMLQKAGPTLQAEILSQL-KHLHSSKELIVTSGYNLPSDF    350
XP_006522818.1[M.musculus]      DVIVNSGYMQ-DFKSGRVAQSILRQAGVEMEKELDKVN--LSTDYQEVWVTKGFKLSCQY    395
NP_001139574.1[H.sapiens]       DVIVNSVNPH-DITVGPVAKSILQQAGVEMKSEFLATKAKQFQRSQLVLVTKGFNLFCKY    390
XP_002813263.1[P.abelii]        DVIVNSVNPH-DITVGPVAKSILQQAGVEMKSEFLATKAKQFQRSRLVLVTKGFNLSCRY    355
XP_041434303.1[X.laevis]        NIIVNSTSSSLDLHQGQISNAILNKAGPELQHEIRKYL--PYPPTGSMLPTLGYRLPCQF    345
XP_014034922.2[S.salar]         SGIVNTISSDLDLSYGAISKAILQKAGKGIQEEISKCY--NYNKYGDVIETTGHKLSCRF    357
                                  **.:     .   . ::  ::*.:** :: *:             :  * *..* . :

XP_040531610.1[G.gallus]        VLHVLWPC-FNH-----VVLLCEQLKEIVNRCLYFVR---NYPLPSIAFPEKNW-SLKLP    400
XP_006522818.1[M.musculus]      VFHVAWHSQINK-----YQILKDAMKSCLEKCL-------KPDINSISFPALGTGLMDLK    443
NP_001139574.1[H.sapiens]       IYHVLWHSEFPK-----PQILKHAMKECLEKCI-------EQNITSISFPALGTGNMEIK    438
XP_002813263.1[P.abelii]        IYHVLWHSEFPK-----PQVLKHAMKECLEKCI-------EQNITSISFPALGTGNMEIK    403
XP_041434303.1[X.laevis]        VYHTILPFYSEWFKGDPKQILNMLI----LQCLTQAANIPSQGQRSISFPTLGTGALGFP    401
XP_014034922.2[S.salar]         VYHTICPNRKQAFE----KILGDVI----SKCLSMAQ---KKTLSSISFPAIGTGMLGFN    406
                                : *.               :*   :     :*:       .    **:**  .   : :

XP_040531610.1[G.gallus]        VAIVAEIMIEEVLDFARKYPETKIDVQFVLHPDDDTTYQVFQEKMNLAASKLENNNRSDY    460
XP_006522818.1[M.musculus]      KSTAAQIMFEEVFAFAKEHKEKTLTVKIVIFPVDVETYKIFYAEMTKRSNELNLSGNSGA    503
NP_001139574.1[H.sapiens]       KETAAEILFDEVLTFAKDHVKHQLTVKFVIFPTDLEIYKAFSSEMAKRSKMLSLNNYSV-    497
XP_002813263.1[P.abelii]        KETAAEILFDEVLTFAKDHVKHQLTVKFVIFPTDLEIYKAFSAEMAKRSKMLSLNNYSV-    462
XP_041434303.1[X.laevis]        IKTVAEIMIHTFLQFAY-NYPTMLDVYFVIHPKDEAYYKVFQDTVQTIQKSNNIRVSAEE    460
XP_014034922.2[S.salar]         KQEVAQIMVDTAVKFAKKNNGTIMDIYYVIYPADTETYRAFEDKLKSLQGATQYSSSFNS    466
                                   .*:*:..  . **       : :  *:.* *   *: *   :       .

XP_040531610.1[G.gallus]        LSTESSSQG----RKE--TTYNKMAIELQGRTHFALKAAELWVQSLVQTQKSHSAVIENN    514
XP_006522818.1[M.musculus]      LALQWSSGE----QRRGGLEAGSPAINLMGVKVGEMCEAQEWIERLLVSLDH--HIIENN    557
NP_001139574.1[H.sapiens]       --PQSTREE----KRENGLEARSPAINLMGFNVEEMYEAHAWIQRILSLQNH--HIIENN    549
XP_002813263.1[P.abelii]        --PQSTREE----KRENGLEARSPAINLMGFNMEEVYEAHAWIQRILSLQNH--HIIENN    514
XP_041434303.1[X.laevis]        PRETSSQDEKI-------------CI-IIRGNSSDVQEAETFLTQNILV--PESIHIQNN    504
XP_014034922.2[S.salar]         ASAQGFQPTTGYFDSRDPRESAQPYIELSSAFDETLREARHWYFSVLYPPFKKDFTIRNN    526
                                                         * :       :  *. :    :         *.**

XP_040531610.1[G.gallus]        YIFSLGKKEYTELSRVK-YSNVSMSEKVKDGKASLEFHGPPDAVIDAVLTTEKLLLFMQE    573
XP_006522818.1[M.musculus]      HILYLGKKEHDVLSELQTSTRVSISETVSPRTATLEIKGPQADLIDAVMRIECMLCDVQE    617
NP_001139574.1[H.sapiens]       HILYLGRKEHDILSQLQKTSSVSITEIISPGRTELEIEGARADLIEVVMNIEDMLCKVQE    609
XP_002813263.1[P.abelii]        HILYLGRKEHDILSQLQKTSSVSITEIISPGRTELEIEGAQADLIEVVMNIEDMLCKVQE    574
XP_041434303.1[X.laevis]        HIFLLGKKEHRSLSSLHF-SQAEISEVLSNGKASLEIKGSQKDLASAAVQVEMMLLDAQE    563
XP_014034922.2[S.salar]         FIQHFGQHEFEKLLTFQTKFKVSIEVFFKDGCAGMNIHGASRDVIAAVFEVEAMCCKVQE    586
                                .*  :*::*.  *  .:    ..:   ..   : :::.*    :  ...  * :    **

XP_040531610.1[G.gallus]        QTTAKQE-ELLRLIGQADTDQLSE--------------GHLDKT----------------    602
XP_006522818.1[M.musculus]      EVAGKREKNLWSLSGQGTNQQ-E----------------KLDKM----------------    644
NP_001139574.1[H.sapiens]       EMARKKERGLWRSLGQWTIQQQK----------------TQDEM----------------    637
XP_002813263.1[P.abelii]        EVARKKERGLWRLLGRRTNQQQK----------------TQDEM----------------    602
XP_041434303.1[X.laevis]        EHAEKLEDELFESAVQWMYEGRKQMDCFPGRDNRKIEEAYMSKSNTELSISGKQMSFDFI    623
XP_014034922.2[S.salar]         DFAKEEERDI--------------------------GLRT-STS----------------    603
                                : : : *  :                               .

XP_040531610.1[G.gallus]        --------------------------------NTTKQFQMLQVDSHLQAFKDRQKQFERT    630
XP_006522818.1[M.musculus]      --------------------------------EESYTFQRY-PASLTQELQDRKKQFEKC    671
NP_001139574.1[H.sapiens]       --------------------------------KENIIFLKC-PVPPTQELLDQKKQFEKC    664
XP_002813263.1[P.abelii]        --------------------------------KENIIFLKY-PAPPTQELLDQKKQFEKC    629
XP_041434303.1[X.laevis]        NLTVKAAGTTYRLYRECHLARDYIPRAHKGGKTVAPLFLLDLVNPSTKEFKDRAKEFMKA    683
XP_014034922.2[S.salar]         ----------------------------------SP---RNPVSESSSDYRHSR--LDFS    624
                                                                               .   .    :

XP_040531610.1[G.gallus]        GLHILKIEKIHNPLLSAAFQQTKKNLEQKGVTSKGTHKLYQRVPAQFCTLVCQTGFHRIY    690
XP_006522818.1[M.musculus]      GLWVVQVEQIDNKVLLAAFQEKKKMMEERTPKGSGSQRLFQQVPHQFCNTVCRVGFHRMY    731
NP_001139574.1[H.sapiens]       GLQVLKVEKIDNEVLMAAFQRKKKMMEEKLHRQPVSHRLFQQVPYQFCNVVCRVGFQRMY    724
XP_002813263.1[P.abelii]        GLQVMKVEKIDNEVLMAAFQRKKKMMEEKLHRQPVSHRLFQQVPYQFCNVVCRVGFQRMY    689
XP_041434303.1[X.laevis]        NLELVKMVKIQNKVLSAVYQSIK---SD-----NKGLQVYKRVPSDFCKLIARVGFNRLY    735
XP_014034922.2[S.salar]         GLEIVRVEKVENSALKQVFELKKKQLAV-----STSQRMYQRLPAQYCGLLNRVGFQREF    679
                                .* :::: ::.*  *  .::  *              :::::* ::*  : :.**:* :
```

```
XP_040531610.1[G.gallus]        SPPPDQRYGAGIYFKRNPNSLIEDDGKK-ETESKIYVFEADVLTGLYTKGKPSYIMPP-A    748
XP_006522818.1[M.musculus]      STSYNPVYGAGIYFTKSLKNLADKVKKTSSTDKLIYVFEAEVLTGSFCQGNSSNIIPP-P    790
NP_001139574.1[H.sapiens]       STPCDPKYGAGIYFTKNLKNLAEKAKKISAADKLIYVFEAEVLTGFFCQGHPLNIVPP-P    783
XP_002813263.1[P.abelii]        SVPCDPKYGAGIYFTKNLKNLAEKAKKISAADKLICVFEAEVLTGSFCQGHLLNIVPP-P    748
XP_041434303.1[X.laevis]        MGSGDAEYEGCICFNKQLDEVLEDPTGT-KGLVCIF--QAEVAPGTLSET---WNMLPTP    789
XP_014034922.2[S.salar]         APPDVQNYGEGIYFSGSVDGAKKQWKRL-ADEVYLYFVEAQVLTGKSTHGSPGLIVPPVI    738
                                       *   * *. . .   ..          :   :*:*  *   .      : *

XP_040531610.1[G.gallus]        VEGDASMLYDSLVDDINNPDTFVICNSLQALPCYLLTCSQVKESPMDL------------    796
XP_006522818.1[M.musculus]      LSPGALDVNDSVVDNVSSPETIVVFNGMQAMPLYLWTCTQDRTFSQHPMWSQGYSSGPGM    850
NP_001139574.1[H.sapiens]       LSPGAIDGHDSVVDNVSSPETFVIFSGMQAIPQYLWTCTQEYVQ------SQDYSSGPMR    837
XP_002813263.1[P.abelii]        LSPGAIDGHDSVVDNVSSPETFVIFSGMQAIPQYLWTCTQEYVQ------SQDYSSGPMR    802
XP_041434303.1[X.laevis]        LASDVFSLYDSYMDSIKDPKKVVIFDGHRANPQYWFICKWKGSKV---------------    834
XP_014034922.2[S.salar]         PSGNPITLYDSVKG---GVDTCVIFNGHQALPEYLIICKNNHAMSSDKN-----------    784
                                   .     **  .   . .. *: .. :* * *   *.

XP_040531610.1[G.gallus]        -----------------          796
XP_006522818.1[M.musculus]      VSSLQSWEWVLNGSSV-          866
NP_001139574.1[H.sapiens]       PFAQHPWRGFASGSPVD          854
XP_002813263.1[P.abelii]        PFAQHPWWGFTSGSPVD          819
XP_041434303.1[X.laevis]        -----------------          834
XP_014034922.2[S.salar]         -----------------          784
```

Percent identity matrix:

```
#
#
#  Percent Identity  Matrix - created by Clustal2.1
#
#

     1: XP_040531610.1[G.gallus]    100.00   37.60   39.84   39.92   36.29   34.29
     2: XP_006522818.1[M.musculus]   37.60  100.00   61.76   62.45   34.83   35.22
     3: NP_001139574.1[H.sapiens]    39.84   61.76  100.00   95.97   36.36   34.93
     4: XP_002813263.1[P.abelii]     39.92   62.45   95.97  100.00   35.95   34.52
     5: XP_041434303.1[X.laevis]     36.29   34.83   36.36   35.95  100.00   34.14
     6: XP_014034922.2[S.salar]      34.29   35.22   34.93   34.52   34.14  100.00
```
