# Supplementary material for: An Evolutionary Perspective on the Origin, Conservation and Binding Partner Acquisition of Tankyrases
Source: Biomolecules. 2022 Nov 15;12(11):1688. doi: 10.3390/biom12111688 (PMC9688111; doi:10.3390/biom12111688)
Supplement: Supplementary file 1 [file biomolecules-12-01688-s001.zip › biomolecules-1913719-supplementary-11.17 -done/Data_2/Seryl-tRNA-synthetase.pdf]

Seryl-tRNA synthase

Sequences:

>NP_006504.2 serine--tRNA ligase, cytoplasmic isoform a [Homo sapiens]
MVLDLDLFRVDKGGDPALIRETQEKRFKDPGLVDQLVKADSEWRRCRFRADNLNKLKNLCSKTIGEKMKK
KEPVGDDESVPENVLSFDDLTADALANLKVSQIKKVRLLIDEAILKCDAERIKLEAERFENLREIGNLLH
PSVPISNDEDVDNKVERIWGDCTVRKKYSHVDLVVMVDGFEGEKGAVVAGSRGYFLKGVLVFLEQALIQY
ALRTLGSRGYIPIYTPFFMRKEVMQEVAQLSQFDEELYKVIGKGSEKSDDNSYDEKYLIATSEQPIAALH
RDEWLRPEDLPIKYAGLSTCFRQEVGSHGRDTRGIFRVHQFEKIEQFVYSSPHDNKSWEMFEEMITTAEE
FYQSLGIPYHIVNIVSGSLNHAASKKLDLEAWFPGSGAFRELVSCSNCTDYQARRLRIRYGQTKKMMDKV
EFVHMLNATMCATTRTICAILENYQTEKGITVPEKLKEFMPPGLQELIPFVKPAPIEQEPSKKQKKQHEG
SKKKAAARDVTLENRLQNMEVTDA

>NP_001125910.1 serine--tRNA ligase, cytoplasmic [Pongo abelii]
MVLDLDLFRVDKGGDPALIRETQEKRFKDPGLVDQLVKADSEWRRCRFRADNLNKLKNLCSKTIGEKMKK
KEPVGDDESVPENVLSFDDLTADALANLKVSQIKKVRLLIDEAILKCDAERIKLEAERFENLREIGNLLH
PSVPISNDEDVDNKVERIWGDCTVRKKYSHVDLVVMVDGFEGEKGAVVAGSRGYFLKGVLVFLEQALIQY
ALRTLGSRGYIPIYTPFFMRKEVMQEVAQLSQFDEELYKVIGKGSEKSDDNSYDEKYLIATSEQPIAALH
RDEWLRPEDLPIKYAGLSTCFRQEVGSHGRDTRGIFRVHQFEKIEQFVYSSPHDNKSWEMFEEMITTAEE
FYQSLGIPYHIVNIVSGSLNHAASKKLDLEAWFPGSGAFRELVSCSNCTDYQARRLRIRYGQTKKMMDKV
EFVHMLNATMCATTRTICAILENYQTEKGITVPEKLKEFMPPGLQELIPFVKPAPIEQEPSKKQKKQHEG
SKKKAAARDATLENRLQNMEVTDA

>NP_001191908.1 serine--tRNA ligase, cytoplasmic isoform 2 [Mus musculus]
MVLDLDLFRVDKGGDPALIRETQEKRFKDPGLVDQLVKADSEWRRCRFRADNLNKLKNLCSKTIGEKMKK
KEAVGDDESVPENVLNFDDLTADALAALKVSQIKKVRLLIDEAIQKCDGERVKLEAERFENLREIGNLLH
PSVPISNDEDADNKVERIWGDCTVRKKYSHVDLVVMVDGFEGEKGAVVAGSRGYFLKGPLVFLEQALIQY
ALRTLGSRGYTPIYTPFFMRKEVMQEVAQLSQFDEELYKVIGKGSEKSDDNSYDEKYLIATSEQPIAALH
RDEWLRPEDLPIKYAGLSTCFRQEVGSHGRDTRGIFRVHQFEKIEQFVYSSPHDNKSWEMFDEMIATAEE
FYQSLGIPYHIVNIVSGSLNHAASKKLDLEAWFPGSGAFRELVSCSNCTDYQARRLRIRYGQTKKMMDKV
EFVHMLNATMCATTRTICAILENYQAEKGIAVPEKLREFMPPGLQELIPFVKPAPIDQEPSKKQKKQHEG
SKKKAKEVPLENQLQSMEVTEA

>NP_001026563.1 serine--tRNA ligase, cytoplasmic [Gallus gallus]
MVLDLDLFRADKGGDPAAVREMQRKRFKDPALVDALVRADGAWRRCRFRADNLNKLKNLCSKTIGDKMKK
KEPVGSDESVPESAQNLDELTADVLGGLQVSQIKKVRLLIDEAILECDAERVRLEAERFESLREIGNLLH
PSVPISNDEDADNKVERIWGDCSCRKKYSHVDLVVMVDGYEGEKGAVVAGSRGYFLKGPLVFLEQALIQY
ALQSLRAKGYTPVYTPFFMRKEVMQEVAQLSQFDEELYKVIGKGSEKAEDSSVDEKYLIATSEQPIAALH
RDEWLKPEDLPIKYAGLSTCFRQEVGSHGRDTRGIFRVHQFEKIEQFVYASPHDNKSWEMFDEMIATAEE
FYQSLGIPYHIVNIVSGALNHAASKKLDLEAWFPGSGAFRELVSCSNCTDYQARRLRIRFGQTKKMMDKV
EFVHMLNATMCATTRTICAILENYQTEEGIVVPERLRDFMPPDLRQIIRFVKPAPIEQELSKKQKKQQEG
GRKKAAGGERVLEEQMQNMGVSSA

>XP_018104878.1 serine--tRNA ligase, cytoplasmic [Xenopus laevis]
MVLDLDLFREDKGGNPELVRETQRKRFKDPGLVDTLLNSDTAWRKSRFQADNLNKQKNLCSKITGEKMKK
KEPLGDSDVLPENIQLDQLTAEVLSALSVTQIKRLRVLIDEAIAATDAERIKLEAERFESLREIGNLLHP
TVPISNDEDNDNKVERTWGDCEIQKKYSHVDLVVMVDGFEGEKGAVVAGSRGYFLKGPLVFLEQALIQFA
LHTLSEKGYTPIYTPFFMRKEVMQEVAQLSQFDEELYKVIGKGSEKSDDNSVDEKYLIATSEQPIAALHR
DEWLKPEDLPVRYAGISTCFRQEVGSHGRDTRGIFRVHQFEKIEQFIYASPHDNKSWEMFEEMIMTAESF
YQMLGIPYRIVNIVSGSLNHAASKKLDLEAWFPGSGAFRELVSCSNCTDYQARRLRIRYGQTKKMMDKVD
FVHMLNATMCATTRAICAILENYQTEEGIIVPEKLRSFMPPGLKDIIKFVKPAPIDQELTKKQKKQQQEK
GKKTENCGLDTQMESMKVHSA

>XP_013991973.1 serine--tRNA ligase, cytoplasmic-like [Salmo salar]
MVLDLDQFRADKGGDPEVIRETQRKRFKDVSLVDKLVHADTEWRKCRFTADNLNKAKNLCSKTVGEKMKK
KEPIGEDDSLPDDAQNLEALTADTLAPLTVTQIKKVRLLVDEAVQKSDSERVKLEEERFQYLREIGNLLH
PSVPISNDEDADNKVERTWGDCTVQKKYSHVDLVVMIDGYDGEKGAIVAGSRGYFLKGPLVFLEQALINY
ALRMLHSKNYQMLYTPFFMRKEVMQEVAQLSQFDDELYKVIGKSSEKSEDTAIDEKYLIATSEQPIAAFL
RDEWLKPEELPIRYAGLSTCFRQEVGSHGRDTRGIFRVHQFEKIEQFVFASPHDNKSWEMMDEMIGTAEE
FYQTLGIPYRIVNIVSGALNHAASKKLDLEAWFPGSAAFRELVSCSNCLDYQARRLRIRYGQTKKMMDKA
DYVHMLNATMCATTRVMCAILETYQTEEGIIIPEVLRNFMPPGMTEMLKFVKPAPIDVEMSKKQKKQQDG
GKKKQGSGDQLQNQVDNMSVHDS

>XP_032819422.1 serine--tRNA ligase, cytoplasmic [Petromyzon marinus]
MVLDLDLFRADKGGDPTRVRENQARRFKDVCLVDTLVQADTQWRACRYRADNLNKLKNLCSKTIGDKMKK
KEDPGQDETLPEGTPGLDELTADTLAALSVAQIKVLRLQIDNAIALCDTERLRLEAVRLDSLREVGNILH
PSVPVSNDEDADNKVERTWGDVTVRKRYSHVDLVVMVDGYEGEKAAIVSGSRAYFLKGPLVFLEQALIQL
ALQRLYSQGYTPLYPPFFMRKEVMQEVAQLSQFDEELYKVVGKGSEHADDSSVDEKYLIATSEQPIAAFH
RDEWLKPEQLPMRYAGLSSCFRQEVGSHGRDTRGIFRVHQFEKIEQFVYTSPHDNKSWEMFDEMVKTAEG
FYQELGIPYRIVNIVSGALNHAAAKKLDLEAWFPGSAAFRELVSCSNCTDYQARRLRVRYGQTKKMMDKA
EFVHMLNATMCATTRTICAILETYQTETGVVVPEALRPFMPPGLKEELKFVKSAPIDQEQIKKQKRGKGD
GLAANLGNMAVNDS

>XP_019634257.1 PREDICTED: serine--tRNA ligase, cytoplasmic-like [Branchiostoma belcheri]
MVLDLDLFRVDRGGDPNKIRENQSKRYKDVTLVDNVVQADEQWRKLRYQADTLNRLKNLCSKTIGEKMKK
KEPVGEESESPPTEDELEKLTPEALQAMTVNQIKQIRTIIDKSILENTAGRAEVEKLRQENLKEIGNILH
ADVPISNDEDEDNRTERTNGDCTVRKKYSHVDLITMIDGYDGERGAVTAGSRGYYMKGIAVFLEQALIQY
ALRILYEKGYTALYTPFFMNKGVMSEVAQLSQFDEELYKVIGKGSEKGDDQTEDVKYLIATSEQPIAAFH
RDEWMKPEDLPIKYAGISTCFRQEVGSHGRDTRGIFRVHQFEKVEQFVLSKPDDSWGLFHEMIGNAEEFY
KTLGIPYRIVNIVSGALNNAAAMKYDLEAWFPGSGAFRELVSCSNCLDYQARRLLVRYGQTKKMNEKPEF
VHMLNATMCATTRVICAILENHQTEEGIVIPEVLRQYMPGGTEFITFVKPAPIEEQATKKKKAGKKKEKV
KPTQDGPEAEKVTEQVQNMAVNEG

>NP_001259982.1 Seryl-tRNA synthetase, isoform B [Drosophila melanogaster]
MVLDLDLFRSDKGGNPDLVRENQKKRFKDVALVETVIAKDTEWRQCRHRADNLNKVKNVCSKVIGEKMKK
KEPVGAMSEDLPADVTKDLTEIVAETLQPLTVNQIKQLRVLIDDAMTENQKSLELAEQTRNTSLREVGNH
LHESVPVSNDEDENRVERTFGDCEKRGKYSHVDLIVMIDGMNAEKGAVVSGGRGYFLTGAAVFLEQALIQ
HALHLLYARDYVPLYTPFFMRKEVMQEVAQLSQFDEELYKVVGKGSEKAEEVGIDEKYLIATSEQPIAAY
HRDEWLPESSLPIKYAGLSTCFRQEVGSHGRDTRGIFRVHQFEKVEQFVLTSPHDNKSWEMMDEMIGNAE
QFCQSLGIPYRVVNIVSGALNHAASKKLDLEAWFGGSGAYRELVSCSNCLDYQARRLLVRFGQTKKMNAA
VDYVHMLNATMCAATRVICAILETHQTETGIKVPEPLKKYMPAKFQDEIPFVKPAPIDLELAAAEKQKGK
KEKTKKDPAAG

>XP_001639063.2 serine--tRNA ligase, cytoplasmic [Nematostella vectensis]
MKFFSPSLHQLRTVRISFVFRRILSSSSSKMVLDLDLYRADKGGNPDKIRENQSKRYSDVTLVDKVVEAD
TQWRKFRFSKDNWNKLRNLCSKQIGDKMKKKEPVGDDETLPKEVLDGLEELTAEMLQQLTVTQIKIVRTL
VDDGMAKCNEEVARLEKLRNEKLFEVGNILHDSVRISNDEDENPVDRIVGDCSVRKKYSHVDLVHMVDGV
DIDRGSVVAGSRGYFLKGPLVFLEHALIQHATRMLYGKGFIPLSTPFFMRKEVMQEVAQLSQFDEELYKV
VGKGADEAGYEEKYLIATSEQPIAAFHRGEWMDKNDLPKKYAGFSTCFRQEAGAHGRDTRGIFRVHQFEK
IEQFVICSPHDNISWEIFDEMIGNAEEFHKSLGIPYRIVSIVSGALNNAAAKKLDLEAWFPASGAFRELV
SCSNCTDYQARRLQVRFGQTKKMGVSTEYVHMLNATMCAVSRVICAILENNQTEEGVVMPQALHPYLPQD
LHFLKFVKPAPIDDKPEQKKQKKKGKNKGANNESVGCLESKLEDVNL

>XP_047126420.1 serine--tRNA ligase, cytoplasmic [Hydra vulgaris]
MLQCHQLKRLHQPNNSLKKLRFSTSSLKMVLDLELFRSDKGCCPEKIRENQSKRFKDVTLVDKVVEADEK

```
WRKLRFQADNWNKLKNLCSKTIGLKMKNKEPEGESDLLPDSLSNIEELQALNPDTLEKFTVLQIKRIRGL
IDDYINKCESERVGCEKVRHECLFEVGNWLHDSVVVSNDEEENEIVRVVGDCTTKKKFSHVDLLAMIDGV
DYERGANVAGNRGYFLKGPGVLLQQALINLALQTLVGSGYTPIYTPFFMRKEVMQEVAQLSQFDEELYKV
VGKGSERDDDTAVEEKYLIATSEQPIAAFHRGEWMSTSDLPKKYAGISSCFRQEVGCHGRDTRGIFRVHQ
FEKVEQFVICSPHDNESWKHLDEMVNNAEKFYQALNIPYRIVGIVSGALNNAAAKKLDLEAWFPGSGAFR
ELCSCSNCTDYQARRLLVRYGQTKKMNDKTEFCHMLNATMCASTRVICCILENNQTEEGIIVPELLRPFM
GNVEFIKYVKTAPIEGNKDVKGKSNKKKPEKIEENLKNLDVKE

>XP_003383114.1 PREDICTED: serine--tRNA ligase, cytoplasmic-like isoform X2 [Amphimedon queenslandica]
MPLDLLLFRADQGGNPDLMRDMQKKRFKDVIHVDKVVEMDTQWRKLRFESDNWNKLKNHCSKTIGEKMKK
KEPIGESADVPESLVTKLDSITPDEIKSLTVTQIKRLQQLIEEASDKNSTKRKELEETRSFHLAQIGNSL
HPSVPISDNEDNNRVERTFGDASIRKKYSHMDLVVMVDGVDMQRGTTVAGNRCYYLKGPLVFLEQALIQF
ALSTLSEKSFVPLYTPFFMKKEVMQEVAHLSDYEEMLYKVIGKSSEIVDDSNVEEKYLIATSEQPIAAFH
RGDWLDPQTLPLKYAGLSSCFRQEVDSHGRDTRGIFRVHQFQKVEQFCLTSPHDDVSWKMFDEMISNAEF
FYQQLGIPYRVVNIVSGELNLAAAMKYDLEGLFPGSGAYRELVSCSNCLDYQVRRLQVRFGLTKKMNAQA
DYVHMLNATMCATTRTICAILENNQTEEGVVVPEILRQYMPPAYKEFIKFVQAAPIHEPITKKQENQMAG
MEKDNEAQS
```

Alignment

```
XP_047126420.1[H.vulgaris]           ----MLQCHQLKRLHQPNNSLKKLRFSTSSLKMVLDLELFRSDKGCCPEKIRENQSKRFK            56
XP_003383114.1[A.queenslandica]      --------------------------------MPLDLLLFRADQGGNPDLMRDMQKKRFK            28
XP_001639063.2[N.vectensis]          MKFFSPSLHQLRTVRI--SFVFRRILSSSSSKMVLDLDLYRADKGGNPDKIRENQSKRYS            58
XP_019634257.1[B.belcheri]           --------------------------------MVLDLDLFRVDRGGDPNKIRENQSKRYK            28
NP_001259982.1[D.melanogaster]       --------------------------------MVLDLDLFRSDKGGNPDLVRENQKKRFK            28
XP_013991973.1[S.salar]              --------------------------------MVLDLDQFRADKGGDPEVIRETQRKRFK            28
XP_032819422.1[P.marinus]            --------------------------------MVLDLDLFRADKGGDPTRVRENQARRFK            28
XP_018104878.1[X.laevis]             --------------------------------MVLDLDLFREDKGGNPELVRETQRKRFK            28
NP_001026563.1[G.gallus]             --------------------------------MVLDLDLFRADKGGDPAAVREMQRKRFK            28
NP_001191908.1[M.musculus]           --------------------------------MVLDLDLFRVDKGGDPALIRETQEKRFK            28
NP_006504.2[H.sapiens]               --------------------------------MVLDLDLFRVDKGGDPALIRETQEKRFK            28
NP_001125910.1[P.abelii]             --------------------------------MVLDLDLFRVDKGGDPALIRETQEKRFK            28
                                                                     * ***  :* *:*  *  :*: * :*:.

XP_047126420.1[H.vulgaris]           DVTLVDKVVEADEKWRKLRFQADNWNKLKNLCSKTIGLKMKNKEPEGESD-LLPDSLSNI            115
XP_003383114.1[A.queenslandica]      DVIHVDKVVEMDTQWRKLRFESDNWNKLKNHCSKTIGEKMKKKEPIGESA-DVPES--LV            85
XP_001639063.2[N.vectensis]          DVTLVDKVVEADTQWRKFRFSKDNWNKLRNLCSKQIGDKMKKKEPVGDDE-TLPKE--VL            115
XP_019634257.1[B.belcheri]           DVTLVDNVVQADEQWRKLRYQADTLNRLKNLCSKTIGEKMKKKEPVGEESESPPTE----            84
NP_001259982.1[D.melanogaster]       DVALVETVIAKDTEWRQCRHRADNLNKVKNVCSKVIGEKMKKKEPVGAMSEDLPAD--VT            86
XP_013991973.1[S.salar]              DVSLVDKLVHADTEWRKCRFTADNLNKAKNLCSKTVGEKMKKKEPIGEDD-SLP-D--DA            84
XP_032819422.1[P.marinus]            DVCLVDTLVQADTQWRACRYRADNLNKLKNLCSKTIGDKMKKKEDPGQDE-TLP-E--GT            84
XP_018104878.1[X.laevis]             DPGLVDTLLNSDTAWRKSRFQADNLNKQKNLCSKITGEKMKKKEPLGDSD-VLP-E--NI            84
NP_001026563.1[G.gallus]             DPALVDALVRADGAWRRCRFRADNLNKLKNLCSKTIGDKMKKKEPVGSDE-SVP-E--SA            84
NP_001191908.1[M.musculus]           DPGLVDQLVKADSEWRRCRFRADNLNKLKNLCSKTIGEKMKKKEAVGDDE-SVP-E--NV            84
NP_006504.2[H.sapiens]               DPGLVDQLVKADSEWRRCRFRADNLNKLKNLCSKTIGEKMKKKEPVGDDE-SVP-E--NV            84
NP_001125910.1[P.abelii]             DPGLVDQLVKADSEWRRCRFRADNLNKLKNLCSKTIGEKMKKKEPVGDDE-SVP-E--NV            84
                                     *   *: ::  *  **  *.  *. *: :* ***  * ***:**  *      * .

XP_047126420.1[H.vulgaris]           EELQALNPDTLEKFTVLQIKRIRGLIDDYINKCESERVGCEKVRHECLFEVGNWLHDSVV            175
XP_003383114.1[A.queenslandica]      TKLDSITPDEIKSLTVTQIKRLQQLIEEASDKNSTKRKELEETRSFHLAQIGNSLHPSVP            145
XP_001639063.2[N.vectensis]          DGLEELTAEMLQQLTVTQIKIVRTLVDDGMAKCNEEVARLEKLRNEKLFEVGNILHDSVR            175
XP_019634257.1[B.belcheri]           DELEKLTPEALQAMTVNQIKQIRTIIDKSILENTAGRAEVEKLRQENLKEIGNILHADVP            144
NP_001259982.1[D.melanogaster]       KDLTEIVAETLQPLTVNQIKQLRVLIDDAMTENQKSLELAEQTRNTSLREVGNHLHESVP            146
XP_013991973.1[S.salar]              QNLEALTADTLAPLTVTQIKKVRLLVDEAVQKSDSERVKLEEERFQYLREIGNLLHPSVP            144
XP_032819422.1[P.marinus]            PGLDELTADTLAALSVAQIKVLRLQIDNAIALCDTERLRLEAVRLDSLREVGNILHPSVP            144
XP_018104878.1[X.laevis]             -QLDQLTAEVLSALSVTQIKRLRVLIDEAIAATDAERIKLEAERFESLREIGNLLHPTVP            143
NP_001026563.1[G.gallus]             QNLDELTADVLGGLQVSQIKKVRLLIDEAILECDAERVRLEAERFESLREIGNLLHPSVP            144
NP_001191908.1[M.musculus]           LNFDDLTADALAALKVSQIKKVRLLIDEAIQKCDGERVKLEAERFENLREIGNLLHPSVP            144
NP_006504.2[H.sapiens]               LSFDDLTADALANLKVSQIKKVRLLIDEAILKCDAERIKLEAERFENLREIGNLLHPSVP            144
NP_001125910.1[P.abelii]             LSFDDLTADALANLKVSQIKKVRLLIDEAILKCDAERIKLEAERFENLREIGNLLHPSVP            144
                                       :  :  : :  : * *** ::  ::.            *  *   * ::** **  *

XP_047126420.1[H.vulgaris]           VSNDEE-ENEIVRVVGDCTTKKKFSHVDLLAMIDGVDYERGANVAGNRGYFLKGPGVLLQ            234
XP_003383114.1[A.queenslandica]      ISDNED-NNRVERTFGDASIRKKYSHMDLVVMVDGVDMQRGTTVAGNRCYYLKGPLVFLE            204
XP_001639063.2[N.vectensis]          ISNDED-ENPVDRIVGDCSVRKKYSHVDLVHMVDGVDIDRGSVVAGSRGYFLKGPLVFLE            234
XP_019634257.1[B.belcheri]           ISNDEDEDNRTERTNGDCTVRKKYSHVDLITMIDGYDGERGAVTAGSRGYYMKGIAVFLE            204
NP_001259982.1[D.melanogaster]       VSNDED-ENRVERTFGDCEKRGKYSHVDLIVMIDGMNAEKGAVVSGGRGYFLTGAAVFLE            205
XP_013991973.1[S.salar]              ISNDEDADNKVERTWGDCTVQKKYSHVDLVVMIDGYDGEKGAIVAGSRGYFLKGPLVFLE            204
XP_032819422.1[P.marinus]            VSNDEDADNKVERTWGDVTVRKRYSHVDLVVMVDGYEGEKAAIVSGSRAYFLKGPLVFLE            204
XP_018104878.1[X.laevis]             ISNDEDNDNKVERTWGDCEIQKKYSHVDLVVMVDGFEGEKGAVVAGSRGYFLKGPLVFLE            203
NP_001026563.1[G.gallus]             ISNDEDADNKVERIWGDCSCRKKYSHVDLVVMVDGYEGEKGAVVAGSRGYFLKGPLVFLE            204
NP_001191908.1[M.musculus]           ISNDEDADNKVERIWGDCTVRKKYSHVDLVVMVDGFEGEKGAVVAGSRGYFLKGPLVFLE            204
NP_006504.2[H.sapiens]               ISNDEDVDNKVERIWGDCTVRKKYSHVDLVVMVDGFEGEKGAVVAGSRGYFLKGVLVFLE            204
NP_001125910.1[P.abelii]             ISNDEDVDNKVERIWGDCTVRKKYSHVDLVVMVDGFEGEKGAVVAGSRGYFLKGVLVFLE            204
                                     :*::*: :*   *  **   : ::**:**: *:** : ::.: .:*.* *::.*  *:*:

XP_047126420.1[H.vulgaris]           QALINLALQTLVGSGYTPIYTPFFMRKEVMQEVAQLSQFDEELYKVVGKGSERDDDTAVE            294
XP_003383114.1[A.queenslandica]      QALIQFALSTLSEKSFVPLYTPFFMKKEVMQEVAHLSDYEEMLYKVIGKSSEIVDDSNVE            264
XP_001639063.2[N.vectensis]          HALIQHATRMLYGKGFIPLSTPFFMRKEVMQEVAQLSQFDEELYKVVGKGAD---EAGYE            291
XP_019634257.1[B.belcheri]           QALIQYALRILYEKGYTALYTPFFMNKGVMSEVAQLSQFDEELYKVIGKGSEKGDDQTED            264
NP_001259982.1[D.melanogaster]       QALIQHALHLLYARDYVPLYTPFFMRKEVMQEVAQLSQFDEELYKVVGKGSEKAEEVGID            265
XP_013991973.1[S.salar]              QALINYALRMLHSKNYQMLYTPFFMRKEVMQEVAQLSQFDDELYKVIGKSSEKSEDTAID            264
XP_032819422.1[P.marinus]            QALIQLALQRLYSQGYTPLYPPFFMRKEVMQEVAQLSQFDEELYKVVGKGSEHADDSSVD            264
XP_018104878.1[X.laevis]             QALIQFALHTLSEKGYTPIYTPFFMRKEVMQEVAQLSQFDEELYKVIGKGSEKSDDNSVD            263
NP_001026563.1[G.gallus]             QALIQYALQSLRAKGYTPVYTPFFMRKEVMQEVAQLSQFDEELYKVIGKGSEKAEDSSVD            264
NP_001191908.1[M.musculus]           QALIQYALRTLGSRGYTPIYTPFFMRKEVMQEVAQLSQFDEELYKVIGKGSEKSDDNSYD            264
NP_006504.2[H.sapiens]               QALIQYALRTLGSRGYIPIYTPFFMRKEVMQEVAQLSQFDEELYKVIGKGSEKSDDNSYD            264
NP_001125910.1[P.abelii]             QALIQYALRTLGSRGYIPIYTPFFMRKEVMQEVAQLSQFDEELYKVIGKGSEKSDDNSYD            264
                                     :***: *   *   .:  :  ****.* **.***:**:::: ****:**.::   :   :

XP_047126420.1[H.vulgaris]           EKYLIATSEQPIAAFHRGEWMSTSDLPKKYAGISSCFRQEVGCHGRDTRGIFRVHQFEKV            354
XP_003383114.1[A.queenslandica]      EKYLIATSEQPIAAFHRGDWLDPQTLPLKYAGLSSCFRQEVDSHGRDTRGIFRVHQFQKV            324
XP_001639063.2[N.vectensis]          EKYLIATSEQPIAAFHRGEWMDKNDLPKKYAGFSTCFRQEAGAHGRDTRGIFRVHQFEKI            351
XP_019634257.1[B.belcheri]           VKYLIATSEQPIAAFHRDEWMKPEDLPIKYAGISTCFRQEVGSHGRDTRGIFRVHQFEKV            324
NP_001259982.1[D.melanogaster]       EKYLIATSEQPIAAYHRDEWLPESSLPIKYAGLSTCFRQEVGSHGRDTRGIFRVHQFEKV            325
XP_013991973.1[S.salar]              EKYLIATSEQPIAAFLRDEWLKPEELPIRYAGLSTCFRQEVGSHGRDTRGIFRVHQFEKI            324
XP_032819422.1[P.marinus]            EKYLIATSEQPIAAFHRDEWLKPEQLPMRYAGLSSCFRQEVGSHGRDTRGIFRVHQFEKI            324
XP_018104878.1[X.laevis]             EKYLIATSEQPIAALHRDEWLKPEDLPVRYAGISTCFRQEVGSHGRDTRGIFRVHQFEKI            323
NP_001026563.1[G.gallus]             EKYLIATSEQPIAALHRDEWLKPEDLPIKYAGLSTCFRQEVGSHGRDTRGIFRVHQFEKI            324
NP_001191908.1[M.musculus]           EKYLIATSEQPIAALHRDEWLRPEDLPIKYAGLSTCFRQEVGSHGRDTRGIFRVHQFEKI            324
NP_006504.2[H.sapiens]               EKYLIATSEQPIAALHRDEWLRPEDLPIKYAGLSTCFRQEVGSHGRDTRGIFRVHQFEKI            324
NP_001125910.1[P.abelii]             EKYLIATSEQPIAALHRDEWLRPEDLPIKYAGLSTCFRQEVGSHGRDTRGIFRVHQFEKI            324
                                      *************  *.:*:  . ** :***:*:*****...*************:*:

XP_047126420.1[H.vulgaris]           EQFVICSPHDNESWKHLDEMVNNAEKFYQALNIPYRIVGIVSGALNNAAAKKLDLEAWFP            414
XP_003383114.1[A.queenslandica]      EQFCLTSPHDDVSWKMFDEMISNAEFFYQQLGIPYRVVNIVSGELNLAAAMKYDLEGLFP            384
XP_001639063.2[N.vectensis]          EQFVICSPHDNISWEIFDEMIGNAEEFHKSLGIPYRIVSIVSGALNNAAAKKLDLEAWFP            411
XP_019634257.1[B.belcheri]           EQFVLSKPDD--SWGLFHEMIGNAEEFYKTLGIPYRIVNIVSGALNNAAAMKYDLEAWFP            382
NP_001259982.1[D.melanogaster]       EQFVLTSPHDNKSWEMMDEMIGNAEQFCQSLGIPYRVVNIVSGALNHAASKKLDLEAWFG            385
XP_013991973.1[S.salar]              EQFVFASPHDNKSWEMMDEMIGTAEEFYQTLGIPYRIVNIVSGALNHAASKKLDLEAWFP            384
XP_032819422.1[P.marinus]            EQFVYTSPHDNKSWEMFDEMVKTAEGFYQELGIPYRIVNIVSGALNHAAAKKLDLEAWFP            384
XP_018104878.1[X.laevis]             EQFIYASPHDNKSWEMFEEMIMTAESFYQMLGIPYRIVNIVSGSLNHAASKKLDLEAWFP            383
NP_001026563.1[G.gallus]             EQFVYASPHDNKSWEMFDEMIATAEEFYQSLGIPYHIVNIVSGALNHAASKKLDLEAWFP            384
NP_001191908.1[M.musculus]           EQFVYSSPHDNKSWEMFDEMIATAEEFYQSLGIPYHIVNIVSGSLNHAASKKLDLEAWFP            384
NP_006504.2[H.sapiens]               EQFVYSSPHDNKSWEMFEEMITTAEEFYQSLGIPYHIVNIVSGSLNHAASKKLDLEAWFP            384
NP_001125910.1[P.abelii]             EQFVYSSPHDNKSWEMFEEMITTAEEFYQSLGIPYHIVNIVSGSLNHAASKKLDLEAWFP            384
                                     ***   .*.*  **  :.**: .** * : *.***::*.**** ** **: * ***. *

XP_047126420.1[H.vulgaris]           GSGAFRELCSCSNCTDYQARRLLVRYGQTKKMNDKTEFCHMLNATMCASTRVICCILENN            474
XP_003383114.1[A.queenslandica]      GSGAYRELVSCSNCLDYQVRRLQVRFGLTKKMNAQADYVHMLNATMCATTRTICAILENN            444
XP_001639063.2[N.vectensis]          ASGAFRELVSCSNCTDYQARRLQVRFGQTKKMGVSTEYVHMLNATMCAVSRVICAILENN            471
XP_019634257.1[B.belcheri]           GSGAFRELVSCSNCLDYQARRLLVRYGQTKKMNEKPEFVHMLNATMCATTRVICAILENH            442
NP_001259982.1[D.melanogaster]       GSGAYRELVSCSNCLDYQARRLLVRFGQTKKMNAAVDYVHMLNATMCAATRVICAILETH            445
XP_013991973.1[S.salar]              GSAAFRELVSCSNCLDYQARRLRIRYGQTKKMMDKADYVHMLNATMCATTRVMCAILETY            444
XP_032819422.1[P.marinus]            GSAAFRELVSCSNCTDYQARRLRVRYGQTKKMMDKAEFVHMLNATMCATTRTICAILETY            444
XP_018104878.1[X.laevis]             GSGAFRELVSCSNCTDYQARRLRIRYGQTKKMMDKVDFVHMLNATMCATTRAICAILENY            443
NP_001026563.1[G.gallus]             GSGAFRELVSCSNCTDYQARRLRIRFGQTKKMMDKVEFVHMLNATMCATTRTICAILENY            444
NP_001191908.1[M.musculus]           GSGAFRELVSCSNCTDYQARRLRIRYGQTKKMMDKVEFVHMLNATMCATTRTICAILENY            444
NP_006504.2[H.sapiens]               GSGAFRELVSCSNCTDYQARRLRIRYGQTKKMMDKVEFVHMLNATMCATTRTICAILENY            444
NP_001125910.1[P.abelii]             GSGAFRELVSCSNCTDYQARRLRIRYGQTKKMMDKVEFVHMLNATMCATTRTICAILENY            444
```

```
                                     .*.*:*** ***** ***.*** :*:* ****    :: ********* :*.:*.***.

XP_047126420.1[H.vulgaris]           QTEEGIIVPELLRPFMGN--VEFIKYVKTAPIEGNKDVKGKSNKKKP-----------EK           521
XP_003383114.1[A.queenslandica]      QTEEGVVVPEILRQYMPPAYKEFIKFVQAAPIHEPITKKQENQMAGMEKDNEAQS-----           499
XP_001639063.2[N.vectensis]          QTEEGVVMPQALHPYLPQDL-HFLKFVKPAPIDDKPEQKKQKKKGKNKGANNE---SVGC           527
XP_019634257.1[B.belcheri]           QTEEGIVIPEVLRQYMPGG-TEFITFVKPAPIEEQATKKKKAGKKKEKVKPTQDGPEAEK           501
NP_001259982.1[D.melanogaster]       QTETGIKVPEPLKKYMPAKFQDEIPFVKPAPIDLELAAAEKQKGKKEKTKKDPAA-G---           501
XP_013991973.1[S.salar]              QTEEGIIIPEVLRNFMPPGMTEMLKFVKPAPIDVEMSKKQKKQQ--DGGKKKQGS-G-DQ           500
XP_032819422.1[P.marinus]            QTETGVVVPEALRPFMPPGLKEELKFVKSAPIDQEQIKKQKRGK--GDG-----------           491
XP_018104878.1[X.laevis]             QTEEGIIVPEKLRSFMPPGLKDIIKFVKPAPIDQELTKKQKKQQ--QEKGKKTE---NCG           498
NP_001026563.1[G.gallus]             QTEEGIVVPERLRDFMPPDLRQIIRFVKPAPIEQELSKKQKKQQ--EGGRKKAAG-GERV           501
NP_001191908.1[M.musculus]           QAEKGIAVPEKLREFMPPGLQELIPFVKPAPIDQEPSKKQKKQH--EGSKKKA---KEVP           499
NP_006504.2[H.sapiens]               QTEKGITVPEKLKEFMPPGLQELIPFVKPAPIEQEPSKKQKKQH--EGSKKKAAA-RDVT           501
NP_001125910.1[P.abelii]             QTEKGITVPEKLKEFMPPGLQELIPFVKPAPIEQEPSKKQKKQH--EGSKKKAAA-RDAT           501
                                     *:* *: :*: *: ::     . : :*: ***.       :

XP_047126420.1[H.vulgaris]           IEENLKNLDVKE-          533
XP_003383114.1[A.queenslandica]      -------------          499
XP_001639063.2[N.vectensis]          LESKLEDVNL---          537
XP_019634257.1[B.belcheri]           VTEQVQNMAVNEG          514
NP_001259982.1[D.melanogaster]       -------------          501
XP_013991973.1[S.salar]              LQNQVDNMSVHDS          513
XP_032819422.1[P.marinus]            LAANLGNMAVNDS          504
XP_018104878.1[X.laevis]             LDTQMESMKVHSA          511
NP_001026563.1[G.gallus]             LEEQMQNMGVSSA          514
NP_001191908.1[M.musculus]           LENQLQSMEVTEA          512
NP_006504.2[H.sapiens]               LENRLQNMEVTDA          514
NP_001125910.1[P.abelii]             LENRLQNMEVTDA          514
```

Percent identity matrix:

```
#
#
#  Percent Identity  Matrix - created by Clustal2.1
#
#
```

1: XP_047126420.1[H.vulgaris]	100.00	61.35	65.20	67.94	63.19	65.60	66.67	65.73	66.00	66.20	65.80	65.80
2: XP_003383114.1[A.queenslandica]	61.35	100.00	61.46	62.75	61.32	63.71	63.88	65.38	64.31	63.56	63.10	63.10
3: XP_001639063.2[N.vectensis]	65.20	61.46	100.00	66.14	65.18	65.21	67.34	64.34	67.46	66.27	66.27	66.27
4: XP_019634257.1[B.belcheri]	67.94	62.75	66.14	100.00	66.53	68.70	67.94	67.39	69.55	69.82	69.74	69.74
5: NP_001259982.1[D.melanogaster]	63.19	61.32	65.18	66.53	100.00	68.61	68.98	69.03	70.02	68.69	69.01	69.01
6: XP_013991973.1[S.salar]	65.60	63.71	65.21	68.70	68.61	100.00	75.79	77.25	78.95	79.45	78.17	78.17
7: XP_032819422.1[P.marinus]	66.67	63.88	67.34	67.94	68.98	75.79	100.00	75.94	79.17	77.78	76.98	76.98
8: XP_018104878.1[X.laevis]	65.73	65.38	64.34	67.39	69.03	77.25	75.94	100.00	81.60	81.96	81.41	81.41
9: NP_001026563.1[G.gallus]	66.00	64.31	67.46	69.55	70.02	78.95	79.17	81.60	100.00	86.72	85.80	85.80
10: NP_001191908.1[M.musculus]	66.20	63.56	66.27	69.82	68.69	79.45	77.78	81.96	86.72	100.00	95.90	95.70
11: NP_006504.2[H.sapiens]	65.80	63.10	66.27	69.74	69.01	78.17	76.98	81.41	85.80	95.90	100.00	99.81
12: NP_001125910.1[P.abelii]	65.80	63.10	66.27	69.74	69.01	78.17	76.98	81.41	85.80	95.70	99.81	100.00
