# Supplementary material for: An Evolutionary Perspective on the Origin, Conservation and Binding Partner Acquisition of Tankyrases
Source: Biomolecules. 2022 Nov 15;12(11):1688. doi: 10.3390/biom12111688 (PMC9688111; doi:10.3390/biom12111688)
Supplement: Supplementary file 1 [file biomolecules-12-01688-s001.zip › biomolecules-1913719-supplementary-11.17 -done/Data_2/TNKS1.pdf]

TNKS1

Sequences:

>NP_003738.2 poly [ADP-ribose] polymerase tankyrase-1 [Homo sapiens]
MAASRRSQHHHHHHQQQLQPAPGASAPPPPPPPPLSPGLAPGTTPASPTASGLAPFASPRHGLALPEGDG
SRDPPDRPRSPDPVDGTSCCSTTSTICTVAAAPVVPAVSTSSAAGVAPNPAGSGSNNSPSSSSSPTSSSS
SSPSSPGSSLAESPEAAGVSSTAPLGPGAAGPGTGVPAVSGALRELLEACRNGDVSRVKRLVDAANVNAK
DMAGRKSSPLHFAAGFGRKDVVEHLLQMGANVHARDDGGLIPLHNACSFGHAEVVSLLLCQGADPNARDN
WNYTPLHEAAIKGKIDVCIVLLQHGADPNIRNTDGKSALDLADPSAKAVLTGEYKKDELLEAARSGNEEK
LMALLTPLNVNCHASDGRKSTPLHLAAGYNRVRIVQLLLQHGADVHAKDKGGLVPLHNACSYGHYEVTEL
LLKHGACVNAMDLWQFTPLHEAASKNRVEVCSLLLSHGADPTLVNCHGKSAVDMAPTPELRERLTYEFKG
HSLLQAAREADLAKVKKTLALEIINFKQPQSHETALHCAVASLHPKRKQVTELLLRKGANVNEKNKDFMT
PLHVAAERAHNDVMEVLHKHGAKMNALDTLGQTALHRAALAGHLQTCRLLLSYGSDPSIISLQGFTAAQM
GNEAVQQILSESTPIRTSDVDYRLLEASKAGDLETVKQLCSSQNVNCRDLEGRHSTPLHFAAGYNRVSVV
EYLLHHGADVHAKDKGGLVPLHNACSYGHYEVAELLVRHGASVNVADLWKFTPLHEAAAKGKYEICKLLL
KHGADPTKKNRDGNTPLDLVKEGDTDIQDLLRGDAALLDAAKKGCLARVQKLCTPENINCRDTQGRNSTP
LHLAAGYNNLEVAEYLLEHGADVNAQDKGGLIPLHNAASYGHVDIAALLIKYNTCVNATDKWAFTPLHEA
AQKGRTQLCALLLAHGADPTMKNQEGQTPLDLATADDIRALLIDAMPPEALPTCFKPQATVVSASLISPA
STPSCLSAASSIDNLTGPLAELAVGGASNAGDGAAGTERKEGEVAGLDMNISQFLKSLGLEHLRDIFETE
QITLDVLADMGHEELKEIGINAYGHRHKLIKGVERLLGGQQGTNPYLTFHCVNQGTILLDLAPEDKEYQS
VEEEMQSTIREHRDGGNAGGIFNRYNVIRIQKVVNKKLRERFCHRQKEVSEENHNHHNERMLFHGSPFIN
AIIHKGFDERHAYIGGMFGAGIYFAENSSKSNQYVYGIGGGTGCPTHKDRSCYICHRQMLFCRVTLGKSF
LQFSTMKMAHAPPGHHSVIGRPSVNGLAYAEYVIYRGEQAYPEYLITYQIMKPEAPSQTATAAEQKT

>XP_024106876.1 tankyrase-1 [Pongo abelii]
MAASRRSQHHHHHHQQQLQPAPGASAPPPPPPPPLSPGLAPGTTPASPTASGLAPFASPRHGLALPEGDG
SRDPPDRPRSPDPVDGTSCCSTTSTICTVTAAPVVPAVSTSSAVGVAPNPAGSGSNNSPSSSSSPTSSSS
SSPSSPGSSLAESPEAAGVSSTAPLGPGAAGPGTGVPAVSGALRELLEACRNGDVSRVKRLVDAANVNAK
DMAGRKSSPLHFAAGFGRKDVVEHLLQMGANVHARDDGGLIPLHNACSFGHAEVVSLLLCQGADPNARDN
WNYTPLHEAAIKGKIDVCIVLLQHGADPNIRNTDGKSALDLADPSAKAVLTGEYKKDELLEAARSGNEEK
LMALLTPLNVNCHASDGRKSTPLHLAAGYNRVRIVQLLLQHGADVHAKDKGGLVPLHNACSYGHYEVTEL
LLKHGACVNAMDLWQFTPLHEAASKNRVEVCSLLLSHGADPTLVNCHGKSAVDMAPTPELRERLTYEFKG
HSLLQAAREADLAKVKKTLALEIINFKQPQSHETALHCAVASLHPKRKQVTELLLRKGANVNEKNKDFMT
PLHVAAERAHNDVMEVLHKHGAKMNALDTLGQTALHRAALAGHLQTCRLLLSYGSDPSIISLQGFTAAQM
GNEAVQQILSESTPIRTSDVDYRLLEASKAGDLETVKQLCSPQNVNCRDLEGRHSTPLHFAAGYNRVSVV
EYLLHHGADVHAKDKGGLVPLHNACSYGHYEVAELLVRHGASVNVADLWKFTPLHEAAAKGKYEICKLLL
KHGADPTKKNRDGNTPLDLVKEGDTDIQDLLRGDAALLDAAKKGCLARVQKLCTPENINCRDTQGRNSTP
LHLAAGYNNLEVAEYLLEHGADVNAQDKGGLIPLHNAASYGHVDIAALLIKYNTCVNATDKWAFTPLHEA
AQKGRTQLCALLLAHGADPTMKNQEGQTPLDLATADDIRALLIDAMPPEALPTCFKPQATVVSASLISPA
STPSCLSAASSIDNLTGPLAELAVGGASNAGDGAAGTERKEGEIAGLDMNISQFLKSLGLEHLRDIFETE
QITLDVLADMGHEELKEIGINAYGHRHKLIKGVERLLGGQQGTNPYLTFHCVNQGTILLDLAPEDKEYQS
VEEEMQSTIREHRDGGNAGGIFNRYNVIRIQKVVNKKLRERFCHRQKEVSEENHNHHNERMLFHGSPFIN
AIIHKGFDERHAYIGGMFGAGIYFAENSSKSNQYVYGIGGGTGCPTHKDRSCYICHRQMLFCRVTLGKSF
LQFSTMKMAHAPPGHHSVIGRPSVNGLAYAEYVIYRGEQAYPEYLITYQIMKPEAPSQTATAAEQKT

>NP_780300.2 poly [ADP-ribose] polymerase tankyrase-1 [Mus musculus]
MAASRRSQHHHHHHQQQLQPAPGASAPPPPPPPPLSPGLAPGPTPASPTAGGLAPFASPRHGLALPEGDG
SRDPPDRPRSPDPVDGAVCTVAAPAAVPAASAAVGVAPTPAGGGGGGGNNSASSASSPTSSSSSSPSSPG
SSLAESPEAAGVGSTATLGAGAAGLGPGVPAVSGALRELLEACRNGDVSRVKRLVDAANVNAKDMAGRKS
SPLHFAAGFGRKDVVEHLLQMGANVHARDDGGLIPLHNACSFGHAEVVSLLLCQGADPNARDNWNYTPLH
EAAIKGKIDVCIVLLQHGADPNIRNTDGKSALDLADPSAKAVLTGEYKKDELLEAARSGNEEKLMALLTP
LNVNCHASDGRKSTPLHLAAGYNRVRIVQLLLQHGADVHAKDKGGLVPLHNACSYGHYEVTELLLKHGAC
VNAMDLWQFTPLHEAASKNRVEVCSLLLSHGADPTLVNCHGKSAVDMAPTPELRERLTYEFKGHSLLQAA
READLAKVKKTLALEIINFKQPQSHETALHCAVASLHPKRKQVAELLLRKGANVNEKNKDFMTPLHVAAE
RAHNDVMEVLHKHGAKMNALDSLGQTALHRAALAGHLQTCRLLLSYGSDPSIISLQGFTAAQMGNEAVQQ
ILSESTPMRTSDVDYRLLEASKAGDLETVKQLCSPQNVNCRDLEGRHSTPLHFAAGYNRVSVVEYLLHHG
ADVHAKDKGGLVPLHNACSYGHYEVAELLVRHGASVNVADLWKFTPLHEAAAKGKYEICKLLLKHGADPT
KKNRDGNTPLDLVKEGDTDIQDLLRGDAALLDAAKKGCLARVQKLCTPENINCRDTQGRNSTPLHLAAGY
NNLEVAEYLLEHGADVNAQDKGGLIPLHNAASYGHVDIAALLIKYNTCVNATDKWAFTPLHEAAQKGRTQ
LCALLLAHGADPTMKNQEGQTPLDLATADDIRALLIDAMPPEALPTCFKPQATVVSASLISPASTPSCLS
AASSIDNLTGPLTDLAVGGASNAGDGAAGAERKEGEVAGLDMNISQFLKSLGLEHLRDIFETEQITLDVL
ADMGHEELKEIGINAYGHRHKLIKGVERLLGGQQGTNPYLTFHCVNQGTILLDLAPEDKEYQSVEEEMQS
TIREHRDGGNAGGIFNRYNVIRIQKVVNKKLRERFCHRQKEVSEENHNHHNERMLFHGSPFINAIIHKGF
DERHAYIGGMFGAGIYFAENSSKSNQYVYGIGGGTGCPTHKDRSCYICHRQMLFCRVTLGKSFLQFSTMK
MAHAPPGHHSVIGRPSVNGLAYAEYVIYRGEQAYPEYLITYQIMKPEAPSQTATAAEQKT

>NP_989671.2 poly [ADP-ribose] polymerase tankyrase-1 [Gallus gallus]
MAAPPRRSQHHHHHHGPPPPPGPASPPAAASPPRSPSLAPAELGPAAQRHSLAGPEGEAPPDAERPPAPE
CSEGAAPGPPPGSGSSSGSSASSSSSSSSTSSSVASSPAAESPEAAGPSGAFRELLEACRNGDVTRVKRL
VDAGNVNAKDMAGRKSTPLHFAAGFGRKDVVEHLLQTGANVHARDDGGLIPLHNACSFGHAEVVSLLLCQ
GADPNARDNWNYTPLHEAAIKGKIDVCIVLLQHGADPNIRNTDGKSALDLADPSAKAVLTGEYKKDELLE
AARSGNEEKLMALLTPLNVNCHASDGRKSTPLHLAAGYNRVRIVQLLLQHGADVHAKDKGGLVPLHNACS
YGHYEVTELLLKHGACVNAMDLWQFTPLHEAASKNRVEVCSLLLSHGADPTLVNCHGKSAVDMAPTPELR
ERLTYEFKGHSLLQAAREADLAKVKKTLALEIINFKQPQSHETALHCAVAAVHPKRKQVTELLLRKGANV
NEKNKDFMTPLHVAAEKAHNDVMEVLHKHGAKMNALDTLGQTALHRAALAGHLQTCRLLLNYGSDPSIIS
LQGFTAAQIGNEAVQQILSESTPVRTSDVDYRLLEASKAGDLETVKQLCSPQNVNCRDLEGRHSTPLHFA
AGYNRVSVVEYLLHHGADVHAKDKGGLVPLHNACSYGHYEVAELLVRHGASVNVADLWKFTPLHEAAAKG
KYEICKLLLKHGADPTKKNRDGNTPLDLVKEGDTDIQDLLRGDAALLDAAKKGCLARVQKLCTQENINCR
DTQGRNSTPLHLAAGYNNLEVAEYLLEHGADVNAQDKGGLIPLHNAASYGHVDIAALLIKYNTCVNATDK
WAFTPLHEAAQKGRTQLCALLLAHGADPTMKNQEGQTPLDLATADDIRALLIDAMPPEALPTCFKPQATV
VSASLISPASTPSCLSAASSIDNLTGPLAELAVGGASNTGDGAAGTERKEGEVSGLDMNITQFLKSLGLE
HLRDIFETEQITLDVLADMGHEELKEIGINAYGHRHKLIKGVERLLGGQQGTNPYLTFHCVSQGTILLDL
APDDKEYQSVEEEMQSTIREHRDGGNAGGIFNRYNVIRIQKVVNKKLRERFCHRQKEVSEENHNHHNERM
LFHGSPFINAIIHKGFDERHAYIGGMFGAGIYFAENSSKSNQYVYGIGGGTGCPTHKDRSCYICHRQMLF
CRVTLGKSFLQFSTMKMAHAPPGHHSVIGRPSVNGLAYAEYVIYRGEQAYPEYLITYQIVKPEAPSQTGT
AAEQKT

>XP_018099068.1 poly [ADP-ribose] polymerase tankyrase-1 isoform X2 [Xenopus laevis]
MAAPSRRSQQQQVPTAAAGQSSQPPSASPPLSPSSRRAASCLSPPCSPETNALSLSREGPDSEPEGEAAS
QPGITPRPLSPPPGEAANILSPTSSLPPAPAPSSGSSSSSSSPSSNSLCTGGSPAESPESGVIGGVSSVG
IGPGDPLSAVSGAFRELFEACRNGDVSRVRRLLEPANVNAKDMAGRKSTPLHFAAGFGRKDVVEHLLQTG
ANVHARDDGGLIPLHNACSFGHAEVVTLLLCQGADPNARDNWNYTPLHEASIKGKIDVCIVLLQHGADPN
IRNTDGKSALDLADPSSKTVLTGEYKKDELLEAARSGNEEKLMALLTPLNVNCHASDGRKSTPLHLAAGY
NRVQIVQLLLQHGADVHAKDKGGLVPLHNACSYGHFEVTESLLKHGACVNAMDLWQFTPLHEAASKNRVE
VCSLLLSHGADPTLVNCHGKSAVDMAPTPELKERLSYEFKGHSLLQAAREADLAKVKKTLALEIINFKQP
QSHETALHCAVASLHPKRKQITELLLRKGASVNEKNKDFMTPLHVAAERAHNDVVEVLHKHGAKMNALDT
LGQTALHRAALGGHLQTCRLLLSFGSDASIVSLQGFTAAQMGNEAVQQILNESTPVRTSDVDYRLLEASK
AGDLDIVKQLCSSQNVNCRDLEGRHSTPLHFAAGYNRVSVVEYLLHHGADVHAKDKGGLVPLHNACSYGH
YEVAELLVRHGASVNVADLWKFTPLHEAAAKGKYEICKLLLKHGADPTKKNRDGNTPLDLVKDGDTDIQD
LLRGDAALLDAAKKGCLARVQKLCTQENINCRDTQGRNSTPLHLAAGYNNLEVAEYLLEHGADVNAQDKG
GLIPLHNAASYGHVDIAALLIKYNTCVNATDKWAFTPLHEAAQKGRTQLCALLLAHGADPTMKNQESQTP
LDLATADDIRALLIDAMPPEALPSCFKPQATVVSASIISPPSTPSCLSAASSIDNLTGPLAELAVGGVSN
AGDGAAGTERKEGEVTVLDININQFLKSLGLEHLRDIFETEQITLDVLADMGHEELKEIGINAYGHRHKL
IKGVERLLGGQQGTNPYLTFHCVSQGTVLLDLASDDKEYQSVEEEMQNSIREHRDGGNAGGVFNRYNVIR
IQKVVNKKLRERFCHRQKEVSEENHNHHNERMLFHGSPFINAIIHKGFDERHAYIGGMFGAGIYFAENSS
KSNQYVYGIGGGTGCPTHKDRSCYICHRQMLFCRVTLGKSFLQFSTMKMAHAPPGHHSVIGRPSVNGLAY
AEYVIYRGEQAYPEYLITYQIMKPETPLQAATTAEQKT

>XP_014017013.1 poly [ADP-ribose] polymerase tankyrase-1-like isoform X4 [Salmo salar]
MAVSRRSSQQQQGNLLSPPRNGSLSVSPPGSPSLNLVTATLVPPDGERGCSTGMENPLASPDLQTSALSS
GSSTSPTTTTSGGGSSSVSSPGSGGTTPGDGSGIGGAFRELFEACRNGDVSRVKRLVDSVNVNAKDMAGR
KSTPLHFAAGFGRKDVVDHLLQTGANVHARDDGGLIPLHNACSFGHAEVVSLLLCQGADPNARDNWNYTP
LHEAAIKGKIDVCIVLLQHGADPNIRNTDGKSALDLADPSAKAVLTGEYKKDELLEAARSGNEEKLMALL
TPLNVNCHASDGRKSTPLHLAAGYNRVRIVQLLLQHGADVHAKDKGGLVPLHNACSYGHFEVTELLLKHG
ACVNAMDLWQFTPLHEAASKNRVEVCSLLLSHGADPTLLNCHSKSAVDMAPTPELKERLTYEFKGHSLLQ
AAREADMAKVKKTLALEIISFKHPQTNETALHCAVASPHPKRKQVTELLLRKGANINEKNKDFMTALHVA
SERAHNDILEVLQKHGAKVNAVDTLGQTALHRAALAGHIQTCRLLLSYGADPAIVSLQGFTASQMGNEAV
QQILNENVPTRNSDVDYRFLEAAKAGDLDTVQQLCTPQNVNCRDLEGRHSTPLHFAAGYNRVAVVEYLLH
HGADVHAKDKGGLVPLHNACSYGHYEVAELLVRHGASVNVADLWKFTPLHEAAAKGKYEICKLLLKHGAD
PTKKNRDGNMPLDMVKDGDTDIQDLLRGDAALLDAAKKGCLARVQKLCSPENINCRDTQGRNSTPLHLAA
GYNNLEVAEYLLEHGADVNAQDKGGLIPLHNAASYGHVDIAALLIKFNTCVNATDKWAFTPLHEAAQKGR
TQLCALLLAHGADPTMKNQEGQTALDLATADDIRALLMDAMPPDALPSCFKPQATVVSAGSVISPASTPS
CLSAASSIDNLAGPLNELGAAGTSGVADGATGSDRKEGELVMLDMNISQFLKSLGLEHLRDIFEREQISL
DVLADMGHEELKEIGINAYGHRHKLIKGVERLLGGQQGANPYLTFHCANQGTVLIDLAPDDKEGQSVEEE
MQSTIREHRDGGNAGGVFSRYNIIKIQKVVNKKLRERYTHRQKEISDENHNHHNERMLFHGSPFINAIIH
KGFDERHAYIGGMFGAGIYFAENSSKSNQYVYGIGGGTGCPTHKDRSCYLCHRQMLFCRVTLGKSFLQFS
AMKMAHAPPGHHSVIGRPSVNGLAYAEYVIYRGEQAYPEYLITYQILKPESTATSAAGEDQKS

>XP_032806710.1 poly [ADP-ribose] polymerase tankyrase-2 [Petromyzon marinus]
MAARRAHLASPSSSSSLSPSSGSLDTTTAAAAAAAATTTAAAAAATTTAAAMTTTAAVSATSSPSTTAAA
AAAVAAAAATGSGPGSGQDSSADGGDAGKELFEACRNGDVGRVKRLVSPLNVNARDTTGRKSTPLHFAAG
FGRKDVVEHLLQNGANVHARDDGGLIPLHNACSFGHAEVVNLLLRQGADPNARDNWNYTPLHEAAIKGKI
DVCIVLLQHGADPGIRNTDGKTALDLAEPTAKAVLTGEYKKDELLEAARSGNEEKLMALLTPLNVNCHAS
DGRKSTPLHLAAGYNRVRIVQLLLQHGADVHAKDKGGLVPLHNACSYGHYEVTELLVKHGASVNAMDLWQ
FTPLHEAASKNRVEVCSLLLSYGADPTLLNCHSKSAIDLAPTSELKERLVYEFRGHSLLQAAREADVTRV
KKHLGLDIINFKHPQTHETALHCAVASPYPKRKQVSELLLRKGANVNEKTKDLLTPLHVASDKAHNDVID
VLLKHGAKINVTDSLGQTSLHRAAHAGHLTTCRLLLSHGCDPNLPSLQGFTAAQLGNESVQQILHESSLP
VRNSDVEHQLLEAAKAGDLDTVKQLCTTQTVNCRDMEGRHSTPLHFAAGYNRVSVVEYLLQHGADVHAKD
KGGLVPLHNACSYGHYEVAELLVKRGAVVNVADLWKFTPLHEAAAKGKYEICKLLLKHGADPTKKNRDGN
TPLDLVKEGDTDIQDLLRGDAALLDAAKKGCLARVQKLCSGENVNCRDSQGRNSTPLHLAAGYNNLEVAE
YLLEHGADVNAQDKGGLIPLHNAASYGHVDIAALLIKYSACVNATDKWAFTPLHEAAQKGRTQLCALLLA
HGADPSMKNQEGQTPLDLATADDVRALLIDAMPPEALPVTHKPPSIPTIAATSAAATAALALGPTPALAS
LAPSPVPSGASASGAGGVPLLASPSSTPASLSAASSMDSLTGACGVAPSPVPAGAIGAGAAGDGTLLGAC
GVSAADGATGPADKPDRSADFEIPGLDMNINMFLKGLGLDHLRDIFEKEQITLDVLADMGHEELKEIGIN
AYGHRHKLIKGVERLLGAQQGGLAYPGANPYLSYHTTAQGTLLIDLPPDDKEYQSVEDEMQSTIREHKDG
GQAGGVFNRYNVIKIQKVRNKKLWERYTHRRKEVTEENHNHHNERMLFHGSPFINAIINKGFDERHAYIG
GMFGAGIYFAENSSKSNQYVYGIGGGTGCPVHKDRSCYICQRQMLFCRVTLGKSFLQFSAMKMAHSPPGH
HSVIGRPSVNGLAFAEYVIYRGEQAYPEYLITYQILKPECTPDLTSAAGQKS

>XP_019641281.1 PREDICTED: tankyrase-1-like isoform X1 [Branchiostoma belcheri]
MAGRRAMLSSDRIHCDESSGCRELFEACRNGDVARVKKLITPQNVNARDTAGRKSTPLHFAAGFGRKDVV
EHLLQNGANVHARDDGGLIPLHNACSFGHAEVVTLLLRNGADPNARDNWNYTPLHEAAIKGKIDVCIVLL
QNGADPSIRNTDGKTALDLAEPSAKTVLTGDYKKDELLEAARSGNEDKLMALLTPLNVNCHASDGRKSTP
LHLAAGYNRVRIVQLILQHGADVHAKDKGGLVPLHNACSYGHFEVTELLLKHGASVNAMDLWQFTPLHEA
ASKSRIEVCSLLLSHGADPTLLNCHSKSAIDVASTPELQEKLSYEFKGHCLLDGARQTDMAKVKKHLQLD
IVNFKHPYTHDTALHCAALSPYPKRRQITELLIRKGANLNDKNKEFLTPLHVASDKCYIDVMEVLLKHGA
KVNALDSLGQTALHRGAHCGHVQACRLLLSFGVDPSIVSLQGVTAAQMATEAVQQMLHEDPPSGPADECD
QLLEAAKAGDLEAVKVLCNPHTVNCRDVNGRHSTPLHFAAGYNRVAVVEYLLQHGADVHAKDKGGLVPLH
NACSYGHYEVCELLLKHGAVVNVADLWKFTPLHEAAAKGKYEICKLLLKHGADPNKKNRDGNTPLDLVRD
GDTDIQDLLRGDAALLDAAKKGNLARVQKLATPENINCRDTQGRNSTPLHLAAGYNNVEVAEFLLENGAD
VNAQDKGGLIPLHNASSYGHVEIAQLLIKYGTCVNATDRWNFTPLHEAAQKGRTQLCALLLAHGADPTMK
NQEGQTPLDLATAEDVRALLVDAMPPQSLPAAITAATVKAASPISSPSTTPSSTPGVLSTASSMDHLVGA
VGGAGQGDGAIDRSAVEGESLLDMTISSFLQQLGLNQLLDIFNKEQITLDILGEMGHEELKEIGVNAYGH
RHKLIKGVERILGGTGTTLNPYLTPALGSHGTILTDLSPDDKEYQSVEEEVQSTIRQHKDQGQAGGIFNR
YNVIKIQKVRNKRLWDRYVHRRKEVSEENHNQSNERMLFHGSPFINAIINKGFDERHAYIGGMFGAGIYF
AENSSKSNQYVYGIGGGTGCPIHKDRSCYICQRQMLFCRVTLGKAFLQFSAMKMAHAPPGHHSVIGRPSV
GGLNFAEYVIYRGEQAFPEYLITYQIVKPEAPAAPSPDQK

>NP_651410.1 tankyrase, isoform A [Drosophila melanogaster]
MANSSRSRAILSVNLDAVMANDPLRELFEACKTGEIAKVKKLITPQTVNARDTAGRKSTPLHFAAGYGRR
EVVEFLLNSGASIQACDEGGLHPLHNCCSFGHAEVVRLLLKAGASPNTTDNWNYTPLHEAASKGKVDVCL
ALLQHGANHTIRNSEQKTPLELADEATRPVLTGEYRKDELLEAARSGAEDRLLALLTPLNVNCHASDGRR
STPLHLAAGYNRIGIVEILLANGADVHAKDKGGLVPLHNACSYGHFDVTKLLIQAGANVNANDLWAFTPL
HEAASKSRVEVCSLLLSRGADPTLLNCHSKSAIDAAPTRELRERIAFEYKGHCLLDACRKCDVSRAKKLV
CAEIVNFVHPYTGDTPLHLAVVSPDGKRKQLMELLTRKGSLLNEKNKAFLTPLHLAAELLHYDAMEVLLK
QGAKVNALDSLGQTPLHRCARDEQAVRLLLSYAADTNIVSLEGLTAAQLASDSVLKLLKNPPDSETHLLE
AAKAGDLDTVRRIVLNNPISVNCRDLDGRHSTPLHFAAGFNRVPVVQFLLEHGAEVYAADKGGLVPLHNA
CSYGHYEVTELLVKHGANVNVSDLWKFTPLHEAAAKGKYDICKLLLKHGADPMKKNRDGATPADLVKESD
HDVAELLRGPSALLDAAKKGNLARVQRLVTPESINCRDAQGRNSTPLHLAAGYNNFECAEYLLENGADVN
AQDKGGLIPLHNASSYGHLDIAALLIKHKTVVNATDKWGFTPLHEAAQKGRTQLCSLLLAHGADAYMKNQ
EGQTPIELATADDVKCLLQDAMATSLSQQALSASTQSLTSSSPAPDATAAAAPGTSSSSSSAILSPTTET
VLLPTGASMILSVPVPLPLSSSTRISPAQGAEANGAEGSSSDDLLPDADTITNVSGFLSSQQLHHLIELF
EREQITLDILAEMGHDDLKQVGVSAYGFRHKILKGIAQLRSTTGIGNNVNLCTLLVDLLPDDKEFVAVEE
EMQATIREHRDNGQAGGYFTRYNIIRVQKVQNRKLWERYAHRRQEIAEENFLQSNERMLFHGSPFINAIV
QRGFDERHAYIGGMFGAGIYFAEHSSKSNQYVYGIGGGIGCPSHKDKSCYVCPRQLLLCRVALGKSFLQY
SAMKMAHAPPGHHSVVGRPSAGGLHFAEYVVYRGEQSYPEYLITYQIVKPDDSSSGTEDTR

>XP_032220531.1 poly [ADP-ribose] polymerase tankyrase-1-like [Nematostella vectensis]
MPCQAKMASKKLASNPTFLSDPDVCEGGRELFEACRNGDVSKVRKLVTNNSNVNVRDTAGRKSSPLHFAA
GFGRKEVVEYLLQCGADVHAMDDGGLIPLHNACSFGHAEVVRILLSHGADANARDNWNYTPLHEAAVKGK
VDVCVVLLQHGADPNIRNTDGKTALDVAEAAAKLVLTGDYKKDELLEGARSGNEEKLMSLLTPLNVNCHA
SDGRKSTPLHLAAGYNRVGVVQLLLKHGADVHAKDKGGLVPLHNACSYGHFEVTELLIKHGASVNAMDLW
QFTPLHEAASKARVEVCALLLGHGADPTLLNCHSKSAIDAAPTKELQEKLLSEFKGHQILEAAKGCDTAK
IKKLITTETINFQHPLTLDTPLHVAVASSSPKKKGVIETLLKKGADPNLQNKSSYAPLHVAAEKGLFEAM
ELLLKHGAKVNAIDSAGQTALHVAAIAGQVQACRVLMSNGADPTCQTFQGYTAFEVAPEPVQKVLHVNEE
SPSPVSDAEKQLLEAAKTGDLETVKNLCTAQTVNCRDLDGRCSTPLHFAAGYNRVRVVEYLLLNGADVHA
KDKGGLVPLHNACSYGHYEVAELLVKHGAIVNVADLWKFTPLHEAAAKGKYEICQLLLKHGADPQKKNRD
GYTPLDLVKEGDSDVADLLRGDAALLDAAKKGNLIRITKLATPENINCRDTQGRNSTPLHLAAGYNHLEV
AEYLLEHGADVNAQDKGGLIPLHNASSYGHVDIAALLIRYNTDVNATDRWLFTPLHEAAQKGRTQLCALL
LAHGAEPSMKNQEGQSPVDLATAEDVKCLLGDAMLSTQTTSVSTAPVAKVPAPVTATAGAATPATGPISG
MAPLSQANSSLLANSPAGHGDGAFSNRSVSEAQGEAVRSISAVHPGLDVDVGQFLDGLQLNNLKEIFERE
QISWDVLVDMGHEELKEIGIHAYGHRHKILKAVKEKISGMGLGLGPFSTSQVQGSVIQELSMVDKDFASV
ADEMQSTIVEHRDSGTAGGVFTSYTILKIERVVNTKLWEKYVYRRREIADSNHNHANERMLFHGSPFINA
IVQKGFDQRHAYIGGMFGAGIYFAENSSKSNQYVYGIGGGSGCPTHKDRSCYSCERQLLLCRVALGKPFY
QFSAVKMAHAPPGHNSVIGRPSSGGLSFAEYVIYRGEQAYPEYLITYKINKPDT

>XP_047128184.1 poly [ADP-ribose] polymerase tankyrase-2-like isoform X1 [Hydra vulgaris]
MALGRPNRAMPSRRNSVASNFMDGGVHENDSGRELFEACRTGDVIRVRYLIQNNWDVNMRDTAGRKSTPL
HFAAGFGRKDIVEYLLDAGADVHARDDGGLIPLHNACSFGHVEVTLSLLRHHADVNARDNWNFTPLHEAA
IKGKVEVCIALLQHGADPLIRNTDGKTARDLAEATAKAVLTGEYKKEELLDSARSGCEEKMLSLITSLNV
NCHAGDGRKSTPLHLAAGYNRTRIVEILLKHGADVHAKDKGGLVPLHNACSYGHYEVTELLVKHGASVNA
MDLWQFTPLHEAASKMRIEVCTLLLSYGADPTVLNCHSKTAIDVAPVQELKDKLLYEYRGHTYLEAVKSS
DLSKIKRIIAPDLVNFVHPLTLNSALHVCALSTSAKRDRVADFLIKKGCNMNFCNKQKLTPLHLCAGNDN
VVVTEILIKAGADCNIVNESLQSPLHIAAQSGHAHICYILLMNGADPTIRNSSGYTPAQVGTESVQKIFQ
ARLEPMLKDVDINLLEASKTGDFELIQSLCTPQNVNCRDVAGRMSTPLHFASGYNRMDIVEHLLRCGADV
HAKDKGGLVPLHNACSYGHFEIAEILVRHGANVNAADYWKFTPLHEAAAKGKFDICKLLLKHGADPKKKN
HEGYTPLDLVKEGFPDVADLLRGDAALLEAAKKGNLAKVMKLVTHQNINCRDTLGRNSTPLHLAAGYNHV
EVAEYLLEFGADVNAQDKGGLIPLHNASSYGHVDIAALLIKYKADVNATDRWLFTPLHEAAQKGRTQLCA
LLLTHGANANMKNQEGQTPIDLATADDVQVLLQDSMVSKSEVMQITSNTCSIKSPINSPQVLPISNDLSP
QLEPQGLTKPSTANQNLLLSEPCNSDGLQTKRLRDQAEGTNPLDIDIGTFLSSLQLEFLHEIFSREQITM
DVLIEMGPDQLKEIGIVAYGHRHKILKGVKERLAGAGCTIVGEPFLSNINASGPNTILQELSMDEKDYQS

VAEEMQSTVVEHKDGGVAGGIFSRYSSLKIERVINKKLWDKYYYQRKQVADANHNHPNERMLFHGSPFVN
AIVQKGFDERHAYIGGMFGAGIYFAEHSSKSNQYVYGIGGGSGCPVHKDRSCYDCKRQIILCRVTLGKPF
FQNSAMKMAHAPPGHHSVIGRPNSGGLSYPEYVVYRGEQAYPEYLITYKIERPNTLSSCQSAVD

>XP_019848937.1 PREDICTED: tankyrase-2-like [Amphimedon queenslandica]
MDTRDHQAKIPGDSRQTSQSIEEGESADKNTGDLARLFEACKNGDIDTVQNLIQQRQSSANERDLHGRKS
TPLHFAAGFGRRDIVKFLIEKGAHVDFRDEGGLIPLHNSCSFGHVDVVQLLLSNGANPNAQDNWKFSPLH
EAAIKGKADVCIVLLQHGADSSLLNTDRKAPIDLANGQAREVLLGTYRQDELLEAAKVGDEQLLMQILTP
LNVNCHASDGRRSTPLHLAAGYNRTSIVQLLLKQGADVHAKDKGGLVPLHNACSYGHYEVAELLLKYGAS
INVTDLWQFTPLQEAASKGRSDVCSLLLAHGANPSIANCHGKTAFNLAPSEEFRKKLDSEYRGYQLLAAA
EDGGIILLKKLLSSQLLKFQHHQTLDTLLHKAVLSKSSNRQSVIDALLKRGINLNIGNKENMTPLICAAK
KGILEVVEQLVQRGANINHQDINGMTSLHWAVQNEHAQICRYLLSSGANPSIVNNQGQTIYQLKTSDTIQ
LILKNEPPVSQFEIEQQLLEAARNSDLEILKKICTPQNVNCRDTKGRMSTPLHFAAGYNRVTVVEFLLEN
GADVHAKDKGGLVPLHNACSYGHYEVAELLVKYKANVNAMDLWKFTPLHEAAAKEKYDICKLLLKNGANV
HSKNRDNLTPIDLVKDPKSDLADLLRGEPALLDAAKKGEIERVKKLLTEDNVNCRDEYGRNSTPLHLAAG
YNHLDVVEYLLENKADVNAKDKGGLVPLHNASSYGHVDVASLLIRYNSVINATDRWNFTPLHEAAQKGRT
QVCSLLIIHGADVYLKNQEGQIPLDLATADDVIALLQDAMMKDIPLTIPPAEKEAKSNIVNKGLTAAGAS
LLASELVLGDGVDDKNGMVTQQRGGPAGVGDGSDKGFMSYRGGGDAPPPWHNVTVKDILTELELGHLVEL
FEREQITIDILIEMNGDDLQSIGITAFGVRHRLLKRIRELVQGNNEEYPVGVTTTKPTQGTQLIELSSDD
KEFIDTADLMQSTICEHRDDGKAGGVFDSYEILKIERIVNTKVWERYKYRRKEVAESNNNCANELMLFHG
SPFVPYIVHNGFDERHAYIGGMFGAGIYFAEHSSKSNQYVYGIGGGNGCPEHKNRSCYTCLRKLLLCRVV
LGKPVEQYTAVRIAHAPPGHHSVIGRPSAGGLNYPEYVIYRGEQAYPEYIITFRIKKPSATDSMSSSSSL
DMSNNT

Alignment

```
XP_019848937.1[A.queenslandica]      ------------------------------------------------------------           0
NP_651410.1[D.melanogaster]          ------------------------------------------------------------           0
XP_047128184.1[H.vulgaris]           ------------------------------------------------------------           0
XP_032220531.1[N.vectensis]          ------------------------------------------------------------           0
XP_032806710.1[P.marinus]            ------------------------------------------------------------           0
XP_019641281.1[B.belcheri]           ------------------------------------------------------------           0
XP_014017013.1[S.salar]              MAVSRRSSQQQQGN-----------------------------------------LLSPP           19
XP_018099068.1[X.laevis]             MAAPSRRSQQ----QQVPTAAAGQSSQPPSASPPLSPSS----RR----AASCLSPPCSP           48
NP_989671.2[G.gallus]                MAAPPRR----------SQ-HHHHHHGPPPPPGPASPPAAASPPRSPSLAPAELG-PAAQ           48
NP_003738.2[H.sapiens]               MAASRRSQHHHHHHQQQLQPAPGASAPPPPPPPPLSPGLAPGTTP-ASPTASGLAPFASP           59
XP_024106876.1[P.abelii]             MAASRRSQHHHHHHQQQLQPAPGASAPPPPPPPPLSPGLAPGTTP-ASPTASGLAPFASP           59
NP_780300.2[M.musculus]              MAASRRSQHHHHHHQQQLQPAPGASAPPPPPPPPLSPGLAPGPTP-ASPTAGGLAPFASP           59

XP_019848937.1[A.queenslandica]      ------------------------------------------------------------           0
NP_651410.1[D.melanogaster]          ------------------------------------------------------------           0
XP_047128184.1[H.vulgaris]           ------------------------------------------------------------           0
XP_032220531.1[N.vectensis]          ------------------------------------------------------------           0
XP_032806710.1[P.marinus]            --------------MAARRAHLASPSSSSSLSPSSGSLDTTTAAAAAAAATTTAAAAAAT           46
XP_019641281.1[B.belcheri]           ------------------------------------------------------------           0
XP_014017013.1[S.salar]              RNGSLSVSPPGSPSLNL---------VTATLVPPDGERGCSTG-----------------           53
XP_018099068.1[X.laevis]             ETNALSLSREGPDSEPEGEAASQPGITPRPLSPPPGEAANIL------------------           90
NP_989671.2[G.gallus]                RHS---------LAGPEGEA---PPDAERPPAPE--------------------------           70
NP_003738.2[H.sapiens]               RHG---------LALPEGDGSRDPPDRPRSPDPVDGTSCCSTTSTICTVAAAPVVPAVST           110
XP_024106876.1[P.abelii]             RHG---------LALPEGDGSRDPPDRPRSPDPVDGTSCCSTTSTICTVTAAPVVPAVST           110
NP_780300.2[M.musculus]              RHG---------LALPEGDGSRDPPDRPRSPDPVDGAVCTVA--------APAAVPAA--           100

XP_019848937.1[A.queenslandica]      ------------------------------------MDTRDHQAKIPGDS------RQ-T           17
NP_651410.1[D.melanogaster]          -----------------------------------------------------MANSSRS           7
XP_047128184.1[H.vulgaris]           ------------------------------------MALGRPNRAMPSRRN-----S---           16
XP_032220531.1[N.vectensis]          ---------------------------------------------MPCQAKMASKKLA--           13
XP_032806710.1[P.marinus]            TTAAAMT----------TTAAVSATSSPSTT--------------AAAAAAVAAAAATGS           82
XP_019641281.1[B.belcheri]           --------------------------------------------------------MAGR           4
XP_014017013.1[S.salar]              -----------------MENPL---ASPDLQTSALSSGSSTSPTTTTSGGGSSSVSSPGS           93
XP_018099068.1[X.laevis]             SPTSSLPPAPAPSSGSSSSS-----SS----PSSNSLCTGGSPAESPESGVIGGVSSVG-           140
NP_989671.2[G.gallus]                -CSEGAAPGPPPGSGSSSGSSASSSSSS---SSTSSSVASSPAAESPE------------           114
NP_003738.2[H.sapiens]               SSAAGVAPNPAGS---GSNNSPSSSSSPTSSSSSSPSSPGSSLAESPEAAGVSSTAPLGP           167
XP_024106876.1[P.abelii]             SSAVGVAPNPAGS---GSNNSPSSSSSPTSSSSSSPSSPGSSLAESPEAAGVSSTAPLGP           167
NP_780300.2[M.musculus]              SAAVGVAPTPAGGGGGGGNNSASSASSPTSSSSSSPSSPGSSLAESPEAAGVGSTATLGA           160

XP_019848937.1[A.queenslandica]      SQSIEEGESADKNTGDLARLFEACKNGDIDTVQNLIQQRQSSANERDLHGRKSTPLHFAA           77
NP_651410.1[D.melanogaster]          RAILSVNLDAVMANDPLRELFEACKTGEIAKVKKLIT-P-QTVNARDTAGRKSTPLHFAA           65
XP_047128184.1[H.vulgaris]           -VASNFMDGGVHENDSGRELFEACRTGDVIRVRYLIQNN-WDVNMRDTAGRKSTPLHFAA           74
XP_032220531.1[N.vectensis]          --SNPTFLSDPDVCEGGRELFEACRNGDVSKVRKLVTNN-SNVNVRDTAGRKSSPLHFAA           70
XP_032806710.1[P.marinus]            G-PGSGQDSSADGGDAGKELFEACRNGDVGRVKRLVS-P-LNVNARDTTGRKSTPLHFAA           139
XP_019641281.1[B.belcheri]           RAMLSSDRIHCDESSGCRELFEACRNGDVARVKKLIT-P-QNVNARDTAGRKSTPLHFAA           62
XP_014017013.1[S.salar]              GGTT-PGDG-SGIGGAFRELFEACRNGDVSRVKRLVD-S-VNVNAKDMAGRKSTPLHFAA           149
XP_018099068.1[X.laevis]             --IG-PGDPLSAVSGAFRELFEACRNGDVSRVRRLLE-P-ANVNAKDMAGRKSTPLHFAA           195
NP_989671.2[G.gallus]                ---------AAGPSGAFRELLEACRNGDVTRVKRLVD-A-GNVNAKDMAGRKSTPLHFAA           163
NP_003738.2[H.sapiens]               GAAG-PGTGVPAVSGALRELLEACRNGDVSRVKRLVD-A-ANVNAKDMAGRKSSPLHFAA           224
XP_024106876.1[P.abelii]             GAAG-PGTGVPAVSGALRELLEACRNGDVSRVKRLVD-A-ANVNAKDMAGRKSSPLHFAA           224
NP_780300.2[M.musculus]              GAAG-LGPGVPAVSGALRELLEACRNGDVSRVKRLVD-A-ANVNAKDMAGRKSSPLHFAA           217
                                                       .*:***:.*::  *: *:      .* :*  ****:******

XP_019848937.1[A.queenslandica]      GFGRRDIVKFLIEKGAHVDFRDEGGLIPLHNSCSFGHVDVVQLLLSNGANPNAQDNWKFS           137
NP_651410.1[D.melanogaster]          GYGRREVVEFLLNSGASIQACDEGGLHPLHNCCSFGHAEVVRLLLKAGASPNTTDNWNYT           125
XP_047128184.1[H.vulgaris]           GFGRKDIVEYLLDAGADVHARDDGGLIPLHNACSFGHVEVTLSLLRHHADVNARDNWNFT           134
XP_032220531.1[N.vectensis]          GFGRKEVVEYLLQCGADVHAMDDGGLIPLHNACSFGHAEVVRILLSHGADANARDNWNYT           130
XP_032806710.1[P.marinus]            GFGRKDVVEHLLQNGANVHARDDGGLIPLHNACSFGHAEVVNLLLRQGADPNARDNWNYT           199
XP_019641281.1[B.belcheri]           GFGRKDVVEHLLQNGANVHARDDGGLIPLHNACSFGHAEVVTLLLRNGADPNARDNWNYT           122
XP_014017013.1[S.salar]              GFGRKDVVDHLLQTGANVHARDDGGLIPLHNACSFGHAEVVSLLLCQGADPNARDNWNYT           209
XP_018099068.1[X.laevis]             GFGRKDVVEHLLQTGANVHARDDGGLIPLHNACSFGHAEVVTLLLCQGADPNARDNWNYT           255
NP_989671.2[G.gallus]                GFGRKDVVEHLLQTGANVHARDDGGLIPLHNACSFGHAEVVSLLLCQGADPNARDNWNYT           223
NP_003738.2[H.sapiens]               GFGRKDVVEHLLQMGANVHARDDGGLIPLHNACSFGHAEVVSLLLCQGADPNARDNWNYT           284
XP_024106876.1[P.abelii]             GFGRKDVVEHLLQMGANVHARDDGGLIPLHNACSFGHAEVVSLLLCQGADPNARDNWNYT           284
NP_780300.2[M.musculus]              GFGRKDVVEHLLQMGANVHARDDGGLIPLHNACSFGHAEVVSLLLCQGADPNARDNWNYT           277
                                     *:**:::*..*:: ** :.  *:*** ****.*****.:*.  **   *. *: ***:::

XP_019848937.1[A.queenslandica]      PLHEAAIKGKADVCIVLLQHGADSSLLNTDRKAPIDLANGQAREVLLGTYRQDELLEAAK           197
NP_651410.1[D.melanogaster]          PLHEAASKGKVDVCLALLQHGANHTIRNSEQKTPLELADEATRPVLTGEYRKDELLEAAR           185
XP_047128184.1[H.vulgaris]           PLHEAAIKGKVEVCIALLQHGADPLIRNTDGKTARDLAEATAKAVLTGEYKKEELLDSAR           194
XP_032220531.1[N.vectensis]          PLHEAAVKGKVDVCVVLLQHGADPNIRNTDGKTALDVAEAAAKLVLTGDYKKDELLEGAR           190
XP_032806710.1[P.marinus]            PLHEAAIKGKIDVCIVLLQHGADPGIRNTDGKTALDLAEPTAKAVLTGEYKKDELLEAAR           259
XP_019641281.1[B.belcheri]           PLHEAAIKGKIDVCIVLLQNGADPSIRNTDGKTALDLAEPSAKTVLTGDYKKDELLEAAR           182
XP_014017013.1[S.salar]              PLHEAAIKGKIDVCIVLLQHGADPNIRNTDGKSALDLADPSAKAVLTGEYKKDELLEAAR           269
XP_018099068.1[X.laevis]             PLHEASIKGKIDVCIVLLQHGADPNIRNTDGKSALDLADPSSKTVLTGEYKKDELLEAAR           315
NP_989671.2[G.gallus]                PLHEAAIKGKIDVCIVLLQHGADPNIRNTDGKSALDLADPSAKAVLTGEYKKDELLEAAR           283
NP_003738.2[H.sapiens]               PLHEAAIKGKIDVCIVLLQHGADPNIRNTDGKSALDLADPSAKAVLTGEYKKDELLEAAR           344
XP_024106876.1[P.abelii]             PLHEAAIKGKIDVCIVLLQHGADPNIRNTDGKSALDLADPSAKAVLTGEYKKDELLEAAR           344
NP_780300.2[M.musculus]              PLHEAAIKGKIDVCIVLLQHGADPNIRNTDGKSALDLADPSAKAVLTGEYKKDELLEAAR           337
                                     *****: *** :**:.***:**:  : *:: *:  ::*:  :: ** * *:::***:.*:

XP_019848937.1[A.queenslandica]      VGDEQLLMQILTPLNVNCHASDGRRSTPLHLAAGYNRTSIVQLLLKQGADVHAKDKGGLV           257
NP_651410.1[D.melanogaster]          SGAEDRLLALLTPLNVNCHASDGRRSTPLHLAAGYNRIGIVEILLANGADVHAKDKGGLV           245
XP_047128184.1[H.vulgaris]           SGCEEKMLSLITSLNVNCHAGDGRKSTPLHLAAGYNRTRIVEILLKHGADVHAKDKGGLV           254
XP_032220531.1[N.vectensis]          SGNEEKLMSLLTPLNVNCHASDGRKSTPLHLAAGYNRVGVVQLLLKHGADVHAKDKGGLV           250
XP_032806710.1[P.marinus]            SGNEEKLMALLTPLNVNCHASDGRKSTPLHLAAGYNRVRIVQLLLQHGADVHAKDKGGLV           319
XP_019641281.1[B.belcheri]           SGNEDKLMALLTPLNVNCHASDGRKSTPLHLAAGYNRVRIVQLILQHGADVHAKDKGGLV           242
XP_014017013.1[S.salar]              SGNEEKLMALLTPLNVNCHASDGRKSTPLHLAAGYNRVRIVQLLLQHGADVHAKDKGGLV           329
XP_018099068.1[X.laevis]             SGNEEKLMALLTPLNVNCHASDGRKSTPLHLAAGYNRVQIVQLLLQHGADVHAKDKGGLV           375
NP_989671.2[G.gallus]                SGNEEKLMALLTPLNVNCHASDGRKSTPLHLAAGYNRVRIVQLLLQHGADVHAKDKGGLV           343
NP_003738.2[H.sapiens]               SGNEEKLMALLTPLNVNCHASDGRKSTPLHLAAGYNRVRIVQLLLQHGADVHAKDKGGLV           404
XP_024106876.1[P.abelii]             SGNEEKLMALLTPLNVNCHASDGRKSTPLHLAAGYNRVRIVQLLLQHGADVHAKDKGGLV           404
NP_780300.2[M.musculus]              SGNEEKLMALLTPLNVNCHASDGRKSTPLHLAAGYNRVRIVQLLLQHGADVHAKDKGGLV           397
                                      * *: :: ::* *******.***:************  :*:::* :*************

XP_019848937.1[A.queenslandica]      PLHNACSYGHYEVAELLLKYGASINVTDLWQFTPLQEAASKGRSDVCSLLLAHGANPSIA           317
NP_651410.1[D.melanogaster]          PLHNACSYGHFDVTKLLIQAGANVNANDLWAFTPLHEAASKSRVEVCSLLLSRGADPTLL           305
XP_047128184.1[H.vulgaris]           PLHNACSYGHYEVTELLVKHGASVNAMDLWQFTPLHEAASKMRIEVCTLLLSYGADPTVL           314
XP_032220531.1[N.vectensis]          PLHNACSYGHFEVTELLIKHGASVNAMDLWQFTPLHEAASKARVEVCALLLGHGADPTLL           310
XP_032806710.1[P.marinus]            PLHNACSYGHYEVTELLVKHGASVNAMDLWQFTPLHEAASKNRVEVCSLLLSYGADPTLL           379
XP_019641281.1[B.belcheri]           PLHNACSYGHFEVTELLLKHGASVNAMDLWQFTPLHEAASKSRIEVCSLLLSHGADPTLL           302
XP_014017013.1[S.salar]              PLHNACSYGHFEVTELLLKHGACVNAMDLWQFTPLHEAASKNRVEVCSLLLSHGADPTLL           389
XP_018099068.1[X.laevis]             PLHNACSYGHFEVTESLLKHGACVNAMDLWQFTPLHEAASKNRVEVCSLLLSHGADPTLV           435
NP_989671.2[G.gallus]                PLHNACSYGHYEVTELLLKHGACVNAMDLWQFTPLHEAASKNRVEVCSLLLSHGADPTLV           403
NP_003738.2[H.sapiens]               PLHNACSYGHYEVTELLLKHGACVNAMDLWQFTPLHEAASKNRVEVCSLLLSHGADPTLV           464
XP_024106876.1[P.abelii]             PLHNACSYGHYEVTELLLKHGACVNAMDLWQFTPLHEAASKNRVEVCSLLLSHGADPTLV           464
NP_780300.2[M.musculus]              PLHNACSYGHYEVTELLLKHGACVNAMDLWQFTPLHEAASKNRVEVCSLLLSHGADPTLV           457
```

```
                                     **********::*:: *:: ** :*. *** ****:***** * :**:***. **:*::

XP_019848937.1[A.queenslandica]      NCHGKTAFNLAPSEEFRKKLDSEYRGYQLLAAAEDGGIILLKKLLSSQLLKFQHHQTLDT         377
NP_651410.1[D.melanogaster]          NCHSKSAIDAAPTRELRERIAFEYKGHCLLDACRKCDVSRAKKLVCAEIVNFVHPYTGDT         365
XP_047128184.1[H.vulgaris]           NCHSKTAIDVAPVQELKDKLLYEYRGHTYLEAVKSSDLSKIKRIIAPDLVNFVHPLTLNS         374
XP_032220531.1[N.vectensis]          NCHSKSAIDAAPTKELQEKLLSEFKGHQILEAAKGCDTAKIKKLITTETINFQHPLTLDT         370
XP_032806710.1[P.marinus]            NCHSKSAIDLAPTSELKERLVYEFRGHSLLQAAREADVTRVKKHLGLDIINFKHPQTHET         439
XP_019641281.1[B.belcheri]           NCHSKSAIDVASTPELQEKLSYEFKGHCLLDGARQTDMAKVKKHLQLDIVNFKHPYTHDT         362
XP_014017013.1[S.salar]              NCHSKSAVDMAPTPELKERLTYEFKGHSLLQAAREADMAKVKKTLALEIISFKHPQTNET         449
XP_018099068.1[X.laevis]             NCHGKSAVDMAPTPELKERLSYEFKGHSLLQAAREADLAKVKKTLALEIINFKQPQSHET         495
NP_989671.2[G.gallus]                NCHGKSAVDMAPTPELRERLTYEFKGHSLLQAAREADLAKVKKTLALEIINFKQPQSHET         463
NP_003738.2[H.sapiens]               NCHGKSAVDMAPTPELRERLTYEFKGHSLLQAAREADLAKVKKTLALEIINFKQPQSHET         524
XP_024106876.1[P.abelii]             NCHGKSAVDMAPTPELRERLTYEFKGHSLLQAAREADLAKVKKTLALEIINFKQPQSHET         524
NP_780300.2[M.musculus]              NCHGKSAVDMAPTPELRERLTYEFKGHSLLQAAREADLAKVKKTLALEIINFKQPQSHET         517
                                     ***.*:*.: *   *::.::  *::*:  * . .   .    *: :  : :.* :  : ::

XP_019848937.1[A.queenslandica]      LLHKAVLSKSSNRQSVIDALLKRGINLNIGNKENMTPLICAAKKGILEVVEQLVQRGANI         437
NP_651410.1[D.melanogaster]          PLHLAVVSPDGKRKQLMELLTRKGSLLNEKNKAFLTPLHLAAELLHYDAMEVLLKQGAKV         425
XP_047128184.1[H.vulgaris]           ALHVCALSTSAKRDRVADFLIKKGCNMNFCNKQKLTPLHLCAGNDNVVVTEILIKAGADC         434
XP_032220531.1[N.vectensis]          PLHVAVASSSPKKKGVIETLLKKGADPNLQNKSSYAPLHVAAEKGLFEAMELLLKHGAKV         430
XP_032806710.1[P.marinus]            ALHCAVASPYPKRKQVSELLLRKGANVNEKTKDLLTPLHVASDKAHNDVIDVLLKHGAKI         499
XP_019641281.1[B.belcheri]           ALHCAALSPYPKRRQITELLIRKGANLNDKNKEFLTPLHVASDKCYIDVMEVLLKHGAKV         422
XP_014017013.1[S.salar]              ALHCAVASPHPKRKQVTELLLRKGANINEKNKDFMTALHVASERAHNDILEVLQKHGAKV         509
XP_018099068.1[X.laevis]             ALHCAVASLHPKRKQITELLLRKGASVNEKNKDFMTPLHVAAERAHNDVVEVLHKHGAKM         555
NP_989671.2[G.gallus]                ALHCAVAAVHPKRKQVTELLLRKGANVNEKNKDFMTPLHVAAEKAHNDVMEVLHKHGAKM         523
NP_003738.2[H.sapiens]               ALHCAVASLHPKRKQVTELLLRKGANVNEKNKDFMTPLHVAAERAHNDVMEVLHKHGAKM         584
XP_024106876.1[P.abelii]             ALHCAVASLHPKRKQVTELLLRKGANVNEKNKDFMTPLHVAAERAHNDVMEVLHKHGAKM         584
NP_780300.2[M.musculus]              ALHCAVASLHPKRKQVAELLLRKGANVNEKNKDFMTPLHVAAERAHNDVMEVLHKHGAKM         577
                                      ** .. :   ::  : : * ::*   *  .*   : *  .:        : * : **.

XP_019848937.1[A.queenslandica]      NHQDINGMTSLHWAVQNEHAQICRYLLSSGANPSIVNNQGQTIYQLKTSDTIQLILKN-E         496
NP_651410.1[D.melanogaster]          NALDSLGQTPLHRCARD--EQAVRLLLSYAADTNIVSLEGLTAAQLASDSVLKLLKNP--         481
XP_047128184.1[H.vulgaris]           NIVNESLQSPLHIAAQSGHAHICYILLMNGADPTIRNSSGYTPAQVGTESVQKIFQARL-         493
XP_032220531.1[N.vectensis]          NAIDSAGQTALHVAAIAGQVQACRVLMSNGADPTCQTFQGYTAFEVAPEPVQKVLHVNEE         490
XP_032806710.1[P.marinus]            NVTDSLGQTSLHRAAHAGHLTTCRLLLSHGCDPNLPSLQGFTAAQLGNESVQQILHES-S         558
XP_019641281.1[B.belcheri]           NALDSLGQTALHRGAHCGHVQACRLLLSFGVDPSIVSLQGVTAAQMATEAVQQMLHED--         480
XP_014017013.1[S.salar]              NAVDTLGQTALHRAALAGHIQTCRLLLSYGADPAIVSLQGFTASQMGNEAVQQILNEN--         567
XP_018099068.1[X.laevis]             NALDTLGQTALHRAALGGHLQTCRLLLSFGSDASIVSLQGFTAAQMGNEAVQQILNES--         613
NP_989671.2[G.gallus]                NALDTLGQTALHRAALAGHLQTCRLLLNYGSDPSIISLQGFTAAQIGNEAVQQILSES--         581
NP_003738.2[H.sapiens]               NALDTLGQTALHRAALAGHLQTCRLLLSYGSDPSIISLQGFTAAQMGNEAVQQILSES--         642
XP_024106876.1[P.abelii]             NALDTLGQTALHRAALAGHLQTCRLLLSYGSDPSIISLQGFTAAQMGNEAVQQILSES--         642
NP_780300.2[M.musculus]              NALDSLGQTALHRAALAGHLQTCRLLLSYGSDPSIISLQGFTAAQMGNEAVQQILSES--         635
                                     *  :    : **  .           *:  . :    . .* *  ::  . . :::

XP_019848937.1[A.queenslandica]      PPVSQFEIEQQLLEAARNSDLEILKKI--CTPQNVNCRDTKGRMSTPLHFAAGYNRVTVV         554
NP_651410.1[D.melanogaster]          -----PDSETHLLEAAKAGDLDTVRRIVLNNPISVNCRDLDGRHSTPLHFAAGFNRVPVV         536
XP_047128184.1[H.vulgaris]           -EPMLKDVDINLLEASKTGDFELIQSL--CTPQNVNCRDVAGRMSTPLHFASGYNRMDIV         550
XP_032220531.1[N.vectensis]          SPSPVSDAEKQLLEAAKTGDLETVKNL--CTAQTVNCRDLDGRCSTPLHFAAGYNRVRVV         548
XP_032806710.1[P.marinus]            LPVRNSDVEHQLLEAAKAGDLDTVKQL--CTTQTVNCRDMEGRHSTPLHFAAGYNRVSVV         616
XP_019641281.1[B.belcheri]           PPSGPADECDQLLEAAKAGDLEAVKVL--CNPHTVNCRDVNGRHSTPLHFAAGYNRVAVV         538
XP_014017013.1[S.salar]              VPTRNSDVDYRFLEAAKAGDLDTVQQL--CTPQNVNCRDLEGRHSTPLHFAAGYNRVAVV         625
XP_018099068.1[X.laevis]             TPVRTSDVDYRLLEASKAGDLDIVKQL--CSSQNVNCRDLEGRHSTPLHFAAGYNRVSVV         671
NP_989671.2[G.gallus]                TPVRTSDVDYRLLEASKAGDLETVKQL--CSPQNVNCRDLEGRHSTPLHFAAGYNRVSVV         639
NP_003738.2[H.sapiens]               TPIRTSDVDYRLLEASKAGDLETVKQL--CSSQNVNCRDLEGRHSTPLHFAAGYNRVSVV         700
XP_024106876.1[P.abelii]             TPIRTSDVDYRLLEASKAGDLETVKQL--CSPQNVNCRDLEGRHSTPLHFAAGYNRVSVV         700
NP_780300.2[M.musculus]              TPMRTSDVDYRLLEASKAGDLETVKQL--CSPQNVNCRDLEGRHSTPLHFAAGYNRVSVV         693
                                           :   .:***:: .*:: :: :   .  .*****  ** *******:*:**: :*

XP_019848937.1[A.queenslandica]      EFLLENGADVHAKDKGGLVPLHNACSYGHYEVAELLVKYKANVNAMDLWKFTPLHEAAAK         614
NP_651410.1[D.melanogaster]          QFLLEHGAEVYAADKGGLVPLHNACSYGHYEVTELLVKHGANVNVSDLWKFTPLHEAAAK         596
XP_047128184.1[H.vulgaris]           EHLLRCGADVHAKDKGGLVPLHNACSYGHFEIAEILVRHGANVNAADYWKFTPLHEAAAK         610
XP_032220531.1[N.vectensis]          EYLLLNGADVHAKDKGGLVPLHNACSYGHYEVAELLVKHGAIVNVADLWKFTPLHEAAAK         608
XP_032806710.1[P.marinus]            EYLLQHGADVHAKDKGGLVPLHNACSYGHYEVAELLVKRGAVVNVADLWKFTPLHEAAAK         676
XP_019641281.1[B.belcheri]           EYLLQHGADVHAKDKGGLVPLHNACSYGHYEVCELLLKHGAVVNVADLWKFTPLHEAAAK         598
XP_014017013.1[S.salar]              EYLLHHGADVHAKDKGGLVPLHNACSYGHYEVAELLVRHGASVNVADLWKFTPLHEAAAK         685
XP_018099068.1[X.laevis]             EYLLHHGADVHAKDKGGLVPLHNACSYGHYEVAELLVRHGASVNVADLWKFTPLHEAAAK         731
NP_989671.2[G.gallus]                EYLLHHGADVHAKDKGGLVPLHNACSYGHYEVAELLVRHGASVNVADLWKFTPLHEAAAK         699
NP_003738.2[H.sapiens]               EYLLHHGADVHAKDKGGLVPLHNACSYGHYEVAELLVRHGASVNVADLWKFTPLHEAAAK         760
XP_024106876.1[P.abelii]             EYLLHHGADVHAKDKGGLVPLHNACSYGHYEVAELLVRHGASVNVADLWKFTPLHEAAAK         760
NP_780300.2[M.musculus]              EYLLHHGADVHAKDKGGLVPLHNACSYGHYEVAELLVRHGASVNVADLWKFTPLHEAAAK         753
                                     :.**  **:*:* ****************:*: *:*::  * **. * ************

XP_019848937.1[A.queenslandica]      EKYDICKLLLKNGANVHSKNRDNLTPIDLVKDPKSDLADLLRGEPALLDAAKKGEIERVK         674
NP_651410.1[D.melanogaster]          GKYDICKLLLKHGADPMKKNRDGATPADLVKESDHDVAELLRGPSALLDAAKKGNLARVQ         656
XP_047128184.1[H.vulgaris]           GKFDICKLLLKHGADPKKKNHEGYTPLDLVKEGFPDVADLLRGDAALLEAAKKGNLAKVM         670
XP_032220531.1[N.vectensis]          GKYEICQLLLKHGADPQKKNRDGYTPLDLVKEGDSDVADLLRGDAALLDAAKKGNLIRIT         668
XP_032806710.1[P.marinus]            GKYEICKLLLKHGADPTKKNRDGNTPLDLVKEGDTDIQDLLRGDAALLDAAKKGCLARVQ         736
XP_019641281.1[B.belcheri]           GKYEICKLLLKHGADPNKKNRDGNTPLDLVRDGDTDIQDLLRGDAALLDAAKKGNLARVQ         658
XP_014017013.1[S.salar]              GKYEICKLLLKHGADPTKKNRDGNMPLDMVKDGDTDIQDLLRGDAALLDAAKKGCLARVQ         745
XP_018099068.1[X.laevis]             GKYEICKLLLKHGADPTKKNRDGNTPLDLVKDGDTDIQDLLRGDAALLDAAKKGCLARVQ         791
NP_989671.2[G.gallus]                GKYEICKLLLKHGADPTKKNRDGNTPLDLVKEGDTDIQDLLRGDAALLDAAKKGCLARVQ         759
NP_003738.2[H.sapiens]               GKYEICKLLLKHGADPTKKNRDGNTPLDLVKEGDTDIQDLLRGDAALLDAAKKGCLARVQ         820
XP_024106876.1[P.abelii]             GKYEICKLLLKHGADPTKKNRDGNTPLDLVKEGDTDIQDLLRGDAALLDAAKKGCLARVQ         820
NP_780300.2[M.musculus]              GKYEICKLLLKHGADPTKKNRDGNTPLDLVKEGDTDIQDLLRGDAALLDAAKKGCLARVQ         813
                                      *::**:****:**:  .**::.  * *:*::   *: :****  ***:***** : ::

XP_019848937.1[A.queenslandica]      KLLTEDNVNCRDEYGRNSTPLHLAAGYNHLDVVEYLLENKADVNAKDKGGLVPLHNASSY         734
NP_651410.1[D.melanogaster]          RLVTPESINCRDAQGRNSTPLHLAAGYNNFECAEYLLENGADVNAQDKGGLIPLHNASSY         716
XP_047128184.1[H.vulgaris]           KLVTHQNINCRDTLGRNSTPLHLAAGYNHVEVAEYLLEFGADVNAQDKGGLIPLHNASSY         730
XP_032220531.1[N.vectensis]          KLATPENINCRDTQGRNSTPLHLAAGYNHLEVAEYLLEHGADVNAQDKGGLIPLHNASSY         728
XP_032806710.1[P.marinus]            KLCSGENVNCRDSQGRNSTPLHLAAGYNNLEVAEYLLEHGADVNAQDKGGLIPLHNAASY         796
XP_019641281.1[B.belcheri]           KLATPENINCRDTQGRNSTPLHLAAGYNNVEVAEFLLENGADVNAQDKGGLIPLHNASSY         718
XP_014017013.1[S.salar]              KLCSPENINCRDTQGRNSTPLHLAAGYNNLEVAEYLLEHGADVNAQDKGGLIPLHNAASY         805
XP_018099068.1[X.laevis]             KLCTQENINCRDTQGRNSTPLHLAAGYNNLEVAEYLLEHGADVNAQDKGGLIPLHNAASY         851
NP_989671.2[G.gallus]                KLCTQENINCRDTQGRNSTPLHLAAGYNNLEVAEYLLEHGADVNAQDKGGLIPLHNAASY         819
NP_003738.2[H.sapiens]               KLCTPENINCRDTQGRNSTPLHLAAGYNNLEVAEYLLEHGADVNAQDKGGLIPLHNAASY         880
XP_024106876.1[P.abelii]             KLCTPENINCRDTQGRNSTPLHLAAGYNNLEVAEYLLEHGADVNAQDKGGLIPLHNAASY         880
NP_780300.2[M.musculus]              KLCTPENINCRDTQGRNSTPLHLAAGYNNLEVAEYLLEHGADVNAQDKGGLIPLHNAASY         873
                                     :* : :.:****  **************:.: .*:***  *****:*****:*****:**

XP_019848937.1[A.queenslandica]      GHVDVASLLIRYNSVINATDRWNFTPLHEAAQKGRTQVCSLLIIHGADVYLKNQEGQIPL         794
NP_651410.1[D.melanogaster]          GHLDIAALLIKHKTVVNATDKWGFTPLHEAAQKGRTQLCSLLLAHGADAYMKNQEGQTPI         776
XP_047128184.1[H.vulgaris]           GHVDIAALLIKYKADVNATDRWLFTPLHEAAQKGRTQLCALLLTHGANANMKNQEGQTPI         790
XP_032220531.1[N.vectensis]          GHVDIAALLIRYNTDVNATDRWLFTPLHEAAQKGRTQLCALLLAHGAEPSMKNQEGQSPV         788
XP_032806710.1[P.marinus]            GHVDIAALLIKYSACVNATDKWAFTPLHEAAQKGRTQLCALLLAHGADPSMKNQEGQTPL         856
XP_019641281.1[B.belcheri]           GHVEIAQLLIKYGTCVNATDRWNFTPLHEAAQKGRTQLCALLLAHGADPTMKNQEGQTPL         778
XP_014017013.1[S.salar]              GHVDIAALLIKFNTCVNATDKWAFTPLHEAAQKGRTQLCALLLAHGADPTMKNQEGQTAL         865
XP_018099068.1[X.laevis]             GHVDIAALLIKYNTCVNATDKWAFTPLHEAAQKGRTQLCALLLAHGADPTMKNQESQTPL         911
NP_989671.2[G.gallus]                GHVDIAALLIKYNTCVNATDKWAFTPLHEAAQKGRTQLCALLLAHGADPTMKNQEGQTPL         879
NP_003738.2[H.sapiens]               GHVDIAALLIKYNTCVNATDKWAFTPLHEAAQKGRTQLCALLLAHGADPTMKNQEGQTPL         940
XP_024106876.1[P.abelii]             GHVDIAALLIKYNTCVNATDKWAFTPLHEAAQKGRTQLCALLLAHGADPTMKNQEGQTPL         940
NP_780300.2[M.musculus]              GHVDIAALLIKYNTCVNATDKWAFTPLHEAAQKGRTQLCALLLAHGADPTMKNQEGQTPL         933
                                     **:::* ***:. : :****:* **************:*:**: ***:  :****.*  :
```

```
XP_019848937.1[A.queenslandica]      DLATADDVIALLQDAMMKDIPLT-IPPAEKEAKSN-------I----------------V           830
NP_651410.1[D.melanogaster]          ELATADDVKCLLQDAMATSLSQQALSASTQSLTSS--------------------SPAPD           816
XP_047128184.1[H.vulgaris]           DLATADDVQVLLQDSMVSKSEVMQITSNTCS-----------------------------           821
XP_032220531.1[N.vectensis]          DLATAEDVKCLLGDAMLSTQTTSVSTAPVAKVPA------------------------PV           824
XP_032806710.1[P.marinus]            DLATADDVRALLIDAMPPEALPVTHKPPSIPTIAATSAAATAALALGPTPALASLAPSPV           916
XP_019641281.1[B.belcheri]           DLATAEDVRALLVDAMPPQSLPAAITAAT-VKAAS-------------------------           812
XP_014017013.1[S.salar]              DLATADDIRALLMDAMPPDALPSCFKPQATVVSAG-------------------------           900
XP_018099068.1[X.laevis]             DLATADDIRALLIDAMPPEALPSCFKPQATVVSA--------------------------           945
NP_989671.2[G.gallus]                DLATADDIRALLIDAMPPEALPTCFKPQATVVSA--------------------------           913
NP_003738.2[H.sapiens]               DLATADDIRALLIDAMPPEALPTCFKPQATVVSA--------------------------           974
XP_024106876.1[P.abelii]             DLATADDIRALLIDAMPPEALPTCFKPQATVVSA--------------------------           974
NP_780300.2[M.musculus]              DLATADDIRALLIDAMPPEALPTCFKPQATVVSA--------------------------           967
                                     :****:*:  ** *:*

XP_019848937.1[A.queenslandica]      NKGLTAAGA-----SLLAS------ELVLGDGVDDKNGMVT------------------           860
NP_651410.1[D.melanogaster]          ATAAAAPGTSSSSSSAILSPTTETVLLPTGAS--------MILSVPVPLPLSSST-----           863
XP_047128184.1[H.vulgaris]           ----------------IKSPINSPQVLPISNDLSPQLEPQ---------GLTKPSTANQN           856
XP_032220531.1[N.vectensis]          TATA-------GAATPATGPISGMAPLSQAN----------------------------S           849
XP_032806710.1[P.marinus]            PSGASASGA--GGVPLLASPSSTPASLSAASSMDSLTGACGVAPSPVPAGAIGAGAAGDG           974
XP_019641281.1[B.belcheri]           -----------PISSPSTTPSSTPGVLSTASSMDHLVGAVG-----------GA-----           844
XP_014017013.1[S.salar]              -----------S----VISPASTPSCLSAASSIDNLAGPLNELGAA------GTS-----           934
XP_018099068.1[X.laevis]             -----------S----IISPPSTPSCLSAASSIDNLTGPLAELAVG------GVS-----           979
NP_989671.2[G.gallus]                -----------S----LISPASTPSCLSAASSIDNLTGPLAELAVG------GAS-----           947
NP_003738.2[H.sapiens]               -----------S----LISPASTPSCLSAASSIDNLTGPLAELAVG------GAS-----           1008
XP_024106876.1[P.abelii]             -----------S----LISPASTPSCLSAASSIDNLTGPLAELAVG------GAS-----           1008
NP_780300.2[M.musculus]              -----------S----LISPASTPSCLSAASSIDNLTGPLTDLAVG------GAS-----           1001
                                                               *  .

XP_019848937.1[A.queenslandica]      -QQRGGPAGVGDGSDKGFMS------YRGGGDAPPPWHNVTVKDILTELELGHLVELFER           913
NP_651410.1[D.melanogaster]          -RISPAQGAEANGAEGSSS-----D-----DLLPDADTITNVSGFLSSQQLHHLIELFER           912
XP_047128184.1[H.vulgaris]           -LLLSEP-CNSDGLQ---------TKRLRDQAEGTNPLDIDIGTFLSSLQLEFLHEIFSR           905
XP_032220531.1[N.vectensis]          SLLANSPAGHGDGAFSNRSVSEAQGEAVRSISAVHPGLDVDVGQFLDGLQLNNLKEIFER           909
XP_032806710.1[P.marinus]            TLLGACGVSAADGATGPA------DKPDRSADFEIPGLDMNINMFLKGLGLDHLRDIFEK           1028
XP_019641281.1[B.belcheri]           --------GQGDGAIDR-----------SAVEGE-SLLDMTISSFLQQLGLNQLLDIFNK           884
XP_014017013.1[S.salar]              --------GVADGATGS-----------DRKEGELVMLDMNISQFLKSLGLEHLRDIFER           975
XP_018099068.1[X.laevis]             --------NAGDGAAGT-----------ERKEGEVTVLDININQFLKSLGLEHLRDIFET           1020
NP_989671.2[G.gallus]                --------NTGDGAAGT-----------ERKEGEVSGLDMNITQFLKSLGLEHLRDIFET           988
NP_003738.2[H.sapiens]               --------NAGDGAAGT-----------ERKEGEVAGLDMNISQFLKSLGLEHLRDIFET           1049
XP_024106876.1[P.abelii]             --------NAGDGAAGT-----------ERKEGEIAGLDMNISQFLKSLGLEHLRDIFET           1049
NP_780300.2[M.musculus]              --------NAGDGAAGA-----------ERKEGEVAGLDMNISQFLKSLGLEHLRDIFET           1042
                                               .:*                            :  :*    *  * ::*.

XP_019848937.1[A.queenslandica]      EQITIDILIEMNGDDLQSIGITAFGVRHRLLKRIRELVQGNNEE------YPVGVTTTKP           967
NP_651410.1[D.melanogaster]          EQITLDILAEMGHDDLKQVGVSAYGFRHKILKGIAQLRSTTGIGN------------NV           959
XP_047128184.1[H.vulgaris]           EQITMDVLIEMGPDQLKEIGIVAYGHRHKILKGVKERLAGAGCTIV--G-EPFLSNINAS           962
XP_032220531.1[N.vectensis]          EQISWDVLVDMGHEELKEIGIHAYGHRHKILKAVKEKISGMGLGL-----GPFS-T--SQ           961
XP_032806710.1[P.marinus]            EQITLDVLADMGHEELKEIGINAYGHRHKLIKGVERLLGAQQGGLAYPGANPYL-SYHTT           1087
XP_019641281.1[B.belcheri]           EQITLDILGEMGHEELKEIGVNAYGHRHKLIKGVERILGGTGT-----TLNPYL-TPALG           938
XP_014017013.1[S.salar]              EQISLDVLADMGHEELKEIGINAYGHRHKLIKGVERLLGGQQ------GANPYL-TFHCA           1028
XP_018099068.1[X.laevis]             EQITLDVLADMGHEELKEIGINAYGHRHKLIKGVERLLGGQQ------GTNPYL-TFHCV           1073
NP_989671.2[G.gallus]                EQITLDVLADMGHEELKEIGINAYGHRHKLIKGVERLLGGQQ------GTNPYL-TFHCV           1041
NP_003738.2[H.sapiens]               EQITLDVLADMGHEELKEIGINAYGHRHKLIKGVERLLGGQQ------GTNPYL-TFHCV           1102
XP_024106876.1[P.abelii]             EQITLDVLADMGHEELKEIGINAYGHRHKLIKGVERLLGGQQ------GTNPYL-TFHCV           1102
NP_780300.2[M.musculus]              EQITLDVLADMGHEELKEIGINAYGHRHKLIKGVERLLGGQQ------GTNPYL-TFHCV           1095
                                     ***: *:* :*. ::*:.:*: *:* **:::* : .

XP_019848937.1[A.queenslandica]      TQGTQLIELSSDDKEFIDTADLMQSTICEHRDDGKAGGVFDSYEILKIERIVNTKVWERY           1027
NP_651410.1[D.melanogaster]          NLCTLLVDLLPDDKEFVAVEEEMQATIREHRDNGQAGGYFTRYNIIRVQKVQNRKLWERY           1019
XP_047128184.1[H.vulgaris]           GPNTILQELSMDEKDYQSVAEEMQSTVVEHKDGGVAGGIFSRYSSLKIERVINKKLWDKY           1022
XP_032220531.1[N.vectensis]          VQGSVIQELSMVDKDFASVADEMQSTIVEHRDSGTAGGVFTSYTILKIERVVNTKLWEKY           1021
XP_032806710.1[P.marinus]            AQGTLLIDLPPDDKEYQSVEDEMQSTIREHKDGGQAGGVFNRYNVIKIQKVRNKKLWERY           1147
XP_019641281.1[B.belcheri]           SHGTILTDLSPDDKEYQSVEEEVQSTIRQHKDQGQAGGIFNRYNVIKIQKVRNKRLWDRY           998
XP_014017013.1[S.salar]              NQGTVLIDLAPDDKEGQSVEEEMQSTIREHRDGGNAGGVFSRYNIIKIQKVVNKKLRERY           1088
XP_018099068.1[X.laevis]             SQGTVLLDLASDDKEYQSVEEEMQNSIREHRDGGNAGGVFNRYNVIRIQKVVNKKLRERF           1133
NP_989671.2[G.gallus]                SQGTILLDLAPDDKEYQSVEEEMQSTIREHRDGGNAGGIFNRYNVIRIQKVVNKKLRERF           1101
NP_003738.2[H.sapiens]               NQGTILLDLAPEDKEYQSVEEEMQSTIREHRDGGNAGGIFNRYNVIRIQKVVNKKLRERF           1162
XP_024106876.1[P.abelii]             NQGTILLDLAPEDKEYQSVEEEMQSTIREHRDGGNAGGIFNRYNVIRIQKVVNKKLRERF           1162
NP_780300.2[M.musculus]              NQGTILLDLAPEDKEYQSVEEEMQSTIREHRDGGNAGGIFNRYNVIRIQKVVNKKLRERF           1155
                                        : : :*   :*:   . : :* :: :*:* * *** *  *  :::::: * :: :::

XP_019848937.1[A.queenslandica]      KYRRKEVAESNNNCANELMLFHGSPFVPYIVHNGFDERHAYIGGMFGAGIYFAEHSSKSN           1087
NP_651410.1[D.melanogaster]          AHRRQEIAEENFLQSNERMLFHGSPFINAIVQRGFDERHAYIGGMFGAGIYFAEHSSKSN           1079
XP_047128184.1[H.vulgaris]           YYQRKQVADANHNHPNERMLFHGSPFVNAIVQKGFDERHAYIGGMFGAGIYFAEHSSKSN           1082
XP_032220531.1[N.vectensis]          VYRRREIADSNHNHANERMLFHGSPFINAIVQKGFDQRHAYIGGMFGAGIYFAENSSKSN           1081
XP_032806710.1[P.marinus]            THRRKEVTEENHNHHNERMLFHGSPFINAIINKGFDERHAYIGGMFGAGIYFAENSSKSN           1207
XP_019641281.1[B.belcheri]           VHRRKEVSEENHNQSNERMLFHGSPFINAIINKGFDERHAYIGGMFGAGIYFAENSSKSN           1058
XP_014017013.1[S.salar]              THRQKEISDENHNHHNERMLFHGSPFINAIIHKGFDERHAYIGGMFGAGIYFAENSSKSN           1148
XP_018099068.1[X.laevis]             CHRQKEVSEENHNHHNERMLFHGSPFINAIIHKGFDERHAYIGGMFGAGIYFAENSSKSN           1193
NP_989671.2[G.gallus]                CHRQKEVSEENHNHHNERMLFHGSPFINAIIHKGFDERHAYIGGMFGAGIYFAENSSKSN           1161
NP_003738.2[H.sapiens]               CHRQKEVSEENHNHHNERMLFHGSPFINAIIHKGFDERHAYIGGMFGAGIYFAENSSKSN           1222
XP_024106876.1[P.abelii]             CHRQKEVSEENHNHHNERMLFHGSPFINAIIHKGFDERHAYIGGMFGAGIYFAENSSKSN           1222
NP_780300.2[M.musculus]              CHRQKEVSEENHNHHNERMLFHGSPFINAIIHKGFDERHAYIGGMFGAGIYFAENSSKSN           1215
                                      :::::::: *    ** ********:  *::.***:*****************:*****

XP_019848937.1[A.queenslandica]      QYVYGIGGGNGCPEHKNRSCYTCLRKLLLCRVVLGKPVEQYTAVRIAHAPPGHHSVIGRP           1147
NP_651410.1[D.melanogaster]          QYVYGIGGGIGCPSHKDKSCYVCPRQLLLCRVALGKSFLQYSAMKMAHAPPGHHSVVGRP           1139
XP_047128184.1[H.vulgaris]           QYVYGIGGGSGCPVHKDRSCYDCKRQIILCRVTLGKPFFQNSAMKMAHAPPGHHSVIGRP           1142
XP_032220531.1[N.vectensis]          QYVYGIGGGSGCPTHKDRSCYSCERQLLLCRVALGKPFYQFSAVKMAHAPPGHNSVIGRP           1141
XP_032806710.1[P.marinus]            QYVYGIGGGTGCPVHKDRSCYICQRQMLFCRVTLGKSFLQFSAMKMAHSPPGHHSVIGRP           1267
XP_019641281.1[B.belcheri]           QYVYGIGGGTGCPIHKDRSCYICQRQMLFCRVTLGKAFLQFSAMKMAHAPPGHHSVIGRP           1118
XP_014017013.1[S.salar]              QYVYGIGGGTGCPTHKDRSCYLCHRQMLFCRVTLGKSFLQFSAMKMAHAPPGHHSVIGRP           1208
XP_018099068.1[X.laevis]             QYVYGIGGGTGCPTHKDRSCYICHRQMLFCRVTLGKSFLQFSTMKMAHAPPGHHSVIGRP           1253
NP_989671.2[G.gallus]                QYVYGIGGGTGCPTHKDRSCYICHRQMLFCRVTLGKSFLQFSTMKMAHAPPGHHSVIGRP           1221
NP_003738.2[H.sapiens]               QYVYGIGGGTGCPTHKDRSCYICHRQMLFCRVTLGKSFLQFSTMKMAHAPPGHHSVIGRP           1282
XP_024106876.1[P.abelii]             QYVYGIGGGTGCPTHKDRSCYICHRQMLFCRVTLGKSFLQFSTMKMAHAPPGHHSVIGRP           1282
NP_780300.2[M.musculus]              QYVYGIGGGTGCPTHKDRSCYICHRQMLFCRVTLGKSFLQFSTMKMAHAPPGHHSVIGRP           1275
                                     ********* *** **::*** * *::::***.*** . * :::::**:****:**:***

XP_019848937.1[A.queenslandica]      SAGGLNYPEYVIYRGEQAYPEYIITFRIKKPSATDSMSSSSSLDMSNNT           1196
NP_651410.1[D.melanogaster]          SAGGLHFAEYVVYRGEQSYPEYLITYQIVKPDDSSSGTEDTR-------           1181
XP_047128184.1[H.vulgaris]           NSGGLSYPEYVVYRGEQAYPEYLITYKIERPNTLSSCQSAVD-------           1184
XP_032220531.1[N.vectensis]          SSGGLSFAEYVIYRGEQAYPEYLITYKINKPDT----------------           1174
XP_032806710.1[P.marinus]            SVNGLAFAEYVIYRGEQAYPEYLITYQILKPECTPDLTSAAGQKS----           1312
XP_019641281.1[B.belcheri]           SVGGLNFAEYVIYRGEQAFPEYLITYQIVKPEAPAAPSPDQK-------           1160
XP_014017013.1[S.salar]              SVNGLAYAEYVIYRGEQAYPEYLITYQILKPESTATSAAGEDQKS----           1253
XP_018099068.1[X.laevis]             SVNGLAYAEYVIYRGEQAYPEYLITYQIMKPETPLQAATTAEQKT----           1298
NP_989671.2[G.gallus]                SVNGLAYAEYVIYRGEQAYPEYLITYQIVKPEAPSQTGTAAEQKT----           1266
NP_003738.2[H.sapiens]               SVNGLAYAEYVIYRGEQAYPEYLITYQIMKPEAPSQTATAAEQKT----           1327
XP_024106876.1[P.abelii]             SVNGLAYAEYVIYRGEQAYPEYLITYQIMKPEAPSQTATAAEQKT----           1327
NP_780300.2[M.musculus]              SVNGLAYAEYVIYRGEQAYPEYLITYQIMKPEAPSQTATAAEQKT----           1320
                                     . .** : ***:*****::***:**::* :*.
```

Percent identity matrix:

```
#
#
#  Percent Identity  Matrix - created by Clustal2.1
#
#

     1: XP_019848937.1[A.queenslandica]  100.00   56.61   56.26   60.24   58.20   58.78   57.98   57.87   58.99   58.15   58.24   58.33
     2: NP_651410.1[D.melanogaster]       56.61  100.00   60.37   64.62   65.53   68.43   66.78   66.17   67.32   66.70   66.78   66.87
     3: XP_047128184.1[H.vulgaris]        56.26   60.37  100.00   68.01   64.65   65.41   64.30   63.79   65.21   64.59   64.68   64.77
     4: XP_032220531.1[N.vectensis]       60.24   64.62   68.01  100.00   70.41   71.77   71.65   70.27   71.72   71.42   71.42   71.50
     5: XP_032806710.1[P.marinus]         58.20   65.53   64.65   70.41  100.00   79.64   81.58   80.45   83.01   80.58   80.50   81.23
     6: XP_019641281.1[B.belcheri]        58.78   68.43   65.41   71.77   79.64  100.00   78.66   79.04   80.28   79.44   79.53   79.53
     7: XP_014017013.1[S.salar]           57.98   66.78   64.30   71.65   81.58   78.66  100.00   86.51   87.95   86.97   87.05   86.47
     8: XP_018099068.1[X.laevis]          57.87   66.17   63.79   70.27   80.45   79.04   86.51  100.00   90.73   89.50   89.35   88.67
     9: NP_989671.2[G.gallus]             58.99   67.32   65.21   71.72   83.01   80.28   87.95   90.73  100.00   93.42   93.42   93.04
    10: NP_003738.2[H.sapiens]            58.15   66.70   64.59   71.42   80.58   79.44   86.97   89.50   93.42  100.00   99.70   97.72
    11: XP_024106876.1[P.abelii]          58.24   66.78   64.68   71.42   80.50   79.53   87.05   89.35   93.42   99.70  100.00   97.80
    12: NP_780300.2[M.musculus]           58.33   66.87   64.77   71.50   81.23   79.53   86.47   88.67   93.04   97.72   97.80  100.00
```
