# Supplementary material for: An Evolutionary Perspective on the Origin, Conservation and Binding Partner Acquisition of Tankyrases
Source: Biomolecules. 2022 Nov 15;12(11):1688. doi: 10.3390/biom12111688 (PMC9688111; doi:10.3390/biom12111688)
Supplement: Supplementary file 1 [file biomolecules-12-01688-s001.zip › biomolecules-1913719-supplementary-11.17 -done/Data_2/TNKS2.pdf]

TNKS2

Sequences:

>NP_079511.1 poly [ADP-ribose] polymerase tankyrase-2 [Homo sapiens]
MSGRRCAGGGAACASAAAEAVEPAARELFEACRNGDVERVKRLVTPEKVNSRDTAGRKSTPLHFAAGFGR
KDVVEYLLQNGANVQARDDGGLIPLHNACSFGHAEVVNLLLRHGADPNARDNWNYTPLHEAAIKGKIDVC
IVLLQHGAEPTIRNTDGRTALDLADPSAKAVLTGEYKKDELLESARSGNEEKMMALLTPLNVNCHASDGR
KSTPLHLAAGYNRVKIVQLLLQHGADVHAKDKGDLVPLHNACSYGHYEVTELLVKHGACVNAMDLWQFTP
LHEAASKNRVEVCSLLLSYGADPTLLNCHNKSAIDLAPTPQLKERLAYEFKGHSLLQAAREADVTRIKKH
LSLEMVNFKHPQTHETALHCAAASPYPKRKQICELLLRKGANINEKTKEFLTPLHVASEKAHNDVVEVVV
KHEAKVNALDNLGQTSLHRAAYCGHLQTCRLLLSYGCDPNIISLQGFTALQMGNENVQQLLQEGISLGNS
EADRQLLEAAKAGDVETVKKLCTVQSVNCRDIEGRQSTPLHFAAGYNRVSVVEYLLQHGADVHAKDKGGL
VPLHNACSYGHYEVAELLVKHGAVVNVADLWKFTPLHEAAAKGKYEICKLLLQHGADPTKKNRDGNTPLD
LVKDGDTDIQDLLRGDAALLDAAKKGCLARVKKLSSPDNVNCRDTQGRHSTPLHLAAGYNNLEVAEYLLQ
HGADVNAQDKGGLIPLHNAASYGHVDVAALLIKYNACVNATDKWAFTPLHEAAQKGRTQLCALLLAHGAD
PTLKNQEGQTPLDLVSADDVSALLTAAMPPSALPSCYKPQVLNGVRSPGATADALSSGPSSPSSLSAASS
LDNLSGSFSELSSVVSSSGTEGASSLEKKEVPGVDFSITQFVRNLGLEHLMDIFEREQITLDVLVEMGHK
ELKEIGINAYGHRHKLIKGVERLISGQQGLNPYLTLNTSGSGTILIDLSPDDKEFQSVEEEMQSTVREHR
DGGHAGGIFNRYNILKIQKVCNKKLWERYTHRRKEVSEENHNHANERMLFHGSPFVNAIIHKGFDERHAY
IGGMFGAGIYFAENSSKSNQYVYGIGGGTGCPVHKDRSCYICHRQLLFCRVTLGKSFLQFSAMKMAHSPP
GHHSVTGRPSVNGLALAEYVIYRGEQAYPEYLITYQIMRPEGMVDG

>XP_024109627.1 tankyrase-2 [Pongo abelii]
MSGRRCAGGGAACASAAAEAMEPAARELFEACRNGDVERVKRLVTPEKVNSRDTAGRKSTPLHFAAGFGR
KDVVEYLLQNGANVQARDDGGLIPLHNACSFGHAEVVNLLLRHGADPNARDNWNYTPLHEAAIKGKIDVC
IVLLQHGAEPTIRNTDGRTALDLADPSAKAVLTGEYKKDELLESARSGNEEKMMALLTPLNVNCHASDGR
KSTPLHLAAGYNRVKIVQLLLQHGADVHAKDKGDLVPLHNACSYGHYEVTELLVKHGACVNAMDLWQFTP
LHEAASKNRVEVCSLLLSYGADPTLLNCHNKSAIDLAPTPQLKERLAYEFKGHSLLQAAREADVTRIKKH
LSLEMVNFKHPQTHETALHCAAASPYPKRKQICELLLRKGANINEKTKEFLTPLHVASEKAHNDVVEVVV
KHEAKVNALDNLGQTSLHRAAYCGHLQTCRLLLSYGCDPNIISLQGFTALQMGNENVQQLLQEGISLGNS
EADRQLLEAAKAGDVETVKKLCTVQSVNCRDIEGRQSTPLHFAAGYNRVSVVEYLLQHGADVHAKDKGGL
VPLHNACSYGHYEVAELLVKHGAVVNVADLWKFTPLHEAAAKGKYEICKLLLQHGADPTKKNRDGNTPLD
LVKDGDTDIQDLLRGDAALLDAAKKGCLARVKKLSSPDNVNCRDTQGRHSTPLHLAAGYNNLEVAEYLLQ
HGADVNAQDKGGLIPLHNAASYGHVDVAALLIKYNACVNATDKWAFTPLHEAAQKGRTQLCALLLAHGAD
PTLKNQEGQTPLDLVSADDVSALLTAAMPPSALPSCYKPQVLNGVRSPGAPADALSSGPSSPSSLSAASS
LDNLSGSFSELSSVVSSSGTEGTSSLEKKEVPGVDFSITQFLRNLGLEHLMDIFEREQITLDVLVEMGHK
ELKEIGINAYGHRHKLIKGVERLISGQQGLNPYLTLNTSGSGTILIDLSPDDKEFQSVEEEMQSTVREHR
DGGHAGGIFNRYNILKIQKVCNKKLWERYTHRRKEVSEENHNHANERMLFHGSPFVNAIIHKGFDERHAY
IGGMFGAGIYFAENSSKSNQYVYGIGGGTGCPVHKDRSCYICHRQLLFCRVTLGKSFLQFSAMKMAHSPP
GHHSVTGRPSVNGLALAEYVIYRGEQAYPEYLITYQIMRPEGMVDG

>NP_001157107.1 poly [ADP-ribose] polymerase tankyrase-2 [Mus musculus]
MSGRRCAGGGAACASAGAEAVEPSARELFEACRNGDVERVKRLVTPEKVNSRDTAGRKSTPLHFAAGFGR
KDVVEYLLQNGANVQARDDGGLIPLHNACSFGHAEVVNLLLQHGADPNARDNWNYTPLHEAAIKGKIDVC
IVLLQHGAEPTIRNTDGRTALDLADPSAKAVLTGDYKKDELLESARSGNEEKMMALLTPLNVNCHASDGR
KSTPLHLAAGYNRVKIVQLLLHHGADVHAKDKGDLVPLHNACSYGHYEVTELLVKHGACVNAMDLWQFTP
LHEAASKNRIEVCSLLLSYGADPTLLNCHNKSAIDLAPTAQLKERLSYEFKGHSLLQAAREADVTRIKKH
LSLEMVNFKHPQTHETALHCAAASPYPKRKQICELLLRKGANTNEKTKEFLTPLHVASENAHNDVVEVVV
KHEAKVNALDSLGQTSLHRAAHCGHLQTCRLLLSYGCDPNIISLQGFTALQMGNENVQQLLQEGASLGHS
EADRQLLEAAKAGDVETVKKLCTVQSVNCRDIEGRQSTPLHFAAGYNRVSVVEYLLQHGADVHAKDKGGL
VPLHNACSYGHYEVAELLVKHGAVVNVADLWKFTPLHEAAAKGKYEICKLLLQHGADPTKKNRDGNTPLD
LVKDGDTDIQDLLRGDAALLDAAKKGCLARVKKLSSPDNVNCRDTQGRHSTPLHLAAGYNNLEVAEYLLQ
HGADVNAQDKGGLIPLHNAASYGHVDVAALLIKYNACVNATDKWAFTPLHEAAQKGRTQLCALLLAHGAD
PTLKNQEGQTPLDLVSADDVSALLTAAMPPSALPTCYKPQVLSGVRGPGATADALSSGPSSPSSLSAASS
LDNLSGSFSELSAVVSSSAAEGATGLQRKEDSGIDFSITQFIRNLGLEHLMDIFEREQITLDVLVEMGHK
ELKEIGINAYGHRHKLIKGVERLISGQQGLNPYLTLNNSGSGTILIDLSPDDKEFQSVEEEMQSTVREHR
DGGHAGGVFNRYNILKIQKVCNKKLWERYTHRRKEVSEENHNHANERMLFHGSPFVNAIIHKGFDERHAY
IGGMFGAGIYFAENSSKSNQYVYGIGGGTGCPIHKDRSCYICHRQLLFCRVTLGKSFLQFSAMKMAHSPP
GHHSVTGRPSVNGLALAEYVIYRGEQAYPEYLITYQIVRPEGMVDG

>NP_989672.2 poly [ADP-ribose] polymerase tankyrase-2 [Gallus gallus]
MAARRCAGGAAALAEAPGCGSAVEPARELFEACRNGDVERVKRLVRPENVNSRDTAGRKSSPLHFAAGFG
RKDVVEYLLQSGANVHARDDGGLIPLHNACSFGHAEVVNLLLRHGADPNARDNWNYTPLHEAAIKGKTDV
CIVLLQHGAEPTIRNTDGRTALDLADPSAKAVLTGEYKKDELLESARSGNEEKMMSLLTPLNVNCHASDG
RKSTPLHLAAGYNRVKIVQLLLQHGADVHAKDKGDLVPLHNACSYGHYEVTELLVKHGACVNAMDLWQFT
PLHEAASKNRVEVCSLLLSYGADPTLLNCHNKSTIDLAPTPQLKERLAYEFKGHSLLQAARESDVARIKK
HLSLETVNFKHPQTHETALHCAAASPYPKRKQVCELLLRKGANINEKTKDFLTPLHVASEKAHNDVVEVV
VKHEAKVNALDNLGQTSLHRAAHCGHLQTCRLLLSSGCDPSIVSLQGFTALQMGNESVQQLLQEGIPLGN
SDADRQLLEAAKAGDVDTVKKLCTVQSVNCRDIEGRQSTPLHFAAGYNRVSVVEYLLQHGADVHAKDKGG
LVPLHNACSYGHYEVAELLVKHGAVVNVADLWKFTPLHEAAAKGKYEICKLLLQHGADPTKKNRDGNTPL
DLVKDGDTDIQDLLRGDAALLDAAKKGCLARVKKLCSPDNVNCRDTQGRHSTPLHLAAGYNNLEVAEYLL
QHGADVNAQDKGGLIPLHNAASYGHVDVAALLIKYNACVNATDKWAFTPLHEAAQKGRTQLCALLLAHGA
DPTLKNQEGQTPLDLVTADDVSALLTAAMPPSALPSCYKPQVISVSQTASSTADPLSSVPSSPSSLSAAS
SLDNLSGSFSELPSVVGTNSAEGATVLEKKEVSGVDFSINQFVRNLGLEHLIDIFEREQITLDVLVEMGH
KELKEIGINAYGHRHKIIKGVERLISGQQGLNPYLTLNTSSSGTLLIDLSSEDKEFQSVEEEMQSTVREH
RDGGHAGGVFNRYNILKIQKVCNKKLWERYTHRRKEVSEENHNHANERMLFHGSPFVNAIIHKGFDERHA
YIGGMFGAGIYFAENSSKSNQYVYGIGGGTGCPIHKDRSCYVCHRQLLFCRVTLGKSFLQFSAMKMAHSP
PGHHSVTGRPSVNGLALAEYVIYRGEQAYPEYLITYQIVKPEATTEA

>XP_018082988.1 poly [ADP-ribose] polymerase tankyrase-2 [Xenopus laevis]
MSGRRCAGASGSAASHGNIGAGGEPARELFEACRNGDVERVRKLVNSDNVNSRDTAGRKSTPLHFAAGFG
RKDVVEHLLQSGANVHARDDGGLIPIHNACSFGHAEVVSLLLRHGADPNARDNWNYTPLHEAAIKGKIDV
CIVLFQHGADPTIRNTDSRTALDLSDPSAKAVLTGEYKKDELLESARSGNEEKMMALLTPLNVNCHASDG
RKSTPLHLAAGYNRVKIVQLLLQHGADVHAKDKGDLVPLHNACSYGHYEVTELLVKHGASVNAMDLWQFT
SLHEAASKNRVEVCSLLLSYGADPTMLNCHNKSAIDLAPTPQLKERLSYEFKGHSLLQAAREADLTRIKK
HLSLEIVNFKHPQTHDTALHCAASSPYPKRKQVCELLFRKGANVNEKTKDFLTPLHIASDKAHNDIVELL
IKHEAKVNALDNLGQTSLHRAAHCGHLQTCRLLLNSGCDPSIVSLQGFTALQMGNESVQQLLQEGLPFSN
SDADSQLLEAAKAGDLDVVKKLCTSQSVNCRDVEGRQSTPLHFAAGYNRVAVVEYLLQHGADVHAKDKGG
LVALHNACSYGHYEVTELLVKHGAVINVADLWKFTPLHEAAAKGKYEICKLLLLHGADSTKKNRDGNTPL
DLVKDGDTDIQDLLRGDAALLDAAKKGCVSRVKKLCTPENVNCRDTQGRHSTPLHLAAGYNNLEVAEYLL
QHGADVNAADKGGLIPLHNAASYGHVDVAALLIKYNACVNANDKWAFTPLHEAAQKGRTQLCALLLAHGA
DPALKNQEGQAPLDLVTADDVRALLTAAMPPAALPTFYKPQLLNVPQPSTTAIAALSSVPLSSPTLSAAS
SLDNLASSFSETTSVSSGGAEGASGLDKKDEGVDLNINQFLRNLGLEHLIDVFEREQITLDVLIEMGHKE
FKEIGISAYGHRHKIIKGVERLISGQHGLNPYLTLNTSNSGTILIDLAPDDKEFLSVEEEMQSTVREHRD
GGHAGGVFTKYNILKIQKVCNKKLWEKYTHRQKEVTEENHNHSNERMLFHGSPFVNAIIHKGFDERHAYI
GGMFGAGIYFAENSSKSNQYVYGIGGGTGCPIHKDRSCYVCHRQLLFCRVTLGKSFLQFSAMKMAHSPPG
HHSVTGRPSVNGLALAEYVIYRGEQAYPEYIITYQIMKPEAVAEG

>XP_014034742.2 poly [ADP-ribose] polymerase tankyrase-2 isoform X1 [Salmo salar]
MASVARFWGSANTNSKLYIYENTTPLMSTRQCSRVLGLGGVSPGVDTPRSGEPNREIFEACRNGDVERVR
RLVRPENVNSRDTAGRKSTPLHFAAGFGRRDVVDYLLQNGANVHARDEGGLVSLHNACSFGHSEVVNLLL
RHGADANSRDNWSYTPLHEAAIKGKSEVCIVLLQHGAEPTIRNTDGRTALDLAEASTKAVLTGEYRKDDL
LESARSGNEDKLMALLTPLNVNCHASDGRKSSPLHLAAGYNRVKTVQLLLKHGADVHAKDKGDLVPLHNA
CSYGHYEVTDILVKQGACVNAMDLWQFTPLHEAASKNRVDVCSLLVSYGADPTFLNCHNQSAIDLSPTPQ
LKERLAYEFRGHALLQAAREADLPRLKKHLSLETITFKHPLTHETALHCAATSPYPKRKQVCELLLRKGA
NVNEKTKDFLTPLHLASEKSHNDIIEVLVKHEAKVNALDNLGQTALHRAAHCGHLQTCRLLLKAGCDPLV
MSLQGFSPSQMGNESVQEILHEGTLIGNSDVDWQLLEASKSGDLEIVKKLCTMQNVNCRDVEGRQSTPLH

FAAGYNRVSVVEYLLLHGADVHAKDKGGLVPLHNACSYGHYEVAELLVIHGAVVNIADLWKFTPLHEAAA
KGKYEICKLLLQHGADPTKKNRDGSTPLDLVKDGDTDMQDLLRGDAALLDAAKKGCLARVEKLCSPDNVN
CRDAQGRHSTLLHLAAGYNNLEVAEYLLQHGAEVNSQDKGGLIPLHNAASYGHVDVAALLIKYDACVNAT
DKWAFTPLHEAAQKGRTQLCALLLAHGADPALRNQEGQSPLDLVTVDDVRALLTAAMPPSALPGCYKPQV
ISMSSPVGVVVPPSLSASSTPLSTLASRNSLDNQATASTCTAFPELPALLGPSGAVGTDNKEVPGVDLSI
GQFLNNLGLEHLLEIFEREQITLDVLVEMGHKELKEIGINAYGHRHKIIKGVERLISGPQSLNPYLTLNT
ANSGTILIDLVSDDKEFQLVEEELQSTIREHRDGGLAGGVFNRYNIVKIQKVCNKKLWERYTHRRKEVSE
ENHNHSNERMLFHGSPFVNAIIHKGFDERHAYIGGMFGAGIYFAENSSKSNQYVYGIGGGTGCPLHKDRS
CYVCQRHLLFCRVTLGKSFLQFSAMKMAHSPPGHHSVTGRPSVNGLSLAEYVIYRGEQAYPEYLITYQIL
KPDASVDG

>XP_032806710.1 poly [ADP-ribose] polymerase tankyrase-2 [Petromyzon marinus]
MAARRAHLASPSSSSSLSPSSGSLDTTTAAAAAAAATTTAAAAAATTTAAAMTTTAAVSATSSPSTTAAA
AAAVAAAAATGSGPGSGQDSSADGGDAGKELFEACRNGDVGRVKRLVSPLNVNARDTTGRKSTPLHFAAG
FGRKDVVEHLLQNGANVHARDDGGLIPLHNACSFGHAEVVNLLLRQGADPNARDNWNYTPLHEAAIKGKI
DVCIVLLQHGADPGIRNTDGKTALDLAEPTAKAVLTGEYKKDELLEAARSGNEEKLMALLTPLNVNCHAS
DGRKSTPLHLAAGYNRVRIVQLLLQHGADVHAKDKGGLVPLHNACSYGHYEVTELLVKHGASVNAMDLWQ
FTPLHEAASKNRVEVCSLLLSYGADPTLLNCHSKSAIDLAPTSELKERLVYEFRGHSLLQAAREADVTRV
KKHLGLDIINFKHPQTHETALHCAVASPYPKRKQVSELLLRKGANVNEKTKDLLTPLHVASDKAHNDVID
VLLKHGAKINVTDSLGQTSLHRAAHAGHLTTCRLLLSHGCDPNLPSLQGFTAAQLGNESVQQILHESSLP
VRNSDVEHQLLEAAKAGDLDTVKQLCTTQTVNCRDMEGRHSTPLHFAAGYNRVSVVEYLLQHGADVHAKD
KGGLVPLHNACSYGHYEVAELLVKRGAVVNVADLWKFTPLHEAAAKGKYEICKLLLKHGADPTKKNRDGN
TPLDLVKEGDTDIQDLLRGDAALLDAAKKGCLARVQKLCSGENVNCRDSQGRNSTPLHLAAGYNNLEVAE
YLLEHGADVNAQDKGGLIPLHNAASYGHVDIAALLIKYSACVNATDKWAFTPLHEAAQKGRTQLCALLLA
HGADPSMKNQEGQTPLDLATADDVRALLIDAMPPEALPVTHKPPSIPTIAATSAAATAALALGPTPALAS
LAPSPVPSGASASGAGGVPLLASPSSTPASLSAASSMDSLTGACGVAPSPVPAGAIGAGAAGDGTLLGAC
GVSAADGATGPADKPDRSADFEIPGLDMNINMFLKGLGLDHLRDIFEKEQITLDVLADMGHEELKEIGIN
AYGHRHKLIKGVERLLGAQQGGLAYPGANPYLSYHTTAQGTLLIDLPPDDKEYQSVEDEMQSTIREHKDG
GQAGGVFNRYNVIKIQKVRNKKLWERYTHRRKEVTEENHNHHNERMLFHGSPFINAIINKGFDERHAYIG
GMFGAGIYFAENSSKSNQYVYGIGGGTGCPVHKDRSCYICQRQMLFCRVTLGKSFLQFSAMKMAHSPPGH
HSVIGRPSVNGLAFAEYVIYRGEQAYPEYLITYQILKPECTPDLTSAAGQKS

>XP_019641281.1 PREDICTED: tankyrase-1-like isoform X1 [Branchiostoma belcheri]
MAGRRAMLSSDRIHCDESSGCRELFEACRNGDVARVKKLITPQNVNARDTAGRKSTPLHFAAGFGRKDVV
EHLLQNGANVHARDDGGLIPLHNACSFGHAEVVTLLLRNGADPNARDNWNYTPLHEAAIKGKIDVCIVLL
QNGADPSIRNTDGKTALDLAEPSAKTVLTGDYKKDELLEAARSGNEDKLMALLTPLNVNCHASDGRKSTP
LHLAAGYNRVRIVQLILQHGADVHAKDKGGLVPLHNACSYGHFEVTELLLKHGASVNAMDLWQFTPLHEA
ASKSRIEVCSLLLSHGADPTLLNCHSKSAIDVASTPELQEKLSYEFKGHCLLDGARQTDMAKVKKHLQLD
IVNFKHPYTHDTALHCAALSPYPKRRQITELLIRKGANLNDKNKEFLTPLHVASDKCYIDVMEVLLKHGA
KVNALDSLGQTALHRGAHCGHVQACRLLLSFGVDPSIVSLQGVTAAQMATEAVQQMLHEDPPSGPADECD
QLLEAAKAGDLEAVKVLCNPHTVNCRDVNGRHSTPLHFAAGYNRVAVVEYLLQHGADVHAKDKGGLVPLH
NACSYGHYEVCELLLKHGAVVNVADLWKFTPLHEAAAKGKYEICKLLLKHGADPNKKNRDGNTPLDLVRD
GDTDIQDLLRGDAALLDAAKKGNLARVQKLATPENINCRDTQGRNSTPLHLAAGYNNVEVAEFLLENGAD
VNAQDKGGLIPLHNASSYGHVEIAQLLIKYGTCVNATDRWNFTPLHEAAQKGRTQLCALLLAHGADPTMK
NQEGQTPLDLATAEDVRALLVDAMPPQSLPAAITAATVKAASPISSPSTTPSSTPGVLSTASSMDHLVGA
VGGAGQGDGAIDRSAVEGESLLDMTISSFLQQLGLNQLLDIFNKEQITLDILGEMGHEELKEIGVNAYGH
RHKLIKGVERILGGTGTTLNPYLTPALGSHGTILTDLSPDDKEYQSVEEEVQSTIRQHKDQGQAGGIFNR
YNVIKIQKVRNKRLWDRYVHRRKEVSEENHNQSNERMLFHGSPFINAIINKGFDERHAYIGGMFGAGIYF
AENSSKSNQYVYGIGGGTGCPIHKDRSCYICQRQMLFCRVTLGKAFLQFSAMKMAHAPPGHHSVIGRPSV
GGLNFAEYVIYRGEQAFPEYLITYQIVKPEAPAAPSPDQK

>NP_651410.1 tankyrase, isoform A [Drosophila melanogaster]
MANSSRSRAILSVNLDAVMANDPLRELFEACKTGEIAKVKKLITPQTVNARDTAGRKSTPLHFAAGYGRR
EVVEFLLNSGASIQACDEGGLHPLHNCCSFGHAEVVRLLLKAGASPNTTDNWNYTPLHEAASKGKVDVCL
ALLQHGANHTIRNSEQKTPLELADEATRPVLTGEYRKDELLEAARSGAEDRLLALLTPLNVNCHASDGRR
STPLHLAAGYNRIGIVEILLANGADVHAKDKGGLVPLHNACSYGHFDVTKLLIQAGANVNANDLWAFTPL
HEAASKSRVEVCSLLLSRGADPTLLNCHSKSAIDAAPTRELRERIAFEYKGHCLLDACRKCDVSRAKKLV
CAEIVNFVHPYTGDTPLHLAVVSPDGKRKQLMELLTRKGSLLNEKNKAFLTPLHLAAELLHYDAMEVLLK
QGAKVNALDSLGQTPLHRCARDEQAVRLLLSYAADTNIVSLEGLTAAQLASDSVLKLLKNPPDSETHLLE
AAKAGDLDTVRRIVLNNPISVNCRDLDGRHSTPLHFAAGFNRVPVVQFLLEHGAEVYAADKGGLVPLHNA
CSYGHYEVTELLVKHGANVNVSDLWKFTPLHEAAAKGKYDICKLLLKHGADPMKKNRDGATPADLVKESD
HDVAELLRGPSALLDAAKKGNLARVQRLVTPESINCRDAQGRNSTPLHLAAGYNNFECAEYLLENGADVN
AQDKGGLIPLHNASSYGHLDIAALLIKHKTVVNATDKWGFTPLHEAAQKGRTQLCSLLLAHGADAYMKNQ
EGQTPIELATADDVKCLLQDAMATSLSQQALSASTQSLTSSSPAPDATAAAAPGTSSSSSSAILSPTTET
VLLPTGASMILSVPVPLPLSSSTRISPAQGAEANGAEGSSSDDLLPDADTITNVSGFLSSQQLHHLIELF
EREQITLDILAEMGHDDLKQVGVSAYGFRHKILKGIAQLRSTTGIGNNVNLCTLLVDLLPDDKEFVAVEE
EMQATIREHRDNGQAGGYFTRYNIIRVQKVQNRKLWERYAHRRQEIAEENFLQSNERMLFHGSPFINAIV
QRGFDERHAYIGGMFGAGIYFAEHSSKSNQYVYGIGGGIGCPSHKDKSCYVCPRQLLLCRVALGKSFLQY
SAMKMAHAPPGHHSVVGRPSAGGLHFAEYVVYRGEQSYPEYLITYQIVKPDDSSSGTEDTR

>XP_032220531.1 poly [ADP-ribose] polymerase tankyrase-1-like [Nematostella vectensis]
MPCQAKMASKKLASNPTFLSDPDVCEGGRELFEACRNGDVSKVRKLVTNNSNVNVRDTAGRKSSPLHFAA
GFGRKEVVEYLLQCGADVHAMDDGGLIPLHNACSFGHAEVVRILLSHGADANARDNWNYTPLHEAAVKGK
VDVCVVLLQHGADPNIRNTDGKTALDVAEAAAKLVLTGDYKKDELLEGARSGNEEKLMSLLTPLNVNCHA
SDGRKSTPLHLAAGYNRVGVVQLLLKHGADVHAKDKGGLVPLHNACSYGHFEVTELLIKHGASVNAMDLW
QFTPLHEAASKARVEVCALLLGHGADPTLLNCHSKSAIDAAPTKELQEKLLSEFKGHQILEAAKGCDTAK
IKKLITTETINFQHPLTLDTPLHVAVASSSPKKKGVIETLLKKGADPNLQNKSSYAPLHVAAEKGLFEAM
ELLLKHGAKVNAIDSAGQTALHVAAIAGQVQACRVLMSNGADPTCQTFQGYTAFEVAPEPVQKVLHVNEE
SPSPVSDAEKQLLEAAKTGDLETVKNLCTAQTVNCRDLDGRCSTPLHFAAGYNRVRVVEYLLLNGADVHA
KDKGGLVPLHNACSYGHYEVAELLVKHGAIVNVADLWKFTPLHEAAAKGKYEICQLLLKHGADPQKKNRD
GYTPLDLVKEGDSDVADLLRGDAALLDAAKKGNLIRITKLATPENINCRDTQGRNSTPLHLAAGYNHLEV
AEYLLEHGADVNAQDKGGLIPLHNASSYGHVDIAALLIRYNTDVNATDRWLFTPLHEAAQKGRTQLCALL
LAHGAEPSMKNQEGQSPVDLATAEDVKCLLGDAMLSTQTTSVSTAPVAKVPAPVTATAGAATPATGPISG
MAPLSQANSSLLANSPAGHGDGAFSNRSVSEAQGEAVRSISAVHPGLDVDVGQFLDGLQLNNLKEIFERE
QISWDVLVDMGHEELKEIGIHAYGHRHKILKAVKEKISGMGLGLGPFSTSQVQGSVIQELSMVDKDFASV
ADEMQSTIVEHRDSGTAGGVFTSYTILKIERVVNTKLWEKYVYRRREIADSNHNHANERMLFHGSPFINA
IVQKGFDQRHAYIGGMFGAGIYFAENSSKSNQYVYGIGGGSGCPTHKDRSCYSCERQLLLCRVALGKPFY
QFSAVKMAHAPPGHNSVIGRPSSGGLSFAEYVIYRGEQAYPEYLITYKINKPDT

>XP_047128184.1 poly [ADP-ribose] polymerase tankyrase-2-like isoform X1 [Hydra vulgaris]
MALGRPNRAMPSRRNSVASNFMDGGVHENDSGRELFEACRTGDVIRVRYLIQNNWDVNMRDTAGRKSTPL
HFAAGFGRKDIVEYLLDAGADVHARDDGGLIPLHNACSFGHVEVTLSLLRHHADVNARDNWNFTPLHEAA
IKGKVEVCIALLQHGADPLIRNTDGKTARDLAEATAKAVLTGEYKKEELLDSARSGCEEKMLSLITSLNV
NCHAGDGRKSTPLHLAAGYNRTRIVEILLKHGADVHAKDKGGLVPLHNACSYGHYEVTELLVKHGASVNA
MDLWQFTPLHEAASKMRIEVCTLLLSYGADPTVLNCHSKTAIDVAPVQELKDKLLYEYRGHTYLEAVKSS
DLSKIKRIIAPDLVNFVHPLTLNSALHVCALSTSAKRDRVADFLIKKGCNMNFCNKQKLTPLHLCAGNDN
VVVTEILIKAGADCNIVNESLQSPLHIAAQSGHAHICYILLMNGADPTIRNSSGYTPAQVGTESVQKIFQ
ARLEPMLKDVDINLLEASKTGDFELIQSLCTPQNVNCRDVAGRMSTPLHFASGYNRMDIVEHLLRCGADV
HAKDKGGLVPLHNACSYGHFEIAEILVRHGANVNAADYWKFTPLHEAAAKGKFDICKLLLKHGADPKKKN
HEGYTPLDLVKEGFPDVADLLRGDAALLEAAKKGNLAKVMKLVTHQNINCRDTLGRNSTPLHLAAGYNHV
EVAEYLLEFGADVNAQDKGGLIPLHNASSYGHVDIAALLIKYKADVNATDRWLFTPLHEAAQKGRTQLCA
LLLTHGANANMKNQEGQTPIDLATADDVQVLLQDSMVSKSEVMQITSNTCSIKSPINSPQVLPISNDLSP
QLEPQGLTKPSTANQNLLLSEPCNSDGLQTKRLRDQAEGTNPLDIDIGTFLSSLQLEFLHEIFSREQITM
DVLIEMGPDQLKEIGIVAYGHRHKILKGVKERLAGAGCTIVGEPFLSNINASGPNTILQELSMDEKDYQS
VAEEMQSTVVEHKDGGVAGGIFSRYSSLKIERVINKKLWDKYYYQRKQVADANHNHPNERMLFHGSPFVN
AIVQKGFDERHAYIGGMFGAGIYFAEHSSKSNQYVYGIGGGSGCPVHKDRSCYDCKRQIILCRVTLGKPF
FQNSAMKMAHAPPGHHSVIGRPNSGGLSYPEYVVYRGEQAYPEYLITYKIERPNTLSSCQSAVD

>XP_019848937.1 PREDICTED: tankyrase-2-like [Amphimedon queenslandica]
MDTRDHQAKIPGDSRQTSQSIEEGESADKNTGDLARLFEACKNGDIDTVQNLIQQRQSSANERDLHGRKS
TPLHFAAGFGRRDIVKFLIEKGAHVDFRDEGGLIPLHNSCSFGHVDVVQLLLSNGANPNAQDNWKFSPLH
EAAIKGKADVCIVLLQHGADSSLLNTDRKAPIDLANGQAREVLLGTYRQDELLEAAKVGDEQLLMQILTP
LNVNCHASDGRRSTPLHLAAGYNRTSIVQLLLKQGADVHAKDKGGLVPLHNACSYGHYEVAELLLKYGAS
INVTDLWQFTPLQEAASKGRSDVCSLLLAHGANPSIANCHGKTAFNLAPSEEFRKKLDSEYRGYQLLAAA

```
EDGGIILLKKLLSSQLLKFQHHQTLDTLLHKAVLSKSSNRQSVIDALLKRGINLNIGNKENMTPLICAAK
KGILEVVEQLVQRGANINHQDINGMTSLHWAVQNEHAQICRYLLSSGANPSIVNNQGQTIYQLKTSDTIQ
LILKNEPPVSQFEIEQQLLEAARNSDLEILKKICTPQNVNCRDTKGRMSTPLHFAAGYNRVTVVEFLLEN
GADVHAKDKGGLVPLHNACSYGHYEVAELLVKYKANVNAMDLWKFTPLHEAAAKEKYDICKLLLKNGANV
HSKNRDNLTPIDLVKDPKSDLADLLRGEPALLDAAKKGEIERVKKLLTEDNVNCRDEYGRNSTPLHLAAG
YNHLDVVEYLLENKADVNAKDKGGLVPLHNASSYGHVDVASLLIRYNSVINATDRWNFTPLHEAAQKGRT
QVCSLLIIHGADVYLKNQEGQIPLDLATADDVIALLQDAMMKDIPLTIPPAEKEAKSNIVNKGLTAAGAS
LLASELVLGDGVDDKNGMVTQQRGGPAGVGDGSDKGFMSYRGGGDAPPPWHNVTVKDILTELELGHLVEL
FEREQITIDILIEMNGDDLQSIGITAFGVRHRLLKRIRELVQGNNEEYPVGVTTTKPTQGTQLIELSSDD
KEFIDTADLMQSTICEHRDDGKAGGVFDSYEILKIERIVNTKVWERYKYRRKEVAESNNNCANELMLFHG
SPFVPYIVHNGFDERHAYIGGMFGAGIYFAEHSSKSNQYVYGIGGGNGCPEHKNRSCYTCLRKLLLCRVV
LGKPVEQYTAVRIAHAPPGHHSVIGRPSAGGLNYPEYVIYRGEQAYPEYIITFRIKKPSATDSMSSSSSL
DMSNNT
```

Alignment

```
XP_019848937.1[A.queenslandica]    ------------------------------------------------------------    0
NP_651410.1[D.melanogaster]        ------------------------------------------------------------    0
XP_047128184.1[H.vulgaris]         ------------------------------------------------------------    0
XP_032220531.1[N.vectensis]        ------------------------------------------------------------    0
XP_014034742.2[S.salar]            MA-------------SVARFWGS---------------------ANT-----NSKLYIYE    21
XP_018082988.1[X.laevis]           ------------------------------------------------------------    0
NP_989672.2[G.gallus]              ------------------------------------------------------------    0
NP_001157107.1[M.musculus]         ------------------------------------------------------------    0
NP_079511.1[H.sapiens]             ------------------------------------------------------------    0
XP_024109627.1[P.abelii]           ------------------------------------------------------------    0
XP_032806710.1[P.marinus]          MAARRAHLASPSSSSSLSPSSGSLDTTTAAAAAAAATTTAAAAAATTTAAAMTTTAAVSA    60
XP_019641281.1[B.belcheri]         ------------------------------------------------------------    0

XP_019848937.1[A.queenslandica]    -----MDTRDHQAKIPGDSRQTSQSIEEGESADKNTGDLARLFEACKNGDIDTVQNLIQQ    55
NP_651410.1[D.melanogaster]        --------------MANSSRSR-AILSVNLDAVMANDPLRELFEACKTGEIAKVKKLIT-    44
XP_047128184.1[H.vulgaris]         -----MALGRPNRAMPSR-RNS-VASNFMDGGVHENDSGRELFEACRTGDVIRVRYLIQN    53
XP_032220531.1[N.vectensis]        --------MPCQAKMAS--KKL-ASNPTFLSDPDVCEGGRELFEACRNGDVSKVRKLVTN    49
XP_014034742.2[S.salar]            NTTPLMSTRQCSR-----VLGL-GGVSPGVDTPRSGEPNREIFEACRNGDVERVRRLVR-    74
XP_018082988.1[X.laevis]           -----MSGRRCAG-----ASGS-AASHG--NIGAGGEPARELFEACRNGDVERVRKLVN-    46
NP_989672.2[G.gallus]              -----MAARRCAG-----GAAA-LAEAP--GCGSAVEPARELFEACRNGDVERVKRLVR-    46
NP_001157107.1[M.musculus]         -----MSGRRCAG-----GGAA-CAS-A--GAEAVEPSARELFEACRNGDVERVKRLVT-    45
NP_079511.1[H.sapiens]             -----MSGRRCAG-----GGAA-CAS-A--AAEAVEPAARELFEACRNGDVERVKRLVT-    45
XP_024109627.1[P.abelii]           -----MSGRRCAG-----GGAA-CAS-A--AAEAMEPAARELFEACRNGDVERVKRLVT-    45
XP_032806710.1[P.marinus]          TSSPSTTAAAAAAVAAAAATGS-GPGSGQDSSADGGDAGKELFEACRNGDVGRVKRLVS-    118
XP_019641281.1[B.belcheri]         -----------------MAGRR-AMLSSDRIHCDESSGCRELFEACRNGDVARVKKLIT-    41
                                                                           .:****:.*::  *: *:

XP_019848937.1[A.queenslandica]    RQSSANERDLHGRKSTPLHFAAGFGRRDIVKFLIEKGAHVDFRDEGGLIPLHNSCSFGHV    115
NP_651410.1[D.melanogaster]        P-QTVNARDTAGRKSTPLHFAAGYGRREVVEFLLNSGASIQACDEGGLHPLHNCCSFGHA    103
XP_047128184.1[H.vulgaris]         N-WDVNMRDTAGRKSTPLHFAAGFGRKDIVEYLLDAGADVHARDDGGLIPLHNACSFGHV    112
XP_032220531.1[N.vectensis]        N-SNVNVRDTAGRKSSPLHFAAGFGRKEVVEYLLQCGADVHAMDDGGLIPLHNACSFGHA    108
XP_014034742.2[S.salar]            P-ENVNSRDTAGRKSTPLHFAAGFGRRDVVDYLLQNGANVHARDEGGLVSLHNACSFGHS    133
XP_018082988.1[X.laevis]           S-DNVNSRDTAGRKSTPLHFAAGFGRKDVVEHLLQSGANVHARDDGGLIPIHNACSFGHA    105
NP_989672.2[G.gallus]              P-ENVNSRDTAGRKSSPLHFAAGFGRKDVVEYLLQSGANVHARDDGGLIPLHNACSFGHA    105
NP_001157107.1[M.musculus]         P-EKVNSRDTAGRKSTPLHFAAGFGRKDVVEYLLQNGANVQARDDGGLIPLHNACSFGHA    104
NP_079511.1[H.sapiens]             P-EKVNSRDTAGRKSTPLHFAAGFGRKDVVEYLLQNGANVQARDDGGLIPLHNACSFGHA    104
XP_024109627.1[P.abelii]           P-EKVNSRDTAGRKSTPLHFAAGFGRKDVVEYLLQNGANVQARDDGGLIPLHNACSFGHA    104
XP_032806710.1[P.marinus]          P-LNVNARDTTGRKSTPLHFAAGFGRKDVVEHLLQNGANVHARDDGGLIPLHNACSFGHA    177
XP_019641281.1[B.belcheri]         P-QNVNARDTAGRKSTPLHFAAGFGRKDVVEHLLQNGANVHARDDGGLIPLHNACSFGHA    100
                                       .* **  ****:*******:**:::*..*:: ** :.  *:***  :**.*****

XP_019848937.1[A.queenslandica]    DVVQLLLSNGANPNAQDNWKFSPLHEAAIKGKADVCIVLLQHGADSSLLNTDRKAPIDLA    175
NP_651410.1[D.melanogaster]        EVVRLLLKAGASPNTTDNWNYTPLHEAASKGKVDVCLALLQHGANHTIRNSEQKTPLELA    163
XP_047128184.1[H.vulgaris]         EVTLSLLRHHADVNARDNWNFTPLHEAAIKGKVEVCIALLQHGADPLIRNTDGKTARDLA    172
XP_032220531.1[N.vectensis]        EVVRILLSHGADANARDNWNYTPLHEAAVKGKVDVCVVLLQHGADPNIRNTDGKTALDVA    168
XP_014034742.2[S.salar]            EVVNLLLRHGADANSRDNWSYTPLHEAAIKGKSEVCIVLLQHGAEPTIRNTDGRTALDLA    193
XP_018082988.1[X.laevis]           EVVSLLLRHGADPNARDNWNYTPLHEAAIKGKIDVCIVLFQHGADPTIRNTDSRTALDLS    165
NP_989672.2[G.gallus]              EVVNLLLRHGADPNARDNWNYTPLHEAAIKGKTDVCIVLLQHGAEPTIRNTDGRTALDLA    165
NP_001157107.1[M.musculus]         EVVNLLLQHGADPNARDNWNYTPLHEAAIKGKIDVCIVLLQHGAEPTIRNTDGRTALDLA    164
NP_079511.1[H.sapiens]             EVVNLLLRHGADPNARDNWNYTPLHEAAIKGKIDVCIVLLQHGAEPTIRNTDGRTALDLA    164
XP_024109627.1[P.abelii]           EVVNLLLRHGADPNARDNWNYTPLHEAAIKGKIDVCIVLLQHGAEPTIRNTDGRTALDLA    164
XP_032806710.1[P.marinus]          EVVNLLLRQGADPNARDNWNYTPLHEAAIKGKIDVCIVLLQHGADPGIRNTDGKTALDLA    237
XP_019641281.1[B.belcheri]         EVVTLLLRNGADPNARDNWNYTPLHEAAIKGKIDVCIVLLQNGADPSIRNTDGKTALDLA    160
                                   :*.  **   *. *: ***.::****** *** :**:.*:*:**:  : *:: ::  :::

XP_019848937.1[A.queenslandica]    NGQAREVLLGTYRQDELLEAAKVGDEQLLMQILTPLNVNCHASDGRRSTPLHLAAGYNRT    235
NP_651410.1[D.melanogaster]        DEATRPVLTGEYRKDELLEAARSGAEDRLLALLTPLNVNCHASDGRRSTPLHLAAGYNRI    223
XP_047128184.1[H.vulgaris]         EATAKAVLTGEYKKEELLDSARSGCEEKMLSLITSLNVNCHAGDGRKSTPLHLAAGYNRT    232
XP_032220531.1[N.vectensis]        EAAAKLVLTGDYKKDELLEGARSGNEEKLMSLLTPLNVNCHASDGRKSTPLHLAAGYNRV    228
XP_014034742.2[S.salar]            EASTKAVLTGEYRKDDLLESARSGNEDKLMALLTPLNVNCHASDGRKSSPLHLAAGYNRV    253
XP_018082988.1[X.laevis]           DPSAKAVLTGEYKKDELLESARSGNEEKMMALLTPLNVNCHASDGRKSTPLHLAAGYNRV    225
NP_989672.2[G.gallus]              DPSAKAVLTGEYKKDELLESARSGNEEKMMSLLTPLNVNCHASDGRKSTPLHLAAGYNRV    225
NP_001157107.1[M.musculus]         DPSAKAVLTGDYKKDELLESARSGNEEKMMALLTPLNVNCHASDGRKSTPLHLAAGYNRV    224
NP_079511.1[H.sapiens]             DPSAKAVLTGEYKKDELLESARSGNEEKMMALLTPLNVNCHASDGRKSTPLHLAAGYNRV    224
XP_024109627.1[P.abelii]           DPSAKAVLTGEYKKDELLESARSGNEEKMMALLTPLNVNCHASDGRKSTPLHLAAGYNRV    224
XP_032806710.1[P.marinus]          EPTAKAVLTGEYKKDELLEAARSGNEEKLMALLTPLNVNCHASDGRKSTPLHLAAGYNRV    297
XP_019641281.1[B.belcheri]         EPSAKTVLTGDYKKDELLEAARSGNEDKLMALLTPLNVNCHASDGRKSTPLHLAAGYNRV    220
                                   :  :: ** * *::::**:.*: * *: :: ::* *******.***:*:**********

XP_019848937.1[A.queenslandica]    SIVQLLLKQGADVHAKDKGGLVPLHNACSYGHYEVAELLLKYGASINVTDLWQFTPLQEA    295
NP_651410.1[D.melanogaster]        GIVEILLANGADVHAKDKGGLVPLHNACSYGHFDVTKLLIQAGANVNANDLWAFTPLHEA    283
XP_047128184.1[H.vulgaris]         RIVEILLKHGADVHAKDKGGLVPLHNACSYGHYEVTELLVKHGASVNAMDLWQFTPLHEA    292
XP_032220531.1[N.vectensis]        GVVQLLLKHGADVHAKDKGGLVPLHNACSYGHFEVTELLIKHGASVNAMDLWQFTPLHEA    288
XP_014034742.2[S.salar]            KTVQLLLKHGADVHAKDKGDLVPLHNACSYGHYEVTDILVKQGACVNAMDLWQFTPLHEA    313
XP_018082988.1[X.laevis]           KIVQLLLQHGADVHAKDKGDLVPLHNACSYGHYEVTELLVKHGASVNAMDLWQFTSLHEA    285
NP_989672.2[G.gallus]              KIVQLLLQHGADVHAKDKGDLVPLHNACSYGHYEVTELLVKHGACVNAMDLWQFTPLHEA    285
NP_001157107.1[M.musculus]         KIVQLLLHHGADVHAKDKGDLVPLHNACSYGHYEVTELLVKHGACVNAMDLWQFTPLHEA    284
NP_079511.1[H.sapiens]             KIVQLLLQHGADVHAKDKGDLVPLHNACSYGHYEVTELLVKHGACVNAMDLWQFTPLHEA    284
XP_024109627.1[P.abelii]           KIVQLLLQHGADVHAKDKGDLVPLHNACSYGHYEVTELLVKHGACVNAMDLWQFTPLHEA    284
XP_032806710.1[P.marinus]          RIVQLLLQHGADVHAKDKGGLVPLHNACSYGHYEVTELLVKHGASVNAMDLWQFTPLHEA    357
XP_019641281.1[B.belcheri]         RIVQLILQHGADVHAKDKGGLVPLHNACSYGHFEVTELLLKHGASVNAMDLWQFTPLHEA    280
                                     *:::* :**********.***********::*:.:*:: ** :*. *** ** *:**

XP_019848937.1[A.queenslandica]    ASKGRSDVCSLLLAHGANPSIANCHGKTAFNLAPSEEFRKKLDSEYRGYQLLAAAEDGGI    355
NP_651410.1[D.melanogaster]        ASKSRVEVCSLLLSRGADPTLLNCHSKSAIDAAPTRELRERIAFEYKGHCLLDACRKCDV    343
XP_047128184.1[H.vulgaris]         ASKMRIEVCTLLLSYGADPTVLNCHSKTAIDVAPVQELKDKLLYEYRGHTYLEAVKSSDL    352
XP_032220531.1[N.vectensis]        ASKARVEVCALLLGHGADPTLLNCHSKSAIDAAPTKELQEKLLSEFKGHQILEAAKGCDT    348
XP_014034742.2[S.salar]            ASKNRVDVCSLLVSYGADPTFLNCHNQSAIDLSPTPQLKERLAYEFRGHALLQAAREADL    373
XP_018082988.1[X.laevis]           ASKNRVEVCSLLLSYGADPTMLNCHNKSAIDLAPTPQLKERLSYEFKGHSLLQAAREADL    345
NP_989672.2[G.gallus]              ASKNRVEVCSLLLSYGADPTLLNCHNKSTIDLAPTPQLKERLAYEFKGHSLLQAARESDV    345
NP_001157107.1[M.musculus]         ASKNRIEVCSLLLSYGADPTLLNCHNKSAIDLAPTAQLKERLSYEFKGHSLLQAAREADV    344
NP_079511.1[H.sapiens]             ASKNRVEVCSLLLSYGADPTLLNCHNKSAIDLAPTPQLKERLAYEFKGHSLLQAAREADV    344
XP_024109627.1[P.abelii]           ASKNRVEVCSLLLSYGADPTLLNCHNKSAIDLAPTPQLKERLAYEFKGHSLLQAAREADV    344
XP_032806710.1[P.marinus]          ASKNRVEVCSLLLSYGADPTLLNCHSKSAIDLAPTSELKERLVYEFRGHSLLQAAREADV    417
XP_019641281.1[B.belcheri]         ASKSRIEVCSLLLSHGADPTLLNCHSKSAIDVASTPELQEKLSYEFKGHCLLDGARQTDM    340
                                   *** * :**:**:. **:*:. ***.::::: :   :::.::  *::*:  * . .  .

XP_019848937.1[A.queenslandica]    ILLKKLLSSQLLKFQHHQTLDTLLHKAVLSKSSNRQSVIDALLKRGINLNIGNKENMTPL    415
NP_651410.1[D.melanogaster]        SRAKKLVCAEIVNFVHPYTGDTPLHLAVVSPDGKRKQLMELLTRKGSLLNEKNKAFLTPL    403
XP_047128184.1[H.vulgaris]         SKIKRIIAPDLVNFVHPLTLNSALHVCALSTSAKRDRVADFLIKKGCNMNFCNKQKLTPL    412
XP_032220531.1[N.vectensis]        AKIKKLITTETINFQHPLTLDTPLHVAVASSSPKKKGVIETLLKKGADPNLQNKSSYAPL    408
XP_014034742.2[S.salar]            PRLKKHLSLETITFKHPLTHETALHCAATSPYPKRKQVCELLLRKGANVNEKTKDFLTPL    433
XP_018082988.1[X.laevis]           TRIKKHLSLEIVNFKHPQTHDTALHCAASSPYPKRKQVCELLFRKGANVNEKTKDFLTPL    405
NP_989672.2[G.gallus]              ARIKKHLSLETVNFKHPQTHETALHCAAASPYPKRKQVCELLLRKGANINEKTKDFLTPL    405
NP_001157107.1[M.musculus]         TRIKKHLSLEMVNFKHPQTHETALHCAAASPYPKRKQICELLLRKGANTNEKTKEFLTPL    404
NP_079511.1[H.sapiens]             TRIKKHLSLEMVNFKHPQTHETALHCAAASPYPKRKQICELLLRKGANINEKTKEFLTPL    404
XP_024109627.1[P.abelii]           TRIKKHLSLEMVNFKHPQTHETALHCAAASPYPKRKQICELLLRKGANINEKTKEFLTPL    404
XP_032806710.1[P.marinus]          TRVKKHLGLDIINFKHPQTHETALHCAVASPYPKRKQVSELLLRKGANVNEKTKDLLTPL    477
XP_019641281.1[B.belcheri]         AKVKKHLQLDIVNFKHPYTHDTALHCAALSPYPKRRQITELLIRKGANLNDKNKEFLTPL    400
                                      *: :  : :.* *  * :: ** .. *   ::  : : * ::*   *  .*   :**
```

```
XP_019848937.1[A.queenslandica]      ICAAKKGILEVVEQLVQRGANINHQDINGMTSLHWAVQNEHAQICRYLLSSGANPSIVNN           475
NP_651410.1[D.melanogaster]          HLAAELLHYDAMEVLLKQGAKVNALDSLGQTPLHRCARD--EQAVRLLLSYAADTNIVSL           461
XP_047128184.1[H.vulgaris]           HLCAGNDNVVVTEILIKAGADCNIVNESLQSPLHIAAQSGHAHICYILLMNGADPTIRNS           472
XP_032220531.1[N.vectensis]          HVAAEKGLFEAMELLLKHGAKVNAIDSAGQTALHVAAIAGQVQACRVLMSNGADPTCQTF           468
XP_014034742.2[S.salar]              HLASEKSHNDIIEVLVKHEAKVNALDNLGQTALHRAAHCGHLQTCRLLLKAGCDPLVMSL           493
XP_018082988.1[X.laevis]             HIASDKAHNDIVELLIKHEAKVNALDNLGQTSLHRAAHCGHLQTCRLLLNSGCDPSIVSL           465
NP_989672.2[G.gallus]                HVASEKAHNDVVEVVVKHEAKVNALDNLGQTSLHRAAHCGHLQTCRLLLSSGCDPSIVSL           465
NP_001157107.1[M.musculus]           HVASENAHNDVVEVVVKHEAKVNALDSLGQTSLHRAAHCGHLQTCRLLLSYGCDPNIISL           464
NP_079511.1[H.sapiens]               HVASEKAHNDVVEVVVKHEAKVNALDNLGQTSLHRAAYCGHLQTCRLLLSYGCDPNIISL           464
XP_024109627.1[P.abelii]             HVASEKAHNDVVEVVVKHEAKVNALDNLGQTSLHRAAYCGHLQTCRLLLSYGCDPNIISL           464
XP_032806710.1[P.marinus]            HVASDKAHNDVIDVLLKHGAKINVTDSLGQTSLHRAAHAGHLTTCRLLLSHGCDPNLPSL           537
XP_019641281.1[B.belcheri]           HVASDKCYIDVMEVLLKHGAKVNALDSLGQTALHRGAHCGHVQACRLLLSFGVDPSIVSL           460
                                       .:        : :::  *. *  :    : **  .          *:  . :    .

XP_019848937.1[A.queenslandica]      QGQTIYQLKTSDTIQLILKNE-PPVSQFEIEQQLLEAARNSDLEILKKI--CTPQNVNCR           532
NP_651410.1[D.melanogaster]          EGLTAAQLASDSVLKLLKNP-------PDSETHLLEAAKAGDLDTVRRIVLNNPISVNCR           514
XP_047128184.1[H.vulgaris]           SGYTPAQVGTESVQKIFQAR--LEPMLKDVDINLLEASKTGDFELIQSL--CTPQNVNCR           528
XP_032220531.1[N.vectensis]          QGYTAFEVAPEPVQKVLHVNEESPSPVSDAEKQLLEAAKTGDLETVKNL--CTAQTVNCR           526
XP_014034742.2[S.salar]              QGFSPSQMGNESVQEILHEG--TLIGNSDVDWQLLEASKSGDLEIVKKL--CTMQNVNCR           549
XP_018082988.1[X.laevis]             QGFTALQMGNESVQQLLQEG--LPFSNSDADSQLLEAAKAGDLDVVKKL--CTSQSVNCR           521
NP_989672.2[G.gallus]                QGFTALQMGNESVQQLLQEG--IPLGNSDADRQLLEAAKAGDVDTVKKL--CTVQSVNCR           521
NP_001157107.1[M.musculus]           QGFTALQMGNENVQQLLQEG--ASLGHSEADRQLLEAAKAGDVETVKKL--CTVQSVNCR           520
NP_079511.1[H.sapiens]               QGFTALQMGNENVQQLLQEG--ISLGNSEADRQLLEAAKAGDVETVKKL--CTVQSVNCR           520
XP_024109627.1[P.abelii]             QGFTALQMGNENVQQLLQEG--ISLGNSEADRQLLEAAKAGDVETVKKL--CTVQSVNCR           520
XP_032806710.1[P.marinus]            QGFTAAQLGNESVQQILHES-SLPVRNSDVEHQLLEAAKAGDLDTVKQL--CTTQTVNCR           594
XP_019641281.1[B.belcheri]           QGVTAAQMATEAVQQMLHED-P-PSGPADECDQLLEAAKAGDLEAVKVL--CNPHTVNCR           516
                                     .* :  ::  . . :::           :   :****:: .*.: :: :   .  .****

XP_019848937.1[A.queenslandica]      DTKGRMSTPLHFAAGYNRVTVVEFLLENGADVHAKDKGGLVPLHNACSYGHYEVAELLVK           592
NP_651410.1[D.melanogaster]          DLDGRHSTPLHFAAGFNRVPVVQFLLEHGAEVYAADKGGLVPLHNACSYGHYEVTELLVK           574
XP_047128184.1[H.vulgaris]           DVAGRMSTPLHFASGYNRMDIVEHLLRCGADVHAKDKGGLVPLHNACSYGHFEIAEILVR           588
XP_032220531.1[N.vectensis]          DLDGRCSTPLHFAAGYNRVRVVEYLLLNGADVHAKDKGGLVPLHNACSYGHYEVAELLVK           586
XP_014034742.2[S.salar]              DVEGRQSTPLHFAAGYNRVSVVEYLLLHGADVHAKDKGGLVPLHNACSYGHYEVAELLVI           609
XP_018082988.1[X.laevis]             DVEGRQSTPLHFAAGYNRVAVVEYLLQHGADVHAKDKGGLVALHNACSYGHYEVTELLVK           581
NP_989672.2[G.gallus]                DIEGRQSTPLHFAAGYNRVSVVEYLLQHGADVHAKDKGGLVPLHNACSYGHYEVAELLVK           581
NP_001157107.1[M.musculus]           DIEGRQSTPLHFAAGYNRVSVVEYLLQHGADVHAKDKGGLVPLHNACSYGHYEVAELLVK           580
NP_079511.1[H.sapiens]               DIEGRQSTPLHFAAGYNRVSVVEYLLQHGADVHAKDKGGLVPLHNACSYGHYEVAELLVK           580
XP_024109627.1[P.abelii]             DIEGRQSTPLHFAAGYNRVSVVEYLLQHGADVHAKDKGGLVPLHNACSYGHYEVAELLVK           580
XP_032806710.1[P.marinus]            DMEGRHSTPLHFAAGYNRVSVVEYLLQHGADVHAKDKGGLVPLHNACSYGHYEVAELLVK           654
XP_019641281.1[B.belcheri]           DVNGRHSTPLHFAAGYNRVAVVEYLLQHGADVHAKDKGGLVPLHNACSYGHYEVCELLLK           576
                                     *  ** *******:*:**: :*:.**  **:*:* ****** *********:*: *:*:

XP_019848937.1[A.queenslandica]      YKANVNAMDLWKFTPLHEAAAKEKYDICKLLLKNGANVHSKNRDNLTPIDLVKDPKSDLA           652
NP_651410.1[D.melanogaster]          HGANVNVSDLWKFTPLHEAAAKGKYDICKLLLKHGADPMKKNRDGATPADLVKESDHDVA           634
XP_047128184.1[H.vulgaris]           HGANVNAADYWKFTPLHEAAAKGKFDICKLLLKHGADPKKKNHEGYTPLDLVKEGFPDVA           648
XP_032220531.1[N.vectensis]          HGAIVNVADLWKFTPLHEAAAKGKYEICQLLLKHGADPQKKNRDGYTPLDLVKEGDSDVA           646
XP_014034742.2[S.salar]              HGAVVNIADLWKFTPLHEAAAKGKYEICKLLLQHGADPTKKNRDGSTPLDLVKDGDTDMQ           669
XP_018082988.1[X.laevis]             HGAVINVADLWKFTPLHEAAAKGKYEICKLLLLHGADSTKKNRDGNTPLDLVKDGDTDIQ           641
NP_989672.2[G.gallus]                HGAVVNVADLWKFTPLHEAAAKGKYEICKLLLQHGADPTKKNRDGNTPLDLVKDGDTDIQ           641
NP_001157107.1[M.musculus]           HGAVVNVADLWKFTPLHEAAAKGKYEICKLLLQHGADPTKKNRDGNTPLDLVKDGDTDIQ           640
NP_079511.1[H.sapiens]               HGAVVNVADLWKFTPLHEAAAKGKYEICKLLLQHGADPTKKNRDGNTPLDLVKDGDTDIQ           640
XP_024109627.1[P.abelii]             HGAVVNVADLWKFTPLHEAAAKGKYEICKLLLQHGADPTKKNRDGNTPLDLVKDGDTDIQ           640
XP_032806710.1[P.marinus]            RGAVVNVADLWKFTPLHEAAAKGKYEICKLLLKHGADPTKKNRDGNTPLDLVKEGDTDIQ           714
XP_019641281.1[B.belcheri]           HGAVVNVADLWKFTPLHEAAAKGKYEICKLLLKHGADPNKKNRDGNTPLDLVRDGDTDIQ           636
                                       * :*  * ************ *::**:*** :**:  .**::. ** ***::   *:

XP_019848937.1[A.queenslandica]      DLLRGEPALLDAAKKGEIERVKKLLTEDNVNCRDEYGRNSTPLHLAAGYNHLDVVEYLLE           712
NP_651410.1[D.melanogaster]          ELLRGPSALLDAAKKGNLARVQRLVTPESINCRDAQGRNSTPLHLAAGYNNFECAEYLLE           694
XP_047128184.1[H.vulgaris]           DLLRGDAALLEAAKKGNLAKVMKLVTHQNINCRDTLGRNSTPLHLAAGYNHVEVAEYLLE           708
XP_032220531.1[N.vectensis]          DLLRGDAALLDAAKKGNLIRITKLATPENINCRDTQGRNSTPLHLAAGYNHLEVAEYLLE           706
XP_014034742.2[S.salar]              DLLRGDAALLDAAKKGCLARVEKLCSPDNVNCRDAQGRHSTLLHLAAGYNNLEVAEYLLQ           729
XP_018082988.1[X.laevis]             DLLRGDAALLDAAKKGCVSRVKKLCTPENVNCRDTQGRHSTPLHLAAGYNNLEVAEYLLQ           701
NP_989672.2[G.gallus]                DLLRGDAALLDAAKKGCLARVKKLCSPDNVNCRDTQGRHSTPLHLAAGYNNLEVAEYLLQ           701
NP_001157107.1[M.musculus]           DLLRGDAALLDAAKKGCLARVKKLSSPDNVNCRDTQGRHSTPLHLAAGYNNLEVAEYLLQ           700
NP_079511.1[H.sapiens]               DLLRGDAALLDAAKKGCLARVKKLSSPDNVNCRDTQGRHSTPLHLAAGYNNLEVAEYLLQ           700
XP_024109627.1[P.abelii]             DLLRGDAALLDAAKKGCLARVKKLSSPDNVNCRDTQGRHSTPLHLAAGYNNLEVAEYLLQ           700
XP_032806710.1[P.marinus]            DLLRGDAALLDAAKKGCLARVQKLCSGENVNCRDSQGRNSTPLHLAAGYNNLEVAEYLLE           774
XP_019641281.1[B.belcheri]           DLLRGDAALLDAAKKGNLARVQKLATPENINCRDTQGRNSTPLHLAAGYNNVEVAEFLLE           696
                                     :****  ***:***** : :: :* : :.:****  **:** ********:.: .*:**:

XP_019848937.1[A.queenslandica]      NKADVNAKDKGGLVPLHNASSYGHVDVASLLIRYNSVINATDRWNFTPLHEAAQKGRTQV           772
NP_651410.1[D.melanogaster]          NGADVNAQDKGGLIPLHNASSYGHLDIAALLIKHKTVVNATDKWGFTPLHEAAQKGRTQL           754
XP_047128184.1[H.vulgaris]           FGADVNAQDKGGLIPLHNASSYGHVDIAALLIKYKADVNATDRWLFTPLHEAAQKGRTQL           768
XP_032220531.1[N.vectensis]          HGADVNAQDKGGLIPLHNASSYGHVDIAALLIRYNTDVNATDRWLFTPLHEAAQKGRTQL           766
XP_014034742.2[S.salar]              HGAEVNSQDKGGLIPLHNAASYGHVDVAALLIKYDACVNATDKWAFTPLHEAAQKGRTQL           789
XP_018082988.1[X.laevis]             HGADVNAADKGGLIPLHNAASYGHVDVAALLIKYNACVNANDKWAFTPLHEAAQKGRTQL           761
NP_989672.2[G.gallus]                HGADVNAQDKGGLIPLHNAASYGHVDVAALLIKYNACVNATDKWAFTPLHEAAQKGRTQL           761
NP_001157107.1[M.musculus]           HGADVNAQDKGGLIPLHNAASYGHVDVAALLIKYNACVNATDKWAFTPLHEAAQKGRTQL           760
NP_079511.1[H.sapiens]               HGADVNAQDKGGLIPLHNAASYGHVDVAALLIKYNACVNATDKWAFTPLHEAAQKGRTQL           760
XP_024109627.1[P.abelii]             HGADVNAQDKGGLIPLHNAASYGHVDVAALLIKYNACVNATDKWAFTPLHEAAQKGRTQL           760
XP_032806710.1[P.marinus]            HGADVNAQDKGGLIPLHNAASYGHVDIAALLIKYSACVNATDKWAFTPLHEAAQKGRTQL           834
XP_019641281.1[B.belcheri]           NGADVNAQDKGGLIPLHNASSYGHVEIAQLLIKYGTCVNATDRWNFTPLHEAAQKGRTQL           756
                                       *:**: *****:*****:****:::* ***:: : :**.*:* **************:

XP_019848937.1[A.queenslandica]      CSLLIIHGADVYLKNQEGQIPLDLATADDVIALLQDAMMKDIPL-TIPPAEKEAKSNIV-           830
NP_651410.1[D.melanogaster]          CSLLLAHGADAYMKNQEGQTPIELATADDVKCLLQDAMATSLSQQALSASTQSLTSSSP-           813
XP_047128184.1[H.vulgaris]           CALLLTHGANANMKNQEGQTPIDLATADDVQVLLQDSMVSKSEVMQITSNTCSIKSPINS           828
XP_032220531.1[N.vectensis]          CALLLAHGAEPSMKNQEGQSPVDLATAEDVKCLLGDAMLSTQTTSVSTAPVAKVPAPVTA           826
XP_014034742.2[S.salar]              CALLLAHGADPALRNQEGQSPLDLVTVDDVRALLTAAMPPSALPGCYKPQVISMSSPVGV           849
XP_018082988.1[X.laevis]             CALLLAHGADPALKNQEGQAPLDLVTADDVRALLTAAMPPAALPTFYKPQLLNVPQPSTT           821
NP_989672.2[G.gallus]                CALLLAHGADPTLKNQEGQTPLDLVTADDVSALLTAAMPPSALPSCYKPQVISVSQTASS           821
NP_001157107.1[M.musculus]           CALLLAHGADPTLKNQEGQTPLDLVSADDVSALLTAAMPPSALPTCYKPQVLSGVRGPGA           820
NP_079511.1[H.sapiens]               CALLLAHGADPTLKNQEGQTPLDLVSADDVSALLTAAMPPSALPSCYKPQVLNGVRSPGA           820
XP_024109627.1[P.abelii]             CALLLAHGADPTLKNQEGQTPLDLVSADDVSALLTAAMPPSALPSCYKPQVLNGVRSPGA           820
XP_032806710.1[P.marinus]            CALLLAHGADPSMKNQEGQTPLDLATADDVRALLIDAMPPEALPVTHKPPSIPTIAATSA           894
XP_019641281.1[B.belcheri]           CALLLAHGADPTMKNQEGQTPLDLATAEDVRALLVDAMPPQSLPAAITAATVKAA-----           811
                                     *:**: ***:  ::***** *::*.:.:**  **  :*

XP_019848937.1[A.queenslandica]      ---------------------N---------------------KGLTAAGASLLASELV           847
NP_651410.1[D.melanogaster]          -------------------APDATAAAAPGTS-------SSSSSAILSPTTETVL-L-PT           845
XP_047128184.1[H.vulgaris]           PQVLP-------ISNDLSPQLE---------------------PQGLT-----------           848
XP_032220531.1[N.vectensis]          TAGAA-----------------------------TPATGPISGMAPLSQANSSLLAN---           854
XP_014034742.2[S.salar]              VVP-------------------------------PSLSASSTPLSTLASRNS--LDN-QA           875
XP_018082988.1[X.laevis]             AIA-------------------------------ALSSVPLSS-PTLSAASS--LDN-L-           845
NP_989672.2[G.gallus]                TAD-------------------------------PLSSVPSSP-SSLSAASS--LDN-L-           845
NP_001157107.1[M.musculus]           TAD-------------------------------ALSSGPSSP-SSLSAASS--LDN-L-           844
NP_079511.1[H.sapiens]               TAD-------------------------------ALSSGPSSP-SSLSAASS--LDN-L-           844
XP_024109627.1[P.abelii]             PAD-------------------------------ALSSGPSSP-SSLSAASS--LDN-L-           844
XP_032806710.1[P.marinus]            AATAALALGPTPALASLAPSPVPSGASASGAGGVPLLASPSSTPASLSAASS--MDS-LT           951
XP_019641281.1[B.belcheri]           -------------------SPIS-----------SPSTTPSSTPGVLSTASS--MDH-LV           838
                                                                                   *:

XP_019848937.1[A.queenslandica]      LGDGVDDKNGMVTQQRGGPAGVGDGSDKGFM-------------SYRGGGDAPPPWHNVT           894
```

```
NP_651410.1[D.melanogaster]          GASMILSVPVPLPLSSSTRISPAQG--------AEANGAEGSSSD----DLLPDADTITN           893
XP_047128184.1[H.vulgaris]           ---------------K--PSTANQNLL-----LSEPCNSDGLQTKRLRDQAEGTNPLDID           886
XP_032220531.1[N.vectensis]          ---------------S--PAGHGDGAFSNRSV-SEA------QGEAVRSISAVHPGLDVD           890
XP_014034742.2[S.salar]              TASTCTAFPELPAL------------------LGPSGAV-------GTDNKEVPGVDLS           909
XP_018082988.1[X.laevis]             ----ASSFSETTSVSS-----------------GGAEGAS-------GLDK-KDEGVDLN           876
NP_989672.2[G.gallus]                ----SGSFSELPSVVG----------------TNSAEGAT-------VLEKKEVSGVDFS           878
NP_001157107.1[M.musculus]           ----SGSFSELSAVVS----------------SSAAEGAT-------GLQRKEDSGIDFS           877
NP_079511.1[H.sapiens]               ----SGSFSELSSVVS----------------SSGTEGAS-------SLEKKEVPGVDFS           877
XP_024109627.1[P.abelii]             ----SGSFSELSSVVS----------------SSGTEGTS-------SLEKKEVPGVDFS           877
XP_032806710.1[P.marinus]            GACGVAPSPVPAGAIG--AGAAGDGTLLGACGVSAADGATGPADKPDRSADFEIPGLDMN           1009
XP_019641281.1[B.belcheri]           GAVGGA--------------GQGDGA-------------------IDRSAVEGESLLDMT           865


XP_019848937.1[A.queenslandica]      VKDILTELELGHLVELFEREQITIDILIEMNGDDLQSIGITAFGVRHRLLKRIRELVQGN           954
NP_651410.1[D.melanogaster]          VSGFLSSQQLHHLIELFEREQITLDILAEMGHDDLKQVGVSAYGFRHKILKGIAQLRSTT           953
XP_047128184.1[H.vulgaris]           IGTFLSSLQLEFLHEIFSREQITMDVLIEMGPDQLKEIGIVAYGHRHKILKGVKERLAGA           946
XP_032220531.1[N.vectensis]          VGQFLDGLQLNNLKEIFEREQISWDVLVDMGHEELKEIGIHAYGHRHKILKAVKEKISGM           950
XP_014034742.2[S.salar]              IGQFLNNLGLEHLLEIFEREQITLDVLVEMGHKELKEIGINAYGHRHKIIKGVERLISGP           969
XP_018082988.1[X.laevis]             INQFLRNLGLEHLIDVFEREQITLDVLIEMGHKEFKEIGISAYGHRHKIIKGVERLISGQ           936
NP_989672.2[G.gallus]                INQFVRNLGLEHLIDIFEREQITLDVLVEMGHKELKEIGINAYGHRHKIIKGVERLISGQ           938
NP_001157107.1[M.musculus]           ITQFIRNLGLEHLMDIFEREQITLDVLVEMGHKELKEIGINAYGHRHKLIKGVERLISGQ           937
NP_079511.1[H.sapiens]               ITQFVRNLGLEHLMDIFEREQITLDVLVEMGHKELKEIGINAYGHRHKLIKGVERLISGQ           937
XP_024109627.1[P.abelii]             ITQFLRNLGLEHLMDIFEREQITLDVLVEMGHKELKEIGINAYGHRHKLIKGVERLISGQ           937
XP_032806710.1[P.marinus]            INMFLKGLGLDHLRDIFEKEQITLDVLADMGHEELKEIGINAYGHRHKLIKGVERLLGAQ           1069
XP_019641281.1[B.belcheri]           ISSFLQQLGLNQLLDIFNKEQITLDILGEMGHEELKEIGVNAYGHRHKLIKGVERILGGT           925
                                     :  ::    *  * ::*.:***: *:* :*. .:::.:*: *:* **:::* : .

XP_019848937.1[A.queenslandica]      NEEY-----PVGVTTTKPTQGTQLIELSSDDKEFIDTADLMQSTICEHRDDGKAGGVFDS           1009
NP_651410.1[D.melanogaster]          GIGN------------NVNLCTLLVDLLPDDKEFVAVEEEMQATIREHRDNGQAGGYFTR           1001
XP_047128184.1[H.vulgaris]           GCTIV--GEPFLSNINASGPNTILQELSMDEKDYQSVAEEMQSTVVEHKDGGVAGGIFSR           1004
XP_032220531.1[N.vectensis]          GLG-----LGPFST--SQVQGSVIQELSMVDKDFASVADEMQSTIVEHRDSGTAGGVFTS           1003
XP_014034742.2[S.salar]              Q------SLNPYLTLNTANSGTILIDLVSDDKEFQLVEEELQSTIREHRDGGLAGGVFNR           1023
XP_018082988.1[X.laevis]             H------GLNPYLTLNTSNSGTILIDLAPDDKEFLSVEEEMQSTVREHRDGGHAGGVFTK           990
NP_989672.2[G.gallus]                Q------GLNPYLTLNTSSSGTLLIDLSSEDKEFQSVEEEMQSTVREHRDGGHAGGVFNR           992
NP_001157107.1[M.musculus]           Q------GLNPYLTLNNSGSGTILIDLSPDDKEFQSVEEEMQSTVREHRDGGHAGGVFNR           991
NP_079511.1[H.sapiens]               Q------GLNPYLTLNTSGSGTILIDLSPDDKEFQSVEEEMQSTVREHRDGGHAGGIFNR           991
XP_024109627.1[P.abelii]             Q------GLNPYLTLNTSGSGTILIDLSPDDKEFQSVEEEMQSTVREHRDGGHAGGIFNR           991
XP_032806710.1[P.marinus]            QGGLAYPGANPYLSYHTTAQGTLLIDLPPDDKEYQSVEDEMQSTIREHKDGGQAGGVFNR           1129
XP_019641281.1[B.belcheri]           GT-----TLNPYLTPALGSHGTILTDLSPDDKEYQSVEEEVQSTIRQHKDQGQAGGIFNR           980
                                                          : : :*   :*::  . : :*:*: :*:* * *** *

XP_019848937.1[A.queenslandica]      YEILKIERIVNTKVWERYKYRRKEVAESNNNCANELMLFHGSPFVPYIVHNGFDERHAYI           1069
NP_651410.1[D.melanogaster]          YNIIRVQKVQNRKLWERYAHRRQEIAEENFLQSNERMLFHGSPFINAIVQRGFDERHAYI           1061
XP_047128184.1[H.vulgaris]           YSSLKIERVINKKLWDKYYYQRKQVADANHNHPNERMLFHGSPFVNAIVQKGFDERHAYI           1064
XP_032220531.1[N.vectensis]          YTILKIERVVNTKLWEKYVYRRREIADSNHNHANERMLFHGSPFINAIVQKGFDQRHAYI           1063
XP_014034742.2[S.salar]              YNIVKIQKVCNKKLWERYTHRRKEVSEENHNHSNERMLFHGSPFVNAIIHKGFDERHAYI           1083
XP_018082988.1[X.laevis]             YNILKIQKVCNKKLWEKYTHRQKEVTEENHNHSNERMLFHGSPFVNAIIHKGFDERHAYI           1050
NP_989672.2[G.gallus]                YNILKIQKVCNKKLWERYTHRRKEVSEENHNHANERMLFHGSPFVNAIIHKGFDERHAYI           1052
NP_001157107.1[M.musculus]           YNILKIQKVCNKKLWERYTHRRKEVSEENHNHANERMLFHGSPFVNAIIHKGFDERHAYI           1051
NP_079511.1[H.sapiens]               YNILKIQKVCNKKLWERYTHRRKEVSEENHNHANERMLFHGSPFVNAIIHKGFDERHAYI           1051
XP_024109627.1[P.abelii]             YNILKIQKVCNKKLWERYTHRRKEVSEENHNHANERMLFHGSPFVNAIIHKGFDERHAYI           1051
XP_032806710.1[P.marinus]            YNVIKIQKVRNKKLWERYTHRRKEVTEENHNHHNERMLFHGSPFINAIINKGFDERHAYI           1189
XP_019641281.1[B.belcheri]           YNVIKIQKVRNKRLWDRYVHRRKEVSEENHNQSNERMLFHGSPFINAIINKGFDERHAYI           1040
                                     *  :::::: * ::*::* :::::::: *    ** ********:  *::.***:*****

XP_019848937.1[A.queenslandica]      GGMFGAGIYFAEHSSKSNQYVYGIGGGNGCPEHKNRSCYTCLRKLLLCRVVLGKPVEQYT           1129
NP_651410.1[D.melanogaster]          GGMFGAGIYFAEHSSKSNQYVYGIGGGIGCPSHKDKSCYVCPRQLLLCRVALGKSFLQYS           1121
XP_047128184.1[H.vulgaris]           GGMFGAGIYFAEHSSKSNQYVYGIGGGSGCPVHKDRSCYDCKRQIILCRVTLGKPFFQNS           1124
XP_032220531.1[N.vectensis]          GGMFGAGIYFAENSSKSNQYVYGIGGGSGCPTHKDRSCYSCERQLLLCRVALGKPFYQFS           1123
XP_014034742.2[S.salar]              GGMFGAGIYFAENSSKSNQYVYGIGGGTGCPLHKDRSCYVCQRHLLFCRVTLGKSFLQFS           1143
XP_018082988.1[X.laevis]             GGMFGAGIYFAENSSKSNQYVYGIGGGTGCPIHKDRSCYVCHRQLLFCRVTLGKSFLQFS           1110
NP_989672.2[G.gallus]                GGMFGAGIYFAENSSKSNQYVYGIGGGTGCPIHKDRSCYVCHRQLLFCRVTLGKSFLQFS           1112
NP_001157107.1[M.musculus]           GGMFGAGIYFAENSSKSNQYVYGIGGGTGCPIHKDRSCYICHRQLLFCRVTLGKSFLQFS           1111
NP_079511.1[H.sapiens]               GGMFGAGIYFAENSSKSNQYVYGIGGGTGCPVHKDRSCYICHRQLLFCRVTLGKSFLQFS           1111
XP_024109627.1[P.abelii]             GGMFGAGIYFAENSSKSNQYVYGIGGGTGCPVHKDRSCYICHRQLLFCRVTLGKSFLQFS           1111
XP_032806710.1[P.marinus]            GGMFGAGIYFAENSSKSNQYVYGIGGGTGCPVHKDRSCYICQRQMLFCRVTLGKSFLQFS           1249
XP_019641281.1[B.belcheri]           GGMFGAGIYFAENSSKSNQYVYGIGGGTGCPIHKDRSCYICQRQMLFCRVTLGKAFLQFS           1100
                                     ************:************** *** **::*** * *::::***.*** . * :

XP_019848937.1[A.queenslandica]      AVRIAHAPPGHHSVIGRPSAGGLNYPEYVIYRGEQAYPEYIITFRIKKPSATDSMSSSSS           1189
NP_651410.1[D.melanogaster]          AMKMAHAPPGHHSVVGRPSAGGLHFAEYVVYRGEQSYPEYLITYQIVKPDDSSSGTEDTR           1181
XP_047128184.1[H.vulgaris]           AMKMAHAPPGHHSVIGRPNSGGLSYPEYVVYRGEQAYPEYLITYKIERPNTLSSCQSAVD           1184
XP_032220531.1[N.vectensis]          AVKMAHAPPGHNSVIGRPSSGGLSFAEYVIYRGEQAYPEYLITYKINKPDT---------           1174
XP_014034742.2[S.salar]              AMKMAHSPPGHHSVTGRPSVNGLSLAEYVIYRGEQAYPEYLITYQILKPDASVDG-----           1198
XP_018082988.1[X.laevis]             AMKMAHSPPGHHSVTGRPSVNGLALAEYVIYRGEQAYPEYIITYQIMKPEAVAEG-----           1165
NP_989672.2[G.gallus]                AMKMAHSPPGHHSVTGRPSVNGLALAEYVIYRGEQAYPEYLITYQIVKPEATTEA-----           1167
NP_001157107.1[M.musculus]           AMKMAHSPPGHHSVTGRPSVNGLALAEYVIYRGEQAYPEYLITYQIVRPEGMVDG-----           1166
NP_079511.1[H.sapiens]               AMKMAHSPPGHHSVTGRPSVNGLALAEYVIYRGEQAYPEYLITYQIMRPEGMVDG-----           1166
XP_024109627.1[P.abelii]             AMKMAHSPPGHHSVTGRPSVNGLALAEYVIYRGEQAYPEYLITYQIMRPEGMVDG-----           1166
XP_032806710.1[P.marinus]            AMKMAHSPPGHHSVIGRPSVNGLAFAEYVIYRGEQAYPEYLITYQILKPECTPDLTSAAG           1309
XP_019641281.1[B.belcheri]           AMKMAHAPPGHHSVIGRPSVGGLNFAEYVIYRGEQAFPEYLITYQIVKPEAPAAPSPDQK           1160
                                     *:::**:****:** ***. .**   ***:*****::***:**::* :*.

XP_019848937.1[A.queenslandica]      LDMSNNT    1196
NP_651410.1[D.melanogaster]          -------    1181
XP_047128184.1[H.vulgaris]           -------    1184
XP_032220531.1[N.vectensis]          -------    1174
XP_014034742.2[S.salar]              -------    1198
XP_018082988.1[X.laevis]             -------    1165
NP_989672.2[G.gallus]                -------    1167
NP_001157107.1[M.musculus]           -------    1166
NP_079511.1[H.sapiens]               -------    1166
XP_024109627.1[P.abelii]             -------    1166
XP_032806710.1[P.marinus]            QKS----    1312
XP_019641281.1[B.belcheri]           -------    1160
```

Percent identity matrix:

```
#
#
#  Percent Identity  Matrix - created by Clustal2.1
#
#

     1: XP_019848937.1[A.queenslandica]  100.00   56.09   56.91   60.57   56.97   58.58   59.06   58.93   58.93   59.02   57.73   58.79
     2: NP_651410.1[D.melanogaster]       56.09  100.00   60.07   65.63   64.35   66.16   66.55   66.52   66.34   66.34   65.70   68.40
     3: XP_047128184.1[H.vulgaris]        56.91   60.07  100.00   67.19   63.13   64.35   65.11   65.26   65.08   65.26   63.82   65.67
     4: XP_032220531.1[N.vectensis]       60.57   65.63   67.19  100.00   67.66   69.42   70.76   70.21   70.21   70.12   70.17   71.87
     5: XP_014034742.2[S.salar]           56.97   64.35   63.13   67.66  100.00   81.77   84.71   83.23   83.92   83.92   76.38   73.16
     6: XP_018082988.1[X.laevis]          58.58   66.16   64.35   69.42   81.77  100.00   88.76   87.29   87.97   87.97   80.43   77.10
     7: NP_989672.2[G.gallus]             59.06   66.55   65.11   70.76   84.71   88.76  100.00   92.80   93.65   93.40   82.26   78.00
     8: NP_001157107.1[M.musculus]        58.93   66.52   65.26   70.21   83.23   87.29   92.80  100.00   97.26   97.00   81.56   78.43
     9: NP_079511.1[H.sapiens]            58.93   66.34   65.08   70.21   83.92   87.97   93.65   97.26  100.00   99.66   81.90   78.16
    10: XP_024109627.1[P.abelii]          59.02   66.34   65.26   70.12   83.92   87.97   93.40   97.00   99.66  100.00   81.90   78.25
    11: XP_032806710.1[P.marinus]         57.73   65.70   63.82   70.17   76.38   80.43   82.26   81.56   81.90   81.90  100.00   80.00
    12: XP_019641281.1[B.belcheri]        58.79   68.40   65.67   71.87   73.16   77.10   78.00   78.43   78.16   78.25   80.00  100.00
```
