# Supplementary material for: An Evolutionary Perspective on the Origin, Conservation and Binding Partner Acquisition of Tankyrases
Source: Biomolecules. 2022 Nov 15;12(11):1688. doi: 10.3390/biom12111688 (PMC9688111; doi:10.3390/biom12111688)
Supplement: Supplementary file 1 [file biomolecules-12-01688-s001.zip › biomolecules-1913719-supplementary-11.17 -done/Data_5.pdf]

>TNKS1[H.sapiens][NP_003738.2]
MAASRRSQHHHHHHQQQLQPAPGASAPPPPPPPPLSPGLAPGTTPASPTASGLAPFASPRHGLALPEGDGSRDPPDRPRSPDP
VDGTSCCSTTSTICTVAAAPVVPAVSTSSAAGVAPNPAGSGSNNSPSSSSSPTSSSSSSPSSPGSSLAESPEAAGVSSTAPLG
PGAAGPGTGVPAVSGALRELLEACRNGDVSRVKRLVDAANVNAKDMAGRKSSPLHFAAGFGRKDVVEHLLQMGANVHARDDGG
LIPLHNACSFGHAEVVSLLLCQGADPNARDNWNYTPLHEAAIKGKIDVCIVLLQHGADPNIRNTDGKSALDLADPSAKAVLTG
EYKKDELLEAARSGNEEKLMALLTPLNVNCHASDGRKSTPLHLAAGYNRVRIVQLLLQHGADVHAKDKGGLVPLHNACSYGHY
EVTELLLKHGACVNAMDLWQFTPLHEAASKNRVEVCSLLLSHGADPTLVNCHGKSAVDMAPTPELRERLTYEFKGHSLLQAAR
EADLAKVKKTLALEIINFKQPQSHETALHCAVASLHPKRKQVTELLLRKGANVNEKNKDFMTPLHVAAERAHNDVMEVLHKHG
AKMNALDTLGQTALHRAALAGHLQTCRLLLSYGSDPSIISLQGFTAAQMGNEAVQQILSESTPIRTSDVDYRLLEASKAGDLE
TVKQLCSSQNVNCRDLEGRHSTPLHFAAGYNRVSVVEYLLHHGADVHAKDKGGLVPLHNACSYGHYEVAELLVRHGASVNVAD
LWKFTPLHEAAAKGKYEICKLLLKHGADPTKKNRDGNTPLDLVKEGDTDIQDLLRGDAALLDAAKKGCLARVQKLCTPENINC
RDTQGRNSTPLHLAAGYNNLEVAEYLLEHGADVNAQDKGGLIPLHNAASYGHVDIAALLIKYNTCVNATDKWAFTPLHEAAQK
GRTQLCALLLAHGADPTMKNQEGQTPLDLATADDIRALLIDAMPPEALPTCFKPQATVVSASLISPASTPSCLSAASSIDNLT
GPLAELAVGGASNAGDGAAGTERKEGEVAGLDMNISQFLKSLGLEHLRDIFETEQITLDVLADMGHEELKEIGINAYGHRHKL
IKGVERLLGGQQGTNPYLTFHCVNQGTILLDLAPEDKEYQSVEEEMQSTIREHRDGGNAGGIFNRYNVIRIQKVVNKKLRERF
CHRQKEVSEENHNHHNERMLFHGSPFINAIIHKGFDERHAYIGGMFGAGIYFAENSSKSNQYVYGIGGGTGCPTHKDRSCYIC
HRQMLFCRVTLGKSFLQFSTMKMAHAPPGHHSVIGRPSVNGLAYAEYVIYRGEQAYPEYLITYQIMKPEAPSQTATAAEQKT

>TNKS2[H.sapiens][AAK13463.1]
MSGRRCAGGGAACASAAAEAVEPAARELFEACRNGDVERVKRLVTPEKVNSRDTAGRKSTPLHFAAGFGRKDVVEYLLQNGAN
VQARDDGGLIPLHNACSFGHAEVVNLLLRHGADPNARDNWNYTPLHEAAIKGKIDVCIVLLQHGAEPTIRNTDGRTALDLADP
SAKAVLTGEYKKDELLESARSGNEEKMMALLTPLNVNCHASDGRKSTPLHLAAGYNRVKIVQLLLQHGADVHAKDKGDLVPLH
NACSYGHYEVTELLVKHGACVNAMDLWQFTPLHEAASKNRVEVCSLLLSYGADPTLLNCHNKSAIDLAPTPQLKERLAYEFKG
HSLLQAAREADVTRIKKHLSLEMVNFKHPQTHETALHCAAASPYPKRKQICELLLRKGANINEKTKEFLTPLHVASEKAHNDV
VEVVVKHEAKVNALDNLGQTSLHRAAYCGHLQTCRLLLSYGCDPNIISLQGFTALQMGNENVQQLLQEGISLGNSEADRQLLE
AAKAGDVETVKKLCTVQSVNCRDIEGRQSTPLHFAAGYNRVSVVEYLLQHGADVHAKDKGGLVPLHNACSYGHYEVAELLVKH
GAVVNVADLWKFTPLHEAAAKGKYEICKLLLQHGADPTKKNRDGNTPLDLVKDGDTDIQDLLRGDAALLDAAKKGCLARVKKL
SSPDNVNCRDTQGRHSTPLHLAAGYNNLEVAEYLLQHGADVNAQDKGGLIPLHNAASYGHVDVAALLIKYNACVNATDKWAFT
PLHEAAQKGRTQLCALLLAHGADPTLKNQEGQTPLDLVSADDVSALLTAAMPPSALPSCYKPQVLNGVRSPGATADALSSGPS
SPSSLSAASSLDNLSGSFSELSSVVSSSGTEGASSLEKKEVPGVDFSITQFVRNLGLEHLMDIFEREQITLDVLVEMGHKELK
EIGINAYGHRHKLIKGVERLISGQQGLNPYLTLNTSGSGTILIDLSPDDKEFQSVEEEMQSTVREHRDGGHAGGIFNRYNILK
IQKVCNKKLWERYTHRRKEVSEENHNHANERMLFHGSPFVNAIIHKGFDERHAYIGGMFGAGIYFAENSSKSNQYVYGIGGGT
GCPVHKDRSCYICHRQLLFCRVTLGKSFLQFSAMKMAHSPPGHHSVTGRPSVNGLALAEYVIYRGEQAYPEYLITYQIMRPEG
MVDG

>TNKS1[P.abelii][XP_024106876.1]
MAASRRSQHHHHHHQQQLQPAPGASAPPPPPPPPLSPGLAPGTTPASPTASGLAPFASPRHGLALPEGDGSRDPPDRPRSPDP
VDGTSCCSTTSTICTVTAAPVVPAVSTSSAVGVAPNPAGSGSNNSPSSSSSPTSSSSSSPSSPGSSLAESPEAAGVSSTAPLG
PGAAGPGTGVPAVSGALRELLEACRNGDVSRVKRLVDAANVNAKDMAGRKSSPLHFAAGFGRKDVVEHLLQMGANVHARDDGG
LIPLHNACSFGHAEVVSLLLCQGADPNARDNWNYTPLHEAAIKGKIDVCIVLLQHGADPNIRNTDGKSALDLADPSAKAVLTG
EYKKDELLEAARSGNEEKLMALLTPLNVNCHASDGRKSTPLHLAAGYNRVRIVQLLLQHGADVHAKDKGGLVPLHNACSYGHY
EVTELLLKHGACVNAMDLWQFTPLHEAASKNRVEVCSLLLSHGADPTLVNCHGKSAVDMAPTPELRERLTYEFKGHSLLQAAR
EADLAKVKKTLALEIINFKQPQSHETALHCAVASLHPKRKQVTELLLRKGANVNEKNKDFMTPLHVAAERAHNDVMEVLHKHG
AKMNALDTLGQTALHRAALAGHLQTCRLLLSYGSDPSIISLQGFTAAQMGNEAVQQILSESTPIRTSDVDYRLLEASKAGDLE
TVKQLCSPQNVNCRDLEGRHSTPLHFAAGYNRVSVVEYLLHHGADVHAKDKGGLVPLHNACSYGHYEVAELLVRHGASVNVAD
LWKFTPLHEAAAKGKYEICKLLLKHGADPTKKNRDGNTPLDLVKEGDTDIQDLLRGDAALLDAAKKGCLARVQKLCTPENINC
RDTQGRNSTPLHLAAGYNNLEVAEYLLEHGADVNAQDKGGLIPLHNAASYGHVDIAALLIKYNTCVNATDKWAFTPLHEAAQK
GRTQLCALLLAHGADPTMKNQEGQTPLDLATADDIRALLIDAMPPEALPTCFKPQATVVSASLISPASTPSCLSAASSIDNLT
GPLAELAVGGASNAGDGAAGTERKEGEIAGLDMNISQFLKSLGLEHLRDIFETEQITLDVLADMGHEELKEIGINAYGHRHKL
IKGVERLLGGQQGTNPYLTFHCVNQGTILLDLAPEDKEYQSVEEEMQSTIREHRDGGNAGGIFNRYNVIRIQKVVNKKLRERF
CHRQKEVSEENHNHHNERMLFHGSPFINAIIHKGFDERHAYIGGMFGAGIYFAENSSKSNQYVYGIGGGTGCPTHKDRSCYIC
HRQMLFCRVTLGKSFLQFSTMKMAHAPPGHHSVIGRPSVNGLAYAEYVIYRGEQAYPEYLITYQIMKPEAPSQTATAAEQKT

>TNKS2[P.abelii][XP_024109627.1]
MSGRRCAGGGAACASAAAEAMEPAARELFEACRNGDVERVKRLVTPEKVNSRDTAGRKSTPLHFAAGFGRKDVVEYLLQNGAN
VQARDDGGLIPLHNACSFGHAEVVNLLLRHGADPNARDNWNYTPLHEAAIKGKIDVCIVLLQHGAEPTIRNTDGRTALDLADP
SAKAVLTGEYKKDELLESARSGNEEKMMALLTPLNVNCHASDGRKSTPLHLAAGYNRVKIVQLLLQHGADVHAKDKGDLVPLH
NACSYGHYEVTELLVKHGACVNAMDLWQFTPLHEAASKNRVEVCSLLLSYGADPTLLNCHNKSAIDLAPTPQLKERLAYEFKG
HSLLQAAREADVTRIKKHLSLEMVNFKHPQTHETALHCAAASPYPKRKQICELLLRKGANINEKTKEFLTPLHVASEKAHNDV
VEVVVKHEAKVNALDNLGQTSLHRAAYCGHLQTCRLLLSYGCDPNIISLQGFTALQMGNENVQQLLQEGISLGNSEADRQLLE
AAKAGDVETVKKLCTVQSVNCRDIEGRQSTPLHFAAGYNRVSVVEYLLQHGADVHAKDKGGLVPLHNACSYGHYEVAELLVKH
GAVVNVADLWKFTPLHEAAAKGKYEICKLLLQHGADPTKKNRDGNTPLDLVKDGDTDIQDLLRGDAALLDAAKKGCLARVKKL
SSPDNVNCRDTQGRHSTPLHLAAGYNNLEVAEYLLQHGADVNAQDKGGLIPLHNAASYGHVDVAALLIKYNACVNATDKWAFT
PLHEAAQKGRTQLCALLLAHGADPTLKNQEGQTPLDLVSADDVSALLTAAMPPSALPSCYKPQVLNGVRSPGAPADALSSGPS
SPSSLSAASSLDNLSGSFSELSSVVSSSGTEGTSSLEKKEVPGVDFSITQFLRNLGLEHLMDIFEREQITLDVLVEMGHKELK
EIGINAYGHRHKLIKGVERLISGQQGLNPYLTLNTSGSGTILIDLSPDDKEFQSVEEEMQSTVREHRDGGHAGGIFNRYNILK
IQKVCNKKLWERYTHRRKEVSEENHNHANERMLFHGSPFVNAIIHKGFDERHAYIGGMFGAGIYFAENSSKSNQYVYGIGGGT

GCPVHKDRSCYICHRQLLFCRVTLGKSFLQFSAMKMAHSPPGHHSVTGRPSVNGLALAEYVIYRGEQAYPEYLITYQIMRPEG
MVDG

>TNKS1[M.musculus][EDL35446.1]
GKMAASRRSQHHHHHHQQQLQPAPGASAPPPPPPPPLSPGLAPGPTPASPTAGGLAPFASPRHGLALPEGDGSRDPPDRPRSP
DPVDGAVCTVAAPAAVPAASAAVGVAPTPAGGGGGGGNNSASSASSPTSSSSSSPSSPGSSLAESPEAAGVGSTATLGAGAAG
LGPGVPAVSGALRELLEACRNGDVSRVKRLVDAANVNAKDMAGRKSSPLHFAAGFGRKDVVEHLLQMGANVHARDDGGLIPLH
NACSFGHAEVVSLLLCQGADPNARDNWNYTPLHEAAIKGKIDVCIVLLQHGADPNIRNTDGKSALDLADPSAKAVLTGEYKKD
ELLEAARSGNEEKLMALLTPLNVNCHASDGRKSTPLHLAAGYNRVRIVQLLLQHGADVHAKDKGGLVPLHNACSYGHYEVTEL
LLKHGACVNAMDLWQFTPLHEAASKNRVEVCSLLLSHGADPTLVNCHGKSAVDMAPTPELRERLTYEFKGHSLLQAAREADLA
KVKKTLALEIINFKQPQSHETALHCAVASLHPKRKQVAELLLRKGANVNEKNKDFMTPLHVAAERAHNDVMEVLHKHGAKMNA
LDSLGQTALHRAALAGHLQTCRLLLSYGSDPSIISLQGFTAAQMGNEAVQQILSESTPMRTSDVDYRLLEASKAGDLETVKQL
CSPQNVNCRDLEGRHSTPLHFAAGYNRVSVVEYLLHHGADVHAKDKGGLVPLHNACSYGHYEVAELLVRHGASVNVADLWKFT
PLHEAAAKGKYEICKLLLKHGADPTKKNRDGNTPLDLVKEGDTDIQDLLRGDAALLDAAKKGCLARVQKLCTPENINCRDTQG
RNSTPLHLAAGYNNLEVAEYLLEHGADVNAQDKGGLIPLHNAASYGHVDIAALLIKYNTCVNATDKWAFTPLHEAAQKGRTQL
CALLLAHGADPTMKNQEGQTPLDLATADDIRALLIDAMPPEALPTCFKPQATVVSASLISPASTPSCLSAASSIDNLTGPLTD
LAVGGASNAGDGAAGAERKEGEVAGLDMNISQFLKSLGLEHLRDIFETEQITLDVLADMGHEELKEIGINAYGHRHKLIKGVE
RLLGGQQGTNPYLTFHCVNQGTILLDLAPEDKEYQSVEEEMQSTIREHRDGGNAGGIFNRYNVIRIQKVVNKKLRERFCHRQK
EVSEENHNHHNERMLFHGSPFINAIIHKGFDERHAYIGGMFGAGIYFAENSSKSNQYVYGIGGGTGCPTHKDRSCYICHRQML
FCRVTLGKSFLQFSTMKMAHAPPGHHSVIGRPSVNGLAYAEYVIYRGEQAYPEYLITYQIMKPEAPSQTATAAEQKT

>TNKS2[M.musculus][NP_001157107.1]
MSGRRCAGGGAACASAGAEAVEPSARELFEACRNGDVERVKRLVTPEKVNSRDTAGRKSTPLHFAAGFGRKDVVEYLLQNGAN
VQARDDGGLIPLHNACSFGHAEVVNLLLQHGADPNARDNWNYTPLHEAAIKGKIDVCIVLLQHGAEPTIRNTDGRTALDLADP
SAKAVLTGDYKKDELLESARSGNEEKMMALLTPLNVNCHASDGRKSTPLHLAAGYNRVKIVQLLLHHGADVHAKDKGDLVPLH
NACSYGHYEVTELLVKHGACVNAMDLWQFTPLHEAASKNRIEVCSLLLSYGADPTLLNCHNKSAIDLAPTAQLKERLSYEFKG
HSLLQAAREADVTRIKKHLSLEMVNFKHPQTHETALHCAAASPYPKRKQICELLLRKGANTNEKTKEFLTPLHVASENAHNDV
VEVVVKHEAKVNALDSLGQTSLHRAAHCGHLQTCRLLLSYGCDPNIISLQGFTALQMGNENVQQLLQEGASLGHSEADRQLLE
AAKAGDVETVKKLCTVQSVNCRDIEGRQSTPLHFAAGYNRVSVVEYLLQHGADVHAKDKGGLVPLHNACSYGHYEVAELLVKH
GAVVNVADLWKFTPLHEAAAKGKYEICKLLLQHGADPTKKNRDGNTPLDLVKDGDTDIQDLLRGDAALLDAAKKGCLARVKKL
SSPDNVNCRDTQGRHSTPLHLAAGYNNLEVAEYLLQHGADVNAQDKGGLIPLHNAASYGHVDVAALLIKYNACVNATDKWAFT
PLHEAAQKGRTQLCALLLAHGADPTLKNQEGQTPLDLVSADDVSALLTAAMPPSALPTCYKPQVLSGVRGPGATADALSSGPS
SPSSLSAASSLDNLSGSFSELSAVVSSSAAEGATGLQRKEDSGIDFSITQFIRNLGLEHLMDIFEREQITLDVLVEMGHKELK
EIGINAYGHRHKLIKGVERLISGQQGLNPYLTLNNSGSGTILIDLSPDDKEFQSVEEEMQSTVREHRDGGHAGGVFNRYNILK
IQKVCNKKLWERYTHRRKEVSEENHNHANERMLFHGSPFVNAIIHKGFDERHAYIGGMFGAGIYFAENSSKSNQYVYGIGGGT
GCPIHKDRSCYICHRQLLFCRVTLGKSFLQFSAMKMAHSPPGHHSVTGRPSVNGLALAEYVIYRGEQAYPEYLITYQIVRPEG
MVDG

>TNKS1[G.gallus][NP_989671.1]
MAAPPRRSQHHHHHHGPPPPPGPASPPAAASPPRSPSLAPAELGPAAQRHSLAGPEGEAPPDAERPPAPECSEGAAPGPPPGS
GSSSGSSASSSSSSSSTSSSVASSPAAESPEAAGPSGAFRELLEACRNGDVTRVKRLVDAGNVNAKDMAGRKSTPLHFAAGFG
RKDVVEHLLQTGANVHARDDGGLIPLHNACSFGHAEVVSLLLCQGADPNARDNWNYTPLHEAAIKGKIDVCIVLLQHGADPNI
RNTDGKSALDLADPSAEAVLTGEYKKDELLEAARSGNEEKLMALLTPLNVNCHASDGRKSTPLHLAAGYNRVRIVQLLLQHGA
DVHAKDKGGLVPLHNACSYGHYEVTELLLKHGACVNAMDLWQFTPLHEAASKNRVEVCSLLLSHGADPTLVNCHGKSAVDMAP
TPELRERLTYEFKGHSLLQAAREADLAKVKKTLALEIINFKQPQSHETALHCAVAAVHPKRKQVTELLLRKGANVNEKNKDFM
TPLHVAAEKAHNDVMEVLHKHGAKMNALDTLGQTALHRAALAGHLQTCRLLLNYGSDPSIISLQGFTAAQIGNEAVQQILSES
TPVRTSDVDYRLLEASKAGDLETVKQLCSPQNVNCRDLEGRHSTPLHFAAGYNRVSVVEYLLHHGADVHAKDKGGLVPLHNAC
SYGHYEVAELLVRHGASVNVADLWKFTPLHEAAAKGKYEICKLLLKHGADPTKKNRDGNTPLDLVKEGDTDIQDLLRGDAALL
DAAKKGCLARVQKLCTPENINCRDTQGRNSTPLHLAAGYNNLEVAEYLLEHGADVNAQDKGGLIPLHNAASYGHVDIAALLIK
YNTCVNATDKWAFTPLHEAAQKGRTQLCALLLAHGADPTMKNQEGQTPLDLATADDIRALLIDAMPPEALPTCFKLQATVVSA
SLISPASTPSCLSAASSIDNLTGPLAELAVGGASNTGDGAAGTERKEGEVSGLDMNITQFLKSLGLEHLRDIFETEQITLDVL
ADMGHEELKEIGINAYGHRHKLIKGVERLLGGQQGTNPYLTFHCVSQGTILLDLAPDDKEYQSVEEEMQSTIREHRDGGNAGG
IFNRYNVIRIQKVVNKKLRERFCHRQKEVSEENHNHHNERMLFHGSPFINAIIHKGFDERHAYIGGMFGAGIYFAENSSKSNQ
YVYGIGGGTGCPTHKDRSCYICHRQMLFCRVTLGKSFLQFSTMKMAHAPPGHHSVIGRPSVNGLAYAEYVIYRGEQAYPEYLI
TYQIVKPEAPSQTGTAAEQKT

>TNKS2[G.gallus][NP_989672.1]
MAARRCAGGAAALAEAPGCGSAVEPARELFEACRNGDVERVKRLVRPENVNSRDTAGRKSSPLHFAAGFGRKDVVEYLLQSGA
NVHARDDGGLIPLHNACSFGHAEVVNLLLRHGANPNARDNWNYTPLHEAAIKGKTDVCIVLLQHGAEPTIRNTDGRTALDLAD
PSAKAVLTGEYKKDELLESARSGNEEKMMSLLTPLNVNCHASDGRKSTPLHLAAGYNRVKIVQLLLQHGADVHAKDKGDLVPL
HNACSYGHYEVTELLVKHGACVNAMDLWQFTPLHEAASKNRVEVCSLLLSYGADPTLLNCHNKSTIDLAPTPQLKERLAYEFK
GHSLLQAARESDVARIKKHLSLETVNFKHPQTHETALHCAAASPYPKRKQVCELLLRKGANINEKTKDFLTPLHVASEKAHND
VVEVVVKHEAKVNALDNLGQTSLHRAAHCGHLQTCRLLLSSGCDPSIVSLQGFTALQMGNESVQQLLQEGIPLGNSDADRQLL
EAAKAGDVDTVKKLCTVQSVNCRDIEGRQSTPLHFAAGYNRVSVVEYLLQHGADVHAKDKGGLVPLHNACSYGHYEVAELLVK
HGAVVNVADLWKFTPLHEAAAKGKYEICKLLLQHGADPPKKNRDGNTPLDLVKDGDTDIQDLLRGDAALLDAAKKGCLARVKK
LCSPDNVNCRDTQGRHSTPLHLAAGYNNLEVAEYLLQHGADVNAQDKGGLIPLHNAASYGHVDVAALLIKYNACVNATDKWAF
TPLHEAAQKGRTQLCALLLAHGADPTLKNQEGQTPLDLVTADDVSALLTAAMPPSALPSCYKPQVISVSQTASSTADPLSSVP
SSPSSLSAASSLDNLSGSFSELPSVVGTNSAEGATVLEKKEVSGVDFSINQFVRNLGLEHLIDIFEREQITLDVLVEMGHKEL

KEIGINAYGHRHKIIKGVERLISGQQGLNPYLTLNTSSSGTLLIDLSSEDKEFQSVEEEMQSTVREHRDGGHAGGVFNRYNIL
KIQKVCNKKLWERYTHRRKEVSEENHNHANERMLFHGSPFVNAIIHKGFDERHAYIGGMFGAGIYFAENSSKSNQYVYGIGGG
TGCPIHKDRSCYVCHRQLLFCRVTLGKSFLQFSAMKMAHSPPGHHSVTGRPSVNGLALAEYVIYRGEQAYPEYLITYQIVKPE
ATTEA

>TNKS1[X.laevis][XP_018099068.1]
MAAPSRRSQQQQVPTAAAGQSSQPPSASPPLSPSSRRAASCLSPPCSPETNALSLSREGPDSEPEGEAASQPGITPRPLSPPP
GEAANILSPTSSLPPAPAPSSGSSSSSSSPSSNSLCTGGSPAESPESGVIGGVSSVGIGPGDPLSAVSGAFRELFEACRNGDV
SRVRRLLEPANVNAKDMAGRKSTPLHFAAGFGRKDVVEHLLQTGANVHARDDGGLIPLHNACSFGHAEVVTLLLCQGADPNAR
DNWNYTPLHEASIKGKIDVCIVLLQHGADPNIRNTDGKSALDLADPSSKTVLTGEYKKDELLEAARSGNEEKLMALLTPLNVN
CHASDGRKSTPLHLAAGYNRVQIVQLLLQHGADVHAKDKGGLVPLHNACSYGHFEVTESLLKHGACVNAMDLWQFTPLHEAAS
KNRVEVCSLLLSHGADPTLVNCHGKSAVDMAPTPELKERLSYEFKGHSLLQAAREADLAKVKKTLALEIINFKQPQSHETALH
CAVASLHPKRKQITELLLRKGASVNEKNKDFMTPLHVAAERAHNDVVEVLHKHGAKMNALDTLGQTALHRAALGGHLQTCRLL
LSFGSDASIVSLQGFTAAQMGNEAVQQILNESTPVRTSDVDYRLLEASKAGDLDIVKQLCSSQNVNCRDLEGRHSTPLHFAAG
YNRVSVVEYLLHHGADVHAKDKGGLVPLHNACSYGHYEVAELLVRHGASVNVADLWKFTPLHEAAAKGKYEICKLLLKHGADP
TKKNRDGNTPLDLVKDGDTDIQDLLRGDAALLDAAKKGCLARVQKLCTQENINCRDTQGRNSTPLHLAAGYNNLEVAEYLLEH
GADVNAQDKGGLIPLHNAASYGHVDIAALLIKYNTCVNATDKWAFTPLHEAAQKGRTQLCALLLAHGADPTMKNQESQTPLDL
ATADDIRALLIDAMPPEALPSCFKPQATVVSASIISPPSTPSCLSAASSIDNLTGPLAELAVGGVSNAGDGAAGTERKEGEVT
VLDININQFLKSLGLEHLRDIFETEQITLDVLADMGHEELKEIGINAYGHRHKLIKGVERLLGGQQGTNPYLTFHCVSQGTVL
LDLASDDKEYQSVEEEMQNSIREHRDGGNAGGVFNRYNVIRIQKVVNKKLRERFCHRQKEVSEENHNHHNERMLFHGSPFINA
IIHKGFDERHAYIGGMFGAGIYFAENSSKSNQYVYGIGGGTGCPTHKDRSCYICHRQMLFCRVTLGKSFLQFSTMKMAHAPPG
HHSVIGRPSVNGLAYAEYVIYRGEQAYPEYLITYQIMKPETPLQAATTAEQKT

>TNKS2[X.laevis][XP_018082988.1]
MSGRRCAGASGSAASHGNIGAGGEPARELFEACRNGDVERVRKLVNSDNVNSRDTAGRKSTPLHFAAGFGRKDVVEHLLQSGA
NVHARDDGGLIPIHNACSFGHAEVVSLLLRHGADPNARDNWNYTPLHEAAIKGKIDVCIVLFQHGADPTIRNTDSRTALDLSD
PSAKAVLTGEYKKDELLESARSGNEEKMMALLTPLNVNCHASDGRKSTPLHLAAGYNRVKIVQLLLQHGADVHAKDKGDLVPL
HNACSYGHYEVTELLVKHGASVNAMDLWQFTSLHEAASKNRVEVCSLLLSYGADPTMLNCHNKSAIDLAPTPQLKERLSYEFK
GHSLLQAAREADLTRIKKHLSLEIVNFKHPQTHDTALHCAASSPYPKRKQVCELLFRKGANVNEKTKDFLTPLHIASDKAHND
IVELLIKHEAKVNALDNLGQTSLHRAAHCGHLQTCRLLLNSGCDPSIVSLQGFTALQMGNESVQQLLQEGLPFSNSDADSQLL
EAAKAGDLDVVKKLCTSQSVNCRDVEGRQSTPLHFAAGYNRVAVVEYLLQHGADVHAKDKGGLVALHNACSYGHYEVTELLVK
HGAVINVADLWKFTPLHEAAAKGKYEICKLLLLHGADSTKKNRDGNTPLDLVKDGDTDIQDLLRGDAALLDAAKKGCVSRVKK
LCTPENVNCRDTQGRHSTPLHLAAGYNNLEVAEYLLQHGADVNAADKGGLIPLHNAASYGHVDVAALLIKYNACVNANDKWAF
TPLHEAAQKGRTQLCALLLAHGADPALKNQEGQAPLDLVTADDVRALLTAAMPPAALPTFYKPQLLNVPQPSTTAIAALSSVP
LSSPTLSAASSLDNLASSFSETTSVSSGGAEGASGLDKKDEGVDLNINQFLRNLGLEHLIDVFEREQITLDVLIEMGHKEFKE
IGISAYGHRHKIIKGVERLISGQHGLNPYLTLNTSNSGTILIDLAPDDKEFLSVEEEMQSTVREHRDGGHAGGVFTKYNILKI
QKVCNKKLWEKYTHRQKEVTEENHNHSNERMLFHGSPFVNAIIHKGFDERHAYIGGMFGAGIYFAENSSKSNQYVYGIGGGTG
CPIHKDRSCYVCHRQLLFCRVTLGKSFLQFSAMKMAHSPPGHHSVTGRPSVNGLALAEYVIYRGEQAYPEYIITYQIMKPEAV
AEG

>TNKS1[S.salar][XP_014017013.1]
MAVSRRSSQQQQGNLLSPPRNGSLSVSPPGSPSLNLVTATLVPPDGERGCSTGMENPLASPDLQTSALSSGSSTSPTTTTSGG
GSSSVSSPGSGGTTPGDGSGIGGAFRELFEACRNGDVSRVKRLVDSVNVNAKDMAGRKSTPLHFAAGFGRKDVVDHLLQTGAN
VHARDDGGLIPLHNACSFGHAEVVSLLLCQGADPNARDNWNYTPLHEAAIKGKIDVCIVLLQHGADPNIRNTDGKSALDLADP
SAKAVLTGEYKKDELLEAARSGNEEKLMALLTPLNVNCHASDGRKSTPLHLAAGYNRVRIVQLLLQHGADVHAKDKGGLVPLH
NACSYGHFEVTELLLKHGACVNAMDLWQFTPLHEAASKNRVEVCSLLLSHGADPTLLNCHSKSAVDMAPTPELKERLTYEFKG
HSLLQAAREADMAKVKKTLALEIISFKHPQTNETALHCAVASPHPKRKQVTELLLRKGANINEKNKDFMTALHVASERAHNDI
LEVLQKHGAKVNAVDTLGQTALHRAALAGHIQTCRLLLSYGADPAIVSLQGFTASQMGNEAVQQILNENVPTRNSDVDYRFLE
AAKAGDLDTVQQLCTPQNVNCRDLEGRHSTPLHFAAGYNRVAVVEYLLHHGADVHAKDKGGLVPLHNACSYGHYEVAELLVRH
GASVNVADLWKFTPLHEAAAKGKYEICKLLLKHGADPTKKNRDGNMPLDMVKDGDTDIQDLLRGDAALLDAAKKGCLARVQKL
CSPENINCRDTQGRNSTPLHLAAGYNNLEVAEYLLEHGADVNAQDKGGLIPLHNAASYGHVDIAALLIKFNTCVNATDKWAFT
PLHEAAQKGRTQLCALLLAHGADPTMKNQEGQTALDLATADDIRALLMDAMPPDALPSCFKPQATVVSAGSVISPASTPSCLS
AASSIDNLAGPLNELGAAGTSGVADGATGSDRKEGELVMLDMNISQFLKSLGLEHLRDIFEREQISLDVLADMGHEELKEIGI
NAYGHRHKLIKGVERLLGGQQGANPYLTFHCANQGTVLIDLAPDDKEGQSVEEEMQSTIREHRDGGNAGGVFSRYNIIKIQKV
VNKKLRERYTHRQKEISDENHNHHNERMLFHGSPFINAIIHKGFDERHAYIGGMFGAGIYFAENSSKSNQYVYGIGGGTGCPT
HKDRSCYLCHRQMLFCRVTLGKSFLQFSAMKMAHAPPGHHSVIGRPSVNGLAYAEYVIYRGEQAYPEYLITYQILKPESTATS
AAGEDQKS

>TNKS2[S.salar][XP_014034742.1]
MASVARFWGSANTNSKLYIYENTTPLMSTRQCSRVLGLGGVFPGVDTPRSGEPNREIFEACRNGDVERVRRLVRPENVNSRDT
AGRKSTPLHFAAGFGRRDVVDYLLQNGANVHARDEGGLVSLHNACSFGHSEVVNLLLRHGADANSRDNWSYTPLHEAAIKGKS
EVCIVLLQHGAEPTIRNTDGRTALDLAEASTKAVLTGEYRKDDLLESARSGNEDKLMALLTPLNVNCHASDGRKSSPLHLAAG
YNRVKTVQLLLKHGADVHAKDKGDLVPLHNACSYGHYEVTDILVKQGACVNAMDLWQFTPLHEAASKNRVDVCSLLVSYGADP
TFLNCHNQSAIDLSPTPQLKERLAYEFRGHALLQAAREADLPRLKKHLSLETITFKHPLTHETALHCAATSPYPKRKQVCELL
LRKGANVNEKTKDFLTPLHLASEKSHNDIIEVLVKHEAKVNALDNLGQTALHRAAHCGHLQTCRLLLKAGCDPLVMSLQGFSP
SQMGNESVQEILHEGTLIGNSDVDWQLLEASKSGDLEIVKKLCTMQNVNCRDVEGRQSTPLHFAAGYNRVSVVEYLLLHGADV
HAKDKGGLVPLHNACSYGHYEVAELLVIHGAVVNIADLWKFTPLHEAAAKGKYEICKLLLQHGADPTKKNRDGSTPLDLVKDG
DTDMQDLLRGDAALLDAAKKGCLARVEKLCSPDNVNCRDAQGRHSTLLHLAAGYNNLEVAEYLLQHGAEVNSQDKGGLIPLHN

AASYGHVDVAALLIKYDACVNATDKWAFTPLHEAAQKGRTQLCALLLAHGADPALRNQEGQSPLDLVTVDDVRALLTAAMPPS
ALPGCYKPQVISMSSPVGVVVPPSLSASSTPLSTLASRNSLDNQATASTCTAFPELPALLGPSGAVGTDNKEVPGVDLSIGQF
LNNLGLEHLLEIFEREQITLDVLVEMGHKELKEIGINAYGHRHKIIKGVERLISGPQSLNPYLTLNTANSGTILIDLVSDDKE
FQLVEEELQSTIREHRDGGLAGGVFNRYNIVKIQKVCNKKLWERYTHRRKEVSEENHNHSNERMLFHGSPFVNAIIHKGFDER
HAYIGGMFGAGIYFAENSSKSNQYVYGIGGGTGCPLHKDRSCYVCQRHLLFCRVTLGKSFLQFSAMKMAHSPPGHHSVTGRPS
VNGLSLAEYVIYRGEQAYPEYLITYQILKPDASVDG

>TNKS[P.marinus][XP_032806710.1]
MAARRAHLASPSSSSSLSPSSGSLDTTTAAAAAAAATTTAAAAAATTTAAAMTTTAAVSATSSPSTTAAAAAAVAAAAATGSG
PGSGQDSSADGGDAGKELFEACRNGDVGRVKRLVSPLNVNARDTTGRKSTPLHFAAGFGRKDVVEHLLQNGANVHARDDGGLI
PLHNACSFGHAEVVNLLLRQGADPNARDNWNYTPLHEAAIKGKIDVCIVLLQHGADPGIRNTDGKTALDLAEPTAKAVLTGEY
KKDELLEAARSGNEEKLMALLTPLNVNCHASDGRKSTPLHLAAGYNRVRIVQLLLQHGADVHAKDKGGLVPLHNACSYGHYEV
TELLVKHGASVNAMDLWQFTPLHEAASKNRVEVCSLLLSYGADPTLLNCHSKSAIDLAPTSELKERLVYEFRGHSLLQAAREA
DVTRVKKHLGLDIINFKHPQTHETALHCAVASPYPKRKQVSELLLRKGANVNEKTKDLLTPLHVASDKAHNDVIDVLLKHGAK
INVTDSLGQTSLHRAAHAGHLTTCRLLLSHGCDPNLPSLQGFTAAQLGNESVQQILHESSLPVRNSDVEHQLLEAAKAGDLDT
VKQLCTTQTVNCRDMEGRHSTPLHFAAGYNRVSVVEYLLQHGADVHAKDKGGLVPLHNACSYGHYEVAELLVKRGAVVNVADL
WKFTPLHEAAAKGKYEICKLLLKHGADPTKKNRDGNTPLDLVKEGDTDIQDLLRGDAALLDAAKKGCLARVQKLCSGENVNCR
DSQGRNSTPLHLAAGYNNLEVAEYLLEHGADVNAQDKGGLIPLHNAASYGHVDIAALLIKYSACVNATDKWAFTPLHEAAQKG
RTQLCALLLAHGADPSMKNQEGQTPLDLATADDVRALLIDAMPPEALPVTHKPPSIPTIAATSAAATAALALGPTPALASLAP
SPVPSGASASGAGGVPLLASPSSTPASLSAASSMDSLTGACGVAPSPVPAGAIGAGAAGDGTLLGACGVSAADGATGPADKPD
RSADFEIPGLDMNINMFLKGLGLDHLRDIFEKEQITLDVLADMGHEELKEIGINAYGHRHKLIKGVERLLGAQQGGLAYPGAN
PYLSYHTTAQGTLLIDLPPDDKEYQSVEDEMQSTIREHKDGGQAGGVFNRYNVIKIQKVRNKKLWERYTHRRKEVTEENHNHH
NERMLFHGSPFINAIINKGFDERHAYIGGMFGAGIYFAENSSKSNQYVYGIGGGTGCPVHKDRSCYICQRQMLFCRVTLGKSF
LQFSAMKMAHSPPGHHSVIGRPSVNGLAFAEYVIYRGEQAYPEYLITYQILKPECTPDLTSAAGQKS

>TNKS[B.belcheri][XP_019641281.1]
MAGRRAMLSSDRIHCDESSGCRELFEACRNGDVARVKKLITPQNVNARDTAGRKSTPLHFAAGFGRKDVVEHLLQNGANVHAR
DDGGLIPLHNACSFGHAEVVTLLLRNGADPNARDNWNYTPLHEAAIKGKIDVCIVLLQNGADPSIRNTDGKTALDLAEPSAKT
VLTGDYKKDELLEAARSGNEDKLMALLTPLNVNCHASDGRKSTPLHLAAGYNRVRIVQLILQHGADVHAKDKGGLVPLHNACS
YGHFEVTELLLKHGASVNAMDLWQFTPLHEAASKSRIEVCSLLLSHGADPTLLNCHSKSAIDVASTPELQEKLSYEFKGHCLL
DGARQTDMAKVKKHLQLDIVNFKHPYTHDTALHCAALSPYPKRRQITELLIRKGANLNDKNKEFLTPLHVASDKCYIDVMEVL
LKHGAKVNALDSLGQTALHRGAHCGHVQACRLLLSFGVDPSIVSLQGVTAAQMATEAVQQMLHEDPPSGPADECDQLLEAAKA
GDLEAVKVLCNPHTVNCRDVNGRHSTPLHFAAGYNRVAVVEYLLQHGADVHAKDKGGLVPLHNACSYGHYEVCELLLKHGAVV
NVADLWKFTPLHEAAAKGKYEICKLLLKHGADPNKKNRDGNTPLDLVRDGDTDIQDLLRGDAALLDAAKKGNLARVQKLATPE
NINCRDTQGRNSTPLHLAAGYNNVEVAEFLLENGADVNAQDKGGLIPLHNASSYGHVEIAQLLIKYGTCVNATDRWNFTPLHE
AAQKGRTQLCALLLAHGADPTMKNQEGQTPLDLATAEDVRALLVDAMPPQSLPAAITAATVKAASPISSPSTTPSSTPGVLST
ASSMDHLVGAVGGAGQGDGAIDRSAVEGESLLDMTISSFLQQLGLNQLLDIFNKEQITLDILGEMGHEELKEIGVNAYGHRHK
LIKGVERILGGTGTTLNPYLTPALGSHGTILTDLSPDDKEYQSVEEEVQSTIRQHKDQGQAGGIFNRYNVIKIQKVRNKRLWD
RYVHRRKEVSEENHNQSNERMLFHGSPFINAIINKGFDERHAYIGGMFGAGIYFAENSSKSNQYVYGIGGGTGCPIHKDRSCY
ICQRQMLFCRVTLGKAFLQFSAMKMAHAPPGHHSVIGRPSVGGLNFAEYVIYRGEQAFPEYLITYQIVKPEAPAAPSPDQK

>TNKS[D.melanogaster][NP_651410.1]
MANSSRSRAILSVNLDAVMANDPLRELFEACKTGEIAKVKKLITPQTVNARDTAGRKSTPLHFAAGYGRREVVEFLLNSGASI
QACDEGGLHPLHNCCSFGHAEVVRLLLKAGASPNTTDNWNYTPLHEAASKGKVDVCLALLQHGANHTIRNSEQKTPLELADEA
TRPVLTGEYRKDELLEAARSGAEDRLLALLTPLNVNCHASDGRRSTPLHLAAGYNRIGIVEILLANGADVHAKDKGGLVPLHN
ACSYGHFDVTKLLIQAGANVNANDLWAFTPLHEAASKSRVEVCSLLLSRGADPTLLNCHSKSAIDAAPTRELRERIAFEYKGH
CLLDACRKCDVSRAKKLVCAEIVNFVHPYTGDTPLHLAVVSPDGKRKQLMELLTRKGSLLNEKNKAFLTPLHLAAELLHYDAM
EVLLKQGAKVNALDSLGQTPLHRCARDEQAVRLLLSYAADTNIVSLEGLTAAQLASDSVLKLLKNPPDSETHLLEAAKAGDLD
TVRRIVLNNPISVNCRDLDGRHSTPLHFAAGFNRVPVVQFLLEHGAEVYAADKGGLVPLHNACSYGHYEVTELLVKHGANVNV
SDLWKFTPLHEAAAKGKYDICKLLLKHGADPMKKNRDGATPADLVKESDHDVAELLRGPSALLDAAKKGNLARVQRLVTPESI
NCRDAQGRNSTPLHLAAGYNNFECAEYLLENGADVNAQDKGGLIPLHNASSYGHLDIAALLIKHKTVVNATDKWGFTPLHEAA
QKGRTQLCSLLLAHGADAYMKNQEGQTPIELATADDVKCLLQDAMATSLSQQALSASTQSLTSSSPAPDATAAAAPGTSSSSS
SAILSPTTETVLLPTGASMILSVPVPLPLSSSTRISPAQGAEANGAEGSSSDDLLPDADTITNVSGFLSSQQLHHLIELFERE
QITLDILAEMGHDDLKQVGVSAYGFRHKILKGIAQLRSTTGIGNNVNLCTLLVDLLPDDKEFVAVEEEMQATIREHRDNGQAG
GYFTRYNIIRVQKVQNRKLWERYAHRRQEIAEENFLQSNERMLFHGSPFINAIVQRGFDERHAYIGGMFGAGIYFAEHSSKSN
QYVYGIGGGIGCPSHKDKSCYVCPRQLLLCRVALGKSFLQYSAMKMAHAPPGHHSVVGRPSAGGLHFAEYVVYRGEQSYPEYL
ITYQIVKPDDSSSGTEDTR

>TNKS[N.vectensis][XP_032220531.1]
MPCQAKMASKKLASNPTFLSDPDVCEGGRELFEACRNGDVSKVRKLVTNNSNVNVRDTAGRKSSPLHFAAGFGRKEVVEYLLQ
CGADVHAMDDGGLIPLHNACSFGHAEVVRILLSHGADANARDNWNYTPLHEAAVKGKVDVCVVLLQHGADPNIRNTDGKTALD
VAEAAAKLVLTGDYKKDELLEGARSGNEEKLMSLLTPLNVNCHASDGRKSTPLHLAAGYNRVGVVQLLLKHGADVHAKDKGGL
VPLHNACSYGHFEVTELLIKHGASVNAMDLWQFTPLHEAASKARVEVCALLLGHGADPTLLNCHSKSAIDAAPTKELQEKLLS
EFKGHQILEAAKGCDTAKIKKLITTETINFQHPLTLDTPLHVAVASSSPKKKGVIETLLKKGADPNLQNKSSYAPLHVAAEKG
LFEAMELLLKHGAKVNAIDSAGQTALHVAAIAGQVQACRVLMSNGADPTCQTFQGYTAFEVAPEPVQKVLHVNEESPSPVSDA
EKQLLEAAKTGDLETVKNLCTAQTVNCRDLDGRCSTPLHFAAGYNRVRVVEYLLLNGADVHAKDKGGLVPLHNACSYGHYEVA
ELLVKHGAIVNVADLWKFTPLHEAAAKGKYEICQLLLKHGADPQKKNRDGYTPLDLVKEGDSDVADLLRGDAALLDAAKKGNL
IRITKLATPENINCRDTQGRNSTPLHLAAGYNHLEVAEYLLEHGADVNAQDKGGLIPLHNASSYGHVDIAALLIRYNTDVNAT

DRWLFTPLHEAAQKGRTQLCALLLAHGAEPSMKNQEGQSPVDLATAEDVKCLLGDAMLSTQTTSVSTAPVAKVPAPVTATAGA
ATPATGPISGMAPLSQANSSLLANSPAGHGDGAFSNRSVSEAQGEAVRSISAVHPGLDVDVGQFLDGLQLNNLKEIFEREQIS
WDVLVDMGHEELKEIGIHAYGHRHKILKAVKEKISGMGLGLGPFSTSQVQGSVIQELSMVDKDFASVADEMQSTIVEHRDSGT
AGGVFTSYTILKIERVVNTKLWEKYVYRRREIADSNHNHANERMLFHGSPFINAIVQKGFDQRHAYIGGMFGAGIYFAENSSK
SNQYVYGIGGGSGCPTHKDRSCYSCERQLLLCRVALGKPFYQFSAVKMAHAPPGHNSVIGRPSSGGLSFAEYVIYRGEQAYPE
YLITYKINKPDT

>TNKS[A.queenslandica][XP_019848937.1]
MDTRDHQAKIPGDSRQTSQSIEEGESADKNTGDLARLFEACKNGDIDTVQNLIQQRQSSANERDLHGRKSTPLHFAAGFGRRD
IVKFLIEKGAHVDFRDEGGLIPLHNSCSFGHVDVVQLLLSNGANPNAQDNWKFSPLHEAAIKGKADVCIVLLQHGADSSLLNT
DRKAPIDLANGQAREVLLGTYRQDELLEAAKVGDEQLLMQILTPLNVNCHASDGRRSTPLHLAAGYNRTSIVQLLLKQGADVH
AKDKGGLVPLHNACSYGHYEVAELLLKYGASINVTDLWQFTPLQEAASKGRSDVCSLLLAHGANPSIANCHGKTAFNLAPSEE
FRKKLDSEYRGYQLLAAAEDGGIILLKKLLSSQLLKFQHHQTLDTLLHKAVLSKSSNRQSVIDALLKRGINLNIGNKENMTPL
ICAAKKGILEVVEQLVQRGANINHQDINGMTSLHWAVQNEHAQICRYLLSSGANPSIVNNQGQTIYQLKTSDTIQLILKNEPP
VSQFEIEQQLLEAARNSDLEILKKICTPQNVNCRDTKGRMSTPLHFAAGYNRVTVVEFLLENGADVHAKDKGGLVPLHNACSY
GHYEVAELLVKYKANVNAMDLWKFTPLHEAAAKEKYDICKLLLKNGANVHSKNRDNLTPIDLVKDPKSDLADLLRGEPALLDA
AKKGEIERVKKLLTEDNVNCRDEYGRNSTPLHLAAGYNHLDVVEYLLENKADVNAKDKGGLVPLHNASSYGHVDVASLLIRYN
SVINATDRWNFTPLHEAAQKGRTQVCSLLIIHGADVYLKNQEGQIPLDLATADDVIALLQDAMMKDIPLTIPPAEKEAKSNIV
NKGLTAAGASLLASELVLGDGVDDKNGMVTQQRGGPAGVGDGSDKGFMSYRGGGDAPPPWHNVTVKDILTELELGHLVELFER
EQITIDILIEMNGDDLQSIGITAFGVRHRLLKRIRELVQGNNEEYPVGVTTTKPTQGTQLIELSSDDKEFIDTADLMQSTICE
HRDDGKAGGVFDSYEILKIERIVNTKVWERYKYRRKEVAESNNNCANELMLFHGSPFVPYIVHNGFDERHAYIGGMFGAGIYF
AEHSSKSNQYVYGIGGGNGCPEHKNRSCYTCLRKLLLCRVVLGKPVEQYTAVRIAHAPPGHHSVIGRPSAGGLNYPEYVIYRG
EQAYPEYIITFRIKKPSATDSMSSSSSLDMSNNT

```
TNKS[A.queenslandica][XP_019848937.1]     ------------------------------------------------------------     0
TNKS[D.melanogaster][NP_651410.1]         ------------------------------------------------------------     0
TNKS[N.vectensis][XP_032220531.1]         ------------------------------------------------------------     0
TNKS[P.marinus][XP_032806710.1]           ------------------------------------------------------------     0
TNKS[B.belcheri][XP_019641281.1]          ------------------------------------------------------------     0
TNKS1[S.salar][XP_014017013.1]            --MAVSRRSSQQQQGN-----------------------------------------LLS     17
TNKS1[X.laevis][XP_018099068.1]           --MAAPSRRSQQQ----QVPTAAAGQSSQPPSASPPLSP----SSRRA----ASCLSPPC     46
TNKS1[G.gallus][NP_989671.1]              --MAAPPRR----------SQ-HHHHHHGPPPPPGPASPPAAASPPRSPSLAPAELG-PA     46
TNKS1[H.sapiens][NP_003738.2]             --MAASRRSQHHHHHHQQQLQPAPGASAPPPPPPPPLSPGLAPGTTP-ASPTASGLAPFA     57
TNKS1[P.abelii][XP_024106876.1]           --MAASRRSQHHHHHHQQQLQPAPGASAPPPPPPPPLSPGLAPGTTP-ASPTASGLAPFA     57
TNKS1[M.musculus][EDL35446.1]             GKMAASRRSQHHHHHHQQQLQPAPGASAPPPPPPPPLSPGLAPGPTP-ASPTAGGLAPFA     59
TNKS2[S.salar][XP_014034742.1]            ------------------------------------------------------------     0
TNKS2[X.laevis][XP_018082988.1]           ------------------------------------------------------------     0
TNKS2[G.gallus][NP_989672.1]              ------------------------------------------------------------     0
TNKS2[M.musculus][NP_001157107.1]         ------------------------------------------------------------     0
TNKS2[H.sapiens][AAK13463.1]              ------------------------------------------------------------     0
TNKS2[P.abelii][XP_024109627.1]           ------------------------------------------------------------     0

TNKS[A.queenslandica][XP_019848937.1]     ------------------------------------------------------------     0
TNKS[D.melanogaster][NP_651410.1]         ------------------------------------------------------------     0
TNKS[N.vectensis][XP_032220531.1]         ------------------------------------------------------------     0
TNKS[P.marinus][XP_032806710.1]           ----------------MAARRAHLASPSSSSSLSPSSGSLDTTTAAAA--------AAAA     36
TNKS[B.belcheri][XP_019641281.1]          ------------------------------------------------------------     0
TNKS1[S.salar][XP_014017013.1]            PPRNGSLSVSPPGSPSLNL---------VTATLVPPDGERGCSTGMEN-PL-ASPDLQTS     66
TNKS1[X.laevis][XP_018099068.1]           SPETNALSLSREGPDSEPEGEAASQPGITPRPLSPPPGEAANI-----------------     89
TNKS1[G.gallus][NP_989671.1]              AQRHS---------LAGPEGEA---PPDAERPPAPE------------------------     70
TNKS1[H.sapiens][NP_003738.2]             SPRHG---------LALPEGDGSRDPPDRPRSPDPVDGTSCCSTTSTICTVAAAPVVPAV     108
TNKS1[P.abelii][XP_024106876.1]           SPRHG---------LALPEGDGSRDPPDRPRSPDPVDGTSCCSTTSTICTVTAAPVVPAV     108
TNKS1[M.musculus][EDL35446.1]             SPRHG---------LALPEGDGSRDPPDRPRSPDPVDGAVCTVA--------APAAVPAA     102
TNKS2[S.salar][XP_014034742.1]            ------------------------------------------------------------     0
TNKS2[X.laevis][XP_018082988.1]           ------------------------------------------------------------     0
TNKS2[G.gallus][NP_989672.1]              ------------------------------------------------------------     0
TNKS2[M.musculus][NP_001157107.1]         ------------------------------------------------------------     0
TNKS2[H.sapiens][AAK13463.1]              ------------------------------------------------------------     0
TNKS2[P.abelii][XP_024109627.1]           ------------------------------------------------------------     0

TNKS[A.queenslandica][XP_019848937.1]     ------------------------------MDTRDHQAKIPGDSR--------------Q     16
TNKS[D.melanogaster][NP_651410.1]         --------------------------------------------------MANS----SR     6
TNKS[N.vectensis][XP_032220531.1]         --------------------------------------------------MPCQAKMASK     10
TNKS[P.marinus][XP_032806710.1]           TTTAAAAAA----------TTTAAAMTTTAAVSATSSPST------TAAAAAAVAAAAAT     80
TNKS[B.belcheri][XP_019641281.1]          --------------------------------------------------MAGRRAMLSS     10
TNKS1[S.salar][XP_014017013.1]            ALSSGSSTSP-----------------------------------TTTTSGGGSSSVSSP     91
TNKS1[X.laevis][XP_018099068.1]           -LSPTSSLPPAPAPSSGSSSSSS---------SPSSNSLCTGGSPAESPESGVIGGVSSV     139
TNKS1[G.gallus][NP_989671.1]              ---CSEGAAPGPPPGSGSSSGSSASSSSSS---SSTSSSVASSPAAESPE----------     114
TNKS1[H.sapiens][NP_003738.2]             STSSAAGVAPNPAGS---GSNNSPSSSSSPTSSSSSSPSSPGSSLAESPEAAGVSSTAPL     165
TNKS1[P.abelii][XP_024106876.1]           STSSAVGVAPNPAGS---GSNNSPSSSSSPTSSSSSSPSSPGSSLAESPEAAGVSSTAPL     165
TNKS1[M.musculus][EDL35446.1]             --SAAVGVAPTPAGGGGGGGNNSASSASSPTSSSSSSPSSPGSSLAESPEAAGVGSTATL     160
TNKS2[S.salar][XP_014034742.1]            -------MA-SVARFWGSANTNSKLYI----------------YENTTPLMSTRQCSRVL     36
TNKS2[X.laevis][XP_018082988.1]           --------------------------------------------------MSGRRCAGAS     10
TNKS2[G.gallus][NP_989672.1]              --------------------------------------------------MAARRCAGGA     10
TNKS2[M.musculus][NP_001157107.1]         --------------------------------------------------MSGRRCAGGG     10
TNKS2[H.sapiens][AAK13463.1]              --------------------------------------------------MSGRRCAGGG     10
TNKS2[P.abelii][XP_024109627.1]           --------------------------------------------------MSGRRCAGGG     10

TNKS[A.queenslandica][XP_019848937.1]     TSQSIEEGESADKNTGDLARLFEACKNGDIDTVQNLIQQRQSSANERDLHGRKSTPLHFA     76
TNKS[D.melanogaster][NP_651410.1]         SRAILSVNLDAVMANDPLRELFEACKTGEIAKVKKLIT-P-QTVNARDTAGRKSTPLHFA     64
TNKS[N.vectensis][XP_032220531.1]         KLASNPTFLSDPDVCEGGRELFEACRNGDVSKVRKLVTNN-SNVNVRDTAGRKSSPLHFA     69
TNKS[P.marinus][XP_032806710.1]           GSGPGSGQDSSADGGDAGKELFEACRNGDVGRVKRLVS-P-LNVNARDTTGRKSTPLHFA     138
TNKS[B.belcheri][XP_019641281.1]          -------DRIHCDESSGCRELFEACRNGDVARVKKLIT-P-QNVNARDTAGRKSTPLHFA     61
TNKS1[S.salar][XP_014017013.1]            GSGGTTPGDG-SGIGGAFRELFEACRNGDVSRVKRLVD-S-VNVNAKDMAGRKSTPLHFA     148
TNKS1[X.laevis][XP_018099068.1]           GI---GPGDPLSAVSGAFRELFEACRNGDVSRVRRLLE-P-ANVNAKDMAGRKSTPLHFA     194
TNKS1[G.gallus][NP_989671.1]              ----------AAGPSGAFRELLEACRNGDVTRVKRLVD-A-GNVNAKDMAGRKSTPLHFA     162
TNKS1[H.sapiens][NP_003738.2]             GPGAAGPGTGVPAVSGALRELLEACRNGDVSRVKRLVD-A-ANVNAKDMAGRKSSPLHFA     223
TNKS1[P.abelii][XP_024106876.1]           GPGAAGPGTGVPAVSGALRELLEACRNGDVSRVKRLVD-A-ANVNAKDMAGRKSSPLHFA     223
TNKS1[M.musculus][EDL35446.1]             GAGAAGLGPGVPAVSGALRELLEACRNGDVSRVKRLVD-A-ANVNAKDMAGRKSSPLHFA     218
TNKS2[S.salar][XP_014034742.1]            GLGGVFPGVDTPRSGEPNREIFEACRNGDVERVRRLVR-P-ENVNSRDTAGRKSTPLHFA     94
TNKS2[X.laevis][XP_018082988.1]           GSAASHGN--IGAGGEPARELFEACRNGDVERVKRLVN-S-DNVNSRDTAGRKSTPLHFA     66
TNKS2[G.gallus][NP_989672.1]              AALAEAPG--CGSAVEPARELFEACRNGDVERVKRLVR-P-ENVNSRDTAGRKSSPLHFA     66
TNKS2[M.musculus][NP_001157107.1]         AACAS-AG--AEAVEPSARELFEACRNGDVERVKRLVT-P-EKVNSRDTAGRKSTPLHFA     65
TNKS2[H.sapiens][AAK13463.1]              AACAS-AA--AEAVEPAARELFEACRNGDVERVKRLVT-P-EKVNSRDTAGRKSTPLHFA     65
TNKS2[P.abelii][XP_024109627.1]           AACAS-AA--AEAMEPAARELFEACRNGDVERVKRLVT-P-EKVNSRDTAGRKSTPLHFA     65
                                                             .::***:.*::  *:.*:     ..* :*  ****:*****

TNKS[A.queenslandica][XP_019848937.1]     AGFGRRDIVKFLIEKGAHVDFRDEGGLIPLHNSCSFGHVDVVQLLLSNGANPNAQDNWKF     136
TNKS[D.melanogaster][NP_651410.1]         AGYGRREVVEFLLNSGASIQACDEGGLHPLHNCCSFGHAEVVRLLLKAGASPNTTDNWNY     124
TNKS[N.vectensis][XP_032220531.1]         AGFGRKEVVEYLLQCGADVHAMDDGGLIPLHNACSFGHAEVVRILLSHGADANARDNWNY     129
TNKS[P.marinus][XP_032806710.1]           AGFGRKDVVEHLLQNGANVHARDDGGLIPLHNACSFGHAEVVNLLLRQGADPNARDNWNY     198
TNKS[B.belcheri][XP_019641281.1]          AGFGRKDVVEHLLQNGANVHARDDGGLIPLHNACSFGHAEVVTLLLRNGADPNARDNWNY     121
TNKS1[S.salar][XP_014017013.1]            AGFGRKDVVDHLLQTGANVHARDDGGLIPLHNACSFGHAEVVSLLLCQGADPNARDNWNY     208
TNKS1[X.laevis][XP_018099068.1]           AGFGRKDVVEHLLQTGANVHARDDGGLIPLHNACSFGHAEVVTLLLCQGADPNARDNWNY     254
TNKS1[G.gallus][NP_989671.1]              AGFGRKDVVEHLLQTGANVHARDDGGLIPLHNACSFGHAEVVSLLLCQGADPNARDNWNY     222
TNKS1[H.sapiens][NP_003738.2]             AGFGRKDVVEHLLQMGANVHARDDGGLIPLHNACSFGHAEVVSLLLCQGADPNARDNWNY     283
TNKS1[P.abelii][XP_024106876.1]           AGFGRKDVVEHLLQMGANVHARDDGGLIPLHNACSFGHAEVVSLLLCQGADPNARDNWNY     283
TNKS1[M.musculus][EDL35446.1]             AGFGRKDVVEHLLQMGANVHARDDGGLIPLHNACSFGHAEVVSLLLCQGADPNARDNWNY     278
TNKS2[S.salar][XP_014034742.1]            AGFGRRDVVDYLLQNGANVHARDEGGLVSLHNACSFGHSEVVNLLLRHGADANSRDNWSY     154
TNKS2[X.laevis][XP_018082988.1]           AGFGRKDVVEHLLQSGANVHARDDGGLIPIHNACSFGHAEVVSLLLRHGADPNARDNWNY     126
TNKS2[G.gallus][NP_989672.1]              AGFGRKDVVEYLLQSGANVHARDDGGLIPLHNACSFGHAEVVNLLLRHGANPNARDNWNY     126
TNKS2[M.musculus][NP_001157107.1]         AGFGRKDVVEYLLQNGANVQARDDGGLIPLHNACSFGHAEVVNLLLQHGADPNARDNWNY     125
TNKS2[H.sapiens][AAK13463.1]              AGFGRKDVVEYLLQNGANVQARDDGGLIPLHNACSFGHAEVVNLLLRHGADPNARDNWNY     125
TNKS2[P.abelii][XP_024109627.1]           AGFGRKDVVEYLLQNGANVQARDDGGLIPLHNACSFGHAEVVNLLLRHGADPNARDNWNY     125
                                          **:**:::*..*:: ** :.  *:***  :**.***** :** :**  **. *: ***.:

TNKS[A.queenslandica][XP_019848937.1]     SPLHEAAIKGKADVCIVLLQHGADSSLLNTDRKAPIDLANGQAREVLLGTYRQDELLEAA     196
TNKS[D.melanogaster][NP_651410.1]         TPLHEAASKGKVDVCLALLQHGANHTIRNSEQKTPLELADEATRPVLTGEYRKDELLEAA     184
TNKS[N.vectensis][XP_032220531.1]         TPLHEAAVKGKVDVCVVLLQHGADPNIRNTDGKTALDVAEAAAKLVLTGDYKKDELLEGA     189
TNKS[P.marinus][XP_032806710.1]           TPLHEAAIKGKIDVCIVLLQHGADPGIRNTDGKTALDLAEPTAKAVLTGEYKKDELLEAA     258
TNKS[B.belcheri][XP_019641281.1]          TPLHEAAIKGKIDVCIVLLQNGADPSIRNTDGKTALDLAEPSAKTVLTGDYKKDELLEAA     181
TNKS1[S.salar][XP_014017013.1]            TPLHEAAIKGKIDVCIVLLQHGADPNIRNTDGKSALDLADPSAKAVLTGEYKKDELLEAA     268
TNKS1[X.laevis][XP_018099068.1]           TPLHEASIKGKIDVCIVLLQHGADPNIRNTDGKSALDLADPSSKTVLTGEYKKDELLEAA     314
```

```
TNKS1[G.gallus][NP_989671.1]             TPLHEAAIKGKIDVCIVLLQHGADPNIRNTDGKSALDLADPSAEAVLTGEYKKDELLEAA   282
TNKS1[H.sapiens][NP_003738.2]            TPLHEAAIKGKIDVCIVLLQHGADPNIRNTDGKSALDLADPSAKAVLTGEYKKDELLEAA   343
TNKS1[P.abelii][XP_024106876.1]          TPLHEAAIKGKIDVCIVLLQHGADPNIRNTDGKSALDLADPSAKAVLTGEYKKDELLEAA   343
TNKS1[M.musculus][EDL35446.1]            TPLHEAAIKGKIDVCIVLLQHGADPNIRNTDGKSALDLADPSAKAVLTGEYKKDELLEAA   338
TNKS2[S.salar][XP_014034742.1]           TPLHEAAIKGKSEVCIVLLQHGAEPTIRNTDGRTALDLAEASTKAVLTGEYRKDDLLESA   214
TNKS2[X.laevis][XP_018082988.1]          TPLHEAAIKGKIDVCIVLFQHGADPTIRNTDSRTALDLSDPSAKAVLTGEYKKDELLESA   186
TNKS2[G.gallus][NP_989672.1]             TPLHEAAIKGKTDVCIVLLQHGAEPTIRNTDGRTALDLADPSAKAVLTGEYKKDELLESA   186
TNKS2[M.musculus][NP_001157107.1]        TPLHEAAIKGKIDVCIVLLQHGAEPTIRNTDGRTALDLADPSAKAVLTGDYKKDELLESA   185
TNKS2[H.sapiens][AAK13463.1]             TPLHEAAIKGKIDVCIVLLQHGAEPTIRNTDGRTALDLADPSAKAVLTGEYKKDELLESA   185
TNKS2[P.abelii][XP_024109627.1]          TPLHEAAIKGKIDVCIVLLQHGAEPTIRNTDGRTALDLADPSAKAVLTGEYKKDELLESA   185
                                         :*****: *** :**:.*:*:**:  : *:: :: :::::  :. ** * *::*:***.*

TNKS[A.queenslandica][XP_019848937.1]    KVGDEQLLMQILTPLNVNCHASDGRRSTPLHLAAGYNRTSIVQLLLKQGADVHAKDKGGL   256
TNKS[D.melanogaster][NP_651410.1]        RSGAEDRLLALLTPLNVNCHASDGRRSTPLHLAAGYNRIGIVEILLANGADVHAKDKGGL   244
TNKS[N.vectensis][XP_032220531.1]        RSGNEEKLMSLLTPLNVNCHASDGRKSTPLHLAAGYNRVGVVQLLLKHGADVHAKDKGGL   249
TNKS[P.marinus][XP_032806710.1]          RSGNEEKLMALLTPLNVNCHASDGRKSTPLHLAAGYNRVRIVQLLLQHGADVHAKDKGGL   318
TNKS[B.belcheri][XP_019641281.1]         RSGNEDKLMALLTPLNVNCHASDGRKSTPLHLAAGYNRVRIVQLILQHGADVHAKDKGGL   241
TNKS1[S.salar][XP_014017013.1]           RSGNEEKLMALLTPLNVNCHASDGRKSTPLHLAAGYNRVRIVQLLLQHGADVHAKDKGGL   328
TNKS1[X.laevis][XP_018099068.1]          RSGNEEKLMALLTPLNVNCHASDGRKSTPLHLAAGYNRVQIVQLLLQHGADVHAKDKGGL   374
TNKS1[G.gallus][NP_989671.1]             RSGNEEKLMALLTPLNVNCHASDGRKSTPLHLAAGYNRVRIVQLLLQHGADVHAKDKGGL   342
TNKS1[H.sapiens][NP_003738.2]            RSGNEEKLMALLTPLNVNCHASDGRKSTPLHLAAGYNRVRIVQLLLQHGADVHAKDKGGL   403
TNKS1[P.abelii][XP_024106876.1]          RSGNEEKLMALLTPLNVNCHASDGRKSTPLHLAAGYNRVRIVQLLLQHGADVHAKDKGGL   403
TNKS1[M.musculus][EDL35446.1]            RSGNEEKLMALLTPLNVNCHASDGRKSTPLHLAAGYNRVRIVQLLLQHGADVHAKDKGGL   398
TNKS2[S.salar][XP_014034742.1]           RSGNEDKLMALLTPLNVNCHASDGRKSSPLHLAAGYNRVKTVQLLLKHGADVHAKDKGDL   274
TNKS2[X.laevis][XP_018082988.1]          RSGNEEKMMALLTPLNVNCHASDGRKSTPLHLAAGYNRVKIVQLLLQHGADVHAKDKGDL   246
TNKS2[G.gallus][NP_989672.1]             RSGNEEKMMSLLTPLNVNCHASDGRKSTPLHLAAGYNRVKIVQLLLQHGADVHAKDKGDL   246
TNKS2[M.musculus][NP_001157107.1]        RSGNEEKMMALLTPLNVNCHASDGRKSTPLHLAAGYNRVKIVQLLLHHGADVHAKDKGDL   245
TNKS2[H.sapiens][AAK13463.1]             RSGNEEKMMALLTPLNVNCHASDGRKSTPLHLAAGYNRVKIVQLLLQHGADVHAKDKGDL   245
TNKS2[P.abelii][XP_024109627.1]          RSGNEEKMMALLTPLNVNCHASDGRKSTPLHLAAGYNRVKIVQLLLQHGADVHAKDKGDL   245
                                         : * *: :: :*************:*:**********   *:::* :**********.*

TNKS[A.queenslandica][XP_019848937.1]    VPLHNACSYGHYEVAELLLKYGASINVTDLWQFTPLQEAASKGRSDVCSLLLAHGANPSI   316
TNKS[D.melanogaster][NP_651410.1]        VPLHNACSYGHFDVTKLLIQAGANVNANDLWAFTPLHEAASKSRVEVCSLLLSRGADPTL   304
TNKS[N.vectensis][XP_032220531.1]        VPLHNACSYGHFEVTELLIKHGASVNAMDLWQFTPLHEAASKARVEVCALLLGHGADPTL   309
TNKS[P.marinus][XP_032806710.1]          VPLHNACSYGHYEVTELLVKHGASVNAMDLWQFTPLHEAASKNRVEVCSLLLSYGADPTL   378
TNKS[B.belcheri][XP_019641281.1]         VPLHNACSYGHFEVTELLLKHGASVNAMDLWQFTPLHEAASKSRIEVCSLLLSHGADPTL   301
TNKS1[S.salar][XP_014017013.1]           VPLHNACSYGHFEVTELLLKHGACVNAMDLWQFTPLHEAASKNRVEVCSLLLSHGADPTL   388
TNKS1[X.laevis][XP_018099068.1]          VPLHNACSYGHFEVTESLLKHGACVNAMDLWQFTPLHEAASKNRVEVCSLLLSHGADPTL   434
TNKS1[G.gallus][NP_989671.1]             VPLHNACSYGHYEVTELLLKHGACVNAMDLWQFTPLHEAASKNRVEVCSLLLSHGADPTL   402
TNKS1[H.sapiens][NP_003738.2]            VPLHNACSYGHYEVTELLLKHGACVNAMDLWQFTPLHEAASKNRVEVCSLLLSHGADPTL   463
TNKS1[P.abelii][XP_024106876.1]          VPLHNACSYGHYEVTELLLKHGACVNAMDLWQFTPLHEAASKNRVEVCSLLLSHGADPTL   463
TNKS1[M.musculus][EDL35446.1]            VPLHNACSYGHYEVTELLLKHGACVNAMDLWQFTPLHEAASKNRVEVCSLLLSHGADPTL   458
TNKS2[S.salar][XP_014034742.1]           VPLHNACSYGHYEVTDILVKQGACVNAMDLWQFTPLHEAASKNRVDVCSLLVSYGADPTF   334
TNKS2[X.laevis][XP_018082988.1]          VPLHNACSYGHYEVTELLVKHGASVNAMDLWQFTSLHEAASKNRVEVCSLLLSYGADPTM   306
TNKS2[G.gallus][NP_989672.1]             VPLHNACSYGHYEVTELLVKHGACVNAMDLWQFTPLHEAASKNRVEVCSLLLSYGADPTL   306
TNKS2[M.musculus][NP_001157107.1]        VPLHNACSYGHYEVTELLVKHGACVNAMDLWQFTPLHEAASKNRIEVCSLLLSYGADPTL   305
TNKS2[H.sapiens][AAK13463.1]             VPLHNACSYGHYEVTELLVKHGACVNAMDLWQFTPLHEAASKNRVEVCSLLLSYGADPTL   305
TNKS2[P.abelii][XP_024109627.1]          VPLHNACSYGHYEVTELLVKHGACVNAMDLWQFTPLHEAASKNRVEVCSLLLSYGADPTL   305
                                         ***********::*:. *:: ** :*. *** ** *:***** * :**:**:. **:*::

TNKS[A.queenslandica][XP_019848937.1]    ANCHGKTAFNLAPSEEFRKKLDSEYRGYQLLAAAEDGGIILLKKLLSSQLLKFQHHQTLD   376
TNKS[D.melanogaster][NP_651410.1]        LNCHSKSAIDAAPTRELRERIAFEYKGHCLLDACRKCDVSRAKKLVCAEIVNFVHPYTGD   364
TNKS[N.vectensis][XP_032220531.1]        LNCHSKSAIDAAPTKELQEKLLSEFKGHQILEAAKGCDTAKIKKLITTETINFQHPLTLD   369
TNKS[P.marinus][XP_032806710.1]          LNCHSKSAIDLAPTSELKERLVYEFRGHSLLQAAREADVTRVKKHLGLDIINFKHPQTHE   438
TNKS[B.belcheri][XP_019641281.1]         LNCHSKSAIDVASTPELQEKLSYEFKGHCLLDGARQTDMAKVKKHLQLDIVNFKHPYTHD   361
TNKS1[S.salar][XP_014017013.1]           LNCHSKSAVDMAPTPELKERLTYEFKGHSLLQAAREADMAKVKKTLALEIISFKHPQTNE   448
TNKS1[X.laevis][XP_018099068.1]          VNCHGKSAVDMAPTPELKERLSYEFKGHSLLQAAREADLAKVKKTLALEIINFKQPQSHE   494
TNKS1[G.gallus][NP_989671.1]             VNCHGKSAVDMAPTPELRERLTYEFKGHSLLQAAREADLAKVKKTLALEIINFKQPQSHE   462
TNKS1[H.sapiens][NP_003738.2]            VNCHGKSAVDMAPTPELRERLTYEFKGHSLLQAAREADLAKVKKTLALEIINFKQPQSHE   523
TNKS1[P.abelii][XP_024106876.1]          VNCHGKSAVDMAPTPELRERLTYEFKGHSLLQAAREADLAKVKKTLALEIINFKQPQSHE   523
TNKS1[M.musculus][EDL35446.1]            VNCHGKSAVDMAPTPELRERLTYEFKGHSLLQAAREADLAKVKKTLALEIINFKQPQSHE   518
TNKS2[S.salar][XP_014034742.1]           LNCHNQSAIDLSPTPQLKERLAYEFRGHALLQAAREADLPRLKKHLSLETITFKHPLTHE   394
TNKS2[X.laevis][XP_018082988.1]          LNCHNKSAIDLAPTPQLKERLSYEFKGHSLLQAAREADLTRIKKHLSLEIVNFKHPQTHD   366
TNKS2[G.gallus][NP_989672.1]             LNCHNKSTIDLAPTPQLKERLAYEFKGHSLLQAARESDVARIKKHLSLETVNFKHPQTHE   366
TNKS2[M.musculus][NP_001157107.1]        LNCHNKSAIDLAPTAQLKERLSYEFKGHSLLQAAREADVTRIKKHLSLEMVNFKHPQTHE   365
TNKS2[H.sapiens][AAK13463.1]             LNCHNKSAIDLAPTPQLKERLAYEFKGHSLLQAAREADVTRIKKHLSLEMVNFKHPQTHE   365
TNKS2[P.abelii][XP_024109627.1]          LNCHNKSAIDLAPTPQLKERLAYEFKGHSLLQAAREADVTRIKKHLSLEMVNFKHPQTHE   365
                                          ***.:::.: : : ::::::  *::*: :* ...  .    ** :  : :.* :  : :

TNKS[A.queenslandica][XP_019848937.1]    TLLHKAVLSKSSNRQSVIDALLKRGINLNIGNKENMTPLICAAKKGILEVVEQLVQRGAN   436
TNKS[D.melanogaster][NP_651410.1]        TPLHLAVVSPDGKRKQLMELLTRKGSLLNEKNKAFLTPLHLAAELLHYDAMEVLLKQGAK   424
TNKS[N.vectensis][XP_032220531.1]        TPLHVAVASSSPKKKGVIETLLKKGADPNLQNKSSYAPLHVAAEKGLFEAMELLLKHGAK   429
TNKS[P.marinus][XP_032806710.1]          TALHCAVASPYPKRKQVSELLLRKGANVNEKTKDLLTPLHVASDKAHNDVIDVLLKHGAK   498
TNKS[B.belcheri][XP_019641281.1]         TALHCAALSPYPKRRQITELLIRKGANLNDKNKEFLTPLHVASDKCYIDVMEVLLKHGAK   421
TNKS1[S.salar][XP_014017013.1]           TALHCAVASPHPKRKQVTELLLRKGANINEKNKDFMTALHVASERAHNDILEVLQKHGAK   508
TNKS1[X.laevis][XP_018099068.1]          TALHCAVASLHPKRKQITELLLRKGASVNEKNKDFMTPLHVAAERAHNDVVEVLHKHGAK   554
TNKS1[G.gallus][NP_989671.1]             TALHCAVAAVHPKRKQVTELLLRKGANVNEKNKDFMTPLHVAAEKAHNDVMEVLHKHGAK   522
TNKS1[H.sapiens][NP_003738.2]            TALHCAVASLHPKRKQVTELLLRKGANVNEKNKDFMTPLHVAAERAHNDVMEVLHKHGAK   583
TNKS1[P.abelii][XP_024106876.1]          TALHCAVASLHPKRKQVTELLLRKGANVNEKNKDFMTPLHVAAERAHNDVMEVLHKHGAK   583
TNKS1[M.musculus][EDL35446.1]            TALHCAVASLHPKRKQVAELLLRKGANVNEKNKDFMTPLHVAAERAHNDVMEVLHKHGAK   578
TNKS2[S.salar][XP_014034742.1]           TALHCAATSPYPKRKQVCELLLRKGANVNEKTKDFLTPLHLASEKSHNDIIEVLVKHEAK   454
TNKS2[X.laevis][XP_018082988.1]          TALHCAASSPYPKRKQVCELLFRKGANVNEKTKDFLTPLHIASDKAHNDIVELLIKHEAK   426
TNKS2[G.gallus][NP_989672.1]             TALHCAAASPYPKRKQVCELLLRKGANINEKTKDFLTPLHVASEKAHNDVVEVVVKHEAK   426
TNKS2[M.musculus][NP_001157107.1]        TALHCAAASPYPKRKQICELLLRKGANTNEKTKEFLTPLHVASENAHNDVVEVVVKHEAK   425
TNKS2[H.sapiens][AAK13463.1]             TALHCAAASPYPKRKQICELLLRKGANINEKTKEFLTPLHVASEKAHNDVVEVVVKHEAK   425
TNKS2[P.abelii][XP_024109627.1]          TALHCAAASPYPKRKQICELLLRKGANINEKTKEFLTPLHVASEKAHNDVVEVVVKHEAK   425
                                         * ** *. :   ::: : : * ::*   *  .*   : *  *:.    : :: : :: *:

TNKS[A.queenslandica][XP_019848937.1]    INHQDINGMTSLHWAVQNEHAQICRYLLSSGANPSIVNNQGQTIYQLKTSDTIQLILKN-   495
TNKS[D.melanogaster][NP_651410.1]        VNALDSLGQTPLHRCAR--DEQAVRLLLSYAADTNIVSLEGLTAAQLASDSVLKLLKNP-   481
TNKS[N.vectensis][XP_032220531.1]        VNAIDSAGQTALHVAAIAGQVQACRVLMSNGADPTCQTFQGYTAFEVAPEPVQKVLHVNE   489
TNKS[P.marinus][XP_032806710.1]          INVTDSLGQTSLHRAAHAGHLTTCRLLLSHGCDPNLPSLQGFTAAQLGNESVQQILHES-   557
TNKS[B.belcheri][XP_019641281.1]         VNALDSLGQTALHRGAHCGHVQACRLLLSFGVDPSIVSLQGVTAAQMATEAVQQMLHED-   480
TNKS1[S.salar][XP_014017013.1]           VNAVDTLGQTALHRAALAGHIQTCRLLLSYGADPAIVSLQGFTASQMGNEAVQQILNEN-   567
TNKS1[X.laevis][XP_018099068.1]          MNALDTLGQTALHRAALGGHLQTCRLLLSFGSDASIVSLQGFTAAQMGNEAVQQILNES-   613
TNKS1[G.gallus][NP_989671.1]             MNALDTLGQTALHRAALAGHLQTCRLLLNYGSDPSIISLQGFTAAQIGNEAVQQILSES-   581
TNKS1[H.sapiens][NP_003738.2]            MNALDTLGQTALHRAALAGHLQTCRLLLSYGSDPSIISLQGFTAAQMGNEAVQQILSES-   642
TNKS1[P.abelii][XP_024106876.1]          MNALDTLGQTALHRAALAGHLQTCRLLLSYGSDPSIISLQGFTAAQMGNEAVQQILSES-   642
TNKS1[M.musculus][EDL35446.1]            MNALDSLGQTALHRAALAGHLQTCRLLLSYGSDPSIISLQGFTAAQMGNEAVQQILSES-   637
TNKS2[S.salar][XP_014034742.1]           VNALDNLGQTALHRAAHCGHLQTCRLLLKAGCDPLVMSLQGFSPSQMGNESVQEILHEG-   513
TNKS2[X.laevis][XP_018082988.1]          VNALDNLGQTSLHRAAHCGHLQTCRLLLNSGCDPSIVSLQGFTALQMGNESVQQLLQEG-   485
TNKS2[G.gallus][NP_989672.1]             VNALDNLGQTSLHRAAHCGHLQTCRLLLSSGCDPSIVSLQGFTALQMGNESVQQLLQEG-   485
```

```
TNKS2[M.musculus][NP_001157107.1]         VNALDSLGQTSLHRAAHCGHLQTCRLLLSYGCDPNIISLQGFTALQMGNENVQQLLQEG-      484
TNKS2[H.sapiens][AAK13463.1]              VNALDNLGQTSLHRAAYCGHLQTCRLLLSYGCDPNIISLQGFTALQMGNENVQQLLQEG-      484
TNKS2[P.abelii][XP_024109627.1]           VNALDNLGQTSLHRAAYCGHLQTCRLLLSYGCDPNIISLQGFTALQMGNENVQQLLQEG-      484
                                          :*  *  * * **  .   .    * *:. . :    . :* :  ::  . . :::

TNKS[A.queenslandica][XP_019848937.1]     EPPVSQFEIEQQLLEAARNSDLEILKKI--CTPQNVNCRDTKGRMSTPLHFAAGYNRVTV      553
TNKS[D.melanogaster][NP_651410.1]         ------PDSETHLLEAAKAGDLDTVRRIVLNNPISVNCRDLDGRHSTPLHFAAGFNRVPV      535
TNKS[N.vectensis][XP_032220531.1]         ESPSPVSDAEKQLLEAAKTGDLETVKNL--CTAQTVNCRDLDGRCSTPLHFAAGYNRVRV      547
TNKS[P.marinus][XP_032806710.1]           SLPVRNSDVEHQLLEAAKAGDLDTVKQL--CTTQTVNCRDMEGRHSTPLHFAAGYNRVSV      615
TNKS[B.belcheri][XP_019641281.1]          -PPSGPADECDQLLEAAKAGDLEAVKVL--CNPHTVNCRDVNGRHSTPLHFAAGYNRVAV      537
TNKS1[S.salar][XP_014017013.1]            -VPTRNSDVDYRFLEAAKAGDLDTVQQL--CTPQNVNCRDLEGRHSTPLHFAAGYNRVAV      624
TNKS1[X.laevis][XP_018099068.1]           -TPVRTSDVDYRLLEASKAGDLDIVKQL--CSSQNVNCRDLEGRHSTPLHFAAGYNRVSV      670
TNKS1[G.gallus][NP_989671.1]              -TPVRTSDVDYRLLEASKAGDLETVKQL--CSPQNVNCRDLEGRHSTPLHFAAGYNRVSV      638
TNKS1[H.sapiens][NP_003738.2]             -TPIRTSDVDYRLLEASKAGDLETVKQL--CSSQNVNCRDLEGRHSTPLHFAAGYNRVSV      699
TNKS1[P.abelii][XP_024106876.1]           -TPIRTSDVDYRLLEASKAGDLETVKQL--CSPQNVNCRDLEGRHSTPLHFAAGYNRVSV      699
TNKS1[M.musculus][EDL35446.1]             -TPMRTSDVDYRLLEASKAGDLETVKQL--CSPQNVNCRDLEGRHSTPLHFAAGYNRVSV      694
TNKS2[S.salar][XP_014034742.1]            -TLIGNSDVDWQLLEASKSGDLEIVKKL--CTMQNVNCRDVEGRQSTPLHFAAGYNRVSV      570
TNKS2[X.laevis][XP_018082988.1]           -LPFSNSDADSQLLEAAKAGDLDVVKKL--CTSQSVNCRDVEGRQSTPLHFAAGYNRVAV      542
TNKS2[G.gallus][NP_989672.1]              -IPLGNSDADRQLLEAAKAGDVDTVKKL--CTVQSVNCRDIEGRQSTPLHFAAGYNRVSV      542
TNKS2[M.musculus][NP_001157107.1]         -ASLGHSEADRQLLEAAKAGDVETVKKL--CTVQSVNCRDIEGRQSTPLHFAAGYNRVSV      541
TNKS2[H.sapiens][AAK13463.1]              -ISLGNSEADRQLLEAAKAGDVETVKKL--CTVQSVNCRDIEGRQSTPLHFAAGYNRVSV      541
TNKS2[P.abelii][XP_024109627.1]           -ISLGNSEADRQLLEAAKAGDVETVKKL--CTVQSVNCRDIEGRQSTPLHFAAGYNRVSV      541
                                                 :   ::***:: .*:: :: :   .  .***** .** *********:*** *

TNKS[A.queenslandica][XP_019848937.1]     VEFLLENGADVHAKDKGGLVPLHNACSYGHYEVAELLVKYKANVNAMDLWKFTPLHEAAA      613
TNKS[D.melanogaster][NP_651410.1]         VQFLLEHGAEVYAADKGGLVPLHNACSYGHYEVTELLVKHGANVNVSDLWKFTPLHEAAA      595
TNKS[N.vectensis][XP_032220531.1]         VEYLLLNGADVHAKDKGGLVPLHNACSYGHYEVAELLVKHGAIVNVADLWKFTPLHEAAA      607
TNKS[P.marinus][XP_032806710.1]           VEYLLQHGADVHAKDKGGLVPLHNACSYGHYEVAELLVKRGAVVNVADLWKFTPLHEAAA      675
TNKS[B.belcheri][XP_019641281.1]          VEYLLQHGADVHAKDKGGLVPLHNACSYGHYEVCELLLKHGAVVNVADLWKFTPLHEAAA      597
TNKS1[S.salar][XP_014017013.1]            VEYLLHHGADVHAKDKGGLVPLHNACSYGHYEVAELLVRHGASVNVADLWKFTPLHEAAA      684
TNKS1[X.laevis][XP_018099068.1]           VEYLLHHGADVHAKDKGGLVPLHNACSYGHYEVAELLVRHGASVNVADLWKFTPLHEAAA      730
TNKS1[G.gallus][NP_989671.1]              VEYLLHHGADVHAKDKGGLVPLHNACSYGHYEVAELLVRHGASVNVADLWKFTPLHEAAA      698
TNKS1[H.sapiens][NP_003738.2]             VEYLLHHGADVHAKDKGGLVPLHNACSYGHYEVAELLVRHGASVNVADLWKFTPLHEAAA      759
TNKS1[P.abelii][XP_024106876.1]           VEYLLHHGADVHAKDKGGLVPLHNACSYGHYEVAELLVRHGASVNVADLWKFTPLHEAAA      759
TNKS1[M.musculus][EDL35446.1]             VEYLLHHGADVHAKDKGGLVPLHNACSYGHYEVAELLVRHGASVNVADLWKFTPLHEAAA      754
TNKS2[S.salar][XP_014034742.1]            VEYLLLHGADVHAKDKGGLVPLHNACSYGHYEVAELLVIHGAVVNIADLWKFTPLHEAAA      630
TNKS2[X.laevis][XP_018082988.1]           VEYLLQHGADVHAKDKGGLVALHNACSYGHYEVTELLVKHGAVINVADLWKFTPLHEAAA      602
TNKS2[G.gallus][NP_989672.1]              VEYLLQHGADVHAKDKGGLVPLHNACSYGHYEVAELLVKHGAVVNVADLWKFTPLHEAAA      602
TNKS2[M.musculus][NP_001157107.1]         VEYLLQHGADVHAKDKGGLVPLHNACSYGHYEVAELLVKHGAVVNVADLWKFTPLHEAAA      601
TNKS2[H.sapiens][AAK13463.1]              VEYLLQHGADVHAKDKGGLVPLHNACSYGHYEVAELLVKHGAVVNVADLWKFTPLHEAAA      601
TNKS2[P.abelii][XP_024109627.1]           VEYLLQHGADVHAKDKGGLVPLHNACSYGHYEVAELLVKHGAVVNVADLWKFTPLHEAAA      601
                                          *::** :**:*:* ****** ************ ***:   * :*  *************

TNKS[A.queenslandica][XP_019848937.1]     KEKYDICKLLLKNGANVHSKNRDNLTPIDLVKDPKSDLADLLRGEPALLDAAKKGEIERV      673
TNKS[D.melanogaster][NP_651410.1]         KGKYDICKLLLKHGADPMKKNRDGATPADLVKESDHDVAELLRGPSALLDAAKKGNLARV      655
TNKS[N.vectensis][XP_032220531.1]         KGKYEICQLLLKHGADPQKKNRDGYTPLDLVKEGDSDVADLLRGDAALLDAAKKGNLIRI      667
TNKS[P.marinus][XP_032806710.1]           KGKYEICKLLLKHGADPTKKNRDGNTPLDLVKEGDTDIQDLLRGDAALLDAAKKGCLARV      735
TNKS[B.belcheri][XP_019641281.1]          KGKYEICKLLLKHGADPNKKNRDGNTPLDLVRDGDTDIQDLLRGDAALLDAAKKGNLARV      657
TNKS1[S.salar][XP_014017013.1]            KGKYEICKLLLKHGADPTKKNRDGNMPLDMVKDGDTDIQDLLRGDAALLDAAKKGCLARV      744
TNKS1[X.laevis][XP_018099068.1]           KGKYEICKLLLKHGADPTKKNRDGNTPLDLVKDGDTDIQDLLRGDAALLDAAKKGCLARV      790
TNKS1[G.gallus][NP_989671.1]              KGKYEICKLLLKHGADPTKKNRDGNTPLDLVKEGDTDIQDLLRGDAALLDAAKKGCLARV      758
TNKS1[H.sapiens][NP_003738.2]             KGKYEICKLLLKHGADPTKKNRDGNTPLDLVKEGDTDIQDLLRGDAALLDAAKKGCLARV      819
TNKS1[P.abelii][XP_024106876.1]           KGKYEICKLLLKHGADPTKKNRDGNTPLDLVKEGDTDIQDLLRGDAALLDAAKKGCLARV      819
TNKS1[M.musculus][EDL35446.1]             KGKYEICKLLLKHGADPTKKNRDGNTPLDLVKEGDTDIQDLLRGDAALLDAAKKGCLARV      814
TNKS2[S.salar][XP_014034742.1]            KGKYEICKLLLQHGADPTKKNRDGSTPLDLVKDGDTDMQDLLRGDAALLDAAKKGCLARV      690
TNKS2[X.laevis][XP_018082988.1]           KGKYEICKLLLLHGADSTKKNRDGNTPLDLVKDGDTDIQDLLRGDAALLDAAKKGCVSRV      662
TNKS2[G.gallus][NP_989672.1]              KGKYEICKLLLQHGADPPKKNRDGNTPLDLVKDGDTDIQDLLRGDAALLDAAKKGCLARV      662
TNKS2[M.musculus][NP_001157107.1]         KGKYEICKLLLQHGADPTKKNRDGNTPLDLVKDGDTDIQDLLRGDAALLDAAKKGCLARV      661
TNKS2[H.sapiens][AAK13463.1]              KGKYEICKLLLQHGADPTKKNRDGNTPLDLVKDGDTDIQDLLRGDAALLDAAKKGCLARV      661
TNKS2[P.abelii][XP_024109627.1]           KGKYEICKLLLQHGADPTKKNRDGNTPLDLVKDGDTDIQDLLRGDAALLDAAKKGCLARV      661
                                          * **:**:*** :**:  .****.  * *:*:: . *: :****  ********* : *:

TNKS[A.queenslandica][XP_019848937.1]     KKLLTEDNVNCRDEYGRNSTPLHLAAGYNHLDVVEYLLENKADVNAKDKGGLVPLHNASS      733
TNKS[D.melanogaster][NP_651410.1]         QRLVTPESINCRDAQGRNSTPLHLAAGYNNFECAEYLLENGADVNAQDKGGLIPLHNASS      715
TNKS[N.vectensis][XP_032220531.1]         TKLATPENINCRDTQGRNSTPLHLAAGYNHLEVAEYLLEHGADVNAQDKGGLIPLHNASS      727
TNKS[P.marinus][XP_032806710.1]           QKLCSGENVNCRDSQGRNSTPLHLAAGYNNLEVAEYLLEHGADVNAQDKGGLIPLHNAAS      795
TNKS[B.belcheri][XP_019641281.1]          QKLATPENINCRDTQGRNSTPLHLAAGYNNVEVAEFLLENGADVNAQDKGGLIPLHNASS      717
TNKS1[S.salar][XP_014017013.1]            QKLCSPENINCRDTQGRNSTPLHLAAGYNNLEVAEYLLEHGADVNAQDKGGLIPLHNAAS      804
TNKS1[X.laevis][XP_018099068.1]           QKLCTQENINCRDTQGRNSTPLHLAAGYNNLEVAEYLLEHGADVNAQDKGGLIPLHNAAS      850
TNKS1[G.gallus][NP_989671.1]              QKLCTPENINCRDTQGRNSTPLHLAAGYNNLEVAEYLLEHGADVNAQDKGGLIPLHNAAS      818
TNKS1[H.sapiens][NP_003738.2]             QKLCTPENINCRDTQGRNSTPLHLAAGYNNLEVAEYLLEHGADVNAQDKGGLIPLHNAAS      879
TNKS1[P.abelii][XP_024106876.1]           QKLCTPENINCRDTQGRNSTPLHLAAGYNNLEVAEYLLEHGADVNAQDKGGLIPLHNAAS      879
TNKS1[M.musculus][EDL35446.1]             QKLCTPENINCRDTQGRNSTPLHLAAGYNNLEVAEYLLEHGADVNAQDKGGLIPLHNAAS      874
TNKS2[S.salar][XP_014034742.1]            EKLCSPDNVNCRDAQGRHSTLLHLAAGYNNLEVAEYLLQHGAEVNSQDKGGLIPLHNAAS      750
TNKS2[X.laevis][XP_018082988.1]           KKLCTPENVNCRDTQGRHSTPLHLAAGYNNLEVAEYLLQHGADVNAADKGGLIPLHNAAS      722
TNKS2[G.gallus][NP_989672.1]              KKLCSPDNVNCRDTQGRHSTPLHLAAGYNNLEVAEYLLQHGADVNAQDKGGLIPLHNAAS      722
TNKS2[M.musculus][NP_001157107.1]         KKLSSPDNVNCRDTQGRHSTPLHLAAGYNNLEVAEYLLQHGADVNAQDKGGLIPLHNAAS      721
TNKS2[H.sapiens][AAK13463.1]              KKLSSPDNVNCRDTQGRHSTPLHLAAGYNNLEVAEYLLQHGADVNAQDKGGLIPLHNAAS      721
TNKS2[P.abelii][XP_024109627.1]           KKLSSPDNVNCRDTQGRHSTPLHLAAGYNNLEVAEYLLQHGADVNAQDKGGLIPLHNAAS      721
                                           :* : :.:****  **:** ********:.: .*:**:: *:**: *****:*****:*

TNKS[A.queenslandica][XP_019848937.1]     YGHVDVASLLIRYNSVINATDRWNFTPLHEAAQKGRTQVCSLLIIHGADVYLKNQEGQIP      793
TNKS[D.melanogaster][NP_651410.1]         YGHLDIAALLIKHKTVVNATDKWGFTPLHEAAQKGRTQLCSLLLAHGADAYMKNQEGQTP      775
TNKS[N.vectensis][XP_032220531.1]         YGHVDIAALLIRYNTDVNATDRWLFTPLHEAAQKGRTQLCALLLAHGAEPSMKNQEGQSP      787
TNKS[P.marinus][XP_032806710.1]           YGHVDIAALLIKYSACVNATDKWAFTPLHEAAQKGRTQLCALLLAHGADPSMKNQEGQTP      855
TNKS[B.belcheri][XP_019641281.1]          YGHVEIAQLLIKYGTCVNATDRWNFTPLHEAAQKGRTQLCALLLAHGADPTMKNQEGQTP      777
TNKS1[S.salar][XP_014017013.1]            YGHVDIAALLIKFNTCVNATDKWAFTPLHEAAQKGRTQLCALLLAHGADPTMKNQEGQTA      864
TNKS1[X.laevis][XP_018099068.1]           YGHVDIAALLIKYNTCVNATDKWAFTPLHEAAQKGRTQLCALLLAHGADPTMKNQESQTP      910
TNKS1[G.gallus][NP_989671.1]              YGHVDIAALLIKYNTCVNATDKWAFTPLHEAAQKGRTQLCALLLAHGADPTMKNQEGQTP      878
TNKS1[H.sapiens][NP_003738.2]             YGHVDIAALLIKYNTCVNATDKWAFTPLHEAAQKGRTQLCALLLAHGADPTMKNQEGQTP      939
TNKS1[P.abelii][XP_024106876.1]           YGHVDIAALLIKYNTCVNATDKWAFTPLHEAAQKGRTQLCALLLAHGADPTMKNQEGQTP      939
TNKS1[M.musculus][EDL35446.1]             YGHVDIAALLIKYNTCVNATDKWAFTPLHEAAQKGRTQLCALLLAHGADPTMKNQEGQTP      934
TNKS2[S.salar][XP_014034742.1]            YGHVDVAALLIKYDACVNATDKWAFTPLHEAAQKGRTQLCALLLAHGADPALRNQEGQSP      810
TNKS2[X.laevis][XP_018082988.1]           YGHVDVAALLIKYNACVNANDKWAFTPLHEAAQKGRTQLCALLLAHGADPALKNQEGQAP      782
TNKS2[G.gallus][NP_989672.1]              YGHVDVAALLIKYNACVNATDKWAFTPLHEAAQKGRTQLCALLLAHGADPTLKNQEGQTP      782
TNKS2[M.musculus][NP_001157107.1]         YGHVDVAALLIKYNACVNATDKWAFTPLHEAAQKGRTQLCALLLAHGADPTLKNQEGQTP      781
TNKS2[H.sapiens][AAK13463.1]              YGHVDVAALLIKYNACVNATDKWAFTPLHEAAQKGRTQLCALLLAHGADPTLKNQEGQTP      781
TNKS2[P.abelii][XP_024109627.1]           YGHVDVAALLIKYNACVNATDKWAFTPLHEAAQKGRTQLCALLLAHGADPTLKNQEGQTP      781
                                          ***:::* ***:. : :**.*:* *************:*:**:  ***:  ::***.*

TNKS[A.queenslandica][XP_019848937.1]     LDLATADDVIALLQDAMMKDIPLT-IPPAEKEAKSNIVN--------KG----------      833
TNKS[D.melanogaster][NP_651410.1]         IELATADDVKCLLQDAMATSLSQQALSASTQSLTSSSPAPDATAAAAPGTSSSSSSAILS      835
```

```
TNKS[N.vectensis][XP_032220531.1]       VDLATAEDVKCLLGDAMLSTQTTSVSTAPVAKVPAPVTAT-AGAA---------------          831
TNKS[P.marinus][XP_032806710.1]         LDLATADDVRALLIDAMPPEALPVTHKPPSIPTIAATSAA-ATAALALGPTPALA--SLA          912
TNKS[B.belcheri][XP_019641281.1]        LDLATAEDVRALLVDAMPPQSLPAAITAATVKAASP------------------------          813
TNKS1[S.salar][XP_014017013.1]          LDLATADDIRALLMDAMPPDALPSCFKPQATVVSAGS-----------------------          901
TNKS1[X.laevis][XP_018099068.1]         LDLATADDIRALLIDAMPPEALPSCFKPQATVVSA-S-----------------------          946
TNKS1[G.gallus][NP_989671.1]            LDLATADDIRALLIDAMPPEALPTCFKLQATVVSA-S-----------------------          914
TNKS1[H.sapiens][NP_003738.2]           LDLATADDIRALLIDAMPPEALPTCFKPQATVVSA-S-----------------------          975
TNKS1[P.abelii][XP_024106876.1]         LDLATADDIRALLIDAMPPEALPTCFKPQATVVSA-S-----------------------          975
TNKS1[M.musculus][EDL35446.1]           LDLATADDIRALLIDAMPPEALPTCFKPQATVVSA-S-----------------------          970
TNKS2[S.salar][XP_014034742.1]          LDLVTVDDVRALLTAAMPPSALPGCYKPQVISMSSPV-----------------------          847
TNKS2[X.laevis][XP_018082988.1]         LDLVTADDVRALLTAAMPPAALPTFYKPQLLNVPQPS-----------------------          819
TNKS2[G.gallus][NP_989672.1]            LDLVTADDVSALLTAAMPPSALPSCYKPQVISVSQTA-----------------------          819
TNKS2[M.musculus][NP_001157107.1]       LDLVSADDVSALLTAAMPPSALPTCYKPQVLSGVRGP-----------------------          818
TNKS2[H.sapiens][AAK13463.1]            LDLVSADDVSALLTAAMPPSALPSCYKPQVLNGVRSP-----------------------          818
TNKS2[P.abelii][XP_024109627.1]         LDLVSADDVSALLTAAMPPSALPSCYKPQVLNGVRSP-----------------------          818
                                        ::*.:.:*: .**  **

TNKS[A.queenslandica][XP_019848937.1]   ------LTAAGASLL--------------ASELVLGDGVDDKNGMVTQ----QRGGPAGV          869
TNKS[D.melanogaster][NP_651410.1]       PTTETVLLPTGASMIL-------SVP--VPLPLSSSTRISP---------------AQG-          870
TNKS[N.vectensis][XP_032220531.1]       --------------------TPATGPISGMAPLSQANSS----------LLANSPAGHG-          860
TNKS[P.marinus][XP_032806710.1]         P----SPVPSGASASGAGGVPLLASPSSTPASLSAASSMDSLTGACGVAPSPVPAGAIGA          968
TNKS[B.belcheri][XP_019641281.1]        ------------------ISSPSTTPSSTPGVLSTASSMDHLVGA-----------V-GG          843
TNKS1[S.salar][XP_014017013.1]          ---------------V-------ISPASTPSCLSAASSIDNLAG-----PLNELGAA-GT          933
TNKS1[X.laevis][XP_018099068.1]         ---------------I-------ISPPSTPSCLSAASSIDNLTG-----PLAELAVG-GV          978
TNKS1[G.gallus][NP_989671.1]            ---------------L-------ISPASTPSCLSAASSIDNLTG-----PLAELAVG-GA          946
TNKS1[H.sapiens][NP_003738.2]           ---------------L-------ISPASTPSCLSAASSIDNLTG-----PLAELAVG-GA          1007
TNKS1[P.abelii][XP_024106876.1]         ---------------L-------ISPASTPSCLSAASSIDNLTG-----PLAELAVG-GA          1007
TNKS1[M.musculus][EDL35446.1]           ---------------L-------ISPASTPSCLSAASSIDNLTG-----PLTDLAVG-GA          1002
TNKS2[S.salar][XP_014034742.1]          ---------------GVVVPPSLSASSTPLSTLASRNSLDNQATASTCTAFPELPAL---          889
TNKS2[X.laevis][XP_018082988.1]         ---------------TTAIAALSSVPLSS-PTLSAASSLDNL-----ASSFSETTSV-SS          857
TNKS2[G.gallus][NP_989672.1]            ---------------SSTADPLSSVPSSP-SSLSAASSLDNL-----SGSFSELPSV-VG          857
TNKS2[M.musculus][NP_001157107.1]       ---------------GATADALSSGPSSP-SSLSAASSLDNL-----SGSFSELSAV-VS          856
TNKS2[H.sapiens][AAK13463.1]            ---------------GATADALSSGPSSP-SSLSAASSLDNL-----SGSFSELSSV-VS          856
TNKS2[P.abelii][XP_024109627.1]         ---------------GAPADALSSGPSSP-SSLSAASSLDNL-----SGSFSELSSV-VS          856
                                                                        *

TNKS[A.queenslandica][XP_019848937.1]   GDGSDKGFMSYR----------------GGGDAPPPWHNVTVKDILTELELGHLVELFER          913
TNKS[D.melanogaster][NP_651410.1]       --------------AEANGAEGSSS---DD-LLPDADTITNVSGFLSSQQLHHLIELFER          912
TNKS[N.vectensis][XP_032220531.1]       ----DGAFSNRS-VSE---AQ---GEAVRSISAVHPGLDVDVGQFLDGLQLNNLKEIFER          909
TNKS[P.marinus][XP_032806710.1]         GAAGDGTLLGACGVSAADGATGPADKPDRSADFEIPGLDMNINMFLKGLGLDHLRDIFEK          1028
TNKS[B.belcheri][XP_019641281.1]        -------------AGQGDGAIDR-----SAVEG-ESLLDMTISSFLQQLGLNQLLDIFNK          884
TNKS1[S.salar][XP_014017013.1]          -------------SGVADGATGS-----DRKEGELVMLDMNISQFLKSLGLEHLRDIFER          975
TNKS1[X.laevis][XP_018099068.1]         -------------SNAGDGAAGT-----ERKEGEVTVLDININQFLKSLGLEHLRDIFET          1020
TNKS1[G.gallus][NP_989671.1]            -------------SNTGDGAAGT-----ERKEGEVSGLDMNITQFLKSLGLEHLRDIFET          988
TNKS1[H.sapiens][NP_003738.2]           -------------SNAGDGAAGT-----ERKEGEVAGLDMNISQFLKSLGLEHLRDIFET          1049
TNKS1[P.abelii][XP_024106876.1]         -------------SNAGDGAAGT-----ERKEGEIAGLDMNISQFLKSLGLEHLRDIFET          1049
TNKS1[M.musculus][EDL35446.1]           -------------SNAGDGAAGA-----ERKEGEVAGLDMNISQFLKSLGLEHLRDIFET          1044
TNKS2[S.salar][XP_014034742.1]          --------------LGPSGAVGT-----DN--KEVPGVDLSIGQFLNNLGLEHLLEIFER          928
TNKS2[X.laevis][XP_018082988.1]         --------------GGAEGASGL-----DK---KDEGVDLNINQFLRNLGLEHLIDVFER          895
TNKS2[G.gallus][NP_989672.1]            -------------TNSAEGATVL-----EK--KEVSGVDFSINQFVRNLGLEHLIDIFER          897
TNKS2[M.musculus][NP_001157107.1]       -------------SSAAEGATGL-----QR--KEDSGIDFSITQFIRNLGLEHLMDIFER          896
TNKS2[H.sapiens][AAK13463.1]            -------------SSGTEGASSL-----EK--KEVPGVDFSITQFVRNLGLEHLMDIFER          896
TNKS2[P.abelii][XP_024109627.1]         -------------SSGTEGTSSL-----EK--KEVPGVDFSITQFLRNLGLEHLMDIFER          896
                                                                                 :  ::    * :* ::*:

TNKS[A.queenslandica][XP_019848937.1]   EQITIDILIEMNGDDLQSIGITAFGVRHRLLKRIRELVQGNNEEY--P---VGVTTTKPT          968
TNKS[D.melanogaster][NP_651410.1]       EQITLDILAEMGHDDLKQVGVSAYGFRHKILKGIAQLRSTTGIG------------NNVN          960
TNKS[N.vectensis][XP_032220531.1]       EQISWDVLVDMGHEELKEIGIHAYGHRHKILKAVKEKISGMGLG-----LGPFST--SQV          962
TNKS[P.marinus][XP_032806710.1]         EQITLDVLADMGHEELKEIGINAYGHRHKLIKGVERLLGAQQGGLAYPGANPYLSYHTTA          1088
TNKS[B.belcheri][XP_019641281.1]        EQITLDILGEMGHEELKEIGVNAYGHRHKLIKGVERILGGTGT-----TLNPYLTPALGS          939
TNKS1[S.salar][XP_014017013.1]          EQISLDVLADMGHEELKEIGINAYGHRHKLIKGVERLLGGQQ------GANPYLTFHCAN          1029
TNKS1[X.laevis][XP_018099068.1]         EQITLDVLADMGHEELKEIGINAYGHRHKLIKGVERLLGGQQ------GTNPYLTFHCVS          1074
TNKS1[G.gallus][NP_989671.1]            EQITLDVLADMGHEELKEIGINAYGHRHKLIKGVERLLGGQQ------GTNPYLTFHCVS          1042
TNKS1[H.sapiens][NP_003738.2]           EQITLDVLADMGHEELKEIGINAYGHRHKLIKGVERLLGGQQ------GTNPYLTFHCVN          1103
TNKS1[P.abelii][XP_024106876.1]         EQITLDVLADMGHEELKEIGINAYGHRHKLIKGVERLLGGQQ------GTNPYLTFHCVN          1103
TNKS1[M.musculus][EDL35446.1]           EQITLDVLADMGHEELKEIGINAYGHRHKLIKGVERLLGGQQ------GTNPYLTFHCVN          1098
TNKS2[S.salar][XP_014034742.1]          EQITLDVLVEMGHKELKEIGINAYGHRHKIIKGVERLISGPQ------SLNPYLTLNTAN          982
TNKS2[X.laevis][XP_018082988.1]         EQITLDVLIEMGHKEFKEIGISAYGHRHKIIKGVERLISGQH------GLNPYLTLNTSN          949
TNKS2[G.gallus][NP_989672.1]            EQITLDVLVEMGHKELKEIGINAYGHRHKIIKGVERLISGQQ------GLNPYLTLNTSS          951
TNKS2[M.musculus][NP_001157107.1]       EQITLDVLVEMGHKELKEIGINAYGHRHKLIKGVERLISGQQ------GLNPYLTLNNSG          950
TNKS2[H.sapiens][AAK13463.1]            EQITLDVLVEMGHKELKEIGINAYGHRHKLIKGVERLISGQQ------GLNPYLTLNTSG          950
TNKS2[P.abelii][XP_024109627.1]         EQITLDVLVEMGHKELKEIGINAYGHRHKLIKGVERLISGQQ------GLNPYLTLNTSG          950
                                        ***: *:* :*. .::::.:*: *:* **:::* : .

TNKS[A.queenslandica][XP_019848937.1]   QGTQLIELSSDDKEFIDTADLMQSTICEHRDDGKAGGVFDSYEILKIERIVNTKVWERYK          1028
TNKS[D.melanogaster][NP_651410.1]       LCTLLVDLLPDDKEFVAVEEEMQATIREHRDNGQAGGYFTRYNIIRVQKVQNRKLWERYA          1020
TNKS[N.vectensis][XP_032220531.1]       QGSVIQELSMVDKDFASVADEMQSTIVEHRDSGTAGGVFTSYTILKIERVVNTKLWEKYV          1022
TNKS[P.marinus][XP_032806710.1]         QGTLLIDLPPDDKEYQSVEDEMQSTIREHKDGGQAGGVFNRYNVIKIQKVRNKKLWERYT          1148
TNKS[B.belcheri][XP_019641281.1]        HGTILTDLSPDDKEYQSVEEEVQSTIRQHKDQGQAGGIFNRYNVIKIQKVRNKRLWDRYV          999
TNKS1[S.salar][XP_014017013.1]          QGTVLIDLAPDDKEGQSVEEEMQSTIREHRDGGNAGGVFSRYNIIKIQKVVNKKLRERYT          1089
TNKS1[X.laevis][XP_018099068.1]         QGTVLLDLASDDKEYQSVEEEMQNSIREHRDGGNAGGVFNRYNVIRIQKVVNKKLRERFC          1134
TNKS1[G.gallus][NP_989671.1]            QGTILLDLAPDDKEYQSVEEEMQSTIREHRDGGNAGGIFNRYNVIRIQKVVNKKLRERFC          1102
TNKS1[H.sapiens][NP_003738.2]           QGTILLDLAPEDKEYQSVEEEMQSTIREHRDGGNAGGIFNRYNVIRIQKVVNKKLRERFC          1163
TNKS1[P.abelii][XP_024106876.1]         QGTILLDLAPEDKEYQSVEEEMQSTIREHRDGGNAGGIFNRYNVIRIQKVVNKKLRERFC          1163
TNKS1[M.musculus][EDL35446.1]           QGTILLDLAPEDKEYQSVEEEMQSTIREHRDGGNAGGIFNRYNVIRIQKVVNKKLRERFC          1158
TNKS2[S.salar][XP_014034742.1]          SGTILIDLVSDDKEFQLVEEELQSTIREHRDGGLAGGVFNRYNIVKIQKVCNKKLWERYT          1042
TNKS2[X.laevis][XP_018082988.1]         SGTILIDLAPDDKEFLSVEEEMQSTVREHRDGGHAGGVFTKYNILKIQKVCNKKLWEKYT          1009
TNKS2[G.gallus][NP_989672.1]            SGTLLIDLSSEDKEFQSVEEEMQSTVREHRDGGHAGGVFNRYNILKIQKVCNKKLWERYT          1011
TNKS2[M.musculus][NP_001157107.1]       SGTILIDLSPDDKEFQSVEEEMQSTVREHRDGGHAGGVFNRYNILKIQKVCNKKLWERYT          1010
TNKS2[H.sapiens][AAK13463.1]            SGTILIDLSPDDKEFQSVEEEMQSTVREHRDGGHAGGIFNRYNILKIQKVCNKKLWERYT          1010
TNKS2[P.abelii][XP_024109627.1]         SGTILIDLSPDDKEFQSVEEEMQSTVREHRDGGHAGGIFNRYNILKIQKVCNKKLWERYT          1010
                                          : : :*   **:   . : :* :: :*:* * *** *  * ::::::: * :: :::

TNKS[A.queenslandica][XP_019848937.1]   YRRKEVAESNNNCANELMLFHGSPFVPYIVHNGFDERHAYIGGMFGAGIYFAEHSSKSNQ          1088
TNKS[D.melanogaster][NP_651410.1]       HRRQEIAEENFLQSNERMLFHGSPFINAIVQRGFDERHAYIGGMFGAGIYFAEHSSKSNQ          1080
TNKS[N.vectensis][XP_032220531.1]       YRRREIADSNHNHANERMLFHGSPFINAIVQKGFDQRHAYIGGMFGAGIYFAENSSKSNQ          1082
TNKS[P.marinus][XP_032806710.1]         HRRKEVTEENHNHHNERMLFHGSPFINAIINKGFDERHAYIGGMFGAGIYFAENSSKSNQ          1208
TNKS[B.belcheri][XP_019641281.1]        HRRKEVSEENHNQSNERMLFHGSPFINAIINKGFDERHAYIGGMFGAGIYFAENSSKSNQ          1059
TNKS1[S.salar][XP_014017013.1]          HRQKEISDENHNHHNERMLFHGSPFINAIIHKGFDERHAYIGGMFGAGIYFAENSSKSNQ          1149
TNKS1[X.laevis][XP_018099068.1]         HRQKEVSEENHNHHNERMLFHGSPFINAIIHKGFDERHAYIGGMFGAGIYFAENSSKSNQ          1194
TNKS1[G.gallus][NP_989671.1]            HRQKEVSEENHNHHNERMLFHGSPFINAIIHKGFDERHAYIGGMFGAGIYFAENSSKSNQ          1162
TNKS1[H.sapiens][NP_003738.2]           HRQKEVSEENHNHHNERMLFHGSPFINAIIHKGFDERHAYIGGMFGAGIYFAENSSKSNQ          1223
```

```
TNKS1[P.abelii][XP_024106876.1]          HRQKEVSEENHNHHNERMLFHGSPFINAIIHKGFDERHAYIGGMFGAGIYFAENSSKSNQ           1223
TNKS1[M.musculus][EDL35446.1]            HRQKEVSEENHNHHNERMLFHGSPFINAIIHKGFDERHAYIGGMFGAGIYFAENSSKSNQ           1218
TNKS2[S.salar][XP_014034742.1]           HRRKEVSEENHNHSNERMLFHGSPFVNAIIHKGFDERHAYIGGMFGAGIYFAENSSKSNQ           1102
TNKS2[X.laevis][XP_018082988.1]          HRQKEVTEENHNHSNERMLFHGSPFVNAIIHKGFDERHAYIGGMFGAGIYFAENSSKSNQ           1069
TNKS2[G.gallus][NP_989672.1]             HRRKEVSEENHNHANERMLFHGSPFVNAIIHKGFDERHAYIGGMFGAGIYFAENSSKSNQ           1071
TNKS2[M.musculus][NP_001157107.1]        HRRKEVSEENHNHANERMLFHGSPFVNAIIHKGFDERHAYIGGMFGAGIYFAENSSKSNQ           1070
TNKS2[H.sapiens][AAK13463.1]             HRRKEVSEENHNHANERMLFHGSPFVNAIIHKGFDERHAYIGGMFGAGIYFAENSSKSNQ           1070
TNKS2[P.abelii][XP_024109627.1]          HRRKEVSEENHNHANERMLFHGSPFVNAIIHKGFDERHAYIGGMFGAGIYFAENSSKSNQ           1070
                                         :*::*:::.*    ** ********:  *::.***:****************:******

TNKS[A.queenslandica][XP_019848937.1]    YVYGIGGGNGCPEHKNRSCYTCLRKLLLCRVVLGKPVEQYTAVRIAHAPPGHHSVIGRPS           1148
TNKS[D.melanogaster][NP_651410.1]        YVYGIGGGIGCPSHKDKSCYVCPRQLLLCRVALGKSFLQYSAMKMAHAPPGHHSVVGRPS           1140
TNKS[N.vectensis][XP_032220531.1]        YVYGIGGGSGCPTHKDRSCYSCERQLLLCRVALGKPFYQFSAVKMAHAPPGHNSVIGRPS           1142
TNKS[P.marinus][XP_032806710.1]          YVYGIGGGTGCPVHKDRSCYICQRQMLFCRVTLGKSFLQFSAMKMAHSPPGHHSVIGRPS           1268
TNKS[B.belcheri][XP_019641281.1]         YVYGIGGGTGCPIHKDRSCYICQRQMLFCRVTLGKAFLQFSAMKMAHAPPGHHSVIGRPS           1119
TNKS1[S.salar][XP_014017013.1]           YVYGIGGGTGCPTHKDRSCYLCHRQMLFCRVTLGKSFLQFSAMKMAHAPPGHHSVIGRPS           1209
TNKS1[X.laevis][XP_018099068.1]          YVYGIGGGTGCPTHKDRSCYICHRQMLFCRVTLGKSFLQFSTMKMAHAPPGHHSVIGRPS           1254
TNKS1[G.gallus][NP_989671.1]             YVYGIGGGTGCPTHKDRSCYICHRQMLFCRVTLGKSFLQFSTMKMAHAPPGHHSVIGRPS           1222
TNKS1[H.sapiens][NP_003738.2]            YVYGIGGGTGCPTHKDRSCYICHRQMLFCRVTLGKSFLQFSTMKMAHAPPGHHSVIGRPS           1283
TNKS1[P.abelii][XP_024106876.1]          YVYGIGGGTGCPTHKDRSCYICHRQMLFCRVTLGKSFLQFSTMKMAHAPPGHHSVIGRPS           1283
TNKS1[M.musculus][EDL35446.1]            YVYGIGGGTGCPTHKDRSCYICHRQMLFCRVTLGKSFLQFSTMKMAHAPPGHHSVIGRPS           1278
TNKS2[S.salar][XP_014034742.1]           YVYGIGGGTGCPLHKDRSCYVCQRHLLFCRVTLGKSFLQFSAMKMAHSPPGHHSVTGRPS           1162
TNKS2[X.laevis][XP_018082988.1]          YVYGIGGGTGCPIHKDRSCYVCHRQLLFCRVTLGKSFLQFSAMKMAHSPPGHHSVTGRPS           1129
TNKS2[G.gallus][NP_989672.1]             YVYGIGGGTGCPIHKDRSCYVCHRQLLFCRVTLGKSFLQFSAMKMAHSPPGHHSVTGRPS           1131
TNKS2[M.musculus][NP_001157107.1]        YVYGIGGGTGCPIHKDRSCYICHRQLLFCRVTLGKSFLQFSAMKMAHSPPGHHSVTGRPS           1130
TNKS2[H.sapiens][AAK13463.1]             YVYGIGGGTGCPVHKDRSCYICHRQLLFCRVTLGKSFLQFSAMKMAHSPPGHHSVTGRPS           1130
TNKS2[P.abelii][XP_024109627.1]          YVYGIGGGTGCPVHKDRSCYICHRQLLFCRVTLGKSFLQFSAMKMAHSPPGHHSVTGRPS           1130
                                         ******** *** **::*** * *::*:***.*** . *:::::**:****:** ****

TNKS[A.queenslandica][XP_019848937.1]    AGGLNYPEYVIYRGEQAYPEYIITFRIKKPSATDSMSSSSSLDMSNNT           1196
TNKS[D.melanogaster][NP_651410.1]        AGGLHFAEYVVYRGEQSYPEYLITYQIVKPDDSSSGTEDTR-------           1181
TNKS[N.vectensis][XP_032220531.1]        SGGLSFAEYVIYRGEQAYPEYLITYKINKPDT----------------           1174
TNKS[P.marinus][XP_032806710.1]          VNGLAFAEYVIYRGEQAYPEYLITYQILKPECTPDLTSAAGQKS----           1312
TNKS[B.belcheri][XP_019641281.1]         VGGLNFAEYVIYRGEQAFPEYLITYQIVKPEAPAAPSPDQK-------           1160
TNKS1[S.salar][XP_014017013.1]           VNGLAYAEYVIYRGEQAYPEYLITYQILKPESTATSAAGEDQKS----           1253
TNKS1[X.laevis][XP_018099068.1]          VNGLAYAEYVIYRGEQAYPEYLITYQIMKPETPLQAATTAEQKT----           1298
TNKS1[G.gallus][NP_989671.1]             VNGLAYAEYVIYRGEQAYPEYLITYQIVKPEAPSQTGTAAEQKT----           1266
TNKS1[H.sapiens][NP_003738.2]            VNGLAYAEYVIYRGEQAYPEYLITYQIMKPEAPSQTATAAEQKT----           1327
TNKS1[P.abelii][XP_024106876.1]          VNGLAYAEYVIYRGEQAYPEYLITYQIMKPEAPSQTATAAEQKT----           1327
TNKS1[M.musculus][EDL35446.1]            VNGLAYAEYVIYRGEQAYPEYLITYQIMKPEAPSQTATAAEQKT----           1322
TNKS2[S.salar][XP_014034742.1]           VNGLSLAEYVIYRGEQAYPEYLITYQILKPDASVDG------------           1198
TNKS2[X.laevis][XP_018082988.1]          VNGLALAEYVIYRGEQAYPEYIITYQIMKPEAVAEG------------           1165
TNKS2[G.gallus][NP_989672.1]             VNGLALAEYVIYRGEQAYPEYLITYQIVKPEATTEA------------           1167
TNKS2[M.musculus][NP_001157107.1]        VNGLALAEYVIYRGEQAYPEYLITYQIVRPEGMVDG------------           1166
TNKS2[H.sapiens][AAK13463.1]             VNGLALAEYVIYRGEQAYPEYLITYQIMRPEGMVDG------------           1166
TNKS2[P.abelii][XP_024109627.1]          VNGLALAEYVIYRGEQAYPEYLITYQIMRPEGMVDG------------           1166
                                          .**   ***:*****::***:**::* :*.
```
