# Supplementary material for: An Evolutionary Perspective on the Origin, Conservation and Binding Partner Acquisition of Tankyrases
Source: Biomolecules. 2022 Nov 15;12(11):1688. doi: 10.3390/biom12111688 (PMC9688111; doi:10.3390/biom12111688)
Supplement: Supplementary file 1 [file biomolecules-12-01688-s001.zip › biomolecules-1913719-supplementary-11.17 -done/Data_7/AMOT.pdf]

AMOT

Homo sapiens:

>Q4VCS5 [Homo sapiens]
MRNSEEQPSGGTTVLQRLLQEQLRYGNPSENRSLLAIHQQATGNGPPFPSGSGNPGPQSD
VLSPQDHHQQLVAHAARQEPQGQEIQSENLIMEKQLSPRMQNNEELPTYEEAKVQSQYFR
GQQHASVGAAFYVTGVTNQKMRTEGRPSVQRLNPGKMHQDEGLRDLKQGHVRSLSERLMQ
MSLATSGVKAHPPVTSAPLSPPQPNDLYKNPTSSSEFYKAQGPLPNQHSLKGMEHRGPPP
EYPFKGMPPQSVVCKPQEPGHFYSEHRLNQPGRTEGQLMRYQHPPEYGAARPAQDISLPL
SARNSQPHSPTSSLTSGGSLPLLQSPPSTRLSPARHPLVPNQGDHSAHLPRPQQHFLPNQ
AHQGDHYRLSQPGLSQQQQQQQQHHHHHHQQQQQQPQQQPGEAYSAMPRAQPSSASY
QPVPADPFAIVSRAQQMVEILSDENRNLRQELEGCYEKVARLQKVETEIQRVSEAYENLV
KSSSKREALEKAMRNKLEGEIRRMHDFNRDLRERLETANKQLAEKEYEGSEDTRKTISQL
FAKNKESQREKEKLEAELATARSTNEDQRRHIEIRDQALSNAQAKVVKLEEELKKKQVYV
DKVEKMQQALVQLQAACEKREQLEHRLRTRLERELESLRIQQRQGNCQPTNVSEYNAAAL
MELLREKEERILALEADMTKWEQKYLEENVMRHFALDAAATVAAQRDTTVISHSPNTSYD
TALEARIQKEEEEILMANKRCLDMEGRIKTLHAQIIEKDAMIKVLQQRSRKEPSKTEQLS
CMRPAKSLMSISNAGSGLLSHSSTLTGSPIMEEKRDDKSWKGSLGILLGGDYRAEYVPST
PSPVPPSTPLLSAHSKTGSRDCSTQTERGTESNKTAAVAPISVPAPVAAAATAAAITATA
ATITTTMVAAAPVAVAAAAAPAAAAAPSPATAAATAAAVSPAAAGQIPAAASVASAAAVA
PSAAAAAAVQVAPAAPAPVPAPALVPVPAPAAAQASAPAQTQAPTSAPAVAPTPAPTPTP
AVAQAEVPASPATGPGPHRLSIPSLTCNPDKTDGPVFHSNTLERKTPIQILGQEPDAEMV
EYLI

Pongo abelii:

>XP_009233461.1 angiomotin isoform X1 [Pongo abelii]
MRNSEEQPSGGTTVLQRLLQEQLRYGNPSENRSLLAIHQQATGNGPPFPSGSGNPGPQSDVLSPQDHHQQ
LVAHAARQEPQGQEIQSENLIMEKQLSPRMQNNEELPTYEEAKVQSQYFRGQQHASVGAAFYVTGVTNQK
MRTEGRPSVQRLNPGKMHQDEGLRDLKQGHVRSLSERLMQMSLATSGVKAHPPVTSAPLSPPQPNDLYKN
PTSSSEFYKAQGPLPNQHSLKGMEHRGPPPEYPFKGMPPQSVVCKPQEPGHFYSEHRLNQPGRTEGQLMR
YQHPPEYGAARPAQDISLPLSARNSQPHSPTSSLTSGGSLPLLQSPPSTRLSPARHPLVPNQGDHSAHLP
RPQQHFLPNQAHQGDHYRLSQPGLSQQQQQQQQHHHHHHQQQQQQPQQQPGEAYSAMPRAQPSSASYQ
PVPADPFAIVSRAQQMVEILSDENRNLRQELEGCYEKVARLQKVETEIQRVSEAYENLVKSSSKREALEK
AMRNKLEGEIRRMHDFNRDLRERLETANKQLAEKEYEGSEDTRKTISQLFAKNKESQREKEKLEAELATA
RSTNEDQRRHIEIRDQALSNAQAKVVKLEEELKKKQVYVDKVEKMQQALVQLQAACEKREQLEHRLRTRL
ERELESLRIQQRQGNCQPTNVSEYNAAALMELLREKEERILALEADMTKWEQKYLEENVMRHFALDAAAT
VAAQRDTTVISHSPNTSYDTALEARIQKEEEEILMANKRCLDMEGRIKTLHAQIIEKDAMIKVLQQRSRK
EPSKTEQLSCMRPAKSLMSISNAGSGLLSHSSTLTGSPIMEEKRDDKSWKGSLGILLGGDYRAEYVPSTP
SPVPPSTPLLSAHSKTGSRDCSTQTERGTESNKTAAVAPISVPAPVAAAATAAAITATAATITTTMVAAA
PVAVAAAAAPAAAAAAAAAPSPATAAAIAAAVSPAAAGQIPAAASVASVAAVAPPPAAAAAAVQVAPAAP
APVPAPALVPVPAPAAAQASAPAQTQTPTSAPAVAPTPAPTPTPAVAQAEVPASPATGPGPHRLSVPSLT
CNPDKTDGPVFHSNTLERKTPIQILGQEPDAEMVEYLI

Mus musculus:

>NP_695231.3 angiomotin isoform 1 [Mus musculus]
MRSSDDQPSGGTTVLQRLLQEQLRYGNPSENRSLLAIHQQATGNSSPFSTGSGNQGPQNDVLSSQDHHQQ
QLVAHPARQEPQGQEIQSENGVMEKQLSPRMQNNEELPTYEEAKVQSQYFRGQQHASVGAAFYVTGVTNQ
KMRTEGRPSVQRLTPGKMHQDEGLRDLKQGHVRSLSERLMQMSLATSGVKAHPPVTSAPLSPPQPNDLYK
NATSSSEFYKAQGPPPSQHSLKGMEHRGPPPEYPFKGVPSQSVVCKSQEPGHFYSEHRLNQPGRTEGQLM
RYQHPPEYGAARATQDISSLSLSARNSQPHSPTSSLTAGASSLPLLQSPPSTRLPPGQHLVSNQGDHSAH
LSRHQQHLLSSQSHQGDHYRHAQASLTSAQQQPGEAYSAMPRAQQSASYQPMPADPFAMVSRAQQMVEIL
SDENRNLRQELDGCYEKVARLQKVETEIQRVSEAYENLVKSSSKREALEKAMRNKLEGEIRRMHDFNRDL
RDRLETANKQLAEKEYEGSEDTRKTISQLFAKHKENQREKEKLEAELATARSTNEDQRRHIEIRDQALSN
AQAKVVKLEEELKKKQVYVDKVEKMQQALVQLQAACEKREQLEHRLRTRLERELESLRIQQRQGNSQPTN
ASEYNAAALMELLREKEERILALEADMTKWEQKYLEENVMRHFALDAAATVAAQRDTTVISHSPNTSYDT
ALEARIQKEEEEILMANKRCLDMEGRIKTLHAQIIEKDAMIKVLQQRSRKEPSKTEQLSSMRPAKSLMSI
SNAGSGLLAHSSTLTGAPIMEEKRDDKSWKGSLGVLLGGDYRVEPVPSTPSPVPPSTPLLSAHSKTGSRD
CSTQTERGPESTKTAAVTPISAPMAGPVAAAAPAAAINATAATNTATAATNTTIMVAAAPVAVAAVAAPA
AAAATPSPANAAALAAAAAPATSVSAATSVSAANSISPAAPVAPAAVVPPAAPVSPAAAVQIPAAASLTP
ATVSPTAATATAAVAAATTAAITAAAAAATTAIQVAPATSAPVPSPASIPAPATAQASAPTPTQASTPAP
TEPPSPVPTPTPALVQTEGPANPGASSGPRRLSTPNLMCNPDKPDAPAFHSSTLERKTPIQILGQEPDAE
MVEYLI

Gallus gallus:

>XP_004940856.2 angiomotin [Gallus gallus]
MRNSEEQAATTVLQRLLQEQLRYGNPNDNRNLLALHQQATGNAPPFNSTGNPASQNEGLSSQDHQLVTHA
ARQEPQGQEIQVDNSIMEKQLSPRLQNSEDLPTYEEAKVQSQYFRGQPHASVGAAFYVTGVTNQKMRTEG
RPTVQRVSPGKVHQDEGLKDLKQGHVRSLSERLMQLSLATSGVKAHAPVTSAPLSPLSPLSPQQSSDPYK
NSGSSEFYKNSVQPPSQPSMKGMEQRGPPPEYPYKNVSTPSMNCKPQDSGHFYSDHRVSQQGRSDGPMMR
YQHPPEYGSVRQNPDVQLQLQQRPPHHHSPTSSLTSLGSLTLLQSPPPSRLSPSQHQQPLTPSQGDPGIL
PRTQQPFLSNQVHQGDLYRLCQPFLGPQQQQGDSYSVMSRAQQIPSPYQQMQVDPFAIVSRAQQMVEILS
EENRSLRQELDGCYEKVARLQKLETEIQQVSEAYKNLEKSSSKRDALEKAMRNKLEGEIRRLHDFNRDLR
ERMETANKQLAEKEFEGSEDNRKTISQLFAQNKETQREKEKLEIELAAARSTNEDQRRHIEIRDQALNNA
QAKVVKLEEELKKKQVYVEKVEKMQQALVQLQAACEKREQLEHRLRTRLERELESLRMQQRQGTSQAANV
SEYNATALMELLREKEERILALEADMTKWEQKYLEESVMRQFALDAAATVAAQRDTTVISHSPNPSYDTS
LEARIQKEEEEILLANRRCLDMEGRIKTLHAQIIEKDAMIKVLQQRSRKEPGKTDQLSSMRPAKSLMSIS
NAGSGLLSHSSTLSSTPILEEKREDKSWKGSLGVLLGTEYRSESIPSTPSPVLPSTPLLSAHSKTGSRDC
STQTDRGNEQSKAAPSPAAPTPGRLPTASLPYSADKTDGPLFHSSTLERKAPVQTMGQDLPDGEMVEYLI

Xenopus laevis:

>XP_018085358.1 PREDICTED: angiomotin-like isoform X1 [Xenopus laevis]
MRTSEEQAATTVLQRLLQEQLRYGNPNENRNLLALHQQATGNVPSYGGGQGSQNEGLTLQDPQLVPHAAR
QEPQGQEIQVENLMEKQIPLRSQNSEDLPTYEEAKVQSQYFRGQPHTSVGAAFYVTGVTNQKMRTEGRPT
VQRVSPGKVHQDDGLKDLKHGHVRSLSERLMQLSLATSGVKAHAPITSAPLSPQPGDFYKNSDSNDMFKN
PPQCGGQKVTEHRGPPPDYPFKNISTLPLQGKSKEVGFHYGEHRGSLQNRSDGPMVRYQPPPEYGASRQN
QDVQQQIQHRPSHYHSPTSSLTSLGSMSLLQSPTIMSPSQQHPPSPSHGDFYTVPSRAHHIPVYNPVFQQ
GDVYRSPQSQFGQQQQMDPVLLRPPPVPSPYSQMQNDPFTIVSRAQNMVSLLSEENRSLRKELEDCYDKV
ARFQKMEMEIQRISEAYENLVKSSSKRETLEKAMRTKLEEEIRRIHDFNRDLRERMDTANKQLAAKEFEG
SEDNRKTISQLLAQNKEIQREKEKLEIEISSLRSTNQDQRRHIEIRDQALNNAQAKVVKLEEELKKKQVY
VDKVEKMQQALVQLQAACEKREQLEHRLRTRLERELESLRIQQRQGNSQSSNVSEYSAATLMELLREKEE
RVLALEADMTKWEQKYLEESVMRQFALDAAATVATQRDTASISHSPSGSFDTSLEARIQKEEEEILLANR
RCVDMEGRIKTLHAQIIEKDAMIKVLQQRSRKDPSKTDQSSTMRPSKSLMSISNAGSGLLSHSSALSSTP
ILEDKREDKSWKGSLGVLLATEYRSESASSTPSPIRPSTPLLCGHSKTGSKDCCTQTDRGAEQNKPPSVS
ASPGPGRLATPSPMYNSERTAETVFQSSTLDRKNPLPPLGQDLTDGELVEYLI

Salmo salar:

>XP_014052684.1 PREDICTED: angiomotin-like isoform X1 [Salmo salar]
MFRKKASSSLRMRRISESNRWNIDRGRSSSFHEVRGLRETEMRPVADETSSSSNSSNNTVLQRLLQEQMR
YGEGRNYQLAMQQHQQQQQQQRQQQEGGVSGGYPGHVGPNDERHDSMVPHIARQEPQGQELQADSGVEKL
GSRGGGSSGGGGMSGGGGGSSQGPNPEDLPTYEEAKVQSQYFRGHPHHQQHQQHQQQQQLPPSVGAAFYV
TAATNNGKVRTEGRPTVQRLSTGGKVHQDDGLKDLKQGHVRSLSERLMQLSLATSGVKAHAPITSAPLSP
QLPPPGPPGDYYKPSGQHRGPPPDYPFKNMGSPSKQQPQHQEQGGHYYQEHRPRDQGRNEVPHVRYQPPP
EYGSFRSNKESSVHSQRSVHHHSPTSSVTSVGSLSRAQSSTLNSLLSASHPSHTPPHPQGDPYASTGPRS
PQGPGSHQGDLYCPGAIHPPLQRGHGFPHDPYGSTSRMHHQQQHHQFQQQQQHQQQQQQQQQQQQQQQQQ
QQQQLQHQQYHSQSSQGHPPQHYPNPQGDPFAMMARAQQMVDMLSEENRLLKQEMAVCSDKVNKLQKLET
EITLVSEAYENLAKSSTKREALEKTMRNKLELEVRRLHDFNRDLRERMETANKQLAAKECEGSEDNRKTI
SQLLTQNKETVREKEKLEIELNALRSTTDDQRRHIEIRDQALNNAQAKVVKLEEELKKKQVYVEKVERMQ
QALAQLQAACEKREQLEHRLRTRLERELESLRMQQRQGVSQSSSSGQEYSATALMEHLREKEERILALEA
DMTKWEQKYLEESVMRQFALDAAASVATQRDTLSSVISHSPSSSYEQQLSVEARIQKEEEEILMANRRCL
DMESRIKNLHAQIIEKDAMIKVLHQRSRKDPNKDRDAQAIMRPSKSLMSIANTGGSGLLSHSLGLSSSPI
TEERKDSSWKGSLGVLLGPEYRTDSLRTESISSSPSPVLPLTGGHSKTGSRDSCTQTDKGQSQGQEASSK
PSTPALQSMTLPSRLSSPSPVYIPDRIADVPVFHSSTLERRAPVQSHPQPPSASQQELDNDMVEVLI

Petromyzon marinus:

>XP_032827792.1 angiomotin-like protein 1 [Petromyzon marinus]
MGDPYARERALEMRAAEDNSGKVLQRLIQEQLRYGGVTDNRGLLAIQQQATTAGSPTPANAATGAGASHG
ALSLAPTSLGAVSLGGAPAAAAGSSISSTESLVQDLRQSARQEPQGQEHQSSETPEKQAQRLALLQQQAL
QQQQYQQQQQCQQQQQYQQQQQYQQHHTATEELPSYEEAKVHSQYYSSLQSQGQGQTVHQARMSNTGPAA
FYVTATVGAQKGRTEGRPAVQRTSPLPIMQDDGLKELKQGHVRSLSERLMQLTLEKNGAKAQLPLGSSPP
SSVPQQSSADFCRLQNQSPPQRPYDPRGPPPEYPFKHKAAFAPQGSPLQLNQFTALQHQHHHVPGAAEQH
LLRYQPPPEYNMARQTQQGMQPMQMQYHSPSASQGSAGSLSIQMSGAGALPGPMQNLGSMTLTTAVAQQT
PMYTGLGGQLSMAHTAGPGEELHFMLLRSQQTVELLTEENQLLRSELESYCEKDIKLQKMEREVLRISEA
YENLEKSTSKRETLEKAMRTKMESEIRRLHDFNRDLRDRLETSNKQLASREYENSEDNKLTIAKLIAQNK
ETLLTKEKLEVELAGLRSANGDQRRHIELLNQAFSSSEAKAAKLQEELKKKQFYVDKVEKLQQALSQLQV
ACEKREQLERRLRTRLERELETLRAQQRQGAPPPGATSEHAAPALMELLREKEERVLALEADMMRWEQKY
LEESAMRQFAMDAAATAATERDTTIIANSPNASFSEEEEFMLASRRCHDMEGRIKNLHAQIIEKDAMIKV
LQQRSRKEQQGKTESSATSLRPAKSVPSIAAAASGSPGGGLASGLAARKPPMAASPSLEERLGARTGSFS
GLVSTELAEEGSTASVGLSPMRALSPLPPPSPHFTHVKSSSKDSSTQTERGAQEGSSSSPHMDPAAAERK
KSNTLPRGSKMLPSLHVLRVDARDRADMKMAAGEKLLDGRPPLHMSGLPQEDQVDNEMVEILI

Branchiostoma belcheri:

No homolog found.

Drosophila melanogaster:

No homolog found.

Hydra vulgaris:

No homolog found.

Nematostella vectensis:

No homolog found.

Amphimedon queenslandica:

No homolog found.

Alignment

```
XP_032827792.1[P.marinus]      ----------------------------------MGDPYARERALEMRAAE---DNSGKV     23
XP_014052684.1[S.salar]        MFRKKASSSLRMRRISESNRWNIDRGRSSSFHEVRGLRETEMRPVADETSSSSNSSNNTV     60
NP_695231.3[M.musculus]        ----------------------------------------------MRSSDDQPSGGTTV     14
Q4VCS5[H.sapiens]              ----------------------------------------------MRNSEEQPSGGTTV     14
XP_009233461.1[P.abelii]       ----------------------------------------------MRNSEEQPSGGTTV     14
XP_004940856.2[G.gallus]       ----------------------------------------------MRNSEEQ--AATTV     12
XP_018085358.1[X.laevis]       ----------------------------------------------MRTSEEQ--AATTV     12
                                                                              . :.       .*

XP_032827792.1[P.marinus]      LQRLIQEQLRYGGVTDNRGLLAIQQQATTAGSPTPANAATGAGASHGALSLAPTSLGAVS     83
XP_014052684.1[S.salar]        LQRLLQEQMRYGEGRNYQLAMQQHQQQQQQQRQQQE-GGVSGG-YPGHV-----------     107
NP_695231.3[M.musculus]        LQRLLQEQLRYGNPSENRSLLAIHQQATGNSSPFSTGSGNQGP-QNDVL-----------     62
Q4VCS5[H.sapiens]              LQRLLQEQLRYGNPSENRSLLAIHQQATGNGPPFPSGSGNPGP-QSDVL-----------     62
XP_009233461.1[P.abelii]       LQRLLQEQLRYGNPSENRSLLAIHQQATGNGPPFPSGSGNPGP-QSDVL-----------     62
XP_004940856.2[G.gallus]       LQRLLQEQLRYGNPNDNRNLLALHQQATGNAPPFNS-TGNPAS-QNEGL-----------     59
XP_018085358.1[X.laevis]       LQRLLQEQLRYGNPNENRNLLALHQQATGNVPSYG---GGQGS-QNEGL-----------     57
                               ****:***:***   : :  :  :**            .  .      :

XP_032827792.1[P.marinus]      LGGAPAAAAGSSISSTESLVQDLRQSARQEPQGQEHQSSETPEKQAQRLALLQQQALQQQ     143
XP_014052684.1[S.salar]        ------------GPNDERHDSMVPHIARQEPQGQELQADSGVEKLGSRGGG---------     146
NP_695231.3[M.musculus]        ------------SSQDHHQQQLVAHPARQEPQGQEIQSENGVMEKQ--------------     96
Q4VCS5[H.sapiens]              ------------SPQDHHQ-QLVAHAARQEPQGQEIQSENLIMEKQ--------------     95
XP_009233461.1[P.abelii]       ------------SPQDHHQ-QLVAHAARQEPQGQEIQSENLIMEKQ--------------     95
XP_004940856.2[G.gallus]       ------------SSQDH---QLVTHAARQEPQGQEIQVDNSIMEKQ--------------     90
XP_018085358.1[X.laevis]       ------------TLQDP---QLVPHAARQEPQGQEIQVEN-LMEKQ--------------     87
                                             .     . : : ********* * ..   :

XP_032827792.1[P.marinus]      QYQQQQQCQQQQQYQQQQQYQQHHTATEELPSYEEAKVHSQYYSSLQSQGQGQT-VHQAR     202
XP_014052684.1[S.salar]        -------SSGGGGMSGGGGGSSQGPNPEDLPTYEEAKVQSQYFRGHPHHQQHQQHQQQQQ     199
NP_695231.3[M.musculus]        -------------------LSPRMQNNEELPTYEEAKVQSQYFRGQQH------------     125
Q4VCS5[H.sapiens]              -------------------LSPRMQNNEELPTYEEAKVQSQYFRGQQH------------     124
XP_009233461.1[P.abelii]       -------------------LSPRMQNNEELPTYEEAKVQSQYFRGQQH------------     124
XP_004940856.2[G.gallus]       -------------------LSPRLQNSEDLPTYEEAKVQSQYFRGQPH------------     119
XP_018085358.1[X.laevis]       -------------------IPLRSQNSEDLPTYEEAKVQSQYFRGQPH------------     116
                                                     :    *:**:******:***: .

XP_032827792.1[P.marinus]      MSNTGPAAFYVTATVGAQKGRTEGRPAVQRTSP-LPIMQDDGLKELKQGHVRSLSERLMQ     261
XP_014052684.1[S.salar]        LPPSVGAAFYVTAATNNGKVRTEGRPTVQRLSTGGKVHQDDGLKDLKQGHVRSLSERLMQ     259
NP_695231.3[M.musculus]        --ASVGAAFYVTGVT-NQKMRTEGRPSVQRLTP-GKMHQDEGLRDLKQGHVRSLSERLMQ     181
Q4VCS5[H.sapiens]              --ASVGAAFYVTGVT-NQKMRTEGRPSVQRLNP-GKMHQDEGLRDLKQGHVRSLSERLMQ     180
XP_009233461.1[P.abelii]       --ASVGAAFYVTGVT-NQKMRTEGRPSVQRLNP-GKMHQDEGLRDLKQGHVRSLSERLMQ     180
XP_004940856.2[G.gallus]       --ASVGAAFYVTGVT-NQKMRTEGRPTVQRVSP-GKVHQDEGLKDLKQGHVRSLSERLMQ     175
XP_018085358.1[X.laevis]       --TSVGAAFYVTGVT-NQKMRTEGRPTVQRVSP-GKVHQDDGLKDLKHGHVRSLSERLMQ     172
                                  :  ******...   * ******:*** .    : **:**::**:************

XP_032827792.1[P.marinus]      LTLEKNGAKAQLPLGSSPPSSVP-------------QQSSADFCRLQNQSPP---QRPYD     305
XP_014052684.1[S.salar]        LSLATSGVKAHAPITSAPLSPQLPP-PGPPGDYYKPS---------------------GQ     297
NP_695231.3[M.musculus]        MSLATSGVKAHPPVTSAPLSPP------QPNDLYKNATSSSEFYKAQGPPPSQHSLKGME     235
Q4VCS5[H.sapiens]              MSLATSGVKAHPPVTSAPLSPP------QPNDLYKNPTSSSEFYKAQGPLPNQHSLKGME     234
XP_009233461.1[P.abelii]       MSLATSGVKAHPPVTSAPLSPP------QPNDLYKNPTSSSEFYKAQGPLPNQHSLKGME     234
XP_004940856.2[G.gallus]       LSLATSGVKAHAPVTSAPLSPLSPLSPQQSSDPYKNSG-SSEFYKNSVQPPSQPSMKGME     234
XP_018085358.1[X.laevis]       LSLATSGVKAHAPITSAPLSPQ-------PGDFYKNSD-SNDMFKN---PPQCGGQKVTE     221
                               ::* ..*.**: *: *:* *                                       :

XP_032827792.1[P.marinus]      PRGPPPEYPFKHKAAFAPQGSPLQLNQFTALQHQHHHVPGAAEQHLLRYQPPPEYNMARQ     365
XP_014052684.1[S.salar]        HRGPPPDYPFKNMGSPSKQQPQHQEQGGHYYQEHRPRDQGRNEVPHVRYQPPPEYGSFRS     357
NP_695231.3[M.musculus]        HRGPPPEYPFKGVPSQSVV--CKSQEPGHFYSEHRLNQPGRTEGQLMRYQHPPEYGAARA     293
Q4VCS5[H.sapiens]              HRGPPPEYPFKGMPPQSVV--CKPQEPGHFYSEHRLNQPGRTEGQLMRYQHPPEYGAARP     292
XP_009233461.1[P.abelii]       HRGPPPEYPFKGMPPQSVV--CKPQEPGHFYSEHRLNQPGRTEGQLMRYQHPPEYGAARP     292
XP_004940856.2[G.gallus]       QRGPPPEYPYKNVSTPSMN--CKPQDSGHFYSDHRVSQQGRSDGPMMRYQHPPEYGSVRQ     292
XP_018085358.1[X.laevis]       HRGPPPDYPFKNISTLPLQ--GKSKEVGFHYGEHRGSLQNRSDGPMVRYQPPPEYGASRQ     279
                                *****:**:*              :      .::    .  :   :*** ****.  *

XP_032827792.1[P.marinus]      TQQGM----QPMQMQYHSPSASQGSAGSLSIQMS-GAGALPG-------PMQNLGSMTLT     413
XP_014052684.1[S.salar]        NKESS--VHSQRSVHHHSPTSSVTSVGSLSRAQSSTLNSLLSASHPSHTPPHPQGDPYAS     415
NP_695231.3[M.musculus]        TQDISSLSLSARNSQPHSPTSSLTAGASSLPLLQSPPSTRLPPGQ--H-LVSNQGDHSAH     350
Q4VCS5[H.sapiens]              AQDIS-LPLSARNSQPHSPTSSLTSGG-SLPLLQSPPSTRLSPAR--HPLVPNQGDHSAH     348
XP_009233461.1[P.abelii]       AQDIS-LPLSARNSQPHSPTSSLTSGG-SLPLLQSPPSTRLSPAR--HPLVPNQGDHSAH     348
XP_004940856.2[G.gallus]       NPDVQ-LQLQQRPPHHHSPTSSLTSLG-SLTLLQSPPPSRLSPSQHQQPLTPSQGDPGIL     350
XP_018085358.1[X.laevis]       NQDVQ-QQIQHRPSHYHSPTSSLTSLG-SMSLLQSPTI--MSPSQ-QHPPSPSHGDFYTV     334
                                 :      .    : ***::*  : .      .                    *.

XP_032827792.1[P.marinus]      TA----------VAQQTPMY----------------------------------------     423
XP_014052684.1[S.salar]        TGPRS---PQGPGSHQGDLYCPGAIHPPLQRGHGFPHDPYGSTSRMHHQQQQHHQFQQQQ     472
NP_695231.3[M.musculus]        LS-RHQQHLLSSQSHQGDHYRHAQ---------------ASLT-----------------     377
Q4VCS5[H.sapiens]              LP-RPQQHFLPNQAHQGDHYRLSQ---------------PGLSQQQQQQQQQHHHHH---     389
XP_009233461.1[P.abelii]       LP-RPQQHFLPNQAHQGDHYRLSQ---------------PGLSQQQQQQQQQHH-HH---     388
XP_004940856.2[G.gallus]       PRT--QQPFLSNQVHQGDLYRLCQ---------------PFLG-----------------     376
XP_018085358.1[X.laevis]       PSRAHHIPVYNPVFQQGDVYRSPQ---------------SQFG-----------------     362
                                             :*   *

XP_032827792.1[P.marinus]      ----------------------------TGLGG-QLSMAHTAGPGEELHFMLLRSQQTVE     454
XP_014052684.1[S.salar]        QQHQQQQQQQQQQQQQQQQQQQLQHQQYHSQSSQGHPPQHYPNPQGDPFAMMARAQQMVD     532
NP_695231.3[M.musculus]        ------------------SAQQQPGEAYSAMPRAQQ-SASYQPMPADPFAMVSRAQQMVE     418
Q4VCS5[H.sapiens]              --H--------HQQQQQQQPQQQPGEAYSAMPRAQPSSASYQPVPADPFAIVSRAQQMVE     439
XP_009233461.1[P.abelii]       --H--------HHQQQQQQPQQQPGEAYSAMPRAQPSSASYQPVPADPFAIVSRAQQMVE     438
XP_004940856.2[G.gallus]       -------------------PQQQQGDSYSVMSRAQQIPSPYQQMQVDPFAIVSRAQQMVE     417
XP_018085358.1[X.laevis]       -------------------QQQQM---DPVLLRPPPVPSPYSQMQNDPFTIVSRAQNMVS     400
                                                                             : . :: *:*: *.

XP_032827792.1[P.marinus]      LLTEENQLLRSELESYCEKDIKLQKMEREVLRISEAYENLEKSTSKRETLEKAMRTKMES     514
XP_014052684.1[S.salar]        MLSEENRLLKQEMAVCSDKVNKLQKLETEITLVSEAYENLAKSSTKREALEKTMRNKLEL     592
NP_695231.3[M.musculus]        ILSDENRNLRQELDGCYEKVARLQKVETEIQRVSEAYENLVKSSSKREALEKAMRNKLEG     478
Q4VCS5[H.sapiens]              ILSDENRNLRQELEGCYEKVARLQKVETEIQRVSEAYENLVKSSSKREALEKAMRNKLEG     499
XP_009233461.1[P.abelii]       ILSDENRNLRQELEGCYEKVARLQKVETEIQRVSEAYENLVKSSSKREALEKAMRNKLEG     498
XP_004940856.2[G.gallus]       ILSEENRSLRQELDGCYEKVARLQKLETEIQQVSEAYKNLEKSSSKRDALEKAMRNKLEG     477
XP_018085358.1[X.laevis]       LLSEENRSLRKELEDCYDKVARFQKMEMEIQRISEAYENLVKSSSKRETLEKAMRTKLEE     460
                               :*::**: *:.*:    :*  ::**:* *:  :****:** **::**::***:**.*:*

XP_032827792.1[P.marinus]      EIRRLHDFNRDLRDRLETSNKQLASREYENSEDNKLTIAKLIAQNKETLLTKEKLEVELA     574
XP_014052684.1[S.salar]        EVRRLHDFNRDLRERMETANKQLAAKECEGSEDNRKTISQLLTQNKETVREKEKLEIELN     652
NP_695231.3[M.musculus]        EIRRMHDFNRDLRDRLETANKQLAEKEYEGSEDTRKTISQLFAKHKENQREKEKLEAELA     538
Q4VCS5[H.sapiens]              EIRRMHDFNRDLRERLETANKQLAEKEYEGSEDTRKTISQLFAKNKESQREKEKLEAELA     559
XP_009233461.1[P.abelii]       EIRRMHDFNRDLRERLETANKQLAEKEYEGSEDTRKTISQLFAKNKESQREKEKLEAELA     558
XP_004940856.2[G.gallus]       EIRRLHDFNRDLRERMETANKQLAEKEFEGSEDNRKTISQLFAQNKETQREKEKLEIELA     537
XP_018085358.1[X.laevis]       EIRRIHDFNRDLRERMDTANKQLAAKEFEGSEDNRKTISQLLAQNKEIQREKEKLEIEIS     520
                               *:**:********:*::*:***** :* *.***.: **::*:::**     ***** *:

XP_032827792.1[P.marinus]      GLRSANGDQRRHIELLNQAFSSSEAKAAKLQEELKKKQFYVDKVEKLQQALSQLQVACEK     634
```

```
XP_014052684.1[S.salar]        ALRSTTDDQRRHIEIRDQALNNAQAKVVKLEEELKKKQVYVEKVERMQQALAQLQAACEK     712
NP_695231.3[M.musculus]        TARSTNEDQRRHIEIRDQALSNAQAKVVKLEEELKKKQVYVDKVEKMQQALVQLQAACEK     598
Q4VCS5[H.sapiens]              TARSTNEDQRRHIEIRDQALSNAQAKVVKLEEELKKKQVYVDKVEKMQQALVQLQAACEK     619
XP_009233461.1[P.abelii]       TARSTNEDQRRHIEIRDQALSNAQAKVVKLEEELKKKQVYVDKVEKMQQALVQLQAACEK     618
XP_004940856.2[G.gallus]       AARSTNEDQRRHIEIRDQALNNAQAKVVKLEEELKKKQVYVEKVEKMQQALVQLQAACEK     597
XP_018085358.1[X.laevis]       SLRSTNQDQRRHIEIRDQALNNAQAKVVKLEEELKKKQVYVDKVEKMQQALVQLQAACEK     580
                                 **:. *******: :**:..::**..**:*******.**:***::**** ***.****

XP_032827792.1[P.marinus]      REQLERRLRTRLERELETLRAQQRQGAPPPGA-TSEHAAPALMELLREKEERVLALEADM     693
XP_014052684.1[S.salar]        REQLEHRLRTRLERELESLRMQQRQGVSQSSSSGQEYSATALMEHLREKEERILALEADM     772
NP_695231.3[M.musculus]        REQLEHRLRTRLERELESLRIQQRQGNSQPTN-ASEYNAAALMELLREKEERILALEADM     657
Q4VCS5[H.sapiens]              REQLEHRLRTRLERELESLRIQQRQGNCQPTN-VSEYNAAALMELLREKEERILALEADM     678
XP_009233461.1[P.abelii]       REQLEHRLRTRLERELESLRIQQRQGNCQPTN-VSEYNAAALMELLREKEERILALEADM     677
XP_004940856.2[G.gallus]       REQLEHRLRTRLERELESLRMQQRQGTSQAAN-VSEYNATALMELLREKEERILALEADM     656
XP_018085358.1[X.laevis]       REQLEHRLRTRLERELESLRIQQRQGNSQSSN-VSEYSAATLMELLREKEERVLALEADM     639
                               *****:***********:** *****        .*: * :*** *******:*******

XP_032827792.1[P.marinus]      MRWEQKYLEESAMRQFAMDAAATAATERDT--TIIANSPNASFS-----------EEEEF     740
XP_014052684.1[S.salar]        TKWEQKYLEESVMRQFALDAAASVATQRDTLSSVISHSPSSSYEQQLSVEARIQKEEEEI     832
NP_695231.3[M.musculus]        TKWEQKYLEENVMRHFALDAAATVAAQRDT--TVISHSPNTSYDT--ALEARIQKEEEEI     713
Q4VCS5[H.sapiens]              TKWEQKYLEENVMRHFALDAAATVAAQRDT--TVISHSPNTSYDT--ALEARIQKEEEEI     734
XP_009233461.1[P.abelii]       TKWEQKYLEENVMRHFALDAAATVAAQRDT--TVISHSPNTSYDT--ALEARIQKEEEEI     733
XP_004940856.2[G.gallus]       TKWEQKYLEESVMRQFALDAAATVAAQRDT--TVISHSPNPSYDT--SLEARIQKEEEEI     712
XP_018085358.1[X.laevis]       TKWEQKYLEESVMRQFALDAAATVATQRDT--ASISHSPSGSFDT--SLEARIQKEEEEI     695
                                :********..**:**:****:.*::***  : *::**. *:.           ****:

XP_032827792.1[P.marinus]      MLASRRCHDMEGRIKNLHAQIIEKDAMIKVLQQRSRKEQQGKTESSATSLRPAKSVPSIA     800
XP_014052684.1[S.salar]        LMANRRCLDMESRIKNLHAQIIEKDAMIKVLHQRSRKDP-NKDRDAQAIMRPSKSLMSIA     891
NP_695231.3[M.musculus]        LMANKRCLDMEGRIKTLHAQIIEKDAMIKVLQQRSRKEP-SKTE-QLSSMRPAKSLMSIS     771
Q4VCS5[H.sapiens]              LMANKRCLDMEGRIKTLHAQIIEKDAMIKVLQQRSRKEP-SKTE-QLSCMRPAKSLMSIS     792
XP_009233461.1[P.abelii]       LMANKRCLDMEGRIKTLHAQIIEKDAMIKVLQQRSRKEP-SKTE-QLSCMRPAKSLMSIS     791
XP_004940856.2[G.gallus]       LLANRRCLDMEGRIKTLHAQIIEKDAMIKVLQQRSRKEP-GKTD-QLSSMRPAKSLMSIS     770
XP_018085358.1[X.laevis]       LLANRRCVDMEGRIKTLHAQIIEKDAMIKVLQQRSRKDP-SKTD-QSSTMRPSKSLMSIS     753
                               ::*.:** ***.***.***************:*****:  .*     : :**:**: **:

XP_032827792.1[P.marinus]      AAASGSPGGGLASGLAARKPPMAASPSLEERLGA--RTGSFSGLVSTELAEEGSTASVGL     858
XP_014052684.1[S.salar]        NTG----G----SGLLSHSLGLSSSPITEE-RKDSSWKGSLGVLLGPEYRTDS----L--     936
NP_695231.3[M.musculus]        NA-----G----SGLLAHSSTLTGAPIMEEKRDDKSWKGSLGVLLGGDYRVEP----V--     816
Q4VCS5[H.sapiens]              NA-----G----SGLLSHSSTLTGSPIMEEKRDDKSWKGSLGILLGGDYRAEY----V--     837
XP_009233461.1[P.abelii]       NA-----G----SGLLSHSSTLTGSPIMEEKRDDKSWKGSLGILLGGDYRAEY----V--     836
XP_004940856.2[G.gallus]       NA-----G----SGLLSHSSTLSSTPILEEKREDKSWKGSLGVLLGTEYRSES----I--     815
XP_018085358.1[X.laevis]       NA-----G----SGLLSHSSALSSTPILEDKREDKSWKGSLGVLLATEYRSES----A--     798
                                :     *    *** ::.  ::.:*  *:       .**:. *:. :   :

XP_032827792.1[P.marinus]      SPMRALSPLPPPSPH-FTHVKSSSKDSSTQTERGAQEGSSSSPHMDPAAAERKKSNTLPR     917
XP_014052684.1[S.salar]        RTESISSSPSPVLPLTGGHSKTGSRDSCTQTDKGQSQGQEASSKPSTPAL---QSMTLPS     993
NP_695231.3[M.musculus]        PST--PSPVPPSTPLLSAHSKTGSRDCSTQTERGPESTKTAAVTPISAP--------MAG     866
Q4VCS5[H.sapiens]              PST--PSPVPPSTPLLSAHSKTGSRDCSTQTERGTESNKTAAVAPISVP--------AP-     886
XP_009233461.1[P.abelii]       PST--PSPVPPSTPLLSAHSKTGSRDCSTQTERGTESNKTAAVAPISVP--------AP-     885
XP_004940856.2[G.gallus]       PST--PSPVLPSTPLLSAHSKTGSRDCSTQTDRGNEQSKAAPSPAA--P--------TPG     863
XP_018085358.1[X.laevis]       SST--PSPIRPSTPLLCGHSKTGSKDCCTQTDRGAEQNKPPSVSAS--P--------GPG     846
                                     *   *  *    * *:.*:*..***::* .. .

XP_032827792.1[P.marinus]      GSKM-LPSLHVLRVDARDRA-------DMKMAAGE-------------------------     944
XP_014052684.1[S.salar]        RLSS--------------------------------------------------------     997
NP_695231.3[M.musculus]        PVAAAAPAAAINATAATNTATAATNTTIMVAAAPVAVAAVAAPAAAAA---TPSPANAAA     923
Q4VCS5[H.sapiens]              -VAAAATAAAI-------TATAATITTTMVAAAPVAVAAAAAPAA----AAAPSPATAAA     934
XP_009233461.1[P.abelii]       -VAAAATAAAI-------TATAATITTTMVAAAPVAVAAAAAPAAAAAAAAAPSPATAAA     937
XP_004940856.2[G.gallus]       RLPT--------------------------------------------------------     867
XP_018085358.1[X.laevis]       RLAT--------------------------------------------------------     850

XP_032827792.1[P.marinus]      ------------------------------------------------------------     944
XP_014052684.1[S.salar]        ------------------------------------------------------------     997
NP_695231.3[M.musculus]        LAAAAAPATSVSAATSVSAANSISPAAPVAPAAVVPPAAPVSPAAAVQIPAAASLTPATV     983
Q4VCS5[H.sapiens]              TA------------------------------------AAVSPAAAGQIPAAASVAS---     955
XP_009233461.1[P.abelii]       IA------------------------------------AAVSPAAAGQIPAAASVAS---     958
XP_004940856.2[G.gallus]       ------------------------------------------------------------     867
XP_018085358.1[X.laevis]       ------------------------------------------------------------     850

XP_032827792.1[P.marinus]      ------------------------------------------------------------     944
XP_014052684.1[S.salar]        ------------------------------------------------------------     997
NP_695231.3[M.musculus]        SPTAATATAAVAAATTAAITAAAAAATTAIQVAPATSAPVPSPASIPA--PATAQASAPT     1041
Q4VCS5[H.sapiens]              ---------------AAAVAPS-AAAAAAVQVAPAAPAPVPAPALVPVPAPAAAQASAPA     999
XP_009233461.1[P.abelii]       ---------------VAAVAPPPAAAAAAVQVAPAAPAPVPAPALVPVPAPAAAQASAPA     1003
XP_004940856.2[G.gallus]       ------------------------------------------------------------     867
XP_018085358.1[X.laevis]       ------------------------------------------------------------     850

XP_032827792.1[P.marinus]      ------------------------------------------------------------     944
XP_014052684.1[S.salar]        -------------------------------------------PSPVYIPDRIADVPVFH     1014
NP_695231.3[M.musculus]        PTQASTPAPTEPPSPVPTPTPALVQTEGPANPGASSGPRRLSTPNLMCNPD-KPDAPAFH     1100
Q4VCS5[H.sapiens]              QTQAPTSAPAVAPTPAPTPTPAVAQAEVPASPATGPGPHRLSIPSLTCNPD-KTDGPVFH     1058
XP_009233461.1[P.abelii]       QTQTPTSAPAVAPTPAPTPTPAVAQAEVPASPATGPGPHRLSVPSLTCNPD-KTDGPVFH     1062
XP_004940856.2[G.gallus]       -------------------------------------------ASLPYSAD-KTDGPLFH     883
XP_018085358.1[X.laevis]       -------------------------------------------PSPMYNSE-RTAETVFQ     866

XP_032827792.1[P.marinus]      -KLLDGRPPLHMSGLPQ---EDQVDNEMVEILI        973
XP_014052684.1[S.salar]        SSTLERRAPVQSHPQPPSASQQELDNDMVEVLI        1047
NP_695231.3[M.musculus]        SSTLERKTPIQILGQ------E-PDAEMVEYLI        1126
Q4VCS5[H.sapiens]              SNTLERKTPIQILGQ------E-PDAEMVEYLI        1084
XP_009233461.1[P.abelii]       SNTLERKTPIQILGQ------E-PDAEMVEYLI        1088
XP_004940856.2[G.gallus]       SSTLERKAPVQTMGQ------DLPDGEMVEYLI        910
XP_018085358.1[X.laevis]       SSTLDRKNPLPPLGQ------DLTDGELVEYLI        893
                                . *: : *:           :  * ::** **
```
