# Supplementary material for: An Evolutionary Perspective on the Origin, Conservation and Binding Partner Acquisition of Tankyrases
Source: Biomolecules. 2022 Nov 15;12(11):1688. doi: 10.3390/biom12111688 (PMC9688111; doi:10.3390/biom12111688)
Supplement: Supplementary file 1 [file biomolecules-12-01688-s001.zip › biomolecules-1913719-supplementary-11.17 -done/Data_7/Arpin.pdf]

Arpin

Homo sapiens:

>NP_872422.1 arpin isoform 1 [Homo sapiens]
MSRIYHDGALRNKAVQSVRLPGAWDPAAHQGGNGVLLEGELIDVSRHSILDTHGRKERYYVLYIRPSHIH
RRKFDAKGNEIEPNFSATRKVNTGFLMSSYKVEAKGDTDRLTPEALKGLVNKPELLALTESLTPDHTVAF
WMPESEMEVMELELGAGVRLKTRGDGPFLDSLAKLEAGTVTKCNFTGDGKTGASWTDNIMAQKCSKGAAA
EIREQGDGAEDEEWDD

Pongo abelii:

>XP_024088103.1 arpin isoform X2 [Pongo abelii]
MSRIYHDGALRNKAVQSVRLPGAWDPAAHQGGNGVLLEGELIDVSRHSILDAHGRKERYYVLYILPSHVH
RRKFDAKGNEIEPNFSATRRVNTGFLMSSYKVEAKGDTDRLTPEALKGLVNKPELLALTESLTPDHTVAF
WMPESEMEAMELELGAGVRLKTRGDGPFLDSLAKLEAGTVTKCNFAGDGKTGASWTDNIMAQKCSEGAAA
EIREQGDGAEDEEWDD

Mus musculus:

>NP_081696.1 arpin [Mus musculus]
MSRIYQDSALRNKAVQSARLPGTWDPATHQGGNGILLEGELVDVSRHSILDAHGRKERYYVLYIQPSCIH
RRKFDPKGNEIEPNFSATRKVNTGFLMSSYKVEAKGDTDRLTLEALKSLVNKPQLLELTESLTPDQAVAF
WMPESEMEVMELELGTGVRLKTRGDGPFIDSLAKLELGTVTKCNFAGDGKTGASWTDNIMAQKSSERNTA
EIREQGDGAEDEEWDD

Gallus gallus:

Not found

Xenopus laevis:

>NP_001087754.1 arpin [Xenopus laevis]
MSRIYQHTALQNKPVHDERFDGSWEPGAFQRGTGVLLEGTLLDFSRHAVTDSKGKKERWYILYLRPSKIH
RRHFDSKGNEIEPNFSDTKKVNTGFLMSSYKVEAKGESDRISLEELNRLVNKVNLMKISEKHTPRETVAF
WLPEADMEKTELELGEQLRVKTMGDGPFLFSLAKVDSGTVTKCNFAGDAQAGASWTDNIMERKSQNTSAP
SEPRGQGDGAEDDEWDD

Salmo salar:

>XP_013979611.1 arpin isoform X3 [Salmo salar]
MSRIYNNTSLQNKPVHNEKIEHAWAPSSYQSGLGVILEGKLVDVSRHVITDVNNQKDRFYVLYIKPSRIH
RRKFDAKGMEIEPNFSDTKKVNTGYLMSSFKVEAKGETDCLTEVQLENLVTKEDLVKVTGKHCLSGTYAF
WYPEGEMSKTELETGQDIRLKTKGDGPFIFSLAKVDGGTVTKCNFAGDKEAGASWTDKIMSNKSDASSAT
KSEGHGEGADEEEWDD

Petromyzon marinus:

>XP_032824636.1 arpin [Petromyzon marinus]
MSRIYDNKKLEARPLHSETWPRPWEPSAFEQGSGVLLEGILGEISRHVILDSAGAKHRYSVLHVRPERAH
RRCWDTGGRELEPNFSETRLVSTGHLFSSYKCEAKGETDRLSESELRRAVAQAKLDSVVGRHGPQGTLPF
WVAEDDVKKTELELGDRIRIKTRGDSPFVFSLARVDAGTVTRCNFAGDAAAGASWTDKILGVKSGLDAPP
PGASGDGEGAADDEWD

Branchiostoma belcheri:

>XP_019625759.1 PREDICTED: arpin-like isoform X1 [Branchiostoma belcheri]
MLYSTVGRTASGINFRCRAEEGEEEQQNTDRPEPKHTEKTEQQPTIAIMSRLYDNQPLMSIPVQTHQLTT
VWNPSNYQKGAGVLVEGRIEGRSRHVIIDPSQTKFRYCVLHVHPTQAHRRKFDAAGKEVEPNFSDTWKVN
TGYLHSSYKVEARGQTDRLTTEELEAVVMSAELDHLTQQVKRHGCLSFWVQEEELAGMELNTGDNVRLKT
RGDGLFIYNVAQLDGATSQVSNFAGGNMVGASWTEKVMAVKRDQQEQRDEENQGVDEDEWDD

Drosophila melanogaster:

Not found

Hydra vulgaris:

Not found

Nematostella vectensis:

Not found

Amphimedon queenslandica:

>XP_019858664.1 PREDICTED: arpin-like [Amphimedon queenslandica]
MSRMHMIYDDTLLQQEGVKVSKWKSEWNSSQLQSGRGILLEGVFHKKTRHIIQDTSNKKYRFCVFHIEFD
GACQRKFDAEGKEIEPNFGQTAKVKTGYLNSSYKTVAKGTPDTLSYAEVCERVHSDLVGFETASHSQSIC
FWGENESYDHVECSPGQRIRLKTRGDSLLIESFTKLDSDQRAHSSNYTGGEKTGQSWTDKVLGHKSTDEP
SQGTGGGGGGEVDDDEWVRIFLSYVLVHCELHLLLF

Alignment

```
XP_019858664.1[A.queenslandica]      ---------------------------------------------MSRMHMIYDDTLLQQ   15
XP_019625759.1[B.belcheri]           MLYSTVGRTASGINFRCRAEEGEEEQQNTDRPEPKHTEKTEQQPTIAIMSRLYDNQPLMS   60
XP_032824636.1[P.marinus]            ------------------------------------------------MSRIYDNKKLEA   12
XP_013979611.1[S.salar]              ------------------------------------------------MSRIYNNTSLQN   12
NP_001087754.1[X.laevis]             ------------------------------------------------MSRIYQHTALQN   12
NP_872422.1[H.sapiens]               ------------------------------------------------MSRIYHDGALRN   12
XP_024088103.1[P.abelii]             ------------------------------------------------MSRIYHDGALRN   12
NP_081696.1[M.musculus]              ------------------------------------------------MSRIYQDSALRN   12
                                                                                     *  :*..  *

XP_019858664.1[A.queenslandica]      EGVKVSKWKSEWNSSQLQSGRGILLEGVFHKKTRHIIQDTSNKKYRFCVFHIEFDGACQR   75
XP_019625759.1[B.belcheri]           IPVQTHQLTTVWNPSNYQKGAGVLVEGRIEGRSRHVIIDPSQTKFRYCVLHVHPTQAHRR   120
XP_032824636.1[P.marinus]            RPLHSETWPRPWEPSAFEQGSGVLLEGILGEISRHVILDSAGAKHRYSVLHVRPERAHRR   72
XP_013979611.1[S.salar]              KPVHNEKIEHAWAPSSYQSGLGVILEGKLVDVSRHVITDVNNQKDRFYVLYIKPSRIHRR   72
NP_001087754.1[X.laevis]             KPVHDERFDGSWEPGAFQRGTGVLLEGTLLDFSRHAVTDSKGKKERWYILYLRPSKIHRR   72
NP_872422.1[H.sapiens]               KAVQSVRLPGAWDPAAHQGGNGVLLEGELIDVSRHSILDTHGRKERYYVLYIRPSHIHRR   72
XP_024088103.1[P.abelii]             KAVQSVRLPGAWDPAAHQGGNGVLLEGELIDVSRHSILDAHGRKERYYVLYILPSHVHRR   72
NP_081696.1[M.musculus]              KAVQSARLPGTWDPATHQGGNGILLEGELVDVSRHSILDAHGRKERYYVLYIQPSCIHRR   72
                                       ::       *  .  : * *:::** :   :** : *    * *: ::::      :*

XP_019858664.1[A.queenslandica]      KFDAEGKEIEPNFGQTAKVKTGYLNSSYKTVAKGTPDTLSYAEVCERVHSDLVGFE--TA   133
XP_019625759.1[B.belcheri]           KFDAAGKEVEPNFSDTWKVNTGYLHSSYKVEARGQTDRLTTEELEAVVMSAELDHLTQQV   180
XP_032824636.1[P.marinus]            CWDTGGRELEPNFSETRLVSTGHLFSSYKCEAKGETDRLSESELRRAVAQAKLDSVVGRH   132
XP_013979611.1[S.salar]              KFDAKGMEIEPNFSDTKKVNTGYLMSSFKVEAKGETDCLTEVQLENLVTKEDLVKVTGKH   132
NP_001087754.1[X.laevis]             HFDSKGNEIEPNFSDTKKVNTGFLMSSYKVEAKGESDRISLEELNRLVNKVNLMKISEKH   132
NP_872422.1[H.sapiens]               KFDAKGNEIEPNFSATRKVNTGFLMSSYKVEAKGDTDRLTPEALKGLVNKPELLALTESL   132
XP_024088103.1[P.abelii]             KFDAKGNEIEPNFSATRRVNTGFLMSSYKVEAKGDTDRLTPEALKGLVNKPELLALTESL   132
NP_081696.1[M.musculus]              KFDPKGNEIEPNFSATRKVNTGFLMSSYKVEAKGDTDRLTLEALKSLVNKPQLLELTESL   132
                                      :*  * *:****. *  *.**.* **:*  *:*  * ::   :   * .  :

XP_019858664.1[A.queenslandica]      SHSQSICFWGENESYDHVECSPGQRIRLKTRGDSLLIESFTKLDSDQRAHSSNYTGGEKT   193
XP_019625759.1[B.belcheri]           KRHGCLSFWVQEEELAGMELNTGDNVRLKTRGDGLFIYNVAQLDGAT-SQVSNFAGGNMV   239
XP_032824636.1[P.marinus]            GPQGTLPFWVAEDDVKKTELELGDRIRIKTRGDSPFVFSLARVDAGT-VTRCNFAGDAAA   191
XP_013979611.1[S.salar]              CLSGTYAFWYPEGEMSKTELETGQDIRLKTKGDGPFIFSLAKVDGGT-VTKCNFAGDKEA   191
NP_001087754.1[X.laevis]             TPRETVAFWLPEADMEKTELELGEQLRVKTMGDGPFLFSLAKVDSGT-VTKCNFAGDAQA   191
NP_872422.1[H.sapiens]               TPDHTVAFWMPESEMEVMELELGAGVRLKTRGDGPFLDSLAKLEAGT-VTKCNFTGDGKT   191
XP_024088103.1[P.abelii]             TPDHTVAFWMPESEMEAMELELGAGVRLKTRGDGPFLDSLAKLEAGT-VTKCNFAGDGKT   191
NP_081696.1[M.musculus]              TPDQAVAFWMPESEMEVMELELGTGVRLKTRGDGPFIDSLAKLELGT-VTKCNFAGDGKT   191
                                            **  : .    * . *  :*:** **. :: ..::::       .*::*.  .

XP_019858664.1[A.queenslandica]      GQSWTDKVLGHKSTDEPSQGTGGGGGGEVDDDEWVRIFLSYVLVHCELHLLLF          246
XP_019625759.1[B.belcheri]           GASWTEKVMAVKRDQQE---QRDEENQGVDEDEWDD-----------------          272
XP_032824636.1[P.marinus]            GASWTDKILGVKSGLDAPPPGASGDGEGAADDEWD------------------          226
XP_013979611.1[S.salar]              GASWTDKIMSNKSDAS-SATKSEGHGEGADEEEWDD-----------------          226
NP_001087754.1[X.laevis]             GASWTDNIMERKSQNTSAPSEPRGQGDGAEDDEWDD-----------------          227
NP_872422.1[H.sapiens]               GASWTDNIMAQKCSKG-AAAEIREQGDGAEDEEWDD-----------------          226
XP_024088103.1[P.abelii]             GASWTDNIMAQKCSEG-AAAEIREQGDGAEDEEWDD-----------------          226
NP_081696.1[M.musculus]              GASWTDNIMAQKSSER-NTAEIREQGDGAEDEEWDD-----------------          226
                                     * ***::::  *             .  . ::**
```
