# Supplementary material for: An Evolutionary Perspective on the Origin, Conservation and Binding Partner Acquisition of Tankyrases
Source: Biomolecules. 2022 Nov 15;12(11):1688. doi: 10.3390/biom12111688 (PMC9688111; doi:10.3390/biom12111688)
Supplement: Supplementary file 1 [file biomolecules-12-01688-s001.zip › biomolecules-1913719-supplementary-11.17 -done/Data_7/ATG9A.pdf]

ATG9A

Homo sapiens:

>NP_001070666.1 autophagy-related protein 9A [Homo sapiens]
MAQFDTEYQRLEASYSDSPPGEEDLLVHVAEGSKSPWHHIENLDLFFSRVYNLHQKNGFTCMLIGEIFEL
MQFLFVVAFTTFLVSCVDYDILFANKMVNHSLHPTEPVKVTLPDAFLPAQVCSARIQENGSLITILVIAG
VFWIHRLIKFIYNICCYWEIHSFYLHALRIPMSALPYCTWQEVQARIVQTQKEHQICIHKRELTELDIYH
RILRFQNYMVALVNKSLLPLRFRLPGLGEAVFFTRGLKYNFELILFWGPGSLFLNEWSLKAEYKRGGQRL
ELAQRLSNRILWIGIANFLLCPLILIWQILYAFFSYAEVLKREPGALGARCWSLYGRCYLRHFNELEHEL
QSRLNRGYKPASKYMNCFLSPLLTLLAKNGAFFAGSILAVLIALTIYDEDVLAVEHVLTTVTLLGVTVTV
CRSFIPDQHMVFCPEQLLRVILAHIHYMPDHWQGNAHRSQTRDEFAQLFQYKAVFILEELLSPIVTPLIL
IFCLRPRALEIIDFFRNFTVEVVGVGDTCSFAQMDVRQHGHPQWLSAGQTEASVYQQAEDGKTELSLMHF
AITNPGWQPPRESTAFLGFLKEQVQRDGAAASLAQGGLLPENALFTSIQSLQSESEPLSLIANVVAGSSC
RGPPLPRDLQGSRHRAEVASALRSFSPLQPGQAPTGRAHSTMTGSGVDARTASSGSSVWEGQLQSLVLSE
YASTEMSLHALYMHQLHKQQAQAEPERHVWHRRESDESGESAPDEGGEGARAPQSIPRSASYPCAAPRPG
APETTALHGGFQRRYGGITDPGTVPRVPSHFSRLPLGGWAEDGQSASRHPEPVPEEGSEDELPPQVHKV

Pongo abelii:

>NP_001127287.1 autophagy-related protein 9A [Pongo abelii]
MAQFDTEYQRLEASYSDSPPGEEDLLVHVAEGSKSPWHHIENLDLFFSRVYNLHQKNGFTCMLIGEIFEL
MQFLFVVAFTTFLVSCVDYDILFANKMVNHSLHPTEPVKVTLPDAFLPAQVCSARIQENGSLITILVIAG
VFWIHRLIKFIYNICCYWEIHSFYLHALRIPMSALPYCTWQEVQARIVQTQKEHQICIHKRELTELDIYH
RILRFQNYMVALVNKSLLPLRFRLPGLGEAVFFTRGLKYNFELILFWGPGSLFLNEWSLKAEYKRGGQRL
ELAQRLSNRILWIGIANFLLCPLILIWQILYAFFSYAEVLKREPGALGARCWSLYGRCYLRHFNELEHEL
QSRLNRGYKPASKYMNCFLSPLLTLLAKNGAFFAGSILAVLIALTIYDEDVLAVEHVLTTVTLLGVTVTV
CRSFIPDQHMVFCPEQLLRVILAHIHYMPDHWQGNAHRSQTRDEFAQLFQYKAVFILEELLSPIVTPLIL
IFCLRPRALEIIDFFRNFTVEVVGVGDTCSFAQMDVRQHGHPQWLSAGQTEASVYQQAEDGKTELSLMHF
AITNPGWQPPRESTAFLGFLKEQVQRDGAAASLAQGGLLPENALFTSIQSLQSESEPLSLIANVVAGSSC
RGPPLPRDLQGSRHRAEVASALRSFSPLQPGQAPTGRAHSTMTGSGVDARTASSGSSVWEGQLQSLVLSE
YASTEMSLHALYMHQLHKQQAQAEPERHLWHRRESDESGESAPDEGGEGARAPQSIPRSASYPCAAPRPG
APETTALHGGFQRRYGGITDPGTVPRAPSHFSRLPLGGWAEDGQSASRHPEPVPEEGSEDELPPQVHKV

Mus musculus:

>NP_001003917.2 autophagy-related protein 9A isoform a [Mus musculus]
MAQFDTEYQRLEASYSDSPPGEEDLLVHVAEGSKSPWHHIENLDLFFSRVYNLHQKNGFTCMLIGEMFEL
MQFLFVVAFTTFLVSCVDYDILFANKMVNHSLHPTEPVKVTLPDAFLPAQVCSARIQENGSLITILVIAG
VFWIHRLIKFIYNICCYWEIHSFYLHALRIPMSALPYCTWQEVQARIVQTQKEHQICIHKRELTELDIYH
RILRFQNYMVALVNKSLLPLRFRLPGLGEVVFFTRGLKYNFELILFWGPGSLFLNEWSLKAEYKRGGQRL
ELAQRLSNRILWIGIANFLLCPLILIWQILYAFFSYAEVLKREPGALGARCWSLYGRCYLRHFNELEHEL
QSRLNRGYKPASKYMNCFLSPLLTLLAKNGAFFAGSILAVLIALTIYDEDVLAVEHVLTTVTLLGVTVTV
CRSFIPDQHMVFCPEQLLRVILAHIHYMPDHWQGNAHRSQTRDEFAQLFQYKAVFILEELLSPIVTPLIL
IFCLRPRALEIIDFFRNFTVEVVGVGDTCSFAQMDVRQHGHPQWLSGGQTEASVYQQAEDGKTELSLMHF
AITNPGWQPPRESTAFLGFLKEQVQRDGAAAGLAQGGLLPENALFTSIQSLQSESEPLSLIANVVAGSSC
RGPSLSRDLQGSRHRADVASALRSFSPLQPGAAPQGRVPSTMTGSGVDARTASSGSSVWEGQLQSLVLSE
YASTEMSLHALYMHQLHKQQTQAEPERHVWHRRESDESGESAPEEGGEGARAPQPIPRSASYPCATPRPG
APETTALHGGFQRRYGGITDPGTVPRGPSHFSRLPLGGWAEDGQPASRHPEPVPEEGSEDELPPQVHKV

Gallus gallus:

>NP_001029993.2 autophagy-related protein 9A [Gallus gallus]
MAHLETQYQRLESSSTESPPGGGDLLVHVPEGAKSPWHHIENLDLFFSRVYNLHQKNGFTCMLIGEIFEL
MQFIFVVAFTTFLISCVDYDILFANKAVNHSQHPSEPIKVTLPDAFLPPNVCSARIQANSFLICILVIAG
VFWIHRLVKFIYNICCYWEIHSFYINALRIPMSNLPYYTWQEVQARIVQIQKEHQICIHKKELTELDIYH
RILRFKNYMVAMVNKSLLPIRFRLPLLGDTVFYTRGLKYNFELIFFWGPGSLFENEWSLKAEYKRAGNRL
ELAEKLSTRILWIGIANFLLCPLILIWQILYAFFSYTEILKREPGSLGARCWSLYGRCYLRHFNELDHEL
QSRLSKGYKPASKYMNCFISPLLTIVAKNVAFFAGSILAVLIALTIYDEDVLAVEHVLTTVTLLGVGITV
CRSFIPDQHLVFCPEQLLRVILAHIHYMPDHWQGNAHRYETRDEFAQLFQYKAVFILEELLSPIITPLIL
IICLRPKSLDIVDFFRNFTVEVVGVGDTCSFAQMDVRQHGHPAWMSAGKTEASIYQQAEDGKTELSLMHF
AITNPKWQPPRESTAFIGFLKERVHRDSSVALAQQAVLPENALFSSIQSLQSESEPHSLIANVIAGSSVL
GFHMGRDGQASRHLSEVASALRSFSPLQSAQQPSGGFQTAGRDGEGTQPRGSSAMTASGADARTMSSGSS
AWEGQLQSMILSEYASTEMSLHALYMHELHKQHAQLEPERHTWHRRESDESGESTHEELDAQRGAPVPLP
RSASYPFSSRQPAEETATLQTGFQRRYGGITDPGTVHRAPSHFSRLPLGGWAEDGQSARHPEPVPEESSE
DELPPQIHKV

Xenopus laevis:

>XP_018094208.1 autophagy-related protein 9A [Xenopus laevis]
MAMYDTPYQRLEASYTDSPLGEDDLLVHVPEGSKSPWHHIENLDLFFSHVYNLHQKNGFTCMLIGEIFEL
LQFIFIVSFTTLLVSCVDYDILFANKMVNHSQSDHVKVTLPDAFLPPGVCRDRMQENGFLVCLLAIAGVF
WIHRLIKFIYNICCYWEIHNFYMQALRIPMSDLPYYTWEEVQSRIVQIQKEHQICIHKKELSELDVSHRI
LRFKNYMVAMVNKNLLPLQHRLPLLGNTVFYTRGLKYNFQLIFFWGPGCLFQNEWSLKPEYKRSGARLEL
SEKLAQRILWIGLANLVLCPLVLVWQILYAFFSYTEVLRREPGSLGARCWSLYGRCYLRHFNELDHQLQA
RLSRAYKPASKYMNCFLSPLLAVVAKHFGFLAGSILAVLIALTVYDEDVLAVEHVLSAVTLLGIVVTICR
SFIPDQHLVFCPEQLLRVILAHIHYMPDHWQRSAHRAETRGEFSQLFQYKAVFILEELLSPLLTPLVLIF

CLRPKSLEIIDFFRNFTVEVVGVGDTCSFAQMDVRQHGNPAWMSAGKTEASIYQQAEDGKTELSLMHFAI
TNPRWQPPRECSAFLSQLRERVQRDSTMGNQKHPTGLMQPLQCSGLSIQSDPEPHSLMANMFSGVSTAGF
PANTEALGSPHVSAVSEVASALHSLSPQQSCHPVQSQSGISADFRNGSFGSNVWEGPMNSGTLSEYASTE
MSLHALYMHELHQQHTRMAPGRHIWHRQESDESGESSQGEGLINTTSNVPSSLSCPSPAAAVWEERLPPL
STHRLSASTDLGSVQRTSLARQPMGGWSEDVQPSRLPEPVPEEGSEDELPPQVQKV

Salmo salar:

>XP_014005943.1 autophagy-related protein 9A [Salmo salar]
MAAHFDTEYQRLEASYSDSPPGEENLLVHIPDGNKSPWHHIENLDLFFQRVYNLHQKNGFTCMLLGEVFE
LVQLLFVVGFTVFLANCVDYDILFANKFVNHTDSSKVTLPDAFLPVDVCSASIRDNVAVMFVLMISGVFW
LHRLIKFIYNVCCYWEIRSFYTNALKMSMAELPYFTWQEVQARIVEIQKEHQICIHKKELTELDIYHRIL
RFKNYMVAMINKSLLPVRFRPPLLGESVFYTRGLKYNFELIFFWGPGSLFESEWSLKPQYKRGGNRLELA
DKLASRILWIGIANLVLCPVVLVWQILYAFFSYTEVIKREPGSLGARCWSLYGRCYLRHFNELDHELMSR
LSKGYKASSRYMNCFLSPLLTVVAKNVAFFAGSLLAVLIALTIYDEDVLAVEHVLSSITLLGVCITVCRS
FIPDKHLVYCPEQLLRVILAHIHYMPDHWQGNAHRYETRDQFSQLFQYKAVFILEELISPLVTPFILIFS
LRRKSLEIIDFFRNFSVEVVGVGDTCSFAQMDIRQHGHPAWMSEGKTEASIYQQAEDGKTELSLMHFAIT
NPQWQPPTETTHFISQLKERVQREATGDRHTLTSLSGSEPRSLIANFLAGPPSLASLHLGRDGSLTNHGP
VGVSDGASALRSLSISSMRGSCSSAHRSAGHASSASRAMPGSSTDARTASSGSSVWEGQLTSLVLSEYAS
TEMSVHALYIHELHKQQSGGELSRHTWHRQDSDDSSDSVTEEGGSGGGNPNPRNPIPRSHTFPISTPITT
PGPIPSSNSGPNPDRGTTPGQDVTPLQSNSQRRYGGNTDSVGPEGRVVRSARVPMGGWAEEGRGGRHPDT
VPEEGSEDDMPPNIHKVT

Petromyzon marinus:

>XP_032801920.1 autophagy-related protein 9A-like isoform X1 [Petromyzon marinus]
MAEAETGYRRLDSYDDEESPNHEREVLVHVPEGSKARWNHIENLDYFFTRIYHFHQKHGFACMMLAEFFE
LVQFLFVVTFTTFLINCVDYDILFANKQVHPQPSRKVTLYDALLSSEKCTERIRGNSWIIFLIVMAGTFW
LYRLIKVIYNFSSYLEIRAFYINALKIPMAELQNFSWQEVQERVLAVQKEQQMCIHKRELTELDMHHRIL
RFKNYLVAMVNKSLLPVRVRVPFAGESALLTQGLKYNLEMLLFWGPGAPFQSAWSLRPEYKRAGCRLELA
ERLGRTILLVGLANLVLCPLVVVWQVLHAFFRYAEVLKREPGSLGARRWSLYGQLYLRHLNELDHELRAR
LGRGYRHAARYMDSFTAPMMAVLAKNVTFFAGSLLAVLLALTVYDEDVLSVEHVLTAITALGVLLTVCRA
FIPDEHLVWNPEHLLQAVLAHIHYMPEHWKGIAHKTETRDEFSQLFQYKAAFIMEELLSPIITPFILIFG
VRPKALEIVDFFRNFTVEVVGVGDVCSFAQMDIRRHGNPTWMSAGQTEASYYQQAEDGKTELSLVHFAIS
NPRWRPPRGSNVYLNQLREQVQRDSTANPQPLLFSSLQTIESQSGPYGLLGSMLGGPSSLTAYRSTRDAT
CSLPEMSAATAAATALMSLSMPSASLTPGPREELAEMPDSTPGNTTVTARPGSSTGAVGGMSMSTGGRGK
VSFGGTDFRSSSVGSSFREGPSQSSILSEFASAEMSLHAIYMHEARRNYATSDAQARYHWQQPENNGDDD
TMDEVNALSGFRESSLQLHNTGSNSGSHSSSVLGSGQRETYVSPSASIGHSGDYMPPNIGSLQDTHGDSL
GLMVTRENTELGAFPFRHYGGTSASENMLQPEQGSPRFGLMPMGGWQEDMAGLGTIDPHGGVPSCMSPNP
QQINEVAYTFHSLFLNT

Branchiostoma belcheri:

>XP_019613418.1 PREDICTED: autophagy-related protein 9A-like isoform X1 [Branchiostoma belcheri]
MAQLETSYRRLDSFYDEEMDSPPNERELLVHAVDSNKAHRWNHIENLDDFFTRVYQYHQRHGFLCMMLAE
VLELLQFIFVLLFTTFLLQCVHYEVLFGDKNPHPDRKVTLPEALVPLDQCASSLDSRIVVCLVLASFFWF
LRFIKVVYNFFRYWEIRTFYTQALKISHNDLPNMTWHEVQRCLRQVQVEQQMCIHKQELTELDIYHRILR
FKNYTVAMVNKSLLPLKFQLPGMGELVFLTNGLKYNLEMILFWGPWSPFANNWHLKEDYKKSSKRLELAN
KLSRHILWVGVANLLLCPFILMWQILYSFFTYAEVLKRQPGSLGARKWSLYGRLYLRHFNELDHELNARL
TRAYKPAEKYMSSFISHVMTLIARHVAFLAGAIFAVLVVLTIYDEDVLTIEHVLTITTLMGVLVAGCRAL
IPDENMVFCPEQLMVCILAQIHYMPDRWKGQAHTYRVRDEFSQLFQYKAVFLLEELLSPIITPLILIFSL
RSKALDIVDFFRNFTVDVVGVGDVCSFAQMDIKRHGNPQWTGEKQQTDVSQYHQAEDGKTELSLMHFTLT
NPTWQPPRHCNKFINHLKEQAHKDVNMLNSAVMVDNPFFQSLQSLSSAGYPSMMTSLMVGRLGPDATLVP
PRDPGTGTEPSQPGVALPGTTAPGRYGLVRGDISSVEGPIQAPNTGLLGSSTMAPRGTASISHTLPPTPE
PVSLQETMAPTELMAAEMSFSALYMHQLHQTQTHHDHQDQEAQRAWRQPLPPAFYGTTMPANQQRPSPGS
VEDSRTSEPGQWHSVGSTTMPGIDEAPSEETDTLSYSPPNV

Drosophila melanogaster:

>NP_611114.1 Autophagy-related 9, isoform A [Drosophila melanogaster]
MSSPHINYRSLAEEAASPFLEHHPSTGQGPSKTQDAKANAAAAHLDPLGEHGLEQPLDEHDTEHEGEDTP
RNSGVMIHMVPETGRARWNHIEDLDSFFSRMYQYQQKHGFTVIVVDEMLQVLEFGFVVWLLAFVMHCVRF
DVLFGDTPPGGLNPNKTTLSDVMYPTGECLANFTWVTYLVVFIAAIYLGIRLLKMVYHITQYADIKRFYN
SALHIEDSDLDNFTWHEVQQRIRRVQAEQHMCIDKESLTELDIYHRVLRFKNYLVALMNKQLLPVRFHIP
LYGEVVSLSRGMLFNIDFILFRGPGSPFQNNWQLRDEFAVRSNQTELAQRLSKLILGVALLNLVLAPVIF
VWQLIYFSFSYANILRKEPGALGLRTWSNYGRLYLRHFNELDHELDARLNRAYDYADRYLNSFSSPLAAV
IAKNLLFISGGLLLLILALGIYEEHVFQVEHLLAILAGLGAIGVVCRTLIPDENLVWCPEQLMTAILAHV
HYLPSEWRQQAHTTKVRQEFSNFFQFKAGYLLSEIFSPFVTPFVLIFVFRPKAIELVRFFRTFTVSVRGV
GNVCSFAQMDVRKHGNPDWQLTSELEEMTRATAQQPQQEPQQQSLAGGKTEMSLLRFTLNNPEWQMPKEA
KQFLRGVREHAVGELVQAKTSMVQENPLTNSLISFGTMGADYCSIANSVLTAQVTPQQLEISQSLRPGLG
PVSGGFPVAASDFRQMLQQNLSASVGPLDSMRRLRLSRAEGRLEGPTDTLLYGLCGVDPRVGSTPLNVGV
ADMCLSALYLHELNQQKRQARQSRIDEAEDERPGTSHWPPRPPAAPSADTGFGSRHTVITSKAAESTPLL
GSIRS

Hydra vulgaris:

>XP_012564588.1 PREDICTED: autophagy-related protein 9A-like [Hydra vulgaris]
MMTSFNSIPYVRVAPEEDDEFSELPPTVVTTKGVDKNGCWTHIENLDEFFTEVYTYHQLQGFVCLLLRDV
FELTQYIFIVLFGTFLLVCVDYKKLFSDPNPVFVEVIHWNNFKQMPSSVILCLVVALLFWLVRLCRVIFR
VYRRWHIRKFFYQVLHITDADLRNMQWNEVQHKLILMQQVHQLCIHVKELTELDIHNRILRQKNYMISFQ
NKGIIPCIYHFPFIGKRTFLTEGLKFNLNLILFNGPGAPFKNSWKLKEEYKDYSCRMVLAASLSHRIFML
GVLNFLLCPFIMIYQVLYSFFMYAELIKRSPDVFGARRWSPFGRLYLRHFNELDHEFQSRLNKAYMPATR

YMNVFTSPIITIFAKNIAFFSGAILAVLLGLSFYDKDVLTAEHVLSFMATLGIIIKICSGFIPDEHMVFC
PETLMKQILSNTHYIPEEWKGRAHTMEVREEFSKMFQYKFICLLEELMSPLLTPFILCFGLRYKALQIVD
FYRNFTVEVAGLGDVCSFAQMDIRKHGAKNWSDEGCSKEDQMLSAESQAENAKTELSLLNFSIKNPDWKP
SSHGKQFIDTIKEQAFQESLSHSLSSNQTPFNAGTSFDFTNFIGLQSIAYPALDPAIMVSRNDDVLRNSI
MYLHEVQNRRPISVTSLKSSYGDNAFENSDLRRTWPSATSGQTSFGVNKENLLPRYSAMKNPADDRL

Nematostella vectensis:

>XP_032240258.1 autophagy-related protein 9A [Nematostella vectensis]
MSEFKTQYQRLNSVDDSEEDLPPQGFHVGTRGLEEIEKGRWNHIENLDEFFIRVYEYHRHDGFACILLTQ
CFELVQFAFVVLFTSFLLLCVDYDKLFKNPDPTFSEVVHFHKIEHMEPAIVVCLVIALLFWFIRLARVFI
YFFKLLEIRMFYKNALKIPESEIVNLQWQDVQKRLIEIQKIHQMCVHKEELTELDIHHRILRWKNYFVAM
QNKGVIPCTYRFPFLGQRTFLTEGMKYNLKMILFWGPGSPFQDNWKLRDDFKNLSKRSALADRLSRRIFW
IGVANFLLCPFILLWVVLYSFFSYAELVKRAPDALGARRWSPYGRLFFRHFNELDHEFQARLNKAYEPAQ
KYLNIFSSPFLAIIAKNVAFLAGAVFAVLTTFTLVDRDVLMAEHMLAVIAGLGILIRGCYVFIPNENLVW
NPEQLMRQILSYTHYIPETWKGKAHTTEVRDQFAQIFQYKLVNVLEELVSPVVTPFLLCFSLRYKALDIV
DFYRNFTVDVVGVGDVCSFAQLDVKKHGNPEWLSEGLSEATQYEQAENGKTELSLLHFSIRNPAWKPSDR
GAHFISTIKENAIHEGLNIAGSVTLSEPCSLQDFRSFDCLPGLPYEPAMATSDPTAGLQGGVKAADTRRS
REQHTEALMNTSMLYMHELHSRQADHSMHEVSYPSGPSTQSPRRPETSGLFEETGLTYQAREQTFEQEMT
YMARSHGPEVAFHPGLSGQLGRVEEGSQALHSTSPTAGNSSSQTNEQRDRDQETMVNF

Amphimedon queenslandica:

>XP_003384679.1 PREDICTED: autophagy-related protein 9A-like [Amphimedon queenslandica]
MSKKTRYMRLETDPESSQSSDDDAPPTHIPLHSSQYVKESKWNHIENLDEFFDWVYQYLINHGLFAIVLN
DILELGQFAFLCIFSAFLITCINFKNLFADDVYTNETGVKDVAFGDVIEIDRLEHINPLLVLILFFAFIF
WFHRLCKTIWRFFKFLGIRRFYIEALDIPPSDLRNYNWRDVLERLKSAQNTHKMNIRKQELTEIDVYHRI
LRFKNYMVAMINKDVLSYKIWIPFYGEKVFFTMGLKYNYELLLFWGPGAPFKNNYHLHEEYKTAAKKYEL
IQKLRFRITVLALVNLVFSPFIFLYQILYSFFTYAELLKRQPGVFGRRRWSLFGKIFLRHFNELDHELIV
RLNRAYLPAGKYMDLFVLPVATILARNVAFVAGSLLAVLFTLTVIQEDLLTAHNILTAITGLGVIVTLCR
IFIPNEHQTRDPKELMTEILEEIHYMPDLWKEKPHTVQVSSQFSQVFQLTIAYLIDELLSPLVTPFILYF
SLRNKSQDIIDFLRNFTIDVSGVGDVCSFAEMNLRKHGHSEWQQNSQLARSTDYERSELGKTEISLLNFT
QKNPNWEAGEDADTFLTQLKESVEELHSSQQQQQQSLTLSREDSPPPLSLSSHQFGVSRLQGSMDTSIW
ASFTSANDEASSLTPLKLHQVHDVMKTSHGQGSSSSAAPPPPPIGSIVPSYHPPPPQPQEVALTVYDHCL
PSDSSLSSADIRLESTIGHTPSSTAGGLTEPFLPRDSEPPPQT

Alignment:

```
NP_611114.1[D.melanogaster]          MSSPHINYRSLAEEAASPFLEHHPSTGQGPSKTQDAKANAAAAHLDPLGEHGLEQPLDEH      60
XP_003384679.1[A.queenslandica]      -------------------------------------------MSKKTRYMRLETDP-ES      16
XP_012564588.1[H.vulgaris]           ----------------------------------------MMTSFNSIPYVRVAPEE-D-      18
XP_032240258.1[N.vectensis]          -----------------------------------------MSEFK-TQYQRLNSV--D-      15
XP_019613418.1[B.belcheri]           -----------------------------------------MAQLE-TSYRRLDSF--Y-      15
XP_032801920.1[P.marinus]            -----------------------------------------MAEAE-TGYRRLDSY--D-      15
XP_014005943.1[S.salar]              ----------------------------------------MAAHFD-TEYQRLEAS--Y-      16
XP_018094208.1[X.laevis]             -----------------------------------------MAMYD-TPYQRLEAS--Y-      15
NP_001029993.2[G.gallus]             -----------------------------------------MAHLE-TQYQRLESS--S-      15
NP_001003917.2[M.musculus]           -----------------------------------------MAQFD-TEYQRLEAS--Y-      15
NP_001070666.1[H.sapiens]            -----------------------------------------MAQFD-TEYQRLEAS--Y-      15
NP_001127287.1[P.abelii]             -----------------------------------------MAQFD-TEYQRLEAS--Y-      15
                                                                                  .      :

NP_611114.1[D.melanogaster]          DTEHEGEDTPRNSGVMIHMVPETGRARWNHIEDLDSFFSRMYQYQQKHGFTVIVVDEMLQ      120
XP_003384679.1[A.queenslandica]      SQSSDDDAPPTH--IPLHSSQYVKESKWNHIENLDEFFDWVYQYLINHGLFAIVLNDILE      74
XP_012564588.1[H.vulgaris]           --DEFSELPPTV--VTTKGV--DKNGCWTHIENLDEFFTEVYTYHQLQGFVCLLLRDVFE      72
XP_032240258.1[N.vectensis]          --DSEEDLPPQGFHVGTRGLEEIEKGRWNHIENLDEFFIRVYEYHRHDGFACILLTQCFE      73
XP_019613418.1[B.belcheri]           --DEEMDSPPNERELLVHAVDSNKAHRWNHIENLDDFFTRVYQYHQRHGFLCMMLAEVLE      73
XP_032801920.1[P.marinus]            --DE--ESPNHEREVLVHVPEGSK-ARWNHIENLDYFFTRIYHFHQKHGFACMMLAEFFE      70
XP_014005943.1[S.salar]              ---S--DSPPGEENLLVHIPDGNK-SPWHHIENLDLFFQRVYNLHQKNGFTCMLLGEVFE      70
XP_018094208.1[X.laevis]             ---T--DSPLGEDDLLVHVPEGSK-SPWHHIENLDLFFSHVYNLHQKNGFTCMLIGEIFE      69
NP_001029993.2[G.gallus]             ---T--ESPPGGGDLLVHVPEGAK-SPWHHIENLDLFFSRVYNLHQKNGFTCMLIGEIFE      69
NP_001003917.2[M.musculus]           ---S--DSPPGEEDLLVHVAEGSK-SPWHHIENLDLFFSRVYNLHQKNGFTCMLIGEMFE      69
NP_001070666.1[H.sapiens]            ---S--DSPPGEEDLLVHVAEGSK-SPWHHIENLDLFFSRVYNLHQKNGFTCMLIGEIFE      69
NP_001127287.1[P.abelii]             ---S--DSPPGEEDLLVHVAEGSK-SPWHHIENLDLFFSRVYNLHQKNGFTCMLIGEIFE      69
                                           :       :  :         * ***:** **  :*      .*:  ::: : ::

NP_611114.1[D.melanogaster]          VLEFGFVVWLLAFVMHCVRFDVLFGDTPPGG----LNPNKTTLSDVMYPTGECLANFTWV      176
XP_003384679.1[A.queenslandica]      LGQFAFLCIFSAFLITCINFKNLFADDVYTNET---GVKDV----AFGDVIEIDRLEHIN      127
XP_012564588.1[H.vulgaris]           LTQYIFIVLFGTFLLVCVDYKKLFSDPNPVFVE----------------VIHWNNFKQMP      116
XP_032240258.1[N.vectensis]          LVQFAFVVLFTSFLLLCVDYDKLFKNPDPTFSE----------------VVHFHKIEHME      117
XP_019613418.1[B.belcheri]           LLQFIFVLLFTTFLLQCVHYEVLFGDK------NPHPDRKVTLPEALVPLDQCASSLDS-      126
XP_032801920.1[P.marinus]            LVQFLFVVTFTTFLINCVDYDILFANKQV----HPQPSRKVTLYDALLSSEKCTERIRGN      126
XP_014005943.1[S.salar]              LVQLLFVVGFTVFLANCVDYDILFANKFVNHT----DSSKVTLPDAFLPVDVCSASIRDN      126
XP_018094208.1[X.laevis]             LLQFIFIVSFTTLLVSCVDYDILFANKMVNHSQ--SDHVKVTLPDAFLPPGVCRDRMQEN      127
NP_001029993.2[G.gallus]             LMQFIFVVAFTTFLISCVDYDILFANKAVNHSQHPSEPIKVTLPDAFLPPNVCSARIQAN      129
NP_001003917.2[M.musculus]           LMQFLFVVAFTTFLVSCVDYDILFANKMVNHSLHPTEPVKVTLPDAFLPAQVCSARIQEN      129
NP_001070666.1[H.sapiens]            LMQFLFVVAFTTFLVSCVDYDILFANKMVNHSLHPTEPVKVTLPDAFLPAQVCSARIQEN      129
NP_001127287.1[P.abelii]             LMQFLFVVAFTTFLVSCVDYDILFANKMVNHSLHPTEPVKVTLPDAFLPAQVCSARIQEN      129
                                     : :  *:  :  ::  *: :. ** :

NP_611114.1[D.melanogaster]          T--YLVVFIAAIYLGIRLLKMVYHITQYADIKRFYNSALHIEDSDLDNFTWHEVQQRIRR      234
XP_003384679.1[A.queenslandica]      PLLVLILFFAFIFWFHRLCKTIWRFFKFLGIRRFYIEALDIPPSDLRNYNWRDVLERLKS      187
XP_012564588.1[H.vulgaris]           SSVILCLVVALLFWLVRLCRVIFRVYRRWHIRKFFYQVLHITDADLRNMQWNEVQHKLIL      176
XP_032240258.1[N.vectensis]          PAIVVCLVIALLFWFIRLARVFIYFFKLLEIRMFYKNALKIPESEIVNLQWQDVQKRLIE      177
XP_019613418.1[B.belcheri]           -RIVVCLVLASFFWFLRFIKVVYNFFRYWEIRTFYTQALKISHNDLPNMTWHEVQRCLRQ      185
XP_032801920.1[P.marinus]            SWIIFLIVMAGTFWLYRLIKVIYNFSSYLEIRAFYINALKIPMAELQNFSWQEVQERVLA      186
XP_014005943.1[S.salar]              VAVMFVLMISGVFWLHRLIKFIYNVCCYWEIRSFYTNALKMSMAELPYFTWQEVQARIVE      186
XP_018094208.1[X.laevis]             GFLVCLLAIAGVFWIHRLIKFIYNICCYWEIHNFYMQALRIPMSDLPYYTWEEVQSRIVQ      187
NP_001029993.2[G.gallus]             SFLICILVIAGVFWIHRLVKFIYNICCYWEIHSFYINALRIPMSNLPYYTWQEVQARIVQ      189
NP_001003917.2[M.musculus]           GSLITILVIAGVFWIHRLIKFIYNICCYWEIHSFYLHALRIPMSALPYCTWQEVQARIVQ      189
NP_001070666.1[H.sapiens]            GSLITILVIAGVFWIHRLIKFIYNICCYWEIHSFYLHALRIPMSALPYCTWQEVQARIVQ      189
NP_001127287.1[P.abelii]             GSLITILVIAGVFWIHRLIKFIYNICCYWEIHSFYLHALRIPMSALPYCTWQEVQARIVQ      189
                                           : .:  :   *: : .  .     *: *:  .* :    :    *.:*   :

NP_611114.1[D.melanogaster]          VQAEQHMCIDKESLTELDIYHRVLRFKNYLVALMNKQLLPVRFHIPLYGEVVSLSRGMLF      294
XP_003384679.1[A.queenslandica]      AQNTHKMNIRKQELTEIDVYHRILRFKNYMVAMINKDVLSYKIWIPFYGEKVFFTMGLKY      247
XP_012564588.1[H.vulgaris]           MQQVHQLCIHVKELTELDIHNRILRQKNYMISFQNKGIIPCIYHFPFIGKRTFLTEGLKF      236
XP_032240258.1[N.vectensis]          IQKIHQMCVHKEELTELDIHHRILRWKNYFVAMQNKGVIPCTYRFPFLGQRTFLTEGMKY      237
XP_019613418.1[B.belcheri]           VQVEQQMCIHKQELTELDIYHRILRFKNYTVAMVNKSLLPLKFQLPGMGELVFLTNGLKY      245
XP_032801920.1[P.marinus]            VQKEQQMCIHKRELTELDMHHRILRFKNYLVAMVNKSLLPVRVRVPFAGESALLTQGLKY      246
XP_014005943.1[S.salar]              IQKEHQICIHKKELTELDIYHRILRFKNYMVAMINKSLLPVRFRPPLLGESVFYTRGLKY      246
XP_018094208.1[X.laevis]             IQKEHQICIHKKELSELDVSHRILRFKNYMVAMVNKNLLPLQHRLPLLGNTVFYTRGLKY      247
NP_001029993.2[G.gallus]             IQKEHQICIHKKELTELDIYHRILRFKNYMVAMVNKSLLPIRFRLPLLGDTVFYTRGLKY      249
NP_001003917.2[M.musculus]           TQKEHQICIHKRELTELDIYHRILRFQNYMVALVNKSLLPLRFRLPGLGEVVFFTRGLKY      249
NP_001070666.1[H.sapiens]            TQKEHQICIHKRELTELDIYHRILRFQNYMVALVNKSLLPLRFRLPGLGEAVFFTRGLKY      249
NP_001127287.1[P.abelii]             TQKEHQICIHKRELTELDIYHRILRFQNYMVALVNKSLLPLRFRLPGLGEAVFFTRGLKY      249
                                      *  ::: :  ..*:*:*: :*:** :** ::: ** ::      *   *. .  : *: :

NP_611114.1[D.melanogaster]          NIDFILFRGPGSPFQNNWQLRDEFAVRSNQTELAQRLSKLILGVALLNLVLAPVIFVWQL      354
XP_003384679.1[A.queenslandica]      NYELLLFWGPGAPFKNNYHLHEEYKTAAKKYELIQKLRFRITVLALVNLVFSPFIFLYQI      307
XP_012564588.1[H.vulgaris]           NLNLILFNGPGAPFKNSWKLKEEYKDYSCRMVLAASLSHRIFMLGVLNFLLCPFIMIYQV      296
XP_032240258.1[N.vectensis]          NLKMILFWGPGSPFQDNWKLRDDFKNLSKRSALADRLSRRIFWIGVANFLLCPFILLWVV      297
XP_019613418.1[B.belcheri]           NLEMILFWGPWSPFANNWHLKEDYKKSSKRLELANKLSRHILWVGVANLLLCPFILMWQI      305
XP_032801920.1[P.marinus]            NLEMLLFWGPGAPFQSAWSLRPEYKRAGCRLELAERLGRTILLVGLANLVLCPLVVVWQV      306
XP_014005943.1[S.salar]              NFELIFFWGPGSLFESEWSLKPQYKRGGNRLELADKLASRILWIGIANLVLCPVVLVWQI      306
XP_018094208.1[X.laevis]             NFQLIFFWGPGCLFQNEWSLKPEYKRSGARLELSEKLAQRILWIGLANLVLCPLVLVWQI      307
NP_001029993.2[G.gallus]             NFELIFFWGPGSLFENEWSLKAEYKRAGNRLELAEKLSTRILWIGIANFLLCPLILIWQI      309
```

```
NP_001003917.2[M.musculus]          NFELILFWGPGSLFLNEWSLKAEYKRGGQRLELAQRLSNRILWIGIANFLLCPLILIWQI      309
NP_001070666.1[H.sapiens]           NFELILFWGPGSLFLNEWSLKAEYKRGGQRLELAQRLSNRILWIGIANFLLCPLILIWQI      309
NP_001127287.1[P.abelii]            NFELILFWGPGSLFLNEWSLKAEYKRGGQRLELAQRLSNRILWIGIANFLLCPLILIWQI      309
                                    * .:::* ** . * . : *: ::   . :  *    *   *  :.: *:::.*.:.:: :

NP_611114.1[D.melanogaster]         IYFSFSYANILRKEPGALGLRTWSNYGRLYLRHFNELDHELDARLNRAYDYADRYLNSFS      414
XP_003384679.1[A.queenslandica]     LYSFFTYAELLKRQPGVFGRRRWSLFGKIFLRHFNELDHELIVRLNRAYLPAGKYMDLFV      367
XP_012564588.1[H.vulgaris]          LYSFFMYAELIKRSPDVFGARRWSPFGRLYLRHFNELDHEFQSRLNKAYMPATRYMNVFT      356
XP_032240258.1[N.vectensis]         LYSFFSYAELVKRAPDALGARRWSPYGRLFFRHFNELDHEFQARLNKAYEPAQKYLNIFS      357
XP_019613418.1[B.belcheri]          LYSFFTYAEVLKRQPGSLGARKWSLYGRLYLRHFNELDHELNARLTRAYKPAEKYMSSFI      365
XP_032801920.1[P.marinus]           LHAFFRYAEVLKREPGSLGARRWSLYGQLYLRHLNELDHELRARLGRGYRHAARYMDSFT      366
XP_014005943.1[S.salar]             LYAFFSYTEVIKREPGSLGARCWSLYGRCYLRHFNELDHELMSRLSKGYKASSRYMNCFL      366
XP_018094208.1[X.laevis]            LYAFFSYTEVLRREPGSLGARCWSLYGRCYLRHFNELDHQLQARLSRAYKPASKYMNCFL      367
NP_001029993.2[G.gallus]            LYAFFSYTEILKREPGSLGARCWSLYGRCYLRHFNELDHELQSRLSKGYKPASKYMNCFI      369
NP_001003917.2[M.musculus]          LYAFFSYAEVLKREPGALGARCWSLYGRCYLRHFNELEHELQSRLNRGYKPASKYMNCFL      369
NP_001070666.1[H.sapiens]           LYAFFSYAEVLKREPGALGARCWSLYGRCYLRHFNELEHELQSRLNRGYKPASKYMNCFL      369
NP_001127287.1[P.abelii]            LYAFFSYAEVLKREPGALGARCWSLYGRCYLRHFNELEHELQSRLNRGYKPASKYMNCFL      369
                                    ::  * *:::::: *. :* * ** :*: ::**:***:*::  ** :.*  : :*:. *

NP_611114.1[D.melanogaster]         SPLAAVIAKNLLFISGGLLLLILALGIYEEHVFQVEHLLAILAGLGAIGVVCRTLIPDEN      474
XP_003384679.1[A.queenslandica]     LPVATILARNVAFVAGSLLAVLFTLTVIQEDLLTAHNILTAITGLGVIVTLCRIFIPNEH      427
XP_012564588.1[H.vulgaris]          SPIITIFAKNIAFFSGAILAVLLGLSFYDKDVLTAEHVLSFMATLGIIIKICSGFIPDEH      416
XP_032240258.1[N.vectensis]         SPFLAIIAKNVAFLAGAVFAVLTTFTLVDRDVLMAEHMLAVIAGLGILIRGCYVFIPNEN      417
XP_019613418.1[B.belcheri]          SHVMTLIARHVAFLAGAIFAVLVVLTIYDEDVLTIEHVLTITTLMGVLVAGCRALIPDEN      425
XP_032801920.1[P.marinus]           APMMAVLAKNVTFFAGSLLAVLLALTVYDEDVLSVEHVLTAITALGVLLTVCRAFIPDEH      426
XP_014005943.1[S.salar]             SPLLTVVAKNVAFFAGSLLAVLIALTIYDEDVLAVEHVLSSITLLGVCITVCRSFIPDKH      426
XP_018094208.1[X.laevis]            SPLLAVVAKHFGFLAGSILAVLIALTVYDEDVLAVEHVLSAVTLLGIVVTICRSFIPDQH      427
NP_001029993.2[G.gallus]            SPLLTIVAKNVAFFAGSILAVLIALTIYDEDVLAVEHVLTTVTLLGVGITVCRSFIPDQH      429
NP_001003917.2[M.musculus]          SPLLTLLAKNGAFFAGSILAVLIALTIYDEDVLAVEHVLTTVTLLGVTVTVCRSFIPDQH      429
NP_001070666.1[H.sapiens]           SPLLTLLAKNGAFFAGSILAVLIALTIYDEDVLAVEHVLTTVTLLGVTVTVCRSFIPDQH      429
NP_001127287.1[P.abelii]            SPLLTLLAKNGAFFAGSILAVLIALTIYDEDVLAVEHVLTTVTLLGVTVTVCRSFIPDQH      429
                                      . ::.*::   *.:*.:: ::  : . :..::  .::*:  : :*     *  :**::

NP_611114.1[D.melanogaster]         LVWCPEQLMTAILAHVHYLPSEWRQQAHTTKVRQEFSNFFQFKAGYLLSEIFSPFVTPFV      534
XP_003384679.1[A.queenslandica]     QTRDPKELMTEILEEIHYMPDLWKEKPHTVQVSSQFSQVFQLTIAYLIDELLSPLVTPFI      487
XP_012564588.1[H.vulgaris]          MVFCPETLMKQILSNTHYIPEEWKGRAHTMEVREEFSKMFQYKFICLLEELMSPLLTPFI      476
XP_032240258.1[N.vectensis]         LVWNPEQLMRQILSYTHYIPETWKGKAHTTEVRDQFAQIFQYKLVNVLEELVSPVVTPFL      477
XP_019613418.1[B.belcheri]          MVFCPEQLMVCILAQIHYMPDRWKGQAHTYRVRDEFSQLFQYKAVFLLEELLSPIITPLI      485
XP_032801920.1[P.marinus]           LVWNPEHLLQAVLAHIHYMPEHWKGIAHKTETRDEFSQLFQYKAAFIMEELLSPIITPFI      486
XP_014005943.1[S.salar]             LVYCPEQLLRVILAHIHYMPDHWQGNAHRYETRDQFSQLFQYKAVFILEELISPLVTPFI      486
XP_018094208.1[X.laevis]            LVFCPEQLLRVILAHIHYMPDHWQRSAHRAETRGEFSQLFQYKAVFILEELLSPLLTPLV      487
NP_001029993.2[G.gallus]            LVFCPEQLLRVILAHIHYMPDHWQGNAHRYETRDEFAQLFQYKAVFILEELLSPIITPLI      489
NP_001003917.2[M.musculus]          MVFCPEQLLRVILAHIHYMPDHWQGNAHRSQTRDEFAQLFQYKAVFILEELLSPIVTPLI      489
NP_001070666.1[H.sapiens]           MVFCPEQLLRVILAHIHYMPDHWQGNAHRSQTRDEFAQLFQYKAVFILEELLSPIVTPLI      489
NP_001127287.1[P.abelii]            MVFCPEQLLRVILAHIHYMPDHWQGNAHRSQTRDEFAQLFQYKAVFILEELLSPIVTPLI      489
                                      .  *: *:  :*   **:*. *:    *  ..  :*::.** .    ::.*:.**.:**::

NP_611114.1[D.melanogaster]         LIFVFRPKAIELVRFFRTFTVSVRGVGNVCSFAQMDVRKHGNPDWQLTSELEEMTRATAQ      594
XP_003384679.1[A.queenslandica]     LYFSLRNKSQDIIDFLRNFTIDVSGVGDVCSFAEMNLRKHGHSEWQQNSQLARSTDY---      544
XP_012564588.1[H.vulgaris]          LCFGLRYKALQIVDFYRNFTVEVAGLGDVCSFAQMDIRKHGAKNWSDEG-CSKEDQML--      533
XP_032240258.1[N.vectensis]         LCFSLRYKALDIVDFYRNFTVDVVGVGDVCSFAQLDVKKHGNPEWLSEG-LSEATQY---      533
XP_019613418.1[B.belcheri]          LIFSLRSKALDIVDFFRNFTVDVVGVGDVCSFAQMDIKRHGNPQWTGEKQQTDVSQY---      542
XP_032801920.1[P.marinus]           LIFGVRPKALEIVDFFRNFTVEVVGVGDVCSFAQMDIRRHGNPTWMSEG-QTEASYY---      542
XP_014005943.1[S.salar]             LIFSLRRKSLEIIDFFRNFSVEVVGVGDTCSFAQMDIRQHGHPAWMSEG-KTEASIY---      542
XP_018094208.1[X.laevis]            LIFCLRPKSLEIIDFFRNFTVEVVGVGDTCSFAQMDVRQHGNPAWMSAG-KTEASIY---      543
NP_001029993.2[G.gallus]            LIICLRPKSLDIVDFFRNFTVEVVGVGDTCSFAQMDVRQHGHPAWMSAG-KTEASIY---      545
NP_001003917.2[M.musculus]          LIFCLRPRALEIIDFFRNFTVEVVGVGDTCSFAQMDVRQHGHPQWLSGG-QTEASVY---      545
NP_001070666.1[H.sapiens]           LIFCLRPRALEIIDFFRNFTVEVVGVGDTCSFAQMDVRQHGHPQWLSAG-QTEASVY---      545
NP_001127287.1[P.abelii]            LIFCLRPRALEIIDFFRNFTVEVVGVGDTCSFAQMDVRQHGHPQWLSAG-QTEASVY---      545
                                    * : .* :: ::: * *.*::.* *:*:.****:::::**   *

NP_611114.1[D.melanogaster]         QPQQEPQQQSLAGGKTEMSLLRFTLNNPEWQMPKEAKQFLRGVREHAVGELVQAKTSM--      652
XP_003384679.1[A.queenslandica]     --------ERSELGKTEISLLNFTQKNPNWEAGEDADTFLTQLKESVEELHSSQQQQQQQ      596
XP_012564588.1[H.vulgaris]          -----SAESQAENAKTELSLLNFSIKNPDWKPSSHGKQFIDTIKEQAFQESLSHSLSSNQ      588
XP_032240258.1[N.vectensis]         --------EQAENGKTELSLLHFSIRNPAWKPSDRGAHFISTIKENAIHEGLNIAGSVTL      585
XP_019613418.1[B.belcheri]          --------HQAEDGKTELSLMHFTLTNPTWQPPRHCNKFINHLKEQAHKDVNML---NSA      591
XP_032801920.1[P.marinus]           --------QQAEDGKTELSLVHFAISNPRWRPPRGSNVYLNQLREQVQRDSTAN------      588
XP_014005943.1[S.salar]             --------QQAEDGKTELSLMHFAITNPQWQPPTETTHFISQLKERVQREATGDRHT---      591
XP_018094208.1[X.laevis]            --------QQAEDGKTELSLMHFAITNPRWQPPRECSAFLSQLRERVQRDSTMGNQKHPT      595
NP_001029993.2[G.gallus]            --------QQAEDGKTELSLMHFAITNPKWQPPRESTAFIGFLKERVHRDSS-VALAQQA      596
NP_001003917.2[M.musculus]          --------QQAEDGKTELSLMHFAITNPGWQPPRESTAFLGFLKEQVQRDGAAAGLAQGG      597
NP_001070666.1[H.sapiens]           --------QQAEDGKTELSLMHFAITNPGWQPPRESTAFLGFLKEQVQRDGAAASLAQGG      597
NP_001127287.1[P.abelii]            --------QQAEDGKTELSLMHFAITNPGWQPPRESTAFLGFLKEQVQRDGAAASLAQGG      597
                                                 .***:**:.*:  ** *.       ::  ::* .

NP_611114.1[D.melanogaster]         -VQENP----LTNSLISFGTMGADYCSIANSVL-----TAQVTPQQLEISQSLRPGLGPV      702
XP_003384679.1[A.queenslandica]     SLT------------LSREDSPPPLSLSSHQF-GVSRLQGSM---DTSIWA--------S      632
XP_012564588.1[H.vulgaris]          -TPFNAGTSFDFTNFIGLQSIAYPAL----------------------------------      613
XP_032240258.1[N.vectensis]         -S--EPCSLQDFRSFDCLPGLPYEPAMA--------------------------------      610
XP_019613418.1[B.belcheri]          VMVDNP----FFQSLQSLSSAGYPSMMTSLMVGRL--------GPDATLVP-------PR      632
XP_032801920.1[P.marinus]           --PQPL----LFSSLQTIESQSGPYGLLGSMLGGPSSLTAYRSTRDATCSL-------PE      635
```

```
XP_014005943.1[S.salar]             ---------------LTSLSGSEPRSLIANFLAGPPSLASLHLGRDGSLTN-------HG      629
XP_018094208.1[X.laevis]            GL-MQP----LQCSGLSIQSDPEPHSLMANMFSGVST-AGFPANTEALGSP-------HV      642
NP_001029993.2[G.gallus]            VLPENA----LFSSIQSLQSESEPHSLIANVIAGSSV-LGFHMGRDGQASR-------HL      644
NP_001003917.2[M.musculus]          LLPENA----LFTSIQSLQSESEPLSLIANVVAGSSC-RGPSLSRDLQGSR-------HR      645
NP_001070666.1[H.sapiens]           LLPENA----LFTSIQSLQSESEPLSLIANVVAGSSC-RGPPLPRDLQGSR-------HR      645
NP_001127287.1[P.abelii]            LLPENA----LFTSIQSLQSESEPLSLIANVVAGSSC-RGPPLPRDLQGSR-------HR      645

NP_611114.1[D.melanogaster]         SGGFPVAASDFRQMLQQNLSASVGPLDSMRRLRLSRA--------------EGRLEGPTD      748
XP_003384679.1[A.queenslandica]     FTSANDEASSLTPLKLHQVHD---------VMKT---------SHGQGSSSSAAPPPPPI      674
XP_012564588.1[H.vulgaris]          ------------------------------------------------------DPAI      617
XP_032240258.1[N.vectensis]         ------------------------------------------------------TSDPTA      616
XP_019613418.1[B.belcheri]          DPGTGT--------EPSQPG---------VALPGTTAPGRYGLVRGDISSVEGPIQAPNT      675
XP_032801920.1[P.marinus]           MSAATAAATALMSLSMPSASLTPGPREELAEMPDS-TPGNTTVT---------ARPGSST      685
XP_014005943.1[S.salar]             PVGVSDGASALRSLSISSMRGSC-------------------SS---------AHRS---      658
XP_018094208.1[X.laevis]            -SAVSEVASALHSLSPQQSCHPV--------------------------------------      664
NP_001029993.2[G.gallus]            -SE---VASALRSFSPLQSAQQP--------------SGGFQTA---------GRDG---      674
NP_001003917.2[M.musculus]          -AD---VASALRSFSPLQPGAAP--------------QG---------------------      666
NP_001070666.1[H.sapiens]           -AE---VASALRSFSPLQPGQAP--------------TG---------------------      666
NP_001127287.1[P.abelii]            -AE---VASALRSFSPLQPGQAP--------------TG---------------------      666

NP_611114.1[D.melanogaster]         TLLYG------------LCGVDPR-------------VGSTPLNVGVADMCLSALYLHEL      783
XP_003384679.1[A.queenslandica]     GSIVPSYHPP-----------------------PPQPQEVAL-----------TVYDHCL      700
XP_012564588.1[H.vulgaris]          --------------M---------------------------VSRNDDVLRNSIMYLHEV      636
XP_032240258.1[N.vectensis]         GLQGGVKAADTRRSR---------------------------EQHTEALMNTSMLYMHEL      649
XP_019613418.1[B.belcheri]          GLLGSSTMAPRGTASISHTLPP---TPE----PVSLQETMAPTELMAAEMSFSALYMHQL      728
XP_032801920.1[P.marinus]           GAVGGMSMSTGGRGKVSFGGTDFRSSSVGSSFREGPSQSSILSEFASAEMSLHAIYMHEA      745
XP_014005943.1[S.salar]             -----AGHASSASRAMPGSSTDARTASSGSSVWEGQLTSLVLSEYASTEMSVHALYIHEL      713
XP_018094208.1[X.laevis]            -------------QSQSGISADFRNGSFGSNVWEGPMNSGTLSEYASTEMSLHALYMHEL      711
NP_001029993.2[G.gallus]            -----EGTQPRGSSAMTASGADARTMSSGSSAWEGQLQSMILSEYASTEMSLHALYMHEL      729
NP_001003917.2[M.musculus]          ----------RVPSTMTGSGVDARTASSGSSVWEGQLQSLVLSEYASTEMSLHALYMHQL      716
NP_001070666.1[H.sapiens]           ----------RAHSTMTGSGVDARTASSGSSVWEGQLQSLVLSEYASTEMSLHALYMHQL      716
NP_001127287.1[P.abelii]            ----------RAHSTMTGSGVDARTASSGSSVWEGQLQSLVLSEYASTEMSLHALYMHQL      716
                                                                                   :* *

NP_611114.1[D.melanogaster]         NQQKRQARQS----RIDEAEDERPGTSHWPPRPPAAPSADT----GFG-------SRHTV      828
XP_003384679.1[A.queenslandica]     PSDSSLSSAD--------------IRLE-------------------------STIGHTP      721
XP_012564588.1[H.vulgaris]          QNRRPISVTSLKSSYGDNAFENSDLRRTWPSATSGQTSFGVNKENLLP----RYSAMKNP      692
XP_032240258.1[N.vectensis]         HSRQADHSMH---------------EVSYPSGPSTQSPRRPETSGLFEETGLTYQAREQT      694
XP_019613418.1[B.belcheri]          HQTQTHHDHQD-----------QEAQRAWRQPLPPAFYGTTMPA---------NQQRPSP      768
XP_032801920.1[P.marinus]           RRNYATSDAQ--------------ARYHWQQPENNGDDDTMDEVNALSGFRESSLQLHNT      791
XP_014005943.1[S.salar]             HKQQSGGE-L--------------SRHTWHRQDSDDSSDSVTEEGGSGGG---NPNPRNP      755
XP_018094208.1[X.laevis]            HQQHTRMA-P--------------GRHIWHRQESDESGESSQGEGLINTT---SNV---P      750
NP_001029993.2[G.gallus]            HKQHAQLE-P--------------ERHTWHRRESDESGESTHEELDAQRG---APV---P      768
NP_001003917.2[M.musculus]          HKQQTQAE-P--------------ERHVWHRRESDESGESAPEEGGEGAR---APQ---P      755
NP_001070666.1[H.sapiens]           HKQQAQAE-P--------------ERHVWHRRESDESGESAPDEGGEGAR---APQ---S      755
NP_001127287.1[P.abelii]            HKQQAQAE-P--------------ERHLWHRRESDESGESAPDEGGEGAR---APQ---S      755

NP_611114.1[D.melanogaster]         ITSKAAESTPLLGSIRS-------------------------------------------      845
XP_003384679.1[A.queenslandica]     SSTAG----------------------GLTEPFLPRDSEP--------------------      739
XP_012564588.1[H.vulgaris]          A-------------DDRL------------------------------------------      697
XP_032240258.1[N.vectensis]         F-------------EQEMTYMARSH--GPEVAFHPGLSGQLG------------------      721
XP_019613418.1[B.belcheri]          GSVEDSR-------------------TSEPGQWHSVGS-----TTMPGIDEAPSEETDTL      804
XP_032801920.1[P.marinus]           GSNSGSHSSSVLGSGQRETYVSPSASIGHSGDYMPPNIGSLQDTHGDSLGLMVTRENTEL      851
XP_014005943.1[S.salar]             I-------------PRSHTFPISTP-ITTPGPIPSSNSGP-----NPDRGTTPGQDVTPL      796
XP_018094208.1[X.laevis]            S---------------SL--SCPSP-AAA------------------------VWE-ERL      767
NP_001029993.2[G.gallus]            L-------------PRSASYPFSSR-QPA-------------------------EETATL      789
NP_001003917.2[M.musculus]          I-------------PRSASYPCATP-RPG------------------------APETTAL      777
NP_001070666.1[H.sapiens]           I-------------PRSASYPCAAP-RPG------------------------APETTAL      777
NP_001127287.1[P.abelii]            I-------------PRSASYPCAAP-RPG------------------------APETTAL      777

NP_611114.1[D.melanogaster]         ------------------------------------------------------------      845
XP_003384679.1[A.queenslandica]     -----------------------------------------------------------P      740
XP_012564588.1[H.vulgaris]          ------------------------------------------------------------      697
XP_032240258.1[N.vectensis]         ------------------RVEEGSQ---------------A---LHSTSPTAGNSSSQTN      745
XP_019613418.1[B.belcheri]          SYS--------------------------------------------------------P      808
XP_032801920.1[P.marinus]           GAFPFRHYGGTSASENMLQPEQGSPRFGLMPMGGWQEDMAGLGTID---PHGGVPSCM-S      907
XP_014005943.1[S.salar]             QSNSQRRYGGNTD---SVGPEGRVVRSARVPMGGWAEEGRG-GRHPDTVPEEGSEDDM-P      851
XP_018094208.1[X.laevis]            PPLSTHRLSASTD---LGSVQR--TSLARQPMGGWSEDVQP-SRLPEPVPEEGSEDEL-P      820
NP_001029993.2[G.gallus]            QTGFQRRYGGITD---PGTVHRAPSHFSRLPLGGWAEDGQS-ARHPEPVPEESSEDEL-P      844
NP_001003917.2[M.musculus]          HGGFQRRYGGITD---PGTVPRGPSHFSRLPLGGWAEDGQPASRHPEPVPEEGSEDEL-P      833
NP_001070666.1[H.sapiens]           HGGFQRRYGGITD---PGTVPRVPSHFSRLPLGGWAEDGQSASRHPEPVPEEGSEDEL-P      833
NP_001127287.1[P.abelii]            HGGFQRRYGGITD---PGTVPRAPSHFSRLPLGGWAEDGQSASRHPEPVPEEGSEDEL-P      833

NP_611114.1[D.melanogaster]         --------------------   845
XP_003384679.1[A.queenslandica]     PQT-----------------   743
XP_012564588.1[H.vulgaris]          --------------------   697
```

```
XP_032240258.1[N.vectensis]          EQRDRDQETMVNF-------   758
XP_019613418.1[B.belcheri]           PNV-----------------   811
XP_032801920.1[P.marinus]            PNPQQINEVAYTFHSLFLNT   927
XP_014005943.1[S.salar]              PN---IHKVT----------   858
XP_018094208.1[X.laevis]             PQ---VQKV-----------   826
NP_001029993.2[G.gallus]             PQ---IHKV-----------   850
NP_001003917.2[M.musculus]           PQ---VHKV-----------   839
NP_001070666.1[H.sapiens]            PQ---VHKV-----------   839
NP_001127287.1[P.abelii]             PQ---VHKV-----------   839
```
