# Supplementary material for: An Evolutionary Perspective on the Origin, Conservation and Binding Partner Acquisition of Tankyrases
Source: Biomolecules. 2022 Nov 15;12(11):1688. doi: 10.3390/biom12111688 (PMC9688111; doi:10.3390/biom12111688)
Supplement: Supplementary file 1 [file biomolecules-12-01688-s001.zip › biomolecules-1913719-supplementary-11.17 -done/Data_7/Axin1.pdf]

Axin1

Homo sapiens:

>NP_003493.1 axin-1 isoform a [Homo sapiens]
MNIQEQGFPLDLGASFTEDAPRPPVPGEEGELVSTDPRPASYSFCSGKGVGIKGETSTATPRRSDLDLGYEPEGSASPTPPYLKWAESLHSLLDDQDGISLFRTFLKQEGCADLLDFWFACTGFRKLEPCDSNEEKRLKLARAIYRKYILDNNGI
VSRQTKPATKSFIKGCIMKQLIDPAMFDQAQTEIQATMEENTYPSFLKSDIYLEYTRTGSESPKVCSDQSSGSGTGKGISGYLPTLNEDEEWKCDQDMDEDDGRDAAPPGRLPQKLLLETAAPRVSSSRRYSEGREFRYGSWREPVNPYYVNAGY
ALAPATSANDSEQQSLSSDADTLSLTDSSVDGIPPYRIRKQHRREMQESVQVNGRVPLPHIPRTYRVPKEVRVEPQKFAEELIHRLEAVQRTREAEEKLEERLKRVRMEEEGEDGDPSSGPPGPCHKLPPAPAWHHFPPRCVDMGCAGLRDAHEE
NPESILDEHVQRVLRTPGRQSPGPGHRSPDSGHVAKMPVALGGAASGHGKHVPKSGAKLDAAGLHHHRHVHHHVHHSTARPKEQVEAEATRRAQSSFAWGLEPHSHGARSRGYSESVGAAPNASDGLAHSGKVGVACKRNAKKAESGKSASTEVP
GASEDAEKNQKIMQWIIEGEKEISRHRRTGHGSSGTRKPQPHENSRPLSLEHPWAGPQLRTSVQPSHLFIQDPTMPPHPAPNPLTQLEEARRRLEEEEKRASRAPSKQRYVQEVMRRGRACVRPACAPVLHVVPAVSDMELSETETRSQRKVGGG
SAQPCDSIVVAYYFCGEPIPYRTLVRGRAVTLGQFKELLTKKGSYRYYFKKVSDEFDCGVVFEEVREDEAVLPVFEEKIIGKVEKVD

Pongo abelii:

>XP_024089076.1 axin-1 isoform X1 [Pongo abelii]
MNIQEQGFPLDLGASFTEDAPRPPVPGEEGELVSTDPRPASYSFCSGKGVGVKGETSTATPRRSDLDLGYEPEGSASPTPPYLKWAESLHSLLDDQDGISLFRTFLKQEGCADLLDFWFACTGFRKLEPCDSNEEKRLKLARAIYRKYILDNNGI
VSRQTKPATKSFIKGCIMKQLIDPAMFDQAQTEIQATMEENTYPSFLKSDIYLEYTRTGSESPKVCSDQSSGSGTGKGISGYLPTLNEDEEWKCDQDMDEDDGRDAAPPGRLPQKLLLETAAPRVSSSRRYSEGREFRYGSWREPVNPYYVNAGY
ALAPATSANDSEQQSLSSDADTLSLPDSSVDGIPPYRIRKQRRREMQESVQVNGRVPLPHIPRTYRVPKEVRVEPQKFAEELIHRLEAVQRTREAEEKLEERLKRVRMEEEGEDGDPSSGPPGPCHKLPPAPAWHHFPPRCVDVGCAGLRDAHEE
NPESILDEHVQRVLRTPGRQSPGPGHRSPDSGHVAKMPVALGGAASGHGKHAPKSGVKLDAAGLHHHRHVHHHVHHSTGRPKEQVEAEATRRAQSSFAWGPEPHSHGAKSRGYSESVGAAPNASDGLAHSGKAGVACKRNAKKAESGKSASTEVP
GASEDVEKNQKIMQWIIEGEKEISRHRRTGHGSSGTRKPQPHENSRPLSLERPGAVHPWAGPQLRTSVQPSHLFIQDPTMPPHPAPNPLTQLEEARRRLEEEEKRASRAPSKQRYVQEVMRRGRACVRPACAPVLRVVPAVSDMELSETETRSQR
KVSGGSAQPCDSIVVAYYFCGEPIPYRTLVRGRAVTLGQFKELLTKKGSYRYYFKKVSDEFDCGVVFEEVREDEAILPVFEEKIIGKVEKVD

Mus musculus:

>EDL22489.1 axin 1, isoform CRA_a [Mus musculus]
MQSPKMNVQEQGFPLDLGASFTEDAPRPPVPGEEGELVSTDSRPVNHSFCSGKGTSIKSETSTATPRRSDLDLGYEPEGSASPTPPYLRWAESLHSLLDDQDGISLFRTFLKQEGCADLLDFWFACSGFRKLEPCDSNEEKRLKLARAIYRKYIL
DSNGIVSRQTKPATKSFIKDCVMKQQIDPAMFDQAQTEIQSTMEENTYPSFLKSDIYLEYTRTGSESPKVCSDQSSGSGTGKGMSGYLPTLNEDEEWKCDQDADEDDGRDPLPPSRLTQKLLLETAAPRAPSSRRYNEGRELRYGSWREPVNPYY
VNSGYALAPATSANDSEQQSLSSDADTLSLTDSSVDGIPPYRIRKQHRREMQESIQVNGRVPLPHIPRTYRMPKEIRVEPQKFAEELIHRLEAVQRTREAEEKLEERLKRVRMVCPYSCSDSTAVSPQEEEGEDGEMPSGPMASHKLPSVPAWHH
FPPRYVDMGCSGLRDAHEENPESILDEHVQRVMRTPGCQSPGPGHRSPDSGHVAKTAVLGGTASGHGKHVPKLGLKLDTAGLHHHRHVHHHVHHNSARPKEQMEAEVARRVQSSFSWGPETHGHAKPRSYSENAGTTLSAGDLAFGGKTSAPSKR
NTKKAESGKNANAEVPSTTEDAEKNQKIMQWIIEGEKEISRHRKAGHGSSGLRKQQAHESSRPLSIERPGAVHPWVSAQLRNSVQPSHLFIQDPTMPPNPAPNPLTQLEEARRRLEEEEKRANKLPSKQRYVQAVMQRGRTCVRPACAPVLSVVP
AVSDLELSETETKSQRKAGGGSAPPCDSIVVAYYFCGEPIPYRTLVRGRAVTLGQFKELLTKKGSYRYYFKKVSDEFDCGVVFEEVREDEAVLPVFEEKIIGKVEKVD

Gallus gallus:

>NP_990275.1 axin-1 [Gallus gallus]
MNIQGKGFPLDLGRSFTEDAPRPPVPGEEGELVSTDPRPVSHGFYSSKSDAVRNETSTATPRRSDLDLGYEPEGSASPTPPYLKWAESLHSLLDDQDGINLFRTFLKQEDCADLLDFWFACSGFRKLEPCVSNEEKRLKLAKAIYKKYILDNNGI
VSRQIKPATKSFIKDCVMKLQIDPDMFDQAQTEIQCMIEDNTYPLFLKSDIYLEYTRTGGESPKIYSDPSSGSGTGKGLPGYLPTLNEDEEWKCDQDTEPEASRDSAPSSRLTQKLLLETATQRATSTRRYSEGREFRHGSWREPVNPYYVNTGY
AMAPATSANDSEQQSMSSDADTMSLTDSSIDGIPPYRLRKQHRREMQESAKANGRVPLPHIPRTYRMPKDIHVEPEKFAAELINRLEEVQKEREAEEKLEERLKRVRAEEEGEDADISSGPSVISHKMPSAQPFHHFAPRYSEMGCAGMQMRDAH
EENPESILDEHVQRVMKTPGCQSPGPGRHSPKPRSPESGHLGKLSGTLGTIPPGHGKHTTKSGMKLDAANLYHHKHVYHHIHHHSMMKPKEQIEAEATQRVQNSFAWNVDSHNYATKSRNYSENLGMAPVPMDSLGYSGKASLLSKRNIKKTDSG
KSDGANYEMPGSPEDVERNQKILQWIIEGEKEISRHKKTNHGSSGVKKQLSHDMVRPLSIERPVAVHPWVSAQLRNVVQPSHPFIQDPTMPPNPAPNPLTQLEEARRRLEEEEKRAGKLPLKQRLKPQKRPGSGASQPCENIVVAYYFCGEPIPY
RTLVKGRVVTLGQFKELLTKKGNYRYYFKKVSDEFDCGVVFEEVREDDTILPIFEEKIIGKVEKID

Xenopus laevis:

>NP_001089251.1 axin 1 S homeolog [Xenopus laevis]
MSVKAKGFPLDLGGSFTEDAPRPPVPGEEGELITTDQRPFSHTYYSLKNDGIKNETSTATPRRPDLDLGYEPEGSASPTPPYLKWAESLHSLLDDQDGINLFRNFLQQENCVDLLDFWFACSGFRKLEPNDSKVEKRLKLAKAIYKKYVLDSNGI
VSRQIKPATKSFIKDCVLRQQIDAAMFDQAQMEIQSMMEDNTYPVFLKSDIYLEYTTIGGESPKNYSDQSSGSGTGKGPSGYLPTLNEDEEWRCDQGGEQERESVPSSLFSQKLILDSSSHCAGSNRRLSDGREFRPGTWREPVNPYYVNTGYSG
APVTSANDSEQQSMSSDADTMSLTDSSVDGIPPYRLRKHYRREMQESANANGRGPLPHIPRTYHMPKDIHVDPEKFAAELINRLEGVLRDREAEQRLEERLKRVRAEEEGDDGDVSSGPSIISHKLPSGPPMHHYNSRYSDSGCVGMQIRDAHEE
NPESILDEHVQRVMKTPGCQSPGTGRHSPKPRSPDGHLSKTLPGSLGTMQTGHGKHSSKSGSKVDSGNLHHHKHVYHHLHHHGGVKPKEQIEAEVTQRVQTNYPWNAESHNYATKSRNYAESMGMAPNPMDSLAYSGKVSMLSKRNAKKADVGKS
ESASYEVPAVPEDAERHQKILQWIMEGEKEISRHKKSNHSSSSAKKQPPPELSRPLSIERPGAVHPWVSAQLRNVVQPSHPFIQDPTMPPNPAPNPLTQLEEARRRLEEEEKRAAKMPQKQRYVQEVIQRGRCSVRPAYIPLLNVVPAVSDMDLS
EAELKPQKKTVSSPSQPCENIVVAYYFCGEPIPYRTMVKGRVVTLGQFKELLTKKGNYRYYFKKVSDEFDCGVVFEEVREDDMILPIYEEKIIGKVEKID

Salmo salar:

>XP_014059686.1 PREDICTED: axin-1-like isoform X1 [Salmo salar]
MDAVNMSVGDKVGGYLVDLGGSFTEDAPRPPVPGEEGELVSSDGRPYGHGFYSFKSEILKNEASTATPRRPDLDLGYEPEGSASPTPPYLKWAESLHSLLDDQDGIHLFRTFLKQEECADVLDFWFACTGFRKLEANEGQGEEKKLKLAKAIYKK
YILDNNGIVSRQIKPATKSFIKDCVMRLHIDPAMFDQAQTEIQTMMEENTYPLFLKSDIYLEYTRTGGESPKLYSDQCSVSGGGKVLPGYLPTLNEDEEWRCDHELEEQQESDPTPSNRLTQKLLQETASQRVTSTKRFQDSHEYRRVLWQEPIN
PYYVNTGYAMAPATSANDSEQQSMSSASDVDTLSLTDSSVDGVPPYKYRKQHRREMHESAKANGRVPLPHIPRTNRIPKDIHVEPEKFAADLISRLEGVQREREAQEKLEERLKRVRLEEEGEDADISTSTSFSSHRLPPGGHPQHYNNPRYSDI
GYSGLALRPDAHEENPESILDDHVQRVMKTPGCQSPGTGRHSPKSRSPDGFPGGKVPGPGMPPPTGPGKHPARLGPKGEAASHQYHHKHVHHIHHPAGGKPKEQVEADAAMRVNGNFHWGTEQHNYGSKSRNYADGTGPSPMDPMGYSSKGSTLS
KRSFKKAEEVRTFEVPVPTEDVERNQKILQWMMEGEAVRNKKSPYGSTTGSKKTPSSHEVSRPSSVERPGAVHPWVSAQLRNNVQPSHPFIQDPTMPPNPAPNPLTQLEEARRRLEEEKKKTGTLQTKQRYVMEVIQRGRAAARPALFPPLSVVP
AVSDTELSESEHKVTKKPACENITVAYYFCGEPIPYRTSVKGRMVMLGQFKELLTKKGSYRYFFKKVSNDFGVVFEEVREDDAILPIFEEKIIGKVEKID

Petromyzon marinus:

>XP_032816874.1 axin-1-like [Petromyzon marinus]
MSVSEKLQRLDLSHIIENAITRRPPVPGREGKVNVWPEHCNDPGLARANGVKGGGSGDGFGVRMMQPRRM
DGDLGYEPEGSASPTPPYVRWAESLPALLEDQDGTSLFRAFLEQEDCVDLLDFWFACSGFRKTDGDKRAK
LAKAIYKKFIKDNAGIVLRQIGSITRTGVKECIASKRIDSTMFDRAQEEVQTAMEENAYPAFLRSDVYLA
YARSGGESPRCFADPLPAGFMAMKGLDSPLGLQTVKEDEVLSYDSQQTHKRWPEVHCGMRPTGGSSCFSV
ASPRAVGMRPSEKGKKGYRNPLWPGNPVNPYHMNSGYGRAPASSANDSEQSLSSDAMTDDTMSMTDSSVD
GIPPYRLRSCASRDTRQSAKDSGRTEVVHIPRTSRLPKDMVVEPAKFASELISRLERLQRERDAQEALEE
RLRRVRAEEEGEEGGPVATATCQASTTQVAAGCGGGGQMAAVPVSFLSPEPDDDPESILDEHVSRVMKTP
GCQSPGHHHGGGGAGRPRSPDSRPHPGVVRPQAALAAIKGEAGTAAAGGASSASQSHHHRTVYHHHHHHI
HHAHAASKAREQLEVEAAQRVYGGGGGGGGGVNGTAESFPYAAKSRNGAENLPPGITNTLPTSNKPGTLG
KKSSKGESGSKAEEAPLEEGPIRVLEEVPDRFRIWQWMVQGEKEAGRHAKTTTTSSGPSSKKGSEATPRA
AHAWSGVATAPQRAPAVGTQPSQPFVQDPGMPPLPAPNPLTQLEEARRRLLEEEKRSARVPPRSRQTQET
LQRNRSFQRQAGSQNPNSSPSIDLDADRELKAGKKSACQVEASDSVVVVYYFCGETIPYRTSVRGHVLTL
GHFKELLTKKGTYRYYFKKASEDFDCGVVYEEVQDDDAILPIYEEKINGKVERIE

Branchiostoma belcheri:

>XP_019639671.1 PREDICTED: axin-1-like isoform X1 [Branchiostoma belcheri]
MSIGVQEYLQDTGGSKFTEDLPTSARPPVPGEETESNHGSQHSGSSNTKSEYSTKSDYSKSDYSHSSAGTPRRTNCDLGFEPEGSASPTPPYLRWADNLQALLSDKDGSQLFGLYLENEDSKHLLDFYFAVNGYKKLDPLDDKTQRLAKVVYAKY
IKEGKGILSHRVKPGTKQYLAKNIDCKPLDISIFDQAQLEIEKFMEENAYRLFLESDIYLQYCRNGGEVSPNQVVTELANTSGNVGNLTTGAILAPLPTLHENVELSTEDTMETRTLPLTAEHLLATSQIRLRSDSRDRERLTRGGNPYHQTYAP
APATSANDSELQSLSSEQTDDTMSLTDSSIDLDGVSNHRHRRRQLKGMRQDAQRNGNVMCYPPFPQPRTQRLPKEMKMMEPKKFAELLIAKLEKVKQDRENKERLDEQLRRMEGDCGASQASGESQGASGADPTQPRLFPLMDDTTSIDDDPES
ILDEHVSRVLQTPGCRSPGRHSPVRPNKSHISGVPHHVSFPGALAGASAMAWHNRSRPKDVSKRDSGLGDSTEAETVTHYHKYYYHHHHHHPKSRDGLEKQTEMETQRKVQRDSEMYTTQSRVHQDSMGGTAESLRHDARREVVGKRGGGRKEEG
EPFLALPVETDRFGKVWDWMEEGERERYRGGVNVREPKFKRSSKTASQATHRSSGKASSSSAKKPLAYNTSRPTSMERPAVTAEVRPMQPFMQDTQMPVLPQPNPLICLEEARRRLVEKSSTKGSSRHVVDSGARKDKGKLPQSKVVPAVSDLEP
EEETRKQPVKKPASMMSVSGDNTVVGYYFCGEPIPYRTTLPGKDITLAIFKNSITKRGNYRYFFKCTSTEFGSNAVYQEVTEDEDLLPLWEGKIVGKVERIE

Drosophila melanogaster:

>AAD24886.1 D-Axin [Drosophila melanogaster]
MSGHPSGIRKHDDNECSGPRPPVPGEESRVKKMTEGVADTSKNSSPSYLNWARTLNHLLEDRDGVELFKKYVEEEAPAYNDHLNFYFACEGLKQQTDPEKIKQIIGAIYRFLRKSQLSISDDLRAQIKAIKTNPEIPLSPHIFDPMQRHVEVTIR
DNIYPTFLCSEMYILYIQQMSAQQERCTSSGATGSGSAGSSGSGGSSLAGACALPPTTASGKQQLPQLVPPGAFINLPVSSVSGPPAGTCSASGSVYGPSTSASSSGSISATDTLPRSSTLPTLHEDSVLSLCDDFEKVQMQEGGGSLGSGSVGA
GARAPDYPIRLTRDLLIATQKRRLEIRPPGAHGYVYNPSTTNTSYVPNSRVDSERASVSSGGRTDSDTMSISSCSMDGRPYIQRRHSSTESKAIRQSAMANKETNTFQVIPRTQRLHSNEHRPLKEEELVSLLIPKLEEVKRKQDLEERARERNP
GAALLTNERSSASDRAFAEAIREKFALDEDNDQDILDQHVSRVWKDQTPHRSPGTMSPCPPIPSRRRTATHDSGMVSDGAMSLSGHSMKHSKSMPDHSSCSRKLTNKWPSMNTDSGISMFSADTVTKYKDASSRSGSSTASKLEEAKRRLEDEPR
RSRRYAQPPMQHLSQQPLASFSSSGGSISLPHQPPPLPAKPPETIVVFSFCEEPVPYRIKIPGTQPTLRQFKDYLPRRGHFRFFFKTHCEDPDSPVIQEEIVNDSDILPLFGDKAMGLVKPSD

Hydra vulgaris:

>XP_002155943.3 PREDICTED: uncharacterized protein LOC100206389 [Hydra vulgaris]
MSSTALRPTPMGSEAVTINFEEKSLKSNINNKSKVANMHYIKENGSYCENFNNTSYPNFEKWKDNFQVLLEDEEGMRTFYEFLGTDQISVIFDCWYSCKTYRKRLPNKAAAKEVYSRFVRIKDSRVPISDQARNNLALRLRANDITETLLLEIEQ
EIFANLRDVCYPKFLKSDFFTFYCETNGQVAHIPSEFHRLNQGVISKMQHNLPTLTEDYADIYHTQASESGEDFHHHCNDYVPEMPTFQSRTQRMDSMKQLAPEQFAKLLTEKLERLLLDREAENFTDPQLLNQNVLSSHGLPIQRSYKPALSDV
DSQSWVMPPASVRYSVAGASSTSGSCVNIEQYPKYRNLAKDFLNAVHHGIRNHLGECQCISCQDCSRNSHHYIQESYFDSQELIKNEIFSKSNFDQSQLFNKLEYNTHNAFIQPAEPPPLVDTSKIYAWMEKNERQEKQNNYVAPSPIVRHKKLQ
NVPEAPIAQDHNMPLLPQPDTGTVLTEVKRVLEEPKSTRRSLRSHQQRPSNHVGDHMSAYSDPSYDQRHRHVGNAWSSFSDTNSVVSYNPSQISSVPSCASRLWNQYGYYEPKNELLYRGRHLSEQDGMKRNENSVNHRDRHSDRDLKSTRRSDV
HSDVSHPLSETRSERTHGSGNSKRTSKNSTTITYYFDTEPIPYRITIPSSEVTLGQFKAETKRGNFRYFFKTISAEDGEIVNEELRSDDEYLPRYKNKIIGKIEKVE

Nematostella vectensis:

>XP_001636924.1 axin-1 [Nematostella vectensis]
MSEELCSTMLRPAPTGQDSSESDFASVSEAGIVKDDDLKSSVAQLTPRRFTSRDLQENRDRLCTDEDKLAPLGFEPEGRAVSSPTYCERWANSFTDLLNDSDGSNLFYKYLRREGASILLDFSRECEAYRRMVPTSTQMRTTAKTLFQKFVYPRQ
FCELLGVKDTTRTQIAHHVNDQPADPALFDGAYAEVVSYMKKCHYVGFVNSNLYKDFTQAHRDTETLTLGERTPQYPGRYHSGYLPTLPEEKVLGFGEEIEEEQFELDQSCKDAIRYPYIRSAHDEQDARKLLHAPMKDIISQYYYPSVPLASRT
ESENQSLSSDALTDDTLSTLSMTTTEGGMTDTSDGLSIPRNSRRRRGAAHKSRNGGPLPHFPFVPRSHRMPKEAKNPLQPEEFAKILISKLEKVKLERELMEREASNLSGICESEYGQSKKSILQEAIENKQRQGKIPLCTMAMSALPPMQDDAS
SLSASNHGSSSSKSTNAQVTAGSTVRDSSSSGSTHRRSRHSHHSTKTPKQEQECADKDSDLASAQGKDPSRSNTKPANTARPDDCIPFLTLNHQRILQWMEMGEEELRQQKTQKVRHQSPSSSSSRGSSKSRQSSSTKGSEHYSNHKPSQPIAQD
PLMPPLPQPEATTVLGEVARRLVATKEMDKKSRHHHHKHRSKTSVDDISDSVSSCQEKIGSSTCSCSATDCSSCAQSMLSYDINHPAPSYIASSTLSSIPSSASNYPSDTKSKSDSGISGTTRKKEKHNATTIIYWLWGEPIAYRTSLPGKHITL
GQFKTLIMRKGEFRYFFKTKTEDRECEVVYEEVKEDKMMLPTFEGKIVGKVEKVDS

Amphimedon queenslandica:

>NP_001266244.1 axin [Amphimedon queenslandica]
MAAIVSGNDHELALEEALQRIGGEPGTDEEDDEELSVVEYDEEEQRPPAPTASVVSGPAPETTTNGDNGAGGGGRYEVPEPYIGTPSNSVPLKEVLGEYRSFMLFRRFLKDQCITRNLQFWLACEYYHTQMPLEGIKAAKAIYCRFLKSSAPLHV
SILEATKRKICTIVQLGSPPGYTLFLEAQQEVYNQMEVNELQQFLCSDSFSECSQFPTRGPTQNMYGSVSGDIGFQPSRYRNGGSLHSSDDSTSVTSFASEDVGSTHTQVLCLPHHTKSVYTPRNAHSDTEHSSSHPRRQQYAGPTTHISRNKVL
TQNQFYEIVCEKLSAVQRDRTAMKQRARNIARIAGKSYEDIMSIDWFDTPEAIKYVCFDDEGTVAGADDGLHSPPLSLPPPHKQSVGKRTTVESSDTPPSSTSGAPSHHLISFGLKEIQEALRDLDIASQVRQKRSSTLMSSSASSYISDSGVGE
SNLPSSGSGGPGPSSGHNSRLSNRVHAYLQNQQRVTSQQSEREAQAQLVATMLMNNSVPSDELNPYHNNGRRSYANYPGAYSSDDSSSCFTATSHSSNSEFFVPRRPHPLYGDSNDESRQQSHHHSRYPPGMRRLAPSNRRPPSSRYLPQQTTPN
QPPQQQLSTSNRAISPSVHPSSKSSSDTLLVAYSWEGKTYANKLQVSCLTLGEFKEKMFKRKGQYRFFFKSFCEELNDVILEEISDNSVTLPLHEGKIVGQVEGITD

Alignment

```
NP_001266244.1[A.queenslandica]      -----MAAIVSGNDHELALEEALQRIGGEPGTDEEDDEELSVVEYDEEEQRPPAPTASVV           55
XP_002155943.3[H.vulgaris]           --------------------------------------------MSSTALRPTPMGSEAV           16
AAD24886.1[D.melanogaster]           -----MS------GHPSG-------I--RKHD-----------DNECSGPRPPVPGEESR           29
XP_001636924.1[N.vectensis]          -----------------------------MSE-----------ELCSTMLRPAPTGQDSS           20
XP_019639671.1[B.belcheri]           -----MSIGVQ--EYLQD-------TGGSKFT-----------EDLPTSARPPVPGEETE           35
XP_032816874.1[P.marinus]            -----MSV--SEKLQRLD-------LSHIIEN-----------AI---TRRPPVPGREGK           32
XP_014059686.1[S.salar]              MDAVNMSVGDKVGGYLVD-------LGGSFT------------ED---APRPPVPGEEGE           38
EDL22489.1[M.musculus]               MQSPKMN--VQEQGFPLD-------LGASFT------------ED---APRPPVPGEEGE           36
NP_003493.1[H.sapiens]               -----MN--IQEQGFPLD-------LGASFT------------ED---APRPPVPGEEGE           31
XP_024089076.1[P.abelii]             -----MN--IQEQGFPLD-------LGASFT------------ED---APRPPVPGEEGE           31
NP_990275.1[G.gallus]                -----MN--IQGKGFPLD-------LGRSFT------------ED---APRPPVPGEEGE           31
NP_001089251.1[X.laevis]             -----MS--VKAKGFPLD-------LGGSFT------------ED---APRPPVPGEEGE           31
                                                                                     **     .

NP_001266244.1[A.queenslandica]      SG-------------------------------PAP-----------------ETTTNGD           67
XP_002155943.3[H.vulgaris]           TINFEEKSLKSNINNK------------SKVANM---------------------HYIKE           43
AAD24886.1[D.melanogaster]           VKKMTEG-----------------------------------------------------           36
XP_001636924.1[N.vectensis]          ---------ESDFASVSEAGIVKDDDLKSSVAQLTPRRFTSRDLQENRDRLCTDEDKLAP           71
XP_019639671.1[B.belcheri]           SNHGSQHSGSSNTKSEYSTKSDYSKSDYSHSSAGTPR------------------RTNCD           77
XP_032816874.1[P.marinus]            VNVWPEHCNDPGLARA-NGVKGGGSGDGFGVRMMQPR------------------RMDGD           73
XP_014059686.1[S.salar]              LVSSDGRPYGHGFY------SFKSEILKNEASTATPR------------------RPDLD           74
EDL22489.1[M.musculus]               LVSTDSRPVNHSFC------SGKGTSIKSETSTATPR------------------RSDLD           72
NP_003493.1[H.sapiens]               LVSTDPRPASYSFC------SGKGVGIKGETSTATPR------------------RSDLD           67
XP_024089076.1[P.abelii]             LVSTDPRPASYSFC------SGKGVGVKGETSTATPR------------------RSDLD           67
NP_990275.1[G.gallus]                LVSTDPRPVSHGFY------SSKSDAVRNETSTATPR------------------RSDLD           67
NP_001089251.1[X.laevis]             LITTDQRPFSHTYY------SLKNDGIKNETSTATPR------------------RPDLD           67

NP_001266244.1[A.queenslandica]      NGAGGGGRYEVPEPYIGTPSNSVPLKEVLGEYRSFMLFRRFLKDQC--ITRNLQFWLACE           125
XP_002155943.3[H.vulgaris]           NGSYCENFNNTSYPNFEKW--KDNFQVLLEDEEGMRTFYEFLGTDQ--ISVIFDCWYSCK           99
AAD24886.1[D.melanogaster]           ---VADTSKNSSPSYL-NW--ARTLNHLLEDRDGVELFKKYVEEEAPAYNDHLNFYFACE           90
XP_001636924.1[N.vectensis]          LGFEPEGRAVSSPTYCERW--ANSFTDLLNDSDGSNLFYKYLRREG--ASILLDFSRECE           127
XP_019639671.1[B.belcheri]           LGFEPEGSASPTPPYL-RW--ADNLQALLSDKDGSQLFGLYLENED--SKHLLDFYFAVN           132
XP_032816874.1[P.marinus]            LGYEPEGSASPTPPYV-RW--AESLPALLEDQDGTSLFRAFLEQED--CVDLLDFWFACS           128
XP_014059686.1[S.salar]              LGYEPEGSASPTPPYL-KW--AESLHSLLDDQDGIHLFRTFLKQEE--CADVLDFWFACT           129
EDL22489.1[M.musculus]               LGYEPEGSASPTPPYL-RW--AESLHSLLDDQDGISLFRTFLKQEG--CADLLDFWFACS           127
NP_003493.1[H.sapiens]               LGYEPEGSASPTPPYL-KW--AESLHSLLDDQDGISLFRTFLKQEG--CADLLDFWFACT           122
XP_024089076.1[P.abelii]             LGYEPEGSASPTPPYL-KW--AESLHSLLDDQDGISLFRTFLKQEG--CADLLDFWFACT           122
NP_990275.1[G.gallus]                LGYEPEGSASPTPPYL-KW--AESLHSLLDDQDGINLFRTFLKQED--CADLLDFWFACS           122
NP_001089251.1[X.laevis]             LGYEPEGSASPTPPYL-KW--AESLHSLLDDQDGINLFRNFLQQEN--CVDLLDFWFACS           122
                                                             :  :* :  .   *  ::  :       ::

NP_001266244.1[A.queenslandica]      YYHTQMPLEG------IKAAKAIYCRFLKSSAP--LHVSILEATKRKICTIVQLGSPPGY           177
XP_002155943.3[H.vulgaris]           TYRKRLPNKA--------AAKEVYSRFVRIKDSRVPISDQA----RNNLALRLRANDITE           147
AAD24886.1[D.melanogaster]           GLKQQTD-----PEKIKQIIGAIYRFLRK-SQ--LSISDDLRAQIK--AIKTNPEIPLSP           140
XP_001636924.1[N.vectensis]          AYRRMVPTSTQ----MRTTAKTLFQKFVYPRQ--FCELLGVKDTTRTQIAHHVNDQPADP           181
XP_019639671.1[B.belcheri]           GYKKLDPLD----DKTQRLAKVVYAKYIKEGK--GILSHRVKPGTKQYLAKNIDCKPLDI           186
XP_032816874.1[P.marinus]            GFRKTD------GDKRAKLAKAIYKKFIKDNA--GIVLRQIGSITRTGVKECIASKRIDS           180
XP_014059686.1[S.salar]              GFRKLEANEGQGEEKKLKLAKAIYKKYILDNN--GIVSRQIKPATKSFIKDCVMRLHIDP           187
EDL22489.1[M.musculus]               GFRKLEPCDS-NEEKRLKLARAIYRKYILDSN--GIVSRQTKPATKSFIKDCVMKQQIDP           184
NP_003493.1[H.sapiens]               GFRKLEPCDS-NEEKRLKLARAIYRKYILDNN--GIVSRQTKPATKSFIKGCIMKQLIDP           179
XP_024089076.1[P.abelii]             GFRKLEPCDS-NEEKRLKLARAIYRKYILDNN--GIVSRQTKPATKSFIKGCIMKQLIDP           179
NP_990275.1[G.gallus]                GFRKLEPCVS-NEEKRLKLAKAIYKKYILDNN--GIVSRQIKPATKSFIKDCVMKLQIDP           179
NP_001089251.1[X.laevis]             GFRKLEPNDS-KVEKRLKLAKAIYKKYVLDSN--GIVSRQIKPATKSFIKDCVLRQQIDA           179
                                       :                   ::                     :

NP_001266244.1[A.queenslandica]      TLFLEAQQEVYNQMEVNELQQFLCSDSFSECSQFPTRGPTQNMY-G--SVSGDIG-----           229
XP_002155943.3[H.vulgaris]           TLLLEIEQEIFANLRDVCYPKFLKSDFFTFYCETNGQVAHIPSEFHRLN-----------           196
AAD24886.1[D.melanogaster]           HIFDPMQRHVEVTIRDNIYPTFLCSEMYILYIQQMSAQQERCTSSGA-TGSGSAGSSGSG           199
XP_001636924.1[N.vectensis]          ALFDGAYAEVVSYMKKCHYVGFVNSNLYKDFTQAHRDTETLTL-------GERTP-----           229
XP_019639671.1[B.belcheri]           SIFDQAQLEIEKFMEENAYRLFLESDIYLQYCRNGGEVSPNQVVTELANTSGNVG-----           241
XP_032816874.1[P.marinus]            TMFDRAQEEVQTAMEENAYPAFLRSDVYLAYARSGGESPRCFA--DPLPAGFMAM-----           233
XP_014059686.1[S.salar]              AMFDQAQTEIQTMMEENTYPLFLKSDIYLEYTRTGGESPKLYS--DQCSVSG-GG-----           239
EDL22489.1[M.musculus]               AMFDQAQTEIQSTMEENTYPSFLKSDIYLEYTRTGSESPKVCS--DQSSGSG-TG-----           236
NP_003493.1[H.sapiens]               AMFDQAQTEIQATMEENTYPSFLKSDIYLEYTRTGSESPKVCS--DQSSGSG-TG-----           231
XP_024089076.1[P.abelii]             AMFDQAQTEIQATMEENTYPSFLKSDIYLEYTRTGSESPKVCS--DQSSGSG-TG-----           231
NP_990275.1[G.gallus]                DMFDQAQTEIQCMIEDNTYPLFLKSDIYLEYTRTGGESPKIYS--DPSSGSG-TG-----           231
NP_001089251.1[X.laevis]             AMFDQAQMEIQSMMEDNTYPVFLKSDIYLEYTTIGGESPKNYS--DQSSGSG-TG-----           231
                                      ::     .:   :.      *: *: :

NP_001266244.1[A.queenslandica]      ------------------------------------------------------------           229
XP_002155943.3[H.vulgaris]           ------------------------------------------------------------           196
AAD24886.1[D.melanogaster]           GSSLAGACALPPTTASGKQQLPQLVPPGAFINLPVSSVSGPPAGTCSASGSVYGPSTSAS           259
XP_001636924.1[N.vectensis]          ------------------------------------------------------------           229
XP_019639671.1[B.belcheri]           ------------------------------------------------------------           241
XP_032816874.1[P.marinus]            ------------------------------------------------------------           233
XP_014059686.1[S.salar]              ------------------------------------------------------------           239
EDL22489.1[M.musculus]               ------------------------------------------------------------           236
NP_003493.1[H.sapiens]               ------------------------------------------------------------           231
XP_024089076.1[P.abelii]             ------------------------------------------------------------           231
NP_990275.1[G.gallus]                ------------------------------------------------------------           231
NP_001089251.1[X.laevis]             ------------------------------------------------------------           231

NP_001266244.1[A.queenslandica]      ------FQPSRYRNGGS--LHS-----SDDSTSVTSF-------A---------------           254
XP_002155943.3[H.vulgaris]           ------QGVISKMQHNLPTLTEDYADIYHTQAS---------------------------           223
AAD24886.1[D.melanogaster]           SSGSISATDTLPRSSTLPTLHEDSVLSLCDDFEKVQMQEGGGSLGSGSVGAGARAPDYPI           319
XP_001636924.1[N.vectensis]          ------QYPGRYHSGYLPTLPEEKVLGFGEEIEEEQFELD--------------------           263
XP_019639671.1[B.belcheri]           ------NLTTGAILAPLPTLHENVELSTEDTMETRT--------L---------------           272
XP_032816874.1[P.marinus]            ------KGL--DSPLGLQTVKEDEVLSYDSQQTHKRWPEVHCGMR---------------           270
XP_014059686.1[S.salar]              ------KVL----PGYLPTLNEDEEWRCDHELEEQQESDPTPSNR---------------           274
EDL22489.1[M.musculus]               ------KGM----SGYLPTLNEDEEWKCDQDADEDDGRDPLPPSR---------------           271
NP_003493.1[H.sapiens]               ------KGI----SGYLPTLNEDEEWKCDQDMDEDDGRDAAPPGR---------------           266
XP_024089076.1[P.abelii]             ------KGI----SGYLPTLNEDEEWKCDQDMDEDDGRDAAPPGR---------------           266
NP_990275.1[G.gallus]                ------KGL----PGYLPTLNEDEEWKCDQDTEPEASRDSAPSSR---------------           266
NP_001089251.1[X.laevis]             ------KGP----SGYLPTLNEDEEWRCDQGGEQ--ERESVPSSL---------------           264
                                                        : .

NP_001266244.1[A.queenslandica]      ----------------------SEDVGS---THTQVLCLPHHTKSVYTPRNAHS--DTEH           287
XP_002155943.3[H.vulgaris]           ----------------------ESGEDFHH---H---CND------Y-------------           236
AAD24886.1[D.melanogaster]           RLTRDLLIATQKRR-----------LEIRPPGAHGYVYNPSTTNTSYVP---NSRVDSER           365
XP_001636924.1[N.vectensis]          ---QSCKD---AIRYPYIRSA-HDEQDARKLLH--APMKDIISQYYYPSVPLASRTESEN           314
XP_019639671.1[B.belcheri]           PLTAEHLLATSQIRL------RSDSRDRERLTRG---GNPYH--QTYAPAPATSANDSEL           321
XP_032816874.1[P.marinus]            PTGGSSCFSVASPRAVGMRPSEKGKKGYRNPLWPGNPVNPYHMNSGYGRAPASSANDSEQ           330
XP_014059686.1[S.salar]              -LTQKLLQETASQRVTSTKRF-QDSHEYRRVLWQ-EPINPYYVNTGYAMAPATSANDSEQ           331
EDL22489.1[M.musculus]               -LTQKLLLETAAPRAPSSRRY-NEGRELRYGSWR-EPVNPYYVNSGYALAPATSANDSEQ           328
NP_003493.1[H.sapiens]               -LPQKLLLETAAPRVSSSRRY-SEGREFRYGSWR-EPVNPYYVNAGYALAPATSANDSEQ           323
XP_024089076.1[P.abelii]             -LPQKLLLETAAPRVSSSRRY-SEGREFRYGSWR-EPVNPYYVNAGYALAPATSANDSEQ           323
NP_990275.1[G.gallus]                -LTQKLLLETATQRATSTRRY-SEGREFRHGSWR-EPVNPYYVNTGYAMAPATSANDSEQ           323
NP_001089251.1[X.laevis]             -FSQKLILDSSSHCAGSNRRL-SDGREFRPGTWR-EPVNPYYVNTGYSGAPVTSANDSEQ           321
                                                                                   *
```

```
NP_001266244.1[A.queenslandica]       SSSH--------------------------P----------RR-------------QQ          296
XP_002155943.3[H.vulgaris]            ------------------------------VPE-------------------------          239
AAD24886.1[D.melanogaster]            ASVSSGGRTDSDTMSI----------SSCSMDGRPYIQR-RHSSTESKAIRQSAMANKET          414
XP_001636924.1[N.vectensis]           QSLSSDA-LTDDTLSTLSMTTTEGGMTDTS-DGLSIPRNSRRR----RGAAHKSRNGGPL          368
XP_019639671.1[B.belcheri]            QSLSSE--QTDDTMSL----------TDSSIDLDGVSNHRHRRRQLKGMRQDAQRNGNV          369
XP_032816874.1[P.marinus]             SLSSDA--MTDDTMSM----------TDSSVDGIPPYRLRSCAS---RDTRQSAKDSGRT          375
XP_014059686.1[S.salar]               QSMSSA--SDVDTLSL----------TDSSVDGVPPYKYRKQHR---REMHESAKANGRV          376
EDL22489.1[M.musculus]                QSLSS----DADTLSL----------TDSSVDGIPPYRIRKQHR---REMQESIQVNGRV          371
NP_003493.1[H.sapiens]                QSLSS----DADTLSL----------TDSSVDGIPPYRIRKQHR---REMQESVQVNGRV          366
XP_024089076.1[P.abelii]              QSLSS----DADTLSL----------PDSSVDGIPPYRIRKQRR---REMQESVQVNGRV          366
NP_990275.1[G.gallus]                 QSMSS----DADTMSL----------TDSSIDGIPPYRLRKQHR---REMQESAKANGRV          366
NP_001089251.1[X.laevis]              QSMSS----DADTMSL----------TDSSVDGIPPYRLRKHYR---REMQESANANGRG          364

NP_001266244.1[A.queenslandica]       YAGPTTHISRN------K--VLTQNQFYEIVCEKLSAVQRDRTAMKQRARNIARIAGKSY          348
XP_002155943.3[H.vulgaris]            ---MPTFQSRTQRMDSMKQ--LAPEQFAKLLTEKLERLLLDREAENFTDPQLLNQNVLS-          293
AAD24886.1[D.melanogaster]            --NTFQVIPRTQRLHSNEHRPLKEEELVSLLIPKLEEVKRKQDLEERARERNPGAALLTN          472
XP_001636924.1[N.vectensis]           --PHFPFVPRSHRMPKEAKNPLQPEEFAKILISKLEKVKLERELMEREASNLSGI---CE          423
XP_019639671.1[B.belcheri]            MCYPPFPQPRTQRLPKEMK-MMEPKKFAELLIAKLEKVKQDRENKERLDEQLRRMEGDCG          428
XP_032816874.1[P.marinus]             ---EVVHIPRTSRLPKDMV--VEPAKFASELISRLERLQRERDAQEALEERLRRVRAE--          428
XP_014059686.1[S.salar]               ---PLPHIPRTNRIPKDIH--VEPEKFAADLISRLEGVQREREAQEKLEERLKRVRLE--          429
EDL22489.1[M.musculus]                ---PLPHIPRTYRMPKEIR--VEPQKFAEELIHRLEAVQRTREAEEKLEERLKRVRMVCP          426
NP_003493.1[H.sapiens]                ---PLPHIPRTYRVPKEVR--VEPQKFAEELIHRLEAVQRTREAEEKLEERLKRVRME--          419
XP_024089076.1[P.abelii]              ---PLPHIPRTYRVPKEVR--VEPQKFAEELIHRLEAVQRTREAEEKLEERLKRVRME--          419
NP_990275.1[G.gallus]                 ---PLPHIPRTYRMPKDIH--VEPEKFAAELINRLEEVQKEREAEEKLEERLKRVRAE--          419
NP_001089251.1[X.laevis]              ---PLPHIPRTYHMPKDIH--VDPEKFAAELINRLEGVLRDREAEQRLEERLKRVRAE--          417
                                               *.          :   ::   :  :*. :   :   :    .

NP_001266244.1[A.queenslandica]       EDIMSIDWFDTPEAIKYVCFDDEGTVAGADDGL----HSP--------------------          384
XP_002155943.3[H.vulgaris]            ------------------------------------SHGLPIQ----RSYKP--------          305
AAD24886.1[D.melanogaster]            ERS----------------------SA----SDRA-------------------------          481
XP_001636924.1[N.vectensis]           SEY--------------------GQSK---------------------------------          430
XP_019639671.1[B.belcheri]            ASQAS------------------GESQGASGADPT-------------------------          445
XP_032816874.1[P.marinus]             ---------------------EEGEEGGPVATATCQA-------------STTQVAAGCG          454
XP_014059686.1[S.salar]               ---------------------EEGEDADISTSTSFSSHRLPPGGHPQHYNNPRYSDIGYS          468
EDL22489.1[M.musculus]                YSCSDSTA--------VSPQEEEGEDGEMPSGP-MASHKLPSVPA-WHHFPPRYVDMGCS          476
NP_003493.1[H.sapiens]                ---------------------EEGEDGDPSSGPPGPCHKLPPAPA-WHHFPPRCVDMGCA          457
XP_024089076.1[P.abelii]              ---------------------EEGEDGDPSSGPPGPCHKLPPAPA-WHHFPPRCVDVGCA          457
NP_990275.1[G.gallus]                 ---------------------EEGEDADISSGPSVISHKMPSAQP-FHHFAPRYSEMGCA          457
NP_001089251.1[X.laevis]              ---------------------EEGDDGDVSSGPSIISHKLPSGPP-MHHYNSRYSDSGCV          455

NP_001266244.1[A.queenslandica]       --------------------PLSLPPPH---KQSVGKR---------------TT-----          401
XP_002155943.3[H.vulgaris]            --------------ALSDVD------------SQSWVM------P----PASVRY-----          324
AAD24886.1[D.melanogaster]            -----FAEAIREKFALDEDNDQDILDQH---VSRVWKDQTPHRSPGTMSPCPPIPS----          529
XP_001636924.1[N.vectensis]           ---------------------KSILQEAIENKQRQGKI------PLCTMAMSALPP----          459
XP_019639671.1[B.belcheri]            ---QPRLFPLMDDTTSIDDDPESILDEH---VSRVLQT------PGCRSPGRHSPV----          489
XP_032816874.1[P.marinus]             GGGQMAAVPVSFLSPEPDDDPESILDEH---VSRVMKT------PGCQSPGHHHGGGGAG          505
XP_014059686.1[S.salar]               G--------LALRPDAHEENPESILDDH---VQRVMKT------PGCQSPGTGRHS---P          508
EDL22489.1[M.musculus]                G--------L--R-DAHEENPESILDEH---VQRVMRT------PGCQSPGPGHR-----          511
NP_003493.1[H.sapiens]                G--------L--R-DAHEENPESILDEH---VQRVLRT------PGRQSPGPGHR-----          492
XP_024089076.1[P.abelii]              G--------L--R-DAHEENPESILDEH---VQRVLRT------PGRQSPGPGHR-----          492
NP_990275.1[G.gallus]                 G--------MQMR-DAHEENPESILDEH---VQRVMKT------PGCQSPGPGRHS---P          496
NP_001089251.1[X.laevis]              G--------MQIR-DAHEENPESILDEH---VQRVMKT------PGCQSPGTGRHS---P          494
                                                                      .

NP_001266244.1[A.queenslandica]       -----------------VESSDTPPSSTSG-------------------APSH-----          418
XP_002155943.3[H.vulgaris]            ---------------------SVAGASSTSGSCVNIEQYPKYRNLAKDFL-NAV------          356
AAD24886.1[D.melanogaster]            --RRRTATH------------------------------DSGMVSDGAMSLSGHSM---          553
XP_001636924.1[N.vectensis]           --MQDDASSLSA---------SNHGSSSSKSTNAQV---TAGSTVRDSSSSGSTHRRS--          503
XP_019639671.1[B.belcheri]            ---RPNKSHISGVPHHVSFPGALAGASAMAWHNRSR---PKDVSKRDSGLGDSTEAETVT          543
XP_032816874.1[P.marinus]             RPRSPDSRPHPG------VVRPQAALAAIKGEAGTA---AAGGASS-AS--QSHHHRTVY          553
XP_014059686.1[S.salar]               KSRSPDGFPGGK------VPGPGMPPPT--GPGKHP---ARLGPKGEAA--SHQYHHKH-          554
EDL22489.1[M.musculus]                ---SPDSGHVAK------TA-VLGGTAS--GHGKHV---PKLGLKLDTA--GLHHHRHV-          553
NP_003493.1[H.sapiens]                ---SPDSGHVAK------MPVALGGAAS--GHGKHV---PKSGAKLDAA--GLHHHRHV-          535
XP_024089076.1[P.abelii]              ---SPDSGHVAK------MPVALGGAAS--GHGKHA---PKSGVKLDAA--GLHHHRHV-          535
NP_990275.1[G.gallus]                 KPRSPESGHLGK------LSGTLGTIPP--GHGKHT---TKSGMKLDAA--NLYHHKHV-          542
NP_001089251.1[X.laevis]              KPRSPDGHLSKT------LPGSLGTMQT--GHGKHS---SKSGSKVDSG--NLHHHKHV-          540

NP_001266244.1[A.queenslandica]       -----HLISFGLKE-IQEALR-DLDIASQVRQKRSSTL------MSSSASSYISDSGVGE          465
XP_002155943.3[H.vulgaris]            ----HHGIRNHLGE--------CQCISCQDCSRN--------------SHHYIQES--YF          388
AAD24886.1[D.melanogaster]            ---------KHSKSMPD-------HSSCSRKLTN--KWP---------------------          574
XP_001636924.1[N.vectensis]           ----RH--SHHSTKTPKQ------EQECAD-----------------------KDSDLA          527
XP_019639671.1[B.belcheri]            HYHKYYYHHHHHHPKSRDGLEKQTEMETQRKVQR-------------DSEMYTTQSRVHQ          590
XP_032816874.1[P.marinus]             HHHHHHIHHAHAASKARE----QLEVEAAQRVYGGGGGGGGGVNGTAESFPYAAKSRNGA          609
XP_014059686.1[S.salar]               ----VHHIHHPAGGKPKE----QVEADAAMRVNGNFHW-------GTEQHNYGSKSRNYA          599
EDL22489.1[M.musculus]                ----HHHVHHN-SARPKE----QMEAEVARRVQSSFSW-------GPETHGHA-KPRSYS          596
NP_003493.1[H.sapiens]                ----HHHVHHS-TARPKE----QVEAEATRRAQSSFAW-------GLEPHSHGARSRGYS          579
XP_024089076.1[P.abelii]              ----HHHVHHS-TGRPKE----QVEAEATRRAQSSFAW-------GPEPHSHGAKSRGYS          579
NP_990275.1[G.gallus]                 ----YHHIHHHSMMKPKE----QIEAEATQRVQNSFAW-------NVDSHNYATKSRNYS          587
NP_001089251.1[X.laevis]              ----YHHLHHHGGVKPKE----QIEAEVTQRVQTNYPW-------NAESHNYATKSRNYA          585

NP_001266244.1[A.queenslandica]       SNLPSSGSGGPGPSS-----GHNSRLSNRVHAYLQNQQ-------R-----VTSQQSERE          508
XP_002155943.3[H.vulgaris]            DSQ----EL-----------IKNEIFSKSNFDQSQLFNKLEYNTHNAFI--QPAEPPPLV          431
AAD24886.1[D.melanogaster]            -SM----NTD------------------------SG-------------ISMFSAD--          588
XP_001636924.1[N.vectensis]           SAQ----GKDPS---------------RSNTKPANTA--------RP----DDCIPFLTL          556
XP_019639671.1[B.belcheri]            DSM----GGT---AESLRHDARREVVGKRGGGRKE--------EGEP----FLALPVETD          631
XP_032816874.1[P.marinus]             ENL----P--PGITNTLPTSNKPGTLGKKSSKGESGSK----AEEAPLEEGPIRVLEEVP          659
XP_014059686.1[S.salar]               DGT------GPSPMDPMGYSSKGSTLSKRSFKKAEEV--------RTFE--VPVPTEDVE          643
EDL22489.1[M.musculus]                ENA----GTTLS-AGDLAFGGKTSAPSKRNTKKAESGK----N--ANAE--VPSTTEDAE          643
NP_003493.1[H.sapiens]                ESV----GAAPNASDGLAHSGKVGVACKRNAKKAESGK----S--ASTE--VPGASEDAE          627
XP_024089076.1[P.abelii]              ESV----GAAPNASDGLAHSGKAGVACKRNAKKAESGK----S--ASTE--VPGASEDVE          627
NP_990275.1[G.gallus]                 ENL----GMAPVPMDSLGYSGKASLLSKRNIKKTDSGK----SDGANYE--MPGSPEDVE          637
NP_001089251.1[X.laevis]              ESM----GMAPNPMDSLAYSGKVSMLSKRNAKKADVGK----SESASYE--VPAVPEDAE          635

NP_001266244.1[A.queenslandica]       AQAQLVATMLMNNSVPSDE-LN----PYHNNGR---RSYANYPGAYSSDDSSSCFTATSH          560
XP_002155943.3[H.vulgaris]            DTSKIYAWMEKNERQEKQNNYVA-------------------------------------          454
AAD24886.1[D.melanogaster]            -------------------TVT----KYKD--------------AS--------------          597
XP_001636924.1[N.vectensis]           NHQRILQWMEMGEEELRQQKTQKVR--------------HQSPSSSSSRGSSK-------          595
XP_019639671.1[B.belcheri]            RFGKVWDWMEEGERERYRGGVNVREPKFKRSSKTASQATHRSSGKASSSSAKKP-LAYNT          690
XP_032816874.1[P.marinus]             DRFRIWQWMVQGEKEAGRHAKT----------------------TTTSSGPSSK------          691
XP_014059686.1[S.salar]               RNQKILQWMMEGEAVRNKKSPY----------------------GSTTGSKKTP-SSHEV          680
EDL22489.1[M.musculus]                KNQKIMQWIIEGEKEISRHRKA----------------------GHGSSGLRKQ-QAHES          680
NP_003493.1[H.sapiens]                KNQKIMQWIIEGEKEISRHRRT----------------------GHGSSGTRKP-QPHEN          664
XP_024089076.1[P.abelii]              KNQKIMQWIIEGEKEISRHRRT----------------------GHGSSGTRKP-QPHEN          664
NP_990275.1[G.gallus]                 RNQKILQWIIEGEKEISRHKKT----------------------NHGSSGVKKQ-LSHDM          674
NP_001089251.1[X.laevis]              RHQKILQWIMEGEKEISRHKKS----------------------NHSSSSAKKQ-PPPEL          672

NP_001266244.1[A.queenslandica]       SSNSEFFVPRRPHPL--------------------YGDSNDESRQQSHHHSRYPPGMRR          599
```

```
XP_002155943.3[H.vulgaris]           --PSPIV----------------RHKKLQNVPEAPIAQDHNMPLLPQPDTGTVLTEVKRV          496
AAD24886.1[D.melanogaster]           SR-------------------------------------------SGSSTASKLEEAKRR          614
XP_001636924.1[N.vectensis]          SRQSS-STKG-------------SEHYSNHKPSQPIAQDPLMPPLPQPEATTVLGEVARR          641
XP_019639671.1[B.belcheri]           SRPTSMERPAV---------------TAEVRPMQPFMQDTQMPVLPQPNPLICLEEARRR          735
XP_032816874.1[P.marinus]            --KGSEATPRAAHAWSGVATAPQRAPAVGTQPSQPFVQDPGMPPLPAPNPLTQLEEARRR          749
XP_014059686.1[S.salar]              SRPSSVERPGAVHPWVSAQL------RNNVQPSHPFIQDPTMPPNPAPNPLTQLEEARRR          734
EDL22489.1[M.musculus]               SRPLSIERPGAVHPWVSAQL------RNSVQPSHLFIQDPTMPPNPAPNPLTQLEEARRR          734
NP_003493.1[H.sapiens]               SRPLSLE-----HPWAGPQL------RTSVQPSHLFIQDPTMPPHPAPNPLTQLEEARRR          713
XP_024089076.1[P.abelii]             SRPLSLERPGAVHPWAGPQL------RTSVQPSHLFIQDPTMPPHPAPNPLTQLEEARRR          718
NP_990275.1[G.gallus]                VRPLSIERPVAVHPWVSAQL------RNVVQPSHPFIQDPTMPPNPAPNPLTQLEEARRR          728
NP_001089251.1[X.laevis]             SRPLSIERPGAVHPWVSAQL------RNVVQPSHPFIQDPTMPPNPAPNPLTQLEEARRR          726
                                                                                               *

NP_001266244.1[A.queenslandica]      LAPSNRRPPSSRYLPQQTTP----------------------------------------          619
XP_002155943.3[H.vulgaris]           LEEPKSTRRSLRSHQQ--RPSNHVGDHMSAYSDPSYDQRHRHVGNAWSSFSDTNSVVSYN          554
AAD24886.1[D.melanogaster]           LEDEPRRSRRYAQPPMQHLSQQPLASFSSSG-----GS----------------------          647
XP_001636924.1[N.vectensis]          LVATKEMDKKSRHHHHKHRSKTSVDDISDSV-----SSCQEKIGSSTCSCSA--------          688
XP_019639671.1[B.belcheri]           LVEKSSTKGSS-------RH---V---VD-S-----GARKDKG-----------------          759
XP_032816874.1[P.marinus]            LLEEEKRSARVPP---RSRQ---TQETLQRN-----RSFQRQA-----------------          781
XP_014059686.1[S.salar]              LEEEKKKTGTLQT---KQRY---VMEVIQRG-----RAAARPA-----------------          766
EDL22489.1[M.musculus]               LEEEEKRANKLPS---KQRY---VQAVMQRG-----RTCVRPA-----------------          766
NP_003493.1[H.sapiens]               LEEEEKRASRAPS---KQRY---VQEVMRRG-----RACVRPA-----------------          745
XP_024089076.1[P.abelii]             LEEEEKRASRAPS---KQRY---VQEVMRRG-----RACVRPA-----------------          750
NP_990275.1[G.gallus]                LEEEEKRAGKLPL---KQR-----------------------------------------          744
NP_001089251.1[X.laevis]             LEEEEKRAAKMPQ---KQRY---VQEVIQRG-----RCSVRPA-----------------          758
                                     *

NP_001266244.1[A.queenslandica]      ------------------------------------------------------------          619
XP_002155943.3[H.vulgaris]           PSQISSVPSCASRLWNQYGYYEPKNELLYRGRHLSEQDGMKRNENSVNHRDRHSDRDLKS          614
AAD24886.1[D.melanogaster]           ------------------------------------------------------------          647
XP_001636924.1[N.vectensis]          ----TDCSSCAQSML-SYDINHPAPSYIA------------------------------S          713
XP_019639671.1[B.belcheri]           ------KL----------------------------------------------------          761
XP_032816874.1[P.marinus]            ------GS----------------------------------------------------          783
XP_014059686.1[S.salar]              ------LF----------------------------------------------------          768
EDL22489.1[M.musculus]               ------CA----------------------------------------------------          768
NP_003493.1[H.sapiens]               ------CA----------------------------------------------------          747
XP_024089076.1[P.abelii]             ------CA----------------------------------------------------          752
NP_990275.1[G.gallus]                ------------------------------------------------------------          744
NP_001089251.1[X.laevis]             ------YI----------------------------------------------------          760

NP_001266244.1[A.queenslandica]      ----NQP-------PQQQLSTSNRAISPSVHPSSKSSSDTLLVAYSWEGK--TYANKLQV          666
XP_002155943.3[H.vulgaris]           TRRSDVHSDVSHPLSE---TRSERTHGSGN--SKRTSKNSTTITYYFDTEPIPYRITIPS          669
AAD24886.1[D.melanogaster]           -----------ISLPH----Q------PP---PLPAKPPETIVVFSFCEEPVPYRIKIPG          683
XP_001636924.1[N.vectensis]          STLSSIPSSASNYPSD-TKSKSDSGISGTT--RKKEKHNATTIIYWLWGEPIAYRTSLPG          770
XP_019639671.1[B.belcheri]           PQSKVVPAVSDLEPEEETRKQPVKK--PAS--MMSVSGDNTVVGYYFCGEPIPYRTTLPG          817
XP_032816874.1[P.marinus]            QNPNSSPSID---LDADRELKA-GK--KSA--CQVEASDSVVVVYYFCGETIPYRTSVRG          835
XP_014059686.1[S.salar]              PPLSVVPAVSDTELSE-SEHKVTKK----------PACENITVAYYFCGEPIPYRTSVKG          817
EDL22489.1[M.musculus]               PVLSVVPAVSDLELSE-TETKSQRK--AGG--GSAPPCDSIVVAYYFCGEPIPYRTLVRG          823
NP_003493.1[H.sapiens]               PVLHVVPAVSDMELSE-TETRSQRK--VGG--GSAQPCDSIVVAYYFCGEPIPYRTLVRG          802
XP_024089076.1[P.abelii]             PVLRVVPAVSDMELSE-TETRSQRK--VSG--GSAQPCDSIVVAYYFCGEPIPYRTLVRG          807
NP_990275.1[G.gallus]                -------------------LKPQKR--PGS--GASQPCENIVVAYYFCGEPIPYRTLVKG          781
NP_001089251.1[X.laevis]             PLLNVVPAVSDMDLSE-AELKPQKK--TVS--SPSQPCENIVVAYYFCGEPIPYRTMVKG          815
                                                                              : :    :   *   :

NP_001266244.1[A.queenslandica]      SCLTLGEFKEKMFKRKGQYRFFFKSFCEELN-DVILEEISDNSVTLPLHEGKIVGQVEGI          725
XP_002155943.3[H.vulgaris]           SEVTLGQFKA--ETKRGNFRYFFKTISAE-DGEIVNEELRSDDEYLPRYKNKIIGKIEKV          726
AAD24886.1[D.melanogaster]           TQPTLRQFKDY-LPRRGHFRFFFKTHCEDPDSPVIQEEIVNDSDILPLFGDKAMGLVKPS          742
XP_001636924.1[N.vectensis]          KHITLGQFKTL-IMRKGEFRYFFKTKTEDRECEVVYEEVKEDKMMLPTFEGKIVGKVEKV          829
XP_019639671.1[B.belcheri]           KDITLAIFKNS-ITKRGNYRYFFKCTSTEFGSNAVYQEVTEDEDLLPLWEGKIVGKVERI          876
XP_032816874.1[P.marinus]            HVLTLGHFKEL-LTKKGTYRYYFKKASEDFDCGVVYEEVQDDDAILPIYEEKINGKVERI          894
XP_014059686.1[S.salar]              RMVMLGQFKEL-LTKKGSYRYFFKKVSNDF--GVVFEEVREDDAILPIFEEKIIGKVEKI          874
EDL22489.1[M.musculus]               RAVTLGQFKEL-LTKKGSYRYYFKKVSDEFDCGVVFEEVREDEAVLPVFEEKIIGKVEKV          882
NP_003493.1[H.sapiens]               RAVTLGQFKEL-LTKKGSYRYYFKKVSDEFDCGVVFEEVREDEAVLPVFEEKIIGKVEKV          861
XP_024089076.1[P.abelii]             RAVTLGQFKEL-LTKKGSYRYYFKKVSDEFDCGVVFEEVREDEAILPVFEEKIIGKVEKV          866
NP_990275.1[G.gallus]                RVVTLGQFKEL-LTKKGNYRYYFKKVSDEFDCGVVFEEVREDDTILPIFEEKIIGKVEKI          840
NP_001089251.1[X.laevis]             RVVTLGQFKEL-LTKKGNYRYYFKKVSDEFDCGVVFEEVREDDMILPIYEEKIIGKVEKI          874
                                         *  **     ::* :*::**    :    : :*: .:.  **    *  * ::

NP_001266244.1[A.queenslandica]      TD         727
XP_002155943.3[H.vulgaris]           E-         727
AAD24886.1[D.melanogaster]           D-         743
XP_001636924.1[N.vectensis]          DS         831
XP_019639671.1[B.belcheri]           E-         877
XP_032816874.1[P.marinus]            E-         895
XP_014059686.1[S.salar]              D-         875
EDL22489.1[M.musculus]               D-         883
NP_003493.1[H.sapiens]               D-         862
XP_024089076.1[P.abelii]             D-         867
NP_990275.1[G.gallus]                D-         841
NP_001089251.1[X.laevis]             D-         875
```
