# Supplementary material for: An Evolutionary Perspective on the Origin, Conservation and Binding Partner Acquisition of Tankyrases
Source: Biomolecules. 2022 Nov 15;12(11):1688. doi: 10.3390/biom12111688 (PMC9688111; doi:10.3390/biom12111688)
Supplement: Supplementary file 1 [file biomolecules-12-01688-s001.zip › biomolecules-1913719-supplementary-11.17 -done/Data_7/CASC3.pdf]

CASC3

Homo sapiens:

>NP_031385.2 protein CASC3 [Homo sapiens]
MADRRRQRASQDTEDEESGASGSDSGGSPLRGGGSCSGSAGGGGSGSLPSQRGGRTGALHLRRVESGGAK
SAEESECESEDGIEGDAVLSDYESAEDSEGEEGEYSEEENSKVELKSEANDAVNSSTKEEKGEEKPDTKS
TVTGERQSGDGQESTEPVENKVGKKGPKHLDDDEDRKNPAYIPRKGLFFEHDLRGQTQEEEVRPKGRQRK
LWKDEGRWEHDKFREDEQAPKSRQELIALYGYDIRSAHNPDDIKPRRIRKPRYGSPPQRDPNWNGERLNK
SHRHQGLGGTLPPRTFINRNAAGTGRMSAPRNYSRSGGFKEGRAGFRPVEAGGQHGGRSGETVKHEISYR
SRRLEQTSVRDPSPEADAPVLGSPEKEEAASEPPAAAPDAAPPPPDRPIEKKSYSRARRTRTKVGDAVKL
AEEVPPPPEGLIPAPPVPETTPTPPTKTGTWEAPVDSSTSGLEQDVAQLNIAEQNWSPGQPSFLQPRELR
GMPNHIHMGAGPPPQFNRMEEMGVQGGRAKRYSSQRQRPVPEPPAPPVHISIMEGHYYDPLQFQGPIYTH
GDSPAPLPPQGMLVQPGMNLPHPGLHPHQTPAPLPNPGLYPPPVSMSPGQPPPQQLLAPTYFSAPGVMNF
GNPSYPYAPGALPPPPPPHLYPNTQAPSQVYGGVTYYNPAQQQVQPKPSPPRRTPQPVTIKPPPPEVVSR
GSS

Pongo abelii:

>XP_024090145.1 protein CASC3 [Pongo abelii]
MADRRRQRASQDTEDEESGASGSDSGGSPLRGGGSCSGSAGGGGSGSLPSQRGGRIGALHLRRVESGGAK
SAEESECESEDGIEGDAVLSDYESAEDSEGEEGEYSEEENSKVELKSEANDAVNSSTKEEKGEEKPDTKS
TVTGERQSGDGQESTEPVENKVGKKGPKHLDDDEDRKNPAYIPRKGLFFEHDLRGQTQEEEVRPKGRQRK
LWKDEGRWEHDKFREDEQAPKSRQELIALYGYDIRSAHNPDDIKPRRIRKPRYGSPPQRDPNWNGERLNK
SHRHQGLGGTLPPRTFINRNAAGTGRMSAPRNYSRSGGFKEGRAGFRPVEAGGQHGGRSGETVKHEISYR
SRHLEQTPVRDPSPEADAPVLGSPEKEEAASPAAAPDAAPPPPDRPIEKKSYSRARRTRTKVGDAVKLAE
EVPPPPEGLIPAPPVPETTPTPPTKTGNWEAPVDSTTGGLEQDVAQLNIAEQSWSPGQPSFLQPRELRGM
PNHIHMGAGPPPQFNRMEEMGVQGGRAKRYSSQRQRPVPEPPAPPVHINIMEGHYYDPLQFQGPIYTHGD
SPAPLPPQGMLVQPEMHLPHPGLHPHQTPAPLPNPGLYPPPVSMSPGQPPPQQLLAPTYFSAPGVMNFGN
PSYPYAPGALPPPPPPHLYPNTQAPSQVYGGVTYYNPAQQQVQPKPSPPRRTPQPVTIKPPPPEVVSRGS
S

Mus musculus:

>NP_619601.2 protein CASC3 [Mus musculus]
MADRRRQRASQDTEDEESGASGSDSGSPARGGGSCSGSVGGGGSGSLPSQRGGRGGGLHLRRVESGGAKS
AEESECESEDGMEGDAVLSDYESAEDSEGEEDYSEEENSKVELKSEANDAADSSAKEKGEEKPESKGTVT
GERQSGDGQESTEPVENKVGKKGPKHLDDDEDRKNPAYIPRKGLFFEHDLRGQTQEEEVRPKGRQRKLWK
DEGRWEHDKFREDEQAPKSRQELIALYGYDIRSAHNPDDIKPRRIRKPRFGSSPQRDPNWIGDRSSKSHR
HQGPGGNLPPRTFINRNTAGTGRMSASRNYSRSGGFKDGRTSFRPVEVAGQHGGRSAETLKHEASYRSRR
LEQTPVRDPSPEPDAPLLGSPEKEEVASETPAAVPDITPPAPDRPIEKKSYSRARRTRTKVGDAVKAAEE
VPPPSEGLASTATVPETTPAAKTGNWEAPVDSTTGGLEQDVAQLNIAEQSWSPSQPSFLQPRELRGVPNH
IHMGAGPPPQFNRMEEMGVQSGRAKRYSSQRQRPVPEPPAPPVHISIMEGHYYDPLQFQGPIYTHGDSPA
PLPPQGMIVQPEMHLPHPGLHPHQSPGPLPNPGLYPPPVSMSPGQPPPQQLLAPTYFSAPGVMNFGNPNY
PYAPGALPPPPPPHLYPNTQAPPQVYGGVTYYNPAQQQVQPKPSPPRRTPQPVSIKPPPPEVVSRGSS

Gallus gallus:

>XP_046789397.1 protein CASC3 isoform X6 [Gallus gallus]
MADRRRQRASQDSEDDSDSAASDSGDSAASAARSRSGSASPRPSHRPPRGAAGALSAGPRGRGADSAAGG
GAAKSAPQSECESEDGIEGDAVLSDYESAEDSEAEEEDYSEEESAKVELKQDSNGSCESAAKAEKGDEKP
DSKGAVTGERQSGDGQESTEPVENKVGKKAPKHLDDDEDRKNPAYIPRKGLFFEHDLRGQTQEEEVRPKG
RQRKLWKDEGRWEHDKFREDEQAPKTRQELIALYGYDIRSAHNPDDIRPRRMRKPRFGSPQRDPNWSNER
PNKPPRHQGTDGTSAPPRTFTSRSSAGTGRMPPPRNYPRVGGYKESRPGYRASEVSAQPSRNGEQSKQES
GYRAKRAEQSPPRDKSPEVEAVHIHGSPVKEEGALENQPDAAQPPPDRPIEKKSYSRARRTRVKAGDAGK
LADDVPALEAPATPKPIQAETSPPPAKSSNWESPVESGLDGLEQEMIQMNLTEQNWSPGQSQFIQPRELR
GIPNHMHVGTGPPPQFNRMEEMAVQGGRVKRYSSQRQRPPVPEPAPPMHISIMEGHYYDPLQFQGPIYTH
GENPAPLPPQGMIVQPEMHLPHPGLHPHQTPAPMANPGLYPPPVSMPPGQPPPQQLLAPTYFSPPGVMNF
GNPGYPYPPGALPPPPPPHLYSNTQAQSQVYGGVTYYNTVQQQVQPKPSPPRRTSQPVTIKPPPPEVVSR
APVNLSF

Xenopus laevis:

>AAH47246.1 MGC54001 protein [Xenopus laevis]
MADRRRRRRRASQDSEELGEEEESASDSAGSGGESVGPIRQERAEQSKTEPARAEQETRRAEPAREGKES
ECESEDGIEGDAVLSDYESADESEEEEAHNSEEDPLKTTLKQENNVEEAPATREQKPKSKGAVTGERQSG
DGQESTEPEENNTSKKSQKQLDDDEDRKNPAYIPRKGLFFEHDLRGHVNDEEVRPKGRHPRKLWKDEGRW
VHDRFREDEQAPKSREELVSIYGYDIRSCRNPEEIHPRRPGKPRFSSPSRREENDEKPSRPSNRYQDSGV
TQPLRPYTNRNAPPSSKVGPSRTYSRQGGYKENRASYQSEEEAAPHTYERRQDYGGHRSRSTDQGPPPPR
EYSPEADPIIKEEAFTEKQTAEPSPPPPDRPVEKKSYSRARRSRIKVGDAGKSMEDTTAAELPPPPPVPP
AVAAEFTPAPLNVKQGNWEPPAEGGMSGIDEELSKMNLSEQSWNQGQPAYISPRGIPNPMHMGAGPPQYS
RMEGMAVQAGRVKRYSSQRQRPVPDPAAMHISLMESHYYDSLQFQGPIYAHGDSSPSMPPQGMIVQPEMH
LSHPGMHPHQSPATISTPNIYPAPVSLTPGQPPPQQLLPPPYFTAPSNVMNFGNPTYPYPPGALPPPPAH
LYPNAQAQSQVYGGVTYYNPVQQQVQQQVLPKPSPPRRTSQPVTIKPPPPEPQSLPVALTF

Salmo salar:

>XP_014047999.1 protein CASC3-like [Salmo salar]
MADRRRRRRRASQDSEEDDESGSGSESGKSLSPTTKPRVRDPEPVEAPAVRVVPKNDAESECESEDGVGE
AVLSDYESADPEENGSHSEGGEEEEEVVPPPAAEPKPSPAADTPAAEGELQEGGGKEEGEKGVSKEVKCE
DKGNLAGERQSGDGQESTEDSENKGAKPGQKLDDDQDRNNPAYIPRKGMFFEHDVRGNAQEEERPKGRNR
KLWKDEGRWEHDRFREEEQAPKSREELVAIYGYDIRNGGVSNERSYRQRKPRHSTSPVRDKRWRDGDGER
PIHTSWQQGGNSNSRSAPLAVALQQSGPPPSAAPNTQRNNNPPRPSSHPPPRGFQGNRPPQAQYRNDRNQ
ESQRPGPKAHPAEPLQTRSLPPMDGERGPRGRGNRGAHTERSSSVVVEEIRSEEDDERNTTTVTTSVYHN
RHYSGKGDRERDSAPRRQEQQRGGSAPPADNPVTRDASPAPERPVEKKSYSLARRTRTRATELDKQASLE
EPASTASSSALTSEPWQGPGQSQGDAGDSSQATVLTGLDQDLARLSLAGQNWAQNPSSFLRAEMRGIPNS
MHMGGAPPQYSNIEELGAGNRAKRYSSQRQRPSPEPAPPMHIGVMESHYYEPMSYQGPIYAHGDSPAPLP

PQGMLVQPEMHLPHPGHPGHPGLHPHQSSGPMPNPALYAAPPVSMSPGQPPPQQLLAPPFYPPPGVMTFG
NTNYPYPAGATLPPMYNPPAQSQVYGQSQVYGGVTYYDPMQQQAQPKPSPPRRSSQPVTVKPPPPEVGYG
PE

Petromyzon marinus:

>XP_032831431.1 protein CASC3 isoform X2 [Petromyzon marinus]
MADRRRRTGASQDSDEEEEDEEEESDLSPGRGPEKAAAIPARAAESECESEGVPERDGVVLSGYESADDN
YSHYSEDEKEEEEGSCSVTDDEVAEEGSSVPPAITGVVAGEKIASTPADKRPERPHRVRKPSHGSQEAPE
DDAPPTGAGGKNSKRDEEEEDQKNPAFVPRKGLFFEHDIRGGNNKEDARAKVHNRKLWRDAGRWEHDRFR
EDEQAPKSREELVALYGYDIRSGAHADDARPLRRPRYRSPTGRWSRESPPVNRGPYQAPKREPVERPARL
RDEDRDTERRPAPRRPGPTTAGPAALPGRPRAYVEQRAYVEPRARAHTQGDSSRDQPPGTPAARDGARSS
PQRHQQRHQQHNGNGEEEGEKRAEILSAGTEAEVGARGAPAAAAAAATVAPATGDAAFAVDLAQDKAPER
KSYSRVRRLRNKPSELPGQASTTDPVSPAPLAAPAPSTVPLVALPETQAATALPHQHNPQQLQLQQPQAP
PLKPLGERTRPIAALLPQPLWDVPVLEPSPVGRLEHGMAQISLGQTWNPGAQPHYLPTQREHRGGATHLP
VNSVPPPFNRIEDLGGQAGRAKRYSSQRSRPVPEPHPTPPMHIPLIEGSYYDPNTAYQAPLYGHGGDSPA
AIPPQGIMVQPDLNLPHPGLHPHQSPTHMYAPPVSMATGQPPPPGQMLGYFPSNIMYPGAALPNPGYTYT
PAPIPPPPPPPAHIFPPPQAQSQVYGGVTYYNTAQQQAQPKPSPPRRPSQPVSVKPPPEEGQAERKE

Branchiostoma belcheri:

>XP_019629453.1 PREDICTED: protein CASC3-like [Branchiostoma belcheri]
MADRRRRRPSESEGEEGEEETLSTSQASLGSTEDPSHSGEDDRDRQSPCESEGEHAEALSEYESAEEDRA
GEADEDSQAEQGDSDTEHEDTRADDDTETEDTQETSEETQETGDTRDLDTSKSTVSTDGSDKGKKLERQS
GDGSEAPDSEDQAENLDDDEDRRNPAFIPRKGDFFEHDIRGEEDDKQQGRPPVKGRRKLWQDEGKWQHDL
FNEDEQRPKPVEELIAIYGYDPRRESSPDNIRPRRGRRPPRGGGRGGRPPPRTLRDFVERPPPPRRGFPP
RNRTDVQTRNRAQYTQDVRQWAANTNNYRDNGPLDNVVSDENRNQPEVSRQKPSVEQAVPNVINNVPSNP
MPKNSENGVTVAPMPVKAPTSTESEHQHPAPQEKRSYTRYRRSTQAPRTSPPQQQNIRKRMENLSLQPGE
ESVAQDRGHADGSPQHPEERHGPNRSDRPKRYSSLRQRPMPEQVVPPNMVKGVTYYNQGYRGEQFARGST
PPVVDGPIQPHPTGPPAPPHQGMIVSDVNLPPGMAHPAMYNPAMTMAPPVPPQLLPPPFFSAGGMVNYAA
APNPVSSYAPIPQAGPTAPTPNVPPVYTSPHPPSAQVVRGVTYFNNQMPPQVLKRSPARRPNAAIPIEPP
PDVRGDPTASEEGAQPTEA

Drosophila melanogaster:

No homolog found.

Hydra vulgaris:

No homolog found.

Nematostella vectensis:

No homolog found.

Amphimedon queenslandica:

No homolog found.

Alignment

```
XP_019629453.1[B.belcheri]      MADRRRRRPSESEGEEGEE--ETLSTSQASLGST--------------------------        32
XP_032831431.1[P.marinus]       MADRRRRT-GASQDSDEEEEDEEE-ESDLSPGRG--------------------------        32
XP_014047999.1[S.salar]         MADRRRRRRRASQDSEEDDESGSGSESGKSLSPTTK-------PRVRD------------        41
AAH47246.1[X.laevis]            MADRRRRRRRASQDSEELG--EEEESASDSAGSGGE----SVGPIRQERAEQSKT--EP-        51
XP_046789397.1[G.gallus]        MADRRRQR--ASQDSEDDSDSAA-SDSGDSAASAARSRSGSASPRPSHRPPRGAAGALSA        57
NP_619601.2[M.musculus]         MADRRRQR--ASQDTEDEESGASGSDS-GSPARGGGSCSGSVG--------GGGSGSLPS        49
NP_031385.2[H.sapiens]          MADRRRQR--ASQDTEDEESGASGSDSGGSPLRGGGSCSGSAG--------GGGSGSLPS        50
XP_024090145.1[P.abelii]        MADRRRQR--ASQDTEDEESGASGSDSGGSPLRGGGSCSGSAG--------GGGSGSLPS        50
                                ******:    *:. :          :  *

XP_019629453.1[B.belcheri]      ----------EDPSHSGEDDRDRQSPCESEGEH---AEALSEYESAEEDRAGEADEDSQA        79
XP_032831431.1[P.marinus]       ------PE-----KAAAIPARAAESECESEGVPERDGVVLSGYESADDNYSHYSEDEKEE        81
XP_014047999.1[S.salar]         ------PEPVEAPAVRVVPKNDAESECESEDGV-GE-AVLSDYESADPEENGSHSEGGEE        93
AAH47246.1[X.laevis]            ------ARAEQETRRAEPAREGKESECESEDGIEGD-AVLSDYESADESEEEEAHNSEED        104
XP_046789397.1[G.gallus]        GPRGRG---ADSAAGGGAAKSAPQSECESEDGIEGD-AVLSDYESAEDSEAEEEDYSEEE        113
NP_619601.2[M.musculus]         QRGGRGGGLHLRRVESGGAKSAEESECESEDGMEGD-AVLSDYESAEDSEGEE-DYSEEE        107
NP_031385.2[H.sapiens]          QRGGRTGALHLRRVESGGAKSAEESECESEDGIEGD-AVLSDYESAEDSEGEEGEYSEEE        109
XP_024090145.1[P.abelii]        QRGGRIGALHLRRVESGGAKSAEESECESEDGIEGD-AVLSDYESAEDSEGEEGEYSEEE        109
                                                       :* ****.       .** ****: .         :

XP_019629453.1[B.belcheri]      EQGDSDTEHEDTR---ADDDTETEDTQET--SEETQETGDTRDLDT-SKSTV--------        125
XP_032831431.1[P.marinus]       EEGSCSVTD---------DEV-----AEEGS-----------SVPPAITGVVAGEKIAST        116
XP_014047999.1[S.salar]         EEEVVPPPAAEPKPSPAADTPAAEGELQEGGGKEEGEKGVSKEVKCEDKGNLAGERQ---        150
AAH47246.1[X.laevis]            PLKTTL------K-------------QENNVEEAP----ATREQKPKSKGAVTGERQ---        138
XP_046789397.1[G.gallus]        SAKVEL------K-------------QDSNGSCESAAKAEKGDEKPDSKGAVTGERQ---        151
NP_619601.2[M.musculus]         NSKVEL------K-------------SEANDAADSSA-KEKGEEKPESKGTVTGERQ---        144
NP_031385.2[H.sapiens]          NSKVEL------K-------------SEANDAVNSSTKEEKGEEKPDTKSTVTGERQ---        147
XP_024090145.1[P.abelii]        NSKVEL------K-------------SEANDAVNSSTKEEKGEEKPDTKSTVTGERQ---        147
                                                           :              .     .. :

XP_019629453.1[B.belcheri]      -STDGSDKGKKLERQSGDGSEAPDSED--------QAENLDDDEDRRNPAFIPRKGDFFE        176
XP_032831431.1[P.marinus]       PADKRPERPHRVRKPSHGSQEAPEDDAPPTGAGGKNSKRDEEEEDQKNPAFVPRKGLFFE        176
XP_014047999.1[S.salar]         ---------------SGDGQESTEDSE--NKGAKP-GQKLDDDQDRNNPAYIPRKGMFFE        192
AAH47246.1[X.laevis]            ---------------SGDGQESTEPEE--NNTSKKSQKQLDDDEDRKNPAYIPRKGLFFE        181
XP_046789397.1[G.gallus]        ---------------SGDGQESTEPVE--NKVGKKAPKHLDDDEDRKNPAYIPRKGLFFE        194
NP_619601.2[M.musculus]         ---------------SGDGQESTEPVE--NKVGKKGPKHLDDDEDRKNPAYIPRKGLFFE        187
NP_031385.2[H.sapiens]          ---------------SGDGQESTEPVE--NKVGKKGPKHLDDDEDRKNPAYIPRKGLFFE        190
XP_024090145.1[P.abelii]        ---------------SGDGQESTEPVE--NKVGKKGPKHLDDDEDRKNPAYIPRKGLFFE        190
                                               * ...*: :             :. ::::*:.***::**** ***

XP_019629453.1[B.belcheri]      HDIRGEEDDKQQGRPPVKGRRKLWQDEGKWQHDLFNEDEQRPKPVEELIAIYGYDPRRES        236
XP_032831431.1[P.marinus]       HDIRGGNNKE-DARAKVHN-RKLWRDAGRWEHDRFREDEQAPKSREELVALYGYDIRSGA        234
XP_014047999.1[S.salar]         HDVRGNAQEE--ERPKGRN-RKLWKDEGRWEHDRFREEEQAPKSREELVAIYGYDIRNGG        249
AAH47246.1[X.laevis]            HDLRGHVNDE-EVRPKGRHPRKLWKDEGRWVHDRFREDEQAPKSREELVSIYGYDIRSCR        240
XP_046789397.1[G.gallus]        HDLRGQTQEE-EVRPKGRQ-RKLWKDEGRWEHDKFREDEQAPKTRQELIALYGYDIRSAH        252
NP_619601.2[M.musculus]         HDLRGQTQEE-EVRPKGRQ-RKLWKDEGRWEHDKFREDEQAPKSRQELIALYGYDIRSAH        245
NP_031385.2[H.sapiens]          HDLRGQTQEE-EVRPKGRQ-RKLWKDEGRWEHDKFREDEQAPKSRQELIALYGYDIRSAH        248
XP_024090145.1[P.abelii]        HDLRGQTQEE-EVRPKGRQ-RKLWKDEGRWEHDKFREDEQAPKSRQELIALYGYDIRSAH        248
                                **:**  :.:   *   :  ****:* *:* ** *.*:** **  :**:::**** *

XP_019629453.1[B.belcheri]      SPDNIRPRRGRRPPRGGGR------GGRPPPRTL--------------------------        264
XP_032831431.1[P.marinus]       HADDARPLR--RPRYRSPT---GRWSRESPPVNRGPYQAPKREPVE--RPARLRDEDRDT        287
XP_014047999.1[S.salar]         VSNE-RSYRQRKPRHSTSPVRDKRWRDGDGERPIHTSWQQGGNSNSRSAPLAVALQQSGP        308
AAH47246.1[X.laevis]            NPEEIHPRRPGKPRFSSPSRREEN--DEKPSRPSNRYQ-DSGVT----QPL---------        284
XP_046789397.1[G.gallus]        NPDDIRPRRMRKPRFGS-PQRDPNWSNERPNKPPRHQG-TDGTS----APP---------        297
NP_619601.2[M.musculus]         NPDDIKPRRIRKPRFGSSPQRDPNWIGDRSSKSHRHQG-PGG-N----LPP---------        290
NP_031385.2[H.sapiens]          NPDDIKPRRIRKPRYGSPPQRDPNWNGERLNKSHRHQG-LGG-T----LPP---------        293
XP_024090145.1[P.abelii]        NPDDIKPRRIRKPRYGSPPQRDPNWNGERLNKSHRHQG-LGG-T----LPP---------        293
                                  :: :  *  :*

XP_019629453.1[B.belcheri]      ------RDFVERPPPP-RRGFPPRNRTDVQTRNRAQYTQDVRQ---------WAANTNNY        308
XP_032831431.1[P.marinus]       ERRPAPRRPGPTTAGPAALPGRPRAYVE-----QRAYVEPRARAHTQ-GDS--------S        333
XP_014047999.1[S.salar]         PPSAAPNTQRNNNPPRPSSHPPPRGFQGNR-PPQAQYRNDRNQESQRPGPKAHPAEPLQT        367
AAH47246.1[X.laevis]            ------RPYTNRNAPPSSKVGPSRTYSR-----QGGYKEN------RASYQSEE------        321
XP_046789397.1[G.gallus]        ------RTFTSRSSAGTGRMPPPRNYPR-----VGGYKES------RPGYRASE------        334
NP_619601.2[M.musculus]         ------RTFINRNTAGTGRMSASRNYSR-----SGGFKDG------RTSFRPVE------        327
NP_031385.2[H.sapiens]          ------RTFINRNAAGTGRMSAPRNYSR-----SGGFKEG------RAGFRPVE------        330
XP_024090145.1[P.abelii]        ------RTFINRNAAGTGRMSAPRNYSR-----SGGFKEG------RAGFRPVE------        330
                                      .                *            : :

XP_019629453.1[B.belcheri]      RDNGPLDN---------VVSDENRNQPEVSRQKPSVEQ---------AVPNV----I---        343
XP_032831431.1[P.marinus]       RDQPPG--------T-PAARDGARSSPQRHQQRHQQHNGNGEEEGEKRAEILS-AGTE--        381
XP_014047999.1[S.salar]         RSLPPMDGERGPRGRGNRGAHTERSSSVV------VEEIRSEEDDERNTTTVTTSVYHNR        421
AAH47246.1[X.laevis]            ----------------EAAPHTYERR---------QDY---------------GGHRSR        340
XP_046789397.1[G.gallus]        ----------------VSAQP-SRNGEQS------KQE---------------SGYRAK        355
NP_619601.2[M.musculus]         ----------------VAGQHGGRSAETL------KHE---------------ASYRSR        349
NP_031385.2[H.sapiens]          ----------------AGGQHGGRSGETV------KHE---------------ISYRSR        352
XP_024090145.1[P.abelii]        ----------------AGGQHGGRSGETV------KHE---------------ISYRSR        352
                                                       .            .

XP_019629453.1[B.belcheri]      -----NNVPSNPMPKNSE-----------NGVTVAPMPVKAPTSTESEHQHPAPQEKRSY        387
XP_032831431.1[P.marinus]       ----------------AEVGARGAPAAAAAAATVAPA--TGDAAFAVDLAQDKAPERKSY        423
XP_014047999.1[S.salar]         HYSGKGDRERDSAPRRQ--------EQQ-RGGSAPPA--DNPVTRDASPAPERPVEKKSY        470
AAH47246.1[X.laevis]            STDQGPPPPREYSPEADPI------IKE-EAFTE--K----QTAEPSPPPPDRPVEKKSY        387
XP_046789397.1[G.gallus]        RAE--QSPPRDKSPEVEAVHIHGSPVKE-EGAL---E----NQPDAAQPPPDRPIEKKSY        405
NP_619601.2[M.musculus]         RLE--QTPVRDPSPEPDAP-LLGSPEKE-EVASETPA----AVPDITPPAPDRPIEKKSY        401
NP_031385.2[H.sapiens]          RLE--QTSVRDPSPEADAP-VLGSPEKE-EAASEPPA----AAPDAAPPPPDRPIEKKSY        404
XP_024090145.1[P.abelii]        HLE--QTPVRDPSPEADAP-VLGSPEKE-EAAS--PA----AAPDAAPPPPDRPIEKKSY        402
                                                                                       *::**

XP_019629453.1[B.belcheri]      TRYRRSTQAPRTSPPQ--------------------------------------------        403
XP_032831431.1[P.marinus]       SRVRRLRNKPSELPGQASTTDPVSPAPLAAPAPSTVPLVALPETQAATALPHQHNPQQLQ        483
```

```
XP_014047999.1[S.salar]         SLARRTRTRATELDKQASLEEP-----AS-------------TAS-------------     497
AAH47246.1[X.laevis]            SRARRSRIKVGDAGKSMEDTTAAEL-PPPPPVPP---AVAAEFTPA-------------     429
XP_046789397.1[G.gallus]        SRARRTRVKAGDAGKLADDVPALEA-PAT----P---KP-IQAETS-------------     442
NP_619601.2[M.musculus]         SRARRTRTKVGDAVKAAEEVPPPSE-GLA----S---TATVPETTP-------------     439
NP_031385.2[H.sapiens]          SRARRTRTKVGDAVKLAEEVPPPPE-GLI----P---APPVPETTP-------------     442
XP_024090145.1[P.abelii]        SRARRTRTKVGDAVKLAEEVPPPPE-GLI----P---APPVPETTP-------------     440
                                :  **

XP_019629453.1[B.belcheri]      ----------------------------------------------QQNIRKRMENLSL-     416
XP_032831431.1[P.marinus]       LQQPQAPPLKPLGERTRPIAALLPQPLWDVPVL---------EPSPVGRLEHGMAQISL-     533
XP_014047999.1[S.salar]         -------------------SSALTSEPWQGPGQSQGDAGDSSQATVLTGLDQDLARLSLA     538
AAH47246.1[X.laevis]            -------------------PLNVKQGNWEPP-----------AEGGMSGIDEELSKMNLS     459
XP_046789397.1[G.gallus]        -------------------PPPAKSSNWESP-----------VESGLDGLEQEMIQMNLT     472
NP_619601.2[M.musculus]         ---------------------AAKTGNWEAP-----------VDSTTGGLEQDVAQLNIA     467
NP_031385.2[H.sapiens]          -------------------TPPTKTGTWEAP-----------VDSSTSGLEQDVAQLNIA     472
XP_024090145.1[P.abelii]        -------------------TPPTKTGNWEAP-----------VDSTTGGLEQDVAQLNIA     470
                                                                                 : . : .:.:

XP_019629453.1[B.belcheri]      ----QPGEESVAQD-RGHADGSPQHPEERH-----------GPNRSDRPKRYSSLRQRPM     460
XP_032831431.1[P.marinus]       GQTWNPGAQPHYLPTQREHRGGATHLPVNSVP-PPFNRIEDLGGQAGRAKRYSSQRSRPV     592
XP_014047999.1[S.salar]         GQNWAQNPSSFLR---AEMRGIPNSMHMGGAP-PQYSNIEELGA-GNRAKRYSSQRQRPS     593
AAH47246.1[X.laevis]            EQSWNQGQPAYISP-----RGIPNPMHMGAGP-PQYSRMEGMAVQAGRVKRYSSQRQRPV     513
XP_046789397.1[G.gallus]        EQNWSPGQSQFIQP--RELRGIPNHMHVGTGPPPQFNRMEEMAVQGGRVKRYSSQRQRPP     530
NP_619601.2[M.musculus]         EQSWSPSQPSFLQP--RELRGVPNHIHMGAGPPPQFNRMEEMGVQSGRAKRYSSQRQRPV     525
NP_031385.2[H.sapiens]          EQNWSPGQPSFLQP--RELRGMPNHIHMGAGPPPQFNRMEEMGVQGGRAKRYSSQRQRPV     530
XP_024090145.1[P.abelii]        EQSWSPGQPSFLQP--RELRGMPNHIHMGAGPPPQFNRMEEMGVQGGRAKRYSSQRQRPV     528
                                      .             *                        ..* ***** *.**

XP_019629453.1[B.belcheri]      PEQVVPPNMVKGV---TYYNQ--GYRGEQFARGSTPPVVDGPIQPHPTGPPAPPHQGMIV     515
XP_032831431.1[P.marinus]       PEPHPTPPMHIPLIEGSYYDPNTAYQAPLYGHGG-------------DSPAAIPPQGIMV     639
XP_014047999.1[S.salar]         PE--PAPPMHIGVMESHYYEPM-SYQGPIYAHG--------------DSPAPLPPQGMLV     636
AAH47246.1[X.laevis]            PDP-A--AMHISLMESHYYDSL-QFQGPIYAHG--------------DSSPSMPPQGMIV     555
XP_046789397.1[G.gallus]        VPE-PAPPMHISIMEGHYYDPL-QFQGPIYTHG--------------ENPAPLPPQGMIV     574
NP_619601.2[M.musculus]         PEP-PAPPVHISIMEGHYYDPL-QFQGPIYTHG--------------DSPAPLPPQGMIV     569
NP_031385.2[H.sapiens]          PEP-PAPPVHISIMEGHYYDPL-QFQGPIYTHG--------------DSPAPLPPQGMLV     574
XP_024090145.1[P.abelii]        PEP-PAPPVHINIMEGHYYDPL-QFQGPIYTHG--------------DSPAPLPPQGMLV     572
                                        :   :    **:    ::.  : :*               .    * **::*

XP_019629453.1[B.belcheri]      S-DVNLP----------------PGMAHPAMYN-PAMTMA--PPVPPQLLPPPFFSAGGM     555
XP_032831431.1[P.marinus]       QPDLNLPH------PGLHPHQSPTHM-----Y-APPVSMATGQPPPPGQMLG-YFP--SN     684
XP_014047999.1[S.salar]         QPEMHLPHPGHPGHPGLHPHQSSGPMPNPALYAAPPVSMSPGQPPPQQLLAPPFYPPP-G     695
AAH47246.1[X.laevis]            QPEMHLSH------PGMHPHQSPATISTPNIYP-APVSLTPGQPPPQQLLPPPYFTAPSN     608
XP_046789397.1[G.gallus]        QPEMHLPH------PGLHPHQTPAPMANPGLYP-PPVSMPPGQPPPQQLLAPTYFSPP-G     626
NP_619601.2[M.musculus]         QPEMHLPH------PGLHPHQSPGPLPNPGLYP-PPVSMSPGQPPPQQLLAPTYFSAP-G     621
NP_031385.2[H.sapiens]          QPGMNLPH------PGLHPHQTPAPLPNPGLYP-PPVSMSPGQPPPQQLLAPTYFSAP-G     626
XP_024090145.1[P.abelii]        QPEMHLPH------PGLHPHQTPAPLPNPGLYP-PPVSMSPGQPPPQQLLAPTYFSAP-G     624
                                .  ::*                   :     *    :::    * *   :   ::

XP_019629453.1[B.belcheri]      VNYA--AAPNPVSSYAPIPQAGPTAPTPNVPPVYTSPHPPSAQVVRGVTYFNNQMPP---     610
XP_032831431.1[P.marinus]       IMYPGAALPNPGYTYTPAPI----PPPPPPPAHIFPPPQAQSQVYGGVTYYNTAQQQ---     737
XP_014047999.1[S.salar]         VM----TFGNTNYPYPAGAT----LPP-MYNPPAQSQVYGQSQVYGGVTYYDPMQQQ---     743
AAH47246.1[X.laevis]            VM----NFGNPTYPYPPGAL----PP---PPAHLYPNAQAQSQVYGGVTYYNPVQQQVQQ     657
XP_046789397.1[G.gallus]        VM----NFGNPGYPYPPGAL----PPP--PPPHLYSNTQAQSQVYGGVTYYNTVQQQ---     673
NP_619601.2[M.musculus]         VM----NFGNPNYPYAPGAL----PPP--PPPHLYPNTQAPPQVYGGVTYYNPAQQQ---     668
NP_031385.2[H.sapiens]          VM----NFGNPSYPYAPGAL----PPP--PPPHLYPNTQAPSQVYGGVTYYNPAQQQ---     673
XP_024090145.1[P.abelii]        VM----NFGNPSYPYAPGAL----PPP--PPPHLYPNTQAPSQVYGGVTYYNPAQQQ---     671
                                :        *    *          *                **   ****::

XP_019629453.1[B.belcheri]      -QVLKRSPARRPNAAIPIEPPPDVRGDPTASEEGAQPTEA        649
XP_032831431.1[P.marinus]       -AQPKPSPPRRPSQPVSVKPPPEEGQAERKE---------        767
XP_014047999.1[S.salar]         -AQPKPSPPRRSSQPVTVKPPPPEVGYGPE----------        772
AAH47246.1[X.laevis]            QVLPKPSPPRRTSQPVTIKPPPPEPQSL-PVALTF-----        691
XP_046789397.1[G.gallus]        -VQPKPSPPRRTSQPVTIKPPPPEVVSRAPVNLSF-----        707
NP_619601.2[M.musculus]         -VQPKPSPPRRTPQPVSIKPPPPEVVSRGSS---------        698
NP_031385.2[H.sapiens]          -VQPKPSPPRRTPQPVTIKPPPPEVVSRGSS---------        703
XP_024090145.1[P.abelii]        -VQPKPSPPRRTPQPVTIKPPPPEVVSRGSS---------        701
                                    * ** **    : ::***
```
