# Supplementary material for: An Evolutionary Perspective on the Origin, Conservation and Binding Partner Acquisition of Tankyrases
Source: Biomolecules. 2022 Nov 15;12(11):1688. doi: 10.3390/biom12111688 (PMC9688111; doi:10.3390/biom12111688)
Supplement: Supplementary file 1 [file biomolecules-12-01688-s001.zip › biomolecules-1913719-supplementary-11.17 -done/Data_7/GMD.pdf]

GMD

Homo sapiens:

>AAC13553.1 GDP-mannose 4,6 dehydratase [Homo sapiens]
MAHAPARCPSARGSGDGEMGKPRNVALITGITGQDGSYLAEFLLEKGYEVHGIVRRSSSFNTGRIEHLYKNPQAHIEGNMKLHYGDLTDSTCLVKIINEVKPTEIYNLGAQ
SHVKISFDLAEYTADVDGVGTLRLLDAVKTCGLINSVKFYQASTSELYGKVQEIPQKETTPFYPRSPYGAAKLYAYWIVVNFREAYNLFAVNGILFNHESPRRGANFVTRK
ISRSVAKIYLGQLECFSLGNLDAKRDWGHAKDYVEAMWLMLQNDEPEDFVIATGEVHSVREFVEKSFLHIGKTIVWEGKNENEVGRCKETGKVHVTVDLKYYRPTEVDFLQ
GDCTKAKQKLNWKPRVAFDELVREMVHADVELMRTNPNA

Pongo abelii:

>XP_002816391.1 GDP-mannose 4,6 dehydratase [Pongo abelii]
MAHAPARCPSARGSGDGEMGKPRNVALITGITGQDGSYLAEFLLEKGYEVHGIVRRSSSFNTGRIEHLYKNPQAHIEGNMKLHYGDLTDSTCLVKIINEVKPTEIYNLGAQ
SHVKISFDLAEYTADVDGVGTLRLLDAVKTCGLINSVKFYQASTSELYGKVQEIPQKETTPFYPRSPYGAAKLYAYWIVVNFREAYNLFAVNGILFNHESPRRGANFVTRK
ISRSVAKIYLGQLECFSLGNLDAKRDWGHAKDYVEAMWLMLQNDEPEDFVIATGEVHSVREFVEKSFLHIGKTIVWEGKNENEVGRCKETGKVHVTVDLKYYRPTEVDFLQ
GDCTKAKQKLNWKPRVAFDELVREMVHADVELMRTNPNA

Mus musculus:

>NP_666153.1 GDP-mannose 4,6 dehydratase [Mus musculus]
MAQAPAKCPSYPGSGDGEMGKLRKVALITGITGQDGSYLAEFLLEKGYEVHGIVRRSSSFNTGRIEHLYKNPQAHIEGNMKLHYGDLTDSTCLVKIINEVKPTEIYNLGAQ
SHVKISFDLAEYTADVDGVGTLRLLDAIKTCGLINSVKFYQASTSELYGKVQEIPQKETTPFYPRSPYGAAKLYAYWIVVNFREAYNLFAVNGILFNHESPRRGANFVTRK
ISRSVAKIYLGQLECFSLGNLDAKRDWGHAKDYVEAMWLMLQNDEPEDFVIATGEVHSVREFVEKSFMHIGKTIVWEGKNENEVGRCKETGKVHVTVDLKYYRPTEVDFLQ
GDCSKAQQKLNWKPRVAFDELVREMVQADVELMRTNPNA

Gallus gallus:

>XP_015137673.1 GDP-mannose 4,6 dehydratase isoform X1 [Gallus gallus]
MGDKKVALITGITGQDGSYLAEFLLEKGYEVHGIVRRSSSFNTGRIEHLYKNSQAHIEGNMKLHYGDLTDSTCLVKIINEVKPNEIYNLGAQSHVKISFDLAEYTADVDGV
GTLRLLDAIKTCGLINSVKFYQASTSELFGKVQEIPQKETTPFYPRSPYGAAKLYAYWIVVNFREAYNLFAVNGILFNHESPRRGANFVTRKISRSVAKIHLGQMDSFSLG
NLDAKRDWGHARDYVEAMWLMLQTDEPEDFVIATGEVHSVREFVEKSFKHIGKTIVWEGKNENEVGRCKETGKIHVTVNHKYYRPTEVDFLQGDCTKARQKLNWKPRVTFD
ELVREMVDADVELMRSNPNA

Xenopus laevis:

>NP_001085613.1 GDP-mannose 4,6-dehydratase S homeolog [Xenopus laevis]
MAQNQGDCSCPPSNSTGKPRKVALITGITGQDGSYLAEFLLEKGYEVHGIVRRSSSFNTGRIEHLYKNPHAHIEGNMKLHYGDLTDSTCLVKIINEVKPTEIYNLGAQSHV
KISFDLAEYTADVDGLGTLRLLDATKTCGLINTVKFYQASTSELYGKVQEIPQKETTPFYPRSPYGAAKLYAYWIVVNFREAYNLFAVNGILFNHESPRRGANFVTRKISR
SVAKIHFGQMESFSLGNLDAKRDWGHAKDYVEAMWLMLQTDEPEDFVISTGEVHSVREFVEKSFKHIGKTIVWEGKNENEVGRCSETGNIHVKVDLKYYRPTEVDFLQGDC
SKAKNKLGWIPKVSFDELVKEMVEADIKLMETNPNA

Salmo salar:

>NP_001134845.1 GDP-mannose 4,6 dehydratase [Salmo salar]
MAQCTEPSTSTGANGELKKPRKVAVITGITGQDGSYLAEFLLEKGYEVHGILRRSSSFNTGRIEHLYQNPQTHTEGNMKLHYGDLTDSTCLVKIINQVKPTEIYNLGAQSH
VKISFELAEYTANVDGVGTLRLLDAIKTCGLTNSVKFYQASTSELYGKVQEIPQKETTPFYPRSPYGAAKLYAYWIVVNFREAYNLFAVNGILFNHESPRRGANFVTRKIS
RSVAKIHLGQLESFSLGNLDSKRDWGHAKDYVEAMWLMLQQEEPEDLVIATGEVHSVREFVEKSFKHVGKTIVWEGKDENEVGRCQETGVIHVKVDSKYYRPTEVEYLQGD
STKALTKLGWKAKITFEELVKEMVDADIHLMKNNPNA

Petromyzon marinus:

>XP_032813332.1 GDP-mannose 4,6 dehydratase [Petromyzon marinus]
MASSSSSSPRPEGPGPDERGAEGSTAEERGAEETGAEETGAEGGEERGAEGGVGGGSGEGVGGTRGGFVDLEKGAQAVVDASPDAERNGERKVALITGITGQDGSYLAEFL
LEKGYEVHGIVRRSSSFNTGRIEHLYKNPATHQEGSMKLHYGDLTDSSCLVKIISAVRPSEIYNLGAQSHVKISFDLAEYTANVDGVGTLRLLDAVRTCGLADRVRFYQAS
TSELYGKVQEIPQSESTPFYPRSPYGAAKLYAYWIVVNFREAYGMYACNGILFNHESPRRGSNFVTRKISRSVAKIHLGQLDCFSLGNLDARRDWGHARDYIEAMWLMLQQ
PEPDDFVIATGEVHSVREFVERAFRCVGRSIVWEGSGLQEVGRCQETGEVHVRVDAKYYRPTEVDFLQGDCSKARAKLGWATRVTFEELVQEMVSADMELMKKNPNA

Branchiostoma belcheri:

>XP_019629718.1 PREDICTED: GDP-mannose 4,6 dehydratase-like isoform X1 [Branchiostoma belcheri]
MAEKRPAEFEEDTKEGNGPRRTRKVALITGISGQDGSYLAEFLIDKGYVVHGIIRRASTFNTGRIEHLYKNPFGHFEGDNFHLHYGDMTDSTCLVKIITEVQPDEIYNLAA
QSHVKVSFDLAEYTADVDGVGVLRMLDAIRTTGLVSKVKFYQASTSEMYGLVQEVPQKETTPFYPRSPYGAAKLYAYWVVVNYREAYNMFACNGILFNHESPRRGETFVTR
KITRGVAKILLGQQDDIVLGNLDSQRDWGHAKDYIRAMWMMLQHQVPEDFVISTGTVHSVREFVVASFKHIGVDIIWEGKGLKEVGKDKATGTVRVRVSQKYYRPTEVDFL
QGDSTKAKKILGWEPSVSFDEMVKEMVDSDIQLMKSHPLA

Drosophila melanogaster:

>NP_608888.2 GDP-mannose 4,6-dehydratase [Drosophila melanogaster]
MLNTRLIAMSTSDGAPETKKQRPESSSNGSKDQNGTEAGAEGDSRDKVALITGITGQDGSYLAEFLLKKDYEVHGIIRRASTFNTTRIEHLYADPKAHKGGRMKLHYGDMT
DSSSLVKIINMVKPTEIYNLAAQSHVKVSFDLSEYTAEVDAVGTLRILDAIRTCGMEKNVRFYQASTSELYGKVVETPQNEQTPFYPRSPYACAKMYGFWIVINYREAYNM
YACNGILFNHESPRRGENFVTRKITRSVAKIYHKQMEYFELGNLDSKRDWGHASDYVEAMWMMLQRESPSDYVIATGETHSVREFVEAAFKHIDREITWKGKGVDEVGVEN
GTGIVRVRINPKYFRPTEVDLLQGDASKANRELNWTPKVTFVELVSDMMKADIELMRKNPIA

Hydra vulgaris:

>XP_002157707.2 PREDICTED: GDP-mannose 4,6 dehydratase-like [Hydra vulgaris]
MSSSTNTRKVALITGITGQDGSYLCEYLLEKGYEVHGIIRRSSSFNTGRIQHLYKDKAGHIGGNMHLHYGDLTDSSCLLNIVSQTRPTEIYNLAAQSHVKVSFDLAEYTAD
ADAMGTLRLLEAVKICNLIGEVRFYQASTSELYGKVHEIPQKETTPFHPRSPYGVAKLYGYWIVTNYREAYGLHASNGILFNHESPRRGETFVTRKITRAVAKIHLGLQDE
LVLGNIDSVRDWGHAKDYVEAMWLMLQQESADDYVIATNETHTVREFVEKAFSLVNVKIRWEGKGANEVGFDDISNKIRVRISPKYYRPAEVEYLQGDPSKAEKVLGWKRK
VSFDELVKEMVEADIQLMKTNPEA

Nematostella vectensis:

>XP_001622549.1 GDP-mannose 4,6 dehydratase [Nematostella vectensis]
MAEASEDQRKVALITGITGQDGSYLAEFLLNKGYQVHGIARRSSSFNTGRIEHLYENCYTHMGGAMKTHYGDLTDSTNLVKIISEVQPTEIYNLGAMSHVKVSFELAEYTA
DVDAVGVLRLLDAIKTCGLANKVKFYQASTSELYGLVQEVPQTEKTPFYPRSPYGAAKLYGYWIVVNYREAYNMFACNGILFNHESPRRGETFVTRKITRAVAKIHLGLQK
ELRLGNLDSKRDWGHAKDYVEAMWMMLQQDKPEDFVIATGETHSVREFVEKSFAHVGTEIVWQGKDEKEIGVDKATNKVLVRVDPKYYRPAEVELLLGCAKKAEEKLGWKP
KIKFEALVKEMVESDLQLMKNNPTA

Amphimedon queenslandica:

>XP_003384422.1 PREDICTED: GDP-mannose 4,6 dehydratase 2-like [Amphimedon queenslandica]
MASGNGALPERINRSSSDETEKVAMITGITGQDGSYLAELLLEKGYKVHGIIRRSSSFNTGRIKHLYSNPALHEGGGLLLHYGDLTDSSCLVKLVSEIRPTEIYNLAAQSH
VKLSFDMSEYTADVNGVGTLRLLDAIRTCGLEKKVKFYQASTSELYGKVQEIPQTEKTPFYPRSPYAAAKLYGYWVVVNYREAYDMFACNGILFNHESPRRGENFVTRKVT
RSVAQVHLGHRESFSLGNLGSQRDWGHAKDYVKAMWMMLQQDKPDDFVIATNKTHSVKKLVELAFKEVGLTISWKGEGLDEVGVDQNDIVRVTVNPKYYRPTEVDFLQGNP
AKAKAILGWEPKIEFEDLVKEMVASDIKLLSADPTA

Alignment:

```
XP_003384422.1[A.queenslandica]     ------------------------------------------------------------      0
NP_608888.2[D.melanogaster]         MLNTRLIAMSTSDGAPETKKQRP---------------------------------ESS-      26
XP_002157707.2[H.vulgaris]          ------------------------------------------------------------      0
XP_019629718.1[B.belcheri]          ------------------------------------------------------------      0
XP_001622549.1[N.vectensis]         ------------------------------------------------------------      0
XP_032813332.1[P.marinus]           ------MA-SSSSSSPRPEGPGPDERGAEGSTAEERGAEETGAEETGAEGGEERGAEGGV      53
NP_001134845.1[S.salar]             ------------------------------------------------------------      0
NP_001085613.1[X.laevis]            ------------------------------------------------------------      0
XP_015137673.1[G.gallus]            ------------------------------------------------------------      0
AAC13553.1[H.sapiens]               ------------------------------------------------------------      0
XP_002816391.1[P.abelii]            ------------------------------------------------------------      0
NP_666153.1[M.musculus]             ------------------------------------------------------------      0


XP_003384422.1[A.queenslandica]     -----------------MASGNGALPERINRSSSDETEKVAMITGITGQDGSYLAELLLE      43
NP_608888.2[D.melanogaster]         ------------------SNGSKDQNGTEAGAEGDSRDKVALITGITGQDGSYLAEFLLK      68
XP_002157707.2[H.vulgaris]          ------------------------------MSSSTNTRKVALITGITGQDGSYLCEYLLE      30
XP_019629718.1[B.belcheri]          -------MAE--------KRPAEFEEDTKEGNGPRRTRKVALITGISGQDGSYLAEFLID      45
XP_001622549.1[N.vectensis]         -----------------------------MAEASEDQRKVALITGITGQDGSYLAEFLLN      31
XP_032813332.1[P.marinus]           GGGSGEGVGGTRGGFVDLEKGAQAVVDASPDAERNGERKVALITGITGQDGSYLAEFLLE      113
NP_001134845.1[S.salar]             -----------------MAQCTEPSTSTGANGELKKPRKVAVITGITGQDGSYLAEFLLE      43
NP_001085613.1[X.laevis]            -------MA------QNQGDCS--CPP---SNSTGKPRKVALITGITGQDGSYLAEFLLE      42
XP_015137673.1[G.gallus]            ----------------------------------MGDKKVALITGITGQDGSYLAEFLLE      26
AAC13553.1[H.sapiens]               -------MA------HAPARCP--SARGSGDGEMGKPRNVALITGITGQDGSYLAEFLLE      45
XP_002816391.1[P.abelii]            -------MA------HAPARCP--SARGSGDGEMGKPRNVALITGITGQDGSYLAEFLLE      45
NP_666153.1[M.musculus]             -------MA------QAPAKCP--SYPGSGDGEMGKLRKVALITGITGQDGSYLAEFLLE      45
                                                                          :**:****:*******.* *:.

XP_003384422.1[A.queenslandica]     KGYKVHGIIRRSSSFNTGRIKHLYSNPALHEGG-GLLLHYGDLTDSSCLVKLVSEIRPTE      102
NP_608888.2[D.melanogaster]         KDYEVHGIIRRASTFNTTRIEHLYADPKAHKGG-RMKLHYGDMTDSSSLVKIINMVKPTE      127
XP_002157707.2[H.vulgaris]          KGYEVHGIIRRSSSFNTGRIQHLYKDKAGHIGG-NMHLHYGDLTDSSCLLNIVSQTRPTE      89
XP_019629718.1[B.belcheri]          KGYVVHGIIRRASTFNTGRIEHLYKNPFGHFEGDNFHLHYGDMTDSTCLVKIITEVQPDE      105
XP_001622549.1[N.vectensis]         KGYQVHGIARRSSSFNTGRIEHLYENCYTHMGG-AMKTHYGDLTDSTNLVKIISEVQPTE      90
XP_032813332.1[P.marinus]           KGYEVHGIVRRSSSFNTGRIEHLYKNPATHQEG-SMKLHYGDLTDSSCLVKIISAVRPSE      172
NP_001134845.1[S.salar]             KGYEVHGILRRSSSFNTGRIEHLYQNPQTHTEG-NMKLHYGDLTDSTCLVKIINQVKPTE      102
NP_001085613.1[X.laevis]            KGYEVHGIVRRSSSFNTGRIEHLYKNPHAHIEG-NMKLHYGDLTDSTCLVKIINEVKPTE      101
XP_015137673.1[G.gallus]            KGYEVHGIVRRSSSFNTGRIEHLYKNSQAHIEG-NMKLHYGDLTDSTCLVKIINEVKPNE      85
AAC13553.1[H.sapiens]               KGYEVHGIVRRSSSFNTGRIEHLYKNPQAHIEG-NMKLHYGDLTDSTCLVKIINEVKPTE      104
XP_002816391.1[P.abelii]            KGYEVHGIVRRSSSFNTGRIEHLYKNPQAHIEG-NMKLHYGDLTDSTCLVKIINEVKPTE      104
NP_666153.1[M.musculus]             KGYEVHGIVRRSSSFNTGRIEHLYKNPQAHIEG-NMKLHYGDLTDSTCLVKIINEVKPTE      104
                                    *.* **** **:*:*** **:*** :   *  *  :  ****:***: *::::.  :* *

XP_003384422.1[A.queenslandica]     IYNLAAQSHVKLSFDMSEYTADVNGVGTLRLLDAIRTCGLEKKVKFYQASTSELYGKVQE      162
NP_608888.2[D.melanogaster]         IYNLAAQSHVKVSFDLSEYTAEVDAVGTLRILDAIRTCGMEKNVRFYQASTSELYGKVVE      187
XP_002157707.2[H.vulgaris]          IYNLAAQSHVKVSFDLAEYTADADAMGTLRLLEAVKICNLIGEVRFYQASTSELYGKVHE      149
XP_019629718.1[B.belcheri]          IYNLAAQSHVKVSFDLAEYTADVDGVGVLRMLDAIRTTGLVSKVKFYQASTSEMYGLVQE      165
XP_001622549.1[N.vectensis]         IYNLGAMSHVKVSFELAEYTADVDAVGVLRLLDAIKTCGLANKVKFYQASTSELYGLVQE      150
XP_032813332.1[P.marinus]           IYNLGAQSHVKISFDLAEYTANVDGVGTLRLLDAVRTCGLADRVRFYQASTSELYGKVQE      232
NP_001134845.1[S.salar]             IYNLGAQSHVKISFELAEYTANVDGVGTLRLLDAIKTCGLTNSVKFYQASTSELYGKVQE      162
NP_001085613.1[X.laevis]            IYNLGAQSHVKISFDLAEYTADVDGLGTLRLLDATKTCGLINTVKFYQASTSELYGKVQE      161
XP_015137673.1[G.gallus]            IYNLGAQSHVKISFDLAEYTADVDGVGTLRLLDAIKTCGLINSVKFYQASTSELFGKVQE      145
AAC13553.1[H.sapiens]               IYNLGAQSHVKISFDLAEYTADVDGVGTLRLLDAVKTCGLINSVKFYQASTSELYGKVQE      164
XP_002816391.1[P.abelii]            IYNLGAQSHVKISFDLAEYTADVDGVGTLRLLDAVKTCGLINSVKFYQASTSELYGKVQE      164
NP_666153.1[M.musculus]             IYNLGAQSHVKISFDLAEYTADVDGVGTLRLLDAIKTCGLINSVKFYQASTSELYGKVQE      164
                                    ****.* ****:**:::****:.:.:*.**:*:* :  .:   *:********::* * *

XP_003384422.1[A.queenslandica]     IPQTEKTPFYPRSPYAAAKLYGYWVVVNYREAYDMFACNGILFNHESPRRGENFVTRKVT      222
NP_608888.2[D.melanogaster]         TPQNEQTPFYPRSPYACAKMYGFWIVINYREAYNMYACNGILFNHESPRRGENFVTRKIT      247
XP_002157707.2[H.vulgaris]          IPQKETTPFHPRSPYGVAKLYGYWIVTNYREAYGLHASNGILFNHESPRRGETFVTRKIT      209
XP_019629718.1[B.belcheri]          VPQKETTPFYPRSPYGAAKLYAYWVVVNYREAYNMFACNGILFNHESPRRGETFVTRKIT      225
XP_001622549.1[N.vectensis]         VPQTEKTPFYPRSPYGAAKLYGYWIVVNYREAYNMFACNGILFNHESPRRGETFVTRKIT      210
XP_032813332.1[P.marinus]           IPQSESTPFYPRSPYGAAKLYAYWIVVNFREAYGMYACNGILFNHESPRRGSNFVTRKIS      292
NP_001134845.1[S.salar]             IPQKETTPFYPRSPYGAAKLYAYWIVVNFREAYNLFAVNGILFNHESPRRGANFVTRKIS      222
NP_001085613.1[X.laevis]            IPQKETTPFYPRSPYGAAKLYAYWIVVNFREAYNLFAVNGILFNHESPRRGANFVTRKIS      221
XP_015137673.1[G.gallus]            IPQKETTPFYPRSPYGAAKLYAYWIVVNFREAYNLFAVNGILFNHESPRRGANFVTRKIS      205
AAC13553.1[H.sapiens]               IPQKETTPFYPRSPYGAAKLYAYWIVVNFREAYNLFAVNGILFNHESPRRGANFVTRKIS      224
XP_002816391.1[P.abelii]            IPQKETTPFYPRSPYGAAKLYAYWIVVNFREAYNLFAVNGILFNHESPRRGANFVTRKIS      224
NP_666153.1[M.musculus]             IPQKETTPFYPRSPYGAAKLYAYWIVVNFREAYNLFAVNGILFNHESPRRGANFVTRKIS      224
                                     **.* ***:*****. **:*.:*:* *:****.:.* ************* .*****::

XP_003384422.1[A.queenslandica]     RSVAQVHLGHRESFSLGNLGSQRDWGHAKDYVKAMWMMLQQDKPDDFVIATNKTHSVKKL      282
NP_608888.2[D.melanogaster]         RSVAKIYHKQMEYFELGNLDSKRDWGHASDYVEAMWMMLQRESPSDYVIATGETHSVREF      307
XP_002157707.2[H.vulgaris]          RAVAKIHLGLQDELVLGNIDSVRDWGHAKDYVEAMWLMLQQESADDYVIATNETHTVREF      269
XP_019629718.1[B.belcheri]          RGVAKILLGQQDDIVLGNLDSQRDWGHAKDYIRAMWMMLQHQVPEDFVISTGTVHSVREF      285
XP_001622549.1[N.vectensis]         RAVAKIHLGLQKELRLGNLDSKRDWGHAKDYVEAMWMMLQQDKPEDFVIATGETHSVREF      270
XP_032813332.1[P.marinus]           RSVAKIHLGQLDCFSLGNLDARRDWGHARDYIEAMWLMLQQPEPDDFVIATGEVHSVREF      352
NP_001134845.1[S.salar]             RSVAKIHLGQLESFSLGNLDSKRDWGHAKDYVEAMWLMLQQEEPEDLVIATGEVHSVREF      282
NP_001085613.1[X.laevis]            RSVAKIHFGQMESFSLGNLDAKRDWGHAKDYVEAMWLMLQTDEPEDFVISTGEVHSVREF      281
XP_015137673.1[G.gallus]            RSVAKIHLGQMDSFSLGNLDAKRDWGHARDYVEAMWLMLQTDEPEDFVIATGEVHSVREF      265
```

```
AAC13553.1[H.sapiens]             RSVAKIYLGQLECFSLGNLDAKRDWGHAKDYVEAMWLMLQNDEPEDFVIATGEVHSVREF      284
XP_002816391.1[P.abelii]          RSVAKIYLGQLECFSLGNLDAKRDWGHAKDYVEAMWLMLQNDEPEDFVIATGEVHSVREF      284
NP_666153.1[M.musculus]           RSVAKIYLGQLECFSLGNLDAKRDWGHAKDYVEAMWLMLQNDEPEDFVIATGEVHSVREF      284
                                  *.**::     . : ***:.: ****** **:.***:***    .* **:*. .*:*:::

XP_003384422.1[A.queenslandica]   VELAFKEVGLTISWKGEGLDEVGVDQ-NDIVRVTVNPKYYRPTEVDFLQGNPAKAKAILG      341
NP_608888.2[D.melanogaster]       VEAAFKHIDREITWKGKGVDEVGVENGTGIVRVRINPKYFRPTEVDLLQGDASKANRELN      367
XP_002157707.2[H.vulgaris]        VEKAFSLVNVKIRWEGKGANEVGFDDISNKIRVRISPKYYRPAEVEYLQGDPSKAEKVLG      329
XP_019629718.1[B.belcheri]        VVASFKHIGVDIIWEGKGLKEVGKDKATGTVRVRVSQKYYRPTEVDFLQGDSTKAKKILG      345
XP_001622549.1[N.vectensis]       VEKSFAHVGTEIVWQGKDEKEIGVDKATNKVLVRVDPKYYRPAEVELLLGCAKKAEEKLG      330
XP_032813332.1[P.marinus]         VERAFRCVGRSIVWEGSGLQEVGRCQETGEVHVRVDAKYYRPTEVDFLQGDCSKARAKLG      412
NP_001134845.1[S.salar]           VEKSFKHVGKTIVWEGKDENEVGRCQETGVIHVKVDSKYYRPTEVEYLQGDSTKALTKLG      342
NP_001085613.1[X.laevis]          VEKSFKHIGKTIVWEGKNENEVGRCSETGNIHVKVDLKYYRPTEVDFLQGDCSKAKNKLG      341
XP_015137673.1[G.gallus]          VEKSFKHIGKTIVWEGKNENEVGRCKETGKIHVTVNHKYYRPTEVDFLQGDCTKARQKLN      325
AAC13553.1[H.sapiens]             VEKSFLHIGKTIVWEGKNENEVGRCKETGKVHVTVDLKYYRPTEVDFLQGDCTKAKQKLN      344
XP_002816391.1[P.abelii]          VEKSFLHIGKTIVWEGKNENEVGRCKETGKVHVTVDLKYYRPTEVDFLQGDCTKAKQKLN      344
NP_666153.1[M.musculus]           VEKSFMHIGKTIVWEGKNENEVGRCKETGKVHVTVDLKYYRPTEVDFLQGDCSKAQQKLN      344
                                  *  :*  :.  * *:*.. .*:*  . .. : * :. **:**:**: * *   **   *.

XP_003384422.1[A.queenslandica]   WEPKIEFEDLVKEMVASDIKLLSADPTA    369
NP_608888.2[D.melanogaster]       WTPKVTFVELVSDMMKADIELMRKNPIA    395
XP_002157707.2[H.vulgaris]        WKRKVSFDELVKEMVEADIQLMKTNPEA    357
XP_019629718.1[B.belcheri]        WEPSVSFDEMVKEMVDSDIQLMKSHPLA    373
XP_001622549.1[N.vectensis]       WKPKIKFEALVKEMVESDLQLMKNNPTA    358
XP_032813332.1[P.marinus]         WATRVTFEELVQEMVSADMELMKKNPNA    440
NP_001134845.1[S.salar]           WKAKITFEELVKEMVDADIHLMKNNPNA    370
NP_001085613.1[X.laevis]          WIPKVSFDELVKEMVEADIKLMETNPNA    369
XP_015137673.1[G.gallus]          WKPRVTFDELVREMVDADVELMRSNPNA    353
AAC13553.1[H.sapiens]             WKPRVAFDELVREMVHADVELMRTNPNA    372
XP_002816391.1[P.abelii]          WKPRVAFDELVREMVHADVELMRTNPNA    372
NP_666153.1[M.musculus]           WKPRVAFDELVREMVQADVELMRTNPNA    372
                                  *   : *  :* :*: :*:.*:  .* *
```
