# Supplementary material for: An Evolutionary Perspective on the Origin, Conservation and Binding Partner Acquisition of Tankyrases
Source: Biomolecules. 2022 Nov 15;12(11):1688. doi: 10.3390/biom12111688 (PMC9688111; doi:10.3390/biom12111688)
Supplement: Supplementary file 1 [file biomolecules-12-01688-s001.zip › biomolecules-1913719-supplementary-11.17 -done/Data_7/Golgin-45.pdf]

Golgin-45

Homo sapiens:

```
>sp|Q9H2G9|GO45_HUMAN Golgin-45 OS=Homo sapiens OX=9606 GN=BLZF1 PE=1 SV=2
MTTKNLETKVTVTSSPIRGAGDGMETEEPPKSVEVTSGVQSRKHHSLQSPWKKAVPSESP
GVLQLGKMLTEKAMEVKAVRILVPKAAITHDIPNKNTKVKSLGHHKGEFLGQSEGVIEPN
KELSEVKNVLEKLKNSERRLLQDKEGLSNQLRVQTEVNRELKKLLVASVGDDLQYHFERL
AREKNQLILENEALGRNTAQLSEQLERMSIQCDVWRSKFLASRVMADELTNSRAALQRQN
RDAHGAIQDLLSEREQFRQEMIATQKLLEELLVSLQWGREQTYSPSVQPHSTAELALTNH
KLAKAVNSHLLGNVGINNQKKIPSTVEFCSTPAEKMAETVLRILDPVTCKESSPDNPFFE
SSPTTLLATKKNIGRFHPYTRYENITFNCCNHCRGELIAL
```

Pongo abelii:

```
>NP_001126836.1 golgin-45 [Pongo abelii]
MTTKNLETKVTVTSSPIRGAGDGMETEEPPKSVEITSGVQSRKHHSLQSPWKKAVPSESPGVLQLGKMLT
EKAMEVKAVRILVPKAAITHDIPNKNTKVKSLGHHKGEFLGQSEGVIEPNKELSEVKNVLEKLKNSERRL
LQDKEGLSNQLRVQTEVNRELKKLLVASVGDDLQYHFERLAREKNQLILENEALGRNTAQLSEQLERMSI
QCDVWRSKFLASRVMADELTNSRAVLQRQNRDAHGAIQDLLSEREQFRQEMIATQKLLEELLVSLQWGRE
QTYSPSVQPHSTAELALTNHKLAKAVNSHLLGNVGINNQKKIPSTVEFCSTPAEKMAETVLRILDPVTCT
ESSPDDPFFESSPTTLLATKKNIGRFHPYTRYENITFNCCNHCQGELIAL
```

Mus musculus:

```
>NP_001153680.1 golgin-45 isoform b [Mus musculus]
MEKMTTLKSSENKGILTSTPIRGAGDGMETEEPPKSVEVTHGVQPINQHVLPSPRKKVSSDSPGVLQLGK
ILNERTVEVEAVRIFVPKAAITHDIPTKNTKVKSLGHHREELHNQAEVVTDPRKELSEVKKVLEKLKNSE
RRLLQDKEGLSNQLRVQTEINRELKKLLVASVGDDPQYHFERLAREKNQLILENEALGRNTAQLSEQLER
MSIQCDVWRSKFLASRVMADELTNFRVVLQRQNRDAQSAIQDLLSEREQFRQEMTSTQKFLEELLVSLQW
GREQTYSPNTQPHSTADLALTNHGLAQAIHAHLLGNVGISHQKKIPTTVEFCSTPAEKMAEKVLRILDPV
ACTESSPDNQFAESSPTTLLTTKKNIGRFHPYTRYENITFNCCNHCQGELIAL
```

Gallus gallus:

```
>XP_004938283.1 golgin-45 [Gallus gallus]
MTSLEKVDYASSPIRGPGDGMETEQMGESLEVSAGASATNHQVRHSPQKKAVSSLSPGVLQLGQIHADKS
VEVEAVRILVPKAAITHVVATKNAKVAKSAGHKGDAFHQSDGTADPKKEQTELKNAIEKLKNSEKRLLQD
KEGLSNQLRIQTEVNRELKKLLVASVGDDLQYHFERMAREKNQLILENEVLGRNLSQLSEQLERMSIQCD
VWRSKFLASRVMADELTNTRAILQRQTRDAQTAIQDLLNERDQFRQEMIHTQKLLEELMVSLQWGRQQTY
YPSAQPYTTTELASVNCKLAKAVSSHLLGNVGTSSPKKTSTAVEFCNTPAEKMAERVLRVLDPAARTETS
TEASFSETSPSSFLSTKKNIGRFHPYTRYEDITFNCCDHCQGELIAL
```

Xenopus laevis:

```
>NP_001088348.2 Golgin-45 [Xenopus laevis]
MAATMETLKTLATSTPLRGTGDGMETEAPLKSEVNSPHTKATQSPRKKSTVSPSLSPGVLQLGKMQSDKA
VEIEAIRILIPKAAITHTATTKNSKHLESNGHSSTELAESVTDPWKQLSELKSTLEKLKNSERRLLQDKE
GLANQLKVQTEVNRELKKLLVASVGDDLQYHFERMAREKNQLILENEVLSQNTAQLSEQLERMSIQCDVW
RSKFLASRVMADELTNIRTNLQRQNRDAQSALQDLLSEREQFRHEMSETQKTLEALLVSLQWGRQKTYAP
IPQPHTTTELIAANLKLAKSIQSHLIGNTGSDHQKPAAKPIEYCSTPAENMAETVLKALDPVSCTDNSSD
ISLSDSSPFLLAKKNIGRFHPYTRYENITFNCCNQCRGELIAV
```

Salmo salar:

```
>XP_014008079.1 PREDICTED: golgin-45-like [Salmo salar]
MSTAAVADRGSVHPVRGPGDGMETDEPPVAKEIGVVDTPLFSVPLLKVASPKQSPKTTHPSPASLQHSPH
STPQQSPRASPRDIASAPQLPGVLHLGKVPREACVVVEAVRIVVPRAAISRSGGHMGPAEEKGEAWAGQQ
MEERPPSPSLPLEDLRGAMEKLQNSERRLLQDKEGLLNQLHVQTEVNRELKKLLVASVGDDLQYHFERLA
REKNQMILDNEALGRSLASTAEQLERMSIQCDVWRTKFLASRVMAEELTNARVSLQHQTREAQSAITDLL
SERDEFSRDMMLTHRSLEQLLVSLQWGRQQTYYPSAQPLSTGELSLANHKLAEAINSRLLGKVGPVGSSS
GAGLRKGRQASELPNTSHTPAEKMAEKVLKILDPISCSDNEEKISPLSISDSSPSAFLTNKKSIGRFHPY
TRYENITFNCCERCSGDILVL
```

Petromyzon marinus:

```
>XP_032834970.1 golgin-45 [Petromyzon marinus]
MATTLTRDGTGWPQAVSRGPADGMEVTADGAGGGDPAKRPAGSSSSPPLPVRSVQPAPRSSWQHRPPAKP
APQHHHLPHQPRPLSPAAAAAATPLPHRPQQQQQQPVPVLAGGQVELTVVQIQVPKEAILHHSARSLPA
LELGVKPDSLEAGGSAAAAAAAAAVTAALRQEVKELKVEVKRLEESERRLMDDKESVVSQLRSQTEVNRE
LKKLLVASVGDDLQFHFERAVRERGRLQAEAQELSRSLASASEQLERIGIQCDVWRSKFLASRVMSEEVA
GARVHALRLFREAQSAVHDLFSERTQLCVDLQDTHRLLEELVLALQWGRQPTYSLGTRRAHNSLELAEAC
TTLASLLTQRLLGGGSGSVKSSPPPAAAGGGGAAGGAGGGGGAAGGGGAAGGGGAGGGGAAVAAQPPAQL
CSTAAERMAQQVLRMTEVRMTDPMLVDDAAGGAAGRLLTSKPSPGRFHPYTRYEHITFTCCSRCTGDIVV
V
```

Branchiostoma belcheri:

```
>XP_019628762.1 PREDICTED: golgin-45-like [Branchiostoma belcheri]
MESAPFAFVKPQAHSSQSKKTTRTHGGGGKDSTLNAMRRSQLSRTRSETRIFGQVQGFQKPKLNDVPLSS
AKITQFKAMEERGSSDLQYQFERMAREKAQLSVDHDNLVQQYADLSEEVERLSIQCDVWRSKFLGSRTQV
SQLIDLRNAIYQQFGKAQAAIDNVMKERAPIRKEMVETYRLLDQLVKALRWGKPQPLQQAANPRNMKELV
QANHRLVSAVSAQLLGNIKPMNSANQETVESGIGLTNAEAAATQVLNMNFDLQLLSPESAAPVGKMSGRF
HPQHKYEEITVNCCNKCKGEIKVV
```

Drosophila melanogaster:

```
>NP_649889.1 uncharacterized protein Dmel_CG9356, isoform A [Drosophila melanogaster]
MEANESAMPGTDKSQACRTSGDGMEQPENSGAAKTESEIPASADVDNKVKRPQLSRKDSSVVQEAVMGRK
TSTSSGHSSNLPLPGLHTLYRRPENVTRSLAPVVPKGELVQMRPRLVTSADSLPEHKKTKAPKFVPYEPY
PGAVNPMISEPTNKHKIHRDKNNLDIAVLVDQVSTLRTQELETETTDIKETDTASSHEVNSLKEELAKMR
DERNYFQAQYKFQTQVNSELKSLLVASVGEDLQTRVNLLTEDKLQLARALLDTANNLTTHTEQIEFLAGQ
CEVWRSKFLASSVMVEELARWKADLTQKNQLLNESTKQLLHATHQIREIQLDMLKQLKFLAKIRFLNLPA
TDVISLSAENLNILQRMVLHTGVGIPEETLKLSSASTSPLCEAEKYAVRALEFISQPLMATDEAIRALFD
QAQRPHYVQSPASEVTPACDNQQLDKMQ
```

Hydra vulgaris:

>XP_002164131.1 PREDICTED: golgin-45-like [Hydra vulgaris]
MEPSILKISRFALNLNQDEITKERFPGDGMESVASKNVLPKDEFSNIEKIKLFKSPPNFQLNQQEQAIRP
HNPDEDLIKYLSSTRPHIPDQELLESLQSKVKFLQEYNEKLIDEKLKVSHQLATQTQINNELKNLLVASI
GDDLETRYEKMILNRIQSDLELKRLINLVEEYQEEKQQAYIQVDVWRSKFLASRVMNEELTQWKSALYYK
YRDSHTALQSLLEEHSKIRVLNDATRIGLIKIAELLNSKDASLDSEMRLTLVDTAFINHDLVESLIEIIE
ESSWAEKPNEIKQNIFFKQFKNHLKEIYVLPSELSTAEQLAHEIVTNTGIQTEELVRMRRDYLAHNRISH
FLSTNYHVTYNCCNSCKGRIIHL

Nematostella vectensis:

>XP_001635606.1 golgin-45 [Nematostella vectensis]
MNSLEPHALKMERIPKPGSDIVDYSKVPRSSGDGMEHSGAALEPTDTRKPHALKQKPQIHFSFGHVELML
ESDTEEKEEGTKNTENDTIRKHPKKATKSTPVLNQIIERTSVDSSKTQGNDTHIFIKEEPKQEILQSSAS
TLCDKPTGSSTEELKKLQSKVEELEEQTSRLTDEKTKLTIQLGIQTEVNSDLKKLLVASMEEELENRMEA
LAHRNAQLRVEVEHWQTLCDEYAEESDRLAIDCDVWRTKFMASRMMSDQLTCWKTTLYTRYRQALAALQR
MLDERDELKHYLVHTTRLLESLDYVVKCSGGSFSDTLMDRHPIQGQSILEMATITSCLAQDVTAQVQGFI
PSGSLPTHALLLNEEKEPALDSTPAEKMALQLLTSSIELQSDDVPSVDSSVLRRMGKQYIGNSYHSRLMT
NSFRVTYDCCDRCTGPVHVV

Amphimedon queenslandica:

>XP_019850840.1 PREDICTED: golgin-45-like [Amphimedon queenslandica]
MESCRRRKQALKEKYLKKAQDLISKKKYEKYKVTDVSWSDPLITSTPLITRTRSTLPSSLRLSPHRDITE
LERMIEDQNKAMKELKRLLLASVGGEMGTEIETLIRDKVTLESKLNHSITELMEMNEEIEGLTIECDVWK
CKVLASRLLIDELREWKGNHYHLSVNTLQTLLDERDQLMNHIKAIHQLVNNISRKLMSTSSTANSTASGI
KIATVTPVQLQPIHQGCNVLELAKNTSVAVQMISDKISARHEATPMPSLLSLNTSLTEGEKLAHKLLYPV
PKETTPTSRGLPHLSFSEKTLMEQRKHQQARTLPWTDDRHDGNNDIANNNSYYFCNKCNGRAIMII

Alignment

```
XP_019850840.1[A.queenslandica]      ------------------------------------------------------------     0
NP_649889.1[D.melanogaster]          -----------MEANESAMPGTDKSQACRTSGDGMEQPENSGAAKTESEIPASADVNKV     49
XP_002164131.1[H.vulgaris]           ---MEPSILKISRFALNLN--QDEITKERFPGDGMESVASKNVLPK--------------     41
XP_001635606.1[N.vectensis]          MNSLEPHALKMERIPKPGSDIVDYSKVPRSSGDGMEHSGAALEPTDTRK-PHA-------     52
XP_019628762.1[B.belcheri]           ------------------------------------------------------------     0
XP_032834970.1[P.marinus]            -----------MATTLTRDGTWPQAVSRGPADGMEVTADGAGGGDPAKRPAGSSSS---     46
XP_014008079.1[S.salar]              -------------MSTAAVADRGSVHPVRGPGDGMETDEPPVAKEI----GVVDTPL--F     41
NP_001088348.2[X.laevis]             -----------MAATMETLKTLATSTPLRGTGDGMETEAPLKSEVN----SP--------     37
XP_004938283.1[G.gallus]             --------------MTSLEKVDYASSPIRGPGDGMETEQMGESLEV----SAGASAT---     39
NP_001153680.1[M.musculus]           ME-------KMTTLKSSENKGILTSTPIRGAGDGMETEEPPKSVEV----THGVQPI---     46
Q9H2G9[H.sapiens]                    -----------MTTKNLETKVTVTSSPIRGAGDGMETEEPPKSVEV----TSGVQSR---     42
NP_001126836.1[P.abelii]             -----------MTTKNLETKVTVTSSPIRGAGDGMETEEPPKSVEI----TSGVQSR---     42

XP_019850840.1[A.queenslandica]      ------------------------------------------------------------     0
NP_649889.1[D.melanogaster]          KRPQLSRKDSSVVQEAVMGRKTSTSSGHSS------------NLPLPGLHTLYRRPENV-     96
XP_002164131.1[H.vulgaris]           ---------------------DEFSN--IE---------KIKLFKSPPNFQLNQQEQA--     67
XP_001635606.1[N.vectensis]          -----------LKQ----KPQIHFSFGHVELMLESDTEEKEEGTKNTENDTIRKHPKK--     95
XP_019628762.1[B.belcheri]           ------------------------------------------------------------     0
XP_032834970.1[P.marinus]            --PPLPVRS--VQP----APRS--SWQHRPPA----------KP-APQHHHLPHQPRPLS     85
XP_014008079.1[S.salar]              SVPLLKVAS--PKQ----SPKT--T--HPSPA----------SLQHSPHSTPQQSPRA-S     80
NP_001088348.2[X.laevis]             ---------------------H---------------------------TKATQSPRK--     47
XP_004938283.1[G.gallus]             ---------------------N---------------------------HQVRHSPQK--     49
NP_001153680.1[M.musculus]           ---------------------N---------------------------QHVLPSPRK--     56
Q9H2G9[H.sapiens]                    ---------------------K---------------------------HHSLQSPWK--     52
NP_001126836.1[P.abelii]             ---------------------K---------------------------HHSLQSPWK--     52

XP_019850840.1[A.queenslandica]      --------------------------------MESCRRRKQALKEKYLKKAQDLI-----     23
NP_649889.1[D.melanogaster]          ----------------------------------TRSLAPVVPKGELVQMRPRLVTSADS     122
XP_002164131.1[H.vulgaris]           -------------------------------------IRPHNPDEDLIKYL---------     81
XP_001635606.1[N.vectensis]          ---ATKSTPVL-----NQIIERTSVDSSKTQGNDTHIFIKEEPKQEILQSSAST------     141
XP_019628762.1[B.belcheri]           --------------------------------MESAPFAFVKPQAHSSQ---SKK-----     20
XP_032834970.1[P.marinus]            PAAAAAATPLPHRPQQQQQQPVPVLAGG--QVELTVVQIQVPKEAILHHSARSL-----     138
XP_014008079.1[S.salar]              PRDIASAPQLP------GVLHLGKVPREA--CVVVEAVRIVVPRAAISRSGGHMG-----     127
NP_001088348.2[X.laevis]             -KSTVSPSLSP------GVLQLGKMQSDK--AVEIEAIRILIPKAAITHTATTKN-----     93
XP_004938283.1[G.gallus]             -K--AVSSLSP------GVLQLGQIHADK--SVEVEAVRILVPKAAITHVVATKN-----     93
NP_001153680.1[M.musculus]           ----KVSSDSP------GVLQLGKILNER--TVEVEAVRIFVPKAAITHDIPTKN-----     99
Q9H2G9[H.sapiens]                    -K--AVPSESP------GVLQLGKMLTEK--AMEVKAVRILVPKAAITHDIPNKN-----     96
NP_001126836.1[P.abelii]             -K--AVPSESP------GVLQLGKMLTEK--AMEVKAVRILVPKAAITHDIPNKN-----     96
                                                                                     :

XP_019850840.1[A.queenslandica]      -SK-K--------KYEKYKV-------------------------TDVSWSDPL-----I     43
NP_649889.1[D.melanogaster]          LPEHKKTKAPKFVPYEPYPGAVNPMISEPTNKHKIHRDKNNLDIAVLVDQVSTLRTQELE     182
XP_002164131.1[H.vulgaris]           ---------------------------------------------------------SST     84
XP_001635606.1[N.vectensis]          ---------------------------------------------------------LCD     144
XP_019628762.1[B.belcheri]           -TTRT-------HGG-----------------------GG---------KDSTL--N---     35
XP_032834970.1[P.marinus]            -PALELGVKPDSLEA-----------------------GG---------SAAAA--AAAA     163
XP_014008079.1[S.salar]              -PAEEK--------G-----------------------EA---------WAG----QQME     142
NP_001088348.2[X.laevis]             -SKHLE--------S-----------------------NG---------HSST----ELA     108
XP_004938283.1[G.gallus]             -AKVAK--------S-----------------------AG---------HKGDA--FHQS     110
NP_001153680.1[M.musculus]           -TKVKS--------L-----------------------GH---------HREEL--HNQA     116
Q9H2G9[H.sapiens]                    -TKVKS--------L-----------------------GH---------HKGEF--LGQS     113
NP_001126836.1[P.abelii]             -TKVKS--------L-----------------------GH---------HKGEF--LGQS     113

XP_019850840.1[A.queenslandica]      TSTPLITRTRSTLP---SSLRLSPHRDITELE---RM----IEDQNKAMKELKRLLLASV     93
NP_649889.1[D.melanogaster]          TETTDIKETDTASSHEVNSLKEELAKMRDERNYFQAQ----YKFQTQVNSELKSLLVASV     238
XP_002164131.1[H.vulgaris]           RPHIPDQELLESLQSKVKFLQEYNEKLIDEKLKVSHQ----LATQTQINNELKNLLVASI     140
XP_001635606.1[N.vectensis]          KPTGSSTEELKKLQSKVEELEEQTSRLTDEKTKLTIQ----LGIQTEVNSDLKKLLVASM     200
XP_019628762.1[B.belcheri]           -----------AMRRSQLSRTRSETRIFGQVQGFQKPKLNDVPLSSA-KITQFKAMEERG     83
XP_032834970.1[P.marinus]            AVTAALRQEVKELKVEVKRLEESERRLMDDKESVVSQ----LRSQTEVNRELKKLLVASV     219
XP_014008079.1[S.salar]              ERPPSPSLPLEDLRGAMEKLQNSERRLLQDKEGLLNQ----LHVQTEVNRELKKLLVASV     198
NP_001088348.2[X.laevis]             ESVTDPWKQLSELKSTLEKLKNSERRLLQDKEGLANQ----LKVQTEVNRELKKLLVASV     164
XP_004938283.1[G.gallus]             DGTADPKKEQTELKNAIEKLKNSEKRLLQDKEGLSNQ----LRIQTEVNRELKKLLVASV     166
NP_001153680.1[M.musculus]           EVVTDPRKELSEVKKVLEKLKNSERRLLQDKEGLSNQ----LRVQTEINRELKKLLVASV     172
Q9H2G9[H.sapiens]                    EGVIEPNKELSEVKNVLEKLKNSERRLLQDKEGLSNQ----LRVQTEVNRELKKLLVASV     169
NP_001126836.1[P.abelii]             EGVIEPNKELSEVKNVLEKLKNSERRLLQDKEGLSNQ----LRVQTEVNRELKKLLVASV     169
                                                              :   :              ..         :

XP_019850840.1[A.queenslandica]      GGEMGTEIETLIRDKVTLESKLNHSITELMEMNEEIEGLTIECDVWKCKVLASRLLIDEL     153
NP_649889.1[D.melanogaster]          GEDLQTRVNLLTEDKLQLARALLDTANNLTTHTEQIEFLAGQCEVWRSKFLASSVMVEEL     298
XP_002164131.1[H.vulgaris]           GDDLETRYEKMILNRIQSDLELKRLINLVEEYQEEKQQAYIQVDVWRSKFLASRVMNEEL     200
XP_001635606.1[N.vectensis]          EEELENRMEALAHRNAQLRVEVEHWQTLCDEYAEESDRLAIDCDVWRTKFMASRMMSDQL     260
XP_019628762.1[B.belcheri]           SSDLQYQFERMAREKAQLSVDHDNLVQQYADLSEEVERLSIQCDVWRSKFLGSRTQVSQL     143
XP_032834970.1[P.marinus]            GDDLQFHFERAVRERGRLQAEAQELSRSLASASEQLERIGIQCDVWRSKFLASRVMSEEV     279
XP_014008079.1[S.salar]              GDDLQYHFERLAREKNQMILDNEALGRSLASTAEQLERMSIQCDVWRTKFLASRVMAEEL     258
NP_001088348.2[X.laevis]             GDDLQYHFERMAREKNQLILENEVLSQNTAQLSEQLERMSIQCDVWRSKFLASRVMADEL     224
XP_004938283.1[G.gallus]             GDDLQYHFERMAREKNQLILENEVLGRNLSQLSEQLERMSIQCDVWRSKFLASRVMADEL     226
NP_001153680.1[M.musculus]           GDDPQYHFERLAREKNQLILENEALGRNTAQLSEQLERMSIQCDVWRSKFLASRVMADEL     232
Q9H2G9[H.sapiens]                    GDDLQYHFERLAREKNQLILENEALGRNTAQLSEQLERMSIQCDVWRSKFLASRVMADEL     229
NP_001126836.1[P.abelii]             GDDLQYHFERLAREKNQLILENEALGRNTAQLSEQLERMSIQCDVWRSKFLASRVMADEL     229
                                       :   . :     .                  *: :    : :**: *.:.*    .::

XP_019850840.1[A.queenslandica]      REWKGNHYHL---SVNTLQTLLDERDQLMNHIKAIHQLVNNISRKLMSTSSTANSTASGI     210
NP_649889.1[D.melanogaster]          ARWKADLTQKNQLLNESTKQLLHATHQIREIQLDMLKQLKFLA-------KIRFLN----     347
XP_002164131.1[H.vulgaris]           TQWKSALYYKYRDSHTALQSLLEEHSKIRVLNDATRIGLIKIAELLNS-KDASLD-----     254
XP_001635606.1[N.vectensis]          TCWKTTLYTRYRQALAALQRMLDERDELKHYLVHTTRLLESLDYVVKCSGGSFSDT----     316
XP_019628762.1[B.belcheri]           IDLRNAIYQQFGKAQAAIDNVMKERAPIRKEMVETYRLLDQLVKALRWGKPQPLQ-----     198
XP_032834970.1[P.marinus]            AGARVHALRLFREAQSAVHDLFSERTQLCVDLQDTHRLLEELVLALQWGRQPTYS-----     334
XP_014008079.1[S.salar]              TNARVSLQHQTREAQSAITDLLSERDEFSRDMMLTHRSLEQLLVSLQWGRQQTYY-----     313
NP_001088348.2[X.laevis]             TNIRTNLQRQNRDAQSALQDLLSEREQFRHEMSETQKTLEALLVSLQWGRQKTYA-----     279
XP_004938283.1[G.gallus]             TNTRAILQRQTRDAQTAIQDLLNERDQFRQEMIHTQKLLEELMVSLQWGRQQTYY-----     281
NP_001153680.1[M.musculus]           TNFRVVLQRQNRDAQSAIQDLLSEREQFRQEMTSTQKFLEELLVSLQWGREQTYS-----     287
Q9H2G9[H.sapiens]                    TNSRAALQRQNRDAHGAIQDLLSEREQFRQEMIATQKLLEELLVSLQWGREQTYS-----     284
NP_001126836.1[P.abelii]             TNSRAVLQRQNRDAHGAIQDLLSEREQFRQEMIATQKLLEELLVSLQWGREQTYS-----     284
                                        :            :   ::     :          :  :

XP_019850840.1[A.queenslandica]      KIATVTPVQLQPIHQGCNVLELAKNTSVAVQMISDK----ISARH---------------     251
NP_649889.1[D.melanogaster]          ------LPATD-------VISLSAENLNILQRMVLH------------------------     370
XP_002164131.1[H.vulgaris]           --------SEMR----LTLVDTAFINHDLVESLIE----IIEESSWAEKPNEI-K-----     292
XP_001635606.1[N.vectensis]          ------LMDRHP-IQGQSILEMATITSCLAQDVTAQVQGFIPSGS---------------     354
XP_019628762.1[B.belcheri]           -------QAANP----RNMKELVQANHRLVSAVSAQLLGNIKPMNSANQETVE-------     240
XP_032834970.1[P.marinus]            -------LGTRR---AHNSLELAEACTTLASLLTQRLLGGGSGSVKSSPPPAAAGGGGAA     384
XP_014008079.1[S.salar]              -------PSAQP----LSTGELSLANHKLAEAINSRLLGKVGPVG---------------     347
NP_001088348.2[X.laevis]             -------PIPQP----HTTTELIAANLKLAKSIQSHLIGNTGSDH---------------     313
XP_004938283.1[G.gallus]             -------PSAQP----YTTTELASVNCKLAKAVSSHLLGNVGTSS---------------     315
NP_001153680.1[M.musculus]           -------PNTQP----HSTADLALTNHGLAQAIHAHLLGNVGISH---------------     321
Q9H2G9[H.sapiens]                    -------PSVQP----HSTAELALTNHKLAKAVNSHLLGNVGINN---------------     318
NP_001126836.1[P.abelii]             -------PSVQP----HSTAELALTNHKLAKAVNSHLLGNVGINN---------------     318
```

```
                                                          .         . :

XP_019850840.1[A.queensladica]       -----------------------EATPMPSLLSLNTSLTEGEKLAHKLLYPV--PKETTP           286
NP_649889.1[D.melanogaster]          ----------------TGVGIPEETLKLSSASTSPLC--EAEKYAVRALEFISQPLMATD           412
XP_002164131.1[H.vulgaris]           ----------------QNIFFKQFKNHLKEIYVLPSELSTAEQLAHEIVTNT-G-IQTEE           334
XP_001635606.1[N.vectensis]          --------------------LPTHALLLNEEKEPALDSTPAEKMALQLLTSS-IELQSDD           393
XP_019628762.1[B.belcheri]           ----------------SGIG-----------------LTNAEAAATQVLNMN-F------           260
XP_032834970.1[P.marinus]            GGAGGGGGAAGGGGAAGGGGAGGGGAAVAAQPPAQLCSTAAERMAQQVLRMT-EVRMTDP           443
XP_014008079.1[S.salar]              --------------SSSGAGL---RKGRQASELPNTSHTPAEKMAEKVLKIL-DPISCSD           389
NP_001088348.2[X.laevis]             --------------Q------------KPAAKPIEYCSTPAENMAETVLKAL-DPVSCTD           346
XP_004938283.1[G.gallus]             --------------P------------KKTSTAVEFCNTPAEKMAERVLRVL-DPAARTE           348
NP_001153680.1[M.musculus]           --------------Q------------KKIPTTVEFCSTPAEKMAEKVLRIL-DPVACTE           354
Q9H2G9[H.sapiens]                    --------------Q------------KKIPSTVEFCSTPAEKMAETVLRIL-DPVTCKE           351
NP_001126836.1[P.abelii]             --------------Q------------KKIPSTVEFCSTPAEKMAETVLRIL-DPVTCTE           351
                                                                             .*  *   :

XP_019850840.1[A.queensladica]       TSRGLPHLSFSEKTLMEQRKHQQARTLPWTD-DRHDGNNDIANNNSYYFCNKCNGRAIMI           345
NP_649889.1[D.melanogaster]          EAIR-------------ALFD-----QAQ---RPH-YVQSPASEVTPACDNQQLDKMQ--           448
XP_002164131.1[H.vulgaris]           L----------------VRMR-----RDYLAHNRISHFLSTNYHVTYNCCNSCKGRIIHL           373
XP_001635606.1[N.vectensis]          VPSV-------DSSVLRR-MG-----KQYIGNSYHSRLMTNSFRVTYDCCDRCTGPVHVV           440
XP_019628762.1[B.belcheri]           --------DLQLLSPESAAPV-----GKMSG-RFH--PQHKYEEITVNCCNKCKGEIKVV           304
XP_032834970.1[P.marinus]            ML----VDDAAGGAAGRLLTS-----KPSPG-RFH--PYTRYEHITFTCCSRCTGDIVVV           491
XP_014008079.1[S.salar]              NEEKISPLSISDSSPSAFLTN-----KKSIG-RFH--PYTRYENITFNCCERCSGDILVL           441
NP_001088348.2[X.laevis]             NSSD---ISLSDSSP--FLLA-----KKNIG-RFH--PYTRYENITFNCCNQCRGELIAV           393
XP_004938283.1[G.gallus]             TSTE---ASFSETSPSSFLST-----KKNIG-RFH--PYTRYEDITFNCCDHCQGELIAL           397
NP_001153680.1[M.musculus]           SSPD---NQFAESSPTTLLTT-----KKNIG-RFH--PYTRYENITFNCCNHCQGELIAL           403
Q9H2G9[H.sapiens]                    SSPD---NPFFESSPTTLLAT-----KKNIG-RFH--PYTRYENITFNCCNHCRGELIAL           400
NP_001126836.1[P.abelii]             SSPD---DPFFESSPTTLLAT-----KKNIG-RFH--PYTRYENITFNCCNHCQGELIAL           400
                                                                                  :    .   .

XP_019850840.1[A.queensladica]       I          346
NP_649889.1[D.melanogaster]          -          448
XP_002164131.1[H.vulgaris]           -          373
XP_001635606.1[N.vectensis]          -          440
XP_019628762.1[B.belcheri]           -          304
XP_032834970.1[P.marinus]            -          491
XP_014008079.1[S.salar]              -          441
NP_001088348.2[X.laevis]             -          393
XP_004938283.1[G.gallus]             -          397
NP_001153680.1[M.musculus]           -          403
Q9H2G9[H.sapiens]                    -          400
NP_001126836.1[P.abelii]             -          400
```
