# Supplementary material for: An Evolutionary Perspective on the Origin, Conservation and Binding Partner Acquisition of Tankyrases
Source: Biomolecules. 2022 Nov 15;12(11):1688. doi: 10.3390/biom12111688 (PMC9688111; doi:10.3390/biom12111688)
Supplement: Supplementary file 1 [file biomolecules-12-01688-s001.zip › biomolecules-1913719-supplementary-11.17 -done/Data_7/IRAP.pdf]

IRAP

Homo sapiens:

```
>sp|Q9UIQ6|LCAP_HUMAN
MEPFTNDRLQLPRNMIENSMFEEEPDVVDLAKEPCLHPLEPDEVEYEPRGSRLLVRGLGE
HEMEEDEEDYESSAKLLGMSFMNRSSGLRNSATGYRQSPDGACSVPSARTMVVCAFVIVV
AVSVIMVIYLLPRCTFTKEGCHKKNQSIGLIQPFATNGKLFPWAQIRLPTAVVPLRYELS
LHPNLTSMTFRGSVTISVQALQVTWNIILHSTGHNISRVTFMSAVSSQEKQAEILEYAYH
GQIAIVAPEALLAGHNYTLKIEYSANISSSYYGFYGFSYTDESNEKKYFAATQFEPLAAR
SAFPCFDEPAFKATFIIKIIRDEQYTALSNMPKKSSVVLDDGLVQDEFSESVKMSTYLVA
FIVGEMKNLSQDVNGTLVSIYAVPEKIGQVHYALETTVKLLEFFQNYFEIQYPLKKLDLV
AIPDFEAGAMENWGLLTFREETLLYDSNTSSMADRKLVTKIIAHELAHQWFGNLVTMKWW
NDLWLNEGFATFMEYFSLEKIFKELSSYEDFLDARFKTMKKDSLNSSHPISSSVQSSEQI
EEMFDSLSYFKGSSLLLMLKTYLSEDVFQHAVVLYLHNHSYASIQSDDLWDSFNEVTNQT
LDVKRMMKTWTLQKGFPLVTVQKKGKELFIQQERFFLNMKPEIQPSDTSYLWHIPLSYVT
EGRNYSKYQSVSLLDKKSGVINLTEEVLWVKVNINMNGYYIVHYADDDWEALIHQLKINP
YVLSDKDRANLINNIFELAGLGKVPLKRAFDLINYLGNENHTAPITEALFQTDLIYNLLE
KLGYMDLASRLVTRVFKLLQNQIQQQTWTDEGTPSMRELRSALLEFACTHNLGNCSTTAM
KLFDDWMASNGTQSLPTDVMTTVFKVGAKTDKGWSFLLGKYISIGSEAEKNKILEALASS
EDVRKLYWLMKSSLNGDNFRTQKLSFIIRTVGRHFPGHLLAWDFVKENWNKLVQKFPLGS
YTIQNIVAGSTYLFSTKTHLSEVQAFFENQSEATFRLRCVQEALEVIQLNIQWMEKNLKS
LTWWL
```

Pongo abelii:

```
>XP_024102718.1 leucyl-cystinyl aminopeptidase [Pongo abelii]
MEPFTNDRLQLPRNMIENSMFEEEPDVVDLAKEPCLHPLEPDEVEYEPRGSRLLVRGLGEHEMEEDEEDY
ESSAKLLGMSFMNRSSGLRNSATGYRQSPDGACSVPSARTMVVCAFVIVVAVSVIMVIYLLPRCTFTKEG
CHKKNQSIGLIQPFATNGKLFPWAQIRLPTAIVPLHYELNLHPNLTSMTFRGSVTISVQALQVTWNIILH
STGHNISRVTFMSAVSSQEKQVEILEYAYHGQIAIVAPEALLAGHNYTLKIEYSANMSRSYFGFYGFSYT
DESNEKKYFAATQFEPLAARSAFPCFDEPAFKATFIIRIIRDEQYTTLSNMPKKSSVILEDGLVQDEFYE
SVKMSTYLVAFIVGEMKNLSQDINGTLVSIYAVPEKIGQVRYALETTVKLLEFYQNYFEIQYPLKKLDLV
AIPDFEAGAMENWGLLTFREETLLYDNNTSSVADRKLVTKIIAHELAHQWFGNLVTMKWWNDLWLNEGFA
TFMEYFSLEKIFKELSSYEDFLDARFKTMKKDSLNSSHPISSSVQSSVQIEEMFDSLSYFKGASLLLMLK
TYLSEDVFQHAVILYLHNHSYASIQSDDLWDSFNEVTNQTLDVKRMMKTWTLQKGFPLVTVQKKGKELFI
QQERFFLNMKPEIQPSDTSYLWHIPLSYVTEGRNYSKYQSVSLLDKKSGVINLTEEVLWVKVNINMNGYY
IVHYADDDWEALIHQLKINPYVLSDKDRANLINNIFELAGLGKVPLKRAFDLINYLGNENHTAPITEALF
QTDLIYNLLEKLGYMDLASRLVTRVFKLLQNQIQQQTWTNEGTPSMRELRSALLEFACIHNLGNCSTTAM
KLFDDWMASNGTQSLPTDVMTTVFKVGAKTDKGWSFLLGKYISIGSEAEKNKILEALASSEDVRKLYWLM
KSSLNGDNFRTQKLSFIIRTVSRHFPGHLLAWDFVKENWNKLVQKFHLGSYTIQNIVAGSTYLFSTKTHL
SEVQAFFENQSEATFRLRCVQEALEVIQLNIQWMEKNLRSLTWWL
```

Mus musculus:

```
>NP_766415.1 leucyl-cystinyl aminopeptidase [Mus musculus]
MESFTNDRLQLPRNMIENSMFEEEPDVVDLAKEPCLHPLEPDEVEYEPRGSRLLVRGLGEHEMDEDEEDY
ESSAKLLGMSFMNRSSGLRNSAAGYRQSPDGTCSLPSARTLVICVFVIVVAVSVIMVIYLLPRCTFTKEG
CHKTNQSAELIQPVATNGKVFPWAQIRLPTAIIPLCYELSLHPNLTSMTFRGSVTISLQALQDTRDIILH
STGHNISRVTFMSAVSSQEKQVEILEYPYHEQIAVVAPEPLLTGHNYTLKIEYSANISNSYYGFYGITYT
DKSNEKKYFAATQFEPLAARSAFPCFDEPAFKATFIIKITRNEHHTALSNMPKKSSVPAEEGLIQDEFSE
SVKMSTYLVAFIVGEMRNLSQDVNGTLVSVYAVPEKIGQVHHALDTTIKLLEFYQTYFEIQYPLKKLDLV
AIPDFEAGAMENWGLLTFREETLLYDNATSSVADRKLVTKIIAHELAHQWFGNLVTMQWWNDLWLNEGFA
TFMEYFSVEKIFKELNSYEDFLDARFKTMRKDSLNSSHPISSSVQSSEQIEEMFDSLSYFKGASLLLMLK
SYLSEDVFRHAVILYLHNHSYAAIQSDDLWDSFNEVTDKTLDVKKMMKTWTLQKGFPLVTVQRKGTELLL
QQERFFLRMQPESQPSDTSHLWHIPISYVTDGRNYSEYRSVSLLDKKSDVINLTEQVQWVKVNSNMTGYY
IVHYAHDDWTALINQLKRNPYVLSDKDRANLINNIFELAGLGKVPLRMAFDLIDYLKNETHTAPITEALF
QTNLIYNLLEKLGHMDLSSRLVARVHKLLQNQIQQQTWTDEGTPSMRELRSALLEFACAHSLENCTTMAT
NLFDSWMASNGTQSLPTDVMVTVFKVGARTEKGWLFLFSMYSSMGSEAEKNKILEALASSEDVHKLYWLM
KSSLDGDIIRTQKLSLIIRTVGRHFPGHLLAWDFVKENWNKLVHKFHLGSYTIQSIVAGSTHLFSTKTHL
SEVQAFFENQSEATLKLRCVQEALEVIQLNIQWMVRNLKTLSQWL
```

Gallus gallus:

```
>XP_424862.3 leucyl-cystinyl aminopeptidase isoform X1 [Gallus gallus]
MEPFPSDQIQLPRNMIENSMFEEEPDVVDLAKEPCLHPLEPDEVEYEPRTSRLLVRGLGEHEMDDDEEDY
ESSAKLLGMSFINRSSGLRNNMSVYRQSPDGSCSVPSTRTMLICTGVLVIAVSVIMVIYLLPKCTFTKEG
CHKKNRTMELIYPLATNGKLFPWAKIRLPSDVVPLHYDLDLQPNLTTLKFTGSVKIVVNVTQVTKKIVLH
SSGLNITKATITSTGGSQEKAVELLEYPLHDQIAVMAPESLLAGQNYTVNLEYSSNLSDTYYGFYRVSYK
DENSKQRWFAATQFEPLAARSAFPCFDEPALKATFSIRIKRDEKLSTLSNMPKKATTPVTKGIVQDEFFV
SLKMSTYLVAFVVADLKNISMETNGSLVSVHAIPQHINQVEYALNTAVKLLEFYEKYFLINYPLEKLDLV
AIPDFQSGAMENWGLITFRETTLLFDNNASSARDKKLITAVIAHELAHQWFGNLVTMEWWNDLWLNEGFA
TFMEYFAMEEIFPELHSDEDFLNLIFKAMMKDSLNSSHPVSSAVQSSEQIEEMFDALSYIKGASLLMMLK
HYLTKDVFQAGIEIYLHNHKYGSAQSDDLWDSMNEITNGTLDVKKLMKTWILHKGFPLVTVNRKGKIISL
HQEKFLYSVEPDNWTSDTSYLWDIPLTYTTNRCNFTHCINAYLLDQKSAVIELPEEVEWIKFNVDMNGYY
IVNYDEDWETLIDLLKKNHTALSAKDRANLINNIFSLASLGKEPLKKAFELIDYLKEESSTAPLSQALFQ
LGLIFGLLEKRGEQQLAARVMYRIECLLGNKIDQQNWTDDGTISERELRSMLLTFACTHDIRNCRTAASK
MFDEWMKSNGTMSLPSDLMKAIFITGAKTNDGWEFLLKMYSSPVPEAEKSKMIEALASTEDVRKMMWLMQ
NSLEGEVIRTQELSHIIATISHSLPGHLLAWDFVKENWEKLTRKFHLGSYTIQNIISSSTSQFATKVHLL
EVKTFFESKSEESSKLRCVKEAIDTIQLNIQWMETNLAKLQEWL
```

Xenopus laevis:

```
>NP_001088429.1 leucyl/cystinyl aminopeptidase L homeolog [Xenopus laevis]
METSNDWAQLPRNMIENSMFEEEPDVVDLAKEPTMNPLESEETEYEPRSSRLLVRGLGEQELDEEEEDYE
SSAKLLGMSFMNRSSGLRNSAANYRQVQDGFCPVPSARTVIVCVVVVIIAVSVVTVVYLLPKCTFTKDGC
HEKKHPLELVYPIATNKKVFPWAKSRLPDSIKPVHYTLTMHPNMTTLSFTGTVQIKLNITTSSKNIVLHS
SGLHIIKASVLVNGGTTMDAEVLEYPPFEEIAIISPESLLKGKECLLTIQYAANFSSTYYGFYKIDYMDK
SNKRSLAATQFEPLAARKAFPCFDEPAFKSTFQINIIRNDESMISLSNMPKAKTSKTSDGLLQDEYSTSV
RMSTYLVAFIVGDIKNTTQKTNDTLVSVYAVPDKTDQVKYALDSTVKLLDFYSNYYGIEYPLEKLDLVAI
PDFQAGAMENWGLITFRETTLLYKENSSSIEDKQSITTVIAHELAHQWFGNLVTMEWWNDLWLNEGFATY
MEYFSVSSVFPELNSDNSFLKMRFMALQKDSLNSSHPISTDVRSPEQIEEMFDDLSYIKGASILLMLKSL
LLEDVFHLCIRGYLKSHEYGSTTSDSLWDSLNVVTGGNPNVKNMMKTWTQKAGYPLVTALRKGEEITVRQ
ERFLRSSDHATNDSTVWHIPLTYVTGKCDAVEPDCEKIHLLKEATGTINVSSELPWVKFNVNMTGYYIVD
YGADGWDALIEQLHRDYTVLHSSDRANLIHDIFMLAGVGKVPLSKAFELLGYLVNETDSAPITQALHQFY
HIHGILLKRGLDDLSDKLMEHGLELLNNTLIKQTWMDEGTLAERQLRSSLLDFACSNGFPSCLEKATELF
NSWRVNKTRIPTDVMKVVFKVGARTTEGWTLLRSTYDSSIYEAEKRKILEALASTDNAKNLQWLMQESLD
GGSIRSQELPAVIGFICKRSPGYLLAWNFIKQNWDLITQKFMPGSFPIQNIVSKTTHQFSTDVHLNEVIA
FFNSTQEKSREMWCVKEAVETIKLNIEWMKNNFDSLKTWLLPSQ
```

Salmo salar:

>XP_014016586.1 PREDICTED: leucyl-cystinyl aminopeptidase isoform X1 [Salmo salar]
MEDPFDSERASLPRNMIENSMFEEEPDVVDLAKDSAAFPALEPDEVVYEPRSSRLLVRGLGENDLDDEEE
DYESSARLLGMSFMNRSSTHNPSSSPYNRQAPPRSCSCPSARMLVVGVFILVLVASMAMVLYFLPGCTFT
KAGCGKANSSTPMEPSYPNSTNGELFPWNELRLPASVRPVNYDLSLTPNLTSMTFTGRTVINMTILHNTK
HVVLHSSELIITKATFQVGEGKSVEVKVLEYKPWQQIAVSFPEDLKVGQVCVLTLDYAASLSHTYDGFYN
SSYNDKTGAKRVLAATQFEPQAARKAFPCFDEPAFKATFLVRITREPGYITLSNMPQAKTTTLPSGLLED
EFEQTAVNMSTYLVAFIVANFTSITRNVSNTLVSVYSVPEKKEHTHYALDTASKLLHFYNTFFKINYPLR
KLDLVAIPDFLAGAMENWGLITFRETSLLVGNQSSPLDKQLIANVVAHELAHQWFGNLVTMRWWNDLWLN
EGFATYWQYMSLMTEFPQLDLGNVFLGVRFKAMAKDSLNSSHPVSVSSQVTTPEQVSEMFDSVTYEKGAS
LLLMLNTTLPDGQFKEGLMKYLDTYRGSNTVTEDLWNSLTQAQVLPQQENVSDMMRTWTLQKGFPLVTVS
RKGGHVTLTQEHFLLSADNTTHTSSLWHIPVTYVNDSCSSAPSCRQIFMLRNKTATLNVSDSVKWLKLNY
MNTGFYIVHYGDEGWTTLIDALKTDINTLTAHDRASLIHNIFSLSRLGRVSFRQVLNLLDYATNETETAP
LTEALSQLSSVYRLLDKRQEQTLVARMKAYIWGHFGQLMDSQDWGEEESVSRQELRSALLEMACSLGRQN
CTDQATALYDQWTNSNQTKQIPGDLQRVVFSVAAQSNLGWLSLMEAYSSTTYDSEKRKILQALASTQDPQ
SIVWILSTGLEGGIIQTQELPLVISTMSDGFAGHLFVWDFVKENWDKIIEKFPVGSYPIQSIIKSTTSQF
STQTHLEEVQGFFSSLKERGSQMRSVQEALETIRLNQLWMDRNLPTLRIWL

Petromyzon marinus:

>XP_032824577.1 endoplasmic reticulum aminopeptidase 1-like isoform X3 [Petromyzon marinus]
MAGDLHGERAPLPKHMIENSMFEDDDEEEEDEEADAGMPNVDPGADPSLGNSVALSSPRTLAASSVPLYT
QHPLISSGGGSADRPTAPEDDSRPHTLSRLPIYAGDPDEFDEDFDDDNEESSRRLLGAAFFNRDPDEPRS
GRGPGGRAAPSTGPGGSDGPGVHLCCQQMSPRTLMFCAAGAIGVIVVIGIALSYVPCALGIGNGCASSNS
SAAGDDHDHRGGTTPSGAGNATFPFTADGEVFPWADDRLPRSVRPLHYHLCLHPNLTTLAVEGYVSVWLL
VDESTDHVVLHANNLTITESILVPADGAPAGGGADGSLVAKLLRYEPRDQLMVCTRKPLTQGQMYLLNIT
FTGRLSTSFLGFYRSTYRAEDGSDRPLAVTQFEPTSARESFPCFDEPDFKASFDISIVRDAAHTTLSNMP
LNETVTRKDGLLEDTFKGSVVMSTYLVAFIVSDFKSISNKTKHGTNVSIYTSPGKEQQGSFALENAIKFL
DFYEELFNISYPLPKQDLVAIPDFAAGAMENWGLVTYRETALLLGPKASGVRDTLWVAQVIAHELAHQWF
GNLVTMNWWNDLWLNEGFATFMEFVATGSLKPDWLMMDVFLTEVVMEALTQDSLPSSHAISSAAVGTMQI
GEMFDSVSYNKGASVLNMLRTFLTPAVFVDGIRDYLLVHSYGNAHTDDLWESLTQSAQQHLGHSVNVTEV
MGTWVRQRGFPLLTLTAVDKELKITYKQEPFPIEPAETSITPSLWQIPLTFITSDNSTSQSKLITDKQGD
IAVTKSTEWIKANTNMTGFYVVDYGKDGWATFANKLTTSKSFLSPADRANLLNDVFYLLNVGRVDVEDAM
PLLSFLPNETSVTPWKVALMHLDRVLLILSPYAHDVQLLQKHIVKLGEELLPRLPWNKSSEEVTVPQRSL
DALLVSVLCAYRSEKCLNRANVAYLQWSASESLSADLMSTVLRVAVRDGDRPTWDSVQEHYHRAKSSTEQ
AKYLEALASTSDEDQQWSLLENAMAGVEVRSQELPRIVGVMARTSWTGRLITWDFLRKRWSLLVEKFSLG
SFALSNMVMSVTSGFSSTYHLDQVEEFVASLRDSSGELRVFKEARDTIRANHHWLATNRVALHKWLTEQS

Branchiostoma belcheri:

>XP_019622707.1 PREDICTED: endoplasmic reticulum aminopeptidase 1-like [Branchiostoma belcheri]
MEEQEEETIDLTGPEEVAEDAEFASDEDSLLPQAVVQDGEGRRRKQRPSSRSGPLSWLSPRSKSAASRPL
RGAQRPLRRKLWQYAPKCSRDRCCFLFAICLTIFIVLVTSAIYMNSKHKSSCPYSFLLDTSEKKSAKKDP
IATNGERFPWDDVRLPSTIVPNSYLLHLHPNLTTFNFTGHVSINISVVKKTDLVVFHVKELNITQSSVKT
MNNVSVPIVKELEYTGNEQYCLRLGQGLSAGQNYTIVVHFSGELNDGLYGFYRSTYTLNKGKDGEESRVL
ATTQFEAANARAAFPCFDEPALKATFQLHMVREGRHSTLFNMPKDHIEDLLGGLKMDVFKKSVAMSTYLV
CFIVSDFRSMQLSTDTSDITVAVHAPEDRISQASFALDVAKTVLEYYETFFGVKYPLPKMDLVAIPDFSS
GAMENWGLVTYRETAILYDADNSSAGDKQWVAVVIAHEFAHQWFGNLVTMKWWNDLWLNEGFASFVEFIG
ADKVSPEWKMLDQFVVSDLQTALSADSLSNSHPISVTVHRPEEVEEIFDSISYDKGASILRMLESFLGRE
TFLQGLTSYLREHQFKNAATDDLWKALDQAAHGAGKSNINIKQVMDRWTNQMGYPVVMVTRQNSHLVLTQ
DRFLQMPPSPACGGVVEFTSPYRYEWIIPLTYITSTSPKEQQTVFMDTTQTTLSLDNNSPEWVKFNVNQT
GFYRVNYDNQNWDAFIHQLQNNHEALNSADRAGLIDDAFHLNRAGLFGLEKSLELVKYLKNERDYVPMAT
ALSGLGYITKLLETEDQDLYNNMKLFVLELLEDTIGEVGWEDQGDQLTKFLRSTVLGAACDYGHEQSTQQ
AHTLFANWMKGTQNVAANLKTVVYTSGVQHGGEEEWNFCWQQYTSATVAAEKRKLLYALANTRDNKLVTK
LLRYTLDSTKIRSQDTVRTITYVSRTAVGYQLAWNFVKSNWQTFLDRYGSGSFNMAELAKIPAHRFSTRR
QLEEVKSFFQSHDISAAARAARQTIETIQSNIFWLEKNKDKVKTWIKNYFMPPEGGKARSSEI

Drosophila melanogaster:

>NP_731786.1 uncharacterized protein Dmel_CG32473, isoform A [Drosophila melanogaster]
MVAWLTVKSVAIFLGLASTAFCVATIVLAVQNADLENDLQDALDKIDALTVDTTSTSSTSTSTSTTTTTA
NPNGPTDDTEPGSTTTPGVGGDTTPSAGTSPGGETSPSSSTGSTTNPIYPTLPTGLPDPEKIEWRLPTEL
TPIKYKVYYHPDLTTGACEGTVSIQFQLNAITNLIVLHAKELNVHSISILNMMARIRVAIDSINLDESRE
LLLITLREVLSMNKAYTLSASFDCDLSSLVGSYISNYTNADGVDRSIISTKFEPTYARQAFPCFDEPALK
AQFTITVARPSGDEYHVLSNMPVASEYVDGDITEVTFAETVPMSTYLAAFVVSDFQYKETTVEGTSIALK
VYAPPAQVEKTQYALDTAAGVMAYYINYFNVSYALPKLDLVAIPDFVSGAMENWGLVTFRETALLYDEST
SSSVNKQRVAIVVAHELAHQWFGNLVTMNWWNDLWLNEGFASFLEYKGVKQMHPEWDMDNQFVIEELHPV
LTIDATLASHPIVKSIESPAEITEYFDTITYSKGAALVRMLENLVGEEKLRNATTRYLVRHIYSTATTED
YLTAVEEEEGLEFDVKQIMQTWTEQMGLPVVEVEKSGSTYKLTQKRFLANEDDYAAEAEASSFNYRWSIP
ITYTSSINSEVQSLIFNHNDNEATITLPEEASWIKINTNQVGYYRVNYGSEQWAELISALKNSRETFSTA
DRAHLLNDANTLAAAGQLNYSVALDLISYLESEQDYVPWSVGTSALATLRNRVYYTDLYTNYTTYARKLL
TPIVEKVTFTVAADHLENRLRIKVLSSACSLGHESSLQQAVTLFNQWLASPETRPNPDIRDVVYYYGLQQ
VNTEAAWDQVWKLYLDESDAQEKLKLMNCLTAVQVPWLLQRYINWAWDESNVRRQDYFTLLGYISTNPVG
QSLVWDYVRENWEKLVDRFGINERTLGRLIPTITARFSTETKLEEMQQFFAKYPEAGAGTAARQQALEAV
KANIKWLAANKAQVGEWLANYVQQSSVTNRIQ

Hydra vulgaris:

>XP_012561211.1 PREDICTED: aminopeptidase N-like [Hydra vulgaris]
MSEASTGLLNESKNKSNKYEGSAWSQHWKLILTGVGVVFLIALGVIFGVILNTDRENKLKNSKREELFPY
SNIRLPLNVIPERYKIYLHPNITDNKFGFTGTVRILINITEETDSVLLHIKDLNISEVKCYHGSSAMSKH
KGPEDSQQVPVKDHLISVEHEFLMIRMKEQHELEVGKQYTLFIRFNGRLSNGLEGFYKSSYTTSKGEKRY
LATTHFEATQARAAFPCFDEPALKALFEIIMVREPQHTALSNMPIVKTINRTDGLKEDHFQQSLMMSTYL
VAFVVCDYGYKSAKTSRGIEVKVWAPKEQIEQANFAIYAAPKVLDYYETFFQVNFPLPKQDLIAIPDFAA
GAMENWGLITYRLTSILYDEKESSSANKQWVAVVIAHELAHQWFGNLVTMKWWNDLWLNEGFAAFVEFIG
ANITEPSWQMMDQFIVDDTQNSLTLDSSSNSHPISVTVNDPAQINEIFDTISYDKGASIIRMMKNFLGSD
VFHTGLTDYLNKYKFKNAVSDDLWACLTKAANNTIDVKSVMDTWTLQMGYPLITITKNHEQSEKGLVTQE
HFLIDVDRKTAASPFNYKWDVPITFYFEHKKEKQLVWFNRSADSINIPMMNASGWIKANIDQLNFYRVNY
DEDNWNLLSKQLQDNHKAFSTSDRSNLIDDAFELAKAGKLDQIKALEMTAYLKNEDEYVPWITALGSLGY
IGGLLQGRSCYSSYQKYIIQQVKPIVDKLGWSDEGTHLNRYLRGAALRSSVMHNDTDSVKRALEIFDRFM
NNHESVAPNLRSTVYLAGIKYGGKEQWEFMLNKYLNSPFPSEQRKIMFALADSSDESILKKYLSWSMNTS
IIRTQDTCGVIEHISTNIKGTKMAEDFVIKNWEKLFERYGKGSFDMSSLIKTVFARMKTKEDLKKVSEIC

Nematostella vectensis:

>XP_032239104.1 aminopeptidase N isoform X1 [Nematostella vectensis]
MDEAYDNGSPNQESSDSSFEDDIDSIALLPERKTRPRRLRRRIRIFEDNGGHLRTTNRMCSRRTIVYST
ITAVLLICVVIISVFVNPSTSDEFEEKTEKRSSEFQDEPEKKDDSSFYEDGHGRKFAWQKIRLPSSVTPE
EYTVILRPKLDPDFTFSGNVSVRVKCNEDTDYIFIHAKQMRLTKFEVLNQGKEPLKIMETANCEKLEMFS
IKVKGGLKKGESYVLQIDFNAVLAEKLTGFYKSSYKDKDGNTRYLATTHFEPTDARAAFPCFDEPALKAV
FNMVIYRKAEHVSLSNMPIKETFKDKESGQVIDVFEPSVKMSTYLVAFVVCDFKSKEATTKRGTLVRVWA
PEDNIDEGDYALSEAVKILSYYEKFFAVRYPLPKQDLIAIPDFAAGAMENWGLITYRLTSLLYDPEVSSD
SNKQWVAVVVAHELAHQWFGNLVTMKWWNDLWLNEGFASFVENIGVNHTTPEWRMMEQFLLDKTQLSMNL
DQLSNSHPISVVVKDPAEINSLFDTISYDKGAAIIRMLKSFLGDDVFQKGLQKYLNKHKFGNAETNQLWD
AFTEESSVSASSTKNFRDVKSVMDTWTLQMGFPVVTIKQRGDSAVASQKHFRIHPKVKPSLRSQFDYKWI
IPFTYYTQNDKTKKKAWIEKDNGREGEGILQFDYNPATSGWIKANYEQHGFYRVNYDAENWERLKQQLDT

```
DHEKLSAADRAGLLDDAFNLARAGELPLTTALDLTKYLTKEEMYVPWAAALSNMGFLESRLCENEEHMTL
YKKYALQQLIPIVRKLGWDDKGSHLQKYLRSYVLKLCARYGDVECATAVKSRFADWMRGESLPPNLRSVI
YDTGVHLGGEKEFKYMYEQYNKSTVAAEKRKLLFAMSATQNPALMKGLLDMSMSTQIRSQDTVSVITSVA
SNCKGRNLAWDFVKKHWKTLFKRYGHGSFDFARLVSRTTTHFSTPQKLKEIQEFFKKHELGSGKLASKQA
EEGISSNIDWMKNNIEVALKWLEEHTQAESKPADGDLGVANVERLENILAPEGFQEEYQDRENQEKPYQN
KRYYSNQHYGRGDDQDRRFSNEYNDDDYEPERRGYEYQ
```

Amphimedon queenslandica:

```
>XP_003386159.1 PREDICTED: glutamyl aminopeptidase-like isoform X1 [Amphimedon queenslandica]
MSRHRNGFEQLDGESSDTDMNLEDNESSALTGKKKRYLVNDEEGPLCSPGSLKLSKRELICILIGGIIII
VAAIVFVIIAVVLSRSSGNGSPGGSDDKPWTNVRLPKTLTPALYRVSLDTDLNTFRVNGFVSVDISVNQS
TDLVIFHAKDMTLNTVSLTKGVRGDQLGISRQFFYSDNDFYVIQLADSLDTNDNLQLNISFNYTLRDDLV
GFYKSSYSLADNEVHYLATTQFEPTDARRAFPCFDEPAMKANFSIELTHANRYNAVSNMPVARRVSKAND
KATTSFNTSYKMSTYLVAFVISDFNCSDSQTVNGHIQVRVCARPDVFSDTSYALSVGKSVIGYYEEFFGV
QYPLPKQDLFAIPDFAVGAMENWGLITYRETALLYNSTQNPAVNKQRVAVVVAHELAHQWFGNLVTMSWW
DGLWLNEGFASYVEYIGTDHVQPDWMMLEQFFIDTVQTAYDADGLNWSHPIIQQVNNPDEINGLFDSISY
DKGASLIQMLRGYIGNESFTNGLTLYLKNNKFGNTETYELWDALNEVSSSDVSVSQMMDTWTKQMGYPVV
TVSASDNNRATVSQKRFFQIPLPEGEQPAASPYNYVWIIPFDYITENGNSVTKKLVSNQQDTITWDSSND
GFIKANANQTGFYRVNYDVGNWQSITAHLMTPPNNRPQILSAVDRAGLLEDAFSLSTSGLLNITVALNLS
RYLVNEEDYAPWMTALRWFSIFSDKLSTNGQYGNFKRYVSSLMGNITRKLSFNKTGLSHLQILLRTYVLL
SGYKYGDISIADTSLTMFRNWMTDGISVPPDLRLVVYRVAIAAGGETEWNYLWSWYKNTTNPYEKQICLS
ALAQSKEYWILSRYLEYSMSQVRSQDTLYVIRSVARNVNGRYLAWNFVRDNYDTIFKKYGGGSFSFSRLI
RSITGSFATSWELQEVESFFGKVDVGSASLALQQSKEIVRGNIAWLDNNESVIGEWMNEYLSGAMSA
```

Alignment

```
XP_032824577.1[P.marinus]             MAGDLHGERAPLPKHMIENSMFEDDDEEEEDEEADAGMPNVDPGADPSLGNSVALSSPRT    60
XP_014016586.1[S.salar]               MEDPFDSERASLPRNMIENSMFEEEPDVVDLAKDSAAFPAL-------------------    41
NP_001088429.1[X.laevis]              --METSNDWAQLPRNMIENSMFEEEPDVVDLAKEPTM-NPL-------------------    38
XP_424862.3[G.gallus]                 -MEPFPSDQIQLPRNMIENSMFEEEPDVVDLAKEPCL-HPL-------------------    39
NP_766415.1[M.musculus]               -MESFTNDRLQLPRNMIENSMFEEEPDVVDLAKEPCL-HPL-------------------    39
Q9UIQ6[H.sapiens]                     -MEPFTNDRLQLPRNMIENSMFEEEPDVVDLAKEPCL-HPL-------------------    39
XP_024102718.1[P.abelii]              -MEPFTNDRLQLPRNMIENSMFEEEPDVVDLAKEPCL-HPL-------------------    39
NP_731786.1[D.melanogaster]           ---------------------------------------------------MVA-----W    4
XP_003386159.1[A.queenslandica]       ------------------------------------------------------------    0
XP_019622707.1[B.belcheri]            ------------------------------------------------------------    0
XP_012561211.1[H.vulgaris]            ------------------------------------------------------------    0
XP_032239104.1[N.vectensis]           ------------------------------------------------------------    0

XP_032824577.1[P.marinus]             LAASSVPLYTQHPLISSGGGSADRPTAPEDDSRPHTLSR---LPIYAGDPDEFDEDFDDD    117
XP_014016586.1[S.salar]               --------------------------EPDEVVYEPRSSR---LLVRGL--GENDLDDEEE    70
NP_001088429.1[X.laevis]              --------------------------ESEETEYEPRSSR---LLVRGL--GEQELDEEEE    67
XP_424862.3[G.gallus]                 --------------------------EPDEVEYEPRTSR---LLVRGL--GEHEMDDDEE    68
NP_766415.1[M.musculus]               --------------------------EPDEVEYEPRGSR---LLVRGL--GEHEMDEDEE    68
Q9UIQ6[H.sapiens]                     --------------------------EPDEVEYEPRGSR---LLVRGL--GEHEMEEDEE    68
XP_024102718.1[P.abelii]              --------------------------EPDEVEYEPRGSR---LLVRGL--GEHEMEEDEE    68
NP_731786.1[D.melanogaster]           LTVKSVAIFLGLAS--TAFCVATIVLAV----QNADLENDLQDALDKIDALTVDTTSTSS    58
XP_003386159.1[A.queenslandica]       ------------------------------------MSRHR-NGFEQLDGESSDTDMNLE    23
XP_019622707.1[B.belcheri]            ------------------------------------MEEQEEETIDLTGPEEVAEDAEFA    24
XP_012561211.1[H.vulgaris]            ------------------------------------------------------------    0
XP_032239104.1[N.vectensis]           ----------------------------------------MDEAYDNGSPNQESSDSSFE    20

XP_032824577.1[P.marinus]             NEESSRRLLGAAFFNRDPDEPRSGRGPGGRA-APSTGPGGSDGPGVHL-----------C    165
XP_014016586.1[S.salar]               DYESSARLLGMSFMNRSSTHNPSSSPYNR-Q-A------------PPR-----------S    105
NP_001088429.1[X.laevis]              DYESSAKLLGMSFMNRSSGLRNSAANY-R-Q-V------------QDG-----------F    101
XP_424862.3[G.gallus]                 DYESSAKLLGMSFINRSSGLRNNMSVY-R-Q-S------------PDG-----------S    102
NP_766415.1[M.musculus]               DYESSAKLLGMSFMNRSSGLRNSAAGY-R-Q-S------------PDG-----------T    102
Q9UIQ6[H.sapiens]                     DYESSAKLLGMSFMNRSSGLRNSATGY-R-Q-S------------PDG-----------A    102
XP_024102718.1[P.abelii]              DYESSAKLLGMSFMNRSSGLRNSATGY-R-Q-S------------PDG-----------A    102
NP_731786.1[D.melanogaster]           TST-S--------------TSTTTTTANPNGPTDDTEPGSTTTPG---------------    88
XP_003386159.1[A.queenslandica]       DNESS----------ALTGKKKRYLVNDEEGPLCS--PGS-------L---------KLS    55
XP_019622707.1[B.belcheri]            SDEDS--LLPQAVVQDGEGRRRKQRPSSRSGPLSWLSPRSKSAASRPLRGAQRPLRRKLW    82
XP_012561211.1[H.vulgaris]            MSEASTGLLNESKNKSNK--------YE--GS---------------------------AW    24
XP_032239104.1[N.vectensis]           DDIDSIALLPERKTRPRRLRRRRIRIFEDNGG-------------HLRT------TNRM    60
                                          *

XP_032824577.1[P.marinus]             CQQMSPRTLMFCAAGAIGVIVV--IGIALSYVPCALGIGNGCASSNSSAAGDDHDHRGGT    223
XP_014016586.1[S.salar]               CSCPSARMLVVGVFILVLVASM--AMV-LYFLPGCTFTKAGCGKANSS------------    150
NP_001088429.1[X.laevis]              CPVPSARTVIVCVVVVIIAVSV--VTV-VYLLPKCTFTKDGCHEKKH-------------    145
XP_424862.3[G.gallus]                 CSVPSTRTMLICTGVLVIAVSV--IMV-IYLLPKCTFTKEGCHKKNR-------------    146
NP_766415.1[M.musculus]               CSLPSARTLVICVFVIVVAVSV--IMV-IYLLPRCTFTKEGCHKTNQ-------------    146
Q9UIQ6[H.sapiens]                     CSVPSARTMVVCAFVIVVAVSV--IMV-IYLLPRCTFTKEGCHKKNQ-------------    146
XP_024102718.1[P.abelii]              CSVPSARTMVVCAFVIVVAVSV--IMV-IYLLPRCTFTKEGCHKKNQ-------------    146
NP_731786.1[D.melanogaster]           ----------VG-GDTTPSAG---------TSPG-------GETSPSSST--------GS    113
XP_003386159.1[A.queenslandica]       KRELICIL--IG-GIIIIVAAIVFVIIAVVLSRS--------------------------    86
XP_019622707.1[B.belcheri]            QYAPKCSRDRCC-FLFAICLT---IFIV-LVTSAIYMNSKHKSSCPYSFL--------LD    129
XP_012561211.1[H.vulgaris]            SQHWKLILTGVG-VVFLIALG---VIFGVILNTDRE------------------------    56
XP_032239104.1[N.vectensis]           CSRRTIVYSTIT-AVLLICV----VIISVFVNPSTSDEFEEKTEKRSSEF--------QD    107

XP_032824577.1[P.marinus]             TPSGAGNATFPFTADGEVFPWADDRLPRSVRPLHYHLCLHPNLT--TLAVEGYVSVWLLV    281
XP_014016586.1[S.salar]               ---TPMEPSYPNSTNGELFPWNELRLPASVRPVNYDLSLTPNLT--SMTFTGRTVINMTI    205
NP_001088429.1[X.laevis]              ----PLELVYPIATNKKVFPWAKSRLPDSIKPVHYTLTMHPNMT--TLSFTGTVQIKLNI    199
XP_424862.3[G.gallus]                 ----TMELIYPLATNGKLFPWAKIRLPSDVVPLHYDLDLQPNLT--TLKFTGSVKIVVNV    200
NP_766415.1[M.musculus]               ----SAELIQPVATNGKVFPWAQIRLPTAIIPLCYELSLHPNLT--SMTFRGSVTISLQA    200
Q9UIQ6[H.sapiens]                     ----SIGLIQPFATNGKLFPWAQIRLPTAVVPLRYELSLHPNLT--SMTFRGSVTISVQA    200
XP_024102718.1[P.abelii]              ----SIGLIQPFATNGKLFPWAQIRLPTAIVPLHYELNLHPNLT--SMTFRGSVTISVQA    200
NP_731786.1[D.melanogaster]           T---TNPIYPTLPTGLPDPEKIEWRLPTELTPIKYKVYYHPDLT--TGACEGTVSIQFQL    168
XP_003386159.1[A.queenslandica]       -------SGNGSPGGSDDKPWTNVRLPKTLTPALYRVSLDTDLN--TFRVNGFVSVDISV    137
XP_019622707.1[B.belcheri]            TSEKKSAKKDPIATNGERFPWDDVRLPSTIVPNSYLLHLHPNLT--TFNFTGHVSINISV    187
XP_012561211.1[H.vulgaris]            -------NKLKNSKREELFPYSNIRLPLNVIPERYKIYLHPNITDNKFGFTGTVRILINI    109
XP_032239104.1[N.vectensis]           EPEKKDDSSFYEDGHGRKFAWQKIRLPSSVTPEEYTVILRPKL-DPDFTFSGNVSVRVKC    166
                                                            . ***  : *  * :    .:        * . : .

XP_032824577.1[P.marinus]             DESTDHVVLHANNLTITESILVPADGA------PAGGGADGSLVAKLLRYEPRDQLMVCT    335
XP_014016586.1[S.salar]               LHNTKHVVLHSSELIITKATFQVGEG-------------K-SVEVKVLEYKPWQQIAVSF    251
NP_001088429.1[X.laevis]              TTSSKNIVLHSSGLHIIKASVLVNGG-------------T-TMDAEVLEYPPFEEIAIIS    245
XP_424862.3[G.gallus]                 TQVTKKIVLHSSGLNITKATITSTGG-------------SQEKAVELLEYPLHDQIAVMA    247
NP_766415.1[M.musculus]               LQDTRDIILHSTGHNISRVTFMSAVS-------------SQEKQVEILEYPYHEQIAVVA    247
Q9UIQ6[H.sapiens]                     LQVTWNIILHSTGHNISRVTFMSAVS-------------SQEKQAEILEYAYHGQIAIVA    247
XP_024102718.1[P.abelii]              LQVTWNIILHSTGHNISRVTFMSAVS-------------SQEKQVEILEYAYHGQIAIVA    247
NP_731786.1[D.melanogaster]           NAITNLIVLHAKELNVHSISILNMMAR------------IRVAIDSINLDESRELLLITL    216
XP_003386159.1[A.queenslandica]       NQSTDLVIFHAKDMTLNTVSLTKGVRG------------DQLGISRQFFYSDNDFYVIQL    185
XP_019622707.1[B.belcheri]            VKKTDLVVFHVKELNITQSSVKTMNNV-----------SVPIV--KELEYTGNEQYCLRL    234
XP_012561211.1[H.vulgaris]            TEETDSVLLHIKDLNISEVKCYHGSSAMSKHKGPEDSQQVPVKDHLISVEHEFLMIRMKE    169
XP_032239104.1[N.vectensis]           NEDTDYIFIHAKQMRLTKFEVLNQGKE-------------PLKIMETANCEKLEMFSIKV    213
                                         :  :.:* .   :                                         :

XP_032824577.1[P.marinus]             RKPLTQGQMYLLNITFTGRLSTSFLGFYRSTYRAEDG----SDRPLAVTQFEPTSARESF    391
XP_014016586.1[S.salar]               PEDLKVGQVCVLTLDYAASLSHTYDGFYNSSYNDKTG----AKRVLAATQFEPQAARKAF    307
NP_001088429.1[X.laevis]              PESLLKGKECLLTIQYAANFSSTYYGFYKIDYMDKSN----K-RSLAATQFEPLAARKAF    300
XP_424862.3[G.gallus]                 PESLLAGQNYTVNLEYSSNLSDTYYGFYRVSYKDENS----KQRWFAATQFEPLAARSAF    303
NP_766415.1[M.musculus]               PEPLLTGHNYTLKIEYSANISNSYYGFYGITYTDKSN----EKKYFAATQFEPLAARSAF    303
Q9UIQ6[H.sapiens]                     PEALLAGHNYTLKIEYSANISSSYYGFYGFSYTDESN----EKKYFAATQFEPLAARSAF    303
XP_024102718.1[P.abelii]              PEALLAGHNYTLKIEYSANMSRSYFGFYGFSYTDESN----EKKYFAATQFEPLAARSAF    303
NP_731786.1[D.melanogaster]           REVLSMNKAYTLSASFDCDLS-SLVGSYISNYTNADG----VDRSIISTKFEPTYARQAF    271
XP_003386159.1[A.queenslandica]       ADSLDTNDNLQLNISFNYTLRDDLVGFYKSSYSLADN----EVHYLATTQFEPTDARRAF    241
XP_019622707.1[B.belcheri]            GQGLSAGQNYTIVVHFSGELNDGLYGFYRSTYTLNKGKDGEESRVLATTQFEAANARAAF    294
XP_012561211.1[H.vulgaris]            QHELEVGKQYTLFIRFNGRLSNGLEGFYKSSYTTSKG----EKRYLATTHFEATQARAAF    225
XP_032239104.1[N.vectensis]           KGGLKKGESYVLQIDFNAVLAEKLTGFYKSSYKDKDG----NTRYLATTHFEPTDARAAF    269
                                         *  ..   :   :   :     * *   *    .      : :  *:**   ** :*

XP_032824577.1[P.marinus]             PCFDEPDFKASFDISIVRD--AAHTTLSNMPLNETVTR-KDGLLEDTFKG-SVVMSTYLV    447
XP_014016586.1[S.salar]               PCFDEPAFKATFLVRITRE--PGYITLSNMPQAKTTTL-PSGLLEDEFEQTAVNMSTYLV    364
NP_001088429.1[X.laevis]              PCFDEPAFKSTFQINIIRN-DESMISLSNMPKAKTSKT-SDGLLQDEYST-SVRMSTYLV    357
XP_424862.3[G.gallus]                 PCFDEPALKATFSIRIKRD--EKLSTLSNMPKKATTPV-TKGIVQDEFFV-SLKMSTYLV    359
NP_766415.1[M.musculus]               PCFDEPAFKATFIIKITRN--EHHTALSNMPKKSSVPA-EEGLIQDEFSE-SVKMSTYLV    359
Q9UIQ6[H.sapiens]                     PCFDEPAFKATFIIKIIRD--EQYTALSNMPKKSSVVL-DDGLVQDEFSE-SVKMSTYLV    359
XP_024102718.1[P.abelii]              PCFDEPAFKATFIIRIIRD--EQYTTLSNMPKKSSVIL-EDGLVQDEFYE-SVKMSTYLV    359
NP_731786.1[D.melanogaster]           PCFDEPALKAQFTITVARPSGDEYHVLSNMPVASEYVD--GDITEVTFAE-TVPMSTYLA    328
XP_003386159.1[A.queenslandica]       PCFDEPAMKANFSIELTHA--NRYNAVSNMPVARRVSK-ANDKATTSFNT-SYKMSTYLV    297
XP_019622707.1[B.belcheri]            PCFDEPALKATFQLHMVRE--GRHSTLFNMPKD-HIEDLLGGLKMDVFKK-SVAMSTYLV    350
XP_012561211.1[H.vulgaris]            PCFDEPALKALFEIIMVRE--PQHTALSNMPIVKTINR-TDGLKEDHFQQ-SLMMSTYLV    281
XP_032239104.1[N.vectensis]           PCFDEPALKAVFNMVIYRK--AEHVSLSNMPIKETFKDKESGQVIDVFEP-SVKMSTYLV    326
                                      ****** :*: * : : :        : ***          .     :   :  *****.
```

```
XP_032824577.1[P.marinus]           AFIVSDFKSISNKT-KHGTNVSIYTSPGKEQQGSFALENAIKFLDFYEELFNISYPLPKQ          506
XP_014016586.1[S.salar]             AFIVANFTSITRNV--SNTLVSVYSVPEKKEHTHYALDTASKLLHFYNTFFKINYPLRKL          422
NP_001088429.1[X.laevis]            AFIVGDIKNTTQKT--NDTLVSVYAVPDKTDQVKYALDSTVKLLDFYSNYYGIEYPLEKL          415
XP_424862.3[G.gallus]               AFVVADLKNISMET--NGSLVSVHAIPQHINQVEYALNTAVKLLEFYEKYFLINYPLEKL          417
NP_766415.1[M.musculus]             AFIVGEMRNLSQDV--NGTLVSVYAVPEKIGQVHHALDTTIKLLEFYQTYFEIQYPLKKL          417
Q9UIQ6[H.sapiens]                   AFIVGEMKNLSQDV--NGTLVSIYAVPEKIGQVHYALETTVKLLEFFQNYFEIQYPLKKL          417
XP_024102718.1[P.abelii]            AFIVGEMKNLSQDI--NGTLVSIYAVPEKIGQVRYALETTVKLLEFYQNYFEIQYPLKKL          417
NP_731786.1[D.melanogaster]         AFVVSDFQYKETTVEGTSIALKVYAPPAQVEKTQYALDTAAGVMAYYINYFNVSYALPKL          388
XP_003386159.1[A.queenslandica]     AFVISDFNCSDSQTVNGHIQVRVCARPDVFSDTSYALSVGKSVIGYYEEFFGVQYPLPKQ          357
XP_019622707.1[B.belcheri]          CFIVSDFRSMQLSTDTSDITVAVHAPEDRISQASFALDVAKTVLEYYETFFGVKYPLPKM          410
XP_012561211.1[H.vulgaris]          AFVVCDYGYKSAKT-SRGIEVKVWAPKEQIEQANFAIYAAPKVLDYYETFFQVNFPLPKQ          340
XP_032239104.1[N.vectensis]         AFVVCDFKSKEATT-KRGTLVRVWAPEDNIDEGDYALSEAVKILSYYEKFFAVRYPLPKQ          385
                                    .*:: :              : : :      .  .*:     .: ::   : : : * *

XP_032824577.1[P.marinus]           DLVAIPDFAAGAMENWGLVTYRETALLLGPKASGVRDTLWVAQVIAHELAHQWFGNLVTM          566
XP_014016586.1[S.salar]             DLVAIPDFLAGAMENWGLITFRETSLLVGNQ-SSPLDKQLIANVVAHELAHQWFGNLVTM          481
NP_001088429.1[X.laevis]            DLVAIPDFQAGAMENWGLITFRETTLLYKENSSSIEDKQSITTVIAHELAHQWFGNLVTM          475
XP_424862.3[G.gallus]               DLVAIPDFQSGAMENWGLITFRETTLLFDNNASSARDKKLITAVIAHELAHQWFGNLVTM          477
NP_766415.1[M.musculus]             DLVAIPDFEAGAMENWGLLTFREETLLYDNATSSVADRKLVTKIIAHELAHQWFGNLVTM          477
Q9UIQ6[H.sapiens]                   DLVAIPDFEAGAMENWGLLTFREETLLYDSNTSSMADRKLVTKIIAHELAHQWFGNLVTM          477
XP_024102718.1[P.abelii]            DLVAIPDFEAGAMENWGLLTFREETLLYDNNTSSVADRKLVTKIIAHELAHQWFGNLVTM          477
NP_731786.1[D.melanogaster]         DLVAIPDFVSGAMENWGLVTFRETALLYDESTSSSVNKQRVAIVVAHELAHQWFGNLVTM          448
XP_003386159.1[A.queenslandica]     DLFAIPDFAVGAMENWGLITYRETALLYNSTQNPAVNKQRVAVVVAHELAHQWFGNLVTM          417
XP_019622707.1[B.belcheri]          DLVAIPDFSSGAMENWGLVTYRETAILYDADNSSAGDKQWVAVVIAHEFAHQWFGNLVTM          470
XP_012561211.1[H.vulgaris]          DLIAIPDFAAGAMENWGLITYRLTSILYDEKESSSANKQWVAVVIAHELAHQWFGNLVTM          400
XP_032239104.1[N.vectensis]         DLIAIPDFAAGAMENWGLITYRLTSLLYDPEVSSDSNKQWVAVVVAHELAHQWFGNLVTM          445
                                    **.*****  ********:*:*  ::*     .   :   :: ::***:***********

XP_032824577.1[P.marinus]           NWWNDLWLNEGFATFMEFVATGSLKPDWLMMDVFLTEVVMEALTQDSLPSSHAISS--AA          624
XP_014016586.1[S.salar]             RWWNDLWLNEGFATYWQYMSLMTEFPQLDLGNVFLG-VRFKAMAKDSLNSSHPVSVSSQV          540
NP_001088429.1[X.laevis]            EWWNDLWLNEGFATYMEYFSVSSVFPELNSDNSFLK-MRFMALQKDSLNSSHPIST--DV          532
XP_424862.3[G.gallus]               EWWNDLWLNEGFATFMEYFAMEEIFPELHSDEDFLN-LIFKAMMKDSLNSSHPVSS--AV          534
NP_766415.1[M.musculus]             QWWNDLWLNEGFATFMEYFSVEKIFKELNSYEDFLD-ARFKTMRKDSLNSSHPISS--SV          534
Q9UIQ6[H.sapiens]                   KWWNDLWLNEGFATFMEYFSLEKIFKELSSYEDFLD-ARFKTMKKDSLNSSHPISS--SV          534
XP_024102718.1[P.abelii]            KWWNDLWLNEGFATFMEYFSLEKIFKELSSYEDFLD-ARFKTMKKDSLNSSHPISS--SV          534
NP_731786.1[D.melanogaster]         NWWNDLWLNEGFASFLEYKGVKQMHPEWDMDNQFVIEELHPVLTIDATLASHPIVK--SI          506
XP_003386159.1[A.queenslandica]     SWWDGLWLNEGFASYVEYIGTDHVQPDWMMLEQFFIDTVQTAYDADGLNWSHPIIQ--QV          475
XP_019622707.1[B.belcheri]          KWWNDLWLNEGFASFVEFIGADKVSPEWKMLDQFVVSDLQTALSADSLSNSHPISV--TV          528
XP_012561211.1[H.vulgaris]          KWWNDLWLNEGFAAFVEFIGANITEPSWQMMDQFIVDDTQNSLTLDSSSNSHPISV--TV          458
XP_032239104.1[N.vectensis]         KWWNDLWLNEGFASFVENIGVNHTTPEWRMMEQFLLDKTQLSMNLDQLSNSHPISV--VV          503
                                     **:.********:: :  .      .    : *.          *     ** :

XP_032824577.1[P.marinus]           VGTMQIGEMFDSVSYNKGASVLNMLRTFLTPAVFVDGIRDYLLVHSYGNAHTDDLWESLT          684
XP_014016586.1[S.salar]             TTPEQVSEMFDSVTYEKGASLLLMLNTTLPDGQFKEGLMKYLDTYRGSNTVTEDLWNSLT          600
NP_001088429.1[X.laevis]            RSPEQIEEMFDDLSYIKGASILLMLKSLLLEDVFHLCIRGYLKSHEYGSTTSDSLWDSLN          592
XP_424862.3[G.gallus]               QSSEQIEEMFDALSYIKGASLLMMLKHYLTKDVFQAGIEIYLHNHKYGSAQSDDLWDSMN          594
NP_766415.1[M.musculus]             QSSEQIEEMFDSLSYFKGASLLLMLKSYLSEDVFRHAVILYLHNHSYAAIQSDDLWDSFN          594
Q9UIQ6[H.sapiens]                   QSSEQIEEMFDSLSYFKGSSLLLMLKTYLSEDVFQHAVVLYLHNHSYASIQSDDLWDSFN          594
XP_024102718.1[P.abelii]            QSSVQIEEMFDSLSYFKGASLLLMLKTYLSEDVFQHAVILYLHNHSYASIQSDDLWDSFN          594
NP_731786.1[D.melanogaster]         ESPAEITEYFDTITYSKGAALVRMLENLVGEEKLRNATTRYLVRHIYSTATTEDYLTAVE          566
XP_003386159.1[A.queenslandica]     NNPDEINGLFDSISYDKGASLIQMLRGYIGNESFTNGLTLYLKNNKFGNTETYELWDALN          535
XP_019622707.1[B.belcheri]          HRPEEVEEIFDSISYDKGASILRMLESFLGRETFLQGLTSYLREHQFKNAATDDLWKALD          588
XP_012561211.1[H.vulgaris]          NDPAQINEIFDTISYDKGASIIRMMKNFLGSDVFHTGLTDYLNKYKFKNAVSDDLWACLT          518
XP_032239104.1[N.vectensis]         KDPAEINSLFDTISYDKGAAIIRMLKSFLGDDVFQKGLQKYLNKHKFGNAETNQLWDAFT          563
                                        ::   ** ::* **:::: *:.  :    :      **         : .   ..

XP_032824577.1[P.marinus]           QSAQQ---HLGHSVNVTEVMGTWVRQRGFPLLTLTAVDK---ELKITYKQEPFPIEPA--          736
XP_014016586.1[S.salar]             QA--Q---VLPQQENVSDMMRTWTLQKGFPLVTVSRKGG---HVTL--TQEHFLLSAD--          648
NP_001088429.1[X.laevis]            V-------VTGGNPNVKNMMKTWTQKAGYPLVTALRKGE---EITV--RQERFLRSSD--          638
XP_424862.3[G.gallus]               E-------ITNGTLDVKKLMKTWILHKGFPLVTVNRKGK---IISL--HQEKFLYSVEP-          641
NP_766415.1[M.musculus]             E-------VTDKTLDVKKMMKTWTLQKGFPLVTVQRKGT---ELLL--QQERFFLRMQP-          641
Q9UIQ6[H.sapiens]                   E-------VTNQTLDVKRMMKTWTLQKGFPLVTVQKKGK---ELFI--QQERFFLNMKP-          641
XP_024102718.1[P.abelii]            E-------VTNQTLDVKRMMKTWTLQKGFPLVTVQKKGK---ELFI--QQERFFLNMKP-          641
NP_731786.1[D.melanogaster]         EEE-------GLEFDVKQIMQTWTEQMGLPVVEVEKSGS---TYKL--TQKRFLANEDDY          614
XP_003386159.1[A.queenslandica]     EVS-------SSDVSVSQMMDTWTKQMGYPVVTVSASDN--NRATV--SQKRFFQIPLP-          583
XP_019622707.1[B.belcheri]          QAAHGA---GKSNINIKQVMDRWTNQMGYPVVMVTRQNS---HLVL--TQDRFLQMPPSP          640
XP_012561211.1[H.vulgaris]          KAA-------NNTIDVKSVMDTWTLQMGYPLITITKNHEQSEKGLV--TQEHFLIDVDR-          568
XP_032239104.1[N.vectensis]         EESSVSASSTKNFRDVKSVMDTWTLQMGFPVVTIKQRGD---SAVA--SQKHFRIHPKV-          617
                                                  .:. :*   *  : * *::                 *. *

XP_032824577.1[P.marinus]           -------ETSITPSLWQIPLTFITSDNSTS-----QSK-LITDKQGDIA--------VTK          775
XP_014016586.1[S.salar]             -------NTTHTSSLWHIPVTYVNDSCSS-APSCRQIF-MLRNKTATLN--------VSD          691
NP_001088429.1[X.laevis]            -------HATNDSTVWHIPLTYVTGKCDAVEPDCEKIH-LLKEATGTIN--------VSS          682
XP_424862.3[G.gallus]               -----DNWTSDTSYLWDIPLTYTTNRCNFT--HCINAY-LLDQKSAVIE--------LPE          685
NP_766415.1[M.musculus]             -----ESQPSDTSHLWHIPISYVTDGRNYS--EYRSVS-LLDKKSDVIN--------LTE          685
Q9UIQ6[H.sapiens]                   -----EIQPSDTSYLWHIPLSYVTEGRNYS--KYQSVS-LLDKKSGVIN--------LTE          685
XP_024102718.1[P.abelii]            -----EIQPSDTSYLWHIPLSYVTEGRNYS--KYQSVS-LLDKKSGVIN--------LTE          685
NP_731786.1[D.melanogaster]         A---AEAEASSFNYRWSIPITYTSSINSEV-----QSLIFNHNDN-EATIT------LPE          659
XP_003386159.1[A.queenslandica]     --EGEQPAASPYNYVWIIPFDYITENGNSV-----TKKLVS-NQ-------QDTITWDSS          628
XP_019622707.1[B.belcheri]          ACGGVVEFTSPYRYEWIIPLTYITSTSPKE-----QQTVFMDTTQ-------TTLSLDNN          688
XP_012561211.1[H.vulgaris]          -----KTAASPFNYKWDVPITFYFEHKKEK-----QLV-WFNRSADSINIPM------MN          611
XP_032239104.1[N.vectensis]         ----KPSLRSQFDYKWIIPFTYYTQNDKTK-----KKA-WIEKDNGREGEGILQFDYNPA          667
                                             :     * :*. :

XP_032824577.1[P.marinus]           STEWIKANTNMTGFYVVDYGKDGWATFANKLT----TSKSFLSPADRANLLNDVFYLLNV          831
XP_014016586.1[S.salar]             SVKWLKLNYMNTGFYIVHYGDEGWTTLIDALK----TDINTLTAHDRASLIHNIFSLSRL          747
NP_001088429.1[X.laevis]            ELPWVKFNVNMTGYYIVDYGADGWDALIEQLH----RDYTVLHSSDRANLIHDIFMLAGV          738
XP_424862.3[G.gallus]               EVEWIKFNVDMNGYYIVNYD-EDWETLIDLLK----KNHTALSAKDRANLINNIFSLASL          740
NP_766415.1[M.musculus]             QVQWVKVNSNMTGYYIVHYAHDDWTALINQLK----RNPYVLSDKDRANLINNIFELAGL          741
Q9UIQ6[H.sapiens]                   EVLWVKVNINMNGYYIVHYADDDWEALIHQLK----INPYVLSDKDRANLINNIFELAGL          741
XP_024102718.1[P.abelii]            EVLWVKVNINMNGYYIVHYADDDWEALIHQLK----INPYVLSDKDRANLINNIFELAGL          741
NP_731786.1[D.melanogaster]         EASWIKINTNQVGYYRVNYGSEQWAELISALK----NSRETFSTADRAHLLNDANTLAAA          715
XP_003386159.1[A.queenslandica]     NDGFIKANANQTGFYRVNYDVGNWQSITAHLMTPPNNRPQILSAVDRAGLLEDAFSLSTS          688
XP_019622707.1[B.belcheri]          SPEWVKFNVNQTGFYRVNYDNQNWDAFIHQLQ----NNHEALNSADRAGLIDDAFHLNRA          744
XP_012561211.1[H.vulgaris]          ASGWIKANIDQLNFYRVNYDEDNWNLLSKQLQ----DNHKAFSTSDRSNLIDDAFELAKA          667
XP_032239104.1[N.vectensis]         TSGWIKANYEQHGFYRVNYDAENWERLKQQLD----TDHEKLSAADRAGLLDDAFNLARA          723
                                       ::* *    .:* *.*    *  :   *          :   **: *:.:   *

XP_032824577.1[P.marinus]           GRVDVEDAMPLLSFLPNETSVTPWKVALMHLDRVLLILSPYAHDV--QLLQKHIVKLGEE          889
XP_014016586.1[S.salar]             GRVSFRQVLNLLDYATNETETAPLTEALSQLSSVYRLLDKRQEQTLVARMKAYIWGHFGQ          807
NP_001088429.1[X.laevis]            GKVPLSKAFELLGYLVNETDSAPITQALHQFYHIHGILLKRGLDDLSDKLMEHGLELLNN          798
XP_424862.3[G.gallus]               GKEPLKKAFELIDYLKEESSTAPLSQALFQLGLIFGLLEKRGEQQLAARVMYRIECLLGN          800
NP_766415.1[M.musculus]             GKVPLRMAFDLIDYLKNETHTAPITEALFQTNLIYNLLEKLGHMDLSSRLVARVHKLLQN          801
Q9UIQ6[H.sapiens]                   GKVPLKRAFDLINYLGNENHTAPITEALFQTDLIYNLLEKLGYMDLASRLVTRVFKLLQN          801
XP_024102718.1[P.abelii]            GKVPLKRAFDLINYLGNENHTAPITEALFQTDLIYNLLEKLGYMDLASRLVTRVFKLLQN          801
NP_731786.1[D.melanogaster]         GQLNYSVALDLISYLESEQDYVPWSVGTSALATLRNRVYY--T-DLYTNYTTYARKLLTP          772
XP_003386159.1[A.queenslandica]     GLLNITVALNLSRYLVNEEDYAPWMTALRWFSIFSDKLST--N-GQYGNFKRYVSSLMGN          745
XP_019622707.1[B.belcheri]          GLFGLEKSLELVKYLKNERDYVPMATALSGLGYITKLLETEDQ-DLYNNMKLFVLELLED          803
XP_012561211.1[H.vulgaris]          GKLDQIKALEMTAYLKNEDEYVPWITALGSLGYIGGLL-Q-GR-SCYSSYQKYIIQQVKP          724
XP_032239104.1[N.vectensis]         GELPLTTALDLTKYLTKEEMYVPWAAALSNMGFLESRLCE-NE-EHMTLYKKYALQQLIP          781
                                    *       : :  :  .*   .*    .      .   :

XP_032824577.1[P.marinus]           LLPRLPWNKSSEEV-TVPQRSLDALLVSVLCAYRSEKCLNRANVAYLQWSASE---SL-S          944
```

```
XP_014016586.1[S.salar]              LMDSQDWG---EEE-SVSRQELRSALLEMACSLGRQNCTDQATALYDQWTNSNQTKQI-P      862
NP_001088429.1[X.laevis]             TLIKQTWM---DEG-TLAERQLRSSLLDFACSNGFPSCLEKATELFNSWRVNK--TRI-P      851
XP_424862.3[G.gallus]                KIDQQNWT---DDG-TISERELRSMLLTFACTHDIRNCRTAASKMFDEWMKSNGTMSL-P      855
NP_766415.1[M.musculus]              QIQQQTWT---DEG-TPSMRELRSALLEFACAHSLENCTTMATNLFDSWMASNGTQSL-P      856
Q9UIQ6[H.sapiens]                    QIQQQTWT---DEG-TPSMRELRSALLEFACTHNLGNCSTTAMKLFDDWMASNGTQSL-P      856
XP_024102718.1[P.abelii]             QIQQQTWT---NEG-TPSMRELRSALLEFACIHNLGNCSTTAMKLFDDWMASNGTQSL-P      856
NP_731786.1[D.melanogaster]          IVEKVTFT---VAA-DHLENRLRIKVLSSACSLGHESSLQQAVTLFNQWLASP--ETRPN      826
XP_003386159.1[A.queenslandica]      ITRKLSFN---KTGLSHLQILLRTYVLLSGYKYGDISIADTSLTMFRNWMTDG--ISV-P      799
XP_019622707.1[B.belcheri]           TIGEVGWE---DQG-DQLTKFLRSTVLGAACDYGHEQSTQQAHTLFANWMKGT--QNV-A      856
XP_012561211.1[H.vulgaris]           IVDKLGWS---DEG-THLNRYLRGAALRSSVMHNDTDSVKRALEIFDRFMNNH--ESV-A      777
XP_032239104.1[N.vectensis]          IVRKLGWD---DKG-SHLQKYLRSYVLKLCARYGDVECATAVKSRFADWM-RG--ESL-P      833
                                           :              *    :         .        :  :

XP_032824577.1[P.marinus]            ADLMSTVLRVAVRD-GDRPTWDSVQEHYHRAKSSTEQAKYLEALASTSDEDQQWSLLENA      1003
XP_014016586.1[S.salar]              GDLQRVVFSVAAQS---NLGWLSLMEAYSSTTYDSEKRKILQALASTQDPQSIVWILSTG      919
NP_001088429.1[X.laevis]             TDVMKVVFKVGART---TEGWTLLRSTYDSSIYEAEKRKILEALASTDNAKNLQWLMQES      908
XP_424862.3[G.gallus]                SDLMKAIFITGAKT---NDGWEFLLKMYSSPVPEAEKSKMIEALASTEDVRKMMWLMQNS      912
NP_766415.1[M.musculus]              TDVMVTVFKVGART---EKGWLFLFSMYSSMGSEAEKNKILEALASSEDVHKLYWLMKSS      913
Q9UIQ6[H.sapiens]                    TDVMTTVFKVGAKT---DKGWSFLLGKYISIGSEAEKNKILEALASSEDVRKLYWLMKSS      913
XP_024102718.1[P.abelii]             TDVMTTVFKVGAKT---DKGWSFLLGKYISIGSEAEKNKILEALASSEDVRKLYWLMKSS      913
NP_731786.1[D.melanogaster]          PDIRDVVYYYGLQQVNTEAAWDQVWKLYLDESDAQEKLKLMNCLTAVQVPWLLQRYINWA      886
XP_003386159.1[A.queenslandica]      PDLRLVVYRVAIAA-GGETEWNYLWSWYKNTTNPYEKQICLSALAQSKEYWILSRYLEYS      858
XP_019622707.1[B.belcheri]           ANLKTVVYTSGVQH-GGEEEWNFCWQQYTSATVAAEKRKLLYALANTRDNKLVTKLLRYT      915
XP_012561211.1[H.vulgaris]           PNLRSTVYLAGIKY-GGKEQWEFMLNKYLNSPFPSEQRKIMFALADSSDESILKKYLSWS      836
XP_032239104.1[N.vectensis]          PNLRSVIYDTGVHL-GGEKEFKYMYEQYNKSTVAAEKRKLLFAMSATQNPALMKGLLDMS      892
                                      ::  .:   .         :      *       *:   : .::           :

XP_032824577.1[P.marinus]            MAGVEVRSQELPRIVGVMARTSWTGRLITWDFLRKRWSLLVEKFSLGSFALSNMVMSVTS      1063
XP_014016586.1[S.salar]              LEGGIIQTQELPLVISTMSD-GFAGHLFVWDFVKENWDKIIEKFPVGSYPIQSIIKSTTS      978
NP_001088429.1[X.laevis]             LDGGSIRSQELPAVIGFICK-RSPGYLLAWNFIKQNWDLITQKFMPGSFPIQNIVSKTTH      967
XP_424862.3[G.gallus]                LEGEVIRTQELSHIIATISH-SLPGHLLAWDFVKENWEKLTRKFHLGSYTIQNIISSSTS      971
NP_766415.1[M.musculus]              LDGDIIRTQKLSLIIRTVGR-HFPGHLLAWDFVKENWNKLVHKFHLGSYTIQSIVAGSTH      972
Q9UIQ6[H.sapiens]                    LNGDNFRTQKLSFIIRTVGR-HFPGHLLAWDFVKENWNKLVQKFPLGSYTIQNIVAGSTY      972
XP_024102718.1[P.abelii]             LNGDNFRTQKLSFIIRTVSR-HFPGHLLAWDFVKENWNKLVQKFHLGSYTIQNIVAGSTY      972
NP_731786.1[D.melanogaster]          WDESNVRRQDYFTLLGYIST-NPVGQSLVWDYVRENWEKLVDRFGINERTLGRLIPTITA      945
XP_003386159.1[A.queenslandica]      MSQ--VRSQDTLYVIRSVAR-NVNGRYLAWNFVRDNYDTIFKKYGGGSFSFSRLIRSITG      915
XP_019622707.1[B.belcheri]           LDSTKIRSQDTVRTITYVSR-TAVGYQLAWNFVKSNWQTFLDRYGSGSFNMAELAKIPAH      974
XP_012561211.1[H.vulgaris]           MNTSIIRTQDTCGVIEHIST-NIKGTKMAEDFVIKNWEKLFERYGKGSFDMSSLIKTVFA      895
XP_032239104.1[N.vectensis]          MSTQ-IRSQDTVSVITSVAS-NCKGRNLAWDFVKKHWKTLFKRYGHGSFDFARLVSRTTT      950
                                          .: *.    :  :      *  :. ::: ..:. :  ::  ..  :  :

XP_032824577.1[P.marinus]            GFSSTYHLDQVEEFVASLRDSSGELRVFKEARDTIRANHHWLATNRVALHKWLTEQS---      1120
XP_014016586.1[S.salar]              QFSTQTHLEEVQGFFSSLKERGSQMRSVQEALETIRLNQLWMDRNLPTLRIWL-------      1031
NP_001088429.1[X.laevis]             QFSTDVHLNEVIAFFNSTQEKSREMWCVKEAVETIKLNIEWMKNNFDSLKTWLLPSQ---      1024
XP_424862.3[G.gallus]                QFATKVHLLEVKTFFESKSEESSKLRCVKEAIDTIQLNIQWMETNLAKLQEWL-------      1024
NP_766415.1[M.musculus]              LFSTKTHLSEVQAFFENQSEATLKLRCVQEALEVIQLNIQWMVRNLKTLSQWL-------      1025
Q9UIQ6[H.sapiens]                    LFSTKTHLSEVQAFFENQSEATFRLRCVQEALEVIQLNIQWMEKNLKSLTWWL-------      1025
XP_024102718.1[P.abelii]             LFSTKTHLSEVQAFFENQSEATFRLRCVQEALEVIQLNIQWMEKNLRSLTWWL-------      1025
NP_731786.1[D.melanogaster]          RFSTETKLEEMQQFFAKYPEAGAGTAARQQALEAVKANIKWLAANKAQVGEWLANYVQQS      1005
XP_003386159.1[A.queenslandica]      SFATSWELQEVESFFGKV-DVGSASLALQQSKEIVRGNIAWLDNNESVIGEWMNEYLSGA      974
XP_019622707.1[B.belcheri]           RFSTRRQLEEVKSFFQSH-DISAAARAARQTIETIQSNIFWLEKNKDKVKTWIKNYFMPP      1033
XP_012561211.1[H.vulgaris]           RMKTKEDLKKVSEIC---------------------------------------------      910
XP_032239104.1[N.vectensis]          HFSTPQKLKEIQEFFKKH-ELGSGKLASKQAEEGISSNIDWMKNNIEVALKWLEEHTQAE      1009
                                      : :  .* ::  :

XP_032824577.1[P.marinus]            ------------------------------------------------------------      1120
XP_014016586.1[S.salar]              ------------------------------------------------------------      1031
NP_001088429.1[X.laevis]             ------------------------------------------------------------      1024
XP_424862.3[G.gallus]                ------------------------------------------------------------      1024
NP_766415.1[M.musculus]              ------------------------------------------------------------      1025
Q9UIQ6[H.sapiens]                    ------------------------------------------------------------      1025
XP_024102718.1[P.abelii]             ------------------------------------------------------------      1025
NP_731786.1[D.melanogaster]          SVTNRIQ-----------------------------------------------------      1012
XP_003386159.1[A.queenslandica]      MSA---------------------------------------------------------      977
XP_019622707.1[B.belcheri]           ---EGGKARSSEI-----------------------------------------------      1043
XP_012561211.1[H.vulgaris]           ------------------------------------------------------------      910
XP_032239104.1[N.vectensis]          SKPADGDLGVANVERLENILAPEGFQEEYQDRENQEKPYQNKRYYSNQHYGRGDDQDRRF      1069


XP_032824577.1[P.marinus]            -------------------    1120
XP_014016586.1[S.salar]              -------------------    1031
NP_001088429.1[X.laevis]             -------------------    1024
XP_424862.3[G.gallus]                -------------------    1024
NP_766415.1[M.musculus]              -------------------    1025
Q9UIQ6[H.sapiens]                    -------------------    1025
XP_024102718.1[P.abelii]             -------------------    1025
NP_731786.1[D.melanogaster]          -------------------    1012
XP_003386159.1[A.queenslandica]      -------------------    977
XP_019622707.1[B.belcheri]           -------------------    1043
XP_012561211.1[H.vulgaris]           -------------------    910
XP_032239104.1[N.vectensis]          SNEYNDDDYEPERRGYEYQ    1088
```
