# Supplementary material for: An Evolutionary Perspective on the Origin, Conservation and Binding Partner Acquisition of Tankyrases
Source: Biomolecules. 2022 Nov 15;12(11):1688. doi: 10.3390/biom12111688 (PMC9688111; doi:10.3390/biom12111688)
Supplement: Supplementary file 1 [file biomolecules-12-01688-s001.zip › biomolecules-1913719-supplementary-11.17 -done/Data_7/Mcl-1.pdf]

Mcl-1

Homo sapiens:

>Q07820 MCL1_HUMAN Induced myeloid leukemia cell differentiation protein Mcl-1 OS=Homo sapiens OX=9606 GN=MCL1 PE=1 SV=3
MFGLKRNAVIGLNLYCGGAGLGAGSGGATRPGGRLLATEKEASARREIGGGEAGAVIGGS
AGASPPSTLTPDSRRVARPPPIGAEVPDVTATPARLLFFAPTRRAAPLEEMEAPAADAIM
SPEEELDGYEPEPLGKRPAVLPLLELVGESGNNTSTDGSLPSTPPPAEEEEDELYRQSLE
IISRYLREQATGAKDTKPMGRSGATSRKALETLRRVGDGVQRNHETAFQGMLRKLDIKNE
DDVKSLSRVMIHVFSDGVTNWGRIVTLISFGAFVAKHLKTINQESCIEPLAESITDVLVR
TKRDWLVKQRGWDGFVEFFHVEDLEGGIRNVLLAFAGVAGVGAGLAYLIR

Pongo abelii:

>XP_002810320.1 induced myeloid leukemia cell differentiation protein Mcl-1 isoform X1 [Pongo abelii]
MFGLKRNAVIGLNLYCGGAGLGAGSGGATPPGGRLLATEKEASARREIGGGEAGAVIGGSAGASPPSTLT
PDSRRVARPPPIGAEVPDVTATPARLFFFAPTRRAAPLEEMEAPAADAIMSPEEELDGYEPEPLGKRPAV
LPLLELVGESGNNTSTDGSLPSTPPPAEEEEDELYRQSLEIISRYLREQATGAKDTKPLGRSGATCRKAL
ETLRRVGDGVQRNHETAFQGMLRKLDIKNEDDVKSLSRVMVHVFSDGVTNWGRIVTLISFGAFVAKHLKT
INQESCIEPLAESITDVLVRTKRDWLVKQRGWDGFVEFFHVEDLEGGIRNVLLAFAGVAGVGAGLAYLIR

Mus musculus:

>NP_032588.1 induced myeloid leukemia cell differentiation protein Mcl-1 homolog [Mus musculus]
MFGLRRNAVIGLNLYCGGASLGAGGGSPAGARLVAEEAKARREGGGEAALLPGARVVARPPPVGAEDPDV
TASAERRLHKSPGLLAVPPEEMAASAAAAIVSPEEELDGCEPEAIGKRPAVLPLLERVSEAAKSSGADGS
LPSTPPPPEEEEDDLYRQSLEIISRYLREQATGSKDSKPLGEAGAAGRRALETLRRVGDGVQRNHETAFQ
GMLRKLDIKNEGDVKSFSRVMVHVFKDGVTNWGRIVTLISFGAFVAKHLKSVNQESFIEPLAETITDVLV
RTKRDWLVKQRGWDGFVEFFHVQDLEGGIRNVLLAFAGVAGVGAGLAYLIR

Gallus gallus:

>NP_001244212.2 induced myeloid leukemia cell differentiation protein Mcl-1 isoform 1 [Gallus gallus]
MFAVKRNAVIGFNLYCGGGSPGLVPASPAGEQTPPPAAAAPAAAAATVAEVPRPLIGSAGLWAAAGRAEA
PRAPIGSGAAPHAPIGSAAARRAPPDSTSRPVALWSPEEELDGCEPESERGPGGDSLPGTPPELPDLIPD
ELRQESLELILRYLREAAGEAEPGVKKLFPGLLGGPGRPCRASSAVMEKALETLRRVGDGVMQKHELAFQ
GMLRKLEIKKEDDLQAVCEVAAHVFNDGVTNWGRVVTLISFGAFVAKHLKSINQEKCITSLAGIITDALV
SSKREWLMSQGGWEGFVDFFRVEDLESSIRNVLMAFAGVAGLGASLAYMIRKWRS

Xenopus laevis:

>NP_001131055.1 MCL1, BCL2 family apoptosis regulator L homeolog [Xenopus laevis]
MMHQSVIAKQRPSTSFLIPCQFYCSGGGSSEKTLSARGASPWDPDMDTHRPQLNGLGFNNGGSLPCSQED
ELDEDMDNGSQGSTSPPDSPVCPKDGLYMDTQQLILAFYRVYSGEESGELEASCLLQHGVHHKALETLLR
VGGEIIEKHHMAFTGMLQRLSIHSREDLQKLSEVPALVFNDGVTNWGRIVTVISFGAFVAKHLKSLNLED
CIGVLAEHFTQFLMMSKKDWIIQEKGWDGFVDFFHIEDYESGLKTVLMAFSSVAVLGAGLAYMIR

Salmo salar:

>NP_001117034.1 myeloid leukemia differentiation protein homologue [Salmo salar]
MSLSNSITRATTTMLHFQNGGSSYLADDARPLYYFQGAGAICAGASPKSKVDLGNGTGDTPPRPTTLGVN
VVKSNGLDNHLSDRSNNDDSLPCTPQMASECGPELSNCPSGDEVLEHDTRQLIENFLGDYTGLSQPRWTQ
SKPLTTMKRVVEDVIAKHRYAYNGMVAKLDLDDRCDDMGVINSVAKTMFSDGITNWGRIASLVAFGAVVS
QHLKERGRGHCVELVGQEIAKYLLSDQSDWLIKNNAWNGFVEFFHVQDPESSVRNTLLAFAGVAGIGATL
AMFIR

Petromyzon marinus:

>XP_032829936.1 anti-apoptotic protein NR13-like [Petromyzon marinus]
MVADGTHAGIFAADRLQQQGAGSPPNSPSPRDNDTATAPAIGEACGAPPPTTPATSADSVSHSAADIQAT
RSGGSRPYNDDALTVETANFITGYMAHLTPTEQPPHQQQLSSASELLKSMGDSLLEQHRAAISGMVSRVE
AQTDPEELRGSALGVAREMFSDGHMNWGRVVALVVFCALLVKRLKASGRLSDAEARAVADEVAAFLVSSR
KAWFVQQGGWNGFVREFERETPDSALKNTLMAVAGFGAMTALAALGVRKILSGFC

Branchiostoma belcheri:

>XP_019634801.1 PREDICTED: bcl-2-related ovarian killer protein homolog B-like [Branchiostoma belcheri]
MDLTRRESAVEQPLPSLDEEHEHDSDGEGPESVDSRREEETGGVPARVWTFLREFSVRDWLRNAKDAALD
LRQYVLDVPETIKGSLWSQAAPTEKSVIDESRQLIRDYINGRLKKSNLTRGRLPDAKRRPTEVSSQLQKM
GGELERMYPHLYRDVSRQINITLSSESILETSFETVANELFSTGITWARIVAMFAVAGAFAVDCVHQGHP
MFTRRLVDVVVDFTDRKLSAWLVQEGGWYGLVKHFRGDGRTHMFWAISGIGAAIGLAATFFVIDP

Drosophila melanogaster:

No homolog found.

Hydra vulgaris:

>XP_001626412.1 apoptosis regulator Bcl-2 [Nematostella vectensis]
MPPIEGYDNANAVAARKMKTPEEKIAHELAKDFIGYKLGKGTRLNTKSALILRKLSNQIENKHELFLRNM
CDRLDIRQHNAPETFKQVADEIFGDGINWGRIVVLYTFAGKVAKYCQENQLDNAENVATWVGNYVASKSD
WVKKAGGWEAFNEQFKDVQEEHEKFWWNSLLCTTLGLGSLAAVLYMKS

Nematostella vectensis:

>XP_047134887.1 apoptosis regulator BAX [Hydra vulgaris]
MGPPAQPKPTIENNQPLNEEAPSLDTVDGRPQRFNNGGTRLRNQPRNVVVNVNNSSNQVIESTPELFTQF
LVNRMENDNIEVPEELRNRSRNGTVNKEVAHCLKRIGDDLVNNHQLNHLISSIQVTRETAYKTFFDVASH
VFADGTINWGRVVTLFYFAYKLAIQVVNQLPLVEIVIGWVQKFVTDRLAQWIAERGGWNAVQEYFGSTTV
QFVGVFAAGFLFAYILTKVFRR

Amphimedon queenslandica:

>XP_003386733.1 PREDICTED: bcl-2-like protein 1 [Amphimedon queenslandica]

MALVVNPAPVQHQYHHKNSPKRRGSPVQDDKFYEETRNLITACIESASGKEPLSVKLSEEALRIVGMVQQ
TLKADEIFFRKLCEELELTATNLFSKLMDVWGGMFSDGQVNIGRLLALLAFCQCVTVYCRSVGLPSIESS
VPHWASIFISTTHLKDWITNRGGWDAISKELMLRTSATNSSTSSRTASNGGWLQWGVSGIALAISVFDAL
HNSS

Alignment

```
XP_019634801.1[B.belcheri]           ------------------------------------------------------------           0
XP_003386733.1[A.queenslandica]      ------------------------------------------------------------           0
XP_047134887.1[H.vulgaris]           ------------------------------------------------------------           0
XP_001626412.1[N.vectensis]          ------------------------------------------------------------           0
XP_032829936.1[P.marinus]            ------------------------------------------------------------           0
NP_001117034.1[S.salar]              MSL-----SNSITRATTTMLHFQNGGSS--------------------------------           23
NP_001131055.1[X.laevis]             MMHQSVIAKQRPSTSFLIPCQFYCSGGGSSEKT---------------------------           33
NP_001244212.2[G.gallus]             --------MFAVKRNAVIGFNLYCGGGSPGLVPAS--PAGEQTPPPAAAAPAAAAATVAE           50
NP_032588.1[M.musculus]              --------MFGLRRNAVIGLNLYCGGASLGAGGGS--PAGARLVAEEA--KARR-EGGGE           47
Q07820[H.sapiens]                    --------MFGLKRNAVIGLNLYCGGAGLGAGSGGATRPGGRLLATEKEASARREIGGGE           52
XP_002810320.1[P.abelii]             --------MFGLKRNAVIGLNLYCGGAGLGAGSGGATPPGGRLLATEKEASARREIGGGE           52

XP_019634801.1[B.belcheri]           ----------------------------------------------------------MD           2
XP_003386733.1[A.queenslandica]      ------------------------------------------------------------           0
XP_047134887.1[H.vulgaris]           ------------------------------------------------------------           0
XP_001626412.1[N.vectensis]          ------------------------------------------------------------           0
XP_032829936.1[P.marinus]            ----------------------------------------------------------MV           2
NP_001117034.1[S.salar]              ------------------------------------YLADDARPLYYFQGAGAI------           41
NP_001131055.1[X.laevis]             -----------------------------------------------------------L           34
NP_001244212.2[G.gallus]             VPRPLIGSAGLW-----AAAGRAEAPRAPIGSGAA-----PHAPI-GSAAARRAPPDS--           97
NP_032588.1[M.musculus]              --------------AALLPGARVVARPPPVGAEDPDVTASAERRLHKSPGLLAVPPEEMA           93
Q07820[H.sapiens]                    AGAVIGGSAGASPPSTLTPDSRRVARPPPIGAEVPDVTATPARLLFFAPTRRAAPLEEME           112
XP_002810320.1[P.abelii]             AGAVIGGSAGASPPSTLTPDSRRVARPPPIGAEVPDVTATPARLFFFAPTRRAAPLEEME           112

XP_019634801.1[B.belcheri]           LT------------------------------RRESAVEQPLPSLDEEHEHDSDGEGPES           32
XP_003386733.1[A.queenslandica]      ------------------------------------------------------------           0
XP_047134887.1[H.vulgaris]           ---------------------MGPPAQPKPTIENNQPLNEEAPSLDTVDGRPQRF-----           34
XP_001626412.1[N.vectensis]          ------------------------------------------------------------           0
XP_032829936.1[P.marinus]            ADGTHAGIFAADRLQQQGA--GSPPNSPSPR-DNDTA---TAPAIGE-------------           43
NP_001117034.1[S.salar]              -----CAGASPKSKVDLGNGTGDTPPRPTTLGVN------VVKSNGLDNHLSDRS-----           85
NP_001131055.1[X.laevis]             SARG-ASPWDPDMDTH--------RPQLNGLGFNNGG---SLPCSQ-EDELDEDM-----           76
NP_001244212.2[G.gallus]             TSRP-VALWSPEEELD--------GCEPESERG---------------------------           121
NP_032588.1[M.musculus]              ASAA-AAIVSPEEELD--------GCEPEAIGKR-PA---VLPLLERVSEAAKSS-----           135
Q07820[H.sapiens]                    APAA-DAIMSPEEELD--------GYEPEPLGKR-PA---VLPLLELVGESGNNT-----           154
XP_002810320.1[P.abelii]             APAA-DAIMSPEEELD--------GYEPEPLGKR-PA---VLPLLELVGESGNNT-----           154

XP_019634801.1[B.belcheri]           VDSRREEETGGVPARVWTFLREFSVRDWLRNAKDAALDLRQYVLDVPETI-------K-G           84
XP_003386733.1[A.queenslandica]      -----------MA-------------------------LVVNPAPVQHQY-----HHKNS           19
XP_047134887.1[H.vulgaris]           -------NNGGTR-------------------------LRNQPRNVVVNVNN--------           54
XP_001626412.1[N.vectensis]          -----------------------------------------MPPIEG-------------           6
XP_032829936.1[P.marinus]            -------ACG-AP-------------------------PPTTPATSADSVSHSAADIQAT           70
NP_001117034.1[S.salar]              -------NND-DS-------------------------LPCTPQMASEC----------G           102
NP_001131055.1[X.laevis]             -------DNG-SQ-------------------------GSTSPPDSPVC----------P           93
NP_001244212.2[G.gallus]             -------PGG-DS-------------------------LPGTPPELPDL----------I           138
NP_032588.1[M.musculus]              -------GAD-GS-------------------------LPSTPPP-PEE----------E           151
Q07820[H.sapiens]                    -------STD-GS-------------------------LPSTPPP-AEE----------E           170
XP_002810320.1[P.abelii]             -------STD-GS-------------------------LPSTPPP-AEE----------E           170

XP_019634801.1[B.belcheri]           SLWSQAAPTEKSVIDESRQLIRDYINGRLKKSNLTRGR------------LPDAKRRPTE           132
XP_003386733.1[A.queenslandica]      PKRRGSPVQDDKFYEETRNLITACIESASGKEPLSV--------------------KLSE           59
XP_047134887.1[H.vulgaris]           --------SSNQVIESTPELFTQFLVNRMENDNIEVPEE--------LRNRSRNGTVNKE           98
XP_001626412.1[N.vectensis]          -------------YDNANAVAARKMKTPEEKIAHELAKDFIGY------KLGKGTRLNTK           47
XP_032829936.1[P.marinus]            RSGGSRPYNDDALTVETANFITGYMAHLTPTEQPPH------------------QQQLSS           112
NP_001117034.1[S.salar]              PELSNCPSGDEVLEHDTRQLIENFLGDYTGLSQP--------------------RWTQSK           142
NP_001131055.1[X.laevis]             K---------DGLYMDTQQLILAFYRVYSGEESGEL-----------EASCLLQHGVHHK           133
NP_001244212.2[G.gallus]             P---------DELRQESLELILRYLREAAGEAEPGVKKLFPGLLGGPGRPCRASSAVMEK           189
NP_032588.1[M.musculus]              E---------DDLYRQSLEIISRYLREQATGSKD-------------SKPLGEAGAAGRR           189
Q07820[H.sapiens]                    E---------DELYRQSLEIISRYLREQATGAKD-------------TKPMGRSGATSRK           208
XP_002810320.1[P.abelii]             E---------DELYRQSLEIISRYLREQATGAKD-------------TKPLGRSGATCRK           208
                                                    .:  .

XP_019634801.1[B.belcheri]           VSSQLQKMGGELERMYPHLYRDVSRQINITLSSESILETSFETVANELFSTGI-TWARIV           191
XP_003386733.1[A.queenslandica]      EALRIVGMVQQTLKADEIFFRKLCEELELTAT--N-LFSKLMDVWGGMFSDGQVNIGRLL           116
XP_047134887.1[H.vulgaris]           VAHCLKRIGDDLVNNHQLN--HLISSIQVTRE--T-AYKTFFDVASHVFADGTINWGRVV           153
XP_001626412.1[N.vectensis]          SALILRKLSNQIENKHELFLRNMCDRLDIRQH-N--APETFKQVADEIFGDGI-NWGRIV           103
XP_032829936.1[P.marinus]            ASELLKSMGDSLLEQHRAAISGMVSRVEAQTDPEE-LRGSALGVAREMFSDGHMNWGRVV           171
NP_001117034.1[S.salar]              PLTTMKRVVEDVIAKHRYAYNGMVAKLDLDDRCDD-MG-VINSVAKTMFSDGITNWGRIA           200
NP_001131055.1[X.laevis]             ALETLLRVGGEIIEKHHMAFTGMLQRLSIHSR-ED-LQ-KLSEVPALVFNDGVTNWGRIV           190
NP_001244212.2[G.gallus]             ALETLRRVGDGVMQKHELAFQGMLRKLEIKKE-DD-LQ-AVCEVAAHVFNDGVTNWGRVV           246
NP_032588.1[M.musculus]              ALETLRRVGDGVQRNHETAFQGMLRKLDIKNE-GD-VK-SFSRVMVHVFKDGVTNWGRIV           246
Q07820[H.sapiens]                    ALETLRRVGDGVQRNHETAFQGMLRKLDIKNE-DD-VK-SLSRVMIHVFSDGVTNWGRIV           265
XP_002810320.1[P.abelii]             ALETLRRVGDGVQRNHETAFQGMLRKLDIKNE-DD-VK-SLSRVMVHVFSDGVTNWGRIV           265
                                         :  :              :   :.               *   :*  *  . .*:

XP_019634801.1[B.belcheri]           AMFAVAGAFAVDCVHQGHPMF--TRRLVDVVVDFTD-RKLSAWLVQEGGWYGLVKHFRGD           248
XP_003386733.1[A.queenslandica]      ALLAFCQCVTVYCRSVGLPSI--ESSVPHWASIFISTTHLKDWITNRGGWDAISKELMLR           174
XP_047134887.1[H.vulgaris]           TLFYFAYKLAIQV--VNQLPL--VEIVIGWVQKFVT-DRLAQWIAERGGWNAVQEYFGST           208
XP_001626412.1[N.vectensis]          VLYTFAGKVAKYCQENQLDNA---ENVATW-VGNYV-ASKSDWVKKAGGWEAFNEQFKDV           158
XP_032829936.1[P.marinus]            ALVVFCALLVKRLKASGRLSDAEARAVADEVAAFLV-SSRKAWFVQQGGWNGFVREFERE           230
NP_001117034.1[S.salar]              SLVAFGAVVSQHLKERGRGHC--VELVGQEIAKYLL-SDQSDWLIKNNAWNGFVEFFHVQ           257
NP_001131055.1[X.laevis]             TVISFGAFVAKHLKSLNLEDC--IGVLAEHFTQFLM-MSKKDWIIQEKGWDGFVDFFHIE           247
NP_001244212.2[G.gallus]             TLISFGAFVAKHLKSINQEKC--ITSLAGIITDALV-SSKREWLMSQGGWEGFVDFFRVE           303
NP_032588.1[M.musculus]              TLISFGAFVAKHLKSVNQESF--IEPLAETITDVLV-RTKRDWLVKQRGWDGFVEFFHVQ           303
Q07820[H.sapiens]                    TLISFGAFVAKHLKTINQESC--IEPLAESITDVLV-RTKRDWLVKQRGWDGFVEFFHVE           322
XP_002810320.1[P.abelii]             TLISFGAFVAKHLKTINQESC--IEPLAESITDVLV-RTKRDWLVKQRGWDGFVEFFHVE           322
                                      :  .   .                 :               *. .  .* ..   :

XP_019634801.1[B.belcheri]           GRTHM---------FWAISGIGAAIGLAATFFVIDP-----      275
XP_003386733.1[A.queenslandica]      TSATNSSTSSRTASNGGWLQWGVS-GIALAISVFDALHNSS      214
XP_047134887.1[H.vulgaris]           T--------------VQFVGVFAA-GFLFAYILTKVFRR--      232
XP_001626412.1[N.vectensis]          QEEHEKFW--WNSLLCTTLGLGSL-AAVLYM---KS-----      188
XP_032829936.1[P.marinus]            TPDSA--L--KNTL-MAVAGFGAM-TALAALGVRKILSGFC      265
NP_001117034.1[S.salar]              DPESS--V--RNTL-LAFAGVAGI-GATLAMFIR-------      285
NP_001131055.1[X.laevis]             DYESG--L--KTVL-MAFSSVAVL-GAGLAYMIR-------      275
NP_001244212.2[G.gallus]             DLESS--I--RNVL-MAFAGVAGL-GASLAYMIRKWRS---      335
NP_032588.1[M.musculus]              DLEGG--I--RNVL-LAFAGVAGV-GAGLAYLIR-------      331
Q07820[H.sapiens]                    DLEGG--I--RNVL-LAFAGVAGV-GAGLAYLIR-------      350
XP_002810320.1[P.abelii]             DLEGG--I--RNVL-LAFAGVAGV-GAGLAYLIR-------      350
```
