# Supplementary material for: An Evolutionary Perspective on the Origin, Conservation and Binding Partner Acquisition of Tankyrases
Source: Biomolecules. 2022 Nov 15;12(11):1688. doi: 10.3390/biom12111688 (PMC9688111; doi:10.3390/biom12111688)
Supplement: Supplementary file 1 [file biomolecules-12-01688-s001.zip › biomolecules-1913719-supplementary-11.17 -done/Data_7/MERIT40.pdf]

MERIT40

Homo sapiens:

>sp|Q9NWV8|BABA1_HUMAN BRISC and BRCA1-A complex member 1 OS=Homo sapiens OX=9606 GN=BABAM1 PE=1 SV=1
MEVAEPSSPTEEEEEEEEHSAEPRPRTRSNPEGAEDRAVGAQASVGSRSEGEGEAASADD
GSLNTSGAGPKSWQVPPPAPEVQIRTPRVNCPEKVIICLDLSEEMSLPKLESFNGSKTNA
LNVSQKMIEMFVRTKHKIDKSHEFALVVVNDDTAWLSGLTSDPRELCSCLYDLETASCST
FNLEGLFSLIQQKTELPVTENVQTIPPPYVVRTILVYSRPPCQPQFSLTEPMKKMFQCPY
FFFDVVYIHNGTEEKEEEMSWKDMFAFMGSLDTKGTSYKYEVALAGPALELHNCMAKLLA
HPLQRPCQSHASYSLLEEEDEAIEVEATV

Pongo abelii:

>NP_001126315.1 BRISC and BRCA1-A complex member 1 [Pongo abelii]
MEVPEPSSPTEEEEEEEEHSAEPRPRTRSNPEGAEDRAVGAQASVGSRSEGEGEAASADDGSPNTSGAGP
KSWQVPPPAPEVQIRTPRVNCPEKVIICLDLSEEMSLPKLESFNGSKTNALNVSQKMIEMFVRTKHKIDK
SHEFALVVVNDDTAWLSGLTSDPRELCSCLYDLETASCSTFNLEGLFSLIQQKTELPVTENVQTIPPPYV
VRTILVYSRPPCQPQFSLTEPMKKMFQCPYFFFDVVYIHNGTEEKEEEMSWKDMFAFMGSLDTKGTSYKY
EVALAGPALELHNCMAKLLAHPLQRPCQSHASYSLLEEEDEATEVEATV

Mus musculus:

>NP_080912.2 BRISC and BRCA1-A complex member 1 [Mus musculus]
MEVAEANSPTEEEEEEEEEGEETISEPRPHTRSNPEGAEDRALGAQASVGSRSEGEGEAATADGGAASVP
GAGPKPWQVPASASEVQIRTPRVNCPEKVIICLDLSEEMSVPKLESFNGSRTNALNVSQKMVEMFVRTKH
KIDKSHEFALVVVNDDSAWLSGLTSDPRELCSCLYDLETASCSTFNLEGLFSLIQQKTELPVTENVQTIP
PPYVVRTILVYSRPPCQPQFSLTEPMKKMFQCPYFFFDIVYIHNGTEEKEEDMSWKDMFAFMGSLDTKGA
SYKYEVALAGPALELHNCMAKLLAHPLQRPCQTHASYSLLEEDEEAGEEEATV

Gallus gallus:

>XP_015155591.1 BRISC and BRCA1-A complex member 1 isoform X3 [Gallus gallus]
MERSCGMDTSGPGSAAEEEEEKAPEQRPRTRSNPEGAEDRALSAQSSVGNRSEGEGEAASSEDSPQGTAA
PDGTAWPGPAHPPPEVQVKTPRVNCPEKVIICLDLAEEMALPKLESFNGSKTNALNISQKMIEMFVRTKH
KIDKSHEFALVVVNNDATWLSGFTSDPREVCSCLYDLETVVCKSFNLDGLFNLIQQKIELPVTDNVQTIP
PPYVVRTILVLGRPGCQPQFSVSEQMKKMLQCPYFFFDVVYIHNGAEEKEDETSWKELYAFFGSLDTKGT
NYRYEVTLTGPAVELHNCMAKLLAHPLQRPFQSHAAYSLLEGDEPPEVEATV

Xenopus laevis:

>NP_001086385.1 BRISC and BRCA1-A complex member 1 [Xenopus laevis]
MDNSTEETFSMDTSEPLEEGEQTHEQRPHTRSNPEGAEDRGVVHQAGVGSRSEGEGEAAQVEDPLPTTTT
VPTNSTPPPTLEFQLKTPRVNCPEKVIICLDLSEEMSTQKLESFNGSKANALNSSQKMIEMFVRTKHKID
KRHEFALVVANNEAMWLSGFTSDPREVCSCLYDLETNVCESFNLEGLFNLIQQRTEFPVTDNVQTIPPPY
VVRIILIYSRPASQPALALTDNMKKMLQCPYFFFDVIYIHNGSEEEELCWKDIFGFFSSLDSKGTSYKYE
VSITGPALELHNCMARLLAHPLQRPFQSHAAYSLLEEEEESPESEVTV

Salmo salar:

>XP_013979057.1 PREDICTED: BRISC and BRCA1-A complex member 1 [Salmo salar]
METPQPGPADGEERSVELRPRTRSNPEGAEDRRSSTGSLGNSNPSTPQPAVGSRVEGEGEASTSDSPPSS
TTTTVSATTAQTVAPIVAATVNAIVGLPTPTPVAKDRPKPTQAPPPLTPPIPPTAEFQLRAPRVNCPEKV
IICLDLSEEMSSQKLESFNGSKTNALNISQKMIEMFVRTKHKIDKRHEFALVVVNDDALWLSGFTSDPRE
LCSCLYDLDTNVCETFNLEDLLSVIRQKIDLPLMDNIQTIPPPFVVRTLLIYSRHAGQLQFSPSDAVNSM
LLSPYFFFDVVYLHNGAEEQGDEASWRDIYTSFCNLDSKGTCYHFEVSLCGPAIELHNCMAKLLSHPLQR
PFQSHASYSLLEGDDPQDVEATV

Petromyzon marinus:

No homolog found.

Branchiostoma belcheri:

>XP_019620522.1 PREDICTED: BRISC and BRCA1-A complex member 1-like [Branchiostoma belcheri]
MSSTASTTEEESVLPDTTVSESARSESDEVVSTQEEEEKEEEEKKDPPRDETPHTENEELNSLASTVSSS
QDSQDSSDGTIHVLLPKVNCPEKIVVCLDMSKEMTSRHVSSRDGSQFSPLSLAKRVIEIFVRTKSRMDRK
HQFALVILHDAPTLMLSELLLSPHLFLDALYVHQPPADDNQSIEIFNAICDLDRKCLSYVYEVATNVTMF
HNHMAKLLAHPLQRPAQSDTYYKLQSNEEVS

Drosophila melanogaster:

No homolog found.

Hydra vulgaris:

No homolog found.

Nematostella vectensis:

>XP_032232271.1 BRISC and BRCA1-A complex member 1 isoform X1 [Nematostella vectensis]
MSKQLPETDLIDLSDDKDADSQRSSPVILTKHPNKHNTGKGRKGEEKVQEKGSKGGHESPSHSIGDSLSL
NSNDSNDPRRLVPWVNCQEKIILCLDLSNEMNEKSFPSKKAPDFTAYQFVRKAAKIFVNNKAMLNSIHEF
GVITLADDATWYQQFESNPDMICTLLDHLNPSEEDNQDTFDMEKLFQTICQRIALPEVEDPALPPPYVIR
VIMIYGRSHCPPHFTDRSRQMLRELLQSPYFFFDVLYVHELPSENNQCEAIYDSFCELDEDEDGYMLEVS
RSTSRLFDHMAILLAHPLQRPKQRDTFYELQNRDDNETT

Amphimedon queenslandica:

>XP_019851282.1 PREDICTED: BRISC and BRCA1-A complex member 1-like [Amphimedon queenslandica]
MADNISVSSSISNASVLSFDFNAQDIHPSCPEKIVFCIDVSEDVSITTVKSKDRGDLPLFTVWKNALTQF
VFNKSMMGKHEFALMILTDKANWFCDFSSDPQLVSDALTSLENVQEKYDSFDTSSIFSAIYENCPPPSVC
VGSSMSPPPFIVRVIILYGRSHCLPVYRGESEAHSELMASPHFFLDVLYSHDPPTGHNVCQSIFDILSEI
YPKPKALVLDWSANLVKLYNFFAKLLAHPLQRPVQSMMDNRLISQKT

Alignment

```
XP_013979057.1[S.salar]              ---------------METPQPGPADGEERSVELRPRTRSNPEGAEDRRSSTGSLGNSNPS           45
NP_001086385.1[X.laevis]             ------MDNSTEETFS-MDTSEPLEEGEQTHEQRPHTRSNPEGAEDRGVV----------           43
XP_015155591.1[G.gallus]             MERSCGMDTSGPG-------SAAEEEEEKAPEQRPRTRSNPEGAEDRALS----------           43
NP_080912.2[M.musculus]              ------MEVAEANSPTEEEEEEEEEGEETISEPRPHTRSNPEGAEDRALG----------           44
Q9NWV8[H.sapiens]                    ------MEVAEPSSPTEEEEEE----EEHSAEPRPRTRSNPEGAEDRAVG----------           40
NP_001126315.1[P.abelii]             ------MEVPEPSSPTEEEEEE----EEHSAEPRPRTRSNPEGAEDRAVG----------           40
XP_019851282.1[A.queenslandica]      ------------------------------------------------------------           0
XP_019620522.1[B.belcheri]           ------MSSTAS----------TTEEESVLPDTTVSESA-----R-S-ESDEVVSTQE--           35
XP_032232271.1[N.vectensis]          ------MS-------------------KQLPETDLIDLSDDKDADSQ-RSSPVILTKHPN           34


XP_013979057.1[S.salar]              TPQPAVGSRVEGEGEASTSDSPPSSTTTTVSATTAQTVAPIVAATVNAIVGLPTPTPVAK           105
NP_001086385.1[X.laevis]             -HQAGVGSRSEGEGEAAQVEDPLPTTTT--------------------------------           70
XP_015155591.1[G.gallus]             -AQSSVGNRSEGEGEAASSEDSPQGTAA--------------------------------           70
NP_080912.2[M.musculus]              -AQASVGSRSEGEGEAATADGGAASVPG--------------------------------           71
Q9NWV8[H.sapiens]                    -AQASVGSRSEGEGEAASADDGSLNTSG--------------------------------           67
NP_001126315.1[P.abelii]             -AQASVGSRSEGEGEAASADDGSPNTSG--------------------------------           67
XP_019851282.1[A.queenslandica]      -------------------------------------M----ADN---------------           4
XP_019620522.1[B.belcheri]           ---------EEEKEEEEKKDPPRDETPHTENE-ELNSL----AST---------------           66
XP_032232271.1[N.vectensis]          KHNTGKGRKGEEKVQ-EKGSKGGHESPS---------------HS---------------           63


XP_013979057.1[S.salar]              DRPKPTQAPPPLTPPIPPTAEFQLRAPRVNCPEKVIICLDLSEEMSSQKLESFNGSKTNA           165
NP_001086385.1[X.laevis]             ---------VPTNSTPPPTLEFQLKTPRVNCPEKVIICLDLSEEMSTQKLESFNGSKANA           121
XP_015155591.1[G.gallus]             ------PDGTAWPGPAHPPPEVQVKTPRVNCPEKVIICLDLAEEMALPKLESFNGSKTNA           124
NP_080912.2[M.musculus]              ------AGPKPWQVPAS-ASEVQIRTPRVNCPEKVIICLDLSEEMSVPKLESFNGSRTNA           124
Q9NWV8[H.sapiens]                    ------AGPKSWQVPPP-APEVQIRTPRVNCPEKVIICLDLSEEMSLPKLESFNGSKTNA           120
NP_001126315.1[P.abelii]             ------AGPKSWQVPPP-APEVQIRTPRVNCPEKVIICLDLSEEMSLPKLESFNGSKTNA           120
XP_019851282.1[A.queenslandica]      -----ISVSSSISNASVLSFDFNAQDIHPSCPEKIVFCIDVSEDVSITTVKSKDRGDLPL           59
XP_019620522.1[B.belcheri]           -------VSSSQDSQDSSDGTIHVLLPKVNCPEKIVVCLDMSKEMTSRHVSSRDGSQFSP           119
XP_032232271.1[N.vectensis]          -------IGDSLSLNSNDSNDPRRLVPWVNCQEKIILCLDLSNEMNEKSFPSKKAPDFTA           116
                                                           .      .* **::.*:*:::::    . * .

XP_013979057.1[S.salar]              LNISQKMIEMFVRTKHKIDKRHEFALVVVNDDALWLSGFTSDPRELCSCLYDLDTN---V           222
NP_001086385.1[X.laevis]             LNSSQKMIEMFVRTKHKIDKRHEFALVVANNEAMWLSGFTSDPREVCSCLYDLETN---V           178
XP_015155591.1[G.gallus]             LNISQKMIEMFVRTKHKIDKSHEFALVVVNNDATWLSGFTSDPREVCSCLYDLETV---V           181
NP_080912.2[M.musculus]              LNVSQKMVEMFVRTKHKIDKSHEFALVVVNDDSAWLSGLTSDPRELCSCLYDLETA---S           181
Q9NWV8[H.sapiens]                    LNVSQKMIEMFVRTKHKIDKSHEFALVVVNDDTAWLSGLTSDPRELCSCLYDLETA---S           177
NP_001126315.1[P.abelii]             LNVSQKMIEMFVRTKHKIDKSHEFALVVVNDDTAWLSGLTSDPRELCSCLYDLETA---S           177
XP_019851282.1[A.queenslandica]      FTVWKNALTQFVFNKSMMG-KHEFALMILTDKANWFCDFSSDPQLVSDALTSLENVQ-EK           117
XP_019620522.1[B.belcheri]           LSLAKRVIEIFVRTKSRMDRKHQFALVILH------------------------------           149
XP_032232271.1[N.vectensis]          YQFVRKAAKIFVNNKAMLNSIHEFGVITLADDATWYQQFESNPDMICTLLDHLNPSEEDN           176
                                         :.    ** .*  :.  *:*.::

XP_013979057.1[S.salar]              CETFNLEDLLSVIRQKIDLPLM-DNIQTIPPPFVVRTLLIYSRHAGQLQFSPS--DAVNS           279
NP_001086385.1[X.laevis]             CESFNLEGLFNLIQQRTEFPVT-DNVQTIPPPYVVRIILIYSRPASQPALALT--DNMKK           235
XP_015155591.1[G.gallus]             CKSFNLDGLFNLIQQKIELPVT-DNVQTIPPPYVVRTILVLGRPGCQPQFSVS--EQMKK           238
NP_080912.2[M.musculus]              CSTFNLEGLFSLIQQKTELPVT-ENVQTIPPPYVVRTILVYSRPPCQPQFSLT--EPMKK           238
Q9NWV8[H.sapiens]                    CSTFNLEGLFSLIQQKTELPVT-ENVQTIPPPYVVRTILVYSRPPCQPQFSLT--EPMKK           234
NP_001126315.1[P.abelii]             CSTFNLEGLFSLIQQKTELPVT-ENVQTIPPPYVVRTILVYSRPPCQPQFSLT--EPMKK           234
XP_019851282.1[A.queenslandica]      YDSFDTSSIFSAIYENCPPPSVCVGSSMSPPPFIVRVIILYGRSHCLPVYRGES-EAHSE           176
XP_019620522.1[B.belcheri]           ----------------------------------------------------DAPTLMLSE           158
XP_032232271.1[N.vectensis]          QDTFDMEKLFQTICQRIALPE--VEDPALPPPYVIRVIMIYGRSHCPPHFTDRSRQMLRE           234
                                                                                        .

XP_013979057.1[S.salar]              MLLSPYFFFDVVYLHNGAEEQGDEASWRDIYTSFCNLDSKGTCYHFEVSLCGPAIELHNC           339
NP_001086385.1[X.laevis]             MLQCPYFFFDVIYIHNGSEE--EELCWKDIFGFFSSLDSKGTSYKYEVSITGPALELHNC           293
XP_015155591.1[G.gallus]             MLQCPYFFFDVVYIHNGAEEKEDETSWKELYAFFGSLDTKGTNYRYEVTLTGPAVELHNC           298
NP_080912.2[M.musculus]              MFQCPYFFFDIVYIHNGTEEKEEDMSWKDMFAFMGSLDTKGASYKYEVALAGPALELHNC           298
Q9NWV8[H.sapiens]                    MFQCPYFFFDVVYIHNGTEEKEEEMSWKDMFAFMGSLDTKGTSYKYEVALAGPALELHNC           294
NP_001126315.1[P.abelii]             MFQCPYFFFDVVYIHNGTEEKEEEMSWKDMFAFMGSLDTKGTSYKYEVALAGPALELHNC           294
XP_019851282.1[A.queenslandica]      LMASPHFFLDVLYSHDPPTG---HNVCQSIFDILSEIYPKPKALVLDWSA--NLVKLYNF           231
XP_019620522.1[B.belcheri]           LLLSPHLFLDALYVHQPPAD---DNQSIEIFNAICDLDRKCLSYVYEVAT--NVTMFHNH           213
XP_032232271.1[N.vectensis]          LLQSPYFFFDVLYVHELPSE---NNQCEAIYDSFCELDEDEDGYMLEVSR--STSRLFDH           289
                                     :: .*::*:* :* *:       .     ::  : .:  .      : :       :.:

XP_013979057.1[S.salar]              MAKLLSHPLQRPFQSHASYSLLEGDDPQDVEATV-            373
NP_001086385.1[X.laevis]             MARLLAHPLQRPFQSHAAYSLLEEEEESPESEVTV            328
XP_015155591.1[G.gallus]             MAKLLAHPLQRPFQSHAAYSLLEGDEPPEVEATV-            332
NP_080912.2[M.musculus]              MAKLLAHPLQRPCQTHASYSLLEEDEEAGEEEATV            333
Q9NWV8[H.sapiens]                    MAKLLAHPLQRPCQSHASYSLLEEEDEAIEVEATV            329
NP_001126315.1[P.abelii]             MAKLLAHPLQRPCQSHASYSLLEEEDEATEVEATV            329
XP_019851282.1[A.queenslandica]      FAKLLAHPLQRPVQSMMDNRLISQKT---------            257
XP_019620522.1[B.belcheri]           MAKLLAHPLQRPAQSDTYYKLQSNEEVS-------            241
XP_032232271.1[N.vectensis]          MAILLAHPLQRPKQRDTFYELQNRDDNETT-----            319
                                     :* **:****** *      * . .
```
