# Supplementary material for: An Evolutionary Perspective on the Origin, Conservation and Binding Partner Acquisition of Tankyrases
Source: Biomolecules. 2022 Nov 15;12(11):1688. doi: 10.3390/biom12111688 (PMC9688111; doi:10.3390/biom12111688)
Supplement: Supplementary file 1 [file biomolecules-12-01688-s001.zip › biomolecules-1913719-supplementary-11.17 -done/Data_7/NKD2.pdf]

NKD2

Homo sapiens:

>NP_149111.1 protein naked cuticle homolog 2 isoform 1 [Homo sapiens]
MGKLQSKHAAAARKRRESPEGDSFVASAYASGRKGAEEAERRARDKQELPNGDPKEGPFREDQCPLQVAL
PAEKAEGREHPGQLLSADDGERAANREGPRGPGGQRLNIDALQCDVSVEEDDRQEWTFTLYDFDNCGKVT
REDMSSLMHTIYEVVDASVNHSSGSSKTLRVKLTVSPEPSSKRKEGPPAGQDREPTRCRMEGELAEEPRV
ADRRLSAHVRRPSTDPQPCSERGPYCVDENTERRNHYLDLAGIENYTSRFGPGSPPVQAKQEPQGRASHL
QARSRSQEPDTHAVHHRRSQVLVEHVVPASEPAARALDTQPRPKGPEKQFLKSPKGSGKPPGVPASSKSG
KAFSYYLPAVLPPQAPQDGHHLPQPPPPPYGHKRYRQKGREGHSPLKAPHAQPATVEHEVVRDLPPTPAG
EGYAVPVIQRHEHHHHHEHHHHHHHHHFHPS

Pongo abelii:

>XP_002815428.2 protein naked cuticle homolog 2 isoform X1 [Pongo abelii]
MGKLQSKHAATARKRRESPEGDSFVASGYACGRKSSEEAERRARDKQELPNGDPKEGPFREDQCPLEVAL
PPEKAEGREHPGQLLSADDGERAANREGPRGPGGQRLNIDALQCDVSVEEDDRQEWTFTLYDFDNCGKVT
REDMSSLMHTIYEVVDASVNHSLGSSKTLRVKLTVSPEPSSKRKEGPPAGQDREPTRCRMEGELAEEPRV
ADRRLSAHVRRPSADPQPCSERGPYCVDENTERRNHYLDLAGIENYTSRFGPGSSPVQAKQEPQGRASHL
QARSRSQEPDAHTVHHRRSQVLVEHVVPASEPAARALDPQPRPKGPEKQFLKSPKGSGKPPGVPASSKSG
KAFSYYLPAVLPHQAPQDGHHLPQPPPQPPPPPYGHKRYRQKGREGHSPLKAPHAQPATVEHEMVRELPP
TPAGEGFAVPVIQRHEHHHHHEHHHHHHHHHFHPS

Mus musculus:

>BAB25908.1 unnamed protein product [Mus musculus]
MGKFQSKHAAAACKRRESPEGDSFVVPAYGSGRRGAEETDRRAGSGVEHRSRDKQELLNGDPKEGPFWDD
KGSLEVVLPPEKSEGHEGQGQLFSTDDGEKAASREGPLRLSKKHLNIDALQCDVSVEEDNRQEWTFTLYD
FDNSGKVTREDMSSLMHTIYEVVDASVNHSSGSSKTLRVKLTVSPEPSSKKECPLTGQDREPTRGRTEIE
LTDEPRVADRRLSAYSRKPNADPQPCSVRVPYCVDENTERRNHYLDLAGIENYTSKFGPGSPPEQARQEH
HGRATHIPSRSRSQESDAHAIHHRRSQVLAEHVIPASEPATRALAAQPRIKGQEKQFLRSPKGPGKPLGT
PGSGKPGKALSYCLQAVPLPQSAQDGHHLPQPPPQPPPQPYGHKRYRQKAREGHSPLKGHGQPTMVEHEV
VRDLPPMLGPEGYVMPVVQRHEHHHHHEHHHHHHHHHFHPS

Gallus gallus:

>XP_040536747.1 protein naked cuticle homolog 1 isoform X1 [Gallus gallus]
MGKLHSKHAAVCKPRESPEGDSFAVNASLARKGLEDWMVKQKYSGGSGSSSGGSSSGLQGCQHRAACGLS
SGRDLSGEGYRDSIGDEHFHLEVALPPEKTDGVCSGDEKKAEKESDTRTGSKKQLKFEELQCDVSVEEDN
RQEWTFTLYDFDNNGRVTREDITSLLHTIYEVVDASVNHSPSSSKTLRVKLTVAPDGSQKKKSILLNHTD
LQSARHRAESKANEELRSSEKKQRASLRYHQSENNPEQSECYRHCVDENIERRNHYLDLAGIENYTSKFG
PGSPPAAQKQDPPSRVVSQTRSRSHEPETFHAHHRKSQVVDPSHINLAESSYAKIAEIQQRLRNQDSNKH
FVRSPKAQGKNVAPVHSGRAVRNKPFQGGAMPMASPSARVGQNLPYLPLQSQQVHKKHKQRAKENHQMCK
TFQSPGTVVEKEHVRDLPTVILYEGQVGQMIQRHEHHHHHEHHHHYHHFYQT

Xenopus laevis:

>XP_041417043.1 protein naked cuticle homolog 1 isoform X6 [Xenopus laevis]
MGKLYSKHAAVCKPRESPEGDSFAVNACLARKGIEDWIVKQKPPNSSLKTGYRITQRDSNGDICKYNIAN
EHFHLEVALPEEKSEGLFTSDDRKVDKDNDGRVKKQHKFEELQCDVSVEEDNRQEWTFTLYDFDNSGRVT
REDITRLLHTIYEVFDASVNHSLSSSKTLRVKLSLAPDATLKKRTVLVNHAELQNGRHRSECKVTENLRG
SEKKQQGSGRLENVTEQTECLRQCVDENIERRNHYLDLAGIENYTSRFGPGSPPMCEKHDSQAKASNQMR
SRSHEPETISFHHRRSQALDTGNSNMTELSHVKVAEVQQRQRGQENNKHFVRSPRASNKNGHLSTSQPGR
TRNKPSQGVSMPVNPPSAFMGQSYLYHPVPPQSVHRKSKQKTKDGHQMRKAFQSPVNVVEKEHVRDLPSM
VLYDDQVGHMIQRHEHHHHHEHHYHYHHFYQK

Salmo salar:

>XP_014031575.1 protein naked cuticle homolog 1 isoform X2 [Salmo salar]
MGKLHSKHAAICKPRESPEGDSFVVNACLARKGIDDWLVKQKYYCTGSRLEQQDCHHTNKCGLSTRDLLD
KACAEGISDEHYRLEVALPPEKTVDSCCVEEKMQGRGSQAPTGTQKQLQFEELECAVSVEEDNRQEWTFT
LYDFDNNGKVTREDITSLLHTIYEVVDTSVNHSPSSSKTLRVKLSVAPDSSQRWQSCTQGTADMPHPRTK
TDKCIEDLKSAEKKSRALLRRHHSDHHTIQHQVLTQQGQGQSRGCQRHCVDENLERRNHYLDLAGIENYT
SRFGAAPTTEPVKAEPQTRPSNQTRSRSHEPENGHTYSHHRRSQVAMESSAAPGPDTLCPPRPSKDSGKH
ALRSPKTHSRTHPTQTTTGRVMRSRGAPPPPALPSQAPPHQSSAPYRRHKQRAKEGQQQYRALGAPVGSV
VEKEQVRVLPSVLLYEGGLAQMVQRHEHHHHHEHHHHYHHFYQS

Petromyzon marinus:

>XP_032830383.1 protein naked cuticle homolog 2 [Petromyzon marinus]
MGKFQSKQACRRRESPEGDSFVVNAYANRRGLEDVPASVLPPAYRDVLYCGGVAKELFPEEVSSLSTLQT
PLSPGEDEAEDDGGGGDATDKKPADKAALNALNFQQELQCDVLVQDNNRQEWTFTLYDFDSSGEVTREDI
SSLMHTIYEVVGTSVNHSTGNSKTLRVKLTVTPDPGQQRRRGAPHGIVDQPPSHGQGEETATPEEARQPQ
KQQQPQQQQQPQQQPQQQQPEHPALRYAAKHGHAVSISGRSRENKNTLTIKTRTPQQERAGWETSPSPRQ
PAGGNPGCRRCGNGGSEMLTPSPGIQEVVGSILTLTPADGYFCVLINLAFHEGDGSVCGGPVVAASHGLA
EGPPPGCPLLPPVRRHCPRVITCPLVLFFPPPFASTVRVQCMPNMSDLFARRRHNSGQPQGSATARHCYC
VDENVERRNHYLDLAGMESYGTPACSPTQAGSPRMSRRRNAQHDPDTPAGQHGRRETVLHQRNRTPQDPS
TAAAAAAAAATTTTAARYVSRSSRTKGPAVTQAASSRPGRQRGRSVPAYVQPLRPVGVQQQQAQQQQQQ
QPQQQHKKHRQRGREGCLSPGRPPYYQTRAPAPGVPDVRWLEQPQHQHHHQHLQQQQQQLHVQQQQPGA
MQTSMLVPVMQRHEHHHYHEHHHHHHYHHYHET

Branchiostoma belcheri:

>XP_019629286.1 PREDICTED: protein naked cuticle homolog 2-like isoform X2 [Branchiostoma belcheri]
MGKLQSKHACKRRESPEGDSFVVNAHFNTGRYRGIEDFIARQKNATGLQLATYKGGRQPPEPPAQEMEKA

GGCKPVPVGMADDCFPLEVQLPPETPDPGRRTPFYVVGPNDGDSKSSVEEGEKPGLHVEEFECGVSVEGS
DKQEWTFTLYDFDGKGKVTQEDFATLLHSIYEMVGNSVHLPSKSSSKGNKNKTLKLRLTVSPPKNGKAPE
KRVETEMEQEVAKDEDGDSGNEGMSEGCETPTRKKSCSANDQMRKLNLHEFVQKSIEKQQQQQHRIAQL
RRHHSDSHNHHHHSADEVRRHRHSHHRPPSRQRQQQPLQRAREPRPPQPLPGPGRRGEPQQRSHHGDVYI
LKFPRKQPFSIKVKRPRGPPQVKVT

Drosophila melanogaster:

>NP_001262029.1 naked cuticle, isoform B [Drosophila melanogaster]
MAGNIVKWWKHKILGGYKQFSVQECTTDSEELMYHQVRASSSCSAPPDLLLVSERDNNIQLRSPVVNIIT
TPPGNASGAGSKQQSHHQTNHHSSGRSHPGHTAHPQDVSSGGSHSKHLRISSTSNGKHGKYSNMQQQLPQ
DEDVVDAAATMQQQQHTGHAHSRHLHHHKEERIRLEEFTCDVSVEGGKSSQPLQFSFTFYDLDGHHGKIT
KDDIVGIVYTIYESIGKSVVVPHCGSKTINVRLTVSPEGKSKSQPVVPVPVAAGFSSSHASKLKKLPTGL
AAMSKPLAGGGVGSGGASALTTSAGNRRQHRYRPRKLIKSDDEDDDSNSEKEKDAAHAPAADQPSGSGTK
ATGKSHHHQSQSARYHQKNNSRAEQCCTEQNTPDNGHNTYENMLNLKCCKPEVDQVDCPSHRQHHQSHPN
HQMRQQDIYMKQATQRVKMLRRARKQKKTPSQCESHTATGSTTTTTTTATPTLQYGLENTHVNYQPQSGR
PAVAAQLQSTHGGAHHPQHSSSSAGHRGQRSAAPPHSSHSSQPPQPPRASPTAHTYLPSAVGH

Hydra vulgaris:

No homolog found.

Nematostella vectensis:

>XP_032232724.1 protein naked cuticle homolog 2 isoform X4 [Nematostella vectensis]
MGKKYSKQAWISREGPEGDCVIENDTTTHSCAVSTSAYEELSRAPSSHTADFTYKGYPVNLEWPLDYEEK
ELPSKKDFFYDDFPLEVVELPDLPDGKNVEISFEREGLVEASTQTKPLKQETPECGMSVKSASSDHQEWS
YTLYDFEGQGQVTREDLRNLVKSIYEVLGKSVNNSRNQPKDPLKKLRVRLSVSKERGKGTEGDVPSRHEY
TISAMATENKAPKKSTRTYHMERCDSMTVNPPSLENIPDKRKMVSLIMEQPGPTNGINCQSHRQKISCKR
SSANRPAKPAVTAANTGNMDTPVVNDRGMYSCPKRMQTGPGECNPPSPKEANDLSRVKQWVDQLCAAHEL
ETVTRSKHRKSYRHTCRQGEPRHDLHRCPQYLDLATDHITYPYHVESQACLFGAASPKPHRRPKRGHHHS
CKYNKDEVMCQRTIVHREHHHSHHHYHHYEKQEPSNK

Amphimedon queenslandica:

No homolog found.

Alignment

```
NP_001262029.1[D.melanogaster]      MAGNIVKWWKHKILGGYKQFS-VQECTTDSEELMYHQVRASSSCSAPPDLLLVSERDNNI    59
XP_032232724.1[N.vectensis]         -------------MGKKYSKQ---AWISR--------------EGPEGDCVIENDTTTHS    30
XP_019629286.1[B.belcheri]          -------------MGKLQSKH---ACKRR--------------ESPEGDSFVVNAHFNTG    30
XP_032830383.1[P.marinus]           -------------MGKFQSKQ---ACRRR--------------ESPEGDSFVVNAYAN--    28
BAB25908.1[M.musculus]              -------------MGKFQSKHAAAACKRR--------------ESPEGDSFVVPAYGSG-    32
NP_149111.1[H.sapiens]              -------------MGKLQSKHAAAARKRR--------------ESPEGDSFVASAYASG-    32
XP_002815428.2[P.abelii]            -------------MGKLQSKHAATARKRR--------------ESPEGDSFVASGYACG-    32
XP_014031575.1[S.salar]             -------------MGKLHSKHAA-ICKPR--------------ESPEGDSFVVNACLA--    30
XP_040536747.1[G.gallus]            -------------MGKLHSKHAA-VCKPR--------------ESPEGDSFAVNASLA--    30
XP_041417043.1[X.laevis]            -------------MGKLYSKHAA-VCKPR--------------ESPEGDSFAVNACLA--    30
                                                 :*   .                         .   * .

NP_001262029.1[D.melanogaster]      QLRSPVVNIIT---TPPGN---ASGAGSKQQSHHQTNH-HSSGRSHPGHTAHPQDVSSGG    112
XP_032232724.1[N.vectensis]         C-AVSTSAYEELSRAPSSHTADFTYKGYPVNLEWPL----------D--YEEKELPSKKD    77
XP_019629286.1[B.belcheri]          R-YRGIEDFIARQ-KNATGLQLATYKGGRQPPEPPAQEM-------E--KAGGCKPVPVG    79
XP_032830383.1[P.marinus]           --RRGLEDVPASV-LPPAYR---------------------------------DVLYCGG    52
BAB25908.1[M.musculus]              --RRGAEETD------------------RR-AGSGVEHRS---RDKQ--ELLNGDPKEGP    66
NP_149111.1[H.sapiens]              --RKGAEEAE------------------R---------RA---RDKQ--ELPNGDPKEGP    58
XP_002815428.2[P.abelii]            --RKSSEEAE------------------R---------RA---RDKQ--ELPNGDPKEGP    58
XP_014031575.1[S.salar]             --RKGIDDWLVKQ-KYYCT-------GSRL-EQQDCHHTNKCGLST---RDLLDKACAEG    76
XP_040536747.1[G.gallus]            --RKGLEDWMVKQ-KYSGGSG-SSSGGSSS-GLQGCQHRAACGLSSG--RDLSGEGYRDS    83
XP_041417043.1[X.laevis]            --RKGIEDWIVKQ-KPPNSSL-KT-------GY----------RITQ--RDSNGDICKYN    67

NP_001262029.1[D.melanogaster]      SHSKHLRISSTSNGKHGKYSNMQQQLPQDEDVVDAAATMQ-QQQHTGHA-HS---RHLHH    167
XP_032232724.1[N.vectensis]         FFYDDFPLEV------VELPDL--------PDGKNVE---ISFEREGLVEASTQT-----    115
XP_019629286.1[B.belcheri]          MADDCFPLEV--------------QLPPETPDPGRRTPFYVVGPNDGDSKSS-----VEE    120
XP_032830383.1[P.marinus]           VAKELFPEEV------SSLSTLQTPLSPGEDEA----------EDDGGGGDATDKKPADK    96
BAB25908.1[M.musculus]              FWDDKGSLEV--------------VLPPEKSEGH-EGQGQLFSTDDGEKAASREG--PLR    109
NP_149111.1[H.sapiens]              FREDQCPLQV--------------ALPAEKAEGR-EHPGQLLSADDGERAANREG--PRG    101
XP_002815428.2[P.abelii]            FREDQCPLEV--------------ALPPEKAEGR-EHPGQLLSADDGERAANREG--PRG    101
XP_014031575.1[S.salar]             ISDEHYRLEV--------------ALPPEKTVDS-CC------VEEKMQGRGSQA---PT    112
XP_040536747.1[G.gallus]            IGDEHFHLEV--------------ALPPEKTDGV-CS------GDEKKAEKESDT---RT    119
XP_041417043.1[X.laevis]            IANEHFHLEV--------------ALPEEKSEGL-FT------SDDRKVDKDNDG---R-    102
                                       .    .

NP_001262029.1[D.melanogaster]      HKEERI-RLEEFTCDVSVEGGKSSQPLQFSFTFYDLDGHHGKITKDDIVGIVYTIYESIG    226
XP_032232724.1[N.vectensis]         ---KPL-KQETPECGMSVKSAS-SDHQEWSYTLYDFEG-QGQVTREDLRNLVKSIYEVLG    169
XP_019629286.1[B.belcheri]          GEKPGL-HVEEFECGVSVE--G-SDKQEWTFTLYDFDG-KGKVTQEDFATLLHSIYEMVG    175
XP_032830383.1[P.marinus]           AALNALNFQQELQCDVLVQ--D-NNRQEWTFTLYDFDS-SGEVTREDISSLMHTIYEVVG    152
BAB25908.1[M.musculus]              LSKKHLN-IDALQCDVSVE--E-DNRQEWTFTLYDFDN-SGKVTREDMSSLMHTIYEVVD    164
NP_149111.1[H.sapiens]              PGGQRLN-IDALQCDVSVE--E-DDRQEWTFTLYDFDN-CGKVTREDMSSLMHTIYEVVD    156
XP_002815428.2[P.abelii]            PGGQRLN-IDALQCDVSVE--E-DDRQEWTFTLYDFDN-CGKVTREDMSSLMHTIYEVVD    156
XP_014031575.1[S.salar]             GTQKQLQ-FEELECAVSVE--E-DNRQEWTFTLYDFDN-NGKVTREDITSLLHTIYEVVD    167
XP_040536747.1[G.gallus]            GSKKQLK-FEELQCDVSVE--E-DNRQEWTFTLYDFDN-NGRVTREDITSLLHTIYEVVD    174
XP_041417043.1[X.laevis]            -VKKQHK-FEELQCDVSVE--E-DNRQEWTFTLYDFDN-SGRVTREDITRLLHTIYEVFD    156
                                             :   * : *:    .:  ::::*:**::.  *.:*::*:  :: :*** ..

NP_001262029.1[D.melanogaster]      KSVVVPHC------GSKTINVRLTVSPEGKSKSQPVVPVPVAAGFSSSHASKLKKLPTGL    280
XP_032232724.1[N.vectensis]         KSVNNSRNQP--KDPLKKLRVRLSVSKERGKGTEGDVPSRH-EY----------------    210
XP_019629286.1[B.belcheri]          NSVHLPSKSSSKGNKNKTLKLRLTVSPPKNGKAPEKRVETEMEQEV--------------    221
XP_032830383.1[P.marinus]           TSVNHSTG------NSKTLRVKLTVTPDPGQQRRRGAPHGIVDQ----------------    190
BAB25908.1[M.musculus]              ASVNHSSG------SSKTLRVKLTVSPEPSSK-KECPLTGQDRE----------------    201
NP_149111.1[H.sapiens]              ASVNHSSG------SSKTLRVKLTVSPEPSSKRKEGPPAGQDRE----------------    194
XP_002815428.2[P.abelii]            ASVNHSLG------SSKTLRVKLTVSPEPSSKRKEGPPAGQDRE----------------    194
XP_014031575.1[S.salar]             TSVNHSPS------SSKTLRVKLSVAPDSSQRWQSCTQGTADMP----------------    205
XP_040536747.1[G.gallus]            ASVNHSPS------SSKTLRVKLTVAPDGSQKKKSILLNHTDLQ----------------    212
XP_041417043.1[X.laevis]            ASVNHSLS------SSKTLRVKLSLAPDATLKKRTVLVNHAELQ----------------    194
                                     **             *.:.::*:::

NP_001262029.1[D.melanogaster]      AAMSKPLAGGGVGSGGASALT-------TSAGNRRQHRYRPRKLIKSDDEDDDSNSE---    330
XP_032232724.1[N.vectensis]         -----------------T-ISAMATENKAPK--KSTRTYHMERC----------------    234
XP_019629286.1[B.belcheri]          ---AKD-EDGDSGNEGMSEGCETPTRKKSCSANDQMRKLNLHEFVQKSIEKQQQQQQHRI    277
XP_032830383.1[P.marinus]           -----------PPSHGQGEETATPEEARQPQ--K---QQQPQQQQQ--PQQQPQQQQPEH    232
BAB25908.1[M.musculus]              -----------PTRG-RTEIELT-DEPRVAD--R---RL---------------------    222
NP_149111.1[H.sapiens]              -----------PTRC-RMEGELA-EEPRVAD--R---RL---------------------    215
XP_002815428.2[P.abelii]            -----------PTRC-RMEGELA-EEPRVAD--R---RL---------------------    215
XP_014031575.1[S.salar]             -----------HPRT-KT-DKCI-EDLKSAE--K---KS---------------------    225
XP_040536747.1[G.gallus]            -----------SARH-RAESKAN-EELRSSE--K---KQ---------------------    233
XP_041417043.1[X.laevis]            -----------NGRH-RSECKVT-ENLRGSE--K---KQ---------------------    215

NP_001262029.1[D.melanogaster]      ------------------------K-E---------------KDAAHAPAADQPSGS---    347
XP_032232724.1[N.vectensis]         --------------------------DSMTVNPPSL---------EN-------------    246
XP_019629286.1[B.belcheri]          AQLRRHHSDSHNHHHHSADEVRRHRHSHH--RPPSRQRQQQPLQRAREPRPPQPL---PG    332
XP_032830383.1[P.marinus]           PALRYAA--KHG---HAVSISGRSRENKNTLTIKTRTPQQERAGWETSPSPRQPAGGNPG    287
BAB25908.1[M.musculus]              -------------------------------SA----------------YSRK-------    228
NP_149111.1[H.sapiens]              -------------------------------SA----------------HVRR-------    221
XP_002815428.2[P.abelii]            -------------------------------SA----------------HVRR-------    221
XP_014031575.1[S.salar]             -------------------------------RA----------------LLRR-------    231
XP_040536747.1[G.gallus]            -------------------------------RA----------------SLRY-------    239
XP_041417043.1[X.laevis]            -------------------------------QG----------------SGRL-------    221

NP_001262029.1[D.melanogaster]      -GTKATGKSHHHQ-SQSARYHQKNNSRAEQCCTEQNTPDNGHNTYENMLN--------LK    397
XP_032232724.1[N.vectensis]         --------------IPDKRK-------MVSLIMEQPGPTNGINCQSHRQKISCKRSSANR    285
XP_019629286.1[B.belcheri]          PGRRGEPQ---------QRS-------HHGDVYILKFPRKQPFSIK--VK--RPRGPPQ-    371
XP_032830383.1[P.marinus]           CRRCGNGGSEMLTPSPGIQE-------VVGSILTL-TPADGYFCVLINLA--FHEGDGSV    337
BAB25908.1[M.musculus]              -----------------------------------------------------PNADPQ-    234
NP_149111.1[H.sapiens]              -----------------------------------------------------PSTDPQ-    227
XP_002815428.2[P.abelii]            -----------------------------------------------------PSADPQ-    227
XP_014031575.1[S.salar]             -----------------------------------------------------HHSDHH-    237
```

```
XP_040536747.1[G.gallus]          -----------------------------------------------------HQSENN-    245
XP_041417043.1[X.laevis]          --------------------------------------------------------ENV-    224


NP_001262029.1[D.melanogaster]    CCKPEVD--------------------------QVDCPSHRQ------------------    413
XP_032232724.1[N.vectensis]       PAKPAVTAANTGNMDTPV-----VN-----DRGMYSCPKRMQTGPGECNPPSPKEANDLS    335
XP_019629286.1[B.belcheri]        -----V-KVT--------------------------------------------------    375
XP_032830383.1[P.marinus]         CGGPVV-AASHGLAEGPPPGCPLLPPVRRHCPRVITCPLVLFFPP------PFA------    384
BAB25908.1[M.musculus]            ------------------------------------------------------------    234
NP_149111.1[H.sapiens]            ------------------------------------------------------------    227
XP_002815428.2[P.abelii]          ------------------------------------------------------------    227
XP_014031575.1[S.salar]           ---------------------------------TI-------------------------    239
XP_040536747.1[G.gallus]          ------------------------------------------------------------    245
XP_041417043.1[X.laevis]          ------------------------------------------------------------    224


NP_001262029.1[D.melanogaster]    ----------------------------------HHQSHPNHQMRQQDIYMKQATQRVKM    439
XP_032232724.1[N.vectensis]       RVKQWVDQLCAAHELETVTRSKHRK----SYRHTCRQGEPRHDLHRCPQYLDLATDHI--    389
XP_019629286.1[B.belcheri]        ------------------------------------------------------------    375
XP_032830383.1[P.marinus]         ---STVRVQCMPNMSDLFARRRHNSGQPQGSATARHCYCVDENVERRNHYLDLAGMESYG    441
BAB25908.1[M.musculus]            ------------------------------PCSVRVPYCVDENTERRNHYLDLAGIENYT    264
NP_149111.1[H.sapiens]            ------------------------------PCSERGPYCVDENTERRNHYLDLAGIENYT    257
XP_002815428.2[P.abelii]          ------------------------------PCSERGPYCVDENTERRNHYLDLAGIENYT    257
XP_014031575.1[S.salar]           -------------------QHQVLTQQGQGQSRGCQRHCVDENLERRNHYLDLAGIENYT    280
XP_040536747.1[G.gallus]          -----------------------------PEQSECYRHCVDENIERRNHYLDLAGIENYT    276
XP_041417043.1[X.laevis]          -----------------------------TEQTECLRQCVDENIERRNHYLDLAGIENYT    255


NP_001262029.1[D.melanogaster]    LRRAR-KQKKTPSQCESHTATGSTT---TTTTTATPTL----QYGL-----ENTHVNYQ-    485
XP_032232724.1[N.vectensis]       ------------------------------------------------------------    389
XP_019629286.1[B.belcheri]        ------------------------------------------------------------    375
XP_032830383.1[P.marinus]         TPACSPTQAGSPRMSR------------RRNAQHDPDTPAG-QHGRRETVLHQRNRTPQD    488
BAB25908.1[M.musculus]            SKFG----PGSPPEQARQEHHGRATHIPSRSRSQESDAHA--IHHRRSQVLAEHVIPASE    318
NP_149111.1[H.sapiens]            SRFG----PGSPPVQAKQEPQGRASHLQARSRSQEPDTHA--VHHRRSQVLVEHVVPASE    311
XP_002815428.2[P.abelii]          SRFG----PGSSPVQAKQEPQGRASHLQARSRSQEPDAHT--VHHRRSQVLVEHVVPASE    311
XP_014031575.1[S.salar]           SRFG----AAPTTEPVKAEPQTRPSN-QTRSRSHEPENGHTYSHHRRSQVAMESSAA---    332
XP_040536747.1[G.gallus]          SKFG----PGSPPAAQKQDPPSRVVS-QTRSRSHEPETFH--AHHRKSQVVDPSHINLAE    329
XP_041417043.1[X.laevis]          SRFG----PGSPPMCEKHDSQAKASN-QMRSRSHEPETIS--FHHRRSQALDTGNSNMTE    308


NP_001262029.1[D.melanogaster]    PQSGRPAVAAQLQSTHGGAHH------------PQHSSSSAG-HRGQRS--AAPP-----    525
XP_032232724.1[N.vectensis]       --------------------------------------------------TYPYHVESQA    399
XP_019629286.1[B.belcheri]        ------------------------------------------------------------    375
XP_032830383.1[P.marinus]         PSTAAAAAAAAAATTTTAARYVSRSSRTKGPAVTQAASSRPGRQRGRSVPAYVQPLRPVG    548
BAB25908.1[M.musculus]            ---PATRALAAQPRIKGQEKQFLRSPKGPGKPLGTPGSGKPGKALSYCLQAVPLP---QS    372
NP_149111.1[H.sapiens]            ---PAARALDTQPRPKGPEKQFLKSPKGSGKPPGVPASSKSGKAFSYYLPAVLPP---QA    365
XP_002815428.2[P.abelii]          ---PAARALDPQPRPKGPEKQFLKSPKGSGKPPGVPASSKSGKAFSYYLPAVLPH---QA    365
XP_014031575.1[S.salar]           ---PGPDTLCPPRPSKDSGKHALRSPKTHSRTHPTQTTT-GRVMRSR---GAPP----PP    381
XP_040536747.1[G.gallus]          SSYAKIAEIQQRLRNQDSNKHFVRSPKAQGKNVAPV-HS-GRAVRNKPFQGGAMPMASPS    387
XP_041417043.1[X.laevis]          LSHVKVAEVQQRQRGQENNKHFVRSPRASNKNGHLSTSQ-PGRTRNKPSQGVSMPVNPPS    367


NP_001262029.1[D.melanogaster]    --HSSHSSQPPQPPRASPTAHTY-----------------------LPSAVGH-------    553
XP_032232724.1[N.vectensis]       CLFGAASPK----------PHRR-----------------------------------PK    414
XP_019629286.1[B.belcheri]        ------------------------------------------------------------    375
XP_032830383.1[P.marinus]         VQQQQAQQQ---QQQQPQQQHKKHRQRGREGCLSPGRPPYYQTRAPAPGVPDVRWLEQPQ    605
BAB25908.1[M.musculus]            AQDGHHLPQPPPQPPPQPYGHKRYRQKAREGHSPL-----KG-HGQ-PTMVEH-------    418
NP_149111.1[H.sapiens]            PQDGHHLPQP----PPPPYGHKRYRQKGREGHSPL-----KAPHAQ-PATVEH-------    408
XP_002815428.2[P.abelii]          PQDGHHLPQPPPQPPPPPYGHKRYRQKGREGHSPL-----KAPHAQ-PATVEH-------    412
XP_014031575.1[S.salar]           ALPSQ---APPHQ---SSAPYRRHKQRAKEGQQQY-----RALGAPVGSVVEK-------    423
XP_040536747.1[G.gallus]          ARVGQNLPYLPLQ---SQQVHKKHKQRAKENHQMC-----KTFQSP-GTVVEK-------    431
XP_041417043.1[X.laevis]          AFMGQSYLYHPVP---PQSVHRKSKQKTKDGHQMR-----KAFQSP-VNVVEK-------    411


NP_001262029.1[D.melanogaster]    ------------------------------------------------------------    553
XP_032232724.1[N.vectensis]       RGHHHSC------------------KY-NKDEVMCQRTIVHR---HEHHHSHHHYHHYEK    452
XP_019629286.1[B.belcheri]        ------------------------------------------------------------    375
XP_032830383.1[P.marinus]         HQHHHQHLQQQQQQQLHVQQQQPGAMQTSMLVPVMQRHEHH---HYHEHHHHHHYHHYHE    662
BAB25908.1[M.musculus]            ---------------EVVRDLPPMLGPEGYVMPVVQRHEHHHHHEHHHHHHHHHFHPS--    461
NP_149111.1[H.sapiens]            ---------------EVVRDLPPTPAGEGYAVPVIQRHEHHHHHEHHHHHHHHHFHPS--    451
XP_002815428.2[P.abelii]          ---------------EMVRELPPTPAGEGFAVPVIQRHEHHHHHEHHHHHHHHHFHPS--    455
XP_014031575.1[S.salar]           ---------------EQVRVLPSVLLYEGGLAQMVQRH-----EHHHHHEHHHHYHHFYQ    463
XP_040536747.1[G.gallus]          ---------------EHVRDLPTVILYEGQVGQMIQRH-----EHHHHHEHHHHYHHFYQ    471
XP_041417043.1[X.laevis]          ---------------EHVRDLPSMVLYDDQVGHMIQRH-----EHHHHHEHHYHYHHFYQ    451


NP_001262029.1[D.melanogaster]    ------        553
XP_032232724.1[N.vectensis]       QEPSNK        458
XP_019629286.1[B.belcheri]        ------        375
XP_032830383.1[P.marinus]         T-----        663
BAB25908.1[M.musculus]            ------        461
NP_149111.1[H.sapiens]            ------        451
XP_002815428.2[P.abelii]          ------        455
XP_014031575.1[S.salar]           S-----        464
XP_040536747.1[G.gallus]          T-----        472
XP_041417043.1[X.laevis]          K-----        452
```
