# Supplementary material for: An Evolutionary Perspective on the Origin, Conservation and Binding Partner Acquisition of Tankyrases
Source: Biomolecules. 2022 Nov 15;12(11):1688. doi: 10.3390/biom12111688 (PMC9688111; doi:10.3390/biom12111688)
Supplement: Supplementary file 1 [file biomolecules-12-01688-s001.zip › biomolecules-1913719-supplementary-11.17 -done/Data_7/Notch2.pdf]

Notch2

Homo sapiens:

>Q04721.3 NOTC2_HUMAN Neurogenic locus notch homolog protein 2 OS=Homo sapiens OX=9606 GN=NOTCH2 PE=1 SV=3
MPALRPALLWALLALWLCCAAPAHALQCRDGYEPCVNEGMCVTYHNGTGYCKCPEGFLGEYCQHRDPCEKNRCQNGGTCVAQAMLGKATCRCASGFTGEDCQYSTSHPCFVSRPCLNGGTCHMLSRDTY
ECTCQVGFTGKECQWTDACLSHPCANGSTCTTVANQFSCKCLTGFTGQKCETDVNECDIPGHCQHGGTCLNLPGSYQCQCPQGFTGQYCDSLYVPCAPSPCVNGGTCRQTGDFTFECNCLPGFEGSTCE
RNIDDCPNHRCQNGGVCVDGVNTYNCRCPPQWTGQFCTEDVDECLLQPNACQNGGTCANRNGGYGCVCVNGWSGDDCSENIDDCAFASCTPGSTCIDRVASFSCMCPEGKAGLLCHLDDACISNPCHKG
ALCDTNPLNGQYICTCPQGYKGADCTEDVDECAMANSNPCEHAGKCVNTDGAFHCECLKGYAGPRCEMDINECHSDPCQNDATCLDKIGGFTCLCMPGFKGVHCELEINECQSNPCVNNGQCVDKVNRF
QCLCPPGFTGPVCQIDIDDCSSTPCLNGAKCIDHPNGYECQCATGFTGVLCEENIDNCDPDPCHHGQCQDGIDSYTCICNPGYMGAICSDQIDECYSSPCLNDGRCIDLVNGYQCNCQPGTSGVNCEIN
FDDCASNPCIHGICMDGINRYSCVCSPGFTGQRCNIDIDECASNPCRKGATCINGVNGFRCICPEGPHHPSCYSQVNECLSNPCIHGNCTGGLSGYKCLCDAGWVGINCEVDKNECLSNPCQNGGTCDN
LVNGYRCTCKKGFKGYNCQVNIDECASNPCLNQGTCFDDISGYTCHCVLPYTGKNCQTVLAPCSPNPCENAAVCKESPNFESYTCLCAPGWQGQRCTIDIDECISKPCMNHGLCHNTQGSYMCECPPGF
SGMDCEEDIDDCLANPCQNGGSCMDGVNTFSCLCLPGFTGDKCQTDMNECLSEPCKNGGTCSDYVNSYTCKCQAGFDGVHCENNINECTESSCFNGGTCVDGINSFSCLCPVGFTGSFCLHEINECSSH
PCLNEGTCVDGLGTYRCSCPLGYTGKNCQTLVNLCSRSPCKNKGTCVQKKAESQCLCPSGWAGAYCDVPNVSCDIAASRRGVLVEHLCQHSGVCINAGNTHYCQCPLGYTGSYCEEQLDECASNPCQHG
ATCSDFIGGYRCECVPGYQGVNCEYEVDECQNQPCQNGGTCIDLVNHFKCSCPPGTRGLLCEENIDDCARGPHCLNGGQCMDRIGGYSCRCLPGFAGERCEGDINECLSNPCSSEGSLDCIQLTNDYLC
VCRSAFTGRHCETFVDVCPQMPCLNGGTCAVASNMPDGFICRCPPGFSGARCQSSCGQVKCRKGEQCVHTASGPRCFCPSPRDCESGCASSPCQHGGSCHPQRQPPYYSCQCAPPFSGSRCELYTAPPS
TPPATCLSQYCADKARDGVCDEACNSHACQWDGGDCSLTMENPWANCSSPLPCWDYINNQCDELCNTVECLFDNFECQGNSKTCKYDKYCADHFKDNHCDQGCNSEECGWDGLDCAADQPENLAEGTLV
IVVLMPPEQLLQDARSFLRALGTLLHTNLRIKRDSQGELMVYPYYGEKSAAMKKQRMTRRSLPGEQEQEVAGSKVFLEIDNRQCVQDSDHCFKNTDAAAALLASHAIQGTLSYPLVSVVSESLTPERTQ
LLYLLAVAVVIILFIILLGVIMAKRKRKHGSLWLPEGFTLRRDASNHKRREPVGQDAVGLKNLSVQVSEANLIGTGTSEHWVDDEGPQPKKVKAEDEALLSEEDDPIDRRPWTQQHLEAADIRRTPSLA
LTPPQAEQEVDVLDVNVRGPDGCTPLMLASLRGGSSDLSDEDEDAEDSSANIITDLVYQGASLQAQTDRTGEMALHLAARYSRADAAKRLLDAGADANAQDNMGRCPLHAAVAADAQGVFQILIRNRVT
DLDARMNDGTTPLILAARLAVEGMVAELINCQADVNAVDDHGKSALHWAAAVNNVEATLLLLKNGANRDMQDNKEETPLFLAAREGSYEAAKILLDHFANRDITDHMDRLPRDVARDRMHHDIVRLLDE
YNVTPSPPGTVLTSALSPVICGPNRSFLSLKHTPMGKKSRRPSAKSTMPTSLPNLAKEAKDAKGSRRKKSLSEKVQLSESSVTLSPVDSLESPHTYVSDTTSSPMITSPGILQASPNPMLATAAPPAPV
HAQHALSFSNLHEMQPLAHGASTVLPSVSQLLSHHHIVSPGSGSAGSLSRLHPVPVPADWMNRMEVNETQYNEMFGMVLAPAEGTHPGIAPQSRPPEGKHITTPREPLPPIVTFQLIPKGSIAQPAGAP
QPQSTCPPAVAGPLPTMYQIPEMARLPSVAFPTAMMPQQDGQVAQTILPAYHPFPASVGKYPTPPSQHSYASSNAAERTPSHSGHLQGEHPYLTPSPESPDQWSSSSPHSASDWSDVTTSPTPGGAGGG
QRGPGTHMSEPPHNNMQVYA

Pongo abelii:

>XP_009243797.2 neurogenic locus notch homolog protein 2 isoform X1 [Pongo abelii]
MPALRPALPWALLALWLCCAAPARALQCRDGYEPCVNEGMCVTYHNGTGYCKCPEGFLGEYCQHRDPCEKNRCQNGGTCVAQAMLGKATCRCASGFTGEDCQYSTSHPCFVSRPCLNGGTCHMLSRDTY
ECTCQVGFTGKECQWTDACLSHPCANGSTCTTVANQFSCKCLPGFTGQKCETDVNECDIPGHCQHGGTCLNLPGSYQCQCPQGFTGQYCDSLYVPCAPSPCVNGGTCRQTGDFTFECSCLPGFEGSTCE
RNIDDCPNHRCQNGGVCVDGVNTYNCRCPPQWTGQFCTEDVDECLLQPNACQNGGTCANRNGGYGCVCVNGWSGDDCSENIDDCAFASCTPGSTCIDRVASFSCMCPEGKAGLLCHLDDACISNPCHKG
ALCDTNPLNGQYICTCPQGYKGADCTEDVDECAMANSNPCEHAGKCVNTDGAFHCECLKGYAGPRCEMDINECHSDPCQNDATCLDKIGGFTCLCMPGFKGVHCELEINECQSNPCVNNGQCVDKVNRF
QCLCPPGFTGPVCQIDIDDCSSTPCLNGAKCIDHPNGYECQCATGFTGVLCEENIDNCDPDPCHHGQCQDGIDSYTCICNPGYMGAICSDQIDECYSSPCLNDGRCIDLVNGYQCNCQPGTSGVNCEIN
FDDCASNPCIHGICMDGINRYSCVCSPGFTGQRCNIDIDECASNPCRKGATCINGVNGFRCICPEGPHHPSCYSQVNECLSNPCIHGNCTGGLSGYKCLCDAGWIGINCEVDKNECLSNPCQNGGTCDN
LVNGYRCTCKKGFKGYNCQVNIDECASNPCLNQGTCFDDISGYTCHCVLPYTGKNCQTVLAPCSPNPCENAAVCKESPNFESYTCLCAPGWQGQRCTIDIDECISKPCMNHGLCHNTQGSYMCECPPGF
SGMDCEEDIDDCLANPCQNGGSCVDGVNAFSCLCLLGFTGDKCQTDMNECLSEPCKNGGTCSDYVNSYTCKCQAGFDGVHCENNINECTESSCFNGGTCVDGINSFSCLCPVGFTGSFCLHEINECSSH
PCLNEGTCVDGLGTYRCSCPLGYTGKNCQTLVNLCSRSPCKNKGTCVQKKAESQCLCPSGWAGAYCDVPNVSCDIAASRGVLVEHLCQHSGVCINAGNTHYCQCPLGYTGSYCEEQLDECVSNPCQHG
ATCSDFIGGYRCECVPGYQGVNCEYEVDECQNQPCQNGGTCIDLVNHFKCSCPPGTRGLLCEENIDDCARGPHCLNGGQCMDRIGGYSCRCLPGFAGERCEGDINECLSNPCSSEGSLDCIQLTNDYLC
VCRSAFTGRHCETFIDVCPQMPCLNGGTCAVASNMPDGFICRCPPGFSGARCQSSCGQVKCRKGEQCVHTASGPRCFCPSPRDCESGCASSPCQHGGSCHPQRQPPYYSCQCAPPFSGSRCELYTAPPS
TPPATCLSQYCADKARDGVCDEACNSHACQWDGGDCSLTMENPWANCSSPLPCWDYINNQCDELCNTAECLFDNFECQGNSKTCKYDKYCADHFKDNHCDQGCNSEECGWDGLDCAADQPENLAEGTLV
IVVLMPPEQLLQDARSFLRALGTLLHTNLRIKRDSQGELMVYPYYGEKSAAMKKQRMTRRSLPGEQEQEVAGSKVFLEIDNRQCVQDSDHCFKNTDAAAALLASHAIQGTLSYPLVSVVSESLTPERTQ
LLYLLAVAVVIILFIILLGVIMAKRKRKHGSLWLPEGFTLRRDASNHKRREPVGQDAVGLKNLSVQVSEANLIGSGTSEHWVDDEGPQPKKVKAEDEALLSEEDDPIDRRPWTQQHLEAADIRRTPSLA
LTPPQAEQEVDVLDVNVRGPDGCTPLMLASLRGGSSDLSDEDEDAEDSSANIITDLVYQGASLQAQTDRTGEMALHLAARYSRADAAKRLLDAGADANAQDNMGRCPLHAAVAADAQGVFQILIRNRVT
DLDARMNDGTTPLILAARLAVEGMVAELINCQADVNAVDDHGKSALHWAAAVNNVEATILLLKNGANRDMQDNKEETPLFLAAREGSYEAAKILLDHFANRDITDHMDRLPRDVARDRMHHDIVRLLDE
YNVTPSPPGTVLTSALSPVICGPNRSFLSLKHTPMGKKSRRPSAKSTMPTSLPNLAKEAKDAKGSRRKKSLSEKVQLSESSVTLSPVDSLESPHTYVSDTTSSPMITSPGILQASPNPMLATAAPPAPV
HAQHALSFSNLHEVQPLAHGASTVLPSVSQLLSHHHIVSPGSGSAGSLSRLHPVPVPADWMNRMEVNETQYNEMFGMVLAPAEGTHPGIAPQSRPPEGKHITTPREPLPPIVTFQLIPKGSIAQPAGAP
QPQSTCPPAVAGPLPTMYQIPEMARLPSVAFPTAMMPQQDGQVAQTILPAYHPFPASMGKYPTPPSQHSYASSNAAERTPSHSGHLQGEHPYLTPSPESPDQWSSSSPHSASDWSDVTTSPTPGGAGGG
QRGPGTHMSEPPHNNMQVYA

Mus musculus:

>NP_035058.2 neurogenic locus notch homolog protein 2 precursor [Mus musculus]
MPALRPAALRALLWLWLCGAGPAHALQCRGGQEPCVNEGTCVTYHNGTGFCRCPEGFLGEYCQHRDPCEKNRCQNGGTCVPQGMLGKATCRCAPGFTGEDCQYSTSHPCFVSRPCQNGGTCHMLSRDTY
ECTCQVGFTGKQCQWTDACLSHPCENGSTCTSVASQFSCKCPAGLTGQKCEADINECDIPGRCQHGGTCLNLPGSYRCQCPQGFTGQHCDSPYVPCAPSPCVNGGTCRQTGDFTFECNCLPGFEGSTCE
RNIDDCPNHKCQNGGVCVDGVNTYNCRCPPQWTGQFCTEDVDECLLQPNACQNGGTCTNRNGGYGCVCVNGWSGDDCSENIDDCAYASCTPGSTCIDRVASFSCLCPEGKAGLLCHLDDACISNPCHKG
ALCDTNPLNGQYICTCPQGYKGADCTEDVDECAMANSNPCEHAGKCVNTDGAFHCECLKGYAGPRCEMDINECHSDPCQNDATCLDKIGGFTCLCMPGFKGVHCELEVNECQSNPCVNNGQCVDKVNRF
QCLCPPGFTGPVCQIDIDDCSSTPCLNGAKCIDHPNGYECQCATGFTGILCDENIDNCDPDPCHHGQCQDGIDSYTCICNPGYMGAICSDQIDECYSSPCLNDGRCIDLVNGYQCNCQPGTSGLNCEIN
FDDCASNPCMHGVCVDGINRYSCVCSPGFTGQRCNIDIDECASNPCRKGATCINDVNGFRCICPEGPHHPSCYSQVNECLSNPCIHGNCTGGLSGYKCLCDAGWVGVNCEVDKNECLSNPCQNGGTCNN
LVNGYRCTCKKGFKGYNCQVNIDECASNPCLNQGTCFDDVSGYTCHCMLPYTGKNCQTVLAPCSPNPCENAAVCKEAPNFESFSCLCAPGWQGKRCTVDVDECISKPCMNNGVCHNTQGSYVCECPPGF
SGMDCEEDINDCLANPCQNGGSCVDHVNTFSCQCHPGFIGDKCQTDMNECLSEPCKNGGTCSDYVNSYTCTCPAGFHGVHCENNIDECTESSCFNGGTCVDGINSFSCLCPVGFTGPFCLHDINECSSN
PCLNAGTCVDGLGTYRCICPLGYTGKNCQTLVNLCSRSPCKNKGTCVQEKARPHCLCPPGWDGAYCDVLNVSCKAAALQKGVPVEHLCQHSGICINAGNTHHCQCPLGYTGSYCEEQLDECASNPCQHG
ATCNDFIGGYRCECVPGYQGVNCEYEVDECQNQPCQNGGTCIDLVNHFKCSCPPGTRGLLCEENIDECAGGPHCLNGGQCVDRIGGYTCRCLPGFAGERCEGDINECLSNPCSSEGSLDCVQLKNNYNC
ICRSAFTGRHCETFLDVCPQKPCLNGGTCAVASNMPDGFICRCPPGFSGARCQSSCGQVKCRRGEQCIHTDSGPRCFCLNPKDCESGCASNPCQHGGTCYPQRQPPHYSCRCPPSFGGSHCELYTAPTS
TPPATCQSQYCADKARDGICDEACNSHACQWDGGDCSLTMEDPWANCTSTLRCWEYINNQCDEQCNTAECLFDNFECQRNSKTCKYDKYCADHFKDNHCDQGCNSEECGWDGLDCASDQPENLAEGTLI
IVVLLPPEQLLQDSRSFLRALGTLLHTNLRIKQDSQGALMVYPYFGEKSAAMKKQKMTRRSLPEEQEQEQEVIGSKIFLEIDNRQCVQDSDQCFKNTDAAAALLASHAIQGTLSYPLVSVFSELESPRN
AQLLYLLAVAVVIILFFILLGVIMAKRKRKHGFLWLPEGFTLRRDSSNHKRREPVGQDAVGLKNLSVQVSEANLIGSGTSEHWVDDEGPQPKKAKAEDEALLSEDDPIDRRPWTQQHLEAADIRHTPSL
ALTPPQAEQEVDVLDVNVRGPDGCTPLMLASLRGGSSDLSDEDEDAEDSSANIITDLVYQGASLQAQTDRTGEMALHLAARYSRADAAKRLLDAGADANAQDNMGRCPLHAAVAADAQGVFQILIRNRV
TDLDARMNDGTTPLILAARLAVEGMVAELINCQADVNAVDDHGKSALHWAAAVNNVEATLLLLKNGANRDMQDNKEETPLFLAAREGSYEAAKILLDHFANRDITDHMDRLPRDVARDRMHHDIVRLLD
EYNVTPSPPGTVLTSALSPVLCGPNRSFLSLKHTPMGKKARRPNTKSTMPTSLPNLAKEAKDAKGSRRKKCLNEKVQLSESSVTLSPVDSLESPHTYVSDATSSPMITSPGILQASPTPLLAAAAPAAP
VHTQHALSFSNLHDMQPLAPGASTVLPSVSQLLSHHHIAPPGSSSAGSLGRLHPVPVPADWMNRVEMNETQYSEMFGMVLAPAEGAHPGIAAPQSRPPEGKHMSTQREPLPPIVTFQLIPKGSIAQAAG
APQTQSSCPPAVAGPLPSMYQIPEMPRLPSVAFPPTMMPQQEGQVAQTIVPTYHPFPASVGKYPTPPSQHSYASSNAAERTPSHGGHLQGEHPYLTPSPESPDQWSSSSPHSASDWSDVTTSPTPGGGG
GGQRGPGTHMSEPPHSNMQVYA

Gallus gallus:

>NP_001238962.2 neurogenic locus notch homolog protein 2 precursor [Gallus gallus]
MARGAGPAFAALFGLVLAVLSRAEPAAALQCMDDSKPCVNEGKCIPYQNGTGYCKCREGYLGDYCQYRNPCESNTCKNGGTCETTSLIGKATCKCAPGFTGEDCQYSESHICYVSQPCLNGGTCHPHSQ
ETYECVCPPGYTGKDCQWIDACTSQPCANGSTCTVSGHKFSCICQSGYTGQKCEIDVNECATSGLCHHGGTCINLPGSFRCQCKPGYTGHRCESNYVPCSPSPCMNGGTCHQTSDFTFECNCLPGFKGS
ICEKNVDDCPNHNCQNGGICVDGVNTYNCRCPPQWTGQFCTEDVDECQLQPNACQNGGTCTNHNGGYACVCVNGWSGDDCSKNIDDCFTASCANGSTCIDRVASFSCICPEGKAGLLCHLDDACVSNPC
QKGALCDTNPVNGHYICTCPQGHKGADCTEDVDECAMANSNPCEHAGKCVNTEGSFHCECLKGYTGPRCEMDINECHSNPCQNDATCLDKIGGFTCLCMPGFKGVHCEEDIDECLSNPCVNNGECLDKV
NRFLCVCPPGFSGAVCQIDIDDCSSTPCLNGAKCIDHPNGYECQCATGFTGVLCEENINNCDPDPCHHGECQDGIDSYTCICNPGYMGAICSEQINECHSNPCLHQGRCIDLVNGYQCNCLPGTSGVNC
ENNFDDCASNPCVHGDCIDGINRYNCACKPGFTGPRCNVDIDECASSPCNNGGTCINEVNGFRCVCPEGYHHPHCQSQADGCLSNPCVHGNCTHIVSGYKCVCDPGWIGDYCSTEGNECKSNPCQNGGT
CEDLLNGYRCTCRKGFKGVNCQVVVAPCSPDPCENSGICQESPDSEGYTCQCAPGWEGERCTVDIDECLSKPCKNHALCHNIQGSYLCECRPGFTGGDCDSNIDDCLSNPCQNGASCVDGINSFSCICL
PGFHGDKCQTDTNECLSEPCRNGGTCTHYVNSYTCKCQPGFEGTNCENNIDECTESSCFNGGTCVDGINSFTCQCPVGFTGPFCLMEINECDSHPCLNKGSCVDSLGKYRCICPLGYTGKNCQTPMDLC
SKSPCKNKGTCFQSGAQTRCDCPSGWTGAYCDVPNVSCQVAASQRGITVDQLCQHSSHCLNVGNTHRCQCRVGYTGSYCEEQLDECDSSPCQNGATCRDHLGGYQCECVPGYQGVNCEYEVDECQFQPC
QNGGTCIDLVNHFRCSCPPGTRGRLCEENVDDCISESGGPRCFNGGQCIDQIGGYSCLCLPGFAGERCEGDINECLSNPCNPRGSLDCIQLINDYTCICRSAFTGRHCESVIDVCPRKPCQNGGTCAVA
SNMPDGFICQCPPGYSGAKCEFSSHSTCGQVKCKKGEQCIHTSSGPRCYCPRLAVGEECQTNTGCASAPCQNGGSCHPRSQPPYYYCQCPAHAQGSQCEQLVHPSQEPLGCLDSQCAEKARDGYCDEDC

NTHACQWDGGDCSLTMEDPWANCSSSLRCWMLFNGQCDEFCNTPECLFDNFECQQNSRMCKYDKYCADHYGDGRCDQGCNSEECGWDGLDCAGDKAERLAEGTLIIVVLMRPDELLRDVRSFLRTLGTL
LHTNLRIKLDSQGNPMVFPYYGEKSAARSRRSVVVVRKHRELEQEVIGTRVFLEIDNRQCAEDSEQCFHNTEAAAALLAAQAIKGMLPYPFVSVQSEPLLPPKTQLLYLLAVAALIILLILLLGVMMAK
RKRKHGSLWLPEGFILRRDPSNHKRREPVGEDAVGLKNLSVQIPEGNMADSGPTEHWAGDGGPQPKRVKTEDQALLPEADEQIDQRQWTQQHLEAADVCGSTSLALTPPQADQEVDVLDVNVRGPDGCT
PLMLASLRVGGSDISEDDEDGEDSSANIITDLIYQGANLQAQTDRTGEMALHLAARYSRADAAKRLLDAGADANARDNMGRTPLHAAVAADAQGVFQILIRNRVTDLDARMNDGTTPLILAARLAVEGM
VAELINCQADVNAVDDHGKSALHWAAAVNNVEATLVLLKNGANRDMQDNKEETPLFLAAREGSFEAAKILLDHFANRDITDHMDRLPRDVAQDRMHHDIVQLLNEYNVAHSPTGHPGAMLNSALSPVIC
GPNRSFLNLKHASLSKKSRKPNAKGIMPTNLTKDAKRRRKKSLSESKGQFSESSVTLSPVDSLESPHAFASEPTSSPVMPSPGVLHSSPSSLLTAPSVQAAHSMSFSNLHEMQPLGRGSSTVLPSVSQL
LSQHRTTPPSSGLGRLRPANVSTEWMNRMEMNESQYNEMFGMVPPVMHSHPSVSQQSGLVQADTKHLGVSRESLPPIMTFQLVPKGGINQQALPQQAQSNCAQNMAGPLSSMYQISDLAQLPSASFSMA
AIPQQDGQVPQTILPAYHQFQSSMGKYPTPPSQHSCYSSATERTPSHNRHLQGEHPYLTPSPESPDQWSSSSPHSASDWSDVATSPGNNQRGPAAAHVPEQQRNNMQVYA

Xenopus laevis:

>XP_018114267.1 neurogenic locus notch homolog protein 2 isoform X1 [Xenopus laevis]
MGVQAVFGLCVLLLGMPCCDGLQCRGASEPCLNEGRCITSDDGSAYCKCPDAFVGEYCQLQNPCQETVCLNGGSCKVSPDLSKGVCTCAPGFSGENCKQPVASVCSLTQPCMNGGTCRLLTLEHYECIC
PFGWRGKDCEFIDACASQPCANGSTCLPNENQYSCTCQAGYTGLKCEMDVNECTSPGLCKNGGTCENLPGSYRCQCHPGYIGQRCESPFIPCSPSPCMNGGTCRQTSEFAYECNCLPGFDGKNCESNID
DCPSHKCQNGGTCVDGVNTYNCRCPPQWTGQFCTEDVDECLLQPNACQNGGTCTNSNGGYNCVCVNGWSGDDCSQNIDDCATAACANGSTCIDRVASFICVCPEGKIGLLCHKNDACFSNPCHIGSLCD
TNPLTGQAICTCPPGYKGTACTEDIDECSLVNSNPCEHAGKCVNTEGSFYCECSKGFTGPRCEMDINECLSEPCQNDATCLDKIGGFTCLCMPGYKGIHCEIEVDECLSNPCVNNGVCVDKVNRFVCIC
PPGFTGPVCQIDIDDCSSTPCQNGAKCVDHPAGYDCLCATGFTGVLCEENINNCEPPPCHYGTCQDGIDSYTCICEPGYMGAICSDQIDECHSNPCLNEGRCVDLVNGYQCNCLPGTSGPRCEKNVDDC
ASNPCTYGNCVDGINRYDCVCSPGFTGPQCKDDIDECASSPCHNGGTCVNEQNRFRCICPEGFRQPSCFSQVDECVNNPCIHGNCTDDVNGYKCLCQPGWNGKKCEVDKNECLSDPCQNGGTCDDLVNG
YRCSCKKGFKGYNCQIDTNECASNPCLNHGTCRDGINGYTCHCALPYTGTNCQNILTPCSPEPCHNGGICQESEDFKSFSCLCATGWQGQRCTIDVDECVTNPCRNYGRCQNTKGGYKCHCPDGFSGFN
CENNIDDCLSNPCQNGASCLDGINTFSCNCLAGFHGDKCQRDVDECASNPCKNGGSCTDYVNSYTCKCQPGFDGIHCDNNIDECTDTSCFNGATCVDGVNSFSCQCPQGFTGPFCLFEINECGSHPCIN
GGTCVDGMGTYHCTCPIGYRGKNCEELVDLCNGFPCKNKGICKQVKTEPKCICPTGWTGIYCDIPDVSCEVAASQRGVAVQDLCQHAGVCINTGFSHRCQCRQGYIGSYCEGELDECASNPCQNSAACV
DRHGGYECKCLPGYQGVNCEYEIDECQLQPCHNGGTCVDLVNQFRCSCPPGTRGALCEENIDDCAPSAEGPHCFNGGRCIDRIGGYRCECLPGFAGERCEGDVNECLSSPCSSKGSLDCIQLANNYLCK
CKPVFTGRHCETIQDLCPSRPCLNGGTCAVAVNMPEGFTCQCPSGFLGSKCQYSNNTCEHYKCKVGENCIQTHMGPRCYCPMGPAGYACQSNLGCASQPCKNNGVCQRKAQPPFYTCHCPKGVVGPQCE
IPIETDRCHKYQCPEKARDGYCDQECNIHECLWDGGDCSLTMGDPWANCSSSLRCWEYFNGQCDELCNTPECLFDNFECQQKEPICKYDKYCEDHYANGHCDKSCNTKECGWDGLDCSTDRPENLAEGT
LVVVVLMPPSELLKNSRNFLRVLGSILRTNLRIKKNPDGSYMVFPYFRENASSQRSRRSLLKLRVIRELEQPIGSKVSLEINNQECVMDSDQCFKTSESAAALLAAHAVRGTLNYPLVSVEVENAPMSH
QTLMYILPVTLAIIIVILLMVVVLAKKKRKHGALWFPEGFFLHRENSNQKRREPVGQDAVGMKNLSVPIVDVNLLDSNQNDHWSDDRGPQPKKSKSEGQALLLGGDDQVDSRQWTQQHLVAADIRMTPS
LALTPPQAEQDIDVIDVNVRGPDGCTPLMLASLRMGSPHLEEDDDEAEDPSANVITDLIYQGACLQAQTDRTGETALHLAARYSRADAAKRLLDAGADANSQDSMGRTPLHAAVSADAQGVFQILIRNR
VTDLDARMNDGTTPLILAARLAVEGMVADLINCQADVNAVDDHGKSALHWAAAVNNVEATMVLLKNGANRDMQDNKEETPLFLAAREGSFEAAKMLLDYFANREITDHMDRLPRDIAKDRMHHDIVRLL
DEYNLVHSTQGQPGSMLSNSLSPAVCGSNRSFLNLKHAAQAKKTRRPSAKNVGPRSLPNLAKEPKDTKNRRRKKSDTDNKGQLSESSVTLSPVDSLESPHATSSPTITSPGTLHTSPGQMHATSTLQPP
HTTMSMTNLQSIGCAANNMLPSVGQLLSQAAQNNVLAGQRQLNLSSDWLSRMERNNTQYNEMFGLVHHDTNNHSCIQQNGIMQADIKPTLHMGVARDALPPIMTFQLIPKSGMNQQTLVQQTSHAQNVA
GMYQMPDRARITGSSVPIAMMHQHDGQLSQTMLTSYHPLQNPMGKYPTPPSQHSYASTDKTPNHNGHLPGEHPYLTPSPESPDQWSSSSPNSASDWSDVTTSPTPNGSQRMPATHMPEQSHNGMQVYA

Salmo salar:

>XP_014025097.1 neurogenic locus notch homolog protein 2-like isoform X3 [Salmo salar]
MGQLPVFSSGKVLVIVVCCVRLSLALQCLDAVKPCVNNATCATFTNGTGYCRCAAGFLGDYCHHKDPCHPGYCLNGGNCSVAVSGIPGSPTCSCPLGYTGQHCQTPQNSTCYPNNPCANQGVCTLLSLD
KYKCQCAHGWTGMRCNREDSCLSGPCANGGTCSSPPGGKFGCTCPPGYLGPRCLNDTDECAASAPVCQNQGRCVNTPGSYRCNCAPGFTGRQCETPYIPCSPSPCLNGGTCRPTSETSYWCHCLPGFNG
TNCENNIDDCPDHQCRNGGTCMDGVNTYNCQCPPEWTGQFCTEDVDECRLQPNTCQNGGTCSNIQGSYTCVCVNGWSGLDCSENIDDCATAACTKGSTCIDRVASFLCVCPYGKTGLLCHVNDACISSP
CRDGAQCDTNPINGMFNCNCAPGYIGSTCNDDVNECIIGPNPCEHGGSCVNTDGSFTCNCARGYDGPRCETDINECASSPCQNDGTCLDRIGDYSCICMEGFEGTHCEIDINECASSPCLNQGTCLDQV
KRYVCQCPLGFSGEMCQIDIDECSSTPCLNGAKCIDRPNGYECECAEGFSGPLCKENIDDCDPEPCHHGVCRDGIATFSCDCDPGYTGSICNVQVMECHSNPCQNRGRCIDLVNKYQCNCLPGTSGVNC
EFNVDDCASNPCEYGECQDGINEYKCVCAPGYTGAKCDVDIHECNSSPCMSGGTCVDKVNGFICQCPPGTHGPLCHSGTDHCAPQPCVHGDCVEQQSGYNCECDSGWVGQHCDQEKDECQSSPCQHSGS
CVDRLNGYSCQCRPGFTGVNCEFNMEECASSPCENHGTCVDGVNTYSCLCDPPYSGKHCEEELVPCASHPCERGGVCQPTPDYTFYTCRCPRGWQGPRCTEDVDECRKNPCQNRARCINSQGSYVCKCR
PGYSGLNCQTNIDDCSPNPCLNGGSCVDEVGGFSCDCRQGFDGERCEAEVDECASQPCQNGAVCRDYVNSFVCVCRPGFDGILCEHNIPECTESSCLNNGTCVDDINTFSCRCRPGFYGTFCEYEQNEC
DSQPCKNGGTCTDGLGSYRCTCPVGYNGQNCQNFVNLCSQPLCQNGGSCSQSETTWLCHCPVGWTGMYCDVPNMSCQDYAARNGIQVELVCKHSGRCVNVGNAHQCQCLPGYTGSYCNEMVDECLSNPC
RNGATCMDYQGTYECMCKAGYQGVNCEYDVDECHSNPCHHGGTCINLINRFSCACPPGTNGVQCEVNVDDCAPKPGSWELRCLNGGQCVDGVGRYTCSCPPGFAGEHCEGDVNECLSGPCHSPGSLDCV
QLANDYQCRCRLGYTGRHCESMVDLCQSKPCHNSGTCSMNMSSVHGYTCICQPGFTGFNCGEVEGYNCAKLRCQNGGHCQETQVGRPHCRCQPGFSGPRCETVHSCQNRPCLNGGTCMKDPHYQYSCHC
PAHFSGRHCENVIFDPSPSTPASCPYVECEQHSGDKVCDPQCNSHECQWDGGDCSLHWRQPWVNCTAPVPCWELFRNGRCDPDCDNSGCLFDSFECQESAQSYCKYDKYCAAHYANKICDKGCNTEACG
WDGLDCSGDTPAEVADGTLVIVVLLQPEELMGDMRGFLRSLGTLLHTNLRVKMDDQQKPMLYPYYGLEHDDGNEGQPSTMTLRGKRELDKEVIGSKVYLEIDNRKCSERSMDCFSSTELAASFIAAEYL
KSELPYPVVSVNSDPTEPYKPNFLLYLAVGAAVIILLILVLGVLAAKRKRKHGVLWLPDGFLAKKDDKRREPVGQDDFGMKNFKTQDGGMIDGGQRWMEEEAPPKKVRTEDKPLLPMGVDGGVDRREWT
LQHHKAADISLTPPQTDLEADCLDVNVKGPDGFTPLMLASLRNGGASDCGLQAEEEEESGGDEPCPNAISDLITQGATLMAQTDRTGETALHLAARYARADAAKRLLDAGADANAHDNMGRTPLHAAVA
ADAQGVFQILIRNRATELDARMNDGTTPLILAARLAVEGMVEELVHCHADINAVDDHGKSALHWAAAVNNVEATLVLLKNGANRDMQDNKEQTPLFLAAREGSFEAAQVLLDHYSNRDITDHLDRLPRD
TAQERMHHDIVRLLDQYNLVHSPHNGPNHMGGGHSSLVCGGNGAGYMGMRPGPQGKKSRRGGAKVGGVGGATAKELKDMKAKRRKKPTGGEGPAVGGASGNGTKAAGGLLESSVTMSPVDSLESPHLYI
GDAAGASKTTNSPLLGSPSSRPLLPPVSHMLGQQQSWVGLKHGYGGHMFSLLPQQMGSGHPSMSQHHGQGLLTPMNVTMSREQLPPIVTFQMMAPGGGQALLKQPQPGQVQSQGQNQGQPQSQQGPTHL
HCPQGMLYQMPNVSLQHSLSHSLPHPHGLAHGMVQDGQSRQQLPPYQAMQSPVDKYPTPPSQHSYATAGSEGTTPGYPAHPPSEHPYLTPSPESPDPWSSSSPHSNSDWSDVTTSPTPLGNPHALPPLH
HTHIPEQLQPQSQQTQQNPQQPQRGNMQVFA

Petromyzon marinus:

>XP_032815028.1 neurogenic locus notch homolog protein 1-like isoform X1 [Petromyzon marinus]
MEPRALLPLLRLLLLLLLALGARLSRGLQCQDSNQPCINGGTCETFANGTGICKCQHNFVGIYCQLDNRCASSPCQNGGTCSTVVRGGVPDYACECPLGFMDPVCLTPADNACLGVPCHNGGTCRLLSL
HRFECHCLLGWSGKTCEKADPCASNPCAHGGSCRAVGSRYACACRPGFSGPMCRADVDECAASPCRNGGSCANVPGSYACACPAAYTGRHCETAYVPCAPSPCLNGGTCRQTGDTAYDCSCLPGFEGRD
CDVNVDDCPGNRCDNGATCVDGVNTYNCQCSPQWTGQYCTEDVDECQLQPNACLNGGTCHNTHGGYNCVCVNGWTGEDCSENIDDCAIAACFNGATCHDRVASFFCQCPLGKTGLLCHLDDACVSDPCH
EGAICDTNPINGKAICTCRAGFTGGACNQDVDECSIGANPCEHGGRCINTQGSFRCQCTAGFTGPRCETDINECNSMPCLNDATCLDKIGEFTCICMPGFEGRHCEIDINECQSGPCLNDGNCTDHVNG
FLCRCPPGFTGPMCQIDMDECASTPCLNGAKCVDRPNAYSCECTEGFAGLVCELNVNECQPDPCHHGKCVDGIASFYCVCSPGYTGFLCDSQLNECQSSPCQNGGKCVDLVNRYHCQCLEGTEGVNCET
NFDDCAGELCQRGKCVDGVNEYRCLCDAGYTGLQCDVELNECESSPCHNGGSCKDLVDAFECVCLPGFHGPLCYSETDACYASPCLHGTCLDHGESYRCACEAGWTGPLCEVDIDECESSPCQNGGACQ
EHLDGYTCRCRDGFKGPTCQKNVNECVSNPCLNRGTCVDGVAGYTCLCTLPFTGRNCEAVMAPCASQPCHNAATCKESVNLSSFSCACPPGWRGATCSLDIDECAGAPCQHGGACMNTQGGFRCSCRPG
YTGPTCQTDIDDCSPNPCQNGGSCTDGVGAFSCKCRPGFRGSRCETEVNECASNPCRNGGSCADYVNSYTCRCRPGFNGIHCEHDITECTDSSCLNGGSCVDGINAYTCLCRGGFTGSYCQFDIDECES
QPCQHGGTCLDGLGTYKCTCPRGYTGGNCQALVDWCSSSPCKNSGSCVQSRTSYRCDCHSGWTGLYCDIPNMSCDTAARNKGLSVSHLCRHGGRCIDAGNTHHCQCSRGFEGSYCEKEVDECTSNPCHN
GATCRNFLGSYKCECPVGYHGLNCDYEINECLSQPCQNGGKCIDLVNRFNCVCPPGTQGILCEINTDDCNPGPDAPMPGPRCFNGGSCVDGVGSFTCSCPPGFVGERCEGDVNECLSNPCDPRGSLDCI
QLVNDYKCQCRPGFTGHNCEVVINTCRLEPCRNGGTCMITVNTLLGFTCLCAPGYAGHACEDNAYACGNLSCRNGGTCLPDRAAGPLCVCPRAWRGPECQYHSGGGGGSSACASSPCENGAACYEEVRP
PFYRCVCPPLFTGPRCRVLGPKVPGDASSGRALPVPPPVLEESCPVAECESHAGDGLCDRDCNNHACAWDGGDCSLHFPSPWRNCSVRLQCWLRFRNGECDEQCNSLGCLFDGFDCQLGMKNCNPLYNA
YCTAHYADQHCDQGCNSDECGWDGGDCAGEVPERLADGVLVLIMLLPPARVHNDSANFLRQLGSLLHATVRFKIDTNGQDMIFPYYGDEARGGGGPRLERAARSLSRFVREMERPLIGSKVYVEIDNRH
CYQTSDRCFSSAADAAAFLAAQATHGSLPLPLLGVSSEVPDVVTAPRVQVYVLLGLVLLVLMVVVLLGVLVGRKRRREQGTLWFPEGFILGPPSDKKKRREPVGQDDVGMKTLSQSSVEDSLIDGNQNE
AWLDGSVPSTKRRKLEEQGMLPEAEEPVDHRPWTQQHLEAADIRTAPALVLALTPPQGEADDGMDVNVRGPNGFTPLMVASFHGGGLDSSLCDEEESSDGLFEDGTDDGSANVITDLICQGAALHAQTD
RTGETALHLAARYARSDAAKRLLDAGADANLPDNMGRSPLHAAVSADAQGVFQILIRNRATDVDARMHDGTTPLILAARLAVEGMAEELINCHADVNKVDDLGKSALHWAAAVNNVEATLVLLKNGANK
DMQDNKEETPLFLAAREGSYETAKLLLDHFASRDITDHVDRLPRDIAQERLHHDIVRLLDEYNVVRNPSGPNGLASPPQANGAYAHGAKAGKKGVRRPGNKSGVAHDAAKCKKAQKKVNGGVASGGGGD
GAPLLGLDGSASLSPVNSESSQSDGTSPSLLTSPHVAHSLYASMEAVGCGPPRVGHPMAFRNGAIAVASGWINPMQNVPLSQFTHQGHSLGLTSSHPQSNHRAMMSPLHPAMINNQHHQMLAFQQNLYS
PRNKPLMEAPQHHPEQARGGAFFQSKMPMIQPQSNLQAASQQSQLLASVSQSNMFPSLQQQHQGIAVPMQSFVGSEFGESFASNDYKPNEKSHSQTSCVLTQTVPPPQEANPSVQSSQLAKREGAAPAE
QFPTPPSQHSRGEGSPGHAPPPGTPGEHPYLTPSPETPERWSTCSPRSGASDWSDGVSSPPGLAARAQRRPADPQQQLHNLQALM

Branchiostoma belcheri:

>XP_019643529.1 PREDICTED: neurogenic locus notch homolog protein 1-like [Branchiostoma belcheri]
MRSLFAVVLLLAAAGPALVQGLVCQANNSPCLNGGICETDSSGQGSCVCTDGWVGEYCQEVDPCASNPCRHGGTCVVHDNATWSCDCPLGYRDMICLTVVDNPCVTNPCNNGGTCELITIDEYRCNCQP
GYTGDTCDVVEHCYSQPCKNGGTCTSTESGYTCACLGGFEGSTCQTDIDECAGTNPCQSGGQCQNTMGSFTCTCPKERTGRLCEEEYIPCSPSPCQHGGTCESVDTYEYTCHCMSGFTGDDCEVNVDDC
VDHLCENGAACVDGVNEYTCTCPSEWQGRYCNEDVDECMQSPNICLNSGTCHNTVGGYSCVCVNGWIGDDCSENFDDCATAACFHGATCHDRVGFFMCECAPGKTGLLCHLDDACESSPCNEGAICDTN
PVNGQPICTCPDGYAGQLCMQDIDECSLGENPCEHDGECNNVPGSFTCTCTDGFTGDRCEVNINECASNPCQNQGTCIDDIGEFRCACMPGFAGDLCETDVDECASSPCLNGICRDGINKYECECDPGY
EGTTCEVNINECASGPCRNGAHCTDLVTAYTCTCLEGFTGTDCEINIDDCQSNQCQHGTCVDEVASFTCSCEPGYNGPLCESPVDECDSDPCQNGGTCEDLVNGYRCNCLPGTSGDNCEVNQDDCTGNL
CVHGVCQDGLNTYTCMCDGGYEGENCESEIDECASSPCHNGGICHDLVNAFSCECPPGYHDLLCYSNVNECESSPCEHGTCQDGINDYTCTCETGYEGKNCDVNIDECASNPCQHEGQCDDGIGRYECQ
CLPGYEGVNCQINTDECGSNPCQNGGQCLDGVNNYQCHCELPFVGINCQIELAPCNPNPCANSGSCIPSADYQTFTCDCAEGFEGETCADDINECQSNPCKNGAPCINLEGDFRCDCLTGFSGELCSVN
IDDCDPDPCHNGGTCNDGINSYTCSCMPGFGGTNCEEDIDECYSNPCQNGGQCIDAVNGYACDCVLGFIGTNCQTNKDDCTSSSCFSGGTCIDGINTFTCQCPPGFTGSNCQHEINECESHPCQNGATC
VDQTGYFSCICTYGYEGVTCQNQKDLCASDPCRNGGTCTQSGDRYECLCEDEWTGLICDMTKVSCAAAASERGVLLGELCQNGGSCVDTGNSHNCNCPAGYRGSYCSEEIDECASSPCQNGAQCRDGLG
TYSCVCRAGYQGVNCEQEINECISNPCQNGGTCIDMVNEYRCSCPPGTQGLLCEINNDNCFAGACYHDGTCVDGIGEFTCRCRPGYVGPRCEGDVNECLSNPCDAEGTQDCVQLENDYRCDCKPGYTGG

RCERTVDSCESDPCLNGGACSQSGNNYVCDCQPGFGGDNCELVCDRECKNRGQCLYTDGEFRCSCPSNYAGDSCQFHESNPCFSLPCYNNGECQQTGIDSYRCMCPEDYNGLVCEIYIPDVTPGPGVTM
IPPHIEPSCPFEHCASKARDGRCDEECNIHECDWDGTDCALGNNPWANCTSSLQCWNYFSNGHCDQQCNNEECLYDGRDCEENPHPTCNPVYDTYCGNFFRDGNCDSGCNNLECGYDGGDCDEDYPPVT
AKGYLVMIVAAPPEVLLNNSISFLRMLSIILRSSMSFKVDDEGNPMVYPWYGEEVDETISKRAIRSVFRALSGRYRRATESEVQGSIVYLEMDNSKCYRVHDDCFETAKEAADFLAALFLHNGLSQLNY
PVKSLYSGDDPEPPSTPTFNPLYVVVAAVGILLVMVGLGVLMTTKRRRESGQLWYPEGFKLTKESRKDARRSTRRRDPVGQEVGMKSFTPQGATAANVDDNDNETVRDSSAWEDDMVPPEAKRMKTQQP
TPGSDATADQTLSDYDDTDHRQWTQQHLHAADIRNPACLAQVALTPPQTEGEADPCSVDVNVRGPDGLTPLMLASFRGGGLEYVEDHQEDGEDESANVITDLLMQGANINAQTDRTGETSLHLAARYAR
ADRAKRLLDAGADANARDNTGRTPLHAAIASDAQGVFQILLRNRATDLDARTNDGTTPLILAARLAVEGVVEELLNSHADANACDDNGKSALHWAAATNNEEAVISLLSHGVNKDVQDNKEETPLFLAA
REGSFQAAKILLDHYANRDITDHMDRLPRDIAQERMHSDIVKLLDEYNLVRSPSQAAANCQNSFGGHHFMNGPTTKQQRAKPKRPNKGMQHHTIARADASKMRRKKKLEGTGTMPRHMDSTALSPDGSL
GGSPHGYNTGETPPPNSMYGVGSQHLALSHPNLHGVDTVPHTTSTPQIVNSFSFTNGQPMPRLSHHTADSNSHQDQSTLRGGETGLGLHLDPPVSVPSDWLASLDSRNHHYTQFSPRMHMTHAGLNHMP
QQSQSPPSGLLSPPHSHGSITGGSPAQMAAFQALSSSNAKQQQLAHQSNHHNNHRMYHDQHGNAHGGSPLGGGGRMHDPGIPLNVIPPGELPPHSHQTVLPPGSCAQQFSSNNPAEQLPTPPSQHSHGN
PQSHECTPTHYLSHAETIVPENYLTPSPDSPDQWSSSSPHSATSDWSDGISSPPAAQSQRQPYIPETHNTRQGGHSALPVFI

Drosophila melanogaster:

>NP_001245510.1 notch, isoform B [Drosophila melanogaster]
MQSQRSRRRSRAPNTWICFWINKMHAVASLPASLPLLLLTLAFANLPNTVRGTDTALVAASCTSVGCQNGGTCVTQLNGKTYCACDSHYVGDYCEHRNPCNSMRCQNGGTCQVTFRNGRPGISCKCPLG
FDESLCEIAVPNACDHVTCLNGGTCQLKTLEEYTCACANGYTGERCETKNLCASSPCRNGATCTALAGSSSFTCSCPPGFTGDTCSYDIEECQSNPCKYGGTCVNTHGSYQCMCPTGYTGKDCDTKYKP
CSPSPCQNGGICRSNGLSYECKCPKGFEGKNCEQNYDDCLGHLCQNGGTCIDGISDYTCRCPPNFTGRFCQDDVDECAQRDHPVCQNGATCTNTHGSYSCICVNGWAGLDCSNNTDDCKQAACFYGATC
IDGVGSFYCQCTKGKTGLLCHLDDACTSNPCHADAICDTSPINGSYACSCATGYKGVDCSEDIDECDQGSPCEHNGICVNTPGSYRCNCSQGFTGPRCETNINECESHPCQNEGSCLDDPGTFRCVCMP
GFTGTQCEIDIDECQSNPCLNDGTCHDKINGFKCSCALGFTGARCQINIDDCQSQPCRNRGICHDSIAGYSCECPPGYTGTSCEININDCDSNPCHRGKCIDDVNSFKCLCDPGYTGYICQKQINECES
NPCQFDGHCQDRVGSYYCQCQAGTSGKNCEVNVNECHSNPCNNGATCIDGINSYKCQCVPGFTGQHCEKNVDECISSPCANNGVCIDQVNGYKCECPRGFYDAHCLSDVDECASNPCVNEGRCEDGINE
FICHCPPGYTGKRCELDIDECSSNPCQHGGTCYDKLNAFSCQCMPGYTGQKCETNIDDCVTNPCGNGGTCIDKVNGYKCVCKVPFTGRDCESKMDPCASNRCKNEAKCTPSSNFLDFSCTCKLGYTGRY
CDEDIDECSLSSPCRNGASCLNVPGSYRCLCTKGYEGRDCAINTDDCASFPCQNGGTCLDGIGDYSCLCVDGFDGKHCETDINECLSQPCQNGATCSQYVNSYTCTCPLGFSGINCQTNDEDCTESSCL
NGGSCIDGINGYNCSCLAGYSGANCQYKLNKCDSNPCLNGATCHEQNNEYTCHCPSGFTGKQCSEYVDWCGQSPCENGATCSQMKHQFSCKCSAGWTGKLCDVQTISCQDAADRKGLSLRQLCNNGTCK
DYGNSHVCYCSQGYAGSYCQKEIDECQSQPCQNGGTCRDLIGAYECQCRQGFQGQNCELNIDDCAPNPCQNGGTCHDRVMNFSCSCPPGTMGIICEINKDDCKPGACHNNGSCIDRVGGFECVCQPGFV
GARCEGDINECLSNPCSNAGTLDCVQLVNNYHCNCRPGHMGRHCEHKVDFCAQSPCQNGGNCNIRQSGHHCICNNGFYGKNCELSGQDCDSNPCRVGNCVVADEGFGYRCECPRGTLGEHCEIDTLDEC
SPNPCAQGAACEDLLGDYECLCPSKWKGKRCDIYDANYPGWNGGSGSGNDRYAADLEQQRAMCDKRGCTEKQGNGICDSDCNTYACNFDGNDCSLGINPWANCTANECWNKFKNGKCNEECNNAACHYD
GHDCERKLKSCDSLFDAYCQKHYGDGFCDYGCNNAECSWDGLDCENKTQSPVLAEGAMSVVMLMNVEAFREIQAQFLRNMSHMLRTTVRLKKDALGHDIIINWKDNVRVPEIEDTDFARKNKILYTQQV
HQTGIQIYLEIDNRKCTECFTHAVEAAEFLAATAAKHQLRNDFQIHSVRGIKNPGDEDNGEPPANVKYVITGIILVIIALAFFGMVLSTQRKRAHGVTWFPEGFRAPAAVMSRRRRDPHGQEMRNLNKQ
VAMQSQGVGQPGAHWSDDESDMPLPKRQRSDPVSGVGLGNNGGYASDHTMVSEYEEADQRVWSQAHLDVVDVRAIMTPPAHQDGGKHDVDARGPCGLTPLMIAAVRGGGLDTGEDIENNEDSTAQVISD
LLAQGAELNATMDKTGETSLHLAARFARADAAKRLLDAGADANCQDNTGRTPLHAAVAADAMGVFQILLRNRATNLNARMHDGTTPLILAARLAIEGMVEDLITADADINAADNSGKTALHWAAAVNNT
EAVNILLMHHANRDAQDDKDETPLFLAAREGSYEACKALLDNFANREITDHMDRLPRDVASERLHHDIVRLLDEHVPRSPQMLSMTPQAMIGSPPPGQQQPQLITQPTVISAGNGGNNGNGNASGKQSN
QTAKQKAAKKAKLIEGSPDNGLDATGSLRRKASSKKTSAASKKAANLNGLNPGQLTGGVSGVPGVPPTNSAAQAAAAAAAAVAAMSHELEGSPVGVGMGGNLPSPYDTSSMYSNAMAAPLANGNPNTGA
KQPPSYEDCIKNAQSMQSLQGNGLDMIKLDNYAYSMGSPFQQELLNGQGLGMNGNGQRNGVGPGVLPGGLCGMGGLSGAGNGNSHEQGLSPPYSNQSPPHSVQSSLALSPHAYLGSPSPAKSRPSLPTS
PTHIQAMRHATQQKQFGGSNLNSLLGGANGGGVVGGGGGGGGVGQGPQNSPVSLGIISPTGSDMGIMLAPPQSSKNSAIMQTISPQQQQQQQQQQQQHQQQQQQQQQQQQQQQQQLGGLEFGSAGLD
LNGFCGSPDSFHSGQMNPPSIQSSMSGSSPSTNMLSPSSQHNQQAFYQYLTPSSQHSGGHTPQHLVQTLDSYPTPSPESPGHWSSSSPRSNSDWSEGVQSPAANNLYISGGHQANKGSEAIYI

Hydra vulgaris:

>XP_012554603.1 PREDICTED: uncharacterized protein LOC100208285 [Hydra vulgaris]
MVFYMLLLFHVLWMVACNTNVKGYVDSKQNNEFYAANYKCPNGWQQRCEKCYLYVNESKQWTDAQYYCKQNGGNLVTIKEDAVNLFLARYVQYSSRLAITIWTGLHARKVHKEFQWVDNSESVHYEFRW
LPGQPNMVTLDEGCVEMYTKSRQYLWDDVSCSKESPFVCQTDGCIIGTQRCDNGKCVSKHLFCDSKNDCGDFSDEKNCTKCNEHYTAQEGSISSPNFPQHYGNNRDCTWLIEVESSYIIEIKFLEFFIE
NSFDWVEVFDGPTLASKSFGKFSGNFKPEVILSSSSKILVHLKSDKSTSDKGFFARYKAVKKGCNVDLIINSNKPEYISSPNFPLNYPDNTKCEWTIRNEDPSKILTFQLLELETEENDFIEVRDGLTE
DNALLGKFFGNFSNIQKRHIFTSNFKMHVKFQSNFQLNQKGFRAVIFSGCTATINATHGTISSPNFGVGNYPNNILCSWTVHAQTDKEVSVIFHSFDTEYKQDTVVLRTCDDVMIKECYGSNKTTLGKE
MLPLEPIVFNSSCFKVIFKSDETYSLEGFNATFSIGCPMLPAINGGSFRQDQKFYGASVEYKCDSRFRIKSENGNVRKCMYGGNWSEPAPFCEEINCGHPGTIENGGFEIYPLNSTFVFMTSVKYSCNV
GYILVGEPNRVCQVNSTWSGGVERKCEVLKCPDPGTPVNGTRSGNEFNVGSSITFSCEKGFRRHGARTITCGITGKWSSETPLCKVVVCQLPSFSLNTIISSSSIGFNTPYKTVLHISCKKGFELIGSV
NITCTESGAWKPDVPQCIDINECLNNPCRGLGSQCTNLMGSYKCTCKSGFRQIDETTCEDIDECKSSGKTVKCNQKCTNIDGGYFCSCEDGYFLYDGDNKTVNRVEAEESFVNHTCLGNPCKLPKIPEN
GYILNYGNRYPTEITFQCDKGWIVDGDLSIKCLKNGSWSGSISDCIAARCNPLPDVPNSSSVVDGGLVKGQYSQGTIYTYTCINGYEIISGNSKRMCSQNATASFWSGSSPVCERVSCGPLENPKNGHV
NITGDKYGDSSKFFCDGGYIITGNDSSTCQANGRWSASSPFCLASPCPDPGIPINGEIIGLISLDQTITFRCLVNGSILIGDNKRTCIFNKSTGLNQWNGSQPFCKDTSTPTFKFCPMSTIEFTLPQSE
KSMMVNWTLPTLTDNVHNLLNITVSPDYVSPMVMPAGLNIITYTATYKDGSTAVCEVKINVQDKEIPKIFCPKNIVIKSDLSSKVFWSDGIYTDNVGIKNVIFNPPNGTILESNKYHKVTMTVEDISGN
SDSCVMEIYVEGCGCTQRTCDYKQRNGQSVCTIAPGVVPDTVIISCNLNCFQNKTTRFVSNTEFEKTSNVMCIPNKGWENKSPSGCVERKPLISCLKGSLSIDFTTTGDNTNEKPFLDIFTFLNNLKNS
TCSFIYQTGNLISNGTSVAGDFKILSHDDGVIPLLNGHLEECKTILLKDLDLFKTNLLQLTSSVDIKLLNISSCCPLNKSYCCPTGHLIKTTGLTVKASYCIPCGLGMYHNSTNNKCLNCPVGFYNDLE
FADQCKRCPNETNNLKVQSDSCTKMCAAGSYSSTGLEPTCRKCDYGEYQSQVGAYNCIKCPYNLTTSRSGSENISECKAQCSPGTYSSTGLEPCTKCAKGLFQNEIGRSYCKLCPSGGIYLGMEGATSE
ANCPSKNACDEKPCRNGATCNAGNYSYTCTCKPGWTGVNCEVDIDECASSPCGRHGTCTNLINDFNCTCIKGIIGKQCETNIDDCKNDTCRNGGICNDLVDDFKCLCAAGFEGKRCEINKNECEPNPCL
KNSTCEDLINDFKCNCIPGYVGKLCDVDIDECAMSPCFNNATCVDKVNAFECKCQPGFNGTLCETDIDECSTQPCANNGSCIDIVNGFLCKCIDGFRGSNCSINIDECDPSPCLHNSTCVDQINGFQCE
CSPGYFGLRCETEINECESQPCSNNATCVDKINDYQCLCNIGFEGKQCEIDINECQPNPCVNGSCKDLVGDYLCECQPGFDGRNCSNLIDNCFSLPCKNNGNCTNKVNNYTCTCQAGFSGSDCETNINE
CDPDPCNSNALNCTDLINGYICYCKLGFRGENCSEIIDNCNPQPCRHNSTCTNRFVTNDFHCNCSLGYEGDRCEIEIDECKALPCKNGGNCTDLPGKYLCTCPPGFTGVDCEINIDDCKNVSCLNGGKC
IDLINSYVCECPVTHKGKHCEKKIPDDVDITFPGGDGSYIKHCVKGNASKEFSISLWFRFLGRSRGVFLNLGFGCETVRPFLIMSHSSIRFQFRNQFSFVMIDNLKINNEKLVINNGVWHHIYIALSSV
TLNFKVVLDGIIVQEEQKAELSGMTFFNESGTILIGKSLLDGKLIESGFIGDVSQVALFKRVLDSTEIAGLVRNCSRNISDSIIPWVGLMADAGPNVSLLETWTCGTNVCPPEFTGKFCQTKIDKIPPT
VVFCPSPIKVYTEESSVFVTWKEPQFDDDVAVVEVIRSHSPNGLFSWGDYVITYIAKDAFENSVSCTFEINVTPFNCSEFQTANNSIHACGTWKYGRFCDYKCHEKYELAVPKPPFYVCGRPGTWSHGP
PTYPENPDLLVSECTATTPFNTITTNKISFPTSSCDDSFKNDFVESFKQLITELQDVWSICGTTCDFKNIKVSCKPDAKRFRRDMDPKWTGPKFILDISFDATNETNLAKIQNGIKTKEFNMTVIVNNV
SISSDLLNVEVKTVCNNGSILKNDICVKCPAGTFKNVTNELCEKCPFGSYSKEAGSEMCINCPANTTTTSEGSTEEKSCKVKCLFGAYLENNECKDCPIGFYKETAGFEKCQPCALGLTTKKTGAVTKD
QCKNDCTLGQELVNNTCQPCRMGYYRNNDSQPVCLPCPYNQSTYQLGATSPVCYAVCEVGKEISSDGLKCMECAIGYYKNDNNAKTCSKCPAGLTTKQLASTSIDQCIEKSSDSKYYDVQVAITSLTWS
DNLYNQNSVEYRDVAYKIIESVYGVYLNMSYLLFVNITKLSSGSVVANLTLGFNSSVKDPLFAFDSAVKSQQFYKLTVDPDSLIADRCKHIVCPMEQVCSYDTRVECKCTVGKQKSPDGEKCYDTCELK
GCPEGQECFSNSTFNECRCKDGKVLSSDKKECIYTCELKGCPEGQECFSNSTFNECRCKDGKVLSPDKKRCINQCSLHYCKNGGSCQLKETGPVCRCSKNYSGDQCQTLQTKSNALMIVLVSLSTVAVV
LIVIYVLKNRKKRIYNECRNDLMYDDEKSLELSVKNSNLDKKSVTLTRYDHLRSSEKLGRINGGATFDDNSPAPLIRNSDQKR

Nematostella vectensis:

>XP_032230096.1 neurogenic locus notch homolog protein 1 [Nematostella vectensis]
MWSVLNIFWFLTIFVNAKAAGFYYQRLKTSEGKTCRPRLEAKLYPKYCGSHTPCENIKDCTKPNDQCICDEDCGNICTDPTTNCTTGWCTNGGVCSEAGGVVTCSCPKNFTGARCEIDVDECTTLSQPC
QNGGTCSNVYGGYMCRCITGWDGADCSVNIDECKQNDPFPRCQHGGTCVDKIGSYTCICPPGKTGLVCNFDDECASNPCSANATCVTSFSGKASCICNSGWTGKNCDVDIKECEGDSSPCYHGGTCRDI
PGSFVCDCVPGFAGARCEDNINECESNPCVHGLCLDYQNKFECACSKGYTGRLCDVEINECDSNPCLNGGQCHDGLGNYSCTCQVGYVGEICQTNYDDCASNPCQNDGTCQDGIAQYTCLCPLGFTGKS
CETNTNECAGNPCMNMGTCIDGINSFRCQCPLGFTGNRCETEIDECESSPCQNGGTCKDKINGYVCICPPGASGTHCENDPNDCPANACQNGGVCIDGMNTYSCKCHPGFTGFSCGVPVNECASSPCRN
GGTCEDGVAQYYCRCRDGYTGKNCEVRIDSCIDHTCQNGASCVSSTPYAYSCQCKPGYTGQYCETDVDECAARPCVNGACVDGVNGFICRCDPGFTGDRCQINVDDCQSSPCVHGGSCIDSINTYTCQC
PKGFTGPRCEIHINECSSDPCQHGGTCSDRIGSYSCYCRPGYTGSNCQHPLDRCANDPCRNGATCRRTGEDLSDFHCECPLGYKGTICDVKEVSCAVAGTICANGGTCFDSNGVQSCTCKPGFTGSYCR
TNIDECAKGPCKYGATCHDAVANYTCTCTAGFTGKNCDININECASNPCQRGSCLDLVNGYLCSCPKGYIGKHCEVNADDCFVNACFNGGSCVDGIAEFKCTCPLGFSGSRCEVDVDECASSPCSALGT
EKCINNIGAYHCQCKQGFLGRHCDLNINECLSYPCRNGGECKDGAGEYTCLCPHGFSGDDCERRVYTCSDQPCLNSGTCRTVHNTYNCSCRPGVYGKQCEWNKDECLSAPCRNGGTCIDGYGSYSCKCP
LGFAGANCEESINECLSQPCKNGGSCRDIVNGYKCDCPSKMIGKNCETVVKDQCPVPNCAKKFDGGKCNPKCNTHECNWDGTTCSLGIEPWSNCTTVTKSGKACYQVFANGVCDRECNTGGCLFDGFDC
KPSVPKCGADKYCAARFANAECDAICNNVACQNDGLDCSFKKPEIVEGTLVLVLLVVPEAFMNGSRVFMRELSRTLNTIAFIKKDSEGKELVKVYPLPPSAPVPAERRKRSAEKIWFIDSDLTESHRFR
RAAVQKNGVQVQINLDNRGCETDCFQSTEQAAKYLGAQQSTGKLNLPYPVYSVKTEEKPTVEPETGFQPEPLWIAILCVGVPLFIVGVLAGGKRVYTKLWLPEGFVRRPVHQRRSLRRDPVGQEHSMRS
MNKSSDLEEEGAVGGDLTPPQEARDAKRVKLEEVDESQRVKVSEKEKDTRQWTRLHREAADVTVRNCTALALTPPQEGESEKPGIDTGVDARGPGGFTPLHLASCRGTLVDGCSIDDDKESDDSGGAMV
SDLLALGASYGARTDIEKETPLHLAARHSRADAAKRLLHAGADPNARDKLGRTPLHLAVGADAQGVFQILLRNRTTDLEAAMEDGTTPLILAARLDLLDIVKDLIKASCKVNNVDAQGKSALHWAAAVN
SHEVTSELCKNGAKKDMQDDKGQTPLFLGAREGSLEAVRILLLSYANRMIADNMDKTPEEVARQRAHNDIVELLSDWSIGCNSPKAAPAPTSPPDQRSPLNGTASPPSMDQISGNKVVTHFPVSAGTTR
PKTNSTSTRGARSKVQNENGAKRKRKRRKDDDLPHGNRKASQGGASSTYSPYGKELSPCSSGANFSPPAFSVVNGLSPQGSSATGISPSNSTTLSPQQHSSLDANSPQFPLSDSPFDDGYLDLDIFEDL
TDQDLHGDFASPACGLRGSTSDLHDSISMLPETTIGPVPQDSSVPTTSACMYLPRHSVAPFGHTAPRLYSAHSSPNLCADNESRQLIVAHSINGYPSNQSSIQACKLSNRLEEHVMMETTLHHNNQSMC
VPVESGMFREKHHTPPSAHSYGTSSYDSSPQKLPISYLTPSPESPKDWSSSPSSHSDWSNC

Amphimedon queenslandica:

>XP_019853114.1 PREDICTED: neurogenic locus notch homolog protein 1-like [Amphimedon queenslandica]
MDTRTKAAILYLLVGCWLSFSVAQNLCFVNTCSNNGTCYIENGSDVCMCPPSYTGMTCEIEVVSGCQSTTCKNGGTCNDEDGVVRCDCLPIFTGQFCDSLLNGCDSNPCFNEATCSNFFGAFVCSCPPG
FTGRQCETDNNDCASNPCADGGTCIDEVNGYTCECPPGFTGSNCSINIDDCRGSDVECHNDGECLDLVDDYYCDCTEEFGGRNCEIESDCPLDNLTQCENNGFCHRNTSSCSCITGYTGTYCETELNEC
DSNPCNNGTCVDKIGSFSCNCFPGYTGQQCEEVIDMCQPQPCYNGAMCVSSINGFDCFCRQGYTGQFCQVDIDECLSSPCQNGGNCTEMVNGFSCGCLPGYSGTQCEIDSCSSQPCQNDGTCIANGLTY

SCVCSLDYTDENCTTFITPCYFEPCLNGATCINEDLDNYTCSCLPGFTEEDCSVNIDDCGSNPCQNEGTCIDLVNDYQCDCSAGYTGSDCQTDIDECLMTPCFNNGTCTDLVNSVSCECPPGFNGSLCQ
NNINECSSSPCSSGSTCIDEIDDFTCLCPMGLTGTQCDISIIDCSDMPCGNNGTCTDTPGGYECSCSSGYTGVHCMVNINDCLPHPCNNGTCIDGINEYVCICPEDYTGDNCETPIDHCDSNPCSSLAT
CITNPGGYQCICPIDFTGTDCFDQINDCQPNPCNNGGTCSDLIGTFNCSCPLGFEGSICEYDINECASLPCLNGGNCTDLVNGYSCSCPHGFNGTNCENSTITTCESVVCENGGMCDDTPTGFECLCPN
GYTGPYCQNNIDDCLSNPCLNNATCIDEIANYTCECTEGFDGRNCAIDTDLCSPQPCFNGGTCSETSDSFFCTCPLGYFGSHCQNTLDPCSSSPCLNSGICTNVNGTNFSCACSQAYGGERCEIKLFPD
CLDMPCLNNGTCAELVGSGNIGGSGAGEPGPRIYCQCPLGYAGEFCENITDLCVSSPCKNNATCIGDSANFTCTCLPGFTGTLCETELTGCHTELYPCLNGGECMEMDGQFMCNCAPGFTGPLCGYGIN
ECRNQPCLNGGTCRDFFRYYVCICPPNFTGTDCESMIDPCTNIDCNNGSCIGDMGTYTCQCDPGWTGLQCESEINECDGVDCTNGTCVDLINNYTCQCSDGFTGQFCEINIDDCAGINCNNGTCVDGIG
SYQCDCLLGYTGPSCDTIIDDCAGMPCMNNASCIDLFNNYTCVCSDGFTGRFCEVNIDDCLNINCNNGSCEDLINDHMCNCFPGFTDQRCETDINECDGNPCNDGTCTDGINSFSCSCPPDYTGDTCDT
EINLCLMEQPCLNNGTCTSDKIEGIPIYNCSCSVPLYSGDNCEQINSCSLSPCQNNATCTGNLTTGDYTCHCSENYYGTHCERFDYCHSNPCQNDGTCINGSPGNLISDTFLCICMPQFNGSDCSMELS
PCSIDPCMNGGTCVEDGSTRYCQCPVGYTGNNCSEVFSSDTPYFNGDSYTLVPYSPNNNNSQSLINLHFRTSSQSGLLLYISEEDHSSYLSLGIEAGLLSLRMRQEGSFIQSVARYHPVSDDRWHYVEV
TQNGLEIKTEIDGYPNSTSLIDMFLIFEPAVTYIGGFDNYTSLPSAVLQSSGFIGYINATIQLGGEALHIIDDSIAGRDIAHGEAAGSCGPETCSNGGICVEESQSSFLCLCPIGFRGDTCEQDTEIIV
PYFNTRSYAIVSNDAFSLYGGIDIDITLHTSSPNGLIYYLYDSTNVTNDYFTLYLEEGGIGIEYSMEGSAHIVSYQGNVSDNEWHLMTVQLNTSGLYLILDGSLVLYSNNATLSSIYLTSPLFLGGLPN
GLIADSVSGSGLNGCIRDVQISNTSLGLIENNVLYGLGLLECPQSLCPLVQCLNGGTCVDSDVSVFGFECSCPSGYNGTYCEVLLSTCIPNPCLFDGVCTDFNNMTYSCQCTLGRQGRICDEELNITVP
AFYGNSYLAYSSLSSITNVQSNLILSLTFKPTSPSGLLLFNGHSNTDFSQYVSISLVNSSVVYKFDLGSGLATISSPITLGLNQWHTITAYRTGRVGFLRVNDEELLTNTSLGTLNGLNIAGDMWLGGT
DRFNIISQHAGVGTGLTGCISSVSVNGISINLVSSAERGLNIGECNMTSCSSFPCFNGGTCTETGSSFICQCPAGYEGGLCGLQTFSCLSSPCGNGGTCIEGVAEGGYNCLCPLGFGGNNCNEMLQVST
PSFNRTSYQEYSSPAPISLSTIISLSFHPTSSNGLILYIGDVSTTRDFLSLSLVSGRIQLRYDLGSGPAIIASPSVIPLNQWTSVTVNRVRKDGVLVVDSVSTNGSSPGFAGLLNPAGNLYIGGGAGGV
GGYQVSPNAGSHVGLTGCVDTATLRVNSFGLGAVISSRGVIQCQVDPCSHSPCQNGGSCVSSDLTYSCVCPLGYSGDQCQEISNATEGAAISQCPTSVACNVNPCLNGGKCIAVLVNGIIEERCQCSLP
FAAGAKCEQRVSFSSAFFAGDGYLLFPAVNIPLSSITIHTEIRLSIIPSNGSGLLLYIGHANPSTGNYLLVGMNEGVLEFRFQLGSGVGLVETSQPLAIGQQHNITLLRTDYRGRILMSQGNTTVFTDG
MSQGSSRHLNLSPYDFVLFLGGAPNVPLISQQVYQTGFSGCLVSVVQLGYSMNPEPVPFDTSSVGENVTTCS

Alignment:

```
 XP_012554603.1[H.vulgaris]           MVFYMLLLFHVLWMVACNTNVKGYVDSKQNNEFYAANYKCPNGWQQRCEKCYLYVNESKQ  60
XP_019853114.1[A.queenslandica]      ------------------------------------------------------------  0
XP_032230096.1[N.vectensis]          ------------------------------------------------------------  0
NP_001245510.1[D.melanogaster]       ------------------------------------------------------------  0
XP_019643529.1[B.belcheri]           ------------------------------------------------------------  0
XP_032815028.1[P.marinus]            ------------------------------------------------------------  0
XP_014025097.1[S.salar]              ------------------------------------------------------------  0
XP_018114267.1[X.laevis]             ------------------------------------------------------------  0
NP_001238962.2[G.gallus]             ------------------------------------------------------------  0
NP_035058.2[M.musculus]              ------------------------------------------------------------  0
Q04721.3[H.sapiens]                  ------------------------------------------------------------  0
XP_009243797.2[P.abelii]             ------------------------------------------------------------  0


XP_012554603.1[H.vulgaris]           WTDAQYYCKQNGGNLVTIKEDAVNLFLARYVQYSSRLAITIWTGLHARKVHKEFQWVDNS  120
XP_019853114.1[A.queenslandica]      ------------------------------------------------------------  0
XP_032230096.1[N.vectensis]          ------------------------------------------------------------  0
NP_001245510.1[D.melanogaster]       ------------------------------------------------------------  0
XP_019643529.1[B.belcheri]           ------------------------------------------------------------  0
XP_032815028.1[P.marinus]            ------------------------------------------------------------  0
XP_014025097.1[S.salar]              ------------------------------------------------------------  0
XP_018114267.1[X.laevis]             ------------------------------------------------------------  0
NP_001238962.2[G.gallus]             ------------------------------------------------------------  0
NP_035058.2[M.musculus]              ------------------------------------------------------------  0
Q04721.3[H.sapiens]                  ------------------------------------------------------------  0
XP_009243797.2[P.abelii]             ------------------------------------------------------------  0


XP_012554603.1[H.vulgaris]           ESVHYEFRWLPGQPNMVTLDEGCVEMYTKSRQYLWDDVSCSKESPFVCQTDGCIIGTQRC  180
XP_019853114.1[A.queenslandica]      ------------------------------------------------------------  0
XP_032230096.1[N.vectensis]          ------------------------------------------------------------  0
NP_001245510.1[D.melanogaster]       ------------------------------------------------------------  0
XP_019643529.1[B.belcheri]           ------------------------------------------------------------  0
XP_032815028.1[P.marinus]            ------------------------------------------------------------  0
XP_014025097.1[S.salar]              ------------------------------------------------------------  0
XP_018114267.1[X.laevis]             ------------------------------------------------------------  0
NP_001238962.2[G.gallus]             ------------------------------------------------------------  0
NP_035058.2[M.musculus]              ------------------------------------------------------------  0
Q04721.3[H.sapiens]                  ------------------------------------------------------------  0
XP_009243797.2[P.abelii]             ------------------------------------------------------------  0


XP_012554603.1[H.vulgaris]           DNGKCVSKHLFCDSKNDCGDFSDEKNCTKCNEHYTAQEGSISSPNFPQHYGNNRDCTWLI  240
XP_019853114.1[A.queenslandica]      ------------------------------------------------------------  0
XP_032230096.1[N.vectensis]          ------------------------------------------------------------  0
NP_001245510.1[D.melanogaster]       ------------------------------------------------------------  0
XP_019643529.1[B.belcheri]           ------------------------------------------------------------  0
XP_032815028.1[P.marinus]            ------------------------------------------------------------  0
XP_014025097.1[S.salar]              ------------------------------------------------------------  0
XP_018114267.1[X.laevis]             ------------------------------------------------------------  0
NP_001238962.2[G.gallus]             ------------------------------------------------------------  0
NP_035058.2[M.musculus]              ------------------------------------------------------------  0
Q04721.3[H.sapiens]                  ------------------------------------------------------------  0
XP_009243797.2[P.abelii]             ------------------------------------------------------------  0


XP_012554603.1[H.vulgaris]           EVESSYIIEIKFLEFFIENSFDWVEVFDGPTLASKSFGKFSGNFKPEVILSSSSKILVHL  300
XP_019853114.1[A.queenslandica]      ------------------------------------------------------------  0
XP_032230096.1[N.vectensis]          ------------------------------------------------------------  0
NP_001245510.1[D.melanogaster]       ------------------------------------------------------------  0
XP_019643529.1[B.belcheri]           ------------------------------------------------------------  0
XP_032815028.1[P.marinus]            ------------------------------------------------------------  0
XP_014025097.1[S.salar]              ------------------------------------------------------------  0
XP_018114267.1[X.laevis]             ------------------------------------------------------------  0
NP_001238962.2[G.gallus]             ------------------------------------------------------------  0
NP_035058.2[M.musculus]              ------------------------------------------------------------  0
Q04721.3[H.sapiens]                  ------------------------------------------------------------  0
XP_009243797.2[P.abelii]             ------------------------------------------------------------  0


XP_012554603.1[H.vulgaris]           KSDKSTSDKGFFARYKAVKKGCNVDLIINSNKPEYISSPNFPLNYPDNTKCEWTIRNEDP  360
XP_019853114.1[A.queenslandica]      ------------------------------------------------------------  0
XP_032230096.1[N.vectensis]          ------------------------------------------------------------  0
NP_001245510.1[D.melanogaster]       ------------------------------------------------------------  0
XP_019643529.1[B.belcheri]           ------------------------------------------------------------  0
XP_032815028.1[P.marinus]            ------------------------------------------------------------  0
XP_014025097.1[S.salar]              ------------------------------------------------------------  0
XP_018114267.1[X.laevis]             ------------------------------------------------------------  0
NP_001238962.2[G.gallus]             ------------------------------------------------------------  0
NP_035058.2[M.musculus]              ------------------------------------------------------------  0
Q04721.3[H.sapiens]                  ------------------------------------------------------------  0
XP_009243797.2[P.abelii]             ------------------------------------------------------------  0


XP_012554603.1[H.vulgaris]           SKILTFQLLELETEENDFIEVRDGLTEDNALLGKFFGNFSNIQKRHIFTSNFKMHVKFQS  420
XP_019853114.1[A.queenslandica]      ------------------------------------------------------------  0
XP_032230096.1[N.vectensis]          ------------------------------------------------------------  0
NP_001245510.1[D.melanogaster]       ------------------------------------------------------------  0
XP_019643529.1[B.belcheri]           ------------------------------------------------------------  0
XP_032815028.1[P.marinus]            ------------------------------------------------------------  0
XP_014025097.1[S.salar]              ------------------------------------------------------------  0
XP_018114267.1[X.laevis]             ------------------------------------------------------------  0
```

```
NP_001238962.2[G.gallus]            ------------------------------------------------------------   0
NP_035058.2[M.musculus]             ------------------------------------------------------------   0
Q04721.3[H.sapiens]                 ------------------------------------------------------------   0
XP_009243797.2[P.abelii]            ------------------------------------------------------------   0


XP_012554603.1[H.vulgaris]          NFQLNQKGFRAVIFSGCTATINATHGTISSPNFGVGNYPNNILCSWTVHAQTDKEVSVIF   480
XP_019853114.1[A.queenslandica]     ------------------------------------------------------------   0
XP_032230096.1[N.vectensis]         ------------------------------------------------------------   0
NP_001245510.1[D.melanogaster]      ------------------------------------------------------------   0
XP_019643529.1[B.belcheri]          ------------------------------------------------------------   0
XP_032815028.1[P.marinus]           ------------------------------------------------------------   0
XP_014025097.1[S.salar]             ------------------------------------------------------------   0
XP_018114267.1[X.laevis]            ------------------------------------------------------------   0
NP_001238962.2[G.gallus]            ------------------------------------------------------------   0
NP_035058.2[M.musculus]             ------------------------------------------------------------   0
Q04721.3[H.sapiens]                 ------------------------------------------------------------   0
XP_009243797.2[P.abelii]            ------------------------------------------------------------   0


XP_012554603.1[H.vulgaris]          HSFDTEYKQDTVVLRTCDDVMIKECYGSNKTTLGKEMLPLEPIVFNSSCFKVIFKSDETY   540
XP_019853114.1[A.queenslandica]     ------------------------------------------------------------   0
XP_032230096.1[N.vectensis]         ------------------------------------------------------------   0
NP_001245510.1[D.melanogaster]      ------------------------------------------------------------   0
XP_019643529.1[B.belcheri]          ------------------------------------------------------------   0
XP_032815028.1[P.marinus]           ------------------------------------------------------------   0
XP_014025097.1[S.salar]             ------------------------------------------------------------   0
XP_018114267.1[X.laevis]            ------------------------------------------------------------   0
NP_001238962.2[G.gallus]            ------------------------------------------------------------   0
NP_035058.2[M.musculus]             ------------------------------------------------------------   0
Q04721.3[H.sapiens]                 ------------------------------------------------------------   0
XP_009243797.2[P.abelii]            ------------------------------------------------------------   0


XP_012554603.1[H.vulgaris]          SLEGFNATFSIGCPMLPAINGGSFRQDQKFYGASVEYKCDSRFRIKSENGNVRKCMYGGN   600
XP_019853114.1[A.queenslandica]     ------------------------------------------------------------   0
XP_032230096.1[N.vectensis]         ------------------------------------------------------------   0
NP_001245510.1[D.melanogaster]      ------------------------------------------------------------   0
XP_019643529.1[B.belcheri]          ------------------------------------------------------------   0
XP_032815028.1[P.marinus]           ------------------------------------------------------------   0
XP_014025097.1[S.salar]             ------------------------------------------------------------   0
XP_018114267.1[X.laevis]            ------------------------------------------------------------   0
NP_001238962.2[G.gallus]            ------------------------------------------------------------   0
NP_035058.2[M.musculus]             ------------------------------------------------------------   0
Q04721.3[H.sapiens]                 ------------------------------------------------------------   0
XP_009243797.2[P.abelii]            ------------------------------------------------------------   0


XP_012554603.1[H.vulgaris]          WSEPAPFCEEINCGHPGTIENGGFEIYPLNSTFVFMTSVKYSCNVGYILVGEPNRVCQVN   660
XP_019853114.1[A.queenslandica]     ------------------------------------------------------------   0
XP_032230096.1[N.vectensis]         ------------------------------------------------------------   0
NP_001245510.1[D.melanogaster]      ------------------------------------------------------------   0
XP_019643529.1[B.belcheri]          ------------------------------------------------------------   0
XP_032815028.1[P.marinus]           ------------------------------------------------------------   0
XP_014025097.1[S.salar]             ------------------------------------------------------------   0
XP_018114267.1[X.laevis]            ------------------------------------------------------------   0
NP_001238962.2[G.gallus]            ------------------------------------------------------------   0
NP_035058.2[M.musculus]             ------------------------------------------------------------   0
Q04721.3[H.sapiens]                 ------------------------------------------------------------   0
XP_009243797.2[P.abelii]            ------------------------------------------------------------   0


XP_012554603.1[H.vulgaris]          STWSGGVERKCEVLKCPDPGTPVNGTRSGNEFNVGSSITFSCEKGFRRHGARTITCGITG   720
XP_019853114.1[A.queenslandica]     ------------------------------------------------------------   0
XP_032230096.1[N.vectensis]         ------------------------------------------------------------   0
NP_001245510.1[D.melanogaster]      ------------------------------------------------------------   0
XP_019643529.1[B.belcheri]          ------------------------------------------------------------   0
XP_032815028.1[P.marinus]           ------------------------------------------------------------   0
XP_014025097.1[S.salar]             ------------------------------------------------------------   0
XP_018114267.1[X.laevis]            ------------------------------------------------------------   0
NP_001238962.2[G.gallus]            ------------------------------------------------------------   0
NP_035058.2[M.musculus]             ------------------------------------------------------------   0
Q04721.3[H.sapiens]                 ------------------------------------------------------------   0
XP_009243797.2[P.abelii]            ------------------------------------------------------------   0


XP_012554603.1[H.vulgaris]          KWSSETPLCKVVVCQLPSFSLNTIISSSSIGFNTPYKTVLHISCKKGFELIGSVNITCTE   780
XP_019853114.1[A.queenslandica]     ------------------------------------------------------------   0
XP_032230096.1[N.vectensis]         ------------------------------------------------------------   0
NP_001245510.1[D.melanogaster]      ------------------------------------------------------------   0
XP_019643529.1[B.belcheri]          ------------------------------------------------------------   0
XP_032815028.1[P.marinus]           ------------------------------------------------------------   0
XP_014025097.1[S.salar]             ------------------------------------------------------------   0
XP_018114267.1[X.laevis]            ------------------------------------------------------------   0
NP_001238962.2[G.gallus]            ------------------------------------------------------------   0
NP_035058.2[M.musculus]             ------------------------------------------------------------   0
Q04721.3[H.sapiens]                 ------------------------------------------------------------   0
XP_009243797.2[P.abelii]            ------------------------------------------------------------   0


XP_012554603.1[H.vulgaris]          SGAWKPDVPQCIDINECLNNPCRGLGSQCTNLMGSYKCTCKSGFRQIDETTCEDIDECKS   840
XP_019853114.1[A.queenslandica]     ------------------------------------------------------------   0
XP_032230096.1[N.vectensis]         ------------------------------------------------------------   0
NP_001245510.1[D.melanogaster]      ------------------------------------------------------------   0
XP_019643529.1[B.belcheri]          ------------------------------------------------------------   0
```

```
XP_032815028.1[P.marinus]          ------------------------------------------------------------   0
XP_014025097.1[S.salar]            ------------------------------------------------------------   0
XP_018114267.1[X.laevis]           ------------------------------------------------------------   0
NP_001238962.2[G.gallus]           ------------------------------------------------------------   0
NP_035058.2[M.musculus]            ------------------------------------------------------------   0
Q04721.3[H.sapiens]                ------------------------------------------------------------   0
XP_009243797.2[P.abelii]           ------------------------------------------------------------   0


XP_012554603.1[H.vulgaris]         SGKTVKCNQKCTNIDGGYFCSCEDGYFLYDGDNKTVNRVEAEESFVNHTCLGNPCKLPKI   900
XP_019853114.1[A.queenslandica]    ------------------------------------------------------------   0
XP_032230096.1[N.vectensis]        ------------------------------------------------------------   0
NP_001245510.1[D.melanogaster]     ------------------------------------------------------------   0
XP_019643529.1[B.belcheri]         ------------------------------------------------------------   0
XP_032815028.1[P.marinus]          ------------------------------------------------------------   0
XP_014025097.1[S.salar]            ------------------------------------------------------------   0
XP_018114267.1[X.laevis]           ------------------------------------------------------------   0
NP_001238962.2[G.gallus]           ------------------------------------------------------------   0
NP_035058.2[M.musculus]            ------------------------------------------------------------   0
Q04721.3[H.sapiens]                ------------------------------------------------------------   0
XP_009243797.2[P.abelii]           ------------------------------------------------------------   0


XP_012554603.1[H.vulgaris]         PENGYILNYGNRYPTEITFQCDKGWIVDGDLSIKCLKNGSWSGSISDCIAARCNPLPDVP   960
XP_019853114.1[A.queenslandica]    ------------------------------------------------------------   0
XP_032230096.1[N.vectensis]        ------------------------------------------------------------   0
NP_001245510.1[D.melanogaster]     ------------------------------------------------------------   0
XP_019643529.1[B.belcheri]         ------------------------------------------------------------   0
XP_032815028.1[P.marinus]          ------------------------------------------------------------   0
XP_014025097.1[S.salar]            ------------------------------------------------------------   0
XP_018114267.1[X.laevis]           ------------------------------------------------------------   0
NP_001238962.2[G.gallus]           ------------------------------------------------------------   0
NP_035058.2[M.musculus]            ------------------------------------------------------------   0
Q04721.3[H.sapiens]                ------------------------------------------------------------   0
XP_009243797.2[P.abelii]           ------------------------------------------------------------   0


XP_012554603.1[H.vulgaris]         NSSSVVDGGLVKGQYSQGTIYTYTCINGYEIISGNSKRMCSQNATASFWSGSSPVCERVS   1020
XP_019853114.1[A.queenslandica]    ------------------------------------------------------------   0
XP_032230096.1[N.vectensis]        ------------------------------------------------------------   0
NP_001245510.1[D.melanogaster]     ------------------------------------------------------------   0
XP_019643529.1[B.belcheri]         ------------------------------------------------------------   0
XP_032815028.1[P.marinus]          ------------------------------------------------------------   0
XP_014025097.1[S.salar]            ------------------------------------------------------------   0
XP_018114267.1[X.laevis]           ------------------------------------------------------------   0
NP_001238962.2[G.gallus]           ------------------------------------------------------------   0
NP_035058.2[M.musculus]            ------------------------------------------------------------   0
Q04721.3[H.sapiens]                ------------------------------------------------------------   0
XP_009243797.2[P.abelii]           ------------------------------------------------------------   0


XP_012554603.1[H.vulgaris]         CGPLENPKNGHVNITGDKYGDSSKFFCDGGYIITGNDSSTCQANGRWSASSPFCLASPCP   1080
XP_019853114.1[A.queenslandica]    ------------------------------------------------------------   0
XP_032230096.1[N.vectensis]        ------------------------------------------------------------   0
NP_001245510.1[D.melanogaster]     ------------------------------------------------------------   0
XP_019643529.1[B.belcheri]         ------------------------------------------------------------   0
XP_032815028.1[P.marinus]          ------------------------------------------------------------   0
XP_014025097.1[S.salar]            ------------------------------------------------------------   0
XP_018114267.1[X.laevis]           ------------------------------------------------------------   0
NP_001238962.2[G.gallus]           ------------------------------------------------------------   0
NP_035058.2[M.musculus]            ------------------------------------------------------------   0
Q04721.3[H.sapiens]                ------------------------------------------------------------   0
XP_009243797.2[P.abelii]           ------------------------------------------------------------   0


XP_012554603.1[H.vulgaris]         DPGIPINGEIIGLISLDQTITFRCLVNGSILIGDNKRTCIFNKSTGLNQWNGSQPFCKDT   1140
XP_019853114.1[A.queenslandica]    ------------------------------------------------------------   0
XP_032230096.1[N.vectensis]        ------------------------------------------------------------   0
NP_001245510.1[D.melanogaster]     ------------------------------------------------------------   0
XP_019643529.1[B.belcheri]         ------------------------------------------------------------   0
XP_032815028.1[P.marinus]          ------------------------------------------------------------   0
XP_014025097.1[S.salar]            ------------------------------------------------------------   0
XP_018114267.1[X.laevis]           ------------------------------------------------------------   0
NP_001238962.2[G.gallus]           ------------------------------------------------------------   0
NP_035058.2[M.musculus]            ------------------------------------------------------------   0
Q04721.3[H.sapiens]                ------------------------------------------------------------   0
XP_009243797.2[P.abelii]           ------------------------------------------------------------   0


XP_012554603.1[H.vulgaris]         STPTFKFCPMSTIEFTLPQSEKSMMVNWTLPTLTDNVHNLLNITVSPDYVSPMVMPAGLN   1200
XP_019853114.1[A.queenslandica]    ------------------------------------------------------------   0
XP_032230096.1[N.vectensis]        ------------------------------------------------------------   0
NP_001245510.1[D.melanogaster]     ------------------------------------------------------------   0
XP_019643529.1[B.belcheri]         ------------------------------------------------------------   0
XP_032815028.1[P.marinus]          ------------------------------------------------------------   0
XP_014025097.1[S.salar]            ------------------------------------------------------------   0
XP_018114267.1[X.laevis]           ------------------------------------------------------------   0
NP_001238962.2[G.gallus]           ------------------------------------------------------------   0
NP_035058.2[M.musculus]            ------------------------------------------------------------   0
Q04721.3[H.sapiens]                ------------------------------------------------------------   0
XP_009243797.2[P.abelii]           ------------------------------------------------------------   0


XP_012554603.1[H.vulgaris]         IITYTATYKDGSTAVCEVKINVQDKEIPKIFCPKNIVIKSDLSSKVFWSDGIYTDNVGIK   1260
XP_019853114.1[A.queenslandica]    -------------------------------------------------MDTR-------   4
```

```
XP_032230096.1[N.vectensis]          ------------------------------------------------------------   0
NP_001245510.1[D.melanogaster]       ---------------MQSQRSRRRSRAPN-------------TWICFWINKMHAV-----   27
XP_019643529.1[B.belcheri]           ------------------------------------------------------------   0
XP_032815028.1[P.marinus]            -------------------------------------------------MEPRAL-----   6
XP_014025097.1[S.salar]              ---------------------------------------------------MGQL-----   4
XP_018114267.1[X.laevis]             ------------------------------------------------------------   0
NP_001238962.2[G.gallus]             -------------------------------------------------MARGAG-----   6
NP_035058.2[M.musculus]              -------------------------------------------------MP---A-----   3
Q04721.3[H.sapiens]                  -------------------------------------------------MP---A-----   3
XP_009243797.2[P.abelii]             -------------------------------------------------MP---A-----   3


XP_012554603.1[H.vulgaris]           NVIFNPPNG--TILESNKYHKVTMTVEDISGNSDSCVMEIYVEGCGCTQRTCDYKQRNGQ   1318
XP_019853114.1[A.queenslandica]      ------TKAAILYLLVGCWLSFS-------------VAQNLCFVNTCSNNGTCYI-ENGS   44
XP_032230096.1[N.vectensis]          ------------------------------------------------------------   0
NP_001245510.1[D.melanogaster]       --ASLPASLPL-LLLTLAFANLPNTV---R-GTDTALVAASCTSVGCQNGGTCVTQLNGK   80
XP_019643529.1[B.belcheri]           ----MRSLFAVVLLLAAAGPALV------Q------GLVCQANNSPCLNGGICETDSSGQ   44
XP_032815028.1[P.marinus]            --LPL-LRL-LLLLLLALGARLS------R------GLQCQDSNQPCINGGTCETFANGT   50
XP_014025097.1[S.salar]              --PVF-SSG-KVLVIVVCCVRLS------L------ALQCLDAVKPCVNNATCATFTNGT   48
XP_018114267.1[X.laevis]             --MGVQAVF-GLCVL-LLGMPCC------D------GLQCRGASEPCLNEGRCITSDDGS   44
NP_001238962.2[G.gallus]             --PAFAALF-GLVLAVLSRAEPA------A------ALQCMDDSKPCVNEGKCIPYQNGT   51
NP_035058.2[M.musculus]              --LRPAALR-ALLWLWLCGAGPA------H------ALQCRGGQEPCVNEGTCVTYHNGT   48
Q04721.3[H.sapiens]                  --LRPALLW-ALLALWLCCAAPA------H------ALQCRDGYEPCVNEGMCVTYHNGT   48
XP_009243797.2[P.abelii]             --LRPALPW-ALLALWLCCAAPA------R------ALQCRDGYEPCVNEGMCVTYHNGT   48


XP_012554603.1[H.vulgaris]           SVCTIAPGVVPDTVII-------------------------------------------S   1335
XP_019853114.1[A.queenslandica]      DVCMCPPSYTGMTCEIEVVSGCQSTTCKNGGTCND----EDGVVRCDCLPIFTGQFCDSL   100
XP_032230096.1[N.vectensis]          ------------------------------------------------------------   0
NP_001245510.1[D.melanogaster]       TYCACDSHYVGDYCEH--RNPCNSMRCQNGGTCQVTFRNGRPGISCKCPLGFDESLCEIA   138
XP_019643529.1[B.belcheri]           GSCVCTDGWVGEYCQE--VDPCASNPCRHGGTCVVH---DNATWSCDCPLGYRDMICLTV   99
XP_032815028.1[P.marinus]            GICKCQHNFVGIYCQL--DNRCASSPCQNGGTCSTVVRGGVPDYACECPLGFMDPVCLTP   108
XP_014025097.1[S.salar]              GYCRCAAGFLGDYCHH--KDPCHPGYCLNGGNCSVAVSGIPGSPTCSCPLGYTGQHCQTP   106
XP_018114267.1[X.laevis]             AYCKCPDAFVGEYCQL--QNPCQETVCLNGGSCKV--SPDLSKGVCTCAPGFSGENCKQP   100
NP_001238962.2[G.gallus]             GYCKCREGYLGDYCQY--RNPCESNTCKNGGTCET--TSLIGKATCKCAPGFTGEDCQYS   107
NP_035058.2[M.musculus]              GFCRCPEGFLGEYCQH--RDPCEKNRCQNGGTCVP--QGMLGKATCRCAPGFTGEDCQYS   104
Q04721.3[H.sapiens]                  GYCKCPEGFLGEYCQH--RDPCEKNRCQNGGTCVA--QAMLGKATCRCASGFTGEDCQYS   104
XP_009243797.2[P.abelii]             GYCKCPEGFLGEYCQH--RDPCEKNRCQNGGTCVA--QAMLGKATCRCASGFTGEDCQYS   104


XP_012554603.1[H.vulgaris]           CNLN------CFQNKTTRFVSNTEFEKTSNVMCIPNKGWENKSPSGCVERKPLISCLKGS   1389
XP_019853114.1[A.queenslandica]      LN-GC-DSNPCFNEATCSNF-FGA------FVCSCPPGFTGRQCETDNNDCASNPCADGG   151
XP_032230096.1[N.vectensis]          -------------------------------------MWSVLNIFWF-LTIFVNAKA---   19
NP_001245510.1[D.melanogaster]       VPNAC-DHVTCLNGGTCQLKTLEE------YTCACANGYTGERCETK-NLCASSPCRNGA   190
XP_019643529.1[B.belcheri]           VDNPC-VTNPCNNGGTCELITIDE------YRCNCQPGYTGDTCDVV-EHCYSQPCKNGG   151
XP_032815028.1[P.marinus]            ADNAC-LGVPCHNGGTCRLLSLHR------FECHCLLGWSGKTCEKA-DPCASNPCAHGG   160
XP_014025097.1[S.salar]              QNSTCYPNNPCANQGVCTLLSLDK------YKCQCAHGWTGMRCNRE-DSCLSGPCANGG   159
XP_018114267.1[X.laevis]             VASVCSLTQPCMNGGTCRLLTLEH------YECICPFGWRGKDCEFI-DACASQPCANGS   153
NP_001238962.2[G.gallus]             ESHICYVSQPCLNGGTCHPHSQET------YECVCPPGYTGKDCQWI-DACTSQPCANGS   160
NP_035058.2[M.musculus]              TSHPCFVSRPCQNGGTCHMLSRDT------YECTCQVGFTGKQCQWT-DACLSHPCENGS   157
Q04721.3[H.sapiens]                  TSHPCFVSRPCLNGGTCHMLSRDT------YECTCQVGFTGKECQWT-DACLSHPCANGS   157
XP_009243797.2[P.abelii]             TSHPCFVSRPCLNGGTCHMLSRDT------YECTCQVGFTGKECQWT-DACLSHPCANGS   157
                                                                           :

XP_012554603.1[H.vulgaris]           LSIDFTTTGDNTNEKPFLDIFTFLNNLKNSTCSFIYQTGNLISNGTSVAGDFKILSHDDG   1449
XP_019853114.1[A.queenslandica]      TCIDE--VNGYT--------CECPPGFTGSNCS---------------------------   174
XP_032230096.1[N.vectensis]          --------AGFY--------YQRLKTSEGKTCR---------------------------   36
NP_001245510.1[D.melanogaster]       TCTALAGSSSFT--------CSCPPGFTGDTCS---------------------------   215
XP_019643529.1[B.belcheri]           TCTS-TE-SGYT--------CACLGGFEGSTCQ---------------------------   174
XP_032815028.1[P.marinus]            SCRA-VG-SRYA--------CACRPGFSGPMCR---------------------------   183
XP_014025097.1[S.salar]              TCSSPPG-GKFG--------CTCPPGYLGPRCL---------------------------   183
XP_018114267.1[X.laevis]             TCLP-NE-NQYS--------CTCQAGYTGLKCE---------------------------   176
NP_001238962.2[G.gallus]             TCTV-SG-HKFS--------CICQSGYTGQKCE---------------------------   183
NP_035058.2[M.musculus]              TCTS-VA-SQFS--------CKCPAGLTGQKCE---------------------------   180
Q04721.3[H.sapiens]                  TCTT-VA-NQFS--------CKCLTGFTGQKCE---------------------------   180
XP_009243797.2[P.abelii]             TCTT-VA-NQFS--------CKCLPGFTGQKCE---------------------------   180
                                                                 .  *

XP_012554603.1[H.vulgaris]           VIPLLNGHLEECKTILLKDLDLFKTNLLQLTSSVDIKLLNISSCCPLNKSYCCPTGHLIK   1509
XP_019853114.1[A.queenslandica]      ------INIDDCRGS-------------------DVECHNDGECLDLVDDYYCDCTEE--   207
XP_032230096.1[N.vectensis]          ------PRLEAKL------------------------------------------YPK--   46
NP_001245510.1[D.melanogaster]       ------YDIEECQSN---------------------PCKYGGTCVNTHGSYQCMCPTG--   246
XP_019643529.1[B.belcheri]           ------TDIDECAGT--------------------NPCQSGGQCQNTMGSFTCTCPKE--   206
XP_032815028.1[P.marinus]            ------ADVDECAAS---------------------PCRNGGSCANVPGSYACACPAA--   214
XP_014025097.1[S.salar]              ------NDTDECAAS-------------------APVCQNQGRCVNTPGSYRCNCAPG--   216
XP_018114267.1[X.laevis]             ------MDVNECTSP--------------------GLCKNGGTCENLPGSYRCQCHPG--   208
NP_001238962.2[G.gallus]             ------IDVNECATS--------------------GLCHHGGTCINLPGSFRCQCKPG--   215
NP_035058.2[M.musculus]              ------ADINECDIP--------------------GRCQHGGTCLNLPGSYRCQCPQG--   212
Q04721.3[H.sapiens]                  ------TDVNECDIP--------------------GHCQHGGTCLNLPGSYQCQCPQG--   212
XP_009243797.2[P.abelii]             ------TDVNECDIP--------------------GHCQHGGTCLNLPGSYQCQCPQG--   212
                                              :

XP_012554603.1[H.vulgaris]           TTGLTVKASY-CIPCG-----LGMYHNSTNN--KCLNCPVGFYNDLEFADQCKRCPNETN   1561
XP_019853114.1[A.queenslandica]      FGGRNCEIESDCPLDNLTQCENNGFCHRNTS---SCSCITGYTGTYC-ETELNECDS---   260
XP_032230096.1[N.vectensis]          YC------------GSHTPCENIKDCTKPND---QCICD-EDCGNIC-TDPTTNCTT---   86
NP_001245510.1[D.melanogaster]       YTGKDCDTKYK--PCSPSPCQNGGICRSNG-LSYECKCPKGFEGKNC-EQNYDDCLG---   299
XP_019643529.1[B.belcheri]           RTGRLCEEEYI--PCSPSPCQHGGTCESVDTYEYTCHCMSGFTGDDC-EVNVDDCVD---   260
XP_032815028.1[P.marinus]            YTGRHCETAYV--PCAPSPCLNGGTCRQTGDTAYDCSCLPGFEGRDC-DVNVDDCPG---   268
XP_014025097.1[S.salar]              FTGRQCETPYI--PCSPSPCLNGGTCRPTSETSYWCHCLPGFNGTNC-ENNIDDCPD---   270
XP_018114267.1[X.laevis]             YIGQRCESPFI--PCSPSPCMNGGTCRQTSEFAYECNCLPGFDGKNC-ESNIDDCPS---   262
NP_001238962.2[G.gallus]             YTGHRCESNYV--PCSPSPCMNGGTCHQTSDFTFECNCLPGFKGSIC-EKNVDDCPN---   269
NP_035058.2[M.musculus]              FTGQHCDSPYV--PCAPSPCVNGGTCRQTGDFTFECNCLPGFEGSTC-ERNIDDCPN---   266
Q04721.3[H.sapiens]                  FTGQYCDSLYV--PCAPSPCVNGGTCRQTGDFTFECNCLPGFEGSTC-ERNIDDCPN---   266
XP_009243797.2[P.abelii]             FTGQYCDSLYV--PCAPSPCVNGGTCRQTGDFTFECSCLPGFEGSTC-ERNIDDCPN---   266
                                                                          *     .          *
```

```
XP_012554603.1[H.vulgaris]          NLKVQSDSCTKMCAAGSYSSTGLEP----------------TCRK--------C-DYGEY   1596
XP_019853114.1[A.queenslandica]     ----------NPCNN-GTCVDKIGSFSCNCFPGYTGQQCEEVIDMCQ---PQPCYNGAMC   306
XP_032230096.1[N.vectensis]         ----------GWCTNGGVCSEAGGVVTCSCPKNFTGARCEIDVDECTTL-SQPCQNGGTC   135
NP_001245510.1[D.melanogaster]      ----------HLCQNGGTCIDGISDYTCRCPPNFTGRFCQDDVDECAQRDHPVCQNGATC   349
XP_019643529.1[B.belcheri]          ----------HLCENGAACVDGVNEYTCTCPSEWQGRYCNEDVDECMQS-PNICLNSGTC   309
XP_032815028.1[P.marinus]           ----------NRCDNGATCVDGVNTYNCQCSPQWTGQYCTEDVDECQLQ-PNACLNGGTC   317
XP_014025097.1[S.salar]             ----------HQCRNGGTCMDGVNTYNCQCPPEWTGQFCTEDVDECRLQ-PNTCQNGGTC   319
XP_018114267.1[X.laevis]            ----------HKCQNGGTCVDGVNTYNCRCPPQWTGQFCTEDVDECLLQ-PNACQNGGTC   311
NP_001238962.2[G.gallus]            ----------HNCQNGGICVDGVNTYNCRCPPQWTGQFCTEDVDECQLQ-PNACQNGGTC   318
NP_035058.2[M.musculus]             ----------HKCQNGGVCVDGVNTYNCRCPPQWTGQFCTEDVDECLLQ-PNACQNGGTC   315
Q04721.3[H.sapiens]                 ----------HRCQNGGVCVDGVNTYNCRCPPQWTGQFCTEDVDECLLQ-PNACQNGGTC   315
XP_009243797.2[P.abelii]            ----------HRCQNGGVCVDGVNTYNCRCPPQWTGQFCTEDVDECLLQ-PNACQNGGTC   315
                                               *   . .                             * : .

XP_012554603.1[H.vulgaris]          QSQVGAYNCIKCPYNLTTSRSGSENISECKAQCSPGTYSSTGLEPCTKCAKGLFQNEIGR   1656
XP_019853114.1[A.queenslandica]     VSSINGFDCFCRQ--GYTGQFCQVDIDECLSS---------------PCQNGGNCTEMVN   349
XP_032230096.1[N.vectensis]         SNVYGGYMCRCIT--GWDGADCSVNIDECKQN-----------DPFPRCQHGGTCVDKIG   182
NP_001245510.1[D.melanogaster]      TNTHGSYSCICVN--GWAGLDCSNNTDDCKQA---------------ACFYGATCIDGVG   392
XP_019643529.1[B.belcheri]          HNTVGGYSCVCVN--GWIGDDCSENFDDCATA---------------ACFHGATCHDRVG   352
XP_032815028.1[P.marinus]           HNTHGGYNCVCVN--GWTGEDCSENIDDCAIA---------------ACFNGATCHDRVA   360
XP_014025097.1[S.salar]             SNIQGSYTCVCVN--GWSGLDCSENIDDCATA---------------ACTKGSTCIDRVA   362
XP_018114267.1[X.laevis]            TNSNGGYNCVCVN--GWSGDDCSQNIDDCATA---------------ACANGSTCIDRVA   354
NP_001238962.2[G.gallus]            TNHNGGYACVCVN--GWSGDDCSKNIDDCFTA---------------SCANGSTCIDRVA   361
NP_035058.2[M.musculus]             TNRNGGYGCVCVN--GWSGDDCSENIDDCAYA---------------SCTPGSTCIDRVA   358
Q04721.3[H.sapiens]                 ANRNGGYGCVCVN--GWSGDDCSENIDDCAFA---------------SCTPGSTCIDRVA   358
XP_009243797.2[P.abelii]            ANRNGGYGCVCVN--GWSGDDCSENIDDCAFA---------------SCTPGSTCIDRVA   358
                                     .  ..: *         .   . : .:*                  *  *    :

XP_012554603.1[H.vulgaris]          SYCKLCPSGGIYLGMEGA---------------------------TSEANCPSKNACDE   1688
XP_019853114.1[A.queenslandica]     GFSCGCLPGYSGTQCEIDSCSSQPCQNGDTCIANGLTYSCVCSLDYTDENCTTFITPCYF   409
XP_032230096.1[N.vectensis]         SYTCICPPGKTGLVCNFD------------------------------------DECAS   205
NP_001245510.1[D.melanogaster]      SFYCQCTKGKTGLLCHLD--------------------------------------DACTS   415
XP_019643529.1[B.belcheri]          FFMCECAPGKTGLLCHLD--------------------------------------DACES   375
XP_032815028.1[P.marinus]           SFFCQCPLGKTGLLCHLD--------------------------------------DACVS   383
XP_014025097.1[S.salar]             SFLCVCPYGKTGLLCHVN--------------------------------------DACIS   385
XP_018114267.1[X.laevis]            SFICVCPEGKIGLLCHKN--------------------------------------DACFS   377
NP_001238962.2[G.gallus]            SFSCICPEGKAGLLCHLD--------------------------------------DACVS   384
NP_035058.2[M.musculus]             SFSCLCPEGKAGLLCHLD--------------------------------------DACIS   381
Q04721.3[H.sapiens]                 SFSCMCPEGKAGLLCHLD--------------------------------------DACIS   381
XP_009243797.2[P.abelii]            SFSCMCPEGKAGLLCHLD--------------------------------------DACIS   381
                                     :   *  *      .                                         *

XP_012554603.1[H.vulgaris]          KPCRNGATCNAGN--YSYTCTCKPGWTGVNCEVDIDEC---ASSPCGRHGTCTNLINDFN   1743
XP_019853114.1[A.queenslandica]     EPCLNGATCINEDL-DNYTCSCLPGFTEEDCSVNIDDC---GSNPCQNEGTCIDLVNDYQ   465
XP_032230096.1[N.vectensis]         NPCSANATCVTS-FSGKASCICNSGWTGKNCDVDIKECEG-DSSPCYHGGTCRDIPGSFV   263
NP_001245510.1[D.melanogaster]      NPCHADAICDTSPINGSYACSCATGYKGVDCSEDIDECDQ--GSPCEHNGICVNTPGSYR   473
XP_019643529.1[B.belcheri]          SPCNEGAICDTNPVNGQPICTCPDGYAGQLCMQDIDECSL-GENPCEHDGECNNVPGSFT   434
XP_032815028.1[P.marinus]           DPCHEGAICDTNPINGKAICTCRAGFTGGACNQDVDECSI-GANPCEHGGRCINTQGSFR   442
XP_014025097.1[S.salar]             SPCRDGAQCDTNPINGMFNCNCAPGYIGSTCNDDVNECII-GPNPCEHGGSCVNTDGSFT   444
XP_018114267.1[X.laevis]            NPCHIGSLCDTNPLTGQAICTCPPGYKGTACTEDIDECSLVNSNPCEHAGKCVNTEGSFY   437
NP_001238962.2[G.gallus]            NPCQKGALCDTNPVNGHYICTCPQGHKGADCTEDVDECAMANSNPCEHAGKCVNTEGSFH   444
NP_035058.2[M.musculus]             NPCHKGALCDTNPLNGQYICTCPQGYKGADCTEDVDECAMANSNPCEHAGKCVNTDGAFH   441
Q04721.3[H.sapiens]                 NPCHKGALCDTNPLNGQYICTCPQGYKGADCTEDVDECAMANSNPCEHAGKCVNTDGAFH   441
XP_009243797.2[P.abelii]            NPCHKGALCDTNPLNGQYICTCPQGYKGADCTEDVDECAMANSNPCEHAGKCVNTDGAFH   441
                                    .**  .: *           * *  *      *  ::.:*      .** . * * :  . :

XP_012554603.1[H.vulgaris]          CTCIKGIIGKQCETNIDDCKNDTCRNGGICNDLVDDFKCLCAAGFEGKRCEINKNECEPN   1803
XP_019853114.1[A.queenslandica]     CDCSAGYTGSDCQTDIDECLMTPCFNNGTCTDLVNSVSCECPPGFNGSLCQNNINECSSS   525
XP_032230096.1[N.vectensis]         CDCVPGFAGARCEDNINECESNPCVH-GLCLDYQNKFECACSKGYTGRLCDVEINECDSN   322
NP_001245510.1[D.melanogaster]      CNCSQGFTGPRCETNINECESHPCQNEGSCLDDPGTFRCVCMPGFTGTQCEIDIDECQSN   533
XP_019643529.1[B.belcheri]          CTCTDGFTGDRCEVNINECASNPCQNQGTCIDDIGEFRCACMPGFAGDLCETDVDECASS   494
XP_032815028.1[P.marinus]           CQCTAGFTGPRCETDINECNSMPCLNDATCLDKIGEFTCICMPGFEGRHCEIDINECQSG   502
XP_014025097.1[S.salar]             CNCARGYDGPRCETDINECASSPCQNDGTCLDRIGDYSCICMEGFEGTHCEIDINECASS   504
XP_018114267.1[X.laevis]            CECSKGFTGPRCEMDINECLSEPCQNDATCLDKIGGFTCLCMPGYKGIHCEIEVDECLSN   497
NP_001238962.2[G.gallus]            CECLKGYTGPRCEMDINECHSNPCQNDATCLDKIGGFTCLCMPGFKGVHCEEDIDECLSN   504
NP_035058.2[M.musculus]             CECLKGYAGPRCEMDINECHSDPCQNDATCLDKIGGFTCLCMPGFKGVHCELEVNECQSN   501
Q04721.3[H.sapiens]                 CECLKGYAGPRCEMDINECHSDPCQNDATCLDKIGGFTCLCMPGFKGVHCELEINECQSN   501
XP_009243797.2[P.abelii]            CECLKGYAGPRCEMDINECHSDPCQNDATCLDKIGGFTCLCMPGFKGVHCELEINECQSN   501
                                    * *  *  *  *: :*::*    * : . * *  .   * *  *: *  *: : :**  .

XP_012554603.1[H.vulgaris]          PCLKNSTCEDLINDFKCNCIPGYVGKLCDVDIDECAMSPCFNNATCVDKVNAFECKCQPG   1863
XP_019853114.1[A.queenslandica]     PCSSGGSTCIDEIDDFTCLCPMGLTGTQCDISIIDCSDMPCGNNGTCTDTPGGYECSCSSG   585
XP_032230096.1[N.vectensis]         PCLNGGQCHDGLGNYSCTCQVGYVGEICQTNYDDCASNPCQNDGTCQDGIAQYTCLCPLG   382
NP_001245510.1[D.melanogaster]      PCLNDGTCHDKINGFKCSCALGFTGARCQINIDDCQSQPCRNRGICHDSIAGYSCECPPG   593
XP_019643529.1[B.belcheri]          PCLN-GICRDGINKYECECDPGYEGTTCEVNINECASGPCRNGAHCTDLVTAYTCTCLEG   553
XP_032815028.1[P.marinus]           PCLNDGNCTDHVNGFLCRCPPGFTGPMCQIDMDECASTPCLNGAKCVDRPNAYSCECTEG   562
XP_014025097.1[S.salar]             PCLNQGTCLDQVKRYVCQCPLGFSGEMCQIDIDECSSTPCLNGAKCIDRPNGYECECAEG   564
XP_018114267.1[X.laevis]            PCVNNGVCVDKVNRFVCICPPGFTGPVCQIDIDDCSSTPCQNGAKCVDHPAGYDCLCATG   557
NP_001238962.2[G.gallus]            PCVNNGECLDKVNRFLCVCPPGFSGAVCQIDIDDCSSTPCLNGAKCIDHPNGYECQCATG   564
NP_035058.2[M.musculus]             PCVNNGQCVDKVNRFQCLCPPGFTGPVCQIDIDDCSSTPCLNGAKCIDHPNGYECQCATG   561
Q04721.3[H.sapiens]                 PCVNNGQCVDKVNRFQCLCPPGFTGPVCQIDIDDCSSTPCLNGAKCIDHPNGYECQCATG   561
XP_009243797.2[P.abelii]            PCVNNGQCVDKVNRFQCLCPPGFTGPVCQIDIDDCSSTPCLNGAKCIDHPNGYECQCATG   561
                                    ** . . * * :  : * *  *  *  *: .  :*   ** * . * *     : * *  *

XP_012554603.1[H.vulgaris]          FNGTLC------------------------------------ETDIDECSTQPCANNGS   1886
XP_019853114.1[A.queenslandica]     YTGVHCMVNINDCLPHPCNNGTCIDGINEYVCICPEDYTGDNCETPIDHCDSNPCSSLAT   645
XP_032230096.1[N.vectensis]         FTGKSC------------------------------------------------------   388
NP_001245510.1[D.melanogaster]      YTGTSCEININDCDSNPCHRGKCIDDVNSFKCLCDPGYTGYICQKQINECESNPCQFDGH   653
XP_019643529.1[B.belcheri]          FTGTDCEINIDDCQSNQCQHGTCVDEVASFTCSCEPGYNGPLCESPVDECDSDPCQNGGT   613
XP_032815028.1[P.marinus]           FAGLVCELNVNECQPDPCHHGKCVDGIASFYCVCSPGYTGFLCDSQLNECQSSPCQNGGK   622
XP_014025097.1[S.salar]             FSGPLCKENIDDCDPEPCHHGVCRDGIATFSCDCDPGYTGSICNVQVMECHSNPCQNRGR   624
XP_018114267.1[X.laevis]            FTGVLCEENINNCEPPPCHYGTCQDGIDSYTCICEPGYMGAICSDQIDECHSNPCLNEGR   617
NP_001238962.2[G.gallus]            FTGVLCEENINNCDPDPCHHGECQDGIDSYTCICNPGYMGAICSEQINECHSNPCLHQGR   624
NP_035058.2[M.musculus]             FTGILCDENIDNCDPDPCHHGQCQDGIDSYTCICNPGYMGAICSDQIDECYSSPCLNDGR   621
```

```
Q04721.3[H.sapiens]               FTGVLCEENIDNCDPDPCHHGQCQDGIDSYTCICNPGYMGAICSDQIDECYSSPCLNDGR   621
XP_009243797.2[P.abelii]          FTGVLCEENIDNCDPDPCHHGQCQDGIDSYTCICNPGYMGAICSDQIDECYSSPCLNDGR   621
                                  : *  *

XP_012554603.1[H.vulgaris]        CIDIVNGFLCKCIDGFRGSNCSINIDECDPSPCLHNSTCVDQINGFQCECSPGYFGLRCE   1946
XP_019853114.1[A.queenslandica]   CITNPGGYQCICPIDFTGTDCFDQINDCQPNPCNNGGTCSDLIGTFNCSCPLGFEGSICE   705
XP_032230096.1[N.vectensis]       ---------------------ETNTNECAGNPCMNMGTCIDGINSFRCQCPLGFTGNRCE   427
NP_001245510.1[D.melanogaster]    CQDRVGSYYCQCQAGTSGKNCEVNVNECHSNPCNNGATCIDGINSYKCQCVPGFTGQHCE   713
XP_019643529.1[B.belcheri]        CEDLVNGYRCNCLPGTSGDNCEVNQDDCTGNLCVHG-VCQDGLNTYTCMCDGGYEGENCE   672
XP_032815028.1[P.marinus]         CVDLVNRYHCQCLEGTEGVNCETNFDDCAGELCQRG-KCVDGVNEYRCLCDAGYTGLQCD   681
XP_014025097.1[S.salar]           CIDLVNKYQCNCLPGTSGVNCEFNVDDCASNPCEYG-ECQDGINEYKCVCAPGYTGAKCD   683
XP_018114267.1[X.laevis]          CVDLVNGYQCNCLPGTSGPRCEKNVDDCASNPCTYG-NCVDGINRYDCVCSPGFTGPQCK   676
NP_001238962.2[G.gallus]          CIDLVNGYQCNCLPGTSGVNCENNFDDCASNPCVHG-DCIDGINRYNCACKPGFTGPRCN   683
NP_035058.2[M.musculus]           CIDLVNGYQCNCQPGTSGLNCEINFDDCASNPCMHG-VCVDGINRYSCVCSPGFTGQRCN   680
Q04721.3[H.sapiens]               CIDLVNGYQCNCQPGTSGVNCEINFDDCASNPCIHG-ICMDGINRYSCVCSPGFTGQRCN   680
XP_009243797.2[P.abelii]          CIDLVNGYQCNCQPGTSGVNCEINFDDCASNPCIHG-ICMDGINRYSCVCSPGFTGQRCN   680
                                                       : ::*  . *     * * :. : * *  *: *  *.

XP_012554603.1[H.vulgaris]        TEINECESQPCSNNATCVDKINDYQCLCNIGFEGKQCEIDINECQPNPCVN-GSCKDLVG   2005
XP_019853114.1[A.queenslandica]   YDINECASLPCLNGGNCTDLVNGYSCSCPHGFNGT-------------------------   740
XP_032230096.1[N.vectensis]       TEIDECESSPCQNGGTCKDKINGYVCICPPGASG--------------------------   461
NP_001245510.1[D.melanogaster]    KNVDECISSPCANNGVCIDQVNGYKCECPRGFYDAHCLSDVDECASNPCVNEGRCEDGIN   773
XP_019643529.1[B.belcheri]        SEIDECASSPCHNGGICHDLVNAFSCECPPGYHDLLCYSNVNECESSPCEH-GTCQDGIN   731
XP_032815028.1[P.marinus]         VELNECESSPCHNGGSCKDLVDAFECVCLPGFHGPLCYSETDACYASPCLH-GTCLDHGE   740
XP_014025097.1[S.salar]           VDIHECNSSPCMSGGTCVDKVNGFICQCPPGTHGPLCHSGTDHCAPQPCVH-GDCVEQQS   742
XP_018114267.1[X.laevis]          DDIDECASSPCHNGGTCVNEQNRFRCICPEGFRQPSCFSQVDECVNNPCIH-GNCTDDVN   735
NP_001238962.2[G.gallus]          VDIDECASSPCNNGGTCINEVNGFRCVCPEGYHHPHCQSQADGCLSNPCVH-GNCTHIVS   742
NP_035058.2[M.musculus]           IDIDECASNPCRKGATCINDVNGFRCICPEGPHHPSCYSQVNECLSNPCIH-GNCTGGLS   739
Q04721.3[H.sapiens]               IDIDECASNPCRKGATCINGVNGFRCICPEGPHHPSCYSQVNECLSNPCIH-GNCTGGLS   739
XP_009243797.2[P.abelii]          IDIDECASNPCRKGATCINGVNGFRCICPEGPHHPSCYSQVNECLSNPCIH-GNCTGGLS   739
                                   ::.** * ** ... * :  : : * *  *

XP_012554603.1[H.vulgaris]        DYLCECQPGFDGRNCSNL-IDNCFSLPCKNNGNCTNKVNNYTCTCQAGFSGSDCETNINE   2064
XP_019853114.1[A.queenslandica]   -------------NCENSTITTCESVVCENGGMCDDTPTGFECLCPNGYTGPYCQNNIDD   787
XP_032230096.1[N.vectensis]       ------------THCEND-PNDCPANACQNGGVCIDGMNTYSCKCHPGFTGFSCGVPVNE   508
NP_001245510.1[D.melanogaster]    EFICHCPPGYTGKRCELD-IDECSSNPCQHGGTCYDKLNAFSCQCMPGYTGQKCETNIDD   832
XP_019643529.1[B.belcheri]        DYTCTCETGYEGKNCDVN-IDECASNPCQHEGQCDDGIGRYECQCLPGYEGVNCQINTDE   790
XP_032815028.1[P.marinus]         SYRCACEAGWTGPLCEVD-IDECESSPCQNGGACQEHLDGYTCRCRDGFKGPTCQKNVNE   799
XP_014025097.1[S.salar]           GYNCECDSGWVGQHCDQE-KDECQSSPCQHSGSCVDRLNGYSCQCRPGFTGVNCEFNMEE   801
XP_018114267.1[X.laevis]          GYKCLCQPGWNGKKCEVD-KNECLSDPCQNGGTCDDLVNGYRCSCKKGFKGYNCQIDTNE   794
NP_001238962.2[G.gallus]          GYKCVCDPGWIGDYCSTE-GNECKSNPCQNGGTCEDLLNGYRCTCRKGFKGVNCQ-----   796
NP_035058.2[M.musculus]           GYKCLCDAGWVGVNCEVD-KNECLSNPCQNGGTCNNLVNGYRCTCKKGFKGYNCQVNIDE   798
Q04721.3[H.sapiens]               GYKCLCDAGWVGINCEVD-KNECLSNPCQNGGTCDNLVNGYRCTCKKGFKGYNCQVNIDE   798
XP_009243797.2[P.abelii]          GYKCLCDAGWIGINCEVD-KNECLSNPCQNGGTCDNLVNGYRCTCKKGFKGYNCQVNIDE   798
                                               *.      * :  *:: * * :    : * *  *: *  *

XP_012554603.1[H.vulgaris]        CDPDPCNSNALNCTDLINGYICYCKLGFRGENCSEIIDNCNPQPCRHNSTCTNRFVTND-   2123
XP_019853114.1[A.queenslandica]   CLSNPCLNN-ATCIDEIANYTCECTEGFDGRNCAIDTDLCSPQPCFNGGTCSETSD--SF   844
XP_032230096.1[N.vectensis]       CASSPCRNG-GTCEDGVAQYYCRCRDGYTGKNCEVRIDSCIDHTCQNGASCVSSTP----   563
NP_001245510.1[D.melanogaster]    CVTNPCGNG-GTCIDKVNGYKCVCKVPFTGRDCESKMDPCASNRCKNEAKCTPSSNFLDF   891
XP_019643529.1[B.belcheri]        CGSNPCQNG-GQCLDGVNNYQCHCELPFVGINCQIELAPCNPNPCANSGSCIPSADYQTF   849
XP_032815028.1[P.marinus]         CVSNPCLNR-GTCVDGVAGYTCLCTLPFTGRNCEAVMAPCASQPCHNAATCKESVNLSSF   858
XP_014025097.1[S.salar]           CASSPCENH-GTCVDGVNTYSCLCDPPYSGKHCEEELVPCASHPCERGGVCQPTPDYTFY   860
XP_018114267.1[X.laevis]          CASNPCLNH-GTCRDGINGYTCHCALPYTGTNCQNILTPCSPEPCHNGGICQESEDFKSF   853
NP_001238962.2[G.gallus]          ----------------------------------VVVAPCSPDPCENSGICQESPDSEGY   822
NP_035058.2[M.musculus]           CASNPCLNQ-GTCFDDVSGYTCHCMLPYTGKNCQTVLAPCSPNPCENAAVCKEAPNFESF   857
Q04721.3[H.sapiens]               CASNPCLNQ-GTCFDDISGYTCHCVLPYTGKNCQTVLAPCSPNPCENAAVCKESPNFESY   857
XP_009243797.2[P.abelii]          CASNPCLNQ-GTCFDDISGYTCHCVLPYTGKNCQTVLAPCSPNPCENAAVCKESPNFESY   857
                                                                        *  . * . . *

XP_012554603.1[H.vulgaris]        ------------------------------------------------------------   2123
XP_019853114.1[A.queenslandica]   FCTCPLGYFGSHCQNTLDPCS-SSPCLNSGICTNVNGTNFSCACSQAYGGERCEIKLFPD   903
XP_032230096.1[N.vectensis]       ------------------------------------------------------------   563
NP_001245510.1[D.melanogaster]    SCTCKLGYTGRYCDEDIDECSLSSPCRNGASCLNVPG-SYRCLCTKGYEGRDCAIN-TDD   949
XP_019643529.1[B.belcheri]        TCDCAEGFEGETCADDINECQ-SNPCKNGAPCINLEG-DFRCDCLTGFSGELCSVN-IDD   906
XP_032815028.1[P.marinus]         SCACPPGWRGATCSLDIDECA-GAPCQHGGACMNTQG-GFRCSCRPGYTGPTCQTD-IDD   915
XP_014025097.1[S.salar]           TCRCPRGWQGPRCTEDVDECR-KNPCQNRARCINSQG-SYVCKCRPGYSGLNCQTN-IDD   917
XP_018114267.1[X.laevis]          SCLCATGWQGQRCTIDVDECV-TNPCRNYGRCQNTKG-GYKCHCPDGFSGFNCENN-IDD   910
NP_001238962.2[G.gallus]          TCQCAPGWEGERCTVDIDECL-SKPCKNHALCHNIQG-SYLCECRPGFTGGDCDSN-IDD   879
NP_035058.2[M.musculus]           SCLCAPGWQGKRCTVDVDECI-SKPCMNNGVCHNTQG-SYVCECPPGFSGMDCEED-IND   914
Q04721.3[H.sapiens]               TCLCAPGWQGQRCTIDIDECI-SKPCMNHGLCHNTQG-SYMCECPPGFSGMDCEED-IDD   914
XP_009243797.2[P.abelii]          TCLCAPGWQGQRCTIDIDECI-SKPCMNHGLCHNTQG-SYMCECPPGFSGMDCEED-IDD   914


XP_012554603.1[H.vulgaris]        --------------------------------FHCNCSLGYEGDRCEIEIDECKALPCKN   2151
XP_019853114.1[A.queenslandica]   CLDMPCLNNGTCAELVGSGNIGGSGAGEPGPRIYCQCPLGYAGEFCENITDLCVSSPCKN   963
XP_032230096.1[N.vectensis]       ----------------Y--------------AYSCQCKPGYTGQYCETDVDECAARPCVN   593
NP_001245510.1[D.melanogaster]    CASFPCQNGGTCLDGIG--------------DYSCLCVDGFDGKHCETDINECLSQPCQN   995
XP_019643529.1[B.belcheri]        CDPDPCHNGGTCNDGIN--------------SYTCSCMPGFGGTNCEEDIDECYSNPCQN   952
XP_032815028.1[P.marinus]         CSPNPCQNGGSCTDGVG--------------AFSCKCRPGFRGSRCETEVNECASNPCRN   961
XP_014025097.1[S.salar]           CSPNPCLNGGSCVDEVG--------------GFSCDCRQGFDGERCEAEVDECASQPCQN   963
XP_018114267.1[X.laevis]          CLSNPCQNGASCLDGIN--------------TFSCNCLAGFHGDKCQRDVDECASNPCKN   956
NP_001238962.2[G.gallus]          CLSNPCQNGASCVDGIN--------------SFSCICLPGFHGDKCQTDTNECLSEPCRN   925
NP_035058.2[M.musculus]           CLANPCQNGGSCVDHVN--------------TFSCQCHPGFIGDKCQTDMNECLSEPCKN   960
Q04721.3[H.sapiens]               CLANPCQNGGSCMDGVN--------------TFSCLCLPGFTGDKCQTDMNECLSEPCKN   960
XP_009243797.2[P.abelii]          CLANPCQNGGSCVDGVN--------------AFSCLCLLGFTGDKCQTDMNECLSEPCKN   960
                                                                 * *  *: *  *:   : * : ** *

XP_012554603.1[H.vulgaris]        GGNCTDLPGKYLCTCPPGFTGVDCEINIDDCKN--VSCLNGGKCIDLINSYVCECPVTHK   2209
XP_019853114.1[A.queenslandica]   NATCIGDSANFTCTCLPGFTGTLCETELTGCHTELYPCLNGGECMEMDGQFMCNCAPGFT   1023
XP_032230096.1[N.vectensis]       G-ACVDGVNGFICRCDPGFTGDRCQINVDDCQS--SPCVHGGSCIDSINTYTCQCPKGFT   650
NP_001245510.1[D.melanogaster]    GATCSQYVNSYTCTCPLGFSGINCQTNDEDCTE--SSCLNGGSCIDGINGYNCSCLAGYS   1053
XP_019643529.1[B.belcheri]        GGQCIDAVNGYACDCVLGFIGTNCQTNKDDCTS--SSCFSGGTCIDGINTFTCQCPPGFT   1010
XP_032815028.1[P.marinus]         GGSCADYVNSYTCRCRPGFNGIHCEHDITECTD--SSCLNGGSCVDGINAYTCLCRGGFT   1019
XP_014025097.1[S.salar]           GAVCRDYVNSFVCVCRPGFDGILCEHNIPECTE--SSCLNNGTCVDDINTFSCRCRPGFY   1021
```

```
XP_018114267.1[X.laevis]            GGSCTDYVNSYTCKCQPGFDGIHCDNNIDECTD--TSCFNGATCVDGVNSFSCQCPQGFT   1014
NP_001238962.2[G.gallus]            GGTCTHYVNSYTCKCQPGFEGTNCENNIDECTE--SSCFNGGTCVDGINSFTCQCPVGFT   983
NP_035058.2[M.musculus]             GGTCSDYVNSYTCTCPAGFHGVHCENNIDECTE--SSCFNGGTCVDGINSFSCLCPVGFT   1018
Q04721.3[H.sapiens]                 GGTCSDYVNSYTCKCQAGFDGVHCENNINECTE--SSCFNGGTCVDGINSFSCLCPVGFT   1018
XP_009243797.2[P.abelii]            GGTCSDYVNSYTCKCQAGFDGVHCENNINECTE--SSCFNGGTCVDGINSFSCLCPVGFT   1018
                                    .  *      : * *  ** *  *: :   *      *. .. *::  . : * *   .

XP_012554603.1[H.vulgaris]          GKHCEKKIPDDVDI------TFPGGDGSYIKHCVKGNASKEFSISLWFRFLGRSRGVFLN   2263
XP_019853114.1[A.queenslandica]     GPLCGYGINECRNQPCLNGGTCRDFFRYYVCICPPNFTGTDCES-------------MID   1070
XP_032230096.1[N.vectensis]         GPRCEIHINECSSDPCQHGGTCSDRIGSYSCYCRPGYTGSNCQH-------------PLD   697
NP_001245510.1[D.melanogaster]      GANCQYKLNKCDSNPCLNGATCHEQNNEYTCHCPSGFTGKQCSE-------------YVD   1100
XP_019643529.1[B.belcheri]          GSNCQHEINECESHPCQNGATCVDQTGYFSCICTYGYEGVTCQN-------------QKD   1057
XP_032815028.1[P.marinus]           GSYCQFDIDECESQPCQHGGTCLDGLGTYKCTCPRGYTGGNCQA-------------LVD   1066
XP_014025097.1[S.salar]             GTFCEYEQNECDSQPCKNGGTCTDGLGSYRCTCPVGYNGQNCQN-------------FVN   1068
XP_018114267.1[X.laevis]            GPFCLFEINECGSHPCINGGTCVDGMGTYHCTCPIGYRGKNCEE-------------LVD   1061
NP_001238962.2[G.gallus]            GPFCLMEINECDSHPCLNKGSCVDSLGKYRCICPLGYTGKNCQT-------------PMD   1030
NP_035058.2[M.musculus]             GPFCLHDINECSSNPCLNAGTCVDGLGTYRCICPLGYTGKNCQT-------------LVN   1065
Q04721.3[H.sapiens]                 GSFCLHEINECSSHPCLNEGTCVDGLGTYRCSCPLGYTGKNCQT-------------LVN   1065
XP_009243797.2[P.abelii]            GSFCLHEINECSSHPCLNEGTCVDGLGTYRCSCPLGYTGKNCQT-------------LVN   1065
                                    *  *     .  .       :       :   *  .  .   .                :

XP_012554603.1[H.vulgaris]          L--GFGCETVRPFLIMSHSSIRFQFRNQFSFVMIDNLKINNEKLVINNGVWHHIYIALSS   2321
XP_019853114.1[A.queenslandica]     PCTNIDCNNGSCI---------GDM---------------GTYTCQCDPGWTGLQCESEI   1106
XP_032230096.1[N.vectensis]         RCANDPCRNGATC---------RRTGEDL-----------SDFHCECPLGYKGTICDVKE   737
NP_001245510.1[D.melanogaster]      WCGQSPCENGATC---------SQMK--------------HQFSCKCSAGWTGKLCDVQT   1137
XP_019643529.1[B.belcheri]          LCASDPCRNGGTC---------TQSG--------------DRYECLCEDEWTGLICDMTK   1094
XP_032815028.1[P.marinus]           WCSSSPCKNSGSC---------VQSR--------------TSYRCDCHSGWTGLYCDIPN   1103
XP_014025097.1[S.salar]             LCSQPLCQNGGSC---------SQSE--------------TTWLCHCPVGWTGMYCDVPN   1105
XP_018114267.1[X.laevis]            LCNGFPCKNKGIC---------KQVK--------------TEPKCICPTGWTGIYCDIPD   1098
NP_001238962.2[G.gallus]            LCSKSPCKNKGTC---------FQSG--------------AQTRCDCPSGWTGAYCDVPN   1067
NP_035058.2[M.musculus]             LCSRSPCKNKGTC---------VQEK--------------ARPHCLCPPGWDGAYCDVLN   1102
Q04721.3[H.sapiens]                 LCSRSPCKNKGTC---------VQKK--------------AESQCLCPSGWAGAYCDVPN   1102
XP_009243797.2[P.abelii]            LCSRSPCKNKGTC---------VQKK--------------AESQCLCPSGWAGAYCDVPN   1102
                                          *..                                     :

XP_012554603.1[H.vulgaris]          VTLNFKVVLDGIIVQEEQKAELSGMTFFNESGTILIGKSLLDGKLIESGFIGDVSQVALF   2381
XP_019853114.1[A.queenslandica]     NECDGVD----------C-----------TNGTCV-DLINNYTCQCSDGFTGQFCEINID   1144
XP_032230096.1[N.vectensis]         VSCAVA--------GTIC----------ANGGTCF-DSNGVQSCTCKPGFTGSYCRTNID   778
NP_001245510.1[D.melanogaster]      ISCQDAADRKGLSLRQLC----------NN-GTCK-DYGNSHVCYCSQGYAGSYCQKEID   1185
XP_019643529.1[B.belcheri]          VSCAAAASERGVLLGELC----------QNGGSCV-DTGNSHNCNCPAGYRGSYCSEEID   1143
XP_032815028.1[P.marinus]           MSCDTAARNKGLSVSHLC----------RHGGRCI-DAGNTHHCQCSRGFEGSYCEKEVD   1152
XP_014025097.1[S.salar]             MSCQDYAARNGIQVELVC----------KHSGRCV-NVGNAHQCQCLPGYTGSYCNEMVD   1154
XP_018114267.1[X.laevis]            VSCEVAASQRGVAVQDLC----------QHAGVCI-NTGFSHRCQCRQGYIGSYCEGELD   1147
NP_001238962.2[G.gallus]            VSCQVAASQRGITVDQLC----------QHSSHCL-NVGNTHRCQCRVGYTGSYCEEQLD   1116
NP_035058.2[M.musculus]             VSCKAAALQKGVPVEHLC----------QHSGICI-NAGNTHHCQCPLGYTGSYCEEQLD   1151
Q04721.3[H.sapiens]                 VSCDIAASRRGVLVEHLC----------QHSGVCI-NAGNTHYCQCPLGYTGSYCEEQLD   1151
XP_009243797.2[P.abelii]            VSCDIAASSRGVLVEHLC----------QHSGVCI-NAGNTHYCQCPLGYTGSYCEEQLD   1151
                                                               .    .           *: *. .   :

XP_012554603.1[H.vulgaris]          KRV-------------LDS---TEIAGL-VRNCSRNISDSIIPWVGLMADAGPNVSLLET   2424
XP_019853114.1[A.queenslandica]     DCAGINCNNG-TCVDGIGSYQCDCLLGYTGPSCDTIIDDCAG---MPCMNNASCIDLFNN   1200
XP_032230096.1[N.vectensis]         ECAKGPCKYGATCHDAVANYTCTCTAGFTGKNCDININECAS---NPCQR-GSCLDLVNG   834
NP_001245510.1[D.melanogaster]      ECQSQPCQNGGTCRDLIGAYECQCRQGFQGQNCELNIDDCAP---NPCQNGGTCHDRVMN   1242
XP_019643529.1[B.belcheri]          ECASSPCQNGAQCRDGLGTYSCVCRAGYQGVNCEQEINECIS---NPCQNGGTCIDMVNE   1200
XP_032815028.1[P.marinus]           ECTSNPCHNGATCRNFLGSYKCECPVGYHGLNCDYEINECLS---QPCQNGGKCIDLVNR   1209
XP_014025097.1[S.salar]             ECLSNPCRNGATCMDYQGTYECMCKAGYQGVNCEYDVDECHS---NPCHHGGTCINLINR   1211
XP_018114267.1[X.laevis]            ECASNPCQNSAACVDRHGGYECKCLPGYQGVNCEYEIDECQL---QPCHNGGTCVDLVNQ   1204
NP_001238962.2[G.gallus]            ECDSSPCQNGATCRDHLGGYQCECVPGYQGVNCEYEVDECQF---QPCQNGGTCIDLVNH   1173
NP_035058.2[M.musculus]             ECASNPCQHGATCNDFIGGYRCECVPGYQGVNCEYEVDECQN---QPCQNGGTCIDLVNH   1208
Q04721.3[H.sapiens]                 ECASNPCQHGATCSDFIGGYRCECVPGYQGVNCEYEVDECQN---QPCQNGGTCIDLVNH   1208
XP_009243797.2[P.abelii]            ECVSNPCQHGATCSDFIGGYRCECVPGYQGVNCEYEVDECQN---QPCQNGGTCIDLVNH   1208
                                    .                         *    .*.  :.:.           .   . .

XP_012554603.1[H.vulgaris]          WTCGTNVCPPEFTGKFCQTKIDKIPPTVVF------------CPSPIKVYTEESSVFVTW   2472
XP_019853114.1[A.queenslandica]     YTC---VCSDGFTGRFCEVNIDDCLNI---------NCNNGSCEDLINDHM--CNCFPGF   1246
XP_032230096.1[N.vectensis]         YLC---SCPKGYIGKHCEVNADDCFVN--------ACFNGGSCVDGIAEFK--CTCPLGF   881
NP_001245510.1[D.melanogaster]      FSC---SCPPGTMGIICEINKDDCKPG--------ACHNNGSCIDRVGGFE--CVCQPGF   1289
XP_019643529.1[B.belcheri]          YRC---SCPPGTQGLLCEINDNCFAG--------ACYHDGTCVDGIGEFT--CRCRPGY   1247
XP_032815028.1[P.marinus]           FNC---VCPPGTQGILCEINTDDCNPGPDAPMPGPRCFNGGSCVDGVGSFT--CSCPPGF   1264
XP_014025097.1[S.salar]             FSC---ACPPGTNGVQCEVNVDDCAPKPG--SWELRCLNGGQCVDGVGRYT--CSCPPGF   1264
XP_018114267.1[X.laevis]            FRC---SCPPGTRGALCEENIDDCAPSAE----GPHCFNGGRCIDRIGGYR--CECLPGF   1255
NP_001238962.2[G.gallus]            FRC---SCPPGTRGRLCEENVDDCISESG----GPRCFNGGQCIDQIGGYS--CLCLPGF   1224
NP_035058.2[M.musculus]             FKC---SCPPGTRGLLCEENIDEC---AG----GPHCLNGGQCVDRIGGYT--CRCLPGF   1256
Q04721.3[H.sapiens]                 FKC---SCPPGTRGLLCEENIDDC---AR----GPHCLNGGQCMDRIGGYS--CRCLPGF   1256
XP_009243797.2[P.abelii]            FKC---SCPPGTRGLLCEENIDDC---AR----GPHCLNGGQCMDRIGGYS--CRCLPGF   1256
                                    : *     *      *  *: : *.                  * . :  .   .     :

XP_012554603.1[H.vulgaris]          KEPQFDDDVAVVEVIRSHSPNGLFSWGDYVITYI-------------------AK-DAFE   2512
XP_019853114.1[A.queenslandica]     TDQRCETDINECDGN-PCNDG---TCTDGINSFSCSCPPDYTGDTCDTEINLCLMEQPCL   1302
XP_032230096.1[N.vectensis]         SGSRCEVDVDECASS-PCSALGTEKCINNIGAYHCQCKQGFLGRHCDLNINECLS-YPCR   939
NP_001245510.1[D.melanogaster]      VGARCEGDINECLSN-PCSNAGTLDCVQLVNNYHCNCRPGHMGRHCEHKVDFCAQ-SPCQ   1347
XP_019643529.1[B.belcheri]          VGPRCEGDVNECLSN-PCDAEGTQDCVQLENDYRCDCKPGYTGGRCERTVDSCES-DPCL   1305
XP_032815028.1[P.marinus]           VGERCEGDVNECLSN-PCDPRGSLDCIQLVNDYKCQCRPGFTGHNCEVVINTCRL-EPCR   1322
XP_014025097.1[S.salar]             AGEHCEGDVNECLSG-PCHSPGSLDCVQLANDYQCRCRLGYTGRHCESMVDLCQS-KPCH   1322
XP_018114267.1[X.laevis]            AGERCEGDVNECLSS-PCSSKGSLDCIQLANNYLCKCKPVFTGRHCETIQDLCPS-RPCL   1313
NP_001238962.2[G.gallus]            AGERCEGDINECLSN-PCNPRGSLDCIQLINDYTCICRSAFTGRHCESVIDVCPR-KPCQ   1282
NP_035058.2[M.musculus]             AGERCEGDINECLSN-PCSSEGSLDCVQLKNNYNCICRSAFTGRHCETFLDVCPQ-KPCL   1314
Q04721.3[H.sapiens]                 AGERCEGDINECLSN-PCSSEGSLDCIQLTNDYLCVCRSAFTGRHCETFVDVCPQ-MPCL   1314
XP_009243797.2[P.abelii]            AGERCEGDINECLSN-PCSSEGSLDCIQLTNDYLCVCRSAFTGRHCETFIDVCPQ-MPCL   1314
                                       : : *:                  :    :

XP_012554603.1[H.vulgaris]          NSVSCTFEI-N-VTPFN--------------------CSE-----------FQTANNSIH   2539
XP_019853114.1[A.queenslandica]     NNGTCTSDKIEGIPIYNCSCSVPLYSGDNCEQIN--SCSLSPCQNNATCTGNLTTGDYTC   1360
XP_032230096.1[N.vectensis]         NGGECKDGA----GEYTCLCP-HGFSGDDCERRV-YTCSDQPCLNSGTCRT--VHNTYNC   991
NP_001245510.1[D.melanogaster]      NGGNCNIRQ----SGHHCICN-NGFYGKNCELSG-QDCDS--------------------   1381
```

```
XP_019643529.1[B.belcheri]          NGGACSQSG----NNYVCDCQ-PGFGGDNCELVC-----D--------------------   1335
XP_032815028.1[P.marinus]           NGGTCMITV-NTLLGFTCLCA-PGYAGHACEDNA-YACGN--------------------   1359
XP_014025097.1[S.salar]             NSGTCSMNM-SSVHGYTCICQ-PGFTGFNCGEVEGYNCAK--------------------   1360
XP_018114267.1[X.laevis]            NGGTCAVAV-NMPEGFTCQCP-SGFLGSKCQYSN-NTCEH--------------------   1350
NP_001238962.2[G.gallus]            NGGTCAVAS-NMPDGFICQCP-PGYSGAKCEFSSHSTCGQ--------------------   1320
NP_035058.2[M.musculus]             NGGTCAVAS-NMPDGFICRCP-PGFSGARCQ----SSCGQ--------------------   1348
Q04721.3[H.sapiens]                 NGGTCAVAS-NMPDGFICRCP-PGFSGARCQ----SSCGQ--------------------   1348
XP_009243797.2[P.abelii]            NGGTCAVAS-NMPDGFICRCP-PGFSGARCQ----SSCGQ--------------------   1348
                                    *.  *           .

XP_012554603.1[H.vulgaris]          ACGTWKYGRFCDYKCH---------------------------------------EKY-   2558
XP_019853114.1[A.queenslandica]     HCSENYYGTHCER-FDYCHSNPCQNDGTCINGSPGNLISDTFLCICMPQFNGSDCSMEL-   1418
XP_032230096.1[N.vectensis]         SCRPGVYGKQCEWNKDECLSAPCRNGGTCIDGYGS------YSCKCPLGFAGANCEESI-   1044
NP_001245510.1[D.melanogaster]      --------------------NPCRVGNCVVADEGF-----GYRCECPRGTLGEHCEIDT-   1415
XP_019643529.1[B.belcheri]          --------------------RECKNRGQCLYTDGE------FRCSCPSNYAGDSCQFHE-   1368
XP_032815028.1[P.marinus]           --------------------LSCRNGGTCLPDRAA-----GPLCVCPRAWRGPECQYHSG   1394
XP_014025097.1[S.salar]             --------------------LRCQNGGHCQETQVG-----RPHCRCQPGFSGPRCETVH-   1394
XP_018114267.1[X.laevis]            --------------------YKCKVGENCIQTHMG------PRCYCPMGPAGYACQSNL-   1383
NP_001238962.2[G.gallus]            --------------------VKCKKGEQCIHTSSG------PRCYCPRLAVGEECQTNT-   1353
NP_035058.2[M.musculus]             --------------------VKCRRGEQCIHTDSG------PRCFCLNPK-----DCES-   1376
Q04721.3[H.sapiens]                 --------------------VKCRKGEQCVHTASG------PRCFCPSPR-----DCES-   1376
XP_009243797.2[P.abelii]            --------------------VKCRKGEQCVHTASG------PRCFCPSPR-----DCES-   1376


XP_012554603.1[H.vulgaris]          ------------------ELAVPKPPFYVCGRPGTWSHGPPT--YPENPDLLVSECT---   2595
XP_019853114.1[A.queenslandica]     -----SPCSIDPCMNGGTCVEDG--STRYCQCPVGYTGNNCSEVFSSDTPYFNGDSY---   1468
XP_032230096.1[N.vectensis]         -----NECLSQPCKNGGSCRDI--VNGYKCDCPSKMIGKNCETVV---------------   1082
NP_001245510.1[D.melanogaster]      ----LDECSPNPCAQGAACEDL--LGDYECLCPSKWKGKRCDIYDANYPGWNGGSGSGND   1469
XP_019643529.1[B.belcheri]          ----SNPCFSLPCYNNGECQQTG-IDSYRCMCPEDYNGLVCEIYIPDVTP-----GP---   1415
XP_032815028.1[P.marinus]           GGGGSSACASSPCENGAACYEEVRPPFYRCVCPPLFTGPRCRVLGPKVPGDASS-GR---   1450
XP_014025097.1[S.salar]             ------SCQNRPCLNGGTCMKDP-HYQYSCHCPAHFSGRHCENVIF--------------   1433
XP_018114267.1[X.laevis]            ------GCASQPCKNNGVCQRKAQPPFYTCHCPKGVVGPQCEIPI---------------   1422
NP_001238962.2[G.gallus]            ------GCASAPCQNGGSCHPRSQPPYYYCQCPAHAQGSQCEQLV---------------   1392
NP_035058.2[M.musculus]             ------GCASNPCQHGGTCYPQRQPPHYSCRCPPSFGGSHCELYT---------------   1415
Q04721.3[H.sapiens]                 ------GCASSPCQHGGSCHPQRQPPYYSCQCAPPFSGSRCELYT---------------   1415
XP_009243797.2[P.abelii]            ------GCASSPCQHGGSCHPQRQPPYYSCQCAPPFSGSRCELYT---------------   1415
                                                              *

XP_012554603.1[H.vulgaris]          --ATTPFNTITTNKISFPTSSCDDSFKNDFV----E----S-------FKQLITELQDVW   2638
XP_019853114.1[A.queenslandica]     --TLVPYSPNNNNSQSLINLHFRTSSQSGLLLYISEEDHSSYLSLGIEAGLLSLRMRQEG   1526
XP_032230096.1[N.vectensis]         ---------KDQCPV----PNCAKKFDGGKC----NPKCNT-HECNWDGTTCSLG-IEPW   1123
NP_001245510.1[D.melanogaster]      RYAADLEQQRAMCDK----RGCTEKQGNGIC----DSDCNT-YACNFDGNDCSLG-INPW   1519
XP_019643529.1[B.belcheri]          GVTMIPPHIEPSCPF----EHCASKARDGRC----DEECNI-HECDWDGTDCALG-NNPW   1465
XP_032815028.1[P.marinus]           ALPVPPPVLEESCPV----AECESHAGDGLC----DRDCNN-HACAWDGGDCSLHFPSPW   1501
XP_014025097.1[S.salar]             ---DPSPSTPASCPY----VECEQHSGDKVC----DPQCNS-HECQWDGGDCSLHWRQPW   1481
XP_018114267.1[X.laevis]            ---E-----TDRCHK----YQCPEKARDGYC----DQECNI-HECLWDGGDCSLTMGDPW   1465
NP_001238962.2[G.gallus]            ---HPSQE-PLGCLD----SQCAEKARDGYC----DEDCNT-HACQWDGGDCSLTMEDPW   1439
NP_035058.2[M.musculus]             ---APTSTPPATCQS----QYCADKARDGIC----DEACNS-HACQWDGGDCSLTMEDPW   1463
Q04721.3[H.sapiens]                 ---APPSTPPATCLS----QYCADKARDGVC----DEACNS-HACQWDGGDCSLTMENPW   1463
XP_009243797.2[P.abelii]            ---APPSTPPATCLS----QYCADKARDGVC----DEACNS-HACQWDGGDCSLTMENPW   1463
                                                           .       :                    .

XP_012554603.1[H.vulgaris]          SICGTTCDFKNIKVSCKPDAKRFRRDMDPK-----WTGPKFILDI-----------SFD   2681
XP_019853114.1[A.queenslandica]     SFIQSVARYHPVSDDRWHYVEVTQNGLEIKTEIDGYPNSTSLIDMFLIFEPAVTYIGGFD   1586
XP_032230096.1[N.vectensis]         SNCTTV---TKSGKACYQVFANGVCDRECNT-------GGCL-------------FDGFD   1160
NP_001245510.1[D.melanogaster]      ANCTA--------NECWNKFKNGKCNEECNN-------AACH-------------YDGHD   1551
XP_019643529.1[B.belcheri]          ANCTSS-------LQCWNYFSNGHCDQQCNN-------EECL-------------YDGRD   1498
XP_032815028.1[P.marinus]           RNCSVR-------LQCWLRFRNGECDEQCNS-------LGCL-------------FDGFD   1534
XP_014025097.1[S.salar]             VNCTAP-------VPCWELFRNGRCDPDCDN-------SGCL-------------FDSFE   1514
XP_018114267.1[X.laevis]            ANCSSS-------LRCWEYF-NGQCDELCNT-------PECL-------------FDNFE   1497
NP_001238962.2[G.gallus]            ANCSSS-------LRCWMLF-NGQCDEFCNT-------PECL-------------FDNFE   1471
NP_035058.2[M.musculus]             ANCTST-------LRCWEYI-NNQCDEQCNT-------AECL-------------FDNFE   1495
Q04721.3[H.sapiens]                 ANCSSP-------LPCWDYI-NNQCDELCNT-------VECL-------------FDNFE   1495
XP_009243797.2[P.abelii]            ANCSSP-------LPCWDYI-NNQCDELCNT-------AECL-------------FDNFE   1495
                                                         .   .                          . :

XP_012554603.1[H.vulgaris]          ATNETNLAKIQNGIKTKEFNMTVIVNNVSISSDLLNVEVKTVCNNGSILKNDICVKCPAG   2741
XP_019853114.1[A.queenslandica]     NYTSLPSAVLQSS----GF-IGYINATIQLGGEAL----------HIIDDSIAGRDIA-   1629
XP_032230096.1[N.vectensis]         CKPSVP-----KC----GA-------------------------------DKYCAARFA-   1179
NP_001245510.1[D.melanogaster]      CERKLK-S--CDS----LF-------------------------------DAYCQKHYG-   1572
XP_019643529.1[B.belcheri]          CEENPHPT--CNP----VY-------------------------------DTYCGNFFR-   1520
XP_032815028.1[P.marinus]           CQLGMK-N--CNP----LY-------------------------------NAYCTAHYA-   1555
XP_014025097.1[S.salar]             CQESAQ-S---YC----KY-------------------------------DKYCAAHYA-   1534
XP_018114267.1[X.laevis]            CQQKE--P---IC----KY-------------------------------DKYCEDHYA-   1516
NP_001238962.2[G.gallus]            CQQNS--R---MC----KY-------------------------------DKYCADHYG-   1490
NP_035058.2[M.musculus]             CQRNS--K---TC----KY-------------------------------DKYCADHFK-   1514
Q04721.3[H.sapiens]                 CQGNS--K---TC----KY-------------------------------DKYCADHFK-   1514
XP_009243797.2[P.abelii]            CQGNS--K---TC----KY-------------------------------DKYCADHFK-   1514
                                                                                  :  .

XP_012554603.1[H.vulgaris]          TFKNVTNELCEKCPFGSYSKEAGSEMC----INCPANT--TTTSEGSTEEKSCKVKCLFG   2795
XP_019853114.1[A.queenslandica]     ------HGEA---AG-----SCGPETCSNGGICVEES----------------------   1652
XP_032230096.1[N.vectensis]         ------NAEC---DA-----ICNNVACQNDGLDCSFKK--PEIVEGTL-----VLV----   1214
NP_001245510.1[D.melanogaster]      ------DGFC---DY-----GCNNAECSWDGLDCENKTQSPVLAEGAM-----SVV----   1609
XP_019643529.1[B.belcheri]          ------DGNC---DS-----GCNNLECGYDGGDCDEDY-PPVTAKGYL-----VMI----   1556
XP_032815028.1[P.marinus]           ------DQHC---DQ-----GCNSDECGWDGGDCAGEV-PERLADGVL-----VLI----   1591
XP_014025097.1[S.salar]             ------NKIC---DK-----GCNTEACGWDGLDCSGDT-PAEVADGTL-----VIV----   1570
XP_018114267.1[X.laevis]            ------NGHC---DK-----SCNTKECGWDGLDCSTDR-PENLAEGTL-----VVV----   1552
NP_001238962.2[G.gallus]            ------DGRC---DQ-----GCNSEECGWDGLDCAGDK-AERLAEGTL-----IIV----   1526
NP_035058.2[M.musculus]             ------DNHC---DQ-----GCNSEECGWDGLDCASDQ-PENLAEGTL-----IIV----   1550
Q04721.3[H.sapiens]                 ------DNHC---DQ-----GCNSEECGWDGLDCAADQ-PENLAEGTL-----VIV----   1550
XP_009243797.2[P.abelii]            ------DNHC---DQ-----GCNSEECGWDGLDCAADQ-PENLAEGTL-----VIV----   1550
                                          .  .             ..   *         .

XP_012554603.1[H.vulgaris]          AYLENNECKDCPIGFYKETAGFEKCQPCALGLTTKKTGAVTKDQCKNDCTLGQELVNNTC   2855
```

```
XP_019853114.1[A.queenslandica]      ------------------QSSFL-------------------------CLC---------   1660
XP_032230096.1[N.vectensis]          LLV-------VPEAFMNGSRVFM-------------------------RELSRTL-----   1237
NP_001245510.1[D.melanogaster]       MLM-------NVEAFREIQAQFL-------------------------RNMSHML-----   1632
XP_019643529.1[B.belcheri]           VAA-------PPEVLLNNSISFL-------------------------RMLSIIL-----   1579
XP_032815028.1[P.marinus]            MLL-------PPARVHNDSANFL-------------------------RQLGSLL-----   1614
XP_014025097.1[S.salar]              VLL-------QPEELMGDMRGFL-------------------------RSLGTLL-----   1593
XP_018114267.1[X.laevis]             VLM-------PPSELLKNSRNFL-------------------------RVLGSIL-----   1575
NP_001238962.2[G.gallus]             VLM-------RPDELLRDVRSFL-------------------------RTLGTLL-----   1549
NP_035058.2[M.musculus]              VLL-------PPEQLLQDSRSFL-------------------------RALGTLL-----   1573
Q04721.3[H.sapiens]                  VLM-------PPEQLLQDARSFL-------------------------RALGTLL-----   1573
XP_009243797.2[P.abelii]             VLM-------PPEQLLQDARSFL-------------------------RALGTLL-----   1573
                                                          *

XP_012554603.1[H.vulgaris]           QPCRMGYYRNNDSQPVCLPCPYNQST-YQLGATSP-VCY-AVCEVGKEIS-SDGLK---C   2908
XP_019853114.1[A.queenslandica]      ---PIGFRGDTCEQDTEIIVPYFNTRSYAIVSNDAFSLYGG---IDIDI------TLHTS   1708
XP_032230096.1[N.vectensis]          ---NTIAFIKKDSEGKELVKVYPLPPSAPVPAERRKRSAEKIWFIDSDLTESHRFRRAAV   1294
NP_001245510.1[D.melanogaster]       ---RTTVRLKKDALGHDIIINWKDNVRVPEIEDTD--FA----------RKNKILYTQQV   1677
XP_019643529.1[B.belcheri]           ---RSSMSFKVDDEGNPMVYPWYGEEVDETISKRAIRSVFR-------ALSGRYRRATES   1629
XP_032815028.1[P.marinus]            ---HATVRFKIDTNGQDMIFPYYGDEARGGGGPRLERAAR---------SLSRFVREMER   1662
XP_014025097.1[S.salar]              ---HTNLRVKMDDQQKPMLYPYYGLEHDDGNEG---QPST---------MTLRGKRELDK   1638
XP_018114267.1[X.laevis]             ---RTNLRIKKNPDGSYMVFPYFRENASSQRSRR--SLLK-----------LRVIREL-E   1618
NP_001238962.2[G.gallus]             ---HTNLRIKLDSQGNPMVFPYYGEKSAARSRR---SVV-----------VVRKHRELEQ   1592
NP_035058.2[M.musculus]              ---HTNLRIKQDSQGALMVYPYFGEKSAAMKKQ---KMTR---------RSLPEEQEQEQ   1618
Q04721.3[H.sapiens]                  ---HTNLRIKRDSQGELMVYPYYGEKSAAMKKQ---RMTR---------RSLPGEQ--EQ   1616
XP_009243797.2[P.abelii]             ---HTNLRIKRDSQGELMVYPYYGEKSAAMKKQ---RMTR---------RSLPGEQ--EQ   1616
                                              .       :   :

XP_012554603.1[H.vulgaris]           MECAIGYYKNDNNAKTCSKCPAGLTTKQLASTSIDQCIEKSSDS-KYYDVQVAITSLTWS   2967
XP_019853114.1[A.queenslandica]      SPNGLIYYL-YDS---------------------------TNVTNDYFTLYLEEGGIG--   1738
XP_032230096.1[N.vectensis]          QKNGVQVQINLDNRG-CE----------------TDCFQSTEQAAKYLGAQQSTGKLN--   1335
NP_001245510.1[D.melanogaster]       HQTGIQIYLEIDNRK-CT-----------------ECFTHAVEAAEFLAATAAKHQLR--   1717
XP_019643529.1[B.belcheri]           EVQGSIVYLEMDNSK-CY-------------RVHDDCFETAKEAADFLAALFLHNGLS--   1673
XP_032815028.1[P.marinus]            PLIGSKVYVEIDNRH-CY-------------QTSDRCFSSAADAAAFLAAQATHG-----   1703
XP_014025097.1[S.salar]              EVIGSKVYLEIDNRK-CS-------------ERSMDCFSSTELAASFIAAEYLKS-----   1679
XP_018114267.1[X.laevis]             QPIGSKVSLEINNQE-CV-------------MDSDQCFKTSESAAALLAAHAVRG-----   1659
NP_001238962.2[G.gallus]             EVIGTRVFLEIDNRQ-CA-------------EDSEQCFHNTEAAAALLAAQAIKG-----   1633
NP_035058.2[M.musculus]              EVIGSKIFLEIDNRQ-CV-------------QDSDQCFKNTDAAAALLASHAIQG-----   1659
Q04721.3[H.sapiens]                  EVAGSKVFLEIDNRQ-CV-------------QDSDHCFKNTDAAAALLASHAIQG-----   1657
XP_009243797.2[P.abelii]             EVAGSKVFLEIDNRQ-CV-------------QDSDHCFKNTDAAAALLASHAIQG-----   1657
                                        .       :.                           :  :

XP_012554603.1[H.vulgaris]           DNLYNQNSVEYRDVAYKIIE----------SVYGVYLNMSYLLFVNITKLSSGSVVANLT   3017
XP_019853114.1[A.queenslandica]      --------IEYSMEGSAHIVSYQ-GNVS-DNEW--HLMTVQLNTSGLYLILDGSLVLYS-   1785
XP_032230096.1[N.vectensis]          --------LPYPVYSVKTEEK---PTVEPETGFQPEPLWIAILCVGVPLFIVGVL--AG-   1381
NP_001245510.1[D.melanogaster]       --------NDFQIHSVRGIKNPGDEDNGEPPANVKYVITG-IILVIIALAFFGMVLSTQ-   1767
XP_019643529.1[B.belcheri]           -------QLNYPVKSLYS---GDDPEPPSTPTFNPLYVVVAAVGILLVMVGLGVLMTTK-   1722
XP_032815028.1[P.marinus]            -------SLPLPLLGVSS---EV-PDVVTAPRVQVYVLLGLVLLVLMVVVLLGVLVGRK-   1751
XP_014025097.1[S.salar]              -------ELPYPVVSVNS---DP-TEPY-KPNFLLYLAVGAA-VIILLILVLGVL-AAK-   1724
XP_018114267.1[X.laevis]             -------TLNYPLVSVEV---EN-APMS-HQTLM-YILPVTL-AIIIVILLMVVV-LAK-   1703
NP_001238962.2[G.gallus]             -------MLPYPFVSVQS---EP-LLPP-KTQLL-YLLAVAA-LIILLILLLGVM-MAK-   1677
NP_035058.2[M.musculus]              -------TLSYPLVSVFS---EL-ESPR-NAQLL-YLLAVAV-VIILFFILLGVI-MAK-   1703
Q04721.3[H.sapiens]                  -------TLSYPLVSVVS---ES-LTPE-RTQLL-YLLAVAV-VIILFIILLGVI-MAK-   1701
XP_009243797.2[P.abelii]             -------TLSYPLVSVVS---ES-LTPE-RTQLL-YLLAVAV-VIILFIILLGVI-MAK-   1701
                                                   .                               :       :

XP_012554603.1[H.vulgaris]           LGFNSSVKDPLFAFDSAVKSQQFYKLTVDPDSLIADRCKHIV--------CPMEQV----   3065
XP_019853114.1[A.queenslandica]      ---NNATLSSIYL-TSP------LFLGGLPNGLIADSVSGSGL-NGCIRDVQISNTSLGL   1834
XP_032230096.1[N.vectensis]          ---GKRVYTKLWL----------------PEGFVRRPVHQ----RRSLRRDPVGQE-HSM   1417
NP_001245510.1[D.melanogaster]       ---RKRAHGVTWF----------------PEGFRAPAAVMSRR-----RRDPHGQE---M   1800
XP_019643529.1[B.belcheri]           ---RRRESGQLWY----------------PEGFKLTKESRKDARRSTRRRDPVGQ-EVGM   1762
XP_032815028.1[P.marinus]            ---RRREQGTLWF----------------PEGFILGPPSDKKK-----RREPVGQDDVGM   1787
XP_014025097.1[S.salar]              ---RKRKHGVLWL----------------PDGFLAKKD---DK-----RREPVGQDDFGM   1757
XP_018114267.1[X.laevis]             ---KKRKHGALWF----------------PEGFFLHRENSNQK-----RREPVGQDAVGM   1739
NP_001238962.2[G.gallus]             ---RKRKHGSLWL----------------PEGFILRRDPSNHK-----RREPVGEDAVGL   1713
NP_035058.2[M.musculus]              ---RKRKHGFLWL----------------PEGFTLRRDSSNHK-----RREPVGQDAVGL   1739
Q04721.3[H.sapiens]                  ---RKRKHGSLWL----------------PEGFTLRRDASNHK-----RREPVGQDAVGL   1737
XP_009243797.2[P.abelii]             ---RKRKHGSLWL----------------PEGFTLRRDASNHK-----RREPVGQDAVGL   1737
                                                :                 *:.:                     :

XP_012554603.1[H.vulgaris]           CSYDTRV-ECKCTVGK--------QKSPDGE-K-CYDTCELKGCPEGQECFSNSTF---N   3111
XP_019853114.1[A.queenslandica]      I-------ENNVLYGL-------------GLLECPQSLCPLVQCLNGGTCVDSDVSVFGF   1874
XP_032230096.1[N.vectensis]          RSMNKSSDLEEEGAVGG-------DLTPPQEARDAKRVKLE------------EVD----   1454
NP_001245510.1[D.melanogaster]       RNLNKQVAMQSQGVGQ------PGAHWSDDESDMPLPKRQR------------SDPVSGV   1842
XP_019643529.1[B.belcheri]           KSFTPQGATAANVDDNDNETVRDSSAWEDDMVPPEAKRMKT------------QQPTPGS   1810
XP_032815028.1[P.marinus]            KTLSQSSVEDSLIDGNQ------NEAWLDGSVPSTKRRKLE------------EQGM---   1826
XP_014025097.1[S.salar]              KNFKTQ--DGGMIDGG--------QRWMEEEAP-PKKVRTE------------DKPL---   1791
XP_018114267.1[X.laevis]             KNLSVPIVDVNLLDSNQ------NDHWSDDRGPQPKKSKSE------------GQAL---   1778
NP_001238962.2[G.gallus]             KNLSVQIPEGNMADSGP------TEHWAGDGGPQPKRVKTE------------DQAL---   1752
NP_035058.2[M.musculus]              KNLSVQVSEANLIGSGT------SEHWVDDEGPQPKKAKAE------------DEAL---   1778
Q04721.3[H.sapiens]                  KNLSVQVSEANLIGTGT------SEHWVDDEGPQPKKVKAE------------DEAL---   1776
XP_009243797.2[P.abelii]             KNLSVQVSEANLIGSGT------SEHWVDDEGPQPKKVKAE------------DEAL---   1776

XP_012554603.1[H.vulgaris]           ECRCKDGK---VLS-----------------SDKKECIYTCEL------KG---------   3136
XP_019853114.1[A.queenslandica]      ECSCPSGYNGTYCEVLLSTCIPNPCLFDGVCTDFNNMTYSCQCTL--GRQGRICDEELNI   1932
XP_032230096.1[N.vectensis]          ------------ESQ-R---------VKVSEKEKDTRQWTRLHREAADVTVRNCT-ALAL   1491
NP_001245510.1[D.melanogaster]       GL----GNNGGYASDHT---------MVSEYEEADQRVWSQAHLDVVDVRA-------IM   1882
XP_019643529.1[B.belcheri]           -----------DATADQ---------TLSDYDDTDHRQWTQQHLHAADIRNPACLAQVAL   1850
XP_032815028.1[P.marinus]            ----------------L----------PEAEEPVDHRPWTQQHLEAADIRTAPAL-VLAL   1859
XP_014025097.1[S.salar]              ----------------L---------PMGVDGGVDRREWTLQHHKAADI---------SL   1817
XP_018114267.1[X.laevis]             ----------------L----------LGGDDQVDSRQWTQQHLVAADIRMTPSL---AL   1809
NP_001238962.2[G.gallus]             ----------------L----------PEADEQIDQRQWTQQHLEAADVCGSTSL---AL   1783
NP_035058.2[M.musculus]              ----------------L----------S-EDDPIDRRPWTQQHLEAADIRHTPSL---AL   1808
Q04721.3[H.sapiens]                  ----------------L----------SEEDDPIDRRPWTQQHLEAADIRRTPSL---AL   1807
XP_009243797.2[P.abelii]             ----------------L----------SEEDDPIDRRPWTQQHLEAADIRRTPSL---AL   1807
```

```
                                                                       .    ::

XP_012554603.1[H.vulgaris]           C-----------------------------------------------------------  3137
XP_019853114.1[A.queenslandica]      TVPAFYGNSYLAYSSLSSITNVQSNLILSLTFKPTSPSGLLLFNGHSNTDFSQYVSISLV  1992
XP_032230096.1[N.vectensis]          TPPQEGES---------------EKPGIDTGVDARGPGGFT-----------PLHLASCR  1525
NP_001245510.1[D.melanogaster]       TPPA-HQ----------------DGG--KHDVDARGPCGLT-----------PLMIAAVR  1912
XP_019643529.1[B.belcheri]           TPPQTEG----------------EADPCSVDVNVRGPDGLT-----------PLMLASFR  1883
XP_032815028.1[P.marinus]            TPPQGEA-----------------DD--GMDVNVRGPNGFT-----------PLMVASFH  1889
XP_014025097.1[S.salar]              TPPQTDL----------------EAD--CLDVNVKGPDGFT-----------PLMLASLR  1848
XP_018114267.1[X.laevis]             TPPQAEQ----------------DID--VIDVNVRGPDGCT-----------PLMLASLR  1840
NP_001238962.2[G.gallus]             TPPQADQ----------------EVD--VLDVNVRGPDGCT-----------PLMLASLR  1814
NP_035058.2[M.musculus]              TPPQAEQ----------------EVD--VLDVNVRGPDGCT-----------PLMLASLR  1839
Q04721.3[H.sapiens]                  TPPQAEQ----------------EVD--VLDVNVRGPDGCT-----------PLMLASLR  1838
XP_009243797.2[P.abelii]             TPPQAEQ----------------EVD--VLDVNVRGPDGCT-----------PLMLASLR  1838


XP_012554603.1[H.vulgaris]           ------------------------------------------------------------  3137
XP_019853114.1[A.queenslandica]      NSSVVY--------------KFDLGSGLATISSPITLGLNQWHTITAYRTGRVG------  2032
XP_032230096.1[N.vectensis]          GTLVDGCSIDD--------DKESDDSGGAMVSDLLALGASYGA--RTDIEKETPLHLAAR  1575
NP_001245510.1[D.melanogaster]       GGGLDTG------E----DIENNEDSTAQVISDLLAQGAELNA--TMDKTGETSLHLAAR  1960
XP_019643529.1[B.belcheri]           GGGLEYV-----ED----HQEDGEDESANVITDLLMQGANINA--QTDRTGETSLHLAAR  1932
XP_032815028.1[P.marinus]            GGGLDSSLCDEEESSDGLFEDGTDDGSANVITDLICQGAALHA--QTDRTGETALHLAAR  1947
XP_014025097.1[S.salar]              NGGASDCGLQAEEE----EESGGDEPCPNAISDLITQGATLMA--QTDRTGETALHLAAR  1902
XP_018114267.1[X.laevis]             MGSPHL------EE----DDDEAEDPSANVITDLIYQGACLQA--QTDRTGETALHLAAR  1888
NP_001238962.2[G.gallus]             VGGSDI------SE----DDEDGEDSSANIITDLIYQGANLQA--QTDRTGEMALHLAAR  1862
NP_035058.2[M.musculus]              GGSSDL------SD----EDEDAEDSSANIITDLVYQGASLQA--QTDRTGEMALHLAAR  1887
Q04721.3[H.sapiens]                  GGSSDL------SD----EDEDAEDSSANIITDLVYQGASLQA--QTDRTGEMALHLAAR  1886
XP_009243797.2[P.abelii]             GGSSDL------SD----EDEDAEDSSANIITDLVYQGASLQA--QTDRTGEMALHLAAR  1886


XP_012554603.1[H.vulgaris]           --------------------------------------------PEGQECFSNSTFN--  3150
XP_019853114.1[A.queenslandica]      FLRVNDEELLTNTSLGTLNGLNIAGDMWLGGTDRFNIISQHAGVGTGLTGCISSVSVNGI  2092
XP_032230096.1[N.vectensis]          HSRADAAKRLLHAGA----------D--PNARDKLGRTPLHLAVGADAQGVFQILLRNR-  1622
NP_001245510.1[D.melanogaster]       FARADAAKRLLDAGA----------D--ANCQDNTGRTPLHAAVAADAMGVFQILLRNR-  2007
XP_019643529.1[B.belcheri]           YARADRAKRLLDAGA----------D--ANARDNTGRTPLHAAIASDAQGVFQILLRNR-  1979
XP_032815028.1[P.marinus]            YARSDAAKRLLDAGA----------D--ANLPDNMGRSPLHAAVSADAQGVFQILIRNR-  1994
XP_014025097.1[S.salar]              YARADAAKRLLDAGA----------D--ANAHDNMGRTPLHAAVAADAQGVFQILIRNR-  1949
XP_018114267.1[X.laevis]             YSRADAAKRLLDAGA----------D--ANSQDSMGRTPLHAAVSADAQGVFQILIRNR-  1935
NP_001238962.2[G.gallus]             YSRADAAKRLLDAGA----------D--ANARDNMGRTPLHAAVAADAQGVFQILIRNR-  1909
NP_035058.2[M.musculus]              YSRADAAKRLLDAGA----------D--ANAQDNMGRCPLHAAVAADAQGVFQILIRNR-  1934
Q04721.3[H.sapiens]                  YSRADAAKRLLDAGA----------D--ANAQDNMGRCPLHAAVAADAQGVFQILIRNR-  1933
XP_009243797.2[P.abelii]             YSRADAAKRLLDAGA----------D--ANAQDNMGRCPLHAAVAADAQGVFQILIRNR-  1933
                                                                                    :.    *

XP_012554603.1[H.vulgaris]           --------ECRCKDGKVLSPDKKRCINQCSLHYCKNGGSCQLKETGPVCRCSKNYSGDQC  3202
XP_019853114.1[A.queenslandica]      SINLVSSAERGLNIGE-------CNMTSCSSFPCFNGGTCTETGSSFICQCPAGYEGGLC  2145
XP_032230096.1[N.vectensis]          ----TTDLEAAMEDGT----------------------------TPLILAARLDLLDIVK  1650
NP_001245510.1[D.melanogaster]       ----ATNLNARMHDGT----------------------------TPLILAARLAIEGMVE  2035
XP_019643529.1[B.belcheri]           ----ATDLDARTNDGT----------------------------TPLILAARLAVEGVVE  2007
XP_032815028.1[P.marinus]            ----ATDVDARMHDGT----------------------------TPLILAARLAVEGMAE  2022
XP_014025097.1[S.salar]              ----ATELDARMNDGT----------------------------TPLILAARLAVEGMVE  1977
XP_018114267.1[X.laevis]             ----VTDLDARMNDGT----------------------------TPLILAARLAVEGMVA  1963
NP_001238962.2[G.gallus]             ----VTDLDARMNDGT----------------------------TPLILAARLAVEGMVA  1937
NP_035058.2[M.musculus]              ----VTDLDARMNDGT----------------------------TPLILAARLAVEGMVA  1962
Q04721.3[H.sapiens]                  ----VTDLDARMNDGT----------------------------TPLILAARLAVEGMVA  1961
XP_009243797.2[P.abelii]             ----VTDLDARMNDGT----------------------------TPLILAARLAVEGMVA  1961
                                             :   . *                             :  :  .     .

XP_012554603.1[H.vulgaris]           QTLQTKSN--AL-------MIVLVSLSTVAVVLIVIYVLKNRKKRIYN---ECRNDLM--  3248
XP_019853114.1[A.queenslandica]      GLQTFSCLSSPCGNGGTCIEGVAEGGYN---C---LCPLGFGGNNCNEMLQVSTPSFNRT  2199
XP_032230096.1[N.vectensis]          DLIKASCKVNNVDAQGKSALHWAAAVNSHEVT---SELCKNGAKKDMQDDKGQTPLFLGA  1707
NP_001245510.1[D.melanogaster]       DLITADADINAADNSGKTALHWAAAVNNTEAV---NILLMHHANRDAQDDKDETPLFLAA  2092
XP_019643529.1[B.belcheri]           ELLNSHADANACDDNGKSALHWAAATNNEEAV---ISLLSHGVNKDVQDNKEETPLFLAA  2064
XP_032815028.1[P.marinus]            ELINCHADVNKVDDLGKSALHWAAAVNNVEAT---LVLLKNGANKDMQDNKEETPLFLAA  2079
XP_014025097.1[S.salar]              ELVHCHADINAVDDHGKSALHWAAAVNNVEAT---LVLLKNGANRDMQDNKEQTPLFLAA  2034
XP_018114267.1[X.laevis]             DLINCQADVNAVDDHGKSALHWAAAVNNVEAT---MVLLKNGANRDMQDNKEETPLFLAA  2020
NP_001238962.2[G.gallus]             ELINCQADVNAVDDHGKSALHWAAAVNNVEAT---LVLLKNGANRDMQDNKEETPLFLAA  1994
NP_035058.2[M.musculus]              ELINCQADVNAVDDHGKSALHWAAAVNNVEAT---LLLLKNGANRDMQDNKEETPLFLAA  2019
Q04721.3[H.sapiens]                  ELINCQADVNAVDDHGKSALHWAAAVNNVEAT---LLLLKNGANRDMQDNKEETPLFLAA  2018
XP_009243797.2[P.abelii]             ELINCQADVNAVDDHGKSALHWAAAVNNVEAT---ILLLKNGANRDMQDNKEETPLFLAA  2018
                                           .                 .  .              :.  :        :

XP_012554603.1[H.vulgaris]           ------------------------------------YDDEKSLELSVKNSNLDKKSV-TL  3271
XP_019853114.1[A.queenslandica]      SYQEYSSPAPISLSTIISLSFHPTSSNGLILYIGDVSTTRDFLSLSLVSGR-------IQ  2252
XP_032230096.1[N.vectensis]          REGSLEAV------RILLLSYAN----------RMIADNMDKTPEEVARQRAHNDIVELL  1751
NP_001245510.1[D.melanogaster]       REGSYEAC------KALLDNFAN----------REITDHMDRLPRDVASERLHHDIVRLL  2136
XP_019643529.1[B.belcheri]           REGSFQAA------KILLDHYAN----------RDITDHMDRLPRDIAQERMHSDIVKLL  2108
XP_032815028.1[P.marinus]            REGSYETA------KLLLDHFAS----------RDITDHVDRLPRDIAQERLHHDIVRLL  2123
XP_014025097.1[S.salar]              REGSFEAA------QVLLDHYSN----------RDITDHLDRLPRDTAQERMHHDIVRLL  2078
XP_018114267.1[X.laevis]             REGSFEAA------KMLLDYFAN----------REITDHMDRLPRDIAKDRMHHDIVRLL  2064
NP_001238962.2[G.gallus]             REGSFEAA------KILLDHFAN----------RDITDHMDRLPRDVAQDRMHHDIVQLL  2038
NP_035058.2[M.musculus]              REGSYEAA------KILLDHFAN----------RDITDHMDRLPRDVARDRMHHDIVRLL  2063
Q04721.3[H.sapiens]                  REGSYEAA------KILLDHFAN----------RDITDHMDRLPRDVARDRMHHDIVRLL  2062
XP_009243797.2[P.abelii]             REGSYEAA------KILLDHFAN----------RDITDHMDRLPRDVARDRMHHDIVRLL  2062
                                                                             .    .    .

XP_012554603.1[H.vulgaris]           TRYDHLRSSEKLG-----------------------------------------------  3284
XP_019853114.1[A.queenslandica]      LRYDLGSGPAIIASPSVIPLNQWTSVTVNRVRKDGVLVVDSVSTNGSSPGFAGLLNPAGN  2312
XP_032230096.1[N.vectensis]          SDWSIGCNSPKAAPAPTSPPDQRSPL--NGTASPPSMDQIS----GN-------------  1792
NP_001245510.1[D.melanogaster]       DEHVP-RSPQMLSMTPQA--MIGSPP--PGQQQPQLITQPTVISAGNG-----------G  2180
XP_019643529.1[B.belcheri]           DEYNLVRSPSQA----------------AANCQNSF--------G--G-----------H  2131
XP_032815028.1[P.marinus]            DEYNVVRNPSGP----------------NGLASPPQ-------ANG--------------  2146
XP_014025097.1[S.salar]              DQYNLVHSPHNG----------------PNHMGGGH---SSLVCGGNG-----------A  2108
XP_018114267.1[X.laevis]             DEYNLVHSTQGQ----------------PGSMLSNS---LSPAVCGSN-----------R  2094
NP_001238962.2[G.gallus]             NEYNVVAHSPTGH---------------PGAMLNSA---LSPVICGPN-----------R  2068
```

```
NP_035058.2[M.musculus]            DEYNVTPSP------------------PGTVLTSA---LSPVLCGPN-----------R   2090
Q04721.3[H.sapiens]                DEYNVTPSP------------------PGTVLTSA---LSPVICGPN-----------R   2089
XP_009243797.2[P.abelii]           DEYNVTPSP------------------PGTVLTSA---LSPVICGPN-----------R   2089
                                          .

XP_012554603.1[H.vulgaris]         -RINGGATFD----------DNSPAPLIRNS------------------DQK--R----   3308
XP_019853114.1[A.queenslandica]    LYIGGGAGGV----GGYQVSPNAGSH--VGL-------TGCV--------DTATLR----   2347
XP_032230096.1[N.vectensis]        KVV---THFP---VSAGTTRPKTNSTSTRGARS-----------KVQNENGAKRKRKRKK   1835
NP_001245510.1[D.melanogaster]     NNGNGNASGKQSNQTAKQK-AAKKAKLIEGSPDNGLDATGSLRRKASSKKTSAASKKAAN   2239
XP_019643529.1[B.belcheri]         HFMNGPTTKQ---QRAKPKRPNKG--MQHHT---------------IARADASKMRRKKK   2171
XP_032815028.1[P.marinus]          AYA-HG-AKA---GKKGVRRPGNKSGVAHDAAK------------------CKKAQ-KKV   2182
XP_014025097.1[S.salar]            GYMGMR-PGP---QGKKSRRGGAKVGGVGG--A-----------TAKELKDMKAKRRKKP   2151
XP_018114267.1[X.laevis]           SFLNLK-HAA---QAKKTRRPSAKNVGPRSLPN-----------LAKEPKDTKNRRRKKS   2139
NP_001238962.2[G.gallus]           SFLNLK-HAS---LSKKSRKPNAKGIMPTNLTK-----------D------AKRRRK-KS   2106
NP_035058.2[M.musculus]            SFLSLK-HTP---MGKKARRPNTKSTMPTSLPN-----------LAKEAKDAKGSRR-KK   2134
Q04721.3[H.sapiens]                SFLSLK-HTP---MGKKSRRPSAKSTMPTSLPN-----------LAKEAKDAKGSRR-KK   2133
XP_009243797.2[P.abelii]           SFLSLK-HTP---MGKKSRRPSAKSTMPTSLPN-----------LAKEAKDAKGSRR-KK   2133
                                                                                         :

XP_012554603.1[H.vulgaris]         ------------------------------------------------------------   3308
XP_019853114.1[A.queenslandica]    ----------------------VNSFGLGAVISSRGVIQCQVDPCSHSPCQNG--GSCV   2382
XP_032230096.1[N.vectensis]        DDDLPHGNRKAS------QGG---ASSTY-----------SPYGKE-LSPC--SSGANFS   1872
NP_001245510.1[D.melanogaster]     LNGLN--PGQLTGGVSGVPGVPPTNSAAQAAAAA-AAAVAAMSHELEGSPVGVGMGGNLP   2296
XP_019643529.1[B.belcheri]         LEGTGTMP-------------RHMDSTAL-----------SPDGSLGGSPHGYNTGE-TP   2206
XP_032815028.1[P.marinus]          NGGVASGGGGDG-----APLLGLDGSASL-----------SPVNSE-SSQ----SDG-TS   2220
XP_014025097.1[S.salar]            TGGEGPAVGGASGNGTKAAGGLLESSVTM-----------SPVDSL-ESPHLYIGDA-AG   2198
XP_018114267.1[X.laevis]           --------------DTDNKGQLSESSVTL-----------SPVDSL-ESPHA------TS   2167
NP_001238962.2[G.gallus]           --------------LSESKGQFSESSVTL-----------SPVDSL-ESPHAFASEP-TS   2139
NP_035058.2[M.musculus]            --------------CLNEKVQLSESSVTL-----------SPVDSL-ESPHTYVSDA-TS   2167
Q04721.3[H.sapiens]                --------------SLSEKVQLSESSVTL-----------SPVDSL-ESPHTYVSDT-TS   2166
XP_009243797.2[P.abelii]           --------------SLSEKVQLSESSVTL-----------SPVDSL-ESPHTYVSDT-TS   2166

XP_012554603.1[H.vulgaris]         ------------------------------------------------------------   3308
XP_019853114.1[A.queenslandica]    SSDLTYSCVCPLGYSGDQCQEISNATEGAAISQCPTSVA--------------CNVNPCL   2428
XP_032230096.1[N.vectensis]        PPAFSVVNGL--------------------SPQGSSATG---------ISPSNSTTLSPQQ   1904
NP_001245510.1[D.melanogaster]     SPYDT--------------SSMYSNAMAAPLANGNPNTGAKQPPSYEDCIKNAQSMQSLQ   2342
XP_019643529.1[B.belcheri]         PPNSMYGVGSQHLALS---HPNLHGVDTVPHTTSTPQIV------NSFSFTNGQPMPRLS   2257
XP_032815028.1[P.marinus]          PSLLT----SPHVAHS-----LYASMEAVGC--GPPRVG------HPMAFRN--------   2255
XP_014025097.1[S.salar]            ASKTT----NSPL-------------LGSP-----------------------------   2211
XP_018114267.1[X.laevis]           SPTIT----SPGTLHT-----SPGQMHATSTLQPPH---------TTM---SMTNLQSIG   2206
NP_001238962.2[G.gallus]           SPVMP----SPGVLHS-----SPSSLLTAP----SVQAA------HSMSFSNLHEMQPLG   2180
NP_035058.2[M.musculus]            SPMIT----SPGILQA-----SPTPLLAAAAPAAPVHTQ------HALSFSNLHDMQPLA   2212
Q04721.3[H.sapiens]                SPMIT----SPGILQA-----SPNPMLATAAPPAPVHAQ------HALSFSNLHEMQPLA   2211
XP_009243797.2[P.abelii]           SPMIT----SPGILQA-----SPNPMLATAAPPAPVHAQ------HALSFSNLHEVQPLA   2211

XP_012554603.1[H.vulgaris]         ------------------------------------------------------------   3308
XP_019853114.1[A.queenslandica]    NGGKCIAVL----------V-NGIIEERCQCSLPFA---AGAKCEQRVSFSSAFFA----   2470
XP_032230096.1[N.vectensis]        HSSLDA----------------------------NSPQFPL---SDSPFDDGYLDLDIF   1932
NP_001245510.1[D.melanogaster]     GNGLDMIKLDNYAYSMGSPFQQELLNG-QGLGMNGNGQRNG---VGPGVLPGGLCGMGGL   2398
XP_019643529.1[B.belcheri]         HH---T----------ADSN-S---HQ-DQSTLRGGETGLGLHLDPPVSVPSDWLASLDS   2299
XP_032815028.1[P.marinus]          ---------------------------------------------GAIAVASGWINPMQN   2270
XP_014025097.1[S.salar]            -SSRPL----------LPPV-SHMLGQ-Q----------------------QSWVG---L   2233
XP_018114267.1[X.laevis]           CAANNM----------LPSV-GQLLSQ-AAQN-------NVLAGQRQLNLSSDWLSRMER   2247
NP_001238962.2[G.gallus]           RGSSTV----------LPSV-SQLLSQ-HRTTPPSS----GLGRLRPANVSTEWMNRMEM   2224
NP_035058.2[M.musculus]            PGASTV----------LPSV-SQLLSH-HHIAPPGSSSAGSLGRLHPVPVPADWMNRVEM   2260
Q04721.3[H.sapiens]                HGASTV----------LPSV-SQLLSH-HHIVSPGSGSAGSLSRLHPVPVPADWMNRMEV   2259
XP_009243797.2[P.abelii]           HGASTV----------LPSV-SQLLSH-HHIVSPGSGSAGSLSRLHPVPVPADWMNRMEV   2259

XP_012554603.1[H.vulgaris]         ------------------------------------------------------------   3308
XP_019853114.1[A.queenslandica]    -------------GDGYLLFPA------VNIPLSSITIHTEIRLSIIPSNGSGL-----L   2506
XP_032230096.1[N.vectensis]        EDLTDQDLHGDFASPACGLRGS------TSDLHDSISMLPETTIGPVPQDSSVPTTSACM   1986
NP_001245510.1[D.melanogaster]     SGA------GNGNSHEQGLSPPYSNQSPPHSVQSSLALSPHAYLGSPSPAKSRP---SLP   2449
XP_019643529.1[B.belcheri]         RNH-----HYTQFSPRMHMTHA----GLNHMPQQ---------------SQSPP---SGL   2332
XP_032815028.1[P.marinus]          VPL----SQFTHQGHSLGLTSSHPQSNHR----------------------------AM   2297
XP_014025097.1[S.salar]            KH--------GYGGHMFSLLPQQMGSGHPSMSQH---------------HGQG------L   2264
XP_018114267.1[X.laevis]           NN--------TQYNEMFGLVHHDTNN-HS-CIQQ---------------NGIMQ---ADI   2279
NP_001238962.2[G.gallus]           NE--------SQYNEMFGMVPPVMH-SHPSVSQQ---------------SGLVQ------   2254
NP_035058.2[M.musculus]            NE--------TQYSEMFGMVLAPAEGAHPGIAA----------------PQSRP------   2290
Q04721.3[H.sapiens]                NE--------TQYNEMFGMVLAPAEGTHPGIA-----------------PQSRP------   2288
XP_009243797.2[P.abelii]           NE--------TQYNEMFGMVLAPAEGTHPGIA-----------------PQSRP------   2288

XP_012554603.1[H.vulgaris]         ------------------------------------------------------------   3308
XP_019853114.1[A.queenslandica]    LYIGHAN------------PSTGNYLLVGMNE--------------------GVLEFRFQ   2534
XP_032230096.1[N.vectensis]        YLPRHS----------------------------------------------VAPFGHT   1999
NP_001245510.1[D.melanogaster]     TSPTHIQAMRHATQQKQFGGSNLNSLLGGANGGGVVGGGGGGGGGVGQGPQNSPVSLGII   2509
XP_019643529.1[B.belcheri]         LSPPHSH-------------------------------------GSITGGSPAQMAAFQAL   2356
XP_032815028.1[P.marinus]          MSPLHP--------------------------------------AMINNQHHQMLAFQQN   2319
XP_014025097.1[S.salar]            LTPMNV---------------------------------------TMSREQLPPIVTFQMM   2286
XP_018114267.1[X.laevis]           KPTLHM--------------------------------------GVARDALPPIMTFQLI   2301
NP_001238962.2[G.gallus]           ADTKHL--------------------------------------GVSRESLPPIMTFQLV   2276
NP_035058.2[M.musculus]            PEGKHM--------------------------------------STQREPLPPIVTFQLI   2312
Q04721.3[H.sapiens]                PEGKHI--------------------------------------TTPREPLPPIVTFQLI   2310
XP_009243797.2[P.abelii]           PEGKHI--------------------------------------TTPREPLPPIVTFQLI   2310

XP_012554603.1[H.vulgaris]         ------------------------------------------------------------   3308
XP_019853114.1[A.queenslandica]    LGSGV--GLVETSQ-----PL----------AIGQQHNITLLRTDYRGRILMSQGNTTVF   2577
XP_032230096.1[N.vectensis]        A------PRLYSAH------------------SSPN-----LCADNESRQLIVAHSINGY   2030
NP_001245510.1[D.melanogaster]     SPTGSDMGIMLAPPQ-------SSKNSAIMQTISPQQQ--QQQQQQQQQQHQQQQQQQQQ   2560
XP_019643529.1[B.belcheri]         SSSNAKQQ----------------------------------QLAHQS---NHHNNHRMY   2379
XP_032815028.1[P.marinus]          LYSPRNKPLMEAPQHHPEQARGGAFFQSKMPMIQPQSN--LQAASQQSQLLASVSQSNMF   2377
```

```
XP_014025097.1[S.salar]              -------------------APGGGQA---------------LLKQPQPGQVQSQGQNQGQ   2312
XP_018114267.1[X.laevis]             -------------------PKSGMNQ---------------QTLVQQT------------   2315
NP_001238962.2[G.gallus]             -------------------PKGGINQ---------------QALPQQA------------   2290
NP_035058.2[M.musculus]              -------------------PKGSIAQ---------------AAGAPQT------------   2326
Q04721.3[H.sapiens]                  -------------------PKGSIAQ---------------PAGAPQP------------   2324
XP_009243797.2[P.abelii]             -------------------PKGSIAQ---------------PAGAPQP------------   2324


XP_012554603.1[H.vulgaris]           ------------------------------------------------------------   3308
XP_019853114.1[A.queenslandica]      TDGMSQGSSRHLNLSPYDFV---LFLGG---APNVPLISQ-----QVYQTGFSGCL----   2622
XP_032230096.1[N.vectensis]          PSN--QSSIQAC--K----------------LS--NRLEEHVMMET-------------T   2055
NP_001245510.1[D.melanogaster]       Q-Q--QQQQQQL--GGLEFGSAGLDLNGFCGSPD--SFHSGQMNPPSIQS----------   2603
XP_019643529.1[B.belcheri]           H-D--QHGNAHG--GS--------PLGGG------GRMHDPGIPLNVIPPGE--------   2412
XP_032815028.1[P.marinus]            PSL--QQQHQGI--AV--------PMQSFVGSEFGESFASNDYKPNEKSHSQTSCVLTQT   2425
XP_014025097.1[S.salar]              PQS--QQGPTHL--HC--------PQGMLYQMPNVSLQHSLSHSLP-HPHG--------L   2351
XP_018114267.1[X.laevis]             --S--H--------AQ--------NVAGMYQMPDRARITGSSVPIA-M------------   2342
NP_001238962.2[G.gallus]             --Q--SNCAQNM--AG--------PLSSMYQISDLAQLPSASFSMA-A------------   2323
NP_035058.2[M.musculus]              --Q--SSCPPAV--AG--------PLPSMYQIPEMPRLPSVAFPPT-M------------   2359
Q04721.3[H.sapiens]                  --Q--STCPPAV--AG--------PLPTMYQIPEMARLPSVAFPTA-M------------   2357
XP_009243797.2[P.abelii]             --Q--STCPPAV--AG--------PLPTMYQIPEMARLPSVAFPTA-M------------   2357


XP_012554603.1[H.vulgaris]           ------------------------------------------------------------   3308
XP_019853114.1[A.queenslandica]      ---------------VSVVQLGYSMNPEPVPFDTSSVGENVTTC-S--------------   2652
XP_032230096.1[N.vectensis]          LH---HN---NQSMCV-PVESGMFREKHHTPPSAHSYGTSSYDS-SPQ--------KLPI   2099
NP_001245510.1[D.melanogaster]       --SMSGSSPSTNMLSPSSQHNQQAFYQYLTPSSQHSGGH------TPQH-----LVQTLD   2650
XP_019643529.1[B.belcheri]           LPPHSHQTVLPPGSCAQQFSSNNPAEQLPTPPSQHSHGNPQSHECTPTHYLSHAETIVPE   2472
XP_032815028.1[P.marinus]            VPPPQEANPSVQSSQLAKREGAAPAEQFPTPPSQHSRGEGSPGH---APPP---GTPGEH   2479
XP_014025097.1[S.salar]              AHGMVQDGQ-SRQQLPPYQAMQSPVDKYPTPPSQHSYATAGSEGTTPGYPA---HPPSEH   2407
XP_018114267.1[X.laevis]             --MHQHDGQLSQTMLTSYHPLQNPMGKYPTPPSQHSYAS---TDKTPNHNG---HLPGEH   2394
NP_001238962.2[G.gallus]             --IPQQDGQVPQTILPAYHQFQSSMGKYPTPPSQHSCYS-SATERTPSHNR---HLQGEH   2377
NP_035058.2[M.musculus]              --MPQQEGQVAQTIVPTYHPFPASVGKYPTPPSQHSYASSNAAERTPSHGG---HLQGEH   2414
Q04721.3[H.sapiens]                  --MPQQDGQVAQTILPAYHPFPASVGKYPTPPSQHSYASSNAAERTPSHSG---HLQGEH   2412
XP_009243797.2[P.abelii]             --MPQQDGQVAQTILPAYHPFPASMGKYPTPPSQHSYASSNAAERTPSHSG---HLQGEH   2412


XP_012554603.1[H.vulgaris]           ------------------------------------------------------------   3308
XP_019853114.1[A.queenslandica]      ------------------------------------------------------------   2652
XP_032230096.1[N.vectensis]          SYLTPSPESPKDWSSS-PSS-HSDWSNC--------------------------------   2125
NP_001245510.1[D.melanogaster]       SYPTPSPESPGHWSSSSPRS-NSDWSEGVQSPAANNLYIS-----GGHQ-----------   2693
XP_019643529.1[B.belcheri]           NYLTPSPDSPDQWSSSSPHSATSDWSDGISSPPAAQSQRQPYI-PETHN-----------   2520
XP_032815028.1[P.marinus]            PYLTPSPETPERWSTCSPRSGASDWSDGVSSPPGLAARAQRRPA----------------   2523
XP_014025097.1[S.salar]              PYLTPSPESPDPWSSSSPHS-NSDWSDVTTSPTPLGNPHALPPLHHTHIPEQLQPQSQQT   2466
XP_018114267.1[X.laevis]             PYLTPSPESPDQWSSSSPNS-ASDWSDVTTSPTPNGS---QRM-PATHMPE---------   2440
NP_001238962.2[G.gallus]             PYLTPSPESPDQWSSSSPHS-ASDWSDVATSPGNN-----QRGPAAAHVPE---------   2422
NP_035058.2[M.musculus]              PYLTPSPESPDQWSSSSPHS-ASDWSDVTTSPTPGGGGGGQRGP-GTHMSE---------   2463
Q04721.3[H.sapiens]                  PYLTPSPESPDQWSSSSPHS-ASDWSDVTTSPTPGGAGGGQRGP-GTHMSE---------   2461
XP_009243797.2[P.abelii]             PYLTPSPESPDQWSSSSPHS-ASDWSDVTTSPTPGGAGGGQRGP-GTHMSE---------   2461


XP_012554603.1[H.vulgaris]           ----------------       3308
XP_019853114.1[A.queenslandica]      ----------------       2652
XP_032230096.1[N.vectensis]          ----------------       2125
NP_001245510.1[D.melanogaster]       ------ANKGSEAIYI       2703
XP_019643529.1[B.belcheri]           ---TRQGGHSALPVFI       2533
XP_032815028.1[P.marinus]            --DPQQQ-LHNLQALM       2536
XP_014025097.1[S.salar]              QQNPQQPQRGNMQVFA       2482
XP_018114267.1[X.laevis]             ------QSHNGMQVYA       2450
NP_001238962.2[G.gallus]             ------QQRNNMQVYA       2432
NP_035058.2[M.musculus]              ------PPHSNMQVYA       2473
Q04721.3[H.sapiens]                  ------PPHNNMQVYA       2471
XP_009243797.2[P.abelii]             ------PPHNNMQVYA       2471
```
