# Supplementary material for: An Evolutionary Perspective on the Origin, Conservation and Binding Partner Acquisition of Tankyrases
Source: Biomolecules. 2022 Nov 15;12(11):1688. doi: 10.3390/biom12111688 (PMC9688111; doi:10.3390/biom12111688)
Supplement: Supplementary file 1 [file biomolecules-12-01688-s001.zip › biomolecules-1913719-supplementary-11.17 -done/Data_7/NuMA.pdf]

NuMA

Homo sapiens:

```
>sp|Q14980|NUMA1_HUMAN Nuclear mitotic apparatus protein 1 OS=Homo sapiens OX=9606 GN=NUMA1 PE=1 SV=2
MTLHATRGAALLSWVNSLHVADPVEAVLQLQDCSIFIKIIDRIHGTEEGQQILKQPVSER
LDFVCSFLQKNRKHPSSPECLVSAQKVLEGSELELAKMTMLLLYHSTMSSKSPRDWEQFE
YKIQAELAVILKFVLDHEDGLNLNEDLENFLQKAPVPSTCSSTFPEELSPPSHQAKREIR
FLELQKVASSSSGNNFLSGSPASPMGDILQTPQFQMRRLKKQLADERSNRDELELELAEN
RKLLTEKDAQIAMMQQRIDRLALLNEKQAASPLEPKELEELRDKNESLTMRLHETLKQCQ
DLKTEKSQMDRKINQLSEENGDLSFKLREFASHLQQLQDALNELTEEHSKATQEWLEKQA
QLEKELSAALQDKKCLEEKNEILQGKLSQLEEHLSQLQDNPPQEKGEVLGDVLQLETLKQ
EAATLAANNTQLQARVEMLETERGQQEAKLLAERGHFEEEKQQLSSLITDLQSSISNLSQ
AKEELEQASQAHGARLTAQVASLTSELTTLNATIQQQDQELAGLKQQAKEKQAQLAQTLQ
QQEQASQGLRHQVEQLSSSLKQKEQQLKEVAEKQEATRQDHAQQLATAAEEREASLRERD
AALKQLEALEKEKAAKLEILQQQLQVANEARDSAQTSVTQAQREKAELSRKVEELQACVE
TARQEQHEAQAQVAELELQLRSEQQKATEKERVAQEKDQLQEQLQALKESLKVTKGSLEE
EKRRAADALEEQQRCISELKAETRSLVEQHKRERKELEEERAGRKGLEARLQQLGEAHQA
ETEVLRRELAEAMAAQHTAESECEQLVKEVAAWRERYEDSQQEEAQYGAMFQEQLMTLKE
ECEKARQELQEAKEKVAGIESHSELQISRQQNELAELHANLARALQQVQEKEVRAQKLAD
DLSTLQEKMAATSKEVARLETLVRKAGEQQETASRELVKEPARAGDRQPEWLEEQQGRQF
CSTQAALQAMEREAEQMGNELERLRAALMESQGQQQEERGQQEREVARLTQERGRAQADL
ALEKAARAELEMRLQNALNEQRVEFATLQEALAHALTEKEGKDQELAKLRGLEAAQIKEL
EELRQTVKQLKEQLAKKEKEHASGSGAQSEAAGRTEPTGPKLEALRAEVSKLEQQCQKQQ
EQADSLERSLEAERASRAERDSALETLQGQLEEKAQELGHSQSALASAQRELAAFRTKVQ
DHSKAEDEWKAQVARGRQEAERKNSLISSLEEEVSILNRQVLEKEGESKELKRLVMAESE
KSQKLEERLRLLQAETASNSARAAERSSALREEVQSLREEAEKQRVASENLRQELTSQAE
RAEELGQELKAWQEKFFQKEQALSTLQLEHTSTQALVSELLPAKHLCQQLQAEQAAAEKR
HREELEQSKQAAGGLRAELLRAQRELGELIPLRQKVAEQERTAQQLRAEKASYAEQLSML
KKAHGLLAEENRGLGERANLGRQFLEVELDQAREKYVQELAAVRADAETRLAEVQREAQS
TARELEVMTAKYEGAKVKVLEERQRFQEERQKLTAQVEQLEVFQREQTKQVEELSKKLAD
SDQASKVQQQKLKAVQAQGGESQQEAQRLQAQLNELQAQLSQKEQAAEHYKLQMEKAKTH
YDAKKQQNQELQEQLRSLEQLQKENKELRAEAERLGHELQQAGLKTKEAEQTCRHLTAQV
RSLEAQVAHADQQLRDLGKFQVATDALKSREPQAKPQLDLSIDSLDLSCEEGTPLSITSK
LPRTQPDGTSVPGEPASPISQRLPPKVESLESLYFTPIPARSQAPLESSLDSLGDVFLDS
GRKTRSARRRTTQIINITMTKKLDVEEPDSANSSFYSTRSAPASQASLRATSSTQSLARL
GSPDYGNSALLSLPGYRPTTRSSARRSQAGVSSGAPPGRNSFYMGTCQDEPEQLDDWNRI
AELQQRNRVCPPHLKTCYPLESRPSLSLGTITDEEMKTGDPQETLRRASMQPIQIAEGTG
ITTRQQRKRVSLEPHQGPGTPESKKATSCFPRPMTPRDRHEGRKQSTTEAQKKAAPASTK
QADRRQSMAFSILNTPKKLGNSLLRRGASKKALSKASPNTRSGTRRSPRIATTTASAATA
AAIGATPRAKGKAKH
```

Pongo abelii:

```
>XP_002822235.1 nuclear mitotic apparatus protein 1 isoform X1 [Pongo abelii]
MTLHTTRGSALLSWVNSLHVADPVEAVLQLQDCSIFIKIIDRIHGTEEGQQILKQPVSERLDFVCSFLQK
NRKHPSSPECLVSAQKVLEGSELELAKMTMLLLYHSTMSSKSPRDWEQFEYKIQAELAVILKFVLDHEDG
LNLNEDLENFLQKAPVPSTCSSTFPEELSPPSHQAKREIRFLELQKVASSSSGNNFLSGSPASPMGDILQ
TPQFQMRRLKKQLADERSNRDELELELAENRKLLTEKDAQIAMMQQRIDRLALLNEKQAASPLEPKELEE
LRDKNESLTMRLHETLKQCQDLKTEKSQMDRKINQLSEENGDLSFKLREFASHLQQLQDALNELTEEHSK
ATQEWLEKQAQLEKELSAALQDKKCLEEKNEILQGKLSQLEEHLSQLRENPPQEKGEVLGDVLQLETLKQ
EAATLAANNTQLQARVETLETERGQQEARLLAERDHFEEEKQQLSSLITDLQSSISNLSQAKEELEQASQ
AHGVRLTAQVASLTCELTTLNATIQQQDQELAGLKQQAKEKQAQLAQTLQQQEQASQGLRHQVEQLSSSL
KQKEQQLKEVVEKQEATRRDHAQQLATAAEEREASLRERDAALKQLEALEKEKAAKLETLQQQLQAANEA
RDSAQTSVTQAQREKAELSQKVEELRACVETARQEQHEAQAQVAELEFQLRSEQQKATEKERVAQEKDQL
QEQLRALKESLKVTKGSLEEEKRRAADALEEQQRCISELKAETRSLVEQHKREQKELEEERAGRKGLEAR
LQQLGEAHQAETEVLRQELAEAIAAQRTAESECEQLVKEVAAWRERYEDSQQEEAQYGAMFQEQLMTLKE
ECEKARQELQEAKEKVAGIESHSELQISRQQNELAELHANLARALQQVQEKEVRAQKLADDLSTLQEKMA
ATSKEVARLETLVRKAGEQQETASRELVKEPARAGDGQPEWLEEQQGRQFCSTQAALQAMEREAEQMGNE
LERLRAALMESQGQQQEERGQQEREVARLTQERGRAQADLALEKAARAELEMRLQNALNEQRVEFATLQE
ALAHALTEKEGKDQELAKLRGLEAAQIKELEELRQTMKQLKEQLAKKEKEHASGSGAQSEAAGRTEPTGP
KLEALRAEVSKLEQQCQQQQKQADSLERSLEAERASRAERDSSLETLRGQLEEKAQELGHSQSVLASAQQ
ELAALRTKVQDHSKAEDEWKAQVARGRQEAERKNSLISSLEEEVSILNRQVLEKEGESKELKQLVMAESE
KSQKLEERLRLLQAETASNSARAAERSCALREEVQSLREEAEKQRVASENLRQELTSQAERAEELGQELK
AWQEKFFQKEQALSTLQLEHTSTQALVSELLPAKHLCQQLQAEQAAAEKRHREELEQSKQAAGGLRAELL
RAQRELGELIPLRQKVAEQERTAQQLRAEKASYAEQLSMLKKAHGLLAEENRGLGERANLGRQFLEVELD
QAREKYVQELAAVRADAETRLAEVQREAQSTARELEVMTAKYEGAKVKVLEERQRFQEERQKLTAQVEQL
EVFQREQTKQVEELSKKLVESDQASKVQQQKLKAVQAQGGESQQEAQRLQAQLNELQAQLSQKEQAAEHY
KLQMEKAKTHYDAKKQQNQELQEQLRSLEQLQKENKELRAEAERLGHELQQAGLKTKEAEQTCRHLTAQV
RSLEAQVAHADQQLRDLGKFQVATDALKSREPQAKPQLDLSIDSLDLSCEEGTPLSITSKLPRTQPDGTS
VPGEPASPISQRLPPKVESLESLYFTPIPARSQAPLESSLDSLGDVFLDSGRKTRSARRRTTQIINITMT
KKLDVEEPDSANSSFYSTRSAPASQASLRATSSTQSLARLGSPDYGNSALLSLPGYRPTTRSSARRSQAG
VSSGAPPGRNSFYMGTCQDEPEQLDDWNRIAELQQRNRVCPPHLKTCYPLESRPSLSLGTITDEEMKTGD
PQETLRRASMQPIQIAEGTGITTRQQRKRVSLEPHQGPGTPESKKATSCFPRPMTPRDRHEGRKQSTTEA
QKKAAPASTKQADRRQSMAFSILNTPKKLGNSLLRRGASKKALSKASPNTRSGTRRSPRIATTTASAATA
AIAATPRAKGKAKH
```

Mus musculus:

```
>NP_001390473.1 nuclear mitotic apparatus protein 1 isoform a [Mus musculus]
MTLHATRAATLLSWVNSLHVADPVETVLQLQDCSIFIKIINTIHDTKEGQQILQQPLPERLDFVCSFLQK
NRKHPSSTQCLVSVQKVIEGSEMELAKMIMLFLYQSTMSSRNLRDWEQFEYGVQAELAVILKFMLDHEES
LNLTEDLESFLEKVPYTHASTLSEELSPPSHQTKRKIRFLEIQRIASSSSENNFLSGSPSSPMGDILQTP
QFQMRRLKKQLADERSNRDDLELELSESLKLLTEKDAQIAMMQQRIDHLALLNEKQAASSQEPSELEELR
GKNESLTVRLHETLKQCQNLKTEKSQMDRKISQLSEENGDLSFKVREFANHLQQLQGAFNDLIEEHSKAS
QEWAEKQARLENELSTALQDKKCLEEKNEILQGKLSQLEDQATRLQESPAPEKGEVLGDALQLDTLKQEA
AKLATDNTQLQTRVETLECERGKQEAQLLAERSRFEDEKQQLASLIADLQSSVSNLSQAKEELEQASQAQ
GAQLTAQLTSMTGLNATLQQRDQELASLKEQAKKEQAQMLQTMQEQEQAAQGLRQQVEQLSSSLKLKEQQ
LEEAAKEQEATRQDHAQQLAIVAEAREASLRERDTARQQLETVEKEKDAKLESLQQQLQAANDARDNAQT
SVTQAQQEKAELSQKIGELHACIEASHQEQRQVQARVTELEAQLKAEQQKTTEREKVVQEKAQLQEQLRA
LEESLKITKGSLEEEKRRAADALKEQQCRATEMEAESRSLMEQREREQKELEQEKAGRKGLEARIQQLEE
AHQAETEALRHELAEATASQHRAESECERLIREVESRQKRFEARQQEEARYGAMFQEQLMALKGEKTGQE
VQEEAVEIHSEGQPGQQQSQLAQLHASLAKAIQQVQEKEVRAQKLVDDLSALQEKMAATNKEVACLKTLV
LKAGEQQETASLELLKEPPRAANRASDQLGEQQGRPFSSTHAAVKAMEREAEQMGGELERLRAALIKSQG
QQQEERGQQEREVARLTQERGQAQADLAQEKAAKAELEMRLQNTLNEQRVEFAALQEALAHALTEKEGTD
QELAKLRGQEAAQRTELKELQQTLEQLKIQLVKKEKEHPAGGASGEDASGPGTQSETAGKTDAPGPELQA
LRAEISKLEQQCQQQQQQVEGLTHSLKSERACRAEQDKALETLQGQLEEKARELGHNQAASASAQRELQA
LRAKAQDHSKAEEEWKAQVARGQQEAERKSSLISSLEEEVSILNRQVLEKEGESKELKRLVVAESEKSQK
LEERLRLLQVETASNSARAAERSSALREEVQSLREEVEKQRVVSENSRQELASQAERAEELGQELKAWQE
KFFQKEQALSALQLEHTSTQALVSELLPAKHLCQQLQAEQAAAEKRFREELEQSKQAAGGLQAELMRAQR
ELGELGSLRQKIVEQERAAQQLRAEKASYAEQLSMLKKAHGLLAEENRGLGERANLGRQFLEVELDQARE
KYVQELAAVRTDAETHLAEMRQEAQSTSRELEVMTAKYEGAKVKVLEERQRFQEERQKLTAQVEQLEVFQ
REQTKQVEELSKKLTEHDQASKVQQQKLKAFQAQRGESQQEVQRLQTQLNELQAQLSQKEQAAEHYKLQM
EKAKTHYDAKKQQNQKLQEQLQDLEELQKENKELRSEAERLGRELQQAGLKTKEAEQTCRHLTAQVRSLE
```

AQVAHADQQLRDLGKFQVATDALKSREPQVKPQLDLSIDSLDLSLEEGTPCSVASKLPRTQPDGTSVPGE
PASPISQRLPPKVESLESLYFTPTPARGQAPLETSLDSLGDAFPDSGRKTRSARRRTTQIINITMTKKLE
LEEPDSANSSFYSTQSAPASQANLRATSSTQSLARLGSPDDGNSALLSLPGYRPTTRSSARRSQARMSSG
APQGRNSFYMGTCQDEPEQLDDWNRIAELQQRNRVCPPHLKTCYPLESRPTLSLATITDEEMKTGDPRET
LRRASMQPAQIAEGVGITTRQQRKRVSSETHQGPGTPESKKATSCFPRPMTPRDRHEGRKQSSTADTQKK
AAPVLKQADRRQSMAFSILNTPKKLGNSLLRRGASKKTPAKVSPNPRSGTRRSPRIATTTTGTATVATTP
RAKGKVKH

Gallus gallus:

>CBN80053.1 nuclear mitotic apparatus protein [Gallus gallus]
MPLHTTRATALLAWVNSTKACAEPLGDLSQLQDCRVFIQIINKIHRTEEGESVLEQSLAERAAFICGFLQ
KLCKHKSATENLVSAQKLLEGEELELAKVAVLLLYHSSMSSKSPRDWNEFDYQIQVELATILKFVLDHEE
SLSENLEVFLQRRAPLSSPGTSSSSSEERSPGLSHPQVRFLELQKIASSSSSMNNMLPGTPASPVGDVLQ
TPQFQLRRLKKQLAIERENRDELEMELAENRKLITEKEAQITMMQHRIDRLALLNEKQAADQLEPREMEE
LRERNESLLVRLHEALKQCQDLKTEKGQMDRKINQLSEENGDLSFKLREIASHLVQLQDALNELSEEHNS
ALMQSQAKQAQLEGELRAVLQEKKCLEEKVEILQGKISLLEDQLVKLGDCSTQEKGEVMGDVLKLEELKQ
EVSSLTTKGIELQASVLRLEEEKSQRDAALQAERNHFEMEKLQLGTHISNLQSSVSELHLAKEKLEQELR
AQEAHLTAQADALTAEVGKLSGFLQQQEKEAAELRMQAEQERAQAEELRHRDMASQEALSELSRRVEQLG
ASLKHSEEELVRVTQEAEGKARQLGAEHEKAVQERDAALQQLQHLQQAKEKQLAALSSQLQSLEAASKAS
SMEMQQEKAELNQKVQELQARVLELSAQCQQSSAQAGSAETLRAQLRELEGKLKDSQQKLADKEKVAKEN
TRLQERLLFLEESVRNTEGILEDEKRRAAESLEGNLARIAELEAEKQQLVQRGEQALQQHSEELARRQAL
ETRLQQVAEERREETAALQRQLAEVAKGEEGERGRLEKRLQELSREHGQACQRLQAEQERVAELEARVAR
MASEQQEQLAALQDQLARAKEKEGKEQEKLASAERVGKLEAEVRKASEALEAVSKELSEERLKFKELEAV
ATQEASEVARLSAALEEAAREHGQELARREEEAKRLQKELEDAKADCAAEKARKVELEVQLQNSINEQRV
ERSAHQQELARSLELIEEKEGELDELRLKNVSRGEELRDLQKTVSKLKGELASVEAVKERASKMDSELQG
FLEAARSRDAEMDSIKAVYAKEASLKNLEEKIRHREQESGSSQDLYQEKLKEAQMLSVEVERLEQKCREQ
QDTIAGLEKAVAEGSQQQQAELEALQREAVRHRETAAELQRLLDASRSAQALQDGTVESLRKELQDKSKE
LAQSKMAVAAAEKEVASLRAAAQEKGHLEEGWKEQLSQCIQELERKNSLLGSLEHEVSILHRQLTEKEGE
SKELKRLIMAESEKSKKLEERLRVLQTEMATAASRAAERCSLMKVEVQRCQEEMEKQRMTIEALKRDRHC
QSEREEELRQEVKVCQDKFFQKEQLLSSLQQELGSAQALAGEVVPLKHLCQQLQAERASLESKHREDLEQ
RAKATTALQAELARARVEVAELPALRDRAAEQERALQRLQKETASSSERLAALQAANSRLAEENRALSES
VSRGQQRLDAELGQAREKHTRELECVRLEAEKAMASSRQEAEEAARKLEAMSNKYENTKVKVLEERQKFQ
EERQKLMAQVEQLEVFQKEQAKQVEELNKKLVQHEKATRSQQQRVKVLEGELQSEATRQQERVAELEEQL
AQKEQAAEHYKAQMEKAKTHYDAKKQQNQDLAEKLKAMEQLQKENAELRTESERLAKELQQSVLQAKEAE
LSCRELSGQVRSLETQLEFADQQLRELGKFPAPKATLKEPESFRQNPSDLSTDSLDLSLDEGQPLNSTRK
ATRSHSDGSAAPGGTEPRASQRLPRKVESLESLYFTPIASRTQPKLESSAGSLADVSLESGCRTRSGRRR
TTQIINITMTKKETTTEEPGGADASFSSILSEQPQKAAPARARLRSARSLASFPSQDSLSKLDTSSPQEP
SGHAALLSLPGYRPATRSSLRRSQAGSSSSLGRSSIYLGTCQDEPEQLDDWNRIAELQQRNRACPPHLKT
CYPLESRPSDVLGTITDEEMKTGDPTETLRRASMQPLQIAEGAATRRGTLGGGWAHGGITTRQQRKRLSD
ESHQGPDTPESKKPVSCFPRPQTPRERRSSHLSRRSEQQAPSKQPERRQSMAFSILNTPKKLGNSLLRRA
ANRKTTPKNSPRGTARRSPRIASTKSPKGKAGRRALKDTKF

Xenopus laevis:

>XP_018103294.2 nuclear mitotic apparatus protein 1 isoform X3 [Xenopus laevis]
MALHSGKMEALICWVNSLKVDEPVERFSQLQDLNILLKVVGVLSGNLEETTSVLQQNPEQRLNFLGSFLQ
KYCRPGSSAENLVQWQKILQGENLELEFSKVIVLLFYYSTMSSKNPKKSEDFDHKTQTELASILRFVLDN
EESLCLDDKLINFLQRKARFPSSGDDASGSSDAMSPYMSHRRKTEVRFLELHRVASSSTIKSLPADAPSS
PMIEVLHTPQFQMRRLKKQLSEVRECRDELEVELAQNHKHLAEKEAQISLLQQQIEHLRILRENQAELQE
PKELEELREKNESLMMRLRDTLKQCQDMKTDKKQLERKNDQWAEENGELTYKVRDLSNRLAQLQEALYET
TEEHERSLSNWQQKQNQLESELSGAVGEKKYLEEHDLILQGKISMLEDQLKKMGEREVQETGDCMGDILK
LDDLKQEVAVHSTQCLSLKEQIRQLEEEKSIAEVEMEAQRSRFESEKAQLQEIVTNLQTSLSEITFQKER
LDNEARAQQEHLMCQMTTLKLEISKIKSSLMHKDKELKDIHHKVEEERSEKNQLLENLKMLEESSKKDRQ
ELIHQVEHLDSSLKVSEGNLVGITQQLESKTKEADYLREEQQKIICERDSSLSTLNEYKCKKDEEFIALN
GTLRTMEQDHQTSLSVIEELKREKAELASKVQELDATILDLIAKCQNLDSENDSQSKSHAITVESLKAQL
SEQESQVKIYQKKLSSNELVSKENSQLKEQLLSMEESVRNLSEHLDKEKMKFAASLDGDSKRISHLEEEM
KKLTESRDAALRNLDEERTAGKKIESQLKNLEEEYHRSNESQHAQLAESCAAIKQREEERDELSKVVDIW
KAKYGESQQKIAQNSCHMQEQIEELKKTHSDVYQQLEGERSKVLMIEAKASETKSSQLEKINQLEGELSA
ANACIKEREAEEKKLVSALHSAEEKLKIAYQGESERLSHLETALSNAKQDLDCLAKELSDEKYKKAEFEA
MVKVLKEQNSERIASLESELKNSLAVVKERKCESEKLSGEVEHLKRQLDDSSQKHKEALAQKNIEIKQLI
DAKEKATSDLAIKSEMGAQLQKAVDTHKSEFSALQNELSRSLDFIALKEGEVERLNKEAALRQEEIQQQQ
QTMTKLTEELTALAALKEKVALQEKEIKQQVQATKGAEEEMANLKSIISEKSKRIECLEQDIKNQKTDLS
SIQEQYQSKLSEAQGHQALIADLEKKCKEQKELICEAQNKAAEAKTLASEKASMSEKQLKGIQTLENEIQ
KERQKTCDLQKQLEVSQALQAEKETELEALKKELFHKVQELEQSQKSFTDSNRELSSMLSEVQERQQALT
EAKEQAEQYQKVIDMKNKEVNSLQAEINILSSKVTSKEEVSADFEQRLLQETSKSAKLEEKLKKLHTEVE
VSSKELVEKNCAVDILRTEAQTFKGEADEQRMAVDCLQQKLSSQAETNNNLQQEIQSWQKNCAEKEQKIC
SLQQNLNSNQSLLEEFASLKRSYQEILAERGLMQQKHQEELLSHKNLTERFQAELEKTKEDMAEIVLLKE
KLNNQELQLHKLQSENSDQSSQISNLQQVNSRLLGENQSLSQISDQDAKKLSIEMSTLKEQHEEEMKTLR
LQYEKTQREGNEQVQDLSLRLETATSKYDHVKSKVLKDQKTFQEEKQMLLLRVEELAAAKKEQSEQVQEL
NKQLRQQEKTLQSQQQKLKQREGETHEEAEKTHKRVIELESQLEQQTQAVEHYKAQMEKAKVHYDAKKKQ
NQELSDELQSHIREQEHLRKENADLKAESEQLSKELQHSLLQSKEAEQNCKNLSNRVRSLEAQVEYSDRQ
LQDLGKFQLATDSTKRRDTFCVPRETRSHADVSIDSLDLSFEEDQLLNSTSKNGRCNQEPATSSVHASSL
DSPASSQLPKKVESLESLYFTPIPTRAQSKLESSIGSIGDLSLDSSKKTRSARRRTTQIINITMTKKTKE
EREPESANTSFYSLRSAPSYQNLHQQNPRKAARPQAAISTPALASLPSQESLAKTEHFSSDDSLNNSVLM
NLPGYRAPARRSSRLSQTGGRSSFYMSTCQDEPDPQEDWTRIAELQARNKTCPPHLKTSYPLESKPSLFS
STLTDEEVKLGDPKETLRRATMLPSQIQDSTTSTRRLTLAGSGAEHLKGHGISTRQQMKRVSEESHYGPD
TPEGKKTATCFPRPMTPKDKHDARRLSTMESKASSSHQQVQPTRRQTSAFSIFNTPKKLGSSLLKRGLNK
KTTPKNSPRGRGTNSSASSTSSKSPHLSLRKSPSRRSPRVSTAKSPKASNKVGQEEQIQFFERKQQRNK

Salmo salar:

>XP_045580741.1 nuclear mitotic apparatus protein 1 isoform X1 [Salmo salar]
MVLHLDKEHALLEWVNHLNVDLPVRSINDLQDGVLLMKLVYKLRKEEPANSYLDQHVQERLKVVSDFLQG
DCRCSTERGALISWDNISNGLNLEVELSKVLVLLYYHSVINNHVDLNQLEYKFEVELASMLRFVLDNENS
LYLSENLEKYLRKKPLFSFNSDISSTSSSSLFNDDESPVFQRRKKMGSVQFLDLQTVASSSVSSPLQDVM
NTPQFQLKKLQRQLRQERDMRDELEKDLTTSATTLTQRESQICQLQHRIEKLLREQAEQEQEPRDELQEL
HSKNEGLRTRLHEVLKECQALKTNSSQMERKVDSLTEENGTLSAQMREVVARLASAEAEVDRLIEAQDSA
QGEWSSRHCHLQDELNRATAQKECLSEQMLILQSKISSLEDELSKAKMQEKGEVMGPILEWEQLKQELAD
ATLRHAECECTIARLKGEKEQAAALHAQERASLQAESQRLQVLVTELQEALSALRADREALELASKEERE
SLTAQLHTLTAEVASLTQTVQQREQEVKALGEEVQQECMQRGELNLAMERQDREAREEIQELTSHVDTMG
ASLRRAEEEVQVREKQLTKQQQESALQREVLQEEMAASEKALKELKKQEEAVREEATRLHQEITTHVTDL
CSLRQEHTALQEQLARQQEEISLEKEAQAAAHREKEAVEAELSRLQEEVRSLGEQMAQLEEAQREKERLL
LQSTENMETLQTERAAASSLAEAKDLELSILREEVRAREEQLAMQQEEYRLRQEELQEELAAQENEINNM
RERLAGLLDQISLLKEVCQEGKNMEALREEHAAQLEQLRLVKEQVEQVQERNEETSAALREKESSLREKE
SSLREKEESLRGLEEELQSTTSLASQRQQEELTSLKEEVISLQEEVERRRAAEALAVEEARELEESVSAL
QQQLDSAIQDNDMKRQKCERLEQDLEQRGTLVEELRQQEHSARLEATRLRQEISTHLSRLEVVQREKEEL
RGEVSLHQQRAAVLQQSLEEQEVAMRVLKEQKESIREETTVKIEALQAQLEVVSSLAAAKDLQLSTLREE
ATALQEQLAKREQEISLQKEVLQEAHREKESLEALREELARQQEELREELILQQQRAQSLEQSLEEQQEA
LKELTLKEERAREEATQLRQQISTHLSRLEEVQRGKEELREQEEKAREETTVKMEALQAQLEVVSSLAAA
KDLQLSTLREEASLLCQENTKRAADLKDVQLEKIRLESLLSEEHRALKEDLAKQQEELRGEVSLHQQRAA
ELQLSLEEKQEALRELKEQLVQQQEDSSLQKVLQEAQAAALQQEAVDALRGEVSLHQQRAAELQHQEAAL
REQEEKSTEEATLKMEALKAAKDLQLSALTEKATALQEQLAKREQEISLQKEVLQEAHREKESVEALREE
LARQQEELREELILQQQRAQSLEQSLEEQQEALKELTLKEERAREEATQLRQQISTHLSRLEEVQRGKEE
LREQEEKAREETTVKMEALQAQLEVVSSLAAAKDLQLSTLREEASLLCQENTKRAADLKDVQLEKIRLES
LLSEEHRALKEDLAKQQEELRGEVSLHQQRAAELQHQEAALREQEEKSTEEATLKMEALKAAKDLQLSAL
TEKAIALQEQLAKREQEISLQKEVLQEAHREKESLEALREELARQQEELREELILQQQRAQSLEQSLEEQ
QEALKELTLKEERAREEATLKMVALQAAKDLELSTLRQEATQLRQEISTHVSHLEEVKSEGPLREEHTAL
QVQLAKQQEENSLQKGLLQEVQATVLQEREAKEALRGEVSLHQQSLEEQQVAVRETLARQKEEGQAAGQV
QKELMEQFSVLQQEKEALLTRALQAEQNQSELEGSMAELRAQAESRESGQRQQLDALLLEKERLTEGHQV
LEMKCSAAQRLEAVLLEELALLREQMEGTEWEKQIRDLREQLAANTEVVEHYKTQVEKAKSHYSGKKQQL

VESQEQVTELQRSLEVREHEVNAVTTEMKLLQKELEKARNKEKSLSSKVNTLEAQLAFTDRHLREQSQVR
PERGPGGIEKMRGGRESVYLKVPQSQTHQETSGDSLDLSLDDSLNTTTRPLGPDESSTPLVRSSERVAAK
RRALGGESLETLYFTPMNNRQINRTSTERRLESSITCLGELALDSARKRPPTSSARRRRTTQVINITMSK
TTPGRGGAGGDSDNEMFYSLSSVRSHPNITGSTHTARPISMEVFHTPGKPAVAVSDQLLSLPGYRRSTVH
VAAPQSTGQFCVGAENEPDHAADDWLRIAELQARNQSCLPHLKSSYPLESRPSLGPSFEFTDDDLRMGDP
TETIRRASVMPGQIQESLSSHRLSLHPGQADSTTASRPAYGSHRLSLMPPKPKASSTLNNQNTHNLRGSN
LSLKRSAKDQEPDTPEVRMEAKRMATSCFPRPLTPKGGRFSSSNNRQPPSPAERRQSMVFSIDNTPRKAA
SKSGFLQRGMNKIRSSTRKSPANKISRVPRSGDVKSPQPGGKAQRKSPRTNSSKSPKNPTSARKEPEVLV
GKPIHLSR

Petromyzon marinus:

>XP_032819484.1 nuclear mitotic apparatus protein 1-like [Petromyzon marinus]
MVNGRKSAALISWVNCMCAEKTESLWDLKDLSTYLHIIQTIKASVDVKPLLKSSEYEKGNYIREFLHQFF
QKNACLQTVDWDALTVGEHVELELAKLTVAFLCCAVVSTEIQSRMKFLDPDTQIQLHEILKFVMDNEESP
QLQVNLELLLNVGCVVRSPGVFSIATEETSGRFSSPLSSLPLGHTAGVRFRDLQRIASSSMNNLLSSPGS
PSSPMLSFFDQPAVEVRRLKQQLRQEWEQQEKLEGELSSKSQLLKQSEQEVAELRGRVERLVQLTQTRPP
SPLPSELAELHEKTQRLSVTVKQCQELKKENELMEKRLTQLAEENGELSSKVRESCTHLAECRSLFEESS
RSAEQRHAEAEVRIHRLEEALGEALNSKASLEETVRILQGKLGALAEKEASLKEQRNLPPGEVMSEVLQM
EEWHMCQKARERADVGLPPLAKAHTNTTHGHDTPTSSREEQSADFNNVSDNDSSTLIVKDRKYLMPNEST
MELEGSMVDQYNVTFREELDGHESKQTGVSQDKCVEATRARSSALKQNSLEGSSTSSRREMGIARGVQEN
FEFKNNGHRDTLEQGRTLLQQERVALSKERDILERERAALEKEQMAVIAMGAGNRSILMGHMLTTEASIA
QQRAILDQEKNKLNLEKAALKAMEDEININLRQQKSRLDQEKRKLEQDMIILKQKWNCVEQEQDNLVKER
AIEDQSKASFTKEIIYLKASLEEETSGMKARVEQERVLLENEREKIIQSITANQNDTASLQRERIAMQQE
VDVREQARSALICVVAALEKIEVMPGNEKGSVICALEEGMTTLKAILGLDKGIACEGTHIDMADNGIVAQ
AVLQQQRTEPAQDNYFISVGKHGMGHRERDTLCQEKDKTNIEETTWDGERSLSQRESLVSLDQSNDVLDR
DQADLETVRDVLLKADTKMAVLAPMQESAAVKDFMEEDCSPLDQEDSDATVRNQKAFTPAETVRLVQANL
DHEQIVFRDSLQHEVAVLEQKRTTLTASLEQERNRLCELEQASMSVRMEADSLKAALQLEEIVFRATLNE
ERTVLEEMKFKVQDNTLLQDQEKVKLQRDRDLLEDEKVSIERERVALDKMKATLEEMKAIWERERVVLVA
PLEQERVTLKRELAALHQERAKWTTESERLVQEQALLEQQRVALQQERESLDQHRGNLKVKSEQERAAQS
ETMYKVEQENACLRTEQKTLIHDMAELRTTVDEEEMSWARKRVALDKEKAENERVKSNLDREMAVIKKQK
SLLDQSMAKFALERTDFLHQKAMIENKSNLKTLIETDCIVLEQEKTGPKQVKGLLDLESATAKQERDVCK
AKQASNTLTQRRAAEALSSSVERHSVVLEQEMYDAVPAKPRQAATQHHETEDTAPPGKVNAALKALQDLE
TDTEIQEMSSPGATEDRKTARSEIRRENVNSSFDQEKGHLELEKNALEKGKENFREDDIMAATMEENLDT
TMQYEVSTDVARSDIVTETGMAPSNTIFTKEECATLQANVSEIEDHFNTGVENTRILSKENLEQTPSEDG
RRHSMVTRSARKRSSRYSQETLYFTPLQTRPSLVGGQAPALDWSLNSLEDLVIDSAKKKPRTSSTRRRTM
HVIDITLNKKVESCRRDSVSSSASTMRSSTTTAYELRSHGDRPTPVQSSNWSLGFPASSASFVTLDTFTS
QNSLQLEDMRSGRLSDASLRSLPGYRPLGPHGTDGRNLEQLQAPPTTRRRKGNASYYMSCVDEPDPEAEW
DELERQGLGAHALPRRQPRAALGTSACYTEGLQENQPPVNCKVDFSEKSDTRNDHLLRQEELGKRNQATL
PHLRSCYPLKVETFMPGEDLCTAKPEMTARRMFLRPALADANADMRKRKSGIGHNTECSPESKKSATMCF
VVPATPRERPPPRASPRRDDHPQRSWPIIRRLRQSMSRKKSPSSKASKP

Branchiostoma belcheri:

>XP_019634543.1 PREDICTED: LOW QUALITY PROTEIN: nuclear mitotic apparatus protein 1-like [Branchiostoma belcheri]
MFNRGKLDALLGWVNLLGVGTVESLKELRDGNILVRIVQLIDPKIEYEAEDPAGRVEVVKSFLEGFHVTS
LGDDTYVRWHLIRQANEDEIAKAATLLLYTAFVSDAREQFMSKMDGRMGKAWEVTIKDILQFNITNNERK
GPRPDFADILKEQSMVSPDAICNGGGGRDGSLTHTNRRHVSVATPMSPGPVVTRLFDSPMSPVRNLMQSP
QMQYKIALTKAQQQVQQLKIELGTKKHIIDENDVQLRKMQRALEQKESEMRELSARLDGLRALQDENDAL
QARESERKQLEQRCEKLTKALHEYEVYKTECQELKKQNDRLFEERHGMEDAMKRCDQMALQVQELQTELH
CTGQQLQALRDSLKAKDQALQHMQQEKAQLLEQARHTEERLRETQLELQETMHSSGMESMGESLGSIVEK
RAQELEQELAYTQENWTKPEEAAKLVESLAAARAANASLEDNIRSLIAEGDKQRKDLTEHLDNAVQKYQT
AEQQLNEEMARHKAEIQQHQQEYQALKTDAAMEREALRSKLGAVSSTKTALEVTLENERWTFKAKEEAYR
EEILSTEKKLEGAKQKVAQLEEENSVTMAVMQDEKDALERQLKEVSAAAEQKCLQLNDQVSLLQEEKAAV
VANLEGYKARVEAEMTGLQKELEQTLHDKTSAESALHNTKEQLTAVSQEKAAITSALEQVKQQKEEEAGQ
LQDQLVTAATENSSLAADLESKAKLCRTLEAEKAMLADAIGKLGASSEKEKSELQSLMSDLQTERDSLQG
QLEDLQQQKEDLTQQNSDLHTELDEHSKDKGAALSKIKEVATSNTELKTSLKHSGKRLDSISDCLDILLS
CQEKAMDSLHQEHTELSLENRDLTSKVSELEKQLEESTGQLTHVTEEKESQVLNLELGLRDLQASLDKAE
QEKWSMQAEISQRERAAAVLQEQHAKRLQHLESESREQLERAEALRVQLLESNSRAAAAEQSCGKLQAEL
QTLQQTQADREAETEKQAADMQTELQFVQDRLILSESAKNRAEAQNADLCNELQTMKEQVLSLEADSKAQ
QQQLRDREAAMAAANKAGKELQGQLEISQVEREAVQQQLDMVHAALDQAEASKCDLKGQLSTAQQQLEQS
KSGSEELRRQLGTAQGQLEEMKTSRDDLQSHLQTAQEQLQQKDAVQGELQKQLQTAQVNLQQSEATRNDL
QNHLQTAQGTAPTKGQVCRVNQKQLQTVEANLQQTEATKNDLQSHLQTAQEQLQQKNTAQAELQKQLQTA
QANVQQTEAARNDLQSHLQIAQEQLKLKDTALGELQNQLQTAQASLQHTEATRNDLQSHLQTAQEHLQQK
DTAQVELQKQLQTAQVNLQQAECTRNDLQSQLQTAQEQLQQKDTVQGELQKQLQAAQANLQQTEATRNNL
QGQLHTAQEHLQQKDADLQNQLQTAAGKLEHSEAVRNDLQSQLQAMQEQLNKKDAAQGVLQIQLKTALAN
LQETEATKSNLQSQLQTAQGQLQEKCTAHDGLQVQLQSSMAKLEQSEASRSDLQGQLQTAQEQLQQKEAA
VGCLNSQLEASQVQLEKAEAGRNDLQSQVDLVQAQYMQSQNSSDETVAELRVQAAHFEQECSQLQEEKQQ
LLKQWEDMMAYKVRVEQQCSTLQRELGEAAAHSQQLELNNAQLCSQQQEMAAKEKSYRQKVEAMQQHFEE
QLRGLKELQGAMEEVQGQKNMLEEQLKMSQSQQQELSEQLHSYQMHYQRKKETIQDLEKSLQKQENAHES
VKNQLQTVQQERNKLKKENSSLSTDVKTAIEKLKEEEATTKKLYQQVRSLEAQVDLANRQLRQQKHQVSF
ATPAARSLNTSTDSIEDTADETFDLEPRSRQRHSTSRDDLDRTLSNSQVAGEEEGDKKNRTISTDSLEGD
SLDTSIFSRTSQDSGMSTRSQRRRTTVSIEMVQTEDSRRLSRTSTEHVDLGSSIGSASSWRGAAPRFFVS
ECEEEPEQYDWDRLSELQRRNTLQPRHLQSCYPTETQGTALEETEDLSKTLRRTTLRSSQAAKTFSITPG
KKRRHEADENSASRKKTVVMPSTPSKTPSVFKTPKKIASAAAGIFKKPTGKTPSSRRRSPRLQGRGKSPA
GRLNKAKTPTKTPNKHQAKQDPRRQSIAFNIGFSPLKGFRKSARKAKEKREAQKEPSQRRMSIANVKSFL
SVNNSKTNKTKAARKPLGTRNNAQTGV

Drosophila melanogaster:

No homolog found.

Hydra vulgaris:

No homolog found.

Nematostella vectensis:

>XP_032223093.1 nuclear mitotic apparatus protein 1 isoform X1 [Nematostella vectensis]
MNNERVTALLEWINSVSGVNVLFIRDFSTVENVKLLVKTIHLIDKDSDEVLPGYDDATVEGLVGFIISSL
EGIYRQKLDGIVINPNLITVDADEVEIGKVIALMLSAAVQCENANYFVKKIQSFEESVQYQIKEIIETLL
QQVEHGGLDASAITKLLKHTTGKNKEKQELPAGTDCVESPLPGKSPTETLFSSSPFLSLMSSPQLKTKQH
QFTMNKLHQKVSKLQLSLDAQMHMQATLEEELADKCKEIDVKDTKMKELNKDVVHLKMVIDELESTQHYK
ADYEKLERESERMKQRVAELEAFREQSCNLDERMADFLEKKAMFKKSVEDMEQLKTKCERYKSQLHEHEF
KASELKAALERRVKDVERLEKERDEALFAASEAMELWKARKSVEIPDEEERLDGDGSPVDTQHTNLDSRI
IELEFTNEKLRAQLASAIDPSEVQRLNDALEDAEQSKRSYEASYVEAKCKILELEDSVCKTETQFKVREK
ELNSRLEELTKNVELSQEKLTHNRKELSRLQSELSLARGEVEAHAIASEKQRATASKALDELLDERKAAE
QAACTRYNELRREMESKERRAEEKVEEIRRDCDHKIRHVTEELHGILETQRQEMRQKRDEFSTREDELKA
AVERVTEERDAVKNAYEEMVGDLKTELENEVKSRVLLEESLQQASTSWESAEAALTEAKAILEKELVSLK
ESQALLNEDNASLNKELVAMCEKEKYIISVNKELEAQISNLKMREEEEKREASRQIADLEEKLAGMVQEI
GRLESNIKGWEEEANVWVEEKAQIGKEVACLKDAFSTEMQTEKQSLEAKHSRDIEGMTKINEELKAKLCS
QKQSLDEVTQNYGRLQREMCATEDKLLQITRERNKIEEQLLAEQQGGEERAARQAEEMDHIKADHAKAMQ
EATDLLNRERDAFKAQSLELERACQGLKVAGDEIEGFKRSQNETQELLIHEKHRAKQMEEELEQLRQDFS
TKQKNLYEDTTKKQTQVRQEYEMERAAWKKKLETANEELVKLRGEMFENSQKHSAELICLRRQTQEKEDE
MEGEKKNMQEHTRQTQEKLQTSETIIKGLRADIKQLEIVKKTLEVEKGQLKSICEDFAKKEKDRLLEVEK
MTKDHEEKIAKIKNENQRALALKDEDAQKLMKETEELKAKMEDEHQKALGLKDEDIQKLMKETDELKAKM
EDEHQKALGLKDEDAQRLIKETEELKAKMAEIEEARKGEVAMAEEERRQKAEEQKKSMDAEIHELKRKLE
QSEESLKESDAKFTKTNDELDKVKKALETTQESWKEKETEMAAKLAQSEQLCEDMKDDQELLERLQDEIA

```
KMEEQVNVLNEEKGLLEEQLEEEQDTNKRLGMQVNSLEAQVKHADMTIREQKAKMQSFENQSEKVKKTRK
MLIEVEKVLPVQEAQELLDESTTESDDSEMRVDDLKLTRQGSSVSFASVNGTDFGKSLSSTSSISRTSLR
STVRPSASRRQSAMYVKGSTPPTRRTTSSAAFFIVGDEFANNMEQESEYEFDWDRLAELERRNTICPPHL
KTSYPVETQTRPSEGNGLHAARSETALTRKRKPEDTLTSSVKERKDMTTSKSEGQIKRSRSKRITDMVSS
KINSLRPSKSNENIKEKQKSGNSLAFEITPPKKRKAEPKAASDTTPRRESTKKETVAFTIENSPPKKCKV
QRTRTINRSTATTQLVRDKDRETFKREKKLETKNRIPLRSSNRKIAK
```

Amphimedon queenslandica:

No homolog found.

Alignment

```
XP_032223093.1[N.vectensis]        --MNNERVTALLEWINSVSGVNVLFIRDFSTVENVKLLVKTIHLIDKDSDEVLPGYDDAT   58
XP_019634543.1[B.belcheri]         -MFNRGKLDALLGWVNLLG--V-GTVESLKELRDGNILVRIVQLIDPK----I-EYEAED   51
XP_032819484.1[P.marinus]          -MVNGRKSAALISWVNCM--CA-EKTESLWDLKDLSTYLHIIQTIKAS-VDVK-PLLKSS   54
XP_045580741.1[S.salar]            MVLHLDKEHALLEWVNHLN-VD-LPVRSINDLQDGVLLMKLVYKLRKE-EPAN-SYLDQH   56
XP_018103294.2[X.laevis]           MALHSGKMEALICWVNSLK-VD-EPVERFSQLQDLNILLKVVGVLSGNLEETT-SVLQQN   57
CBN80053.1[G.gallus]               MPLHTTRATALLAWVNSTKACA-EPLGDLSQLQDCRVFIQIINKIHRT-EEGE-SVLEQS   57
NP_001390473.1[M.musculus]         MTLHATRAATLLSWVNSLH-VA-DPVETVLQLQDCSIFIKIINTIHDT-KEGQ-QILQQP   56
Q14980[H.sapiens]                  MTLHATRGAALLSWVNSLH-VA-DPVEAVLQLQDCSIFIKIIDRIHGT-EEGQ-QILKQP   56
XP_002822235.1[P.abelii]           MTLHTTRGSALLSWVNSLH-VA-DPVEAVLQLQDCSIFIKIIDRIHGT-EEGQ-QILKQP   56
                                     .:  :  :*: *:*            .  :.:    :: :  :

XP_032223093.1[N.vectensis]        VEGLVGFIISSLEGIYRQKLD--GIVINPNLITVDADEVEIGKVIALMLSAAVQCENANY   116
XP_019634543.1[B.belcheri]         PAGRVEVVKSFLEGFHVTSLGDDTYVRWHLIRQANED--EIAKAATLLLYTAFVSDAREQ   109
XP_032819484.1[P.marinus]          EYEKGNYIREFLHQFFQKN-ACLQTVDWDALTVGEHVELELAKLTVAFLCCAVVSTEIQS   113
XP_045580741.1[S.salar]            VQERLKVVSDFLQGDCRCSTERGALISWDNISNGLNLEVELSKVLVLLYYHSVIN--NHV   114
XP_018103294.2[X.laevis]           PEQRLNFLGSFLQKYCRPGSSAENLVQWQKILQGENLELEFSKVIVLLFYYSTMSSKNPK   117
CBN80053.1[G.gallus]               LAERAAFICGFLQKLCKHKSATENLVSAQKLLEGEEL--ELAKVAVLLLYHSSMSSKSPR   115
NP_001390473.1[M.musculus]         LPERLDFVCSFLQKNRKHPSSTQCLVSVQKVIEGSEM--ELAKMIMLFLYQSTMSSRNLR   114
Q14980[H.sapiens]                  VSERLDFVCSFLQKNRKHPSSPECLVSAQKVLEGSEL--ELAKMTMLLLYHSTMSSKSPR   114
XP_002822235.1[P.abelii]           VSERLDFVCSFLQKNRKHPSSPECLVSAQKVLEGSEL--ELAKMTMLLLYHSTMSSKSPR   114
                                          :   *.            :    :        *:.*    :   :

XP_032223093.1[N.vectensis]        FVKKI-QSFEESVQYQIKEIIETLLQQVEHGGLDASAITKLLKHT----TGK--------   163
XP_019634543.1[B.belcheri]         FMSKMDGRMGKAWEVTIKDILQFNITNNERKGPRPDFADILKEQSMVSPD-AICNGG---   165
XP_032819484.1[P.marinus]          RMKFL----DPDTQIQLHEILKFVMDNEESPQLQVNLELLLNVGCVVRSPGVFSIATEET   169
XP_045580741.1[S.salar]            DLNQL----EYKFEVELASMLRFVLDNENSLYLSENLEKYLRKKPLFSFNSDISSTS--S   168
XP_018103294.2[X.laevis]           KSEDF----DHKTQTELASILRFVLDNEESLCLDDKLINFLQRKARFPSSGD--------   165
CBN80053.1[G.gallus]               DWNEF----DYQIQVELATILKFVLDHEESLS--ENLEVFLQRRAPLSSPGT--------   161
NP_001390473.1[M.musculus]         DWEQF----EYGVQAELAVILKFMLDHEESLNLTEDLESFLEKV-PY--THA--------   159
Q14980[H.sapiens]                  DWEQF----EYKIQAELAVILKFVLDHEDGLNLNEDLENFLQKA-PVPSTCS--------   161
XP_002822235.1[P.abelii]           DWEQF----EYKIQAELAVILKFVLDHEDGLNLNEDLENFLQKA-PVPSTCS--------   161
                                     . :        :  :  ::.  : : :      .    *

XP_032223093.1[N.vectensis]        ---NKEKQELP-------AGTDCVESPLPGKSP--TET---LFSSSPFLSLMSSPQLKTK   208
XP_019634543.1[B.belcheri]         ---GGRDGSLT------HTNRRHVSVATPM-SPGPVVTRLFDSPMSPVRNLMQSPQMQYK   215
XP_032819484.1[P.marinus]          SGRFSSPLSSL--PLGHTAGVRFRDLQRIASSSMNNLLSSPGSPSSPMLSFFDQPAVEVR   227
XP_045580741.1[S.salar]            SSLFNDDESPVFQRRKKMGSVQFLDLQTVASSS----------VSSPLQDVMNTPQFQLK   218
XP_018103294.2[X.laevis]           DASGSSDAMSPYMSHRRKTEVRFLELHRVASSS-TIKSLPADAPSSPMIEVLHTPQFQMR   224
CBN80053.1[G.gallus]               SSSSSEERS-P---GLSHPQVRFLELQKIASSSSSMNNMLPGTPASPVGDVLQTPQFQLR   217
NP_001390473.1[M.musculus]         ST-LSEELS-P-PSHQTKRKIRFLEIQRIASSS-SENNFLSGSPSSPMGDILQTPQFQMR   215
Q14980[H.sapiens]                  ST-FPEELS-P-PSHQAKREIRFLELQKVASSS-SGNNFLSGSPASPMGDILQTPQFQMR   217
XP_002822235.1[P.abelii]           ST-FPEELS-P-PSHQAKREIRFLELQKVASSS-SGNNFLSGSPASPMGDILQTPQFQMR   217
                                                           .      *             **. ..:  * .: :

XP_032223093.1[N.vectensis]        QHQFTMNKLHQKVSKLQLSLDAQMHMQATLEEELADKCKEIDVKDTKMKELNKDVVHLKM   268
XP_019634543.1[B.belcheri]         IA---LTKAQQQVQQLKIELGTKKHIIDENDVQLRKMQRALEQKESEMRELSARLDGLRA   272
XP_032819484.1[P.marinus]          R--------------LKQQLRQEWEQQEKLEGELSSKSQLLKQSEQEVAELRGRVERLVQ   273
XP_045580741.1[S.salar]            K--------------LQRQLRQERDMRDELEKDLTTSATTLTQRESQICQLQHRIEKLL-   263
XP_018103294.2[X.laevis]           R--------------LKKQLSEVRECRDELEVELAQNHKHLAEKEAQISLLQQQIEHLRI   270
CBN80053.1[G.gallus]               R--------------LKKQLAIERENRDELEMELAENRKLITEKEAQITMMQHRIDRLAL   263
NP_001390473.1[M.musculus]         R--------------LKKQLADERSNRDDLELELSESLKLLTEKDAQIAMMQQRIDHLAL   261
Q14980[H.sapiens]                  R--------------LKKQLADERSNRDELELELAENRKLLTEKDAQIAMMQQRIDRLAL   263
XP_002822235.1[P.abelii]           R--------------LKKQLADERSNRDELELELAENRKLLTEKDAQIAMMQQRIDRLAL   263
                                                  *: .*          : :*      :   : ::  :   :  *

XP_032223093.1[N.vectensis]        VIDELESTQHYKADYEKLERESERMKQRVAELEAFREQS-------CNLDERMADFLEKK   321
XP_019634543.1[B.belcheri]         LQDENDALQARESERKQLEQRCEKLTKALHEYEVYKTECQELKKQNDR-------LFEER   325
XP_032819484.1[P.marinus]          LTQTRPPS-PLPSELAELHEKTQ----RLSV---TVKQCQELKKENELMEKRLTQLAEEN   325
XP_045580741.1[S.salar]            -REQAEQEQEPRDELQELHSKNEGLRTRLHE---VLKECQALKTNSSQMERKVDSLTEEN   319
XP_018103294.2[X.laevis]           LRENQA-ELQEPKELEELREKNESLMMRLRD---TLKQCQDMKTDKKQLERKNDQWAEEN   326
CBN80053.1[G.gallus]               LNEKQAADQLEPREMEELRERNESLLVRLHE---ALKQCQDLKTEKGQMDRKINQLSEEN   320
NP_001390473.1[M.musculus]         LNEKQAASSQEPSELEELRGKNESLTVRLHE---TLKQCQNLKTEKSQMDRKISQLSEEN   318
Q14980[H.sapiens]                  LNEKQAASPLEPKELEELRDKNESLTMRLHE---TLKQCQDLKTEKSQMDRKINQLSEEN   320
XP_002822235.1[P.abelii]           LNEKQAASPLEPKELEELRDKNESLTMRLHE---TLKQCQDLKTEKSQMDRKINQLSEEN   320
                                     :          :  :*. . :     :        :.                  *:.

XP_032223093.1[N.vectensis]        AMFKKSVEDMEQLKTKCERYKSQLHEH----------------------------EFKAS   353
XP_019634543.1[B.belcheri]         HGME-------DAMKRCDQMALQVQELQTELHCTGQQLQALRDSLKAKDQALQHMQQEKA   378
XP_032819484.1[P.marinus]          GELS-----------------SKVRESCTHLAECRS---LFEESSRSAEQRHAEAEVRIH   365
XP_045580741.1[S.salar]            GTLS-----------------AQMREVVARLASAEA---EVDRLIEAQDSAQGEWSSRHC   359
XP_018103294.2[X.laevis]           GELT-----------------YKVRDLSNRLAQLQE---ALYETTEEHERSLSNWQQKQN   366
CBN80053.1[G.gallus]               GDLS-----------------FKLREIASHLVQLQD---ALNELSEEHNSALMQSQAKQA   360
NP_001390473.1[M.musculus]         GDLS-----------------FKVREFANHLQQLQG---AFNDLIEEHSKASQEWAEKQA   358
Q14980[H.sapiens]                  GDLS-----------------FKLREFASHLQQLQD---ALNELTEEHSKATQEWLEKQA   360
XP_002822235.1[P.abelii]           GDLS-----------------FKLREFASHLQQLQD---ALNELTEEHSKATQEWLEKQA   360
                                     :                   ::::                               .

XP_032223093.1[N.vectensis]        ELKAALERRVKDVERLEKE-----------------------RDEALFAASEAMELW--   387
XP_019634543.1[B.belcheri]         QLL---EQARHTEERLRETQLELQE----------TMHSSGMESMGESLGSIVEKRAQEL   425
XP_032819484.1[P.marinus]          RLEEALGEALNSKASLEETVRILQGKLGALAEKEASLKEQRNLPPGEVMSEVLQMEEW--   423
XP_045580741.1[S.salar]            HLQDELNRATAQKECLSEQMLILQSKISSLEDELSK---AKMQEKGEVMGPILEWEQL--   414
XP_018103294.2[X.laevis]           QLESELSGAVGEKKYLEEHDLILQGKISMLEDQLKKMGEREVQETGDCMGDILKLDDL--   424
CBN80053.1[G.gallus]               QLEGELRAVLQEKKCLEEKVEILQGKISLLEDQLVKLGDCSTQEKGEVMGDVLKLEEL--   418
NP_001390473.1[M.musculus]         RLENELSTALQDKKCLEEKNEILQGKLSQLEDQATRLQESPAPEKGEVLGDALQLDTL--   416
Q14980[H.sapiens]                  QLEKELSAALQDKKCLEEKNEILQGKLSQLEEHLSQLQDNPPQEKGEVLGDVLQLETL--   418
XP_002822235.1[P.abelii]           QLEKELSAALQDKKCLEEKNEILQGKLSQLEEHLSQLRENPPQEKGEVLGDVLQLETL--   418
                                   .*             * :                              :.

XP_032223093.1[N.vectensis]        -------------------------KARKSVEIPDEEERLDGDGSP------------   408
XP_019634543.1[B.belcheri]         EQELAYTQENWTKPEEAAKLVESLAAARAANASLEDNIRSLIAEGDK-------------   472
XP_032819484.1[P.marinus]          HMCQKA--------------------RERADVGL----PPLAKAHTNTTHGHDTPTSSRE   459
XP_045580741.1[S.salar]            KQELAD--------------------ATLRHAECECTIARLKGEKEQAAALHAQERASLQ   454
XP_018103294.2[X.laevis]           KQEVAV--------------------HSTQCLSLKEQIRQLEEEKSIAEVEMEAQRSRFE   464
CBN80053.1[G.gallus]               KQEVSS--------------------LTTKGIELQASVLRLEEEKSQRDAALQAERNHFE   458
NP_001390473.1[M.musculus]         KQEAAK--------------------LATDNTQLQTRVETLECERGKQEAQLLAERSRFE   456
Q14980[H.sapiens]                  KQEAAT--------------------LAANNTQLQARVEMLETERGQQEAKLLAERGHFE   458
XP_002822235.1[P.abelii]           KQEAAT--------------------LAANNTQLQARVETLETERGQQEARLLAERDHFE   458
                                                                           *

XP_032223093.1[N.vectensis]        -----VDTQHTNLDSRIIELEFTNEKLRAQ-------LASAID--PSEVQRLNDALEDAE   454
XP_019634543.1[B.belcheri]         ----QRKDLTEHLDNAVQKYQ------------------TAEQQLNEEMARHKAEIQQHQ   510
XP_032819484.1[P.marinus]          EQSADFNNVS-DNDSSTL-IVK-----------DRKYLMPNESTMELEGSMV--------   498
XP_045580741.1[S.salar]            AESQRLQVLVTELQEALSALRADREALELASKEERESLTAQLHTLTAEVASLTQTVQQRE   514
XP_018103294.2[X.laevis]           SEKAQLQEIVTNLQTSLSEITFQKERLDNEARAQQEHLMCQMTTLKLEISKIKSSLMHKD   524
CBN80053.1[G.gallus]               MEKLQLGTHISNLQSSVSELHLAKEKLEQELRAQEAHLTAQADALTAEVGKLSGFLQQQE   518
NP_001390473.1[M.musculus]         DEKQQLASLIADLQSSVSNLSQAKEELEQASQAQGAQLTAQLTSM----TGLNATLQQRD   512
Q14980[H.sapiens]                  EEKQQLSSLITDLQSSISNLSQAKEELEQASQAHGARLTAQVASLTSELTTLNATIQQQD   518
XP_002822235.1[P.abelii]           EEKQQLSSLITDLQSSISNLSQAKEELEQASQAHGVRLTAQVASLTCELTTLNATIQQQD   518
                                              . :
```

```
XP_032223093.1[N.vectensis]      QSKRSYEAS------------------YVEAKCKILELEDSVCKTETQFKVREKELNSRL   496
XP_019634543.1[B.belcheri]       QEYQALKTDAAMEREALRSKLGAV-----SSTKT--ALEVTLENERWTFKAKEEAYREEI   563
XP_032819484.1[P.marinus]        ------------------------------------------DQYNVTFR-------EEL   509
XP_045580741.1[S.salar]          QEVKALGEEVQQECMQRGELNLAMERQDREAREEIQELTSHVDTMGASLRRAE----EEV   570
XP_018103294.2[X.laevis]         KELKDIHHKVEEERSEKNQLLENLKMLEESSKKDRQELIHQVEHLDSSLKVSE----GNL   580
CBN80053.1[G.gallus]             KEAAELRMQAEQERAQAEELRH----RDMASQEALSELSRRVEQLGASLKHSE----EEL   570
NP_001390473.1[M.musculus]       QELASLKEQAKKEQAQMLQTMQ-------EQEQAAQGLRQQVEQLSSSLKLKE----QQL   561
Q14980[H.sapiens]                QELAGLKQQAKEKQAQLAQTLQ-------QQEQASQGLRHQVEQLSSSLKQKE----QQL   567
XP_002822235.1[P.abelii]         QELAGLKQQAKEKQAQLAQTLQ-------QQEQASQGLRHQVEQLSSSLKQKE----QQL   567
                                                                                ::        .:

XP_032223093.1[N.vectensis]      EELTKNVE----------------------------------------------------   504
XP_019634543.1[B.belcheri]       LSTEKKLEGAKQKVAQLEEENSVTMAVMQDEK-------DALERQLKEVSAAAEQKCLQL   616
XP_032819484.1[P.marinus]        DGHESKQTGVSQDKC-----------------VEATRARSSALKQNSLEGSS---TSSR-   548
XP_045580741.1[S.salar]          QVREKQLTKQQQESA-------LQREVLQEEMA----ASEKALKELKKQEEAVREEATR-   618
XP_018103294.2[X.laevis]         VGITQQLESKTKE-----------ADYLREEQQKIICERDSSLSTLNEYKC---------   620
CBN80053.1[G.gallus]             VRVTQEAEGK--------------ARQLGAEHEKAVQERDAALQQLQHLQQ---------   607
NP_001390473.1[M.musculus]       EEAAKEQEATRQDHA-------QQLAIVAEAREASLRERDTARQQLETVEK---------   605
Q14980[H.sapiens]                KEVAEKQEATRQDHA-------QQLATAAEEREASLRERDAALKQLEALEK---------   611
XP_002822235.1[P.abelii]         KEVVEKQEATRRDHA-------QQLATAAEEREASLRERDAALKQLEALEK---------   611
                                     .:

XP_032223093.1[N.vectensis]      -----LSQEKLT----------------HNRKELSRLQSELSLARGEVE----------A   533
XP_019634543.1[B.belcheri]       NDQVSLLQEEKAAVVANL-----EGYKARVEAEMTGLQKELEQTLHDKTSAESALHNTKE   671
XP_032819484.1[P.marinus]        ------REMGIARGVQENFEFKNNGHRDTLEQGRTLLQQERVALSKER------------   590
XP_045580741.1[S.salar]          ------LHQEITTHVTDLC---------SLRQEHTALQEQLARQQEEI-------SLEKE   656
XP_018103294.2[X.laevis]         ----------------------------KKDEEFIALNGTLRTMEQDH-------QTSLS   645
CBN80053.1[G.gallus]             ----------------------------AKEKQLAALSSQLQSLEAAS----------KA   629
NP_001390473.1[M.musculus]       ----------------------------EKDAKLESLQQQLQAANDAR-------DNAQT   630
Q14980[H.sapiens]                ----------------------------EKAAKLEILQQQLQVANEAR-------DSAQT   636
XP_002822235.1[P.abelii]         ----------------------------EKAAKLETLQQQLQAANEAR-------DSAQT   636
                                                                      *.

XP_032223093.1[N.vectensis]      HAIASEKQRATASKALDELLDERKAAEQAACTRY--------------------------   567
XP_019634543.1[B.belcheri]       QLTAVSQEKAAITSALEQVKQQ--------------------------------------   693
XP_032819484.1[P.marinus]        --DILERERAALEKE----QMA-VIAMGAGN-----------------------------   614
XP_045580741.1[S.salar]          AQAAAHREKEAVEAELSRLQEE-VRSLGEQMAQLEEAQREKERLLLQSTENMETLQTERA   715
XP_018103294.2[X.laevis]         VIEELKREKAELASKVQELDAT-ILDLIAKCQNLDS------------------------   680
CBN80053.1[G.gallus]             SSMEMQQEKAELNQKVQELQAR-VLELSAQCQQSSA------------------------   664
NP_001390473.1[M.musculus]       SVTQAQQEKAELSQKIGELHAC-IEASH--------------------------------   657
Q14980[H.sapiens]                SVTQAQREKAELSRKVEELQAC-VETAR--------------------------------   663
XP_002822235.1[P.abelii]         SVTQAQREKAELSQKVEELRAC-VETAR--------------------------------   663
                                       :::

XP_032223093.1[N.vectensis]      ----------------------------------------------------------N-   568
XP_019634543.1[B.belcheri]       ------------------K---------------------EEEAGQLQDQLVTAATENSS   714
XP_032819484.1[P.marinus]        --RSILMGHMLTTEASIAQQRA---ILD--QE----KNKLNLEKA----ALKAMEDEIN-   658
XP_045580741.1[S.salar]          AASSLAEAKDLELSILREEVRAREEQLAMQQEEY------RLRQEELQEELAAQENEIN-   768
XP_018103294.2[X.laevis]         ENDSQSKSHAITVESLKAQLSEQESQVKIYQKKLSSNELVSKENSQLKEQLLSMEESVR-   739
CBN80053.1[G.gallus]             QAG--------SAETLRAQLRELEGKLKDSQQKLADKEKVAKENTRLQERLLFLEESVR-   715
NP_001390473.1[M.musculus]       --Q--------EQRQVQARVTELEAQLKAEQQKTTEREKVVQEKAQLQEQLRALEESLK-   706
Q14980[H.sapiens]                --Q--------EQHEAQAQVAELELQLRSEQQKATEKERVAQEKDQLQEQLQALKESLK-   712
XP_002822235.1[P.abelii]         --Q--------EQHEAQAQVAELEFQLRSEQQKATEKERVAQEKDQLQEQLRALKESLK-   712

XP_032223093.1[N.vectensis]      ---------ELRREMESKERRAEEKVE-------EIRRDCDHK---IRHV----------   599
XP_019634543.1[B.belcheri]       LAADLESKAKLCRTLEAEKAMLADAI-------GKLGASSEKEKSELQSL----------   757
XP_032819484.1[P.marinus]        --INLR---QQKSRLDQEKRKLEQDMIIL-----KQKWNCVE-QEQ-DNL----------   696
XP_045580741.1[S.salar]          ---------NMRE-----------RLAGLLDQISLLKEVCQE-GKNMEALREEHAAQLEQ   807
XP_018103294.2[X.laevis]         ---------NLSEHLDKEKMKFAASLDGDSKRISHLEE---E----MKKL----------   773
CBN80053.1[G.gallus]             ---------NTEGILEDEKRRAAESLEGNLARIAELEA---E----KQQL----------   749
NP_001390473.1[M.musculus]       ---------ITKGSLEEEKRRAADALKEQQCRATEMEA---E----SRSL----------   740
Q14980[H.sapiens]                ---------VTKGSLEEEKRRAADALEEQQRCISELKA---E----TRSL----------   746
XP_002822235.1[P.abelii]         ---------VTKGSLEEEKRRAADALEEQQRCISELKA---E----TRSL----------   746
                                                          :               .       :

XP_032223093.1[N.vectensis]      ----TEELH----------GILETQRQEMRQKRDEFSTREDELKAAVERVTEERDA----   641
XP_019634543.1[B.belcheri]       --------------------------------MSDLQTERDSLQGQLEDLQQQ-------   778
XP_032819484.1[P.marinus]        ----------------------------------------------VKE----RAIEDQS   706
XP_045580741.1[S.salar]          LRLVKEQVEQVQERNEETSAALREKESSLREKESSLREKEESLRGLEEELQSTTSLASQR   867
XP_018103294.2[X.laevis]         -----------------------------------TESRDAALRNLDEERTAGKKIESQL   798
CBN80053.1[G.gallus]             -----------------------------------VQRGEQALQQHSEELARRQALETRL   774
NP_001390473.1[M.musculus]       -----------------------------------MEQREREQKELEQEKAGRKGLEARI   765
Q14980[H.sapiens]                -----------------------------------VEQHKRERKELEEERAGRKGLEARL   771
XP_002822235.1[P.abelii]         -----------------------------------VEQHKREQKELEEERAGRKGLEARL   771
                                                                                :

XP_032223093.1[N.vectensis]      -------V------------------------KNAYEEMVGDLKTELENEVK--------   662
XP_019634543.1[B.belcheri]       --------------------------------KEDLTQQNSDLHTELDEHSKDKGAALSK   806
XP_032819484.1[P.marinus]        K----ASFTKEIIYLKA----------------------------SLEEETSGMK-----   729
XP_045580741.1[S.salar]          QQEELTSLKEEVISLQEEVERRRAAEALAVEEARELEESVSALQQQLDSAIQDNDMKRQK   927
XP_018103294.2[X.laevis]         KN-----L------------------------EEEYHRSNESQHAQLAESCAAIKQREEE   829
CBN80053.1[G.gallus]             QQ-----V------------------------AEERREETAALQRQLAEVAKGEEGERGR   805
NP_001390473.1[M.musculus]       QQ-----L------------------------EEAHQAETEALRHELAEATASQHRAESE   796
Q14980[H.sapiens]                QQ-----L------------------------GEAHQAETEVLRRELAEAMAAQHTAESE   802
XP_002822235.1[P.abelii]         QQ-----L------------------------GEAHQAETEVLRQELAEAIAAQRTAESE   802
                                                                              .* .

XP_032223093.1[N.vectensis]      ---------SRVLLEESLQQASTSWESA-EAALTEAKAILEKELVSLKESQ---------   703
XP_019634543.1[B.belcheri]       I----KEVAT----------------------------SNTELKTSLKHSGKRLDSISDC   834
XP_032819484.1[P.marinus]        -----ARVEQERVLLEN---------------------EREKIIQSITANQ---------   754
XP_045580741.1[S.salar]          CERLEQDLEQRGTLVEELRQQEHS-----------ARLEATRLRQEISTHLSRLEVVQRE   976
XP_018103294.2[X.laevis]         RDELSKVVDIWKAKYGESQQKIAQNSCHMQEQIEELKKTHSDVYQQLEGERSKVLMIEAK   889
CBN80053.1[G.gallus]             LEKRLQE----------------------------LSREHGQACQRLQAEQERVAELEAR   837
NP_001390473.1[M.musculus]       CERLIREVESRQKRFEARQQEEARYGAMFQEQLMALKGEKTGQ--EVQ-----EEAVEIH   849
Q14980[H.sapiens]                CEQLVKEVAAWRERYEDSQQEEAQYGAMFQEQLMTLKEECEKARQELQEAKEKVAGIESH   862
XP_002822235.1[P.abelii]         CEQLVKEVAAWRERYEDSQQEEAQYGAMFQEQLMTLKEECEKARQELQEAKEKVAGIESH   862
                                                                               :

XP_032223093.1[N.vectensis]      -----ALLNEDNASLNKELVAMCE---------------------------------KEK   725
XP_019634543.1[B.belcheri]       LDILLSCQEKAMDSLHQEHTELS--------LENRDLTSKVSELEKQLEE-------STG   879
XP_032819484.1[P.marinus]        ---------NDTASLQRERIAMQQ-EVDVREQARSALICVVAA-LEKIEV----MPGNE-   798
XP_045580741.1[S.salar]          KEELRG-----EVSLHQQRAAVLQQSLEEQEVAMRVLKEQKESIREETTVKIEALQAQLE   1031
XP_018103294.2[X.laevis]         ASETKSSQLEKINQLEGELSAANA-CIKEREAEEKKLVSALHSAEEKLKIA---YQGESE   945
CBN80053.1[G.gallus]             VARMASEQQEQLAALQDQLARAKE-K----EGKE----------QEKL-A-------SAE   874
NP_001390473.1[M.musculus]       SEGQPGQQQSQLAQLHASLAKAIQ-QVQEKEVRAQKLVDDLSALQEKMAA-------TNK   901
Q14980[H.sapiens]                SELQISRQQNELAELHANLARALQ-QVQEKEVRAQKLADDLSTLQEKMAA-------TSK   914
XP_002822235.1[P.abelii]         SELQISRQQNELAELHANLARALQ-QVQEKEVRAQKLADDLSTLQEKMAA-------TSK   914
                                                 *. .

XP_032223093.1[N.vectensis]      YIISVNKELEAQISNLKMRE----------EEEKREASRQIADLEE----KLAGMVQEIG   771
XP_019634543.1[B.belcheri]       QLTHVTEEKESQVLNLELGLRDLQASLDKAEQEKWSMQAEISQRERAAAVLQEQHAKRLQ   939
XP_032819484.1[P.marinus]        -KGSV-------ICALEEGMTTLKAIL--------GLDKGIACEGTH----ID-------   831
XP_045580741.1[S.salar]          VVSSLAAAKDLQLSTLREEATALQEQLAKREQ-EISLQKEVLQEAHREKESLEAL-----   1085
```

```
XP_018103294.2[X.laevis]         RLSHL------------------ETALSNAKQDLDCLAKELSDEKYKKA-EFEAM-----   981
CBN80053.1[G.gallus]             RVGKL------------------EAEVRKASEALEAVSKELSEE----------------   900
NP_001390473.1[M.musculus]       EVACL------------------KTLVLKAGEQQETASLELLKEPPRAA-NRA-------   935
Q14980[H.sapiens]                EVARL------------------ETLVRKAGEQQETASRELVKEPARAG-DRQ-------   948
XP_002822235.1[P.abelii]         EVARL------------------ETLVRKAGEQQETASRELVKEPARAG-DGQ-------   948
                                     :                                   :

XP_032223093.1[N.vectensis]      RLESNIKGWEEEANV---------------------------------------WVEEK   791
XP_019634543.1[B.belcheri]       HLESESREQLER-AEALRVQLLESNSRAAAAEQSCGKLQAELQTLQQTQADREAETEKQA   998
XP_032819484.1[P.marinus]        -----------MADNGIVAQAVLQQQRTEP------------------------------   850
XP_045580741.1[S.salar]          ------REELARQQEELREELILQQQRAQSLEQSLEEQQEALKEL-------T----LKE   1128
XP_018103294.2[X.laevis]         ------VKVLKEQNS----------ERIASLESELKNSLAVVKER-------K----CES   1014
CBN80053.1[G.gallus]             ---------------------------------------RLKFKEL-------E----AVA   911
NP_001390473.1[M.musculus]       ------SDQLGEQQG----------RPF-------SSTHAAVKAM-------E----REA   961
Q14980[H.sapiens]                ------PEWLEEQQG----------RQF-------CSTQAALQAM-------E----REA   974
XP_002822235.1[P.abelii]         ------PEWLEEQQG----------RQF-------CSTQAALQAM-------E----REA   974


XP_032223093.1[N.vectensis]      AQIGKEVACLKDAFSTEMQTEKQSLEA-------------KHSRDI--------------   824
XP_019634543.1[B.belcheri]       ADMQTELQFVQDRLILSESAKN----RAEAQNADLCNELQTMKEQVLSLEADSKAQQQQL   1054
XP_032819484.1[P.marinus]        -------A--QDNYFISVGKHGM--GHRE--RDTLCQEKDKT--NI---E----------   882
XP_045580741.1[S.salar]          ERAREEATQLRQQIST-------------------------HLSRL---E----------   1150
XP_018103294.2[X.laevis]         EKLSGEVEHLKRQLDDSSQKHKEALAQKNIEIKQLIDAKEKATSDL--------------   1060
CBN80053.1[G.gallus]             TQEASEVARLSAALEEAAREHGQELARREEEAKRLQKELEDAKADC---A----------   958
NP_001390473.1[M.musculus]       EQMGGELERLRAALIKSQGQQQEERGQQEREVARLTQERGQAQADL---A----------   1008
Q14980[H.sapiens]                EQMGNELERLRAALMESQGQQQEERGQQEREVARLTQERGRAQADL---A----------   1021
XP_002822235.1[P.abelii]         EQMGNELERLRAALMESQGQQQEERGQQEREVARLTQERGRAQADL---A----------   1021


XP_032223093.1[N.vectensis]      -------EGMTKINEELKAKL-------CSQKQSLDEVTQNYGRLQREMCATEDKLL---   867
XP_019634543.1[B.belcheri]       RDREAAMAAANKAGKELQGQLEISQVEREAVQQQLDMVHAALDQAEASKCDLKGQLS---   1111
XP_032819484.1[P.marinus]        ---ETTWDGERSLSQ------RESLVS-------LDQSNDVLDRDQ--------------   912
XP_045580741.1[S.salar]          ---EVQ-RGKEELREQEEKAREETTVKMEALQAQLEVVSSLAAAKDLQLSTLREEASLLC   1206
XP_018103294.2[X.laevis]         -------AIKSEMGAQLQKAVDTHKSEFSALQNELSRSLDFIALKEGEVERLNKEAA---   1110
CBN80053.1[G.gallus]             ---AEK-ARKVELEVQLQNSINEQRVERSAHQQELARSLELIEEKEGELDELRLKNV---   1011
NP_001390473.1[M.musculus]       ---QEK-AAKAELEMRLQNTLNEQRVEFAALQEALAHALTEKEGTDQELAKLRGQEA---   1061
Q14980[H.sapiens]                ---LEK-AARAELEMRLQNALNEQRVEFATLQEALAHALTEKEGKDQELAKLRGLEA---   1074
XP_002822235.1[P.abelii]         ---LEK-AARAELEMRLQNALNEQRVEFATLQEALAHALTEKEGKDQELAKLRGLEA---   1074
                                            .                      *          :

XP_032223093.1[N.vectensis]      -----------QITRERNKIEEQLL--A---------------------EQQGGEERAAR   893
XP_019634543.1[B.belcheri]       -----------TAQQQLEQ-------------------------SKSGSEELRRQLGTAQG   1136
XP_032819484.1[P.marinus]        -------ADLETVRDVLLKADTKMAVLAPMQESAAVKDFMEEDCSPLDQEDS--------   957
XP_045580741.1[S.salar]          QENTKRAADLKDVQLEKIRLESLLS---E--EHRALK----EDLAKQQEELRGEVSLHQQ   1257
XP_018103294.2[X.laevis]         ----LRQEEIQQQQQTMTKLTEELT------ALAALK----EKVALQEKEIKQQVQATKG   1156
CBN80053.1[G.gallus]             ----SRGEELRDLQKTVSKLKGELA------SVEAVK----ERASKMDSELQGFLEAARS   1057
NP_001390473.1[M.musculus]       ----AQRTELKELQQTLEQLKIQLV------KKEKEH----PAGGASGEDASG-------   1100
Q14980[H.sapiens]                ----AQIKELEELRQTVKQLKEQLA------KKEKEH----A---------SG-------   1104
XP_002822235.1[P.abelii]         ----AQIKELEELRQTMKQLKEQLA------KKEKEH----A---------SG-------   1104
                                                   :

XP_032223093.1[N.vectensis]      QAEEMDHIKADHAK---A-----------------MQEATDLLNRERDAFKAQSLELERA   933
XP_019634543.1[B.belcheri]       QLEEMKT---SRDDLQSHLQTAQEQLQQKDAVQGELQKQLQTAQV----------NLQQ-   1182
XP_032819484.1[P.marinus]        -DATVRNQKAFTPA--ETVRLVQANLDHEQI-------VF------RDSLQHEVAVLEQK   1001
XP_045580741.1[S.salar]          RAAELQ---LSLEEKQEALRELKEQLVQQQE-DSSL------------------------   1289
XP_018103294.2[X.laevis]         AEEEMANLKSIISEKSKRIECLEQDIKNQKTDLSSIQEQYQSKLSEAQGHQALIADLEKK   1216
CBN80053.1[G.gallus]             RDAEMDSIKAVYAK-EASLKNLEEKIRHREQESGSSQDLYQEKLKEAQMLSVEVERLEQK   1116
NP_001390473.1[M.musculus]       -------------------PGTQSET------AG----KTDAPGPELQALRAEISKLEQQ   1131
Q14980[H.sapiens]                -------------------SGAQSEA------AG----RTEPTGPKLEALRAEVSKLEQQ   1135
XP_002822235.1[P.abelii]         -------------------SGAQSEA------AG----RTEPTGPKLEALRAEVSKLEQQ   1135


XP_032223093.1[N.vectensis]      CQGLKVAGDEIEGF--------------------------------KRSQNETQELLIHE   961
XP_019634543.1[B.belcheri]       ---SEATRNDLQNHLQTAQGTAPTKGQVCRVNQKQLQTVEANLQQTEATKNDLQSHLQTA   1239
XP_032819484.1[P.marinus]        R-----------------------------------TTLTASLEQERNRLCELEQASMSV   1026
XP_045580741.1[S.salar]          -----------QKVLQEA--------QAAALQQEAVDALRGEVSLHQQRAAELQHQEAAL   1330
XP_018103294.2[X.laevis]         CKEQKELICEAQNKAAEAKTLASEKASMSEKQLKGIQTLENEIQKERQKTCDLQKQLEVS   1276
CBN80053.1[G.gallus]             CREQQDTIAGLEKAVAEG----------SQQQQAELEALQREAVRHRETAAELQRLLDAS   1166
NP_001390473.1[M.musculus]       CQQQQQQVEGLTHS------------------------------------------LKSE   1149
Q14980[H.sapiens]                CQKQQEQADSLERS------------------------------------------LEAE   1153
XP_002822235.1[P.abelii]         CQQQQKQADSLERS------------------------------------------LEAE   1153


XP_032223093.1[N.vectensis]      K----------------------------HRAKQMEEELEQLRQDFSTKQKNLYEDTTK   992
XP_019634543.1[B.belcheri]       -----------------------------------QEQLQQKN-------TAQAELQKQ   1256
XP_032819484.1[P.marinus]        RME-----------ADSLKAA--LQLEEI-----VFRATL--------------NEERTV   1054
XP_045580741.1[S.salar]          REQEEKSTEEATLKMEALKAAKDLQLSALTEKATALQEQLAKREQEISLQKEVLQEAHRE   1390
XP_018103294.2[X.laevis]         Q---------------ALQAEKETEL-------EALKKELFHKVQELEQSQKSFTDSNRE   1314
CBN80053.1[G.gallus]             R---------------SAQALQDGTV-------ESLRKELQDKSKELAQSKMAVAAAEKE   1204
NP_001390473.1[M.musculus]       R---------------ACRAEQDKAL-------ETLQGQLEEKARELGHNQAASASAQRE   1187
Q14980[H.sapiens]                R---------------ASRAERDSAL-------ETLQGQLEEKAQELGHSQSALASAQRE   1191
XP_002822235.1[P.abelii]         R---------------ASRAERDSSL-------ETLRGQLEEKAQELGHSQSVLASAQQE   1191
                                                                    .  *

XP_032223093.1[N.vectensis]      K--QTQVRQEYEMERAAWKKKLETANEEL-------------------------------   1019
XP_019634543.1[B.belcheri]       LQTAQANVQQTEAARNDLQSHLQIAQEQLKLKDTALGELQNQLQTAQASLQHTEATR---   1313
XP_032819484.1[P.marinus]        LEEMKFKVQDNTLLQDQEKVK-----------------LQRDRDLLEDEKVSIERERV--   1095
XP_045580741.1[S.salar]          KESVEALREELARQQEELREELIL---QQQRAQSLEQSLEEQQEALKELTLKEERAREEA   1447
XP_018103294.2[X.laevis]         LSSMLSEVQERQQALTEAKEQAEQYQKVIDMKNKEVNSLQAEINILSSKVTSKEEVS---   1371
CBN80053.1[G.gallus]             VASLRAAAQEKGHLEEGWKEQLSQCIQELERKNSLLGSLEHEVSILHRQLTEKEGES---   1261
NP_001390473.1[M.musculus]       LQALRAKAQDHSKAEEEWKAQVARGQQEAERKSSLISSLEEEVSILNRQVLEKEGES---   1244
Q14980[H.sapiens]                LAAFRTKVQDHSKAEDEWKAQVARGRQEAERKNSLISSLEEEVSILNRQVLEKEGES---   1248
XP_002822235.1[P.abelii]         LAALRTKVQDHSKAEDEWKAQVARGRQEAERKNSLISSLEEEVSILNRQVLEKEGES---   1248
                                         ::        : .

XP_032223093.1[N.vectensis]      ------------------------------------------------------------   1019
XP_019634543.1[B.belcheri]       ----NDLQSHLQTAQ------EHL-QQKDTAQVEL-------QKQLQT--AQVNLQQAEC   1353
XP_032819484.1[P.marinus]        ---------ALDK-----------------MKA-TLEEMKAIWERERVVLVAPLEQE---   1125
XP_045580741.1[S.salar]          TQLRQQISTHLSRLEEVQRGKEELREQEEKAREETTVKMEALQAQLEV--VSSLAAAKDL   1505
XP_018103294.2[X.laevis]         --------ADFEQ----------RLLQETSKSAKLEEKLKKLHTEVEV--SSKELVEKNC   1411
CBN80053.1[G.gallus]             --------KELKR----------LIMAESEKSKKLEERLRVLQTEMAT--AASRAAER--   1299
NP_001390473.1[M.musculus]       --------KELKR----------LVVAESEKSQKLEERLRLLQVETAS--NSARAAER--   1282
Q14980[H.sapiens]                --------KELKR----------LVMAESEKSQKLEERLRLLQAETAS--NSARAAER--   1286
XP_002822235.1[P.abelii]         --------KELKQ----------LVMAESEKSQKLEERLRLLQAETAS--NSARAAER--   1286


XP_032223093.1[N.vectensis]      ---------VKLRGEMFENSQK---------------HSAELICLRRQT---QEKEDEME   1052
XP_019634543.1[B.belcheri]       TRNDLQSQLQTAQEQLQQKDTV----QGE--------LQKQLQAAQANLQQTEATRNNLQ   1401
XP_032819484.1[P.marinus]        -RVTLKRELAALHQERAKWTTE-----SERLVQEQALLEQQRVALQQERESLD----QHR   1175
XP_045580741.1[S.salar]          QLSTLREEASLLCQENTKRAADLKDVQLEKIRL-ESLLSEEHRALKEDL---AKQQEELR   1561
XP_018103294.2[X.laevis]         AVDILRTEAQTFKGEADEQRMA-----VD-------------CLQQKLSSQAETNNNLQ   1452
CBN80053.1[G.gallus]             -CSLMKVEVQRCQEEMEKQRMT-----IE-------------ALKRDRHCQSEREEELR   1339
NP_001390473.1[M.musculus]       -SSALREEVQSLREEVEKQRVV-----SE-------------NSRQELASQAERAEELG   1322
Q14980[H.sapiens]                -SSALREEVQSLREEAEKQRVA-----SE-------------NLRQELTSQAERAEELG   1326
```

```
XP_002822235.1[P.abelii]         -SCALREEVQSLREEAEKQRVA-----SE-------------NLRQELTSQAERAEELG   1326
                                               :  :                           : .         :

XP_032223093.1[N.vectensis]      GE--KKNMQEHTR---------QTQEKLQT------SE-TIIKGLRADIKQLEIVKKTLE   1094
XP_019634543.1[B.belcheri]       GQ--LHTAQEHL---------QQKDADLQNQLQTAAG--KLE--HSEAVRN---------   1437
XP_032819484.1[P.marinus]        GNLKVKSEQERAAQSETMYKVEQENACLRT------EQKTLI-HDMAELRTT--------   1220
XP_045580741.1[S.salar]          GE--VSLHQQRAA------ELQHQEAALREQEEKSTEEATLKMEALKAAKDLQLSAL---   1610
XP_018103294.2[X.laevis]         QE--IQSWQKNCA------EKEQKICSLQ-QNLNSNQ--SLL-EEFASLKRS--------   1492
CBN80053.1[G.gallus]             QE--VKVCQDKFF------QKEQLLSSLQ-QELGSAQ--ALA-GEVVPLKHL--------   1379
NP_001390473.1[M.musculus]       QE--LKAWQEKFF------QKEQALSALQ-LEHTSTQ--ALV-SELLPAKHL--------   1362
Q14980[H.sapiens]                QE--LKAWQEKFF------QKEQALSTLQ-LEHTSTQ--ALV-SELLPAKHL--------   1366
XP_002822235.1[P.abelii]         QE--LKAWQEKFF------QKEQALSTLQ-LEHTSTQ--ALV-SELLPAKHL--------   1366
                                  :      *..           :    *:           :        :

XP_032223093.1[N.vectensis]      VEKGQLKSICEDFAKKEKDRLLEVEKMTKDHEEKIAKIKNENQRALALKDEDAQKLMKET   1154
XP_019634543.1[B.belcheri]       -----------DLQ---SQL--------QAMQEQLNKKDAAQGV-LQIQLKTALANLQET   1474
XP_032819484.1[P.marinus]        ------------VD--EEEM------SWARKRVALDKEKAENER-VKSNLDRE-------   1252
XP_045580741.1[S.salar]          --TEKAIALQEQLAKREQEISLQKEVLQEAH-----REKESLEA-LREELARQQEELREE   1662
XP_018103294.2[X.laevis]         ----------------YQEILAERGLMQQKHQEELLSHKNLTER-FQAELEKTKEDMAEI   1535
CBN80053.1[G.gallus]             ----------------CQQLQAERASLESKHREDLEQRAKATTA-LQAELARARVEVAEL   1422
NP_001390473.1[M.musculus]       ----------------CQQLQAEQAAAEKRFREELEQSKQAAGG-LQAELMRAQRELGEL   1405
Q14980[H.sapiens]                ----------------CQQLQAEQAAAEKRHREELEQSKQAAGG-LRAELLRAQRELGEL   1409
XP_002822235.1[P.abelii]         ----------------CQQLQAEQAAAEKRHREELEQSKQAAGG-LRAELLRAQRELGEL   1409
                                                  .:                          .  :

XP_032223093.1[N.vectensis]      EELKAKM--------E--------------------------------------------   1162
XP_019634543.1[B.belcheri]       EATKSNL-----------------------------------------------------   1481
XP_032819484.1[P.marinus]        MAVIKKQKSLLDQSMA-------------------------------------------K   1269
XP_045580741.1[S.salar]          LILQQQRAQSLEQSLEEQQEALKELTLKEERAREEATLKMVALQAAKDLELSTLRQEATQ   1722
XP_018103294.2[X.laevis]         VLLKEKL--------NNQELQLH------------------------------------K   1551
CBN80053.1[G.gallus]             PALRDRA--------AEQERALQ------------------------------------R   1438
NP_001390473.1[M.musculus]       GSLRQKI--------VEQERAAQ------------------------------------Q   1421
Q14980[H.sapiens]                IPLRQKV--------AEQERTAQ------------------------------------Q   1425
XP_002822235.1[P.abelii]         IPLRQKV--------AEQERTAQ------------------------------------Q   1425
                                      .

XP_032223093.1[N.vectensis]      ------D-EH-------------QKALGLKDEDIQKLMKETDELKAKMEDE--------   1193
XP_019634543.1[B.belcheri]       --------QSQL-----------QTAQGQLQE----KCTAHDGLQVQLQSSMAKLEQSE   1517
XP_032819484.1[P.marinus]        FALERTDFLHQKAMIENKSNLKTLIETDCIVLE----Q--EKTGPKQ--VK---------   1312
XP_045580741.1[S.salar]          LRQEISTHVSHLEEV----------KSEGPLRE-------EHTALQVQLAKQQ-------   1758
XP_018103294.2[X.laevis]         LQSENSDQSSQISNL---------QQVNSRLLG-------ENQSLSQISDQDA-------   1588
CBN80053.1[G.gallus]             LQKETASSSERLAAL---------QAANSRLAE-------ENRALSESVSRGQ-------   1475
NP_001390473.1[M.musculus]       LRAEKASYAEQLSML---------KKAHGLLAE-------ENRGLGERANLGR-------   1458
Q14980[H.sapiens]                LRAEKASYAEQLSML---------KKAHGLLAE-------ENRGLGERANLGR-------   1462
XP_002822235.1[P.abelii]         LRAEKASYAEQLSML---------KKAHGLLAE-------ENRGLGERANLGR-------   1462


XP_032223093.1[N.vectensis]      -----HQKALGLKDE--------DAQRLIKETEELKA-------KMAEIEEAR--KGEVA   1231
XP_019634543.1[B.belcheri]       ASRSDLQGQLQTAQEQ-----------LQQKEAAVGCLNSQLEASQVQLEKAEAGRNDLQ   1566
XP_032819484.1[P.marinus]        ---GLLDLESATAKQERDVCKAKQASNTLTQRRAAEALSSSVERHSVVLEQE--------   1361
XP_045580741.1[S.salar]          ---EENSLQKGLLQE-------VQ-ATVLQEREAKEALRGEVSLHQQSL--EE---QQVA   1802
XP_018103294.2[X.laevis]         ---KKLSIEMSTLKE-------Q-------HEEEMKTLRLQYEKTQREGNEQV---QDLS   1628
CBN80053.1[G.gallus]             ---QRLDAELGQARE-------K-------HTRELECVRLEAEKAMASSRQEA---EEAA   1515
NP_001390473.1[M.musculus]       ---QFLEVELDQARE-------K-------YVQELAAVRTDAETHLAEMRQEA---QSTS   1498
Q14980[H.sapiens]                ---QFLEVELDQARE-------K-------YVQELAAVRADAETRLAEVQREA---QSTA   1502
XP_002822235.1[P.abelii]         ---QFLEVELDQARE-------K-------YVQELAAVRADAETRLAEVQREA---QSTA   1502
                                       .       :

XP_032223093.1[N.vectensis]      MAEEERRQKAEE-----------------------------------------QKKSMD   1249
XP_019634543.1[B.belcheri]       SQVDLVQAQYMQSQNSSDETVAELRVQAAHFEQECSQLQEEKQQLLKQWEDMMAYKVRVE   1626
XP_032819484.1[P.marinus]        --------MYDA-------------------------------VPAKPRQAATQHHETE   1381
XP_045580741.1[S.salar]          VRETLARQKEEGQ--------AAGQV-QKELMEQFSVLQQEKEALLTRALQAEQNQSELE   1853
XP_018103294.2[X.laevis]         LRLETATSKYDH-------------V-KSKVLKDQKTFQEEKQMLLLRVEELAAAKKEQS   1674
CBN80053.1[G.gallus]             RKLEAMSNKYEN-------------T-KVKVLEERQKFQEERQKLMAQVEQLEVFQKEQA   1561
NP_001390473.1[M.musculus]       RELEVMTAKYEG-------------A-KVKVLEERQRFQEERQKLTAQVEQLEVFQREQT   1544
Q14980[H.sapiens]                RELEVMTAKYEG-------------A-KVKVLEERQRFQEERQKLTAQVEQLEVFQREQT   1548
XP_002822235.1[P.abelii]         RELEVMTAKYEG-------------A-KVKVLEERQRFQEERQKLTAQVEQLEVFQREQT   1548
                                                                                        :

XP_032223093.1[N.vectensis]      AEIHELKRKLEQSEESLK---ESDAKFTKTND----------------ELDKV-------   1283
XP_019634543.1[B.belcheri]       QQCSTLQRELGEAAAHSQQLELNNAQLCSQQQ-----EMAAKEKSYRQKVEAMQQHFEEQ   1681
XP_032819484.1[P.marinus]        --------------DTAPPGKVN-AALKALQDLETDTEIQEMSSP--------------   1411
XP_045580741.1[S.salar]          GSMAELRAQAE-SRESGQRQQLD-ALLLEKERLTEGHQVLEMKCSAAQRLEAVLL---EE   1908
XP_018103294.2[X.laevis]         EQVQE----------------LN-KQLRQQEKTLQ---------SQQQKLKQR-------   1701
CBN80053.1[G.gallus]             KQVEE----------------LN-KKLVQHEKATR---------SQQQRVKVL-------   1588
NP_001390473.1[M.musculus]       KQVEE----------------LS-KKLTEHDQASK---------VQQQKLKAFQA---Q-   1574
Q14980[H.sapiens]                KQVEE----------------LS-KKLADSDQASK---------VQQQKLKAVQA---Q-   1578
XP_002822235.1[P.abelii]         KQVEE----------------LS-KKLVESDQASK---------VQQQKLKAVQA---Q-   1578
                                                       .   :   :

XP_032223093.1[N.vectensis]      ---KKALETTQESWKEKETEMAAKLAQSEQLCEDMKDD--------------Q------   1319
XP_019634543.1[B.belcheri]       LRGLKELQGAMEEVQGQKNMLEEQLKMSQSQQQELSEQLHSYQMHYQRKKETIQDLEKSL   1741
XP_032819484.1[P.marinus]        --GA-TEDRKTARSEIRRENVNSSFDQEKGHLELEKNALEKGKENFREDDIMAATMEENL   1468
XP_045580741.1[S.salar]          LALL-REQMEGTEWEKQIRDLREQLAANTEVVEHYKTQVEKAKSHYSGKKQQLVESQEQV   1967
XP_018103294.2[X.laevis]         --EG-ETHEEAEKTHKRVIELESQLEQQTQAVEHYKAQMEKAKVHYDAKKKQNQELSDEL   1758
CBN80053.1[G.gallus]             --EG-ELQSEATRQQERVAELEEQLAQKEQAAEHYKAQMEKAKTHYDAKKQQNQDLAEKL   1645
NP_001390473.1[M.musculus]       --RG-ESQQEVQRLQTQLNELQAQLSQKEQAAEHYKLQMEKAKTHYDAKKQQNQKLQEQL   1631
Q14980[H.sapiens]                --GG-ESQQEAQRLQAQLNELQAQLSQKEQAAEHYKLQMEKAKTHYDAKKQQNQELQEQL   1635
XP_002822235.1[P.abelii]         --GG-ESQQEAQRLQAQLNELQAQLSQKEQAAEHYKLQMEKAKTHYDAKKQQNQELQEQL   1635
                                        .      . :   :  .:  .    :  .

XP_032223093.1[N.vectensis]      ------------------ELLERLQDEIAKMEEQVNV--LNEEKGLLEEQLEEEQDTNKR   1359
XP_019634543.1[B.belcheri]       QKQENAHESVKNQLQTVQQERNKLKKENSS---------LSTDVKTAIEKLKEEEATTKK   1792
XP_032819484.1[P.marinus]        D--------------TTM------QYE---VSTDVARSDIVTETGMAPSNTIFTKEECAT   1505
XP_045580741.1[S.salar]          T--------------ELQRSLEVREHEVNAVTTEMKL--LQKELEK-------ARNKEKS   2004
XP_018103294.2[X.laevis]         Q--------------SHIREQEHLRKENADLKAESEQ--LSKELQHSLLQSKEAEQNCKN   1802
CBN80053.1[G.gallus]             ------------------KAMEQLQKENAELRTESER--LAKELQQSVLQAKEAELSCRE   1685
NP_001390473.1[M.musculus]       ------------------QDLEELQKENKELRSEAER--LGRELQQAGLKTKEAEQTCRH   1671
Q14980[H.sapiens]                ------------------RSLEQLQKENKELRAEAER--LGHELQQAGLKTKEAEQTCRH   1675
XP_002822235.1[P.abelii]         ------------------RSLEQLQKENKELRAEAER--LGHELQQAGLKTKEAEQTCRH   1675
                                                         . *            :  :           .

XP_032223093.1[N.vectensis]      LGMQVNSLEAQVKHADMTIREQKAKMQSFENQS-EKVKK-TR------------------   1399
XP_019634543.1[B.belcheri]       LYQQVRSLEAQVDLANRQLRQQKHQVSFATPAA-------------------RSLNTSTD   1833
XP_032819484.1[P.marinus]        LQANVSEIED--------------------------------------------------   1515
XP_045580741.1[S.salar]          LSSKVNTLEAQLAFTDRHLREQSQVRPERGPGGIEKMRGGRESVYLKVPQSQTHQETSGD   2064
XP_018103294.2[X.laevis]         LSNRVRSLEAQVEYSDRQLQDLGKFQLATDS-------TKRRDTFCVPRETRSHADVSID   1855
CBN80053.1[G.gallus]             LSGQVRSLETQLEFADQQLRELGKFPAPKAT-------LKEPESFRQN-----PSDLSTD   1733
NP_001390473.1[M.musculus]       LTAQVRSLEAQVAHADQQLRDLGKFQVATDA-------LKSREPQVKP-----QLDLSID   1719
Q14980[H.sapiens]                LTAQVRSLEAQVAHADQQLRDLGKFQVATDA-------LKSREPQAKP-----QLDLSID   1723
XP_002822235.1[P.abelii]         LTAQVRSLEAQVAHADQQLRDLGKFQVATDA-------LKSREPQAKP-----QLDLSID   1723
                                 *  .*  :*

XP_032223093.1[N.vectensis]      KMLIEVEKVLPVQEAQELLDESTTESDD-SEMR------------V----DDLKLTRQGS   1442
```

```
XP_019634543.1[B.belcheri]          SIEDTADETFDLEPRSRQRHS---TSRDDLDRTLSNSQVAGEEEGDKKNRTISTDSLEGD   1890
XP_032819484.1[P.marinus]           HFNTGVENTRILSKENLEQTP---SEDG----R-RHSMVT----------RSARKRSSRY   1557
XP_045580741.1[S.salar]             SLDLSLDDSLNTTTRPLGPDE---SS----------TPLVRSSERV----AAKRRALGGE   2107
XP_018103294.2[X.laevis]            SLDLSFEEDQLLNSTSKNGRC---NQEP----A-TSSVHASSLDSP----ASSQLPKKVE   1903
CBN80053.1[G.gallus]                SLDLSLDEGQPLNSTRKATRS---HSDG--------SAAPGGTEPR----ASQRLPRKVE   1778
NP_001390473.1[M.musculus]          SLDLSLEEGTPCSVASKLPRT---QPDG--------TSVPGEPASP----ISQRLPPKVE   1764
Q14980[H.sapiens]                   SLDLSCEEGTPLSITSKLPRT---QPDG--------TSVPGEPASP----ISQRLPPKVE   1768
XP_002822235.1[P.abelii]            SLDLSCEEGTPLSITSKLPRT---QPDG--------TSVPGEPASP----ISQRLPPKVE   1768
                                     :    :.

XP_032223093.1[N.vectensis]         ---SVSFASVNGTD------FGKSLSSTSSISRTSL------------------------   1469
XP_019634543.1[B.belcheri]          SLDTSIFS------------------------RTSQDSGMSTR----SQRRRTTVSIEMV   1922
XP_032819484.1[P.marinus]           SQETLYFTPLQTRPSLVGGQAPALDWSLNSLEDLVIDSAKKKPR-TSSTRRRTMHVIDIT   1616
XP_045580741.1[S.salar]             SLETLYFTPMNNRQINRTSTERRLESSITCLGELALDSARKRPPTSSARRRRTTQVINIT   2167
XP_018103294.2[X.laevis]            SLESLYFTPIPTRA------QSKLESSIGSIGDLSLDSSKKTR----SARRRTTQIINIT   1953
CBN80053.1[G.gallus]                SLESLYFTPIASRT------QPKLESSAGSLADVSLESGCRTR----SGRRRTTQIINIT   1828
NP_001390473.1[M.musculus]          SLESLYFTPTPARG------QAPLETSLDSLGDAFPDSGRKTR----SARRRTTQIINIT   1814
Q14980[H.sapiens]                   SLESLYFTPIPARS------QAPLESSLDSLGDVFLDSGRKTR----SARRRTTQIINIT   1818
XP_002822235.1[P.abelii]            SLESLYFTPIPARS------QAPLESSLDSLGDVFLDSGRKTR----SARRRTTQIINIT   1818
                                       :  *:

XP_032223093.1[N.vectensis]         --------------------------------------------RSTVRPSA--------   1477
XP_019634543.1[B.belcheri]          QT--EDSRRL--------------------------------------------------   1930
XP_032819484.1[P.marinus]           LNKKVESCRRDSVSSSASTMRSSTTTAYELRSHGDRPTPVQSSNWSLGFPASSASFVTLD   1676
XP_045580741.1[S.salar]             MSKTTPGRGGAGGDSD-------NEMFYSLSSVRSHPNITGSTH--TARPIS--------   2210
XP_018103294.2[X.laevis]            MTKKTKEERE--PESA-------NTSFYSLRSAPSYQNLHQQNPRKAARPQAAISTPALA   2004
CBN80053.1[G.gallus]                MTKKETTTEE--PGGA-------DASFSSILSEQPQKAAPARAR--------LRSARSLA   1871
NP_001390473.1[M.musculus]          MTKKLEL-EE--PDSA-------NSSFYSTQSAPAS-----QAN--------LRAT----   1847
Q14980[H.sapiens]                   MTKKLDV-EE--PDSA-------NSSFYSTRSAPAS-----QAS--------LRAT----   1851
XP_002822235.1[P.abelii]            MTKKLDV-EE--PDSA-------NSSFYSTRSAPAS-----QAS--------LRAT----   1851

XP_032223093.1[N.vectensis]         -------------------SRRQSAMYVKGSTPPTRRTTSSA------------AFFIVG   1506
XP_019634543.1[B.belcheri]          ------------------------------SRTSTEHVDLGSS-----IGSASSWRGAAP   1955
XP_032819484.1[P.marinus]           TFTSQNSLQLEDMR--SGRLSDASLRSLPGYRPLGPHGTDGRNLEQLQAPPTTRRRKGNA   1734
XP_045580741.1[S.salar]             -------MEVFHTPGKPAVAVSDQLLSLPGYRRSTVHV---------------AAPQSTG   2248
XP_018103294.2[X.laevis]            SLPSQESLAKTEHFSSDDSLNNSVLMNLPGYRAPARRSSRLSQ-----T-------GGRS   2052
CBN80053.1[G.gallus]                SFPSQDSLSKLDTSSPQEPSGHAALLSLPGYRPATRSSLRRSQ-----AGSSS--SLGRS   1924
NP_001390473.1[M.musculus]          --SSTQSLARLG----SPDDGNSALLSLPGYRPTTRSSARRSQ-----ARMSSGAPQGRN   1896
Q14980[H.sapiens]                   --SSTQSLARLG----SPDYGNSALLSLPGYRPTTRSSARRSQ-----AGVSSGAPPGRN   1900
XP_002822235.1[P.abelii]            --SSTQSLARLG----SPDYGNSALLSLPGYRPTTRSSARRSQ-----AGVSSGAPPGRN   1900

XP_032223093.1[N.vectensis]         DEFANNMEQESEYEFDWD------------------------------------------   1524
XP_019634543.1[B.belcheri]          RFFVSECEEEPEQ-YDWD------------------------------------------   1972
XP_032819484.1[P.marinus]           SYYM-SCVDEPDPEAEWDELERQGLGAHALPRRQPRAALGTSACYTEGLQENQPPVNCKV   1793
XP_045580741.1[S.salar]             QFCVGAENEPDHAADDWL------------------------------------------   2266
XP_018103294.2[X.laevis]            SFYMSTCQDEPDPQEDWT------------------------------------------   2070
CBN80053.1[G.gallus]                SIYLGTCQDEPEQLDDWN------------------------------------------   1942
NP_001390473.1[M.musculus]          SFYMGTCQDEPEQLDDWN------------------------------------------   1914
Q14980[H.sapiens]                   SFYMGTCQDEPEQLDDWN------------------------------------------   1918
XP_002822235.1[P.abelii]            SFYMGTCQDEPEQLDDWN------------------------------------------   1918
                                            :  .   :*

XP_032223093.1[N.vectensis]         --------------RLAELERRNTICPPHLKTSYPVETQTRPSEGNGLHAARSETALTRK   1570
XP_019634543.1[B.belcheri]          --------------RLSELQRRNTLQPRHLQSCYPTETQGTALEE--------------T   2004
XP_032819484.1[P.marinus]           DFSEKSDTRNDHLLRQEELGKRNQATLPHLRSCYPLKVETF------MPGEDLC-----T   1842
XP_045580741.1[S.salar]             --------------RIAELQARNQSCLPHLKSSYPLESRPSLGPSFEFTDDDLR-----M   2307
XP_018103294.2[X.laevis]            --------------RIAELQARNKTCPPHLKTSYPLESKPSLFSS-TLTDEEVK-----L   2110
CBN80053.1[G.gallus]                --------------RIAELQQRNRACPPHLKTCYPLESRPSDVLG-TITDEEMK-----T   1982
NP_001390473.1[M.musculus]          --------------RIAELQQRNRVCPPHLKTCYPLESRPTLSLA-TITDEEMK-----T   1954
Q14980[H.sapiens]                   --------------RIAELQQRNRVCPPHLKTCYPLESRPSLSLG-TITDEEMK-----T   1958
XP_002822235.1[P.abelii]            --------------RIAELQQRNRVCPPHLKTCYPLESRPSLSLG-TITDEEMK-----T   1958
                                                  *  **  **     **::.** : .

XP_032223093.1[N.vectensis]         RKPEDTLTSS------VKERKDMTTSKS-EGQIKR-------SRSKRITDMVSSK-----   1611
XP_019634543.1[B.belcheri]          EDLSKTLRRTTLRSSQAAKTFSITPGKKRRHEADENSA-----SRKKTVVMPSTPS----   2055
XP_032819484.1[P.marinus]           AKPEMTARRMFLRPALADANADM-------------------------------------   1865
XP_045580741.1[S.salar]             GDPTETIRRASVMPGQIQESLSSHRLSLHPGQADSTTASRPAYGSHRLSLMPPKPKASST   2367
XP_018103294.2[X.laevis]            GDPKETLRRATMLPSQIQDSTTSTRRLTLAGSGAEH------LKGHGISTRQQ-------   2157
CBN80053.1[G.gallus]                GDPTETLRRASMQPLQIAEGAATRRG-TLG----GG------WAHGGITTRQQ-------   2024
NP_001390473.1[M.musculus]          GDPRETLRRASMQPAQIAEGV--------------------------GITTRQQ-------   1982
Q14980[H.sapiens]                   GDPQETLRRASMQPIQIAEGT--------------------------GITTRQQ-------   1986
XP_002822235.1[P.abelii]            GDPQETLRRASMQPIQIAEGT--------------------------GITTRQQ-------   1986
                                     .   *

XP_032223093.1[N.vectensis]         -------------------INSLRPSKSNENIKEKQK--SGNSLAFEI----TPPKKR--   1644
XP_019634543.1[B.belcheri]          -------------------------K-TPSVFKTPKKIASAAAGIFKKPTGKTPSSRRRS   2089
XP_032819484.1[P.marinus]           -------------RKRKSGIGHNTEC-SPE----SKK---SATMCFVVPA--TPRERP--   1900
XP_045580741.1[S.salar]             LNNQNTHNLRGSNLSLKRSAKDQEPD-TPEVRMEAKR---MATSCFPRPL--TPKGGR--   2419
XP_018103294.2[X.laevis]            -------------MKRVSEESHYGPD-TPEG-----K---KTATCFPRPM--TPKDKH--   2191
CBN80053.1[G.gallus]                -------------RKRLSDESHQGPD-TPES-----K---KPVSCFPRPQ--TPRERR--   2058
NP_001390473.1[M.musculus]          -------------RKRVSSETHQGPG-TPES-----K---KATSCFPRPM--TPRDRH--   2016
Q14980[H.sapiens]                   -------------RKRVSLEPHQGPG-TPES-----K---KATSCFPRPM--TPRDRH--   2020
XP_002822235.1[P.abelii]            -------------RKRVSLEPHQGPG-TPES-----K---KATSCFPRPM--TPRDRH--   2020
                                                               : .      :        *      **

XP_032223093.1[N.vectensis]         ----------------KAEPKAASDTTPRRESTKKETVAFTIENSPPKKCKVQR--TRTI   1686
XP_019634543.1[B.belcheri]          PRLQGRGKSPAGRLNKAKTPTKTPNKHQAKQDPRRQSIAFNIGFSPLKGFRKSA--RKAK   2147
XP_032819484.1[P.marinus]           ----------PPRASPRRDDH----PQRSWPIIRR--------------------LRQSM   1926
XP_045580741.1[S.salar]             ----------FSSS---------NNRQPPSPAERRQSMVFSIDNTPRKAASKSGFLQRGM   2460
XP_018103294.2[X.laevis]            ----------DARRLSTMESK-ASSSHQQVQPTRRQTSAFSIFNTPKKLGS--SLLKRGL   2238
CBN80053.1[G.gallus]                ----------SSHLSRR-----SEQQAPSKQPERRQSMAFSILNTPKKLGN--SLLRRAA   2101
NP_001390473.1[M.musculus]          ----------EGRKQSSTADTQKKAAPVLKQADRRQSMAFSILNTPKKLGN--SLLRRGA   2064
Q14980[H.sapiens]                   ----------EGRKQSTTEAQKKAAPASTKQADRRQSMAFSILNTPKKLGN--SLLRRGA   2068
XP_002822235.1[P.abelii]            ----------EGRKQSTTEAQKKAAPASTKQADRRQSMAFSILNTPKKLGN--SLLRRGA   2068
                                                                     ::                      :

XP_032223093.1[N.vectensis]         NRSTATTQ-------LV---RDKDRETFKREKKLETKNRIPLRSSNRKIAK---------   1727
XP_019634543.1[B.belcheri]          EKREA--QKEPSQRRMSI----ANVKSF-LSV------NNSKT-NKTKAARKPLGTRNNA   2193
XP_032819484.1[P.marinus]           SRKKSPSSKASKP-----------------------------------------------   1939
XP_045580741.1[S.salar]             NKIRSSTRKSPAN-KISRVPRSGDVKSPQ--PGGKAQRKSPRT-NSSKSPKNPTSARKEP   2516
XP_018103294.2[X.laevis]            NKKTTPKN-SPRGRGTNSSASSTSSKSPHLSLRKSPSRRSPRV-STAKSPKASNKV-GQ-   2294
CBN80053.1[G.gallus]                NRKTTPKN-SP--RGTAR--------------------RSPRI-ASTKSPKGKAGRRALK   2137
NP_001390473.1[M.musculus]          SKKTPAKV-SPNPRSGTR--------------------RSPRI-ATTTTGTA---TVATT   2099
Q14980[H.sapiens]                   SKKALSKA-SPNTRSGTR--------------------RSPRI-ATTTASAATAAAIGAT   2106
XP_002822235.1[P.abelii]            SKKALSKA-SPNTRSGTR--------------------RSPRI-ATTTASAAT-AAIAAT   2105
                                    .:

XP_032223093.1[N.vectensis]         ------------------         1727
XP_019634543.1[B.belcheri]          QTGV--------------         2197
XP_032819484.1[P.marinus]           ------------------         1939
XP_045580741.1[S.salar]             EVLVGKPIHLSR------         2528
XP_018103294.2[X.laevis]            ---EEQIQFFERKQQRNK         2309
```

```
CBN80053.1[G.gallus]            D-----TKF---------         2141
NP_001390473.1[M.musculus]      PRAKGKVKH---------         2108
Q14980[H.sapiens]               PRAKGKAKH---------         2115
XP_002822235.1[P.abelii]        PRAKGKAKH---------         2114
```
