# Supplementary material for: An Evolutionary Perspective on the Origin, Conservation and Binding Partner Acquisition of Tankyrases
Source: Biomolecules. 2022 Nov 15;12(11):1688. doi: 10.3390/biom12111688 (PMC9688111; doi:10.3390/biom12111688)
Supplement: Supplementary file 1 [file biomolecules-12-01688-s001.zip › biomolecules-1913719-supplementary-11.17 -done/Data_7/PEX14.pdf]

PEX14

Homo sapiens:

>O75381 PEX14 [Homo sapiens]
MASSEQAEQPSQPSSTPGSENVLPREPLIATAVKFLQNSRVRQSPLATRRAFLKKKGLTD
EEIDMAFQQSGTAADEPSSLGPATQVVPVQPPHLISQPYSPAGSRWRDYGALAIIMAGIA
FGFHQLYKKYLLPLILGGREDRKQLERMEAGLSELSGSVAQTVTQLQTTLASVQELLIQQ
QQKIQELAHELAAAKATTSTNWILESQNINELKSEINSLKGLLLNRRQFPPSPSAPKIPS
WQIPVKSPSPSSPAAVNHHSSSDISPVSNESTSSSPGKEGHSPEGSTVTYHLLGPQEEGE
GVVDVKGQVRMEVQGEEEKREDKEDEEDEEDDDVSHVDEEDCLGVQREDRRGGDGQINEQ
VEKLRRPEGASNESERD

Pongo abelii:

>XP_024109019.1 peroxisomal membrane protein PEX14 isoform X1 [Pongo abelii]
MASSEQAEQPSQPSSTPGSENVLPREPLIATAVKFLQNSRVRQSPLATRRAFLKKKGLTDEEIDMAFQQS
GTAADEPSSLGPATQVVPVQPPHLVSQPYSPAGSRWRDYGALAIIMAGIAFGFHQLYKKYLLPLILGGRE
DRKQLERMEAGLSELSGSVAQTVTQLQTTLASVQELLIQQQQKIQELAHELAAAKATTSTNWILESQNIN
ELKSEINSLKGLLLNRRQFPPSPSAPKIPSWQIPVKSPSPSSPAAVNHHSSSDISPVSNESTSSSPGKEG
HSPEGSTVTYHLLGPQEEGEGVVDVKGQVRMEVQGEEEKREDKEDEEDEEDDDVSHVDEEDCLGVQREDR
RGGDGQINEQVEKLRRPEGASNESERD

Mus musculus:

>NP_062755.1 peroxisomal membrane protein PEX14 [Mus musculus]
MASSEQAEQPNQPSSPPGSENVVPREPLIATAVKFLQNSRVRQSPLATRRAFLKKKGLTDEEIDLAFQQS
GTAADEPSPLGPATPVVPVQPPHLTPQPYSPRGSRWRDYGALAIIMAGIAFGFHQLYKRYLLPLILGGRE
DRKQLERMAASLSELSGTVAQTVTQVQTTLASVQELLRQQQQKVQELAHELATAKATTSTNWILESQNIN
ELKSEINSLKGLLLNRRQFPPSPSAPKIPSWQIPVKSSSPSSPAAVNHHSSSDISPVSNESTSSSPGKDS
HSPEGSTATYHLLGPQEEGEGVLDVKGQVRMEVQGEEEKREDKEDEDDEDDDVSHVDEEDVLGVQREDRR
GGDGQINEQVEKLRRPEGASNETERD

Gallus gallus:

>XP_001234697.3 peroxisomal membrane protein PEX14 [Gallus gallus]
MASSEQAEQPSQAGSSPATENAASREPLIVTAVKFLQNPRVRQSPIATRRAFLKKKGLTDEEIDLAFQQS
GTSADEPQSPGPSSQLVPAQPAHPVVYNPPGSRWRDYGALAIIMAGIAFGFHQLYKKYLLPLIMGGKEDR
KQLQRIESNIAEMSGSVTQTVTQLQTTLAAVQEMLIQQQQKIQELTQELAASKATTSTNWILESQNINEL
KSEIYSLKGLLLNRRQFPPSPSAPKIPSWQIPVKPSSPSNPVVANHNSSSDISPVSNESTTSSPVKENHS
PEGSKVSCHLLSTEEGNKAVIDVKSQVRMEVQGEEEKRENKRNEEEEEDDEDDDVSHVDEEECMGVQTED
RRGGDGQINEQVEKLRRPEGASNENEID

Xenopus laevis:

>XP_018081136.1 PREDICTED: peroxisomal membrane protein PEX14-like [Xenopus laevis]
MASSDQTDQSVQGGPSLINEKIVPRDQLIATAVKFLQNPRVRQSPVATRKEFLKKKGLSNEEIELALQQS
GTVHDDPGLVTHTGIPHTGPSSQLAVQQFSPPGSRWREYGALAIILAGIAFGFHQLYKRYLLPLIIGGRE
SRKQLQRIESGVSEMSGSVTQTVTQLQTTLAAVQELLIQQQQKIQELSLELSASKASSSTNTILESQNIQ
ELKSEIYSLKGLLLNRRQFPPSPSASKIPAWQIPVKPPTLPSPAVLNHNSSSDISPVSNESGSSSPVKEI
HSPEGTKGSSHHLGLTEEESEIDVQTQVRMEVQGEEENEDDVVQPDEDCIGVQTEDRRGGDGQINEQVDK
LRRPEGASNEMDC

Salmo salar:

>XP_014001298.1 PREDICTED: peroxisomal membrane protein PEX14 [Salmo salar]
MASSEQPDPQTQPRAPSRDDVAPREAMIATAVKFLQNHKVRQSPLETRKVFLKKKGLTDKEIELAIQRSG
STDEPLALTPVGPQYPLLDPQLAPIPYSPRGYRWRDYGALTVIMMGMAFGFHHLYKKYILPLIMGSKEDK
KHLQRIESNIAEMSGTLTQTVTQLQQTLATVQDLLVQQQQKIHDLSQELSTSEASSSTNRILDTQTIGDL
KAEIGSLKGLLLGRRQFPASPSVPKIPSWQMPLKPDTISTSSPSINHTNSSSDISPVSNESASSSPIKDG
HHSPQDALGGPDGAHGLNGKNGVGLGGGATLPPLDLKDQVRMEVQGEEEKKEEDVKEEKEEEEEEEDDVS
HVDEEEHLSVPAEDRRGGDGQINEQVDKLRRPEGASNESEVD

Petromyzon marinus:

>XP_032819505.1 peroxisomal membrane protein PEX14-like isoform X3 [Petromyzon marinus]
MADTEREVDAVIGNASREHLVDTAFKFLKNPRVAGSPPNIQRAFLRKKGLTDVEIEDVFLRAGVHRGTSL
AHAQHLAQSPAGPPASRHSWRWRDVGALSAVIAAIGFGINRLYKWLAAVRAAEAQRLTDVESTVSAMAAS
LTHAVTQLQTSVAGTHELLSRQQKQLERLTDDTAVSKASGPLLADLRTEIASIKGLMLGRKQFPCPPNSA
AILPSWQLALKPSTSTGTTISSTMTSQDVNAATNKEQQLQPRENTDTLTGSSENVSSSDISPISHESASP
GLDSEVHTLKAEGKVNSLQSLTQNGEVGDGFIHQHQDEDEDVITEVCVLTKVEAQGENEVD

Branchiostoma belcheri:

>XP_019628140.1 PREDICTED: LOW QUALITY PROTEIN: peroxisomal membrane protein PEX14-like [Branchiostoma belcheri]
MAAPDPKSSPKDEGSPSDVPLNPEPRQQMIETAVKFLQNPRVRQSAFAQRRAFLQKKGLTNEEIQLAIEK
SGTASDETNPPPPPPAQVAQSTAPVPLQPPVIVPVSPWSKWRDMGAVIVLVGGALYGLYRLFQAYLVPLW
KTRQEERQRLERIEAAVTELSASVNKTVGELQTSMGVVQQALTKQSDRVQEISMELAAGKSTARVFESQS
VSELKAEITSLKGLLLSRXVTKYLTDHYTFPPTPSPTPIIPSWQMEPVDSASSSSSKPATTSSEVSPAEQ
PLPADSNHAAETLDVAQNNGPSAQKTVSTSESSEDTSSSIVHVSNPLDDLSSGEEMEAKGQMEVKGQMEV
KGEEVTAEVNHQGEEDNREETVIKEA

Drosophila melanogaster:

>NP_649253.1 peroxin 14 [Drosophila melanogaster]
MSSNNTDTGDTTVMATATSVQNDVEAGVDEQLPRESLITTAVSFLQNTKVRHTTLIQKQQFLRSKGLTAH
EIQLACERAGVFTQDPNKPNPNPNTVISIGSQLHALQPQPTVLGRIREIIHSAALFSGVVYAVYIFWKQY
IAPYLFGKSKKKAVDEVLDDIDKKVETRTNDLNKEILAVRDLITTQQREHAQQLNREFSNFRSDLDAIKG
LLLNRKQFAGPVAPIAVPSIPAWQLAGSPHHHHRHSGSDDNEKGDDAGSGSGSSETEVVTKNSDSSLEIM

Hydra vulgaris:

>XP_004208685.1 PREDICTED: peroxisomal membrane protein PEX14-like [Hydra vulgaris]
MKVDSNEDVDSKKVDVAVRFLSNPNVKSTNKESKEAFLKSKGLNDMEILNAMQKASSECEIAESNKWSIF
TYFKGIVIGAGILSAANYAYKAYILPYVAHEIKDDGRIERLGESVQAMKDDIKQHTYELSSTLKDIQTVL
EKQHKVIVLLQESIQNSPFDHTTLADIKAELSNVKTMMLSRKQFPKAPFTQQSNEIPAWQRVDNKKVTDV

STDDTSSSETI

Nematostella vectensis:

>XP_032228851.1 peroxisomal membrane protein PEX14-like [Nematostella vectensis]
MADPKEANMEEATNADSKQQKIATAVKFLQNPRVRSTPMSQRRAFLEKKGLTKEEIDLAVEQSGTAGDVV
SVQAPSAITPYQTQVQRVAPLPYTWRGYLGAAVIGGSLGYIIVTVFKKFILPLITHKKQKDERLRAIENS
VGELSNSVMDTVGGVQTTLDSVQKLLIEQQKQIICLSADLSSTQEKVEQLPTESYSVNDLKSEIISLKGL
LLNRKAFPSPLDNSTPAGIPSWQRAVTAGADSRTTDSASTSFAASKRNKDGGQITESGTYNSSRKKASYE
SEHKSMNGEISSEKKKKTPEKKETPPEVPQEMEHSHHSNESSDTDTPNTVKSAITNENLDDAVLVDSQDI
SLQSNDTSASSLSDMSANASFNEESIAFSSTCIKVPAEASKSEELPSASAIEVD

Amphimedon queenslandica:

>XP_003385343.1 PREDICTED: peroxisomal membrane protein PEX14-like [Amphimedon queenslandica]
MAAMSSESTSAASSPEVNGASTDPIKGSFVPRDSSYSEFMRRLLPERQKLLDIAVKFLINPRVVNSNDDD
KIAFLNKKGLNEEEIYWVRQEAASRPAPSLAVPAPPTSLALTTPTQATPTLTHLVIRGVLVGSMFGIFIY
WIKGVVSKWFDSLKVQQERHQVLEERVQEIRTTLETTMQELQQTTLSIKVLLERQQEQITQLSSTQSNVV
ASNELRTINSELSTLKGLLLNRHQFPTPQKDNLSLPPGIPSWQRNTSLPPLNTTNGSSKEQEEASGDGGG
GGGESSTGTTVQQNSNNDNNDEEDDD

Alignment

```
NP_649253.1[D.melanogaster]          MSSNNTD----------TG-D-----TTVMATATSVQNDVEAGVDEQLPRESLITTAVSF           44
XP_003385343.1[A.queenslandica]      MAAMSSESTSAASSPEVNGASTDPIKGSFVPRDSS---YSEFMRRLLPERQKLLDIAVKF           57
XP_032819505.1[P.marinus]            MADT-----ER---------E-------------------VDAVIGNASREHLVDTAFKF           27
XP_004208685.1[H.vulgaris]           ----------------------------------------MKVDSNEDVDSKKVDVAVRF           20
XP_032228851.1[N.vectensis]          MADP-----KEA--------N-------------------MEEATNADSKQQKIATAVKF           28
XP_019628140.1[B.belcheri]           MAAP-----DPKSSPKDEGSP-------------------SDVPLNPEPRQQMIETAVKF           36
XP_014001298.1[S.salar]              MASS-----EQP-DPQTQP-R-------------------APSRDDVAPREAMIATAVKF           34
XP_018081136.1[X.laevis]             MASS-----DQT-DQSVQGGP-------------------SLINEKIVPRDQLIATAVKF           35
XP_001234697.3[G.gallus]             MASS-----EQA-EQPSQAGS-------------------SPATENAASREPLIVTAVKF           35
O75381[H.sapiens]                    MASS-----EQA-EQPSQPSS-------------------TPGSENVLPREPLIATAVKF           35
XP_024109019.1[P.abelii]             MASS-----EQA-EQPSQPSS-------------------TPGSENVLPREPLIATAVKF           35
NP_062755.1[M.musculus]              MASS-----EQA-EQPNQPSS-------------------PPGSENVVPREPLIATAVKF           35
                                                                                  .  :  *. *

NP_649253.1[D.melanogaster]          LQNTKVRHTTLIQKQQFLRSKGLTAHEIQLACERAGVFTQDPNKPNPNPNTVISIGSQLH           104
XP_003385343.1[A.queenslandica]      LINPRVVNSNDDDKIAFLNKKGLNEEEIYWVRQEAASRPAPSLAVPAPPTSLALTT-PTQ           116
XP_032819505.1[P.marinus]            LKNPRVAGSPPNIQRAFLRKKGLTDVEIEDVFLRAGVHRGTSLAHAQH-------LAQSP           80
XP_004208685.1[H.vulgaris]           LSNPNVKSTNKESKEAFLKSKGLNDMEILNAMQKASSECEIA------------------           62
XP_032228851.1[N.vectensis]          LQNPRVRSTPMSQRRAFLEKKGLTKEEIDLAVEQSGTAGDVVSVQAPS-----------A           77
XP_019628140.1[B.belcheri]           LQNPRVRQSAFAQRRAFLQKKGLTNEEIQLAIEKSGTASDETNPPPPPPAQVAQSTAPVP           96
XP_014001298.1[S.salar]              LQNHKVRQSPLETRKVFLKKKGLTDKEIELAIQRSGST-DEPLALTPV-------GPQYP           86
XP_018081136.1[X.laevis]             LQNPRVRQSPVATRKEFLKKKGLSNEEIELALQQSGTVHDDPGLVTHT-------GIPHT           88
XP_001234697.3[G.gallus]             LQNPRVRQSPIATRRAFLKKKGLTDEEIDLAFQQSGTSADEPQSPGPS-------SQLVP           88
O75381[H.sapiens]                    LQNSRVRQSPLATRRAFLKKKGLTDEEIDMAFQQSGTAADEPSSLGPA-------TQVVP           88
XP_024109019.1[P.abelii]             LQNSRVRQSPLATRRAFLKKKGLTDEEIDMAFQQSGTAADEPSSLGPA-------TQVVP           88
NP_062755.1[M.musculus]              LQNSRVRQSPLATRRAFLKKKGLTDEEIDLAFQQSGTAADEPSPLGPA-------TPVVP           88
                                     * * .*  :    :  **..***.  **  .  .:.

NP_649253.1[D.melanogaster]          ALQ------PQPTVLGRIREIIHSAALFSGVVYAVYIFWKQYIAPYLF-GKSKK----KA           153
XP_003385343.1[A.queenslandica]      ATP-------------TLTH-LVIRGVLVGSMFGIFIYWIKGVVSKWFDSLKVQQERHQV           162
XP_032819505.1[P.marinus]            AGP------PASRHSWRWRDVGALSAVIAAIGFGINRLYKWLA----A-VRAAEAQRLTD           129
XP_004208685.1[H.vulgaris]           ----------ESNKWSIF-TYFKGIVIGAGILSAANYAYKAYILPYVA-HEIKDDGRIER           110
XP_032228851.1[N.vectensis]          ITPYQTQVQRVAPLPYTWRGYLGAAVIGGSLGYIIVTVFKKFILPLIT-HKKQKDERLRA           136
XP_019628140.1[B.belcheri]           LQPPVIV---PVSPWSKWRDMGAVIVLVGGALYGLYRLFQAYLVPLWK-TRQEERQRLER           152
XP_014001298.1[S.salar]              LLDPQLAPIPYSPRGYRWRDYGALTVIMMGMAFGFHHLYKKYILPLIM-GSKEDKKHLQR           145
XP_018081136.1[X.laevis]             GPSSQLAVQQFSPPGSRWREYGALAIILAGIAFGFHQLYKRYLLPLII-GGRESRKQLQR           147
XP_001234697.3[G.gallus]             AQPAHPV--VYNPPGSRWRDYGALAIIMAGIAFGFHQLYKKYLLPLIM-GGKEDRKQLQR           145
O75381[H.sapiens]                    VQPPHLISQPYSPAGSRWRDYGALAIIMAGIAFGFHQLYKKYLLPLIL-GGREDRKQLER           147
XP_024109019.1[P.abelii]             VQPPHLVSQPYSPAGSRWRDYGALAIIMAGIAFGFHQLYKKYLLPLIL-GGREDRKQLER           147
NP_062755.1[M.musculus]              VQPPHLTPQPYSPRGSRWRDYGALAIIMAGIAFGFHQLYKRYLLPLIL-GGREDRKQLER           147
                                                               :  .        :              .

NP_649253.1[D.melanogaster]          VDEVLDDIDKKVETRTNDLNKEILAVRDLITTQQREHAQQL------------------N           195
XP_003385343.1[A.queenslandica]      LEERVQEIRTTLETTMQELQQTTLSIKVLLERQQEQI-TQLSSTQ---------SNVVAS           212
XP_032819505.1[P.marinus]            VESTVSAMAASLTHAVTQLQTSVAGTHELLSRQQKQL-ERLTDDTAVSKAS--------G           180
XP_004208685.1[H.vulgaris]           LGESVQAMKDDIKQHTYELSSTLKDIQTVLEKQHKVI-VLLQES--------IQNSPFDH           161
XP_032228851.1[N.vectensis]          IENSVGELSNSVMDTVGGVQTTLDSVQKLLIEQQKQI-ICLSADLSSTQEK-VEQLPTES           194
XP_019628140.1[B.belcheri]           IEAAVTELSASVNKTVGELQTSMGVVQQALTKQSDRV-QEISMELAAGK---STARVFES           208
XP_014001298.1[S.salar]              IESNIAEMSGTLTQTVTQLQQTLATVQDLLVQQQQKI-HDLSQELSTSEASSSTNRILDT           204
XP_018081136.1[X.laevis]             IESGVSEMSGSVTQTVTQLQTTLAAVQELLIQQQQKI-QELSLELSASKASSSTNTILES           206
XP_001234697.3[G.gallus]             IESNIAEMSGSVTQTVTQLQTTLAAVQEMLIQQQQKI-QELTQELAASKATTSTNWILES           204
O75381[H.sapiens]                    MEAGLSELSGSVAQTVTQLQTTLASVQELLIQQQQKI-QELAHELAAAKATTSTNWILES           206
XP_024109019.1[P.abelii]             MEAGLSELSGSVAQTVTQLQTTLASVQELLIQQQQKI-QELAHELAAAKATTSTNWILES           206
NP_062755.1[M.musculus]              MAASLSELSGTVAQTVTQVQTTLASVQELLRQQQQKV-QELAHELATAKATTSTNWILES           206
                                     :   :  :   :      :.      :  :  *       :

NP_649253.1[D.melanogaster]          REFSNFRSDLDAIKGLLLNRK---------QFAGPVAP--IAVPSIPAWQLAGSPHHHHR           244
XP_003385343.1[A.queenslandica]      NELRTINSELSTLKGLLLNRH---------QFPTPQKDNLSLPPGIPSWQRNTSLPPLNT           263
XP_032819505.1[P.marinus]            PLLADLRTEIASIKGLMLGRK---------QFPC--PP--NSAAILPSWQLALKPSTSTG           227
XP_004208685.1[H.vulgaris]           TTLADIKAELSNVKTMMLSRK---------QFPKAPFT--QQSNEIPAWQRVDNKKVTDV           210
XP_032228851.1[N.vectensis]          YSVNDLKSEIISLKGLLLNRK---------AFPSPLDN--STPAGIPSWQRAVTAGADSR           243
XP_019628140.1[B.belcheri]           QSVSELKAEITSLKGLLLSRXVTKYLTDHYTFPP--TP--SPTPIIPSWQMEPVDSASSS           264
XP_014001298.1[S.salar]              QTIGDLKAEIGSLKGLLLGRR---------QFPA--SP--S-VPKIPSWQMPLKPDTI-S           249
XP_018081136.1[X.laevis]             QNIQELKSEIYSLKGLLLNRR---------QFPP--SP--S-ASKIPAWQIPVKPPTL--           250
XP_001234697.3[G.gallus]             QNINELKSEIYSLKGLLLNRR---------QFPP--SP--S-APKIPSWQIPVKPSSP--           248
O75381[H.sapiens]                    QNINELKSEINSLKGLLLNRR---------QFPP--SP--S-APKIPSWQIPVKSPSP--           250
XP_024109019.1[P.abelii]             QNINELKSEINSLKGLLLNRR---------QFPP--SP--S-APKIPSWQIPVKSPSP--           250
NP_062755.1[M.musculus]              QNINELKSEINSLKGLLLNRR---------QFPP--SP--S-APKIPSWQIPVKSSSP--           250
                                       .  :.:::  :* ::*.*           *             :*:**

NP_649253.1[D.melanogaster]          HSGSDDNEKG-------------------------------------------DDAGSGS           261
XP_003385343.1[A.queenslandica]      TNGSSKE----------------------------------------QEEASGDGGGGGG           283
XP_032819505.1[P.marinus]            TTISSTMTSQDVNAATNKEQQLQPRENTDTLTGSSENVSSSDISPISHESASPGLDSEVH           287
XP_004208685.1[H.vulgaris]           STDDTS--------------------------------SSETI-----------------           221
XP_032228851.1[N.vectensis]          TTDSAST----------------------------SFAAS-------------KRNKDGG           262
XP_019628140.1[B.belcheri]           SSKPAT--------------------------------TSSEVSPAEQPL----PADSN-           287
XP_014001298.1[S.salar]              TSSPSIN----------------------------HTNSSSDISPVSNESASSSPIKDG-           280
XP_018081136.1[X.laevis]             -PSPAVL----------------------------NHNSSSDISPVSNESGSSSPVKEI-           280
XP_001234697.3[G.gallus]             -SNPVVA----------------------------NHNSSSDISPVSNESTTSSPVKEN-           278
O75381[H.sapiens]                    -SSPAAV----------------------------NHHSSSDISPVSNESTSSSPGKEG-           280
XP_024109019.1[P.abelii]             -SSPAAV----------------------------NHHSSSDISPVSNESTSSSPGKEG-           280
NP_062755.1[M.musculus]              -SSPAAV----------------------------NHHSSSDISPVSNESTSSSPGKDS-           280


NP_649253.1[D.melanogaster]          GSSETEVVTKNSDS-SL--------------EIM--------------------------           280
XP_003385343.1[A.queenslandica]      ESSTGTTVQQNSNN----------------------------------------------           297
XP_032819505.1[P.marinus]            TLKAEG-----------------------------KVNSLQSLTQNGEVGD---------           309
XP_004208685.1[H.vulgaris]           ------------------------------------------------------------           221
XP_032228851.1[N.vectensis]          QITESG---TYNSS-RK-----------------KASYESEHKSMNGEISSEKKKKTPEK           301
XP_019628140.1[B.belcheri]           HAAETLDVAQNNGPSAQKTVSTSESSEDTSSSIVHVSNPLDDLSSGEEMEA--KGQMEVK           345
XP_014001298.1[S.salar]              HHSPQDALG---GPDGA-----------------HGLNGKNGVGLGG-GAT--LPPLDLK           317
XP_018081136.1[X.laevis]             HSPEGT----------------------------KGSSHHLGLTEE-------ESEIDVQ           305
XP_001234697.3[G.gallus]             HSPEGS----------------------------KVSCHLLSTEEG----N--KAVIDVK           304
O75381[H.sapiens]                    HSPEGS----------------------------TVTYHLLGPQEE----G--EGVVDVK           306
XP_024109019.1[P.abelii]             HSPEGS----------------------------TVTYHLLGPQEE----G--EGVVDVK           306
NP_062755.1[M.musculus]              HSPEGS----------------------------TATYHLLGPQEE----G--EGVLDVK           306


NP_649253.1[D.melanogaster]          ------------------------------------------------------------           280
XP_003385343.1[A.queenslandica]      ------------------DN----------------NDEEDDD-----------------           306
XP_032819505.1[P.marinus]            ---------GFI---HQHQD-------------------EDEDVI---------------           323
XP_004208685.1[H.vulgaris]           ------------------------------------------------------------           221
XP_032228851.1[N.vectensis]          KETPPEVPQEMEH--SHHSNESSDTDTPNTVKSAITNENLDDAVLVDSQDISLQSNDTSA           359
XP_019628140.1[B.belcheri]           G--QMEVKGEEVTAEVNHQG---E------------EDNREETVI---------------           373
XP_014001298.1[S.salar]              DQVRMEVQGEEEKKEEDVKEEKEE------------EEEEEDDVS---------------           350
XP_018081136.1[X.laevis]             TQVRMEVQGEEENEDD----------------------------V---------------           322
XP_001234697.3[G.gallus]             SQVRMEVQGEEEKRENK-RNEEEE------------EDDEDDDVS---------------           336
O75381[H.sapiens]                    GQVRMEVQGEEEKREDK-ED---E------------EDEEDDDVS---------------           335
XP_024109019.1[P.abelii]             GQVRMEVQGEEEKREDK-ED---E------------EDEEDDDVS---------------           335
NP_062755.1[M.musculus]              GQVRMEVQGEEEKREDK-ED---E------------D-DEDDDVS---------------           334
```

```
NP_649253.1[D.melanogaster]          ---------------------------------------------------------- 280
XP_003385343.1[A.queenslandica]      ---------------------------------------------------------- 306
XP_032819505.1[P.marinus]            -------------------TEVCVLTKVEAQGENEVD--------------------- 341
XP_004208685.1[H.vulgaris]           ---------------------------------------------------------- 221
XP_032228851.1[N.vectensis]          SSLSDMSANASFNEESIAFSSTCIKVPAEASKSEELPSAS-------------AIEVD 404
XP_019628140.1[B.belcheri]           ----------------K---EA------------------------------------ 376
XP_014001298.1[S.salar]              ----------------HVDEEEHLSVPAEDRRGGDGQINEQVDKLRRPEGASNESEVD 392
XP_018081136.1[X.laevis]             ----------------VQPDEDCIGVQTEDRRGGDGQINEQVDKLRRPEGASNEMDC- 363
XP_001234697.3[G.gallus]             ----------------HVDEEECMGVQTEDRRGGDGQINEQVEKLRRPEGASNENEID 378
O75381[H.sapiens]                    ----------------HVDEEDCLGVQREDRRGGDGQINEQVEKLRRPEGASNESERD 377
XP_024109019.1[P.abelii]             ----------------HVDEEDCLGVQREDRRGGDGQINEQVEKLRRPEGASNESERD 377
NP_062755.1[M.musculus]              ----------------HVDEEDVLGVQREDRRGGDGQINEQVEKLRRPEGASNETERD 376
```
