# Supplementary material for: An Evolutionary Perspective on the Origin, Conservation and Binding Partner Acquisition of Tankyrases
Source: Biomolecules. 2022 Nov 15;12(11):1688. doi: 10.3390/biom12111688 (PMC9688111; doi:10.3390/biom12111688)
Supplement: Supplementary file 1 [file biomolecules-12-01688-s001.zip › biomolecules-1913719-supplementary-11.17 -done/Data_7/RNF146.pdf]

RNF146

Homo sapiens:

>sp|Q9NTX7|RN146_HUMAN E3 ubiquitin-protein ligase RNF146 OS=Homo sapiens OX=9606 GN=RNF146 PE=1 SV=1
MMAGCGEIDHSINMLPTNRKANESCSNTAPSLTVPECAICLQTCVHPVSLPCKHVFCYLC
VKGASWLGKRCALCRQEIPEDFLDKPTLLSPEELKAASRGNGEYAWYYEGRNGWWQYDER
TSRELEDAFSKGKKNTEMLIAGFLYVADLENMVQYRRNEHGRRRKIKRDIIDIPKKGVAG
LRLDCDANTVNLARESSADGADSVSAQSGASVQPLVSSVRPLTSVDGQLTSPATPSPDAS
TSLEDSFAHLQLSGDNTAERSHRGEGEEDHESPSSGRVPAPDTSIEETESDASSDSEDVS
AVVAQHSLTQQRLLVSNANQTVPDRSDRSGTDRSVAGGGTVSVSVRSRRPDGQCTVTEV

Pongo abelii:

>NP_001128751.1 E3 ubiquitin-protein ligase RNF146 [Pongo abelii]
MAGCGEIDHSINMLPTNRKANESCSNTAPSLTVPECAICLQTCVHPVSLPCKHVFCYLCVKGASWLGKRC
ALCRQEIPEDFLDKPTLLSPEELKAASRGNGEYAWYYEGRNGWWQYGERTSRELEDAFSKGKKNTEMLIA
GFLYVADLENMVQYRRNEHGRRRKIKRDIIDIPKKGVAGLRLDCDANTVNLARESSADGADSVSAQSGAS
VQPLVSSVRPLTSVDGQLTSPATPSPDASTSLEDSFAHLQLSGDNTAERSHRGEGEEDHESPSSGRVPAP
DTSIEETESDASSDSEDVSAVVAQHSLTQQRLLVSNANQTVPDRSDRSGTDRSVAGGGTVSVSVRSRRPD
GQCTVTEV

Mus musculus:

>BAE31549.1 unnamed protein product [Mus musculus]
MEMAGCGEIDHSINMLPTNKKANESCSNTAPSLTVPECAICLQTCVHPVSLPCKHVFCYLCVKGASWLGK
RCALCRQEIPEDFLDKPTLLSPEELKAASRGNGEYAWYYEGRNGWWQYDERTSRELEDAFSKGKKNTEML
IAGFLYVADLENMVQYRRNEHGRRRKIKRDIIDIPKKGVAGLRLDCDTNTVNLARESSADGADSGSAQTG
ASVQLAVPSSTRPLTSVDGQLTSPVTPSPDAGISLEDSFAHLQLSGDSIAERSHRGEGEEDHESPSSGRV
PDTSVEETESDASSDSEDAPVVVAQHSLTQQRPLVPNGNQTVADQSDRSGTDRSVAGGRTMSVNVRSRRP
DGQCTVTEV

Gallus gallus:

>XP_015139929.3 E3 ubiquitin-protein ligase RNF146 [Gallus gallus]
MAGCGEIDHSINMLPTNRKTNESCANAAPSLTVPECAICLQTCVHPVSLPCKHVFCYLCVKGASWLGKRC
ALCRQEIPEDFLDKPTLLSPEELKAASRGNGEYAWYYEGRNGWWQYDERTSRELEDAFSKGKKSTEMLIA
GFLYIADLENMVQYRRNEHGRRRKIKRDIIDIPKKGVAGLRLDCDTNSVNLARESSADGADSTLTGGAAA
VQPLVPVSARPLPSLDGQLMSPSTPSPDASTSLENSFAHLQINGDSMAERSHRGEGEEDHESSSSGRVPA
PDTSVEETESDASSDSEDVSAPLQQHPSSAQQRHLNANANQSGADRPVAGGGVANASVRSRRPDGQCTVT
EV

Xenopus laevis:

>XP_018118669.1 E3 ubiquitin-protein ligase rnf146 [Xenopus laevis]
MAGCGEVSHSANMLPTNKKLGEPCSNSAPSSSVPECAICLQICVHPVSLPCKHIFCYLCVKGASWLGRRC
ALCRQEIPEDFLDKPTLLSPEELKSASRGNGEYAWYYEGRNGWWQYDERTSRELEDAFTKGKKSTEMLIA
GFLYVADLENMVQYRRNEHGRRRKIKRDIVDIPKKGVAGLRLECDPANLNLARESSADGADNMAALGASS
SQPAPVLPARLHTSLSTTASQVLSHSDVTNSLENSFAELQIGDPVIGRNNIGEGEEGQPLINPRMPPPNA
VLEEPEPSDSNDHGSPALQHHSFLGNP

Salmo salar:

>NP_001167373.1 E3 ubiquitin-protein ligase rnf146 [Salmo salar]
MASCGEVDHSVSSLPSSKKGSGDSACSGSSGSSPALPVPECAICLQSCVHPVQLPCRHVFCFLCVKGASW
QSKRCALCRQEVPEDFLEHPTLLSPEELKTGGRGATGDNAWYYEGRNGWWQYDERTSRELEDAFSKGKKT
AEMLIAGFLYVADLENMVQYRRNEHGRRRKIKRDVVDIPKKGVAGLRLDTEGGVQGSAAAGRGNSADGAD
TSAAAVQQAAAAPAATTVLSAPARPPTSLGGQPGSPTSPSLEDTLALLHISPTDAPERAEVGEGEEEATA
TPSMSSSPNTYADGSGDWSDDEGDGEAVEPREQRLRLGESLVDRSPPGAEASSSSSVRSRRPDGQCTEV

Petromyzon marinus:

>tr|S4R4W7|S4R4W7_PETMA E3 ubiquitin-protein ligase OS=Petromyzon marinus OX=7757 PE=4 SV=1
VPDCAICLQPCVHPARLPCRHVFCFLCAKGVALQSRRCALCRSEIPPDFLERPTLLCSTS
DGVPLKPRTANAWYYEGRNGWWQYDERTSAELEEAFARGSTTMEMLIAGYLYVADLEAMV
QYRRSEPSRRRKMKRDLLSAPKKGVAGLRLDLTAPAVSPAHASGVDGVDRAVAGTSSGSG
SVVAGGLLASAGPSTITVASPSSPVSSVATSSTLSVDPLPDSGATSSFPSRPATFAPQHQ
MRAVVGPPSPADSDDSSDRDDEEQDYEDNEGAASISRQLAGMRLGRSGAVIDTAEAQQQS
AAPRSATLHVRSRDGRPDGQYTVTDV

Branchiostoma belcheri:

>XP_019623976.1 PREDICTED: E3 ubiquitin-protein ligase rnf146-like [Branchiostoma belcheri]
MAESRSVASAETSLRQSSAKKRREKEEMAETATGQKPGGEDMIPECAVCLQTCVQPVRLPCTHIFCYLCV
KGVANQSKRCALCRQEIPIEYLERPELVKPIKVTEEQEGEEEEEVYCWFYEGRNGWWQYDERTNAELEDA
YTKKKKSCELLIAGFLYIIDFVNMLQFRRNDPSRRRRIKRDLTNTPKKGVAGVRLPAPAPPRNAPADGVE
GANEGATGGSRSVAVTSRATKSTRGPADHDEDDSNEDTNNRAEEDSTSEDVTDQSARGNARQSPDSLRQH
TPTQEEDDDGDTNSTGGAEMSEMVRMLQRSRLGRGSDSSRSGVLQAVLQGQVSSVANVAMAREEIPGDSS
DESD

Drosophila melanogaster:

>tr|B7Z078|B7Z078_DROME E3 ubiquitin-protein ligase OS=Drosophila melanogaster OX=7227 GN=Rnf146 PE=2 SV=1
MSQQRSTEQIAASNNNTSDVIALDDDEDTDVQFVAALRPTAPVIDLCVSPSTSAAAAAAA
AAAAHSGGGSGEDPAVGSASGAAASDTDSLASSPDEGALATGVDAAAAVSGEDSPSAAAA
ALECPICLQTCIHPARLPCGHIFCFLCVKGVAYKNRRCAMCRREIPAEFLDHPQLVNGIE
DICTTRATEDGFQWYYEGRNGWWQYDDRTSQDIEDAFKKGDKSCTILVAGYVYIVDLEQL
VQQRQNEPTRCRRVKRDLATIPKKGVAGLRIEGNQVTSDTVFSRPTNTANPTTVAAAASS
FISTIAATDAAIRIASDIIGSTLAHADELTRGLTASNLNDDLSSSGEQSNSTNSNDAGRS
PASSPASSSMQDLITRDLRNTQQVIAHNQHTIDLFEQALNDFQALAMRNYVDSSDEENND
QDQEHELREREQLEAGEQPQSNDNPQRF

Hydra vulgaris:

>XP_012555516.1 E3 ubiquitin-protein ligase rnf146 [Hydra vulgaris]

MTYCWVDSNPDCAVCLQQCLQPVQLPCKHIFCFLCLKGFAYRSKRCALCRSVIELEYFNNPIIIKVREKE
DVSSSTSNKVSVNLSTPSITYNWFYEGRNGWWQYDERASLILEKAFSNSKKNCEILIAGFLYVVDFERMV
QYRKDNLARLRKVKRDSDSTDLKGVAGIKLPKEIENKPDSKNVINKKELLTNTSDNQLNNNQETLATERP
FINLASDLSQNSSGVNEVLYGIQQVALKD

Nematostella vectensis:

>XP_048581887.1 E3 ubiquitin-protein ligase rnf146 isoform X1 [Nematostella vectensis]
MAEGPENVKERQSDISSSKEDVSDSAEPVKNAVYVYLSTPRYLVSYCCIFGDTEEEEVLQNIFELDYQPD
CPVCLQQASYPVRLPCGHMFCFLCIKGVALRSRKCAICRQPISPDYLDKPTLVKVVSGQSSSSDKAPSDP
PADEYVWFYEGRNGWWQYDTKTSKEVESAFKGGKRSCTLLIAGFLYLIDFENMFQMRRNEPGRRRRIKRD
KPNADRKGVAGIRLKGLTVSELADLKNQTTETSTADNQWTSTEDVSVQALSGSPTEAANRDCDDSASSTS
DKESAPDETTHEFACFMLMQPLIAHSESYHNSPVLGDVNAPS

Amphimedon queenslandica:

>XP_003383131.1 PREDICTED: E3 ubiquitin-protein ligase RNF146-like [Amphimedon queenslandica]
MADSENSKKGQYGDSKSLEEKEKKRKSESLDKEGDRVVQVEKDLSKLKIKEDIVPFDMKCPICQDKSRHP
LTLPCGHTFCYLCIKGVYARQKVCALCRQAIPQHCIVSPPGTGEREEEEGRKTCWYYEAKSGGWWEYEER
VRQELEIHYQSLQINNEDNQDDGQFVIYVSGFPYVVDFAKMIQYRQDKPNRSRRIKREKGVAEEGEELVR
GVAGIPIAPSIRQDDN

Alignment

```
Q9NTX7[H.sapiens]                    ------------------------------------------------MMAGC-------           5
NP_001128751.1[P.abelii]             -------------------------------------------------MAGC-------           4
BAE31549.1[M.musculus]               -----------------------------------------------MEMAGC-------           6
XP_015139929.3[G.gallus]             -------------------------------------------------MAGC-------           4
XP_018118669.1[X.laevis]             -------------------------------------------------MAGC-------           4
NP_001167373.1[S.salar]              -------------------------------------------------MASC-------           4
XP_032825805.1[P.marinus]            -------------------------------------------------MSGC-------           4
XP_019623976.1[B.belcheri]           MAESRS-------------VASAETSLRQSSAK------------KR-------------           22
AAL48063.1[D.melanogaster]           MSQQRSTEQIAASNNNTSDVIALDDD-----ED-TDVQFVAALRPTAPVIDLCVSPSTSA           54
XP_012555516.1[H.vulgaris]           ------------------------------------------------------------           0
XP_048581887.1[N.vectensis]          MAEGPENVK-----ERQSDISSSKEDVSDSAEPVKNAVYVY-LSTPRYLVSYC-------           47
XP_003383131.1[A.queenslandica]      -------------------------------------------------MA---------           2

Q9NTX7[H.sapiens]                    --------------------------GEIDHSINMLPTNRK------ANES-----CSN-           27
NP_001128751.1[P.abelii]             --------------------------GEIDHSINMLPTNRK------ANES-----CSN-           26
BAE31549.1[M.musculus]               --------------------------GEIDHSINMLPTNKK------ANES-----CSN-           28
XP_015139929.3[G.gallus]             --------------------------GEIDHSINMLPTNRK------TNES-----CAN-           26
XP_018118669.1[X.laevis]             --------------------------GEVSHSANMLPTNKK------LGEP-----CSN-           26
NP_001167373.1[S.salar]              --------------------------GEVDHSVSSLPSSKK------GSGDSACSGSSG-           31
XP_032825805.1[P.marinus]            --------------------------RVLGTDEP--P--------------SAGVGPGEA           22
XP_019623976.1[B.belcheri]           --------------------------REKEEMA--------------------ETATGQK           36
AAL48063.1[D.melanogaster]           AAAAAAAAAAHSGGGSGEDPAVGSASGAAASDTDSLASSPDEGALATGVDAAAAVSGEDS           114
XP_012555516.1[H.vulgaris]           ----------------------------------------------------------MT           2
XP_048581887.1[N.vectensis]          --------------------------CIFGDTE--------------------EEEVLQN           61
XP_003383131.1[A.queenslandica]      ------DSENSKKGQYGDSKSLEE--KEKKRKSESL---DKEGDRVVQVEKD-LSKLKIK           50

Q9NTX7[H.sapiens]                    TAPSLTVPECAICLQTCVHPVSLPCKHVFCYLCVKGASWLGKRCALCRQEIPEDFLDKPT           87
NP_001128751.1[P.abelii]             TAPSLTVPECAICLQTCVHPVSLPCKHVFCYLCVKGASWLGKRCALCRQEIPEDFLDKPT           86
BAE31549.1[M.musculus]               TAPSLTVPECAICLQTCVHPVSLPCKHVFCYLCVKGASWLGKRCALCRQEIPEDFLDKPT           88
XP_015139929.3[G.gallus]             AAPSLTVPECAICLQTCVHPVSLPCKHVFCYLCVKGASWLGKRCALCRQEIPEDFLDKPT           86
XP_018118669.1[X.laevis]             SAPSSSVPECAICLQICVHPVSLPCKHIFCYLCVKGASWLGRRCALCRQEIPEDFLDKPT           86
NP_001167373.1[S.salar]              SSPALPVPECAICLQSCVHPVQLPCRHVFCFLCVKGASWQSKRCALCRQEVPEDFLEHPT           91
XP_032825805.1[P.marinus]            PPARPVVPDCAICLQPCVHPARLPCRHVFCFLCAKGVALQSRRCALCRSEIPPDFLERPT           82
XP_019623976.1[B.belcheri]           PGGEDMIPECAVCLQTCVQPVRLPCTHIFCYLCVKGVANQSKRCALCRQEIPIEYLERPE           96
AAL48063.1[D.melanogaster]           PSAAAAALECPICLQTCIHPARLPCGHIFCFLCVKGVAYKNRRCAMCRREIPAEFLDHPQ           174
XP_012555516.1[H.vulgaris]           YCWVDSNPDCAVCLQQCLQPVQLPCKHIFCFLCLKGFAYRSKRCALCRSVIELEYFNNPI           62
XP_048581887.1[N.vectensis]          IFELDYQPDCPVCLQQASYPVRLPCGHMFCFLCIKGVALRSRKCAICRQPISPDYLDKPT           121
XP_003383131.1[A.queenslandica]      EDIVPFDMKCPICQDKSRHPLTLPCGHTFCYLCIKGVYARQKVCALCRQAIPQHCIVSPP           110
                                             .* :* : .  *  *** * **:** **     : **:**  :  . :  *

Q9NTX7[H.sapiens]                    LLSPEELK-----------AASR------------G-NGEYAWYYEGR-NGWWQYDERTS           122
NP_001128751.1[P.abelii]             LLSPEELK-----------AASR------------G-NGEYAWYYEGR-NGWWQYGERTS           121
BAE31549.1[M.musculus]               LLSPEELK-----------AASR------------G-NGEYAWYYEGR-NGWWQYDERTS           123
XP_015139929.3[G.gallus]             LLSPEELK-----------AASR------------G-NGEYAWYYEGR-NGWWQYDERTS           121
XP_018118669.1[X.laevis]             LLSPEELK-----------SASR------------G-NGEYAWYYEGR-NGWWQYDERTS           121
NP_001167373.1[S.salar]              LLSPEELK-----------TGGR------------GATGDNAWYYEGR-NGWWQYDERTS           127
XP_032825805.1[P.marinus]            LLCSTSDGQPLAQQQQRRPSASMGVAGGASEGVPLKPRTANAWYYEGR-NGWWQYDERTS           141
XP_019623976.1[B.belcheri]           LVKPIKVTEEQ---------E------------GEEEEEVYCWFYEGR-NGWWQYDERTN           134
AAL48063.1[D.melanogaster]           LVNGIEDICTT-----------------------RATEDGFQWYYEGR-NGWWQYDDRTS           210
XP_012555516.1[H.vulgaris]           IIKVREKEDVSSSTSNKVSVN------------LSTPSITYNWFYEGR-NGWWQYDERAS           109
XP_048581887.1[N.vectensis]          LVKVVSGQSSSSD-----KAP------------SDPPADEYVWFYEGR-NGWWQYDTKTS           163
XP_003383131.1[A.queenslandica]      GTGERE----------------------------EEEGRKTCWYYEAKSGGWWEYEERVR           142
                                          .                                    *:**.: .***:*  :.

Q9NTX7[H.sapiens]                    RELEDAFSKG---------KKNTEMLIAGFLYVADLENMVQYRRNEHGRRRKIKRDIIDI           173
NP_001128751.1[P.abelii]             RELEDAFSKG---------KKNTEMLIAGFLYVADLENMVQYRRNEHGRRRKIKRDIIDI           172
BAE31549.1[M.musculus]               RELEDAFSKG---------KKNTEMLIAGFLYVADLENMVQYRRNEHGRRRKIKRDIIDI           174
XP_015139929.3[G.gallus]             RELEDAFSKG---------KKSTEMLIAGFLYIADLENMVQYRRNEHGRRRKIKRDIIDI           172
XP_018118669.1[X.laevis]             RELEDAFTKG---------KKSTEMLIAGFLYVADLENMVQYRRNEHGRRRKIKRDIVDI           172
NP_001167373.1[S.salar]              RELEDAFSKG---------KKTAEMLIAGFLYVADLENMVQYRRNEHGRRRKIKRDVVDI           178
XP_032825805.1[P.marinus]            AELEEAFARG---------STTMEMLIAGYLYVADLEAMVQYRRSEPSRRRKMKRDLLSA           192
XP_019623976.1[B.belcheri]           AELEDAYTKK---------KKSCELLIAGFLYIIDFVNMLQFRRNDPSRRRRIKRDLTNT           185
AAL48063.1[D.melanogaster]           QDIEDAFKKG---------DKSCTILVAGYVYVVDLEQLVQQRQNEPTRCRRVKRDLATI           261
XP_012555516.1[H.vulgaris]           LILEKAFSNS---------KKNCEILIAGFLYVVDFERMVQYRKDNLARLRKVKRDSDST           160
XP_048581887.1[N.vectensis]          KEVESAFKGG---------KRSCTLLIAGFLYLIDFENMFQMRRNEPGRRRRIKRDKPNA           214
XP_003383131.1[A.queenslandica]      QELEIHYQSLQINNEDNQDDGQFVIYVSGFPYVVDFAKMIQYRQDKPNRSRRIKREKGVA           202
                                       :*  :            .    : ::*: *: *:  :.* *:..  * *::**:

Q9NTX7[H.sapiens]                    P-----KKGVAGLRLDCDANTVNL---ARESSADGADSVSAQS-----------------           208
NP_001128751.1[P.abelii]             P-----KKGVAGLRLDCDANTVNL---ARESSADGADSVSAQS-----------------           207
BAE31549.1[M.musculus]               P-----KKGVAGLRLDCDTNTVNL---ARESSADGADSGSAQT-----------------           209
XP_015139929.3[G.gallus]             P-----KKGVAGLRLDCDTNSVNL---ARESSADGADSTLTGG-----------------           207
XP_018118669.1[X.laevis]             P-----KKGVAGLRLECDPANLNL---ARESSADGADNMAALG-----------------           207
NP_001167373.1[S.salar]              P-----KKGVAGLRLDTEGGVQGSAAAGRGNSADGADTSAAAVQQAA-------------           220
XP_032825805.1[P.marinus]            P-----KKGVAGLRLDLTAPAVSPA---HASGVDGVDRAVAGTSSGSGSVVAGGFSMLSS           244
XP_019623976.1[B.belcheri]           P-----KKGVAGVRLPAPAPPRNAP----ADGVEGANEGATGGSRS---VAVTSRATKST           233
AAL48063.1[D.melanogaster]           P-----KKGVAGLRIEGNQVTSDTVFS-RPTNTANPTTVAAAASS-----------FIST           304
XP_012555516.1[H.vulgaris]           D-----LKGVAGIKLPKEIENKP-D----SKNVINKKELLTNTSDN--------------           196
XP_048581887.1[N.vectensis]          D-----RKGVAGIRLKGLTVSELAD----LKNQT----TETSTADN--------------           247
XP_003383131.1[A.queenslandica]      EEGEELVRGVAGIPIAPSIRQDDN------------------------------------           226
                                            :****: :

Q9NTX7[H.sapiens]                    --------------------------------GASVQPLV-SSVRPLTSVDGQ--LTSPA           233
NP_001128751.1[P.abelii]             --------------------------------GASVQPLV-SSVRPLTSVDGQ--LTSPA           232
BAE31549.1[M.musculus]               --------------------------------GASVQLAVPSSTRPLTSVDGQ--LTSPV           235
XP_015139929.3[G.gallus]             --------------------------------AAAVQPLVPVSARPLPSLDGQ--LMSPS           233
XP_018118669.1[X.laevis]             --------------------------------ASSSQPAPVLPARLHTSLS-T--TASQV           232
NP_001167373.1[S.salar]              -------------------------------AAPAATTVLSAPARPPTSLGGQ--PGSPT           247
XP_032825805.1[P.marinus]            AASVISSGLSAALGVSSAAFGGSVASGGLLASAGPSTITVASPSSPVSSVATS--STLSV           302
XP_019623976.1[B.belcheri]           RGPADHDEDDSNEDTNNR--------------AE---EDSTSEDVTDQSARGN--ARQSP           274
AAL48063.1[D.melanogaster]           IAA-----TDAAIRIASDIIGSTLAHADELT------RGLTASN-LNDDLSSSDSFSICT           352
XP_012555516.1[H.vulgaris]           -------QLNNNQET------------------------------------------LA-           206
XP_048581887.1[N.vectensis]          -------QWTSTEDV------------------------------SVQALSGS--PTEA-           267
XP_003383131.1[A.queenslandica]      ------------------------------------------------------------           226

Q9NTX7[H.sapiens]                    TPSPDAST------SLEDSFAHLQL----SGDNTAE----RSH--RGEGEE---DHESP-           273
NP_001128751.1[P.abelii]             TPSPDAST------SLEDSFAHLQL----SGDNTAE----RSH--RGEGEE---DHESP-           272
BAE31549.1[M.musculus]               TPSPDAGI------SLEDSFAHLQL----SGDSIAE----RSH--RGEGEE---DHESP-           275
XP_015139929.3[G.gallus]             TPSPDAST------SLENSFAHLQI----NGDSMAE----RSH--RGEGEE---DHESS-           273
XP_018118669.1[X.laevis]             LSHSDVTN------SLENSFAELQI----GD-PVIG----RNN--IGEGEE---GQPL--           270
NP_001167373.1[S.salar]              S------P------SLEDTLALLHI----SPTDAPE----RAE--VGEGEE---EATAT-           281
XP_032825805.1[P.marinus]            DPLPDSGATSSFP-SRPATFAPQHQMRAVVGPPSPADSDDSSD--RDDEEQDYEDNEGAA           359
XP_019623976.1[B.belcheri]           D-----------------SL---RQ-----HTPTQEEDDDGDTNSTGGAEM-SE------           302
AAL48063.1[D.melanogaster]           PPLLLT-AKCVFVASLISTV-YLKLY----------------------------------           376
XP_012555516.1[H.vulgaris]           ------------------------T-----ERPFINLASDLSQNSSGVNEVLYG------           231
XP_048581887.1[N.vectensis]          ------------------------A-----NRDCDDSASSTSDKESAPDETTHE------           292
XP_003383131.1[A.queenslandica]      ------------------------------------------------------------           226
```

```
Q9NTX7[H.sapiens]                    -SS-----GR--------------------VPA-PD--TSIEETESDASSDSE--D-VSA     301
NP_001128751.1[P.abelii]             -SS-----GR--------------------VPA-PD--TSIEETESDASSDSE--D-VSA     300
BAE31549.1[M.musculus]               -SS-----GR--------------------VP---D--TSVEETESDASSDSE--D-APV     301
XP_015139929.3[G.gallus]             -SS-----GR--------------------VPA-PD--TSVEETESDASSDSE--D-VSA     301
XP_018118669.1[X.laevis]             -IN-----PR--------------------MPP-PN--AVLEEPEPSDSND------HGS     295
NP_001167373.1[S.salar]              -PS-----MS---------------------SS-PN--TYAD-GSGDWSDDEG--D-GEA     307
XP_032825805.1[P.marinus]            SISRQLAGMRLGRSGQISLAGEGGGSSRAVYAA-PH--LAADEDGSRLLGRRRRTNMPPP     416
XP_019623976.1[B.belcheri]           -MVRMLQRSRLGRGSDSSRSGVLQAVLQGQVSSVANVAMAREEIPGDSSDESD-------     354
AAL48063.1[D.melanogaster]           ------------------------------------------------------------     376
XP_012555516.1[H.vulgaris]           -----IQQVALK------------------D-----------------------------     239
XP_048581887.1[N.vectensis]          FACFMLMQPLIAHSESYHNSPVLG-----DVNA-----------P------S--------     322
XP_003383131.1[A.queenslandica]      ------------------------------------------------------------     226

Q9NTX7[H.sapiens]                    VV-AQHSLTQQRLLVSN------------ANQTVPDRSDRSGTDRSV-A-----------     336
NP_001128751.1[P.abelii]             VV-AQHSLTQQRLLVSN------------ANQTVPDRSDRSGTDRSV-A-----------     335
BAE31549.1[M.musculus]               VV-AQHSLTQQRPLVPN------------GNQTVADQSDRSGTDRSV-A-----------     336
XP_015139929.3[G.gallus]             PL-QQHPSSAQQR-------------------HLNANANQSGADRPV-A-----------     329
XP_018118669.1[X.laevis]             PA-LQHHSFLGNP-----------------------------------------------     307
NP_001167373.1[S.salar]              VEPREQRLRLGES----------------------------LVDRSPPG-----------     328
XP_032825805.1[P.marinus]            PPPHRQPALLPDSLAARQDSGTGRLPLRASERLIARQHGAHGGAESSSGGGGTGGAVIDT     476
XP_019623976.1[B.belcheri]           ------------------------------------------------------------     354
AAL48063.1[D.melanogaster]           ------------------------------------------------------------     376
XP_012555516.1[H.vulgaris]           ------------------------------------------------------------     239
XP_048581887.1[N.vectensis]          ------------------------------------------------------------     322
XP_003383131.1[A.queenslandica]      ------------------------------------------------------------     226

Q9NTX7[H.sapiens]                    ----------GGGTVSVSVRSRRPDGQCTVTEV---------------------------     359
NP_001128751.1[P.abelii]             ----------GGGTVSVSVRSRRPDGQCTVTEV---------------------------     358
BAE31549.1[M.musculus]               ----------GGRTMSVNVRSRRPDGQCTVTEV---------------------------     359
XP_015139929.3[G.gallus]             ----------GGGVANASVRSRRPDGQCTVTEV---------------------------     352
XP_018118669.1[X.laevis]             ------------------------------------------------------------     307
NP_001167373.1[S.salar]              ----------AEASSSSSVRSRRPDGQCTEV-----------------------------     349
XP_032825805.1[P.marinus]            AEAQQQSAAPRSATLHVRSRDGRPDGQYTVTDVVNSAILLLLYMWVLGEPLGTQRGARAA     536
XP_019623976.1[B.belcheri]           ------------------------------------------------------------     354
AAL48063.1[D.melanogaster]           ------------------------------------------------------------     376
XP_012555516.1[H.vulgaris]           ------------------------------------------------------------     239
XP_048581887.1[N.vectensis]          ------------------------------------------------------------     322
XP_003383131.1[A.queenslandica]      ------------------------------------------------------------     226

Q9NTX7[H.sapiens]                    ------------------------------------------------------------     359
NP_001128751.1[P.abelii]             ------------------------------------------------------------     358
BAE31549.1[M.musculus]               ------------------------------------------------------------     359
XP_015139929.3[G.gallus]             ------------------------------------------------------------     352
XP_018118669.1[X.laevis]             ------------------------------------------------------------     307
NP_001167373.1[S.salar]              ------------------------------------------------------------     349
XP_032825805.1[P.marinus]            SSYRRVRGGLCTYTFVIPPDLSACASLAQSMRDADPDEEDSDRIHNKDRTRSPDRKGDLE     596
XP_019623976.1[B.belcheri]           ------------------------------------------------------------     354
AAL48063.1[D.melanogaster]           ------------------------------------------------------------     376
XP_012555516.1[H.vulgaris]           ------------------------------------------------------------     239
XP_048581887.1[N.vectensis]          ------------------------------------------------------------     322
XP_003383131.1[A.queenslandica]      ------------------------------------------------------------     226

Q9NTX7[H.sapiens]                    ------------------------------------------------------------     359
NP_001128751.1[P.abelii]             ------------------------------------------------------------     358
BAE31549.1[M.musculus]               ------------------------------------------------------------     359
XP_015139929.3[G.gallus]             ------------------------------------------------------------     352
XP_018118669.1[X.laevis]             ------------------------------------------------------------     307
NP_001167373.1[S.salar]              ------------------------------------------------------------     349
XP_032825805.1[P.marinus]            AASPVDGDVSKKDLDETSERIQQLESALGNATLRLHKLESAVREAGGARGHSGITRSLQK     656
XP_019623976.1[B.belcheri]           ------------------------------------------------------------     354
AAL48063.1[D.melanogaster]           ------------------------------------------------------------     376
XP_012555516.1[H.vulgaris]           ------------------------------------------------------------     239
XP_048581887.1[N.vectensis]          ------------------------------------------------------------     322
XP_003383131.1[A.queenslandica]      ------------------------------------------------------------     226

Q9NTX7[H.sapiens]                    ------------------------------------------------------------     359
NP_001128751.1[P.abelii]             ------------------------------------------------------------     358
BAE31549.1[M.musculus]               ------------------------------------------------------------     359
XP_015139929.3[G.gallus]             ------------------------------------------------------------     352
XP_018118669.1[X.laevis]             ------------------------------------------------------------     307
NP_001167373.1[S.salar]              ------------------------------------------------------------     349
XP_032825805.1[P.marinus]            KVLEQARNIQTLQHSNRHLERKVTELESRREVTRRKILNRQPYTSTTTNDDDGDDDDDED     716
XP_019623976.1[B.belcheri]           ------------------------------------------------------------     354
AAL48063.1[D.melanogaster]           ------------------------------------------------------------     376
XP_012555516.1[H.vulgaris]           ------------------------------------------------------------     239
XP_048581887.1[N.vectensis]          ------------------------------------------------------------     322
XP_003383131.1[A.queenslandica]      ------------------------------------------------------------     226

Q9NTX7[H.sapiens]                    ------------------------------------------------------------     359
NP_001128751.1[P.abelii]             ------------------------------------------------------------     358
BAE31549.1[M.musculus]               ------------------------------------------------------------     359
XP_015139929.3[G.gallus]             ------------------------------------------------------------     352
XP_018118669.1[X.laevis]             ------------------------------------------------------------     307
NP_001167373.1[S.salar]              ------------------------------------------------------------     349
XP_032825805.1[P.marinus]            DEDDNDDDDEEDDDAHDDDDDKNNEVSIRHQSSDEAQPKNRLLHGGKSQHNTTAVQGRAR     776
XP_019623976.1[B.belcheri]           ------------------------------------------------------------     354
AAL48063.1[D.melanogaster]           ------------------------------------------------------------     376
XP_012555516.1[H.vulgaris]           ------------------------------------------------------------     239
XP_048581887.1[N.vectensis]          ------------------------------------------------------------     322
XP_003383131.1[A.queenslandica]      ------------------------------------------------------------     226

Q9NTX7[H.sapiens]                    ------------------------------------------------------------     359
NP_001128751.1[P.abelii]             ------------------------------------------------------------     358
BAE31549.1[M.musculus]               ------------------------------------------------------------     359
XP_015139929.3[G.gallus]             ------------------------------------------------------------     352
XP_018118669.1[X.laevis]             ------------------------------------------------------------     307
NP_001167373.1[S.salar]              ------------------------------------------------------------     349
XP_032825805.1[P.marinus]            GAQVTRELRGATASAETGRDTTAAGVPAGPGGPYQDCAEIYRSGQTRSGIYVLHVFNTSH     836
XP_019623976.1[B.belcheri]           ------------------------------------------------------------     354
AAL48063.1[D.melanogaster]           ------------------------------------------------------------     376
XP_012555516.1[H.vulgaris]           ------------------------------------------------------------     239
XP_048581887.1[N.vectensis]          ------------------------------------------------------------     322
XP_003383131.1[A.queenslandica]      ------------------------------------------------------------     226

Q9NTX7[H.sapiens]                    ------------------------------------------------------------     359
```

```
NP_001128751.1[P.abelii]            ------------------------------------------------------------           358
BAE31549.1[M.musculus]              ------------------------------------------------------------           359
XP_015139929.3[G.gallus]            ------------------------------------------------------------           352
XP_018118669.1[X.laevis]            ------------------------------------------------------------           307
NP_001167373.1[S.salar]             ------------------------------------------------------------           349
XP_032825805.1[P.marinus]           TLKVYCDMQTEGGGWTVFQRREDGHINFNRPWQDYKLGFGSADREYWLGNEAVHVLTSRR           896
XP_019623976.1[B.belcheri]          ------------------------------------------------------------           354
AAL48063.1[D.melanogaster]          ------------------------------------------------------------           376
XP_012555516.1[H.vulgaris]          ------------------------------------------------------------           239
XP_048581887.1[N.vectensis]         ------------------------------------------------------------           322
XP_003383131.1[A.queenslandica]     ------------------------------------------------------------           226

Q9NTX7[H.sapiens]                   ------------------------------------------------------------           359
NP_001128751.1[P.abelii]            ------------------------------------------------------------           358
BAE31549.1[M.musculus]              ------------------------------------------------------------           359
XP_015139929.3[G.gallus]            ------------------------------------------------------------           352
XP_018118669.1[X.laevis]            ------------------------------------------------------------           307
NP_001167373.1[S.salar]             ------------------------------------------------------------           349
XP_032825805.1[P.marinus]           PQALRILLWDWDENQAYASYARFTLRGEAARYRLLVSGFSGTAGRYSSLAQSGAEFSTRD           956
XP_019623976.1[B.belcheri]          ------------------------------------------------------------           354
AAL48063.1[D.melanogaster]          ------------------------------------------------------------           376
XP_012555516.1[H.vulgaris]          ------------------------------------------------------------           239
XP_048581887.1[N.vectensis]         ------------------------------------------------------------           322
XP_003383131.1[A.queenslandica]     ------------------------------------------------------------           226

Q9NTX7[H.sapiens]                   ------------------------------------------------------------           359
NP_001128751.1[P.abelii]            ------------------------------------------------------------           358
BAE31549.1[M.musculus]              ------------------------------------------------------------           359
XP_015139929.3[G.gallus]            ------------------------------------------------------------           352
XP_018118669.1[X.laevis]            ------------------------------------------------------------           307
NP_001167373.1[S.salar]             ------------------------------------------------------------           349
XP_032825805.1[P.marinus]           QDHDRCQCKCAQLSSGGWWFEACGPSNLNGQFFPKGSHYGHFNGIKWHYWRGASYSLKAS           1016
XP_019623976.1[B.belcheri]          ------------------------------------------------------------           354
AAL48063.1[D.melanogaster]          ------------------------------------------------------------           376
XP_012555516.1[H.vulgaris]          ------------------------------------------------------------           239
XP_048581887.1[N.vectensis]         ------------------------------------------------------------           322
XP_003383131.1[A.queenslandica]     ------------------------------------------------------------           226

Q9NTX7[H.sapiens]                   ---------  359
NP_001128751.1[P.abelii]            ---------  358
BAE31549.1[M.musculus]              ---------  359
XP_015139929.3[G.gallus]            ---------  352
XP_018118669.1[X.laevis]            ---------  307
NP_001167373.1[S.salar]             ---------  349
XP_032825805.1[P.marinus]           TMMIRPADF  1025
XP_019623976.1[B.belcheri]          ---------  354
AAL48063.1[D.melanogaster]          ---------  376
XP_012555516.1[H.vulgaris]          ---------  239
XP_048581887.1[N.vectensis]         ---------  322
XP_003383131.1[A.queenslandica]     ---------  226
```
