# Supplementary material for: An Evolutionary Perspective on the Origin, Conservation and Binding Partner Acquisition of Tankyrases
Source: Biomolecules. 2022 Nov 15;12(11):1688. doi: 10.3390/biom12111688 (PMC9688111; doi:10.3390/biom12111688)
Supplement: Supplementary file 1 [file biomolecules-12-01688-s001.zip › biomolecules-1913719-supplementary-11.17 -done/Data_7/SH3BP2.pdf]

SH3BP2

Homo sapiens:

>sp|P78314|3BP2_HUMAN SH3 domain-binding protein 2 OS=Homo sapiens OX=9606 GN=SH3BP2 PE=1 SV=2
MAAEEMHWPVPMKAIGAQNLLTMPGGVAKAGYLHKKGGTQLQLLKWPLRFVIIHKRCVYY
FKSSTSASPQGAFSLSGYNRVMRAAEETTSNNVFPFKIIHISKKHRTWFFSASSEEERKS
WMALLRREIGHFHEKKDLPLDTSDSSSDTDSFYGAVERPVDISLSPYPTDNEDYEHDDED
DSYLEPDSPEPGRLEDALMHPPAYPPPPVPTPRKPAFSDMPRAHSFTSKGPGPLLPPPPP
KHGLPDVGLAAEDSKRDPLCPRRAEPCPRVPATPRRMSDPPLSTMPTAPGLRKPPCFRES
ASPSPEPWTPGHGACSTSSAAIMATATSRNCDKLKSFHLSPRGPPTSEPPPVPANKPKFL
KIAEEDPPREAAMPGLFVPPVAPRPPALKLPVPEAMARPAVLPRPEKPQLPHLQRSPPDG
QSFRSFSFEKPRQPSQADTGGDDSDEDYEKVPLPNSVFVNTTESCEVERLFKATSPRGEP
QDGLYCIRNSSTKSGKVLVVWDETSNKVRNYRIFEKDSKFYLEGEVLFVSVGSMVEHYHT
HVLPSHQSLLLRHPYGYTGPR

Pongo abelii:

>PNJ23454.1 SH3BP2 isoform 3 [Pongo abelii]
MASLGPRTPALSRSRGRRAMCWVSTISFMAAEETHWPVPMKAIGAQNLLTMPGGVAKAGYLHKKGGTQLQ
LLKWPLRFVIIHKRCVYYFKSSTSASPQGAFSLSGYNRVMRAAEETTSNNVFPFKIIHISKKHRTWFFSA
SSEDERKSWMALLRREIGHFHEKKDLPLDTSDSSSDTDSFYGAVERPVDISLSPYPTDNEDYEHDDEDDS
YMEPDSPEPGRLEDALMHPPAYPPPPVPTPRKPAFSDMPRAHSFTSKGPGPLLPPPPPKHGLPDVGLAAE
DSKRDPLCPRRAEPCPRVPATPRRMSDPPLSTMPTAPGLRKPPCFRESASPSPSPEPWTPGHGACSTSSA
AIMATATSRNCDKLKSFHLSPRGPPTSQPPPVPANKPKFLKTAEEDPPREAAMPGLFVSPVAPRPPALKL
PVPEATARPAVLPRPEKPQLPHLQRSPPDGQSFRSFSFEKPRQPLPADTGGDDSDEDYEKVPLPNSVFIN
TTESCEVERLFKATSPRGEPQDGLYCIRNSSTKSGKVLVVWDETSNKVRNYRIFEKDSKFYLEGEVLFVS
VGSMVEHYHTHVLPSHQSLLLRHPYGYTGPR

Mus musculus:

>NP_001139330.1 SH3 domain-binding protein 2 isoform c [Mus musculus]
MSGGCRLQSLLIAPELNSLSGFKDTCPQQVTGSCTMCWVSAMSFMAAEEMQWPVPMKAIGAQNLLTMPGG
VAKAGYLHKKGGTQLQLLKWPLRFVIIHKRCIYYFKSSTSASPQGAFSLSGYNRVMRAAEETTSNNVFPF
KIIHISKKHRTWFFSASSEDERKSWMALLRREIGHFHEKKELPLDTSDSSSDTDSFYGAVERPIDISLSS
YPMDNEDYEHEDEDDSYLEPDSPGPMKLEDALTYPPAYPPPPVPVPRKPAFSDLPRAHSFTSKSPSPLLP
PPPPKRGLPDTGSAPEDAKDALGLRRVEPGLRVPATPRRMSDPPMSNVPTVPNLRKHPCFRDSVNPGLEP
WTPGHGTSSVSSSTTMAVATSRNCDKLKSFHLSSRGPPTSEPPPVPANKPKFLKIAEEPSPREAAKFAPV
PPVAPRPPVQKMPMPEATVRPAVLPRPENTPLPHLQRSPPDGQSFRGFSFEKARQPSQADTGEEDSDEDY
EKVPLPNSVFVNTTESCEVERLFKATDPRGEPQDGLYCIRNSSTKSGKVLVVWDESSNKVRNYRIFEKDS
KFYLEGEVLFASVGSMVEHYHTHVLPSHQSLLLRHPYGYAGPR

Gallus gallus:

>XP_015141266.1 SH3 domain-binding protein 2 isoform X1 [Gallus gallus]
MPERGYQLLMALSSQRKLSYGESVSRRTMCRLGTVTRKMASEEQVWPVPMKAIGAQNLLTMPGGVTKSGY
LHKKGGTQLQILKWPLRFVIIHEGCIYYFKTSTSASPQGAFSLNGYNRVMRAAEETTSSNVFPFKLVHIS
KKHRTWFFSASSEDERKNWMLSLRREIDHYHDKKETVTEFSDSGSDADSFYGSVERPIDIKYSHHSADNE
DYDQEEDDESYLQPDTSDIVKDDFMVLPPAYPPPPVPHVRKAAYSESRSHSFSGKTAGSTPPPPPPKRSL
PEIKTEDFFGVREPQLPCRAEPNLKIQSSSRRPSEQPPPVPPLPLFKKPFCVKEPSSLPPELPLSHVLTT
TEGCEKLKTLNLSPRTPPPLPSNKPKLSQITEKTVENKVPREHGKPGLFVPPVLPKPPVPGHQHSVLKPR
PEKPSCPQLQRSPPDGQSFRSFSFEKPALPSKPNQVNDDSDDDYEKVELPTSIFLNTSESLEVERIFKAT
SPKGQPQNGLYCIRNSSTKAGKVLVVWDQSAEKVRNYRIFEKDCKFYLDADIMFLNMGSLVEYYSTHVLP
SHGSLILRCPYGYSKPR

Xenopus laevis:

>XP_041443552.1 SH3 domain-binding protein 2 isoform X3 [Xenopus laevis]
MASDEPSWPTPMKAIGAQNLLTMPGGVAISGYLHKKGGTQLQIMKWPLRFVIIHKGCVYYFKTSTSATSQ
GAFSLNGYNRVMRAAEETTSNNVFPFKMVHISKKQRTWYFSAASEEERKKWMLSLRKEIDHYHEKKETVT
DLSDPDSDSDSFYGSVERPVPINYTHNPADDSYEDDDDEDDDYVKPDGADENAPTYPPPPVPRNPRNGND
SVKPRTMSDVGLPYRPPPPPTPPLIKTLPHLSDQARKESAIHQGFSCGPPPHKIEEFTPKLPPHKKTSFD
LIGKEPPRREDLIGSSDSVPGYDLCGPTIKPPSPSLQLGARHVDPPAPPPVSYPKKNGSSCKPMTNPSTQ
LISNTVSKELTDKFRMMPVPSPAPPPKPSFLSPVVKPSSSSNAPPPPPPVKPSIPPSTPPSVTIASISPP
VPPIKPRMLNDQKAERIPQKPPPIPRQPGRKSESDKLEPSPKLRSPPDGQSFRGLSTEGPAHPIKPKRKL
TRNDSDEDYEKVPLPVSVFVDTNDSVEVERIFKAASPGGTPQNGLFCIRNSAKAGKVLVVWDTATEKTRN
YRIFEKDSKYFLEAELLFPDVESLVEHYYTNKLPSHSTLVLQHAYGCSFPR

Salmo salar:

>XP_013984441.1 PREDICTED: SH3 domain-binding protein 2-like isoform X1 [Salmo salar]
MASAENYWPVPMRAIGAQNLLTMPGGVCHSGYLHKKGGSQFSLMKWPLRYIIIHRGCVYYFKSSTSPAPQ
GAFSLNGYNRVMRAAEETTSSNVFPFKIVHFSKKHRTYCFSAANEEERRKWMRNLRKEIDHYNDRRDSHI
PNDSESDADSFYGSIECPMDITYAHDDPGDNYMLEDEEDDEDEDDYEKPDSPPTYKGRLTVPPPSYHPPP
LPCQFRQESMSGSCKGLPQPAPVPNRTLSSPLPKKMSPLLPRPKLGEVGHNHHGHKDVPNKDSPERSVRG
PKAPHPTHIASLQKQMMMGKTSISTLPGGDHRDKKPHAPVTLKSPATLPICINLERQMGLNPSPPGCHGN
TPSHIPSVPNYCHSTRPTPDLPPQPSPALNHKLGPYKPPLLTKPPCPATMPKRPRGSAQRSPPDGLSSQT
SVDQVPASLRKNRDPSKYIDDDSDEDYENVQLPDSVFVDITETSSVEKMFMESSSSPQDGLYCFRNSASK
TSKVLVVWDVDLCKARNYRLFEEDSRVFLEFEMTFASLSALVEHYHSHPLPNHDSLCLQQPYRYIMPR

Petromyzon marinus:

>XP_032800705.1 SH3 domain-binding protein 2-like [Petromyzon marinus]
MSAACRDWPLPMRSVGAQSILTMPGGVAHSGYLHKKGGTQVSFFAWPLRYVVLHAGCLYYYKSSTATIPR
GSFSLRGYNRVMRALQETSMGNVYPFKLVHHNVRHRTWIFSAANEGERKVWMSAIRSEIENYLRPQSSNE
RRNSSSSDKFYEQVEMPLPLLDSTWSGGSQDADEYDEDSGDDDDDGDYLQPDDYLTTHLESGTGGGLSPQ
PKPQQQPPPPQLLLASSPSPSPANKFTATTATTKRPALMPPKPPPKPPPQQPQQQQQPQQQQPQQHHHHQ
ELGYFKRPIAGTAIGPQQRSPPLPWAAVAAAAAASTTTTTTTATTTTTTARGKALMPAATTTTITTTKAP
PRPLSRPQPEGESFHKLQIDSDSDDYIQPEDAMPSTIFLDTTSGDEAEWRMKNAGLTHGLYCIRNSASSD
QQVLVVWDGECRKLRNFKIFRAGPLVSLDVSDGAPHFKLLSGLLDHYRVAPLPRQSSLRLTQPLVV

Branchiostoma belcheri:

>XP_019631975.1 PREDICTED: SH3 domain-binding protein 2-like [Branchiostoma belcheri]
MISLALALPHPLRMAGAKDSKNRLSLSAGLGGVKKSFLSALGQQDKHVIPGVTEPHITISAQELLSDGKA
IFYGWLRKKGNDVLSQRFMIWKHRYVILKDGCVYYFKNETSEKPCGAFSLSNYTKIMRAVDVETTELPWP
FLLVGRFPQMRTWYFSASNEQDMQHWMLNMKNEMDAVNSSVQGAGHGGRGSNPEVIVPDPAYGDDSGLPP
IYQDTDEEEHYYEDPDEHLQEEEDYLQVLEDGPRVTPVGAEAAGDVGAFREMPLPPTPTSKMASPSKGWY

GQEGAARPPVPLPKKQSSFPTKPLPATPQEGTRSPKKALVPPHMRGVSTSPPHQTESKGKVPSPPWKELK
GPGVKKASEPINITSPGWTAGKTVGQRETGEGRGRDVKDEKKEKSGMARALAAEMTQKFGSNWQAAGGTA
KPRTPDKPVPAAKPGLHPKPGLAPKPGTSPKPGLAPKPGHPSRQGMKPLVPPHKPQVEVQGIMEDRYEHE
EEEEPVEDPGIRRTPPEGNSFSKKNFNENPESETDDVMPADVSDDVDVEKAESCLRSGQVNGKYFLRPSK
QDKGKMVLVVYDCAGQKCRKFKMYGLGSQLYLHKGAPTFSSMSDLLRHYQYHNLPVTDGVEIRLTEPYQG
H

Drosophila melanogaster:

No homolog found.

Hydra vulgaris:

No homolog found.

Nematostella vectensis:

>XP_032222077.1 SH3 domain-binding protein 2 isoform X3 [Nematostella vectensis]
MSNGPTWRTCSMPTTRHNECSVPGNRIRSRTDTNITAPKHPEPYMSIPAQSLVQDECHIHCGTVRKENKY
NMWQDRYLVLHKGCMYYYKNYLDKSAKGQFSLSGYRVSTAPERAVKLPWVFKLTHLQPEKRTFYFAAKSE
KELNEWMSKIKEDIDEYCEPLATSGFNPECEATRQSSSGSSDDGDSFKYDYPLFAPEFALAKALTLEKLS
ESQNSSSASSEPEYCPPPSFDTLPNGSPPPPPARNQSGKERAQPIGSVPTPVMMGKSGKSSVDISAVILG
KAALKPVQDNSLPPLPKPRSKLRQSPRVHAGSHPSSPSPPTKPPKPSVHKYASDPELACLPQQTSLPPAN
PALVDSSDSEEDDYLKILPDEESTPNHGNNNDEFIPRLSTLERLRPEGKSFRTPPDEKEQIHIDVLPATS
VKLDIERTKAIAMLEGRNGMYILHKSRSGDIGQCLSVCIEDRVRHFLVFYSKEEGGYALDRDAQRFPTLE
EVIRHHYTTPLPKQQSATLEQPYRWPH

Amphimedon queenslandica:

No homolog found.

Alignment

```
XP_032222077.1[N.vectensis]      -MSNGPTW--------------------RTC-SMPTTRHNECSVPGNRIRSRTDTNITAP   38
XP_019631975.1[B.belcheri]       MIS------LALALPHPLRMAGAKDSKNRLSLSAGLGGVKKSFLSAL----GQQDKHVIP   50
XP_032800705.1[P.marinus]        ------------------------------------------------------MSAACR   6
XP_013984441.1[S.salar]          ------------------------------------------------------MASAEN   6
XP_041443552.1[X.laevis]         ------------------------------------------------------MASDEP   6
XP_015141266.1[G.gallus]         MPERGYQ--------LLMALSSQR----KLSYGESVSRRTMCRLGTV----TRKMASEEQ   44
NP_001139330.1[M.musculus]       -MSGGCRLQSLLIAPELNSLSGFK----DTCPQQVTGSCTMCWVSAM-----SFMAAEEM   50
P78314[H.sapiens]                ------------------------------------------------------MAAEEM   6
PNJ23454.1[P.abelii]             -----------------MASLGPR----TPALSRSRGRRAMCWVSTI-----SFMAAEET   34


XP_032222077.1[N.vectensis]      KHPEPYMSIPAQSLVQ-DECHIHCGTVRKEN------KYNMWQDRYLVLHKGCMYYYKNY   91
XP_019631975.1[B.belcheri]       GVTEPHITISAQELLS-DGKAIFYGWLRKKGNDVLSQRFMIWKHRYVILKDGCVYYFKNE   109
XP_032800705.1[P.marinus]        DWPLPMRSVGAQSILTMPGGVAHSGYLHKKGGTQV--SFFAWPLRYVVLHAGCLYYYKSS   64
XP_013984441.1[S.salar]          YWPVPMRAIGAQNLLTMPGGVCHSGYLHKKGGSQF--SLMKWPLRYIIIHRGCVYYFKSS   64
XP_041443552.1[X.laevis]         SWPTPMKAIGAQNLLTMPGGVAISGYLHKKGGTQL--QIMKWPLRFVIIHKGCVYYFKTS   64
XP_015141266.1[G.gallus]         VWPVPMKAIGAQNLLTMPGGVTKSGYLHKKGGTQL--QILKWPLRFVIIHEGCIYYFKTS   102
NP_001139330.1[M.musculus]       QWPVPMKAIGAQNLLTMPGGVAKAGYLHKKGGTQL--QLLKWPLRFVIIHKRCIYYFKSS   108
P78314[H.sapiens]                HWPVPMKAIGAQNLLTMPGGVAKAGYLHKKGGTQL--QLLKWPLRFVIIHKRCVYYFKSS   64
PNJ23454.1[P.abelii]             HWPVPMKAIGAQNLLTMPGGVAKAGYLHKKGGTQL--QLLKWPLRFVIIHKRCVYYFKSS   92
                                     *  :: **.::         * ::*:.          *  *:::::  *:**:*.

XP_032222077.1[N.vectensis]      LDKSAKGQFSLSGYRVS--TAPERAVKLPWVFKLTHLQPEKRTFYFAAKSEKELNEWMSK   149
XP_019631975.1[B.belcheri]       TSEKPCGAFSLSNYTKIMRAVDVETTELPWPFLLVGRFPQMRTWYFSASNEQDMQHWMLN   169
XP_032800705.1[P.marinus]        TATIPRGSFSLRGYNRVMRALQETSMGNVYPFKLVHHNVRHRTWIFSAANEGERKVWMSA   124
XP_013984441.1[S.salar]          TSPAPQGAFSLNGYNRVMRAAEETTSSNVFPFKIVHFSKKHRTYCFSAANEEERRKWMRN   124
XP_041443552.1[X.laevis]         TSATSQGAFSLNGYNRVMRAAEETTSNNVFPFKMVHISKKQRTWYFSAASEEERKKWMLS   124
XP_015141266.1[G.gallus]         TSASPQGAFSLNGYNRVMRAAEETTSSNVFPFKLVHISKKHRTWFFSASSEDERKNWMLS   162
NP_001139330.1[M.musculus]       TSASPQGAFSLSGYNRVMRAAEETTSNNVFPFKIIHISKKHRTWFFSASSEDERKSWMAL   168
P78314[H.sapiens]                TSASPQGAFSLSGYNRVMRAAEETTSNNVFPFKIIHISKKHRTWFFSASSEEERKSWMAL   124
PNJ23454.1[P.abelii]             TSASPQGAFSLSGYNRVMRAAEETTSNNVFPFKIIHISKKHRTWFFSASSEDERKSWMAL   152
                                       * *** .*     :    :    : * :     . **: *:* .* : . **

XP_032222077.1[N.vectensis]      IKEDIDEYCEPLATSGFNPECEATRQSSSGSSDDGDSFKYDY------------------   191
XP_019631975.1[B.belcheri]       MKNEMDAVNSSVQGAGHGGRGSNP------EVIVPDPAYGDDSGLPPI-YQDTD---EEE   219
XP_032800705.1[P.marinus]        IRSEIENYLRPQSSNE--RRN----------SSSSDKFYEQVEMPLPLLDSTWSGGSQDA   172
XP_013984441.1[S.salar]          LRKEIDHYNDRRDSHI-PNDS----------ESDADSFYGSIECPMDITYAHDDPG---D   170
XP_041443552.1[X.laevis]         LRKEIDHYHEKKETVTDLSDP----------DSDSDSFYGSVERPVPINYTHNPAD---D   171
XP_015141266.1[G.gallus]         LRREIDHYHDKKETVTEFSDS----------GSDADSFYGSVERPIDIKYSHHSAD---N   209
NP_001139330.1[M.musculus]       LRREIGHFHEKKELPLDTSDS----------SSDTDSFYGAVERPIDISLSSYPMD---N   215
P78314[H.sapiens]                LRREIGHFHEKKDLPLDTSDS----------SSDTDSFYGAVERPVDISLSPYPTD---N   171
PNJ23454.1[P.abelii]             LRREIGHFHEKKDLPLDTSDS----------SSDTDSFYGAVERPVDISLSPYPTD---N   199
                                 :: ::                              *

XP_032222077.1[N.vectensis]      --------------PLFAPEFALAKALTLEKLSESQNSSSASSEPEYCPPPSFDTLPN--   235
XP_019631975.1[B.belcheri]       HYYEDPDEHLQEEEDYLQVLEDGPRVTPVGAEAA-GDVGA---FREMPLPPTPTSK---M   272
XP_032800705.1[P.marinus]        DEYDEDSGDDDDDGDYLQPDDYL--TTHLESGTG-GGLSPQPKPQQQPPPPQLLLASSPS   229
XP_013984441.1[S.salar]          NYMLEDEEDDEDEDDYEKPDSPP--TY-KGRLTV-P-------PPSYHPPPLPCQFRQES   219
XP_041443552.1[X.laevis]         SY----EDDDDEDDDYVKPDGAD--E---------N-------APTYPPPPVPRNPRNGN   209
XP_015141266.1[G.gallus]         ED----YDQEEDDESYLQPDTSD--IV-KDDFMV-L-------PPAYPPPPVPHVRKAAY   254
NP_001139330.1[M.musculus]       ED----YEHEDEDDSYLEPDSPG--PMKLEDALT-Y-------PPAYPPPPVPVPRKPAF   261
P78314[H.sapiens]                ED----YEHDDEDDSYLEPDSPE--PGRLEDALM-H-------PPAYPPPPVPTPRKPAF   217
PNJ23454.1[P.abelii]             ED----YEHDDEDDSYMEPDSPE--PGRLEDALM-H-------PPAYPPPPVPTPRKPAF   245
                                                                                  **

XP_032222077.1[N.vectensis]      -----------------GS--PPPPPARN-QSGK-ERAQ----------PIGSVPTPVMM   264
XP_019631975.1[B.belcheri]       ASPS-----KGWYGQEGAARPPVPLPKK--QSSFPTKPLPATPQEGTRSPKKALVPPHMR   325
XP_032800705.1[P.marinus]        PSPANKFTATTATTKRPALMPPKPPPKPPPQQPQ--QQQ---------------QPQQQ   271
XP_013984441.1[S.salar]          -----------MSGSCKGLPQPAPVPNRTLSSPLPKKMSPLLPR------------PKLG   256
XP_041443552.1[X.laevis]         D--SVKPRTMSDVG---LPYRPPPPPTPPLIKTLPHLSDQARKE----------SAIHQG   254
XP_015141266.1[G.gallus]         S---E-SRSHSFSGKTAGSTPPPPPPKR----SLPEIKTEDFFG----------VREPQ-   295
NP_001139330.1[M.musculus]       S---DLPRAHSFTSKSPSPLLPPPPPKR----GLPDTGSAPEDA-----------KDAL-   302
P78314[H.sapiens]                S---DMPRAHSFTSKGPGPLLPPPPPKH----GLPDVGLAAEDS----------KRDPL-   259
PNJ23454.1[P.abelii]             S---DMPRAHSFTSKGPGPLLPPPPPKH----GLPDVGLAAEDS----------KRDPL-   287
                                                      * * *

XP_032222077.1[N.vectensis]      GKSGKSSV-------DI------SAVILGKAALK---------PV------QDNSLPPLP   296
XP_019631975.1[B.belcheri]       GVSTSPPHQTE-SKGKVPS-------------------------------------PPWK   347
XP_032800705.1[P.marinus]        QPQQH--HHHQEL-------------------------GYFKRPIAGTA-----------   293
XP_013984441.1[S.salar]          E-VGHNHHGH----KDVPNKDSPERSVRGPKAPHPTHIASLQK----QMMMGKTSISTLP   307
XP_041443552.1[X.laevis]         FSCGPPPHKIEEFTPKLPPHKKTSFDLIGKEPPRREDLIGSSDSVPGYDLCGPTIKPPSP   314
XP_015141266.1[G.gallus]         LPCRAEP------NLKIQSS------------SRRPS-----EQ-------PP-PVPPLP   324
NP_001139330.1[M.musculus]       GLRRVEP------GLRVPAT------------PRRMS-----DP-------PMSNVPTVP   332
P78314[H.sapiens]                CPRRAEP------CPRVPAT------------PRRMS-----DP-------PLSTMPTAP   289
PNJ23454.1[P.abelii]             CPRRAEP------CPRVPAT------------PRRMS-----DP-------PLSTMPTAP   317


XP_032222077.1[N.vectensis]      KPR--------------------------------------------------------S   300
XP_019631975.1[B.belcheri]       ELKGPGVKKASEPINITSPGWTAGKTVGQRETGEGRGRDVKDEKKEKSGMARALAAEMTQ   407
XP_032800705.1[P.marinus]        ---IGPQQRSP------PLPWAAVAAAAAASTTTTT---------------TTATTTTT-   328
XP_013984441.1[S.salar]          GG----DHRDKKP-----------HAPVTLKSP--------------------ATLPICI   332
XP_041443552.1[X.laevis]         SLQLGARHVDPPAPPPVSYPKKNGSSCKPMTNPSTQ------------LISNTVSKELTD   362
XP_015141266.1[G.gallus]         LFKKPFCVKEPSSLPPEL--------------PL---------------SHVLTTTEGCE   355
NP_001139330.1[M.musculus]       NLRKHPCFRDSVNP--GLEPWTPGHGTSSVSSST---------------TMAVATSRNCD   375
P78314[H.sapiens]                GLRKPPCFRESASP--SPEPWTPGHGACSTSSAA---------------IMATATSRNCD   332
PNJ23454.1[P.abelii]             GLRKPPCFRESASPSPSPEPWTPGHGACSTSSAA---------------IMATATSRNCD   362


XP_032222077.1[N.vectensis]      KLRQSPRVHA-GSHPSSPSPPTKPPKPSVHKYASDPEL---------------ACLPQ--   342
XP_019631975.1[B.belcheri]       KFGSNWQAAGGTAKPRTPDKPVPAAKPGLHPK------------------PGLAPKPG--   447
XP_032800705.1[P.marinus]        ------------------------------------------------------------   328
XP_013984441.1[S.salar]          NLERQMGLNP-SP------PGCHGNTPSHIPSVPNYCH-STRP------TPDLPPQPSP-   377
XP_041443552.1[X.laevis]         KF-RMMPV----------PSPAPPPKPSFLSPVVKPSSSSNAPPPPPPVKPSIPPSTPPS   411
XP_015141266.1[G.gallus]         KL-KTLNLSP-RTP-----PPLPSNKPKLSQITEKTVE---NKVPREH------------   393
NP_001139330.1[M.musculus]       KL-KSFHLSS-RGPPTSEPPPVPANKPKFLKIA----E---EPSPREA------------   414
P78314[H.sapiens]                KL-KSFHLSP-RGPPTSEPPPVPANKPKFLKIA----E---EDPPREA------------   371
PNJ23454.1[P.abelii]             KL-KSFHLSP-RGPPTSQPPPVPANKPKFLKTA----E---EDPPREA------------   401


XP_032222077.1[N.vectensis]      ------QTSLPPANPALVDSSDSEEDDYL-KILPDEESTPN----HGNNNDEFIPRLSTL   391
XP_019631975.1[B.belcheri]       ------TSPKPGLAPKPGHPSRQGMKPLVPPHKPQVEVQGIMEDRYEHEEEEEPVEDPGI   501
XP_032800705.1[P.marinus]        ---TARGKA----------------------LMPAAT---------TTTITTTKAPPRPL   354
XP_013984441.1[S.salar]          ALNHKLGPYKPPLLTKPPCP--------------------------ATM---PKRPRGSA   408
XP_041443552.1[X.laevis]         VTIASISPPVPPIKPRMLNDQKAERIPQKPPPIPR---QP------GRKSESDKLEPSPK   462
XP_015141266.1[G.gallus]         ---GKPGLFVPPVLPKPP---------VPGH------QHS------VLKPRPEKPSCPQL   429
NP_001139330.1[M.musculus]       ---AKF-APVPPVAPRPP---------VQKMPMPEATVRP------AVLPRPENTPLPHL   455
P78314[H.sapiens]                ---AMPGLFVPPVAPRPP---------ALKLPVPEAMARP------AVLPRPEKPQLPHL   413
PNJ23454.1[P.abelii]             ---AMPGLFVSPVAPRPP---------ALKLPVPEATARP------AVLPRPEKPQLPHL   443
```

```
XP_032222077.1[N.vectensis]      ERLRPEGKSFRTPPDEKEQ-------------------------IHIDVLPATSVKLDIE   426
XP_019631975.1[B.belcheri]       RRTPPEGNSFSKKNFNENPES------------ETDD------VMP----ADVSDDVDVE   539
XP_032800705.1[P.marinus]        SRPQPEGESFHKLQID----------------SDSDDYIQPEDAMPSTIFLDTTSGDEAE   398
XP_013984441.1[S.salar]          QRSPPDGLSSQTSVDQVPASLRKNRDPSKYIDDDSDE-DYENVQLPDSVFVDITETSSVE   467
XP_041443552.1[X.laevis]         LRSPPDGQSFRGLSTEGPAHP--IKPKRKLTRNDSDE-DYEKVPLPVSVFVDTNDSVEVE   519
XP_015141266.1[G.gallus]         QRSPPDGQSFRSFSFEKPALP--SKPN--QVNDDSDD-DYEKVELPTSIFLNTSESLEVE   484
NP_001139330.1[M.musculus]       QRSPPDGQSFRGFSFEKARQP--SQAD--TGEEDSDE-DYEKVPLPNSVFVNTTESCEVE   510
P78314[H.sapiens]                QRSPPDGQSFRSFSFEKPRQP--SQAD--TGGDDSDE-DYEKVPLPNSVFVNTTESCEVE   468
PNJ23454.1[P.abelii]             QRSPPDGQSFRSFSFEKPRQP--LPAD--TGGDDSDE-DYEKVPLPNSVFINTTESCEVE   498
                                  *  *:* *      :                            :        .   . *

XP_032222077.1[N.vectensis]      RTKAIAM--LEGRNGMYILHKSRSGDIGQCLS--VCIEDRVRHFLVFYSKEEGGYALDRD   482
XP_019631975.1[B.belcheri]       K--AESCLRSGQVNGKYFLRPSKQDKGKMVLVVYDCAGQKCRKFKMYGLGSQLYLH--KG   595
XP_032800705.1[P.marinus]        WRMKNA----GLTHGLYCIRNSASSDQ-QVLVVWDGECRKLRNFKIFRAGPLVSLDVSDG   453
XP_013984441.1[S.salar]          KMFMESS--SSPQDGLYCFRNSASKTS-KVLVVWDVDLCKARNYRLFEEDSRVFLE---F   521
XP_041443552.1[X.laevis]         RIFKAASPGGTPQNGLFCIRNS-AKAG-KVLVVWDTATEKTRNYRIFEKDSKYFLE---A   574
XP_015141266.1[G.gallus]         RIFKATSPKGQPQNGLYCIRNSSTKAG-KVLVVWDQSAEKVRNYRIFEKDCKFYLD---A   540
NP_001139330.1[M.musculus]       RLFKATDPRGEPQDGLYCIRNSSTKSG-KVLVVWDESSNKVRNYRIFEKDSKFYLE---G   566
P78314[H.sapiens]                RLFKATSPRGEPQDGLYCIRNSSTKSG-KVLVVWDETSNKVRNYRIFEKDSKFYLE---G   524
PNJ23454.1[P.abelii]             RLFKATSPRGEPQDGLYCIRNSSTKSG-KVLVVWDETSNKVRNYRIFEKDSKFYLE---G   554
                                      :       .* : :: *        *        : *:: ::

XP_032222077.1[N.vectensis]      AQRFPTLEEVIRHHYTTPLPKQQSATLEQPYRWPH--  517
XP_019631975.1[B.belcheri]       APTFSSMSDLLRHYQYHNLPVTDGVEIRLTEPYQGH-  631
XP_032800705.1[P.marinus]        APHFKLLSGLLDHYRVAPLPRQSSLRLTQPLVV----  486
XP_013984441.1[S.salar]          EMTFASLSALVEHYHSHPLPNHDSLCLQQPYRYIMPR  558
XP_041443552.1[X.laevis]         ELLFPDVESLVEHYYTNKLPSHSTLVLQHAYGCSFPR  611
XP_015141266.1[G.gallus]         DIMFLNMGSLVEYYSTHVLPSHGSLILRCPYGYSKPR  577
NP_001139330.1[M.musculus]       EVLFASVGSMVEHYHTHVLPSHQSLLLRHPYGYAGPR  603
P78314[H.sapiens]                EVLFVSVGSMVEHYHTHVLPSHQSLLLRHPYGYTGPR  561
PNJ23454.1[P.abelii]             EVLFVSVGSMVEHYHTHVLPSHQSLLLRHPYGYTGPR  591
                                    *  :  :: ::    **      :
```
