# Supplementary material for: An Evolutionary Perspective on the Origin, Conservation and Binding Partner Acquisition of Tankyrases
Source: Biomolecules. 2022 Nov 15;12(11):1688. doi: 10.3390/biom12111688 (PMC9688111; doi:10.3390/biom12111688)
Supplement: Supplementary file 1 [file biomolecules-12-01688-s001.zip › biomolecules-1913719-supplementary-11.17 -done/Data_7/SH3BP5.pdf]

SH3BP5

Homo sapiens:

>sp|O60239|3BP5_HUMAN SH3 domain-binding protein 5 OS=Homo sapiens OX=9606 GN=SH3BP5 PE=1 SV=2
MDAALKRSRSEEPAEILPPARDEEEEEEEGMEQGLEEEEEVDPRIQGELEKLNQSTDDIN
RRETELEDARQKFRSVLVEATVKLDELVKKIGKAVEDSKPYWEARRVARQAQLEAQKATQ
DFQRATEVLRAAKETISLAEQRLLEDDKRQFDSAWQEMLNHATQRVMEAEQTKTRSELVH
KETAARYNAAMGRMRQLEKKLKRAINKSKPYFELKAKYYVQLEQLKKTVDDLQAKLTLAK
GEYKMALKNLEMISDEIHERRRSSAMGPRGCGVGAEGSSTSVEDLPGSKPEPDAISVASE
AFEDDSCSNFVSEDDSETQSVSSFSSGPTSPSEMPDQFPAVVRPGSLDLPSPVSLSEFGM
MFPVLGPRSECSGASSPECEVERGDRAEGAENKTSDKANNNRGLSSSSGSGGSSKSQSST
SPEGQALENRMKQLSLQCSKGRDGIIADIKMVQIG

Pongo abelii:

>XP_002814083.1 SH3 domain-binding protein 5 [Pongo abelii]
MDAALKRSRSEEPAELLPPARDEEEEEEEGMEQGLEEEEEVDPRIQGELEKLNQSTDDINRRETELEDAR
QKFRSVLVEATVKLDELVKKIGKAVEDSKPYWEARRVARQAQLEAQKATQDFQRATEVLRAAKETISLAE
QRLLEDDKRQFDSAWQEMLNHATQRVMEAEQTKTRSELVHKETAARYNAAMGRMRQLEKKLKRAINKSKP
YFELKAKYYVQLEQLKKTVDDLQAKLTLAKGEYKMALKNLEMISDEIHERRRSSAMGPRGCGVGAEGSST
SVEDLPGSKPEPDAISVASEAFEDDSCSNFVSEDDSETQSVSSFSSGPTSPSEMPDQFPAVVRPGSLDLP
SPVSLSEFGMMFPVLGPRSECSGASSPECEVERGDRAEGAENKTSDKANNNRGLSNSSGSGGSSKSQSSI
SPEGQALENRMKQLSLQCSKGRDGIIADIKMVQIG

Mus musculus:

>NP_036024.2 SH3 domain-binding protein 5 isoform 1 [Mus musculus]
MDTALKRSRSDEPAELPPPAREVEEKEEEEERMEQGLEEEEEEVDPRIQGELEKLNQSTDDINRRETELE
DARQKFRSVLVEATVKLDELAKKIGKAVEDSKPYWEARRVARQAQLEAQKATQDFQRATEVLRAAKETIS
LAEQRLLEDDKRQFDSAWQEMLNHATQRVMEAEQTKTRSELVHKETAARYNAAMGRMRQLEKKLKRAINK
SKPYFELKAKYYVQLEQLKKTVDDLQAKLALAKGEYKAALKSLERISDEIHERRRSNAMGPRGCGVGAEG
SIASVENLPVSKPEPDAISVASEAFEDDNCSNLVSEDDSETQSVSSFSSGPTSPSEMPDQFPAVARPGSL
DLPSPVSLSEFGMMFPILGPRSECSGASSPECEVERGDRAEGAENKMSDKANNNRVLGSTNGGSGRSRSQ
SSTSLESQALETRMKQLSLQCSKGRDGIIADIKMVQIG

Gallus gallus:

>NP_001026160.3 SH3 domain-binding protein 5 [Gallus gallus]
MEPGGRRGRAAEEAPRPEEEEVDPRIQGELEKLNQSTDVINRRETELEDARQKFRSVLVEATVKLDELVK
KIGKAVEDSKPYWEARRVARQAQLEAQRATQDFQRATEVLRAAKETISLAEQRLLEDDKRQFDSAWQEML
NHATQRVMEAEQTKTRSELVHKETAAKYNAAMGRMKQLEKKLKRAINKSKPYFELKAKYYVQLEQLKKTV
DDLQAKLSLAKGEYKTALKNLEMISDEIHERRRSAAMGPRGCGVGAEGSNTSVEDLSTSKPELDAVSMAS
EVFEDDNGSSFVSEEDSETQSVSSFSSGPTSPSEVPAPFPPATRPGTLDLPSPVSLSEFGAIFPILGPRS
ECSGASSPECEAERGDRAEGAENKASDRVNKNRSSNRSGSTEGLALDNRMKQLSLQCMKIKEGICAGRKA
AQIG

Xenopus laevis:

>XP_018122983.1 SH3 domain-binding protein 5 [Xenopus laevis]
METILIKNTGQQENEQLKEEEEEEVDPRIQGELEKLNQSTDDINRREIELEDARQKFRSVLTEATLKLDE
MVKKIGKAVEDSKPYWEARKVARQAQLEAQKATQDFQRATEVLRAAKETISLAEQRLLEDDKRQFDSAWQ
EMLNHATQRVMEEEQKKTRSELLHKETAAKYNAAMGRMKQLEKKLKRTINKSKPYFELKAKYYLQLEQLK
KTVDDLQAKLSLAKGEYKTALKNLEMISDEIHEKRRTSSMGPRGRGVGSEGNNSLEDLSACKLDSDNISM
TSEVFDDDNLSSLVSEEDSETQSMSSFSSVPTSPAEIPSPFPNVPRPGSLDLSSPLSLSDFGLISPILGP
RSECSGASSPECDLERGNRAEGSENAAQDRINNNSKSNSRKAMALPVLDNKIKQITLECTKTGNGTNTER
KKCSRSQSLASTLLVNAV

Salmo salar:

>XP_014056350.2 SH3 domain-binding protein 5 [Salmo salar]
MDNLEKENRSDEDSEYEEEEVDPRIQGELEKLNQSTDDINRCETQLEDARQKFRTVLVEATVKLDELVKK
IGKAVEESKPYWEARRVARQAQLEAQRATQDFQRATEVLRAAKETIALAEQRLLEEDTRQFDSAWQEMLN
HATQRVMEAEHTKTRSELVHKETAAKYTAAMGRMKQLEKKLKRTINKSKPYFELKAKYYLQLEHLKKNVD
ELQAKLSQAKEGYKTALRNLEVISDEIHERRRNSAMGPRGRGVGADGDSASVDDISSFKMESDGISMTSE
SFEDETCSSSAMSEEDSETRSTCSPQDLPSPCPSASSSSPAPSSSSSPAPSSRPCSLDLPSPVSLSDFGL
ISPVLGPRSECSGASSPECDLERGDRAEGAEGELDNTVNNNSTTVPNTNKTTGLEVRLSFLSLRRSRNDS
TNNTKRERQSRSQTPTQTVVLINGL

Petromyzon marinus:

>XP_032831288.1 SH3 domain-binding protein 5-like [Petromyzon marinus]
MVMTKMIKMMIMTMMMVVMTMMCLQVMKAEQDKGRSELVHKQTAERYSVTMVHMNQLERKLKRTIKKSRP
YYELKAKYYLELEHQKQRVEGLQEAVTATKGQYRAALDSLERISDEIHARRKASAVEEPRGRGVGAETTG
AFSMELGFFSMDGDSFSVASDACESDACSLMASKDDNYLESCSPCAGGAVPPAPLPTRPPALQLAASEPG
SGSRSGSSSGSGSRSSSGSGADPDSGFFSPVQDERLGPRSDCSGASSPECEGLPPLTGAGSSLPPSEAEA
EPAEKTRAAETGREGFCVDSVTPRGEEEEEDGSDAAASGASGTHTAIADGGVGGGERERDMRAAERREQE
EEEDGADSGEATAKESGDGQVAEGEGRSVSGTDGGAAMHEASPEEEPAAAWAVRAEDLSVGGVKLEQKDE
EVEDEGVEVEKVEVEEVEEDEEVDLQGDIGDRGGEGVAGAGFTAGALGASVRDVAL

Branchiostoma belcheri:

>KAI8492211.1 SH3 domain-binding protein 5 [Branchiostoma belcheri]
MSEQGQPETSSGDEGLDPRIQEELERLNAASNEINKLEAELDEARALFRQTLTESTQKLNLLSKKMGKSV
QKARPYYDAKKMAKQAQVEAQKAAVEYQRANGIHRAARETISLAEQRLFSDADEKRQFDSAWQEMLNHAT
MKVMEAEQMRSKSEHDHQQKAEAYNTANHKLQQLEKSLKKVIAKTKPYFDVKTSFELQLLQQKQNVEDLQ
KAVSASKVKYREALKNLESISDEIHRNRQLLKEPRSAGVGAEADRDDLPEINLDDVDSCSQYSDLDDGNS
LDTDSTTDDASMIAQTDDASLSRRDRGGSFTSHSSSVGESVSSSSAVGAACSQDVVEKTAEGGCTNEGTA
TESGQAVEGGSYTEGTAPAVQAVEGGLTEKVTAGDGDKAEDSMVVAGTVTCREEEQHRMEEIQMLTEGNS
QNTSQNADTTEIETDKTDKEDIDVHVQQEAVDDVEKSFEDVVEEGYKEDMSHNADVPTPVAIPGNVNAEE
VVEQQQVTPTEVVKAESESFPTLKNEGDRTNMPNVAVACDPSLKDEVKVSECREEVIEQKVGQDEVKFES
TEGSALENQEPKVAFEDVVVKETVEAIEESQTAGSVTGRNDEEDNAKREEGTEVRSVESQETSVPDTPVE
AVSNASIEEVSNTSTEEKPPEIRQTDKSSSVEKIRGRNDS

Drosophila melanogaster:

```
>NP_611010.4 parcas [Drosophila melanogaster]
MSSAEDGELDPQIQIELENLNSATDEINKLEIELEEANSTFRILLNESTRRLKVSSKKLGNCIEKARPYY
EALDKAREAQIECQKAAVKFQRANEIHAAAKETVALAEQRFMSNSHEWQFDNAWQEMLNHATQKVMDAET
QKADCHAEHQRLTKLFNAAEQKLQQLEDRFRRSINKSRPYFEEKQVCQDQLQTQKNRIQELQQQVAGAKS
TYSTALRNLERISEDIHRQRGDFPTPPGPREPGVGAELNSPTSSALPSLPDFQLELEKCDYPSIAGSQMS
LGAKTPQAAAETEDEEDACDYDETGAGELRGVVDERDLEALRQKVKILAVRPIEGGDGQQQNDVWEHELK
ATVDKLDHLMMLKETAKRQQTNRLKSTEQRPDSLGAEALKRHCDVVEVKVTSCATTASLPVTPHHQLNHL
APPTPIKKLQQQLAPLPSVNVSMRELPLLARLSNELLDRSSAAFGGVRKTLRRRSLE
```

Hydra vulgaris:

```
>XP_047136805.1 SH3 domain-binding protein 5-like isoform X1 [Hydra vulgaris]
MYDSLVLEMISKENEARQKSKYNNVEENEEEEELDPRIKCELERLNAATDGINHLETEFGEAQALFQQLM
QDAAFNLKVMHQKLGKAVDKSRPYYEALKCAKISHSELHEAALKFEQAQTRHFNAKKLVTDTERIVFSST
DRKLDPALQEMLNQATVEVMEAATQSQKAWEVHRNASVEFEKKNKVALDLYDSLKKHIVKSRPYFDLKSS
FNQQLDLQKTRLEKVQEGLQVSKNEYAKILHNLELISDDIHQSREMKRKVCSEMRGEGVGSESQSNNNVL
VYKDVEKQEEKNNSNLYLNQRDDNIGDKENLDKNLSQVSGQTELVNLGESLSQSSDLFLNQNDENIGCNE
NLGESLSQVSGPTELVKIRKQVQSLLETSAEQKSKNRLSQTERLINVNKTLIENCFDKSYDVITSHSIPF
SHPQEEFVDSSINEYHNISKFLKKSFILPEFEIIPRRHTISGEKDSFQLLNHNKKLFCKSSSALI
```

Nematostella vectensis:

```
>XP_001637874.3 SH3 domain-binding protein 5-like [Nematostella vectensis]
MSSLTLSTDDTKLSHKFSDDEVDSEESLDPRVKGELERLNSATTEINCLEKALEEMHALFRQTLTESAFV
LKSHANRYGKSINQSRPYYEALERSKKAQIATQKAALKYERACCTHQAAKEMVARAEEKLVSTSKEHKVL
DPTWQDMLNKAVMKVMESEQEKCHAEKEHLETAHLFTKAGKELQDLKTKLKTAINKSRVYFDLKTKYMDQ
LEAHKKEVISLQGKLKVAKKDYSMSLRKLEGISDEIHEVRQSRISLCLPSRESGVGAECPGHQAPSEGRT
ARDWDEDGMMVLPDGRCDDSDKSTTDSTRGDTSESIPDAVCNDTSKPIPDGVCNDTSKSIPDAVCNDTSK
SIPDAVCDDTSKSIPDPVCDNLDESPKISTMDRASDVSPASGACNDTAESALDAVCGDLGELPSEGTIDD
FSEST
```

Amphimedon queenslandica:

No homolog found.

Alignment

```
O60239[H.sapiens]                MDAALKRSRSEEPAEILPPARDEEE-------EEEEGMEQGLEE-EEEVDPRIQGELEKL   52
XP_002814083.1[P.abelii]         MDAALKRSRSEEPAELLPPARDEEE-------EEEEGMEQGLEE-EEEVDPRIQGELEKL   52
NP_036024.2[M.musculus]          MDTALKRSRSDEPAELPPPAREVEE-----KEEEEERMEQGLEEEEEEVDPRIQGELEKL   55
NP_001026160.3[G.gallus]         ---------------MEPG------------GRRGRAAEEAPRPEEEEVDPRIQGELEKL   33
XP_018122983.1[X.laevis]         ME-------------------TILI-----KNTGQQENEQLKEEEEEEVDPRIQGELEKL   36
XP_014056350.2[S.salar]          -----------------------MD-----NLEKENRSDEDSEYEEEEVDPRIQGELEKL   32
XP_032831288.1[P.marinus]        ------------------------------------------------------------   0
KAI8492211.1[B.belcheri]         ---------------------------------MSEQGQPETSSGDEGLDPRIQEELERL   27
NP_611010.4[D.melanogaster]      ----------------------------------------MSSAEDGELDPQIQIELENL   20
XP_047136805.1[H.vulgaris]       ---------------MYDSLVLEMISKENEARQKSKYNNVEENEEEEELDPRIKCELERL   45
XP_001637874.3[N.vectensis]      ---------------MSS-LTLSTD-----DTKLSHKFSDDEVDSEESLDPRVKGELERL   39


O60239[H.sapiens]                NQSTDDINRRETELEDARQKFRSVLVEATVKLDELVKKIGKAVEDSKPYWEARRVARQAQ   112
XP_002814083.1[P.abelii]         NQSTDDINRRETELEDARQKFRSVLVEATVKLDELVKKIGKAVEDSKPYWEARRVARQAQ   112
NP_036024.2[M.musculus]          NQSTDDINRRETELEDARQKFRSVLVEATVKLDELAKKIGKAVEDSKPYWEARRVARQAQ   115
NP_001026160.3[G.gallus]         NQSTDVINRRETELEDARQKFRSVLVEATVKLDELVKKIGKAVEDSKPYWEARRVARQAQ   93
XP_018122983.1[X.laevis]         NQSTDDINRREIELEDARQKFRSVLTEATLKLDEMVKKIGKAVEDSKPYWEARKVARQAQ   96
XP_014056350.2[S.salar]          NQSTDDINRCETQLEDARQKFRTVLVEATVKLDELVKKIGKAVEESKPYWEARRVARQAQ   92
XP_032831288.1[P.marinus]        ------------------------------------------------------------   0
KAI8492211.1[B.belcheri]         NAASNEINKLEAELDEARALFRQTLTESTQKLNLLSKKMGKSVQKARPYYDAKKMAKQAQ   87
NP_611010.4[D.melanogaster]      NSATDEINKLEIELEEANSTFRILLNESTRRLKVSSKKLGNCIEKARPYYEALDKAREAQ   80
XP_047136805.1[H.vulgaris]       NAATDGINHLETEFGEAQALFQQLMQDAAFNLKVMHQKLGKAVDKSRPYYEALKCAKISH   105
XP_001637874.3[N.vectensis]      NSATTEINCLEKALEEMHALFRQTLTESAFVLKSHANRYGKSINQSRPYYEALERSKKAQ   99


O60239[H.sapiens]                LEAQKATQDFQRATEVLRAAKETISLAEQRLLE--DDKRQFDSAWQEMLNHATQRVMEAE   170
XP_002814083.1[P.abelii]         LEAQKATQDFQRATEVLRAAKETISLAEQRLLE--DDKRQFDSAWQEMLNHATQRVMEAE   170
NP_036024.2[M.musculus]          LEAQKATQDFQRATEVLRAAKETISLAEQRLLE--DDKRQFDSAWQEMLNHATQRVMEAE   173
NP_001026160.3[G.gallus]         LEAQRATQDFQRATEVLRAAKETISLAEQRLLE--DDKRQFDSAWQEMLNHATQRVMEAE   151
XP_018122983.1[X.laevis]         LEAQKATQDFQRATEVLRAAKETISLAEQRLLE--DDKRQFDSAWQEMLNHATQRVMEEE   154
XP_014056350.2[S.salar]          LEAQRATQDFQRATEVLRAAKETIALAEQRLLE--EDTRQFDSAWQEMLNHATQRVMEAE   150
XP_032831288.1[P.marinus]        ------------------------------MVMTKMIKMMIMTMMMVVMTMMCLQVMKAE   30
KAI8492211.1[B.belcheri]         VEAQKAAVEYQRANGIHRAARETISLAEQRLFSDADEKRQFDSAWQEMLNHATMKVMEAE   147
NP_611010.4[D.melanogaster]      IECQKAAVKFQRANEIHAAAKETVALAEQRFMSNSH-EWQFDNAWQEMLNHATQKVMDAE   139
XP_047136805.1[H.vulgaris]       SELHEAALKFEQAQTRHFNAKKLVTDTERIVFS--STDRKLDPALQEMLNQATVEVMEAA   163
XP_001637874.3[N.vectensis]      IATQKAALKYERACCTHQAAKEMVARAEEKLVSTSKEHKVLDPTWQDMLNKAVMKVMESE   159
                                                               ..        :      ::.    .**.

O60239[H.sapiens]                QTKTRSELVHKETAARYNAAMGRMRQLEKKLKRAINKSKPYFELKAKYYVQLEQLKKTVD   230
XP_002814083.1[P.abelii]         QTKTRSELVHKETAARYNAAMGRMRQLEKKLKRAINKSKPYFELKAKYYVQLEQLKKTVD   230
NP_036024.2[M.musculus]          QTKTRSELVHKETAARYNAAMGRMRQLEKKLKRAINKSKPYFELKAKYYVQLEQLKKTVD   233
NP_001026160.3[G.gallus]         QTKTRSELVHKETAAKYNAAMGRMKQLEKKLKRAINKSKPYFELKAKYYVQLEQLKKTVD   211
XP_018122983.1[X.laevis]         QKKTRSELLHKETAAKYNAAMGRMKQLEKKLKRTINKSKPYFELKAKYYLQLEQLKKTVD   214
XP_014056350.2[S.salar]          HTKTRSELVHKETAAKYTAAMGRMKQLEKKLKRTINKSKPYFELKAKYYLQLEHLKKNVD   210
XP_032831288.1[P.marinus]        QDKGRSELVHKQTAERYSVTMVHMNQLERKLKRTIKKSRPYYELKAKYYLELEHQKQRVE   90
KAI8492211.1[B.belcheri]         QMRSKSEHDHQQKAEAYNTANHKLQQLEKSLKKVIAKTKPYFDVKTSFELQLLQQKQNVE   207
NP_611010.4[D.melanogaster]      TQKADCHAEHQRLTKLFNAAEQKLQQLEDRFRRSINKSRPYFEEKQVCQDQLQTQKNRIQ   199
XP_047136805.1[H.vulgaris]       TQSQKAWEVHRNASVEFEKKNKVALDLYDSLKKHIVKSRPYFDLKSSFNQQLDLQKTRLE   223
XP_001637874.3[N.vectensis]      QEKCHAEKEHLETAHLFTKAGKELQDLKTKLKTAINKSRVYFDLKTKYMDQLEAHKKEVI   219
                                      .   * . :  :        :*   ::  * *:: *:: *     :*   *  :

O60239[H.sapiens]                DLQAKLTLAKGEYKMALKNLEMISDEIHERRRSS---AMGPRGCGVGAEGSSTSVE---D   284
XP_002814083.1[P.abelii]         DLQAKLTLAKGEYKMALKNLEMISDEIHERRRSS---AMGPRGCGVGAEGSSTSVE---D   284
NP_036024.2[M.musculus]          DLQAKLALAKGEYKAALKSLERISDEIHERRRSN---AMGPRGCGVGAEGSIASVE---N   287
NP_001026160.3[G.gallus]         DLQAKLSLAKGEYKTALKNLEMISDEIHERRRSA---AMGPRGCGVGAEGSNTSVE---D   265
XP_018122983.1[X.laevis]         DLQAKLSLAKGEYKTALKNLEMISDEIHEKRRTS---SMGPRGRGVGSEGNN-SLE---D   267
XP_014056350.2[S.salar]          ELQAKLSQAKEGYKTALRNLEVISDEIHERRRNS---AMGPRGRGVGADGDSASVD---D   264
XP_032831288.1[P.marinus]        GLQEAVTATKGQYRAALDSLERISDEIHARRKA--SAVEEPRGRGVGAETTGAFSM---E   145
KAI8492211.1[B.belcheri]         DLQKAVSASKVKYREALKNLESISDEIHRNRQ-L---LKEPRSAGVGAEADRDDLP---E   260
NP_611010.4[D.melanogaster]      ELQQQVAGAKSTYSTALRNLERISEDIHRQRGD-FPTPPGPREPGVGAELNSPTSSALPS   258
XP_047136805.1[H.vulgaris]       KVQEGLQVSKNEYAKILHNLELISDDIHQSREMKRKVCSEMRGEGVGSESQSNNNVLVYK   283
XP_001637874.3[N.vectensis]      SLQGKLKVAKKDYSMSLRKLEGISDEIHEVRQSRISLCLPSRESGVGAECPGHQAP---S   276
                                  :*  :  :*  *   * .** **::**  *          *  ***::          .

O60239[H.sapiens]                LPGSKPEP-----------DAISVASEAFEDDSCSNF---VSEDDSET--QSVSSFSSGP   328
XP_002814083.1[P.abelii]         LPGSKPEP-----------DAISVASEAFEDDSCSNF---VSEDDSET--QSVSSFSSGP   328
NP_036024.2[M.musculus]          LPVSKPEP-----------DAISVASEAFEDDNCSNL---VSEDDSET--QSVSSFSSGP   331
NP_001026160.3[G.gallus]         LSTSKPEL-----------DAVSMASEVFEDDNGSSF---VSEEDSET--QSVSSFSSGP   309
XP_018122983.1[X.laevis]         LSACKLDS-----------DNISMTSEVFDDDNLSSL---VSEEDSET--QSMSSFSSVP   311
XP_014056350.2[S.salar]          ISSFKMES-----------DGISMTSESFEDETCSSS--AMSEEDSET--RSTCSPQDLP   309
XP_032831288.1[P.marinus]        LGFFSMDG-----------DSFSVASDACESDACSLM---ASKDDNYLESCSPCAGGAVP   191
KAI8492211.1[B.belcheri]         INLDDVDS--CSQY-SDLDDGNSLDTDSTTDDA-SMI--AQTDDASLSRRDRGGSFTSHS   314
NP_611010.4[D.melanogaster]      LPDFQLELEKCDYP-SIAGSQMSLGAKT--PQAA-----AETEDE-----EDACDYD---   302
XP_047136805.1[H.vulgaris]       DVEKQEEKNNSNLYLNQRDDNIG--DKENLDKNLSQ----VSGQTELV------------   325
XP_001637874.3[N.vectensis]      EGRTARDW-------D--EDGMMVLPDGRCDDSDKSTTDSTRGDTSESIPDAVCNDTSKP   327
                                       :            .      .    .           :

O60239[H.sapiens]                -T----------SPSEM------PDQFPAVVRPGSLDLPSPV------------SLSEFG   359
XP_002814083.1[P.abelii]         -T----------SPSEM------PDQFPAVVRPGSLDLPSPV------------SLSEFG   359
NP_036024.2[M.musculus]          -T----------SPSEM------PDQFPAVARPGSLDLPSPV------------SLSEFG   362
NP_001026160.3[G.gallus]         -T----------SPSEV------PAPFPPATRPGTLDLPSPV------------SLSEFG   340
XP_018122983.1[X.laevis]         -T----------SPAEI------PSPFPNVPRPGSLDLSSPL------------SLSDFG   342
XP_014056350.2[S.salar]          -SPCPSASSSSPAPSSS------S-SPAPSSRPCSLDLPSPV------------SLSDFG   349
XP_032831288.1[P.marinus]        PAPLPTR------P---------PALQLAASEPGSGSRSGSSSGSGSRSSSGSGADPDSG   236
KAI8492211.1[B.belcheri]         -S----------SVGES------VSSSSAVGAACSQDVVEKT------------A-----   340
NP_611010.4[D.melanogaster]      ------------------------------------------------------------   302
XP_047136805.1[H.vulgaris]       ------------NLGES------------------LSQSSDL------------------   337
XP_001637874.3[N.vectensis]      -IPDGVCNDTSKSIPDAVCNDTSKSIPDAVCDDTSKSIPDPVC----DNLDESPKISTMD   382


O60239[H.sapiens]                MMFP----VLGPRSECSGAS----SPECEVERGDRAEGAENKTSDKAN------------   399
XP_002814083.1[P.abelii]         MMFP----VLGPRSECSGAS----SPECEVERGDRAEGAENKTSDKAN------------   399
NP_036024.2[M.musculus]          MMFP----ILGPRSECSGAS----SPECEVERGDRAEGAENKMSDKAN------------   402
NP_001026160.3[G.gallus]         AIFP----ILGPRSECSGAS----SPECEAERGDRAEGAENKASDRVN------------   380
XP_018122983.1[X.laevis]         LISP----ILGPRSECSGAS----SPECDLERGNRAEGSENAAQDRIN------------   382
XP_014056350.2[S.salar]          LISP----VLGPRSECSGAS----SPECDLERGDRAEGAEGELDNTVN------------   389
XP_032831288.1[P.marinus]        FFSPVQDERLGPRSDCSGAS----SPECEGLPPLTGAGSSLPPSEAEAEPAEKTRAAETG   292
KAI8492211.1[B.belcheri]         ------------EGGCTNEGTA-TESGQAVEGGSYTEGTAPAVQAVE-------------   374
NP_611010.4[D.melanogaster]      ------------------------ETGAGELRGVVDERDLEALRQKVK------------   326
XP_047136805.1[H.vulgaris]       -FLN----QNDENIGCNENLGESLSQVSGPT-------ELVKIRKQVQ------------   373
XP_001637874.3[N.vectensis]      RASD----VSPASGACNDTAESALDAVCGDLGELPSEGTIDDFSEST-------------   425
                                                         .

O60239[H.sapiens]                -NNRGLSSSSGSGGSSKSQSSTSPEG-----QALENRMKQLSLQCSKGRDGI--IAD---   448
XP_002814083.1[P.abelii]         -NNRGLSNSSGSGGSSKSQSSISPEG-----QALENRMKQLSLQCSKGRDGI--IAD---   448
NP_036024.2[M.musculus]          -NNRVLGSTNGGSGRSRSQSSTSLES-----QALETRMKQLSLQCSKGRDGI--IAD---   451
NP_001026160.3[G.gallus]         -KNRSSNR------------SGSTEG-----LALDNRMKQLSLQCMKIKEGI--CAG---   417
XP_018122983.1[X.laevis]         -NNSKSNS------------RKAMAL-----PVLDNKIKQITLECTKTGNGT--NTE---   419
XP_014056350.2[S.salar]          -NNSTTVPNTNK-------------T-----TGLEVRLSFLSLRRSRNDSTN--NTK---   425
XP_032831288.1[P.marinus]        REGFCVDSVTPRGEEEEEDGSDAAASGA---SGT-----HTAI-ADGG-------VGGGE   336
```

```
KAI8492211.1[B.belcheri]         -GG-LTEKVTAGDGDKAED-SMVVAGTVTCREEEQHRMEEIQMLTEGNSQNTSQNADTTE   431
NP_611010.4[D.melanogaster]      --ILAVRPIEGGDGQQQNDVWE---HEL---KATVDKLDHLMMLKETAKRQQTNRLKSTE   378
XP_047136805.1[H.vulgaris]       -SL--L------------------ET--SAEQKSKNRLSQTERLINVNKTLIENCFD---   407
XP_001637874.3[N.vectensis]      ------------------------------------------------------------   425

O60239[H.sapiens]                IKM-----VQIG------------------------------------------------   455
XP_002814083.1[P.abelii]         IKM-----VQIG------------------------------------------------   455
NP_036024.2[M.musculus]          IKM-----VQIG------------------------------------------------   458
NP_001026160.3[G.gallus]         RKA-----AQIG------------------------------------------------   424
XP_018122983.1[X.laevis]         RKK-----CSRS-QSLA----------------------------------S-T-LLVNA   437
XP_014056350.2[S.salar]          RER-----QSRS-QTPT---------------------------------QT-V-VLING   444
XP_032831288.1[P.marinus]        RERDMRAAERRE-QEEEEDGADSGEATAKESGDGQVAEGEGRSVSG-----------TDG   384
KAI8492211.1[B.belcheri]         IETDKTDKEDID-VHVQQEAVDDVEKSF----EDVVEEGYKEDMSHNADVPT-P-VAIPG   484
NP_611010.4[D.melanogaster]      QRP-----DSLG-AEALKRHC------------DVVEVKVTS-CATTASLPVTPHHQLNH   419
XP_047136805.1[H.vulgaris]       KSYDVITSHSIPFSHPQEEFVDSSINEY----HN-----------------------ISK   440
XP_001637874.3[N.vectensis]      ------------------------------------------------------------   425

O60239[H.sapiens]                ------------------------------------------------------------   455
XP_002814083.1[P.abelii]         ------------------------------------------------------------   455
NP_036024.2[M.musculus]          ------------------------------------------------------------   458
NP_001026160.3[G.gallus]         ------------------------------------------------------------   424
XP_018122983.1[X.laevis]         V-----------------------------------------------------------   438
XP_014056350.2[S.salar]          L-----------------------------------------------------------   445
XP_032831288.1[P.marinus]        GAAMHEASPEEE---PAAA----------------------------WAVRAEDLSVGGV   413
KAI8492211.1[B.belcheri]         NVNAEE-VVEQQQVTPTEVVKAESESFPTLKNEGDRTNMPNVAVACDPSLKDE-VKVSEC   542
NP_611010.4[D.melanogaster]      LAPPTPIKKLQQQLAPLPSVNVSMRELPLLARLSN------------ELLDRSSAAFGGV   467
XP_047136805.1[H.vulgaris]       FLKKSFILPEFEIIPRRHTISGEKDSFQLLNHNKK--------LFCKSS-----------   481
XP_001637874.3[N.vectensis]      ------------------------------------------------------------   425

O60239[H.sapiens]                ------------------------------------------------------------   455
XP_002814083.1[P.abelii]         ------------------------------------------------------------   455
NP_036024.2[M.musculus]          ------------------------------------------------------------   458
NP_001026160.3[G.gallus]         ------------------------------------------------------------   424
XP_018122983.1[X.laevis]         ------------------------------------------------------------   438
XP_014056350.2[S.salar]          ------------------------------------------------------------   445
XP_032831288.1[P.marinus]        KLEQKDEEVEDEGVEVE-----------------KVEVEE-VEEDEEVDLQGDIGDRGGE   455
KAI8492211.1[B.belcheri]         REEVIEQKVGQDEVKFESTEGSALENQEPKVAFEDVVVKETVEAIEESQTAGSVTGRNDE   602
NP_611010.4[D.melanogaster]      RKTLRRRS---------------LE-----------------------------------   477
XP_047136805.1[H.vulgaris]       -SALI-------------------------------------------------------   485
XP_001637874.3[N.vectensis]      ------------------------------------------------------------   425

O60239[H.sapiens]                ------------------------------------------------------------   455
XP_002814083.1[P.abelii]         ------------------------------------------------------------   455
NP_036024.2[M.musculus]          ------------------------------------------------------------   458
NP_001026160.3[G.gallus]         ------------------------------------------------------------   424
XP_018122983.1[X.laevis]         ------------------------------------------------------------   438
XP_014056350.2[S.salar]          ------------------------------------------------------------   445
XP_032831288.1[P.marinus]        GVAGAGFTAGALGASVRDVAL---------------------------------------   476
KAI8492211.1[B.belcheri]         EDN----AKREEGTEVRSVESQETSVPDTPVEAVSNASIEEVSNTSTEEKPPEIRQTDKS   658
NP_611010.4[D.melanogaster]      ------------------------------------------------------------   477
XP_047136805.1[H.vulgaris]       ------------------------------------------------------------   485
XP_001637874.3[N.vectensis]      ------------------------------------------------------------   425

O60239[H.sapiens]                ------------   455
XP_002814083.1[P.abelii]         ------------   455
NP_036024.2[M.musculus]          ------------   458
NP_001026160.3[G.gallus]         ------------   424
XP_018122983.1[X.laevis]         ------------   438
XP_014056350.2[S.salar]          ------------   445
XP_032831288.1[P.marinus]        ------------   476
KAI8492211.1[B.belcheri]         SSVEKIRGRNDS   670
NP_611010.4[D.melanogaster]      ------------   477
XP_047136805.1[H.vulgaris]       ------------   485
XP_001637874.3[N.vectensis]      ------------   425
```
