# Supplementary material for: An Evolutionary Perspective on the Origin, Conservation and Binding Partner Acquisition of Tankyrases
Source: Biomolecules. 2022 Nov 15;12(11):1688. doi: 10.3390/biom12111688 (PMC9688111; doi:10.3390/biom12111688)
Supplement: Supplementary file 1 [file biomolecules-12-01688-s001.zip › biomolecules-1913719-supplementary-11.17 -done/Data_7/Sox9.pdf]

SOX9

Homo sapiens:

>NP_000337.1 transcription factor SOX-9 [Homo sapiens]
MNLLDPFMKMTDEQEKGLSGAPSPTMSEDSAGSPCPSGSGSDTENTRPQENTFPKGEPDLKKESEEDKFPVCIREAVSQVLKGYDWTLVPMPVRVNGSSKNKPHVKRPMNAFMVWAQAARRKLADQYPHLHNAELSKTLGKLWRLLNESEKRPFV
EEAERLRVQHKKDHPDYKYQPRRRKSVKNGQAEAEEATEQTHISPNAIFKALQADSPHSSSGMSEVHSPGEHSGQSQGPPTPPTTPKTDVQPGKADLKREGRPLPEGGRQPPIDFRDVDIGELSSDVISNIETFDVNEFDQYLPPNGHPGVPATH
GQVTYTGSYGISSTAATPASAGHVWMSKQQAPPPPPQQPPQAPPAPQAPPQPQAAPPQQPAAPPQQPQAHTLTTLSSEPGQSQRTHIKTEQLSPSHYSEQQQHSPQQIAYSPFNLPHYSPSYPPITRSQYDYTDHQNSSSYYSHAAGQGTGLYST
FTYMNPAQRPMYTPIADTSGVPSIPQTHSPQHWEQPVYTQLTRP

Pongo abelii:

>XP_009250264.1 transcription factor SOX-9 [Pongo abelii]
MNLLDPFMKMTDEQEKGLSGAPSPTMSEDSAGSPCPSGSGSDTENTRPQENTFPKGEPDLKKESEEDKFPVCIREAVSQVLKGYDWTLVPMPVRVNGSSKNKPHVKRPMNAFMVWAQAARRKLADQYPHLHNAELSKTLGKLWRLLNESEKRPFV
EEAERLRVQHKKDHPDYKYQPRRRKSVKNGQAEAEEATEQTHISPNAIFKALQADSPHSSSGMSEVHSPGEHSGQSQGPPTPPTTPKTDVQPGKADLKREGRPLPEGGRQPPIDFRDVDIGELSSDVISNIETFDVNEFDQYLPPNGHPGVPATH
GQVTYTGSYGISSTAATPAGAGHVWMSKQQAPPPPPQQPPQAPPAPQAPPQPQAAPPQQPAAPPQQPQAHTLTTLSSEPGQSQRTHIKTEQLSPSHYSEQQQHSPQQIAYSPFNLPHYSPSYPPITRSQYDYTDHQNSSSYYSHAAGQGTGLYST
FTYMNPAQRPMYTPIADTSGVPSIPQTHSPQHWEQPVYTQLTRP

Mus musculus:

>NP_035578.3 transcription factor SOX-9 [Mus musculus]
MNLLDPFMKMTDEQEKGLSGAPSPTMSEDSAGSPCPSGSGSDTENTRPQENTFPKGEPDLKKESEEDKFPVCIREAVSQVLKGYDWTLVPMPVRVNGSSKNKPHVKRPMNAFMVWAQAARRKLADQYPHLHNAELSKTLGKLWRLLNESEKRPFV
EEAERLRVQHKKDHPDYKYQPRRRKSVKNGQAEAEEATEQTHISPNAIFKALQADSPHSSSGMSEVHSPGEHSGQSQGPPTPPTTPKTDVQAGKVDLKREGRPLAEGGRQPPIDFRDVDIGELSSDVISNIETFDVNEFDQYLPPNGHPGVPATH
GQVTYTGSYGISSTAPTPATAGHVWMSKQQAPPPPPQQPPQAPQAPQAPPQQQAPPQQPQAPQQQQAHTLTTLSSEPGQSQRTHIKTEQLSPSHYSEQQQHSPQQISYSPFNLPHYSPSYPPITRSQYDYADHQNSGSYYSHAAGQGSGLYSTFT
YMNPAQRPMYTPIADTSGVPSIPQTHSPQHWEQPVYTQLTRP

Gallus gallus:

>NP_989612.1 transcription factor SOX-9 [Gallus gallus]
MNLLDPFMKMTEEQDKCISDAPSPTMSDDSAGSPCPSGSGSDTENTRPQENTFPKGDPDLKKESDEDKFPVCIREAVSQVLKGYDWTLVPMPVRVNGSSKNKPHVKRPMNAFMVWAQAARRKLADQYPHLHNAELSKTLGKLWRLLNESEKRPFV
EEAERLRVQHKKDHPDYKYQPRRRKSVKNGQSEQEEGSEQTHISPNAIFKALQADSPQSSSSISEVHSPGEHSGQSQGPPTPPTTPKTDAQQPGKQDLKREGRPLAEGGRQPPHIDFRDVDIGELSSDVISNIETFDVNEFDQYLPPNGHPGVPA
THGQVTTYSGTYGISSSASSPAGAGHAWMAKQQPQPPQPPAQPPAQHTLPALSGEQGPAQQRPHIKTEQLSPSHYSEQQQHSPQQQQQQQQQLGYGSFNLQHYGSSYPPITRSQYDYTEHQNSGSYYSHAAGQSGGLYSTFTYMNPTQRPMYTPI
ADTSGVPSIPQTHSPQHWEQPVYTQLTRP

Xenopus laevis:

>NP_001087942.1 transcription factor Sox-9-B [Xenopus laevis]
MNLLDPFMKMSEEQDKCLSGAPSPTMSEDSAGSPCPSGSGSDTENTRPQENTFPKGDQEMKKETEDEKFPVCIREAVSQVLKGYDWTLVPMPVRVNGSSKNKPHVKRPMNAFMVWAQAARRKLADQYPHLHNAELSKTLGKLWRLLNEGEKRPFV
EEAERLRVQHKKDHPDYKYQPRRRKSVKNGQSEQDDSAEQTHISPNAIFKALQADSPHSTSSMSEVHSPGEHSGQSQGPPTPPTTPKTDVQPGKPDLKREGRPLQESGRQPPHIDFRDVDIGELSSEVISTIETFDVNEFDQYLPPNGHPGVGSA
QAPYTGSYGISSTPSATTGAGSAWMSKQQQQPQQHSLSTINSEQSQSQQRTHIKTEQLSPSHYSDQQQQHSPQQLNYSSFNLQHYSSSYPTITRAQYDYTEHQGSNSYYTHASGQNSGLYSNFTYMNPSQRPMYTPIADTTGVPSIPQTHSPQHW
EQPVYTQLTRP

Salmo salar:

>XP_014034661.1 transcription factor Sox-9-A-like [Salmo salar]
MNLLDPFLKMTDEQDKCLSDAPSPSMSEDSAGSPCPSGSGSDTENTRPSENGLLMGPDGPLVEFKKDDDDKFPVCIRDAVSQVLKGYDWTLVPMPVRVNGSSKNKPHVKRPMNAFMVWAQAARRKLADQYPHLHNAELSKTLGKLWRLLNEGEKR
PFVEEAERLRVQHKKDHPDYKYQPRRRKSMKNGQSESEDGSEQTHISPGAIFKALQQADSPASSIGEVHSPGEHSGQSQGPPTPPTTPKTDIQAGKVDLKREGRPLQEGTGRQLNIDFRDVDIGELSSDVISHIDTFDVNEFDQYLPPNGHPGAP
GTAAGQVSYNGTYGISSSAVSQAAGGTAGHSWMTKSQSQQQHSLTTLGSGGEQGQNQRTPTHIKTEQLSPSHYNDQQNSSPPQHVTYGSFNLQHYSSSSYPSITRAQYDFSDHQGGTNSYYSHAAGGQGSGLYSFSSYMSPSQRPMYTPIADTTG
VPSVPQTHSPQQHWDQQPVYTQLSRP

Petromyzon marinus:

>XP_032830402.1 transcription factor Sox-9-A [Petromyzon marinus]
MMSDEQHDKHMSDVPSPDMSENCSVGSPADSLAGSDSDSQSCGSKRCSDREDGAGASGLLLAGGPGGVLDGGVVFGLGRDAGSGKKMDEDDKFPACIREAVSQVLKGYDWTLVPMPVRVNGSSKSKPHVKRPMNAFMVWAQAARRKLADQYPHLH
NAELSKTLGKLWRLLSENEKRPFVEEAERLRVQHKKDHPDYKYQPRRRKSGKNGQSESDSSGEQTHITTNAIYKALQADSPSADVHSPGEHSGQSQGPPTPPTTPKTDVQSNKLDIKREGRPLQEGGRQQIDFSNVDIRELSREVISNMESFDVN
EFDQYLPPNGHPGHGQSVAASYGGTGYSINGHAWLSKQQQQQQQQQQHTLSSPPPPPPAISSPEQRAHVKTEQLSPSHYSDQQQQQQPQQQQQHSPQQQQQQQQQQPQQAQQAQQQVQQQQQLGGYSPFSIQHYGAAVVPAISRSQYSYADHH
HHHHHHQSSAAAAAYYSGHSAGQTAGLYSGFSYMGPSQRPSYTPIADATGVPSIPQPHSPPSWEQPVYTQLTRP

Branchiostoma belcheri:

>XP_019645392.1 PREDICTED: transcription factor Sox-10-like [Branchiostoma belcheri]
MSETETPVEVKEEPAELESLKREREDTDDEESSSGSEDGSGPSDRKRHKFPSQIREAVSNVLKGYDWTLVPMPVRVNGSSKSKPHVKRPMNAFMVWAQAARRKLADQYPHLHNAELSKTLGKLWRMLNEDEKRPFIEEAERLRVQHKKDHPDYKY
QPRRRKNSKANQGSGDEAGSEASPISANTLFKALQAESPTGSEPHSPEDLKGPSPHDGSVGVTPSSQAPPTPPTTPKQDQGMTALKADGMKRDPTSNTLTAIHRDGPHHHHHHPQGHGHPNIDFSNVDIGPLDVMSSMESFDVEEFDQYLPPNGH
PASASGHHPSHPPYTSYSQMSSSATTTVTSSSSWMAKQNTSPRDNSQEQRLPVKMEQEHLPPPPPQYTPHPPASSYNYQPQYSSYQHSPPRPQYTDYPPPAHSPQQFYSPHPTSSSIPPPYNYMAPPQRSLYPTVAGAPSTWEPSYTQLARP

Drosophila melanogaster:

>CAB63903.1 Sox100B protein [Drosophila melanogaster]
MSDSSSSNCTKDRAKPVEALVLANYALKAEQKEAQGQGGRKEDERITTAVMKVLEGYDWNLVQASAKAPSDRKKEHIKRPMNAFMVWAQAARRVMSKQYPHLQNSELSKSLGKLWKNLKDSDKKPFMEFAEKLRMTHKQEHPDYKYQPRRKKARV
LPSQHSGDGGSPGPEMTLSATMGSSGKPRSSNSNGQRRAGKGNAAADLGSCAATISHANVGSSSSDVFSNEAFMKSLNSACAASLMEQSLIETGLDSPCSTASSMSSLTPPATPYNVAPSNAKASAANNPSLLLRQLSEPVANAGDGYGVLLEAG
REYVAIGEVNYQGQSAGVQSGAEGGGAGQEMDFLENINGYGGYTGSRVSYPAYSYPANGGHFATEEQQQQQALQASEALNYKPAAADIDPKEIDQYFMDQMLPMTQHHHPHHTHPLHHPLHHSPPLNSSASLSSACSSASSQQPVAEYYEHLGYS
PAASSASQNPNFGPQQPYANGAASMTPTLGDPAPQQELQSQQQEQQHQNPSQHHLWGTYTYVNP

Hydra vulgaris:

>XP_002161752.2 PREDICTED: transcription factor SOX-18-like [Hydra vulgaris]
MMAASETNSNFATHVIQNNNITHAVSQEQRKGEGYGSTTISLSEAAQCLKNLGEEGILCDDALLRLTMSSTSEHGETEFLDTKNSRVKRPMNSFMVWAQTARKKLAEKYPHLHNAHLSKMLGKLWKMLSPDEKQPYVLEASRLDKLHKDEHPEYK
YRPRRRPKGLKRGYSTPTMIVSPTTTSKPYTIPSNWARIVHAPENENLISPRAEAAQIVYATDGMQYYAVQKGSGFTPTMAHHHGNSVIVSPTNQPITLVQSTSAPTSNYPAYVTVPMPATSTAIFHPIAIPVQGVHTPAFVLRSSYPGSNILSP
SAIPIAYHNIDHRTIESHMQPNQHIKTESTNTIVKTPDGETIVIIQRPIDGHVVPFQLREVPSGATNTITVVPQSQVIQQHIVQTPNNQETKIL

Nematostella vectensis:

>XP_001630037.1 transcription factor SOX-9 [Nematostella vectensis]
MDKKVTEQQVQAVLGLDTDGSQVRNHQLSNAIASAVNHVLDGYDWSLIPLPVRVNGIKTQKPHVKRPMNAFMVWAQAARRKLADQYPHLHNAELSKTLGKLWKLLNDSEKKPFIEEAERLRIKHKREHPDYKYQPRRKKQKGNGNGDAGDATISA
DDLLKVLKGDSKLVPNNGDASASCASPESVSDGEVSSSESCSVPSPETPTAVPVKNEDVKNDEALSAQPGFPSCSKKDDSNSHAIDFDVGDLTTDLMAMGDVDSTEFDQYLPTYSQALLDSTLTKAINTTQINTQSLSNSRFTTSQAVQSPPPLP
SSYREFMVQLQKLPSEGSFPPNRVAPSATMQQRNQPDSNSFFPFSESEVNVCSSLAATRQPAFLSSPSTSGTLSSSSNSGRTHTLIWK

Amphimedon queenslandica:

>NP_001266245.1 uncharacterized protein LOC100631786 [Amphimedon queenslandica]
MSAGDLQYTVILQQAPSPSCSSLSSPSSPYQSNLLDVCDVGTATHVHHNGGSRGGGNGGSAGKKEHIKRPMNAFMVWAQLERRKMTTEFPDMHNAEISRRLGKLWRLLSDREKQPYIEESERLRIQHMKQYPDYKYRPRKKGGKKPKPVSNTSYL
GGNDSGSEEYYPTTMPTSSSNSCSCGAGIRRAPVPTCSIAVQCSMELGEHVIEREPSSPKQTAEISIQVGNGSAHLQQNRRRSFSSFAGDKRPRDLSLSCPPIKKRALIPVSSPPEVLPTSLDDIFPPSPPSSDSSPISHQHREDTLPTLHFEEF
MDPLMPYDPSAVDLALSGSSSALSSSTAASNVLSLNTFPLQSSAATSIFSPFNIDSCSTFDFPELPSDFADIFAQNASEFDTSITTLLST

Alignment

```
XP_002161752.2[H.vulgaris]           ------MMAASETNSNFATHVIQNNNITHAVSQEQRKGEGYGSTTISLSEAAQCLKN---     51
NP_001266245.1[A.queenslandica]      -------MSAGDLQYT----VILQQAPSPSCSSLSSPSSPY-------------------     30
CAB63903.1[D.melanogaster]           ---------------------------------------------MSDSSSSNCTKDR--     13
XP_001630037.1[N.vectensis]          -----------------------------------------------------------M     1
XP_019645392.1[B.belcheri]           ----------------------MSETETPVEVKEE----PAEL--------ES--LK--R     22
XP_032830402.1[P.marinus]            -------MMSDE-QHD----KHMSDVPSPDMSENCSVGSPADSLAGSDSDSQSCGSKRCS     48
XP_014034661.1[S.salar]              MNLLDPFLKMTD-EQD----KCLSDAPSPSMSEDS-AGSPCPSGSGSDT-----------     43
NP_989612.1[G.gallus]                MNLLDPFMKMTE-EQD----KCISDAPSPTMSDDS-AGSPCPSGSGSDT-----------     43
NP_001087942.1[X.laevis]             MNLLDPFMKMSE-EQD----KCLSGAPSPTMSEDS-AGSPCPSGSGSDT-----------     43
NP_000337.1[H.sapiens]               MNLLDPFMKMTD-EQE----KGLSGAPSPTMSEDS-AGSPCPSGSGSDT-----------     43
XP_009250264.1[P.abelii]             MNLLDPFMKMTD-EQE----KGLSGAPSPTMSEDS-AGSPCPSGSGSDT-----------     43
NP_035578.3[M.musculus]              MNLLDPFMKMTD-EQE----KGLSGAPSPTMSEDS-AGSPCPSGSGSDT-----------     43

XP_002161752.2[H.vulgaris]           ----------------------------LG----E--------EGILCDDA-----LLRL     66
NP_001266245.1[A.queenslandica]      ----------------------------------------QSNLLDVCDVGTATHVHH--     48
CAB63903.1[D.melanogaster]           ----AKPVEALVLANYALK-AEQ------KEAQGQ-----GGRKEDERITTAVMKVLEGY     57
XP_001630037.1[N.vectensis]          D----------------KKVTEQQVQAVLGLD--TDGSQVRNHQLSNAIASAVNHVLDGY     43
XP_019645392.1[B.belcheri]           EREDTDD-----------------EESSSGSEDGSGPSDRKRHKFPSQIREAVSNVLKGY     65
XP_032830402.1[P.marinus]            DREDGAGASGLLLAGGPGGVLDGGVVFGLGRDAGSGKKMDEDDKFPACIREAVSQVLKGY     108
XP_014034661.1[S.salar]              --ENTRPSENGLLMGPDGP--------------LVEFKKDDDDKFPVCIRDAVSQVLKGY     87
NP_989612.1[G.gallus]                --ENTRPQENTFPKGDP-----------------DLKKESDEDKFPVCIREAVSQVLKGY     84
NP_001087942.1[X.laevis]             --ENTRPQENTFPKGDQ-----------------EMKKETEDEKFPVCIREAVSQVLKGY     84
NP_000337.1[H.sapiens]               --ENTRPQENTFPKGEP-----------------DLKKESEEDKFPVCIREAVSQVLKGY     84
XP_009250264.1[P.abelii]             --ENTRPQENTFPKGEP-----------------DLKKESEEDKFPVCIREAVSQVLKGY     84
NP_035578.3[M.musculus]              --ENTRPQENTFPKGEP-----------------DLKKESEEDKFPVCIREAVSQVLKGY     84

XP_002161752.2[H.vulgaris]           TMSSTSEHGETEFLDTKNSRVKRPMNSFMVWAQTARKKLAEKYPHLHNAHLSKMLGKLWK     126
NP_001266245.1[A.queenslandica]      --NGGSRGGGNGGSAGKKEHIKRPMNAFMVWAQLERRKMTTEFPDMHNAEISRRLGKLWR     106
CAB63903.1[D.melanogaster]           DWNLVQASAKAP-SDRKKEHIKRPMNAFMVWAQAARRVMSKQYPHLQNSELSKSLGKLWK     116
XP_001630037.1[N.vectensis]          DWSLIPLPVRVNGIKTQKPHVKRPMNAFMVWAQAARRKLADQYPHLHNAELSKTLGKLWK     103
XP_019645392.1[B.belcheri]           DWTLVPMPVRVNGSSKSKPHVKRPMNAFMVWAQAARRKLADQYPHLHNAELSKTLGKLWR     125
XP_032830402.1[P.marinus]            DWTLVPMPVRVNGSSKSKPHVKRPMNAFMVWAQAARRKLADQYPHLHNAELSKTLGKLWR     168
XP_014034661.1[S.salar]              DWTLVPMPVRVNGSSKNKPHVKRPMNAFMVWAQAARRKLADQYPHLHNAELSKTLGKLWR     147
NP_989612.1[G.gallus]                DWTLVPMPVRVNGSSKNKPHVKRPMNAFMVWAQAARRKLADQYPHLHNAELSKTLGKLWR     144
NP_001087942.1[X.laevis]             DWTLVPMPVRVNGSSKNKPHVKRPMNAFMVWAQAARRKLADQYPHLHNAELSKTLGKLWR     144
NP_000337.1[H.sapiens]               DWTLVPMPVRVNGSSKNKPHVKRPMNAFMVWAQAARRKLADQYPHLHNAELSKTLGKLWR     144
XP_009250264.1[P.abelii]             DWTLVPMPVRVNGSSKNKPHVKRPMNAFMVWAQAARRKLADQYPHLHNAELSKTLGKLWR     144
NP_035578.3[M.musculus]              DWTLVPMPVRVNGSSKNKPHVKRPMNAFMVWAQAARRKLADQYPHLHNAELSKTLGKLWR     144
                                       .             .: ::*****:******  *: :: ::*.::*:.:*: *****:

XP_002161752.2[H.vulgaris]           MLSPDEKQPYVLEASRLDKLHKDEHPEYKYRPRRRPKGLKRGYSTPTMIVSPTTTSKPYT     186
NP_001266245.1[A.queenslandica]      LLSDREKQPYIEESERLRIQHMKQYPDYKYRPRKKGGKKPKPVSNTSYLGGNDSGSEEY-     165
CAB63903.1[D.melanogaster]           NLKDSDKKPFMEFAEKLRMTHKQEHPDYKYQPRRKKARVLPSQHS------GDGGSPGPE     170
XP_001630037.1[N.vectensis]          LLNDSEKKPFIEEAERLRIKHKREHPDYKYQPRRKKQKGNGNGDA------GD-----AT     152
XP_019645392.1[B.belcheri]           MLNEDEKRPFIEEAERLRVQHKKDHPDYKYQPRRRKNSKANQGSG------DEAGSEASP     179
XP_032830402.1[P.marinus]            LLSENEKRPFVEEAERLRVQHKKDHPDYKYQPRRRKSGKNGQSES-------DSSGEQTH     221
XP_014034661.1[S.salar]              LLNEGEKRPFVEEAERLRVQHKKDHPDYKYQPRRRKSMKNGQSES-------EDGSEQTH     200
NP_989612.1[G.gallus]                LLNESEKRPFVEEAERLRVQHKKDHPDYKYQPRRRKSVKNGQSEQ-------EEGSEQTH     197
NP_001087942.1[X.laevis]             LLNEGEKRPFVEEAERLRVQHKKDHPDYKYQPRRRKSVKNGQSEQ-------DDSAEQTH     197
NP_000337.1[H.sapiens]               LLNESEKRPFVEEAERLRVQHKKDHPDYKYQPRRRKSVKNGQAEA-------EEATEQTH     197
XP_009250264.1[P.abelii]             LLNESEKRPFVEEAERLRVQHKKDHPDYKYQPRRRKSVKNGQAEA-------EEATEQTH     197
NP_035578.3[M.musculus]              LLNESEKRPFVEEAERLRVQHKKDHPDYKYQPRRRKSVKNGQAEA-------EEATEQTH     197
                                      *.  :*:*::  :.:*   *  ::*:***:**::

XP_002161752.2[H.vulgaris]           IPSN-WARIVHA-PENENLISPRAEAAQIVYATDGMQ-----------------------     221
NP_001266245.1[A.queenslandica]      -YPT-TM-------PTSSS-NSCSCGAGIRRAPVPTCSIAVQCSMELGEHVIEREPSSPK     215
CAB63903.1[D.melanogaster]           MTLSATMGSSGK-PRSSNSNGQRRAGKGNAAADLGSCAATIS------HAN---------     214
XP_001630037.1[N.vectensis]          ISADDLLKVLKGDSKLVPNNGDASA----------SCA---S------------------     181
XP_019645392.1[B.belcheri]           ISANTLFKALQAESPTGSE-----------------PH---S------------------     201
XP_032830402.1[P.marinus]            ITTNAIYKALQADSPS--------------------------------------------     237
XP_014034661.1[S.salar]              ISPGAIFKALQQADS-PAS-----------------SI----------------------     220
NP_989612.1[G.gallus]                ISPNAIFKALQADSPQSSS-----------------SI----------------------     218
NP_001087942.1[X.laevis]             ISPNAIFKALQADSPHSTS-----------------SM----------------------     218
NP_000337.1[H.sapiens]               ISPNAIFKALQADSPHSSS-----------------GM----------------------     218
XP_009250264.1[P.abelii]             ISPNAIFKALQADSPHSSS-----------------GM----------------------     218
NP_035578.3[M.musculus]              ISPNAIFKALQADSPHSSS-----------------GM----------------------     218

XP_002161752.2[H.vulgaris]           ---YYAVQKGSG-------------------FTPTMAHHHGNS----VIVSPTN---QPI     252
NP_001266245.1[A.queenslandica]      QTAEISIQVGNGSAHLQQNRRRSFSSFAGDKRPRDLSLSCPPI-KKRALIP---VSSPPE     271
CAB63903.1[D.melanogaster]           --------VGSSSSDVF----------SNEAFMKSLNSACAASLMEQSLIETGLDS--PC     254
XP_001630037.1[N.vectensis]          ------------PE-SV----------S-----------------------DGEVSSSES     195
XP_019645392.1[B.belcheri]           ------------PEDLK----------G-----------------------PSPHDGSVG     216
XP_032830402.1[P.marinus]            -------------ADVH----------S-----------------------PGEHS----     247
XP_014034661.1[S.salar]              -------------GEVH----------S-----------------------PGEHS----     230
NP_989612.1[G.gallus]                -------------SEVH----------S-----------------------PGEHS----     228
NP_001087942.1[X.laevis]             -------------SEVH----------S-----------------------PGEHS----     228
NP_000337.1[H.sapiens]               -------------SEVH----------S-----------------------PGEHS----     228
XP_009250264.1[P.abelii]             -------------SEVH----------S-----------------------PGEHS----     228
NP_035578.3[M.musculus]              -------------SEVH----------S-----------------------PGEHS----     228

XP_002161752.2[H.vulgaris]           TLVQSTSAPTSNYPAYVTVPMPATSTAIFHPIAIPVQGVH-TPAFVLRSSYPG-------     304
NP_001266245.1[A.queenslandica]      VLPTSLDDIFPPSPPSSDSS----------------------------------------     291
CAB63903.1[D.melanogaster]           STASSMSSLTPPATPYNVAPSNAKA-------SAA-----NNPSLLLRQLSEPVANAGDG     302
XP_001630037.1[N.vectensis]          C--SVPSPETPTAVPV------------------KNEDVKNDEALSAQ---PGF------     226
XP_019645392.1[B.belcheri]           VTPSSQAPPTPPTTPKQDQGM---T-------ALKADGMKRDPTSNTL---TAIHRDGPH     263
XP_032830402.1[P.marinus]            --GQSQGPPTPPTTPKTDV-QSNKL-------DIKREG----------------------     275
XP_014034661.1[S.salar]              --GQSQGPPTPPTTPKTDI-QAGKV-------DLKREG----------------------     258
NP_989612.1[G.gallus]                --GQSQGPPTPPTTPKTDAQQPGKQ-------DLKREG----------------------     257
NP_001087942.1[X.laevis]             --GQSQGPPTPPTTPKTDV-QPGKP-------DLKREG----------------------     256
NP_000337.1[H.sapiens]               --GQSQGPPTPPTTPKTDV-QPGKA-------DLKREG----------------------     256
XP_009250264.1[P.abelii]             --GQSQGPPTPPTTPKTDV-QPGKA-------DLKREG----------------------     256
NP_035578.3[M.musculus]              --GQSQGPPTPPTTPKTDV-QAGKV-------DLKREG----------------------     256

XP_002161752.2[H.vulgaris]           SNILSPS---AIPIAYHNIDHRTIESHMQPNQHIKTESTNTIVKTPDGETIVIIQRPIDG     361
NP_001266245.1[A.queenslandica]      --PISHQHRE------------------------DT------LPTLHFEEFMDPLMPYDP     319
CAB63903.1[D.melanogaster]           YGVLLEAGREY-------VAIGEVNYQGQSAGVQSG------A-------------EGGG     336
XP_001630037.1[N.vectensis]          --P-SCSKKDDSNSHAIDFDVGDLTT-----DLM-A------MGDVDSTEFDQYLPTYSQ     271
XP_019645392.1[B.belcheri]           HHHHHPQGHGHPNIDFSNVDIGPL-------DVMSS------MESFDVEEFDQYLPPNGH     310
XP_032830402.1[P.marinus]            -RPLQEGGRQQ--IDFSNVDIRELSR-----EVISN------MESFDVNEFDQYLPPNGH     321
XP_014034661.1[S.salar]              -RPLQEGTGRQLNIDFRDVDIGELSS-----DVISH------IDTFDVNEFDQYLPPNGH     306
NP_989612.1[G.gallus]                -RPLAEGGRQPPHIDFRDVDIGELSS-----DVISN------IETFDVNEFDQYLPPNGH     305
NP_001087942.1[X.laevis]             -RPLQESGRQPPHIDFRDVDIGELSS-----EVIST------IETFDVNEFDQYLPPNGH     304
NP_000337.1[H.sapiens]               -RPLPEGGRQPP-IDFRDVDIGELSS-----DVISN------IETFDVNEFDQYLPPNGH     303
```

```
XP_009250264.1[P.abelii]            -RPLPEGGRQPP-IDFRDVDIGELSS-----DVISN------IETFDVNEFDQYLPPNGH          303
NP_035578.3[M.musculus]             -RPLAEGGRQPP-IDFRDVDIGELSS-----DVISN------IETFDVNEFDQYLPPNGH          303
                                                                                           .

XP_002161752.2[H.vulgaris]          HVVPFQLREVP--SGATNTITVVPQ---SQVIQQHIVQTPNNQE--------TKIL----          404
NP_001266245.1[A.queenslandica]     SAVDLALSGSS------SALSSSTAAS--NVLSLNTF---------PLQ-----------          351
CAB63903.1[D.melanogaster]          AGQEMDFLENINGYGGYTGSRVSYPAY-SYPANGGHFATEEQQQQQALQASEALNYKPAA          395
XP_001630037.1[N.vectensis]         ALLDSTLTKAINT----TQINTQ-------SLSNSRFTTSQAVQSPPPLPSSYREFMVQ-          319
XP_019645392.1[B.belcheri]          PASASGHHPSHPPYTSYSQMSSSATT---TVTSSSSWMAKQNTS--PRDNSQEQRLPVK-          364
XP_032830402.1[P.marinus]           PGHGQSVAA---SYGGT-----------GYSINGHAWLSKQQQQ--QQQQQQQH------          359
XP_014034661.1[S.salar]             PGAPGTAAGQV-SYNGTYGISSSAVSQAAGGTAGHSWMTKSQSQ----------------          349
NP_989612.1[G.gallus]               PGVPATHGQVT-TYSGTYGISSSASSP---AGAGHAWMAKQQPQ--PPQPP---------          350
NP_001087942.1[X.laevis]            PGVGS--AQA--PYTGSYGISSTPSAT---TGAGSAWMSKQQQQ--P-------------          342
NP_000337.1[H.sapiens]              PGVPATHGQV--TYTGSYGISSTAATP---ASAGHVWMSKQQAP--PPPPQQPPQAPPA-          355
XP_009250264.1[P.abelii]            PGVPATHGQV--TYTGSYGISSTAATP---AGAGHVWMSKQQAP--PPPPQQPPQAPPA-          355
NP_035578.3[M.musculus]             PGVPATHGQV--TYTGSYGISSTAPTP---ATAGHVWMSKQQAP--PPPPQQPPQAPQA-          355


XP_002161752.2[H.vulgaris]          ------------------------------------------------------------          404
NP_001266245.1[A.queenslandica]     ------------------------------------------------------------          351
CAB63903.1[D.melanogaster]          ADIDPKEIDQYFMDQMLPMTQHHHPHHTHPLHHPLHHSPPLNSSASLSS-ACSSASSQQP          454
XP_001630037.1[N.vectensis]         -------LQKLPSEGSFPPNR-------------------------------------VA          335
XP_019645392.1[B.belcheri]          -------MEQ-------------------------EHLPPPPPQYTP-----H-----PP          382
XP_032830402.1[P.marinus]           ---------------------------------TLSSPPPPPPAISSPEQRAHVKTEQLS          386
XP_014034661.1[S.salar]             ------------------------------QQHSLTTLGSGGEQGQNQRTPTHIKTEQLS          379
NP_989612.1[G.gallus]               -------------------------A-QPPAQHTLPALSGE--QG-PAQQRPHIKTEQLS          381
NP_001087942.1[X.laevis]            ------------------------------QQHSLSTINSE--QS-QSQQRTHIKTEQLS          369
NP_000337.1[H.sapiens]              -------PQAPPQPQAAPPQQPAAPP-QQPQAHTLTTLSSE--P--GQSQRTHIKTEQLS          403
XP_009250264.1[P.abelii]            -------PQAPPQPQAAPPQQPAAPP-QQPQAHTLTTLSSE--P--GQSQRTHIKTEQLS          403
NP_035578.3[M.musculus]             -------PQAPPQQQAP-PQQPQ-AP-QQQQAHTLTTLSSE--P--GQSQRTHIKTEQLS          401


XP_002161752.2[H.vulgaris]          ------------------------------------------------------------          404
NP_001266245.1[A.queenslandica]     --------------------------------------------SSAATSIFSPFNIDSC          367
CAB63903.1[D.melanogaster]          VAEYYEHLGYSPAASS-----------------------------ASQNPNFGPQQPYAN          485
XP_001630037.1[N.vectensis]         PSATMQQ------------------------------------RNQPDSNSFFPFSESEV          359
XP_019645392.1[B.belcheri]          ASSYNYQPQYS------------------------------SYQHSPPRPQYT-------          405
XP_032830402.1[P.marinus]           PSHYSDQQQQQQQPQQQQQHSPQQQQQQQQQQPQQAQQAQQQVQQQQQLGGYSPFSIQHY          446
XP_014034661.1[S.salar]             PSHYNDQQNSS----------------------------------PPQHVTYGSFNLQHY          405
NP_989612.1[G.gallus]               PSHYSEQQQHSPQ----------QQ------------------QQQQQQLGYGSFNLQHY          413
NP_001087942.1[X.laevis]            PSHYSDQQQ----------------------------------QHSPQQLNYSSFNLQHY          395
NP_000337.1[H.sapiens]              PSHYSEQ-Q----------------------------------QHSPQQIAYSPFNLPHY          428
XP_009250264.1[P.abelii]            PSHYSEQ-Q----------------------------------QHSPQQIAYSPFNLPHY          428
NP_035578.3[M.musculus]             PSHYSEQ-Q----------------------------------QHSPQQISYSPFNLPHY          426


XP_002161752.2[H.vulgaris]          ------------------------------------------------------------          404
NP_001266245.1[A.queenslandica]     STFDFPELPSDFADIFAQNAS-----------EFD-----TSITTLLST-----------          400
CAB63903.1[D.melanogaster]          GAASMTPTLGDPAPQQE-----------LQSQQQEQQHQNPSQHHLWGTYTYVNP-----          529
XP_001630037.1[N.vectensis]         NVCSSLAATRQ--------------------PAFLSS---PSTSGTLSSSSNSGRTHT--          394
XP_019645392.1[B.belcheri]          ---DYPPPAHS-------------------PQQFYSPH--PTSSSIPPPYNYMAPPQRSL          441
XP_032830402.1[P.marinus]           GAAVVPAISRSQYSYADHHHHHHHHQSSAAAAAYYSGHSAGQTAGLYSGFSYMGPSQRPS          506
XP_014034661.1[S.salar]             SSSSYPSITRAQYDFSDHQGG---------TNSYYSHAAGGQGSGLYSFSSYMSPSQRPM          456
NP_989612.1[G.gallus]               GS-SYPPITRSQYDYTEHQNS----------GSYYSHA-AGQSGGLYSTFTYMNPTQRPM          461
NP_001087942.1[X.laevis]            SS-SYPTITRAQYDYTEHQGS----------NSYYTHA-SGQNSGLYSNFTYMNPSQRPM          443
NP_000337.1[H.sapiens]              SP-SYPPITRSQYDYTDHQNS----------SSYYSHA-AGQGTGLYSTFTYMNPAQRPM          476
XP_009250264.1[P.abelii]            SP-SYPPITRSQYDYTDHQNS----------SSYYSHA-AGQGTGLYSTFTYMNPAQRPM          476
NP_035578.3[M.musculus]             SP-SYPPITRSQYDYADHQNS----------GSYYSHA-AGQGSGLYSTFTYMNPAQRPM          474


XP_002161752.2[H.vulgaris]          -----------------------------------            404
NP_001266245.1[A.queenslandica]     -----------------------------------            400
CAB63903.1[D.melanogaster]          -----------------------------------            529
XP_001630037.1[N.vectensis]         --------------------LIWK-----------            398
XP_019645392.1[B.belcheri]          YPTVAGAP------------STWE--PSYTQLARP            462
XP_032830402.1[P.marinus]           YTPIADATGVPSIPQPHSPP-SWE-QPVYTQLTRP            539
XP_014034661.1[S.salar]             YTPIADTTGVPSVPQTHSPQQHWDQQPVYTQLSRP            491
NP_989612.1[G.gallus]               YTPIADTSGVPSIPQTHSPQ-HWE-QPVYTQLTRP            494
NP_001087942.1[X.laevis]            YTPIADTTGVPSIPQTHSPQ-HWE-QPVYTQLTRP            476
NP_000337.1[H.sapiens]              YTPIADTSGVPSIPQTHSPQ-HWE-QPVYTQLTRP            509
XP_009250264.1[P.abelii]            YTPIADTSGVPSIPQTHSPQ-HWE-QPVYTQLTRP            509
NP_035578.3[M.musculus]             YTPIADTSGVPSIPQTHSPQ-HWE-QPVYTQLTRP            507
```
