# Supplementary material for: An Evolutionary Perspective on the Origin, Conservation and Binding Partner Acquisition of Tankyrases
Source: Biomolecules. 2022 Nov 15;12(11):1688. doi: 10.3390/biom12111688 (PMC9688111; doi:10.3390/biom12111688)
Supplement: Supplementary file 1 [file biomolecules-12-01688-s001.zip › biomolecules-1913719-supplementary-11.17 -done/Data_7/Striatin.pdf]

Striatin

Homo sapiens:

```
>sp|O43815|STRN_HUMAN Striatin OS=Homo sapiens OX=9606 GN=STRN PE=1 SV=4
MDEQAGPGVFFSNNHPGAGGAKGLGPLAEAAAAGDGAAAAGAARAQYSLPGILHFLQHEW
ARFEVERAQWEVERAELQAQIAFLQGERKGQENLKKDLVRRIKMLEYALKQERAKYHKLK
YGTELNQGDMKPPSYDSDEGNETEVQPQQNSQLMWKQGRQLLRQYLQEVGYTDTILDVKS
KRVRALLGFSSDVTDREDDKNQDSVVNGTEAEVKETAMIAKSELTDSASVLDNFKFLESA
AADFSDEDEDDDVDGREKSVIDTSTIVRKKALPDSGEDRDTKEALKEFDFLVTSEEGDNE
SRSAGDGTDWEKEDQCLMPEAWNVDQGVITKLKEQYKKERKGKKGVKRPNRSKLQDMLAN
LRDVDELPSLQPSVGSPSRPSSSRLPEHEINRADEVEALTFPPSSGKSFIMGADEALESE
LGLGELAGLTVANEADSLTYDIANNKDALRKTWNPKFTLRSHFDGIRALAFHPIEPVLIT
ASEDHTLKMWNLQKTAPAKKSTSLDVEPIYTFRAHKGPVLCVVMSSNGEQCYSGGTDGLI
QGWNTTNPNIDPYDSYDPSVLRGPLLGHTDAVWGLAYSAAHQRLLSCSADGTLRLWNTTE
VAPALSVFNDTKELGIPASVDLVSSDPSHMVASFSKGYTSIFNMETQQRILTLESNVDTT
ANSSCQINRVISHPTLPISITAHEDRHIKFYDNNTGKLIHSMVAHLEAVTSLAVDPNGLY
LMSGSHDCSIRLWNLESKTCIQEFTAHRKKFEESIHDVAFHPSKCYIASAGADALAKVFV
```

Pongo abelii:

```
>XP_002812184.3 striatin isoform X1 [Pongo abelii]
MDEQAGPGVFFSNNHPGAGGAKGLGPLAEAAAAGDGAAAAGAARAQYSLPGILHFLQHEWARFEVERAQW
EVERAELQAQIAFLQGERKGQENLKKDLVRRIKMLEYALKQERAKYHKLKYGTELNQGDMKPPSYDSDEG
NETEVQPQQNSQLMWKQGRQLLRQYLQEVGYTDTILDVKSKRVRALLGFSSDVTDREDDKNQDSVVNGTE
AEVKETAMIGKSELTDSASVLDNFKFLESAAADFSDEDEDDDVDGREKSVIDTSTIVRKKALPDSGEDRD
TKEALKEFDFLVTSEEGDNESRSAGDGTDWEKEDQCLMPEAWNVDQGVITKLKEQYKKERKGKKGVKRPN
RSKLQDMLANLRDVDELPSLQPSVGSPSRPSSSRLPEHEINRADEVEALTFPPSSGKSFIMGADEALESE
LGLGELAGLTVANEADSLTYDIANNKDALRKTWNPKFTLRSHFDGIRALAFHPIEPVLITASEDHTLKMW
NLQKTAPAKKSTSLDVEPIYTFRAHKGPVLCVVMSSNGEQCYSGGTDGLIQGWNTTNPNIDPYDSYDPSV
LRGPLLGHTDAVWGLAYSAAHQRLLSCSADGTLRLWNTTEVAPALSVFNDTKELGIPASVDLVSSDPSHM
VASFSKGYTSIFNMETQQRILTLESNVDTTANSSCQINRVISHPTLPISITAHEDRHIKFYDNNTGKLIH
SMVAHLEAVTSLAVDPNGLYLMSGSHDCSIRLWNLESKTCIQEFTAHRKKFEESIHDVAFHPSKCYIASA
GADALAKVFV
```

Mus musculus:

```
>NP_035630.2 striatin [Mus musculus]
MDEQAGPGVFFSNNHPGAGGAKGLGPLAEAAAAGDGAAAAGAARAQYSLPGILHFLQHEWARFEVERAQW
EVERAELQAQIAFLQGERKGQENLKKDLVRRIKMLEYALKQERAKYHKLKYGTELNQGDMKPPSYDSDEG
NETEVQPQQNSQLMWKQGRQLLRQYLQEVGYTDTILDVKSKRVRALLGFSSDVTDREDDKNQDSVINGTE
AEVKETAMIGKSELTDSASVLDNFKFLESAAADVSDEDEDEDTDGRAKSVIDTSTIVRKKALPDTSEDRD
TKEALKEFDFLVTSEEGDNESRSAGDGTDWEKEDQCLTPEAWNVDQGVISKLKEQYKKERKGKKGVKRPN
RSKLQDMLANLRDVDELPSLQPSVGSPSRPSSSRLPEQELSRADEVEALTFPPSSGKSFIMGADEALESE
LGLGELAGLTVANEADSLAYDIANNKDALRKTWNPKFTLRSHFDGIRALAFHPIEPVLITASEDHTLKMW
NLQKTAPAKKSTSLDVEPIYTFRAHKGPVLCVVMSSNGEQCYSGGTDGRIQSWSTTNPNVDPYDAYDPSV
LRGPLLGHTDAVWGLAYSAAHQRLLSCSADGTLRLWNTTEVAPALSVFNDNQELGIPASVDLVSSDPSHM
VASFSKGYTSIFNMETQQRVLTLESNVDSTSSSSCQINRVISHPTLPISITAHEDRHIKFYDNNTGKLIH
SMVAHLEAVTSLAVDPNGLYLMSGSHDCSIRLWNLESKTCIQEFTAHRKKFEESIHDVAFHPSKCYIASA
GADALAKVFV
```

Gallus gallus:

```
>XP_040524727.1 striatin isoform X3 [Gallus gallus]
MDEQAGPGVFFSNNNNNNALLLPPPTGGPGLEPGEAAAAAAAAAAGGGGGGVSAGGAAAPVSASRAQYSV
PGILHFLQHEWGRFEAERAEWEAERAELQAQIAFLQGERKGQENLKKDLVRRIKMLEYALKQERAKYHKL
KYGTELNQGDMKPPNYDSDEGNETEIQPQQNSQLIWKQGRQLLRQYLQEVGYTDTILDVKSKQVRALLGL
SSDASEKENRNQEPMVNGTEGQIKENAMIGKPELTDSASLLETFKFLENAAADFSDEEDEEDIEGREKTI
IDTATIVRKRVLSDSSEDRDTEEALKEFDFLVTSDEGDGESRSSGDGTDWEKEDQCLMPEAWNVDQGVIT
KLKEQYKKERKGKKGVKRPNRSKLQDMLANLRDVDDLPSLQPSVAPSSRPSSSRLIEHEINRTDEVEALT
FPPSSGKSFIMGTDETLENELGLGELAGLTVANEADSLTYDIGNNTDALRKTWNPKFTLRSHFDGIRGLA
FHPIEPVLITASEDHTLKMWNLQKTAPAKKSASLDVEPIYTFRAHSGPVLCVVMSSNGEQCYSGGTDGLI
HGWNTTNPNIDPYDSYDPSVLRGAFVGHTDAVWGLVYSGTHQRLLSCSADGTVRLWKATEVAPALNVFND
NQEMGIPSSVDLVSSDPSLMVASFNTGHTSIFNMETRQRILTLESGVDNTVSTSCQINRVISHPTLPISI
TAHEDRHIKFYDNNTGKLIHSMVAHLDAVTSLAVDPNGLYLMSGSHDCSIRLWNLESKTCIQEFTAHRKK
FDESIHDVAFHPSKCYIASAGADALAKVFV
```

Xenopus laevis:

```
>XP_041418671.1 striatin isoform X1 [Xenopus laevis]
MDEQAGPGVFFNNNNNSLLSAGTGPGGAGKGFEAGAADAASVAGGGGGGSAGRAQYSIPGILHFLQHEWA
RFEVERAQWEVERAEMQAQIAFLQGERKGQENLKKDLVRRIKMLEYALKQERAKYHKLKYGTELNQGDMK
PPNYDSDEANENEIQPPQQNSQFIWKQSRQLLRQYLQEVGYTDTILDVKSKRVRSLLGLSADSSDRLTDT
NQDSMVNGTETELPNPLVLGKPDMTDSASVLETFKFLENAAAEFSDEEDEEENESRDKTIIDSATIVRRR
IIPDSNDDTEEALKEFDFLGSDDSEGGESRSAGDGTEWEKEDQCPITESWNVDQGVITKLKEQYKKERKG
KKGVKRPNRSKLQDMLANLREVDELPSLQPSVVSSSRPASSRISEQEINRADEVEALTFPPSSGKSFIMG
ADEALENELGLGELAGLTVANEADSLTYDIANNKDALRKTWNPKFTLRSHFDGIRGLAFHPSEPVLITAS
EDHTLKMWNLQKTAPAKKSTSLDIEPIYTFRAHRGPVLCVVMSTNGEQCYSGGTDGVIQSWNTPNSNVDP
YDSYDPTVLRGAYEGHTDAVWGLVYSAVHQRLLSAAADGTIRIWNATEVSPALSIFNENQELGIPSSVDL
VSCDPAHLVASFNNGHTNIFNMETHQKVLTLESCIDTAVNSTCQINRVISHPTLPISITAHEDRHIKFYD
NNTGKLIHSMVAHLDAVTSLAVDPNGLYLMSGSHDCSIRLWNLESKTCIQEFTAHRKKSDESIHDVAFHP
SKCYIASAGADALAKVFV
```

Salmo salar:

```
>XP_045558221.1 striatin-like isoform X1 [Salmo salar]
MDEQAGPGVFFNNNNSILAGGGKVLLPEGDAGEAARAPYSIPGILHFLQHEWARFEVERAQWEVQRAELQ
AQTAFLQGERKGQENLKKDLVRRIKMLEYALKQERAKYHKLKYGTELNQGDMKPPSYDSDEVNENETTGS
LNNQLSWKQGRQLLRQYLQEVGYTDTILDVKSQRVKTLLAMAGDGGGRPGERTGSETPLVNGTDTVPKGT
GTGGRKTEVSESSAVLDTFRFYCDEDDDEDGAGLDRTIMEQTGPTGTMVRKKPPSSSSPALSLSMDTSED
PDAEDALKGFDFLSSPDNMDTSPESRSAGDGTDWEKEEQSAMSEAWDVDQGLITKLKEEYRKERKGKKGG
KRPNRSKLQDMLANLRDQEDMSPREPSSSPQARSNAPRLNEQHQDNRTDEVEALTFPPSSGKAFIMGPDE
NMEAELGLGELAGLTVANEADSLAYDMSNNKDALRRTWNPKFTLRSHFDGIRGLAFHPVEPVLVTASEDH
TLKMWNLQKTAPAKKSASVDMEPMYTFRAHQGAVLCVVMSSTGEQCFSGGVDGTIQSWNTPNPNLDPYDS
YEPTVLRGALCGHTDLVWGLVYSGVHQRLLSCSGDGTVRLWDASNTKPALATFQNKEFGVPSSVDLVCSD
PAHMVTSFTSGQIGLFNMETQQLVLSLESAAETGTPCQINKVLSHPTLPITITAEEDRHIKFFDNNTGKL
IHSMVAHLDAVTSLAVDPNGLYLMSGSHDCSIRLWNLETKTCIQEFTAHRKKNDESIHDVAFHPSKAYIA
SAGADALAKVFV
```

Petromyzon marinus:

>XP_032803414.1 striatin-3-like isoform X1 [Petromyzon marinus]
MDEHPALQVGSPGLKQLQQQPSGGSGQQQQQQQQQQLLQQQLQMHQQQQGGEGGSESGRPHYSMPGILH
YIQHEWARFEMERASWDMERAELQARIAFLQGERKGQENLKKDLVRRIKMLEYALKQERAKYHKLKYGTE
LNQGDMKPPTFDAEETNDTDTPVVTSNSQLTWRQGRQLLRQYLQEVGYTDTILDVRSQRVRSLLGLSGPP
DQNGAPDAKNIEQILNGRDIMSIKPKEDDAKRNGDKSRSEPLVPSVVDTFQFLQEEEDSDDGEDDDDDDE
SVGLVDERDRPRASKKAKIGNEGLAADLADDPDTEEALKEFDFLVTTEDGEGAGEARSSGDGTEWDKEEL
SPSGEEWDIDHGLITKLKEQYKKERKNKKGAKRPNRSALQDMLANLREVDELSPLAASSPPRQPEERAAP
LPHDNARSDEGESPFPRGTGKMFISGPEEAMDTTGLGELADLTVTNDADSPSYDIGANKDAFRKTWNPKF
TLRSHFDGVRALAFHPSEHVLVTASEDHTLKLWNLQKTISGKKTASLDVEPIYTFRAHTGPVLSLAISTS
GEQCFSGGTDASIRCWNMPSPNIDPYDTFEASVLGQTLLGHSDAVWGLAYSSLRRRLLSCSADGTVRLWD
PSCQDSPPCLNTYNPDKEHGIPVSVDFVSDASHLVATFGGGDTLVFDMETAKPVITLESRIETDTPLGNQ
INRVVSHPTLPITVTAHEDRHIKFFDNNTGKQVHSMVAHLDAVTCLAVDPNGLYLMSGSHDCSIRLWNLE
SKTCVQEITAHRKKFDEAIHDVAFHPSKAFIASAGADALAKVFV

Branchiostoma belcheri:

>XP_019631056.1 PREDICTED: striatin-3-like [Branchiostoma belcheri]
MDDGSTGNPHAVPPGSNQPGMIPNKPQTLQDEQSQQTKYSIPGILHFIQHEWARFEMERAHWEVERAELQ
ARIAFLQGERKGQENLKRDLVRRIKMLEYALKQERAKYHKLKYGTELNQGEMRPPTPPSDEERETDSPLS
QNSQVTWRQGRQLLRQYLQEIGYSDTILDVRSNRVRTLLGLTPNESTKQDGEAGEVLVNGEQEREARRDA
DRRPVEKKRAGIPEIDSEASVLATFDFLSSEAVDDEDEENLSDGEEDGGAADDESEEREQRRRREKGKKA
LVQMMENESLAGMEDVLADPETEEALAEFDFLVSEAGEGAGEAKSTDGAEWGLDKSPTTEEWAMDKNLLN
KLKEEFKKERKSKKGAKRPNRSTLQAMLANLGGDDDIVMPASGPPRSAINNPDLGPLDEPRKEGGPEEQV
LGVGGPEEESLEAALGLGDLAGLTVTNEAEPLQYDVAATKEAFRKTWNPKYTLRSHFDAVRALAFHPIEP
VLITASEDHTLKMWNLQKTVPAKKSASLDVEPIYTFRAHSGPVTCLCVSSNGDQCYSGSTDSTIRCWNIP
SPNIDPYDAFDPTVLGQALVGHTDAIWGVTIHSVKQQLLSCSADGTVRLWSLQNKSPLLNTYTAEKDDGA
PTSVDFVRCELSQMVAAYSSSVAVLYDLETAQPVAKLDARQGSDSGVSSQINRVVAHPTLPITITAHEDR
HIKFFDNNSGKMVNTMVAHLDAVTSLAVDPNGLYLLSGSHDCSIRLWNLDSKTCVQEITSHRKKFDESIF
DVAFHPSKPYIASAGADALAKVFV

Drosophila melanogaster:

>NP_609177.1 connector of kinase to AP-1, isoform A [Drosophila melanogaster]
MGTNSGATAGINNKPVGGATGAGVLVGGGVGGANSSIGGVLSNSLGGGGSGGLSISGLNAGGQNANVGGM
GNVGGDDGGNGMVGGGVNNQQATTPQYTIPGILHFIQHEWSRFELERSQWDVDRAELQARIAMLLGERKC
LESLKSDLTRRIKMLEYALRQERAKFYRLKYGTDPPQLNEFKPSNEDAGLAGEVATDSEVPYSSVSNTTW
RQGRQMLRQYLAEIGYTDNIIDVRSNRVRSILGLNNNAEHDGSGGGLGGGLGGGTGGENLSPNINGNESN
KRASETEGRHTPAKKVQQSIDEIIVDTEAAVMANFEFLGATEMSDDDEISDDLEMVATDNDDTDVKLAKR
AKSGKDMLTEEVDGSLGLGELAQLTVNNESDGAYDANSKDGTGGSAGGAGYRKTWNAKYTLRSHFDGVRS
LIFHPEEPVLITASEDHTLKLWNLQKTVQAKKSASLDVEPLYTFRAHTGPVLCLGMSSSGETCYSGGLDG
NIECWQLPSPNIDPYDCYDPNVHSGTLEGHTDAVWGLTTMQSNIVSCSADGTVKLWSPYNKEPLLRTYTA
SEAEGVPSSVDFVRNEVDHIVVAYNSAHCIVYDTETGKQVVRLEAAQEMSGNTGKFINKVVSHPTLPITI
TAHEDRHIRFWDNTSGTLVHSMVAHLEPVTSLAVDAHGLYLLSGSHDCSIRLWNLDNKTCVQEITAHRKK
FDESIFDVAFHATKPYIASAGADGLAKVFV

Hydra vulgaris:

>NP_001296588.1 striatin-3-like [Hydra vulgaris]
MEIDAKIINGVNKSNGNVESSSNPMVYSIPGILHYIQHEWARFEIERAHWEVERAELQARIAFLQGERKG
QENLKRDLIRRIKMLEFALRKERQKFHKLKYGTELKLPDIKDDEDHDLANNEIISNKNHDLVFNYKQGRQ
LLRQYLQEIGFTDKVLEMRSARIRSLLGITTDDLPTEDASTKSKKNEKRQEDFTVSLNQEQNCFDNPVDT
DSTEVIEDSVTKNSLESKKVEHETGDTFVLKDFDFLGNEELNVDEASNIQNEEQDDWSNVDSEKLQNKIK
QFKNETKDRGKKKRPNKDVMKNMLQTFNAGEEEILPPPPQASTPSVPRVSGGVVDDEIPVIGDTVLPSSG
YSPRQVKLVAVNHSGVSDVTEVAMGLGDLADLSVSNELGNLDSKDDMRKTWGPKYTLRSHFDGVRSLCFH
PYEAALITGSEDHTLKLWSLQKNVQGKRGNISDLEPVYTFRGHSDGVLCCAINSNGTVCYSGGLDFKIQC
WNLPSLNIDLFGPYDPNVLGDALIGHSDSVWDLAYHIDTDFLLSCSADGTCKLWNPSIKSPLLETYKSPE
SGTPTSVSFVCTDTGQVAASYTSESCVVYDIETAKPIVTFSPEKTYGLSQINKVISHPTHPILITAHEDR
HVRFFDLSSGKEVHSMVAHLDAVTSLSIDPSGLYILSGSHDGSIRLWSMENKTCIQEITAHRKKFDEAVF
DVAFHPSQPYIASAGADALAKVFV

Nematostella vectensis:

>XP_032221713.1 striatin [Nematostella vectensis]
MYSIDMDSDSSGKLPQSSQNIENDSGTKFTIPGILHFIQHEWARFEMERAHWEVERAELQARIAFLQGER
KGQENLKHDLIRRIKMLEFALKQERAKYHRLKYGTDLGQERSPPKEQEQKEEHPNEVTGSSNKPSMNWRQ
GRQLLRQYLQEVGYTDAILDVRSSRVRSILGLSAAEPLAPAQMNGTSPRKPDDGPEFIREPSPQSREPVS
VRQQAPSSHDVVAPNPIRRAPPHIPPIGKKPADEDFESEAAVMANFDFLENDIEDEEENEDEVDDGGSAD
DEDDERDVLKTERRIIKKPKTDSNEDLSPEEEEALDEFKFVMGDDDDMDSDSTVIEVPTGKDLDEVNIHM
PDGEEGMAEQDHLHPDGTPKDEWDVDPSMLAKLKEQYKKERKSKKNVKRPPRSALQVMIANLGGDEEMPP
QPQSTPAPATTGPSPVRLVGSRAMSEIPPPIGSVKIPTKTEAAPAASSPPKNEPFEDALGLGELAELTVN
NEAEALSYEINKKDEFRKTWNPKFTLRSHFDSIRSVYFHPQEAAVITASEDHTMKLWNLQKTVPQKKSNI
LDVEPIYTFRGHSAAVLSVVISSTSDMCFSGGADGTILCWNIPSLDLDPYGPYSPNVTGALLEVHTDAVW
GLAVQTNSLQLLSCSSDGTCRLWNPTLKSPLLNTFILDKESSCTPTSIDFLRMDTSQMVASYSSARAAIF
DLETAEAVVNLDSAKTYNNSPSTQINKVVSHPTLPVTITAHEDRHIRFFDNNTGKQIHSMVAHLDAVTSL
AVDPNGLYLLSGSHDCSIRLWNLDSKTCVQEITSHRKKFDESIYDVAFHPTKPYIASAGADGLAKVFV

Amphimedon queenslandica:

>XP_019858798.1 PREDICTED: striatin-3-like isoform X1 [Amphimedon queenslandica]
MSIEEAAPVTRPFMMGPNGPSGPIPSDPGQPQAGYSVPGVLQFIKQEFGRFERERASWEVERAELQGKIA
FLQGERKGHDNLMRDLVRRIKMLEFALKQERNKVYKLTHGTDSGLSLKPPQLDTETNPVTEESSLPSLVN
QKESRQILRDYLREVGFPDNVLEARVARLNLYRTLMSSSTNQNEPPLPPPAVEAPSETYSPKPPHPPESS
TGGGSDIVPNGTDMQEQIGGRRDTEAESEDDAVKQVQNTFDFLSNEDSDDEDEETEWQQVATKDLFQGLE
NTTEPAGLDKGAIMDGDFVYEDRYSSKSDNPPDLPIGEEDNEWEFKRQVERYQEYKRSNKSKNISSRPPR
EQLRQLQQYANTNQPAEEMSSVMGLMGMPVRGHHDLEGVRVSPHRIPDERPEVSLGIGELVSIANPSDFI
MNPSTSGSSKRWEHKYTMRNHYDAVTCVQFHPMDDMLVTGSEDATIKLWSIPKSSQKKSAIIDVEPAFTF
RGHTGPVLSLAVSSDGEVVYSGSADGQLRMWQTPNDLSDPFDIYDLDIQKGVLEGHTDAIWGLVFNQSNG
LLASASADGHCILWDPVNSSQIKSIVSEEALGSPTSIDFLHGESIVVSYSTAKVVVYDVETGKPVVTLDS
ALTYNGTPTTQINKVLSHPTLPIVITAHEDKYICFFDSKSGQVTHSMTAHMDAVTGLAIDPHGLYILSGS
HDGSLRFWSMETKTCVQEITAHRKRFDESIYNVTCHLTKPFFASAGADGIAKVLL

Alignment

```
XP_019858798.1[A.queenslandica]      ----------------------------------------------------MS--IEEA      6
NP_001296588.1[H.vulgaris]           ------------------------------------------------------------      0
NP_609177.1[D.melanogaster]          MGTNSGATAGINNKPVGGATGAGVLVGGGVGGANSSIGGVLSNSLGGGGSGGLSISGLNA      60
XP_032221713.1[N.vectensis]          ------------------------------------------------------------      0
XP_019631056.1[B.belcheri]           -------------------------MDDGSTGN------PHAVPPGSNQPGMIP--NKPQ      27
XP_032803414.1[P.marinus]            ---------------MDEHPALQ----VGSPGL------KQLQQQPSGGSGQQQ--QQQQ      33
XP_045558221.1[S.salar]              ---------------MDEQAGPGVFFNNNNSIL------A------GGGKVLLP--E---      28
XP_041418671.1[X.laevis]             ---------------MDEQAGPGVFFNNNNNSL---------LSAGTGPGGAGK--GFEA      34
XP_040524727.1[G.gallus]             ---------------MDEQAGPGVFFSNNNNNN------ALLLPPPTGGPGLEP--GEAA      37
NP_035630.2[M.musculus]              ---------------MDEQAGPGVFFSNNHPG-------------AGGAKGLGP--LAEA      30
O43815[H.sapiens]                    ---------------MDEQAGPGVFFSNNHPG-------------AGGAKGLGP--LAEA      30
XP_002812184.3[P.abelii]             ---------------MDEQAGPGVFFSNNHPG-------------AGGAKGLGP--LAEA      30


XP_019858798.1[A.queenslandica]      A---PVTRPFMMGP-------NGPSGPIPSDPGQPQAGYSVPGVLQFIKQEFGRFERERA      56
NP_001296588.1[H.vulgaris]           -----------MEIDAKI-INGVNKSNGNVESSSNPMVYSIPGILHYIQHEWARFEIERA      48
NP_609177.1[D.melanogaster]          GGQN-AN-VGGMGNVGGDDGGNGMVGGGVNNQQATTPQYTIPGILHFIQHEWSRFELERS      118
XP_032221713.1[N.vectensis]          ----------MYSIDMDSDSSGKLPQSSQNIENDSGTKFTIPGILHFIQHEWARFEMERA      50
XP_019631056.1[B.belcheri]           T---------------------------LQDEQSQQTKYSIPGILHFIQHEWARFEMERA      60
XP_032803414.1[P.marinus]            QQQQLLQQQLQMHQQQQGG---------EGGSESGRPHYSMPGILHYIQHEWARFEMERA      84
XP_045558221.1[S.salar]              ----------------------------GDAGEAARAPYSIPGILHFLQHEWARFEVERA      60
XP_041418671.1[X.laevis]             G--------AADAASVAGGGGGG---------SAGRAQYSIPGILHFLQHEWARFEVERA      77
XP_040524727.1[G.gallus]             A--------AAAAAAAGGGGGGVSAGGAAAPVSASRAQYSVPGILHFLQHEWGRFEAERA      89
NP_035630.2[M.musculus]              A----------------------AAGDGAAAAGAARAQYSLPGILHFLQHEWARFEVERA      68
O43815[H.sapiens]                    A----------------------AAGDGAAAAGAARAQYSLPGILHFLQHEWARFEVERA      68
XP_002812184.3[P.abelii]             A----------------------AAGDGAAAAGAARAQYSLPGILHFLQHEWARFEVERA      68
                                                                           :::**:*:::::*:.*** **:

XP_019858798.1[A.queenslandica]      SWEVERAELQGKIAFLQGERKGHDNLMRDLVRRIKMLEFALKQERNKVYKLTHGTDSGLS      116
NP_001296588.1[H.vulgaris]           HWEVERAELQARIAFLQGERKGQENLKRDLIRRIKMLEFALRKERQKFHKLKYGTELKLP      108
NP_609177.1[D.melanogaster]          QWDVDRAELQARIAMLLGERKCLESLKSDLTRRIKMLEYALRQERAKFYRLKYGTDPPQL      178
XP_032221713.1[N.vectensis]          HWEVERAELQARIAFLQGERKGQENLKHDLIRRIKMLEFALKQERAKYHRLKYGTDLGQE      110
XP_019631056.1[B.belcheri]           HWEVERAELQARIAFLQGERKGQENLKRDLVRRIKMLEYALKQERAKYHKLKYGTELNQG      120
XP_032803414.1[P.marinus]            SWDMERAELQARIAFLQGERKGQENLKKDLVRRIKMLEYALKQERAKYHKLKYGTELNQG      144
XP_045558221.1[S.salar]              QWEVQRAELQAQTAFLQGERKGQENLKKDLVRRIKMLEYALKQERAKYHKLKYGTELNQG      120
XP_041418671.1[X.laevis]             QWEVERAEMQAQIAFLQGERKGQENLKKDLVRRIKMLEYALKQERAKYHKLKYGTELNQG      137
XP_040524727.1[G.gallus]             EWEAERAELQAQIAFLQGERKGQENLKKDLVRRIKMLEYALKQERAKYHKLKYGTELNQG      149
NP_035630.2[M.musculus]              QWEVERAELQAQIAFLQGERKGQENLKKDLVRRIKMLEYALKQERAKYHKLKYGTELNQG      128
O43815[H.sapiens]                    QWEVERAELQAQIAFLQGERKGQENLKKDLVRRIKMLEYALKQERAKYHKLKYGTELNQG      128
XP_002812184.3[P.abelii]             QWEVERAELQAQIAFLQGERKGQENLKKDLVRRIKMLEYALKQERAKYHKLKYGTELNQG      128
                                      *: :***:*.: *:* ****  :.*  ** *******:**::** * ::*.:**:

XP_019858798.1[A.queenslandica]      LKPPQLDTET--------NPVTEESSLPSLVNQKESRQILRDYLREVGFPDNVLEARVAR      168
NP_001296588.1[H.vulgaris]           DIKDDEDH------DLANNEIISNKNHDLVFNYKQGRQLLRQYLQEIGFTDKVLEMRSAR      162
NP_609177.1[D.melanogaster]          NEFKPSNEDAGLAGEVATDSEV-PYSSVSNTTWRQGRQMLRQYLAEIGYTDNIIDVRSNR      237
XP_032221713.1[N.vectensis]          RSPPK-EQEQKE----EHPNEVTGSSNKPSMNWRQGRQLLRQYLQEVGYTDAILDVRSSR      165
XP_019631056.1[B.belcheri]           EMRPP-TPPSDE----ERETDSPL-SQNSQVTWRQGRQLLRQYLQEIGYSDTILDVRSNR      174
XP_032803414.1[P.marinus]            DMKPP-TFDAEE----TNDTDTPVVTSNSQLTWRQGRQLLRQYLQEVGYTDTILDVRSQR      199
XP_045558221.1[S.salar]              DMKPP-SYDSDE----VNENETTG-SLNNQLSWKQGRQLLRQYLQEVGYTDTILDVKSQR      174
XP_041418671.1[X.laevis]             DMKPP-NYDSDE----ANENEIQPPQQNSQFIWKQSRQLLRQYLQEVGYTDTILDVKSKR      192
XP_040524727.1[G.gallus]             DMKPP-NYDSDE----GNETEIQP-QQNSQLIWKQGRQLLRQYLQEVGYTDTILDVKSKQ      203
NP_035630.2[M.musculus]              DMKPP-SYDSDE----GNETEVQP-QQNSQLMWKQGRQLLRQYLQEVGYTDTILDVKSKR      182
O43815[H.sapiens]                    DMKPP-SYDSDE----GNETEVQP-QQNSQLMWKQGRQLLRQYLQEVGYTDTILDVKSKR      182
XP_002812184.3[P.abelii]             DMKPP-SYDSDE----GNETEVQP-QQNSQLMWKQGRQLLRQYLQEVGYTDTILDVKSKR      182
                                                                      ::.**:**:** *:*: * ::: :  :

XP_019858798.1[A.queenslandica]      LNLYRTLMSSSTNQNEPPL--P-----------PPAVEAPSETYSPKPPHPPESSTGGGS      215
NP_001296588.1[H.vulgaris]           IRSLLGITTDDLPTEDASTKSK--KNEKRQEDFTVSLNQEQ-N-----------------      202
NP_609177.1[D.melanogaster]          VRSILGLNNNAEHDGSGGGLGGGLGGGTGGENLSPNINGNE-SNKRASETE---------      287
XP_032221713.1[N.vectensis]          VRSILGLSAAEPLAPAQMNGTS-PRKPDDG---PEFIREPS-PQSREPVSVRQQAPSSHD      220
XP_019631056.1[B.belcheri]           VRTLLGLTPNESTKQDGEA--G-----------EVLVNGEQ-EREARR------------      208
XP_032803414.1[P.marinus]            VRSLLGLSGPPDQNGAPDAKNI-----------EQILNGRD-IMSIKP------------      235
XP_045558221.1[S.salar]              VKTLLAMAGDGGGRPGERTGSE-----------TPLVNGTD-TVPKGT------------      210
XP_041418671.1[X.laevis]             VRSLLGLSADSSDRLTDT--NQ-----------DSMVNGTE-TELPNP------------      226
XP_040524727.1[G.gallus]             VRALLGLSSDASEKE-NR--NQ-----------EPMVNGTE-GQIKEN------------      236
NP_035630.2[M.musculus]              VRALLGFSSDVTDREDDK--NQ-----------DSVINGTE-AEVKET------------      216
O43815[H.sapiens]                    VRALLGFSSDVTDREDDK--NQ-----------DSVVNGTE-AEVKET------------      216
XP_002812184.3[P.abelii]             VRALLGFSSDVTDREDDK--NQ-----------DSVVNGTE-AEVKET------------      216
                                     :.    :                             :.  .

XP_019858798.1[A.queenslandica]      DIVPNGTD----MQEQIGGRRDTEAESEDDAVKQVQNTFDFLSNEDSDDEDEETEWQQVA      271
NP_001296588.1[H.vulgaris]           ----------------------------------------CFDNPVDT----------DS      212
NP_609177.1[D.melanogaster]          -------GRHTP-AKKV-QQ--SIDEIIVDTEAAV-------------------------      311
XP_032221713.1[N.vectensis]          VVAPNPIRRAPPHIPPI-GK--KPADEDFESEAAVMANFDFLENDIEDEEENEDEVDDGG      277
XP_019631056.1[B.belcheri]           -----DADRRPVEKKRA-G------IPEIDSEASVLATFDFLSSEAVDDEDEENLSDGEE      256
XP_032803414.1[P.marinus]            ---------KEDDAKRN-G----DKSRSEPLVPSVVDTFQFLQEEEDSDDGEDDDDDDES      281
XP_045558221.1[S.salar]              ---------GTG------G-----RKTEVSESSAVLDTFRFYC-------DEDDDEDGAG      243
XP_041418671.1[X.laevis]             ---------LVL------G------KPDMTDSASVLETFKFLENAAAEFSDEEDEEENES      265
XP_040524727.1[G.gallus]             ---------AMI------G------KPELTDSASLLETFKFLENAAADFSDEEDEEDIEG      275
NP_035630.2[M.musculus]              ---------AMI------G------KSELTDSASVLDNFKFLESAAADVSDEDEDEDTDG      255
O43815[H.sapiens]                    ---------AMI------A------KSELTDSASVLDNFKFLESAAADFSDEDEDDDVDG      255
XP_002812184.3[P.abelii]             ---------AMI------G------KSELTDSASVLDNFKFLESAAADFSDEDEDDDVDG      255


XP_019858798.1[A.queenslandica]      T-----------------------KDLFQGLENTT-------EPAGLDKGAIMDGDFVYE      301
NP_001296588.1[H.vulgaris]           --TEVIEDS------VTKNSL----------------ESKKVEHETGDTFVLKDFDFLGN      248
NP_609177.1[D.melanogaster]          ---------------------------------------------------MANFEFLGA      320
XP_032221713.1[N.vectensis]          ---SADDED-DERDVLKTERRIIK--------KPK--TDSNEDLSPEEEEALDEFKFVMG      323
XP_019631056.1[B.belcheri]           DGGAADDES-EERE-QRRRREKGKKALVQMMENESLAGMEDVLADPETEEALAEFDFLVS      314
XP_032803414.1[P.marinus]            --VGLVDE--------RDRPRASKKA-----KIGNEGLAADLADDPDTEEALKEFDFLVT      326
XP_045558221.1[S.salar]              LDRTIMEQTGPTGTMVRKKPPSS--------SSPALSLSMDTSEDPDAEDALKGFDFLSS      295
XP_041418671.1[X.laevis]             RDKTIIDS----ATIVRRR------------------IIPDSND--DTEEALKEFDFLGS      301
XP_040524727.1[G.gallus]             REKTIIDT----ATIVRKR------------------VLSDSSEDRDTEEALKEFDFLVT      313
NP_035630.2[M.musculus]              RAKSVIDT----STIVRKK------------------ALPDTSEDRDTKEALKEFDFLVT      293
O43815[H.sapiens]                    REKSVIDT----STIVRKK------------------ALPDSGEDRDTKEALKEFDFLVT      293
XP_002812184.3[P.abelii]             REKSVIDT----STIVRKK------------------ALPDSGEDRDTKEALKEFDFLVT      293
                                                                                    :   .*:

XP_019858798.1[A.queenslandica]      DRYSSKSDN-P---------------------------PDLPIGEEDNEWEFKRQ--VER      331
NP_001296588.1[H.vulgaris]           EELNVDEASN--------------IQNEEQDDW----------------SNVDSEKLQNK      278
NP_609177.1[D.melanogaster]          TEMSDDDEI-S-----------------DDLE----------------------------      334
XP_032221713.1[N.vectensis]          DDDDMDSDSTVIEVPTGKDLDEVNIHMPDGEEGMAEQDHLHPDGTPKDEWDVDPSMLAKL      383
XP_019631056.1[B.belcheri]           EA-GEGAGE-AK-ST-------------DGAEW-----GL-DKSPTTEEWAMDKNLLNKL      352
XP_032803414.1[P.marinus]            TEDGEGAGE-ARSSG-------------DGTEW-----DKEELSPSGEEWDIDHGLITKL      367
XP_045558221.1[S.salar]              PDNMDTSPE-SRSAG-------------DGTDW-----EKEEQSAMSEAWDVDQGLITKL      336
XP_041418671.1[X.laevis]             DDSE--GGE-SRSAG-------------DGTEW-----EKEDQCPITESWNVDQGVITKL      340
XP_040524727.1[G.gallus]             SDEG--DGE-SRSSG-------------DGTDW-----EKEDQCLMPEAWNVDQGVITKL      352
NP_035630.2[M.musculus]              SEEG--DNE-SRSAG-------------DGTDW-----EKEDQCLTPEAWNVDQGVISKL      332
O43815[H.sapiens]                    SEEG--DNE-SRSAG-------------DGTDW-----EKEDQCLMPEAWNVDQGVITKL      332
XP_002812184.3[P.abelii]             SEEG--DNE-SRSAG-------------DGTDW-----EKEDQCLMPEAWNVDQGVITKL      332
```

```
XP_019858798.1[A.queenslandica]      YQEYKRSNKSKNISSRPPREQLRQLQQYANTNQPAEEMS--------SVMGLMGMPVRGH           383
NP_001296588.1[H.vulgaris]           IKQFKNETKDRGKKKRPNKDVMKNMLQTFNAG---EEEILPPPPQASTPSV---PRVSGG           332
NP_609177.1[D.melanogaster]          ------------------------MVATDNDD---TDVK-L-----AKR-----------           350
XP_032221713.1[N.vectensis]          KEQYKKERKSKKNVKRPPRSALQVMIANLGGD---EEMP-PQPQSTPAPATTGPSPVRLV           439
XP_019631056.1[B.belcheri]           KEEFKKERKSKKGAKRPNRSTLQAMLANLGGD---DDIV-MPA-SGPPRSAINNPDLG--           405
XP_032803414.1[P.marinus]            KEQYKKERKNKKGAKRPNRSALQDMLANLREV---DELS-PLAASSPPR-QPEERAAPLP           422
XP_045558221.1[S.salar]              KEEYRKERKGKKGGKRPNRSKLQDMLANLRDQ---EDMS-PREPSSSPQARSNAPRLNEQ           392
XP_041418671.1[X.laevis]             KEQYKKERKGKKGVKRPNRSKLQDMLANLREV---DELP-SLQPSVVSSSRPASSRISEQ           396
XP_040524727.1[G.gallus]             KEQYKKERKGKKGVKRPNRSKLQDMLANLRDV---DDLP-SLQPSVAPSSRPSSSRLIEH           408
NP_035630.2[M.musculus]              KEQYKKERKGKKGVKRPNRSKLQDMLANLRDV---DELP-SLQPSVGSPSRPSSSRLPEQ           388
O43815[H.sapiens]                    KEQYKKERKGKKGVKRPNRSKLQDMLANLRDV---DELP-SLQPSVGSPSRPSSSRLPEH           388
XP_002812184.3[P.abelii]             KEQYKKERKGKKGVKRPNRSKLQDMLANLRDV---DELP-SLQPSVGSPSRPSSSRLPEH           388
                                                             :           :

XP_019858798.1[A.queenslandica]      HDLEGV-------------------------RVSPHRIPDERPEVSLGIGELVSIANPS           417
NP_001296588.1[H.vulgaris]           ---VVDDEIPVIGDTVLPSSGYSPRQVK---LVAVNHSGVSDVTEVAMGLGDLADLSVSN           386
NP_609177.1[D.melanogaster]          -----------------A---------------KSGKDMLTEEVDGSLGLGELAQLTVNN           378
XP_032221713.1[N.vectensis]          ----GSR----AMSEIPPPIGSVKIPTKTEAAPAASSPPKNEPFEDALGLGELAELTVNN           491
XP_019631056.1[B.belcheri]           ----PLDEP--RKEGGPEEQV------------LGVGGPEEESLEAALGLGDLAGLTVTN           447
XP_032803414.1[P.marinus]            HDNARSD-E--GESPFPRGTG------------KMFISGPEEAMD-TTGLGELADLTVTN           466
XP_045558221.1[S.salar]              HQDNRTDEV--EALTFPPSSG------------KAFIMGPDENMEAELGLGELAGLTVAN           438
XP_041418671.1[X.laevis]             -EINRADEV--EALTFPPSSG------------KSFIMGADEALENELGLGELAGLTVAN           441
XP_040524727.1[G.gallus]             -EINRTDEV--EALTFPPSSG------------KSFIMGTDETLENELGLGELAGLTVAN           453
NP_035630.2[M.musculus]              -ELSRADEV--EALTFPPSSG------------KSFIMGADEALESELGLGELAGLTVAN           433
O43815[H.sapiens]                    -EINRADEV--EALTFPPSSG------------KSFIMGADEALESELGLGELAGLTVAN           433
XP_002812184.3[P.abelii]             -EINRADEV--EALTFPPSSG------------KSFIMGADEALESELGLGELAGLTVAN           433
                                                                              :  :   *:*:*. ::  .

XP_019858798.1[A.queenslandica]      --D---FIMN--------PSTSGSSKRWEHKYTMRNHYDAVTCVQFHPMDDMLVTGSEDA           464
NP_001296588.1[H.vulgaris]           ELGNL-------------DSKDDMRKTWGPKYTLRSHFDGVRSLCFHPYEAALITGSEDH           433
NP_609177.1[D.melanogaster]          ESDGA-YDANSKDGTGGSAGGAGYRKTWNAKYTLRSHFDGVRSLIFHPEEPVLITASEDH           437
XP_032221713.1[N.vectensis]          EAEALSYEIN---------KKDEFRKTWNPKFTLRSHFDSIRSVYFHPQEAAVITASEDH           542
XP_019631056.1[B.belcheri]           EAEPLQYDVA--------ATKEAFRKTWNPKYTLRSHFDAVRALAFHPIEPVLITASEDH           499
XP_032803414.1[P.marinus]            DADSPSYDIG--------ANKDAFRKTWNPKFTLRSHFDGVRALAFHPSEHVLVTASEDH           518
XP_045558221.1[S.salar]              EADSLAYDMS--------NNKDALRRTWNPKFTLRSHFDGIRGLAFHPVEPVLVTASEDH           490
XP_041418671.1[X.laevis]             EADSLTYDIA--------NNKDALRKTWNPKFTLRSHFDGIRGLAFHPSEPVLITASEDH           493
XP_040524727.1[G.gallus]             EADSLTYDIG--------NNTDALRKTWNPKFTLRSHFDGIRGLAFHPIEPVLITASEDH           505
NP_035630.2[M.musculus]              EADSLAYDIA--------NNKDALRKTWNPKFTLRSHFDGIRALAFHPIEPVLITASEDH           485
O43815[H.sapiens]                    EADSLTYDIA--------NNKDALRKTWNPKFTLRSHFDGIRALAFHPIEPVLITASEDH           485
XP_002812184.3[P.abelii]             EADSLTYDIA--------NNKDALRKTWNPKFTLRSHFDGIRALAFHPIEPVLITASEDH           485
                                                              : *  *:*:*.*:*.:  : *** :  ::*.***

XP_019858798.1[A.queenslandica]      TIKLWSIPKSS-QKKSAIIDVEPAFTFRGHTGPVLSLAVSSDGEVVYSGSADGQLRMWQT           523
NP_001296588.1[H.vulgaris]           TLKLWSLQKNVQGKRGNISDLEPVYTFRGHSDGVLCCAINSNGTVCYSGGLDFKIQCWNL           493
NP_609177.1[D.melanogaster]          TLKLWNLQKTVQAKKSASLDVEPLYTFRAHTGPVLCLGMSSSGETCYSGGLDGNIECWQL           497
XP_032221713.1[N.vectensis]          TMKLWNLQKTVPQKKSNILDVEPIYTFRGHSAAVLSVVISSTSDMCFSGGADGTILCWNI           602
XP_019631056.1[B.belcheri]           TLKMWNLQKTVPAKKSASLDVEPIYTFRAHSGPVTCLCVSSNGDQCYSGSTDSTIRCWNI           559
XP_032803414.1[P.marinus]            TLKLWNLQKTISGKKTASLDVEPIYTFRAHTGPVLSLAISTSGEQCFSGGTDASIRCWNM           578
XP_045558221.1[S.salar]              TLKMWNLQKTAPAKKSASVDMEPMYTFRAHQGAVLCVVMSSTGEQCFSGGVDGTIQSWNT           550
XP_041418671.1[X.laevis]             TLKMWNLQKTAPAKKSTSLDIEPIYTFRAHRGPVLCVVMSTNGEQCYSGGTDGVIQSWNT           553
XP_040524727.1[G.gallus]             TLKMWNLQKTAPAKKSASLDVEPIYTFRAHSGPVLCVVMSSNGEQCYSGGTDGLIHGWNT           565
NP_035630.2[M.musculus]              TLKMWNLQKTAPAKKSTSLDVEPIYTFRAHKGPVLCVVMSSNGEQCYSGGTDGRIQSWST           545
O43815[H.sapiens]                    TLKMWNLQKTAPAKKSTSLDVEPIYTFRAHKGPVLCVVMSSNGEQCYSGGTDGLIQGWNT           545
XP_002812184.3[P.abelii]             TLKMWNLQKTAPAKKSTSLDVEPIYTFRAHKGPVLCVVMSSNGEQCYSGGTDGLIQGWNT           545
                                     *:*:*.: *.   *:    *:** :***.*   * .  :.: .   :**. *  :  *.

XP_019858798.1[A.queenslandica]      PNDLSDPFDIYDLDIQKGVLEGHTDAIWGLVFNQSNGLLASASADGHCILWDPV--NSSQ           581
NP_001296588.1[H.vulgaris]           PSLNIDLFGPYDPNVLGDALIGHSDSVWDLAYHIDTDFLLSCSADGTCKLWNPS--IKSP           551
NP_609177.1[D.melanogaster]          PSPNIDPYDCYDPNVHSGTLEGHTDAVWGLTT--MQSNIVSCSADGTVKLWSPY--NKEP           553
XP_032221713.1[N.vectensis]          PSLDLDPYGPYSPNVTGALLEVHTDAVWGLAVQTNSLQLLSCSSDGTCRLWNPT--LKSP           660
XP_019631056.1[B.belcheri]           PSPNIDPYDAFDPTVLGQALVGHTDAIWGVTIHSVKQQLLSCSADGTVRLWSLQ--NKSP           617
XP_032803414.1[P.marinus]            PSPNIDPYDTFEASVLGQTLLGHSDAVWGLAYSSLRRRLLSCSADGTVRLWDPSCQDSPP           638
XP_045558221.1[S.salar]              PNPNLDPYDSYEPTVLRGALCGHTDLVWGLVYSGVHQRLLSCSGDGTVRLWDAS--NTKP           608
XP_041418671.1[X.laevis]             PNSNVDPYDSYDPTVLRGAYEGHTDAVWGLVYSAVHQRLLSAAADGTIRIWNAT--EVSP           611
XP_040524727.1[G.gallus]             TNPNIDPYDSYDPSVLRGAFVGHTDAVWGLVYSGTHQRLLSCSADGTVRLWKAT--EVAP           623
NP_035630.2[M.musculus]              TNPNVDPYDAYDPSVLRGPLLGHTDAVWGLAYSAAHQRLLSCSADGTLRLWNTT--EVAP           603
O43815[H.sapiens]                    TNPNIDPYDSYDPSVLRGPLLGHTDAVWGLAYSAAHQRLLSCSADGTLRLWNTT--EVAP           603
XP_002812184.3[P.abelii]             TNPNIDPYDSYDPSVLRGPLLGHTDAVWGLAYSAAHQRLLSCSADGTLRLWNTT--EVAP           603
                                      .   * :. :.  :       *:* :*.:.       : *.:.**   :*.

XP_019858798.1[A.queenslandica]      IKS-IV-SEEALGSPTSIDFLHGE--SIVVSYSTAKVVVYDVETGKPVVTLDSALTYNGT           637
NP_001296588.1[H.vulgaris]           LLETYKSP--ESGTPTSVSFVCTDTGQVAASYTSESCVVYDIETAKPIVTFSPEKTYG--           607
NP_609177.1[D.melanogaster]          LLRTYTASEAE-GVPSSVDFVRNEVDHIVVAYNSAHCIVYDTETGKQVVRLEAAQEMSGN           612
XP_032221713.1[N.vectensis]          LLNTFILDKESSCTPTSIDFLRMDTSQMVASYSSARAAIFDLETAEAVVNLDSAKTYNNS           720
XP_019631056.1[B.belcheri]           LLNTYTAEKDD-GAPTSVDFVRCELSQMVAAYSSSVAVLYDLETAQPVAKLDARQGSDSG           676
XP_032803414.1[P.marinus]            CLNTYNPDKEH-GIPVSVDFVS-DASHLVATFGGGDTLVFDMETAKPVITLESRIETDTP           696
XP_045558221.1[S.salar]              ALATFQ-NKEF-GVPSSVDLVCSDPAHMVTSFTSGQIGLFNMETQQLVLSLESAAE--TG           664
XP_041418671.1[X.laevis]             ALSIFNENQEL-GIPSSVDLVSCDPAHLVASFNNGHTNIFNMETHQKVLTLESCIDTAVN           670
XP_040524727.1[G.gallus]             ALNVFNDNQEM-GIPSSVDLVSSDPSLMVASFNTGHTSIFNMETRQRILTLESGVDNTVS           682
NP_035630.2[M.musculus]              ALSVFNDNQEL-GIPASVDLVSSDPSHMVASFSKGYTSIFNMETQQRVLTLESNVDSTSS           662
O43815[H.sapiens]                    ALSVFNDTKEL-GIPASVDLVSSDPSHMVASFSKGYTSIFNMETQQRILTLESNVDTTAN           662
XP_002812184.3[P.abelii]             ALSVFNDTKEL-GIPASVDLVSSDPSHMVASFSKGYTSIFNMETQQRILTLESNVDTTAN           662
                                                   * *:.::  :   :..::      ::: ** : :  :.

XP_019858798.1[A.queenslandica]      PTTQINKVLSHPTLPIVITAHEDKYICFFDSKSGQVTHSMTAHMDAVTGLAIDPHGLYIL           697
NP_001296588.1[H.vulgaris]           -LSQINKVISHPTHPILITAHEDRHVRFFDLSSGKEVHSMVAHLDAVTSLSIDPSGLYIL           666
NP_609177.1[D.melanogaster]          TGKFINKVVSHPTLPITITAHEDRHIRFWDNTSGTLVHSMVAHLEPVTSLAVDAHGLYLL           672
XP_032221713.1[N.vectensis]          PSTQINKVVSHPTLPVTITAHEDRHIRFFDNNTGKQIHSMVAHLDAVTSLAVDPNGLYLL           780
XP_019631056.1[B.belcheri]           VSSQINRVVAHPTLPITITAHEDRHIKFFDNNSGKMVNTMVAHLDAVTSLAVDPNGLYLL           736
XP_032803414.1[P.marinus]            LGNQINRVVSHPTLPITVTAHEDRHIKFFDNNTGKQVHSMVAHLDAVTCLAVDPNGLYLM           756
XP_045558221.1[S.salar]              TPCQINKVLSHPTLPITITAEEDRHIKFFDNNTGKLIHSMVAHLDAVTSLAVDPNGLYLM           724
XP_041418671.1[X.laevis]             STCQINRVISHPTLPISITAHEDRHIKFYDNNTGKLIHSMVAHLDAVTSLAVDPNGLYLM           730
XP_040524727.1[G.gallus]             TSCQINRVISHPTLPISITAHEDRHIKFYDNNTGKLIHSMVAHLDAVTSLAVDPNGLYLM           742
NP_035630.2[M.musculus]              SSCQINRVISHPTLPISITAHEDRHIKFYDNNTGKLIHSMVAHLEAVTSLAVDPNGLYLM           722
O43815[H.sapiens]                    SSCQINRVISHPTLPISITAHEDRHIKFYDNNTGKLIHSMVAHLEAVTSLAVDPNGLYLM           722
XP_002812184.3[P.abelii]             SSCQINRVISHPTLPISITAHEDRHIKFYDNNTGKLIHSMVAHLEAVTSLAVDPNGLYLM           722
                                         **:*::*** *: :**.**::: *:* .:*   ::*.**:: ** *::*  ***::

XP_019858798.1[A.queenslandica]      SGSHDGSLRFWSMETKTCVQEITAHRKRFDESIYNVTCHLTKPFFASAGADGIAKVLL 755
NP_001296588.1[H.vulgaris]           SGSHDGSIRLWSMENKTCIQEITAHRKKFDEAVFDVAFHPSQPYIASAGADALAKVFV 724
NP_609177.1[D.melanogaster]          SGSHDCSIRLWNLDNKTCVQEITAHRKKFDESIFDVAFHATKPYIASAGADGLAKVFV 730
XP_032221713.1[N.vectensis]          SGSHDCSIRLWNLDSKTCVQEITSHRKKFDESIYDVAFHPTKPYIASAGADGLAKVFV 838
XP_019631056.1[B.belcheri]           SGSHDCSIRLWNLDSKTCVQEITSHRKKFDESIFDVAFHPSKPYIASAGADALAKVFV 794
XP_032803414.1[P.marinus]            SGSHDCSIRLWNLESKTCVQEITAHRKKFDEAIHDVAFHPSKAFIASAGADALAKVFV 814
XP_045558221.1[S.salar]              SGSHDCSIRLWNLETKTCIQEFTAHRKKNDESIHDVAFHPSKAYIASAGADALAKVFV 782
XP_041418671.1[X.laevis]             SGSHDCSIRLWNLESKTCIQEFTAHRKKSDESIHDVAFHPSKCYIASAGADALAKVFV 788
XP_040524727.1[G.gallus]             SGSHDCSIRLWNLESKTCIQEFTAHRKKFDESIHDVAFHPSKCYIASAGADALAKVFV 800
NP_035630.2[M.musculus]              SGSHDCSIRLWNLESKTCIQEFTAHRKKFEESIHDVAFHPSKCYIASAGADALAKVFV 780
O43815[H.sapiens]                    SGSHDCSIRLWNLESKTCIQEFTAHRKKFEESIHDVAFHPSKCYIASAGADALAKVFV 780
XP_002812184.3[P.abelii]             SGSHDCSIRLWNLESKTCIQEFTAHRKKFEESIHDVAFHPSKCYIASAGADALAKVFV 780
                                     ***** *:*:*.::.***:**:*:***: :*::.:*: * :: ::******.:***::
```
