# Supplementary material for: An Evolutionary Perspective on the Origin, Conservation and Binding Partner Acquisition of Tankyrases
Source: Biomolecules. 2022 Nov 15;12(11):1688. doi: 10.3390/biom12111688 (PMC9688111; doi:10.3390/biom12111688)
Supplement: Supplementary file 1 [file biomolecules-12-01688-s001.zip › biomolecules-1913719-supplementary-11.17 -done/Data_7/TAB182.pdf]

TAB182

Homo sapiens:

>NP_203754.2 182 kDa tankyrase-1-binding protein [Homo sapiens]
MKVSTLRESSAMASPLPREMEEELVPTGSEPGDTRAKPPVKPKPRALPAKPALPAKPSLLVPVGPRPPRG
PLAELPSARKMNMLAGPQPYGGSKRPLPFAPRPAVEASTGGEATQETGKEEAGKEEPPPLTPPARCAAPG
GVRKAPAPFRPASERFAATTVEEILAKMEQPRKEVLASPDRLWGSRLTFNHDGSSRYGPRTYGTTTAPRD
EDGSTLFRGWSQEGPVKSPAECREEHSKTPEERSLPSDLAFNGDLAKAASSELPADISKPWIPSSPAPSS
ENGGPASPGLPAEASGSGPGSPHLHPPDKSSPCHSQLLEAQTPEASQASPCPAVTPSAPSAALPDEGSRH
TPSPGLPAEGAPEAPRPSSPPPEVLEPHSLDQPPATSPRPLIEVGELLDLTRTFPSGGEEEAKGDAHLRP
TSLVQRRFSEGVLQSPSQDQEKLGGSLAALPQGQGSQLALDRPFGAESNWSLSQSFEWTFPTRPSGLGVW
RLDSPPPSPITEASEAAEAAEAGNLAVSSREEGVSQQGQGAGSAPSGSGSSWVQGDDPSMSLTQKGDGES
QPQFPAVPLEPLPTTEGTPGLPLQQAEERYESQEPLAGQESPLPLATREAALPILEPVLGQEQPAAPDQP
CVLFADAPEPGQALPVEEEAVTLARAETTQARTEAQDLCRASPEPPGPESSSRWLDDLLASPPPSGGGAR
RGAGAELKDTQSPSTCSEGLLGWSQKDLQSEFGITGDPQPSSFSPSSWCQGASQDYGLGGASPRGDPGLG
ERDWTSKYGQGAGEGSTREWASRCGIGQEEMEASSSQDQSKVSAPGVLTAQDRVVGKPAQLGTQRSQEAD
VQDWEFRKRDSQGTYSSRDAELQDQEFGKRDSLGTYSSRDVSLGDWEFGKRDSLGAYASQDANEQGQDLG
KRDHHGRYSSQDADEQDWEFQKRDVSLGTYGSRAAEPQEQEFGKSAWIRDYSSGGSSRTLDAQDRSFGTR
PLSSGFSPEEAQQQDEEFEKKIPSVEDSLGEGSRDAGRPGERGSGGLFSPSTAHVPDGALGQRDQSSWQN
SDASQEVGGHQERQQAGAQGPGSADLEDGEMGKRGWVGEFSLSVGPQREAAFSPGQQDWSRDFCIEASER
SYQFGIIGNDRVSGAGFSPSSKMEGGHFVPPGKTTAGSVDWTDQLGLRNLEVSSCVGSGGSSEARESAVG
QMGWSGGLSLRDMNLTGCLESGGSEEPGGIGVGEKDWTSDVNVKSKDLAEVGEGGGHSQARESGVGQTDW
SGVEAGEFLKSRERGVGQADWTPDLGLRNMAPGAVCSPGESKELGVGQMDWGNNLGLRDLEVTCDPDSGG
SQGLRGCGVGQMDWTQDLAPQNVELFGAPSEAREHGVGGVSQCPEPGLRHNGSLSPGLEARDPLEARELG
VGETSGPETQGEDYSSSSLEPHPADPGMETGEALSFGASPGRCPARPPPSGSQGLLEEMLAASSSKAVAR
RESAASGLGGLLEEEGAGAGAAQEEVLEPGRDSPPSWRPQPDGEASQTEDVDGTWGSSAARWSDQGPAQT
SRRPSQGPPARSPSQDFSFIEDTEILDSAMYRSRANLGRKRGHRAPVIRPGGTLGLSEAADSDAHLFQDS
TEPRASRVPSSDEEVVEEPQSRRTRMSLGTKGLKVNLFPGLSPSALKAKLRPRNRSAEEGELAESKSSQK
ESAVQRSKSCKVPGLGKPLTLPPKPEKSSGSEGSSPNWLQALKLKKKKV

Pongo abelii:

>XP_002821835.2 182 kDa tankyrase-1-binding protein [Pongo abelii]
MKVSTLRESSAMASPLPREMEEELVPTGSEPGDTRAKPPVKPKPRALPAKPALPAKPSLLVPVGPRPPRG
PLAELPSARKMNMLAGPQPYGGSKRPLPFAPRPAVEASTGGEATQETGKEEAGKEEPPPLTPPARCAAPG
GVRKAPAPFRPASERFAATTVEEILAKMEQPRKEVPASPDRLWGSRLTFNHDGSSRYGPRTYGTTTAPRD
EDGSTLSRGWSQEGPVKFPAECQEEHRKTPEERSLPSDLAFNGDLAKAASSELPADISKPWIPSSPAPSS
ENGGPASPGLPAEASGSGPGSPHLHSPDKSSPCHSQLLEAQSPEASQASPCSPVTPTAPSAALPDEGSRH
TPSPGLPAEGAPEAPRPSSPPPEVLEPHSLDQPPATSPQPVIEVGELLDLTRTFPSGGEEEAKGDAHLRP
TSLVQRRFSEGVLQSPSQDQEKLGGSLAALPQGQGSQLALDRPFGAESNWSLSESFEWTFPTRPSGLGVW
RLDSPPPSPITEASEAAEAAEAGNLAVSSGEEGVSQQGQGAGSAPSGSGSSWVQGDDPSMSLTQKGDGES
QPQFPAVPLEPLPTTEGTPGLPLQQAEERYESQEPLAGQESPLPLATREAALPILEPVLGQEQPAPPDQP
CVLFADVPEPGQALPVEEEAMTLDRAETTQPRTEAQDLCRASPEPPGPESSSRWLDDLLASPPPSGGSAR
RGAGSELKDAQSPSTCSEGLLGWSQKDLQSEFGITGDPQPSSFSPSSWCQGASQDYGLGGASPRGDPGLG
ERDWTSKYGQGAGEGSTRDWASRCGIGQEEMEASSSQDQSEVSAPGALTAQDRVVGKPAQLGTQRSQEAD
VQDWEFRKRDSQGTYSSRDAELQDQEFGKRDSLGTYSSRDVSLGDWEFGKRDSLGTYASQDVNEQGQDLG
KRDHRGRYSSQDADEQDWKFQKRDVSLGTYGSRGAEPQEQEFGKSAWIRDYSSGGSSRTLDAQDRSFGTR
PLSSGFSPEEAQQQDEEFEKKIPSVEDSLGEGSRDAGRPGERESGGLFSPSTAHVPDGALGQRDQSSWQN
SDASQEVGGHQERQQAGAHGPGSADLEDGEMGKRGWVGEFSLSVGPRREAAFSPGQQDWSRDFCVEASER
SYQFGIIGNDRVSGAGFSPSSKMGGGHFVPPGKTTAGSVDWTDQLGLRNLEVSSCVGSGGSSEARENAVG
QMGWSGGLSLRDMNLAGCLESGGSEEPGGIGVGEKDWTSDVNVKSKDLAEVGEGGGHSQARESGVGQTDW
SGVEAGEFLKSRERGVGQADWTPDLGLRNMAPGAVCSPGEHKELGVGQMDWGNNLGLRDLEVTCDPESGD
SQGLRGCGVGQMDWTQDLAPRNVELFGAPSEAREHGVGGVSQCPEPGLRHNGSLSPGLEARDPLEARELG
VGETSGPETQGEDDSSFSLEPHPADPGMETGEALSFGASPGRRPARPPPSGSQGLLEEMLAASSSKAVAQ
RESAASGLGGLLEEEGAGAGAAQEEVLEPGRDSPPSWRPQPDGEASQTEDVDGTWGSSAARWSDQGPAQT
SRRPSQGPPARSPSQDFSFIEDTEILDSAMYRSRANLGRKRGHRAPAIRPGGTLGLSEAADSDARLFQDS
TEPRASRVPSSDEEVVEEPQSRRTRMSLGTKGLKVNLFPGLSPSALKAKLRPRNRSAEEGEPAESKSSQK
ESAVQRSKSCKVSGLGKPLTLPPKPEKSSGSEGSSPNWLQALKLKKKKV

Mus musculus:

>NP_001074729.1 182 kDa tankyrase-1-binding protein [Mus musculus]
MKGSTLREGTAMASPLPQDMEEELAPVGSEPGDPRAKPPVKPKPRGLPSKPALPAKPSLLVPVGPRPPRG
PLAELPSARKMNMLAGPQPYGVSKRPLPFAPRPSAEATAGGDVTQESGKEDAGKEDLPPLTPPARCAALG
GVRKAPAPFRPSSERFAACTVEEILAKMEQPRKEILASPDRLWGSRLTFNHDGSSRYGPRTYGAPCPREE
DSKSPAKGRSQEGTAEIPAECQEEHSKTPEERNLTSSPAMNGDLAKLACSEAPTDVSKTWVTSSADPVSE
HGGSTSAVRLANISVPASESPRLSSRPSSPCHSQLSETQSPAASEASSICLPVTPASPSAVLPAEPPGHS
PSSELPAEAAPETLSPNSSPVETVSGHHSPEQPPVLLPQLLTEGAELPDITRTFPCGEEAAARGHTESRP
SSLAQRRFSEGVLQPPSQDQEKLGGSLATLPQGQGSQSALDRPFGSGTESNWSLSQSFEWTFPTRPSGLG
VWRLDSPPPSPITEASEAAEAAEADSWAVSGRGEGVSQVGPGTPPAPESPRKPISGVQGNDPGISLPQRD
DGESQPRSPALLPSTVEGPPGAPLLQAKENYEDQEPLVGHESPITLAAREAALPVLEPALGQQQPTPSDQ
PCILFVDVPDPEQALSTEEDVVTLGWAETTLPMTEAQEPCSVSPEPTGPESSSRWLDDLLASPPPNSGSA
RRAAGAELKDRQSPSTCSEGLLGWAQKDLQSEFGVATDSHHSSFGSSSWSQDTSQNYSLGGRSPVGDTGL
GKRDWSSKCGQGSGEGSTREWASRHSLGQEVIGIGGSQDESEVPVRERAVGRPAQLGAQGLEADAQQWEF
GKRESQDPHSIHDKELQDQEFGKRDSLGSFSTRDASLQDWEFGKRASVSTNQDTDENDQELGMKNLSRGY
SSQDAEEQDREFEKRDSVLDIHGSRATAQQNQEFGKSAWFQDYSSGGGGSRVLGSQERGFGIRSLSSGFS
PEEAQQQDEEFEKKTPVGEDRFCEASRDVGHLEEGASGGLLSPSTPHSRDGAARPKDEGSWQDGDSSQEI
TRLQGRMQAESQSPTNVDLEDKEREQRGWAGEFSLGVAAQSEAAFSPGRQDWSRDVCVEASESSYQFGII
GNDRVSGAGLSPSRKSGGGHFVPPGETKAGAVDWTDQLGLRNLEVSSCVSSEGPSEARENVVGQMGWSDS
LGLNNGDLARRLGTGESEEPRSLGVGEKDWTSSVEARNRDLPGQAEVGRHSQARESGVGEPDWSGAEAGE
FLKSRERGVGQADWTPDLGLRNMAPGAGCSPGEPRELGVGQVDWGDDLGLRNLEVSCDLESGGSRGCGVG
QMDWAQDLGLRNLRLCGAPSEVRECGVGRVGPDLELDPKSSGSLSPGLETEDPLEARELGVGEISGPETQ
GEDSSSPSFETPSEDTGMDTGEAPSLGASPSSCLTRSPPSGSQSLLEGIMTASSSKGAPQRESAASGSRV
LLEEEGLAAGAGQGEPQEPSRAPLPSSRPQPDGEASQVEEVDGTWSLTGAARQNEQASAPPPRRPPRGLL
PSCPSEDFSFIEDTEILDSAMYRSRANLGRKRGHRAPAIRPGGTLGLSETADSDTRLFQDSTEPRASRVP
SSDEEVVEEPQSRRTRMSLGTKGLKVNLFPGLSPSALKAKLRSRNRSAEEGEVTESKSSQKESSVQRSKS
CKVPGLGKPLTLPPKPEKSSGSEGSSPNWLQALKLKKKKI

Gallus gallus:

```
>XP_040557690.1 182 kDa tankyrase-1-binding protein isoform X1 [Gallus gallus]
MASQPQSLHPPLPCASGGAGLAAGSPEKGSPRPKPPVRPKPRVLPKPAVPAKPCLPHPPPAPRHSRPELP
SAEKINRLAGPQPYSGGGSGAALRRPSFTIKSPETPNGKGLPSPPVLTPEDSCSTPSDEVPLAPSTPSRK
GPAPFKVTPVPVATKLERFPGTTVEEILAKMDSKEGLGSPDRARLSPFCPDSSSRFGSKTFTAFRRRPSG
EADGDSPGEARQLLQQAEGELGTGGDRCLMVETSSSSPAGPSCAGDPCGLRRPPSPPDLSSLQLGPPGSP
RPPACPPPGAPFQPSASAPGSPDAPPELLAPGSPTMAPESPESPAQRPTEELLATSIQAPGAPSSVAEPW
LGASHSPGSPHTPSEGSPTPASPSTPGTSQLSPKATCPPGSPESATEPSPPPSPPPELPARASCPPGSPG
GPDDSLVSSQTPEGPDFSLPPSNRDSGLRRSSEGVLRPPPSEGQGMGLLGGSLGALPQPGDLPAEHLLSG
ESSWSLSQSFEWTFPTRGVRGLPSPPRSPIQEAADSGLSEEDESDREGAATGSHGDSGDTDGPGLAVTSS
WGAEGAPCPGGPVAQQEAESSEEEEEEGEAELDSTELHIVEPSLDSAEPEPPVKAAPPDPAPTEADASCE
PTGDFPASLQGLGGPGQDEGSSKGPDAHTDPRWLTELLASPRARASGRDPLNTEEQEGLLGWSRKDLCSE
FGIGRSHQAGAFDWSHKAVARQGDWPGEMEQDQKFRTNSSRNNSCGISDMNQQDRAFGAAERDWSSNYKG
TELMGDTRLGSSDWSKSHGAGESCLQDQDFSKPTWGTGYGLDSAGSREEVGSGKAEWSSPYSVGHGQQQD
KELSSRQPGWAGKYGSGDLETHSGDAAPVWSSEYGTGDADIKDREVTPDWASKYSSRDAETKDKDFTLGW
AGRSSTGDAGTLDKEFCSSRSAWDSKYSTRDMESQDREFSPSRPAWSGEYSTQDMESQDREFSPSRLAEA
SEYSSRDMEIQDREFSPSRPACAEEHSTSDMESQDREFSTSRPTWHREYSSRDMESQDREFSPSRPACTN
EYSTSDAETQDREFNPSRPTWHSEYSSRDMETQDREFSPSRPACADEYITKNMESQDREFSPNRPAEASE
YSARDVETQDRELKSNKPAWDDEYITKNMESRDREFGPSRSVWASDYDSMNTEKGEFSSSRLSWAGECSI
GQTQLANAFGVRKEDLPGLCAPSRPGQESSWGSTDQQESGTRDWAEELHLGGAEHQNQFGIIGTERVSDP
SSTAASADGLMSWANKMRSEVPQEPRQPLGDWHSDLSFSTSGITGKADEGEPSQMAWDEGLAAVGSSAQP
QQAKVEEPGWSQDLEEAGWSEELREAEARRQEWASAFGARCAARSQDFSAGEQSLGGDGGSADGNPENIH
LSESSPPLGDDAVAEPAAAEPIWGESATPRTDPMEQQDLPELAAAPLPAEAAGETPETESEEAPLDSPDG
KTPVSWEEKRLSVSASQPEGLLDLSGQDFTFLEDTEVLDSTMYRSKANLGRKRRHRAPVLRPGATSEGDS
WIFQDSTEPRPARAAASSDEEAAEEPKSRRVRASPSGKGVKVPLFPGLTTSALKAKLRGRNRSVEEGASP
VDSKATPTKDPHVQRSKSCKIPGLSGKPLALPPKPEKSSGSDASPPHWLQALKLKKKKS
```

Xenopus laevis:

No homolog found.

Salmo salar:

No homolog found.

Petromyzon marinus:

No homolog found.

Branchiostoma belcheri:

No homolog found.

Drosophila melanogaster:

No homolog found.

Hydra vulgaris:

No homolog found.

Nematostella vectensis:

No homolog found.

Amphimedon queenslandica:

No homolog found.

Alignment

```
XP_040557690.1[G.gallus]        ----MASQPQSLHPPLPCASGGAGLAAGSPEKGSPRPKPPVRPKPRVLP-KPAVPAKPCL        55
NP_001074729.1[M.musculus]      MKGSTLREGTAMASPLPQDME-EELAPVGSEPGDPRAKPPVKPKPRGLPSKPALPAKPSL        59
NP_203754.2[H.sapiens]          MKVSTLRESSAMASPLPREME-EELVPTGSEPGDTRAKPPVKPKPRALPAKPALPAKPSL        59
XP_002821835.2[P.abelii]        MKVSTLRESSAMASPLPREME-EELVPTGSEPGDTRAKPPVKPKPRALPAKPALPAKPSL        59
                                       :  ::  ***       *.  . * *. * ****:**** ** ***:****.*

XP_040557690.1[G.gallus]        P--HPPPAPRHSRPELPSAEKINRLAGPQPYSGGGSGAALR-RPSFTIK------SPETP        106
NP_001074729.1[M.musculus]      LVPVGPRPPRGPLAELPSARKMNMLAGPQPYGVSKRPLPFAPRPSAEATAGGDVTQESGK        119
NP_203754.2[H.sapiens]          LVPVGPRPPRGPLAELPSARKMNMLAGPQPYGGSKRPLPFAPRPAVEASTGGEATQETGK        119
XP_002821835.2[P.abelii]        LVPVGPRPPRGPLAELPSARKMNMLAGPQPYGGSKRPLPFAPRPAVEASTGGEATQETGK        119
                                      *  **    *****.*:* *******. .     :  **:   .      .

XP_040557690.1[G.gallus]        NGKGLPSPPVLTPEDSCSTPSDEVPLAPSTPSRKGPAPFKVTPVPVATKLERFPGTTVEE        166
NP_001074729.1[M.musculus]      EDAGKEDLPPLTPPARCAALG----------------GVRKAPAPFRPSSERFAACTVEE        163
NP_203754.2[H.sapiens]          EEAGKEEPPPLTPPARCAAPG----------------GVRKAPAPFRPASERFAATTVEE        163
XP_002821835.2[P.abelii]        EEAGKEEPPPLTPPARCAAPG----------------GVRKAPAPFRPASERFAATTVEE        163
                                :  *  . * ***   *:: .                 .: :*.*.    *** . ****

XP_040557690.1[G.gallus]        ILAKMDS--KEGLGSPDRARL--SPFCPDSSSRFGSKTFTAFRRRPSGEADGDSPGEARQ        222
NP_001074729.1[M.musculus]      ILAKMEQPRKEILASPDRLWGSRLTFNHDGSSRYGPRTYGAP-CPRE--EDSKSPAKGR-        219
NP_203754.2[H.sapiens]          ILAKMEQPRKEVLASPDRLWGSRLTFNHDGSSRYGPRTYGTTTAPRD--EDGSTLFRGW-        220
XP_002821835.2[P.abelii]        ILAKMEQPRKEVPASPDRLWGSRLTFNHDGSSRYGPRTYGTTTAPRD--EDGSTLSRGW-        220
                                *****:.  **  .****       *  *.***:* :*: :     .   *..:  ..

XP_040557690.1[G.gallus]        LLQQAEGELGTGGDRCLMVE----TSSSSPAGPSCAGDPCGLRRPPSPPDLSSLQLGPPG        278
NP_001074729.1[M.musculus]      -SQ--EGTAEI-PAECQEEHSKTPEERNLTSSPAMNGDLAKLACSEAPTDVSKTWVTSSA        275
NP_203754.2[H.sapiens]          -SQ--EGPVKS-PAECREEHSKTPEERSLPSDLAFNGDLAKAASSELPADISKPWIPSSP        276
XP_002821835.2[P.abelii]        -SQ--EGPVKF-PAECQEEHRKTPEERSLPSDLAFNGDLAKAASSELPADISKPWIPSSP        276
                                  *  **       .*   .     . .  :. :  ** .       * *:*.  :

XP_040557690.1[G.gallus]        SPRPP--ACPPPGAPFQPSASAPGSPDAPPELLAPGSPTMAPESPESPAQRPTEELL---        333
NP_001074729.1[M.musculus]      DPVSEHGGSTSAVRLANIS------------VPASESPRL-SSRPSSPCHSQLSETQSPA        322
NP_203754.2[H.sapiens]          APSSENGGPASPGLPAEAS------------GSGPGSPHLHPPDKSSPCHSQLLEAQTPE        324
XP_002821835.2[P.abelii]        APSSENGGPASPGLPAEAS------------GSGPGSPHLHSPDKSSPCHSQLLEAQSPE        324
                                 *     .        : *              .  ** :     .**.:    *

XP_040557690.1[G.gallus]        ---ATSIQ---APGAPSSVAEPWLGASHSPGSPHTPSEGSPTPASPSTPGTSQLSPKATC        387
NP_001074729.1[M.musculus]      ASEASSICLPVTPASPSAV-LP------AEPPGHSPSSELPAEAAPETLSPNSSPVE---        372
NP_203754.2[H.sapiens]          ASQA-SPCPVTPSAPSAA-LP------DEGSRHTPSPGLPAEGAPEAPRPSSPPPE---        373
XP_002821835.2[P.abelii]        ASQA-SPCSPVTPTAPSAA-LP------DEGSRHTPSPGLPAEGAPEAPRPSSPPPE---        373
                                   * *     :* :**:.  *           *:**   *: .:*.:   ..   :

XP_040557690.1[G.gallus]        PPGSPESATEPSPPPSPPPELPARASCPPGSPGGPDDSLVSSQTPEGPDFS----LPPSN        443
NP_001074729.1[M.musculus]      ------TVSGHHSPEQPPVLLPQLLT-----EGAELP-DITRTFPCGEEAAARGHTESRP        420
NP_203754.2[H.sapiens]          ------VL-EPHSLDQPPATSPRPLI-----EVGELL-DLTRTFPSGGEEEAKGDAHLRP        420
XP_002821835.2[P.abelii]        ------VL-EPHSLDQPPATSPQPVI-----EVGELL-DLTRTFPSGGEEEAKGDAHLRP        420
                                              .**   *           .     ::   * * :

XP_040557690.1[G.gallus]        RDSGLRRSSEGVLRPPPSEGQGMGLLGGSLGALPQPGDL----PAEHLLSGESSWSLSQS        499
NP_001074729.1[M.musculus]      SSLAQRRFSEGVLQPPSQD---QEKLGGSLATLPQGQGSQSALDRPFGSGTESNWSLSQS        477
NP_203754.2[H.sapiens]          TSLVQRRFSEGVLQSPSQD---QEKLGGSLAALPQGQGSQLALDRPFG--AESNWSLSQS        475
XP_002821835.2[P.abelii]        TSLVQRRFSEGVLQSPSQD---QEKLGGSLAALPQGQGSQLALDRPFG--AESNWSLSES        475
                                 .   ** *****: * .:      *****.:***  .        .     **.****:*

XP_040557690.1[G.gallus]        FEWTFPTR----GVRGLPSPPRSPIQEAADSGLSEEDESD-----REGAAT---------        541
NP_001074729.1[M.musculus]      FEWTFPTRPSGLGVWRLDSPPPSPITEASEAAEAAEADSWAVSGRGEGVSQVGPGTPPAP        537
NP_203754.2[H.sapiens]          FEWTFPTRPSGLGVWRLDSPPPSPITEASEAAEAAEAGNLAVSSREEGVSQQGQGAGSAP        535
XP_002821835.2[P.abelii]        FEWTFPTRPSGLGVWRLDSPPPSPITEASEAAEAAEAGNLAVSSGEEGVSQQGQGAGSAP        535
                                ********    **  * *** *** **:::. : *  .       **.:

XP_040557690.1[G.gallus]        -----GSHGDSGDTDGPGLAVTSSWGAEG---------------APCPGGPVAQQEAESS        581
NP_001074729.1[M.musculus]      ESPRKPISGVQG--NDPGISLPQRDDGESQPRSPALLP---STVEGPPGAPLLQAKENYE        592
NP_203754.2[H.sapiens]          SGS--GSSWVQG--DDPSMSLTQKGDGESQPQFPAVPLEPLPTTEGTPGLPLQQAEERYE        591
XP_002821835.2[P.abelii]        SGS--GSSWVQG--DDPSMSLTQKGDGESQPQFPAVPLEPLPTTEGTPGLPLQQAEERYE        591
                                          .*  :.*.::: .  ..*.                 ** *: * : . .

XP_040557690.1[G.gallus]        EEEEEEGE-----AELDSTELHIVEPSLDSAEPEPPVKAAPPDPAPTEADASCEPTGDFP        636
NP_001074729.1[M.musculus]      DQEPLVGHESPITLAAREAALPVLEPALGQQQPTPS-------------DQPCILFVDVP        639
NP_203754.2[H.sapiens]          SQEPLAGQESPLPLATREAALPILEPVLGQEQPAAP-------------DQPCVLFADAP        638
XP_002821835.2[P.abelii]        SQEPLAGQESPLPLATREAALPILEPVLGQEQPAPP-------------DQPCVLFADVP        638
                                .:*   *.         .: * ::** *.. :*                *  *    * *

XP_040557690.1[G.gallus]        ASLQGLGG-------------------PGQDEGSSKGPDAHTDPRWLTELLASPRARASG        677
NP_001074729.1[M.musculus]      DPEQALSTEEDVVTLGWAETTLPMTEAQEPCSVSPEPTGPESSSRWLDDLLASPPPNSGS        699
NP_203754.2[H.sapiens]          EPGQALPVEEEAVTLARAETTQARTEAQDLCRASPEPPGPESSSRWLDDLLASPPPSGGG        698
XP_002821835.2[P.abelii]        EPGQALPVEEEAMTLDRAETTQPRTEAQDLCRASPEPPGPESSSRWLDDLLASPPPSGGS        698
                                   *.*                               * :  . .:. *** :*****   ...

XP_040557690.1[G.gallus]        RDP----------LNTEEQEGLLGWSRKDLCSEFGIGRSHQAGAFDWSHKAVARQGDWPG        727
NP_001074729.1[M.musculus]      ARRAAGAELKDRQSPSTCSEGLLGWAQKDLQSEFGVATDSHHSSFGSS--------SWSQ        751
NP_203754.2[H.sapiens]          ARRGAGAELKDTQSPSTCSEGLLGWSQKDLQSEFGITGDPQPSSFSPS--------SWCQ        750
XP_002821835.2[P.abelii]        ARRGAGSELKDAQSPSTCSEGLLGWSQKDLQSEFGITGDPQPSSFSPS--------SWCQ        750
                                              :  .******::*** ****:  . : .:*. *        .*

XP_040557690.1[G.gallus]        EMEQDQKFRTNSSRNNSCGISDMNQQDRAFGAAERDWSSNYKGTELMGDTRLGSSDWSKS        787
NP_001074729.1[M.musculus]      DTSQNY--------------------------------SLGGRSPVGDTGLGKRDWSSK        778
NP_203754.2[H.sapiens]          GASQDY---------------------------------GLGGASPRGDPGLGERDWTSK        777
XP_002821835.2[P.abelii]        GASQDY---------------------------------GLGGASPRGDPGLGERDWTSK        777
                                  .*:                                  .  *  .  **  **. **:..

XP_040557690.1[G.gallus]        HGAGESCLQDQDFSKPTWGTGYGLDSAGSREEVGSGKAEWSSPYSVGHGQQQDKELSSRQ        847
```

```
NP_001074729.1[M.musculus]      CGQGSGEGS-----TREWASRHSLGQ----EVIGIGGSQDESEV-------P---VRERA    819
NP_203754.2[H.sapiens]          YGQGAGEGS-----TREWASRCGIGQ----EEMEASSSQDQSKVS-APGVLT---AQDRV    824
XP_002821835.2[P.abelii]        YGQGAGEGS-----TRDWASRCGIGQ----EEMEASSSQDQSEVS-APGALT---AQDRV    824
                                 * * .  .     .  *.:  .:..    * :  . :: .*               .*

XP_040557690.1[G.gallus]        PGWAGKYGSGDLETHSGDAAPVWSSEYGTGDADIKDREVT-PDWASKYSSRDAETKDKDF    906
NP_001074729.1[M.musculus]      VGRPAQLGA-----------------QG-LEADAQQWEFGKRESQDPHSIHDKELQDQEF    861
NP_203754.2[H.sapiens]          VGKPAQLGT-----------------QRSQEADVQDWEFRKRDSQGTYSSRDAELQDQEF    867
XP_002821835.2[P.abelii]        VGKPAQLGT-----------------QRSQEADVQDWEFRKRDSQGTYSSRDAELQDQEF    867
                                 *  .: *:                     :** :: *.   :  . :* :* * :*::*

XP_040557690.1[G.gallus]        TLGWAGRSSTGDAGTLDKEFCSSRSAWDSKYSTRDMESQDREFSPSRPAWSGEYSTQDME    966
NP_001074729.1[M.musculus]      GKR----DSL------------------GSFSTRDASLQDWEFGKR--ASVSTN--QDTD    895
NP_203754.2[H.sapiens]          GKR----DSL------------------GTYSSRDVSLGDWEFGKR--DSLGAYASQDAN    903
XP_002821835.2[P.abelii]        GKR----DSL------------------GTYSSRDVSLGDWEFGKR--DSLGTYASQDVN    903
                                       .*                   ..:*:** .  * **.       .    ** :

XP_040557690.1[G.gallus]        SQDREFSPSRLAEASEYSSRDMEIQDREFSPSRPACAEEHSTSDMESQDREFSTSRPTWH    1026
NP_001074729.1[M.musculus]      ENDQELGMK--NLSRGYSSQDAEEQDREFEKRDSV-LDIHGSRATAQQNQEF--GKSAWF    950
NP_203754.2[H.sapiens]          EQGQDLGKR--DHHGRYSSQDADEQDWEFQKRDVS-LGTYGSRAAEPQEQEF--GKSAWI    958
XP_002821835.2[P.abelii]        EQGQDLGKR--DHRGRYSSQDADEQDWKFQKRDVS-LGTYGSRGAEPQEQEF--GKSAWI    958
                                .:.:::.         ***:* : ** :*.         :.:     *::**  .: :*

XP_040557690.1[G.gallus]        REYSSR-----DMESQDREFSPSRPACTNEYSTSDAETQDREFNPSRPTWHSEYSSRDME    1081
NP_001074729.1[M.musculus]      QDYSSGGGGSRVLGSQERGFGIRS--LSSGFSPEEAQQQDEEFEKKTPVGEDRFCEASRD    1008
NP_203754.2[H.sapiens]          RDYSSGG-SSRTLDAQDRSFGTRP--LSSGFSPEEAQQQDEEFEKKIPSVEDSLGEGSRD    1015
XP_002821835.2[P.abelii]        RDYSSGG-SSRTLDAQDRSFGTRP--LSSGFSPEEAQQQDEEFEKKIPSVEDSLGEGSRD    1015
                                ::***       : :*:* *.      :. :* .:*: **.**: . *  ..   . . :

XP_040557690.1[G.gallus]        -------TQDREFSPSRPACADEYITKNMESQ--DREFSPN--R---PAEASEYSARDVE    1127
NP_001074729.1[M.musculus]      VGHLEEGASGGLLSPSTPHSRDGAARPKDEGSWQDGDSSQEITRLQGRMQAESQSPTNVD    1068
NP_203754.2[H.sapiens]          AGRPGERGSGGLFSPSTAHVPDGALGQRDQSSWQNSDASQEVGGHQERQQAGAQGPGSAD    1075
XP_002821835.2[P.abelii]        AGRPGERESGGLFSPSTAHVPDGALGQRDQSSWQNSDASQEVGGHQERQQAGAHGPGSAD    1075
                                        ..  :***     *     . :..  : : * :        :*   .  ..:

XP_040557690.1[G.gallus]        TQDRELKSNKPAWDDEYITKNMESRDREFGPSRSVWASDYDSMNTEKGEFSSSRLSWAGE    1187
NP_001074729.1[M.musculus]      LED--KEREQRGWAGEFSLGVAAQSEAAFSPG----------------------------    1098
NP_203754.2[H.sapiens]          LED--GEMGKRGWVGEFSLSVGPQREAAFSPG----------------------------    1105
XP_002821835.2[P.abelii]        LED--GEMGKRGWVGEFSLSVGPRREAAFSPG----------------------------    1105
                                 :*   :  : .* .*:        :  *.*.

XP_040557690.1[G.gallus]        CSIGQTQLANAFGVRKEDLPGLCAPSRPGQESSWGSTDQQESGTRDWAEELHLGGAEHQN    1247
NP_001074729.1[M.musculus]      -------------------------------------------RQDWSRDVCVEASESSY    1115
NP_203754.2[H.sapiens]          -------------------------------------------QQDWSRDFCIEASERSY    1122
XP_002821835.2[P.abelii]        -------------------------------------------QQDWSRDFCVEASERSY    1122
                                                                            :**:.:. : .:* .

XP_040557690.1[G.gallus]        QFGIIGTERVSDPSSTA-------------------------------------------    1264
NP_001074729.1[M.musculus]      QFGIIGNDRVSGAGLSPSRKSGGGHFVPPGETKAGAVDWTDQLGLRNLEVSSCVSSEGPS    1175
NP_203754.2[H.sapiens]          QFGIIGNDRVSGAGFSPSSKMEGGHFVPPGKTTAGSVDWTDQLGLRNLEVSSCVGSGGSS    1182
XP_002821835.2[P.abelii]        QFGIIGNDRVSGAGFSPSSKMGGGHFVPPGKTTAGSVDWTDQLGLRNLEVSSCVGSGGSS    1182
                                ******.:***. . :

XP_040557690.1[G.gallus]        ---ASADGLMSWANKMRS--------------EVPQEPRQPLGDWHSDLSFSTSGITGKA    1307
NP_001074729.1[M.musculus]      EARENVVGQMGWSDSLGLNNGDLARRLGTGESEEPRSLGVGEKDWTSSVEARNRDLPGQA    1235
NP_203754.2[H.sapiens]          EARESAVGQMGWSGGLSLRDMNLTGCLESGGSEEPGGIGVGEKDWTSDVNVKSKDLAEVG    1242
XP_002821835.2[P.abelii]        EARENAVGQMGWSGGLSLRDMNLAGCLESGGSEEPGGIGVGEKDWTSDVNVKSKDLAEVG    1242
                                    .. * *.*:. :                * *        ** *.:.  . .:   .

XP_040557690.1[G.gallus]        DEGEPSQMAWDEGLAAVGSSAQPQQAKVEEPGWSQD------------LEEAGWSEEL--    1353
NP_001074729.1[M.musculus]      EVG---------------RHSQARESGVGEPDWSGAEAGEFLKSRERGVGQADWTPDLGL    1280
NP_203754.2[H.sapiens]          EGG---------------GHSQARESGVGQTDWSGVEAGEFLKSRERGVGQADWTPDLGL    1287
XP_002821835.2[P.abelii]        EGG---------------GHSQARESGVGQTDWSGVEAGEFLKSRERGVGQADWTPDLGL    1287
                                : *                 :* ::: * : .**              : :*.*: :*

XP_040557690.1[G.gallus]        --------------REAEARRQEWASAFGARCAARSQDFSAG-EQSLGGDGGS-----AD    1393
NP_001074729.1[M.musculus]      RNMAPGAGCSPGEPRELGVGQVDWGDDLGLRNLEVSCDLESGGS---RGCGVGQMDWAQD    1337
NP_203754.2[H.sapiens]          RNMAPGAVCSPGESKELGVGQMDWGNNLGLRDLEVTCDPDSGGSQGLRGCGVGQMDWTQD    1347
XP_002821835.2[P.abelii]        RNMAPGAVCSPGEHKELGVGQMDWGNNLGLRDLEVTCDPESGDSQGLRGCGVGQMDWTQD    1347
                                              :*  . : :*.. :* *    : * .:* .    * * .      *

XP_040557690.1[G.gallus]        GNPENIHLSE--------------------------SSPPL-----------GDDAVAEP    1416
NP_001074729.1[M.musculus]      LGLRNLRLCGAPSEVRECGVGRVGPDLELDPKSSGSLSPGLETEDPLEARELGVGEISGP    1397
NP_203754.2[H.sapiens]          LAPQNVELFGAPSEAREHGVGGVSQCPEPGLRHNGSLSPGLEARDPLEARELGVGETSGP    1407
XP_002821835.2[P.abelii]        LAPRNVELFGAPSEAREHGVGGVSQCPEPGLRHNGSLSPGLEARDPLEARELGVGETSGP    1407
                                   .*:.*                             ** *           * .  : *

XP_040557690.1[G.gallus]        A---------------------AAEPIWGESA---TPRTDPMEQQDLPE-LAAAP----    1446
NP_001074729.1[M.musculus]      ETQGEDSSSPSFETPSEDTGMDTGEAPSLGASPSSCLTRSPPSGSQSLLEGIMTASSSKG    1457
NP_203754.2[H.sapiens]          ETQGEDYSSSSLEPHPADPGMETGEALSFGASPGRCPARPPPSGSQGLLEEMLAASSSKA    1467
XP_002821835.2[P.abelii]        ETQGEDDSSFSLEPHPADPGMETGEALSFGASPGRRPARPPPSGSQGLLEEMLAASSSKA    1467
                                                       .     * *      *  *  .*.* * : :*

XP_040557690.1[G.gallus]        --------------LPAEAA--G----E------------TPETESEEAPLDSPDGK---    1471
NP_001074729.1[M.musculus]      APQRESAASGSRVLLEEEGLAAGAGQGEPQEPSRAPLPSSRPQPDGEASQVEEVDGTWSL    1517
NP_203754.2[H.sapiens]          VARRESAASGLGGLLEEEGAGAGAAQEEVLEPGRDSPPSWRPQPDGEASQTEDVDGTWGS    1527
XP_002821835.2[P.abelii]        VAQRESAASGLGGLLEEEGAGAGAAQEEVLEPGRDSPPSWRPQPDGEASQTEDVDGTWGS    1527
                                              *  *.   *    *             *: :.* :  :. **.

XP_040557690.1[G.gallus]        -TPVSWEEKRLSV-SASQPEGL-LDLSGQDFTFLEDTEVLDSTMYRSKANLGRKRRHRAP    1528
NP_001074729.1[M.musculus]      TGAARQNEQASAPPPRRPPRGLLPSCPSEDFSFIEDTEILDSAMYRSRANLGRKRGHRAP    1577
NP_203754.2[H.sapiens]          SAA-RWSDQGPAQTSRRPSQGPPARSPSQDFSFIEDTEILDSAMYRSRANLGRKRGHRAP    1586
XP_002821835.2[P.abelii]        SAA-RWSDQGPAQTSRRPSQGPPARSPSQDFSFIEDTEILDSAMYRSRANLGRKRGHRAP    1586
                                      .::  :       .*      .:**:*:****:***:****:******* ****
```

```
XP_040557690.1[G.gallus]        VLRPGAT------SEGDSWIFQDSTEPRPARAAASSDEEAAEEPKSRRVRASPSGKGVKV        1582
NP_001074729.1[M.musculus]      AIRPGGTLGLSETADSDTRLFQDSTEPRASRV-PSSDEEVVEEPQSRRTRMSLGTKGLKV        1636
NP_203754.2[H.sapiens]          VIRPGGTLGLSEAADSDAHLFQDSTEPRASRV-PSSDEEVVEEPQSRRTRMSLGTKGLKV        1645
XP_002821835.2[P.abelii]        AIRPGGTLGLSEAADSDARLFQDSTEPRASRV-PSSDEEVVEEPQSRRTRMSLGTKGLKV        1645
                                .:***.*      ::.*: :******** :*.  *****..***:***.* * . **:**

XP_040557690.1[G.gallus]        PLFPGLTTSALKAKLRGRNRSVEEGASPVDSKATPTKDPHVQRSKSCKIPGLSGKPLALP        1642
NP_001074729.1[M.musculus]      NLFPGLSPSALKAKLRSRNRSAEEGEVTE--SKSSQKESSVQRSKSCKVPGL-GKPLTLP        1693
NP_203754.2[H.sapiens]          NLFPGLSPSALKAKLRPRNRSAEEGELAE--SKSSQKESAVQRSKSCKVPGL-GKPLTLP        1702
XP_002821835.2[P.abelii]        NLFPGLSPSALKAKLRPRNRSAEEGEPAE--SKSSQKESAVQRSKSCKVSGL-GKPLTLP        1702
                                 *****: ******** ****.***      . :  *:  ********: ** ****:**

XP_040557690.1[G.gallus]        PKPEKSSGSDASPPHWLQALKLKKKKS 1669
NP_001074729.1[M.musculus]      PKPEKSSGSEGSSPNWLQALKLKKKKI 1720
NP_203754.2[H.sapiens]          PKPEKSSGSEGSSPNWLQALKLKKKKV 1729
XP_002821835.2[P.abelii]        PKPEKSSGSEGSSPNWLQALKLKKKKV 1729
                                *********:.* *:***********
```
