# Supplementary material for: An Evolutionary Perspective on the Origin, Conservation and Binding Partner Acquisition of Tankyrases
Source: Biomolecules. 2022 Nov 15;12(11):1688. doi: 10.3390/biom12111688 (PMC9688111; doi:10.3390/biom12111688)
Supplement: Supplementary file 1 [file biomolecules-12-01688-s001.zip › biomolecules-1913719-supplementary-11.17 -done/Data_7/TRF1.pdf]

TRF1

Homo sapiens:

>sp|P54274.3|TERF1_HUMAN RecName: Full=Telomeric repeat-binding factor 1; AltName: Full=NIMA-interacting protein 2; AltName: Full=TTAGGG repeat-binding factor 1; AltName: Full=Telomeric protein Pin2/TRF1
MAEDVSSAAPSPRGCADGRDADPTEEQMAETERNDEEQFECQELLECQVQVGAPEEEEEEEEDAGLVAEA
EAVAAGWMLDFLCLSLCRAFRDGRSEDFRRTRNSAEAIIHGLSSLTACQLRTIYICQFLTRIAAGKTLDA
QFENDERITPLESALMIWGSIEKEHDKLHEEIQNLIKIQAIAVCMENGNFKEAEEVFERIFGDPNSHMPF
KSKLLMIISQKDTFHSFFQHFSYNHMMEKIKSYVNYVLSEKSSTFLMKAAAKVVESKRTRTITSQDKPSG
NDVEMETEANLDTRKSVSDKQSAVTESSEGTVSLLRSHKNLFLSKLQHGTQQQDLNKKERRVGTPQSTKK
KKESRRATESRIPVSKSQPVTPEKHRARKRQAWLWEEDKNLRSGVRKYGEGNWSKILLHYKFNNRTSVML
KDRWRTMKKLKLISSDSED

Pongo abelii:

>PNJ54419.1 TERF1 isoform 3 [Pongo abelii]
MAEDVASAAPSPRGCADGRDADPTEEQMAETERNDEEQFECQELLECQVQVGAPEEEEEDAGLVAEAEAV
AAGWMLDFLCLSLCRAFRDGRSEDFRRTRNSAEAIIHGLSSLTAYQLRTIYICQFLTRIAAGKTLDAQFE
NDERITPLESALMIWGSIEKEHDKLHEEIQNLIKIQAIAVCMENGNFKEAEEVFERIFGDPNSYMPFKSK
LLMIISQKDTFHSFFQHFSYNHMMEKIKSYVNYVLSEKSSTFLMKAAAKVVESKRTGTITSQDKPNGNDV
EMETEANLDTRKSVSDKQSAVTESSEGTVSLLRSHKNLFLPKLQHGTEQQDLNKKERRVGTLQSTKKKKE
SRRATESRIPVSKSQLVTPEKHRARKRQAWLWEEDKNLRSGVRKYGEGNWSKILLHYKFNNRTSVMLKDR
WRTMKKLKLICSDSED

Mus musculus:

>NP_033378.1 telomeric repeat-binding factor 1 isoform 1 [Mus musculus]
MAETVSSAARDAPSREGWTDSDSPEQEEVGDDAELLQCQLQLGTPREMENAELVAEVEAVAAGWMLDFLC
LSLCRAFRDGRSEDFRRTRDSAEAIIHGLHRLTAYQLKTVYICQFLTRVASGKALDAQFEVDERITPLES
ALMIWNSIEKEHDKLHDEIKNLIKIQAVAVCMEIGSFKEAEEVFERIFGDPEFYTPLERKLLKIISQKDV
FHSLFQHFSYSCMMEKIQSYVGDVLSEKSSTFLMKAATKVVENEKARTQASKDRPDATNTGMDTEVGLNK
EKSVNGQQSTETEPLVDTVSSIRSHKNALSQLKHRRAPSDFSRNEARTGTLQCETTMERNRRTSGRNRLC
VSENQPDTDDKSGRRKRQTWLWEEDRILKCGVKKYGEGNWAKILSHYKFNNRTSVMLKDRWRTMKRLKLI
S

Gallus gallus:

>NP_989711.2 telomeric repeat-binding factor 1 [Gallus gallus]
MSEAGREREGGLVPFLPSALAEAVAADWVLEFSCCCLCRYFVEECEAEFRRWRDVAQAVSNGFSKVTTHQ
KKMVYLCQLLIRIAEGKRLECHFENDTTISPLESALSFWTLLEREESKLNTLHEEIRRLIQIQVVAVYME
KGYYKEAAEVLERLFTDSESHKPLRMKLAAIVKSKDPYVPLLQSFSYSLLLSKVKSYVKLFLKENRTNFL
LQAATKQVESEGGEVRVLQNKTLNVKEERENNLEAKQRPAEELRSTTDWLTGDISSRVRPPSKKGCRTSS
VQRLKDLKNVEERGDDLPCSRRRQPWTYEEDKKLKSGVREFGVGNWTKILIHGDFNNRTSVMLKDRWRTL
CKIK

Xenopus laevis:

>ABA29525.1 telomeric repeat binding factor 1 [Xenopus laevis]
MEEETDGPPFDDTAAVATNWMCDFMFASMCFYFREDRTEDFQRSTHMLEWLLEGSQKIDAHRKTIPIAQF
LMRVAEGKNLDSQFDTDESLTPLETALMAFNQIEEEEDLKHLHEEIELLLKVQAVVTCMEKGRFKLSAEI
LDRLFKESGSNKYLRMKLTMLIEKKDPYHEFLQNFTYAQMMKKIKSYIALKMKERPSVFLLKAAAKVVEA
TAKEELDIQSQESEDCEQQTNESLENKDDNSSSEYEERDVLSLSNINHVENKDVSSSDYEEATEQLKVCN
RDINQNELTNTTNIQETTEKSTKRHQRRLFSIAQRTPWNPDKPCTSKRLLSSINIGKNSKENQENVKDSR
TEKPLNSKKRQHWTWEEDELLKKGVRKFGVGNWSKILLHYEFRNRTGVMLKDRWRTMKRLKIVDSDCDL

Salmo salar:

>XP_014015369.1 PREDICTED: telomeric repeat-binding factor 1 [Salmo salar]
MEADPNNRTLTITSTTDENVCFSDVATVAKRWMIDFSFVSLCQFFKEGKFEEFNQTISTLETIIDGSPHL
NTEQKQKRQICGFLARIMHGKHLDVSFDRDERLSPLMSAVRVWASQKETVANLSLFQHVTNLLYVQSVAV
CLEKGDCGMASSALKWLEEECEIPQNLGIKLSLIVSKRDTYHPFLLNFSWNRLLENIQTFLDRFLEEHPS
DFLLQAATKVVKAGQETGEDSETQEASYTTSRSSEHSKVNQETSVLMRPKKKLLSTRNIHPWKPESGKKP
ALTFMRKRVSTRLTYRRALPETTLNTTSLSNTTLNTNATRKTHGRWPWDLDMALKAGVKRHGEGKWSRIL
QEHDFQGRTGVQLKDRWRVLKKAHKVNSS

Petromyzon marinus:

>XP_032808952.1 uncharacterized protein LOC116941715 isoform X1 [Petromyzon marinus]
MAEVRDGLVSEYLSAPLLRERGDRLLFDHLYRLAAAYFELGDYERLSGVYRAASGALQFLREEQPRQVQW
KLIQLMEMVMRGQFFDPVREGNQMLLEGAVEMVDMLGRECQLEPEVTQEVTRILRLKLLTEARVQVQRNE
QLQTDLVDPWYSLDLKPHILRFIDSVPQSSQASSYVQLLKESLPQETPPANKTNGGKFLHKDHIKEAFLS
IADCREDAFNTFEVLLLTDVPPLSLKLALPAMGAAESSPGSERREDSSVPEGAAANNSVAPARVISVPQK
QLSPAKRLMGPETPRHVPEDSREEKEKVVAPLQATPPSPPRPPPLSPLLLSDSEELLPMRIVQSKRSDQS
TAEAACDDAHQMSTTDFIPSTYEEIPPTAPAKDVAGPGSTPPILAIIGRKRTRSDSPRVVEEAAATPRLV
EDGEVAGPSKPRAATISPAKATCGKWDKGHGHPTRNHVERNGRFPGDGFSSAEIRRLSPLMIKDEWSDEE
DLFGPSKVAGRQSKFGSSARKKKMWTETETQCLKEGVARFGVGNWSKIKRHYSLSRTAVQLKDRWRNISK

Branchiostoma belcheri:

>XP_019634930.1 PREDICTED: telomeric repeat-binding factor 2-like [Branchiostoma belcheri]
MIIFICRYEEDGDITALESALPIFDSLVSDFAVPEDQVSEARGLVMTQSVLVCCRGGDFQAAEQVFSRVW
VPGAATTAKRKKLRSKVQEVMRSGNPDHSLVQVRCSYDKFLTWMIQFLHLIVDGFSLPFLLKAAQRVLRA
RIDAGDGTEAPEESLSQSESDSDMYETPSENEAPPKEQEEEFVFNSPALLRECFLVMAKEERSGSEWTDV
NGYDYRNLRGDLAKSREAKTSGAASEKDLEQNREEKTSGSGVRKSPRKHQQAAKNSEPTERRSPRKKTSS
ETTGRHEFATHGAVQSRGEKRKQQTDEENFEKTENRKKTQTTSAPSTDRNNPGRMQKSGDTGKDIRYWMT
SDQPSTSGVRKKVHKQAERRTLVESDSESSEEETRKENVTQGSKKRPRDRPEYGNGVKETWSSDEETFRA
PVKKKYYIYDSDSDSSENFPSALPCSAASLVVPRRLVQKREPLDITSKGQVRSRFRWAEYEVENLIKGVQ
EYGVGHWSEILDNYRFKNRTNVNLKDKWRQLVKSGTVREFP

Drosophila melanogaster:

No homolog found.

Hydra vulgaris:

```
>XP_012558972.1 PREDICTED: putative uncharacterized protein DDB_G0282499 [Hydra vulgaris]
MSEQQDTLYNTDFLIMEYFAHKIIKMFLEEYKGCINNNMVHLMNAWKVMVHSSDGIFSDEMRNLYILISL
IVTCCFSSISQVDQLNLMLEMINTQGQEFQSATEDVSNVCKFLHTKIFLKLLDNGCGKGAKQYLRDHDVL
CRDSLEQLVSKDSLPENLKPENLKNVCFSYVSEVYKKIPRSRLVLLFDNFMKEKNNTRFKGKSFLDKVDE
SDIDISKEVENALPQSQVMTLIPKETDTTYSSSSEPMPNSYQSSTTYVEKESGTFKDSPCIPTKHKLSND
SFKETVDLKYSKNHLRKSFKYKGSYKIYNTRSDKKVTSNLNSHMYISDFKKSFTRDNSKKSDDDMIFDDV
RSDISDSQTENLGIKCYVDINKVISPKYIEKKNNDTQNIYCEQNRSNNDNNETEKNNTSNHSSKLQIGCQ
NLTELSNNENFCSVVSNNSIFGGKKKVKVENIHTNNKACHILTNNQINYNYKNLKTKNSDQQTSADSDDI
YIKTSADLACVKNIDSLREDVTSKDTSIVTPASLKKLLGDEQQNDSKVVFECVCQSSKEKTVSSSVANKK
SIVNSDESGTSCKWSDDEFEVKYNPTVRVPFSKDEEKYIKEGIKKFGIGNWCKIKRSYPFHPNRTNVSIK
DKYRTMKRQGIL
```

Nematostella vectensis:

No homolog found.

Amphimedon queenslandica:

No homolog found.

Alignment

```
XP_012558972.1[H.vulgaris]      ------------------------------------------------------------    0
XP_019634930.1[B.belcheri]      ------------------------------------------------------------    0
XP_032808952.1[P.marinus]       ------------------------------------------------------------    0
XP_014015369.1[S.salar]         ---------------------MEADPNNRTLTI---------------------------    12
ABA29525.1[X.laevis]            ------------------------------------------------------------    0
NP_989711.2[G.gallus]           ---------------------------------------------------------MSE    3
NP_033378.1[M.musculus]         MAETVSSAARDAPSREGWTDSD------------SPEQEEVGDDAELLQCQLQLGTPREM    48
P54274.3[H.Sapiens]             MAEDVSSAA---PSPRGCADGRDADPTEEQMAETERNDEEQFECQELLECQVQVGAPEEE    57
PNJ54419.1[P.abelii]            MAEDVASAA---PSPRGCADGRDADPTEEQMAETERNDEEQFECQELLECQVQVGAPEEE    57


XP_012558972.1[H.vulgaris]      MSEQQDTLYN-----------TDFLIMEYFAHKIIKMFL----EEYKGCINNNMVHLMNA    45
XP_019634930.1[B.belcheri]      ------------------------------------------------------------    0
XP_032808952.1[P.marinus]       MAEVRDGLVSEYLSAPLLRERGDRLLFDHLYRLAAAYFELGDYERLSGV-----------    49
XP_014015369.1[S.salar]         -----TSTTDENVCFSDVATVAKRWMIDFSFVSLCQFFKEGKFEEFNQTI--------ST    59
ABA29525.1[X.laevis]            MEEETDG-----PPFDDTAAVATNWMCDFMFASMCFYFREDRTEDFQRST--------HM    47
NP_989711.2[G.gallus]           AGREREGGLVPFLPSALAEAVAADWVLEFSCCCLCRYFVEECEAEFRRWR--------DV    55
NP_033378.1[M.musculus]         ----ENAEL-----VAEVEAVAAGWMLDFLCLSLCRAFRDGRSEDFRRTR--------DS    91
P54274.3[H.Sapiens]             EEEEEDAGL-----VAEAEAVAAGWMLDFLCLSLCRAFRDGRSEDFRRTR--------NS    104
PNJ54419.1[P.abelii]            ---EEDAGL-----VAEAEAVAAGWMLDFLCLSLCRAFRDGRSEDFRRTR--------NS    101


XP_012558972.1[H.vulgaris]      WKVMVHSSDGIFSDEMRNL---------YILISLIVTCCFSSISQVDQLNLMLEMINTQG    96
XP_019634930.1[B.belcheri]      --------------------------------MIIFICRYEEDGDITALESALPIFDSLV    28
XP_032808952.1[P.marinus]       YRAASGALQFLREEQPRQVQWKLIQLMEMVMRGQFFDPVREGNQM--LLEGAVEMVDMLG    107
XP_014015369.1[S.salar]         LETIIDGSPHLNTEQKQKR--QICGFLARIMHGKHLDVSFDRDERLSPLMSAVRVWASQK    117
ABA29525.1[X.laevis]            LEWLLEGSQKIDAH-RKTI--PIAQFLMRVAEGKNLDSQFDTDESLTPLETALMAFNQIE    104
NP_989711.2[G.gallus]           AQAVSNGFSKVTTHQKKMV--YLCQLLIRIAEGKRLECHFENDTTISPLESALSFWTLLE    113
NP_033378.1[M.musculus]         AEAIIHGLHRLTAYQLKTV--YICQFLTRVASGKALDAQFEVDERITPLESALMIWNSIE    149
P54274.3[H.Sapiens]             AEAIIHGLSSLTACQLRTI--YICQFLTRIAAGKTLDAQFENDERITPLESALMIWGSIE    162
PNJ54419.1[P.abelii]            AEAIIHGLSSLTAYQLRTI--YICQFLTRIAAGKTLDAQFENDERITPLESALMIWGSIE    159
                                                                   .    .       *   :

XP_012558972.1[H.vulgaris]      QEFQSATEDVSNVCKFLHTKIFLKLLDNGCGKGAK----------------QYLRDHDVL    140
XP_019634930.1[B.belcheri]      SDFAVPEDQVSEARGLVMTQSVLVC----CRGG----------DF--QAAEQVFSRVWVP    72
XP_032808952.1[P.marinus]       RECQLEPEVTQEVTRILRLKLLTEA-RVQVQRNEQLQTDLVDPWYSLDLKPHILRFIDSV    166
XP_014015369.1[S.salar]         ETVANL-SLFQHVTNLLYVQSVAVC----LEKG----------DC--GMASSALKWLEEE    160
ABA29525.1[X.laevis]            EEED-LKHLHEEIELLLKVQAVVTC----MEKG----------RF--KLSAEILDRLFKE    147
NP_989711.2[G.gallus]           REESKLNTLHEEIRRLIQIQVVAVY----MEKG----------YY--KEAAEVLERLFTD    157
NP_033378.1[M.musculus]         KEHD---KLHDEIKNLIKIQAVAVC----MEIG----------SF--KEAEEVFERIFGD    190
P54274.3[H.Sapiens]             KEHD---KLHEEIQNLIKIQAIAVC----MENG----------NF--KEAEEVFERIFGD    203
PNJ54419.1[P.abelii]            KEHD---KLHEEIQNLIKIQAIAVC----MENG----------NF--KEAEEVFERIFGD    200
                                          ..   ::  : .          .                    :

XP_012558972.1[H.vulgaris]      CRDS---LEQLVSKDSLPENLKPENLKNVCFSYVSEVYKKIPRSRLVL---LFDNFMKEK    194
XP_019634930.1[B.belcheri]      GAA-----------------TTAKRKKL-RSKVQEVMRSGNPDHSLVQVRCSYDKFLTWM    114
XP_032808952.1[P.marinus]       PQSSQASSYVQLLKESLPQETPPANKTN-GGKFLH----KDHIKEAFL-SIA-DCREDAF    219
XP_014015369.1[S.salar]         C---------------------EIPQNL-GIKLSLIVSKRDTYHPFLL-NFSWNRLLENI    197
ABA29525.1[X.laevis]            S---------------------GSNKYL-RMKLTMLIEKKDPYHEFLQ-NFTYAQMMKKI    184
NP_989711.2[G.gallus]           S---------------------ESHKPL-RMKLAAIVKSKDPYVPLLQ-SFSYSLLLSKV    194
NP_033378.1[M.musculus]         P---------------------EFYTPL-ERKLLKIISQKDVFHSLFQ-HFSYSCMMEKI    227
P54274.3[H.Sapiens]             P---------------------NSHMPF-KSKLLMIISQKDTFHSFFQ-HFSYNHMMEKI    240
PNJ54419.1[P.abelii]            P---------------------NSYMPF-KSKLLMIISQKDTFHSFFQ-HFSYNHMMEKI    237
                                                               .              .

XP_012558972.1[H.vulgaris]      N---NTRFKG--KSFLDKVD----ESDIDIS---KEVENALPQSQVMTLIPKETDTT-YS    241
XP_019634930.1[B.belcheri]      IQFLHLIVDGFSLPFLLKAAQRVLRARIDAGDGTEAPEESLSQ--------SESDSDMYE    166
XP_032808952.1[P.marinus]       NTFEVLLLTDVPPLSLKLALPAMGAAESS--PGSERREDSSVP--------EGAAANNSV    269
XP_014015369.1[S.salar]         QTFLDRFLEEHPSDFLLQAATK--VVKAG--Q--ETGEDSETQ--------E---ASYT-    239
ABA29525.1[X.laevis]            KSYIALKMKERPSVFLLKAAAK--VVEAT--A--KEELDIQSQ--------E---SED--    225
NP_989711.2[G.gallus]           KSYVKLFLKENRTNFLLQAATK--QVESE--G--GEVRVLQNK--------T---LNV--    235
NP_033378.1[M.musculus]         QSYVGDVLSEKSSTFLMKAATK--VVENE--K--ARTQASKDR--------P---DATN-    269
P54274.3[H.Sapiens]             KSYVNYVLSEKSSTFLMKAAAK--VVESK--R--TRTITSQDK--------P---SGND-    282
PNJ54419.1[P.abelii]            KSYVNYVLSEKSSTFLMKAAAK--VVESK--R--TGTITSQDK--------P---NGND-    279
                                       .       *  .

XP_012558972.1[H.vulgaris]      SSS--EPMPNSYQSSTTYVEKESGTFKDSPCIP--TKHKLSNDSFKETVDLKYSKNHLRK    297
XP_019634930.1[B.belcheri]      TPSENEAPPKEQ--EEEFVFNSPAL--LRECFLVMAKEERSGSEWTDVNGYDY--RNLRG    220
XP_032808952.1[P.marinus]       APARVISVPQKQ---------------LSPAKRLMGPE----------------------    292
XP_014015369.1[S.salar]         ----------------------------------TSRS----------------------    243
ABA29525.1[X.laevis]            ----------------------------------CEQQ----------------------    229
NP_989711.2[G.gallus]           -----------------------------------KEE----------------------    238
NP_033378.1[M.musculus]         ----------------------------------TGMD----------------------    273
P54274.3[H.Sapiens]             ----------------------------------VEME----------------------    286
PNJ54419.1[P.abelii]            ----------------------------------VEME----------------------    283


XP_012558972.1[H.vulgaris]      SFKYKGSYKIYNTRSDKKVTSNLNSHMYISDFKKSFTRDNSKKSDDDMIF---DDVRSDI    354
XP_019634930.1[B.belcheri]      DLAKSREAKTSGAASEKDLEQNREEKTSGSGVRKSP-----RKHQQAAKNSEPTERRSPR    275
XP_032808952.1[P.marinus]       ---------TPRH----VPEDSREEKEKVVAP---------------LQATPPSPPRPPP    324
XP_014015369.1[S.salar]         ------------------SEHS---------K----------------VN---QETSV--    255
ABA29525.1[X.laevis]            ------------------TNESLENKDDNSSS----------------EY---EERDVLS    252
NP_989711.2[G.gallus]           ------------------RENNLEAKQR-------------------------------P    249
NP_033378.1[M.musculus]         ------------------TEVGLNKEKSVNGQ----------------QSTE-TEPLVDT    298
P54274.3[H.Sapiens]             ------------------TEANLDTRKSVSDK----------------QSAV-TESSEGT    311
PNJ54419.1[P.abelii]            ------------------TEANLDTRKSVSDK----------------QSAV-TESSEGT    308
                                                     .

XP_012558972.1[H.vulgaris]      SDSQTENLGIKCYVDINKVISPKYIEKKNNDTQNIYCEQNRSNNDNNETEKNN-----TS    409
XP_019634930.1[B.belcheri]      KKTSSETTGRHEFATHGAVQSR----GEKR---------KQQTDEENFEKTENRKKTQTT    322
XP_032808952.1[P.marinus]       LSPL-LLSDSEELLPMRIVQSK----RSDQSTAEAACDDAHQMSTTDFIPSTY--EEIPP    377
XP_014015369.1[S.salar]         ------------------------------------------------------------    255
ABA29525.1[X.laevis]            LSNI------------------------------------NHVENKDVSSSDY--EEATE    274
NP_989711.2[G.gallus]           AEEL------------------------------------RST--TDWLTGDI--SS---    266
NP_033378.1[M.musculus]         VSSI------------------------------------RSH--KNAL-SQL--KH---    314
P54274.3[H.Sapiens]             VSLL------------------------------------RSH--KNLFLSKL--QH---    328
PNJ54419.1[P.abelii]            VSLL------------------------------------RSH--KNLFLPKL--QH---    325


XP_012558972.1[H.vulgaris]      NHSSKLQ--IG-CQNLTELSNNENFCSVVSNNSI---------------------FGGKK    445
XP_019634930.1[B.belcheri]      SAPSTDRNNPGRMQKSGDTGKDIRYWMTSDQPSTSGVRKKVHKQAERRTLVESDSESSEE    382
XP_032808952.1[P.marinus]       TAPAKDVAGPGSTP------PILAIIG--RKRTRSDSPRVVEEAAATPRLVEDGEVAGPS    429
XP_014015369.1[S.salar]         ----------------------------------------------------------LM    257
ABA29525.1[X.laevis]            QL--------KVCN------RDIN-----QNELTN-----------TTNIQET-TEKSTK    303
NP_989711.2[G.gallus]           ----------RVRP------P--S-----KKGCR--------------------------    277
NP_033378.1[M.musculus]         ----------RRAP------SDFS-----RNEART-----------GT------------    330
P54274.3[H.Sapiens]             ----------GTQQ------QDLN-----KKERRV-----------GT------------    344
PNJ54419.1[P.abelii]            ----------GTEQ------QDLN-----KKERRV-----------GT------------    341


XP_012558972.1[H.vulgaris]      KVKVENIHTNNKACHILTNNQINYNYKNLKTKNSDQQTSADSDDIYIKTSADLACVKNI-    504
```

```
XP_019634930.1[B.belcheri]      ETRKENVTQGS----------KKR------P----------R-------------DRPEY    403
XP_032808952.1[P.marinus]       KPRAATISPAKATCGKWDKGHGHP------TRNH-----VERNGRFPGDGFSSAEIRRLS    478
XP_014015369.1[S.salar]         RPKKKLLS--TRNIHPWKPESGKK------PALT-----FMRKRV--STRL--TYRRALP    300
ABA29525.1[X.laevis]            RHQRRLFS--IAQRTPWNPDKPCT------SKRL-----LSS------IN---IGKNSKE    341
NP_989711.2[G.gallus]           -------------------------------------------------T---SSVQRLK    285
NP_033378.1[M.musculus]         --------------------LQCE------TTME-----RNR------RT---SGRNRLC    350
P54274.3[H.Sapiens]             --------------------PQST------KKKK------ES------RR---ATESRIP    363
PNJ54419.1[P.abelii]            --------------------LQST------KKKK------ES------RR---ATESRIP    360


XP_012558972.1[H.vulgaris]      -DSLREDVTSKDTSIVTPASLKKLLGDEQQNDSKVVFEC--VCQSSKEKTVSSSVANKKS    561
XP_019634930.1[B.belcheri]      GNGVKETWSSDEETFRAPVKKKYYIYDS-DSDSSENFPSALPCSA-ASLVVPRRLVQKRE    461
XP_032808952.1[P.marinus]       PLMIKDEWSDEEDLFGP-----------------------------------SKVAGR--    501
XP_014015369.1[S.salar]         -----------ETTLNT-----------------------------------TSLSNT--    312
ABA29525.1[X.laevis]            ---------------NQ-----------------------------------ENVKDS--    349
NP_989711.2[G.gallus]           ---------------D------------------------------------LKNVEE--    292
NP_033378.1[M.musculus]         ----------------------------------------------------VSENQP--    356
P54274.3[H.Sapiens]             ----------------------------------------------------VSKSQP--    369
PNJ54419.1[P.abelii]            ----------------------------------------------------VSKSQL--    366


XP_012558972.1[H.vulgaris]      IVNSDESGTSCKWSDDEFEVKYNPTVRVPFSKDEEKYIKEGIKKFGIGNWCKIKRSYPFH    621
XP_019634930.1[B.belcheri]      PL--------------DITSKGQVRSRFRWAEYEVENLIKGVQEYGVGHWSEILDNYRFK    507
XP_032808952.1[P.marinus]       ----------------QSKFGSSARKKKMWTETETQCLKEGVARFGVGNWSKIKRHYSL-    544
XP_014015369.1[S.salar]         ----------------TLNTNATRKTHGRWPWDLDMALKAGVKRHGEGKWSRILQEHDFQ    356
ABA29525.1[X.laevis]            ----------------RTEKPLNSKKRQHWTWEEDELLKKGVRKFGVGNWSKILLHYEFR    393
NP_989711.2[G.gallus]           ----------------RGDDLPCSRRRQPWTYEEDKKLKSGVREFGVGNWTKILIHGDFN    336
NP_033378.1[M.musculus]         ----------------DTDDKSGRRKRQTWLWEEDRILKCGVKKYGEGNWAKILSHYKFN    400
P54274.3[H.Sapiens]             ----------------VTPEKHRARKRQAWLWEEDKNLRSGVRKYGEGNWSKILLHYKFN    413
PNJ54419.1[P.abelii]            ----------------VTPEKHRARKRQAWLWEEDKNLRSGVRKYGEGNWSKILLHYKFN    410
                                                          :  :       :  *: ..* *:* .*     :

XP_012558972.1[H.vulgaris]      PNRTNVSIKDKYRTMKRQGIL------ 642
XP_019634930.1[B.belcheri]      -NRTNVNLKDKWRQLVKSGTVREFP-- 531
XP_032808952.1[P.marinus]       -SRTAVQLKDRWRNISK---------- 560
XP_014015369.1[S.salar]         -GRTGVQLKDRWRVLKKAHKVNSS--- 379
ABA29525.1[X.laevis]            -NRTGVMLKDRWRTMKRLKIVDSDCDL 419
NP_989711.2[G.gallus]           -NRTSVMLKDRWRTLCKIK-------- 354
NP_033378.1[M.musculus]         -NRTSVMLKDRWRTMKRLKLIS----- 421
P54274.3[H.Sapiens]             -NRTSVMLKDRWRTMKKLKLISSDSED 439
PNJ54419.1[P.abelii]            -NRTSVMLKDRWRTMKKLKLICSDSED 436
                                 .** * :**::* : :
```
