# Supplementary material for: An Evolutionary Perspective on the Origin, Conservation and Binding Partner Acquisition of Tankyrases
Source: Biomolecules. 2022 Nov 15;12(11):1688. doi: 10.3390/biom12111688 (PMC9688111; doi:10.3390/biom12111688)
Supplement: Supplementary file 1 [file biomolecules-12-01688-s001.zip › biomolecules-1913719-supplementary-11.17 -done/Data_7/USP25.pdf]

USP25

Homo sapiens:

```
>Q9UHP3 [Homo sapiens]
MTVEQNVLQQSAAQKHQQTFLNQLREITGINDTQILQQALKDSNGNLELAVAFLTAKNAK
TPQQEETTYYQTALPGNDRYISVGSQADTNVIDLTGDDKDDLQRAIALSLAESNRAFRET
GITDEEQAISRVLEASIAENKACLKRTPTEVWRDSRNPYDRKRQDKAPVGLKNVGNTCWF
SAVIQSLFNLLEFRRLVLNYKPPSNAQDLPRNQKEHRNLPFMRELRYLFALLVGTKRKYV
DPSRAVEILKDAFKSNDSQQQDVSEFTHKLLDWLEDAFQMKAEEETDEEKPKNPMVELFY
GRFLAVGVLEGKKFENTEMFGQYPLQVNGFKDLHECLEAAMIEGEIESLHSENSGKSGQE
HWFTELPPVLTFELSRFEFNQALGRPEKIHNKLEFPQVLYLDRYMHRNREITRIKREEIK
RLKDYLTVLQQRLERYLSYGSGPKRFPLVDVLQYALEFASSKPVCTSPVDDIDASSPPSG
SIPSQTLPSTTEQQGALSSELPSTSPSSVAAISSRSVIHKPFTQSRIPPDLPMHPAPRHI
TEEELSVLESCLHRWRTEIENDTRDLQESISRIHRTIELMYSDKSMIQVPYRLHAVLVHE
GQANAGHYWAYIFDHRESRWMKYNDIAVTKSSWEELVRDSFGGYRNASAYCLMYINDKAQ
FLIQEEFNKETGQPLVGIETLPPDLRDFVEEDNQRFEKELEEWDAQLAQKALQEKLLASQ
KLRESETSVTTAQAAGDPEYLEQPSRSDFSKHLKEETIQIITKASHEHEDKSPETVLQSA
IKLEYARLVKLAQEDTPPETDYRLHHVVVYFIQNQAPKKIIEKTLLEQFGDRNLSFDERC
HNIMKVAQAKLEMIKPEEVNLEEYEEWHQDYRKFRETTMYLIIGLENFQRESYIDSLLFL
ICAYQNNKELLSKGLYRGHDEELISHYRRECLLKLNEQAAELFESGEDREVNNGLIIMNE
FIVPFLPLLLVDEMEEKDILAVEDMRNRWCSYLGQEMEPHLQEKLTDFLPKLLDCSMEIK
SFHEPPKLPSYSTHELCERFARIMLSLSRTPADGR
```

Pongo abelii:

```
>XP_024095245.1 ubiquitin carboxyl-terminal hydrolase 25 isoform X3 [Pongo abelii]
MTVEQNVLQQSAAQKHQQTFLNQLREITGINDTQILQQALKDSNGNLELAVAFLTAKNAKTPQQEETTYY
QTALPGNDRYISVGSQADTNVIDLTGDDKDDLQRAIALSLAESNRAFRETGITDEEQAISRVLEASIAEN
KACLKRTPAEVWRDSRNPYDRKRQDKAPVGLKNVGNTCWFSAVIQSLFNLLEFRRLVLNYKPPSNAQDLP
RNQKEHRNLPFMRELRYLFALLVGTKRKYVDPSRAVEILKDAFKSNDSQQQDVSEFTHKLLDWLEDAFQM
KAEEETDEEKPKNPMVELFYGRFLAVGVLEGKKFENTEMFGQYPLQVNGFKDLHECLEAAMIEGEIESLH
SENSGKSGQEHWFTELPPVLTFELSRFEFNQALGRPEKIHNKLEFPQVLYLDRYMHRNREITRIKREEIK
RLKDYLTVLQQRLERYLSYGSGPKRFPLVDVLQYALEFASSKPVCTSPVDDIDASSPPSGSIPSQTLPST
TEQQGAPSSELPSTSPSSVAAISSRSVIHKPFTQSRIPPDLPMHPAPRHITEEELSVLESCLHRWRTEIE
NDTRDLQESISRIHRTIELMYSDKSMIQVPYRLHAVLVHEGQANAGHYWAYIFDHRESRWMKYNDIAVTK
SSWEELVRDSFGGYRNASAYCLMYINDKAQFLIQEEFNKETGQPLVGIETLPPDLRDFVEEDNQRFEKEL
EEWDAQLAQKALQEKLLASQKLRESETSVTTAQAAGDPEYLEQPSRSDFSKHLKEETIRIITKASHEHED
KSPETVLQSAIKLEYARLVKLAQEDTPPETDYRLHHVVVYFIQNQAPKKIIEKTLLEQFGDRNLSFDERC
HNIMKVAQSKLEMIKPEEVNLEEYEEWHQDYRKFRETTMYLIIGLENFQRESYIDSLLFLICAYQNNKGL
LSKGLYRGHDEELISHYRRECLLKLNEQAAELFESGEDREVNNGLIIMNEFIVPFLPLLLVDEMEEKDIL
AVEDMRNRWCSYLGQEMEPHLQEKLTDFLPKLLDCSMEIKSFHEPPKLPSYSTHELCERFARIMLSLSRT
PADGR
```

Mus musculus:

```
>NP_038946.2 ubiquitin carboxyl-terminal hydrolase 25 [Mus musculus]
MTVEQNVLQQSAAQKHQQTFLNQLREITGINDAQILQQALKDSNGNLELAVAFLTAKNAKTPPQEETGYY
QTALPGNDRYISVGSQADANVIDLTGDDKDDLQRAIALSLAESNRAFRETGITDEEQAISRVLEASIAEN
KACLKRTPIEVWRDSRNPYDRKRQEKAPVGLKNVGNTCWFSAVIQSLFNLLEFRRLVLNYKPPSNAQDLP
RNQKEHRNLPFMRELRYLFALLVGTKRKYVDPSRAVEILKDAFKSNDSQQQDVSEFTHKLLDWLEDAFQM
KAEEETDEEKPKNPMVELFYGRFLAMGVLEGKKFENTEMFGQYPLQVNGFKDLHECLEAAMIEGEIESLH
SDNSGKSGQEHWFTELPPVLTFELSRFEFNQALGRPEKIHNKLEFPQVLYLDRYMHRNREITRIKREEIK
RLKDYLTVLQQRLERYLSYGSGPKRFPLVDVLQYALEFASSKPVCTSPVDDIDASSSASGPLPSQSLPST
TEQQGPCASDLPGSSSPASGAALPLRSVIHKPFTQSRIPPDLPMHPAPRHITEEELCVLESCLHRWRTEI
ENDTRDLQESISRIHRTIELMYSDKSMIQVPYRLHAVLVHEGQANAGHYWAYIFDHRESRWMKYNDIAVT
KSSWEELVRDSFGGYRNASAYCLMYIDDKAQFLIQEEFNKETGQALVGMETLPPDLRDFVEEDNQRFEKE
LEEWDTQLAQRSLQEKLLAAPKLREAEASATTAQAGGADYLEQPSRSDLSKHWKEETLRVIAKASHDLED
KGPETVLQSAIKLEYSRLVKLAQEDTPPETDYRLHHVLVYFIQNQAPKKIIEKTLLEQFGDRNLSFDERC
HNIMKVAQAKLEMIKPEEVNLEEYEEWHADYKKFRETTMYLITGLENFQRESYIDSLLFLLCAYQNNKEL
LSKGPYRGHDGELISHYRRECLLKLNEQAAELFESGEDGDVNNGLIIMNEFIVPFLPLLLVDDMEEKDIL
AVEDMRNRWCSYLGQEMEANLQEKLTDFLPKLLDCSTEIKGFHEPPKLPSYSAHELCERFARIMLSLSRT
PADGR
```

Gallus gallus:

```
>XP_040518387.1 ubiquitin carboxyl-terminal hydrolase 25 isoform X5 [Gallus gallus]
MTVEQNVLQPGGAAKHQQTFLNQLREITGINDIQVLQQALKDSNGNLELAVAFLTAKNAKVPQQEEATYY
QTALPGNDRYISVGSQADTNVIDLTGDDKDDLQRAIALSLEESNRAFRETGITDEEQAISRVLEASIAEN
KASLKRTHPEVWSDSPNPYDRKRQDNCPVGLKNVGNTCWFSAVIQSLFNLLEFRRLVLNFSPPANAQDLP
RNQKEHRNLPFMRELRYLFALLVGSKRKYVDPSRAVEILKDAFKSNDSQQQDVSEFTHKLLDWLEDAFQI
KAEEERDGEKPKNPMVELFYGRFLAVGVLEGKKFENTEMFGQYPLQVNGFKDLHECLEAAMIEGEIESLH
SENSAKSGQEHWFTELPPVLTFELSRFEFNQALGRPEKIHNKLEFPPILYLDRYMHKNRELTRIKRDEIK
RLKEYLTVLQQRLERYLSYGSGPKRFPLVDVLQYALEFASSKPVCTSPVDDLGASAPSSGTLPAQTSPST
IEQQGPSSSDVPSTSPVQRSVIHKPFTQSRIPPDLPMHPAPRHITEEELSVLEGCLHRWRTEVENDTRDL
QESISRIHRTIELMYFDKTMIQVPYRLHAVLVHEGQANAGHYWAYIYDHHQNRWMKYNDISVTKSTWEEL
ERDSFGGYRNASAYCLMYINDKEQYLIEEEFNKETGQILVGMDTLPSDLRDYVKEDNKRFEKELEEWDAE
LAQKAQQEKLLSQIPRAPAPSPAPVQAGEPEYLEQPSRTDLSKHLKEDSVQAINKALAEQEDRGPEAIMD
TAIKLEYTRLLKLAQEDPPPECDYRLRHAIVYFIQNQAPKKIIERTLLEQFGDHNLSFDERCRNIMKVAQ
AKLEMIKPDEVNMEEYERWHQDYRNFRETTMFLMVGLEFFQKKSYMEALLYLIYAYQNNKELLSKGPYRG
HDEELISHYRRECLLKLNEHAAALFESGDDQEVNNGLIIMNELIVPCLPLLLVDEMEEKDIVAVEDMRNR
WCSYLGQEMEPNLQEKLTDFLPKLLDCSTEIKGFNDPPKLPSYSTHELCERYARIMLSLSRTPADGR
```

Xenopus laevis:

```
>OCT93923.1 hypothetical protein XELAEV_18011587mg [Xenopus laevis]
MTVEQNVLQQNSQKHQQTFLNQLREITGINDLQVLQQALKDSNGNLELAVAFLTAKNAKLPQPEEATYYQ
TALPNNDRYISVGSQADTNVIDLTVDDKDDLQRAIALSLEESNRTFRETGITDEEQAISRVLEASIAENK
ACLKRTHTEVWSDSPNPYDRKRQENCPVGLKNVGNTCWFSAVIQSLFNLLEFRRLVLNYNPPSSAQDVPR
NQKEHRNLPFMRELRYLFSLMVSSKRKYVDPSRAVEILKDAFKSNDSQQQDVSEFTHKLLDWLEDAFQIK
AEEERDGEKPKNPMVELFYGRFLAVGVHEGKKFENTEMFGQYPLQVNGFKDLHECLEAAMIEGEIESLHS
ENSGKSGQEHWFTELPPVLTFELSRFEFNQALGRPEKIHNKLEFPPCLYMDRYMHKNREITRLKRDEIKR
LKDHLTVLQQRLERYLSYGSGPKRFPLADVLQYALEFASSKPVCTSPVEDIDVSAPPSGCVATQTMQSTT
EHQGSSSASETQLPTQRSVIHKPFTQSRIPPDLPMHPAPRNITDEELSVLEGCLHRWRTEVETDTRDLQD
SIGRIHRTIELMYSDKQMNQVPYKLHAVLVHEGQANAGHYWAYIFDHHEQRWMKYNDISVTKSSWEELER
DSFGGYRNASAYCLMYINDEEKCLMQEEYDKETGQVLKGMDILPLDLREYIKEDNRRFEKELEAWDKEQL
RKAEQEKMILAQKALKQAPLVVAAPPNEPEYMEQPARADAPNETSEETTNVITQAATESEDKGPEAVLQM
AIKTEYARLVKLAQEDPLPEIDYRLQHAIIYFIQSQAPKKIIERTLLEQFADRNLSFDERCRNIMKVAQA
KLEMIKPDEVNMEEYRKWHKDYRMFRETTMYILIGLELFQNKSYVEALLYLIYGYQYNKELLSKGLYRGH
DEQLISHYRRECFLKLNEQAAAMFESGDMDEVNNGLASMNELIVPCLPLLLVDDMEEKDIVAVEDMRNRW
CSYLGQEMEPNLQEKLTDFLPKLLDCSTEIKGLNDPPKLPNFSTHELCERFARIMLSLSRMPADGR
```

Salmo salar:

>XP_013993323.1 PREDICTED: ubiquitin carboxyl-terminal hydrolase 25-like isoform X9 [Salmo salar]
MTVEQNVLQQHSQKHQQTLLNQLREVTGTTDVQLLQQALQVSNGDLAEAVAFLTEKNAKVPQQDEAAYYQ
TTQVGNDRYISVGSQADTNVIDLTGDDKDDLQRAIALSLEESSRAFRETGITDEEQAISRVLEASIAENK
ASLKRTHTEVWSDSPNPHDRKRMENCPVGLKNVGNTCWFSAVIQSLFNLLEFQRLVLHYSPPARVQDLPR
NQKEHRNLPFMQELRNLFSLMVGSKRKYVDPSRAVEILKDAFKSSESQQQDVSEFTHKLLDWLEDAFQMK
AEEDREGEKPKNPMVELFYGRFQAVGVLEGKKFENTEMFGQYPLQVNGFKDLHECLEAAMIEGEIESLHS
SAENSAKSGQEHWFTELPPVLTFELSRFEFNQALGRPEKIHNKLEFPSMLYMDRYMDRNREITRIKREEI
RRLKEQLTLLQQRLERYLSYGSGPKRFPLADVLQYAMEFASSKPVCTSPLEDIDTSAPPGGTTGQLLPAL
STAEQGPSCTLPEGLAAAPTQAPSTQQRVPIHKPFTHSRLPPDLPMHPAPRHITEEELRVLEGCLHRWRS
EVENDTHDLQGSISRIHRTIELMYSDKSMMQVPFRLHAVLVHEGQANAGHYWAYIYDPHQQRWMKYNDIS
VTKSSWEELVRDSFGGYRNASAYCLMYINDKKPFLIEEEFDKETGQMLSGLDKLPSDLKAYVKEDNGLFD
KEMEEWDKLQARKAQQEKLALAAAAAATAETTTSHQPMSTEPSPPDSWGPQQDPEYMEQPSPTDDSKHLQ
EDTERAISKAAAEQQEERSPEGLLTAAIKVEYTRLLRFAQEDTAPENDYRLQHVIVYFIHNQAPKKILER
TLLMQFADRNLSFDERCKSIMKVARAKLDLVKPEEVNMEEYEMWHQDYRNFRETTIFLMIGLELFQKKCF
VEALMYLIYSYQYNRELLVKGLYRGHNDELIGHYRRECLLKLNENAARMFESGEEPEVCNGLSIMNELVV
PCIPLLLVHDTEKDLLAVEDMRNRWCSYLGQEMEPNLQEKLTDFLPKLLDCSTEIKSFHDPPKLPTYSTL
ELVERYGRVMASLCRVPADGR

Petromyzon marinus:

>XP_032826770.1 ubiquitin carboxyl-terminal hydrolase 25-like isoform X3 [Petromyzon marinus]
MADRSQWTVMTVEHRETAGIPSRNNQIILNQLREITGNSDVIALQQALQAAQGDVSQAVALLTETPNQNA
GCALSSSQSHRSLPNQSQNSTGVIDLTGDDKDDLQRAIALSLQETSERAVRADLTTEEQDISRVLEESLA
EASGPGGVKRKRDSPCACSCPREGDGASGGGAAEGEVAVSAVRAPGAPVGLKNVGNTCWFSAVVQSLFHL
PGFRRLILGFTLPDSRLQNPQSENERVGLCFMRELRVLFALMVGSVRGFVDPSPAVHILKGAFRGADTQQ
DVSEFTHKLLDWLEDAFRISAEESRDDSLKTVNPMVDLFYGRARSIGTNQGKRFAVLEGFGPLPVRVSGF
HDLHECLEHATMAGDIEQQQQQPPACASSGQELWFSELPPVLTLELSRFRFNTEVGRPEKIHSRLEFPDT
IYMDRYMESNKEVTRIKRDEIRRLSEQQSHLQQTLARYQSYGSGPQRYPLVDILSCALQFASTPPATVLL
PPTSLVNHKGPSSAAKPPCTLSTNHVTTSPTVSSSQVVSTNNIALFPIVESTPIPSTNHVVSSSAASPIP
VLTTNHSDLSSTVPPTPMQSTNRVVLSPTSLPIPVPLTNHVAPSCATTSTPTTSINHTAPSPAVLPIHIA
STNHVAPPTVVKSPADRALLVDDTGPVLINGVAELGVALPPSDESSKQPKAEALDINGDLSESESTAAPG
CVVADGEVHQLRQQPQPPSAAAAVACSAPEPHHTQERHGARARQEVPAQQKSGEQNRTGDPPPPGTEQRT
ADSQQASVLQQGSLQQEAMQVDTLQENVQQGEEEGSQQKDITQQQQELAQGESNQQTETQQRFDLQSEAN
QVSLLQDMQQQQHRGNVQQEHVQQGNPQQGNFQQENSEQCKEQQEGSTQQCDVQRVQAVEARQEGGRLEW
HGCPAPQAVTALELELVRAVLSRWNSQVQRELQDLQDSICRIKRTIDLMYTDKTFRQLPYRLHAVLVHDG
QAMAGHYWAYVREHARLPAAAAASSSSSSSGNSASSNSNSVGASDESSGSDSCWRKFNDVRVSRASWAEL
VRDSYGGGARSASAYCLVYVNARAHDFTSELAGEAVGDLSSLSPALRDLVAKDNHRFLSEMAMRGNGPER
PAEPDADAREPGQRAEPREHEQEERRHEHGGEQAPARAAPPAAAAAAAASREGEGSGAAREQERADKHSQ
TNSLEQEAEPTRERVDGRARAGEREETRVHEGRAPDVASISETQRQGSEADGSASLPGREGTSAPSPSPS
GTEEYMKVGPDTPTEKPRVERVVEVSIPDVGTIVVQAEEHGYNDEVMLTPAMQGVLMAVARARLVREAQG
PEATLIEAFRVEYARLRTLAQESYPDASSPASPPLAPAGASGAAATAWRGREERLEHVVIYLFQNEAPNR
VVERALLEQFADRNLSYDEQLIKIMKVAQAKLQEISPEDMDPAEYQKWHQEYELFRQVCVYLVMGIEKYQ
EGRYTEALPCLMHAHAVNEELLARGQRRGVRRDVLARYRRVCVRRVNEACALTFGTGDVAQATRSLAVMT
ELVLPAMALLAPLGSSCEGGDAGDEAAVARESDLCAVELMRDRWCSYLGRDDMASELQELLTDFLPRLLD
YQPEPAPPTQLTPPPLQGPPPPPPPPSEPRKCESSGENWRGLRAPPRLKAYSPHTLAEKMAEVLRVNRPR
EA

Branchiostoma belcheri:

>XP_019624636.1 PREDICTED: ubiquitin carboxyl-terminal hydrolase 25-like [Branchiostoma belcheri]
MSEPLDHAYQAGKGDLAEVVSILTNQNVSLVTNRNDEVVQVQVQVRNAQRPSGQAGRPDGRANTQEPAGV
IDLTADTEDKDQEDLQRAIALSLQEAQTANTANRPQGECRPKRRKSSVSWDNVQETPREPGWPVGLKNVG
NTCWFSAVIQSLFHLPVFRDLVLGFRFFPKDWPANSRESLVLHFMQELRCLFARMQGSNRKYIDPTCVVK
SLRELIPFRAGETQQDVSEFTHKLLDLLEEAFIIVQRRQSTQPGSSASANTSENGGDKKSNPMLDLFYGR
IQAEGMHAGKMFTNQQTVGQYTLQVNGFKDLHESLEGATAQGEIETGHEEGATKSGLEHWFTHLPPVLTF
ELSRFEFNQKLGKAEKIHHKLEFPKFLYMDRYLVKNKELTRLKREEMKSMKEEMDTLQAKLDSFLNYGSG
YKRLPLPDILKYTLEFAEKSTVQVTDGQVQRSNGSGSSKAANGNGHNGSEKKTTDEEDGSSDDFEVISAV
HSADSLACLSKKTLDAVATKKAAPVPTSEEMAIPSKVHIPCPHHVTKEELAVLQTCLRRWRHEAEKEVKG
LQDRILYLQAKIENLYEDNSLKQVPYQLHAVLVHEGQASAGHYWAYVRDHTQNRWLKFNDVTVTGMSWGE
LLRESEGGYHHASAYCLMYIDITRKDLVEVKLQWSQYDELKLMVLAENERFIAQQRDLESKQRRTMVVRE
EDPPANHGRGQGTTSESSSGEVADQPIQGQEHRVDVQRKQPPQVKPKPVTTETQQGVRLGRTSQQSTPLD
HSGHVLQATMKTILEVSNIQEQQGPEKALIKAVNIEYHRLRSLVEKGISRQYDPRLEHCVVYLILNGAAR
VIVERTLVEQFADRGLSGDSKSIEIMKVAQSKLKIMQLSEENLKAYEKWHADYGLFQKTVMFLLRGIEFF
HQERFPEALTYLVHAWTYNQQLLGEGKNYIMAADSSLITHYRTQCLKSLSEQACSLFESGDTESVDEGLQ
LMVELVVPCMALLQELGGTDSDQAIAEEIRSNWCDYLGQDLPDWCQEKLQDFLPQLLDCSGDLQQLRTPP
AVWPSQHLAEWFSTVMQAVVLAEPDTVD

Drosophila melanogaster:

No homolog found.

Hydra vulgaris:

No homolog found.

Nematostella vectensis:

>XP_032229076.1 ubiquitin carboxyl-terminal hydrolase 25 [Nematostella vectensis]
MTVEYPSSMKKVTQENEAIHQVCEITGVSIEEAQNAFAAGNYNVDEAISILTQSNLKSSTTHHVVKKTPV
EQEEVIDLTKDKDDRDDDLEKAIALSLQESQAGQPQGITAEEQDISRVLEASLIENKPGQKRKRGDPWMM
FVDPVNPYDRKRVEGTPVGFKNVGNTCWFSAVIQALFHLPVFRKLVLGYQPPAANEQKDAHQLLFMLELR
KLFALLVASKRKYVDPIKAIEILKEALQYNGSDSNPGNQQDVSEFTHKLLEWLEDTFNTESKEEEKSQAN
SSSSNPVVELFFGQSKVDGVYEGNRFSNIETFGACPLQVQGYSNLHDSLEGGMAARЕIEPANHGDTQNSG
QEHWFTRLPLVLTFMLSRFLFNQSIGRAEKIHEKFLFDKCIYMDRYMERNKELSRRRREEVKTLKERHQL
LKSSLDRYTNYGSGAKRSPIQDILDSALNFVRSTPPSDSTDVEMHTPIPGSPVTFGTTSAGPSTLSDTRT
PSLHPAPRYVTESELETIATSLRRWRTEVEQDVLDISTSLKEVEEAISVMYNDEEMKTIPYRLYAVLVHE
GQANGGHYWAYINDPLDRRWRKFNDITVSEVTWEEVERESLGGYRNVSAYCLLYCDARREEVIRAGNTPE
VLSAELQQMVNEDNASFEQELRDWDEKQAKAAKEAKSQALVVARPDLVLDAEASCAVSPRAHSPAAARSE
SVLTANLGVLPSLVDSSGKKLTIDEAITQWCNNEKTRLDKLAREYPPEYPGDVRLEHLGIYLMRCSAPDF
TVECAILESVIDEVKAKDQSLMPYRKRVQEFLEREKGTDRAADYKRWKEMYMTFKLINSCFLTGLEHFHS
QKYKESLPYLVHAYELNRKLSDPTNNARSMDQRLLAHCRRVCFLKLNELAISLFESDDYKTLCEGLDLVS
EQVVPCIPQLVTSPYAEDREAVEHMRNNWFTYLGGTVDEKRQEKLQDFLPKLLDGSTDGNFEEMRPPPVM
RPTNSKDLCERFTRAMEIVHANMPFAAKA

Amphimedon queenslandica:

>XP_019852971.1 PREDICTED: ubiquitin carboxyl-terminal hydrolase 25-like isoform X2 [Amphimedon queenslandica]
MTSTKAEVMSDKAKQDIMQLQEIFGITSTEAENSYQYYKNSLSDAVVGLSSLQENGRVSLEEAIAKSPHS
SESTSGDTSQGLVATCANNPMAAPAGGGGGMADSDQPTGSTNTNPIDLTQESQDDDLQKALALSLQDMQH
QGGGISLEDQELSKALEASIADTHTEYGPVSRFVDPLNPHERKKIEGFPLGLKNIGNTCWFSAVIQSLFN
IPAFRDSILHFNFKTSDQTEEDLPHCVLFVLELQKLFALLLESHQRYIDPKQAIDLLNASFQTLGGNHMQ
QDVSEFKHKLLEWIEDAFNYQDKTKNNPIIELFTGECVTEGKINETSFVNRSQFGAFPVQIEGHAHLHDS

LEAATVNTEMEQETWFTHLPPVLVLELSRFKYNQLTGQAEKIHDSLSFDKTLYLDRYLHSNASQTRLLRK
EMGLLRQKMRKIEETLKSSRLEGVLGAEGASPNVQEEGRPSPRSGSKEEVEFVRKWLEQWRSEMDTEISE
LKSSLSATRESLDAVYSDPSMNRTSYHLHSVLVHQGQASRGHYWAYVRKKKTKKRKATREMGIQASYLDD
EQTEEEGVDKSSSEEKREEGEGHEDMEVTTTGGDTDVETFSSPLSSCEPSSGPASVAPPEVKREEGSKED
EEEIWLKYNDVSVTTVDWEEVKRESFGGRGGESACNSNGPGTNTSAYCLLYVNSEAAKTWREEVHLLSLG
LQRYIAYHNLAFERELKAYDLKVQLQQLKTSSEGKELIKPAKAVRFEYPKGEEDPGASTNGGHDMMALAS
DAKRARAGSPSNLEDMQIDLDNLPPVDPALLNQLSMRLSDKLQMFIQDVPGTIIESRLLTDFHYAHACQT
SEKVRQAILYEHVIQDLKRENDLNMSLIARKYFVEICKDPQIYQELQDVRLLYSRFCSCCYHFIQSVNEA
KRNSWCLSLAHIINCFYLNSSISAQAGDKYSLDQQLLGRFRCKALLMVSEIGTVAFTSGEVSSSIVIGND
YIVPGLKAFSLHPFPGDDSILEKVREEWCQCLDQSSLTEDDSDKLADLLMKIVEDYSKAPNIKIPQAPSN
FDTQRLAAQYTTAYKTLTSEILL

Alignment

```
XP_019852971.1[A.queenslandica]      ---------MTSTKAEVM----SDKAKQDIMQLQEIFGITSTEAE-NSYQYYKNSLSDAV     46
XP_019624636.1[B.belcheri]           ----------------------------------------MSEPLDHAYQAGKGDLAEV-     19
XP_032826770.1[P.marinus]            MADRSQWTVMTVEHRETAG-IPSRNNQIILNQLREITGNSDVIALQQALQAAQGDVSQA-     58
XP_032229076.1[N.vectensis]          ---------MTVEYPSSMKK--VTQENEAIHQVCEITGVSI-EEAQNAFAAGNYNVDEA-     47
XP_013993323.1[S.salar]              ---------MTVEQNVLQQH-SQKHQQTLLNQLREVTGTTDVQLLQQALQVSNGDLAEA-     49
OCT93923.1[X.laevis]                 ---------MTVEQNVLQQN-SQKHQQTFLNQLREITGINDLQVLQQALKDSNGNLELA-     49
XP_040518387.1[G.gallus]             ---------MTVEQNVLQPGGAAKHQQTFLNQLREITGINDIQVLQQALKDSNGNLELA-     50
NP_038946.2[M.musculus]              ---------MTVEQNVLQQSAAQKHQQTFLNQLREITGINDAQILQQALKDSNGNLELA-     50
Q9UHP3[H.sapiens]                    ---------MTVEQNVLQQSAAQKHQQTFLNQLREITGINDTQILQQALKDSNGNLELA-     50
XP_024095245.1[P.abelii]             ---------MTVEQNVLQQSAAQKHQQTFLNQLREITGINDTQILQQALKDSNGNLELA-     50
                                                                                   ::    : .:  .

XP_019852971.1[A.queenslandica]      VGLSSLQENGRVSLEEAIAKSPHSSESTSGDTSQGLVATCAN-NPMAAPAGGGGGMADSD     105
XP_019624636.1[B.belcheri]           -----------------VSILTNQNVSLVTNRNDEVVQVQVQVRNAQRPSGQAGRPD--G     60
XP_032826770.1[P.marinus]            -----------------VALLTETPNQNAG------CALSSS----------QSHRS--L     83
XP_032229076.1[N.vectensis]          -----------------ISILTQSNLKSSTTHH----------------------V--V     65
XP_013993323.1[S.salar]              -----------------VAFLTEKNAKVPQQDEAAYYQTTQV----------GNDRY--I     80
OCT93923.1[X.laevis]                 -----------------VAFLTAKNAKLPQPEEATYYQTALP----------NNDRY--I     80
XP_040518387.1[G.gallus]             -----------------VAFLTAKNAKVPQQEEATYYQTALP----------GNDRY--I     81
NP_038946.2[M.musculus]              -----------------VAFLTAKNAKTPPQEETGYYQTALP----------GNDRY--I     81
Q9UHP3[H.sapiens]                    -----------------VAFLTAKNAKTPQQEETTYYQTALP----------GNDRY--I     81
XP_024095245.1[P.abelii]             -----------------VAFLTAKNAKTPQQEETTYYQTALP----------GNDRY--I     81
                                                      ::       .

XP_019852971.1[A.queenslandica]      QPTGSTNTNPIDLTQE---SQDDDLQKALALSLQDMQHQ--GGGISLEDQELSKALEASI     160
XP_019624636.1[B.belcheri]           RANTQEPAGVIDLTADTEDKDQEDLQRAIALSLQEAQTANTANRPQGECR----------     110
XP_032826770.1[P.marinus]            PNQSQNSTGVIDLTG----DDKDDLQRAIALSLQETSERAVRADLTTEEQDISRVLEESL     139
XP_032229076.1[N.vectensis]          KKTPVEQEEVIDLTKDKD-DRDDDLEKAIALSLQESQA-GQPQGITAEEQDISRVLEASL     123
XP_013993323.1[S.salar]              SVGSQADTNVIDLTG----DDKDDLQRAIALSLEESSRAFRETGITDEEQAISRVLEASI     136
OCT93923.1[X.laevis]                 SVGSQADTNVIDLTV----DDKDDLQRAIALSLEESNRTFRETGITDEEQAISRVLEASI     136
XP_040518387.1[G.gallus]             SVGSQADTNVIDLTG----DDKDDLQRAIALSLEESNRAFRETGITDEEQAISRVLEASI     137
NP_038946.2[M.musculus]              SVGSQADANVIDLTG----DDKDDLQRAIALSLAESNRAFRETGITDEEQAISRVLEASI     137
Q9UHP3[H.sapiens]                    SVGSQADTNVIDLTG----DDKDDLQRAIALSLAESNRAFRETGITDEEQAISRVLEASI     137
XP_024095245.1[P.abelii]             SVGSQADTNVIDLTG----DDKDDLQRAIALSLAESNRAFRETGITDEEQAISRVLEASI     137
                                               ****     . .:**::*:**** : .          * :

XP_019852971.1[A.queenslandica]      ADTHTEYGPVSR-------------------FVDPLNPHERKKIEGFPLGLKNIGNTCW     200
XP_019624636.1[B.belcheri]           -------PKRRKSSVSW-------------------DNVQETPREPGWPVGLKNVGNTCW     144
XP_032826770.1[P.marinus]            AEASGPGGVKRKRDSPCACSCPREGDGASGGGAAEGEVAVSAVRAPGAPVGLKNVGNTCW     199
XP_032229076.1[N.vectensis]          IENKP--GQKRKRGDPWMMF-------------VDPVNPYDRKRVEGTPVGFKNVGNTCW     168
XP_013993323.1[S.salar]              AENKA--SLKRTHTEVW----------------SDSPNPHDRKRMENCPVGLKNVGNTCW     178
OCT93923.1[X.laevis]                 AENKA--CLKRTHTEVW----------------SDSPNPYDRKRQENCPVGLKNVGNTCW     178
XP_040518387.1[G.gallus]             AENKA--SLKRTHPEVW----------------SDSPNPYDRKRQDNCPVGLKNVGNTCW     179
NP_038946.2[M.musculus]              AENKA--CLKRTPIEVW----------------RDSRNPYDRKRQEKAPVGLKNVGNTCW     179
Q9UHP3[H.sapiens]                    AENKA--CLKRTPTEVW----------------RDSRNPYDRKRQDKAPVGLKNVGNTCW     179
XP_024095245.1[P.abelii]             AENKA--CLKRTPAEVW----------------RDSRNPYDRKRQDKAPVGLKNVGNTCW     179
                                                                             .  :    *:*:**:*****

XP_019852971.1[A.queenslandica]      FSAVIQSLFNIPAFRDSILHFNFKTSDQT---EEDLPHCVLFVLELQKLFALLLESHQRY     257
XP_019624636.1[B.belcheri]           FSAVIQSLFHLPVFRDLVLGFRFFP--KDWPANSRESLVLHFMQELRCLFARMQGSNRKY     202
XP_032826770.1[P.marinus]            FSAVVQSLFHLPGFRRLILGFTLPDSRLQNPQSENERVGLCFMRELRVLFALMVGSVRGF     259
XP_032229076.1[N.vectensis]          FSAVIQALFHLPVFRKLVLGYQPPAAN-----EQKDAHQLLFMLELRKLFALLVASKRKY     223
XP_013993323.1[S.salar]              FSAVIQSLFNLLEFQRLVLHYSPPARVQDLPRNQKEHRNLPFMQELRNLFSLMVGSKRKY     238
OCT93923.1[X.laevis]                 FSAVIQSLFNLLEFRRLVLNYNPPSSAQDVPRNQKEHRNLPFMRELRYLFSLMVSSKRKY     238
XP_040518387.1[G.gallus]             FSAVIQSLFNLLEFRRLVLNFSPPANAQDLPRNQKEHRNLPFMRELRYLFALLVGSKRKY     239
NP_038946.2[M.musculus]              FSAVIQSLFNLLEFRRLVLNYKPPSNAQDLPRNQKEHRNLPFMRELRYLFALLVGTKRKY     239
Q9UHP3[H.sapiens]                    FSAVIQSLFNLLEFRRLVLNYKPPSNAQDLPRNQKEHRNLPFMRELRYLFALLVGTKRKY     239
XP_024095245.1[P.abelii]             FSAVIQSLFNLLEFRRLVLNYKPPSNAQDLPRNQKEHRNLPFMRELRYLFALLVGTKRKY     239
                                     ****:*:**::  *:  :* :           ..     : *: **: **: :  : : :

XP_019852971.1[A.queenslandica]      IDPKQAIDLLNASFQTLGG---NHMQQDVSEFKHKLLEWIEDAFNYQDK----------     303
XP_019624636.1[B.belcheri]           IDPTCVVKSLRELIPFR----AGETQQDVSEFTHKLLDLLEEAFIIVQRRQSTQPGSSAS     258
XP_032826770.1[P.marinus]            VDPSPAVHILKGAFRG------ADTQQDVSEFTHKLLDWLEDAFRISAEES---------     304
XP_032229076.1[N.vectensis]          VDPIKAIEILKEALQYNGSDSNPGNQQDVSEFTHKLLEWLEDTFNTESKEE---------     274
XP_013993323.1[S.salar]              VDPSRAVEILKDAFKS-----SESQQQDVSEFTHKLLDWLEDAFQMKAEED---------     284
OCT93923.1[X.laevis]                 VDPSRAVEILKDAFKS-----NDSQQQDVSEFTHKLLDWLEDAFQIKAEEE---------     284
XP_040518387.1[G.gallus]             VDPSRAVEILKDAFKS-----NDSQQQDVSEFTHKLLDWLEDAFQIKAEEE---------     285
NP_038946.2[M.musculus]              VDPSRAVEILKDAFKS-----NDSQQQDVSEFTHKLLDWLEDAFQMKAEEE---------     285
Q9UHP3[H.sapiens]                    VDPSRAVEILKDAFKS-----NDSQQQDVSEFTHKLLDWLEDAFQMKAEEE---------     285
XP_024095245.1[P.abelii]             VDPSRAVEILKDAFKS-----NDSQQQDVSEFTHKLLDWLEDAFQMKAEEE---------     285
                                     :**  .:. *.  :           *******.****: :*::*    .

XP_019852971.1[A.queenslandica]      ---------TKNNPIIELFTGECVTEGKINETSFVNRSQFGAFPVQIEGHAHLHDSLEAA     354
XP_019624636.1[B.belcheri]           ANTSENGGDKKSNPMLDLFYGRIQAEGMHAGKMFTNQQTVGQYTLQVNGFKDLHESLEGA     318
XP_032826770.1[P.marinus]            --RDD--SLKTVNPMVDLFYGRARSIGTNQGKRFAVLEGFGPLPVRVSGFHDLHECLEHA     360
XP_032229076.1[N.vectensis]          --EKSQANSSSSNPVVELFFGQSKVDGVYEGNRFSNIETFGACPLQVQGYSNLHDSLEGG     332
XP_013993323.1[S.salar]              --RE---GEKPKNPMVELFYGRFQAVGVLEGKKFENTEMFGQYPLQVNGFKDLHECLEAA     339
OCT93923.1[X.laevis]                 --RD---GEKPKNPMVELFYGRFLAVGVHEGKKFENTEMFGQYPLQVNGFKDLHECLEAA     339
XP_040518387.1[G.gallus]             --RD---GEKPKNPMVELFYGRFLAVGVLEGKKFENTEMFGQYPLQVNGFKDLHECLEAA     340
NP_038946.2[M.musculus]              --TD---EEKPKNPMVELFYGRFLAMGVLEGKKFENTEMFGQYPLQVNGFKDLHECLEAA     340
Q9UHP3[H.sapiens]                    --TD---EEKPKNPMVELFYGRFLAVGVLEGKKFENTEMFGQYPLQVNGFKDLHECLEAA     340
XP_024095245.1[P.abelii]             --TD---EEKPKNPMVELFYGRFLAVGVLEGKKFENTEMFGQYPLQVNGFKDLHECLEAA     340
                                              .  **:::** *.    *    . *   . .*   :::.*. .**:.** .

XP_019852971.1[A.queenslandica]      TVNTEME------------QETWFTHLPPVLVLELSRFKYNQLTGQAEKIHDSLSFDKT     401
XP_019624636.1[B.belcheri]           TAQGEIETGHE--EGATKSGLEHWFTHLPPVLTFELSRFEFNQKLGKAEKIHHKLEFPKF     376
XP_032826770.1[P.marinus]            TMAGDIEQQQQQPPACASSGQELWFSELPPVLTLELSRFRFNTEVGRPEKIHSRLEFPDT     420
XP_032229076.1[N.vectensis]          MAAREIEPANH--GDTQNSGQEHWFTRLPLVLTFMLSRFLFNQSIGRAEKIHEKFLFDKC     390
XP_013993323.1[S.salar]              MIEGEIESLHSSAENSAKSGQEHWFTELPPVLTFELSRFEFNQALGRPEKIHNKLEFPSM     399
OCT93923.1[X.laevis]                 MIEGEIESLHS--ENSGKSGQEHWFTELPPVLTFELSRFEFNQALGRPEKIHNKLEFPPC     397
XP_040518387.1[G.gallus]             MIEGEIESLHS--ENSAKSGQEHWFTELPPVLTFELSRFEFNQALGRPEKIHNKLEFPPI     398
NP_038946.2[M.musculus]              MIEGEIESLHS--DNSGKSGQEHWFTELPPVLTFELSRFEFNQALGRPEKIHNKLEFPQV     398
Q9UHP3[H.sapiens]                    MIEGEIESLHS--ENSGKSGQEHWFTELPPVLTFELSRFEFNQALGRPEKIHNKLEFPQV     398
XP_024095245.1[P.abelii]             MIEGEIESLHS--ENSGKSGQEHWFTELPPVLTFELSRFEFNQALGRPEKIHNKLEFPQV     398
                                         ::*              * **:.** **.: **** :*   *: ****  : *

XP_019852971.1[A.queenslandica]      LYLDRYLHSNASQTRLLRKEMGLLRQKMRKIEETLKSSRLE------------------     442
XP_019624636.1[B.belcheri]           LYMDRYLVKNKELTRLKREEMKSMKEEMDTLQAKLDSFLNYGSGYKRLPLPDILKYTLEF     436
XP_032826770.1[P.marinus]            IYMDRYMESNKEVTRIKRDEIRRLSEQQSHLQQTLARYQSYGSGPQRYPLVDILSCALQF     480
XP_032229076.1[N.vectensis]          IYMDRYMERNKELSRRRREEVKTLKERHQLLKSSLDRYTNYGSGAKRSPIQDILDSALNF     450
XP_013993323.1[S.salar]              LYMDRYMDRNREITRIKREEIRRLKEQLTLLQQRLERYLSYGSGPKRFPLADVLQYAMEF     459
OCT93923.1[X.laevis]                 LYMDRYMHKNREITRLKRDEIKRLKDHLTVLQQRLERYLSYGSGPKRFPLADVLQYALEF     457
XP_040518387.1[G.gallus]             LYLDRYMHKNRELTRIKRDEIKRLKEYLTVLQQRLERYLSYGSGPKRFPLVDVLQYALEF     458
NP_038946.2[M.musculus]              LYLDRYMHRNREITRIKREEIKRLKDYLTVLQQRLERYLSYGSGPKRFPLVDVLQYALEF     458
Q9UHP3[H.sapiens]                    LYLDRYMHRNREITRIKREEIKRLKDYLTVLQQRLERYLSYGSGPKRFPLVDVLQYALEF     458
XP_024095245.1[P.abelii]             LYLDRYMHRNREITRIKREEIKRLKDYLTVLQQRLERYLSYGSGPKRFPLVDVLQYALEF     458
                                     :*:***:  * . :*  *.*:  : :    ::  *

XP_019852971.1[A.queenslandica]      ------------------------------------------------------------     442
XP_019624636.1[B.belcheri]           AEKSTVQVTDGQVQRSNGSGSSKAANGN--------------------------------     464
```

```
XP_032826770.1[P.marinus]           ASTPPATVLLPPTSLVNHKGPSSAAKPPCTLSTNHVTTSPTVSSSQVVSTNNIALFPIVE    540
XP_032229076.1[N.vectensis]         VRSTPP----------------------SDSTDVEMHT----------------------    466
XP_013993323.1[S.salar]             ASSKPV----------------------CTSPLEDIDT----------------------    475
OCT93923.1[X.laevis]                ASSKPV----------------------CTSPVEDIDV----------------------    473
XP_040518387.1[G.gallus]            ASSKPV----------------------CTSPVDDLGA----------------------    474
NP_038946.2[M.musculus]             ASSKPV----------------------CTSPVDDIDA----------------------    474
Q9UHP3[H.sapiens]                   ASSKPV----------------------CTSPVDDIDA----------------------    474
XP_024095245.1[P.abelii]            ASSKPV----------------------CTSPVDDIDA----------------------    474

XP_019852971.1[A.queenslandica]     ------------------------------------GVL---------------------    445
XP_019624636.1[B.belcheri]          ------------------------------------GHNG--------------------    468
XP_032826770.1[P.marinus]           STPIPSTNHVVSSSAASPIPVLTTNHSDLSSTVPPTPMQSTNRVVLSPTSLPIPVPLTNH    600
XP_032229076.1[N.vectensis]         -------------------------------PIPGSP-----------------------    472
XP_013993323.1[S.salar]             -------------------------------SAPPGGTTG--------------------    484
OCT93923.1[X.laevis]                -------------------------------SAPPSGCVA--------------------    482
XP_040518387.1[G.gallus]            -------------------------------SAPSSGTLP--------------------    483
NP_038946.2[M.musculus]             -------------------------------SSSASGPLP--------------------    483
Q9UHP3[H.sapiens]                   -------------------------------SSPPSGSIP--------------------    483
XP_024095245.1[P.abelii]            -------------------------------SSPPSGSIP--------------------    483

XP_019852971.1[A.queenslandica]     ---------------GAEG-----------------------------------------    449
XP_019624636.1[B.belcheri]          --------SEKKTTDEEDGSSDDFEVI---SAV---------------------------    490
XP_032826770.1[P.marinus]           VAPSCATTSTPTTSINHTAPSPAVLPIHIASTNHVAPPTVVKSPADRALLVDDTGPVLIN    660
XP_032229076.1[N.vectensis]         ----------VTFGTTSAGPST--------------------------------------    484
XP_013993323.1[S.salar]             -------QLLPALSTAEQGPSCTLP-----------------------------------    502
OCT93923.1[X.laevis]                -------TQTMQSTTEHQGSSSAS------------------------------------    499
XP_040518387.1[G.gallus]            -------AQTSPSTIEQQGPSSSDV-----------------------------------    501
NP_038946.2[M.musculus]             -------SQSLPSTTEQQGPCASDL-----------------------------------    501
Q9UHP3[H.sapiens]                   -------SQTLPSTTEQQGALSSEL-----------------------------------    501
XP_024095245.1[P.abelii]            -------SQTLPSTTEQQGAPSSEL-----------------------------------    501
                                                      .

XP_019852971.1[A.queenslandica]     ------------------------------------------------------------    449
XP_019624636.1[B.belcheri]          ---------------------------------H-------------------------S    492
XP_032826770.1[P.marinus]           GVAELGVALPPSDESSKQPKAEALDINGDLSESESTAAPGCVVADGEVHQLRQQPQPPSA    720
XP_032229076.1[N.vectensis]         ------------------------------------------------------------    484
XP_013993323.1[S.salar]             --------------------------------------EGL-------------------    505
OCT93923.1[X.laevis]                ------------------------------------------------------------    499
XP_040518387.1[G.gallus]            --------------------------------------P-S-------------------    503
NP_038946.2[M.musculus]             --------------------------------------P-G-------------------    503
Q9UHP3[H.sapiens]                   --------------------------------------P-S-------------------    503
XP_024095245.1[P.abelii]            --------------------------------------P-S-------------------    503

XP_019852971.1[A.queenslandica]     ------------------------------------------------------------    449
XP_019624636.1[B.belcheri]          ADSLACLSKKT-------------------------------------------------    503
XP_032826770.1[P.marinus]           AAAVACSAPEPHHTQERHGARARQEVPAQQKSGEQNRTGDPPPPGTEQRTADSQQASVLQ    780
XP_032229076.1[N.vectensis]         ------------------------------------------------------------    484
XP_013993323.1[S.salar]             ----------------------------------------AAAPTQAP---STQQRVPIH    522
OCT93923.1[X.laevis]                ---------------------------------------------ETQ---LPTQRSVIH    511
XP_040518387.1[G.gallus]            ----------------------------------------TSP----------VQRSVIH    513
NP_038946.2[M.musculus]             ----------------------------------------SSSPASGA---ALPLRSVIH    520
Q9UHP3[H.sapiens]                   ----------------------------------------T-SPSSVA---AISSRSVIH    519
XP_024095245.1[P.abelii]            ----------------------------------------T-SPSSVA---AISSRSVIH    519

XP_019852971.1[A.queenslandica]     ------------------------------------------------------------    449
XP_019624636.1[B.belcheri]          ------------------------------------------------------------    503
XP_032826770.1[P.marinus]           QGSLQQEAMQVDTLQENVQQGEEEGSQQKDITQQQQELAQGESNQQTETQQRFDLQSEAN    840
XP_032229076.1[N.vectensis]         ------------------------------------------------------------    484
XP_013993323.1[S.salar]             ----------------------------K-------------------------------    523
OCT93923.1[X.laevis]                ----------------------------K-------------------------------    512
XP_040518387.1[G.gallus]            ----------------------------K-------------------------------    514
NP_038946.2[M.musculus]             ----------------------------K-------------------------------    521
Q9UHP3[H.sapiens]                   ----------------------------K-------------------------------    520
XP_024095245.1[P.abelii]            ----------------------------K-------------------------------    520

XP_019852971.1[A.queenslandica]     ------------------------------------------------------------    449
XP_019624636.1[B.belcheri]          -------------------------------LDA----------------VATKKAAPVP    516
XP_032826770.1[P.marinus]           QVSLLQDMQQQQHRGNVQQEHVQQGNPQQGNFQQENSEQCKEQQEGSTQQCDVQRVQAVE    900
XP_032229076.1[N.vectensis]         -------------------------------LSD--------------------------    487
XP_013993323.1[S.salar]             ------------------------------PFTH--------------------------    527
OCT93923.1[X.laevis]                ------------------------------PFTQ--------------------------    516
XP_040518387.1[G.gallus]            ------------------------------PFTQ--------------------------    518
NP_038946.2[M.musculus]             ------------------------------PFTQ--------------------------    525
Q9UHP3[H.sapiens]                   ------------------------------PFTQ--------------------------    524
XP_024095245.1[P.abelii]            ------------------------------PFTQ--------------------------    524

XP_019852971.1[A.queenslandica]     ---ASPNVQEEGRPSPRSGSKEEVEFVRKWLEQWRSEMDTEISELKSSLSATRESLDAVY    506
XP_019624636.1[B.belcheri]          TSEEMAIPSKVHIPCPHHVTKEELAVLQTCLRRWRHEAEKEVKGLQDRILYLQAKIENLY    576
XP_032826770.1[P.marinus]           ARQEGGRLEWHGCPAPQAVTALELELVRAVLSRWNSQVQRELQDLQDSICRIKRTIDLMY    960
XP_032229076.1[N.vectensis]         ---T---RTPSLHPAPRYVTESELETIATSLRRWRTEVEQDVLDISTSLKEVEEAISVMY    541
XP_013993323.1[S.salar]             ---SRLPPDLPMHPAPRHITEEELRVLEGCLHRWRSEVENDTHDLQGSISRIHRTIELMY    584
OCT93923.1[X.laevis]                ---SRIPPDLPMHPAPRNITDEELSVLEGCLHRWRTEVETDTRDLQDSIGRIHRTIELMY    573
XP_040518387.1[G.gallus]            ---SRIPPDLPMHPAPRHITEEELSVLEGCLHRWRTEVENDTRDLQESISRIHRTIELMY    575
NP_038946.2[M.musculus]             ---SRIPPDLPMHPAPRHITEEELCVLESCLHRWRTEIENDTRDLQESISRIHRTIELMY    582
Q9UHP3[H.sapiens]                   ---SRIPPDLPMHPAPRHITEEELSVLESCLHRWRTEIENDTRDLQESISRIHRTIELMY    581
XP_024095245.1[P.abelii]            ---SRIPPDLPMHPAPRHITEEELSVLESCLHRWRTEIENDTRDLQESISRIHRTIELMY    581
                                                 *.*:  :  *:  :   * :*. : : :   :.  :   .  :. :*

XP_019852971.1[A.queenslandica]     SDPSMNRTSYHLHSVLVHQGQASRGHYWAYVRKKKTKKRKATREMGIQASYLDDEQTEEE    566
XP_019624636.1[B.belcheri]          EDNSLKQVPYQLHAVLVHEGQASAGHYWAYVRDHT-------------------------    611
XP_032826770.1[P.marinus]           TDKTFRQLPYRLHAVLVHDGQAMAGHYWAYVREHARLPAAAAASSSS----------S--    1008
XP_032229076.1[N.vectensis]         NDEEMKTIPYRLYAVLVHEGQANGGHYWAYINDPL-------------------------    576
XP_013993323.1[S.salar]             SDKSMMQVPFRLHAVLVHEGQANAGHYWAYIYDPH-------------------------    619
OCT93923.1[X.laevis]                SDKQMNQVPYKLHAVLVHEGQANAGHYWAYIFDHH-------------------------    608
XP_040518387.1[G.gallus]            FDKTMIQVPYRLHAVLVHEGQANAGHYWAYIYDHH-------------------------    610
NP_038946.2[M.musculus]             SDKSMIQVPYRLHAVLVHEGQANAGHYWAYIFDHR-------------------------    617
Q9UHP3[H.sapiens]                   SDKSMIQVPYRLHAVLVHEGQANAGHYWAYIFDHR-------------------------    616
XP_024095245.1[P.abelii]            SDKSMIQVPYRLHAVLVHEGQANAGHYWAYIFDHR-------------------------    616
                                     *  :    ::*::****:***  ******: .

XP_019852971.1[A.queenslandica]     GVDKSSSEEKREEGEGHEDMEVTTTGGDTDVETFSSPLSSCEPSSGPASVAPPEVKREEG    626
XP_019624636.1[B.belcheri]          ------------------------------------------------------------    611
XP_032826770.1[P.marinus]           -------------------------------------------SSGNSASSNSNSVGASD    1025
XP_032229076.1[N.vectensis]         ------------------------------------------------------------    576
XP_013993323.1[S.salar]             ------------------------------------------------------------    619
OCT93923.1[X.laevis]                ------------------------------------------------------------    608
XP_040518387.1[G.gallus]            ------------------------------------------------------------    610
NP_038946.2[M.musculus]             ------------------------------------------------------------    617
```

```
Q9UHP3[H.sapiens]                  ------------------------------------------------------------          616
XP_024095245.1[P.abelii]           ------------------------------------------------------------          616


XP_019852971.1[A.queenslandica]    SKEDEEEIWLKYNDVSVTTVDWEEVKRESFGGRGGESACNSNGPGTNTSAYCLLYVNSEA          686
XP_019624636.1[B.belcheri]         -----QNRWLKFNDVTVTGMSWGELLRESEGG-Y-----------HHASAYCLMYIDITR          654
XP_032826770.1[P.marinus]          ESSGSDSCWRKFNDVRVSRASWAELVRDSYGGGA-----------RSASAYCLVYVNARA          1074
XP_032229076.1[N.vectensis]        -----DRRWRKFNDITVSEVTWEEVERESLGG-Y-----------RNVSAYCLLYCDARR          619
XP_013993323.1[S.salar]            -----QQRWMKYNDISVTKSSWEELVRDSFGG-Y-----------RNASAYCLMYINDKK          662
OCT93923.1[X.laevis]               -----EQRWMKYNDISVTKSSWEELERDSFGG-Y-----------RNASAYCLMYINDEE          651
XP_040518387.1[G.gallus]           -----QNRWMKYNDISVTKSTWEELERDSFGG-Y-----------RNASAYCLMYINDKE          653
NP_038946.2[M.musculus]            -----ESRWMKYNDIAVTKSSWEELVRDSFGG-Y-----------RNASAYCLMYIDDKA          660
Q9UHP3[H.sapiens]                  -----ESRWMKYNDIAVTKSSWEELVRDSFGG-Y-----------RNASAYCLMYINDKA          659
XP_024095245.1[P.abelii]           -----ESRWMKYNDIAVTKSSWEELVRDSFGG-Y-----------RNASAYCLMYINDKA          659
                                        :  * *:**: *:   * *: *:* **               .*****:* :

XP_019852971.1[A.queenslandica]    AKTWREE-----------VHLLSLGLQRYIAYHNLAFERELKAYDLKVQLQQLKTSS--E          733
XP_019624636.1[B.belcheri]         KDLVEVKLQW----------SQYDELKLMVLAENERFIAQQRDLESKQRRTMVVR-----          699
XP_032826770.1[P.marinus]          HDFTSEL----AGEAVGDLSSLSPALRDLVAKDNHRFLSEMAMRGNGPERPAEPDADARE          1130
XP_032229076.1[N.vectensis]        EEVIRAGN---------TPEVLSAELQQMVNEDNASFEQELRDWDEKQAKAAKEAKS---          667
XP_013993323.1[S.salar]            PFLIEEEFDKETGQMLSGLDKLPSDLKAYVKEDNGLFDKEMEEWDKLQARKAQQEKL---          719
OCT93923.1[X.laevis]               KCLMQEEYDKETGQVLKGMDILPLDLREYIKEDNRRFEKELEAWDKEQLRKAEQEKM---          708
XP_040518387.1[G.gallus]           QYLIEEEFNKETGQILVGMDTLPSDLRDYVKEDNKRFEKELEEWDAELAQKAQQEKL---          710
NP_038946.2[M.musculus]            QFLIQEEFNKETGQALVGMETLPPDLRDFVEEDNQRFEKELEEWDTQLAQRSLQEKL---          717
Q9UHP3[H.sapiens]                  QFLIQEEFNKETGQPLVGIETLPPDLRDFVEEDNQRFEKELEEWDAQLAQKALQEKL---          716
XP_024095245.1[P.abelii]           QFLIQEEFNKETGQPLVGIETLPPDLRDFVEEDNQRFEKELEEWDAQLAQKALQEKL---          716
                                                            *:  :  .*  *  :

XP_019852971.1[A.queenslandica]    -G------------------KEL-----IKPAKAVRFE-YPK------------------          750
XP_019624636.1[B.belcheri]         --------------------------EEDPPAN--------HGRGQGTTSESSSGEVA--          723
XP_032826770.1[P.marinus]          PGQRAEPREHEQEERRHEHGGEQAPARAAPPAAAAAAAASREGEGSGAAREQERADKHSQ          1190
XP_032229076.1[N.vectensis]        ----------------------QALVVARPDLVLDA-------EA---------------          683
XP_013993323.1[S.salar]            ----------------------ALAAAAA----ATA-------ET---------------          731
OCT93923.1[X.laevis]               ----------------------ILAQKAL----K--------------------------          716
XP_040518387.1[G.gallus]           ----------------------LSQIPR--------------------------------          716
NP_038946.2[M.musculus]            ----------------------LAAPKLR----E--------------------------          725
Q9UHP3[H.sapiens]                  ----------------------LASQKLR----E--------------------------          724
XP_024095245.1[P.abelii]           ----------------------LASQKLR----E--------------------------          724


XP_019852971.1[A.queenslandica]    ------------------------------------------------------------          750
XP_019624636.1[B.belcheri]         -----------DQPIQ-----GQEHRVDVQRKQ--------------------------P          741
XP_032826770.1[P.marinus]          TNSLEQEAEPTRERVDGRARAGEREETRVHEGRAPDVASISETQRQGSEADGSASLPGRE          1250
XP_032229076.1[N.vectensis]        --------------------------SCAVSP------------------------RAHS          693
XP_013993323.1[S.salar]            --------------------------TTSHQP------------------------MSTE          741
OCT93923.1[X.laevis]               ------------------------------------------------------------          716
XP_040518387.1[G.gallus]           -----------------------------------------------------------A          717
NP_038946.2[M.musculus]            -----------------------------------------------------------A          726
Q9UHP3[H.sapiens]                  -----------------------------------------------------------S          725
XP_024095245.1[P.abelii]           -----------------------------------------------------------S          725


XP_019852971.1[A.queenslandica]    GEEDPGASTNGGHDMMALASDAKRARAGSPSNLEDMQIDLDNLPPVDPALLNQ-------          803
XP_019624636.1[B.belcheri]         PQVKPKPVTTETQQGVRLGRTSQQSTP---LD------------HSGH------------          774
XP_032826770.1[P.marinus]          GTSAPSPSPSGTEEYMKVGPDTPTEKP---RVERVVEV---SIPDVGTIVVQAEEHGYND          1304
XP_032229076.1[N.vectensis]        PAAAR-------SES---------------VLTA----------NLGVL-----------          710
XP_013993323.1[S.salar]            PSPPDSWGPQQDPEYMEQPSPTDDSKH---LQED----------TERAI-----------          777
OCT93923.1[X.laevis]               QAPLVVAAPPNEPEYMEQPARADAPNE---TSEE----------TTNVI-----------          752
XP_040518387.1[G.gallus]           PAPSPAPVQAGEPEYLEQPSRTDLSKH---LKED----------SVQAI-----------          753
NP_038946.2[M.musculus]            EASATTAQA-GGADYLEQPSRSDLSKH---WKEE----------TLRVI-----------          761
Q9UHP3[H.sapiens]                  ETSVTTAQAAGDPEYLEQPSRSDFSKH---LKEE----------TIQII-----------          761
XP_024095245.1[P.abelii]           ETSVTTAQAAGDPEYLEQPSRSDFSKH---LKEE----------TIRII-----------          761
                                                :

XP_019852971.1[A.queenslandica]    ----------LSMRLSDKLQMFIQDVPGTIIESRLL------------------------          829
XP_019624636.1[B.belcheri]         --VL----QATMKTILEVSNIQEQQGPEK----ALIKAVNIEYHRLRSLVEKGISR----          820
XP_032826770.1[P.marinus]          EVMLTPAMQGVLMAVARARLVREAQGPEA----TLIEAFRVEYARLRTLAQESYPDASSP          1360
XP_032229076.1[N.vectensis]        ------------P--S----LVDSSGKKLTIDEAITQWCNNEKTRLDKLAREYPPEY---          749
XP_013993323.1[S.salar]            ------------S--KAAAEQQEERSPEG----LLTAAIKVEYTRLLRFAQEDTA-P---          815
OCT93923.1[X.laevis]               ------------T--QAATE-SEDKGPEA----VLQMAIKTEYARLVKLAQEDPL-P---          789
XP_040518387.1[G.gallus]           ------------N--KALAE-QEDRGPEA----IMDTAIKLEYTRLLKLAQEDPP-P---          790
NP_038946.2[M.musculus]            ------------A--KASHD-LEDKGPET----VLQSAIKLEYSRLVKLAQEDTP-P---          798
Q9UHP3[H.sapiens]                  ------------T--KASHE-HEDKSPET----VLQSAIKLEYARLVKLAQEDTP-P---          798
XP_024095245.1[P.abelii]           ------------T--KASHE-HEDKSPET----VLQSAIKLEYARLVKLAQEDTP-P---          798
                                                                     :

XP_019852971.1[A.queenslandica]    --------------------------TDFHYAHACQTSEKVRQAILYEHVIQD-LKR-EN          861
XP_019624636.1[B.belcheri]         -------------------QYDPRLEHCVVYLILNGAARVIVERTLVEQFADRGLSGDSK          861
XP_032826770.1[P.marinus]          ASPPLAPAGASGAAATAWRGREERLEHVVIYLFQNEAPNRVVERALLEQFADRNLSYDEQ          1420
XP_032229076.1[N.vectensis]        -------------------PGDVRLEHLGIYLMRCSAPDFTVECAILESVIDEVKAKDQS          790
XP_013993323.1[S.salar]            -------------------ENDYRLQHVIVYFIHNQAPKKILERTLLMQFADRNLSFDER          856
OCT93923.1[X.laevis]               -------------------EIDYRLQHAIIYFIQSQAPKKIIERTLLEQFADRNLSFDER          830
XP_040518387.1[G.gallus]           -------------------ECDYRLRHAIVYFIQNQAPKKIIERTLLEQFGDHNLSFDER          831
NP_038946.2[M.musculus]            -------------------ETDYRLHHVLVYFIQNQAPKKIIEKTLLEQFGDRNLSFDER          839
Q9UHP3[H.sapiens]                  -------------------ETDYRLHHVVVYFIQNQAPKKIIEKTLLEQFGDRNLSFDER          839
XP_024095245.1[P.abelii]           -------------------ETDYRLHHVVVYFIQNQAPKKIIEKTLLEQFGDRNLSFDER          839
                                                                 *     :     :  :   . :      .

XP_019852971.1[A.queenslandica]    DLNMSLIARKYFVEICKDPQIYQELQDVRLLYSRFCSCCYHFIQSVNEAKRNSWCLSLAH          921
XP_019624636.1[B.belcheri]         SIEIMKVAQSKLKIMQLSEENLKAYEKWHADYGLFQKTVMFLLRGIEFFHQERFPEALTY          921
XP_032826770.1[P.marinus]          LIKIMKVAQAKLQEISPEDMDPAEYQKWHQEYELFRQVCVYLVMGIEKYQEGRYTEALPC          1480
XP_032229076.1[N.vectensis]        LMPYRKRVQEFLERE-KGTDRAADYKRWKEMYMTFKLINSCFLTGLEHFHSQKYKESLPY          849
XP_013993323.1[S.salar]            CKSIMKVARAKLDLVKPEEVNMEEYEMWHQDYRNFRETTIFLMIGLELFQKKCFVEALMY          916
OCT93923.1[X.laevis]               CRNIMKVAQAKLEMIKPDEVNMEEYRKWHKDYRMFRETTMYILIGLELFQNKSYVEALLY          890
XP_040518387.1[G.gallus]           CRNIMKVAQAKLEMIKPDEVNMEEYERWHQDYRNFRETTMFLMVGLEFFQKKSYMEALLY          891
NP_038946.2[M.musculus]            CHNIMKVAQAKLEMIKPEEVNLEEYEEWHADYKKFRETTMYLITGLENFQRESYIDSLLF          899
Q9UHP3[H.sapiens]                  CHNIMKVAQAKLEMIKPEEVNLEEYEEWHQDYRKFRETTMYLIIGLENFQRESYIDSLLF          899
XP_024095245.1[P.abelii]           CHNIMKVAQSKLEMIKPEEVNLEEYEEWHQDYRKFRETTMYLIIGLENFQRESYIDSLLF          899
                                          .:  :             .  :  *  *      :: .::  :   :  :*

XP_019852971.1[A.queenslandica]    IINCFYLNSSISAQAGDK-YSLDQQLLGRFRCKALLMVSEIGTVAFTSGEVS---SSIVI          977
XP_019624636.1[B.belcheri]         LVHAWTYNQQLLGEGKNYIMAADSSLITHYRTQCLKSLSEQACSLFESGDTESVDEGLQL          981
XP_032826770.1[P.marinus]          LMHAHAVNEELLAR-GQR-RGVRRDVLARYRRVCVRRVNEACALTFGTGDVAQATRSLAV          1538
XP_032229076.1[N.vectensis]        LVHAYELNRKLSDPTNNA-RSMDQRLLAHCRRVCFLKLNELAISLFESDDYKTLCEGLDL          908
XP_013993323.1[S.salar]            LIYSYQYNRELLVK-GLY-RGHNDELIGHYRRECLLKLNENAARMFESGEEPEVCNGLSI          974
OCT93923.1[X.laevis]               LIYGYQYNKELLSK-GLY-RGHDEQLISHYRRECFLKLNEQAAAMFESGDMDEVNNGLAS          948
XP_040518387.1[G.gallus]           LIYAYQNNKELLSK-GPY-RGHDEELISHYRRECLLKLNEHAAALFESGDDQEVNNGLII          949
NP_038946.2[M.musculus]            LLCAYQNNKELLSK-GPY-RGHDGELISHYRRECLLKLNEQAAELFESGEDGDVNNGLII          957
Q9UHP3[H.sapiens]                  LICAYQNNKELLSK-GLY-RGHDEELISHYRRECLLKLNEQAAELFESGEDREVNNGLII          957
XP_024095245.1[P.abelii]           LICAYQNNKGLLSK-GLY-RGHDEELISHYRRECLLKLNEQAAELFESGEDREVNNGLII          957
                                   ::     *  :         .    :: : *  ..  :.*     * :.:      .:

XP_019852971.1[A.queenslandica]    GNDYIVPGLKAFSLHP---------------FPGDDSILEKVREEWCQCLDQSSLTEDDS          1022
XP_019624636.1[B.belcheri]         MVELVVPCMALLQELG--------------GTDSDQAIAEEIRSNWCDYLGQ-DLPDWCQ          1026
```

```
XP_032826770.1[P.marinus]          MTELVLPAMALLAPLGSSCEGGDAGDEAAVARESDLCAVELMRDRWCSYLGRDDMASELQ          1598
XP_032229076.1[N.vectensis]        VSEQVVPCIPQLVTSP--------------Y-AEDREAVEHMRNNWFTYLGG-TVDEKRQ          952
XP_013993323.1[S.salar]            MNELVVPCIPLLLVHD--------------T-EKDLLAVEDMRNRWCSYLGQ-EMEPNLQ          1018
OCT93923.1[X.laevis]               MNELIVPCLPLLLVDD--------------MEEKDIVAVEDMRNRWCSYLGQ-EMEPNLQ          993
XP_040518387.1[G.gallus]           MNELIVPCLPLLLVDE--------------MEEKDIVAVEDMRNRWCSYLGQ-EMEPNLQ          994
NP_038946.2[M.musculus]            MNEFIVPFLPLLLVDD--------------MEEKDILAVEDMRNRWCSYLGQ-EMEANLQ          1002
Q9UHP3[H.sapiens]                  MNEFIVPFLPLLLVDE--------------MEEKDILAVEDMRNRWCSYLGQ-EMEPHLQ          1002
XP_024095245.1[P.abelii]           MNEFIVPFLPLLLVDE--------------MEEKDILAVEDMRNRWCSYLGQ-EMEPHLQ          1002
                                     : ::* :  :                      *    * :*..*   *.   :    .

XP_019852971.1[A.queenslandica]    DKLADLLMKIVEDYSKAPNIK-----------------------------------IPQA          1047
XP_019624636.1[B.belcheri]         EKLQDFLPQLLDCSGD-----------------------------------LQQLRTPPA          1051
XP_032826770.1[P.marinus]          ELLTDFLPRLLDYQPE-PAPPTQLTPPPLQGPPPPPPPPSEPRKCESSGENWRGLRAPPR          1657
XP_032229076.1[N.vectensis]        EKLQDFLPKLLDGSTD---------------------------------GNFEEMRPPPV          979
XP_013993323.1[S.salar]            EKLTDFLPKLLDCSTE-----------------------------------IKSFHDPPK          1043
OCT93923.1[X.laevis]               EKLTDFLPKLLDCSTE-----------------------------------IKGLNDPPK          1018
XP_040518387.1[G.gallus]           EKLTDFLPKLLDCSTE-----------------------------------IKGFNDPPK          1019
NP_038946.2[M.musculus]            EKLTDFLPKLLDCSTE-----------------------------------IKGFHEPPK          1027
Q9UHP3[H.sapiens]                  EKLTDFLPKLLDCSME-----------------------------------IKSFHEPPK          1027
XP_024095245.1[P.abelii]           EKLTDFLPKLLDCSME-----------------------------------IKSFHEPPK          1027
                                   : * *:* ::::   .                                         *

XP_019852971.1[A.queenslandica]    PSNFDTQRLAAQYTTAYKTLTSEILL----     1073
XP_019624636.1[B.belcheri]         V--WPSQHLAEWFSTVMQAVVLAEPDTVD-     1078
XP_032826770.1[P.marinus]          LKAYSPHTLAEKMAEVLRVNR---PREA--     1682
XP_032229076.1[N.vectensis]        MRPTNSKDLCERFTRAMEIVHANMPFAAKA     1009
XP_013993323.1[S.salar]            LPTYSTLELVERYGRVMASLCR-VPADGR-     1071
OCT93923.1[X.laevis]               LPNFSTHELCERFARIMLSLSR-MPADGR-     1046
XP_040518387.1[G.gallus]           LPSYSTHELCERYARIMLSLSR-TPADGR-     1047
NP_038946.2[M.musculus]            LPSYSAHELCERFARIMLSLSR-TPADGR-     1055
Q9UHP3[H.sapiens]                  LPSYSTHELCERFARIMLSLSR-TPADGR-     1055
XP_024095245.1[P.abelii]           LPSYSTHELCERFARIMLSLSR-TPADGR-     1055
                                           *
```
