# Supplementary material for: An Evolutionary Perspective on the Origin, Conservation and Binding Partner Acquisition of Tankyrases
Source: Biomolecules. 2022 Nov 15;12(11):1688. doi: 10.3390/biom12111688 (PMC9688111; doi:10.3390/biom12111688)
Supplement: Supplementary file 1 [file biomolecules-12-01688-s001.zip › biomolecules-1913719-supplementary-11.17 -done/Supplementary-proofed.pdf]

An Evolutionary Perspective on the Origin, Conservation and Binding Partner Acquisition of Tankyrases

Sven T. Sowa [1], Chiara Bosetti [1], Albert Galera-Prat [1], Mark S. Johnson [2] and Lari Lehtiö [1,*]

[1] Faculty for Biochemistry and Molecular Medicine & Biocenter Oulu, University of Oulu, Oulu 90220, Finland
[2] Structural Bioinformatics Laboratory, Biochemistry, Faculty of Science and Engineering and InFLAMES Research Flagship Center, Åbo Akademi University, Turku 20520, Finland
* Correspondence: lari.lehtio@oulu.fi

Supplementary material content

Table S1. **Protein sequence identifiers from the analysis of tankyrase distribution and origin. Related to Figure 2.**

Figure S1. **Phylogenetic analysis of tankyrases. Related to Figure 2.**

Figure S2. **Predicted structure of the TNKS-like ankyrin-repeat domain from the archaeon *Acidianus manzaensis*. Related to Figure 2.**

Figure S3. **Predicted structure of the TNKS-like ART domain from the amoeba *Naegleria fowleri*. Related to Figure 2.**

Figure S4. **Conservation of TNKS ARC domain residues mapped to the structures. Related to Figure 4.**

Figure S5. **The ARC3 domains in human and early-diverging tankyrases are unlikely to bind TBM peptides. Related to Figure 4.**

Figure S6. **Size-exclusion chromatography of TNKS1 and *Aq*TNKS constructs. Related to Figure 5.**

Figure S7. **Distribution of possible tankyrase binding motifs in orthologs of RNF146. Related to Figure 6.**

Table S1. Protein sequence identifiers from the analysis of tankyrase distribution and origin. Related to Figure 2. Accession identifiers for the NCBI protein database are shown for each species. ANK = Ankyrin-repeat domain; ART = ADP-ribosyltransferase domain. *Data from transcriptome shotgun assembly; identifier refers to the NCBI nucleotide databank. **Identifier refers to the transcript in the EnsemblGenomes database.

Phylogenetic group	Species	Common name	Accession identifier
Archaea	*Acidianus manzaensis*	N/A	ANK: WP_148690368.1
Percolozoa	*Naegleria fowleri*	Brain-eating amoeba	TNKS-like ART: XP_044559609.1
Choanoflagellata	*Salpingoeca kvevrii*	N/A	TNKS*: GGOX01000296.1
	Salpingoeca macrocollata	N/A	TNKS*: GGOT01036632.1
Porifera (Sponges)	*Amphimedon queenslandica*	N/A	TNKS: XP_019848937.1
Placozoa	*Trichoplax adhaerens*	N/A	No TNKS ortholog found.
Ctenophora (Comb jellies)	*Mnemiopsis leidyi*	Sea walnut	TNKS**: ML097525a-RA
Cnidaria (Cnidarians)	*Nematostella vectensis*	N/A	TNKS: XP_032220531.1
	Hydra vulgaris	N/A	TNKS: XP_047128184.1
Mollusca (Molluscs)	*Octopus bimaculoides*	California two-spot octopus	TNKS: XP_014782906.1
	Crassostrea gigas	Pacific oyster	TNKS: XP_019923312.2
Nematoda (Roundworms)	*Brugia malayi*	N/A	TNKS: CDP94254.1
	Trichinella spiralis	Pork worm	TNKS: KRY42746.1
	Caenorhabditis elegans	N/A	No TNKS ortholog found.
Arthropoda (Arthropods)	*Drosophila melanogaster*	Fruit fly	TNKS: NP_651410.1
	Daphnia pulex	Water flea	TNKS: XP_046457952.1
	Ixodes scapularis	Deer tick	TNKS: XP_029842287.2
Echinodermata	*Asterias rubens*	Common starfish	TNKS: XP_033632864.1
	Acanthaster planci	Crown-of-thorns starfish	TNKS: XP_022094330.1
Cephalochordata (Lancelets)	*Branchiostoma belcheri*	Belcher's lancelet	TNKS: XP_019641281.1
	Branchiostoma lanceolatum	European lancelet	TNKS: CAH1257770.1
Tunicata (Tunicates)	*Ciona intestinalis*	Vase tunicate	TNKS: XP_002121662.3
	Oikopleura dioica	N/A	TNKS: CBY09100.1
Agnatha (Jawless fish)	*Petromyzon marinus*	Sea lamprey	TNKS: XP_032806710.1
	Eptatretus burgeri	Inshore hagfish	TNKS**: ENSEBUT00000023950.1
Chondrichthyes (Cartilaginous fish)	*Callorhinchus milii*	Australian ghostshark	TNKS1: XP_042200715.1<br>TNKS2: XP_007894887.1
	Amblyraja radiata	Thorny skate	TNKS1: XP_032873799.1<br>TNKS2: XP_032889463.1
	Carcharodon carcharias	Great white shark	TNKS1: XP_041041558.1<br>TNKS2: XP_041066021.1
Osteichthyes (Bony fish)	*Salmo salar*	Atlantic salmon	TNKS1: XP_014017013.1<br>TNKS2: XP_014034742.2
	Protopterus annectens	West African lungfish	TNKS1: XP_043916872.1<br>TNKS2: XP_043912589.1
	Latimeria chalumnae	Coelacanth	TNKS1: XP_006007641.1<br>TNKS2: XP_006006371.1
Amphibia (Amphibians)	*Xenopus laevis*	African clawed frog	TNKS1: XP_018099068.1<br>TNKS2: XP_018082988.1
	Nanorana parkeri	High Himalaya Frog	TNKS1: XP_018421084.1<br>TNKS2: XP_018427335.1
Reptilia (Reptiles)	*Crocodylus porosus*	Saltwater crocodile	TNKS1: XP_019389515.1<br>TNKS2: XP_019411065.1
	Chelonia mydas	Green sea turtle	TNKS1: XP_037752450.1<br>TNKS2: XP_007059469.2
Aves (Birds)	*Gallus gallus*	Red junglefowl	TNKS1: NP_989671.2<br>TNKS2: NP_989672.2
	Tyto alba	Barn owl	TNKS1: XP_042664360.1<br>TNKS2: XP_032840041.2
Mammalia (Mammals)	*Ornithorhynchus anatinus*	Platypus	TNKS1: XP_001508887.3<br>TNKS2: XP_028915823.1
	Sarcophilus harrisii	Tasmanian devil	TNKS1: XP_031799023.1<br>TNKS2: XP_031811897.1
	Bos taurus	Cattle	TNKS1: NP_001193089.1<br>TNKS2: XP_024841840.1
	Mus musculus	House mouse	TNKS1: NP_780300.2

			TNKS2: NP_001157107.1
	Homo sapiens	Modern human	TNKS1: NP_003738.2<br>TNKS2: NP_079511.1

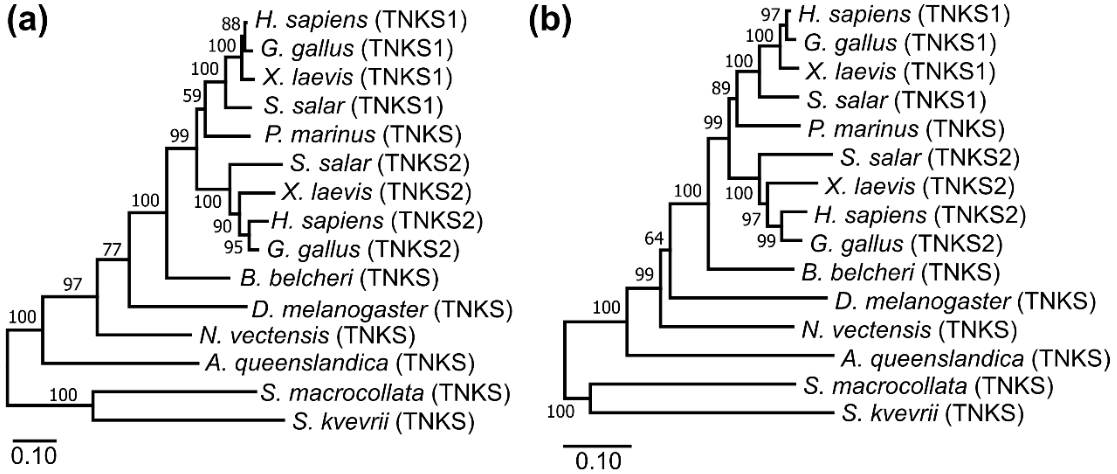

Figure S1. Phylogenetic analysis of tankyrases. Related to Figure 2. Construction of the phylogenetic trees was done with Maximum Likelihood (**a**) or Neighbor Joining (**b**). Percentages for bootstrap support values are shown for each node. Bootstrap tests were done with 1000 bootstrap replicates. The analysis was performed with truncated sequences starting from the ARC3 domain to match the likely incomplete sequence from *S. macrocollata*.

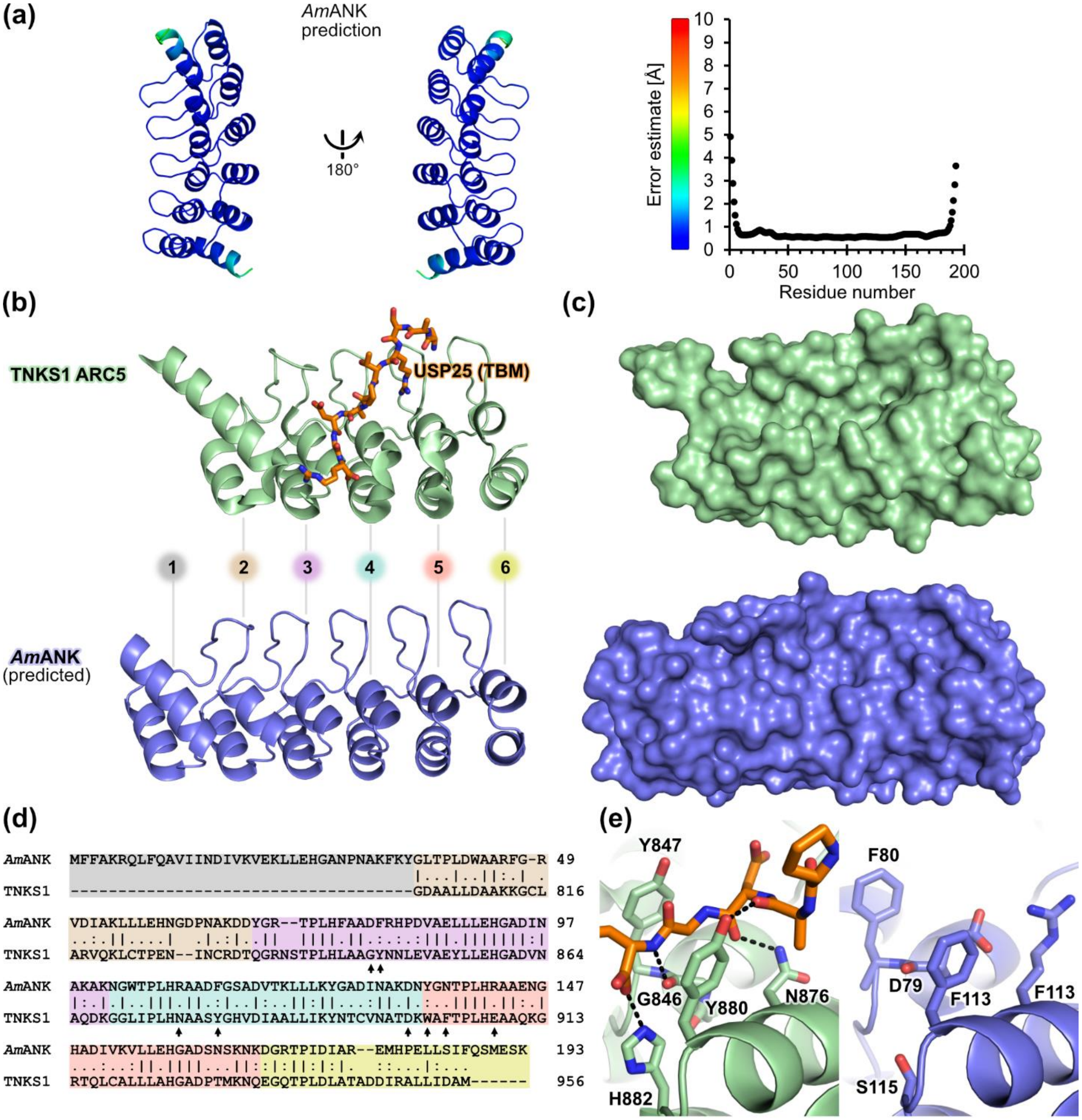

Figure S2. Predicted structure of the TNKS-like ankyrin-repeat domain from the archaeon *Acidianus manzaensis*. Related to Figure 2. (**a**) The structure of the *A. manzaensis* ankyrin-repeat domain (*Am*ANK) was predicted by RoseTTAFold. Coloring of the model corresponds to the estimation of errors in Å. (**b**) Side-by-side view of the human TNKS1 ARC5 domain in complex with an USP25 TBM peptide (PDB: 5GP7) and the predicted structure of *Am*ANK. ARC5 contains 5 and *Am*ANK contains 6 ankyrin-repeat units. The ankyrin-repeat units are numbered. (**c**) Surface representations of TNKS1 ARC5 and *Am*ANK. While both structures are similar, the peptide-binding pocket in ARC5 appears more well-defined. (**d**) Pairwise sequence alignment of *Am*ANK and the ARC5 domain from TNKS1. The ankyrin-repeat units are highlighted in different colors. Important residues for TBM binding are indicated by arrows for TNKS1. (**e**) Close-up view of the aromatic glycine-sandwich sub-site in ARC5 binding the USP25 TBM peptide (left)

and corresponding view of *Am*ANK (right). While the two tyrosines are replaced with phenylalanines in *Am*ANK, other residues facilitating this interaction differ and would likely not permit this mode of peptide-binding.

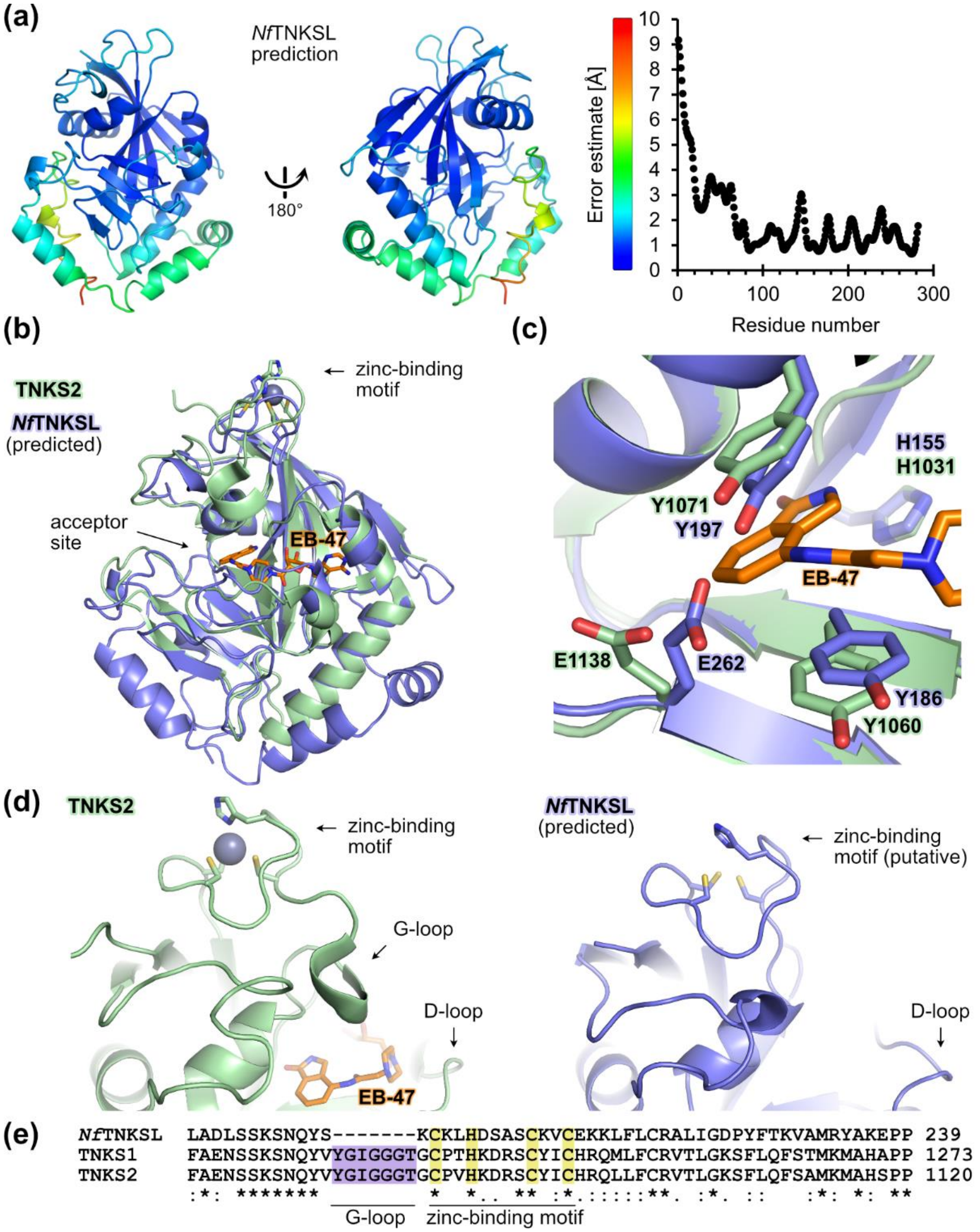

Figure S3. Predicted structure of the TNKS-like ART domain from the amoeba *Naegleria fowleri*. Related to Figure 2. (**a**) The structure of the *N. fowleri* TNKS-like (*Nf*TNKSL) ART domain was predicted by RoseTTAFold. Coloring of the model corresponds to the estimation of errors in Å. (**b**) Superposition of the predicted *Nf*TNKSL structure and the crystal structure of TNKS2 ART domain in complex with the NAD+-mimic EB-47 (PDB: 4BJ9). (**c**) Close-up view of the nicotinamide sub-pocket for the superimposed structures. (**d**) Side-by-side comparison of the structures from TNKS2 and *Nf*TNKSL. The unique zinc-binding motif in TNKSs was modelled in very similar conformation in the predicted *Nf*TNKSL structure, while the Glycine-rich loop (G-loop) present in TNKSs is absent in *Nf*TNKSL. (**e**) Multiple sequence alignment of ART domains from human TNKS1, human TNKS2 and *Nf*TNKSL. The G-loop region is

colored in violet and not present in *Nf*TNKSL; the ligand residues from the zinc-binding motif are highlighted in yellow.

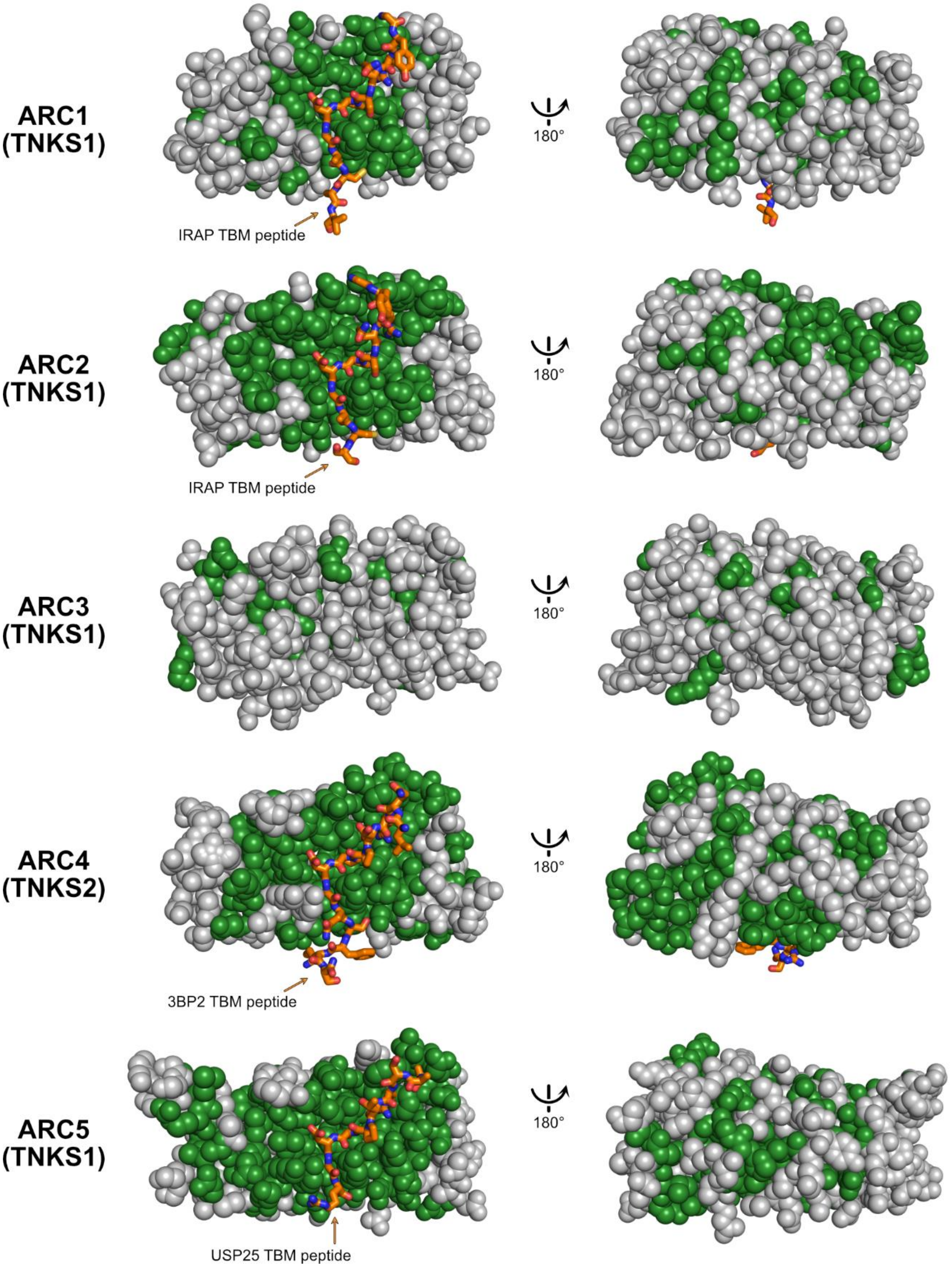

Figure S4. Conservation of TNKS ARC domain residues mapped to the structures. Related to Figure 4. A multiple sequence alignment of TNKSs from 11 metazoan species (as exemplified in Figure 4b) was used to map all identical residues to the structures of TNKS ARC domains, shown in green: TNKS1 ARC1 (PDB:

5JHQ), TNKS1 ARC2 (PDB: 5JHQ), TNKS1 ARC3 (PDB: 5JHQ), TNKS2 ARC4 (PDB: 3TWR), TNKS1 ARC5 (PDB: 5GP7).

(a)

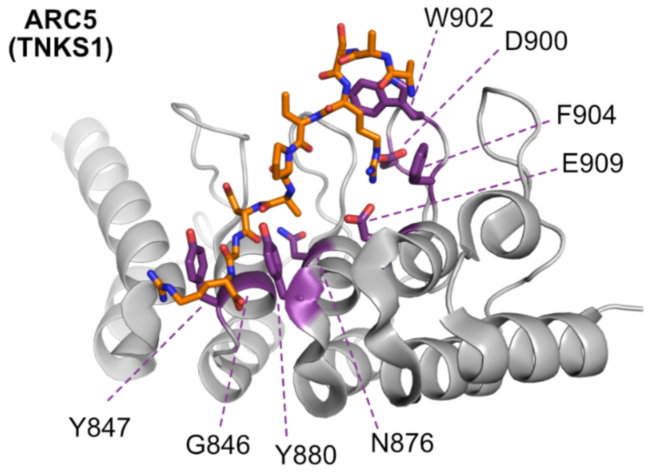

(b)

```
                                                                G846
                                                                 Y847
TNKS1_ARC5       --GDAALLDAAKKGCLARVQKLCTPENINCRDTQGRNSTPLHLAA---GYNNLEVAEYLL
TNKS1_ARC3       EFKGHSLLQAAREADLAKVKKTLALEIINFKQPQSH-ETALHCAVASLHPKRKQVTELLL
PmTNKS_ARC3      EFRGHSLLQAAREADVTRVKKHLGLDIINFKHPQTH-ETALHCAVASPYPKRKQVSELLL
BbTNKS_ARC3      EFKGHCLLDGARQTDMAKVKKHLQLDIVNFKHPYTH-DTALHCAALSPYPKRRQITELLI
DmTNKS_ARC3      EYKGHCLLDACRKCDVSRAKKLVCAEIVNFVHPYTG-DTPLHLAVVSPDGKRKQLMELLT
AqTNKS_ARC3      EYRGYQLLAAAEDGGIILLKKLLSSQLLKFQHHQTL-DTLLHKAVLSKSSNRQSVIDALL
                    .  ** ....  :   :*    : ::  .     .* ** *.     :. .: : *

                                   N876 Y880                D900 W902 F904 E909
TNKS1_ARC5       EHGADVNAQDKGGLIPLHNAASYGHVDIAALLIKYNTCVNATDKWAFTPLHEAAQKGRTQ
TNKS1_ARC3       RKGANVNEKNKDFMTPLHVAAERAHNDVMEVLHKHGAKMNALDTLGQTALHRAALAGHLQ
PmTNKS_ARC3      RKGANVNEKTKDLLTPLHVASDKAHNDVIDVLLKHGAKINVTDSLGQTSLHRAAHAGHLT
BbTNKS_ARC3      RKGANLNDKNKEFLTPLHVASDKCYIDVMEVLLKHGAKVNALDSLGQTALHRGAHCGHVQ
DmTNKS_ARC3      RKGSLLNEKNKAFLTPLHLAAELLHYDAMEVLLKQGAKVNALDSLGQTPLHRCAR--DEQ
AqTNKS_ARC3      KRGINLNIGNKENMTPLICAAKKGILEVVEQLVQRGANINHQDINGMTSLHWAVQNEHAQ
                 .:*  :*   *  : **  *:.    :    * : .: :*  *  . * **  .

TNKS1_ARC5       LCALLLAHGADPTMKNQEGQTPLDLATADDIRALLIDAM
TNKS1_ARC3       TCRLLLSYGSDPSIISLQGFTAAQMGNEAVQQILS----
PmTNKS_ARC3      TCRLLLSHGCDPNLPSLQGFTAAQLGNESVQQILH----
BbTNKS_ARC3      ACRLLLSFGVDPSIVSLQGVTAAQMATEAVQQMLH----
DmTNKS_ARC3      AVRLLLSYAADTNIVSLEGLTAAQLASDSVLKLLK----
AqTNKS_ARC3      ICRYLLSSGANPSIVNNQGQTIYQLKTSDTIQLIL----
                     **: . : .: . :* *  :: .    : :
```

Figure S5. The ARC3 domains in human and early-diverging tankyrases are unlikely to bind TBM peptides. Related to Figure 4. (**a**) Residues important for binding TBMs by TNKS ARC domains are colored purple and mapped to the structure of ARC5 from human TNKS1 (PDB: 5GP7). The TBM peptide of USP25 is shown in orange. (**b**) Multiple sequence alignment of ARC5 from human TNKS1 and ARC3 sequences from human TNKS1, *Petromyzon marinus* TNKS (*Pm*TNKS), *Branchiostoma belcheri* TNKS (*Bb*TNKS), *Drosophila melanogaster* TNKS (*Dm*TNKS) and *Amphimedon queenslandica* TNKS (*Am*TNKS). Residues important for binding TBMs are highlighted in purple for TNKS1 ARC5. Non-identical residues in these positions are marked in gray.

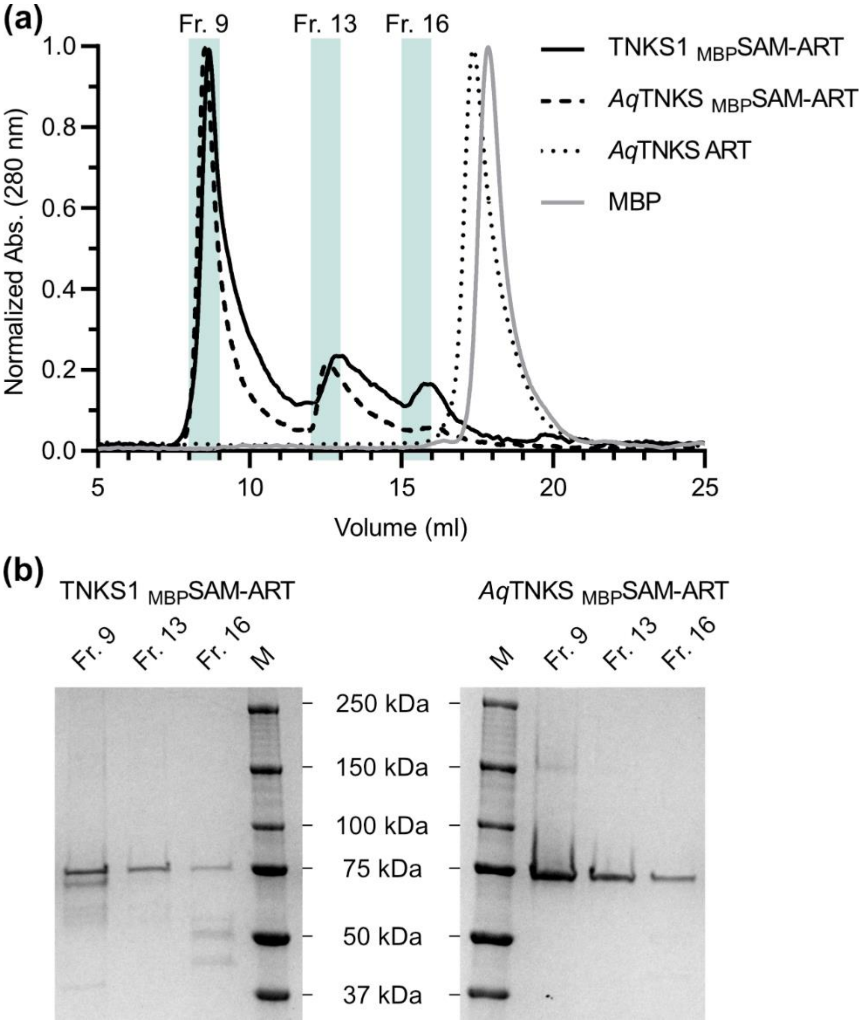

Figure S6. Size-exclusion chromatography of TNKS1 and *Aq*TNKS constructs. Related to Figure 5. (**a**) Size-exclusion chromatogram of TNKS constructs or MBP. In separate runs, 0.5 mg of each protein was loaded to a Superdex 200 Increase 10/300 GL column at a flowrate of 0.5 ml/min at 4°C. The absorbance at 280 nm was monitored and fractions (Fr.) of 1 ml were taken. (**b**) For the SAM-ART constructs of TNKS1 or *Aq*TNKS, fractions 9, 13 and 16 were analyzed by SDS-PAGE. Precision Plus Protein All Blue (BioRad) was used as protein weight marker (M).

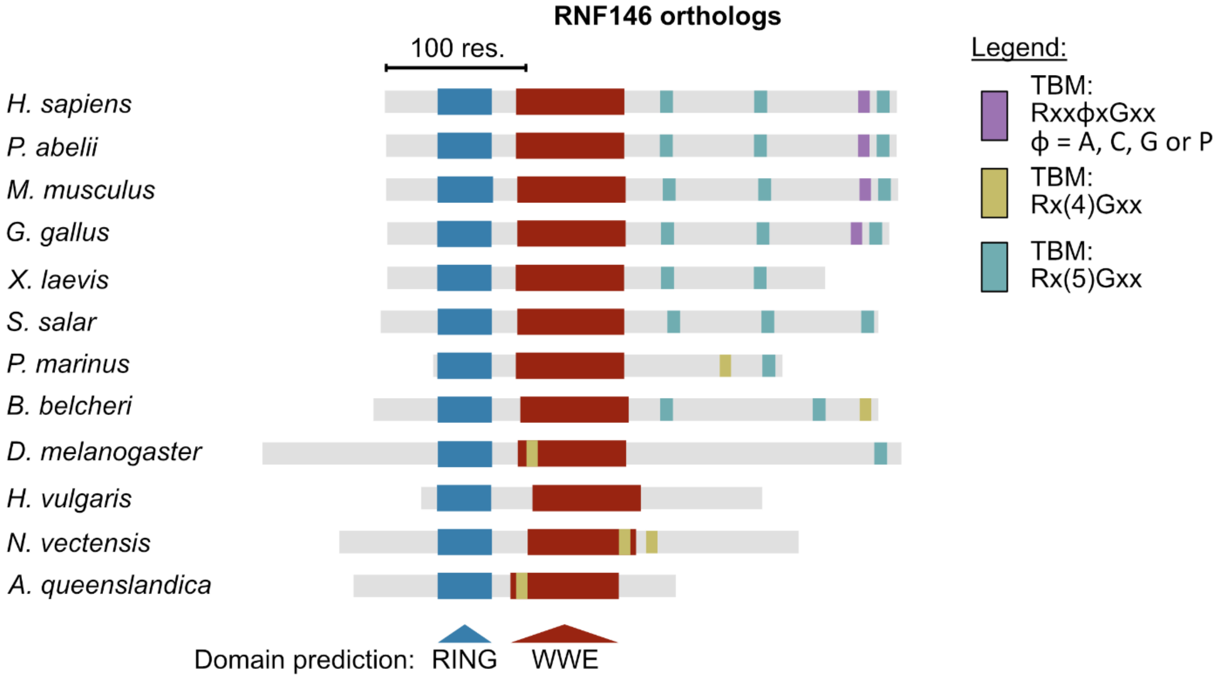

Figure S7. Distribution of possible tankyrase binding motifs in orthologs of RNF146. Related to Figure 6. Orthologs of RNF146 were examined for presence and location of possible TBM sequences. Three configurations Rxx[ACGP]xGxx, Rx(4)Gxx and Rx(5)Gxx were considered as TBM. The prediction of the domain positions for RING finger (blue) and WWE (red) domains was done using InterPro. Parts of the proteins without domain predictions are likely disordered regions and are shown in gray. A scale corresponding to 100 amino acid residues is shown.
